# Reprogramming in *Candida albicans* Gene Expression Network under Butanol Stress Abrogates Hyphal Development

**DOI:** 10.3390/ijms242417227

**Published:** 2023-12-07

**Authors:** Rajesh Anand, Mohammad Kashif, Awadhesh Pandit, Ram Babu, Agam P. Singh

**Affiliations:** 1 Infectious Disease Laboratory, National Institute of Immunology, New Delhi 110067, India; rajesh.anand@nii.ac.in (R.A.);; 2 Next Generation Sequencing Facility, National Institute of Immunology, New Delhi 110067, India; 3 Department of Botany, Kirori Mal College, University of Delhi, Delhi 110007, India

**Keywords:** whole human serum, *Candida albicans*, transcriptome, butanol, hypha

## Abstract

*Candida albicans* is the causative agent of invasive fungal infections. Its hyphae-forming ability is regarded as one of the important virulence factors. To unravel the impact of butanol on *Candida albicans*, it was placed in O^+ve^ complete human serum with butanol (1% *v*/*v*). The *Candida* transcriptome under butanol stress was then identified by mRNA sequencing. Studies including electron microscopy demonstrated the inhibition of hyphae formation in *Candida* under the influence of butanol, without any significant alteration in growth rate. The numbers of genes upregulated in the butanol in comparison to the serum alone were 1061 (20 min), 804 (45 min), and 537 (120 min). *Candida* cells exhibited the downregulation of six hypha-specific transcription factors and the induction of four repressor/regulator genes. Many of the hypha-specific genes exhibited repression in the medium with butanol. The genes related to adhesion also exhibited repression, whereas, among the heat-shock genes, three showed inductions in the presence of butanol. The fungal-specific genes exhibited induction as well as repression in the butanol-treated *Candida* cells. Furthermore, ten upregulated genes formed the core stress gene set in the presence of butanol. In the gene ontology analysis, enrichment of the processes related to non-coding RNA, ribosome biosynthesis, and metabolism was observed in the induced gene set. On the other side, a few GO biological process terms, including biofilm formation and filamentous growth, were enriched in the repressed gene set. Taken together, under butanol stress, *Candida albicans* is unable to extend hyphae and shows growth by budding. Many of the genes with perturbed expression may have fitness or virulence attributes and may provide prospective sites of antifungal targets against *C. albicans.*

## 1. Introduction

In nosocomial settings, opportunistic fungal infections are increasing among immunocompromised or hospitalized patients with serious primary diseases [1,2,3,4,5]. A study suggested that *Candida* spp. caused the majority of nosocomial fungal infections in the United States [4]. In India also, invasive candidiasis is the most common among mycotic infections. *Candida* was the fourth most common causative agent of bloodstream infection [6,7], particularly from intensive care units [6,7,8]. Despite the accessibility of antifungal drugs, the death percentage associated with invasive candidiasis or candidemia remains high [1,4,6].

As the pathogen *Candida albicans* is associated with warm-blooded animals only, it has rapidly evolved its virulence factors and fitness characteristics associated with pathogenicity [9,10]. These alterations have had a major impact on the strategies developed by *C albicans* to stress [11]. 

Fungal determinants that transact directly with host elements are designated as virulence attributes or factors [12]. The common factors of *Candida albicans* causing virulence are the agglutin-like sequence (ALS) gene family members (ALS1 to ALS9), and others that initiate the colonization of host cells, the secreted lipases and proteases that assist in invasion during the yeast-to-hypha transition and those that regulate morphogenesis and biofilm formation [13,14]. Morphological plasticity is argued to be a key virulence regulator. Hyphal stages play a critical part in the disease by escaping from the defense mechanism and supporting tissue penetration [15,16]. The magnitude of the hypha-specific transcription factor Ume6 controls the extent and span of the transcription, characteristics to the hypha [17,18,19]. Hypha-associated G1-type cyclin1 (Hgc1) is responsible for growth at the hyphal tips and cell-chain formation [20,21,22,23,24,25,26,27]. Yeast-to-hypha metamorphosis is initiated by several nutritional and environmental cues, including serum [28], *N*-acetylglucosamine [29], neutral pH [30], high temperature, nutrient starvation [31], hypoxia, CO_2_ [32], and adherence [33]. Various strong hypha-inducing signals are recognized and sensed at the adenylyl cyclase Cyr1, which is essential for the hypha extension under all conditions that induce hyphal growth [34,35,36,37]. Virulence is related to hyphal growth, as genes that control hyphal development coincide with genes related to virulence factors [38,39].

Other factors that contribute to the virulence of *C. albicans* in the absence of direct interaction with the host are defined as fitness attributes [40]. These attributes include functions that improve the physiological fitness of *C. albicans* through metabolic and stress adaptation in the microenvironment [41]. Several studies have underlined the importance of stress adaptation for *C. albicans*. These include genome-wide expression profiles that have demonstrated the niche-specific induction of stress genes [42,43,44]. Previous work has displayed that, in *Candida albicans*, a core stress response comprised a set of about 25 genes [45]. The core stress response is synchronized by Hog1 and Cap1 [45]. In an interesting study, under combined cationic and oxidative stresses, *C. albicans* was killed [46]. The study of stress resistance or susceptibility is important, as adaptation to stress contributes to the virulence of *Candida albicans.* Thus, investigations of stress adaptation or susceptibility are significant, as they can unveil sites of vulnerability in *C. albicans* that could help in the development of novel antifungal therapies by providing novel targets.

Intriguingly, in the presence of alcoholic stress, *Candida albicans* failed to produce hyphae under favorable conditions like the presence of serum, a temperature of 37 °C, and CO_2_ [47,48]. Thus, butanol, being a stressor with the ability to inhibit hypha formation, was selected for the present study. There has been no such report on the gene expression pattern demonstrated by *Candida albicans* during growth when butanol is added in the medium. Therefore, we aimed to analyze the morphology, growth kinetics, and gene expression signatures during the growth of *C. albicans* in the presence of whole human serum with butanol, which may provide the putative targets for antifungal therapy.

## 2. Results

### 2.1. Inhibition of Hypha Formation by Butanol

**Figure 1:**
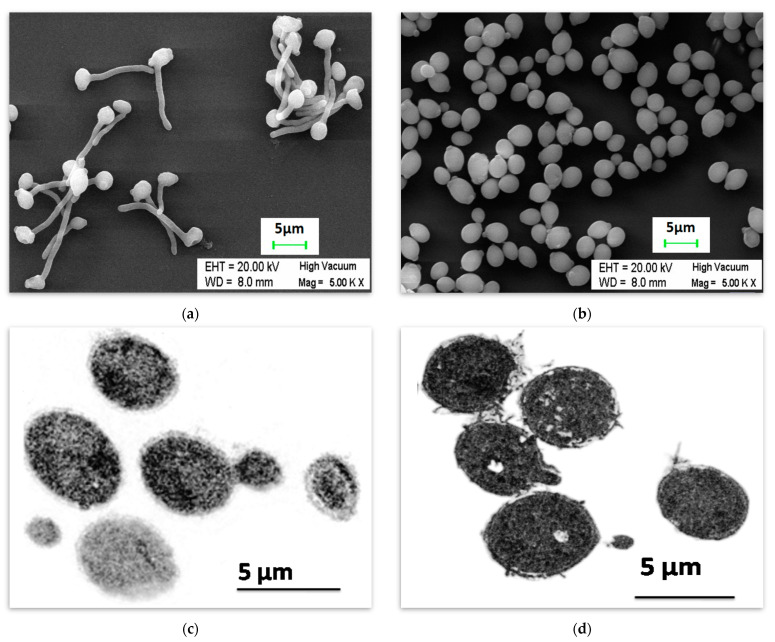
*Candida albicans* cells were observed using a scanning electron microscope, when the overnight grown cultures of *C. albicans* were incubated at 37 °C in 5% CO_2_ for 120 min in (**a**) the whole human serum resulting in true hypha formation and (**b**) whole human serum with butanol (1%) showing yeast-like cells. The cells were also viewed under the transmission electron microscope when the *Candida albicans* was incubated at 37 °C for 60 min in (**c**) the serum with butanol (1%) and (**d**) YPD medium.

**Figure 2:**
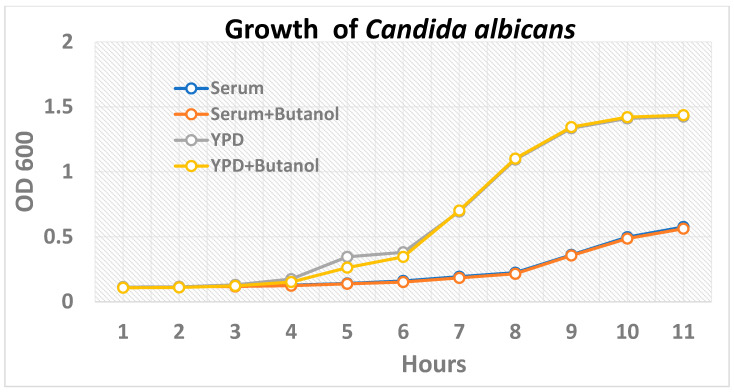
For the growth kinetics study, overnight grown cultures of *Candida albicans* were incubated at 37 °C for 10 h in (i) the whole human serum (blue line), (ii) whole human serum with butanol (1% *v*/*v*) (red line), (iii) YPD medium (grey line), and (iv) YPD with butanol (1% *v*/*v*) (yellow line).

Whole O^+ve^ human serum (hereafter serum) with butanol was selected as the medium for the inhibition of hyphae formation in *Candida albicans*. The yeast cells formed wild-type/true hyphae when the *C. albicans* cultures grown for 16–18 h (overnight) were placed in the serum and incubated at 37 °C with 5% CO_2_ (Figure 1a). *Candida albicans* grown in the presence of serum with butanol demonstrated the inhibition of hyphae formation when observed under a scanning electron microscope (Figure 1b). A similar morphology was exhibited by *Candida albicans* when grown either in serum with butanol or YPD alone (Figure 1c,d). The growth kinetics study up to 10 h showed similar growth whether butanol was added to the medium or not. (Figure 2).

### 2.2. Differential Gene Expression Pattern of C. albicans in Serum and the Serum with Butanol

**Figure 3:**
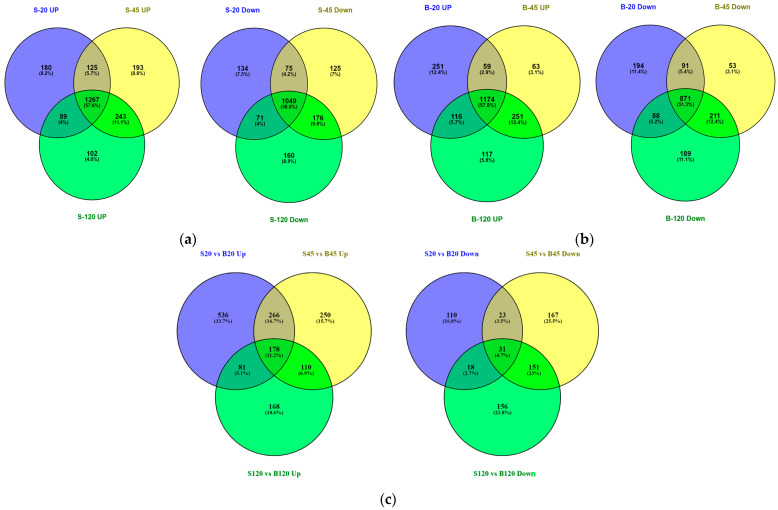
The number of genes up and downregulated in *Candida albicans* when placed in either whole human serum alone or in serum with butanol (1% *v*/*v*) and incubated at 37 °C with 5% CO_2_. RNA were extracted at three time points and subjected to mRNA sequencing using Illumina Genome Analyzer II. The genes showing a fold change of ≥2 and *p* ≤ 0.05 at 20 min, 45 min, and 120 min in comparison to the control sample (0 min) were considered. (**a**) The number of induced and repressed genes in the whole human serum. (**b**) The number of induced and downregulated genes in whole human serum with butanol. (**c**) The comparative analysis using the RPKM values of serum and serum with butanol samples showed upregulation of numerous genes. (S = Serum, UP = Upregulated, Down = Downregulated, 20 = 20 min, 45 = 45 min, and 120 = 120 min).

*Candida albicans* showed differential gene expression patterns in the serum and the serum with butanol at 20 min, 45 min, and 120 min. A genome-wide transcriptomic study was conducted to investigate the sets of genes reprogrammed in response to the butanol. Excellent, high-coverage transcripts information with ~5 million sequencing reads per sample exclusively mapped to ~6453 transcripts, corresponding to open-reading frames, was obtained. The dataset has been deposited at the Gene Expression Omnibus (GEO) with Accession No. GSE168619 (whole human serum) and GSE168874 (whole human serum with butanol). The numbers of genes induced in the serum with a fold change of ≥2 and *p* ≤ 0.05 were 1661 (20 min), 1828 (45 min), and 1701 (120 min) when compared to the control sample (0 min). Altogether, 1267 genes were commonly induced and 1049 genes were commonly inhibited in the serum samples (Figure 3a). On the other hand, the numbers of genes induced in the serum with butanol having fold change of ≥2 and *p* ≤ 0.05 were 1600 (20 min), 1547 (45 min), and 1658 (120 min) in comparison to the control sample. Taken together, 1174 genes were commonly upregulated at all the time points and 871 genes were repressed in the serum with butanol (Figure 3b, Table S1). With a further comparative analysis using the RPKM values of the serum and the serum with butanol, a butanol-specific gene expression network was found. On the whole, 1061 genes (20 min), 804 genes (45 min), and 537 genes (120 min) were upregulated, whereas only 182 genes (20 min), 372 (45 min), and 356 (120 min) were downregulated in the medium with butanol (Figure 3c). The number of commonly upregulated genes was 178, however, the number of commonly downregulated genes was reduced to 31 only (Figure 3c).

### 2.3. Altered Gene Expression Signatures of Many Hyphae-Associated Transcription Factors Repressors and Other Genes in Serum with Butanol Compared to the Serum

Since butanol prevented true hypha formation, the perturbation in the gene expression of transcription factors specific to the hyphal stage was sought. The search identified several genes which demonstrated altered expression compared to the gene expression pattern in the serum alone. Among these, six genes showed a decreased expression, while four repressors/regulators: NRG1, FGR17, SFL1, and FCR1, exhibited induction in the presence of butanol (Table 1). Many hyphae-specific genes also showed altered gene expression in the medium with butanol in comparison to the serum. Among those prominent hypha-specific genes were HWP1 (hypha cell wall protein), ECE1, SAP5, HYR1, RBT1, SOD5, SAP6, the hypha-specific cyclin HGC1, and GPR1, the cAMP/PKA pathway gene (Table 1). The adhesion (virulence factor co-expressed with hypha)-associated transcription factors like AHR1 and RFX2, along with many adhesion-related genes, were also repressed in the presence of butanol (Table 1). The detailed gene expression pattern of the serum versus the serum with butanol samples is presented in Table S2.

In the butanol-treated samples, only four transcription factors, RLM1, SUC1, AHR1, and C2_00640W_A, showed decreased expression compared to the serum (Table 2). Twelve other TFs showed induction when compared to the serum (Table 2). Among the heat-shock-related genes, three exhibited induced expression (Table 2). Among fungal-specific genes, nine showed upregulation, including MET16, FLU1, and C1_13130C_A (Table 2).

### 2.4. Gene Ontology (GO) Analysis of Comparative Gene Expression Data Obtained Using RPKM Values of Serum and Serum with Butanol Indicated Induction and Repression of Specific Processes in Presence of Butanol

The gene ontology processes were obtained using the induced and repressed set of genes found in the comparative analysis of the serum versus the serum with butanol gene expression data. In the GO processes analysis with the induced genes, 126 processes were enriched at 20 min with a *p* value of less than 0.05. However, at 45 and 120 min, altogether, 98 and 51 biological processes were enriched, respectively (Table S3). With the repressed genes obtained in the comparative analysis; 8, 41, and 58 GO processes were mapped at 20, 45, and 120 min, respectively.

**Figure 4:**
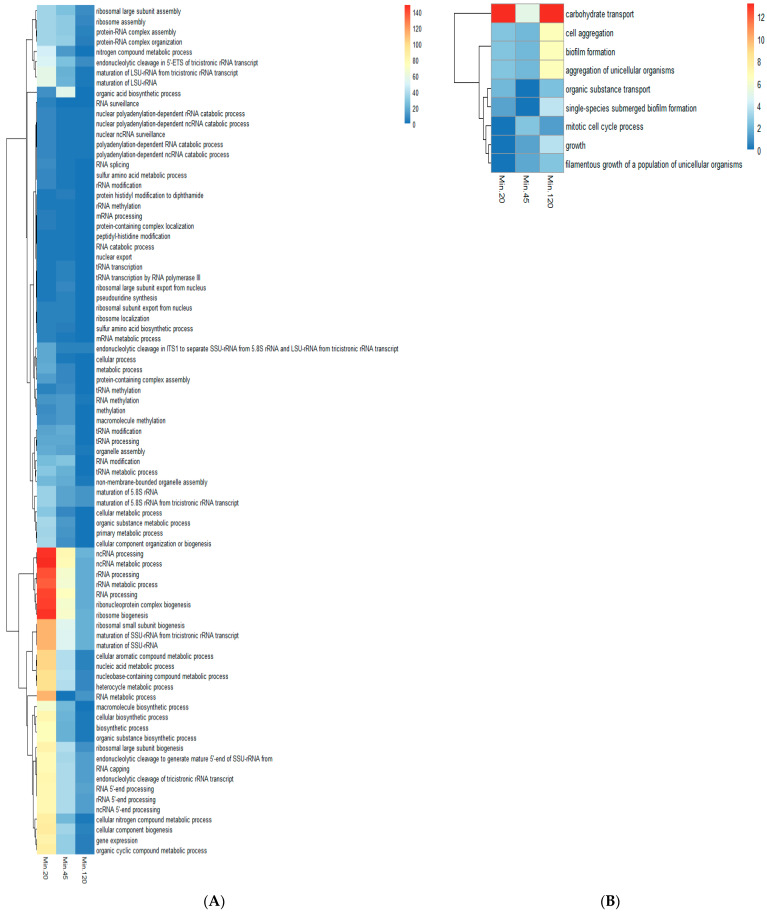
The hierarchical clustering of Gene Ontology Biological Process terms of the genes enriched in the genes set obtained during comparative analysis of the gene expression data of serum and serum with butanol samples. Only GO terms with *p* value less than 0.05 at at least two time points (20, 45, and 120 minutes) were considered in the heat map. (**A**) The GO biological processes enriched with the upregulated set of genes. (**B**) GO biological processes mapped with repressed gene set. The heat map color was coded based on corrected *p* values. (Min denotes minutes; 20, 45, and 120 denote times in minutes at which RNA seq was performed).

Upon close examination, it was found that, with the induced gene set, the biological processes pertaining to non-coding RNA and ribosome biosynthesis were mapped at all three time points (Figure 4A). Those GO processes which were enriched at a minimum of two sampling time points were considered further in the study. The other GO processes, like the cellular nitrogen compound metabolic process, organic cyclic compound metabolic process, organic substance biosynthetic process, macromolecule biosynthetic process, nitrogen compound metabolic process, organic substance metabolic process, and peptidyl-histidine modification, were also observed in the medium with butanol when compared to the serum gene expression (Figure 4A).

With the inhibited or repressed gene set, nine GO biological processes were enriched. The biological processes related to biofilm formation and the filamentous growth of a population of unicellular organisms was enriched with about 25 and 50 genes, respectively (Figure 4B). Intriguingly, the filamentous growth biological process with 66 downregulated genes was observed only at 120 min (Table S2). Further, two processes related to carbohydrate transport and organic substance transport were also enriched in the repressed gene set (Figure 4B).

### 2.5. Unique Stress Associated Transcripts in Serum with Butanol Compared to Serum

The comparative gene expression in the serum with butanol compared to the serum was investigated. In the description of differentially regulated genes, the term “stress” was searched. Altogether, 76 genes were found to be upregulated in the butanol-treated serum samples. However, 30 genes with the description “repressed in core stress response” were found induced in the presence of butanol (Table S4). The transcription factors associated with stress, like RPN4, CTA8, CTA4, and CR_02510W_A, showed induced expression in the presence of butanol. On the other hand, the transcription factor HOG1 was induced in both the serum and serum with butanol when compared to the 0 minute control. The expression of the CAP1 transcription regulator was downregulated in both the serum and serum with butanol samples (Table S1). The gene expression values of the genes which have been reported to be upregulated in stress conditions [45,49,50,51] were compared to the gene expression obtained in the present study. The regulation of the genes in the present study differed from that reported earlier, suggesting that the serum and serum with butanol stress were unique among all the four sets of stress conditions reported (Table 3).

### 2.6. RNA Seq Gene-Expression Data and qRT-PCR Data Correlated with Each Other

The gene expressions were quantified following the 2^−ΔΔCt^ method. We had altogether selected six upregulated and four downregulated genes observed in the RNA seq data of the 120 minute serum sample. The genes showed similar levels of expression when compared to the RNA-seq data of the serum samples (Table 4). Genes showing upregulation in the NGS analysis (ALS3, ECE1, HWP1, SAP5, and UME6) also exhibited induced expression in the qRT-PCR results. Similarly, genes showing downregulation in the RNA-sequencing (RME1, YWP1, C4_05010W_A, and C5_03440W_A) showed decreased expression in the qRT-PCR analysis when compared to the levels of the control (Table 3). Both the approaches to the quantification of gene expression thus indicated the robustness of the methodologies and protocols used for the analysis of the gene expressions.

## 3. Discussion

There is a lack of information regarding the impact of butanol on the gene expression in *Candida albicans*. In this study, we investigated the effect of butanol on the growth and gene expression of *Candida albicans* in O^+ve^ whole human serum. The morphological study in the present work indicated that the *Candida* cells remained in yeast form when butanol was added to the serum, which was the sole growth medium. Earlier studies have also reported yeast-like growth in response to alcohols, including butanol [47,48]. In a study, attachment to tissue culture dishes and polystyrene dishes was shown to be reduced by butanol in *Pseudomonas* species [52]. The growth kinetics study indicated the normal growth of *C. albicans* in yeast form when butanol was added to the medium. In the current study, once the inhibition of hyphae formation in the presence of butanol was confirmed by microscopy, gene expression reprogramming was studied at 20, 45, and 120 min.

### 3.1. Hyphae Transcripts under the Influence of Butanol

The gene expressions were found to be significantly induced when the RPKM values of the serum samples were compared to the serum with butanol samples. The number of genes showing upregulation was more than 500 at all three sampling time points. The presence of butanol resulted in the abolishment of true hypha formation and, altogether, six transcription factors related to hypha extension were repressed. The downregulation of UME6, the master regulator that controls the magnitude and duration of hypha-specific transcription [17,18,19], suggested that butanol suppressed the signaling events that occurred during the growth in the serum. The repression of the transcription factor Cph1, a positive regulator of filamentation [53,54], suggested the inactivation of MAPK-pathway-induced hyphal development. Another transcription factor, SFL2, was also repressed in the medium with butanol. The filamentation is additionally regulated through the transcription factor Sfl2 that senses elevated temperature [55]. IRO1 has been shown to be induced during hypha formation and IRO1 expression was inhibited in the presence of butanol [56]. TEC1, which regulates hyphal development [57], was downregulated in the presence of butanol. The repression of UME6, CPH1, SFL2, IRO1, and TEC1 in the present study indicated an unknown mechanism by which butanol target the expressions of these factors, leading to the inhibition of hypha formation.

Four genes, NRG1, FGR17, FCR1, and SFL1, which are negative regulators of filamentation, exhibited increased expression in the presence of butanol. FRG17 is a filamentous growth regulator and a homozygous null mutant of FGR17,, showed increased filamentation [55]. NRG1 is a DNA binding protein that represses filamentous growth in *C. albicans* and hyphal growth is induced by the inhibition of NRG1 [58,59]. FCR1 is a transcription factor/repressor that represses fluconazole/ketoconazole/brefeldin A resistance, and it also negatively regulates hyphal development [60,61]. Further, SFL1 is a transcription factor involved in the negative regulation of morphogenesis, flocculation, and virulence, which suppresses hyphal growth [62]. Altogether, the upregulation of all the above four genes in the presence of butanol indicated a hitherto unknown mechanism through which butanol suppresses hyphal growth.

The hyphal-specific G1 cyclin Hgc1 showed repression in the presence of butanol. HGC1 plays a critical role in hyphal morphogenesis and is positively regulated by the transcription factor Ume6. HGC1 is also accountable for specific growth at the hyphal tips and the formation of the chain of cells [20,21,22,23,24,25,26,27]. In addition, many hypha-specific genes were repressed in the presence of butanol. Among those prominent were HWP1, ECE1, SAP5, HYR1, RBT1, SOD5, SAP6, GPR1, and HGC1. On the whole, the downregulation of the hypha-specific transcription factors and hypha-specific genes, along with the upregulation of the repressors/regulators of filamentous growth, indicated that butanol reprogrammed *Candida albicans* cells, resulting in the abrogation of hypha formation.

The yeast-to-hypha transformation is activated by numerous nutritional and environmental signals, including serum [28]. However, the hypha-associated gene expression pattern suggested that, in the presence of butanol, *Candida* appears not to sense the hyphal cues present in serum.

Many of the adhesion-related genes were either downregulated or showed no significant difference in expression. Among them, AHR1, which is the Zn(II)2Cys6 transcription factor responsible for the modulation of adhesion genes [63], was also repressed, suggesting an overall decrease in the expression of adhesion-associated genes. Agglutinin-Like Sequence (ALS) genes code a group of cell surface glycoproteins known as adhesins which are linked to glycosylphosphatidylinositol (GPI). ALS3 has a role in cell wall adhesion, epithelial adhesion, endothelial invasion, and immunoprotection [64]. The repression of ALS3 and other adhesion-associated genes suggested the absence of adhesion to the surfaces, as evident also by the planktonic state of the yeast cells in the medium with butanol.

During the growth of *Candida albicans* in the serum with butanol, many transcription factors showed induced expression. Among those, one of the transcription factors was RPN4. RPN4 is a C2H2 transcription factor regulating proteasome genes and has been shown to be induced in the core stress response [45,65]. Another transcription factor upregulated in the butanol was NDT80, which activates CDR1 induction during antifungal drug treatment and has been shown to be induced by antifungal drugs [66]. Taken together, the induction of these two TFs indicated that butanol acted as a stressor for *Candida albicans*.

Among the fungal-specific genes, the upregulation of FLU1, a multidrug efflux pump of the plasma membrane [67], and C3_03070W_A, putative transporter similar to MDR proteins, was observed [68]. The upregulation of these two fungal-specific genes indicated that butanol acts as a stressor for *Candida albicans*, which, in turn, induced the genes to efflux the butanol. Along with the above two genes, other fungal-specific genes related to metabolism like MET16, sulfur amino acid metabolism [69]; HOM3, putative L-aspartate 4-P-transferase [70]; C1_13130C_A, putative histidine permease [71]; LEU42, putative alpha-isopropyl malate synthase [72]; amd DUR4, putative urea permease [73] were also induced. The above genes thus may act as virulence factors or fitness attributes and may be important in tolerating butanol stress. On the other hand, many of the fungal-specific genes such as ATO1, coding for putative fungal-specific transmembrane protein [74]; OPT6, putative oligopeptide transporter [75]; and PLB5 and PLB3, putative GPI-linked phospholipase B [76,77] were downregulated in the serum growth medium with butanol. Overall, these genes behaving like virulence or fitness attributes may act as prospective antifungal targets.

In the presence of butanol heat shock genes, C7_01360C_A, CTA8, and SSB1 were upregulated. The behavior of the cells was akin to a temperature rise or heat shock in the presence of butanol [78]. The microbes responded to solvents by upregulating the heat shock proteins [79]. The heat shock proteins mentioned above thus may act to stabilize the proteins in the presence of butanol and therefore may be important as fitness attributes or as virulence factors. Therefore, these heat shock genes/proteins may be important drug targets.

### 3.2. Induction and Repression of Specific GO Processes in the Serum with Butanol

Gene Ontology furnishes a framework and set of concepts for delineating the functions of gene products from organisms. The process or biological process terms in GO represent a particular objective that *Candida* is genetically programmed to attain.

Many of the GO biological processes were enriched in the set of genes which were found to be significantly induced in the comparative analysis of the serum and serum with butanol samples. The majority of the GO biological processes induced in *Candida* when placed in the medium with butanol belonged to two groups. The first major group of GO terms belonged to the genesis of non-coding RNA, ribosomal units, and their exodus from the nucleus (Figure 4). The other group of GO biological processes were related to the metabolic process, including the primary metabolic process, nitrogen compound metabolic process, organic substance metabolic process, cellular metabolic process, metabolic process, and macromolecule biosynthetic process, etc. Overall, with the biogenesis of ribosome and their transport to the cytoplasm, along with the activation of metabolic processes, suggested that the protein/other macromolecule metabolism has a role in overcoming butanol stress. In the case of *Saccharomyces cerevisiae*, protein degradation was observed as the major factor for butanol tolerance [80]. *Candida* cells thus appeared to promote the mechanisms for synthesizing the protein content to restore the damaged protein repertoire. The enrichment of the macromolecule biosynthetic biological process was also observed in the present study. It has been shown that the exposure of butanol to the membrane bilayer led to a more extensive disruption than to ethanol [81]. Thus, under butanol stress, the cell wall alteration/disruption might have resulted in the upregulation of the process.

The gene ontology analysis using repressed genes demonstrated the inhibition of several specific biological processes, including carbohydrate transport and organic substance transport. Butanol has been shown to interfere with the active uptake of glucose and other nutrients [78].

Other biological processes, including biofilm formation and single-species submerged biofilm formation observed in the the GO analysis with repressed genes, suggested the obstruction of adhesion and biofilm formation in the medium with butanol. Intriguingly, in *Candida albicans*, alcohol dehydrogenase are repressed in the biofilm [82]. The gene IFE1 (putative medium-chain alcohol dehydrogenase) was induced in the presence of butanol. NADPH-dependent alcohol dehydrogenase activity has been shown to increase during the growth of *Candida utilis* in the presence of alcohol and nitrate/ammonia [83]. Serum with nitrate and ammonia along with the addition of butanol may have caused the upregulation of alcohol dehydrogenase, which, in turn, resulted in the inhibition of biofilm formation. Lastly, the enrichment of biological processes among the repressed genes, such as the filamentous growth of a population of unicellular organisms and filamentous growth at 120 min, indicated the inhibition of hypha formation, which was also evident during the morphological observations in the present study.

### 3.3. Stress Response of Candida albicans in the Serum with Butanol

The differentially expressed stress-responsive genes in the serum with butanol indicated a unique stress response, in which more than 75 genes were upregulated (Table S4). In earlier studies with osmotic and oxidative stresses, the upregulation of 44 genes with a 1.5 or more fold change was observed [45,49]. In the serum with butanol medium, out of those genes, the upregulation of fourteen genes and downregulation of six genes were observed (Table 3). However, 20 of those genes showed a similar expression in the serum and serum with butanol, suggesting that the serum present in both the mediums may have resulted in certain stress signaling. Gene MLS1 coding for the malate synthase that has no mammalian homologue [84] initially showed downregulation, followed by induction at two hours. Another fungal-specific gene, CR_03710C_A, reported to be induced by nitric oxide or oxidative stress [85], was also induced in the present study. These two genes with no mammalian homologue could be regarded as potential antifungal target. In the next group, a combination of oxidative and heavy metal stress has been shown to cause the upregulation of seventeen genes [45,50]. However, in the serum with butanol medium, upregulation of eight genes and the downregulation of a single gene were observed. Six genes, however, showed similar expression patterns in both the serum and serum with butanol. Another set of stress combinations consisting of osmotic stress and heavy metal stress has been reported to cause the upregulation of 18 genes [45,49]. In the present study, the combination of serum and butanol resulted in the upregulation of seven genes and downregulation of two genes out of those eighteen genes. In contrast, eight genes had similar expression patterns in the serum or serum with butanol, indicating that the effect may have occurred due to the presence of serum or its component present in both the media. In the heat shock response, it was shown that, altogether, 23 genes were induced [45,50,51]. Out of those 23 genes, 10 genes showed upregulated expression in the serum with butanol medium and 2 showed inhibition in the same medium. Out of the remaining eleven genes, nine showed similar expressions in the serum or serum with butanol, indicating the signaling event commonly present in both mediums.

Intriguingly, the genes expressed in all three stress conditions (osmotic, oxidative, and heavy metal) were proposed as the core stress response genes and comprised roughly 25 genes [45]. Among those core genes, AHP1, CDR4, MRF1, CAT1 (CTA1), HGT8, NRG1, and RPN4 were induced in butanol stress compared to the serum alone condition (Table 2 and Table S2). Another set of genes, G-K1, C1_11270W_A and HGT6, were detected in the presence of butanol stress, but were absent from the serum alone medium (Table S1). Intriguingly, the majority of the core stress genes; TPS2, PHO15, DAP1, CRP1, GRP2, ZWF1, C6_02480W_A, ECM4, GLX3, GPD2, and NPR1, exhibited similar expression patterns in both the serum and serum with butanol medium (Table S1).

The difference in the expression patterns of the core stress genes in the present study compared to the earlier study could have been due to the difference in the agent used as stressor. In a previous study, core stress gene signature was reported for three stresses: osmotic, cadmium, and oxidative stress [45], whereas, in the present study, butanol and serum were used as stressors. In conclusion, it may be safely stated that, under the butanol stress, ten genes (AHP1, CDR4, MRF1, CAT1, RPN4, HGT8, NRG1, GLK1, C1_11270W_A, and HGT6) may be considered as core gene signatures of *Candida albicans*. One of these genes, C1_11270W_A is a cell wall protein that has been reported to be regulated by iron and induced by amphotericin B, ketoconazole, and hypoxia [86,87,88] and has no significant homology to human proteins. The other gene, HGT6, is a putative high-affinity MFS glucose transporter, which has been previously reported to be induced in core stress response or by fluconazole [89,90], and also does not show any significant homology to human proteins. Thus, these two proteins may be regarded as fitness attributes and may be considered as potential antifungal targets.

The majority of the GO terms enriched in the upregulated set of genes in the serum with butanol were related to ribosome biosynthesis. A similar induction of GO process terms associated with ribosome biogenesis has been reported in the presence of arachidonic acid [91]. In *Candida albicans*, the process of ribosome biogenesis is downregulated under the stress due to reactive oxygen species and a limited nutrient supply [92]. Furthermore, in the presence of hypha inducers like serum, a reduction in ribosomal RNA has been reported [93]. Therefore, it may be concluded that, since butanol inhibited yeast to hyphae transition in *C. albicans*, the opposite effect of an increase in rRNA and ribosome-biogenesis-related GO terms was observed.

## 4. Materials and Methods

### 4.1. Strains and Culture Conditions

*Candida albicans* strains isolated from patients (NCCPF 400034, NCCPF 400039, and NCCPF 400040) were obtained from the National Culture Collection of Pathogenic Fungi (NCCPF), [NCCPF Core Facility, Chandigarh, India, RRID:SCR_018954]. Yeast was grown in Sabouraud Dextrose Agar Yeast (Dextrose—40 g, peptone—10 g, yeast extract—10 g, and agar—20 g per litre) medium, and glycerol stock of the cultures was cryopreserved at −80 °C for further use.

### 4.2. Selection of Strain and Preparation of Inoculum

The strains 400034 and 400039 killed BALB/c mice within 4 days post-infection when treated with an intravenous dose of 5 × 10^5^ Colony-Forming Units (CFU). However, the third strain 400040 could kill the mice only after 10 days post-infection. The strain 400034 was selected for further studies as a representative of pathogenic strains. YPD-grown yeast cells were used for inoculum preparation, as described earlier [94], with minor modification. Briefly, the yeast cells were grown overnight (16–18 h) in YPD medium at 37 °C with shaking at 150 RPM. The cells were washed at least two times with sterilized PBS and then re-suspended in PBS. A haemocytometer was used for counting, and, finally, a suspension of 1 × 10^8^ Cells/mL was prepared for further study.

### 4.3. Microscopy for the Determination of Inhibition of Hyphae Formation

#### 4.3.1. Visualization of *Candida albicans* under Scanning Electron Microscope

O^+ve^ whole human serum sterilized by filtration (0.22 µm filters) was selected as the medium for the induction of hypha and butanol (1% *v*/*v*) for the inhibition of hypha formation. Yeast cells were placed in six-well tissue culture plates (Corning, NY, USA) containing either serum or serum with butanol and incubated at 37 °C with 5% CO_2_. Scanning Electron Microscopy (SEM) was performed as per the standard protocol described earlier [95], with a modification. The incubation for 18 h post-treatment with 2.5% glutaraldehyde was completed at 4 °C instead of 8 °C. The prepared samples were visualized using a Carl Zeiss Scanning Electron Microscope (Model EVO LS10), Oberkochen, Germany with a Lanthanum hexaboride (LaB6) emitting cathode and Zeiss SmartSEM software, Version 5.06.

#### 4.3.2. Transmission Electron Microscopy for the Observation of Morphology of *Candida albicans* under the Influence of Butanol

The transmission electron microscopy was performed as per the protocol described elsewhere for the yeast [96], with modifications. The *Candida* cells grown in YPD or the serum with butanol were prefixed with fixative containing 4% formaldehyde and 0.5% glutaraldehyde in PIPES buffer for 30 min at 4 °C. Post-fixation was performed with 2% KMnO_4_ solution. The en bloc staining was performed with 1% Uranyl acetate for 1 h. Samples were then dehydrated using gradually increasing ethanol concentrations. In the next step, infiltration and embedding in LR white resin was performed, followed by hardening at 45 °C. Sections in the range of 80–150 nm were cut and, in the last step, staining with Reynold’s citrate was performed. After air drying, the grids were processed for transmission electron microscopy using the FEI Technai G2-20 system.

### 4.4. Growth Kinetics of Candida albicans in Presence of Butanol

For the growth kinetics study, *Candida albicans* cells were grown overnight in YPD medium. Then, yeast culture was added in either fresh YPD or the serum with or without butanol (1% *v*/*v*), so that the final concentration of *Candida* was 1% (*v*/*v*). The optical density (OD) at 600 nm of the culture grown at 37 °C under stationary conditions was recorded with a Nanoquant M200 (Tecan) spectrophotometer. Six-well culture plates were used for culturing the yeast cells. The cultures were mixed properly before the OD measurement.

### 4.5. Extraction of Total RNA

A Ribopure Yeast Kit (Ambion, Austin, TX, USA) was utilized for the extraction of RNA at 0, 20, 45, and 120 min. The inoculum prepared as above in YPD was taken as the 0 min sample/control. The total RNA was extracted from the *Candida* cells grown in the serum, serum with butanol, and in the YPD (the control). For the isolation of RNA, the yeast cells were centrifuged at 6000 RPM and the procedures described in the kit manual were followed. The total RNA in the samples was estimated using the Nanodrop spectrophotometer and the RNA 6000 nano kit was used for the estimation of the RNA integration numbers of the samples on an Agilent 2100 Bioanalyzer.

### 4.6. RNA Library Preparation, Sequencing, and Data Analyses

For mRNA libraries, seven *Candida albicans* samples, three each from cells grown either in the serum or the serum with butanol, along with zero minutes, was processed. Two biological replicates at each time point and 0 minutes were sequenced on the Illumina Genome analyzer II. The libraries of mRNA for each sample were prepared as per the IlluminaTrueseq RNA sample preparation kit V2 using a low-throughput protocol [97]. The sequencing of the libraries was executed for 34 cycles using *GA II_x_* of Next Generation Sequencing Facility at the National Institute of Immunology. CLC Genomic Workbench 6.5.1 [98] with default settings was implied for the independent alignment of the sequence reads against the *Candida albicans* genome assembly 21 obtained from *the Candida* genome database (*Candida* Genome Database, RRID:SCR_002036). The differential expression values, *p*-values, and fold changes for each gene were also obtained using CLC Genomic Workbench 6.5.1 working at default parameters. A Gene Ontology (GO) enrichment analysis for biological processes was performed with the *Candida* genome database Gene Ontology Term Finder [99]. Other statistical analyses were performed in Microsoft Excel 2007 or another tool, as indicated in the figure legends. For the comparison, the mRNA gene expression data of the samples grown in the serum were utilized. The R program [100] was used for the generation of heatmaps using the *p* values associated with the GO biological process terms. The GO terms not found at any sampling time point were given a value of 1, the log10 values of *p* were quantified using Excel, and then negatives of log10 values were utilized in generating hierarchical heatmaps.

### 4.7. Real-Time PCR Analysis of Gene Expression

Cultures were set up as identical to those used for RNA sequencing and RNA was extracted for quantitative reverse transcription-PCR (qRT-PCR). The total RNA for qRT-PCR was isolated using theRibopure Yeast Kit (Ambion, TX, USA), which included DNAse treatment. The synthesis of cDNA was carried out using the iScript cDNA synthesis kit (Bio-Rad, CA, USA). Primers were designed using the Primer BLAST of NCBI and are listed in Table S5. For the real-time detection of amplicons, the GoTaq^®^ qPCR Master Mix (Promega, WI, USA) was used and the reaction was run on an Eppendorf Mastercycler Realplex4 system. Separate reactions were performed for each gene. Gene expression levels were normalized against the expression levels of the WOR3 gene, which did not show any significant variation throughout the RNA sequencing data obtained in our study. Only the control (0 min) and 120 min samples were used for the q-RT-PCR analysis.

## 5. Conclusions

Taken together, under the butanol stress, *Candida albicans* is unable to extend hyphae and showed growth while remaining in yeast form. The downregulation of hyphae-associated genes suggested that, in the presence of butanol, the formation of hypha/filaments, which is an important virulence factor, is abrogated. Further repression of many adhesion-associated genes and the enrichment of Gene Ontology biological processes related to biofilm formation (in the repressed gene set) indicated the non-adherence of *Candida albicans* to the surface. The upregulation of certain heat shock genes and the ten core stress genes suggested a distinct stress response due to butanol. The gene ontology analysis exhibited the enrichment of processes related to ncRNA metabolism, ribosome biosynthesis, and metabolism, suggesting the impact of butanol on the protein and cell wall. The enrichment of the GO processes observed with the repressed gene set was related to the transport of carbohydrates and other organic molecule, suggesting that butanol hampered the absorption of glucose and other organic nutrients. Intriguingly, the GO process associated with filamentous growth was also enriched in the repressed set of genes, validating the absence of hypha formation/filamentation in the presence of butanol, which was also evident with microscopy. Further, many fungal-specific genes related to multidrug efflux pump, metabolism, and transport were induced in the *Candida* cells treated with butanol. On the other hand, fungal-specific genes for transmembrane protein, transport, and phospholipase were inhibited in the medium with butanol.

The current study performed under butanol stress may thus provide sites for antifungal targets against *C. albicans*, including fungal-specific genes FLU1 (multidrug efflux pump of the plasma membrane), C3_03070W_A (transporter similar to MDR proteins), MET16 (amino acid metabolism), LEU42 (alpha-isopropyl malate synthase), HOM3 (L-aspartate 4-P-transferase), C1_13130C_A (putative histidine permease), DUR4 (putative urea permease), ATO1 (putative fungal-specific transmembrane protein), PLB5 (Putative GPI-linked phospholipase), PLB3 (GPI-anchored cell surface phospholipase B), C1_13130C_A (Putative histidine permease), OPT6 (Putative oligopeptide transporter), and the stress genes MLS1 (malate synthase), CR_03710C_A, C1_11270W_A, and HGT6.

## Figures and Tables

**Table 1:** The relative expression of hyphae-related genes, adhesion-specific transcription factors (TFs), and genes when *Candida albicans* was placed in either serum with butanol or serum alone and incubated at 37 °C in 5% CO_2_. NS = Not significant (less than 2-fold change or a *p* value of more than 0.05).

Descriptions	Fold Change in Serum with Butanol Medium Compared to the Serum Alone Medium							
	Gene Name/ID	20 min	45 min	120 min	Gene Name/ID	20 min	45 min	120 min
Hypha-Specific TFs/ Repressor	UME6	−677.49	−264.33	−75.17	NRG1	2.45	8.44	11.72
	CSR1	−5.79	NS	−2.52	FGR17	NS	3.7	4.06
	IRO1	−4.55	−8.58	−6.38	FCR1	NS	3.56	8.41
	SFL2	−2.45	−4.19	NS	SFL1	NS	2.93	2.84
	CPH1	NS	−5.8	−4.54	TEC1	−2.98	−3.62	NS
Hypha-Specific Genes	ECE1	NS	−1800.63	−1732.65	SHE3	NS	−4.4	−4.03
	HWP1	−1265.6	−2361.3	−528.71	RSR1	NS	−4.65	−6.12
	SAP5	NS	−511.46	−425.61	CLA4	NS	−6.32	−6.43
	CDC11	NS	−2.62	−2.83	C1_06250W_A	NS	−7.71	−3.07
	HYR1	NS	−38.73	−22.14	DCK1	NS	−2.97	−4.02
	CDC10	NS	−2.5	−2.28	SOD5	NS	−51.19	−52.42
	IHD1	−42.14	−63.83	−145.49	GPR1	−7.84	−3.23	−3.78
	C6_02330W_A	−4.86	−17.36	−31.74	SAP6	NS	−418.36	−222.69
	RBT1	−27.61	−57.1	NS	C7_03480W_A	−2.29	−6.01	−7.15
	RAX2	NS	−2.15	−3.04	TUB2	NS	−3.28	−3.92
	HGC1	NS	−10.23	−10	PHR1	−2.05	−2.46	−3.12
	PGA54	NS	−7.49	−4.25	CHS2	−5.34	NS	−2.25
	RAS1	−3.77	−4.87	−3.4	AXL2	NS	−9.14	−7.88
	PGA56	NS	−2.88	−3.73	C4_01860C_A	−2.7	−3.44	−2.87
	PST1	NS	−3.93	−2.36	PTP3	−4.01	NS	−3.5
	RBT4	NS	−3.76	−4.77	SUN41	NS	−4.49	−3.57
	PHO13	NS	−3.50	−7.07	SHE3	NS	−4.40	−4.03
	SNG4	NS	−3.37	−2.49	MYO2	NS	−2.60	−2.39
	IHD2	NS	−3.14	−3.6	CAS4	NS	−2.49	−2.14
	RHO3	NS	−2.19	−2.4	MRP8	NS	−2.47	−2.12
	KIP4	NS	−2.31	−5.52				
Adhesion-Specific TFs/Repressor	UME6	−677.49	−264.33	−75.17	RFX2	NS	−15.85	−26.78
	AHR1	−3.73	−5.14	−5.68				
Adhesion-Specific Genes	C1_13100W_A	−5.1	−7.06	−12.61	MSB2	NS	−3.14	−2.69
	ALS3	NS	−105.50	−96.27	EAP1	NS	−9.27	−4.88
	ECM33	NS	−2.37	−3.38	PGA59	−2.25	−2.23	NS
	FAV2	NS	−20.73	−18.53				

**Table 2:** The relative expression of other transcription factors (TFs), fungal-specific, and heat shock genes of *Candida albicans* in the serum with butanol compared to the serum alone medium. NS = Not significant (less than 2-fold change or a *p* value more than 0.05).

Descriptions	Fold Changein Serum with Butanol Medium Compared to the Serum Alone Medium							
	Gene Name/ID	20 min	45 min	120 min	Gene Name/ID	20 min	45 min	120 min
Other TFs/Repressor	RLM1	−3.49	−3.33	−4.27	RPN4	2.38	6.15	
	SUC1	NS	−2.44	−2.59	CTA4	2.35	3.78	2.02
	AHR1	−3.73	−5.14	−5.68	ZCF5	NS	4.49	6.02
	C2_00640W_A	NS	−2.15	−3.11	UGA3	NS	3.97	3.15
	ZCF25	5.8	NS	13.8	LYS144	NS	3.03	2.34
	WOR2	5.73	10.45	NS	C4_03160C_A	NS	2.44	3.8
	CTA8	3.25	3.04	NS	NDT80	NS	2.19	2.38
	C3_04860W_A	3.11	4.72	3.81	C2_01870C_A	2.92	2.06	2.58
Fungal-Specific Genes	ATO1	NS	−35.11	−23.01	FLU1	14.33	7.25	NS
	PLB3	NS	−2.51	−2.12	MET16	5.56	2.77	2.33
	PLB5	NS	−4.74	−6.03	HOM3	5.1	NS	5.56
	OPT6	NS	−3.92	−5.75	C1_13130C_A	3.08	4.18	NS
	LSP1	NS	−2.20	−2.13	LEU42	2.77	2.38	3.44
	CR_03710C_A	NS	4.67	2.38	DUR4	2.25	2.84	NS
	RIT1	2.66	2.50	NS	C3_03070W_A	2.05	2.54	NS
Heat-shock Genes	C7_01360C_A	4.72	4.25	NS	SSB1	2.01	2.09	NS
	CTA8	3.25	3.04	NS				

**Table 3:** Expression of the stress-associated genes (reported to be upregulated under different stress conditions) in the present study.

	Serum vs. Serum with Butanol		Expression in Serum vs. Control (0 min) and Serum with Butanol vs. Control (0 min)			Expression in Serum with Butanol vs. Control (0 min)	
**Genes as observed in Osmotic Stress + Oxidative Stress** [45,49]	**Upregulated**	**Downregulated**	**Upregulated in Both**	**Downregulated in Both**	**No Change**	**Upregulated**	**Downregulated**
	IFR2	RHD3	C1_02040C_A	C2_07640W_A		AGP2	CR_01090W_A
	CR_03710C_A	CRG1	GLC3	C7_01430C_A		C3_06490W_A	
	C4_06710W_A	C1_07980C_A	ARE2	MSN4			
	TPO3	CDR11	PST2	C6_03370W_A			
	RAD16	CBP1	HRT2	LPI9			
	C7_01940C_A	GSY1	STV1	C1_06660W_A			
	RHR2		C1_06670W_A	C2_06600W_A			
	C1_07990C_A			C2_04280W_A			
	C6_03320W_A			TPS3			
	CR_02460W_A			C1_14250C_A			
	C3_06860C_A			MHP1			
	C6_02420W_A			PRB1			
	C2_07630C_A			C6_03780C_A			
	AHP1						
	MLS1						
**Genes as observed in heavy metal Stress + Oxidative Stress** [45,50]	OYE32	HSP21	ZRT2	CAP1	C3_03230C_A		
	GST2			OYE23	ZRT2		
	OPT8			TPS2	C1_11530C_A		
	GCS1			HSP78			
	CR_07480W_A						
	LEU42						
	ALP1						
	NRG1						
**Genes as observed in heavy metal Stress + Osmotic Stress** [45,49]	SOU1	RCT1	SRG1	C3_01540W_A			SLK19
	C2_03020C_A	HMX1	ALD5				
	HSP70		ASR1				
	HSP12		C1_00310W_A				
	DLD1		C1_10460W_A				
	PIR1		C4_02160C_A				
	GAD1		CIT1				
**Genes as observed in heat shock Stress** [45,50,51]	MRF1	C7_00350C_A	PHO15	TPS2			HGT6
	RPN4	C1_11270W_A	GLX3	C1_11670W_A			GLK1
	C6_02480W_A		DAP1				
	CAT1		GRP2				
	CDR4		GPD2				
	HGT8		ZWF1				
	NPR1		ECM4				
	RHR2						
	AHP1						
	HSP12						

**Table 4:** Comparison of gene expression values using qRT-PCR and RNA Seq.

Gene	120 min (qRT-PCR) ± SEM *	120 min (RNA Seq)	120 min *p* Value (RNA Seq)
ACT1	1.8 ± 0.08	17.0	4.3 × 10^−36^
ALS3	125 ± 3	215.3	1.3 × 10^−130^
ECE1	26,851 ± 3749	25,907.7	1.0 × 10^−8^
HWP1	1540 ± 288	7404.9	2.0 × 10^−23^
RME1	−98 ± 5	−270.0	6.6 × 10^−59^
SAP5	1669 ± 305	1156.0	7.7 × 10^−5^
UME6	22 ± 2	87.0	6.1 × 10^−7^
YWP1	−129 ± 23	−88.9	1.9 × 10^−34^
C4_05010W_A	−1.6 ± 0.2	−2.1	0.015604
C5_03440W_A	−20 ± 2	−28.4	1.9 × 10^−12^

* SEM = Standard error of mean.

## Data Availability

All the data generated in the present study are included in the main manuscript or in the supplementary files. The high throughput dataset has been deposited at Gene Expression Omnibus (GEO) with Accession No. GSE168619 (Whole Human Serum) and GSE168874 (Whole human serum + Butanol). The other supporting data may be requested from the corresponding author without reservation.

## Supplementary Materials

The following supporting information can be downloaded at: https://www.mdpi.com/article/10.3390/ijms242417227/s1.

## Abbreviations

TF	Transcription Factor

## References

[1] Horn D.L., Neofytos D., Anaissie E.J., Fishman J.A., Steinbach W.J., Olyaei A.J., Marr K.A., Pfaller M.A., Chang C.H., Webster K.M. (2009). Epidemiology and outcomes of candidemia in 2019 patients: Data from the prospective antifungal therapy alliance registry. Clin. Infect. Dis..

[2] Neofytos D., Horn D., Anaissie E., Steinbach W., Olyaei A., Fishman J., Pfaller M., Chang C., Webster K., Marr K. (2009). Epidemiology and outcome of invasive fungal infection in adult hematopoietic stem cell transplant recipients: Analysis of Multicenter Prospective Antifungal Therapy (PATH) Alliance registry. Clin. Infect. Dis..

[3] Pfaller M.A., Diekema D.J. (2007). Epidemiology of invasive candidiasis: A persistent public health problem. Clin. Microbiol. Rev..

[4] Pfaller M.A., Diekema D.J. (2010). Epidemiology of invasive mycoses in North America. Crit. Rev. Microbiol..

[5] Ciurea C.N., Kosovski I.-B., Mare A.D., Toma F., Pintea-Simon I.A., Man A. (2020). *Candida* and Candidiasis—Opportunism versus Pathogenicity: A Review of the Virulence Traits. Microorganisms.

[6] Chakrabarti A., Ghosh A., Batra R., Kaushal A., Roy P., Singh H. (1996). Antifungal susceptibility pattern of non-albicans Candida species & distribution of species isolated from Candidaemia cases over a 5 year period. Indian J. Med. Res..

[7] Chakrabarti A., Mohan B., Shrivastava S.K., Marak R.S.K. (2002). Change in distribution & antifungal susceptibility of Candida species isolated from candidaemia cases in a tertiary care centre during 1996–2000. Indian J. Med. Res..

[8] Verma A.K., Prasad K.N., Singh M., Dixit A.K., Ayyagari A. (2003). Candidaemia in patients of a tertiary health care hospital from north India. Indian J. Med.Res..

[9] Butler G., Rasmussen M.D., Lin M.F., Santos M.A.S., Sakthikumar S., Munro C.A., Rheinbay E., Grabherr M., Forche A., Reedy J.L. (2009). Evolution of pathogenicity and sexual reproduction in eight *Candida* genomes. Nature.

[10] Nikolaou E., Agrafioti I., Stumpf M., Quinn J., Stansfield I., Brown A.J.P. (2009). Phylogenetic diversity of stress signalling pathways in fungi. BMC Evol. Biol..

[11] Brown A.J.P., Haynes K., Gow N.A.R., Quinn J., Calderone R.A., Clancy C.J. (2012). Stress responses in *Candida*. Candida and Candidiasis.

[12] Odds F.C., Calderone R.A., Hube B., Nombela C. (2003). Virulence in *Candida* species: Views and suggestions from a peer-group workshop. ASM News.

[13] Mayer F.L., Wilson D., Hube B. (2013). *Candida albicans* pathogenicity mechanisms. Virulence.

[14] Hoyer L.L., Green C.B., Oh S.-H., Zhao X. (2008). Discovering the secrets of the *Candida albicans* agglutinin-like sequence (ALS) gene family—A sticky pursuit. Med. Mycol..

[15] Dalle F., Wächtler B., L’Ollivier C., Holland G., Bannert N., Wilson D., Labruère C., Bonnin A., Hube B. (2010). Cellular interactions of *Candida albicans* with human oral epithelial cells and enterocytes. Cell. Microbiol..

[16] Lorenz M.C., Bender J.A., Fink G.R. (2004). Transcriptional response of *Candida albicans* upon internalization by macrophages. Eukaryot. Cell.

[17] Banerjee M., Thompson D.S., Lazzell A., Carlisle P.L., Pierce C., Monteagudo C., Lopez-Ribot J.L., Kadosh D. (2008). UME6, a novel filament-specific regulator of *Candida albicans* hyphal extension and virulence. Mol. Biol. Cell..

[18] Zeidler U., Lettner T., Lassnig C., Müller M., Lajko R., Hintner H., Breitenbach M., Bito A. (2009). UME6 is a crucial downstream target of other transcriptional regulators of true hyphal development in *Candida albicans*. FEMS Yeast Res..

[19] Carlisle P.L., Banerjee M., Lazzell A., Monteagudo C., López-Ribot J.L., Kadosh D. (2009). Expression levels of a filament-specific transcriptional regulator are sufficient to determine *Candida albicans* morphology and virulence. Proc. Natl. Acad. Sci. USA.

[20] Zheng X., Wang Y., Wang Y. (2004). Hgc1, a novel hypha-specific G1 cyclin-related protein regulates *Candida albicans* hyphal morphogenesis. EMBO J..

[21] Zheng X.D., Lee R.T.H., Wang Y.M., Lin Q.S., Wang Y. (2007). Phosphorylation of Rga2, a Cdc42 GAP, by CDK/Hgc1 is crucial for *Candida albicans* hyphal growth. EMBO J..

[22] Wang A., Raniga P.P., Lane S., Lu Y., Liu H. (2009). Hyphal chain formation in *Candida albicans*: Cdc28-Hgc1 phosphorylation of Efg1 represses cell separation genes. Mol. Cell. Biol..

[23] Bishop A., Lane R., Beniston R., Chapa-y-Lazo B., Smythe C., Sudbery P. (2010). Hyphal growth in *Candida albicans* requires the phosphorylation of Sec2 by the Cdc28-Ccn1/Hgc1 kinase. EMBO J..

[24] Gutiérrez-Escribano P., González-Novo A., Suárez M.B., Li C.R., Wang Y., de Aldana C.R.V., Correa-Bordes J. (2011). CDK-dependent phosphorylation of Mob2 is essential for hyphal development in *Candida albicans*. Mol. Biol. Cell..

[25] González-Novo A., Correa-Bordes J., Labrador L., Sánchez M., Vazquez de Aldana C.R., Jiménez J. (2008). Sep7 is essential to modify septin ring dynamics and inhibit cell separation during *Candida albicans* hyphal growth. Mol. Biol. Cell..

[26] Caballero-Lima D., Sudbery P.E. (2014). In *Candida albicans*, phosphorylation of Exo84 by Cdk1-Hgc1 is necessary for efficient hyphal extension. Mol. Biol. Cell..

[27] Sinha I., Wang Y.M., Philp R., Li C.R., Yap W.H., Wang Y. (2007). Cyclin-dependent kinases control septin phosphorylation in *Candida albicans* hyphal development. Dev. Cell.

[28] Taschdjian C.L., Burchall J.J., Kozinn P.J. (1960). Rapid identification of *Candida albicans* by filamentation on serum and serum substitutes. AMA J. Dis. Child..

[29] Simonetti N., Strippoli V., Cassone A. (1974). Yeast-mycelialconversioninduced by N-acetyl-D-glucosamine in *Candida albicans*. Nature.

[30] Buffo J., Herman M.A., Soll D.R. (1984). A characterization of pH-regulated dimorphism in *Candida albicans*. Mycopathologia.

[31] Lu Y., Su C., Wang A., Liu H. (2011). Hyphal development in *Candida albicans* requires two temporally linked changes in promoter chromatin for initiation and maintenance. PLoS Biol..

[32] Klengel T., Liang W.J., Chaloupka J., Ruoff C., Schröppel K., Naglik J.R., Eckert S.E., Mogensen E.G., Haynes K., Tuite M.F. (2005). Fungal adenylyl cyclase integrates CO_2_ sensing with cAMP signaling and virulence. Curr. Biol..

[33] Brown D.H., Giusani A.D., Chen X., Kumamoto C.A. (1999). Filamentous growth of *Candida albicans* in response to physical environmental cues and its regulation by the unique CZF1 gene. Mol. Microbiol..

[34] Rocha C.R., Schroppel K., Harcus D., Marcil A., Dignard D., Taylor B.N., Thomas D.Y., Whiteway M., Leberer E. (2001). Signaling through adenylyl cyclase is essential for hyphal growth and virulence in the pathogenic fungus *Candida albicans*. Mol. Biol. Cell..

[35] Bahn Y.S., Sundstrom P. (2001). CAP1, an adenylatecyclase-associated protein gene, regulates bud-hypha transitions, filamentous growth, and cyclic AMP levels and is required for virulence of *Candida albicans*. J. Bacteriol..

[36] Zou H., Fang H.M., Zhu Y., Wang Y. (2010). *Candida albicans* Cyr1, Cap1 and G-actin form a sensor/effector apparatus for activating cAMP synthesis in hyphal growth. Mol. Microbiol..

[37] Hogan D.A., Sundstrom P. (2009). The Ras/cAMP/PKA signaling pathway and virulence in *Candida albicans*. Future Microbiol..

[38] Kumamoto C.A., Vinces M.D. (2005). Contributions of hyphae and hypha-co-regulated genes to *Candida albicans* virulence. Cell. Microbiol..

[39] Whiteway M., Bachewich C. (2007). Morphogenesis in *Candida albicans*. Annu. Rev. Microbiol..

[40] Brown A.J.P., Brown A.J.P., Esser K. (2005). Integration of metabolism with virulence in *Candida albicans*. Fungal Genomics (The Mycota).

[41] Brown A.J., Budge S., Kaloriti D., Tillmann A., Jacobsen M.D., Yin Z., Ene I.V., Bohovych I., Sandai D., Kastora S. (2014). Stress adaptation in a pathogenic fungus. J. Exp. Biol..

[42] Enjalbert B., MacCallum D.M., Odds F.C., Brown A.J.P. (2007). Niche-specific activation of the oxidative stress response by the pathogenic fungus *Candida albicans*. Infect. Immun..

[43] Miramón P., Dunker C., Windecker H., Bohovych I.M., Brown A.J.P., Kurzai O., Hube B. (2012). Cellular responses of *Candida albicans* to phagocytosis and the extracellular activities of neutrophils are critical to counteract carbohydrate starvation, oxidative and nitrosative stress. PLoS ONE.

[44] Cottier F., Tan A.S., Chen J., Lum J., Zolezzi F., Poidinger M., Pavelka N. (2015). The transcriptional stress response of *Candida albicans* to weak organic acids. G3.

[45] Enjalbert B., Smith D.A., Cornell M.J., Alam I., Nicholls S., Brown A.J.P., Quinn J. (2006). Role of the Hog1 stress-activated protein kinase in the global transcriptional response to stress in the fungal pathogen *Candida albicans*. Mol. Biol. Cell.

[46] Kaloriti D., Tillmann A., Cook E., Jacobsen M.D., You T., Lenardon M.D., Ames L., Barahona M., Chandrasekaran K., Coghill G. (2012). Combinatorial stresses kill pathogenic *Candida* species. Med. Mycol..

[47] Chauhan N.M., Raut J.S., Karuppayil S.M. (2011). A morphogenetic regulatory role for ethyl alcohol in *Candida albicans*. Mycoses.

[48] Chauhan N.M., Shinde R.B., Karuppayil S.M. (2013). Effect of alcohols on filamentation, growth, viability and biofilm development in *Candida albicans*. Braz. J. Microbiol..

[49] Hohmann S. (2002). Osmotic stress signaling and osmoadaptation in yeasts. Microbiol. Mol. Biol..

[50] Enjalbert B., Nantel A., Whiteway M. (2003). Stress-induced gene expression in *Candida albicans*: Absence of a general stress response. Mol. Biol. Cell.

[51] Smith D.A., Nicholls S., Morgan B.A., Brown A.J., Quinn J. (2004). A conserved stress-activated protein kinase regulates a core stress response in the human pathogen *Candida Albicans*. Mol. Biol. Cell.

[52] Fletcher M. (1983). Effects of methanol, ethanol, propanol, and butanol on bacterial attachment to surfaces. J. Gen. Microbiol..

[53] Leberer E., Harcus D., Broadbent I.D., Clark K.L., Dignard D., Ziegelbauer K., Schmit A., Gow N.A.R., Brown A.J.P., Thomas D.Y. (1996). Homologs of the Ste20p and Ste7p protein kinases are involved in hyphal formation of *Candida albicans*. Proc. Natl. Acad. Sci. USA.

[54] Leng P. (1999). Gene Regulation During Morphogenesis in *Candida albicans*. Ph.D. Thesis.

[55] Song W., Wang H., Chen J. (2011). *Candida albicans* Sfl2, a temperature-induced transcriptional regulator, is required for virulence in a murine gastrointestinal infection model. FEMS Yeast Res..

[56] Chibana H., Uno J., Cho T., Mikami Y. (2005). Mutation in IRO1 tightly linked with URA3 gene reduces virulence of *Candida albicans*. Microbiol. Immunol..

[57] Schweizer A., Rupp S., Taylor B.N., Röllinghoff M., Schröppel K. (2000). The TEA/ATTS transcription factor CaTec1p regulates hyphal development and virulence in *Candida albicans*. Mol. Microbiol..

[58] Murad A.M.A., Leng P., Straffon M., Wishart J., Macaskill S., MacCallum D., Schnell N., Talibi D., Marechal D., Tekaia F. (2001). NRG1 represses yeast–hypha morphogenesis and hypha-specific gene expression in *Candida albicans*. EMBO J..

[59] Braun B.R., Kadosh D., Johnson A.D. (2001). NRG1, a repressor of filamentous growth in *C. albicans*, is down-regulated during filament induction. EMBO J..

[60] Talibi D., Raymond M. (1999). Isolation of a putative *Candida albicans* transcriptional regulator involved in pleiotropic drug resistance by functional complementation of a pdr1 pdr3 mutation in Saccharomyces cerevisiae. J. Bacteriol..

[61] Uhl M.A., Biery M., Craig N., Johnson A.D. (2003). Haploinsufficiency-based large-scale forward genetic analysis of filamentous growth in the diploid human fungal pathogen *C. albicans*. EMBO J..

[62] Bauer J., Wendland J. (2007). *Candida albicans* Sfl1 suppresses flocculation and filamentation. Eukar. Cell.

[63] Askew C., Sellam A., Epp E., Mallick J., Hogues H., Mullick A., Nantel A., Whiteway M. (2011). The zinc cluster transcription factor Ahr1p directs Mcm1p regulation of *Candida albicans* adhesion. Mol. Microbiol..

[64] Cleary I.A., Reinhard S.M., Miller C.L., Murdoch C., Thornhill M.H., Lazzell A.L., Monteagudo C., Thomas D.P., Saville S.P. (2011). *Candida albicans* adhesin Als3p is dispensable for virulence in the mouse model of disseminated candidiasis. Microbiology.

[65] Yau K.P.S., Weerasinghe H., Olivier F.A.B., Lo T.L., Powell D.R., Koch B., Beilharz T.H., Traven A. (2023). The proteasome regulator Rpn4 controls antifungal drug tolerance by coupling protein homeostasis with metabolic responses to drug stress. PLoS Pathog..

[66] Chen C.G., Yang Y.L., Shih H.I., Su C.L., Lo H.J. (2004). CaNdt80 is involved in drug resistance in *Candida albicans* by regulating CDR1. Antimicrob. Agents Chemother..

[67] Li R., Kumar R., Tati S., Puri S., Edgerton M. (2013). *Candida albicans* flu1-mediated efflux of salivary histatin 5 reduces its cytosolic concentration and fungicidal activity. Antimicrob. Agents Chemother..

[68] Nobile C.J., Fox E.P., Nett J.E., Sorrells T.R., Mitrovich Q.M., Hernday A.D., Tuch B.B., Andes D.R., Johnson A.D. (2012). A recently evolved transcriptional network controls biofilm development in *Candida albicans*. Cell.

[69] García-Sánchez S., Aubert S., Iraqui I., Janbon G., Ghigo J.M., d’Enfert C. (2004). *Candida albicans* biofilms: A developmental state associated with specific and stable gene expression patterns. Eukaryot. Cell.

[70] Tournu H., Tripathi G., Bertram G., Macaskill S., Mavor A., Walker L., Odds F.C., Gow N.A., Brown A.J. (2005). Global role of the protein kinase Gcn2 in the human pathogen Candida albicans. Eukaryot. Cell.

[71] Singh R.P., Prasad H.K., Sinha I., Agarwal N., Natarajan K. (2011). Cap2-HAP complex is a critical transcriptional regulator that has dual but contrasting roles in regulation of iron homeostasis in *Candida albicans*. J. Biol. Chem..

[72] Fradin C., De Groot P., MacCallum D., Schaller M., Klis F., Odds F.C., Hube B. (2005). Granulocytes govern the transcriptional response, morphology and proliferation of *Candida albicans* in human blood. Mol. Microbiol..

[73] Navarathna D.H.M.L.P., Das A., Morschhäuser J., Nickerson K.W., Roberts D.D. (2011). Dur3 is the major urea transporter in Candida albicans and is co-regulated with the urea amidolyase Dur1, 2. Microbiology.

[74] Vylkova S., Carman A.J., Danhof H.A., Collette J.R., Zhou H., Lorenz M.C. (2011). The fungal pathogen *Candida albicans* autoinduces hyphal morphogenesis by raising extracellular pH. mBio.

[75] Reuss O., Morschhäuser J. (2006). A family of oligopeptide transporters is required for growth of Candida albicans on proteins. Mol. Microbiol..

[76] Theiss S., Ishdorj G., Brenot A., Kretschmar M., Lan C.Y., Nichterlein T., Hacker J., Nigam S., Agabian N., Köhler G.A. (2006). Inactivation of the phospholipase B gene PLB5 in wild-type Candida albicans reduces cell-associated phospholipase A2 activity and attenuates virulence. IJMM.

[77] Niewerth M., Kunze D., Seibold M., Schaller M., Korting H.C., Hube B. (2003). Ciclopiroxolamine treatment affects the expression pattern of *Candida albicans* genes encoding virulence factors, iron metabolism proteins, and drug resistance factors. Antimicrob. Agents Chemother..

[78] Bowles L.K., Ellefson W.L. (1985). Effects of butanol on *Clostridium acetobutylicum*. Appl. Environ. Microbiol..

[79] Heipieper H.J., Neumann G., Cornelissen S., Meinhardt F. (2007). Solvent-tolerant bacteria for biotransformations in two-phase fermentation systems. Appl. Microbiol. Biotechnol..

[80] González-Ramos D., van den Broek M., van Maris A.J., Pronk J.T., Daran J.-M.G. (2013). Genome-scale analyses of butanol tolerance in *Saccharomyces cerevisiae* reveal an essential role of protein degradation. Biotechnol. Biofuels.

[81] Setiawan I., Blanchard G.J. (2014). Structural disruption of phospholipid bilayers over a range of length scales by n-butanol. J. Phys. Chem. B.

[82] Mukherjee P.K., Mohamed S., Chandra J., Kuhn D., Liu S., Antar O.S., Munyon R., Mitchell A.P., Andes D., Chance M.R. (2006). Alcohol dehydrogenase restricts the ability of the pathogen Candida albicans to form a biofilm on catheter surfaces through an ethanol-based mechanism. Infect. Immun..

[83] Bruinenberg P.M., van Dijken J.P., Scheffers W.A. (1983). An enzymic analysis of NADPH production and consumption in *Candida utilis*. J. Gen. Microbiol..

[84] Piekarska K., Hardy G., Mol E., van den Burg J., Strijbis K., van Roermund C., van den Berg M., Distel B. (2008). The activity of the glyoxylate cycle in peroxisomes of Candida albicans depends on a functional beta-oxidation pathway: Evidence for reduced metabolite transport across the peroxisomal membrane. Microbiology.

[85] Hromatka B.S., Noble S.M., Johnson A.D. (2005). Transcriptional response of Candida albicans to nitric oxide and the role of the YHB1 gene in nitrosative stress and virulence. Mol. Biol. Cell.

[86] Liu T.T., Lee R.E., Barker K.S., Lee R.E., Wei L., Homayouni R., Rogers P.D. (2005). Genome-wide expression profiling of the response to azole, polyene, echinocandin, and pyrimidine antifungal agents in Candida albicans. Antimicrob. Agents Chemother..

[87] Lan C.Y., Rodarte G., Murillo L.A., Jones T., Davis R.W., Dungan J., Newport G., Agabian N. (2004). Regulatory networks affected by iron availability in Candida albicans. Mol. Microbiol..

[88] Synnott J.M., Guida A., Mulhern-Haughey S., Higgins D.G., Butler G. (2010). Regulation of the hypoxic response in *Candida albicans*. Eukaryot. Cell.

[89] Fan J., Chaturvedi V., Shen S.H. (2002). Identification and phylogenetic analysis of a glucose transporter gene family from the human pathogenic yeast *Candida albicans*. J. Mol. Evol..

[90] Copping V.M., Barelle C.J., Hube B., Gow N.A., Brown A.J., Odds F.C. (2005). Exposure of *Candida albicans* to antifungal agents affects expression of SAP2 and SAP9 secreted proteinase genes. J. Antimicrob. Agents Chemother..

[91] Kuloyo O., Fourie R., Cason E., Albertyn J., Pohl C.H. (2020). Transcriptome Analyses of *Candida albicans* Biofilms, Exposed to Arachidonic Acid and Fluconazole, Indicates Potential Drug Targets. G3.

[92] Kos-Braun I.C., Koš M. (2017). Post-transcriptional Regulation of Ribosome Biogenesis in Yeast. Microb. Cell.

[93] Fleischmann J., Rocha M.A. (2015). Decrease in Ribosomal RNA in Candida albicans Induced by Serum Exposure. PLoS ONE.

[94] O’Connor L., Caplice N., Coleman D.C., Sullivan D.J., Moran G.P. (2010). Differential filamentation of *Candida albicans* and *Candida dubliniensis* is governed by nutrient regulation of UME6 expression. Eukaryot. Cell..

[95] Oliveira M.T., Specian A.F., Andrade C.G., França E.J., Furlaneto-Maia L., Furlaneto M.C. (2010). Interaction of *Candida parapsilosis* isolates with human hair and nail surfaces revealed by scanning electron microscopy analysis. Micron.

[96] Wright R. (2000). Transmission electron microscopy of yeast. Microsc. Res. Tech..

[97] (2014). IlluminaTruSeq® RNA Sample Preparation v2 Guide. https://support.illumina.com/content/dam/illumina-support/documents/documentation/chemistry_documentation/samplepreps_truseq/truseqrna/truseq-rna-sample-prep-v2-guide-15026495-f.pdf.

[98] (2015). CLC Genomics Workbench Manuals. https://resources.qiagenbioinformatics.com/manuals/clcgenomicsworkbench/852/index.php?manual=Sequence_alignment.html.

[99] Inglis D.O., Arnaud M.B., Binkley J., Shah P., Skrzypek M.S., Wymore F., Binkley G., Miyasato S.R., Simison M., Sherlock G. (2012). The Candida genome database incorporates multiple *Candida* species: Multispecies search and analysis tools with curated gene and protein information for *Candida albicans* and *Candida glabrata*. Nucleic Acids Res..

[100] R Core Team (2013). R: A Language and Environment for Statistical Computing.

