# Supplementary material for: Reprogramming in Candida albicans Gene Expression Network under Butanol Stress Abrogates Hyphal Development
Source: Int J Mol Sci. 2023 Dec 7;24(24):17227. doi: 10.3390/ijms242417227 (PMC10743114; doi:10.3390/ijms242417227)
Supplement: Supplementary file 1 [file ijms-24-17227-s001.zip › Table S2.pdf]

Table S2 Differential gene expression when the serum and serum with butanol data were compared

S20 vs B20 Upregulated genes

Gene I D	Fold change	Standard gene name	Name of *S. cerevisiae* ortholog(s)
**C2_06970W_A**	**57.6**	AAH1	AAH1
**C1_13870W_A**	**55.51**	MET3	MET3
**C5_00430W_A**	**25.47**	MET14	MET14
**C6_03320W_A**	**23.75**		YHI9
**C2_06520C_A**	**16.09**		TPC1
**CR_07740W_A**	**14.76**		
**C7_01520W_A**	**14.33**	FLU1	TPO1
**C7_03850W_A**	**12.91**		NOC3
**C2_05620W_A**	**12.16**	KTI11	KTI11
**C3_06220C_A**	**12.04**	OAC1	OAC1
**C1_02450C_A**	**10.31**		BUD20
**C1_03870C_A**	**10.1**		YNL234W
**C5_03860W_A**	**9.79**	HAM1	HAM1
**C5_04030W_A**	**9.67**		
**C4_02610C_A**	**9.65**	SUL2	SUL1
**C1_05150C_A**	**9.32**		
**C2_04700C_A**	**8.75**		UTP30
**C6_03390W_A**	**8.66**		DIC1
**C4_04100C_A**	**8.6**	ECM1	ECM1
**C6_03440W_A**	**8.53**		CCM1
**C2_07960C_A**	**8.49**	NSA1	NSA1
**C6_01770W_A**	**8.41**	RAD18	RAD18
**C1_13820C_A**	**8.35**		
**C1_00170W_A**	**8.29**	LEU4	LEU4
**C7_02340C_A**	**8.28**		TRM82
**C1_07880C_A**	**8.27**	GCS1	GSH1
**C6_01180C_A**	**8.16**	EBP1	
**CR_04110W_A**	**8.06**		NAF1
**CR_04710W_A**	**7.99**		CWC27
**C4_00800W_A**	**7.92**		NSE4
**C2_06660W_A**	**7.87**		RRP46
**C1_11410C_A**	**7.83**		
**C1_07790C_A**	**7.76**		CMS1
**C1_00900W_A**	**7.75**	RRS1	RRS1
**C6_04320C_A**	**7.45**		
**C1_03060C_A**	**7.44**	TSR2	TSR2
**C7_03310W_A**	**7.43**		
**C2_06850W_A**	**7.39**		SLX9
**C2_08530C_A**	**7.35**		YML096W

**C4_01200C_A**	**7.32**	AAT22	
**C2_07410W_A**	**7.23**		YMR210W
**C3_01560W_A**	**7.22**		PRP43
**C7_03540C_A**	**7.22**	ENP2	ENP2
**C2_04080W_A**	**7.18**		
**C2_02940W_A**	**7.14**	MET1	MET1
**C2_04570W_A**	**7.13**		EBP2
**CR_04500C_A**	**7.13**		
**C2_06530W_A**	**7.01**		TAH11
**C1_07660W_A**	**7**		URB1
**C5_02590C_A**	**7**		SLS1
**C4_00200C_A**	**6.86**	MET15	MET17
**C2_09160W_A**	**6.82**		RRP45
**CR_08340W_A**	**6.75**	CYS3	CYS3
**C2_02030W_A**	**6.66**	ARO3	ARO3
**C2_05080C_A**	**6.66**		BMT2
**C4_06710W_A**	**6.62**		
**C1_02370C_A**	**6.59**		
**C6_01890C_A**	**6.54**		DBP9
**C2_06360C_A**	**6.53**	MAK16	MAK16
**C3_04210W_A**	**6.5**		
**C2_04190C_A**	**6.49**	UGA1	UGA1
**C2_04120C_A**	**6.46**		NOP12
**C2_10270W_A**	**6.4**	PUS7	PUS7
**C4_05230C_A**	**6.4**		RIX1
**C1_12310C_A**	**6.35**	CSI2	BRX1
**C5_00450C_A**	**6.35**	IFG3	
**C3_01150C_A**	**6.34**		APD1
**CR_06570C_A**	**6.33**		
**C1_05060W_A**	**6.32**	REI1	REI1
**C1_12440W_A**	**6.31**		REX4
**C4_01360W_A**	**6.22**	PGA53	
**C6_04330W_A**	**6.21**	NOP8	NOP8
**C4_02830C_A**	**6.19**	HCA4	HCA4
**CR_02060W_A**	**6.17**		
**C3_01200W_A**	**6.11**	UTP20	UTP20
**C6_02770W_A**	**6.11**	MRT4	MRT4
**C2_08550C_A**	**6.07**	MSU1	DSS1
**C4_00510C_A**	**6.07**	RLP24	RLP24
**CR_04750W_A**	**6.06**		YLR363W-A
**C4_04990C_A**	**6.05**		
**C1_12790C_A**	**6.03**	FEN1	ELO2
**C2_01830W_A**	**6.03**	PRP39	PRP39
**C6_01530C_A**	**6**		PMP1
**CR_10470C_A**	**6**		BFR2
**C2_08480W_A**	**5.97**	RRP8	RRP8
**C3_02130W_A**	**5.93**	UTP4	UTP4

**C6_01200W_A**	**5.92**		
**CR_00900W_A**	**5.92**	TRM1	TRM1
**C1_14080W_A**	**5.91**		UTP10
**C2_09660W_A**	**5.89**		NOP16
**C1_12670C_A**	**5.87**		PRP6
**C4_07150W_A**	**5.84**		ATC1
**C1_09510W_A**	**5.8**	YTM1	YTM1
**C4_02220C_A**	**5.8**	ZCF25	
**C2_02580W_A**	**5.79**		
**C5_01710C_A**	**5.79**		
**C4_00810C_A**	**5.77**		QRI7
**CR_10240W_A**	**5.77**	UTP5	UTP5
**C3_05170W_A**	**5.73**	WOR2	
**CR_01410C_A**	**5.73**		YNL035C
**CR_07310W_A**	**5.63**		
**C2_09920W_A**	**5.62**		RRG9
**CR_07030C_A**	**5.61**		RRP36
**C1_09040C_A**	**5.59**		HPM1
**C2_04230W_A**	**5.59**	BAT21	BAT2
**C1_04040C_A**	**5.58**		NOP9
**CR_02420W_A**	**5.58**		UTP23
**C2_00410C_A**	**5.57**		ECM16
**C4_07030W_A**	**5.56**	MET16	MET16
**C2_05160C_A**	**5.51**		PUF6
**C2_10240W_A**	**5.5**	GPD1	GPD1
**C3_02020W_A**	**5.48**		MRD1
**C2_01240C_A**	**5.47**		CHL1
**C3_01680C_A**	**5.47**		YDL183C
**C1_04160C_A**	**5.45**		YMR087W
**C6_01600W_A**	**5.45**		
**CR_05520W_A**	**5.44**	NOC2	NOC2
**C1_10970W_A**	**5.42**		ESF1
**C2_02450C_A**	**5.42**	UTP22	UTP22
**C4_05010W_A**	**5.4**		RSA4
**C7_01030C_A**	**5.39**		RRP12
**CR_05630W_A**	**5.39**	DBP8	DBP8
**C1_06740C_A**	**5.34**	NOP6	NOP6
**C5_03630C_A**	**5.34**		
**CR_09800C_A**	**5.34**		UTP14
**C2_01970C_A**	**5.33**	ROD1	
**C1_05740C_A**	**5.31**		
**C6_03540W_A**	**5.31**		
**C2_09060C_A**	**5.29**	ASN1	ASN2
**C7_00690W_A**	**5.29**	NOP15	NOP15
**C1_04130W_A**	**5.28**	ERB1	ERB1
**C4_03830W_A**	**5.26**		TRM3
**C1_03230C_A**	**5.25**	ARX1	ARX1

**C2_05230C_A**	**5.25**	RPF2	RPF2
**C6_03250W_A**	**5.25**	BMT4	
**CR_04160C_A**	**5.24**		GCD10
**C1_00160C_A**	**5.22**		NSR1
**CR_01600C_A**	**5.22**		
**CR_08490W_A**	**5.22**	TSR1	TSR1
**C7_03700C_A**	**5.21**	ENP1	ENP1
**C2_04860W_A**	**5.18**	PZF1	PZF1
**C3_01430W_A**	**5.18**		RRT14
**C4_02080W_A**	**5.16**		
**C4_06950W_A**	**5.14**		PUS5
**C6_03640W_A**	**5.14**	NOG2	NOG2
**C3_02950C_A**	**5.12**	MET13	MET13
**C4_05540W_A**	**5.1**	HOM3	HOM3
**C3_04690C_A**	**5.09**		
**C1_09710C_A**	**5.06**		UTP7
**CR_03570C_A**	**5.06**	YVH1	YVH1
**C7_00570W_A**	**5.04**	RPA135	RPA135
**C2_05090W_A**	**5.03**	MAK5	MAK5
**CR_02710W_A**	**5.03**	RRP9	RRP9
**C1_03800W_A**	**4.99**	RPP1	RPP1
**C5_03250W_A**	**4.99**	RMS1	RKM4
**C5_04910W_A**	**4.99**		EFG1
**C1_02620C_A**	**4.98**	HOM6	HOM6
**C1_05000W_A**	**4.98**	RHD1	
**C6_03930W_A**	**4.98**	OPT8	YGL114W
**CR_05660W_A**	**4.97**	SDA1	SDA1
**C4_03840C_A**	**4.96**	RRP42	RRP42
**C1_04390C_A**	**4.95**	NOP4	NOP4
**C4_02260C_A**	**4.92**		YMR310C
**C1_05500W_A**	**4.91**	RSM22	RSM22
**C2_06170C_A**	**4.91**	ECM17	MET5
**C1_04120C_A**	**4.85**		IST3
**C5_01090C_A**	**4.85**		
**C1_11000C_A**	**4.83**		CGR1
**C7_04190C_A**	**4.81**	UTP18	UTP18
**C6_02150C_A**	**4.8**		MFM1
**C1_02590C_A**	**4.78**	SNZ1	SNZ1
**C1_12910W_A**	**4.75**		
**C3_01000W_A**	**4.75**	BMS1	BMS1
**C5_03920C_A**	**4.72**		FCF1
**C7_01360C_A**	**4.72**		ZIM17
**C1_09610W_A**	**4.71**		
**C2_09320C_A**	**4.71**	PES1	NOP7
**C2_08280W_A**	**4.7**	EAF3	EAF3
**C3_02750W_A**	**4.7**		
**CR_07750C_A**	**4.69**		DBP6

**C1_11420W_A**	**4.68**	KRR1	KRR1
**C1_04510W_A**	**4.65**		
**CR_04170W_A**	**4.64**		NOP2
**C3_05770C_A**	**4.63**	SAM50	SAM50
**C5_01600C_A**	**4.63**	SPB4	SPB4
**C6_03110C_A**	**4.62**		ASA1
**C3_05590C_A**	**4.61**	BAT22	
**C3_04560W_A**	**4.59**	MAK21	MAK21
**CR_03650W_A**	**4.59**	SGD1	SGD1
**C2_05750W_A**	**4.57**		NOP53
**C3_04900W_A**	**4.57**	NOP14	NOP14
**C6_01860C_A**	**4.56**	HCH1	HCH1
**C6_02230W_A**	**4.53**		LSG1
**C2_07910C_A**	**4.51**		
**C4_05360C_A**	**4.5**		PET54
**C7_03590C_A**	**4.5**		ERC1
**C4_06720W_A**	**4.48**	NOP1	NOP1
**C5_03080C_A**	**4.48**		ESBP6
**C6_00370C_A**	**4.48**	NOP5	NOP58
**C6_01170W_A**	**4.48**	CIC1	CIC1
**C2_00960C_A**	**4.46**	MXR1	MXR1
**C6_04160C_A**	**4.46**	SPB1	SPB1
**C1_05050C_A**	**4.45**	RRP6	RRP6
**C1_06760C_A**	**4.41**		IPI3
**C3_02350W_A**	**4.41**		KRI1
**C1_12630C_A**	**4.4**		CWC15
**C2_04510W_A**	**4.4**	RIM2	RIM2
**C3_06160C_A**	**4.39**		
**C5_04750C_A**	**4.39**	HAS1	HAS1
**CR_01620C_A**	**4.39**	MET6	MET6
**C1_03830C_A**	**4.38**		CSL4
**C5_03530C_A**	**4.38**		COQ10
**C2_08200W_A**	**4.37**		
**C3_07570C_A**	**4.37**		CTO1
**C1_08170C_A**	**4.36**	BUL1	
**C5_02550C_A**	**4.36**	UTP13	UTP13
**C7_03400C_A**	**4.36**		MTR4
**C7_02920W_A**	**4.35**		
**C5_00310C_A**	**4.34**		
**C2_10090C_A**	**4.33**		MRX1
**C4_03030C_A**	**4.33**	NAN1	NAN1
**C4_00540C_A**	**4.32**	NMD5	NMD5
**C2_04350C_A**	**4.31**	SUV3	SUV3
**C7_01200C_A**	**4.3**	DBP7	DBP7
**C1_05990C_A**	**4.28**		YDL144C
**C2_09500W_A**	**4.28**		DUS1
**CR_06230W_A**	**4.28**		

**C3_07300W_A**	**4.27**	NOP13	NOP13
**C1_12030W_A**	**4.26**	SER33	SER3
**C1_12750C_A**	**4.26**		CDC40
**C1_11070W_A**	**4.25**	PET127	PET127
**CR_03380W_A**	**4.25**	MTG1	MTG1
**CR_06770C_A**	**4.25**		
**C1_07960W_A**	**4.24**		LRP1
**C1_02600W_A**	**4.23**	SNO1	SNO1
**C3_05160C_A**	**4.22**		DBP10
**C4_06210C_A**	**4.22**		NUG1
**C1_09490C_A**	**4.21**	GUA1	GUA1
**C2_09380W_A**	**4.2**	RRP15	RRP15
**CR_01710W_A**	**4.2**		TMA23
**C1_07140C_A**	**4.19**	RIA1	RIA1
**C1_13380W_A**	**4.19**		BUD13
**C3_03650W_A**	**4.18**	HBR3	NOB1
**C5_02440C_A**	**4.18**		BUD32
**C5_01610W_A**	**4.17**		DEG1
**CR_02980C_A**	**4.17**	SRP40	SRP40
**C1_01530C_A**	**4.15**		YDR341C
**C1_07930C_A**	**4.15**	ERD1	ERD1
**C1_08290C_A**	**4.15**	DIP2	DIP2
**C1_10800C_A**	**4.15**	ALP1	
**C6_02910W_A**	**4.13**	POP4	POP4
**C3_06380W_A**	**4.12**	NSA2	NSA2
**CR_02030C_A**	**4.12**		RCM1
**C2_08180C_A**	**4.11**		RRP14
**C7_02660C_A**	**4.11**		LSM12
**CR_08890C_A**	**4.11**	ASR2	
**C7_01570C_A**	**4.09**		
**C4_00650W_A**	**4.08**	HIS5	HIS5
**C4_02800W_A**	**4.06**	RPC53	RPC53
**C4_02790C_A**	**4.05**	SAS10	SAS10
**C5_05190W_A**	**4.05**	PCL5	PCL5
**C1_01160C_A**	**4.04**		RRP40
**C1_00280C_A**	**4.03**	RFC52	
**C6_00940C_A**	**4.03**		PRM5
**CR_10540C_A**	**4.03**	MCD1	MCD1
**C1_10250C_A**	**4.02**		
**C2_02540W_A**	**4.02**		RRP5
**CR_01780W_A**	**4.01**		BMT6
**CR_06480C_A**	**4.01**	RRN11	RRN11
**C1_10670C_A**	**3.99**	RPA190	RPA190
**C2_07450C_A**	**3.99**	RCL1	RCL1
**C4_02770C_A**	**3.98**		CDC123
**CR_06110C_A**	**3.98**	RFX1	RFX1
**CR_10690W_A**	**3.98**	POP3	POP3

**C2_06480W_A**	**3.96**		TRM10
**CR_03370C_A**	**3.95**	KTI12	KTI12
**C6_00680C_A**	**3.94**		
**CR_09660W_A**	**3.94**		YLR287C
**C2_05070W_A**	**3.93**	FRE9	
**C2_08000C_A**	**3.92**	KRE30	ARB1
**C3_05460W_A**	**3.92**		
**C3_02670W_A**	**3.91**		RSA1
**C3_04340W_A**	**3.89**	ATF1	
**C3_06720W_A**	**3.89**	PRP5	PRP5
**C1_11450C_A**	**3.88**	SAM2	SAM2
**C5_01430C_A**	**3.88**		RBA50
**CR_04240C_A**	**3.88**		KRE33
**CR_06720W_A**	**3.88**	NMD3	NMD3
**CR_10550W_A**	**3.88**	DRS1	DRS1
**C1_04710C_A**	**3.87**		RSA3
**C1_08190C_A**	**3.87**	CAT8	CAT8
**C1_08870C_A**	**3.87**	JIP5	JIP5
**C1_06440C_A**	**3.86**		
**C1_13370W_A**	**3.86**		YDR161W
**C3_00370C_A**	**3.86**	BUD21	BUD21
**C3_05070W_A**	**3.86**	CEM1	CEM1
**C5_03310C_A**	**3.86**	PCL1	PCL1
**C6_01040C_A**	**3.86**		DHR2
**CR_03550W_A**	**3.86**	NCS2	NCS2
**C1_01150C_A**	**3.85**		PPM2
**C1_10030W_A**	**3.85**	DBP3	DBP3
**C1_02970W_A**	**3.84**		
**C1_11870W_A**	**3.84**	MUP1	MUP1
**C4_01450W_A**	**3.84**	PWP1	PWP1
**C7_01270C_A**	**3.84**		CMR1
**C2_02630W_A**	**3.83**		RRN7
**C5_03970W_A**	**3.83**		
**C5_01480W_A**	**3.81**	FYV5	FYV7
**C2_05830C_A**	**3.79**		SHQ1
**C3_02040C_A**	**3.79**		LOC1
**C4_07140W_A**	**3.79**		UPS2
**C7_01300C_A**	**3.79**	PSF1	PSF1
**C7_01950W_A**	**3.79**		RBD2
**CR_03940W_A**	**3.79**		IPI1
**C2_07920W_A**	**3.78**		RRT2
**C1_14160W_A**	**3.76**		RKM3
**C2_03700W_A**	**3.76**		NTA1
**C3_06370C_A**	**3.76**		LCP5
**CR_09730C_A**	**3.76**	SSF1	SSF1
**C4_06790W_A**	**3.75**		GPN3
**CR_03980W_A**	**3.75**		YJR056C

**C7_00330C_A**	**3.74**		SUA5
**CR_05620C_A**	**3.74**	MTG2	MTG2
**C2_01060C_A**	**3.73**		JJJ3
**C2_02430W_A**	**3.73**	UTP21	UTP21
**C3_04380C_A**	**3.73**		SNU13
**C5_01680C_A**	**3.73**	CCN1	
**C1_01750W_A**	**3.72**		YCR023C
**C1_07150W_A**	**3.72**		
**C1_07950C_A**	**3.72**		RRP17
**C2_00840W_A**	**3.72**		TAH18
**C1_12760W_A**	**3.71**		BCP1
**C2_09180W_A**	**3.7**		RNH202
**C1_14320C_A**	**3.69**		FMP41
**C4_01370W_A**	**3.69**	ILV6	ILV6
**C1_12680W_A**	**3.68**		RRB1
**C3_01300C_A**	**3.67**	NCE103	NCE103
**C3_05440C_A**	**3.67**		AIM23
**C3_06030W_A**	**3.67**	NOG1	NOG1
**C1_05630C_A**	**3.66**		KAP123
**C1_06250W_A**	**3.66**		
**C1_12800W_A**	**3.66**		
**C6_03630W_A**	**3.66**	PAD1	PAD1
**CR_04210C_A**	**3.66**	QDR1	QDR1
**C2_03690C_A**	**3.65**		
**CR_09150W_A**	**3.65**		
**C2_01740C_A**	**3.64**		MRPL31
**C2_08800C_A**	**3.64**	APE3	APE3
**C4_04880W_A**	**3.64**	FGR10	
**C1_02250W_A**	**3.63**	CWH8	CAX4
**C1_01870C_A**	**3.62**	CYS4	CYS4
**C1_04880C_A**	**3.62**	MRPL37	MRPL37
**C4_02010C_A**	**3.62**	HSX11	
**C5_03640W_A**	**3.62**		
**C5_03840W_A**	**3.62**		THI80
**C2_06210C_A**	**3.61**	SER1	SER1
**C3_05570W_A**	**3.61**	QDR2	
**C3_07550C_A**	**3.61**		GEP3
**CR_02170W_A**	**3.6**	MET2	MET2
**CR_04560C_A**	**3.6**		RAD27
**CR_07860C_A**	**3.6**	ASF1	ASF1
**C1_01030W_A**	**3.59**	HMT1	HMT1
**C1_02380C_A**	**3.58**		CIN4
**C2_01070W_A**	**3.58**		RPA49
**C1_10620W_A**	**3.57**		CBF5
**C3_07540C_A**	**3.57**		
**C4_05650W_A**	**3.57**		FAF1
**C6_02490C_A**	**3.56**		

**C1_02080W_A**	**3.55**	SET6	SET6
**C3_05140C_A**	**3.55**		TRM11
**CR_06330C_A**	**3.54**		YML108W
**CR_08500W_A**	**3.54**		SYO1
**C1_04340C_A**	**3.53**		YMR010W
**C1_08810C_A**	**3.53**	MSS116	MSS116
**C5_04570C_A**	**3.53**	URA7	URA7
**C4_03190W_A**	**3.52**		
**C5_05410C_A**	**3.52**	ILV3	ILV3
**C7_03930C_A**	**3.51**	MAM33	MAM33
**C1_10110W_A**	**3.5**		
**CR_09950C_A**	**3.5**	SIK1	NOP56
**CR_10190C_A**	**3.5**		
**C1_00560W_A**	**3.49**	CNS1	CNS1
**C4_04840C_A**	**3.49**	AGP3	AGP3
**C1_11550W_A**	**3.48**	GAR1	GAR1
**C2_05190W_A**	**3.47**	CDC21	CDC21
**C4_04470W_A**	**3.47**	SAP10	
**C1_05110C_A**	**3.46**	ARO4	ARO4
**C2_05860C_A**	**3.46**		XBP1
**C6_00320C_A**	**3.45**	DNA2	DNA2
**C7_03570W_A**	**3.45**	ARG4	ARG4
**C1_03540C_A**	**3.44**		ILM1
**C1_13400C_A**	**3.44**	AGC1	AGC1
**C4_03170W_A**	**3.44**		NSE3
**C3_07660W_A**	**3.43**		RAD4
**C6_01290C_A**	**3.43**	QDR3	QDR3
**CR_01950W_A**	**3.42**		RPA43
**C1_12280C_A**	**3.41**		MRPL13
**C3_07580W_A**	**3.41**		
**C5_03470C_A**	**3.41**		
**C6_01420C_A**	**3.41**		
**C1_11380W_A**	**3.4**	NEP1	EMG1
**CR_02430C_A**	**3.4**		FAP1
**CR_07600W_A**	**3.4**		FUN30
**C2_03200W_A**	**3.39**	PRP3	PRP3
**C3_07750W_A**	**3.39**	UTP9	UTP9
**CR_09370W_A**	**3.39**	ELF1	NEW1
**C2_04200W_A**	**3.38**	TBF1	TBF1
**C2_02420C_A**	**3.37**		DUS3
**C2_05840W_A**	**3.37**		DPH1
**C4_04160W_A**	**3.37**		
**C1_11230W_A**	**3.36**	FGR39	
**C7_01370W_A**	**3.36**		
**C2_00390C_A**	**3.35**		STR3
**C4_04520W_A**	**3.35**		PXR1
**C6_00760W_A**	**3.34**		FES1

**CR_09900C_A**	**3.34**	DPB2	DPB2
**C2_02950W_A**	**3.33**		BNA3
**C3_05510W_A**	**3.33**		
**C5_00680W_A**	**3.33**	RMT2	RMT2
**C5_01060C_A**	**3.33**	BUD23	BUD23
**C6_01120C_A**	**3.33**		STE14
**C5_04580C_A**	**3.32**		VPS25
**C1_05020C_A**	**3.31**	ARG2	ARG2
**CR_00390W_A**	**3.31**		
**C1_00340W_A**	**3.3**	HBR1	FAP7
**C3_00320W_A**	**3.28**	RHR2	GPP1
**C3_04370C_A**	**3.28**		URB2
**C5_03410C_A**	**3.28**		MRPL20
**C2_06830C_A**	**3.27**	FHL1	FHL1
**C5_03810C_A**	**3.27**		
**CR_00080W_A**	**3.27**	MIS12	
**C1_10080W_A**	**3.26**		CWC21
**C4_02750W_A**	**3.26**	HIT1	HIT1
**C1_03840W_A**	**3.25**		
**C1_09170W_A**	**3.25**	CTA8	HSF1
**C2_05050C_A**	**3.25**		GNT1
**C6_02960W_A**	**3.25**		YGR012W
**C1_12570C_A**	**3.24**		ELP4
**C2_06700W_A**	**3.24**	AMO2	
**C4_03730C_A**	**3.24**		TRM5
**C4_02850W_A**	**3.22**		ELP2
**C4_03740W_A**	**3.22**		RRP4
**CR_01480W_A**	**3.22**	PRS	AIM10
**C7_01170C_A**	**3.21**		
**CR_06840W_A**	**3.21**		UTP25
**CR_09760W_A**	**3.2**	MED21	SRB7
**C1_11610C_A**	**3.19**		
**C2_06640C_A**	**3.19**	VAS1	VAS1
**C2_07000W_A**	**3.18**		YCR051W
**C3_03550C_A**	**3.18**	ULP1	
**C4_00750C_A**	**3.18**		
**C5_02120C_A**	**3.18**	RIX7	RIX7
**CR_06450W_A**	**3.18**		GRC3
**C2_00070C_A**	**3.17**	MPP10	MPP10
**C4_00960W_A**	**3.16**	PTC8	
**C6_00950C_A**	**3.16**	TOP1	TOP1
**C6_02300C_A**	**3.16**		
**C4_06960W_A**	**3.15**		
**CR_06300C_A**	**3.15**	FGR50	
**C3_01770C_A**	**3.14**		SCM3
**C3_07140C_A**	**3.14**		
**C4_00140C_A**	**3.14**	HIS4	HIS4

**CR_07710W_A**	**3.14**	ARO2	ARO2
**C4_00700C_A**	**3.13**		
**C5_00630C_A**	**3.13**	EXO1	EXO1
**C5_05340W_A**	**3.13**		SQT1
**C6_02420W_A**	**3.13**		PGC1
**C6_02440C_A**	**3.13**	PRP45	PRP45
**C7_03880C_A**	**3.13**		
**C3_06390W_A**	**3.12**		
**C4_00740W_A**	**3.12**		POP7
**C7_03720C_A**	**3.12**	HIS7	HIS7
**C3_04860W_A**	**3.11**		SFP1
**C4_04810C_A**	**3.11**		TRM8
**C1_05330C_A**	**3.1**		YDR306C
**C2_08670C_A**	**3.1**	UTP15	UTP15
**C3_03810W_A**	**3.1**	RAD53	RAD53
**CR_08940W_A**	**3.1**		GCD14
**CR_10410C_A**	**3.1**		PNO1
**C2_04820W_A**	**3.09**		MRM1
**C2_06750C_A**	**3.09**		
**CR_06970C_A**	**3.09**		LIN1
**C1_13130C_A**	**3.08**		
**C1_13200C_A**	**3.08**	REX3	REX3
**C3_03470W_A**	**3.08**		UGA2
**C1_05510C_A**	**3.07**	RPS27A	RPS27A
**C1_12210W_A**	**3.07**	TRP3	TRP3
**C3_02180C_A**	**3.07**		DYS1
**CR_02890C_A**	**3.07**		RPC82
**C1_00640C_A**	**3.06**	RPO41	RPO41
**C1_03400W_A**	**3.06**		ART5
**C2_10740C_A**	**3.06**		EMW1
**C7_03170W_A**	**3.06**	IFA21	
**CR_03340C_A**	**3.06**		SLD2
**CR_07950W_A**	**3.06**	IMP4	IMP4
**C2_04530W_A**	**3.05**	MED8	MED8
**C2_06130W_A**	**3.05**		CSM3
**C3_05800W_A**	**3.05**		SEN54
**C3_06540C_A**	**3.05**		
**CR_00810W_A**	**3.05**		
**CR_01700C_A**	**3.05**		PRP24
**C2_07340W_A**	**3.04**	NOC4	NOC4
**C3_05090C_A**	**3.04**		
**C3_07400W_A**	**3.04**		TRM7
**C6_04260C_A**	**3.04**		JLP2
**C1_02880C_A**	**3.03**	MIA40	MIA40
**C1_05930C_A**	**3.03**		DUG2
**C2_01400C_A**	**3.03**	ESC4	RTT107
**C4_07060W_A**	**3.03**		RPO31

**C7_04090C_A**	**3.03**		
**C1_03790C_A**	**3.02**		FPR3
**C1_06540C_A**	**3.01**		RRP43
**C2_07360W_A**	**3.01**		TGS1
**C3_02320W_A**	**3.01**	ILV2	ILV2
**C3_07650C_A**	**3.01**		
**C5_03360W_A**	**3.01**	RPO26	RPO26
**C7_01400C_A**	**3.01**		RRN5
**C3_02370C_A**	**3**	ALR1	ALR1
**CR_03760W_A**	**3**		NNT1
**C1_13360C_A**	**2.99**	RMP1	RMP1
**C5_00920W_A**	**2.99**		DIB1
**C7_01490W_A**	**2.99**		
**C1_00840C_A**	**2.98**	NRM1	
**C1_10640C_A**	**2.98**	PEA2	PEA2
**C2_05980C_A**	**2.98**		TES1
**C7_00370W_A**	**2.98**	DCC1	DCC1
**CR_03200C_A**	**2.98**		POP1
**C2_03070C_A**	**2.97**	SMP3	SMP3
**C3_02390W_A**	**2.97**		
**CR_00800C_A**	**2.97**	PWP2	PWP2
**CR_01590C_A**	**2.97**	TRP2	TRP2
**CR_10260W_A**	**2.97**		MRM2
**C1_09910C_A**	**2.96**		
**C3_02700W_A**	**2.96**	SMC3	SMC3
**CR_05570C_A**	**2.96**	RNR3	
**C3_01450C_A**	**2.95**	MET18	MET18
**CR_01570W_A**	**2.95**		PTH2
**C2_02690W_A**	**2.94**	SER2	SER2
**C2_07130C_A**	**2.94**	RCK2	RCK2
**C3_00100W_A**	**2.94**		YJU2
**C3_04270C_A**	**2.94**	CTP1	CTP1
**C4_06670W_A**	**2.94**		
**CR_01670W_A**	**2.94**		
**CR_03110W_A**	**2.94**		MSE1
**C1_03460C_A**	**2.93**		
**C1_05210C_A**	**2.93**		YNR040W
**C2_04990W_A**	**2.93**		SLD7
**C2_06190W_A**	**2.93**	ILV1	ILV1
**C1_06750W_A**	**2.92**		
**C2_01870C_A**	**2.92**		
**C4_03870C_A**	**2.92**		NUR1
**C5_04290C_A**	**2.92**		
**C6_01300W_A**	**2.92**		CST26
**C4_01560C_A**	**2.9**		BUL1
**C1_01110C_A**	**2.89**	ZPR1	ZPR1
**C2_08030W_A**	**2.89**		

**CR_04300W_A**	**2.89**		NCL1
**CR_10650W_A**	**2.89**	LTV1	LTV1
**C3_01480C_A**	**2.88**	RKI1	RKI1
**C1_10200C_A**	**2.87**		HOL1
**C3_05280C_A**	**2.87**		
**C4_06940C_A**	**2.87**		
**C2_06650C_A**	**2.86**		PRP31
**C4_00210W_A**	**2.86**		YPL034W
**C6_02170C_A**	**2.86**	SWD3	SWD3
**C1_07200W_A**	**2.85**	YMC1	YMC1
**C5_02410C_A**	**2.85**		
**C5_03700C_A**	**2.85**		DPH5
**CR_00680W_A**	**2.85**	BUD22	BUD22
**CR_01550C_A**	**2.85**		YBL055C
**CR_06980W_A**	**2.85**		LTO1
**CR_10750C_A**	**2.85**		YCR087C-A
**C1_07470C_A**	**2.84**		IRC3
**C1_11880W_A**	**2.84**		MRPL51
**C2_09600C_A**	**2.84**		YCR090C
**C4_04110W_A**	**2.84**		
**C6_00530C_A**	**2.84**		SME1
**C6_02900C_A**	**2.84**		AIM22
**C1_00510W_A**	**2.83**		ECM31
**C1_04350C_A**	**2.82**		
**C1_05820C_A**	**2.82**	RAD10	RAD10
**C3_06530W_A**	**2.82**		
**C5_00820W_A**	**2.82**		MRPL17
**C1_00990C_A**	**2.81**	NMA111	NMA111
**C2_10230W_A**	**2.81**		MET32
**C3_05380W_A**	**2.81**		YJL163C
**CR_01690C_A**	**2.81**	CDC60	CDC60
**C1_01450W_A**	**2.8**	ABC1	COQ8
**C1_13330C_A**	**2.8**		SPE4
**C5_05010W_A**	**2.8**		SNN1
**C6_04240W_A**	**2.8**		
**CR_08100C_A**	**2.8**	RIO2	RIO2
**C1_12110C_A**	**2.79**		
**C2_01050W_A**	**2.79**		
**C2_05890C_A**	**2.79**	IDP1	IDP1
**C2_10140W_A**	**2.79**		UBP8
**C4_01130C_A**	**2.79**		VPS71
**C5_04650C_A**	**2.79**	MSM1	MSM1
**C6_00670W_A**	**2.79**	CDC14	CDC14
**C7_03210W_A**	**2.79**		
**C1_09540W_A**	**2.78**		
**C2_02490C_A**	**2.78**		YPK9
**C3_03200C_A**	**2.78**		YDR286C

**C6_00380C_A**	**2.78**		
**CR_08020C_A**	**2.78**	NOP10	NOP10
**C1_13470W_A**	**2.77**	KNS1	KNS1
**C2_09750W_A**	**2.77**	LEU42	
**C3_00330W_A**	**2.77**	UTP8	UTP8
**CR_01030W_A**	**2.77**	DAL81	DAL81
**CR_02370W_A**	**2.77**	ERG25	ERG25
**CR_08000C_A**	**2.77**		
**CR_08880C_A**	**2.77**		
**C1_00910W_A**	**2.76**		TRI1
**C1_09010W_A**	**2.76**	NAR1	NAR1
**C1_10340W_A**	**2.76**		
**C2_00080C_A**	**2.76**	FAV3	
**C2_09140C_A**	**2.76**	MET10	MET10
**C3_02560W_A**	**2.76**	DDC1	DDC1
**C3_04710W_A**	**2.76**	ARV1	ARV1
**C4_03350C_A**	**2.76**		
**C4_05460C_A**	**2.76**	OFD1	TPA1
**C5_03460C_A**	**2.76**		
**C6_03220W_A**	**2.76**		
**C7_00160C_A**	**2.76**		ALB1
**CR_06510W_A**	**2.76**		
**CR_07510W_A**	**2.76**		SMB1
**CR_08580C_A**	**2.76**		
**C4_07090C_A**	**2.75**	TRP4	TRP4
**C6_00660C_A**	**2.75**		AIM7
**C7_00220W_A**	**2.75**		
**C1_03260W_A**	**2.74**		MGS1
**C1_06510C_A**	**2.74**		MCH4
**C2_01410C_A**	**2.74**	CLB2	CLB2
**C2_02930C_A**	**2.74**		ETT1
**C2_06460W_A**	**2.74**	RTA3	
**C5_05280C_A**	**2.74**	SDH4	TIM18
**C1_02190W_A**	**2.73**	ABP140	ABP140
**C1_06350W_A**	**2.73**		
**C2_01430W_A**	**2.73**	APT1	APT1
**C5_03010W_A**	**2.73**		SNU66
**C6_02690C_A**	**2.73**		MRN1
**CR_06120W_A**	**2.73**	RPL7	RLP7
**C1_02470W_A**	**2.72**		
**C4_02870C_A**	**2.72**	ZUO1	ZUO1
**C5_00700C_A**	**2.72**	SPE2	SPE2
**C5_03550W_A**	**2.72**		NUP2
**C1_11910W_A**	**2.71**		THP1
**C3_02910W_A**	**2.71**		
**C4_03900C_A**	**2.71**	RAM1	RAM1
**CR_03680C_A**	**2.71**	SMC1	SMC1

**C1_07710C_A**	**2.7**	ADE4	ADE4
**C6_01980C_A**	**2.7**		MRPS18
**C7_03530C_A**	**2.7**	ISY1	ISY1
**C2_03950W_A**	**2.69**		MRPL24
**C2_09900C_A**	**2.69**	TIM23	TIM23
**C3_02850C_A**	**2.69**		ATF2
**C4_01280C_A**	**2.69**		PRP21
**C4_02990C_A**	**2.69**	GST2	
**C5_02540C_A**	**2.69**	SPR28	
**C6_02350C_A**	**2.69**		PUS1
**CR_09310W_A**	**2.69**		RKM1
**C2_05340C_A**	**2.68**	RPC40	RPC40
**C2_07680W_A**	**2.68**		MRPL7
**C6_01030W_A**	**2.68**	HAL22	
**C1_07500C_A**	**2.67**	LHP1	LHP1
**C2_04300C_A**	**2.67**		MRX11
**C2_05330C_A**	**2.67**		
**C3_01310W_A**	**2.67**		IRC5
**C3_02460C_A**	**2.67**	PHA2	PHA2
**C4_04730W_A**	**2.67**	BAS1	BAS1
**CR_05480W_A**	**2.67**		ODC1
**CR_08960C_A**	**2.67**	MPS1	MPS1
**C3_07620C_A**	**2.66**	RIT1	RIT1
**C4_00880W_A**	**2.66**		CCA1
**C2_05200C_A**	**2.65**	SGO1	SGO1
**C2_06810C_A**	**2.65**	RPL11	RPL11B
**C4_00690C_A**	**2.65**		DPH2
**C6_02430W_A**	**2.65**		ITC1
**C1_09390W_A**	**2.64**		MOD5
**C2_03920C_A**	**2.64**	GPI13	GPI13
**C4_04220W_A**	**2.64**	DUO1	
**C6_03210C_A**	**2.64**		RPC34
**C7_01020C_A**	**2.64**		MRPS16
**C4_06410W_A**	**2.63**		SYF1
**C7_00560C_A**	**2.63**	THG1	THG1
**C1_02090C_A**	**2.62**		DRN1
**C2_03830W_A**	**2.62**		DPH6
**C7_00530C_A**	**2.62**	PSF3	PSF3
**CR_06910W_A**	**2.62**	BUD5	BUD5
**C4_06850C_A**	**2.61**		MAP1
**C5_02290W_A**	**2.61**	PDE1	PDE1
**C5_05080W_A**	**2.61**	GRF10	PHO2
**C1_05220C_A**	**2.6**		
**C1_08630W_A**	**2.6**		MTF2
**CR_05550C_A**	**2.6**		RET1
**C2_00810C_A**	**2.59**		
**C4_05000W_A**	**2.59**		

**C5_02010C_A**	**2.59**		BUD27
**CR_07640C_A**	**2.59**		VFA1
**C1_11030W_A**	**2.58**	RPL82	
**C3_01710C_A**	**2.58**	PPT1	PPT1
**C6_00690W_A**	**2.58**		
**CR_02110W_A**	**2.58**	MCM6	MCM6
**C1_12380C_A**	**2.57**	WRS1	WRS1
**C4_01500W_A**	**2.57**		NCS6
**C6_00360C_A**	**2.57**		MIX23
**C7_01660C_A**	**2.57**		NEL1
**C2_00820W_A**	**2.56**		MSS1
**C3_03110W_A**	**2.56**		SNG1
**C4_03380C_A**	**2.56**	DAO2	
**CR_02600W_A**	**2.56**		
**C1_06210W_A**	**2.55**	BUD16	BUD16
**C1_03240W_A**	**2.54**		
**C1_12640W_A**	**2.54**		SOM1
**C2_00870W_A**	**2.54**		
**C2_07020C_A**	**2.54**		
**C3_01850W_A**	**2.54**		MRP1
**C1_10690W_A**	**2.53**		IMO32
**C1_12350W_A**	**2.53**		DIS3
**C3_02760C_A**	**2.53**		
**C7_02460C_A**	**2.53**		NPA3
**CR_03440W_A**	**2.53**		
**C4_00940W_A**	**2.52**		ELP6
**C5_00420C_A**	**2.52**	MRP17	MRP17
**CR_04740C_A**	**2.52**	ADE6	ADE6
**C1_02840W_A**	**2.51**	PDE2	PDE2
**C2_10100W_A**	**2.51**	CWC22	CWC22
**C3_02840W_A**	**2.51**		POP5
**C3_06150W_A**	**2.51**		YPR022C
**C4_02880C_A**	**2.51**		SDO1
**CR_02720C_A**	**2.51**	RPC31	RPC31
**C3_05900W_A**	**2.5**		YPQ1
**C4_01080W_A**	**2.5**	RAT1	RAT1
**C4_02590C_A**	**2.5**		SLX4
**C6_02290C_A**	**2.5**		TYW1
**CR_03100W_A**	**2.5**	SMC5	SMC5
**CR_05100W_A**	**2.5**		BIR1
**C1_05090W_A**	**2.49**	TRY2	
**C1_08890C_A**	**2.49**		MSL1
**C1_10440W_A**	**2.49**		YEL043W
**C2_06000W_A**	**2.49**	LAS1	LAS1
**C5_00320W_A**	**2.49**		YDR115W
**CR_10120C_A**	**2.49**		RRG7
**C1_05010C_A**	**2.48**		IZH1

**C1_07000W_A**	**2.48**	MEF2	MEF2
**C2_08560W_A**	**2.48**	SMC2	SMC2
**C6_02970C_A**	**2.48**		PEX25
**C7_03790W_A**	**2.48**	MRP7	MRP7
**C7_03820C_A**	**2.48**		
**CR_07000C_A**	**2.48**	POL2	POL2
**C2_09890W_A**	**2.47**	SMM1	SMM1
**C4_03550W_A**	**2.47**	RAD2	RAD2
**C5_04340W_A**	**2.47**		MPD1
**C6_02270C_A**	**2.47**	ALG11	ALG11
**CR_01960C_A**	**2.47**	MRS2	MRS2
**C1_02900C_A**	**2.46**		SPP382
**C1_03630W_A**	**2.46**		TTI1
**C1_09970C_A**	**2.46**	PDC2	PDC2
**C2_01370C_A**	**2.46**		
**CR_08450C_A**	**2.46**		
**C1_04970W_A**	**2.45**		CLU1
**C5_03390C_A**	**2.45**	FUR1	FUR1
**C7_04230W_A**	**2.45**	NRG1	NRG2
**CR_04920W_A**	**2.45**		YDL119C
**C1_10650W_A**	**2.44**	APN2	APN2
**C3_00840C_A**	**2.44**		AIM11
**C4_00890W_A**	**2.44**	ARO1	ARO1
**C7_02930C_A**	**2.44**		TSR4
**CR_01260W_A**	**2.44**		TMN3
**C1_10920W_A**	**2.43**		TFC6
**C2_02270C_A**	**2.43**		MRPS8
**C2_09310C_A**	**2.43**		GTF1
**C3_05860C_A**	**2.43**		BMT5
**C5_02070C_A**	**2.43**		ESF2
**C5_02490C_A**	**2.43**	TIF5	TIF5
**C1_00520W_A**	**2.42**		YOR283W
**C1_07340W_A**	**2.42**		ISW1
**C1_07510W_A**	**2.42**		YBL036C
**C2_01420C_A**	**2.42**		
**C3_06240C_A**	**2.42**		MRPL39
**CR_01560W_A**	**2.42**		
**C1_11790W_A**	**2.41**		CDC8
**C2_05270W_A**	**2.41**		KAP104
**C3_05390C_A**	**2.41**	GPI1	GPI1
**C3_05980C_A**	**2.41**	FAA2	
**C4_03910W_A**	**2.41**	FGR28	
**C5_04310W_A**	**2.41**		
**C5_04740C_A**	**2.41**	HYS2	POL31
**C5_05320C_A**	**2.41**	HIS1	HIS1
**CR_07010W_A**	**2.41**	MIS11	MIS1
**CR_10580W_A**	**2.41**	PRO1	PRO1

**C1_02390W_A**	**2.4**		PSO2
**C5_00250C_A**	**2.4**		BER1
**C6_02280W_A**	**2.4**		STB1
**CR_08190W_A**	**2.4**		
**C1_10950C_A**	**2.39**		RIO1
**C2_02160W_A**	**2.39**	SAM37	SAM37
**C6_00560W_A**	**2.39**		NDC1
**C1_04330W_A**	**2.38**	RPN4	RPN4
**C2_09020W_A**	**2.38**	CDC47	MCM7
**C3_01350C_A**	**2.38**	URA3	URA3
**C5_02780W_A**	**2.38**		SPT21
**CR_03230W_A**	**2.38**		SQS1
**C1_07850C_A**	**2.37**		DOC1
**C5_03400C_A**	**2.37**		LSM7
**C5_05000C_A**	**2.37**		ROG1
**CR_01440C_A**	**2.37**		
**C2_04730W_A**	**2.36**		
**C7_00950W_A**	**2.36**	YML6	YML6
**CR_03750C_A**	**2.36**	PRP42	PRP42
**C1_05650W_A**	**2.35**		DAS2
**C1_08920W_A**	**2.35**		RSM25
**C2_02680W_A**	**2.35**	PDR17	
**C3_05930W_A**	**2.35**	CTA4	OAF1
**C3_07800C_A**	**2.35**		FCF2
**C4_02140C_A**	**2.35**	TIM10	TIM10
**C5_02770W_A**	**2.35**	MSW1	MSW1
**C6_04530C_A**	**2.35**		RTT10
**C1_08520C_A**	**2.34**		MRPL28
**C3_00560C_A**	**2.34**	SOF1	SOF1
**C4_04820C_A**	**2.34**		MRPL11
**C5_00260W_A**	**2.34**		PRS1
**C5_01180W_A**	**2.34**	PUS4	PUS4
**CR_09740W_A**	**2.34**		RRP3
**C1_07690C_A**	**2.33**		CSE1
**C1_08410C_A**	**2.33**	SAM4	SAM4
**C2_09790W_A**	**2.33**		
**CR_09010C_A**	**2.33**		YHR112C
**C1_07830C_A**	**2.32**		ECO1
**C1_09880C_A**	**2.32**	BUD31	BUD31
**C3_03330C_A**	**2.32**		CLF1
**C3_06570C_A**	**2.32**	OPI3	OPI3
**C4_07080C_A**	**2.32**		
**C5_01100C_A**	**2.32**	CLN3	CLN3
**C1_05270C_A**	**2.31**		MHR1
**C1_11900C_A**	**2.31**		UPF3
**C2_10360C_A**	**2.31**	HEM3	HEM3
**C3_05890W_A**	**2.31**	TRM12	TRM12

**C3_06830C_A**	**2.31**		YLR419W
**C5_01700W_A**	**2.31**	MPRL36	MRPL36
**C6_03330C_A**	**2.31**		YPR097W
**C7_01580W_A**	**2.31**	SMC6	SMC6
**C7_01600W_A**	**2.31**		OXA1
**C1_08750W_A**	**2.3**	YMC2	
**C2_10770W_A**	**2.3**	RPC11	RPC11
**C3_05310W_A**	**2.3**	USO6	
**C4_03720C_A**	**2.3**		
**C2_04640C_A**	**2.29**	SNU114	SNU114
**C6_01630W_A**	**2.29**	TIF3	TIF3
**CR_02540W_A**	**2.29**	CHO2	CHO2
**C1_08420W_A**	**2.28**	GCR3	STO1
**C5_02740W_A**	**2.28**		CYC2
**C6_03380W_A**	**2.28**		HEK2
**CR_00460C_A**	**2.28**		IMP3
**CR_03360W_A**	**2.28**		UTP11
**CR_06060W_A**	**2.28**		
**C1_10070C_A**	**2.27**	MNN23	
**C3_06640W_A**	**2.27**		
**C5_03290C_A**	**2.27**		MRP10
**CR_07720C_A**	**2.27**	CDC45	CDC45
**CR_10570C_A**	**2.27**	YHB4	
**C2_00200W_A**	**2.26**		PRP18
**C1_05640C_A**	**2.25**	FAL1	FAL1
**C1_12820C_A**	**2.25**		RPB5
**C2_03560C_A**	**2.25**		MRPL35
**C3_03560W_A**	**2.25**		ICP55
**C4_00480W_A**	**2.25**	DUR4	
**C4_04900W_A**	**2.25**	RPL30	RPL30
**C4_05890W_A**	**2.25**		
**C1_05360C_A**	**2.24**		PAP2
**C1_07130C_A**	**2.24**		
**C1_07910C_A**	**2.24**	MRPL3	MRPL3
**C1_08210C_A**	**2.24**	MLT1	
**C3_03000W_A**	**2.24**		
**C4_06040W_A**	**2.24**		
**C4_06110C_A**	**2.24**	TRP5	TRP5
**C7_01250W_A**	**2.24**	ASC1	ASC1
**CR_02550C_A**	**2.24**		MTR3
**CR_06950C_A**	**2.24**	ATX1	ATX1
**CR_07050C_A**	**2.24**	URA2	URA2
**C1_06380C_A**	**2.23**		
**C1_10610W_A**	**2.23**	FCY23	TPN1
**C2_04980C_A**	**2.23**	CKA2	CKA2
**C2_09300W_A**	**2.23**	MED18	SRB5
**CR_04050C_A**	**2.23**	MEX67	MEX67

**CR_08320W_A**	**2.23**	ATS1	ATS1
**CR_08520C_A**	**2.23**		
**CR_10180W_A**	**2.23**		
**C1_01380C_A**	**2.22**	TRM2	TRM2
**C1_05200C_A**	**2.22**		
**C1_05470W_A**	**2.22**	MTM1	MTM1
**C1_09370W_A**	**2.22**	CEF1	CEF1
**C1_13520C_A**	**2.22**	MCU1	
**C4_00790C_A**	**2.22**	MLH1	MLH1
**C5_04720C_A**	**2.22**		FOL2
**C7_02060W_A**	**2.22**	LIG1	TRL1
**C1_07890C_A**	**2.21**	ADE5,7	ADE5,7
**C1_09840C_A**	**2.21**		STS1
**C1_12340C_A**	**2.21**	MRPS9	MRPS9
**C2_00650W_A**	**2.21**		
**C2_08730W_A**	**2.21**	MFG1	MFG1
**C3_06200C_A**	**2.21**	MMS21	MMS21
**C4_06590W_A**	**2.21**		
**C5_02270W_A**	**2.21**	THR4	THR4
**C5_02760W_A**	**2.21**	CKB2	CKB2
**CR_00410W_A**	**2.21**	RTT109	RTT109
**C3_00660W_A**	**2.2**		RSE1
**C4_01470W_A**	**2.2**		HRT3
**C5_03600W_A**	**2.2**	MDJ1	MDJ1
**C2_02400W_A**	**2.19**	ECM3	ECM3
**C2_07860W_A**	**2.19**		CUE3
**C4_04700W_A**	**2.19**	SSZ1	SSZ1
**C4_05970W_A**	**2.19**		
**C5_03280W_A**	**2.19**	CAM1-1	
**C6_00620W_A**	**2.19**	FCA1	FCY1
**C6_02240C_A**	**2.19**	RPL10A	RPL1A
**C1_04530C_A**	**2.18**		TRM44
**C1_10700C_A**	**2.18**	NIT3	NIT3
**C3_06300W_A**	**2.18**	DOT1	DOT1
**C4_03700W_A**	**2.18**		VBA1
**CR_06490C_A**	**2.18**	HSP60	HSP60
**C1_09790C_A**	**2.17**		AIR2
**C1_13050W_A**	**2.17**	RPL14	RPL14B
**C2_06330C_A**	**2.17**	AVT1	
**C5_00030W_A**	**2.17**		MRPL1
**C7_02940C_A**	**2.17**	IFM1	IFM1
**CR_02580W_A**	**2.17**	PAN6	PAN6
**CR_04010C_A**	**2.17**	MRPL40	MRPL40
**C1_04720W_A**	**2.16**		
**C1_13840W_A**	**2.16**		SMF2
**C2_01580W_A**	**2.16**	REX2	REX2
**C3_00090W_A**	**2.16**	RPS24	RPS24B

**C5_03850W_A**	**2.16**		
**C6_02370C_A**	**2.16**		RSM23
**CR_00830W_A**	**2.16**		TAH1
**C2_04320W_A**	**2.15**		
**C3_06230W_A**	**2.15**	CYM1	CYM1
**C4_03410W_A**	**2.15**		MRPL22
**C5_00290W_A**	**2.15**	DUS4	DUS4
**CR_00430C_A**	**2.15**		FRA2
**CR_03400W_A**	**2.15**		ISM1
**CR_06680C_A**	**2.15**		ECM32
**CR_08410W_A**	**2.15**		REH1
**CR_09990W_A**	**2.15**		
**C2_01820C_A**	**2.14**		SNU71
**C3_04680W_A**	**2.14**	RPP2B	RPP2B
**C4_02720C_A**	**2.14**		SFK1
**C6_00640C_A**	**2.14**		TFB4
**C6_03450C_A**	**2.14**	NAM2	NAM2
**CR_01150C_A**	**2.14**	CTF18	CTF18
**C2_02190C_A**	**2.13**		
**C2_07270W_A**	**2.13**		
**C2_09350W_A**	**2.13**	TAZ1	TAZ1
**C3_02870C_A**	**2.13**		
**C4_03140C_A**	**2.13**		IKI1
**C1_04850C_A**	**2.12**		
**C1_13960W_A**	**2.12**	YBP1	YBP1
**C3_01490W_A**	**2.12**	RPS7A	RPS7A
**C5_03100C_A**	**2.12**		
**C7_01210C_A**	**2.12**		RPC37
**C1_00150C_A**	**2.11**	RIM8	RIM8
**C1_01910W_A**	**2.11**		YER156C
**C1_04000C_A**	**2.11**	KTR4	KTR4
**C2_06950C_A**	**2.11**		ATP23
**C3_06630W_A**	**2.11**		SLD3
**C4_02400C_A**	**2.11**		MPS3
**C7_01450C_A**	**2.11**	PRT1	PRT1
**C1_13560W_A**	**2.1**		SAE2
**C2_02120W_A**	**2.1**		MTF1
**C2_08050C_A**	**2.1**	SIT1	ARN1
**C2_10810W_A**	**2.1**		ECM2
**C3_01520C_A**	**2.1**		TMA46
**C4_03800C_A**	**2.1**	NUP84	NUP84
**C4_05570C_A**	**2.1**	TUB4	TUB4
**C5_02670W_A**	**2.1**	BUR2	BUR2
**C5_02820C_A**	**2.1**		MDE1
**C5_05250C_A**	**2.1**		RSM7
**C7_01420W_A**	**2.1**	GIR2	GIR2
**C7_01680C_A**	**2.1**		

**CR_07480W_A**	**2.1**		YBR287W
**C1_00740C_A**	**2.09**	SPE1	SPE1
**C1_06820W_A**	**2.09**		
**C2_03940C_A**	**2.09**	SCH9	SCH9
**C3_04520C_A**	**2.09**	ADE2	ADE2
**C4_05260W_A**	**2.09**		IKI3
**C5_04120C_A**	**2.09**		
**C1_01680C_A**	**2.08**		FYV4
**C1_05380C_A**	**2.08**		RIF1
**C1_09620C_A**	**2.08**		FMC1
**C3_04140C_A**	**2.08**	EGD2	EGD2
**C3_06310C_A**	**2.08**	ISW2	ISW2
**C5_01140C_A**	**2.08**		MSC6
**C6_04520W_A**	**2.08**	ILS1	ILS1
**CR_06740W_A**	**2.08**		SPC97
**CR_07940W_A**	**2.08**		
**CR_08330W_A**	**2.08**		OPI10
**C2_02510W_A**	**2.07**	PRS1	PRS3
**C2_03130W_A**	**2.07**		SFM1
**C3_00280C_A**	**2.07**	ULP3	ULP2
**C4_07130W_A**	**2.07**		
**C7_04150W_A**	**2.07**		RTP1
**C1_08600C_A**	**2.06**	GCD6	GCD6
**C2_01010W_A**	**2.06**	HGT8	
**C2_04400W_A**	**2.06**		
**C2_07260C_A**	**2.06**	DQD1	
**C3_00830C_A**	**2.06**		CDC9
**CR_08360C_A**	**2.06**	RPP2A	RPP2A
**C1_03370W_A**	**2.05**		PAB1
**C1_08090C_A**	**2.05**	FUN12	FUN12
**C1_13060C_A**	**2.05**		MEF1
**C2_01860C_A**	**2.05**		UTR4
**C2_05670C_A**	**2.05**		SEN34
**C2_07160W_A**	**2.05**	SMF12	SMF1
**C3_03070W_A**	**2.05**		
**C3_04670C_A**	**2.05**	RPS15	RPS15
**C3_06620W_A**	**2.05**	FUN31	PSK1
**C4_02680C_A**	**2.05**		
**C4_04130W_A**	**2.05**		COA1
**C6_01070C_A**	**2.05**	CIP1	
**C1_06530C_A**	**2.04**		UBP12
**C1_08460C_A**	**2.04**	UPC2	UPC2
**C1_13410W_A**	**2.04**	COX19	COX19
**C1_14330W_A**	**2.04**		SLM3
**C2_04340C_A**	**2.04**		YDR248C
**C2_07890W_A**	**2.04**	PRS5	PRS5
**C3_06270C_A**	**2.04**		RAD33

**C3_07150C_A**	**2.04**	RPS12	RPS12
**C4_01490W_A**	**2.04**	NIP1	NIP1
**C4_04390W_A**	**2.04**		RPL22B
**CR_08370W_A**	**2.04**	GSH2	GSH2
**C1_08100W_A**	**2.03**	DRG1	RBG1
**C1_08670W_A**	**2.03**	SWI6	SWI6
**C2_06960W_A**	**2.03**	SPE3	SPE3
**C2_07820C_A**	**2.03**	PHO86	PHO86
**C2_08040C_A**	**2.03**	RPS10	RPS10A
**C3_03390C_A**	**2.03**	YAH1	YAH1
**C3_06600C_A**	**2.03**		DML1
**C5_00280C_A**	**2.03**		NUC1
**C5_04270C_A**	**2.03**	SAH1	SAH1
**CR_00480W_A**	**2.03**	MRPL6	MRPL6
**C1_02820W_A**	**2.02**	LYS2	LYS2
**C3_01030W_A**	**2.02**	ARP9	ARP9
**C3_07600W_A**	**2.02**		YCR016W
**C5_04840C_A**	**2.02**		NOP19
**CR_03060W_A**	**2.02**	ARC1	ARC1
**C2_00620C_A**	**2.01**		EFM3
**C2_09290W_A**	**2.01**		VMA21
**C3_05240C_A**	**2.01**	RPL8B	RPL8B
**C4_01680W_A**	**2.01**	TOA2	TOA2
**C5_01130W_A**	**2.01**	CYC3	CYC3
**C6_03350C_A**	**2.01**	ARP4	ARP4
**CR_00510C_A**	**2.01**	ADE1	ADE1
**CR_04780W_A**	**2.01**	RPB8	RPB8
**CR_07360W_A**	**2.01**	PIF1	PIF1
**CR_08090W_A**	**2.01**	SSB1	SSB2
**C1_13010W_A**	**2**		PET309
**C2_00280C_A**	**2**		CWC24
**C2_01220W_A**	**2**		NIC96
**C4_06450W_A**	**2**		IES6
**C5_01930W_A**	**2**		PRP9
**CR_01320C_A**	**2**		YGR125W

S45 vs B45 Upregulated genes

**Gene I D**	**Fold change**	**Standard gene nan**	**Name of *S. cerevisiae* ortholog(s)**
**C5_03440W_A**	**45.64**		
**CR_02920C_A**	**33.65**	AQY1	AQY1
**C1_04750W_A**	**24.32**	IFE1	BDH2
**C2_05070W_A**	**22.96**	FRE9	
**C1_06740C_A**	**22.68**	NOP6	NOP6
**C4_04100C_A**	**21.24**	ECM1	ECM1
**C4_05010W_A**	**21.10**		RSA4
**C2_04120C_A**	**19.39**		NOP12
**C3_01430W_A**	**19.12**		RRT14

**C6_04130C_A**	**18.99**	ALS4	
**C2_07960C_A**	**18.54**	NSA1	NSA1
**C1_05000W_A**	**18.47**	RHD1	
**C1_06760C_A**	**18.34**		IPI3
**CR_03570C_A**	**18.31**	YVH1	YVH1
**CR_06300C_A**	**17.68**	FGR50	
**C2_05620W_A**	**17.64**	KTI11	KTI11
**C7_03540C_A**	**17.52**	ENP2	ENP2
**C1_12440W_A**	**17.45**		REX4
**C6_03250W_A**	**17.09**	BMT4	
**C2_04570W_A**	**16.88**		EBP2
**C5_03530C_A**	**16.70**		COQ10
**CR_04110W_A**	**16.30**		NAF1
**C4_04840C_A**	**16.09**	AGP3	AGP3
**C5_04030W_A**	**15.51**		
**C2_05090W_A**	**15.30**	MAK5	MAK5
**C2_06460W_A**	**15.11**	RTA3	
**C1_00900W_A**	**14.94**	RRS1	RRS1
**C7_00690W_A**	**14.94**	NOP15	NOP15
**C3_03650W_A**	**14.87**	HBR3	NOB1
**CR_08500W_A**	**14.54**		SYO1
**C2_06850W_A**	**14.24**		SLX9
**C7_02340C_A**	**13.98**		TRM82
**C1_12310C_A**	**13.49**	CSI2	BRX1
**C1_02450C_A**	**13.45**		BUD20
**C1_04040C_A**	**13.39**		NOP9
**C1_11420W_A**	**13.25**	KRR1	KRR1
**C7_01030C_A**	**13.24**		RRP12
**C3_02040C_A**	**13.07**		LOC1
**CR_05380C_A**	**12.83**	YCP4	YCP4
**C3_06760W_A**	**12.42**		RRP1
**CR_02980C_A**	**12.31**	SRP40	SRP40
**C2_05160C_A**	**12.30**		PUF6
**CR_09730C_A**	**12.07**	SSF1	SSF1
**CR_03370C_A**	**11.92**	KTI12	KTI12
**CR_05520W_A**	**11.85**	NOC2	NOC2
**CR_08490W_A**	**11.80**	TSR1	TSR1
**C7_04190C_A**	**11.62**	UTP18	UTP18
**C7_01200C_A**	**11.53**	DBP7	DBP7
**C1_10030W_A**	**11.46**	DBP3	DBP3
**C3_01560W_A**	**11.35**		PRP43
**CR_10240W_A**	**11.31**	UTP5	UTP5
**CR_05660W_A**	**11.17**	SDA1	SDA1
**CR_01410C_A**	**11.10**		YNL035C
**CR_04170W_A**	**11.09**		NOP2
**C3_04510W_A**	**11.00**		YBL028C
**C3_02020W_A**	**10.95**		MRD1

**C6_04160C_A**	**10.56**	SPB1	SPB1
**C1_03840W_A**	**10.54**		
**C3_05170W_A**	**10.45**	WOR2	
**C1_12420C_A**	**10.42**		
**C5_01610W_A**	**10.40**		DEG1
**C6_01170W_A**	**10.34**	CIC1	CIC1
**C1_13370W_A**	**10.33**		YDR161W
**C3_00370C_A**	**10.23**	BUD21	BUD21
**C2_00860C_A**	**10.17**		
**C1_10970W_A**	**10.13**		ESF1
**C2_07340W_A**	**10.09**	NOC4	NOC4
**CR_07030C_A**	**10.09**		RRP36
**C3_07300W_A**	**10.03**	NOP13	NOP13
**C3_06160C_A**	**9.94**		
**C4_00510C_A**	**9.94**	RLP24	RLP24
**C3_02130W_A**	**9.92**	UTP4	UTP4
**C6_01180C_A**	**9.88**	EBP1	
**CR_05630W_A**	**9.75**	DBP8	DBP8
**C1_09510W_A**	**9.69**	YTM1	YTM1
**CR_04240C_A**	**9.51**		KRE33
**C1_04390C_A**	**9.46**	NOP4	NOP4
**CR_04750W_A**	**9.43**		YLR363W-A
**C1_09330W_A**	**9.41**		TMA16
**C6_02230W_A**	**9.36**		LSG1
**C1_03060C_A**	**9.34**	TSR2	TSR2
**C3_05160C_A**	**9.33**		DBP10
**C2_09320C_A**	**9.31**	PES1	NOP7
**C5_03250W_A**	**9.24**	RMS1	RKM4
**C4_03830W_A**	**9.22**		TRM3
**C1_08070W_A**	**9.20**	CDR4	
**C5_01430C_A**	**9.19**		RBA50
**C7_02240W_A**	**9.12**	FMP45	FMP45
**C6_02360W_A**	**9.10**	NIP7	NIP7
**C1_03230C_A**	**8.97**	ARX1	ARX1
**C6_03210C_A**	**8.93**		RPC34
**CR_07060C_A**	**8.93**	CRZ2	
**C3_06030W_A**	**8.81**	NOG1	NOG1
**C1_03830C_A**	**8.79**		CSL4
**C5_02550C_A**	**8.79**	UTP13	UTP13
**CR_06720W_A**	**8.75**	NMD3	NMD3
**C2_00410C_A**	**8.70**		ECM16
**C2_09660W_A**	**8.66**		NOP16
**C1_05050C_A**	**8.64**	RRP6	RRP6
**C2_04080W_A**	**8.59**		
**CR_02030C_A**	**8.55**		RCM1
**C5_01600C_A**	**8.50**	SPB4	SPB4
**C7_04230W_A**	**8.44**	NRG1	NRG2

**C7_00570W_A**	**8.43**	RPA135	RPA135
**C1_06570W_A**	**8.38**	ANT1	ANT1
**C3_01000W_A**	**8.37**	BMS1	BMS1
**C3_00800W_A**	**8.35**	MIH1	MIH1
**C3_06380W_A**	**8.31**	NSA2	NSA2
**CR_10550W_A**	**8.31**	DRS1	DRS1
**C5_04750C_A**	**8.27**	HAS1	HAS1
**C3_04560W_A**	**8.26**	MAK21	MAK21
**C4_06720W_A**	**8.25**	NOP1	NOP1
**C1_07880C_A**	**8.21**	GCS1	GSH1
**C6_01890C_A**	**8.14**		DBP9
**C1_00160C_A**	**8.10**		NSR1
**C6_04380W_A**	**7.98**	ALS2	
**C4_04810C_A**	**7.93**		TRM8
**C1_11000C_A**	**7.88**		CGR1
**C2_02540W_A**	**7.87**		RRP5
**CR_02420W_A**	**7.82**		UTP23
**C1_05480C_A**	**7.75**		
**C2_08200W_A**	**7.74**		
**C4_06210C_A**	**7.74**		NUG1
**C1_06440C_A**	**7.73**		
**C1_13480W_A**	**7.73**	HSP70	SSA4
**C4_00810C_A**	**7.73**		QRI7
**C6_01440C_A**	**7.72**	FRP5	
**CR_06550C_A**	**7.71**		
**C2_10240W_A**	**7.70**	GPD1	GPD1
**C1_01110C_A**	**7.69**	ZPR1	ZPR1
**C1_07950C_A**	**7.62**		RRP17
**C2_06700W_A**	**7.61**	AMO2	
**C3_02350W_A**	**7.58**		KRI1
**CR_05620C_A**	**7.50**	MTG2	MTG2
**C2_08480W_A**	**7.46**	RRP8	RRP8
**CR_03940W_A**	**7.45**		IPI1
**C1_08040W_A**	**7.42**		
**C6_00480C_A**	**7.42**	FET3	
**CR_10470C_A**	**7.41**		BFR2
**C2_09500W_A**	**7.37**		DUS1
**C4_02260C_A**	**7.34**		YMR310C
**C1_09710C_A**	**7.26**		UTP7
**C7_01520W_A**	**7.25**	FLU1	TPO1
**CR_03650W_A**	**7.24**	SGD1	SGD1
**C4_02720C_A**	**7.21**		SFK1
**CR_03550W_A**	**7.21**	NCS2	NCS2
**C2_03690C_A**	**7.17**		
**C2_05080C_A**	**7.15**		BMT2
**C6_00370C_A**	**7.11**	NOP5	NOP58
**C1_07990C_A**	**7.10**		

**C7_00160C_A**	**7.01**		ALB1
**CR_09370W_A**	**6.95**	ELF1	NEW1
**C2_05230C_A**	**6.88**	RPF2	RPF2
**CR_08940W_A**	**6.86**		GCD14
**C5_03920C_A**	**6.83**		FCF1
**C1_14080W_A**	**6.81**		UTP10
**CR_02520W_A**	**6.80**	RPC19	RPC19
**C2_00070C_A**	**6.78**	MPP10	MPP10
**C5_01060C_A**	**6.78**	BUD23	BUD23
**C6_03710W_A**	**6.77**	ALS9	FLO1
**C2_02420C_A**	**6.75**		DUS3
**C4_04500C_A**	**6.66**		EFM5
**C3_05230W_A**	**6.45**	SAP3	
**C7_04090C_A**	**6.42**		
**C2_01050W_A**	**6.36**		
**C2_09990C_A**	**6.36**	YOR1	YOR1
**CR_00680W_A**	**6.33**	BUD22	BUD22
**C1_12680W_A**	**6.30**		RRB1
**C1_14160W_A**	**6.30**		RKM3
**C1_08810C_A**	**6.29**	MSS116	MSS116
**C2_09380W_A**	**6.27**	RRP15	RRP15
**C6_01420C_A**	**6.26**		
**CR_09800C_A**	**6.20**		UTP14
**C1_04330W_A**	**6.15**	RPN4	RPN4
**C4_04320W_A**	**6.15**	FRE10	
**CR_03760W_A**	**6.15**		NNT1
**C3_04370C_A**	**6.10**		URB2
**C1_09550W_A**	**6.09**		
**C4_05650W_A**	**6.09**		FAF1
**C1_01150C_A**	**6.06**		PPM2
**C1_01750W_A**	**6.05**		YCR023C
**C3_05570W_A**	**6.00**	QDR2	
**C5_00910C_A**	**6.00**	TRX2	
**C3_06370C_A**	**5.95**		LCP5
**C4_02850W_A**	**5.91**		ELP2
**CR_01950W_A**	**5.86**		RPA43
**C3_06390W_A**	**5.85**		
**C5_05340W_A**	**5.85**		SQT1
**CR_05390W_A**	**5.83**	PST3	PST2
**C1_06810W_A**	**5.77**	CAT1	CTA1
**C2_10740C_A**	**5.75**		EMW1
**C5_02120C_A**	**5.75**	RIX7	RIX7
**CR_10410C_A**	**5.72**		PNO1
**CR_00900W_A**	**5.69**	TRM1	TRM1
**C3_01680C_A**	**5.68**		YDL183C
**CR_03360W_A**	**5.67**		UTP11
**C1_13870W_A**	**5.66**	MET3	MET3

**C2_08000C_A**	**5.66**	KRE30	ARB1
**C1_03390W_A**	**5.63**	EHT1	EHT1
**C2_06480W_A**	**5.63**		TRM10
**C2_10270W_A**	**5.63**	PUS7	PUS7
**C3_04200W_A**	**5.63**	AFP99	
**C4_01450W_A**	**5.60**	PWP1	PWP1
**C3_07070C_A**	**5.55**		
**C4_06710W_A**	**5.54**		
**C3_05140C_A**	**5.53**		TRM11
**C1_01160C_A**	**5.49**		RRP40
**C2_05660W_A**	**5.48**	PNG2	
**C4_02050W_A**	**5.48**	HGH1	HGH1
**C2_10810W_A**	**5.45**		ECM2
**C3_03470W_A**	**5.44**		UGA2
**CR_00080W_A**	**5.41**	MIS12	
**C3_05460W_A**	**5.35**		
**C1_10360C_A**	**5.32**		YCR061W
**CR_07470W_A**	**5.32**		DSS4
**CR_07750C_A**	**5.32**		DBP6
**C2_06890C_A**	**5.22**		
**C4_03110W_A**	**5.21**		DNF3
**C5_00920W_A**	**5.17**		DIB1
**C2_07450C_A**	**5.16**	RCL1	RCL1
**C6_04530C_A**	**5.09**		RTT10
**CR_07480W_A**	**5.06**		YBR287W
**C6_01040C_A**	**5.04**		DHR2
**C4_04470W_A**	**5.00**	SAP10	
**CR_07950W_A**	**4.98**	IMP4	IMP4
**C7_04140C_A**	**4.97**		YAR1
**C2_10100W_A**	**4.96**	CWC22	CWC22
**C4_02990C_A**	**4.96**	GST2	
**C5_00680W_A**	**4.96**	RMT2	RMT2
**C1_01220C_A**	**4.95**		
**C4_07050W_A**	**4.95**	YDC1	YDC1
**CR_04160C_A**	**4.94**		GCD10
**CR_01780W_A**	**4.93**		BMT6
**CR_08340W_A**	**4.93**	CYS3	CYS3
**CR_00460C_A**	**4.92**		IMP3
**CR_06590C_A**	**4.92**	RGS2	RGS2
**C2_00080C_A**	**4.91**	FAV3	
**C2_08840W_A**	**4.91**		TRM112
**C3_02180C_A**	**4.90**		DYS1
**C6_02350C_A**	**4.87**		PUS1
**C2_08820C_A**	**4.86**	SPO11	SPO11
**C4_06620C_A**	**4.86**		
**C4_03840C_A**	**4.85**	RRP42	RRP42
**C4_02080W_A**	**4.84**		

**C1_05230W_A**	**4.82**		RPC25
**CR_08890C_A**	**4.81**	ASR2	
**C2_07020C_A**	**4.80**		
**C4_00750C_A**	**4.78**		
**C3_01150C_A**	**4.74**		APD1
**C4_00690C_A**	**4.74**		DPH2
**C6_02940C_A**	**4.73**		
**C1_13940W_A**	**4.72**	SIM1	UTH1
**C3_04860W_A**	**4.72**		SFP1
**CR_06120W_A**	**4.71**	RPL7	RLP7
**C1_10620W_A**	**4.67**		CBF5
**C2_05340C_A**	**4.67**	RPC40	RPC40
**CR_03710C_A**	**4.67**		
**C1_08870C_A**	**4.66**	JIP5	JIP5
**C2_02980C_A**	**4.65**	DLD1	
**CR_06680C_A**	**4.64**		ECM32
**C1_12570C_A**	**4.60**		ELP4
**C4_03900C_A**	**4.60**	RAM1	RAM1
**C7_00330C_A**	**4.59**		SUA5
**C3_00330W_A**	**4.57**	UTP8	UTP8
**C6_03180C_A**	**4.56**		ALY2
**CR_10750C_A**	**4.55**		YCR087C-A
**C1_02800W_A**	**4.54**		
**CR_06330C_A**	**4.53**		YML108W
**C1_02190W_A**	**4.51**	ABP140	ABP140
**C2_01070W_A**	**4.51**		RPA49
**C4_05700W_A**	**4.49**	ZCF5	
**C3_07340W_A**	**4.47**	GCY1	GCY1
**CR_05550C_A**	**4.46**		RET1
**C2_06000W_A**	**4.41**	LAS1	LAS1
**C3_06830C_A**	**4.40**		YLR419W
**C6_03390W_A**	**4.40**		DIC1
**C1_09810W_A**	**4.39**		YGR210C
**CR_09950C_A**	**4.39**	SIK1	NOP56
**C4_04140W_A**	**4.37**		
**C5_00310C_A**	**4.36**		
**C4_06790W_A**	**4.35**		GPN3
**CR_02530W_A**	**4.35**	DBP2	DBP2
**C1_00560W_A**	**4.33**	CNS1	CNS1
**CR_07820W_A**	**4.32**		
**C2_07130C_A**	**4.31**	RCK2	RCK2
**C5_03470C_A**	**4.30**		
**C6_02420W_A**	**4.30**		PGC1
**C7_01670W_A**	**4.30**		
**C7_01360C_A**	**4.25**		ZIM17
**C2_07920W_A**	**4.24**		RRT2
**C6_02770W_A**	**4.24**	MRT4	MRT4

**C3_03450C_A**	**4.22**	BMT7	
**C4_03190W_A**	**4.21**		
**C2_09260C_A**	**4.20**	GLO2	GLO2
**C1_13130C_A**	**4.18**		
**C2_05960C_A**	**4.17**		
**C3_05800W_A**	**4.17**		SEN54
**C1_12670C_A**	**4.15**		PRP6
**C3_07750W_A**	**4.15**	UTP9	UTP9
**C4_03350C_A**	**4.15**		
**C1_05510C_A**	**4.13**	RPS27A	RPS27A
**C1_05840W_A**	**4.13**	PRN1	
**C4_00970C_A**	**4.13**	MDN1	MDN1
**C3_01520C_A**	**4.12**		TMA46
**C6_04320C_A**	**4.12**		
**C2_03830W_A**	**4.11**		DPH6
**C1_04130W_A**	**4.08**	ERB1	ERB1
**C2_03040W_A**	**4.08**	PLC2	
**C4_02300W_A**	**4.04**	HOS3	HOS3
**C1_01530C_A**	**4.03**		YDR341C
**C1_09490C_A**	**4.03**	GUA1	GUA1
**C1_12600C_A**	**4.00**	CHR1	ROK1
**C2_05970C_A**	**4.00**	TES1	
**C3_07400W_A**	**3.99**		TRM7
**C4_03740W_A**	**3.99**		RRP4
**C5_03010W_A**	**3.97**		SNU66
**CR_09960C_A**	**3.97**	UGA3	UGA3
**CR_00740C_A**	**3.96**	BMT3	
**C1_05520W_A**	**3.95**		
**C1_07330W_A**	**3.94**	RME1	RME1
**C6_04050W_A**	**3.94**		
**C4_01080W_A**	**3.93**	RAT1	RAT1
**C1_06770W_A**	**3.92**		YNL181W
**C2_06930C_A**	**3.90**		
**C4_06470W_A**	**3.90**		YMR295C
**C6_01870C_A**	**3.90**		YHK8
**C1_01910W_A**	**3.88**		YER156C
**C1_06540C_A**	**3.86**		RRP43
**C4_06390W_A**	**3.86**	SOU1	
**C6_04200C_A**	**3.86**		
**C6_00530C_A**	**3.85**		SME1
**C1_11700C_A**	**3.84**	MRF1	
**C5_02010C_A**	**3.84**		BUD27
**C2_06470W_A**	**3.83**	RTA2	RSB1
**C1_02090C_A**	**3.82**		DRN1
**C1_13720W_A**	**3.82**		KEL3
**C3_00100W_A**	**3.82**		YJU2
**C5_02280C_A**	**3.81**		CTR86

**C1_05360C_A**	**3.80**		PAP2
**C1_10780C_A**	**3.80**	HNT1	HNT1
**C2_02510W_A**	**3.79**	PRS1	PRS3
**C2_07300C_A**	**3.79**	RPA12	RPA12
**C3_05930W_A**	**3.78**	CTA4	OAF1
**CR_02890C_A**	**3.78**		RPC82
**C1_08630W_A**	**3.76**		MTF2
**C7_02490W_A**	**3.76**	PEX1	PEX1
**CR_00670C_A**	**3.76**		TRM13
**C5_04350C_A**	**3.74**		FSH3
**C2_04820W_A**	**3.73**		MRM1
**C3_03870C_A**	**3.72**	SAP9	YPS1
**CR_08100C_A**	**3.72**	RIO2	RIO2
**CR_09140C_A**	**3.71**		MSC1
**C6_03610W_A**	**3.70**	FGR17	
**C5_01480W_A**	**3.69**	FYV5	FYV7
**C6_02910W_A**	**3.69**	POP4	POP4
**C1_10110W_A**	**3.68**		
**CR_07160C_A**	**3.68**		
**C4_02870C_A**	**3.67**	ZUO1	ZUO1
**C6_00290W_A**	**3.67**		
**C1_11910W_A**	**3.66**		THP1
**C7_02460C_A**	**3.66**		NPA3
**CR_00800C_A**	**3.66**	PWP2	PWP2
**C1_05330C_A**	**3.63**		YDR306C
**CR_04920W_A**	**3.63**		YDL119C
**C1_13330C_A**	**3.62**		SPE4
**C4_00540C_A**	**3.59**	NMD5	NMD5
**C3_00560C_A**	**3.58**	SOF1	SOF1
**C2_02740C_A**	**3.57**	HPT1	HPT1
**C3_06850W_A**	**3.56**	FCR1	
**C5_04990W_A**	**3.56**		RPC17
**C6_03630W_A**	**3.56**	PAD1	PAD1
**C2_00650W_A**	**3.52**		
**C1_05640C_A**	**3.47**	FAL1	FAL1
**C2_01010W_A**	**3.45**	HGT8	
**C4_06410W_A**	**3.44**		SYF1
**C1_11200W_A**	**3.43**		
**CR_01700C_A**	**3.42**		PRP24
**C3_07100C_A**	**3.41**		NGR1
**C4_00490W_A**	**3.41**		
**CR_10690W_A**	**3.41**	POP3	POP3
**C6_04260C_A**	**3.38**		JLP2
**C2_03940C_A**	**3.37**	SCH9	SCH9
**C7_03930C_A**	**3.37**	MAM33	MAM33
**C1_01030W_A**	**3.35**	HMT1	HMT1
**C1_04350C_A**	**3.35**		

**C4_06990W_A**	**3.35**		
**C1_11550W_A**	**3.33**	GAR1	GAR1
**C2_02900W_A**	**3.33**		
**C4_00940W_A**	**3.32**		ELP6
**C1_12760W_A**	**3.31**		BCP1
**C2_06390C_A**	**3.31**	IMH3	IMD4
**C2_09000C_A**	**3.30**		
**C4_07060W_A**	**3.30**		RPO31
**C6_02560W_A**	**3.28**		YMR315W
**C2_06750C_A**	**3.27**		
**C3_01230C_A**	**3.27**		
**C3_02460C_A**	**3.27**	PHA2	PHA2
**C4_04550C_A**	**3.27**		OMA1
**C2_01060C_A**	**3.26**		JJJ3
**C3_07600W_A**	**3.26**		YCR016W
**C3_07670W_A**	**3.26**		YDR090C
**C4_07140W_A**	**3.26**		UPS2
**C7_00880C_A**	**3.26**		
**CR_06770C_A**	**3.26**		
**CR_06840W_A**	**3.26**		UTP25
**C2_07410W_A**	**3.25**		YMR210W
**C1_06120C_A**	**3.24**		
**CR_09150W_A**	**3.24**		
**C2_07070W_A**	**3.23**		ZTA1
**C6_03840C_A**	**3.23**	SNQ2	SNQ2
**C3_04380C_A**	**3.21**		SNU13
**CR_08020C_A**	**3.21**	NOP10	NOP10
**C1_00990C_A**	**3.20**	NMA111	NMA111
**C1_05210C_A**	**3.19**		YNR040W
**C2_02600C_A**	**3.18**	FGR22	
**C2_00880W_A**	**3.17**		
**C7_00380W_A**	**3.17**		PET8
**C3_05070W_A**	**3.16**	CEM1	CEM1
**C1_12140W_A**	**3.15**		
**C3_06720W_A**	**3.15**	PRP5	PRP5
**C1_07500C_A**	**3.14**	LHP1	LHP1
**C1_07710C_A**	**3.13**	ADE4	ADE4
**C1_04740W_A**	**3.12**	LPG20	YPL088W
**C1_07140C_A**	**3.12**	RIA1	RIA1
**C5_01110W_A**	**3.12**		OTU2
**CR_02060W_A**	**3.12**		
**C2_03130W_A**	**3.11**		SFM1
**C4_06540W_A**	**3.11**	MNN4	
**C5_04580C_A**	**3.11**		VPS25
**C3_05380W_A**	**3.10**		YJL163C
**C1_00340W_A**	**3.08**	HBR1	FAP7
**C3_06230W_A**	**3.08**	CYM1	CYM1

**C5_01180W_A**	**3.08**	PUS4	PUS4
**C7_00490C_A**	**3.08**		TAE1
**CR_04420C_A**	**3.08**	RBR2	TIP1
**C5_03360W_A**	**3.07**	RPO26	RPO26
**C7_03230C_A**	**3.07**		
**CR_09670C_A**	**3.07**		YJL068C
**C2_01410C_A**	**3.05**	CLB2	CLB2
**C2_04230W_A**	**3.05**	BAT21	BAT2
**C6_00590W_A**	**3.05**	SLY41	SLY41
**C1_09170W_A**	**3.04**	CTA8	HSF1
**C6_00760W_A**	**3.04**		FES1
**C7_02100W_A**	**3.04**		
**C1_02840W_A**	**3.03**	PDE2	PDE2
**C3_00750W_A**	**3.03**	LYS144	
**C3_05860C_A**	**3.03**		BMT5
**C4_06780C_A**	**3.03**	OYE32	
**C5_00280C_A**	**3.03**		NUC1
**C4_01010C_A**	**3.02**	DAG7	
**C6_04300W_A**	**3.02**		
**CR_08580C_A**	**3.02**		
**C2_00170C_A**	**3.01**		TCD2
**C2_01880C_A**	**3.01**	ZCF6	
**C7_03210W_A**	**3.01**		
**C1_12350W_A**	**3.00**		DIS3
**C4_03150W_A**	**3.00**		HSH155
**C5_03390C_A**	**3.00**	FUR1	FUR1
**C5_03640W_A**	**3.00**		
**C6_03200W_A**	**2.99**		
**CR_05570C_A**	**2.99**	RNR3	
**C1_05650W_A**	**2.97**		DAS2
**C2_09900C_A**	**2.97**	TIM23	TIM23
**C3_04520C_A**	**2.96**	ADE2	ADE2
**C1_00220W_A**	**2.95**	PHR2	GAS1
**C1_09440W_A**	**2.95**		PPA2
**C4_01280C_A**	**2.95**		PRP21
**CR_05990C_A**	**2.93**	SFL1	SFL1
**C1_01610C_A**	**2.92**		
**C5_03700C_A**	**2.92**		DPH5
**CR_06980W_A**	**2.92**		LTO1
**CR_08330W_A**	**2.89**		OPI10
**CR_08920W_A**	**2.89**		
**C2_07360W_A**	**2.88**		TGS1
**C4_05770C_A**	**2.88**	CFL1	
**C5_03970W_A**	**2.88**		
**C7_00310C_A**	**2.88**		
**C1_07690C_A**	**2.87**		CSE1
**C2_02710C_A**	**2.87**		RRP7

**C1_06560W_A**	**2.86**		YGR117C
**C7_02930C_A**	**2.86**		TSR4
**C4_00480W_A**	**2.84**	DUR4	
**C4_01470W_A**	**2.84**		HRT3
**C3_07760C_A**	**2.83**		
**C5_02070C_A**	**2.83**		ESF2
**C2_02960C_A**	**2.82**		EFM2
**C2_03020C_A**	**2.82**		YJL016W
**C4_03050C_A**	**2.82**		CPS1
**C6_01300W_A**	**2.82**		CST26
**CR_03110W_A**	**2.82**		MSE1
**C2_03030W_A**	**2.81**	LIG4	DNL4
**CR_06570C_A**	**2.81**		
**C4_00360C_A**	**2.80**	RCH1	YMR034C
**CR_04300W_A**	**2.80**		NCL1
**C4_04700W_A**	**2.79**	SSZ1	SSZ1
**CR_07640C_A**	**2.79**		VFA1
**C1_13470W_A**	**2.78**	KNS1	KNS1
**C3_00950C_A**	**2.77**		CPR7
**C3_07550C_A**	**2.77**		GEP3
**C4_07030W_A**	**2.77**	MET16	MET16
**CR_03750C_A**	**2.77**	PRP42	PRP42
**C1_03200C_A**	**2.76**	ARO80	ARO80
**C1_13270W_A**	**2.75**		POF1
**C3_04480C_A**	**2.75**	RAS2	
**C1_04140W_A**	**2.74**	IFD6	
**C3_00480C_A**	**2.74**	DOT5	DOT5
**C1_00500C_A**	**2.73**	GLO1	GLO1
**C1_01240W_A**	**2.73**	IFD3	
**C1_04720W_A**	**2.73**		
**CR_03230W_A**	**2.73**		SQS1
**C1_09610W_A**	**2.72**		
**C6_01000C_A**	**2.72**		AEP1
**C1_06380C_A**	**2.71**		
**C1_06800W_A**	**2.71**		RAI1
**C2_07000W_A**	**2.71**		YCR051W
**C3_05980C_A**	**2.71**	FAA2	
**C5_02430W_A**	**2.71**	ZCF22	
**CR_10270C_A**	**2.71**	AHA1	AHA1
**C5_00260W_A**	**2.70**		PRS1
**C1_03400W_A**	**2.69**		ART5
**C2_04640C_A**	**2.69**	SNU114	SNU114
**C3_03680W_A**	**2.69**		DPL1
**CR_02720C_A**	**2.69**	RPC31	RPC31
**CR_05750W_A**	**2.69**		
**CR_06000W_A**	**2.69**	SOK1	SOK1
**C1_09400C_A**	**2.68**	FTH1	FTH1

**C2_08620W_A**	**2.68**		YPL191C
**C5_03860W_A**	**2.68**	HAM1	HAM1
**C1_00150C_A**	**2.67**	RIM8	RIM8
**C1_01900C_A**	**2.67**		TMA64
**C1_11220C_A**	**2.67**	TIM50	TIM50
**C2_03200W_A**	**2.66**	PRP3	PRP3
**C3_03550C_A**	**2.66**	ULP1	
**C6_02500C_A**	**2.66**	GCV1	GCV1
**C6_01690W_A**	**2.65**	ACF2	ACF2
**C1_02620C_A**	**2.64**	HOM6	HOM6
**C4_01490W_A**	**2.64**	NIP1	NIP1
**C1_05220C_A**	**2.63**		
**C3_07380W_A**	**2.63**		YBR062C
**C5_04570C_A**	**2.63**	URA7	URA7
**CR_07250C_A**	**2.63**		
**C1_03790C_A**	**2.62**		FPR3
**C3_02570W_A**	**2.62**		YIL108W
**CR_08000C_A**	**2.62**		
**C3_06100C_A**	**2.61**	DED1	DED1
**C3_06860C_A**	**2.61**		YJR096W
**C4_06850C_A**	**2.61**		MAP1
**C1_01470W_A**	**2.60**		NAB2
**C1_08090C_A**	**2.60**	FUN12	FUN12
**C2_05440W_A**	**2.60**	PEX6	PEX6
**C4_03720C_A**	**2.60**		
**C6_03480W_A**	**2.60**	YMX6	
**C7_00590W_A**	**2.58**		
**C2_06070W_A**	**2.57**	FAA2-1	
**CR_08320W_A**	**2.57**	ATS1	ATS1
**C3_03330C_A**	**2.55**		CLF1
**C4_03730C_A**	**2.55**		TRM5
**C6_01980C_A**	**2.55**		MRPS18
**CR_07780W_A**	**2.55**		
**C1_03370W_A**	**2.54**		PAB1
**C2_01920C_A**	**2.54**		
**C3_03070W_A**	**2.54**		
**C5_03550W_A**	**2.54**		NUP2
**CR_09930W_A**	**2.54**		YDR124W
**C2_02690W_A**	**2.53**	SER2	SER2
**C1_07090C_A**	**2.52**		RNH70
**C2_10640C_A**	**2.52**	MTR2	MTR2
**C4_01680W_A**	**2.52**	TOA2	TOA2
**C5_01930W_A**	**2.52**		PRP9
**C2_00220C_A**	**2.51**		TFG2
**C3_05790C_A**	**2.51**		SGF11
**C4_05260W_A**	**2.51**		IKI3
**C1_02310C_A**	**2.50**		GON7

**C1_11020W_A**	**2.50**		
**C3_07620C_A**	**2.50**	RIT1	RIT1
**C2_03090C_A**	**2.49**	ADE8	ADE8
**C2_00810C_A**	**2.48**		
**C2_09140C_A**	**2.48**	MET10	MET10
**C1_14130W_A**	**2.47**	FTR1	FTR1
**C2_01510C_A**	**2.47**		TAD2
**C5_02490C_A**	**2.47**	TIF5	TIF5
**CR_04050C_A**	**2.47**	MEX67	MEX67
**C2_06810C_A**	**2.46**	RPL11	RPL11B
**C2_08050C_A**	**2.46**	SIT1	ARN1
**C6_02730W_A**	**2.46**	SAC6	SAC6
**CR_09310W_A**	**2.46**		RKM1
**C4_02880C_A**	**2.45**		SDO1
**CR_04740C_A**	**2.45**	ADE6	ADE6
**C1_11560C_A**	**2.44**		MRX8
**C2_05810W_A**	**2.44**		BOP3
**C4_00160C_A**	**2.44**	CAR2	CAR2
**C4_03160C_A**	**2.44**		
**C1_08600C_A**	**2.43**	GCD6	GCD6
**C1_13400C_A**	**2.43**	AGC1	AGC1
**C2_00280C_A**	**2.43**		CWC24
**C2_09930W_A**	**2.43**		UBX4
**C6_00640C_A**	**2.43**		TFB4
**C7_01220W_A**	**2.43**	GCN3	GCN3
**C1_02910C_A**	**2.42**		ATG17
**C1_07510W_A**	**2.42**		YBL036C
**C2_03700W_A**	**2.42**		NTA1
**C1_02440C_A**	**2.41**		OTU1
**C1_03240W_A**	**2.41**		
**C4_01130C_A**	**2.41**		VPS71
**CR_02430C_A**	**2.41**		FAP1
**C5_00140C_A**	**2.39**		YHR140W
**C2_02880C_A**	**2.38**	EBP7	
**C2_06020W_A**	**2.38**	CNT	
**C2_09750W_A**	**2.38**	LEU42	
**C1_01770W_A**	**2.37**	MAK32	MAK32
**C2_00100C_A**	**2.37**	PGA52	YJL171C
**C5_01910W_A**	**2.37**	GIS2	GIS2
**C1_02880C_A**	**2.36**	MIA40	MIA40
**C1_09060C_A**	**2.36**		FUN14
**C7_03220C_A**	**2.36**	ZCF29	
**CR_04360C_A**	**2.36**	NHP2	NHP2
**CR_08130W_A**	**2.36**		ATP10
**C4_05100C_A**	**2.35**	MYO5	MYO5
**C6_03750C_A**	**2.35**	SBA1	SBA1
**C6_03800C_A**	**2.35**		IZH3

**C7_01940C_A**	**2.35**		PRM10
**C2_08240W_A**	**2.34**	ORF298	
**CR_09990W_A**	**2.34**		
**C1_11370C_A**	**2.33**		LCD1
**C4_04090C_A**	**2.33**		CNE1
**C2_04480W_A**	**2.32**		
**C2_06960W_A**	**2.32**	SPE3	SPE3
**C6_01160W_A**	**2.32**	RCN1	
**CR_03630W_A**	**2.32**	IFF3	
**C2_09950W_A**	**2.31**	FCY21	FCY2
**C5_00290W_A**	**2.31**	DUS4	DUS4
**C5_02730C_A**	**2.31**		CIA1
**C6_03330C_A**	**2.31**		YPR097W
**C7_02020W_A**	**2.31**	RAD14	RAD14
**C1_00520W_A**	**2.30**		YOR283W
**CR_07080W_A**	**2.30**		TIF6
**C1_00510W_A**	**2.29**		ECM31
**C1_10220C_A**	**2.29**	TPK1	
**C2_03990W_A**	**2.29**	GCD7	GCD7
**C4_01670C_A**	**2.29**		TAP42
**C5_01940W_A**	**2.29**		RIB2
**C7_02290W_A**	**2.29**		
**C1_08100W_A**	**2.28**	DRG1	RBG1
**C3_02030W_A**	**2.28**		PCP1
**C3_04220C_A**	**2.28**	URK1	URK1
**C3_05310W_A**	**2.28**	USO6	
**C4_04720W_A**	**2.28**		MTD1
**C6_03690W_A**	**2.28**	ALS5	
**CR_10400W_A**	**2.28**		ARD1
**C2_05490W_A**	**2.27**		
**C2_09170W_A**	**2.27**	SLP2	
**C5_05430W_A**	**2.27**	PEX4	PEX4
**C1_12410C_A**	**2.26**	PKH2	PKH2
**C3_07290W_A**	**2.26**		OCA5
**C4_05400C_A**	**2.26**		
**C1_10450W_A**	**2.25**	GLY1	
**C2_00960C_A**	**2.25**	MXR1	MXR1
**C2_06170C_A**	**2.25**	ECM17	MET5
**CR_10170C_A**	**2.25**		ASP1
**C1_06910C_A**	**2.24**		CAF130
**C1_13260W_A**	**2.24**		FPR2
**C1_14490C_A**	**2.24**	SAM35	SAM35
**C2_07570W_A**	**2.24**	RNR22	
**C2_07890W_A**	**2.24**	PRS5	PRS5
**C1_00530C_A**	**2.23**		
**C1_05930C_A**	**2.23**		DUG2
**C1_10340W_A**	**2.23**		

**C1_13160W_A**	**2.23**	PSA2	
**C2_08860W_A**	**2.23**		FPK1
**C3_05590C_A**	**2.23**	BAT22	
**C3_07430W_A**	**2.23**		DET1
**C4_01840C_A**	**2.23**		AIM2
**C6_02590C_A**	**2.23**	MOB1	MOB1
**CR_02460W_A**	**2.23**		
**C3_00890C_A**	**2.22**		
**C4_04510W_A**	**2.22**		TFC3
**C5_03680W_A**	**2.22**	FGR27	
**C1_01520C_A**	**2.21**	SOD2	SOD2
**C2_05520W_A**	**2.21**		EFM4
**C6_04590C_A**	**2.20**	NAG1	
**C1_04990C_A**	**2.19**		
**C1_07390W_A**	**2.19**		UBX5
**C2_00140W_A**	**2.19**	NDT80	NDT80
**C2_04520C_A**	**2.19**		
**C2_08090W_A**	**2.19**		
**C5_03740W_A**	**2.19**		
**CR_09240C_A**	**2.19**		YPL225W
**C1_04340C_A**	**2.18**		YMR010W
**C2_04190C_A**	**2.18**	UGA1	UGA1
**C2_10770W_A**	**2.18**	RPC11	RPC11
**C4_00700C_A**	**2.18**		
**C4_02620C_A**	**2.18**		YDR109C
**CR_04780W_A**	**2.18**	RPB8	RPB8
**CR_08370W_A**	**2.18**	GSH2	GSH2
**C1_03110W_A**	**2.17**	RPL6	RPL6B
**C1_11900C_A**	**2.17**		UPF3
**C2_08460C_A**	**2.17**		
**C4_07100C_A**	**2.17**		BRR2
**C1_00550W_A**	**2.16**	CIS2	ECM38
**C1_07890C_A**	**2.16**	ADE5,7	ADE5,7
**C1_09000W_A**	**2.16**		YTP1
**C1_10410W_A**	**2.16**		
**C1_01490W_A**	**2.15**		
**C7_03990C_A**	**2.15**		TAN1
**CR_08180C_A**	**2.15**	TRM9	TRM9
**C1_03300C_A**	**2.14**	MAS1	MAS1
**C1_09390W_A**	**2.14**		MOD5
**C2_00620C_A**	**2.14**		EFM3
**C3_06060W_A**	**2.14**	ELP3	ELP3
**C4_05810W_A**	**2.14**		CAJ1
**CR_00590W_A**	**2.14**	PHO81	PHO81
**C1_13910C_A**	**2.13**	MODF	YDR061W
**C2_03910C_A**	**2.13**		MNL1
**C3_06340W_A**	**2.13**	HNT2	HNT2

**C3_06680C_A**	**2.13**		
**C3_07480W_A**	**2.13**		PRP19
**CR_00980C_A**	**2.13**		CAB3
**CR_09110C_A**	**2.13**		PGA3
**C1_01130W_A**	**2.12**		MET30
**C1_07240W_A**	**2.12**	YPD1	YPD1
**C2_07610C_A**	**2.12**		RQC1
**C3_06150W_A**	**2.12**		YPR022C
**C5_00950C_A**	**2.12**		SET5
**C6_02480W_A**	**2.12**		ADH7
**C1_05560W_A**	**2.11**	RIB4	RIB4
**C1_06110C_A**	**2.11**	NPL4	NPL4
**C2_04930C_A**	**2.11**	CRK1	SGV1
**C2_05150W_A**	**2.11**	PEX2	PEX2
**C4_04630C_A**	**2.11**		
**C7_03360W_A**	**2.11**	CPY1	PRC1
**CR_08660W_A**	**2.11**	PRP8	PRP8
**C1_02430C_A**	**2.10**		YGR054W
**C1_03430W_A**	**2.10**		TGL2
**C1_05540C_A**	**2.10**		
**C1_07290W_A**	**2.10**		
**C2_10190C_A**	**2.10**	OCA1	OCA1
**C3_01140W_A**	**2.10**		
**C3_02870C_A**	**2.10**		
**C5_00180W_A**	**2.10**		VIP1
**CR_07700W_A**	**2.10**		
**C1_11010C_A**	**2.09**		PEX32
**C2_09600C_A**	**2.09**		YCR090C
**C3_05510W_A**	**2.09**		
**C7_02330W_A**	**2.09**	YCF1	YCF1
**CR_02510W_A**	**2.09**		AZF1
**CR_03990C_A**	**2.09**	GCD1	GCD1
**CR_08090W_A**	**2.09**	SSB1	SSB2
**C4_02740W_A**	**2.08**		
**C5_03670C_A**	**2.08**		
**C5_04610W_A**	**2.08**		YJR084W
**C6_02410W_A**	**2.08**		NPY1
**CR_05890C_A**	**2.08**		VPS75
**CR_10430C_A**	**2.08**		SPP2
**C1_05630C_A**	**2.07**		KAP123
**C3_06940W_A**	**2.07**		
**C6_00660C_A**	**2.07**		AIM7
**C1_01100W_A**	**2.06**	CCH1	CCH1
**C1_06790C_A**	**2.06**	MRPL19	MRPL19
**C1_11660W_A**	**2.06**	GAD1	GAD1
**C2_01870C_A**	**2.06**		
**C2_03360W_A**	**2.06**		AMD1

**C2_07380W_A**	**2.06**	SSC1	SSC1
**C2_09880C_A**	**2.06**		YOR338W
**C3_01350C_A**	**2.06**	URA3	URA3
**C4_05590W_A**	**2.06**		YPR117W
**C6_03760C_A**	**2.06**	SHM2	SHM2
**CR_00210W_A**	**2.06**	ALK2	
**CR_08250C_A**	**2.06**	HSP104	HSP104
**C1_07570C_A**	**2.05**		
**C2_10710W_A**	**2.05**	TIF11	TIF11
**C5_01640W_A**	**2.05**	MAS2	MAS2
**C5_05190W_A**	**2.05**	PCL5	PCL5
**C1_04120C_A**	**2.04**		IST3
**C1_07820W_A**	**2.04**		YFR045W
**C1_08710W_A**	**2.04**		YNL050C
**C1_12780W_A**	**2.04**	RBK1	RBK1
**C2_03550C_A**	**2.04**		NMD2
**C2_04370W_A**	**2.04**		
**C2_05100C_A**	**2.04**	ERF1	SUP45
**C3_04680W_A**	**2.04**	RPP2B	RPP2B
**C6_04080W_A**	**2.04**		MAP2
**C2_09070C_A**	**2.03**		
**C4_05170C_A**	**2.03**	NBP35	NBP35
**C5_00900C_A**	**2.03**	URA4	URA4
**C1_06510C_A**	**2.02**		MCH4
**C1_06960W_A**	**2.02**	SUI2	SUI2
**C1_07420C_A**	**2.02**	PEX3	PEX3
**C4_04880W_A**	**2.02**	FGR10	
**C4_06420W_A**	**2.02**		
**C5_01520C_A**	**2.02**	GLR1	GLR1
**C1_07130C_A**	**2.01**		
**C1_07200W_A**	**2.01**	YMC1	YMC1
**C2_08760C_A**	**2.01**	TIF4631	TIF4631
**C3_03890W_A**	**2.01**	CSC25	CDC25
**C4_01460C_A**	**2.01**		
**C7_03530C_A**	**2.01**	ISY1	ISY1
**C1_12890W_A**	**2.00**		
**C2_05370C_A**	**2.00**	RXT3	RXT3
**C2_07140W_A**	**2.00**		YNL010W
**C2_08780W_A**	**2.00**	RPT6	RPT6
**C4_03040W_A**	**2.00**		RPB10
**C4_04270W_A**	**2.00**		DAL3
**C5_02170C_A**	**2.00**	GCD11	GCD11
**C6_00970C_A**	**2.00**	HAL21	MET22

S120 vs B120 Upregulated genes

**Gene I D**	**Fold change**	**Standard gene nan**	**Name of *S. cerevisiae* ortholog(s)**
**C1_14120C_A**	**37.69**	RBE1	PRY2

**C1_00170W_A**	**35.29**	LEU4	LEU4
**CR_06300C_A**	**30.47**	FGR50	
**C2_08590W_A**	**22.16**	YWP1	
**C1_07330W_A**	**20.22**	RME1	RME1
**C5_03440W_A**	**18.99**		
**C1_05000W_A**	**18.33**	RHD1	
**C1_11990W_A**	**17.22**		
**C6_03250W_A**	**15.95**	BMT4	
**CR_05210W_A**	**15.7**		
**C2_06460W_A**	**15.06**	RTA3	
**C6_04130C_A**	**14.43**	ALS4	
**C4_02220C_A**	**13.8**	ZCF25	
**CR_01200W_A**	**13.47**	OYE22	
**C2_06970W_A**	**13.1**	AAH1	AAH1
**C1_02270C_A**	**12.9**		
**C4_03960W_A**	**12.7**		
**C4_06530C_A**	**12.45**	XUT1	
**C1_04750W_A**	**12.25**	IFE1	BDH2
**C7_04230W_A**	**11.72**	NRG1	NRG2
**C1_06810W_A**	**11.65**	CAT1	CTA1
**C6_01180C_A**	**11.31**	EBP1	
**CR_01160W_A**	**11.23**		
**C3_05580C_A**	**10.48**	GAP2	GAP1
**C1_13870W_A**	**10.32**	MET3	MET3
**C3_01460C_A**	**9.37**	FOX3	
**C2_03690C_A**	**9.25**		
**C4_01010C_A**	**9.05**	DAG7	
**C1_08070W_A**	**8.87**	CDR4	
**CR_00150C_A**	**8.77**	POT1	POT1
**CR_08890C_A**	**8.73**	ASR2	
**C3_06850W_A**	**8.41**	FCR1	
**C5_03530C_A**	**8.29**		COQ10
**C4_06390W_A**	**8.12**	SOU1	
**C3_04210W_A**	**8.05**		
**C7_02240W_A**	**7.78**	FMP45	FMP45
**C6_01420C_A**	**7.75**		
**C2_08170W_A**	**7.73**		SAY1
**C1_02800W_A**	**7.28**		
**C1_01220C_A**	**7.16**		
**C1_01750W_A**	**7.12**		YCR023C
**C2_03120W_A**	**7.12**	AMO1	
**C1_09610W_A**	**7.1**		
**C5_00430W_A**	**7.1**	MET14	MET14
**C2_08400C_A**	**6.95**		
**C6_01010W_A**	**6.85**	KAR5	KAR5
**C2_07570W_A**	**6.84**	RNR22	
**C1_06570W_A**	**6.83**	ANT1	ANT1

**C3_02310W_A**	**6.68**	MEP1	MEP3
**C3_04200W_A**	**6.67**	AFP99	
**C3_02480C_A**	**6.39**	CCP1	CCP1
**C6_04190C_A**	**6.37**		
**CR_01810C_A**	**6.1**		
**CR_09140C_A**	**6.04**		MSC1
**C4_05700W_A**	**6.02**	ZCF5	
**C6_00960W_A**	**5.98**	CAN1	
**C6_00480C_A**	**5.77**	FET3	
**C6_03360C_A**	**5.77**		
**C3_00410C_A**	**5.66**		
**C2_08870C_A**	**5.64**	PIR1	PIR1
**CR_05380C_A**	**5.6**	YCP4	YCP4
**C1_05480C_A**	**5.59**		
**C2_00180C_A**	**5.56**		
**C3_01960C_A**	**5.56**	POX1-3	POX1
**C4_05540W_A**	**5.56**	HOM3	HOM3
**C5_02080C_A**	**5.54**	HSP12	
**C5_02110W_A**	**5.51**		HSP12
**C1_08240C_A**	**5.43**		
**C1_10110W_A**	**5.41**		
**C1_05060W_A**	**5.33**	REI1	REI1
**C1_08840W_A**	**5.32**		
**C1_04660W_A**	**5.24**	DUR1,2	DUR1,2
**CR_02880W_A**	**5.24**		
**CR_04220C_A**	**5.21**		
**C6_00870C_A**	**5.16**	ILV5	ILV5
**C4_06620C_A**	**5.09**		
**CR_09150W_A**	**5.09**		
**C1_07990C_A**	**5.04**		
**C4_00160C_A**	**5.03**	CAR2	CAR2
**C5_04030W_A**	**5.03**		
**C4_04840C_A**	**4.99**	AGP3	AGP3
**C4_02200C_A**	**4.96**		
**C6_04610C_A**	**4.93**	NAG3	
**C1_13600W_A**	**4.92**		YBL029W
**C3_03710W_A**	**4.89**	CCC1	CCC1
**C4_02610C_A**	**4.89**	SUL2	SUL1
**C3_00810C_A**	**4.84**	FOX2	FOX2
**C6_02940C_A**	**4.79**		
**C1_06740C_A**	**4.72**	NOP6	NOP6
**C2_04460W_A**	**4.72**	LYS22	
**C3_04580C_A**	**4.63**	STP1	
**CR_00360C_A**	**4.61**	LEU1	LEU1
**C1_02980W_A**	**4.58**	GOR1	GOR1
**C1_01150C_A**	**4.57**		PPM2
**C6_04380W_A**	**4.56**	ALS2	

**C2_09920W_A**	**4.5**		RRG9
**CR_06550C_A**	**4.48**		
**C3_02810C_A**	**4.43**	FAA21	FAA2
**C4_06380W_A**	**4.41**	SOU2	
**C5_00140C_A**	**4.4**		YHR140W
**CR_00310C_A**	**4.39**		
**C6_03630W_A**	**4.36**	PAD1	PAD1
**C4_03050C_A**	**4.3**		CPS1
**C5_03470C_A**	**4.22**		
**C4_04140W_A**	**4.18**		
**C2_00080C_A**	**4.17**	FAV3	
**C6_00290W_A**	**4.08**		
**C1_04460C_A**	**4.06**		
**C6_03610W_A**	**4.06**	FGR17	
**C2_04230W_A**	**4**	BAT21	BAT2
**C1_06720C_A**	**3.99**		
**C1_08790W_A**	**3.94**	TPO3	TPO2
**C2_10240W_A**	**3.89**	GPD1	GPD1
**C2_05890C_A**	**3.87**	IDP1	IDP1
**CR_05620C_A**	**3.87**	MTG2	MTG2
**C4_01470W_A**	**3.84**		HRT3
**C7_00110W_A**	**3.82**	SOD3	
**C7_03850W_A**	**3.82**		NOC3
**C3_04860W_A**	**3.81**		SFP1
**C4_03160C_A**	**3.8**		
**C1_02620C_A**	**3.76**	HOM6	HOM6
**C6_02500C_A**	**3.74**	GCV1	GCV1
**C6_00520W_A**	**3.73**		
**CR_05390W_A**	**3.73**	PST3	PST2
**C6_03530C_A**	**3.71**		
**C2_00130W_A**	**3.67**		
**C1_11070W_A**	**3.65**	PET127	PET127
**C4_03110W_A**	**3.65**		DNF3
**C6_03710W_A**	**3.65**	ALS9	FLO1
**C4_03190W_A**	**3.63**		
**C4_07050W_A**	**3.63**	YDC1	YDC1
**C4_06120W_A**	**3.61**	GDH3	GDH3
**CR_08490W_A**	**3.58**	TSR1	TSR1
**C3_00320W_A**	**3.56**	RHR2	GPP1
**C2_06850W_A**	**3.55**		SLX9
**C3_03870C_A**	**3.49**	SAP9	YPS1
**C1_07820W_A**	**3.48**		YFR045W
**C2_09590C_A**	**3.48**		PIC2
**C3_02670W_A**	**3.48**		RSA1
**CR_06770C_A**	**3.46**		
**C2_09750W_A**	**3.44**	LEU42	
**C7_04090C_A**	**3.42**		

**C3_06760W_A**	**3.4**		RRP1
**C4_04810C_A**	**3.4**		TRM8
**C2_04480W_A**	**3.37**		
**C2_07300C_A**	**3.36**	RPA12	RPA12
**CR_07480W_A**	**3.35**		YBR287W
**C2_02910W_A**	**3.34**		
**C2_07130C_A**	**3.34**	RCK2	RCK2
**C5_05410C_A**	**3.27**	ILV3	ILV3
**C3_00100W_A**	**3.26**		YJU2
**C6_03180C_A**	**3.26**		ALY2
**C1_08890C_A**	**3.25**		MSL1
**C4_02720C_A**	**3.22**		SFK1
**C7_03880C_A**	**3.22**		
**C1_03180W_A**	**3.21**		LIP2
**C3_02040C_A**	**3.21**		LOC1
**C6_01890C_A**	**3.21**		DBP9
**C2_08200W_A**	**3.2**		
**C1_13670W_A**	**3.18**	OSM2	OSM1
**CR_04110W_A**	**3.16**		NAF1
**CR_09960C_A**	**3.15**	UGA3	UGA3
**C1_10780C_A**	**3.14**	HNT1	HNT1
**C3_03990C_A**	**3.11**	AAP1	
**C1_09190C_A**	**3.09**	DAK2	DAK2
**C2_01570W_A**	**3.09**		
**C3_03140C_A**	**3.08**	DAL4	DAL4
**C4_02050W_A**	**3.08**	HGH1	HGH1
**C3_06860C_A**	**3.07**		YJR096W
**C1_08290C_A**	**3.05**	DIP2	DIP2
**C1_08530W_A**	**3.05**		
**C5_01080C_A**	**3.05**	GDS1	GDS1
**C2_00880W_A**	**3.03**		
**C5_00100C_A**	**3.03**		
**C3_07360W_A**	**3.02**	DLD2	DLD1
**C6_01980C_A**	**3**		MRPS18
**CR_01470W_A**	**3**	CSP37	OM45
**C1_06350W_A**	**2.99**		
**C2_04570W_A**	**2.99**		EBP2
**C3_05380W_A**	**2.99**		YJL163C
**C1_09600C_A**	**2.98**	LIP6	
**C4_06710W_A**	**2.97**		
**C2_09500W_A**	**2.96**		DUS1
**C4_00510C_A**	**2.96**	RLP24	RLP24
**CR_03240C_A**	**2.96**		JJJ1
**C1_13060C_A**	**2.95**		MEF1
**C2_08130W_A**	**2.95**	ARA1	ARA1
**C5_01110W_A**	**2.95**		OTU2
**C1_07000W_A**	**2.94**	MEF2	MEF2

**C6_04310W_A**	**2.94**	PEX11	PEX11
**CR_04210C_A**	**2.94**	QDR1	QDR1
**C2_04120C_A**	**2.92**		NOP12
**C6_02230W_A**	**2.91**		LSG1
**C4_02880C_A**	**2.9**		SDO1
**C4_01170C_A**	**2.87**	BMT9	
**C1_06550W_A**	**2.86**	GLT1	GLT1
**C1_08060W_A**	**2.86**	OFR1	
**C4_03350C_A**	**2.86**		
**C5_03800W_A**	**2.86**		RCF1
**CR_01570W_A**	**2.86**		PTH2
**C1_04740W_A**	**2.84**	LPG20	YPL088W
**C3_04510W_A**	**2.84**		YBL028C
**CR_05790C_A**	**2.84**	ACO2	ACO2
**CR_05990C_A**	**2.84**	SFL1	SFL1
**C2_09000C_A**	**2.83**		
**CR_02420W_A**	**2.83**		UTP23
**CR_03450W_A**	**2.83**	HXT5	
**C1_07960W_A**	**2.82**		LRP1
**C1_01520C_A**	**2.81**	SOD2	SOD2
**C2_02640C_A**	**2.81**		
**C2_07960C_A**	**2.81**	NSA1	NSA1
**C4_01090C_A**	**2.81**		
**C4_04500C_A**	**2.81**		EFM5
**CR_01980C_A**	**2.81**	CRC1	CRC1
**C1_01240W_A**	**2.8**	IFD3	
**CR_01410C_A**	**2.8**		YNL035C
**C4_01840C_A**	**2.79**		AIM2
**C4_03710C_A**	**2.79**		
**C3_05070W_A**	**2.78**	CEM1	CEM1
**CR_01400W_A**	**2.78**	LYS12	LYS12
**C1_09160W_A**	**2.77**	AOX1	
**C7_03540C_A**	**2.77**	ENP2	ENP2
**C2_07450C_A**	**2.76**	RCL1	RCL1
**C1_01160C_A**	**2.75**		RRP40
**C1_03400W_A**	**2.75**		ART5
**C1_06120C_A**	**2.75**		
**C2_09140C_A**	**2.75**	MET10	MET10
**C2_08050C_A**	**2.73**	SIT1	ARN1
**C2_00410C_A**	**2.72**		ECM16
**C3_03800W_A**	**2.72**	PTR22	PTR2
**C5_04650C_A**	**2.72**	MSM1	MSM1
**C7_03530C_A**	**2.72**	ISY1	ISY1
**C4_06790W_A**	**2.71**		GPN3
**C7_02340C_A**	**2.71**		TRM82
**C1_01490W_A**	**2.7**		
**C5_05130C_A**	**2.7**		CAB5

**C3_05140C_A**	**2.69**		TRM11
**C4_00210W_A**	**2.69**		YPL034W
**C4_05460C_A**	**2.69**	OFD1	TPA1
**C3_01560W_A**	**2.68**		PRP43
**C1_06440C_A**	**2.67**		
**C1_09910C_A**	**2.66**		
**C2_07630C_A**	**2.66**		RTC3
**C3_07300W_A**	**2.66**	NOP13	NOP13
**C4_04880W_A**	**2.66**	FGR10	
**CR_02710W_A**	**2.66**	RRP9	RRP9
**CR_09370W_A**	**2.66**	ELF1	NEW1
**C1_12190W_A**	**2.65**	BUL4	
**C6_04530C_A**	**2.65**		RTT10
**CR_02030C_A**	**2.65**		RCM1
**C1_03660W_A**	**2.64**		
**C1_09710C_A**	**2.64**		UTP7
**C6_00800C_A**	**2.64**		
**C1_09690W_A**	**2.63**	MLS1	MLS1
**C2_06470W_A**	**2.63**	RTA2	RSB1
**C3_00370C_A**	**2.63**	BUD21	BUD21
**C7_00590W_A**	**2.63**		
**C2_09160W_A**	**2.62**		RRP45
**C5_00310C_A**	**2.62**		
**C7_04190C_A**	**2.62**	UTP18	UTP18
**CR_08940W_A**	**2.62**		GCD14
**C1_07880C_A**	**2.61**	GCS1	GSH1
**C1_13630W_A**	**2.61**	CYB2	CYB2
**C6_02560W_A**	**2.61**		YMR315W
**CR_00210W_A**	**2.61**	ALK2	
**CR_07030C_A**	**2.61**		RRP36
**C3_03200C_A**	**2.6**		YDR286C
**C4_01370W_A**	**2.6**	ILV6	ILV6
**C6_03930W_A**	**2.6**	OPT8	YGL114W
**CR_10470C_A**	**2.6**		BFR2
**C7_00690W_A**	**2.59**	NOP15	NOP15
**C7_02100W_A**	**2.59**		
**C1_05270C_A**	**2.58**		MHR1
**C1_10970W_A**	**2.58**		ESF1
**C2_00840W_A**	**2.58**		TAH18
**C2_01870C_A**	**2.58**		
**CR_04810W_A**	**2.58**	RPS3	RPS3
**CR_09670C_A**	**2.58**		YJL068C
**C1_09810W_A**	**2.57**		YGR210C
**C2_07070W_A**	**2.56**		ZTA1
**C2_09380W_A**	**2.56**	RRP15	RRP15
**C5_05280C_A**	**2.56**	SDH4	TIM18
**C6_01000C_A**	**2.56**		AEP1

**CR_08000C_A**	**2.56**		
**C1_06800W_A**	**2.55**		RAI1
**C4_02300W_A**	**2.55**	HOS3	HOS3
**C4_03030C_A**	**2.55**	NAN1	NAN1
**C5_04290C_A**	**2.55**		
**C1_11700C_A**	**2.54**	MRF1	
**C2_01740C_A**	**2.54**		MRPL31
**C4_02790C_A**	**2.54**	SAS10	SAS10
**C4_00700C_A**	**2.53**		
**C5_02270W_A**	**2.53**	THR4	THR4
**CR_06590C_A**	**2.53**	RGS2	RGS2
**CR_02690W_A**	**2.52**		ALF1
**CR_07700W_A**	**2.52**		
**C2_01010W_A**	**2.51**	HGT8	
**C2_09660W_A**	**2.51**		NOP16
**C3_03390C_A**	**2.51**	YAH1	YAH1
**C4_01730C_A**	**2.51**		PBY1
**C4_03900C_A**	**2.51**	RAM1	RAM1
**CR_00670C_A**	**2.51**		TRM13
**CR_03000C_A**	**2.51**		
**C1_02850W_A**	**2.5**		VTS1
**C1_02880C_A**	**2.5**	MIA40	MIA40
**C2_06480W_A**	**2.5**		TRM10
**C4_05010W_A**	**2.5**		RSA4
**CR_03010C_A**	**2.5**	ERV1	ERV1
**C3_07550C_A**	**2.49**		GEP3
**C1_04040C_A**	**2.48**		NOP9
**C2_07340W_A**	**2.48**	NOC4	NOC4
**C3_02140C_A**	**2.48**		
**C3_04370C_A**	**2.48**		URB2
**C4_02410C_A**	**2.48**	AHP1	AHP1
**C5_02280C_A**	**2.48**		CTR86
**C1_12360C_A**	**2.47**	RIB3	RIB3
**C3_06380W_A**	**2.47**	NSA2	NSA2
**C7_01490W_A**	**2.47**		
**C1_11020W_A**	**2.46**		
**C4_04470W_A**	**2.46**	SAP10	
**C4_06820C_A**	**2.46**	CZF1	
**C6_04200C_A**	**2.46**		
**C1_11420W_A**	**2.45**	KRR1	KRR1
**C2_08240W_A**	**2.45**	ORF298	
**C3_05800W_A**	**2.45**		SEN54
**C4_04820C_A**	**2.45**		MRPL11
**C1_11380W_A**	**2.44**	NEP1	EMG1
**C2_01760C_A**	**2.44**		
**C2_06000W_A**	**2.44**	LAS1	LAS1
**C2_08000C_A**	**2.44**	KRE30	ARB1

**C4_02140C_A**	**2.44**	TIM10	TIM10
**C6_00640C_A**	**2.44**		TFB4
**CR_03470W_A**	**2.44**		YPL014W
**CR_10240W_A**	**2.44**	UTP5	UTP5
**C1_03830C_A**	**2.43**		CSL4
**CR_06720W_A**	**2.43**	NMD3	NMD3
**C2_08670C_A**	**2.41**	UTP15	UTP15
**C4_04110W_A**	**2.41**		
**CR_08990C_A**	**2.41**		
**C1_10880W_A**	**2.4**		UTP6
**C2_04930C_A**	**2.4**	CRK1	SGV1
**C1_11970C_A**	**2.39**		
**C1_13720W_A**	**2.39**		KEL3
**C2_02980C_A**	**2.39**	DLD1	
**C4_01080W_A**	**2.39**	RAT1	RAT1
**C5_02120C_A**	**2.39**	RIX7	RIX7
**C5_02730C_A**	**2.39**		CIA1
**C6_04240W_A**	**2.39**		
**C2_00140W_A**	**2.38**	NDT80	NDT80
**C4_02620C_A**	**2.38**		YDR109C
**CR_03710C_A**	**2.38**		
**CR_07050C_A**	**2.38**	URA2	URA2
**C1_08870C_A**	**2.37**	JIP5	JIP5
**C2_02880C_A**	**2.37**	EBP7	
**C2_08460C_A**	**2.37**		
**C4_05230C_A**	**2.37**		RIX1
**CR_10550W_A**	**2.37**	DRS1	DRS1
**C5_01430C_A**	**2.36**		RBA50
**CR_04760C_A**	**2.36**		YMR221C
**C1_11010C_A**	**2.35**		PEX32
**C1_02820W_A**	**2.34**	LYS2	LYS2
**C1_03840W_A**	**2.34**		
**C1_14320C_A**	**2.34**		FMP41
**C3_00750W_A**	**2.34**	LYS144	
**C3_03650W_A**	**2.33**	HBR3	NOB1
**C4_06210C_A**	**2.33**		NUG1
**C4_07030W_A**	**2.33**	MET16	MET16
**C5_03710C_A**	**2.33**		
**C6_03470W_A**	**2.33**		CSR1
**C5_03390C_A**	**2.32**	FUR1	FUR1
**C6_00360C_A**	**2.32**		MIX23
**C7_01030C_A**	**2.32**		RRP12
**C7_03120W_A**	**2.32**	TIM54	TIM54
**C1_02810W_A**	**2.31**	RAD16	RAD16
**C1_06820W_A**	**2.31**		
**C1_07950C_A**	**2.31**		RRP17
**C4_01740W_A**	**2.31**	TIM12	TIM12

**CR_02060W_A**	**2.31**		
**C2_05090W_A**	**2.3**	MAK5	MAK5
**C6_00760W_A**	**2.3**		FES1
**C6_01040C_A**	**2.3**		DHR2
**CR_10410C_A**	**2.3**		PNO1
**C1_02090C_A**	**2.29**		DRN1
**C2_00480C_A**	**2.29**	PHHB	YHL018W
**C3_01680C_A**	**2.29**		YDL183C
**C3_03500W_A**	**2.29**	SKN2	
**C4_02830C_A**	**2.29**	HCA4	HCA4
**C5_00930C_A**	**2.29**	TFS1	TFS1
**CR_03750C_A**	**2.29**	PRP42	PRP42
**C2_02430W_A**	**2.28**	UTP21	UTP21
**C2_04510W_A**	**2.28**	RIM2	RIM2
**C3_00960W_A**	**2.28**		
**C5_03920C_A**	**2.28**		FCF1
**C7_03400C_A**	**2.28**		MTR4
**C7_03520W_A**	**2.28**	PNC1	PNC1
**CR_03070W_A**	**2.28**		DOM34
**C2_01970C_A**	**2.27**	ROD1	
**C3_05570W_A**	**2.27**	QDR2	
**C7_03930C_A**	**2.27**	MAM33	MAM33
**CR_05630W_A**	**2.27**	DBP8	DBP8
**C1_05820C_A**	**2.26**	RAD10	RAD10
**C2_06360C_A**	**2.26**	MAK16	MAK16
**C3_02950C_A**	**2.26**	MET13	MET13
**CR_03280W_A**	**2.26**	IFR2	
**C2_00820W_A**	**2.25**		MSS1
**C2_02190C_A**	**2.25**		
**C2_09170W_A**	**2.25**	SLP2	
**C2_09260C_A**	**2.25**	GLO2	GLO2
**C3_06830C_A**	**2.25**		YLR419W
**C5_03030W_A**	**2.25**		
**CR_08690C_A**	**2.25**	ECI1	ECI1
**C5_00550C_A**	**2.24**		
**C5_01420W_A**	**2.24**		SNA2
**C5_02010C_A**	**2.24**		BUD27
**C5_03410C_A**	**2.24**		MRPL20
**CR_02810W_A**	**2.24**	CIRT4B	
**CR_07950W_A**	**2.24**	IMP4	IMP4
**C2_02710C_A**	**2.23**		RRP7
**C5_01140C_A**	**2.23**		MSC6
**CR_03370C_A**	**2.23**	KTI12	KTI12
**CR_05750W_A**	**2.23**		
**C1_04320W_A**	**2.22**	GPM2	
**CR_05020W_A**	**2.22**	LYP1	
**C1_04340C_A**	**2.21**		YMR010W

**C2_03130W_A**	**2.21**		SFM1
**C3_01790C_A**	**2.21**		
**C3_07290W_A**	**2.21**		OCA5
**C4_01830C_A**	**2.21**		
**CR_09310W_A**	**2.21**		RKM1
**C1_11220C_A**	**2.2**	TIM50	TIM50
**C1_03150C_A**	**2.19**		
**C1_13940W_A**	**2.19**	SIM1	UTH1
**C2_07520C_A**	**2.19**		NAM8
**C2_10640C_A**	**2.19**	MTR2	MTR2
**C4_06780C_A**	**2.19**	OYE32	
**C5_01910W_A**	**2.19**	GIS2	GIS2
**CR_00570W_A**	**2.18**		COQ1
**CR_07640C_A**	**2.18**		VFA1
**C1_00230C_A**	**2.17**	BFA1	BFA1
**C7_00950W_A**	**2.17**	YML6	YML6
**C2_06070W_A**	**2.16**	FAA2-1	
**CR_03760W_A**	**2.16**		NNT1
**CR_04170W_A**	**2.16**		NOP2
**CR_10400W_A**	**2.16**		ARD1
**C1_05030C_A**	**2.15**	PSF2	PSF2
**C1_08430W_A**	**2.15**	CAF16	CAF16
**C2_01580W_A**	**2.15**	REX2	REX2
**C3_01000W_A**	**2.15**	BMS1	BMS1
**C3_06230W_A**	**2.15**	CYM1	CYM1
**CR_03650W_A**	**2.15**	SGD1	SGD1
**C1_09720W_A**	**2.14**	URA1	URA1
**C2_02540W_A**	**2.14**		RRP5
**C2_07920W_A**	**2.14**		RRT2
**C3_02320W_A**	**2.14**	ILV2	ILV2
**C5_01520C_A**	**2.14**	GLR1	GLR1
**C1_02890C_A**	**2.13**		
**C1_08810C_A**	**2.12**	MSS116	MSS116
**C2_03560C_A**	**2.12**		MRPL35
**C2_08480W_A**	**2.12**	RRP8	RRP8
**C3_02130W_A**	**2.12**	UTP4	UTP4
**C6_02430W_A**	**2.12**		ITC1
**C7_03030W_A**	**2.12**		
**CR_09800C_A**	**2.12**		UTP14
**C3_03820C_A**	**2.11**		PNT1
**C5_05340W_A**	**2.11**		SQT1
**C1_01480C_A**	**2.1**	RPS21	RPS2
**C1_02840W_A**	**2.1**	PDE2	PDE2
**C1_08260C_A**	**2.1**	LTP1	LTP1
**C2_04820W_A**	**2.1**		MRM1
**C3_07100C_A**	**2.1**		NGR1
**C5_04580C_A**	**2.1**		VPS25

**C7_01970C_A**	**2.1**	TOM40	TOM40
**CR_02260C_A**	**2.1**		
**CR_08570W_A**	**2.1**	SKI2	SKI2
**C1_10950C_A**	**2.09**		RIO1
**C2_04160W_A**	**2.09**		YGR021W
**C3_00420W_A**	**2.09**		TRZ1
**C5_05440C_A**	**2.09**		
**CR_03200C_A**	**2.09**		POP1
**C1_00530C_A**	**2.08**		
**C1_03430W_A**	**2.08**		TGL2
**C1_03900W_A**	**2.08**	SEN15	SEN15
**C1_06760C_A**	**2.08**		IPI3
**C1_13260W_A**	**2.08**		FPR2
**C2_06750C_A**	**2.08**		
**C7_03130C_A**	**2.08**	DFR1	DFR1
**CR_04880W_A**	**2.08**		GLY1
**CR_08410W_A**	**2.08**		REH1
**C1_00560W_A**	**2.07**	CNS1	CNS1
**C1_05990C_A**	**2.07**		YDL144C
**C1_07210C_A**	**2.07**		
**C2_05160C_A**	**2.07**		PUF6
**C2_10270W_A**	**2.07**	PUS7	PUS7
**C3_06730W_A**	**2.07**		
**CR_00680W_A**	**2.07**	BUD22	BUD22
**C1_05840W_A**	**2.06**	PRN1	
**C1_12890W_A**	**2.06**		
**C4_02870C_A**	**2.06**	ZUO1	ZUO1
**C4_06680C_A**	**2.06**		SMY2
**C6_02970C_A**	**2.06**		PEX25
**CR_08500W_A**	**2.06**		SYO1
**CR_08840C_A**	**2.06**	NIT2	NIT2
**C1_13820C_A**	**2.05**		
**C2_05340C_A**	**2.05**	RPC40	RPC40
**C3_07660W_A**	**2.05**		RAD4
**C6_02410W_A**	**2.05**		NPY1
**C7_04210C_A**	**2.05**		
**C1_05050C_A**	**2.04**	RRP6	RRP6
**C2_02490C_A**	**2.04**		YPK9
**C2_03700W_A**	**2.04**		NTA1
**C2_03830W_A**	**2.04**		DPH6
**C3_07040C_A**	**2.04**	YSA1	YSA1
**C4_06850C_A**	**2.04**		MAP1
**C7_01200C_A**	**2.04**	DBP7	DBP7
**C7_03630C_A**	**2.04**	TIM9	TIM9
**C1_06780W_A**	**2.03**	NPR1	NPR1
**C2_09510C_A**	**2.03**		SAL1
**C3_06350W_A**	**2.03**		MED6

**C4_00690C_A**	**2.03**		DPH2
**C5_00030W_A**	**2.03**		MRPL1
**C2_05060C_A**	**2.02**		SRX1
**C2_09280C_A**	**2.02**		
**C3_05930W_A**	**2.02**	CTA4	OAF1
**C5_00800C_A**	**2.02**		BNA2
**C5_02690W_A**	**2.02**		
**C5_04360C_A**	**2.02**		SDT1
**C1_00590W_A**	**2.01**	TUF1	TUF1
**C1_01110C_A**	**2.01**	ZPR1	ZPR1
**C3_04560W_A**	**2.01**	MAK21	MAK21
**C3_05120C_A**	**2.01**		PAC10
**CR_01950W_A**	**2.01**		RPA43
**CR_04240C_A**	**2.01**		KRE33
**CR_06000W_A**	**2.01**	SOK1	SOK1
**CR_08330W_A**	**2.01**		OPI10
**CR_08600C_A**	**2.01**	PEX19	PEX19
**C1_13330C_A**	**2**		SPE4
**CR_08860W_A**	**2**	PDK2	PKP1

S20 Vs B20 Downregulated genes

**Gene I D**	**Fold change**	**Standard gene nan**	**Name of *S. cerevisiae* ortholog(s)**
**C4_03570W_A**	**-1,265.60**	HWP1	
**C1_06280C_A**	**-677.49**	UME6	
**C6_02100W_A**	**-428.43**		
**C4_04080C_A**	**-91.97**	PGA31	
**C2_02390W_A**	**-59.95**		
**C6_03850C_A**	**-42.14**	IHD1	YFL067W
**C5_04250W_A**	**-38.85**	MRV8	
**C4_03520C_A**	**-27.61**	RBT1	
**C5_04370C_A**	**-25.29**	PGA37	
**CR_05430W_A**	**-21.8**	CSP1	
**C2_03220C_A**	**-19.91**	STP4	STP3
**C4_02900C_A**	**-19.31**	CRH11	CRH1
**C5_04190W_A**	**-18.41**	MRV2	YDL218W
**C4_04050C_A**	**-16.04**	RHD3	
**CR_09180W_A**	**-13.76**	CDG1	
**C6_02210W_A**	**-11.91**		
**C1_02110C_A**	**-11.24**	HGT2	
**C6_04420W_A**	**-10.94**		
**C5_03430W_A**	**-9.95**		
**CR_03890W_A**	**-9.84**	WOR3	
**C2_03120W_A**	**-8.85**	AMO1	
**CR_08990C_A**	**-8.85**		
**CR_09090C_A**	**-8.75**		
**CR_07790C_A**	**-8.03**	YHB1	YHB1
**C5_01250W_A**	**-7.84**	GPR1	GPR1

**C3_01640C_A**	**-7.79**		
**CR_02900W_A**	**-7.65**		
**C3_04070C_A**	**-7.6**	CDR11	
**CR_09680C_A**	**-7.6**	RTA4	
**C2_04010C_A**	**-7.44**	HSP21	
**CR_05170C_A**	**-6.37**	FDH1	FDH1
**C1_06000W_A**	**-6.21**		
**CR_10330W_A**	**-6.07**	PGA11	
**CR_02070C_A**	**-6.02**	ADH5	
**C3_01440C_A**	**-5.84**		
**C3_02650W_A**	**-5.84**		
**C5_03730W_A**	**-5.83**		
**C4_04850C_A**	**-5.79**	CSR1	ZAP1
**CR_01910C_A**	**-5.78**		BIO4
**C2_09220W_A**	**-5.71**	DDR48	DDR48
**C4_06970C_A**	**-5.69**	ZRT1	
**C3_03990C_A**	**-5.54**	AAP1	
**C1_06160W_A**	**-5.47**		
**CR_09020C_A**	**-5.34**	CHS2	CHS1
**C1_13100W_A**	**-5.1**		
**C6_00160W_A**	**-5.01**	PGA48	
**C6_02330W_A**	**-4.86**		
**C4_04790W_A**	**-4.83**	FAV1	FUS2
**C1_04010C_A**	**-4.67**		
**C1_11270W_A**	**-4.62**		
**C3_01360C_A**	**-4.55**	IRO1	
**C1_03970W_A**	**-4.49**		
**C1_00270W_A**	**-4.46**		
**C4_05830W_A**	**-4.46**		
**C1_07160C_A**	**-4.45**		
**C2_04430W_A**	**-4.38**		
**C1_07980C_A**	**-4.37**		SAE3
**C4_04860W_A**	**-4.26**		
**C1_10060C_A**	**-4.2**		
**CR_09100C_A**	**-4.16**		YPR127W
**CR_10340W_A**	**-4.01**	PTP3	PTP3
**C1_02130C_A**	**-3.97**	GAL1	GAL1
**C3_02280C_A**	**-3.97**	GFA1	GFA1
**C3_00140W_A**	**-3.92**		
**C3_05750C_A**	**-3.85**		
**CR_05340C_A**	**-3.84**	IFE2	
**C1_04500W_A**	**-3.83**	ICL1	ICL1
**C2_10210C_A**	**-3.77**	RAS1	RAS1
**C3_06000W_A**	**-3.73**	AHR1	
**C3_03020W_A**	**-3.68**		
**C3_07830W_A**	**-3.67**	FBP1	FBP1
**C7_00350C_A**	**-3.66**		

**C4_00450C_A**	**-3.64**	PGA10	
**C3_02790W_A**	**-3.62**		
**C4_02020W_A**	**-3.62**	CAT2	CAT2
**C5_04980W_A**	**-3.62**		
**C1_14180W_A**	**-3.6**		
**C4_02360W_A**	**-3.58**	AMS1	AMS1
**C7_03420C_A**	**-3.57**	SNG3	
**C4_01260W_A**	**-3.49**	RLM1	RLM1
**C1_10180C_A**	**-3.47**	ECM21	ECM21
**C1_05440C_A**	**-3.34**		
**C1_00290W_A**	**-3.33**	POL93	
**C1_10290W_A**	**-3.33**	GCA1	
**C2_07900W_A**	**-3.33**	GDH2	GDH2
**C5_04560C_A**	**-3.23**	CUP1	
**CR_04960C_A**	**-3.17**	CRG1	
**C1_01210W_A**	**-3.15**		
**C1_09690W_A**	**-3.15**	MLS1	MLS1
**C1_12850W_A**	**-3.13**	BLP1	
**C1_02150W_A**	**-3.12**	GAL10	GAL10
**C1_11850W_A**	**-3.09**		
**C3_05710W_A**	**-3.03**	RCT1	YNL208W
**CR_02740W_A**	**-3.03**	INN1	INN1
**C3_06470W_A**	**-3.02**	AAF1	
**C4_04380C_A**	**-3.01**		
**C3_04530C_A**	**-2.98**	TEC1	TEC1
**C2_04050C_A**	**-2.97**	DCK1	DCK1
**C3_02290W_A**	**-2.94**		MRX10
**C1_05160C_A**	**-2.93**		YKR070W
**C1_10300W_A**	**-2.92**	MNN12	MNT2
**C1_00190C_A**	**-2.9**		
**C2_05130W_A**	**-2.82**		YML131W
**C1_05140W_A**	**-2.81**	BRG1	
**C2_08330W_A**	**-2.77**		
**C3_04640W_A**	**-2.76**	ZCF31	
**CR_03260W_A**	**-2.74**		YGR127W
**C2_04440W_A**	**-2.73**		
**C2_04450W_A**	**-2.73**		
**CR_02360W_A**	**-2.72**	IDP2	IDP2
**C1_12140W_A**	**-2.71**		
**C4_01860C_A**	**-2.7**		
**C1_03320C_A**	**-2.69**	EHD3	EHD3
**C4_06150C_A**	**-2.68**		HOP2
**C3_07640C_A**	**-2.65**	FUM12	
**C5_04470C_A**	**-2.64**		
**CR_00090C_A**	**-2.61**		YMR090W
**C2_09810C_A**	**-2.6**		
**C4_00860C_A**	**-2.59**		PIN2

**C5_03050C_A**	**-2.59**	PGA58	
**CR_04480C_A**	**-2.58**	LAP3	LAP3
**C3_02810C_A**	**-2.56**	FAA21	FAA2
**CR_04730W_A**	**-2.54**		YJR085C
**C3_04630W_A**	**-2.53**		
**CR_00780C_A**	**-2.52**	GSY1	GSY1
**CR_09690C_A**	**-2.51**	PLB3	PLB1
**C6_01900C_A**	**-2.47**	DOG1	DOG2
**C3_05550C_A**	**-2.46**		
**C5_04830W_A**	**-2.45**	SFL2	MGA1
**CR_08690C_A**	**-2.45**	ECI1	ECI1
**C4_03430W_A**	**-2.44**	MOH1	MOH1
**C1_03720C_A**	**-2.43**		
**C5_03240W_A**	**-2.43**	BUB3	BUB3
**C1_11730W_A**	**-2.38**		
**C3_04910C_A**	**-2.37**	NCE102	NCE102
**C5_04510W_A**	**-2.37**	GDE1	GDE1
**C2_07730W_A**	**-2.35**	YVC1	YVC1
**C3_00880W_A**	**-2.35**	KGD1	KGD1
**CR_02020C_A**	**-2.34**	OPT1	OPT1
**C4_06510C_A**	**-2.31**	CSO99	
**CR_07140C_A**	**-2.31**		
**C2_06470W_A**	**-2.29**	RTA2	RSB1
**C7_03480W_A**	**-2.29**		YEL1
**CR_06270W_A**	**-2.29**		
**C5_02800C_A**	**-2.28**		
**C1_01760W_A**	**-2.26**	FGR38	
**C1_04100C_A**	**-2.25**	POR1	POR1
**C1_08350C_A**	**-2.25**		
**C2_07750W_A**	**-2.25**	RHO2	RHO2
**C4_02370C_A**	**-2.25**	PGA59	
**C4_02390W_A**	**-2.25**	PGA62	
**C2_09650W_A**	**-2.24**		SGN1
**C3_02890C_A**	**-2.24**		
**C1_01710W_A**	**-2.23**		
**C6_00790C_A**	**-2.23**	CTR1	CTR1
**C4_00150C_A**	**-2.22**	PEX5	PEX5
**CR_07290W_A**	**-2.22**	FRE7	
**C2_03210W_A**	**-2.19**		
**C6_03700W_A**	**-2.19**	ALS1	
**CR_03730C_A**	**-2.15**		
**CR_05730C_A**	**-2.15**		MDM36
**C1_03270W_A**	**-2.13**		YMR114C
**C1_11950W_A**	**-2.13**		
**C1_14440C_A**	**-2.13**	GDI1	GDI1
**C6_00840W_A**	**-2.12**	GPX2	
**C7_02040C_A**	**-2.11**	CUP9	TOS8

**C3_02710W_A**	**-2.1**		
**C6_00810C_A**	**-2.1**		
**CR_03780C_A**	**-2.1**		
**C5_03150W_A**	**-2.08**		PRR1
**C1_08030W_A**	**-2.06**	PFY1	PFY1
**C1_09650W_A**	**-2.05**		
**C1_14420W_A**	**-2.05**	DDI1	DDI1
**C4_04530C_A**	**-2.05**	PHR1	
**C7_01860W_A**	**-2.05**		
**CR_05670C_A**	**-2.05**	HMO1	HMO1
**CR_07280W_A**	**-2.05**	FRE30	
**C4_05110C_A**	**-2.04**	HAP41	
**C3_04920C_A**	**-2.03**		NAS6
**C2_09030W_A**	**-2.02**		
**C4_03590C_A**	**-2.02**	OSH3	OSH3
**C5_02520W_A**	**-2.01**		

S45 vs B45 Downregulated genes

**Gene I D**	**Fold change**	**Standard gene nan**	**Name of *S. cerevisiae* ortholog(s)**
**C4_03570W_A**	**-2361.30**	HWP1	
**C4_03470C_A**	**-1800.63**	ECE1	
**C6_03030W_A**	**-511.46**	SAP5	
**CR_06670W_A**	**-424.18**	CFL11	TUB2
**C6_02710C_A**	**-418.36**	SAP6	CLN2
**C1_06280C_A**	**-264.33**	UME6	
**C4_04070C_A**	**-113.74**	PGA30	
**CR_07070C_A**	**-105.50**	ALS3	
**C1_05920W_A**	**-73.11**		
**C6_03850C_A**	**-63.83**	IHD1	DUT1
**C4_03520C_A**	**-57.10**	RBT1	
**C2_00680C_A**	**-51.19**	SOD5	GAL1
**C1_13450W_A**	**-38.73**	HYR1	GAL10
**C3_00920W_A**	**-35.11**	ATO1	EXG2
**C5_04930C_A**	**-31.35**	MAL31	
**CR_02490W_A**	**-28.32**	OPT4	ECM33
**CR_03890W_A**	**-25.94**	WOR3	CDC12
**C6_00840W_A**	**-21.12**	GPX2	
**C5_03750W_A**	**-20.73**	FAV2	
**C2_08430C_A**	**-20.69**	PGA46	
**C5_03430W_A**	**-19.39**		
**C4_04380C_A**	**-19.21**		CHL4
**C6_02330W_A**	**-17.36**		
**C4_02030W_A**	**-15.85**	RFX2	
**C2_02390W_A**	**-15.71**		LAS21
**C2_07720C_A**	**-13.35**		YOR238W
**C4_03500C_A**	**-13.32**		
**C7_00280W_A**	**-12.69**	HGT12	POL32

**C3_05990C_A**	**-10.58**		
**C5_03930C_A**	**-10.49**		
**C1_00780C_A**	**-10.23**	HGC1	
**C7_02500C_A**	**-10.22**	DPP3	
**C3_06400C_A**	**-9.77**		
**C2_09530W_A**	**-9.27**	EAP1	
**C4_04170C_A**	**-9.14**	AXL2	
**C4_03690C_A**	**-9.10**		CTF4
**C7_03150W_A**	**-8.76**		RHO5
**C3_01470W_A**	**-8.74**	ARF3	
**C3_01360C_A**	**-8.58**	IRO1	
**C4_01220C_A**	**-7.98**		ALG9
**C2_07730W_A**	**-7.79**	YVC1	STE12
**C1_06250W_A**	**-7.71**		DOC1
**C1_06630W_A**	**-7.64**		DPM1
**C2_04050C_A**	**-7.62**	DCK1	TAD3
**C5_02460C_A**	**-7.56**	ECM331	
**C4_03070W_A**	**-7.49**	PGA54	
**CR_10790W_A**	**-7.35**	MAL2	KEX2
**C1_02630C_A**	**-7.12**	EXG2	
**C1_13100W_A**	**-7.06**		
**CR_06510W_A**	**-7.06**		CLA4
**C1_06140C_A**	**-6.91**		MNT2
**C4_01240C_A**	**-6.89**		
**C4_06490C_A**	**-6.87**	PRI2	
**C6_01960W_A**	**-6.79**		ERP5
**C1_12550C_A**	**-6.62**	CDC54	CSG2
**C7_01500W_A**	**-6.58**		GIN4
**C7_01380W_A**	**-6.56**		
**CR_06500C_A**	**-6.55**		SMY1
**C3_06670C_A**	**-6.50**		
**C6_04420W_A**	**-6.44**		MCD4
**C1_10210C_A**	**-6.32**	CLA4	CDC6
**C2_04440W_A**	**-6.16**		RAX2
**CR_07790C_A**	**-6.12**	YHB1	
**C2_06250C_A**	**-6.11**	CDC46	MCM4
**CR_01530C_A**	**-6.08**		
**C2_02850W_A**	**-6.04**	UGA6	ELO2
**C7_03480W_A**	**-6.01**		
**C1_06340W_A**	**-5.90**		
**C4_01250W_A**	**-5.83**	NAT4	SPC3
**C1_07370C_A**	**-5.80**	CPH1	
**C2_01330C_A**	**-5.76**	SIR2	MSH6
**C1_01980W_A**	**-5.74**	HGT1	YPR098C
**C5_01460W_A**	**-5.66**	YBL053	
**C7_00770W_A**	**-5.61**		
**CR_01910C_A**	**-5.57**		RAD54

**C4_04790W_A**	**-5.55**	FAV1	MYO2
**C2_01250W_A**	**-5.48**		LCB2
**CR_03680C_A**	**-5.43**	SMC1	IRR1
**C2_03160C_A**	**-5.34**	RNR1	RFA1
**C2_01400C_A**	**-5.22**	ESC4	SAS2
**C2_07350W_A**	**-5.19**	MCM3	
**C2_01980C_A**	**-5.15**	SRD1	
**C3_06000W_A**	**-5.14**	AHR1	
**C2_03010C_A**	**-5.09**	RNR21	
**C1_13170C_A**	**-5.04**	MSH6	
**C3_04300C_A**	**-5.04**	POL1	PTM1
**C4_01770W_A**	**-5.00**	POL30	RTT107
**C2_06260W_A**	**-4.93**		MPO1
**C4_02600C_A**	**-4.92**		MSB2
**C2_10210C_A**	**-4.87**	RAS1	YOL019W
**C4_05250W_A**	**-4.87**		FAB1
**C5_00740W_A**	**-4.87**	CTF8	
**C2_04450W_A**	**-4.81**		
**C5_04250W_A**	**-4.80**	MRV8	
**C2_07180W_A**	**-4.76**		
**C1_08230C_A**	**-4.74**	PLB5	SUR2
**C5_02800C_A**	**-4.72**		RNR2
**C3_06880W_A**	**-4.71**		RNR1
**CR_00300W_A**	**-4.69**	CDA2	
**C1_12200W_A**	**-4.66**	MCD4	
**CR_02140W_A**	**-4.65**	RSR1	DCK1
**C3_05350C_A**	**-4.63**	SMI1B	YPR011C
**C2_07660W_A**	**-4.61**	PDS5	
**C3_02880W_A**	**-4.61**		
**C1_07160C_A**	**-4.58**		
**C2_00440W_A**	**-4.58**	SAS2	HIS3
**C4_05900C_A**	**-4.56**		ITR2
**C1_01330C_A**	**-4.55**	DUT1	
**C1_04590W_A**	**-4.55**		RHO3
**C4_00720W_A**	**-4.53**	CSP2	CDC21
**C6_00820W_A**	**-4.49**	SUN41	ERP3
**CR_01060W_A**	**-4.46**	LAG1	MCM5
**C6_03100W_A**	**-4.40**	SHE3	YLR281C
**C6_02210W_A**	**-4.35**		PIL1
**C3_01550C_A**	**-4.32**	TOS1	LTE1
**C3_02330C_A**	**-4.29**		
**C3_00170C_A**	**-4.27**		RFA2
**C5_04830W_A**	**-4.19**	SFL2	
**C7_01550W_A**	**-4.14**	BEM2	MCM3
**C6_00830C_A**	**-4.11**	CAN3	
**C1_00580W_A**	**-4.07**		PDS5
**C2_05190W_A**	**-4.06**	CDC21	

**CR_02360W_A**	**-4.06**	IDP2	YVC1
**CR_00920W_A**	**-4.04**	CCE1	RHO2
**C5_05350W_A**	**-3.95**		
**C2_06870C_A**	**-3.93**	PST1	AIF1
**C4_01320C_A**	**-3.92**	OPT6	
**CR_09070C_A**	**-3.89**		DPB11
**CR_09530C_A**	**-3.88**		
**C6_02990W_A**	**-3.86**		
**C3_04610W_A**	**-3.82**	MEA1	
**C5_02780W_A**	**-3.81**		
**CR_09050C_A**	**-3.81**		
**C1_06270W_A**	**-3.78**		SGN1
**C1_07030C_A**	**-3.76**	RBT4	
**CR_01920W_A**	**-3.73**		RAS1
**C3_04740C_A**	**-3.71**		
**C2_07750W_A**	**-3.68**	RHO2	SGS1
**C3_04530C_A**	**-3.62**	TEC1	
**CR_04650W_A**	**-3.57**		RDH54
**CR_09440C_A**	**-3.55**		VTC1
**C7_01100C_A**	**-3.53**		
**C2_09490W_A**	**-3.50**	PHO13	
**C6_01940W_A**	**-3.45**		
**C4_01860C_A**	**-3.44**		
**CR_07810W_A**	**-3.44**	YHB5	TAO3
**C2_04970W_A**	**-3.43**	ABP2	TOS1
**C6_00180C_A**	**-3.43**		DIG2
**CR_04680C_A**	**-3.43**		ERG6
**C7_03080W_A**	**-3.40**	LMO1	
**C6_00440C_A**	**-3.39**	FET34	
**C1_14230C_A**	**-3.37**	IRR1	
**C7_03440W_A**	**-3.37**	SNG4	
**C4_04250W_A**	**-3.35**		ALT1
**C6_03670C_A**	**-3.34**		
**C4_01260W_A**	**-3.33**	RLM1	
**C4_04590W_A**	**-3.29**	SCS7	
**C6_03310W_A**	**-3.29**		POL1
**C1_00710C_A**	**-3.28**	TUB2	VAM3
**C6_02090C_A**	**-3.28**		TEC1
**C3_00650W_A**	**-3.24**	NGT1	AFI1
**C5_01250W_A**	**-3.23**	GPR1	TOP3
**C7_01580W_A**	**-3.23**	SMC6	RDI1
**CR_02910W_A**	**-3.19**		
**CR_04660C_A**	**-3.16**	UGP1	
**C1_00850W_A**	**-3.14**	IHD2	
**C1_10940C_A**	**-3.14**	ERP5	ERV25
**C2_01780W_A**	**-3.14**	MSB2	UNG1
**C6_03990C_A**	**-3.07**		OST6

**C2_10840W_A**	**-3.06**	RDH54	BNI5
**C6_00400C_A**	**-3.05**		
**C2_10420W_A**	**-3.04**	FGR6-4	
**C1_08140W_A**	**-3.02**		SEC61
**C4_07120C_A**	**-3.02**	MSH2	
**C2_04810W_A**	**-3.01**	HIS3	SPC2
**C4_02900C_A**	**-3.01**	CRH11	
**C4_03990C_A**	**-3.01**		
**C4_05530W_A**	**-3.01**	FGR6-10	
**CR_09600C_A**	**-3.01**	YKE2	
**C2_07120W_A**	**-3.00**	RFA2	NAT4
**CR_00390W_A**	**-2.98**		RLM1
**CR_05690W_A**	**-2.97**	MCT1	
**C1_10630C_A**	**-2.96**		
**CR_01150C_A**	**-2.96**	CTF18	POL30
**CR_02100C_A**	**-2.96**		
**CR_09810W_A**	**-2.92**		
**C4_01420W_A**	**-2.91**		
**C5_03580C_A**	**-2.91**	CAC2	PHO89
**C1_09080C_A**	**-2.88**	PGA6	
**C2_02570W_A**	**-2.88**		ERV29
**C5_03900W_A**	**-2.88**	PGA56	
**C7_02540W_A**	**-2.88**		RLF2
**CR_09560C_A**	**-2.86**		CRH1
**CR_07220C_A**	**-2.84**		
**CR_09120C_A**	**-2.84**	TUB1	
**C2_10830W_A**	**-2.83**		HTD2
**C1_10300W_A**	**-2.79**	MNN12	
**C4_02930W_A**	**-2.79**		
**C1_07850C_A**	**-2.78**		
**C1_02130C_A**	**-2.77**	GAL1	
**C1_01210W_A**	**-2.76**		
**C1_04570C_A**	**-2.76**	CHL4	
**CR_10700W_A**	**-2.76**	RFC4	
**C2_05290C_A**	**-2.75**		
**C3_03490W_A**	**-2.75**	RSN1	AXL2
**C5_00630C_A**	**-2.75**	EXO1	YMR155W
**CR_02070C_A**	**-2.75**	ADH5	
**C7_02750W_A**	**-2.71**	FGR6-1	MRP8
**C1_00830W_A**	**-2.70**		
**C2_03790C_A**	**-2.70**	ASR3	SCS7
**C4_01230C_A**	**-2.69**		FUS2
**C5_01590W_A**	**-2.68**		
**C1_05070C_A**	**-2.66**	GPI7	
**CR_06270W_A**	**-2.66**		TRS130
**C1_07250C_A**	**-2.65**	ALG9	
**C4_07080C_A**	**-2.65**		

**C7_04320W_A**	**-2.65**		
**C2_06860W_A**	**-2.63**	LTE1	
**C3_02150C_A**	**-2.63**	ERG6	PRI2
**C7_04120W_A**	**-2.63**		MSC2
**C5_00070W_A**	**-2.62**	CDC11	
**CR_09180W_A**	**-2.62**	CDG1	MSH2
**C1_13780W_A**	**-2.60**	MYO2	CDC1
**C3_02890C_A**	**-2.59**		SAS4
**C1_12700W_A**	**-2.58**		APC4
**C3_01800C_A**	**-2.58**		CDC11
**C1_11140W_A**	**-2.56**		EXO1
**C3_06410C_A**	**-2.55**		CTF8
**C1_05850W_A**	**-2.54**	POL32	
**C1_12110C_A**	**-2.54**		GPR1
**C3_02910W_A**	**-2.54**		TOF1
**CR_05730C_A**	**-2.54**		ULA1
**C1_03210C_A**	**-2.52**	CDC12	
**C1_06730W_A**	**-2.51**	RAC1	ACH1
**C1_11400C_A**	**-2.51**	GIN4	
**C2_08740W_A**	**-2.51**		SPT21
**C4_01940W_A**	**-2.51**	PHO89	
**C5_00060C_A**	**-2.51**		YNK1
**C5_03950W_A**	**-2.50**		SUT1
**CR_02350C_A**	**-2.50**	GEF2	
**CR_04570C_A**	**-2.50**	CDC10	MLC2
**C2_08450W_A**	**-2.49**		
**C3_01530C_A**	**-2.49**	CAS4	CAC2
**C6_01880W_A**	**-2.49**		
**C1_12790C_A**	**-2.48**	FEN1	
**C2_10570W_A**	**-2.48**	SGS1	SIA1
**C3_04070C_A**	**-2.48**	CDR11	
**C2_04180C_A**	**-2.47**		RFC3
**C4_04430W_A**	**-2.47**	MRP8	
**C4_04530C_A**	**-2.46**	PHR1	MGA1
**C1_13190W_A**	**-2.45**		MAL31
**C2_00690W_A**	**-2.45**		
**C2_04720C_A**	**-2.45**	FGR51	PRI1
**C6_02130C_A**	**-2.45**	CTF5	GAS5
**C1_04490W_A**	**-2.44**		
**C4_01850C_A**	**-2.44**	PDC12	
**CR_09210W_A**	**-2.44**	SUC1	
**C1_00290W_A**	**-2.43**	POL93	
**C2_02860W_A**	**-2.43**	SUR2	FET3
**C1_13020C_A**	**-2.42**	SPC3	MMS4
**C2_00380C_A**	**-2.42**	RFA1	SUN4
**CR_10530W_A**	**-2.42**		
**C4_07200C_A**	**-2.41**		

**C5_05210W_A**	**-2.41**		SCJ1
**C6_03160C_A**	**-2.41**	BMT6	
**CR_09270C_A**	**-2.41**	CBP1	DOG2
**C2_08100W_A**	**-2.40**		
**C5_05390C_A**	**-2.40**	PGA4	
**C7_03160W_A**	**-2.40**		
**CR_10570C_A**	**-2.40**	YHB4	PXL1
**C1_05950C_A**	**-2.39**		MCM21
**C5_03260C_A**	**-2.39**		
**C6_00770C_A**	**-2.39**		
**C7_03800W_A**	**-2.38**		
**C1_03190C_A**	**-2.37**	ECM33	CRT10
**C1_03720C_A**	**-2.37**		
**C2_09650W_A**	**-2.37**		
**C1_02870W_A**	**-2.34**	YPT7	SHE3
**C1_12880C_A**	**-2.34**		
**CR_07590W_A**	**-2.34**	EMP24	
**C1_04620W_A**	**-2.33**		
**C5_02890W_A**	**-2.33**	YNK1	YFL067W
**C7_00820W_A**	**-2.32**	RFC2	
**C1_12010C_A**	**-2.31**	KIP4	
**C3_06250W_A**	**-2.30**	ERV25	
**C3_07810C_A**	**-2.30**	SEC61	
**C4_03200C_A**	**-2.30**		
**C1_12870C_A**	**-2.29**		
**C6_02790C_A**	**-2.29**		
**C1_05740C_A**	**-2.28**		RFC2
**C2_03480C_A**	**-2.28**		
**C3_04190W_A**	**-2.28**		
**C5_03130W_A**	**-2.28**	SUT1	
**C4_03860C_A**	**-2.27**		
**C1_12220W_A**	**-2.26**	CDC6	BEM2
**C4_07210W_A**	**-2.25**		SMC6
**C1_04060W_A**	**-2.24**	IFI3	TOM7
**C4_06080W_A**	**-2.24**		DPP1
**CR_02790C_A**	**-2.24**	ALG5	
**C4_02370C_A**	**-2.23**	PGA59	
**C5_02000C_A**	**-2.23**	ACH1	LMO1
**C5_04000W_A**	**-2.23**	RCF3	
**C6_02000W_A**	**-2.23**	PLB2	
**C7_00100W_A**	**-2.23**	FRP2	
**CR_00180C_A**	**-2.23**	CHT1	YEL1
**CR_06740W_A**	**-2.23**		YNL217W
**C3_04320W_A**	**-2.22**	VAM3	RER1
**CR_02860W_A**	**-2.22**	RHO1	
**C4_02120W_A**	**-2.21**	ERV29	
**C7_01890C_A**	**-2.21**	TOM7	

**CR_08460W_A**	**-2.21**	TPM2	PCK1
**CR_09570W_A**	**-2.21**		CDA2
**C1_12530C_A**	**-2.20**		
**C1_13900C_A**	**-2.20**	LCB2	GSY1
**C2_06730W_A**	**-2.20**	LSP1	
**C4_06340W_A**	**-2.20**	AGO1	CCE1
**C7_01510W_A**	**-2.20**		
**C7_03500W_A**	**-2.20**		LAG1
**C2_05030C_A**	**-2.19**	RHO3	CTF18
**C2_07770C_A**	**-2.19**		
**C3_06610W_A**	**-2.19**		MDM1
**CR_00200W_A**	**-2.19**	PCK1	BIO4
**CR_00910W_A**	**-2.19**		BIO3
**C1_08350C_A**	**-2.18**		
**CR_00780C_A**	**-2.18**	GSY1	
**C2_01340W_A**	**-2.17**		RSR1
**C2_08340C_A**	**-2.17**		RAD51
**C2_08380C_A**	**-2.17**		
**C5_05480W_A**	**-2.17**	DES1	IDP2
**C6_01900C_A**	**-2.17**	DOG1	
**C1_08990C_A**	**-2.16**	KEX2	ALG5
**C1_13510C_A**	**-2.16**	TGL99	RHO1
**CR_05340C_A**	**-2.16**	IFE2	ATG13
**C1_12510W_A**	**-2.15**	RAX2	SMC1
**C2_00640W_A**	**-2.15**		
**C7_00360W_A**	**-2.15**	DFI1	CDC10
**CR_00930W_A**	**-2.15**	ATO10	RBL2
**CR_10610C_A**	**-2.15**		UGP1
**C2_01660C_A**	**-2.14**		
**C5_01790C_A**	**-2.14**		
**C5_03370C_A**	**-2.14**		MCT1
**C6_00270W_A**	**-2.14**		MDM36
**C1_00360W_A**	**-2.13**	CDC13	CDC28
**C1_03950C_A**	**-2.13**		
**C1_00790W_A**	**-2.12**		
**C3_03480C_A**	**-2.12**	ALT1	
**C4_06770W_A**	**-2.12**		
**C4_00620C_A**	**-2.11**	SPC2	SPC97
**C4_01920W_A**	**-2.11**		
**C2_01990C_A**	**-2.10**	FAB1	NDL1
**C4_05790W_A**	**-2.10**		EMP24
**C4_00120W_A**	**-2.09**	PGA7	YHB1
**CR_02200C_A**	**-2.09**	RAD51	
**C1_02150W_A**	**-2.07**	GAL10	TPM1
**C1_08010W_A**	**-2.07**	DPM1	
**C2_04940C_A**	**-2.07**	ITR1	
**C4_06050W_A**	**-2.07**	MED3	TUB1

**CR_06050W_A**	**-2.06**	CDC28	
**C1_13660W_A**	**-2.05**	RAD54	MAL13
**C3_05000W_A**	**-2.05**	RDI1	
**C6_04410C_A**	**-2.05**		
**C1_05100W_A**	**-2.04**		
**C1_11940C_A**	**-2.04**		
**CR_01720W_A**	**-2.04**		YOR292C
**C2_07650C_A**	**-2.03**		YKE2
**C2_09910C_A**	**-2.03**		
**C6_01460C_A**	**-2.03**		
**C1_09130W_A**	**-2.02**		
**C2_02660W_A**	**-2.02**		
**C1_10510W_A**	**-2.01**		APC11
**CR_10000C_A**	**-2.01**		RFC4
**C5_00890C_A**	**-2.00**	GIT2	IMA1

S120 vs B120 Downregulated genes

**Gene I D**	**Fold change**	**Standard gene name (locus name)**
**C4_03470C_A**	**-1,732.65**	ECE1
**C4_03570W_A**	**-528.71**	HWP1
**C6_03030W_A**	**-425.61**	SAP5
**C6_02710C_A**	**-222.69**	SAP6
**C1_07040C_A**	**-153.08**	
**C6_03850C_A**	**-145.49**	IHD1
**CR_07070C_A**	**-96.27**	ALS3
**C1_06280C_A**	**-75.17**	UME6
**C2_00680C_A**	**-52.42**	SOD5
**C5_04930C_A**	**-48.54**	MAL31
**C6_03090W_A**	**-45.97**	
**C2_08890W_A**	**-37.55**	
**C1_05920W_A**	**-34.62**	
**C6_02330W_A**	**-31.74**	
**C3_06660C_A**	**-31.35**	
**C4_02030W_A**	**-26.78**	RFX2
**C4_04080C_A**	**-24.43**	PGA31
**C3_00920W_A**	**-23.01**	ATO1
**C1_13450W_A**	**-22.14**	HYR1
**CR_08510W_A**	**-20.14**	PGA13
**C6_00840W_A**	**-18.61**	GPX2
**C5_03750W_A**	**-18.53**	FAV2
**C4_04070C_A**	**-15.85**	PGA30
**C4_03460C_A**	**-14.54**	
**C7_00630C_A**	**-14.29**	
**C5_03930C_A**	**-14.11**	
**C2_00670C_A**	**-13.51**	
**C1_00680W_A**	**-13.5**	ASM3
**C5_02460C_A**	**-13.31**	ECM331

**C1_06140C_A**	**-13.13**	
**C3_03460C_A**	**-12.7**	
**C1_13100W_A**	**-12.61**	
**C2_07720C_A**	**-12.59**	
**C3_07470W_A**	**-12.29**	
**C3_02330C_A**	**-11.91**	
**C4_00720W_A**	**-11.8**	CSP2
**C4_04380C_A**	**-11.42**	
**C2_02390W_A**	**-10.94**	
**CR_03890W_A**	**-10.63**	WOR3
**CR_08730W_A**	**-10.48**	tE(UUC)7
**C4_03520C_A**	**-10.31**	RBT1
**C3_05990C_A**	**-10.01**	
**C1_00780C_A**	**-10**	HGC1
**C1_10300W_A**	**-9.97**	MNN12
**C1_02730W_A**	**-9.87**	
**C7_02500C_A**	**-9.43**	DPP3
**C1_02630C_A**	**-8.55**	EXG2
**C3_05750C_A**	**-8.49**	
**CR_02210W_A**	**-8.46**	
**C7_03110W_A**	**-8.45**	PGA28
**C6_04420W_A**	**-8.26**	
**CR_06510W_A**	**-8.17**	
**C5_04220W_A**	**-8.09**	MRV5
**CR_06500C_A**	**-7.97**	
**C4_04170C_A**	**-7.88**	AXL2
**CR_10100C_A**	**-7.81**	INO1
**C6_02100W_A**	**-7.66**	
**C5_00010W_A**	**-7.44**	TLO11
**C4_05560C_A**	**-7.37**	ARO9
**CR_04680C_A**	**-7.31**	
**C7_03150W_A**	**-7.24**	
**C7_03480W_A**	**-7.15**	
**C2_09490W_A**	**-7.07**	PHO13
**C5_03430W_A**	**-7.01**	
**CR_09440C_A**	**-6.85**	
**C2_01330C_A**	**-6.72**	SIR2
**C2_00690W_A**	**-6.54**	
**C7_00090C_A**	**-6.5**	CSA1
**C1_10210C_A**	**-6.43**	CLA4
**C3_01360C_A**	**-6.38**	IRO1
**C3_01020W_A**	**-6.22**	
**C6_00440C_A**	**-6.2**	FET34
**CR_09530C_A**	**-6.2**	
**C2_06680W_A**	**-6.14**	FRP3
**CR_02140W_A**	**-6.12**	RSR1
**C1_08230C_A**	**-6.03**	PLB5

**C1_12790C_A**	**-5.88**	FEN1
**C1_14130W_A**	**-5.79**	FTR1
**C4_05250W_A**	**-5.76**	
**C4_01320C_A**	**-5.75**	OPT6
**C3_06130W_A**	**-5.71**	
**C3_06000W_A**	**-5.68**	AHR1
**C1_12010C_A**	**-5.52**	KIP4
**C3_02280C_A**	**-5.48**	GFA1
**CR_07220C_A**	**-5.45**	
**C7_00770W_A**	**-5.3**	
**CR_01530C_A**	**-5.23**	
**CR_06660W_A**	**-5.05**	SEO1
**C3_05330C_A**	**-4.97**	
**C1_05950C_A**	**-4.96**	
**C2_07180W_A**	**-4.92**	
**CR_07680C_A**	**-4.91**	
**C2_09530W_A**	**-4.88**	EAP1
**C6_01070C_A**	**-4.87**	CIP1
**C5_01260W_A**	**-4.85**	
**C3_00650W_A**	**-4.83**	NGT1
**C6_02210W_A**	**-4.81**	
**C1_07030C_A**	**-4.77**	RBT4
**C5_02600W_A**	**-4.64**	PUT1
**C1_07370C_A**	**-4.54**	CPH1
**C4_01220C_A**	**-4.54**	
**C2_06860W_A**	**-4.53**	LTE1
**C1_01210W_A**	**-4.49**	
**C5_01990W_A**	**-4.41**	
**C7_01680C_A**	**-4.41**	
**CR_02910W_A**	**-4.35**	
**C4_01260W_A**	**-4.27**	RLM1
**C4_03070W_A**	**-4.25**	PGA54
**C5_01240W_A**	**-4.16**	AUR1
**C4_04790W_A**	**-4.13**	FAV1
**C3_01550C_A**	**-4.1**	TOS1
**CR_09070C_A**	**-4.07**	
**C3_01470W_A**	**-4.06**	ARF3
**CR_04440C_A**	**-4.04**	RBR1
**C6_03100W_A**	**-4.03**	SHE3
**C2_04050C_A**	**-4.02**	DCK1
**C5_01590W_A**	**-3.99**	
**C1_08900W_A**	**-3.98**	
**CR_01060W_A**	**-3.98**	LAG1
**C2_08750W_A**	**-3.97**	
**C1_00710C_A**	**-3.92**	TUB2
**C7_01500W_A**	**-3.87**	
**C4_04860W_A**	**-3.86**	

**C6_00830C_A**	**-3.83**	CAN3
**C5_01250W_A**	**-3.78**	GPR1
**C1_07220W_A**	**-3.76**	
**C5_03900W_A**	**-3.73**	PGA56
**C1_06270W_A**	**-3.67**	
**C5_01070C_A**	**-3.65**	
**C1_00850W_A**	**-3.6**	IHD2
**C6_03500C_A**	**-3.59**	SAP4
**C2_07750W_A**	**-3.57**	RHO2
**C6_00820W_A**	**-3.57**	SUN41
**CR_07240C_A**	**-3.57**	CLG1
**C1_10270C_A**	**-3.56**	SFH5
**C3_04070C_A**	**-3.54**	CDR11
**CR_06920W_A**	**-3.53**	
**CR_07810W_A**	**-3.52**	YHB5
**C4_04980W_A**	**-3.51**	
**CR_10340W_A**	**-3.5**	PTP3
**C4_06490C_A**	**-3.49**	PRI2
**C1_06340W_A**	**-3.45**	
**C7_00100W_A**	**-3.45**	FRP2
**C3_01730C_A**	**-3.43**	UTR2
**C6_02090C_A**	**-3.43**	
**CR_09120C_A**	**-3.43**	TUB1
**C6_01710C_A**	**-3.41**	PTK2
**C1_00400W_A**	**-3.4**	SVF1
**C2_04970W_A**	**-3.4**	ABP2
**C2_10210C_A**	**-3.4**	RAS1
**C5_03260C_A**	**-3.4**	
**C1_03190C_A**	**-3.38**	ECM33
**CR_09060W_A**	**-3.38**	
**C2_09810C_A**	**-3.36**	
**C1_09320C_A**	**-3.34**	
**C1_01980W_A**	**-3.33**	HGT1
**C3_06670C_A**	**-3.27**	
**C1_10580C_A**	**-3.24**	
**C7_00360W_A**	**-3.24**	DFI1
**C5_01220W_A**	**-3.23**	
**CR_09600C_A**	**-3.22**	YKE2
**C2_07730W_A**	**-3.2**	YVC1
**C2_08450W_A**	**-3.2**	
**C2_06930C_A**	**-3.19**	
**C3_02720W_A**	**-3.19**	
**C5_00620W_A**	**-3.18**	
**C1_09870W_A**	**-3.16**	HCM1
**C6_01530C_A**	**-3.16**	
**C4_04530C_A**	**-3.12**	PHR1
**CR_09270C_A**	**-3.12**	CBP1

**C2_00640W_A**	**-3.11**	
**C3_04350C_A**	**-3.09**	
**C3_04610W_A**	**-3.09**	MEA1
**C5_03020W_A**	**-3.09**	CCC2
**CR_03790C_A**	**-3.09**	KRE1
**C7_03500W_A**	**-3.08**	
**CR_09570W_A**	**-3.08**	
**C1_06250W_A**	**-3.07**	
**CR_09880W_A**	**-3.06**	DEF1
**C1_12510W_A**	**-3.04**	RAX2
**C7_01550W_A**	**-3.04**	BEM2
**CR_02370W_A**	**-2.97**	ERG25
**C5_01440C_A**	**-2.96**	
**C4_01240C_A**	**-2.94**	
**C2_09480W_A**	**-2.93**	
**C3_06400C_A**	**-2.92**	
**C3_01800C_A**	**-2.9**	
**C4_01420W_A**	**-2.89**	
**C4_01860C_A**	**-2.87**	
**CR_09560C_A**	**-2.87**	
**C2_04430W_A**	**-2.86**	
**C4_02320C_A**	**-2.86**	SOD1
**C5_00070W_A**	**-2.83**	CDC11
**C5_02530W_A**	**-2.83**	UAP1
**CR_00910W_A**	**-2.83**	
**C2_05740W_A**	**-2.82**	SPO7
**CR_02860W_A**	**-2.81**	RHO1
**C1_13110C_A**	**-2.79**	CHS3
**C2_00790C_A**	**-2.79**	
**C2_04450W_A**	**-2.78**	
**C2_10310C_A**	**-2.78**	KIP2
**C2_01980C_A**	**-2.77**	SRD1
**CR_09580C_A**	**-2.77**	
**CR_07790C_A**	**-2.76**	YHB1
**C3_02290W_A**	**-2.75**	
**C4_02930W_A**	**-2.75**	
**CR_00620C_A**	**-2.74**	ARG1
**C1_01780C_A**	**-2.73**	HOL4
**C3_05220W_A**	**-2.71**	CDR1
**C5_03950W_A**	**-2.71**	
**C2_01890W_A**	**-2.7**	CTF1
**C5_02470W_A**	**-2.7**	INT1
**C5_03170C_A**	**-2.7**	
**C6_03070C_A**	**-2.7**	
**C2_01780W_A**	**-2.69**	MSB2
**C1_00830W_A**	**-2.68**	
**C1_02470W_A**	**-2.68**	

**C5_01100C_A**	**-2.68**	CLN3
**C1_00800C_A**	**-2.67**	ERG2
**C2_06740W_A**	**-2.65**	
**C3_02820C_A**	**-2.65**	
**CR_08460W_A**	**-2.65**	TPM2
**C7_03080W_A**	**-2.62**	LMO1
**CR_01280C_A**	**-2.61**	
**C1_12200W_A**	**-2.6**	MCD4
**C7_01230C_A**	**-2.6**	
**CR_09210W_A**	**-2.59**	SUC1
**C1_14180W_A**	**-2.58**	
**C6_02160W_A**	**-2.58**	
**CR_04830C_A**	**-2.58**	
**C6_01960W_A**	**-2.57**	
**CR_06790C_A**	**-2.57**	
**C1_05980W_A**	**-2.56**	
**C2_08100W_A**	**-2.56**	
**C5_00890C_A**	**-2.56**	GIT2
**C4_01250W_A**	**-2.55**	NAT4
**C2_09420W_A**	**-2.54**	
**C5_00230C_A**	**-2.54**	
**CR_02350C_A**	**-2.54**	GEF2
**C4_00120W_A**	**-2.53**	PGA7
**C4_01380W_A**	**-2.53**	ZCF27
**C7_02540W_A**	**-2.53**	
**C2_09740W_A**	**-2.52**	SSO2
**C4_04850C_A**	**-2.52**	CSR1
**CR_09520C_A**	**-2.51**	
**C3_05600W_A**	**-2.5**	ATO6
**C6_00400C_A**	**-2.5**	
**C1_06320W_A**	**-2.49**	
**C1_07770W_A**	**-2.49**	FGR6-3
**C5_01500C_A**	**-2.49**	ZCF20
**C7_03440W_A**	**-2.49**	SNG4
**C1_09430W_A**	**-2.47**	
**C3_02150C_A**	**-2.47**	ERG6
**C6_01620W_A**	**-2.47**	
**C2_09700W_A**	**-2.46**	YEA4
**C5_02370C_A**	**-2.46**	
**C6_03260W_A**	**-2.45**	
**C7_01840W_A**	**-2.44**	IQG1
**C2_08340C_A**	**-2.43**	
**CR_04630C_A**	**-2.43**	DIT2
**C1_04620W_A**	**-2.42**	
**C4_04250W_A**	**-2.42**	
**C4_05530W_A**	**-2.42**	FGR6-10
**C1_08620W_A**	**-2.41**	CTR2

**C7_03410C_A**	**-2.41**	
**C2_05030C_A**	**-2.4**	RHO3
**C1_13780W_A**	**-2.39**	MYO2
**C5_03650C_A**	**-2.39**	
**C2_09760W_A**	**-2.38**	
**C4_05900C_A**	**-2.38**	
**C1_10060C_A**	**-2.36**	
**C2_06870C_A**	**-2.36**	PST1
**C1_11730W_A**	**-2.34**	
**C2_07220W_A**	**-2.34**	
**C5_01650C_A**	**-2.34**	MYO1
**C1_09120W_A**	**-2.33**	IPT1
**C1_11400C_A**	**-2.33**	GIN4
**C2_05260W_A**	**-2.33**	BUD14
**C7_03420C_A**	**-2.32**	SNG3
**CR_04660C_A**	**-2.32**	UGP1
**C2_03790C_A**	**-2.31**	ASR3
**C2_03320W_A**	**-2.3**	CHK1
**C2_09220W_A**	**-2.3**	DDR48
**C4_04760C_A**	**-2.3**	
**C5_02780W_A**	**-2.3**	
**CR_05690W_A**	**-2.3**	MCT1
**C2_08140C_A**	**-2.29**	PHM7
**C5_01680C_A**	**-2.29**	CCN1
**C6_03310W_A**	**-2.29**	
**C1_11250W_A**	**-2.28**	
**CR_04570C_A**	**-2.28**	CDC10
**C1_05960W_A**	**-2.27**	PGA45
**C3_01940C_A**	**-2.27**	
**C4_00130W_A**	**-2.27**	RBT5
**C1_00350C_A**	**-2.26**	HMX1
**C1_03710C_A**	**-2.26**	MSB1
**C3_02930W_A**	**-2.26**	
**CR_06740W_A**	**-2.26**	
**C1_00580W_A**	**-2.25**	
**C1_03210C_A**	**-2.25**	CDC12
**C4_05980C_A**	**-2.25**	
**C4_06770W_A**	**-2.25**	
**CR_09020C_A**	**-2.25**	CHS2
**C1_01260C_A**	**-2.24**	TRS20
**C2_04310W_A**	**-2.23**	ERG10
**C3_05650W_A**	**-2.23**	YCK2
**CR_04770C_A**	**-2.22**	
**C3_05350C_A**	**-2.21**	SMI1B
**CR_09050C_A**	**-2.21**	
**C1_13900C_A**	**-2.2**	LCB2
**C1_01620C_A**	**-2.19**	

**C1_12220W_A**	**-2.19**	CDC6
**C4_01920W_A**	**-2.18**	
**CR_06440C_A**	**-2.18**	BCR1
**C1_00840C_A**	**-2.17**	NRM1
**C3_07200C_A**	**-2.17**	ZFU2
**C1_13740W_A**	**-2.16**	KAR9
**C3_00760W_A**	**-2.16**	ERG4
**C3_04490W_A**	**-2.16**	RAX1
**C1_02870W_A**	**-2.14**	YPT7
**C2_01340W_A**	**-2.14**	
**C3_01530C_A**	**-2.14**	CAS4
**C3_04320W_A**	**-2.14**	VAM3
**C4_00110C_A**	**-2.14**	FRP1
**C2_05580W_A**	**-2.13**	
**C2_06730W_A**	**-2.13**	LSP1
**C1_04210C_A**	**-2.12**	CDC3
**C4_04430W_A**	**-2.12**	MRP8
**C4_04590W_A**	**-2.12**	SCS7
**CR_09690C_A**	**-2.12**	PLB3
**C3_05880C_A**	**-2.11**	
**C5_04840C_A**	**-2.1**	
**C6_02860W_A**	**-2.1**	STE23
**C6_02200C_A**	**-2.09**	
**C1_12470W_A**	**-2.07**	
**C4_01230C_A**	**-2.07**	
**C5_04260W_A**	**-2.07**	
**C7_00230W_A**	**-2.07**	
**CR_02360W_A**	**-2.07**	IDP2
**C1_03080C_A**	**-2.06**	TEM1
**CR_03780C_A**	**-2.05**	
**CR_10610C_A**	**-2.05**	
**C2_03610W_A**	**-2.04**	FMO2
**C3_02270W_A**	**-2.04**	
**C3_05000W_A**	**-2.04**	RDI1
**C4_05440C_A**	**-2.04**	
**C6_02130C_A**	**-2.04**	CTF5
**C7_02770W_A**	**-2.04**	CHS1
**CR_05540C_A**	**-2.04**	
**C3_06420C_A**	**-2.02**	
**C7_00760C_A**	**-2.02**	
**CR_02650C_A**	**-2.01**	DRE2
**C1_12740W_A**	**-2**	
**C4_00620C_A**	**-2**	SPC2
**C4_05080C_A**	**-2**	
**C6_03230W_A**	**-2**	ARG3
