# Supplementary material for: Reprogramming in Candida albicans Gene Expression Network under Butanol Stress Abrogates Hyphal Development
Source: Int J Mol Sci. 2023 Dec 7;24(24):17227. doi: 10.3390/ijms242417227 (PMC10743114; doi:10.3390/ijms242417227)
Supplement: Supplementary file 1 [file ijms-24-17227-s001.zip › Table S3.pdf]

Table S3: Gene Ontology processes enriched in the induced and repressed genes sets obtained by comparing serum vs serum with butanol gene expression RPKM values

S20 vs B20 Upregulated genes

GOID	GO_term	Cluster frequency	Background frequency	Corrected P-value	False discovery rate	Gene(s) annotated to the term
34660	ncRNA metabolic process	464 out of 1061 genes, 43.7%	935 out of 6473 background genes, 14.4%	3.02E-149	0.00%	HBR1:CNS1:RPO41:NRM1:RRS1:TRI1:C1_01150C_A:C1_01160C_A:TRM2:C1_01530C_A:C1_02090C_A:ABP140:C1_02450C_A:PDE2:C1_02900C_A:TSR2:C1_03630W_A:RPP1:C1_03830C_A:C1_04040C_A:C1_04120C_A:ERB1:NOP4:C1_04510W_A:C1_04530C_A:C1_04710C_A:C1_04970W_A:RRP6:REI1:TRY2:C1_05220C_A:C1_05360C_A:C1_05380C_A:RSM22:RPS27A:FAL1:C1_05650W_A:C1_06540C_A:NOP6:C1_06760C_A:RIA1:LHP1:C1_07510W_A:C1_07660W_A:ERD1:C1_07950C_A:C1_07960W_A:FUN12:MLT1:DIP2:GCR3:GCD6:C1_08630W_A:MSS116:CEF1:C1_09390W_A:C1_09710C_A:C1_09790C_A:PDC2:DBP3:MNN23:FCY23:C1_10620W_A:RPA190:C1_10920W_A:C1_10950C_A:C1_10970W_A:C1_11000C_A:NEP1:KRR1:GAR1:MUP1:C1_11900C_A:C1_11910W_A:TRP3:C1_12280C_A:CSI2:C1_12350W_A:WRS1:C1_12440W_A:C1_12570C_A:C1_12630C_A:C1_12750C_A:C1_12760W_A:C1_12820C_A:REX3:RMP1:C1_13380W_A:MCU1:C1_13820C_A:C1_14080W_A:C1_14330W_A:MPP10:FAV3:C2_00200W_A:C2_00280C_A:C2_00410C_A:C2_00820W_A:C2_00840W_A:C2_01070W_A:C2_01220W_A:C2_01420C_A:REX2:C2_01820C_A:C2_01860C_A:C2_02120W_A:C2_02420C_A:UTP21:UTP22:PRS1:C2_02540W_A:C2_02630W_A:PDR17:MET1:PRP3:SCH9:C2_04120C_A:TBF1:SUV3:RIM2:C2_04570W_A:SNU114:C2_04700C_A:C2_04820W_A:PZF1:CKA2:C2_05050C_A:C2_05080C_A:MAK5:C2_05160C_A:RPF2:RPC40:KTI11:C2_05670C_A:C2_05750W_A:C2_05830C_A:C2_05840W_A:LAS1:ECM17:MAK16:C2_06480W_A:C2_06520C_A:C2_06530W_A:VAS1:C2_06660W_A:FHL1:C2_06850W_A:NOC4:C2_07360W_A:RCL1:PRS5:C2_07920W_A:NSA1:C2_08180C_A:EAF3:RRP8:C2_08530C_A:MSU1:UTP15:C2_09160W_A:C2_09180W_A:C2_09310C_A:PES1:TAZ1:RRP15:C2_09500W_A:C2_09660W_A:SMM1:C2_09920W_A:CWC22:PUS7:C2_10740C_A:RPC11:C2_10810W_A:RPS24:C3_00100W_A:UTP8:BUD21:SOF1:C3_00660W_A:C3_00830C_A:BMS1:ARP9:UTP20:NCE103:C3_01310W_A:C3_01560W_A:C3_02020W_A:C3_02040C_A:UTP4:ILV2:C3_02350W_A:C3_02670W_A:C3_02840W_A:C3_02850C_A:C3_03070W_A:C3_03110W_A:C3_03330C_A:HBR3:C3_04370C_A:C3_04380C_A:MAK21:ARV1:SFP1:NOP14:CEM1:C3_05140C_A:C3_05160C_A:RPL8B:C3_05280C_A:C3_05380W_A:C3_05440C_A:QDR2:C3_05800W_A:C3_05860C_A:TRM12:C3_05900W_A:NOG1:C3_06150W_A:DOT1:ISW2:C3_06370C_A:NSA2:PRP5:NOP13:C3_07400W_A:C3_07550C_A:C3_07570C_A:RIT1:RAD4:UTP9:C3_

						07800C_A:DUR4:RLP24:NMD5:C4_00690C_A:C4_00740W_A:C4_00800W_A:C4_00810C_A:C4_00880W_A:C4_00940W_A:RAT1:C4_01280C_A:PWP1:C4_01500W_A:SLX4:HIT1:SAS10:RPC53:HCA4:C4_02850W_A:ZUO1:C4_02880C_A:NAN1:C4_03140C_A:C4_03170W_A:DAO2:C4_03720C_A:C4_03730C_A:C4_03740W_A:NUP84:C4_03830W_A:RRP42:RAM1:ECM1:DUO1:C4_04520W_A:SSZ1:BAS1:C4_04810C_A:AGP3:RPL30:C4_05230C_A:C4_05260W_A:C4_05360C_A:C4_05650W_A:C4_06210C_A:C4_06410W_A:NOP1:C4_06790W_A:C4_06950W_A:C4_07060W_A:C4_07150W_A:DUS4:MRP17:RMT2:C5_00920W_A:BUD23:CYC3:PUS4:C5_01430C_A:FYV5:SPB4:C5_01610W_A:C5_01930W_A:C5_02010C_A:C5_02070C_A:RIX7:C5_02440C_A:SPR28:UTP13:CKB2:MSW1:C5_03010W_A:RMS1:RPO26:C5_03400C_A:C5_03920C_A:C5_04120C_A:MSM1:HAS1:C5_04840C_A:C5_04910W_A:GRF10:C5_05340W_A:NOP5:CDC14:TOP1:C6_01040C_A:C6_01120C_A:CIC1:C6_01890C_A:SWD3:C6_02230W_A:ALG11:C6_02290C_A:C6_02350C_A:C6_02430W_A:MRT4:C6_02900C_A:POP4:C6_03210C_A:C6_03380W_A:C6_03390W_A:C6_03440W_A:NAM2:SPB1:C6_04240W_A:NOP8:ILS1:C6_04530C_A:C7_00160C_A:C7_00220W_A:C7_00330C_A:THG1:RPA135:NOP15:C7_01030C_A:DBP7:C7_01210C_A:C7_01270C_A:C7_01360C_A:C7_01400C_A:GIR2:FLU1:C7_01570C_A:C7_01950W_A:LIG1:C7_02340C_A:C7_02460C_A:C7_02660C_A:C7_02930C_A:C7_03400C_A:ISY1:ENP2:C7_03590C_A:ENP1:C7_03850W_A:C7_03880C_A:C7_04150W_A:UTP18:CR_00430C_A:CR_00460C_A:BUD22:PWP2:CR_00830W_A:TRM1:DAL81:CR_01320C_A:CR_01410C_A:PRS:CR_01550C_A:CR_01600C_A:CR_01670W_A:CDC60:CR_01700C_A:CR_01710W_A:CR_01780W_A:CR_01950W_A:CR_02030C_A:MCM6:CR_02420W_A:CR_02550C_A:RRP9:RPC31:CR_02890C_A:SRP40:ARC1:SMC5:CR_03110W_A:CR_03200C_A:CR_03230W_A:CR_03360W_A:KTI12:CR_03400W_A:NCS2:YVH1:SMC1:PRP42:CR_03940W_A:CR_04110W_A:CR_04160C_A:CR_04170W_A:CR_04240C_A:CR_04300W_A:CR_04500C_A:CR_04710W_A:RPB8:NOC2:CR_05550C_A:RNR3:MTG2:DBP8:RFX1:RPL7:CR_06230W_A:FGR50:CR_06450W_A:RRN11:CR_06680C_A:NMD3:CR_06840W_A:CR_06970C_A:POL2:CR_07030C_A:CR_07310W_A:PIF1:FUN30:CR_07640C_A:DBP6:IMP4:CR_08000C_A:NOP10:SSB1:RIO2:ATS1:CR_08330W_A:CR_08410W_A:TSR1:CR_08940W_A:MPS1:SSF1:CR_09740W_A:MED21:CR_09800C_A:DPB2:SIK1:UTP5:CR_10260W_A:CR_10410C_A:CR_10470C_A:DRS1:LTV1:POP3

34470	ncRNA processing	441 out of 1061 genes, 41.6%	860 out of 6473 background genes, 13.3%	3.45E-147	0.00%	HBR1:CNS1:RPO41:NRM1:RRS1:TRI1:C1_01150C_A:C1_01160C_A:TRM2:C1_02090C_A:ABP140:C1_02450C_A:PDE2:C1_02900C_A:TSR2:C1_03630W_A:RPP1:C1_03830C_A:C1_04040C_A:C1_04120C_A:ERB1:NOP4:C1_04510W_A:C1_04530C_A:C1_04710C_A:C1_04970W_A:RRP6:REI1:TRY2:C1_05220C_A:C1_05360C_A:C1_05380C_A:RSM22:RPS27A:FAL1:C1_05650W_A:C1_06540C_A:NOP6:C1_06760C_A:RIA1:LHP1:C1_07510W_A:C1_07660W_A:ERD1:C1_07950C_A:C1_07960W_A:FUN12:MLT1:DIP2:GCR3:GCD6:C1_08630W_A:MSS116:CEF1:C1_09390W_A:C1_09710C_A:C1_09790C_A:PDC2:DBP3:MNN23:FCY23:C1_10620W_A:C1_10950C_A:C1_10970W_A:C1_11000C_A:NEP1:KRR1:MUP1:C1_11900C_A:C1_11910W_A:TRP3:C1_12280C_A:CSI2:C1_12350W_A:C1_12440W_A:C1_12570C_A:C1_12630C_A:C1_12750C_A:C1_12760W_A:C1_12820C_A:REX3:RMP1:C1_13380W_A:MCU1:C1_13820C_A:C1_14080W_A:C1_14330W_A:MPP10:FAV3:C2_00200W_A:C2_00280C_A:C2_00410C_A:C2_00820W_A:C2_00840W_A:C2_01070W_A:C2_01220W_A:C2_01420C_A:REX2:C2_01820C_A:C2_01860C_A:C2_02120W_A:C2_02420C_A:UTP21:UTP22:PRS1:C2_02540W_A:C2_02630W_A:PDR17:MET1:PRP3:SCH9:C2_04120C_A:TBF1:SUV3:RIM2:C2_04570W_A:SNU114:C2_04700C_A:C2_04820W_A:PZF1:CKA2:C2_05050C_A:C2_05080C_A:MAK5:C2_05160C_A:RPF2:KTI11:C2_05670C_A:C2_05750W_A:C2_05830C_A:C2_05840W_A:LAS1:ECM17:MAK16:C2_06480W_A:C2_06520C_A:C2_06530W_A:C2_06660W_A:FHL1:C2_06850W_A:NOC4:C2_07360W_A:RCL1:PRS5:C2_07920W_A:NSA1:C2_08180C_A:EAF3:RRP8:C2_08530C_A:MSU1:UTP15:C2_09160W_A:C2_09180W_A:C2_09310C_A:PES1:TAZ1:RRP15:C2_09500W_A:C2_09660W_A:SMM1:C2_09920W_A:CWC22:PUS7:C2_10740C_A:C2_10810W_A:RPS24:C3_00100W_A:UTP8:BUD21:SOF1:C3_00660W_A:C3_00830C_A:BMS1:ARP9:UTP20:NCE103:C3_01310W_A:C3_01560W_A:C3_02020W_A:C3_02040C_A:UTP4:ILV2:C3_02350W_A:C3_02670W_A:C3_02840W_A:C3_02850C_A:C3_03070W_A:C3_03110W_A:C3_03330C_A:HBR3:C3_04370C_A:C3_04380C_A:MAK21:ARV1:SFP1:NOP14:CEM1:C3_05140C_A:C3_05160C_A:RPL8B:C3_05280C_A:C3_05380W_A:C3_05440C_A:QDR2:C3_05800W_A:C3_05860C_A:TRM12:C3_05900W_A:NOG1:C3_06150W_A:DOT1:ISW2:C3_06370C_A:NSA2:PRP5:NOP13:C3_07400W_A:C3_07550C_A:C3_07570C_A:RIT1:RAD4:UTP9:C3_07800C_A:DUR4:RLP24:NMD5:C4_00690C_A:C4_00740W_A:C4_00800W_A:C4_00810C_A:C4_00880W_A:C4_00940W_A:RAT1:C4_01280C_A:PWP1:C4_01500W_A:SLX4:HIT1:SAS10:RPC53:HCA4:C4_02850W_A:ZUO1:C4_02880C_A:NAN1:C4_03140C_A:C4_03170W_A:DAO2:C4_03720C_A:C4_03730C_A:C4_03740W_A:NUP84:C4_03830W_A:RRP42:RAM1:ECM1:DUO1:C4_04520W_A:SSZ1:BAS1:C4_04810C_A:AGP3:RPL30:C4_05230C_A:C4_05260W_A:C4_05360C_A:C4_05650W_A:C4_06210C_A:C4_06410W_A:NOP1:C4_06790W_A:C4_06950W_A:C4_07060W_A:C4_07150W_A:DUS4:MRP17:RMT2:C5_00920W_A:BUD23:CYC3:PUS4:C5_01430C_A:FYV5:SPB4:C5_01610W_A:C5_01930W_A:C5_02010C_A:C5_02070C_A:RIX7:C5_024

						40C_A:SPR28:UTP13:CKB2:C5_03010W_A:RMS1:C5_03400C_A:C5_03920C_A:C5_04120C_A:HAS1:C5_04840C_A:C5_04910W_A:GRF10:C5_05340W_A:NOP5:CDC14:TOP1:C6_01040C_A:C6_01120C_A:CIC1:C6_01890C_A:SWD3:C6_02230W_A:ALG11:C6_02290C_A:C6_02350C_A:C6_02430W_A:MRT4:C6_02900C_A:POP4:C6_03210C_A:C6_03380W_A:C6_03390W_A:C6_03440W_A:SPB1:C6_04240W_A:NOP8:C6_04530C_A:C7_00160C_A:C7_00220W_A:C7_00330C_A:THG1:NOP15:C7_01030C_A:DBP7:C7_01210C_A:C7_01270C_A:C7_01360C_A:GIR2:FLU1:C7_01570C_A:C7_01950W_A:LIG1:C7_02340C_A:C7_02460C_A:C7_02660C_A:C7_02930C_A:C7_03400C_A:ISY1:ENP2:C7_03590C_A:ENP1:C7_03850W_A:C7_03880C_A:C7_04150W_A:UTP18:CR_00430C_A:CR_00460C_A:BUD22:PWP2:CR_00830W_A:TRM1:DAL81:CR_01320C_A:CR_01410C_A:CR_01550C_A:CR_01600C_A:CR_01670W_A:CR_01700C_A:CR_01710W_A:CR_01780W_A:CR_02030C_A:MCM6:CR_02420W_A:CR_02550C_A:RRP9:CR_02890C_A:SRP40:SMC5:CR_03200C_A:CR_03230W_A:CR_03360W_A:KTI12:NCS2:YVH1:SMC1:PRP42:CR_03940W_A:CR_04110W_A:CR_04160C_A:CR_04170W_A:CR_04240C_A:CR_04300W_A:CR_04500C_A:CR_04710W_A:NOC2:CR_05550C_A:RNR3:MTG2:DBP8:RFX1:RPL7:CR_06230W_A:FGR50:CR_06450W_A:RRN11:CR_06680C_A:NMD3:CR_06840W_A:CR_06970C_A:POL2:CR_07030C_A:CR_07310W_A:PIF1:FUN30:CR_07640C_A:DBP6:IMP4:CR_08000C_A:NOP10:SSB1:RIO2:ATS1:CR_08330W_A:CR_08410W_A:TSR1:CR_08940W_A:MPS1:SSF1:CR_09740W_A:MED21:CR_09800C_A:DPB2:SIK1:UTP5:CR_10260W_A:CR_10410C_A:CR_10470C_A:DRS1:LTV1:POP3

42254	ribosome biogenesis	448 out of 1061 genes, 42.2%	886 out of 6473 background genes, 13.7%	6.16E-147	0.00%	HBR1:CNS1:RPO41:NRM1:RRS1:TRI1:C1_01160C_A:C1_02090C_A:ABP140:C1_02450C_A:PDE2:C1_02900C_A:TSR2:ARX1:C1_03630W_A:RPP1:C1_03830C_A:C1_04040C_A:C1_04120C_A:ERB1:NOP4:C1_04510W_A:C1_04710C_A:C1_04970W_A:RRP6:REI1:TRY2:C1_05220C_A:C1_05360C_A:C1_05380C_A:RSM22:RPS27A:FAL1:C1_05650W_A:C1_06530C_A:C1_06540C_A:NOP6:C1_06760C_A:RIA1:C1_07510W_A:C1_07660W_A:ERD1:C1_07950C_A:C1_07960W_A:FUN12:MLT1:DIP2:GCR3:GCD6:C1_08630W_A:MSS116:JIP5:C1_09040C_A:CEF1:C1_09390W_A:YTM1:C1_09710C_A:C1_09790C_A:PDC2:DBP3:MNN23:FCY23:C1_10620W_A:RPA190:C1_10920W_A:C1_10950C_A:C1_10970W_A:C1_11000C_A:NEP1:KRR1:GAR1:MUP1:C1_11900C_A:C1_11910W_A:TRP3:C1_12280C_A:CSI2:C1_12350W_A:C1_12440W_A:C1_12570C_A:C1_12630C_A:C1_12680W_A:C1_12750C_A:C1_12760W_A:C1_12820C_A:REX3:RMP1:C1_13370W_A:C1_13380W_A:MCU1:C1_13820C_A:C1_14080W_A:C1_14330W_A:MPP10:FAV3:C2_00200W_A:C2_00280C_A:C2_00410C_A:C2_00820W_A:C2_00840W_A:C2_01070W_A:C2_01220W_A:C2_01420C_A:REX2:C2_01820C_A:C2_01860C_A:C2_02120W_A:C2_02420C_A:UTP21:UTP22:PRS1:C2_02540W_A:C2_02630W_A:PDR17:MET1:PRP3:SCH9:C2_04120C_A:TBF1:SUV3:RIM2:C2_04570W_A:SNU114:C2_04700C_A:C2_04820W_A:PZF1:CKA2:C2_05050C_A:C2_05080C_A:MAK5:C2_05160C_A:RPF2:C2_05670C_A:C2_05750W_A:C2_05830C_A:C2_05840W_A:LAS1:ECM17:MAK16:C2_06520C_A:C2_06530W_A:C2_06660W_A:RPL11:FHL1:C2_06850W_A:NOC4:C2_07360W_A:RCL1:PRS5:C2_07920W_A:NSA1:KRE30:RPS10:C2_08180C_A:EAF3:RRP8:C2_08530C_A:MSU1:UTP15:MFG1:C2_09160W_A:C2_09180W_A:MED18:C2_09310C_A:PES1:TAZ1:RRP15:C2_09660W_A:SMM1:C2_09920W_A:CWC22:PUS7:C2_10740C_A:C2_10810W_A:RPS24:C3_00100W_A:UTP8:BUD21:SOF1:C3_00660W_A:C3_00830C_A:BMS1:ARP9:UTP20:NCE103:C3_01310W_A:RPS7A:C3_01560W_A:C3_02020W_A:C3_02040C_A:UTP4:ILV2:C3_02350W_A:C3_02670W_A:SMC3:C3_02840W_A:C3_02850C_A:C3_03070W_A:C3_03110W_A:C3_03330C_A:HBR3:C3_04370C_A:C3_04380C_A:MAK21:RPS15:ARV1:SFP1:NOP14:CEM1:C3_05160C_A:RPL8B:C3_05280C_A:C3_05380W_A:C3_05440C_A:QDR2:C3_05800W_A:C3_05860C_A:C3_05900W_A:NOG1:C3_06150W_A:DOT1:ISW2:C3_06370C_A:NSA2:PRP5:NOP13:C3_07550C_A:C3_07570C_A:RAD4:UTP9:C3_07800C_A:DUR4:RLP24:NMD5:C4_00690C_A:C4_00740W_A:C4_00800W_A:C4_00810C_A:C4_00880W_A:RAT1:C4_01280C_A:PWP1:C4_01500W_A:SLX4:HIT1:SAS10:RPC53:HCA4:ZUO1:C4_02880C_A:NAN1:C4_03140C_A:C4_03170W_A:DAO2:C4_03720C_A:C4_03740W_A:NUP84:C4_03830W_A:RRP42:RAM1:ECM1:DUO1:C4_04520W_A:SSZ1:BAS1:C4_04820C_A:AGP3:RPL30:C4_05010W_A:C4_05230C_A:C4_05260W_A:C4_05360C_A:C4_05650W_A:C4_06210C_A:C4_06410W_A:NOP1:C4_06790W_A:C4_06950W_A:C4_07060W_A:C4_07150W_A:MRP17:RMT2:C5_00920W_A:BUD23:CYC3:PUS4:C5_01430C_A:FYV5:SPB4:C5_01930W_A:C5_02010C_A:C5_02070C_A:RIX7:C5_02440C_A:TIF5:S

						PR28:UTP13:BUR2:CKB2:C5_03010W_A:RMS1:C5_03400C_A:C5_03920C_A:C5_04120C_A:C5_04720C_A:HAS1:C5_04840C_A:C5_04910W_A:GRF10:C5_05340W_A:NOP5:CDC14:TOP1:C6_01040C_A:C6_01120C_A:CIC1:C6_01890C_A:SWD3:C6_02230W_A:RPL10A:ALG11:C6_02290C_A:C6_02430W_A:MRT4:C6_02900C_A:POP4:C6_03210C_A:C6_03380W_A:C6_03390W_A:C6_03440W_A:NOG2:SPB1:C6_04240W_A:NOP8:C7_00160C_A:C7_00220W_A:C7_00330C_A:THG1:RPA135:NOP15:C7_01030C_A:DBP7:C7_01210C_A:C7_01270C_A:C7_01360C_A:GIR2:FLU1:C7_01570C_A:C7_01950W_A:LIG1:C7_02460C_A:C7_02660C_A:C7_02930C_A:C7_03400C_A:ISY1:ENP2:C7_03590C_A:ENP1:C7_03850W_A:C7_03880C_A:C7_04150W_A:UTP18:CR_00430C_A:CR_00460C_A:BUD22:PWP2:CR_00830W_A:DAL81:CR_01320C_A:CR_01410C_A:CR_01550C_A:CR_01600C_A:CR_01670W_A:CR_01700C_A:CR_01710W_A:CR_01780W_A:CR_02030C_A:MCM6:CR_02420W_A:CR_02550C_A:RRP9:CR_02890C_A:SRP40:SMC5:CR_03200C_A:CR_03230W_A:CR_03360W_A:KTI12:NCS2:YVH1:SGD1:SMC1:PRP42:CR_03940W_A:MEX67:CR_04110W_A:CR_04170W_A:CR_04240C_A:CR_04500C_A:CR_04710W_A:NOC2:CR_05550C_A:RNR3:MTG2:DBP8:SDA1:RFX1:RPL7:CR_06230W_A:FGR50:CR_06450W_A:RRN11:CR_06680C_A:NMD3:CR_06840W_A:CR_06970C_A:POL2:CR_07030C_A:CR_07310W_A:PIF1:FUN30:CR_07640C_A:DBP6:IMP4:CR_08000C_A:NOP10:SSB1:RIO2:ATS1:CR_08330W_A:CR_08410W_A:TSR1:CR_08500W_A:MPS1:ELF1:SSF1:CR_09740W_A:MED21:CR_09800C_A:DPB2:SIK1:UTP5:CR_10260W_A:CR_10410C_A:CR_10470C_A:DRS1:LTV1:POP3

22613	ribonucleoprotein complex biogenesis	453 out of 1061 genes, 42.7%	915 out of 6473 background genes, 14.1%	3.24E-144	0.00%	HBR1:CNS1:RPO41:NRM1:RRS1:TRI1:C1_01160C_A:C1_02090C_A:ABP140:C1_02450C_A:PDE2:C1_02900C_A:TSR2:ARX1:C1_03630W_A:RPP1:C1_03830C_A:C1_04040C_A:C1_04120C_A:ERB1:NOP4:C1_04510W_A:C1_04710C_A:C1_04970W_A:RRP6:REI1:TRY2:C1_05220C_A:C1_05360C_A:C1_05380C_A:RSM22:RPS27A:FAL1:C1_05650W_A:C1_06530C_A:C1_06540C_A:NOP6:C1_06760C_A:RIA1:C1_07510W_A:C1_07660W_A:ERD1:C1_07950C_A:C1_07960W_A:FUN12:MLT1:DIP2:GCR3:GCD6:C1_08630W_A:MSS116:JIP5:C1_09040C_A:CEF1:C1_09390W_A:YTM1:C1_09710C_A:C1_09790C_A:PDC2:DBP3:MNN23:FCY23:C1_10620W_A:RPA190:C1_10920W_A:C1_10950C_A:C1_10970W_A:C1_11000C_A:NEP1:KRR1:GAR1:MUP1:C1_11900C_A:C1_11910W_A:TRP3:C1_12280C_A:CSI2:C1_12350W_A:C1_12440W_A:C1_12570C_A:C1_12630C_A:C1_12680W_A:C1_12750C_A:C1_12760W_A:C1_12820C_A:REX3:RMP1:C1_13370W_A:C1_13380W_A:MCU1:C1_13820C_A:C1_14080W_A:C1_14330W_A:MPP10:FAV3:C2_00200W_A:C2_00280C_A:C2_00410C_A:C2_00820W_A:C2_00840W_A:C2_01070W_A:C2_01220W_A:C2_01420C_A:REX2:C2_01820C_A:PRP39:C2_01860C_A:C2_02120W_A:C2_02420C_A:UTP21:UTP22:PRS1:C2_02540W_A:C2_02630W_A:PDR17:MET1:PRP3:SCH9:C2_04120C_A:TBF1:SUV3:RIM2:C2_04570W_A:SNU114:C2_04700C_A:C2_04820W_A:PZF1:CKA2:C2_05050C_A:C2_05080C_A:MAK5:C2_05160C_A:RPF2:C2_05670C_A:C2_05750W_A:C2_05830C_A:C2_05840W_A:LAS1:ECM17:MAK16:C2_06520C_A:C2_06530W_A:C2_06650C_A:C2_06660W_A:RPL11:FHL1:C2_06850W_A:NOC4:C2_07360W_A:RCL1:PRS5:C2_07920W_A:NSA1:KRE30:RPS10:C2_08180C_A:EAF3:RRP8:C2_08530C_A:MSU1:UTP15:MFG1:C2_09160W_A:C2_09180W_A:MED18:C2_09310C_A:PES1:TAZ1:RRP15:C2_09660W_A:SMM1:C2_09920W_A:CWC22:PUS7:C2_10740C_A:C2_10810W_A:RPS24:C3_00100W_A:UTP8:BUD21:SOF1:C3_00660W_A:C3_00830C_A:BMS1:ARP9:UTP20:NCE103:C3_01310W_A:RPS7A:C3_01560W_A:C3_02020W_A:C3_02040C_A:UTP4:ILV2:C3_02350W_A:C3_02670W_A:SMC3:C3_02840W_A:C3_02850C_A:C3_03070W_A:C3_03110W_A:C3_03330C_A:HBR3:C3_04370C_A:C3_04380C_A:MAK21:RPS15:ARV1:SFP1:NOP14:CEM1:C3_05160C_A:RPL8B:C3_05280C_A:C3_05380W_A:C3_05440C_A:QDR2:C3_05800W_A:C3_05860C_A:C3_05900W_A:NOG1:C3_06150W_A:DOT1:ISW2:C3_06370C_A:NSA2:PRP5:NOP13:C3_07550C_A:C3_07570C_A:RAD4:UTP9:C3_07800C_A:DUR4:RLP24:NMD5:C4_00690C_A:C4_00740W_A:C4_00800W_A:C4_00810C_A:C4_00880W_A:RAT1:C4_01280C_A:PWP1:C4_01500W_A:SLX4:HIT1:SAS10:RPC53:HCA4:ZUO1:C4_02880C_A:NAN1:C4_03140C_A:C4_03170W_A:DAO2:C4_03720C_A:C4_03740W_A:NUP84:C4_03830W_A:RRP42:RAM1:ECM1:DUO1:C4_04520W_A:SSZ1:BAS1:C4_04820C_A:AGP3:RPL30:C4_05010W_A:C4_05230C_A:C4_05260W_A:C4_05360C_A:C4_05650W_A:C4_06210C_A:C4_06410W_A:NOP1:C4_06790W_A:C4_06950W_A:C4_07060W_A:C4_07150W_A:MRP17:RMT2:C5_00920W_A:BUD23:CYC3:PUS4:C5_01430C_A:FYV5:SPB4:C5_01930W_A:C5_02010C_A:C5_02070C_A:RIX7:C5_0

						2440C_A:TIF5:SPR28:UTP13:BUR2:CKB2:C5_03010W_A:RMS1:C5_03400C_A:C5_03920C_A:C5_04120C_A:C5_04720C_A:HAS1:C5_04840C_A:C5_04910W_A:GRF10:C5_05340W_A:NOP5:CDC14:TOP1:C6_01040C_A:C6_01120C_A:CIC1:TIF3:C6_01890C_A:SWD3:C6_02230W_A:RPL10A:ALG11:C6_02290C_A:C6_02430W_A:PRP45:MRT4:C6_02900C_A:POP4:C6_03210C_A:C6_03380W_A:C6_03390W_A:C6_03440W_A:NOG2:SPB1:C6_04240W_A:NOP8:C7_00160C_A:C7_00220W_A:C7_00330C_A:THG1:RPA135:NOP15:C7_01030C_A:DBP7:C7_01210C_A:C7_01270C_A:C7_01360C_A:GIR2:FLU1:C7_01570C_A:C7_01950W_A:LIG1:C7_02460C_A:C7_02660C_A:C7_02930C_A:C7_03400C_A:ISY1:ENP2:C7_03590C_A:ENP1:C7_03850W_A:C7_03880C_A:C7_04150W_A:UTP18:CR_00430C_A:CR_00460C_A:BUD22:PWP2:CR_00830W_A:DAL81:CR_01320C_A:CR_01410C_A:CR_01550C_A:CR_01600C_A:CR_01670W_A:CR_01700C_A:CR_01710W_A:CR_01780W_A:CR_02030C_A:MCM6:CR_02420W_A:CR_02550C_A:RRP9:CR_02890C_A:SRP40:ARC1:SMC5:CR_03200C_A:CR_03230W_A:CR_03360W_A:KTI12:NCS2:YVH1:SGD1:SMC1:PRP42:CR_03940W_A:MEX67:CR_04110W_A:CR_04170W_A:CR_04240C_A:CR_04500C_A:CR_04710W_A:NOC2:CR_05550C_A:RNR3:MTG2:DBP8:SDA1:RFX1:RPL7:CR_06230W_A:FGR50:CR_06450W_A:RRN11:CR_06680C_A:NMD3:CR_06840W_A:CR_06970C_A:POL2:CR_07030C_A:CR_07310W_A:PIF1:FUN30:CR_07640C_A:DBP6:IMP4:CR_08000C_A:NOP10:SSB1:RIO2:ATS1:CR_08330W_A:CR_08410W_A:TSR1:CR_08500W_A:MPS1:ELF1:SSF1:CR_09740W_A:MED21:CR_09800C_A:DPB2:SIK1:UTP5:CR_10260W_A:CR_10410C_A:CR_10470C_A:DRS1:LTV1:POP3

6396	RNA processing	456 out of 1061 genes, 43.0%	931 out of 6473 background genes, 14.4%	5.28E-143	0.00%	HBR1:CNS1:RPO41:NRM1:RRS1:TRI1:C1_01150C_A:C1_01160C_A:TRM2:C1_02090C_A:ABP140:C1_02450C_A:PDE2:C1_02900C_A:TSR2:C1_03370W_A:C1_03630W_A:RPP1:C1_03830C_A:C1_04040C_A:C1_04120C_A:ERB1:NOP4:C1_04510W_A:C1_04530C_A:C1_04710C_A:C1_04970W_A:RRP6:REI1:TRY2:C1_05220C_A:C1_05360C_A:C1_05380C_A:RSM22:RPS27A:FAL1:C1_05650W_A:C1_06540C_A:NOP6:C1_06760C_A:RIA1:LHP1:C1_07510W_A:C1_07660W_A:MRPL3:ERD1:C1_07950C_A:C1_07960W_A:FUN12:MLT1:DIP2:GCR3:GCD6:C1_08630W_A:MSS116:C1_08890C_A:CEF1:C1_09390W_A:C1_09710C_A:C1_09790C_A:BUD31:PDC2:DBP3:MNN23:C1_10080W_A:FCY23:C1_10620W_A:C1_10950C_A:C1_10970W_A:C1_11000C_A:PET127:NEP1:KRR1:MUP1:C1_11900C_A:C1_11910W_A:TRP3:C1_12280C_A:CSI2:C1_12350W_A:C1_12440W_A:C1_12570C_A:C1_12630C_A:C1_12670C_A:C1_12750C_A:C1_12760W_A:C1_12820C_A:REX3:RMP1:C1_13380W_A:MCU1:C1_13820C_A:C1_14080W_A:C1_14330W_A:MPP10:FAV3:C2_00200W_A:C2_00280C_A:C2_00410C_A:C2_00820W_A:C2_00840W_A:C2_01070W_A:C2_01220W_A:C2_01420C_A:REX2:C2_01820C_A:PRP39:C2_01860C_A:C2_02120W_A:C2_02420C_A:UTP21:UTP22:PRS1:C2_02540W_A:C2_02630W_A:PDR17:MET1:PRP3:SCH9:C2_04120C_A:TBF1:SUV3:RIM2:C2_04570W_A:SNU114:C2_04700C_A:C2_04820W_A:PZF1:CKA2:C2_05050C_A:C2_05080C_A:MAK5:C2_05160C_A:RPF2:KTI11:C2_05670C_A:C2_05750W_A:C2_05830C_A:C2_05840W_A:LAS1:ECM17:MAK16:C2_06480W_A:C2_06520C_A:C2_06530W_A:C2_06650C_A:C2_06660W_A:FHL1:C2_06850W_A:NOC4:C2_07360W_A:RCL1:PRS5:C2_07920W_A:NSA1:C2_08180C_A:EAF3:RRP8:C2_08530C_A:MSU1:UTP15:C2_09160W_A:C2_09180W_A:C2_09310C_A:PES1:TAZ1:RRP15:C2_09500W_A:C2_09660W_A:SMM1:C2_09920W_A:CWC22:UBP8:PUS7:C2_10740C_A:C2_10810W_A:RPS24:C3_00100W_A:UTP8:BUD21:SOF1:C3_00660W_A:C3_00830C_A:BMS1:ARP9:UTP20:NCE103:C3_01310W_A:C3_01560W_A:C3_02020W_A:C3_02040C_A:UTP4:ILV2:C3_02350W_A:C3_02670W_A:C3_02750W_A:C3_02840W_A:C3_02850C_A:C3_03070W_A:C3_03110W_A:C3_03330C_A:HBR3:C3_04370C_A:C3_04380C_A:MAK21:ARV1:SFP1:NOP14:CEM1:C3_05140C_A:C3_05160C_A:RPL8B:C3_05280C_A:C3_05380W_A:C3_05440C_A:QDR2:C3_05800W_A:C3_05860C_A:TRM12:C3_05900W_A:NOG1:C3_06150W_A:DOT1:ISW2:C3_06370C_A:NSA2:PRP5:NOP13:C3_07400W_A:C3_07550C_A:C3_07570C_A:RIT1:RAD4:UTP9:C3_07800C_A:DUR4:RLP24:NMD5:C4_00690C_A:C4_00740W_A:C4_00800W_A:C4_00810C_A:C4_00880W_A:C4_00940W_A:RAT1:C4_01280C_A:PWP1:C4_01500W_A:SLX4:HIT1:SAS10:RPC53:HCA4:C4_02850W_A:ZUO1:C4_02880C_A:NAN1:C4_03140C_A:C4_03170W_A:DAO2:C4_03720C_A:C4_03730C_A:C4_03740W_A:NUP84:C4_03830W_A:RRP42:RAM1:ECM1:DUO1:C4_04520W_A:SSZ1:BAS1:C4_04810C_A:AGP3:RPL30:C4_05230C_A:C4_05260W_A:C4_05360C_A:C4_05650W_A:C4_06210C_A:C4_06410W_A:NOP1:C4_06790W_A:C4_06950W_A:C4_07060W_A:C4_07150W_A:DUS4:MRP17:RMT2:C5_00920

						W_A:BUD23:CYC3:PUS4:C5_01430C_A:FYV5:SPB4:C5_01610W_A:C5_01930W_A:C5_02010C_A:C5_02070C_A:RIX7:C5_02440C_A:SPR28:UTP13:CKB2:C5_03010W_A:RMS1:C5_03400C_A:C5_03920C_A:C5_04120C_A:HAS1:C5_04840C_A:C5_04910W_A:GRF10:C5_05340W_A:NOP5:C6_00530C_A:CDC14:TOP1:C6_01040C_A:C6_01120C_A:CIC1:C6_01890C_A:SWD3:C6_02230W_A:ALG11:C6_02290C_A:C6_02350C_A:C6_02430W_A:PRP45:C6_02690C_A:MRT4:C6_02900C_A:POP4:C6_03210C_A:C6_03380W_A:C6_03390W_A:C6_03440W_A:NAM2:SPB1:C6_04240W_A:NOP8:C6_04530C_A:C7_00160C_A:C7_00220W_A:C7_00330C_A:THG1:NOP15:C7_01030C_A:DBP7:C7_01210C_A:C7_01270C_A:C7_01360C_A:GIR2:FLU1:C7_01570C_A:C7_01950W_A:LIG1:C7_02340C_A:C7_02460C_A:C7_02660C_A:C7_02930C_A:C7_03400C_A:ISY1:ENP2:C7_03590C_A:ENP1:C7_03850W_A:C7_03880C_A:C7_04150W_A:UTP18:CR_00430C_A:CR_00460C_A:BUD22:PWP2:CR_00830W_A:TRM1:DAL81:CR_01320C_A:CR_01410C_A:CR_01550C_A:CR_01600C_A:CR_01670W_A:CR_01700C_A:CR_01710W_A:CR_01780W_A:CR_02030C_A:MCM6:CR_02420W_A:CR_02550C_A:RRP9:CR_02890C_A:SRP40:SMC5:CR_03200C_A:CR_03230W_A:CR_03360W_A:KTI12:NCS2:YVH1:SMC1:PRP42:CR_03940W_A:CR_04110W_A:CR_04160C_A:CR_04170W_A:CR_04240C_A:CR_04300W_A:CR_04500C_A:CR_04710W_A:NOC2:CR_05550C_A:RNR3:MTG2:DBP8:RFX1:RPL7:CR_06230W_A:FGR50:CR_06450W_A:RRN11:CR_06680C_A:NMD3:CR_06840W_A:CR_06970C_A:POL2:CR_07030C_A:CR_07310W_A:PIF1:FUN30:CR_07640C_A:DBP6:IMP4:CR_08000C_A:NOP10:SSB1:RIO2:ATS1:CR_08330W_A:CR_08410W_A:TSR1:CR_08940W_A:MPS1:SSF1:CR_09740W_A:MED21:CR_09800C_A:DPB2:SIK1:UTP5:CR_10260W_A:CR_10410C_A:CR_10470C_A:DRS1:LTV1:POP3

6364	rRNA processing	417 out of 1061 genes, 39.3%	806 out of 6473 background genes, 12.5%	3.66E-139	0.00%	HBR1:CNS1:RPO41:NRM1:RRS1:TRI1:C1_01160C_A:C1_02090C_A:ABP140:C1_02450C_A:PDE2:C1_02900C_A:TSR2:C1_03630W_A:RPP1:C1_03830C_A:C1_04040C_A:C1_04120C_A:ERB1:NOP4:C1_04510W_A:C1_04710C_A:C1_04970W_A:RRP6:REI1:TRY2:C1_05220C_A:C1_05360C_A:C1_05380C_A:RSM22:RPS27A:FAL1:C1_05650W_A:C1_06540C_A:NOP6:C1_06760C_A:RIA1:C1_07510W_A:C1_07660W_A:ERD1:C1_07950C_A:C1_07960W_A:FUN12:MLT1:DIP2:GCR3:GCD6:C1_08630W_A:MSS116:CEF1:C1_09390W_A:C1_09710C_A:C1_09790C_A:PDC2:DBP3:MNN23:FCY23:C1_10620W_A:C1_10950C_A:C1_10970W_A:C1_11000C_A:NEP1:KRR1:MUP1:C1_11900C_A:C1_11910W_A:TRP3:C1_12280C_A:CSI2:C1_12350W_A:C1_12440W_A:C1_12570C_A:C1_12630C_A:C1_12750C_A:C1_12760W_A:C1_12820C_A:REX3:RMP1:C1_13380W_A:MCU1:C1_13820C_A:C1_14080W_A:C1_14330W_A:MPP10:FAV3:C2_00200W_A:C2_00280C_A:C2_00410C_A:C2_00820W_A:C2_00840W_A:C2_01070W_A:C2_01220W_A:C2_01420C_A:REX2:C2_01820C_A:C2_01860C_A:C2_02120W_A:C2_02420C_A:UTP21:UTP22:PRS1:C2_02540W_A:C2_02630W_A:PDR17:MET1:PRP3:SCH9:C2_04120C_A:TBF1:SUV3:RIM2:C2_04570W_A:SNU114:C2_04700C_A:C2_04820W_A:PZF1:CKA2:C2_05050C_A:C2_05080C_A:MAK5:C2_05160C_A:RPF2:C2_05670C_A:C2_05750W_A:C2_05830C_A:C2_05840W_A:LAS1:ECM17:MAK16:C2_06520C_A:C2_06530W_A:C2_06660W_A:FHL1:C2_06850W_A:NOC4:C2_07360W_A:RCL1:PRS5:C2_07920W_A:NSA1:C2_08180C_A:EAF3:RRP8:C2_08530C_A:MSU1:UTP15:C2_09160W_A:C2_09180W_A:C2_09310C_A:PES1:TAZ1:RRP15:C2_09660W_A:SMM1:C2_09920W_A:CWC22:PUS7:C2_10740C_A:C2_10810W_A:RPS24:C3_00100W_A:UTP8:BUD21:SOF1:C3_00660W_A:C3_00830C_A:BMS1:ARP9:UTP20:NCE103:C3_01310W_A:C3_01560W_A:C3_02020W_A:C3_02040C_A:UTP4:ILV2:C3_02350W_A:C3_02670W_A:C3_02840W_A:C3_02850C_A:C3_03070W_A:C3_03110W_A:C3_03330C_A:HBR3:C3_04370C_A:C3_04380C_A:MAK21:ARV1:SFP1:NOP14:CEM1:C3_05160C_A:RPL8B:C3_05280C_A:C3_05380W_A:C3_05440C_A:QDR2:C3_05800W_A:C3_05860C_A:C3_05900W_A:NOG1:C3_06150W_A:DOT1:ISW2:C3_06370C_A:NSA2:PRP5:NOP13:C3_07550C_A:C3_07570C_A:RAD4:UTP9:C3_07800C_A:DUR4:RLP24:NMD5:C4_00690C_A:C4_00740W_A:C4_00800W_A:C4_00810C_A:C4_00880W_A:RAT1:C4_01280C_A:PWP1:C4_01500W_A:SLX4:HIT1:SAS10:RPC53:HCA4:ZUO1:C4_02880C_A:NAN1:C4_03140C_A:C4_03170W_A:DAO2:C4_03720C_A:C4_03740W_A:NUP84:C4_03830W_A:RRP42:RAM1:ECM1:DUO1:C4_04520W_A:SSZ1:BAS1:AGP3:RPL30:C4_05230C_A:C4_05260W_A:C4_05360C_A:C4_05650W_A:C4_06210C_A:C4_06410W_A:NOP1:C4_06790W_A:C4_06950W_A:C4_07060W_A:C4_07150W_A:MRP17:RMT2:C5_00920W_A:BUD23:CYC3:PUS4:C5_01430C_A:FYV5:SPB4:C5_01930W_A:C5_02010C_A:C5_02070C_A:RIX7:C5_02440C_A:SPR28:UTP13:CKB2:C5_03010W_A:RMS1:C5_03400C_A:C5_03920C_A:C5_04120C_A:HAS1:C5_04840C_A:C5_04910W_A:GRF10:C5_05340W_A:NOP5:CDC14:TOP1:C6_01040C_A:C6_01120C_A:CIC1:C6_

						01890C_A:SWD3:C6_02230W_A:ALG11:C6_02290C_A:C6_02430W_A:MRT4:C6_02900C_A:POP4:C6_03210C_A:C6_03380W_A:C6_03390W_A:C6_03440W_A:SPB1:C6_04240W_A:NOP8:C7_00160C_A:C7_00220W_A:C7_00330C_A:THG1:NOP15:C7_01030C_A:DBP7:C7_01210C_A:C7_01270C_A:C7_01360C_A:GIR2:FLU1:C7_01570C_A:C7_01950W_A:LIG1:C7_02460C_A:C7_02660C_A:C7_02930C_A:C7_03400C_A:ISY1:ENP2:C7_03590C_A:ENP1:C7_03850W_A:C7_03880C_A:C7_04150W_A:UTP18:CR_00430C_A:CR_00460C_A:BUD22:PWP2:CR_00830W_A:DAL81:CR_01320C_A:CR_01410C_A:CR_01550C_A:CR_01600C_A:CR_01670W_A:CR_01700C_A:CR_01710W_A:CR_01780W_A:CR_02030C_A:MCM6:CR_02420W_A:CR_02550C_A:RRP9:CR_02890C_A:SRP40:SMC5:CR_03200C_A:CR_03230W_A:CR_03360W_A:KTI12:NCS2:YVH1:SMC1:PRP42:CR_03940W_A:CR_04110W_A:CR_04170W_A:CR_04240C_A:CR_04500C_A:CR_04710W_A:NOC2:CR_05550C_A:RNR3:MTG2:DBP8:RFX1:RPL7:CR_06230W_A:FGR50:CR_06450W_A:RRN11:CR_06680C_A:NMD3:CR_06840W_A:CR_06970C_A:POL2:CR_07030C_A:CR_07310W_A:PIF1:FUN30:CR_07640C_A:DBP6:IMP4:CR_08000C_A:NOP10:SSB1:RIO2:ATS1:CR_08330W_A:CR_08410W_A:TSR1:MPS1:SSF1:CR_09740W_A:MED21:CR_09800C_A:DPB2:SIK1:UTP5:CR_10260W_A:CR_10410C_A:CR_10470C_A:DRS1:LTV1:POP3

16072	rRNA metabolic process	422 out of 1061 genes, 39.8%	835 out of 6473 background genes, 12.9%	3.15E-136	0.00%	HBR1:CNS1:RPO41:NRM1:RRS1:TRI1:C1_01160C_A:C1_02090C_A:ABP140:C1_02450C_A:PDE2:C1_02900C_A:TSR2:C1_03630W_A:RPP1:C1_03830C_A:C1_04040C_A:C1_04120C_A:ERB1:NOP4:C1_04510W_A:C1_04710C_A:C1_04970W_A:RRP6:REI1:TRY2:C1_05220C_A:C1_05360C_A:C1_05380C_A:RSM22:RPS27A:FAL1:C1_05650W_A:C1_06540C_A:NOP6:C1_06760C_A:RIA1:C1_07510W_A:C1_07660W_A:ERD1:C1_07950C_A:C1_07960W_A:FUN12:MLT1:DIP2:GCR3:GCD6:C1_08630W_A:MSS116:CEF1:C1_09390W_A:C1_09710C_A:C1_09790C_A:PDC2:DBP3:MNN23:FCY23:C1_10620W_A:RPA190:C1_10920W_A:C1_10950C_A:C1_10970W_A:C1_11000C_A:NEP1:KRR1:MUP1:C1_11900C_A:C1_11910W_A:TRP3:C1_12280C_A:CSI2:C1_12350W_A:C1_12440W_A:C1_12570C_A:C1_12630C_A:C1_12750C_A:C1_12760W_A:C1_12820C_A:REX3:RMP1:C1_13380W_A:MCU1:C1_13820C_A:C1_14080W_A:C1_14330W_A:MPP10:FAV3:C2_00200W_A:C2_00280C_A:C2_00410C_A:C2_00820W_A:C2_00840W_A:C2_01070W_A:C2_01220W_A:C2_01420C_A:REX2:C2_01820C_A:C2_01860C_A:C2_02120W_A:C2_02420C_A:UTP21:UTP22:PRS1:C2_02540W_A:C2_02630W_A:PDR17:MET1:PRP3:SCH9:C2_04120C_A:TBF1:SUV3:RIM2:C2_04570W_A:SNU114:C2_04700C_A:C2_04820W_A:PZF1:CKA2:C2_05050C_A:C2_05080C_A:MAK5:C2_05160C_A:RPF2:C2_05670C_A:C2_05750W_A:C2_05830C_A:C2_05840W_A:LAS1:ECM17:MAK16:C2_06520C_A:C2_06530W_A:C2_06660W_A:FHL1:C2_06850W_A:NOC4:C2_07360W_A:RCL1:PRS5:C2_07920W_A:NSA1:C2_08180C_A:EAF3:RRP8:C2_08530C_A:MSU1:UTP15:C2_09160W_A:C2_09180W_A:C2_09310C_A:PES1:TAZ1:RRP15:C2_09660W_A:SMM1:C2_09920W_A:CWC22:PUS7:C2_10740C_A:C2_10810W_A:RPS24:C3_00100W_A:UTP8:BUD21:SOF1:C3_00660W_A:C3_00830C_A:BMS1:ARP9:UTP20:NCE103:C3_01310W_A:C3_01560W_A:C3_02020W_A:C3_02040C_A:UTP4:ILV2:C3_02350W_A:C3_02670W_A:C3_02840W_A:C3_02850C_A:C3_03070W_A:C3_03110W_A:C3_03330C_A:HBR3:C3_04370C_A:C3_04380C_A:MAK21:ARV1:SFP1:NOP14:CEM1:C3_05160C_A:RPL8B:C3_05280C_A:C3_05380W_A:C3_05440C_A:QDR2:C3_05800W_A:C3_05860C_A:C3_05900W_A:NOG1:C3_06150W_A:DOT1:ISW2:C3_06370C_A:NSA2:PRP5:NOP13:C3_07550C_A:C3_07570C_A:RAD4:UTP9:C3_07800C_A:DUR4:RLP24:NMD5:C4_00690C_A:C4_00740W_A:C4_00800W_A:C4_00810C_A:C4_00880W_A:RAT1:C4_01280C_A:PWP1:C4_01500W_A:SLX4:HIT1:SAS10:RPC53:HCA4:ZUO1:C4_02880C_A:NAN1:C4_03140C_A:C4_03170W_A:DAO2:C4_03720C_A:C4_03740W_A:NUP84:C4_03830W_A:RRP42:RAM1:ECM1:DUO1:C4_04520W_A:SSZ1:BAS1:AGP3:RPL30:C4_05230C_A:C4_05260W_A:C4_05360C_A:C4_05650W_A:C4_06210C_A:C4_06410W_A:NOP1:C4_06790W_A:C4_06950W_A:C4_07060W_A:C4_07150W_A:MRP17:RMT2:C5_00920W_A:BUD23:CYC3:PUS4:C5_01430C_A:FYV5:SPB4:C5_01930W_A:C5_02010C_A:C5_02070C_A:RIX7:C5_02440C_A:SPR28:UTP13:CKB2:C5_03010W_A:RMS1:C5_03400C_A:C5_03920C_A:C5_04120C_A:HAS1:C5_04840C_A:C5_04910W_A:GRF10:C5_05340W_A:NOP5:CDC14:TOP1:C6_01040C

						_A:C6_01120C_A:CIC1:C6_01890C_A:SWD3:C6_02230W_A:ALG11:C6_02290C_A:C6_02430W_A:MRT4:C6_02900C_A:POP4:C6_03210C_A:C6_03380W_A:C6_03390W_A:C6_03440W_A:SPB1:C6_04240W_A:NOP8:C7_00160C_A:C7_00220W_A:C7_00330C_A:THG1:RPA135:NOP15:C7_01030C_A:DBP7:C7_01210C_A:C7_01270C_A:C7_01360C_A:C7_01400C_A:GIR2:FLU1:C7_01570C_A:C7_01950W_A:LIG1:C7_02460C_A:C7_02660C_A:C7_02930C_A:C7_03400C_A:ISY1:ENP2:C7_03590C_A:ENP1:C7_03850W_A:C7_03880C_A:C7_04150W_A:UTP18:CR_00430C_A:CR_00460C_A:BUD22:PWP2:CR_00830W_A:DAL81:CR_01320C_A:CR_01410C_A:CR_01550C_A:CR_01600C_A:CR_01670W_A:CR_01700C_A:CR_01710W_A:CR_01780W_A:CR_01950W_A:CR_02030C_A:MCM6:CR_02420W_A:CR_02550C_A:RRP9:CR_02890C_A:SRP40:SMC5:CR_03200C_A:CR_03230W_A:CR_03360W_A:KTI12:NCS2:YVH1:SMC1:PRP42:CR_03940W_A:CR_04110W_A:CR_04170W_A:CR_04240C_A:CR_04500C_A:CR_04710W_A:NOC2:CR_05550C_A:RNR3:MTG2:DBP8:RFX1:RPL7:CR_06230W_A:FGR50:CR_06450W_A:RRN11:CR_06680C_A:NMD3:CR_06840W_A:CR_06970C_A:POL2:CR_07030C_A:CR_07310W_A:PIF1:FUN30:CR_07640C_A:DBP6:IMP4:CR_08000C_A:NOP10:SSB1:RIO2:ATS1:CR_08330W_A:CR_08410W_A:TSR1:MPS1:SSF1:CR_09740W_A:MED21:CR_09800C_A:DPB2:SIK1:UTP5:CR_10260W_A:CR_10410C_A:CR_10470C_A:DRS1:LTV1:POP3

16070	RNA metabolic process	498 out of 1061 genes, 46.9%	1245 out of 6473 background genes, 19.2%	2.24E-113	0.00%	HBR1:CNS1:RPO41:NRM1:RRS1:TRI1:C1_01150C_A:C1_01160C_A:TRM2:C1_01530C_A:C1_02090C_A:ABP140:C1_02450C_A:PDE2:C1_02900C_A:TSR2:C1_03370W_A:C1_03630W_A:RPP1:C1_03830C_A:C1_04040C_A:C1_04120C_A:ERB1:NOP4:C1_04510W_A:C1_04530C_A:C1_04710C_A:C1_04970W_A:RRP6:REI1:TRY2:C1_05220C_A:C1_05360C_A:C1_05380C_A:RSM22:RPS27A:FAL1:C1_05650W_A:C1_06540C_A:NOP6:C1_06760C_A:RIA1:C1_07340W_A:LHP1:C1_07510W_A:C1_07660W_A:MRPL3:ERD1:C1_07950C_A:C1_07960W_A:FUN12:CAT8:MLT1:DIP2:GCR3:GCD6:C1_08630W_A:MSS116:C1_08890C_A:CEF1:C1_09390W_A:C1_09710C_A:C1_09790C_A:BUD31:PDC2:DBP3:MNN23:C1_10080W_A:FCY23:C1_10620W_A:RPA190:C1_10920W_A:C1_10950C_A:C1_10970W_A:C1_11000C_A:PET127:NEP1:KRR1:GAR1:MUP1:C1_11900C_A:C1_11910W_A:TRP3:C1_12280C_A:CSI2:C1_12350W_A:WRS1:C1_12440W_A:C1_12570C_A:C1_12630C_A:C1_12670C_A:C1_12750C_A:C1_12760W_A:C1_12820C_A:REX3:RMP1:C1_13380W_A:MCU1:C1_13820C_A:C1_14080W_A:C1_14330W_A:MPP10:FAV3:C2_00200W_A:C2_00280C_A:C2_00410C_A:C2_00820W_A:C2_00840W_A:C2_01070W_A:C2_01220W_A:C2_01420C_A:REX2:C2_01820C_A:PRP39:C2_01860C_A:C2_01870C_A:C2_02120W_A:C2_02420C_A:UTP21:UTP22:PRS1:C2_02540W_A:C2_02630W_A:PDR17:MET1:PRP3:SCH9:C2_04120C_A:TBF1:SUV3:RIM2:C2_04570W_A:SNU114:C2_04700C_A:C2_04820W_A:PZF1:CKA2:C2_05050C_A:C2_05080C_A:MAK5:C2_05160C_A:RPF2:RPC40:KTI11:C2_05670C_A:C2_05750W_A:C2_05830C_A:C2_05840W_A:LAS1:ECM17:MAK16:C2_06480W_A:C2_06520C_A:C2_06530W_A:VAS1:C2_06650C_A:C2_06660W_A:FHL1:C2_06850W_A:NOC4:C2_07360W_A:RCL1:PRS5:C2_07920W_A:NSA1:C2_08180C_A:EAF3:RRP8:C2_08530C_A:MSU1:UTP15:MFG1:C2_09160W_A:C2_09180W_A:MED18:C2_09310C_A:PES1:TAZ1:RRP15:C2_09500W_A:C2_09660W_A:SMM1:C2_09920W_A:CWC22:UBP8:PUS7:C2_10740C_A:RPC11:C2_10810W_A:RPS24:C3_00100W_A:ULP3:UTP8:BUD21:SOF1:C3_00660W_A:C3_00830C_A:BMS1:ARP9:UTP20:NCE103:C3_01310W_A:C3_01560W_A:C3_02020W_A:C3_02040C_A:UTP4:ILV2:C3_02350W_A:C3_02670W_A:C3_02750W_A:C3_02840W_A:C3_02850C_A:C3_03070W_A:C3_03110W_A:C3_03330C_A:HBR3:C3_04370C_A:C3_04380C_A:MAK21:ARV1:SFP1:NOP14:CEM1:C3_05140C_A:C3_05160C_A:RPL8B:C3_05280C_A:C3_05380W_A:C3_05440C_A:QDR2:C3_05800W_A:C3_05860C_A:TRM12:C3_05900W_A:CTA4:NOG1:C3_06150W_A:DOT1:ISW2:C3_06370C_A:NSA2:PRP5:NOP13:C3_07400W_A:C3_07550C_A:C3_07570C_A:RIT1:RAD4:UTP9:C3_07800C_A:DUR4:RLP24:NMD5:C4_00690C_A:C4_00740W_A:C4_00800W_A:C4_00810C_A:C4_00880W_A:C4_00940W_A:RAT1:C4_01280C_A:PWP1:C4_01500W_A:TOA2:ZCF25:C4_02400C_A:SLX4:HIT1:SAS10:RPC53:HCA4:C4_02850W_A:ZUO1:C4_02880C_A:NAN1:C4_03140C_A:C4_03170W_A:DAO2:RAD2:C4_03720C_A:C4_03730C_A:C4_03740W_A:NUP84:C4_03830W_A:RRP42:RAM1:ECM1:DUO1:C4_04520W_A:SSZ1:BAS1:C4_04810C_A:AGP3:RPL30:C4_05230C_A:C4_

						05260W_A:C4_05360C_A:OFD1:C4_05650W_A:C4_06210C_A:C4_06410W_A:NOP1:C4_06790W_A:C4_06950W_A:C4_07060W_A:C4_07150W_A:C5_00280C_A:DUS4:MRP17:RMT2:C5_00920W_A:BUD23:CYC3:PUS4:C5_01430C_A:FYV5:SPB4:C5_01610W_A:C5_01930W_A:C5_02010C_A:C5_02070C_A:RIX7:C5_02440C_A:TIF5:SPR28:UTP13:BUR2:CKB2:MSW1:C5_03010W_A:RMS1:RPO26:C5_03400C_A:C5_03920C_A:C5_04120C_A:MSM1:HYS2:HAS1:C5_04840C_A:C5_04910W_A:GRF10:C5_05340W_A:NOP5:C6_00530C_A:C6_00640C_A:CDC14:TOP1:C6_01040C_A:C6_01120C_A:CIC1:C6_01890C_A:SWD3:C6_02230W_A:ALG11:C6_02290C_A:C6_02300C_A:C6_02350C_A:C6_02430W_A:PRP45:C6_02690C_A:MRT4:C6_02900C_A:POP4:C6_03210C_A:C6_03380W_A:C6_03390W_A:C6_03440W_A:NAM2:SPB1:C6_04240W_A:NOP8:ILS1:C6_04530C_A:C7_00160C_A:C7_00220W_A:C7_00330C_A:THG1:RPA135:NOP15:C7_01030C_A:DBP7:C7_01210C_A:C7_01270C_A:C7_01360C_A:C7_01400C_A:GIR2:FLU1:C7_01570C_A:C7_01950W_A:LIG1:C7_02340C_A:C7_02460C_A:C7_02660C_A:C7_02930C_A:C7_03400C_A:ISY1:ENP2:C7_03590C_A:ENP1:C7_03850W_A:C7_03880C_A:C7_04150W_A:UTP18:CR_00430C_A:CR_00460C_A:BUD22:PWP2:CR_00830W_A:TRM1:DAL81:CR_01320C_A:CR_01410C_A:PRS:CR_01550C_A:CR_01600C_A:CR_01670W_A:CDC60:CR_01700C_A:CR_01710W_A:CR_01780W_A:CR_01950W_A:CR_02030C_A:MCM6:CR_02420W_A:CR_02550C_A:RRP9:RPC31:CR_02890C_A:SRP40:ARC1:SMC5:CR_03110W_A:CR_03200C_A:CR_03230W_A:CR_03360W_A:KTI12:CR_03400W_A:NCS2:YVH1:SGD1:SMC1:PRP42:CR_03940W_A:CR_04110W_A:CR_04160C_A:CR_04170W_A:CR_04240C_A:CR_04300W_A:CR_04500C_A:CR_04560C_A:CR_04710W_A:RPB8:NOC2:CR_05550C_A:RNR3:MTG2:DBP8:RFX1:RPL7:CR_06230W_A:FGR50:CR_06450W_A:RRN11:CR_06680C_A:NMD3:CR_06840W_A:CR_06970C_A:POL2:CR_07030C_A:CR_07310W_A:PIF1:FUN30:CR_07640C_A:DBP6:IMP4:CR_08000C_A:NOP10:SSB1:RIO2:ATS1:CR_08330W_A:CR_08410W_A:TSR1:CR_08940W_A:MPS1:SSF1:CR_09740W_A:MED21:CR_09800C_A:DPB2:SIK1:UTP5:CR_10260W_A:CR_10410C_A:CR_10470C_A:DRS1:LTV1:POP3

30490	maturation of SSU-rRNA	336 out of 1061 genes, 31.7%	627 out of 6473 background genes, 9.7%	3.43E-113	0.00%	HBR1:CNS1:RPO41:NRM1:RRS1:C1_01160C_A:C1_02090C_A:ABP140:C1_02450C_A:C1_02900C_A:TSR2:C1_03630W_A:RPP1:C1_03830C_A:C1_04040C_A:C1_04120C_A:NOP4:C1_04510W_A:C1_04710C_A:REI1:TRY2:C1_05220C_A:C1_05360C_A:C1_05380C_A:RSM22:RPS27A:FAL1:C1_05650W_A:C1_06540C_A:NOP6:RIA1:C1_07510W_A:C1_07660W_A:C1_07960W_A:FUN12:MLT1:DIP2:GCD6:C1_08630W_A:MSS116:CEF1:C1_09390W_A:C1_09710C_A:C1_09790C_A:PDC2:MNN23:FCY23:C1_10950C_A:C1_11000C_A:NEP1:KRR1:MUP1:C1_11900C_A:C1_12280C_A:C1_12350W_A:C1_12440W_A:C1_12570C_A:C1_12630C_A:C1_12750C_A:C1_12820C_A:RMP1:C1_13380W_A:C1_13820C_A:C1_14080W_A:C1_14330W_A:MPP10:FAV3:C2_00200W_A:C2_00280C_A:C2_00410C_A:C2_00820W_A:C2_00840W_A:C2_01070W_A:C2_01220W_A:C2_01420C_A:C2_01820C_A:C2_02120W_A:C2_02420C_A:PRS1:C2_02540W_A:C2_02630W_A:PDR17:MET1:PRP3:SCH9:C2_04120C_A:TBF1:SUV3:RIM2:C2_04570W_A:C2_04700C_A:C2_04820W_A:PZF1:CKA2:C2_05050C_A:C2_05080C_A:MAK5:C2_05160C_A:C2_05670C_A:C2_05750W_A:C2_05830C_A:C2_05840W_A:LAS1:MAK16:C2_06520C_A:C2_06660W_A:FHL1:C2_06850W_A:NOC4:C2_07360W_A:RCL1:PRS5:C2_07920W_A:NSA1:C2_08180C_A:RRP8:C2_08530C_A:MSU1:UTP15:C2_09160W_A:C2_09310C_A:PES1:TAZ1:RRP15:C2_09660W_A:SMM1:C2_09920W_A:CWC22:C2_10740C_A:C2_10810W_A:RPS24:C3_00100W_A:UTP8:BUD21:SOF1:C3_00660W_A:BMS1:UTP20:NCE103:C3_01310W_A:C3_01560W_A:C3_02020W_A:C3_02040C_A:UTP4:C3_02350W_A:C3_02670W_A:C3_02840W_A:C3_02850C_A:C3_03070W_A:C3_03110W_A:C3_03330C_A:HBR3:C3_04370C_A:C3_04380C_A:MAK21:SFP1:NOP14:CEM1:C3_05160C_A:QDR2:C3_05800W_A:C3_06150W_A:DOT1:ISW2:C3_06370C_A:NSA2:PRP5:NOP13:C3_07550C_A:RAD4:UTP9:C3_07800C_A:DUR4:RLP24:NMD5:C4_00690C_A:C4_00740W_A:C4_00800W_A:C4_00810C_A:C4_00880W_A:RAT1:C4_01280C_A:C4_01500W_A:SLX4:HIT1:SAS10:RPC53:HCA4:C4_02880C_A:NAN1:C4_03140C_A:C4_03170W_A:C4_03720C_A:C4_03740W_A:NUP84:C4_03830W_A:ECM1:DUO1:C4_04520W_A:BAS1:C4_05260W_A:C4_05650W_A:C4_06790W_A:C4_07060W_A:C4_07150W_A:MRP17:C5_00920W_A:BUD23:CYC3:PUS4:C5_01430C_A:FYV5:SPB4:C5_02010C_A:C5_02070C_A:RIX7:C5_02440C_A:SPR28:UTP13:C5_03010W_A:RMS1:C5_03400C_A:C5_03920C_A:C5_04120C_A:HAS1:C5_04840C_A:C5_04910W_A:GRF10:C5_05340W_A:NOP5:CDC14:TOP1:C6_01040C_A:CIC1:C6_01890C_A:C6_02230W_A:ALG11:C6_02290C_A:C6_02430W_A:C6_02900C_A:POP4:C6_03210C_A:C6_03380W_A:C6_03440W_A:C6_04240W_A:NOP8:C7_00160C_A:C7_00220W_A:C7_00330C_A:THG1:NOP15:C7_01030C_A:DBP7:C7_01210C_A:C7_01270C_A:C7_01360C_A:C7_01950W_A:LIG1:C7_02460C_A:C7_02930C_A:C7_03400C_A:ISY1:ENP2:C7_03590C_A:ENP1:C7_03850W_A:C7_03880C_A:C7_04150W_A:UTP18:CR_00430C_A:CR_00460C_A:BUD22:PWP2:CR_00830W_A:DAL81:CR_01410C_A:CR_01600C_A:CR_01700C_A:CR_01710W_A:CR_01780W_A:CR_02030C_A:

						MCM6:CR_02420W_A:CR_02550C_A:RRP9:CR_02890C_A:SRP40:SMC5:CR_03200C_A:CR_03230W_A:CR_03360W_A:KTI12:NCS2:YVH1:SMC1:CR_03940W_A:CR_04110W_A:CR_04240C_A:CR_04710W_A:NOC2:CR_05550C_A:RNR3:MTG2:DBP8:RFX1:CR_06230W_A:FGR50:CR_06450W_A:RRN11:CR_06680C_A:NMD3:CR_06840W_A:POL2:CR_07030C_A:CR_07310W_A:PIF1:FUN30:DBP6:IMP4:CR_08000C_A:RIO2:ATS1:CR_08330W_A:CR_08410W_A:TSR1:MPS1:CR_09740W_A:CR_09800C_A:DPB2:UTP5:CR_10260W_A:CR_10410C_A:CR_10470C_A:DRS1:LTV1:POP3

42274	ribosomal small subunit biogenesis	338 out of 1061 genes, 31.9%	636 out of 6473 background genes, 9.8%	1.52E-112	0.00%	HBR1:CNS1:RPO41:NRM1:RRS1:C1_01160C_A:C1_02090C_A:ABP140:C1_02450C_A:C1_02900C_A:TSR2:C1_03630W_A:RPP1:C1_03830C_A:C1_04040C_A:C1_04120C_A:NOP4:C1_04510W_A:C1_04710C_A:REI1:TRY2:C1_05220C_A:C1_05360C_A:C1_05380C_A:RSM22:RPS27A:FAL1:C1_05650W_A:C1_06540C_A:NOP6:RIA1:C1_07510W_A:C1_07660W_A:C1_07960W_A:FUN12:MLT1:DIP2:GCD6:C1_08630W_A:MSS116:CEF1:C1_09390W_A:C1_09710C_A:C1_09790C_A:PDC2:MNN23:FCY23:C1_10950C_A:C1_11000C_A:NEP1:KRR1:MUP1:C1_11900C_A:C1_12280C_A:C1_12350W_A:C1_12440W_A:C1_12570C_A:C1_12630C_A:C1_12750C_A:C1_12820C_A:RMP1:C1_13380W_A:C1_13820C_A:C1_14080W_A:C1_14330W_A:MPP10:FAV3:C2_00200W_A:C2_00280C_A:C2_00410C_A:C2_00820W_A:C2_00840W_A:C2_01070W_A:C2_01220W_A:C2_01420C_A:C2_01820C_A:C2_02120W_A:C2_02420C_A:PRS1:C2_02540W_A:C2_02630W_A:PDR17:MET1:PRP3:SCH9:C2_04120C_A:TBF1:SUV3:RIM2:C2_04570W_A:C2_04700C_A:C2_04820W_A:PZF1:CKA2:C2_05050C_A:C2_05080C_A:MAK5:C2_05160C_A:C2_05670C_A:C2_05750W_A:C2_05830C_A:C2_05840W_A:LAS1:MAK16:C2_06520C_A:C2_06660W_A:FHL1:C2_06850W_A:NOC4:C2_07360W_A:RCL1:PRS5:C2_07920W_A:NSA1:C2_08180C_A:RRP8:C2_08530C_A:MSU1:UTP15:C2_09160W_A:C2_09310C_A:PES1:TAZ1:RRP15:C2_09660W_A:SMM1:C2_09920W_A:CWC22:C2_10740C_A:C2_10810W_A:RPS24:C3_00100W_A:UTP8:BUD21:SOF1:C3_00660W_A:BMS1:UTP20:NCE103:C3_01310W_A:C3_01560W_A:C3_02020W_A:C3_02040C_A:UTP4:C3_02350W_A:C3_02670W_A:C3_02840W_A:C3_02850C_A:C3_03070W_A:C3_03110W_A:C3_03330C_A:HBR3:C3_04370C_A:C3_04380C_A:MAK21:SFP1:NOP14:CEM1:C3_05160C_A:QDR2:C3_05800W_A:C3_06150W_A:DOT1:ISW2:C3_06370C_A:NSA2:PRP5:NOP13:C3_07550C_A:RAD4:UTP9:C3_07800C_A:DUR4:RLP24:NMD5:C4_00690C_A:C4_00740W_A:C4_00800W_A:C4_00810C_A:C4_00880W_A:RAT1:C4_01280C_A:C4_01500W_A:SLX4:HIT1:SAS10:RPC53:HCA4:C4_02880C_A:NAN1:C4_03140C_A:C4_03170W_A:C4_03720C_A:C4_03740W_A:NUP84:C4_03830W_A:ECM1:DUO1:C4_04520W_A:BAS1:C4_05260W_A:C4_05650W_A:C4_06790W_A:C4_07060W_A:C4_07150W_A:MRP17:C5_00920W_A:BUD23:CYC3:PUS4:C5_01430C_A:FYV5:SPB4:C5_02010C_A:C5_02070C_A:RIX7:C5_02440C_A:SPR28:UTP13:C5_03010W_A:RMS1:C5_03400C_A:C5_03920C_A:C5_04120C_A:HAS1:C5_04840C_A:C5_04910W_A:GRF10:C5_05340W_A:NOP5:CDC14:TOP1:C6_01040C_A:CIC1:C6_01890C_A:C6_02230W_A:ALG11:C6_02290C_A:C6_02430W_A:C6_02900C_A:POP4:C6_03210C_A:C6_03380W_A:C6_03440W_A:C6_04240W_A:NOP8:C7_00160C_A:C7_00220W_A:C7_00330C_A:THG1:NOP15:C7_01030C_A:DBP7:C7_01210C_A:C7_01270C_A:C7_01360C_A:C7_01950W_A:LIG1:C7_02460C_A:C7_02930C_A:C7_03400C_A:ISY1:ENP2:C7_03590C_A:ENP1:C7_03850W_A:C7_03880C_A:C7_04150W_A:UTP18:CR_00430C_A:CR_00460C_A:BUD22:PWP2:CR_00830W_A:DAL81:CR_01410C_A:CR_01600C_A:CR_01700C_A:CR_01710W_A:CR_01780W_A:CR_02030C_A:

						MCM6:CR_02420W_A:CR_02550C_A:RRP9:CR_02890C_A:SRP40:SMC5:CR_03200C_A:CR_03230W_A:CR_03360W_A:KTI12:NCS2:YVH1:SGD1:SMC1:CR_03940W_A:CR_04110W_A:CR_04240C_A:CR_04710W_A:NOC2:CR_05550C_A:RNR3:MTG2:DBP8:RFX1:CR_06230W_A:FGR50:CR_06450W_A:RRN11:CR_06680C_A:NMD3:CR_06840W_A:POL2:CR_07030C_A:CR_07310W_A:PIF1:FUN30:DBP6:IMP4:CR_08000C_A:RIO2:ATS1:CR_08330W_A:CR_08410W_A:TSR1:MPS1:ELF1:CR_09740W_A:CR_09800C_A:DPB2:UTP5:CR_10260W_A:CR_10410C_A:CR_10470C_A:DRS1:LTV1:POP3

462	maturation of SSU-rRNA from tricistronic rRNA transcript (SSU-rRNA, 5.8S rRNA, LSU-rRNA)	333 out of 1061 genes, 31.4%	620 out of 6473 background genes, 9.6%	2.18E-112	0.00%	HBR1:CNS1:RPO41:NRM1:RRS1:C1_01160C_A:C1_02090C_A:ABP140:C1_02450C_A:C1_02900C_A:TSR2:C1_03630W_A:RPP1:C1_03830C_A:C1_04040C_A:C1_04120C_A:NOP4:C1_04510W_A:C1_04710C_A:REI1:TRY2:C1_05220C_A:C1_05360C_A:C1_05380C_A:RSM22:RPS27A:FAL1:C1_05650W_A:C1_06540C_A:NOP6:RIA1:C1_07510W_A:C1_07660W_A:C1_07960W_A:FUN12:MLT1:DIP2:GCD6:C1_08630W_A:MSS116:CEF1:C1_09390W_A:C1_09710C_A:C1_09790C_A:PDC2:MNN23:FCY23:C1_10950C_A:C1_11000C_A:NEP1:KRR1:MUP1:C1_11900C_A:C1_12280C_A:C1_12350W_A:C1_12440W_A:C1_12570C_A:C1_12630C_A:C1_12750C_A:C1_12820C_A:RMP1:C1_13380W_A:C1_13820C_A:C1_14080W_A:C1_14330W_A:MPP10:FAV3:C2_00200W_A:C2_00280C_A:C2_00410C_A:C2_00820W_A:C2_00840W_A:C2_01070W_A:C2_01220W_A:C2_01420C_A:C2_01820C_A:C2_02120W_A:C2_02420C_A:PRS1:C2_02540W_A:C2_02630W_A:PDR17:MET1:PRP3:SCH9:C2_04120C_A:TBF1:SUV3:RIM2:C2_04570W_A:C2_04700C_A:C2_04820W_A:PZF1:CKA2:C2_05050C_A:C2_05080C_A:MAK5:C2_05160C_A:C2_05670C_A:C2_05750W_A:C2_05830C_A:C2_05840W_A:LAS1:MAK16:C2_06520C_A:C2_06660W_A:FHL1:C2_06850W_A:NOC4:C2_07360W_A:RCL1:PRS5:C2_07920W_A:NSA1:C2_08180C_A:RRP8:C2_08530C_A:MSU1:UTP15:C2_09160W_A:C2_09310C_A:PES1:TAZ1:RRP15:C2_09660W_A:SMM1:C2_09920W_A:CWC22:C2_10740C_A:C2_10810W_A:RPS24:C3_00100W_A:UTP8:BUD21:SOF1:C3_00660W_A:BMS1:UTP20:NCE103:C3_01310W_A:C3_01560W_A:C3_02020W_A:C3_02040C_A:UTP4:C3_02350W_A:C3_02670W_A:C3_02840W_A:C3_02850C_A:C3_03070W_A:C3_03110W_A:C3_03330C_A:HBR3:C3_04370C_A:C3_04380C_A:MAK21:SFP1:NOP14:CEM1:C3_05160C_A:QDR2:C3_05800W_A:C3_06150W_A:DOT1:ISW2:C3_06370C_A:NSA2:PRP5:NOP13:C3_07550C_A:RAD4:UTP9:C3_07800C_A:DUR4:RLP24:NMD5:C4_00690C_A:C4_00740W_A:C4_00800W_A:C4_00810C_A:C4_00880W_A:RAT1:C4_01280C_A:C4_01500W_A:SLX4:HIT1:SAS10:RPC53:HCA4:C4_02880C_A:NAN1:C4_03140C_A:C4_03170W_A:C4_03720C_A:C4_03740W_A:NUP84:C4_03830W_A:ECM1:DUO1:C4_04520W_A:BAS1:C4_05260W_A:C4_05650W_A:C4_06790W_A:C4_07060W_A:C4_07150W_A:MRP17:C5_00920W_A:BUD23:CYC3:PUS4:C5_01430C_A:FYV5:SPB4:C5_02010C_A:C5_02070C_A:RIX7:C5_02440C_A:SPR28:UTP13:C5_03010W_A:RMS1:C5_03920C_A:C5_04120C_A:HAS1:C5_04840C_A:C5_04910W_A:GRF10:C5_05340W_A:NOP5:CDC14:TOP1:C6_01040C_A:CIC1:C6_01890C_A:C6_02230W_A:ALG11:C6_02290C_A:C6_02430W_A:C6_02900C_A:POP4:C6_03210C_A:C6_03380W_A:C6_03440W_A:C6_04240W_A:NOP8:C7_00160C_A:C7_00220W_A:C7_00330C_A:THG1:NOP15:C7_01030C_A:DBP7:C7_01210C_A:C7_01270C_A:C7_01360C_A:C7_01950W_A:LIG1:C7_02460C_A:C7_02930C_A:C7_03400C_A:ISY1:ENP2:C7_03590C_A:ENP1:C7_03850W_A:C7_03880C_A:C7_04150W_A:UTP18:CR_00430C_A:CR_00460C_A:BUD22:PWP2:CR_00830W_A:DAL81:CR_01410C_A:CR_01600C_A:CR_01700C_A:CR_01710W_A:CR_01780W_A:CR_02030C_A:MCM6:CR_024

						20W_A:CR_02550C_A:RRP9:CR_02890C_A:SRP40:SMC5:CR_03200C_A:CR_03360W_A:KTI12:NCS2:YVH1:SMC1:CR_03940W_A:CR_04110W_A:CR_04710W_A:NOC2:CR_05550C_A:RNR3:MTG2:DBP8:RFX1:CR_06230W_A:FGR50:CR_06450W_A:RRN11:CR_06680C_A:NMD3:CR_06840W_A:POL2:CR_07030C_A:CR_07310W_A:PIF1:FUN30:DBP6:IMP4:CR_08000C_A:RIO2:ATS1:CR_08330W_A:CR_08410W_A:TSR1:MPS1:CR_09740W_A:CR_09800C_A:DPB2:UTP5:CR_10260W_A:CR_10410C_A:CR_10470C_A:DRS1:LTV1:POP3

90304	nucleic acid metabolic process	542 out of 1061 genes, 51.1%	1509 out of 6473 background genes, 23.3%	6.01E-103	0.00%	HBR1:CNS1:RPO41:NRM1:RRS1:TRI1:C1_01150C_A:C1_01160C_A:TRM2:C1_01530C_A:C1_02090C_A:ABP140:C1_02380C_A:C1_02390W_A:C1_02450C_A:PDE2:C1_02900C_A:TSR2:C1_03260W_A:C1_03370W_A:C1_03630W_A:RPP1:C1_03830C_A:C1_04040C_A:C1_04120C_A:ERB1:NOP4:C1_04510W_A:C1_04530C_A:C1_04710C_A:C1_04970W_A:RRP6:REI1:TRY2:C1_05220C_A:C1_05270C_A:C1_05360C_A:C1_05380C_A:RSM22:RPS27A:FAL1:C1_05650W_A:RAD10:C1_06540C_A:NOP6:C1_06760C_A:RIA1:C1_07340W_A:C1_07470C_A:LHP1:C1_07510W_A:C1_07660W_A:ECO1:MRPL3:ERD1:C1_07950C_A:C1_07960W_A:FUN12:CAT8:MLT1:DIP2:GCR3:GCD6:C1_08630W_A:MSS116:C1_08890C_A:CEF1:C1_09390W_A:C1_09710C_A:C1_09790C_A:BUD31:PDC2:DBP3:MNN23:C1_10080W_A:FCY23:C1_10620W_A:APN2:RPA190:C1_10920W_A:C1_10950C_A:C1_10970W_A:C1_11000C_A:PET127:NEP1:KRR1:GAR1:MUP1:C1_11900C_A:C1_11910W_A:TRP3:C1_12280C_A:CSI2:C1_12350W_A:WRS1:C1_12440W_A:C1_12570C_A:C1_12630C_A:C1_12670C_A:C1_12750C_A:C1_12760W_A:C1_12820C_A:REX3:RMP1:C1_13380W_A:MCU1:C1_13560W_A:C1_13820C_A:C1_14080W_A:C1_14330W_A:MPP10:FAV3:C2_00200W_A:C2_00280C_A:C2_00410C_A:C2_00820W_A:C2_00840W_A:C2_01070W_A:C2_01220W_A:C2_01240C_A:ESC4:C2_01420C_A:REX2:C2_01820C_A:PRP39:C2_01860C_A:C2_01870C_A:C2_02120W_A:C2_02420C_A:UTP21:UTP22:PRS1:C2_02540W_A:C2_02630W_A:PDR17:MET1:PRP3:SCH9:C2_04120C_A:TBF1:SUV3:RIM2:C2_04570W_A:SNU114:C2_04700C_A:C2_04820W_A:PZF1:CKA2:C2_04990W_A:C2_05050C_A:C2_05080C_A:MAK5:C2_05160C_A:CDC21:RPF2:RPC40:KTI11:C2_05670C_A:C2_05750W_A:C2_05830C_A:C2_05840W_A:LAS1:CSM3:ECM17:MAK16:C2_06480W_A:C2_06520C_A:C2_06530W_A:VAS1:C2_06650C_A:C2_06660W_A:FHL1:C2_06850W_A:NOC4:C2_07360W_A:RCL1:PRS5:C2_07920W_A:NSA1:C2_08180C_A:EAF3:RRP8:C2_08530C_A:MSU1:UTP15:MFG1:CDC47:C2_09160W_A:C2_09180W_A:MED18:C2_09310C_A:PES1:TAZ1:RRP15:C2_09500W_A:C2_09660W_A:SMM1:C2_09920W_A:CWC22:UBP8:PUS7:C2_10740C_A:RPC11:C2_10810W_A:RPS24:C3_00100W_A:ULP3:UTP8:BUD21:SOF1:C3_00660W_A:C3_00830C_A:BMS1:ARP9:UTP20:NCE103:C3_01310W_A:C3_01560W_A:SCM3:C3_02020W_A:C3_02040C_A:UTP4:ILV2:C3_02350W_A:DDC1:C3_02670W_A:SMC3:C3_02750W_A:C3_02840W_A:C3_02850C_A:C3_03070W_A:C3_03110W_A:C3_03330C_A:HBR3:RAD53:C3_04370C_A:C3_04380C_A:MAK21:ARV1:SFP1:NOP14:CEM1:C3_05140C_A:C3_05160C_A:WOR2:RPL8B:C3_05280C_A:C3_05380W_A:C3_05440C_A:QDR2:C3_05800W_A:C3_05860C_A:TRM12:C3_05900W_A:CTA4:NOG1:C3_06150W_A:MMS21:C3_06270C_A:DOT1:ISW2:C3_06370C_A:NSA2:C3_06630W_A:PRP5:NOP13:C3_07400W_A:C3_07550C_A:C3_07570C_A:RIT1:RAD4:UTP9:C3_07800C_A:DUR4:RLP24:NMD5:C4_00690C_A:C4_00700C_A:C4_00740W_A:MLH1:C4_00800W_A:C4_00810C_A:C4_00880W_A:C4_00940W_A:RAT1:C4_01280C_A:PWP1:C4_01500W_A:TOA2:ZCF25:C4_02400C_A:SLX4:HIT1:C4_02770C_A:S

						AS10:RPC53:HCA4:C4_02850W_A:ZUO1:C4_02880C_A:NAN1:C4_03140C_A:C4_03170W_A:DAO2:RAD2:C4_03720C_A:C4_03730C_A:C4_03740W_A:NUP84:C4_03830W_A:RRP42:C4_03870C_A:RAM1:ECM1:DUO1:C4_04520W_A:SSZ1:BAS1:C4_04810C_A:AGP3:RPL30:C4_05230C_A:C4_05260W_A:C4_05360C_A:OFD1:C4_05650W_A:C4_06210C_A:C4_06410W_A:NOP1:C4_06790W_A:C4_06950W_A:C4_07060W_A:C4_07150W_A:C5_00280C_A:DUS4:MRP17:EXO1:RMT2:C5_00920W_A:BUD23:CYC3:C5_01140C_A:PUS4:C5_01430C_A:FYV5:SPB4:C5_01610W_A:C5_01930W_A:C5_02010C_A:C5_02070C_A:RIX7:C5_02440C_A:TIF5:SPR28:UTP13:BUR2:CKB2:MSW1:C5_03010W_A:RMS1:RPO26:C5_03400C_A:C5_03920C_A:C5_04120C_A:MSM1:HYS2:HAS1:C5_04840C_A:C5_04910W_A:GRF10:C5_05340W_A:DNA2:NOP5:C6_00530C_A:C6_00640C_A:CDC14:TOP1:C6_01040C_A:C6_01120C_A:CIC1:RAD18:C6_01890C_A:SWD3:C6_02230W_A:ALG11:C6_02290C_A:C6_02300C_A:C6_02350C_A:C6_02430W_A:PRP45:C6_02690C_A:MRT4:C6_02900C_A:POP4:C6_03210C_A:ARP4:C6_03380W_A:C6_03390W_A:C6_03440W_A:NAM2:SPB1:C6_04240W_A:NOP8:ILS1:C6_04530C_A:C7_00160C_A:C7_00220W_A:C7_00330C_A:DCC1:PSF3:THG1:RPA135:NOP15:C7_01030C_A:DBP7:C7_01210C_A:C7_01270C_A:PSF1:C7_01360C_A:C7_01400C_A:GIR2:FLU1:C7_01570C_A:SMC6:C7_01950W_A:LIG1:C7_02340C_A:C7_02460C_A:C7_02660C_A:C7_02930C_A:C7_03400C_A:ISY1:ENP2:C7_03590C_A:ENP1:C7_03850W_A:C7_03880C_A:C7_04150W_A:UTP18:MMS22:RTT109:CR_00430C_A:CR_00460C_A:BUD22:PWP2:CR_00830W_A:TRM1:DAL81:CTF18:CR_01320C_A:CR_01410C_A:PRS:CR_01550C_A:CR_01600C_A:CR_01670W_A:CDC60:CR_01700C_A:CR_01710W_A:CR_01780W_A:CR_01950W_A:CR_02030C_A:MCM6:CR_02420W_A:CR_02550C_A:RRP9:RPC31:CR_02890C_A:SRP40:ARC1:SMC5:CR_03110W_A:CR_03200C_A:CR_03230W_A:CR_03340C_A:CR_03360W_A:KTI12:CR_03400W_A:CR_03440W_A:NCS2:YVH1:SGD1:SMC1:PRP42:CR_03940W_A:CR_04110W_A:CR_04160C_A:CR_04170W_A:CR_04240C_A:CR_04300W_A:CR_04500C_A:CR_04560C_A:CR_04710W_A:RPB8:NOC2:CR_05550C_A:RNR3:MTG2:DBP8:RFX1:RPL7:CR_06230W_A:FGR50:CR_06450W_A:RRN11:CR_06680C_A:NMD3:CR_06840W_A:CR_06970C_A:POL2:CR_07030C_A:CR_07310W_A:PIF1:FUN30:CR_07640C_A:CDC45:DBP6:IMP4:CR_08000C_A:NOP10:SSB1:RIO2:ATS1:CR_08330W_A:CR_08410W_A:TSR1:CR_08940W_A:MPS1:SSF1:CR_09740W_A:MED21:CR_09800C_A:DPB2:SIK1:CR_10180W_A:UTP5:CR_10260W_A:CR_10410C_A:CR_10470C_A:MCD1:DRS1:LTV1:POP3

6725	cellular aromatic compound metabolic process	586 out of 1061 genes, 55.2%	1727 out of 6473 background genes, 26.7%	4.04E-102	0.00%	HBR1:CNS1:RPO41:NRM1:RRS1:TRI1:HMT1:C1_01150C_A:C1_01160C_A:TRM2:C1_01530C_A:C1_02090C_A:ABP140:C1_02380C_A:C1_02390W_A:C1_02450C_A:SNZ1:SNO1:PDE2:C1_02900C_A:TSR2:C1_03260W_A:C1_03370W_A:C1_03630W_A:RPP1:C1_03830C_A:C1_04040C_A:C1_04120C_A:ERB1:NOP4:C1_04510W_A:C1_04530C_A:C1_04710C_A:C1_04970W_A:RRP6:REI1:TRY2:ARO4:C1_05220C_A:C1_05270C_A:C1_05360C_A:C1_05380C_A:RSM22:RPS27A:FAL1:C1_05650W_A:RAD10:BUD16:C1_06540C_A:NOP6:C1_06760C_A:RIA1:YMC1:C1_07340W_A:C1_07470C_A:LHP1:C1_07510W_A:C1_07660W_A:ADE4:ECO1:ADE5,7:MRPL3:ERD1:C1_07950C_A:C1_07960W_A:FUN12:CAT8:MLT1:DIP2:SAM4:GCR3:GCD6:C1_08630W_A:MSS116:C1_08890C_A:CEF1:C1_09390W_A:GUA1:C1_09710C_A:C1_09790C_A:BUD31:PDC2:DBP3:MNN23:C1_10080W_A:FCY23:C1_10620W_A:APN2:RPA190:C1_10920W_A:C1_10950C_A:C1_10970W_A:C1_11000C_A:PET127:NEP1:KRR1:GAR1:C1_11790W_A:MUP1:C1_11900C_A:C1_11910W_A:TRP3:C1_12280C_A:CSI2:C1_12350W_A:WRS1:C1_12440W_A:C1_12570C_A:C1_12630C_A:C1_12670C_A:C1_12750C_A:C1_12760W_A:C1_12820C_A:REX3:RMP1:C1_13380W_A:MCU1:C1_13560W_A:C1_13820C_A:C1_14080W_A:C1_14330W_A:MPP10:FAV3:C2_00200W_A:C2_00280C_A:C2_00410C_A:C2_00820W_A:C2_00840W_A:C2_01070W_A:C2_01220W_A:C2_01240C_A:ESC4:C2_01420C_A:APT1:REX2:C2_01820C_A:PRP39:C2_01860C_A:C2_01870C_A:ARO3:C2_02120W_A:C2_02420C_A:UTP21:UTP22:PRS1:C2_02540W_A:C2_02630W_A:PDR17:MET1:BNA31:PRP3:SCH9:C2_04120C_A:TBF1:SUV3:RIM2:C2_04570W_A:SNU114:C2_04700C_A:C2_04820W_A:PZF1:CKA2:C2_04990W_A:C2_05050C_A:C2_05080C_A:MAK5:C2_05160C_A:CDC21:RPF2:RPC40:KTI11:C2_05670C_A:C2_05750W_A:C2_05830C_A:C2_05840W_A:LAS1:CSM3:ECM17:SER1:MAK16:C2_06480W_A:C2_06520C_A:C2_06530W_A:VAS1:C2_06650C_A:C2_06660W_A:FHL1:C2_06850W_A:AAH1:NOC4:C2_07360W_A:RCL1:PRS5:C2_07920W_A:NSA1:C2_08180C_A:EAF3:RRP8:C2_08530C_A:MSU1:UTP15:MFG1:CDC47:C2_09160W_A:C2_09180W_A:MED18:C2_09310C_A:PES1:TAZ1:RRP15:C2_09500W_A:C2_09660W_A:SMM1:C2_09920W_A:CWC22:UBP8:PUS7:HEM3:C2_10740C_A:RPC11:C2_10810W_A:RPS24:C3_00100W_A:ULP3:UTP8:BUD21:SOF1:C3_00660W_A:C3_00830C_A:BMS1:ARP9:UTP20:NCE103:C3_01310W_A:URA3:RKI1:C3_01560W_A:SCM3:C3_02020W_A:C3_02040C_A:UTP4:ILV2:C3_02350W_A:PHA2:DDC1:C3_02670W_A:SMC3:C3_02750W_A:C3_02840W_A:C3_02850C_A:C3_03070W_A:C3_03110W_A:C3_03330C_A:YAH1:HBR3:RAD53:C3_04370C_A:C3_04380C_A:ADE2:MAK21:ARV1:SFP1:NOP14:CEM1:C3_05140C_A:C3_05160C_A:WOR2:RPL8B:C3_05280C_A:C3_05380W_A:C3_05440C_A:QDR2:C3_05800W_A:C3_05860C_A:TRM12:C3_05900W_A:CTA4:NOG1:C3_06150W_A:MMS21:C3_06270C_A:DOT1:ISW2:C3_06370C_A:NSA2:C3_06630W_A:PRP5:NOP13:C3_07400W_A:C3_07550C_A:C3_07570C_A:RIT1:RAD4:UTP9:C3_07800C_A:DUR4:RLP24:NMD5:C4_00690C_A:C4_00700C_A:C4_00740W_A:MLH1:C4_00800

						W_A:C4_00810C_A:C4_00880W_A:ARO1:C4_00940W_A:RAT1:C4_01280C_A:PWP1:C4_01500W_A:TOA2:ZCF25:C4_02400C_A:SLX4:HIT1:C4_02770C_A:SAS10:RPC53:HCA4:C4_02850W_A:ZUO1:C4_02880C_A:NAN1:C4_03140C_A:C4_03170W_A:DAO2:RAD2:C4_03720C_A:C4_03730C_A:C4_03740W_A:NUP84:C4_03830W_A:RRP42:C4_03870C_A:RAM1:ECM1:DUO1:C4_04520W_A:SSZ1:BAS1:C4_04810C_A:AGP3:RPL30:C4_05230C_A:C4_05260W_A:C4_05360C_A:OFD1:C4_05650W_A:TRP5:C4_06210C_A:C4_06410W_A:NOP1:C4_06790W_A:C4_06950W_A:C4_07060W_A:TRP4:C4_07150W_A:C5_00260W_A:C5_00280C_A:DUS4:MRP17:EXO1:RMT2:C5_00920W_A:BUD23:CYC3:C5_01140C_A:PUS4:C5_01430C_A:FYV5:SPB4:C5_01610W_A:C5_01930W_A:C5_02010C_A:C5_02070C_A:RIX7:PDE1:C5_02440C_A:TIF5:SPR28:UTP13:BUR2:CKB2:MSW1:C5_03010W_A:RMS1:RPO26:FUR1:C5_03400C_A:C5_03840W_A:HAM1:C5_03920C_A:C5_04120C_A:SAH1:URA7:MSM1:C5_04720C_A:HYS2:HAS1:C5_04840C_A:C5_04910W_A:GRF10:C5_05340W_A:DNA2:NOP5:C6_00530C_A:FCA1:C6_00640C_A:CDC14:TOP1:C6_01040C_A:C6_01120C_A:CIC1:RAD18:C6_01890C_A:SWD3:C6_02230W_A:ALG11:C6_02290C_A:C6_02300C_A:C6_02350C_A:C6_02430W_A:PRP45:C6_02690C_A:MRT4:C6_02900C_A:POP4:C6_03210C_A:ARP4:C6_03380W_A:C6_03390W_A:C6_03440W_A:NAM2:PAD1:SPB1:C6_04240W_A:NOP8:ILS1:C6_04530C_A:C7_00160C_A:C7_00220W_A:C7_00330C_A:DCC1:PSF3:THG1:RPA135:NOP15:C7_01030C_A:DBP7:C7_01210C_A:C7_01270C_A:PSF1:C7_01360C_A:C7_01400C_A:GIR2:FLU1:C7_01570C_A:SMC6:C7_01950W_A:LIG1:C7_02340C_A:C7_02460C_A:C7_02660C_A:C7_02930C_A:C7_03400C_A:ISY1:ENP2:C7_03590C_A:ENP1:C7_03850W_A:C7_03880C_A:C7_04150W_A:UTP18:MIS12:MMS22:RTT109:CR_00430C_A:CR_00460C_A:ADE1:BUD22:PWP2:CR_00830W_A:TRM1:DAL81:CTF18:CR_01320C_A:CR_01410C_A:PRS:CR_01550C_A:TRP2:CR_01600C_A:MET6:CR_01670W_A:CDC60:CR_01700C_A:CR_01710W_A:CR_01780W_A:CR_01950W_A:CR_02030C_A:MCM6:CR_02420W_A:CR_02550C_A:RRP9:RPC31:CR_02890C_A:SRP40:ARC1:SMC5:CR_03110W_A:CR_03200C_A:CR_03230W_A:CR_03340C_A:CR_03360W_A:KTI12:CR_03400W_A:CR_03440W_A:NCS2:YVH1:SGD1:SMC1:PRP42:CR_03940W_A:CR_04110W_A:CR_04160C_A:CR_04170W_A:CR_04240C_A:CR_04300W_A:CR_04500C_A:CR_04560C_A:CR_04710W_A:ADE6:RPB8:CR_04920W_A:NOC2:CR_05550C_A:RNR3:MTG2:DBP8:RFX1:RPL7:CR_06230W_A:FGR50:CR_06450W_A:RRN11:CR_06680C_A:NMD3:CR_06840W_A:CR_06970C_A:POL2:MIS11:CR_07030C_A:URA2:CR_07310W_A:PIF1:FUN30:CR_07640C_A:ARO2:CDC45:DBP6:IMP4:CR_08000C_A:NOP10:SSB1:RIO2:ATS1:CR_08330W_A:CR_08410W_A:TSR1:CR_08940W_A:MPS1:SSF1:CR_09740W_A:MED21:CR_09800C_A:DPB2:SIK1:CR_10180W_A:UTP5:CR_10260W_A:CR_10410C_A:CR_10470C_A:MCD1:DRS1:LTV1:POP3

6139	nucleobase-containing compound metabolic process	566 out of 1061 genes, 53.3%	1659 out of 6473 background genes, 25.6%	4.79E-98	0.00%	HBR1:CNS1:RPO41:NRM1:RRS1:TRI1:C1_01150C_A:C1_01160C_A:TRM2:C1_01530C_A:C1_02090C_A:ABP140:C1_02380C_A:C1_02390W_A:C1_02450C_A:PDE2:C1_02900C_A:TSR2:C1_03260W_A:C1_03370W_A:C1_03630W_A:RPP1:C1_03830C_A:C1_04040C_A:C1_04120C_A:ERB1:NOP4:C1_04510W_A:C1_04530C_A:C1_04710C_A:C1_04970W_A:RRP6:REI1:TRY2:C1_05220C_A:C1_05270C_A:C1_05360C_A:C1_05380C_A:RSM22:RPS27A:FAL1:C1_05650W_A:RAD10:C1_06540C_A:NOP6:C1_06760C_A:RIA1:C1_07340W_A:C1_07470C_A:LHP1:C1_07510W_A:C1_07660W_A:ADE4:ECO1:ADE5,7:MRPL3:ERD1:C1_07950C_A:C1_07960W_A:FUN12:CAT8:MLT1:DIP2:SAM4:GCR3:GCD6:C1_08630W_A:MSS116:C1_08890C_A:CEF1:C1_09390W_A:GUA1:C1_09710C_A:C1_09790C_A:BUD31:PDC2:DBP3:MNN23:C1_10080W_A:FCY23:C1_10620W_A:APN2:RPA190:C1_10920W_A:C1_10950C_A:C1_10970W_A:C1_11000C_A:PET127:NEP1:KRR1:GAR1:C1_11790W_A:MUP1:C1_11900C_A:C1_11910W_A:TRP3:C1_12280C_A:CSI2:C1_12350W_A:WRS1:C1_12440W_A:C1_12570C_A:C1_12630C_A:C1_12670C_A:C1_12750C_A:C1_12760W_A:C1_12820C_A:REX3:RMP1:C1_13380W_A:MCU1:C1_13560W_A:C1_13820C_A:C1_14080W_A:C1_14330W_A:MPP10:FAV3:C2_00200W_A:C2_00280C_A:C2_00410C_A:C2_00820W_A:C2_00840W_A:C2_01070W_A:C2_01220W_A:C2_01240C_A:ESC4:C2_01420C_A:APT1:REX2:C2_01820C_A:PRP39:C2_01860C_A:C2_01870C_A:C2_02120W_A:C2_02420C_A:UTP21:UTP22:PRS1:C2_02540W_A:C2_02630W_A:PDR17:MET1:BNA31:PRP3:SCH9:C2_04120C_A:TBF1:SUV3:RIM2:C2_04570W_A:SNU114:C2_04700C_A:C2_04820W_A:PZF1:CKA2:C2_04990W_A:C2_05050C_A:C2_05080C_A:MAK5:C2_05160C_A:CDC21:RPF2:RPC40:KTI11:C2_05670C_A:C2_05750W_A:C2_05830C_A:C2_05840W_A:LAS1:CSM3:ECM17:SER1:MAK16:C2_06480W_A:C2_06520C_A:C2_06530W_A:VAS1:C2_06650C_A:C2_06660W_A:FHL1:C2_06850W_A:AAH1:NOC4:C2_07360W_A:RCL1:PRS5:C2_07920W_A:NSA1:C2_08180C_A:EAF3:RRP8:C2_08530C_A:MSU1:UTP15:MFG1:CDC47:C2_09160W_A:C2_09180W_A:MED18:C2_09310C_A:PES1:TAZ1:RRP15:C2_09500W_A:C2_09660W_A:SMM1:C2_09920W_A:CWC22:UBP8:PUS7:C2_10740C_A:RPC11:C2_10810W_A:RPS24:C3_00100W_A:ULP3:UTP8:BUD21:SOF1:C3_00660W_A:C3_00830C_A:BMS1:ARP9:UTP20:NCE103:C3_01310W_A:URA3:RKI1:C3_01560W_A:SCM3:C3_02020W_A:C3_02040C_A:UTP4:ILV2:C3_02350W_A:DDC1:C3_02670W_A:SMC3:C3_02750W_A:C3_02840W_A:C3_02850C_A:C3_03070W_A:C3_03110W_A:C3_03330C_A:HBR3:RAD53:C3_04370C_A:C3_04380C_A:ADE2:MAK21:ARV1:SFP1:NOP14:CEM1:C3_05140C_A:C3_05160C_A:WOR2:RPL8B:C3_05280C_A:C3_05380W_A:C3_05440C_A:QDR2:C3_05800W_A:C3_05860C_A:TRM12:C3_05900W_A:CTA4:NOG1:C3_06150W_A:MMS21:C3_06270C_A:DOT1:ISW2:C3_06370C_A:NSA2:C3_06630W_A:PRP5:NOP13:C3_07400W_A:C3_07550C_A:C3_07570C_A:RIT1:RAD4:UTP9:C3_07800C_A:DUR4:RLP24:NMD5:C4_00690C_A:C4_00700C_A:C4_00740W_A:MLH1:C4_00800W_A:C4_00810C_A:C4_00880W_A:C4_00940W_A:RAT1:C4_0

						1280C_A:PWP1:C4_01500W_A:TOA2:ZCF25:C4_02400C_A:SLX4:HIT1:C4_02770C_A:SAS10:RPC53:HCA4:C4_02850W_A:ZUO1:C4_02880C_A:NAN1:C4_03140C_A:C4_03170W_A:DAO2:RAD2:C4_03720C_A:C4_03730C_A:C4_03740W_A:NUP84:C4_03830W_A:RRP42:C4_03870C_A:RAM1:ECM1:DUO1:C4_04520W_A:SSZ1:BAS1:C4_04810C_A:AGP3:RPL30:C4_05230C_A:C4_05260W_A:C4_05360C_A:OFD1:C4_05650W_A:C4_06210C_A:C4_06410W_A:NOP1:C4_06790W_A:C4_06950W_A:C4_07060W_A:C4_07150W_A:C5_00260W_A:C5_00280C_A:DUS4:MRP17:EXO1:RMT2:C5_00920W_A:BUD23:CYC3:C5_01140C_A:PUS4:C5_01430C_A:FYV5:SPB4:C5_01610W_A:C5_01930W_A:C5_02010C_A:C5_02070C_A:RIX7:PDE1:C5_02440C_A:TIF5:SPR28:UTP13:BUR2:CKB2:MSW1:C5_03010W_A:RMS1:RPO26:FUR1:C5_03400C_A:HAM1:C5_03920C_A:C5_04120C_A:SAH1:URA7:MSM1:HYS2:HAS1:C5_04840C_A:C5_04910W_A:GRF10:C5_05340W_A:DNA2:NOP5:C6_00530C_A:FCA1:C6_00640C_A:CDC14:TOP1:C6_01040C_A:C6_01120C_A:CIC1:RAD18:C6_01890C_A:SWD3:C6_02230W_A:ALG11:C6_02290C_A:C6_02300C_A:C6_02350C_A:C6_02430W_A:PRP45:C6_02690C_A:MRT4:C6_02900C_A:POP4:C6_03210C_A:ARP4:C6_03380W_A:C6_03390W_A:C6_03440W_A:NAM2:SPB1:C6_04240W_A:NOP8:ILS1:C6_04530C_A:C7_00160C_A:C7_00220W_A:C7_00330C_A:DCC1:PSF3:THG1:RPA135:NOP15:C7_01030C_A:DBP7:C7_01210C_A:C7_01270C_A:PSF1:C7_01360C_A:C7_01400C_A:GIR2:FLU1:C7_01570C_A:SMC6:C7_01950W_A:LIG1:C7_02340C_A:C7_02460C_A:C7_02660C_A:C7_02930C_A:C7_03400C_A:ISY1:ENP2:C7_03590C_A:ENP1:C7_03850W_A:C7_03880C_A:C7_04150W_A:UTP18:MMS22:RTT109:CR_00430C_A:CR_00460C_A:ADE1:BUD22:PWP2:CR_00830W_A:TRM1:DAL81:CTF18:CR_01320C_A:CR_01410C_A:PRS:CR_01550C_A:CR_01600C_A:MET6:CR_01670W_A:CDC60:CR_01700C_A:CR_01710W_A:CR_01780W_A:CR_01950W_A:CR_02030C_A:MCM6:CR_02420W_A:CR_02550C_A:RRP9:RPC31:CR_02890C_A:SRP40:ARC1:SMC5:CR_03110W_A:CR_03200C_A:CR_03230W_A:CR_03340C_A:CR_03360W_A:KTI12:CR_03400W_A:CR_03440W_A:NCS2:YVH1:SGD1:SMC1:PRP42:CR_03940W_A:CR_04110W_A:CR_04160C_A:CR_04170W_A:CR_04240C_A:CR_04300W_A:CR_04500C_A:CR_04560C_A:CR_04710W_A:ADE6:RPB8:NOC2:CR_05550C_A:RNR3:MTG2:DBP8:RFX1:RPL7:CR_06230W_A:FGR50:CR_06450W_A:RRN11:CR_06680C_A:NMD3:CR_06840W_A:CR_06970C_A:POL2:MIS11:CR_07030C_A:URA2:CR_07310W_A:PIF1:FUN30:CR_07640C_A:CDC45:DBP6:IMP4:CR_08000C_A:NOP10:SSB1:RIO2:ATS1:CR_08330W_A:CR_08410W_A:TSR1:CR_08940W_A:MPS1:SSF1:CR_09740W_A:MED21:CR_09800C_A:DPB2:SIK1:CR_10180W_A:UTP5:CR_10260W_A:CR_10410C_A:CR_10470C_A:MCD1:DRS1:LTV1:POP3

46483	heterocycle metabolic process	581 out of 1061 genes, 54.8%	1733 out of 6473 background genes, 26.8%	4.93E-98	0.00%	RIM8:HBR1:CNS1:RPO41:NRM1:RRS1:TRI1:HMT1:C1_01150C_A:C1_01160C_A:TRM2:C1_01530C_A:C1_02090C_A:ABP140:C1_02380C_A:C1_02390W_A:C1_02450C_A:SNZ1:SNO1:PDE2:C1_02900C_A:TSR2:C1_03260W_A:C1_03370W_A:C1_03630W_A:RPP1:C1_03830C_A:C1_04040C_A:C1_04120C_A:ERB1:NOP4:C1_04510W_A:C1_04530C_A:C1_04710C_A:C1_04970W_A:RRP6:REI1:TRY2:C1_05220C_A:C1_05270C_A:C1_05360C_A:C1_05380C_A:RSM22:RPS27A:FAL1:C1_05650W_A:RAD10:BUD16:C1_06540C_A:NOP6:C1_06760C_A:RIA1:YMC1:C1_07340W_A:C1_07470C_A:LHP1:C1_07510W_A:C1_07660W_A:ADE4:ECO1:ADE5,7:MRPL3:ERD1:C1_07950C_A:C1_07960W_A:FUN12:CAT8:MLT1:DIP2:SAM4:GCR3:GCD6:C1_08630W_A:MSS116:C1_08890C_A:CEF1:C1_09390W_A:GUA1:C1_09710C_A:C1_09790C_A:BUD31:PDC2:DBP3:MNN23:C1_10080W_A:FCY23:C1_10620W_A:APN2:RPA190:C1_10920W_A:C1_10950C_A:C1_10970W_A:C1_11000C_A:PET127:NEP1:KRR1:GAR1:C1_11790W_A:MUP1:C1_11900C_A:C1_11910W_A:TRP3:C1_12280C_A:CSI2:C1_12350W_A:WRS1:C1_12440W_A:C1_12570C_A:C1_12630C_A:C1_12670C_A:C1_12750C_A:C1_12760W_A:C1_12820C_A:REX3:RMP1:C1_13380W_A:MCU1:C1_13560W_A:C1_13820C_A:C1_14080W_A:C1_14330W_A:MPP10:FAV3:C2_00200W_A:C2_00280C_A:C2_00410C_A:C2_00820W_A:C2_00840W_A:C2_01070W_A:C2_01220W_A:C2_01240C_A:ESC4:C2_01420C_A:APT1:REX2:C2_01820C_A:PRP39:C2_01860C_A:C2_01870C_A:C2_02120W_A:C2_02420C_A:UTP21:UTP22:PRS1:C2_02540W_A:C2_02630W_A:PDR17:MET1:BNA31:PRP3:SCH9:C2_04120C_A:TBF1:SUV3:RIM2:C2_04570W_A:SNU114:C2_04700C_A:C2_04820W_A:PZF1:CKA2:C2_04990W_A:C2_05050C_A:C2_05080C_A:MAK5:C2_05160C_A:CDC21:RPF2:RPC40:KTI11:C2_05670C_A:C2_05750W_A:C2_05830C_A:C2_05840W_A:LAS1:CSM3:ECM17:SER1:MAK16:C2_06480W_A:C2_06520C_A:C2_06530W_A:VAS1:C2_06650C_A:C2_06660W_A:FHL1:C2_06850W_A:AAH1:NOC4:C2_07360W_A:RCL1:PRS5:C2_07920W_A:NSA1:C2_08180C_A:EAF3:RRP8:C2_08530C_A:MSU1:UTP15:MFG1:CDC47:C2_09160W_A:C2_09180W_A:MED18:C2_09310C_A:PES1:TAZ1:RRP15:C2_09500W_A:C2_09660W_A:SMM1:C2_09920W_A:CWC22:UBP8:PUS7:HEM3:C2_10740C_A:RPC11:C2_10810W_A:RPS24:C3_00100W_A:ULP3:UTP8:BUD21:SOF1:C3_00660W_A:C3_00830C_A:BMS1:ARP9:UTP20:NCE103:C3_01310W_A:URA3:RKI1:C3_01560W_A:SCM3:C3_02020W_A:C3_02040C_A:UTP4:ILV2:C3_02350W_A:DDC1:C3_02670W_A:SMC3:C3_02750W_A:C3_02840W_A:C3_02850C_A:C3_03070W_A:C3_03110W_A:C3_03330C_A:YAH1:HBR3:RAD53:C3_04370C_A:C3_04380C_A:ADE2:MAK21:ARV1:SFP1:NOP14:CEM1:C3_05140C_A:C3_05160C_A:WOR2:RPL8B:C3_05280C_A:C3_05380W_A:C3_05440C_A:QDR2:C3_05800W_A:C3_05860C_A:TRM12:C3_05900W_A:CTA4:NOG1:C3_06150W_A:MMS21:C3_06270C_A:DOT1:ISW2:C3_06370C_A:NSA2:C3_06630W_A:PRP5:NOP13:C3_07400W_A:C3_07550C_A:C3_07570C_A:RIT1:RAD4:UTP9:C3_07800C_A:DUR4:RLP24:NMD5:C4_00690C_A:C4_00700C_A:C4_00740W_A:MLH1:C4_00800W_A:C4_0

						0810C_A:C4_00880W_A:C4_00940W_A:RAT1:C4_01280C_A:PWP1:C4_01500W_A:TOA2:ZCF25:C4_02400C_A:SLX4:HIT1:C4_02770C_A:SAS10:RPC53:HCA4:C4_02850W_A:ZUO1:C4_02880C_A:NAN1:C4_03140C_A:C4_03170W_A:DAO2:RAD2:C4_03720C_A:C4_03730C_A:C4_03740W_A:NUP84:C4_03830W_A:RRP42:C4_03870C_A:RAM1:ECM1:DUO1:C4_04520W_A:SSZ1:BAS1:C4_04810C_A:AGP3:RPL30:C4_05230C_A:C4_05260W_A:C4_05360C_A:OFD1:C4_05650W_A:TRP5:C4_06210C_A:C4_06410W_A:NOP1:C4_06790W_A:C4_06950W_A:C4_07060W_A:TRP4:C4_07150W_A:C5_00260W_A:C5_00280C_A:DUS4:MRP17:EXO1:RMT2:C5_00920W_A:BUD23:CYC3:C5_01140C_A:PUS4:C5_01430C_A:FYV5:SPB4:C5_01610W_A:C5_01930W_A:C5_02010C_A:C5_02070C_A:RIX7:PDE1:C5_02440C_A:TIF5:SPR28:UTP13:BUR2:CKB2:MSW1:C5_03010W_A:RMS1:RPO26:FUR1:C5_03400C_A:C5_03840W_A:HAM1:C5_03920C_A:C5_04120C_A:SAH1:URA7:MSM1:C5_04720C_A:HYS2:HAS1:C5_04840C_A:C5_04910W_A:GRF10:C5_05340W_A:DNA2:NOP5:C6_00530C_A:FCA1:C6_00640C_A:CDC14:TOP1:C6_01040C_A:C6_01120C_A:CIC1:RAD18:C6_01890C_A:SWD3:C6_02230W_A:ALG11:C6_02290C_A:C6_02300C_A:C6_02350C_A:C6_02430W_A:PRP45:C6_02690C_A:MRT4:C6_02900C_A:POP4:C6_03210C_A:ARP4:C6_03380W_A:C6_03390W_A:C6_03440W_A:NAM2:SPB1:C6_04240W_A:NOP8:ILS1:C6_04530C_A:C7_00160C_A:C7_00220W_A:C7_00330C_A:DCC1:PSF3:THG1:RPA135:NOP15:C7_01030C_A:DBP7:C7_01210C_A:C7_01270C_A:PSF1:C7_01360C_A:C7_01400C_A:GIR2:FLU1:C7_01570C_A:SMC6:C7_01950W_A:LIG1:C7_02340C_A:C7_02460C_A:C7_02660C_A:C7_02930C_A:C7_03400C_A:ISY1:ENP2:C7_03590C_A:ENP1:C7_03850W_A:C7_03880C_A:C7_04150W_A:UTP18:MIS12:MMS22:RTT109:CR_00430C_A:CR_00460C_A:ADE1:BUD22:PWP2:CR_00830W_A:TRM1:DAL81:CTF18:CR_01320C_A:CR_01410C_A:PRS:CR_01550C_A:TRP2:CR_01600C_A:MET6:CR_01670W_A:CDC60:CR_01700C_A:CR_01710W_A:CR_01780W_A:CR_01950W_A:CR_02030C_A:MCM6:CR_02420W_A:CR_02550C_A:RRP9:RPC31:CR_02890C_A:SRP40:ARC1:SMC5:CR_03110W_A:CR_03200C_A:CR_03230W_A:CR_03340C_A:CR_03360W_A:KTI12:CR_03400W_A:CR_03440W_A:NCS2:YVH1:SGD1:SMC1:PRP42:CR_03940W_A:CR_04110W_A:CR_04160C_A:CR_04170W_A:CR_04240C_A:CR_04300W_A:CR_04500C_A:CR_04560C_A:CR_04710W_A:ADE6:RPB8:CR_04920W_A:NOC2:CR_05550C_A:RNR3:MTG2:DBP8:RFX1:RPL7:CR_06230W_A:FGR50:CR_06450W_A:RRN11:CR_06680C_A:NMD3:CR_06840W_A:CR_06970C_A:POL2:MIS11:CR_07030C_A:URA2:CR_07310W_A:PIF1:FUN30:CR_07640C_A:CDC45:DBP6:IMP4:CR_08000C_A:NOP10:SSB1:RIO2:ATS1:CR_08330W_A:CR_08410W_A:TSR1:CR_08940W_A:MPS1:SSF1:CR_09740W_A:MED21:CR_09800C_A:DPB2:SIK1:CR_10180W_A:UTP5:CR_10260W_A:CR_10410C_A:CR_10470C_A:MCD1:DRS1:LTV1:POP3

44085	cellular component biogenesis	487 out of 1061 genes, 45.9%	1340 out of 6473 background genes, 20.7%	1.15E-90	0.00%	HBR1:CNS1:RPO41:NRM1:RRS1:TRI1:C1_01160C_A:C1_02090C_A:ABP140:C1_02380C_A:C1_02450C_A:PDE2:C1_02900C_A:TSR2:ARX1:C1_03630W_A:C1_03790C_A:RPP1:C1_03830C_A:C1_04040C_A:C1_04120C_A:ERB1:NOP4:C1_04510W_A:C1_04710C_A:C1_04970W_A:RRP6:REI1:TRY2:C1_05220C_A:C1_05360C_A:C1_05380C_A:RSM22:RPS27A:FAL1:C1_05650W_A:C1_06530C_A:C1_06540C_A:NOP6:C1_06760C_A:RIA1:C1_07510W_A:C1_07660W_A:ERD1:C1_07950C_A:C1_07960W_A:FUN12:MLT1:DIP2:GCR3:GCD6:C1_08630W_A:MSS116:JIP5:NAR1:C1_09040C_A:CEF1:C1_09390W_A:YTM1:C1_09620C_A:C1_09710C_A:C1_09790C_A:PDC2:DBP3:MNN23:FCY23:C1_10620W_A:RPA190:C1_10920W_A:C1_10950C_A:C1_10970W_A:C1_11000C_A:NEP1:KRR1:GAR1:MUP1:C1_11900C_A:C1_11910W_A:TRP3:C1_12280C_A:CSI2:C1_12350W_A:C1_12440W_A:C1_12570C_A:C1_12630C_A:C1_12640W_A:C1_12680W_A:C1_12750C_A:C1_12760W_A:C1_12820C_A:C1_13010W_A:REX3:RMP1:C1_13370W_A:C1_13380W_A:COX19:MCU1:C1_13820C_A:C1_14080W_A:C1_14330W_A:MPP10:FAV3:C2_00200W_A:C2_00280C_A:C2_00410C_A:C2_00810C_A:C2_00820W_A:C2_00840W_A:C2_01070W_A:C2_01220W_A:C2_01420C_A:REX2:C2_01820C_A:PRP39:C2_01860C_A:C2_02120W_A:SAM37:C2_02420C_A:UTP21:UTP22:PRS1:C2_02540W_A:C2_02630W_A:PDR17:MET1:SMP3:PRP3:MNN42:SCH9:C2_04120C_A:TBF1:SUV3:RIM2:C2_04570W_A:SNU114:C2_04700C_A:C2_04820W_A:PZF1:CKA2:C2_05050C_A:C2_05080C_A:MAK5:C2_05160C_A:SGO1:RPF2:C2_05670C_A:C2_05750W_A:C2_05830C_A:C2_05840W_A:LAS1:ECM17:MAK16:C2_06520C_A:C2_06530W_A:C2_06650C_A:C2_06660W_A:RPL11:FHL1:C2_06850W_A:C2_06950C_A:NOC4:C2_07360W_A:RCL1:PRS5:C2_07920W_A:NSA1:KRE30:RPS10:C2_08180C_A:EAF3:RRP8:C2_08530C_A:MSU1:UTP15:MFG1:CDC47:C2_09160W_A:C2_09180W_A:C2_09290W_A:MED18:C2_09310C_A:PES1:TAZ1:RRP15:C2_09660W_A:SMM1:C2_09920W_A:CWC22:PUS7:C2_10740C_A:C2_10810W_A:RPS24:C3_00100W_A:UTP8:BUD21:SOF1:C3_00660W_A:C3_00830C_A:BMS1:ARP9:UTP20:NCE103:C3_01310W_A:RPS7A:C3_01560W_A:SCM3:C3_02020W_A:C3_02040C_A:UTP4:ILV2:C3_02350W_A:C3_02670W_A:SMC3:C3_02840W_A:C3_02850C_A:C3_03070W_A:C3_03110W_A:C3_03330C_A:YAH1:HBR3:C3_04370C_A:C3_04380C_A:MAK21:RPS15:ARV1:SFP1:NOP14:CEM1:C3_05160C_A:RPL8B:C3_05280C_A:C3_05380W_A:C3_05440C_A:QDR2:C3_05800W_A:C3_05860C_A:C3_05900W_A:NOG1:C3_06150W_A:DOT1:ISW2:C3_06370C_A:NSA2:C3_06600C_A:PRP5:NOP13:C3_07550C_A:C3_07570C_A:RAD4:UTP9:C3_07800C_A:DUR4:RLP24:NMD5:C4_00690C_A:C4_00740W_A:C4_00800W_A:C4_00810C_A:C4_00880W_A:RAT1:C4_01280C_A:PWP1:C4_01500W_A:TOA2:C4_02400C_A:SLX4:HIT1:C4_02770C_A:SAS10:RPC53:HCA4:ZUO1:C4_02880C_A:NAN1:C4_03140C_A:C4_03170W_A:DAO2:C4_03720C_A:C4_03740W_A:NUP84:C4_03830W_A:RRP42:RAM1:ECM1:C4_04130W_A:DUO1:C4_04520W_A:SSZ1:BAS1:C4_04820C_A:AGP3:RPL30:C4_05010W_A:C4_05230C_A:C4_05260W_A:C4_0

						5360C_A:TUB4:C4_05650W_A:C4_06210C_A:C4_06410W_A:NOP1:C4_06790W_A:C4_06950W_A:C4_07060W_A:C4_07150W_A:MRP17:RMT2:C5_00920W_A:BUD23:CYC3:PUS4:C5_01430C_A:FYV5:SPB4:C5_01930W_A:C5_02010C_A:C5_02070C_A:RIX7:C5_02440C_A:TIF5:SPR28:UTP13:BUR2:C5_02740W_A:CKB2:C5_03010W_A:RMS1:C5_03400C_A:C5_03920C_A:C5_04120C_A:C5_04720C_A:HAS1:C5_04840C_A:C5_04910W_A:GRF10:C5_05340W_A:NOP5:C6_00560W_A:CDC14:TOP1:C6_01040C_A:C6_01120C_A:CIC1:QDR3:TIF3:C6_01890C_A:SWD3:C6_02230W_A:RPL10A:ALG11:C6_02290C_A:C6_02430W_A:PRP45:MRT4:C6_02900C_A:POP4:C6_03210C_A:ARP4:C6_03380W_A:C6_03390W_A:C6_03440W_A:NOG2:SPB1:C6_04240W_A:NOP8:C7_00160C_A:C7_00220W_A:C7_00330C_A:THG1:RPA135:NOP15:C7_01030C_A:DBP7:C7_01210C_A:C7_01270C_A:C7_01360C_A:GIR2:FLU1:C7_01570C_A:C7_01600W_A:C7_01950W_A:LIG1:C7_02460C_A:C7_02660C_A:C7_02930C_A:C7_03400C_A:ISY1:ENP2:C7_03590C_A:ENP1:C7_03850W_A:C7_03880C_A:C7_04150W_A:UTP18:CR_00430C_A:CR_00460C_A:BUD22:PWP2:CR_00830W_A:DAL81:CR_01320C_A:CR_01410C_A:CR_01550C_A:CR_01600C_A:CR_01670W_A:CR_01700C_A:CR_01710W_A:CR_01780W_A:CR_02030C_A:MCM6:CR_02420W_A:CR_02550C_A:RRP9:CR_02890C_A:SRP40:ARC1:SMC5:CR_03200C_A:CR_03230W_A:CR_03340C_A:CR_03360W_A:KTI12:NCS2:YVH1:SGD1:SMC1:PRP42:CR_03940W_A:MEX67:CR_04110W_A:CR_04170W_A:QDR1:CR_04240C_A:CR_04500C_A:CR_04710W_A:NOC2:CR_05550C_A:RNR3:MTG2:DBP8:SDA1:RFX1:RPL7:CR_06230W_A:FGR50:CR_06450W_A:RRN11:HSP60:CR_06680C_A:NMD3:CR_06740W_A:CR_06840W_A:CR_06970C_A:POL2:CR_07030C_A:CR_07310W_A:PIF1:FUN30:CR_07640C_A:CDC45:DBP6:IMP4:CR_08000C_A:NOP10:SSB1:RIO2:ATS1:CR_08330W_A:CR_08410W_A:TSR1:CR_08500W_A:MPS1:ELF1:SSF1:CR_09740W_A:MED21:CR_09800C_A:DPB2:SIK1:CR_10180W_A:UTP5:CR_10260W_A:CR_10410C_A:CR_10470C_A:DRS1:LTV1:POP3

34641	cellular nitrogen compound metabolic process	671 out of 1061 genes, 63.2%	2297 out of 6473 background genes, 35.5%	1.26E-87	0.00%	RIM8:HBR1:C1_00510W_A:CNS1:RPO41:SPE1:NRM1:RRS1:TRI1:HMT1:C1_01150C_A:C1_01160C_A:TRM2:C1_01530C_A:C1_02090C_A:ABP140:C1_02380C_A:C1_02390W_A:C1_02450C_A:SNZ1:SNO1:HOM6:PDE2:MIA40:C1_02900C_A:C1_02970W_A:TSR2:C1_03260W_A:C1_03370W_A:C1_03540C_A:C1_03630W_A:RPP1:C1_03830C_A:C1_04040C_A:C1_04120C_A:ERB1:NOP4:C1_04510W_A:C1_04530C_A:C1_04710C_A:MRPL37:C1_04970W_A:RRP6:REI1:TRY2:C1_05220C_A:C1_05270C_A:C1_05360C_A:C1_05380C_A:RSM22:RPS27A:C1_05630C_A:FAL1:C1_05650W_A:RAD10:C1_05990C_A:BUD16:C1_06540C_A:NOP6:C1_06760C_A:RIA1:YMC1:C1_07340W_A:C1_07470C_A:LHP1:C1_07510W_A:C1_07660W_A:ADE4:ECO1:GCS1:ADE5,7:MRPL3:ERD1:C1_07950C_A:C1_07960W_A:FUN12:DRG1:CAT8:MLT1:DIP2:SAM4:GCR3:C1_08520C_A:GCD6:C1_08630W_A:MSS116:C1_08890C_A:CEF1:C1_09390W_A:GUA1:C1_09710C_A:C1_09790C_A:BUD31:C1_09910C_A:PDC2:DBP3:MNN23:C1_10080W_A:C1_10200C_A:FCY23:C1_10620W_A:APN2:RPA190:C1_10690W_A:C1_10920W_A:C1_10950C_A:C1_10970W_A:C1_11000C_A:PET127:FGR39:NEP1:KRR1:SAM2:GAR1:C1_11790W_A:MUP1:C1_11880W_A:C1_11900C_A:C1_11910W_A:SER33:TRP3:C1_12280C_A:CSI2:MRPS9:C1_12350W_A:WRS1:C1_12440W_A:C1_12570C_A:C1_12630C_A:C1_12670C_A:C1_12750C_A:C1_12760W_A:FEN1:C1_12820C_A:RPL14:C1_13060C_A:REX3:C1_13330C_A:RMP1:C1_13380W_A:AGC1:MCU1:C1_13560W_A:C1_13820C_A:C1_14080W_A:C1_14330W_A:MPP10:FAV3:C2_00200W_A:C2_00280C_A:C2_00410C_A:C2_00820W_A:C2_00840W_A:C2_01070W_A:C2_01220W_A:C2_01240C_A:ESC4:C2_01420C_A:APT1:REX2:C2_01740C_A:C2_01820C_A:PRP39:C2_01860C_A:C2_01870C_A:C2_02120W_A:C2_02270C_A:C2_02420C_A:UTP21:UTP22:PRS1:C2_02540W_A:C2_02630W_A:PDR17:C2_02930C_A:MET1:BNA31:SMP3:PRP3:C2_03560C_A:SCH9:C2_03950W_A:C2_04120C_A:TBF1:SUV3:RIM2:C2_04570W_A:SNU114:C2_04700C_A:C2_04820W_A:PZF1:CKA2:C2_04990W_A:C2_05050C_A:C2_05080C_A:MAK5:C2_05160C_A:CDC21:RPF2:C2_05270W_A:RPC40:KTI11:C2_05670C_A:C2_05750W_A:C2_05830C_A:C2_05840W_A:IDP1:LAS1:CSM3:ECM17:SER1:MAK16:C2_06480W_A:C2_06520C_A:C2_06530W_A:VAS1:C2_06650C_A:C2_06660W_A:RPL11:FHL1:C2_06850W_A:C2_06950C_A:SPE3:AAH1:NOC4:C2_07360W_A:RCL1:C2_07680W_A:PRS5:C2_07920W_A:NSA1:C2_08180C_A:EAF3:RRP8:C2_08530C_A:MSU1:UTP15:MFG1:CDC47:C2_09160W_A:C2_09180W_A:MED18:C2_09310C_A:PES1:TAZ1:RRP15:C2_09500W_A:C2_09660W_A:SMM1:C2_09920W_A:CWC22:UBP8:PUS7:HEM3:C2_10740C_A:RPC11:C2_10810W_A:RPS24:C3_00100W_A:ULP3:UTP8:BUD21:SOF1:C3_00660W_A:C3_00830C_A:BMS1:ARP9:UTP20:NCE103:C3_01310W_A:URA3:RKI1:RPS7A:C3_01520C_A:C3_01560W_A:SCM3:C3_02020W_A:C3_02040C_A:UTP4:ILV2:C3_02350W_A:DDC1:C3_02670W_A:SMC3:C3_02750W_A:C3_02840W_A:C3_02850C_A:C3_03070W_A:C3_03110W_A:C3_03330C_A:YAH1:HBR3:RAD53:C3_04370C_A:C3_04380C_A:ADE2:MAK21:RPS15:RPP2B:ARV

						1:SFP1:NOP14:CEM1:C3_05140C_A:C3_05160C_A:WOR2:RPL8B:C3_05280C_A:C3_05380W_A:C3_05440C_A:QDR2:C3_05800W_A:C3_05860C_A:TRM12:C3_05900W_A:CTA4:NOG1:C3_06150W_A:MMS21:C3_06240C_A:C3_06270C_A:DOT1:ISW2:C3_06370C_A:NSA2:C3_06630W_A:PRP5:C3_06830C_A:RPS12:NOP13:C3_07400W_A:C3_07550C_A:C3_07570C_A:RIT1:RAD4:UTP9:C3_07800C_A:DUR4:RLP24:NMD5:HIS5:C4_00690C_A:C4_00700C_A:C4_00740W_A:MLH1:C4_00800W_A:C4_00810C_A:C4_00880W_A:ARO1:C4_00940W_A:RAT1:C4_01280C_A:PGA53:PWP1:NIP1:C4_01500W_A:TOA2:HSX11:TIM10:ZCF25:C4_02400C_A:SLX4:HIT1:C4_02770C_A:SAS10:RPC53:HCA4:C4_02850W_A:ZUO1:C4_02880C_A:NAN1:C4_03140C_A:C4_03170W_A:DAO2:C4_03410W_A:RAD2:C4_03720C_A:C4_03730C_A:C4_03740W_A:NUP84:C4_03830W_A:RRP42:C4_03870C_A:RAM1:ECM1:C4_04130W_A:DUO1:C4_04390W_A:C4_04520W_A:SSZ1:BAS1:C4_04810C_A:AGP3:RPL30:C4_05230C_A:C4_05260W_A:C4_05360C_A:OFD1:C4_05650W_A:TRP5:C4_06210C_A:C4_06410W_A:NOP1:C4_06790W_A:C4_06950W_A:C4_07060W_A:TRP4:C4_07140W_A:C4_07150W_A:C5_00030W_A:C5_00260W_A:C5_00280C_A:DUS4:C5_00320W_A:MRP17:MET14:EXO1:RMT2:SPE2:C5_00820W_A:C5_00920W_A:BUD23:CYC3:C5_01140C_A:PUS4:C5_01430C_A:FYV5:SPB4:C5_01610W_A:C5_01700W_A:C5_01930W_A:C5_02010C_A:C5_02070C_A:RIX7:PDE1:C5_02440C_A:TIF5:SPR28:UTP13:C5_02590C_A:BUR2:CKB2:MSW1:C5_02780W_A:C5_02820C_A:C5_03010W_A:RMS1:CAM1-1:C5_03290C_A:RPO26:FUR1:C5_03400C_A:C5_03530C_A:C5_03840W_A:HAM1:C5_03920C_A:C5_04120C_A:SAH1:C5_04290C_A:URA7:CSU57:MSM1:C5_04720C_A:HYS2:HAS1:C5_04840C_A:C5_04910W_A:GRF10:C5_05250C_A:SDH4:C5_05340W_A:DNA2:NOP5:C6_00530C_A:FCA1:C6_00640C_A:CDC14:TOP1:C6_01040C_A:C6_01120C_A:CIC1:PMP1:TIF3:RAD18:C6_01890C_A:C6_01980C_A:SWD3:C6_02230W_A:RPL10A:ALG11:C6_02290C_A:C6_02300C_A:C6_02350C_A:C6_02370C_A:C6_02430W_A:PRP45:C6_02690C_A:MRT4:C6_02900C_A:POP4:C6_03210C_A:ARP4:C6_03380W_A:C6_03390W_A:C6_03440W_A:NAM2:SPB1:C6_04240W_A:NOP8:ILS1:C6_04530C_A:C7_00160C_A:C7_00220W_A:C7_00330C_A:DCC1:PSF3:THG1:RPA135:NOP15:YML6:C7_01020C_A:C7_01030C_A:DBP7:C7_01210C_A:ASC1:C7_01270C_A:PSF1:C7_01360C_A:C7_01400C_A:GIR2:PRT1:FLU1:C7_01570C_A:SMC6:C7_01600W_A:C7_01950W_A:LIG1:C7_02340C_A:C7_02460C_A:C7_02660C_A:C7_02930C_A:IFM1:C7_03400C_A:ISY1:ENP2:C7_03590C_A:ENP1:HIS7:MRP7:C7_03850W_A:C7_03880C_A:C7_04150W_A:UTP18:MIS12:MMS22:RTT109:CR_00430C_A:CR_00460C_A:MRPL6:ADE1:BUD22:PWP2:CR_00830W_A:TRM1:DAL81:CTF18:CR_01320C_A:CR_01410C_A:PRS:CR_01550C_A:TRP2:CR_01600C_A:MET6:CR_01670W_A:CDC60:CR_01700C_A:CR_01710W_A:CR_01780W_A:CR_01950W_A:CR_02030C_A:MCM6:ERG25:CR_02420W_A:CR_02430C_A:CR_02550C_A:PAN6:RRP9:RPC31:CR_02890C_A:SRP40:ARC1:SMC5:CR_03110W_A:CR_03200C_A:CR_03230W_A:CR_03340C_A:CR_033

						60W_A:KTI12:MTG1:CR_03400W_A:CR_03440W_A:NCS2:YVH1:SGD1:SMC1:PRP42:CR_03940W_A:CR_04110W_A:CR_04160C_A:CR_04170W_A:CR_04240C_A:CR_04300W_A:CR_04500C_A:CR_04560C_A:CR_04710W_A:ADE6:RPB8:CR_04920W_A:NOC2:CR_05550C_A:RNR3:MTG2:DBP8:RFX1:RPL7:CR_06230W_A:FGR50:CR_06450W_A:RRN11:CR_06680C_A:NMD3:CR_06840W_A:CR_06970C_A:POL2:MIS11:CR_07030C_A:URA2:CR_07310W_A:PIF1:FUN30:CR_07640C_A:CDC45:DBP6:IMP4:CR_08000C_A:NOP10:SSB1:RIO2:ATS1:CR_08330W_A:RPP2A:GSH2:CR_08410W_A:TSR1:CR_08940W_A:MPS1:SSF1:CR_09740W_A:MED21:CR_09800C_A:DPB2:SIK1:CR_10180W_A:UTP5:CR_10260W_A:CR_10410C_A:CR_10470C_A:MCD1:DRS1:LTV1:POP3

2E+06	organic cyclic compound metabolic process	597 out of 1061 genes, 56.3%	1903 out of 6473 background genes, 29.4%	4.81E-87	0.00%	RIM8:HBR1:CNS1:RPO41:NRM1:RRS1:TRI1:HMT1:C1_01150C_A:C1_01160C_A:TRM2:C1_01530C_A:C1_02090C_A:ABP140:CWH8:C1_02380C_A:C1_02390W_A:C1_02450C_A:SNZ1:SNO1:PDE2:C1_02900C_A:TSR2:C1_03260W_A:C1_03370W_A:C1_03630W_A:RPP1:C1_03830C_A:C1_04040C_A:C1_04120C_A:ERB1:NOP4:C1_04510W_A:C1_04530C_A:C1_04710C_A:C1_04970W_A:RRP6:REI1:TRY2:ARO4:C1_05220C_A:C1_05270C_A:C1_05360C_A:C1_05380C_A:RSM22:RPS27A:FAL1:C1_05650W_A:RAD10:BUD16:C1_06540C_A:NOP6:C1_06760C_A:RIA1:YMC1:C1_07340W_A:C1_07470C_A:LHP1:C1_07510W_A:C1_07660W_A:ADE4:ECO1:ADE5,7:MRPL3:ERD1:C1_07950C_A:C1_07960W_A:FUN12:CAT8:MLT1:DIP2:SAM4:GCR3:UPC2:GCD6:C1_08630W_A:MSS116:C1_08890C_A:CEF1:C1_09390W_A:GUA1:C1_09610W_A:C1_09710C_A:C1_09790C_A:BUD31:PDC2:DBP3:MNN23:C1_10080W_A:FCY23:C1_10620W_A:APN2:RPA190:C1_10920W_A:C1_10950C_A:C1_10970W_A:C1_11000C_A:PET127:NEP1:KRR1:GAR1:C1_11790W_A:MUP1:C1_11900C_A:C1_11910W_A:TRP3:C1_12280C_A:CSI2:C1_12350W_A:WRS1:C1_12440W_A:C1_12570C_A:C1_12630C_A:C1_12670C_A:C1_12750C_A:C1_12760W_A:C1_12820C_A:REX3:RMP1:C1_13380W_A:MCU1:C1_13560W_A:C1_13820C_A:C1_14080W_A:C1_14330W_A:MPP10:FAV3:C2_00200W_A:C2_00280C_A:C2_00410C_A:C2_00820W_A:C2_00840W_A:C2_01070W_A:C2_01220W_A:C2_01240C_A:ESC4:C2_01420C_A:APT1:REX2:C2_01820C_A:PRP39:C2_01860C_A:C2_01870C_A:ARO3:C2_02120W_A:C2_02420C_A:UTP21:UTP22:PRS1:C2_02540W_A:C2_02630W_A:PDR17:MET1:BNA31:PRP3:SCH9:C2_04120C_A:TBF1:SUV3:RIM2:C2_04570W_A:SNU114:C2_04700C_A:C2_04820W_A:PZF1:CKA2:C2_04990W_A:C2_05050C_A:C2_05080C_A:MAK5:C2_05160C_A:CDC21:RPF2:RPC40:KTI11:C2_05670C_A:C2_05750W_A:C2_05830C_A:C2_05840W_A:LAS1:CSM3:ECM17:SER1:MAK16:C2_06480W_A:C2_06520C_A:C2_06530W_A:VAS1:C2_06650C_A:C2_06660W_A:FHL1:C2_06850W_A:AAH1:NOC4:C2_07360W_A:RCL1:PRS5:C2_07920W_A:NSA1:C2_08180C_A:EAF3:RRP8:C2_08530C_A:MSU1:UTP15:MFG1:CDC47:C2_09160W_A:C2_09180W_A:MED18:C2_09310C_A:PES1:TAZ1:RRP15:C2_09500W_A:C2_09660W_A:SMM1:C2_09920W_A:CWC22:UBP8:PUS7:HEM3:C2_10740C_A:RPC11:C2_10810W_A:RPS24:C3_00100W_A:ULP3:UTP8:BUD21:SOF1:C3_00660W_A:C3_00830C_A:BMS1:ARP9:UTP20:NCE103:C3_01310W_A:URA3:RKI1:C3_01560W_A:SCM3:C3_02020W_A:C3_02040C_A:UTP4:ILV2:C3_02350W_A:PHA2:DDC1:C3_02670W_A:SMC3:C3_02750W_A:C3_02840W_A:C3_02850C_A:C3_03070W_A:C3_03110W_A:C3_03330C_A:YAH1:HBR3:RAD53:C3_04370C_A:C3_04380C_A:ADE2:MAK21:ARV1:SFP1:NOP14:CEM1:C3_05140C_A:C3_05160C_A:WOR2:RPL8B:C3_05280C_A:C3_05380W_A:C3_05440C_A:QDR2:C3_05800W_A:C3_05860C_A:TRM12:C3_05900W_A:CTA4:NOG1:C3_06150W_A:MMS21:C3_06270C_A:DOT1:ISW2:C3_06370C_A:NSA2:C3_06630W_A:PRP5:NOP13:C3_07400W_A:C3_07550C_A:C3_07570C_A:RIT1:RAD4:UTP9:C3_07800C_A:DUR4:RLP24:NMD5:C4_00690C_A:C4_00700C

						_A:C4_00740W_A:MLH1:C4_00800W_A:C4_00810C_A:C4_00880W_A:ARO1:C4_00940W_A:RAT1:C4_01280C_A:PWP1:C4_01500W_A:TOA2:ZCF25:C4_02400C_A:SLX4:HIT1:C4_02770C_A:SAS10:RPC53:HCA4:C4_02850W_A:ZUO1:C4_02880C_A:NAN1:C4_03140C_A:C4_03170W_A:DAO2:RAD2:C4_03720C_A:C4_03730C_A:C4_03740W_A:NUP84:C4_03830W_A:RRP42:C4_03870C_A:RAM1:ECM1:DUO1:C4_04520W_A:SSZ1:BAS1:C4_04810C_A:AGP3:RPL30:C4_05230C_A:C4_05260W_A:C4_05360C_A:OFD1:C4_05650W_A:TRP5:C4_06210C_A:C4_06410W_A:NOP1:C4_06790W_A:C4_06950W_A:C4_07060W_A:TRP4:C4_07150W_A:C5_00260W_A:C5_00280C_A:DUS4:MRP17:EXO1:RMT2:C5_00920W_A:BUD23:CYC3:C5_01140C_A:PUS4:C5_01430C_A:FYV5:SPB4:C5_01610W_A:CCN1:C5_01930W_A:C5_02010C_A:C5_02070C_A:RIX7:PDE1:C5_02440C_A:TIF5:SPR28:UTP13:BUR2:CKB2:MSW1:C5_03010W_A:RMS1:PCL1:RPO26:FUR1:C5_03400C_A:C5_03840W_A:HAM1:C5_03920C_A:C5_04120C_A:SAH1:URA7:MSM1:C5_04720C_A:HYS2:HAS1:C5_04840C_A:C5_04910W_A:GRF10:C5_05340W_A:DNA2:NOP5:C6_00530C_A:FCA1:C6_00640C_A:CDC14:TOP1:C6_01040C_A:C6_01120C_A:CIC1:EBP1:RAD18:C6_01890C_A:SWD3:C6_02230W_A:ALG11:C6_02290C_A:C6_02300C_A:C6_02350C_A:C6_02430W_A:PRP45:C6_02690C_A:MRT4:C6_02900C_A:POP4:C6_03210C_A:BMT4:ARP4:C6_03380W_A:C6_03390W_A:C6_03440W_A:NAM2:PAD1:SPB1:C6_04240W_A:NOP8:ILS1:C6_04530C_A:C7_00160C_A:C7_00220W_A:C7_00330C_A:DCC1:PSF3:THG1:RPA135:NOP15:C7_01030C_A:DBP7:C7_01210C_A:C7_01270C_A:PSF1:C7_01360C_A:C7_01400C_A:GIR2:FLU1:C7_01570C_A:SMC6:C7_01950W_A:LIG1:C7_02340C_A:C7_02460C_A:C7_02660C_A:C7_02930C_A:C7_03400C_A:ISY1:ENP2:C7_03590C_A:ENP1:C7_03850W_A:C7_03880C_A:C7_04150W_A:UTP18:MIS12:MMS22:RTT109:CR_00430C_A:CR_00460C_A:ADE1:BUD22:PWP2:CR_00830W_A:TRM1:DAL81:CTF18:CR_01260W_A:CR_01320C_A:CR_01410C_A:PRS:CR_01550C_A:TRP2:CR_01600C_A:MET6:CR_01670W_A:CDC60:CR_01700C_A:CR_01710W_A:CR_01780W_A:CR_01950W_A:CR_02030C_A:MCM6:ERG25:CR_02420W_A:CR_02550C_A:RRP9:RPC31:CR_02890C_A:SRP40:ARC1:SMC5:CR_03110W_A:CR_03200C_A:CR_03230W_A:CR_03340C_A:CR_03360W_A:KTI12:CR_03400W_A:CR_03440W_A:NCS2:YVH1:SGD1:SMC1:PRP42:CR_03940W_A:CR_04110W_A:CR_04160C_A:CR_04170W_A:QDR1:CR_04240C_A:CR_04300W_A:CR_04500C_A:CR_04560C_A:CR_04710W_A:ADE6:RPB8:CR_04920W_A:NOC2:CR_05550C_A:RNR3:MTG2:DBP8:RFX1:RPL7:CR_06230W_A:FGR50:CR_06450W_A:RRN11:CR_06680C_A:NMD3:CR_06840W_A:CR_06970C_A:POL2:MIS11:CR_07030C_A:URA2:CR_07310W_A:PIF1:FUN30:CR_07640C_A:ARO2:CDC45:DBP6:IMP4:CR_08000C_A:NOP10:SSB1:RIO2:ATS1:CR_08330W_A:CR_08410W_A:TSR1:CR_08940W_A:MPS1:SSF1:CR_09740W_A:MED21:CR_09800C_A:DPB2:SIK1:CR_10180W_A:UTP5:CR_10260W_A:CR_10410C_A:CR_10470C_A:MCD1:DRS1:LTV1:POP3

42273	ribosomal large subunit biogenesis	279 out of 1061 genes, 26.3%	546 out of 6473 background genes, 8.4%	1.39E-84	0.00%	RRS1:TRI1:C1_02090C_A:C1_02450C_A:PDE2:C1_02900C_A:RPP1:C1_03830C_A:C1_04040C_A:C1_04120C_A:ERB1:NOP4:C1_04510W_A:C1_04710C_A:C1_04970W_A:RRP6:REI1:TRY2:C1_05220C_A:C1_05360C_A:C1_05650W_A:C1_06540C_A:NOP6:C1_06760C_A:RIA1:C1_07510W_A:C1_07660W_A:ERD1:C1_07960W_A:GCR3:C1_08630W_A:JIP5:C1_09040C_A:CEF1:C1_09390W_A:YTM1:C1_09710C_A:PDC2:DBP3:FCY23:RPA190:C1_10920W_A:C1_10970W_A:C1_11000C_A:MUP1:C1_11900C_A:C1_11910W_A:TRP3:C1_12280C_A:CSI2:C1_12350W_A:C1_12440W_A:C1_12570C_A:C1_12630C_A:C1_12750C_A:C1_12760W_A:C1_12820C_A:RMP1:C1_13370W_A:C1_13380W_A:MCU1:C1_14080W_A:C2_00200W_A:C2_00280C_A:C2_00820W_A:C2_01070W_A:C2_01220W_A:C2_01820C_A:C2_01860C_A:C2_02120W_A:UTP22:C2_02540W_A:C2_02630W_A:PRP3:SCH9:C2_04120C_A:SUV3:C2_04570W_A:SNU114:C2_04700C_A:PZF1:CKA2:C2_05050C_A:MAK5:C2_05160C_A:RPF2:C2_05750W_A:C2_05830C_A:LAS1:ECM17:MAK16:C2_06520C_A:C2_06530W_A:C2_06660W_A:RPL11:C2_06850W_A:NSA1:C2_08180C_A:EAF3:RRP8:C2_08530C_A:MSU1:UTP15:MFG1:C2_09160W_A:C2_09180W_A:MED18:PES1:RRP15:C2_09660W_A:SMM1:C2_09920W_A:CWC22:C2_10740C_A:C3_00660W_A:C3_00830C_A:BMS1:ARP9:NCE103:C3_01310W_A:C3_01560W_A:C3_02040C_A:ILV2:C3_02350W_A:C3_02670W_A:SMC3:C3_02840W_A:C3_02850C_A:C3_03330C_A:C3_04370C_A:MAK21:ARV1:SFP1:NOP14:CEM1:C3_05160C_A:RPL8B:C3_05280C_A:C3_05380W_A:C3_05440C_A:QDR2:C3_05800W_A:C3_05860C_A:C3_05900W_A:NOG1:C3_06150W_A:C3_06370C_A:NSA2:PRP5:C3_07550C_A:C3_07570C_A:RAD4:DUR4:RLP24:NMD5:C4_00740W_A:RAT1:C4_01280C_A:SLX4:HIT1:C4_02880C_A:DAO2:C4_03720C_A:NUP84:C4_03830W_A:RAM1:AGP3:C4_05010W_A:C4_05230C_A:C4_05260W_A:C4_05360C_A:C4_06210C_A:C4_06410W_A:C4_06790W_A:C4_06950W_A:C4_07060W_A:RMT2:C5_00920W_A:C5_01430C_A:SPB4:C5_01930W_A:C5_02010C_A:RIX7:C5_02440C_A:SPR28:BUR2:CKB2:C5_03010W_A:RMS1:C5_03920C_A:C5_04120C_A:C5_04720C_A:HAS1:C5_04910W_A:GRF10:C5_05340W_A:C6_01120C_A:CIC1:C6_01890C_A:SWD3:C6_02230W_A:ALG11:C6_02430W_A:MRT4:C6_03210C_A:C6_03390W_A:C6_03440W_A:SPB1:C6_04240W_A:NOP8:C7_00160C_A:RPA135:NOP15:DBP7:C7_01360C_A:GIR2:FLU1:C7_01570C_A:LIG1:C7_02460C_A:C7_02660C_A:C7_02930C_A:C7_03400C_A:ISY1:ENP2:C7_03590C_A:ENP1:C7_03850W_A:C7_03880C_A:C7_04150W_A:UTP18:BUD22:PWP2:CR_00830W_A:DAL81:CR_01320C_A:CR_01410C_A:CR_01550C_A:CR_01600C_A:CR_01670W_A:CR_01700C_A:CR_02030C_A:CR_02890C_A:CR_03200C_A:CR_03360W_A:YVH1:SGD1:PRP42:CR_03940W_A:CR_04170W_A:CR_04500C_A:CR_04710W_A:NOC2:CR_05550C_A:RNR3:SDA1:RPL7:CR_06230W_A:CR_06450W_A:RRN11:CR_06680C_A:CR_06970C_A:PIF1:CR_07640C_A:DBP6:CR_08000C_A:CR_08330W_A:CR_08410W_A:TSR1:CR_08500W_A:ELF1:SSF1:CR_09740W_A:MED21:CR_09800C_A:CR_10260W_A:CR_10470C_A:DRS1:LTV1

10467	gene expression	602 out of 1061 genes, 56.7%	1951 out of 6473 background genes, 30.1%	1.63E-84	0.00%	RIM8:HBR1:CNS1:RPO41:NRM1:RRS1:TRI1:HMT1:C1_01150C_A:C1_01160C_A:TRM2:C1_01530C_A:C1_02090C_A:ABP140:C1_02380C_A:C1_02450C_A:HOM6:PDE2:MIA40:C1_02900C_A:TSR2:C1_03370W_A:C1_03540C_A:C1_03630W_A:C1_03790C_A:RPP1:C1_03830C_A:C1_04040C_A:C1_04120C_A:ERB1:NOP4:C1_04510W_A:C1_04530C_A:C1_04710C_A:MRPL37:C1_04970W_A:RRP6:REI1:TRY2:C1_05220C_A:C1_05270C_A:C1_05360C_A:C1_05380C_A:RSM22:RPS27A:C1_05630C_A:FAL1:C1_05650W_A:C1_06530C_A:C1_06540C_A:NOP6:C1_06760C_A:RIA1:C1_07340W_A:C1_07470C_A:LHP1:C1_07510W_A:C1_07660W_A:MRPL3:ERD1:C1_07950C_A:C1_07960W_A:FUN12:DRG1:CAT8:MLT1:DIP2:GCR3:C1_08520C_A:GCD6:C1_08630W_A:MSS116:C1_08890C_A:CEF1:C1_09390W_A:C1_09710C_A:C1_09790C_A:BUD31:C1_09910C_A:PDC2:DBP3:MNN23:C1_10080W_A:C1_10200C_A:FCY23:C1_10620W_A:RPA190:C1_10690W_A:C1_10920W_A:C1_10950C_A:C1_10970W_A:C1_11000C_A:PET127:FGR39:NEP1:KRR1:SAM2:MUP1:C1_11880W_A:C1_11900C_A:C1_11910W_A:SER33:TRP3:C1_12280C_A:CSI2:MRPS9:C1_12350W_A:WRS1:C1_12440W_A:C1_12570C_A:C1_12630C_A:C1_12670C_A:C1_12750C_A:C1_12760W_A:FEN1:C1_12820C_A:RPL14:C1_13060C_A:REX3:RMP1:C1_13380W_A:MCU1:C1_13820C_A:C1_14080W_A:C1_14330W_A:MPP10:FAV3:C2_00200W_A:C2_00280C_A:C2_00410C_A:C2_00820W_A:C2_00840W_A:C2_01070W_A:C2_01220W_A:C2_01420C_A:REX2:C2_01740C_A:C2_01820C_A:PRP39:C2_01860C_A:C2_01870C_A:C2_02120W_A:C2_02270C_A:C2_02420C_A:UTP21:UTP22:PRS1:C2_02540W_A:C2_02630W_A:PDR17:C2_02930C_A:MET1:SMP3:PRP3:C2_03560C_A:SCH9:C2_03950W_A:C2_04120C_A:TBF1:SUV3:RIM2:C2_04570W_A:SNU114:C2_04700C_A:C2_04820W_A:PZF1:CKA2:C2_05050C_A:C2_05080C_A:MAK5:C2_05160C_A:RPF2:C2_05270W_A:RPC40:KTI11:C2_05670C_A:C2_05750W_A:C2_05830C_A:C2_05840W_A:IDP1:LAS1:ECM17:SER1:MAK16:C2_06480W_A:C2_06520C_A:C2_06530W_A:VAS1:C2_06650C_A:C2_06660W_A:RPL11:FHL1:C2_06850W_A:C2_06950C_A:SPE3:NOC4:C2_07360W_A:RCL1:C2_07680W_A:PHO86:PRS5:C2_07920W_A:NSA1:C2_08180C_A:EAF3:RRP8:C2_08530C_A:MSU1:UTP15:MFG1:C2_09160W_A:C2_09180W_A:MED18:C2_09310C_A:PES1:TAZ1:RRP15:C2_09500W_A:C2_09660W_A:SMM1:C2_09920W_A:CWC22:UBP8:PUS7:HEM3:C2_10740C_A:RPC11:C2_10810W_A:RPS24:C3_00100W_A:ULP3:UTP8:BUD21:SOF1:C3_00660W_A:C3_00830C_A:BMS1:ARP9:UTP20:NCE103:C3_01310W_A:MET18:RPS7A:C3_01520C_A:C3_01560W_A:C3_02020W_A:C3_02040C_A:UTP4:C3_02180C_A:ILV2:C3_02350W_A:C3_02670W_A:C3_02750W_A:C3_02840W_A:C3_02850C_A:C3_03070W_A:C3_03110W_A:C3_03330C_A:C3_03560W_A:HBR3:C3_04370C_A:C3_04380C_A:MAK21:RPS15:RPP2B:ARV1:SFP1:NOP14:CEM1:C3_05140C_A:C3_05160C_A:RPL8B:C3_05280C_A:C3_05380W_A:C3_05440C_A:QDR2:C3_05800W_A:C3_05860C_A:TRM12:C3_05900W_A:CTA4:NOG1:C3_06150W_A:C3_06240C_A:DOT1:ISW2:C3_06370C_A:NSA2:PRP5:C3_06830C_A:RPS12:NOP13:C3_

						07400W_A:C3_07550C_A:C3_07570C_A:RIT1:RAD4:UTP9:C3_07800C_A:DUR4:RLP24:NMD5:HIS5:C4_00690C_A:C4_00740W_A:C4_00800W_A:C4_00810C_A:C4_00880W_A:ARO1:C4_00940W_A:RAT1:C4_01280C_A:PGA53:PWP1:NIP1:C4_01500W_A:TOA2:HSX11:TIM10:ZCF25:C4_02400C_A:SLX4:HIT1:SAS10:RPC53:HCA4:C4_02850W_A:ZUO1:C4_02880C_A:NAN1:C4_03140C_A:C4_03170W_A:DAO2:C4_03410W_A:RAD2:C4_03720C_A:C4_03730C_A:C4_03740W_A:NUP84:C4_03830W_A:RRP42:RAM1:ECM1:C4_04130W_A:DUO1:C4_04390W_A:C4_04520W_A:SSZ1:BAS1:C4_04810C_A:AGP3:RPL30:C4_05230C_A:C4_05260W_A:C4_05360C_A:OFD1:C4_05650W_A:C4_06210C_A:C4_06410W_A:NOP1:C4_06790W_A:C4_06950W_A:C4_07060W_A:C4_07140W_A:C4_07150W_A:C5_00030W_A:C5_00280C_A:DUS4:C5_00320W_A:MRP17:MET14:RMT2:C5_00820W_A:C5_00920W_A:BUD23:CYC3:C5_01140C_A:PUS4:C5_01430C_A:FYV5:SPB4:C5_01610W_A:C5_01700W_A:C5_01930W_A:C5_02010C_A:C5_02070C_A:RIX7:C5_02440C_A:TIF5:SPR28:UTP13:C5_02590C_A:BUR2:CKB2:MSW1:C5_02780W_A:C5_02820C_A:C5_03010W_A:RMS1:CAM1-1:C5_03290C_A:RPO26:FUR1:C5_03400C_A:C5_03530C_A:C5_03550W_A:MDJ1:C5_03920C_A:C5_04120C_A:C5_04290C_A:C5_04340W_A:CSU57:MSM1:HAS1:C5_04840C_A:C5_04910W_A:GRF10:C5_05250C_A:SDH4:C5_05340W_A:NOP5:C6_00530C_A:C6_00640C_A:CDC14:TOP1:C6_01040C_A:C6_01120C_A:CIC1:PMP1:TIF3:HCH1:C6_01890C_A:C6_01980C_A:SWD3:C6_02230W_A:RPL10A:ALG11:C6_02290C_A:C6_02300C_A:C6_02350C_A:C6_02370C_A:C6_02430W_A:PRP45:C6_02690C_A:MRT4:C6_02900C_A:POP4:C6_03210C_A:C6_03380W_A:C6_03390W_A:C6_03440W_A:NAM2:SPB1:C6_04240W_A:NOP8:ILS1:C6_04530C_A:C7_00160C_A:C7_00220W_A:C7_00330C_A:THG1:RPA135:NOP15:YML6:C7_01020C_A:C7_01030C_A:DBP7:C7_01210C_A:ASC1:C7_01270C_A:C7_01360C_A:C7_01400C_A:GIR2:PRT1:FLU1:C7_01570C_A:C7_01600W_A:C7_01950W_A:LIG1:C7_02340C_A:C7_02460C_A:C7_02660C_A:C7_02930C_A:IFM1:C7_03400C_A:ISY1:ENP2:C7_03590C_A:ENP1:HIS7:MRP7:C7_03850W_A:C7_03880C_A:C7_04150W_A:UTP18:CR_00430C_A:CR_00460C_A:MRPL6:ADE1:BUD22:PWP2:CR_00830W_A:TRM1:DAL81:CR_01320C_A:CR_01410C_A:PRS:CR_01550C_A:CR_01600C_A:CR_01670W_A:CDC60:CR_01700C_A:CR_01710W_A:CR_01780W_A:CR_01950W_A:CR_02030C_A:MCM6:ERG25:CR_02420W_A:CR_02430C_A:CR_02550C_A:RRP9:RPC31:CR_02890C_A:SRP40:ARC1:SMC5:CR_03110W_A:CR_03200C_A:CR_03230W_A:CR_03360W_A:KTI12:MTG1:CR_03400W_A:NCS2:YVH1:SMC1:PRP42:CR_03940W_A:MEX67:CR_04110W_A:CR_04160C_A:CR_04170W_A:CR_04240C_A:CR_04300W_A:CR_04500C_A:CR_04710W_A:RPB8:CR_04920W_A:NOC2:CR_05550C_A:RNR3:MTG2:DBP8:RFX1:RPL7:CR_06230W_A:FGR50:CR_06450W_A:RRN11:HSP60:CR_06680C_A:NMD3:CR_06840W_A:CR_06970C_A:CR_06980W_A:POL2:CR_07030C_A:URA2:CR_07310W_A:PIF1:FUN30:CR_07640C_A:CDC45:DBP6:IMP4:CR_08000C_A:NOP10:SSB1:RIO2:ATS1:CR_08330W_A:RPP2A:GSH

						2:CR_08410W_A:TSR1:CR_08940W_A:MPS1:ELF1:SSF1:CR_09740W_A:MED21:CR_09800C_A:DPB2:SIK1:UTP5:CR_10260W_A:CR_10410C_A:CR_10470C_A:DRS1:LTV1:POP3

44249	cellular biosynthetic process	694 out of 1061 genes, 65.4%	2485 out of 6473 background genes, 38.4%	9.94E-82	0.00%	RIM8:LEU4:HBR1:C1_00510W_A:CNS1:RPO41:SPE1:NRM1:RRS1:TRI1:HMT1:C1_01150C_A:C1_01160C_A:TRM2:ABC1:C1_01530C_A:CYS4:C1_02090C_A:ABP140:CWH8:C1_02380C_A:C1_02450C_A:SNZ1:SNO1:HOM6:LYS2:PDE2:MIA40:C1_02900C_A:C1_02970W_A:TSR2:C1_03370W_A:C1_03540C_A:C1_03630W_A:C1_03790C_A:RPP1:C1_03830C_A:KTR4:C1_04040C_A:C1_04120C_A:ERB1:NOP4:C1_04510W_A:C1_04530C_A:C1_04710C_A:MRPL37:C1_04970W_A:RHD1:ARG2:RRP6:REI1:TRY2:ARO4:C1_05220C_A:C1_05270C_A:C1_05360C_A:C1_05380C_A:RSM22:RPS27A:C1_05630C_A:FAL1:C1_05650W_A:RAD10:C1_05990C_A:BUD16:C1_06530C_A:C1_06540C_A:NOP6:C1_06760C_A:RIA1:YMC1:C1_07340W_A:C1_07470C_A:LHP1:C1_07510W_A:C1_07660W_A:ADE4:GCS1:ADE5,7:MRPL3:ERD1:C1_07950C_A:C1_07960W_A:FUN12:DRG1:CAT8:MLT1:DIP2:SAM4:GCR3:C1_08520C_A:GCD6:C1_08630W_A:MSS116:C1_08890C_A:CEF1:C1_09390W_A:GUA1:C1_09710C_A:C1_09790C_A:BUD31:C1_09910C_A:PDC2:DBP3:MNN23:C1_10080W_A:C1_10200C_A:C1_10340W_A:FCY23:C1_10620W_A:RPA190:C1_10690W_A:C1_10920W_A:C1_10950C_A:C1_10970W_A:C1_11000C_A:PET127:FGR39:NEP1:KRR1:SAM2:C1_11610C_A:C1_11790W_A:MUP1:C1_11880W_A:C1_11900C_A:C1_11910W_A:SER33:TRP3:C1_12280C_A:CSI2:MRPS9:C1_12350W_A:WRS1:C1_12440W_A:C1_12570C_A:C1_12630C_A:C1_12670C_A:C1_12750C_A:C1_12760W_A:FEN1:C1_12820C_A:RPL14:C1_13060C_A:REX3:C1_13330C_A:RMP1:C1_13380W_A:AGC1:MCU1:C1_13820C_A:C1_14080W_A:C1_14330W_A:MPP10:FAV3:C2_00200W_A:C2_00280C_A:C2_00390C_A:C2_00410C_A:C2_00820W_A:C2_00840W_A:MXR1:C2_01070W_A:C2_01220W_A:C2_01420C_A:APT1:REX2:C2_01740C_A:C2_01820C_A:PRP39:C2_01860C_A:C2_01870C_A:ARO3:C2_02120W_A:C2_02270C_A:C2_02420C_A:UTP21:UTP22:PRS1:C2_02540W_A:C2_02630W_A:PDR17:SER2:C2_02930C_A:MET1:BNA31:SMP3:PRP3:C2_03560C_A:MNN42:GPI13:SCH9:C2_03950W_A:C2_04120C_A:TBF1:BAT21:SUV3:RIM2:C2_04570W_A:SNU114:C2_04700C_A:C2_04820W_A:PZF1:CKA2:C2_05050C_A:C2_05080C_A:MAK5:C2_05160C_A:CDC21:RPF2:C2_05270W_A:RPC40:KTI11:C2_05670C_A:C2_05750W_A:C2_05830C_A:C2_05840W_A:IDP1:LAS1:ECM17:ILV1:SER1:MAK16:C2_06480W_A:C2_06520C_A:C2_06530W_A:VAS1:C2_06650C_A:C2_06660W_A:RPL11:FHL1:C2_06850W_A:C2_06950C_A:SPE3:AAH1:NOC4:C2_07360W_A:C2_07410W_A:RCL1:C2_07680W_A:PHO86:PRS5:C2_07920W_A:NSA1:C2_08180C_A:EAF3:RRP8:C2_08530C_A:MSU1:UTP15:MFG1:ASN1:C2_09160W_A:C2_09180W_A:MED18:C2_09310C_A:PES1:TAZ1:RRP15:C2_09500W_A:C2_09660W_A:LEU42:SMM1:C2_09920W_A:CWC22:UBP8:PUS7:HEM3:C2_10740C_A:RPC11:C2_10810W_A:RPS24:C3_00100W_A:ULP3:UTP8:BUD21:SOF1:C3_00660W_A:C3_00830C_A:BMS1:ARP9:UTP20:NCE103:C3_01310W_A:URA3:MET18:RKI1:RPS7A:C3_01520C_A:C3_01560W_A:C3_02020W_A:C3_02040C_A:UTP4:C3_02180C_A:ILV2:C3_02350W_A:PHA2:C3_02670W_A:C3_02750W_A:C3_02840W_A:C3_02850C_A:MET13:C3_03070W_A:C3_0311

						0W_A:C3_03330C_A:YAH1:C3_03560W_A:HBR3:RAD53:C3_04370C_A:C3_04380C_A:ADE2:MAK21:RPS15:RPP2B:ARV1:SFP1:NOP14:CEM1:C3_05140C_A:C3_05160C_A:RPL8B:C3_05280C_A:C3_05380W_A:GPI1:C3_05440C_A:QDR2:C3_05800W_A:C3_05860C_A:TRM12:C3_05900W_A:CTA4:NOG1:C3_06150W_A:C3_06240C_A:DOT1:ISW2:C3_06370C_A:NSA2:OPI3:PRP5:C3_06830C_A:RPS12:NOP13:C3_07400W_A:C3_07550C_A:C3_07570C_A:RIT1:RAD4:UTP9:C3_07800C_A:HIS4:MET15:DUR4:RLP24:NMD5:HIS5:C4_00690C_A:C4_00740W_A:C4_00800W_A:C4_00810C_A:C4_00880W_A:ARO1:C4_00940W_A:PTC8:RAT1:C4_01280C_A:PGA53:ILV6:PWP1:NIP1:C4_01500W_A:TOA2:HSX11:TIM10:ZCF25:C4_02400C_A:SLX4:HIT1:SAS10:RPC53:HCA4:C4_02850W_A:ZUO1:C4_02880C_A:NAN1:C4_03140C_A:C4_03170W_A:DAO2:C4_03410W_A:RAD2:C4_03720C_A:C4_03730C_A:C4_03740W_A:NUP84:C4_03830W_A:RRP42:RAM1:ECM1:C4_04130W_A:DUO1:C4_04390W_A:C4_04520W_A:SSZ1:BAS1:C4_04810C_A:AGP3:RPL30:C4_05230C_A:C4_05260W_A:C4_05360C_A:OFD1:HOM3:C4_05650W_A:TRP5:C4_06210C_A:C4_06410W_A:NOP1:C4_06790W_A:C4_06950W_A:MET16:C4_07060W_A:TRP4:C4_07140W_A:C4_07150W_A:C5_00030W_A:C5_00260W_A:C5_00280C_A:DUS4:C5_00320W_A:MRP17:MET14:RMT2:SPE2:C5_00820W_A:C5_00920W_A:BUD23:CYC3:C5_01140C_A:PUS4:C5_01430C_A:FYV5:SPB4:C5_01610W_A:CCN1:C5_01700W_A:C5_01930W_A:C5_02010C_A:C5_02070C_A:RIX7:THR4:C5_02440C_A:TIF5:SPR28:UTP13:C5_02590C_A:BUR2:CKB2:MSW1:C5_02780W_A:C5_02820C_A:C5_03010W_A:RMS1:CAM1-1:C5_03290C_A:PCL1:RPO26:FUR1:C5_03400C_A:C5_03460C_A:C5_03530C_A:C5_03550W_A:MDJ1:C5_03840W_A:C5_03920C_A:C5_03970W_A:C5_04120C_A:SAH1:C5_04290C_A:C5_04340W_A:URA7:CSU57:MSM1:C5_04720C_A:HYS2:HAS1:C5_04840C_A:C5_04910W_A:GRF10:C5_05250C_A:SDH4:HIS1:C5_05340W_A:ILV3:NOP5:C6_00530C_A:C6_00560W_A:FCA1:C6_00640C_A:CDC14:TOP1:HAL22:C6_01040C_A:C6_01120C_A:CIC1:PMP1:TIF3:RAD18:HCH1:C6_01890C_A:C6_01980C_A:SWD3:C6_02230W_A:RPL10A:ALG11:C6_02280W_A:C6_02290C_A:C6_02300C_A:C6_02350C_A:C6_02370C_A:C6_02430W_A:PRP45:C6_02690C_A:MRT4:C6_02900C_A:POP4:C6_03210C_A:C6_03380W_A:C6_03390W_A:C6_03440W_A:NAM2:SPB1:C6_04240W_A:NOP8:ILS1:C6_04530C_A:C7_00160C_A:C7_00220W_A:C7_00330C_A:THG1:RPA135:NOP15:YML6:C7_01020C_A:C7_01030C_A:DBP7:C7_01210C_A:ASC1:C7_01270C_A:C7_01360C_A:C7_01400C_A:GIR2:PRT1:FLU1:C7_01570C_A:C7_01600W_A:C7_01950W_A:LIG1:C7_02340C_A:C7_02460C_A:C7_02660C_A:C7_02930C_A:IFM1:C7_03400C_A:ISY1:ENP2:ARG4:C7_03590C_A:ENP1:HIS7:MRP7:C7_03850W_A:C7_03880C_A:C7_04150W_A:UTP18:MIS12:CR_00430C_A:CR_00460C_A:MRPL6:ADE1:BUD22:PWP2:CR_00830W_A:TRM1:DAL81:CR_01260W_A:CR_01320C_A:CR_01410C_A:PRS:CR_01550C_A:TRP2:CR_01600C_A:MET6:CR_01670W_A:CDC60:CR_01700C_A:CR_01710W_A:CR_01780W_A:CR_01950W_A:CR_02030C_A:MCM6:MET

						2:ERG25:CR_02420W_A:CR_02430C_A:CHO2:CR_02550C_A:PAN6:RRP9:RPC31:CR_02890C_A:SRP40:ARC1:SMC5:CR_03110W_A:CR_03200C_A:CR_03230W_A:CR_03360W_A:KTI12:MTG1:CR_03400W_A:NCS2:YVH1:SMC1:PRP42:CR_03940W_A:MEX67:CR_04110W_A:CR_04160C_A:CR_04170W_A:CR_04240C_A:CR_04300W_A:CR_04500C_A:CR_04710W_A:ADE6:RPB8:CR_04920W_A:NOC2:CR_05550C_A:RNR3:MTG2:DBP8:RFX1:RPL7:CR_06230W_A:FGR50:CR_06450W_A:RRN11:HSP60:CR_06680C_A:NMD3:CR_06840W_A:CR_06970C_A:CR_06980W_A:POL2:MIS11:CR_07030C_A:URA2:CR_07310W_A:PIF1:FUN30:CR_07640C_A:ARO2:CDC45:DBP6:IMP4:CR_08000C_A:NOP10:SSB1:RIO2:ATS1:CR_08330W_A:CYS3:RPP2A:GSH2:CR_08410W_A:TSR1:CR_08940W_A:MPS1:ELF1:SSF1:CR_09740W_A:MED21:CR_09800C_A:DPB2:SIK1:UTP5:CR_10260W_A:CR_10410C_A:CR_10470C_A:DRS1:PRO1:LTV1:POP3

479	endonucleolytic cleavage of tricistronic rRNA transcript (SSU-rRNA, 5.8S rRNA, LSU-rRNA)	253 out of 1061 genes, 23.8%	473 out of 6473 background genes, 7.3%	2.63E-81	0.00%	HBR1:CNS1:RPO41:RRS1:C1_02090C_A:C1_02900C_A:C1_03630W_A:RPP1:C1_03830C_A:C1_04040C_A:C1_04120C_A:NOP4:C1_04510W_A:REI1:TRY2:C1_05360C_A:C1_05380C_A:RSM22:C1_05650W_A:C1_06540C_A:NOP6:RIA1:C1_07660W_A:C1_07960W_A:MLT1:DIP2:GCD6:C1_08630W_A:MSS116:CEF1:C1_09390W_A:C1_09710C_A:C1_09790C_A:PDC2:DBP3:MNN23:FCY23:C1_10950C_A:C1_11000C_A:NEP1:KRR1:MUP1:C1_11900C_A:CSI2:C1_12350W_A:C1_12440W_A:C1_12570C_A:C1_12630C_A:C1_12750C_A:C1_12820C_A:RMP1:C1_13380W_A:C1_13820C_A:C1_14080W_A:C1_14330W_A:MPP10:FAV3:C2_00200W_A:C2_00280C_A:C2_00410C_A:C2_00820W_A:C2_01220W_A:C2_01820C_A:C2_02120W_A:C2_02420C_A:C2_02540W_A:C2_02630W_A:MET1:PRP3:SCH9:C2_04120C_A:TBF1:SUV3:C2_04700C_A:PZF1:CKA2:C2_05080C_A:MAK5:C2_05160C_A:C2_05830C_A:LAS1:MAK16:C2_06520C_A:C2_06660W_A:FHL1:C2_06850W_A:NOC4:RCL1:PRS5:C2_07920W_A:NSA1:C2_08180C_A:RRP8:C2_08530C_A:MSU1:RRP15:SMM1:C2_09920W_A:CWC22:C2_10740C_A:C2_10810W_A:C3_00100W_A:BUD21:C3_00660W_A:BMS1:UTP20:NCE103:C3_01310W_A:C3_02020W_A:C3_02040C_A:C3_02350W_A:C3_02670W_A:C3_02840W_A:C3_02850C_A:C3_03330C_A:HBR3:C3_04370C_A:MAK21:SFP1:NOP14:CEM1:C3_05160C_A:QDR2:C3_05800W_A:C3_06150W_A:ISW2:C3_06370C_A:NSA2:PRP5:C3_07550C_A:RAD4:C3_07800C_A:DUR4:RLP24:NMD5:C4_00690C_A:C4_00740W_A:RAT1:C4_01280C_A:C4_01500W_A:SLX4:HIT1:SAS10:RPC53:HCA4:C4_02880C_A:C4_03140C_A:C4_03720C_A:NUP84:C4_03830W_A:ECM1:C4_04520W_A:BAS1:C4_05260W_A:C4_05650W_A:C4_06790W_A:C4_07060W_A:C4_07150W_A:C5_00920W_A:BUD23:CYC3:C5_01430C_A:FYV5:SPB4:C5_02010C_A:C5_02070C_A:RIX7:C5_02440C_A:SPR28:UTP13:C5_03010W_A:RMS1:C5_03920C_A:C5_04120C_A:C5_04840C_A:C5_04910W_A:GRF10:C5_05340W_A:NOP5:C6_02230W_A:ALG11:C6_02430W_A:C6_02900C_A:POP4:C6_03440W_A:C6_04240W_A:NOP8:C7_00160C_A:C7_00220W_A:C7_00330C_A:DBP7:C7_01360C_A:C7_01950W_A:LIG1:C7_02460C_A:C7_02930C_A:C7_03400C_A:ISY1:C7_03590C_A:ENP1:C7_03850W_A:C7_03880C_A:C7_04150W_A:UTP18:CR_00460C_A:PWP2:CR_00830W_A:DAL81:CR_01410C_A:CR_01600C_A:CR_01700C_A:CR_02030C_A:CR_02420W_A:RRP9:CR_02890C_A:SRP40:SMC5:CR_03200C_A:CR_03360W_A:NCS2:YVH1:SMC1:CR_03940W_A:CR_04110W_A:CR_04710W_A:NOC2:CR_05550C_A:RNR3:DBP8:RFX1:CR_06230W_A:FGR50:CR_06450W_A:RRN11:CR_06680C_A:POL2:PIF1:FUN30:DBP6:IMP4:CR_08000C_A:RIO2:ATS1:CR_08330W_A:CR_08410W_A:TSR1:MPS1:CR_09740W_A:CR_09800C_A:CR_10260W_A:CR_10410C_A:DRS1:POP3

966	RNA 5'-end processing	236 out of 1061 genes, 22.2%	421 out of 6473 background genes, 6.5%	8.14E-81	0.00%	HBR1:CNS1:RPO41:C1_02090C_A:C1_02900C_A:RPP1:C1_03830C_A:C1_04040C_A:C1_04120C_A:NOP4:C1_04510W_A:REI1:TRY2:C1_05360C_A:C1_05380C_A:RSM22:C1_05650W_A:C1_06540C_A:NOP6:RIA1:C1_07660W_A:C1_07960W_A:MLT1:DIP2:C1_08630W_A:MSS116:CEF1:C1_09390W_A:C1_09710C_A:C1_09790C_A:FCY23:C1_10950C_A:C1_11000C_A:PET127:NEP1:MUP1:C1_11900C_A:CSI2:C1_12350W_A:C1_12440W_A:C1_12570C_A:C1_12630C_A:C1_12750C_A:C1_12820C_A:RMP1:C1_13380W_A:C1_13820C_A:C1_14080W_A:C1_14330W_A:MPP10:FAV3:C2_00200W_A:C2_00280C_A:C2_00410C_A:C2_00820W_A:C2_01820C_A:C2_02120W_A:C2_02420C_A:C2_02540W_A:C2_02630W_A:MET1:PRP3:SCH9:C2_04120C_A:TBF1:SUV3:C2_04700C_A:PZF1:CKA2:C2_05080C_A:MAK5:C2_05160C_A:C2_05830C_A:LAS1:MAK16:C2_06520C_A:C2_06660W_A:FHL1:C2_06850W_A:NOC4:RCL1:PRS5:C2_07920W_A:NSA1:C2_08180C_A:RRP8:MSU1:RRP15:SMM1:C2_09920W_A:CWC22:C2_10740C_A:C2_10810W_A:C3_00100W_A:BUD21:C3_00660W_A:BMS1:UTP20:NCE103:C3_01310W_A:C3_02020W_A:C3_02040C_A:C3_02670W_A:C3_02840W_A:C3_02850C_A:C3_03330C_A:HBR3:C3_04370C_A:MAK21:SFP1:NOP14:CEM1:C3_05160C_A:C3_05800W_A:C3_06150W_A:ISW2:C3_06370C_A:NSA2:PRP5:C3_07550C_A:RAD4:C3_07800C_A:DUR4:RLP24:NMD5:C4_00690C_A:C4_00740W_A:RAT1:C4_01280C_A:C4_01500W_A:SLX4:HIT1:SAS10:RPC53:HCA4:C4_02880C_A:C4_03140C_A:C4_03720C_A:C4_03830W_A:ECM1:C4_04520W_A:BAS1:C4_05260W_A:C4_05650W_A:C4_06790W_A:C4_07060W_A:C4_07150W_A:C5_00920W_A:CYC3:C5_01430C_A:FYV5:SPB4:C5_02010C_A:C5_02070C_A:RIX7:C5_02440C_A:SPR28:UTP13:C5_03010W_A:RMS1:C5_03920C_A:C5_04120C_A:C5_04840C_A:C5_04910W_A:GRF10:C5_05340W_A:NOP5:C6_02230W_A:ALG11:C6_02430W_A:C6_02900C_A:POP4:C6_03440W_A:C6_04240W_A:NOP8:C7_00160C_A:C7_00220W_A:C7_00330C_A:DBP7:C7_01360C_A:C7_01950W_A:C7_02460C_A:C7_02930C_A:C7_03400C_A:ISY1:C7_03590C_A:C7_03850W_A:C7_03880C_A:C7_04150W_A:UTP18:CR_00460C_A:PWP2:CR_00830W_A:DAL81:CR_01410C_A:CR_01600C_A:CR_01700C_A:CR_02030C_A:CR_02420W_A:RRP9:CR_02890C_A:SRP40:SMC5:CR_03200C_A:CR_03360W_A:NCS2:YVH1:CR_03940W_A:CR_04110W_A:CR_04710W_A:NOC2:CR_05550C_A:RNR3:DBP8:RFX1:RPL7:CR_06230W_A:FGR50:CR_06450W_A:RRN11:PIF1:FUN30:DBP6:IMP4:CR_08000C_A:RIO2:ATS1:CR_08330W_A:CR_08410W_A:MPS1:CR_09740W_A:CR_09800C_A:CR_10260W_A:CR_10410C_A:DRS1:POP3

967	rRNA 5'-end processing	235 out of 1061 genes, 22.1%	419 out of 6473 background genes, 6.5%	1.70E-80	0.00%	HBR1:CNS1:RPO41:C1_02090C_A:C1_02900C_A:RPP1:C1_03830C_A:C1_04040C_A:C1_04120C_A:NOP4:C1_04510W_A:REI1:TRY2:C1_05360C_A:C1_05380C_A:RSM22:C1_05650W_A:C1_06540C_A:NOP6:RIA1:C1_07660W_A:C1_07960W_A:MLT1:DIP2:C1_08630W_A:MSS116:CEF1:C1_09390W_A:C1_09710C_A:C1_09790C_A:FCY23:C1_10950C_A:C1_11000C_A:NEP1:MUP1:C1_11900C_A:CSI2:C1_12350W_A:C1_12440W_A:C1_12570C_A:C1_12630C_A:C1_12750C_A:C1_12820C_A:RMP1:C1_13380W_A:C1_13820C_A:C1_14080W_A:C1_14330W_A:MPP10:FAV3:C2_00200W_A:C2_00280C_A:C2_00410C_A:C2_00820W_A:C2_01820C_A:C2_02120W_A:C2_02420C_A:C2_02540W_A:C2_02630W_A:MET1:PRP3:SCH9:C2_04120C_A:TBF1:SUV3:C2_04700C_A:PZF1:CKA2:C2_05080C_A:MAK5:C2_05160C_A:C2_05830C_A:LAS1:MAK16:C2_06520C_A:C2_06660W_A:FHL1:C2_06850W_A:NOC4:RCL1:PRS5:C2_07920W_A:NSA1:C2_08180C_A:RRP8:MSU1:RRP15:SMM1:C2_09920W_A:CWC22:C2_10740C_A:C2_10810W_A:C3_00100W_A:BUD21:C3_00660W_A:BMS1:UTP20:NCE103:C3_01310W_A:C3_02020W_A:C3_02040C_A:C3_02670W_A:C3_02840W_A:C3_02850C_A:C3_03330C_A:HBR3:C3_04370C_A:MAK21:SFP1:NOP14:CEM1:C3_05160C_A:C3_05800W_A:C3_06150W_A:ISW2:C3_06370C_A:NSA2:PRP5:C3_07550C_A:RAD4:C3_07800C_A:DUR4:RLP24:NMD5:C4_00690C_A:C4_00740W_A:RAT1:C4_01280C_A:C4_01500W_A:SLX4:HIT1:SAS10:RPC53:HCA4:C4_02880C_A:C4_03140C_A:C4_03720C_A:C4_03830W_A:ECM1:C4_04520W_A:BAS1:C4_05260W_A:C4_05650W_A:C4_06790W_A:C4_07060W_A:C4_07150W_A:C5_00920W_A:CYC3:C5_01430C_A:FYV5:SPB4:C5_02010C_A:C5_02070C_A:RIX7:C5_02440C_A:SPR28:UTP13:C5_03010W_A:RMS1:C5_03920C_A:C5_04120C_A:C5_04840C_A:C5_04910W_A:GRF10:C5_05340W_A:NOP5:C6_02230W_A:ALG11:C6_02430W_A:C6_02900C_A:POP4:C6_03440W_A:C6_04240W_A:NOP8:C7_00160C_A:C7_00220W_A:C7_00330C_A:DBP7:C7_01360C_A:C7_01950W_A:C7_02460C_A:C7_02930C_A:C7_03400C_A:ISY1:C7_03590C_A:C7_03850W_A:C7_03880C_A:C7_04150W_A:UTP18:CR_00460C_A:PWP2:CR_00830W_A:DAL81:CR_01410C_A:CR_01600C_A:CR_01700C_A:CR_02030C_A:CR_02420W_A:RRP9:CR_02890C_A:SRP40:SMC5:CR_03200C_A:CR_03360W_A:NCS2:YVH1:CR_03940W_A:CR_04110W_A:CR_04710W_A:NOC2:CR_05550C_A:RNR3:DBP8:RFX1:RPL7:CR_06230W_A:FGR50:CR_06450W_A:RRN11:PIF1:FUN30:DBP6:IMP4:CR_08000C_A:RIO2:ATS1:CR_08330W_A:CR_08410W_A:MPS1:CR_09740W_A:CR_09800C_A:CR_10260W_A:CR_10410C_A:DRS1:POP3

34471	ncRNA 5'-end processing	235 out of 1061 genes, 22.1%	420 out of 6473 background genes, 6.5%	3.34E-80	0.00%	HBR1:CNS1:RPO41:C1_02090C_A:C1_02900C_A:RPP1:C1_03830C_A:C1_04040C_A:C1_04120C_A:NOP4:C1_04510W_A:REI1:TRY2:C1_05360C_A:C1_05380C_A:RSM22:C1_05650W_A:C1_06540C_A:NOP6:RIA1:C1_07660W_A:C1_07960W_A:MLT1:DIP2:C1_08630W_A:MSS116:CEF1:C1_09390W_A:C1_09710C_A:C1_09790C_A:FCY23:C1_10950C_A:C1_11000C_A:NEP1:MUP1:C1_11900C_A:CSI2:C1_12350W_A:C1_12440W_A:C1_12570C_A:C1_12630C_A:C1_12750C_A:C1_12820C_A:RMP1:C1_13380W_A:C1_13820C_A:C1_14080W_A:C1_14330W_A:MPP10:FAV3:C2_00200W_A:C2_00280C_A:C2_00410C_A:C2_00820W_A:C2_01820C_A:C2_02120W_A:C2_02420C_A:C2_02540W_A:C2_02630W_A:MET1:PRP3:SCH9:C2_04120C_A:TBF1:SUV3:C2_04700C_A:PZF1:CKA2:C2_05080C_A:MAK5:C2_05160C_A:C2_05830C_A:LAS1:MAK16:C2_06520C_A:C2_06660W_A:FHL1:C2_06850W_A:NOC4:RCL1:PRS5:C2_07920W_A:NSA1:C2_08180C_A:RRP8:MSU1:RRP15:SMM1:C2_09920W_A:CWC22:C2_10740C_A:C2_10810W_A:C3_00100W_A:BUD21:C3_00660W_A:BMS1:UTP20:NCE103:C3_01310W_A:C3_02020W_A:C3_02040C_A:C3_02670W_A:C3_02840W_A:C3_02850C_A:C3_03330C_A:HBR3:C3_04370C_A:MAK21:SFP1:NOP14:CEM1:C3_05160C_A:C3_05800W_A:C3_06150W_A:ISW2:C3_06370C_A:NSA2:PRP5:C3_07550C_A:RAD4:C3_07800C_A:DUR4:RLP24:NMD5:C4_00690C_A:C4_00740W_A:RAT1:C4_01280C_A:C4_01500W_A:SLX4:HIT1:SAS10:RPC53:HCA4:C4_02880C_A:C4_03140C_A:C4_03720C_A:C4_03830W_A:ECM1:C4_04520W_A:BAS1:C4_05260W_A:C4_05650W_A:C4_06790W_A:C4_07060W_A:C4_07150W_A:C5_00920W_A:CYC3:C5_01430C_A:FYV5:SPB4:C5_02010C_A:C5_02070C_A:RIX7:C5_02440C_A:SPR28:UTP13:C5_03010W_A:RMS1:C5_03920C_A:C5_04120C_A:C5_04840C_A:C5_04910W_A:GRF10:C5_05340W_A:NOP5:C6_02230W_A:ALG11:C6_02430W_A:C6_02900C_A:POP4:C6_03440W_A:C6_04240W_A:NOP8:C7_00160C_A:C7_00220W_A:C7_00330C_A:DBP7:C7_01360C_A:C7_01950W_A:C7_02460C_A:C7_02930C_A:C7_03400C_A:ISY1:C7_03590C_A:C7_03850W_A:C7_03880C_A:C7_04150W_A:UTP18:CR_00460C_A:PWP2:CR_00830W_A:DAL81:CR_01410C_A:CR_01600C_A:CR_01700C_A:CR_02030C_A:CR_02420W_A:RRP9:CR_02890C_A:SRP40:SMC5:CR_03200C_A:CR_03360W_A:NCS2:YVH1:CR_03940W_A:CR_04110W_A:CR_04710W_A:NOC2:CR_05550C_A:RNR3:DBP8:RFX1:RPL7:CR_06230W_A:FGR50:CR_06450W_A:RRN11:PIF1:FUN30:DBP6:IMP4:CR_08000C_A:RIO2:ATS1:CR_08330W_A:CR_08410W_A:MPS1:CR_09740W_A:CR_09800C_A:CR_10260W_A:CR_10410C_A:DRS1:POP3

472	endonucleolytic cleavage to generate mature 5'-end of SSU-rRNA from (SSU-rRNA, 5.8S rRNA, LSU-rRNA)	233 out of 1061 genes, 22.0%	417 out of 6473 background genes, 6.4%	2.87E-79	0.00%	HBR1:CNS1:RPO41:C1_02090C_A:C1_02900C_A:RPP1:C1_03830C_A:C1_04040C_A:C1_04120C_A:NOP4:C1_04510W_A:REI1:TRY2:C1_05360C_A:C1_05380C_A:RSM22:C1_05650W_A:C1_06540C_A:NOP6:RIA1:C1_07660W_A:C1_07960W_A:MLT1:DIP2:C1_08630W_A:MSS116:CEF1:C1_09390W_A:C1_09710C_A:C1_09790C_A:FCY23:C1_10950C_A:C1_11000C_A:NEP1:MUP1:C1_11900C_A:C1_12350W_A:C1_12440W_A:C1_12570C_A:C1_12630C_A:C1_12750C_A:C1_12820C_A:RMP1:C1_13380W_A:C1_13820C_A:C1_14080W_A:C1_14330W_A:MPP10:FAV3:C2_00200W_A:C2_00280C_A:C2_00410C_A:C2_00820W_A:C2_01820C_A:C2_02120W_A:C2_02420C_A:C2_02540W_A:C2_02630W_A:MET1:PRP3:SCH9:C2_04120C_A:TBF1:SUV3:C2_04700C_A:PZF1:CKA2:C2_05080C_A:MAK5:C2_05160C_A:C2_05830C_A:LAS1:MAK16:C2_06520C_A:C2_06660W_A:FHL1:C2_06850W_A:NOC4:RCL1:PRS5:C2_07920W_A:NSA1:C2_08180C_A:RRP8:MSU1:RRP15:SMM1:C2_09920W_A:CWC22:C2_10740C_A:C2_10810W_A:C3_00100W_A:BUD21:C3_00660W_A:BMS1:UTP20:NCE103:C3_01310W_A:C3_02020W_A:C3_02040C_A:C3_02670W_A:C3_02840W_A:C3_02850C_A:C3_03330C_A:HBR3:C3_04370C_A:MAK21:SFP1:NOP14:CEM1:C3_05160C_A:C3_05800W_A:C3_06150W_A:ISW2:C3_06370C_A:NSA2:PRP5:C3_07550C_A:RAD4:C3_07800C_A:DUR4:RLP24:NMD5:C4_00690C_A:C4_00740W_A:RAT1:C4_01280C_A:C4_01500W_A:SLX4:HIT1:SAS10:RPC53:HCA4:C4_02880C_A:C4_03140C_A:C4_03720C_A:C4_03830W_A:ECM1:C4_04520W_A:BAS1:C4_05260W_A:C4_05650W_A:C4_06790W_A:C4_07060W_A:C4_07150W_A:C5_00920W_A:CYC3:C5_01430C_A:FYV5:SPB4:C5_02010C_A:C5_02070C_A:RIX7:C5_02440C_A:SPR28:UTP13:C5_03010W_A:RMS1:C5_03920C_A:C5_04120C_A:C5_04840C_A:C5_04910W_A:GRF10:C5_05340W_A:NOP5:C6_02230W_A:ALG11:C6_02430W_A:C6_02900C_A:POP4:C6_03440W_A:C6_04240W_A:NOP8:C7_00160C_A:C7_00220W_A:C7_00330C_A:DBP7:C7_01360C_A:C7_01950W_A:C7_02460C_A:C7_02930C_A:C7_03400C_A:ISY1:C7_03590C_A:C7_03850W_A:C7_03880C_A:C7_04150W_A:UTP18:CR_00460C_A:PWP2:CR_00830W_A:DAL81:CR_01410C_A:CR_01600C_A:CR_01700C_A:CR_02030C_A:CR_02420W_A:RRP9:CR_02890C_A:SRP40:SMC5:CR_03200C_A:CR_03360W_A:NCS2:YVH1:CR_03940W_A:CR_04110W_A:CR_04710W_A:NOC2:CR_05550C_A:RNR3:DBP8:RFX1:CR_06230W_A:FGR50:CR_06450W_A:RRN11:PIF1:FUN30:DBP6:IMP4:CR_08000C_A:RIO2:ATS1:CR_08330W_A:CR_08410W_A:MPS1:CR_09740W_A:CR_09800C_A:CR_10260W_A:CR_10410C_A:DRS1:POP3

36260	RNA capping	236 out of 1061 genes, 22.2%	427 out of 6473 background genes, 6.6%	4.43E-79	0.00%	HBR1:CNS1:RPO41:C1_02090C_A:C1_02900C_A:RPP1:C1_03830C_A:C1_04040C_A:C1_04120C_A:NOP4:C1_04510W_A:REI1:TRY2:C1_05360C_A:C1_05380C_A:RSM22:C1_05650W_A:C1_06540C_A:NOP6:RIA1:C1_07660W_A:C1_07960W_A:MLT1:DIP2:C1_08630W_A:MSS116:CEF1:C1_09390W_A:C1_09710C_A:C1_09790C_A:FCY23:C1_10950C_A:C1_11000C_A:NEP1:MUP1:C1_11900C_A:CSI2:C1_12350W_A:C1_12440W_A:C1_12570C_A:C1_12630C_A:C1_12750C_A:C1_12820C_A:RMP1:C1_13380W_A:C1_13820C_A:C1_14080W_A:C1_14330W_A:MPP10:FAV3:C2_00200W_A:C2_00280C_A:C2_00410C_A:C2_00820W_A:C2_01820C_A:C2_02120W_A:C2_02420C_A:C2_02540W_A:C2_02630W_A:MET1:PRP3:SCH9:C2_04120C_A:TBF1:SUV3:C2_04700C_A:PZF1:CKA2:C2_05080C_A:MAK5:C2_05160C_A:C2_05830C_A:LAS1:MAK16:C2_06520C_A:C2_06660W_A:FHL1:C2_06850W_A:NOC4:C2_07360W_A:RCL1:PRS5:C2_07920W_A:NSA1:C2_08180C_A:RRP8:MSU1:RRP15:SMM1:C2_09920W_A:CWC22:C2_10740C_A:C2_10810W_A:C3_00100W_A:BUD21:C3_00660W_A:BMS1:UTP20:NCE103:C3_01310W_A:C3_02020W_A:C3_02040C_A:C3_02670W_A:C3_02840W_A:C3_02850C_A:C3_03330C_A:HBR3:C3_04370C_A:MAK21:SFP1:NOP14:CEM1:C3_05160C_A:C3_05800W_A:C3_06150W_A:ISW2:C3_06370C_A:NSA2:PRP5:C3_07550C_A:RAD4:C3_07800C_A:DUR4:RLP24:NMD5:C4_00690C_A:C4_00740W_A:RAT1:C4_01280C_A:C4_01500W_A:SLX4:HIT1:SAS10:RPC53:HCA4:C4_02880C_A:C4_03140C_A:C4_03720C_A:C4_03830W_A:ECM1:C4_04520W_A:BAS1:C4_05260W_A:C4_05650W_A:C4_06790W_A:C4_07060W_A:C4_07150W_A:C5_00920W_A:CYC3:C5_01430C_A:FYV5:SPB4:C5_02010C_A:C5_02070C_A:RIX7:C5_02440C_A:SPR28:UTP13:C5_03010W_A:RMS1:C5_03920C_A:C5_04120C_A:C5_04840C_A:C5_04910W_A:GRF10:C5_05340W_A:NOP5:C6_02230W_A:ALG11:C6_02430W_A:C6_02900C_A:POP4:C6_03440W_A:C6_04240W_A:NOP8:C7_00160C_A:C7_00220W_A:C7_00330C_A:DBP7:C7_01360C_A:C7_01950W_A:C7_02460C_A:C7_02930C_A:C7_03400C_A:ISY1:C7_03590C_A:C7_03850W_A:C7_03880C_A:C7_04150W_A:UTP18:CR_00460C_A:PWP2:CR_00830W_A:DAL81:CR_01410C_A:CR_01600C_A:CR_01700C_A:CR_02030C_A:CR_02420W_A:RRP9:CR_02890C_A:SRP40:SMC5:CR_03200C_A:CR_03360W_A:NCS2:YVH1:CR_03940W_A:CR_04110W_A:CR_04710W_A:NOC2:CR_05550C_A:RNR3:DBP8:RFX1:RPL7:CR_06230W_A:FGR50:CR_06450W_A:RRN11:PIF1:FUN30:DBP6:IMP4:CR_08000C_A:RIO2:ATS1:CR_08330W_A:CR_08410W_A:MPS1:CR_09740W_A:CR_09800C_A:CR_10260W_A:CR_10410C_A:DRS1:POP3

9058	biosynthetic process	702 out of 1061 genes, 66.2%	2586 out of 6473 background genes, 40.0%	2.00E-76	0.00%	RIM8:LEU4:HBR1:C1_00510W_A:CNS1:RPO41:SPE1:NRM1:RRS1:TRI1:HMT1:C1_01150C_A:C1_01160C_A:TRM2:ABC1:C1_01530C_A:CYS4:C1_02090C_A:ABP140:CWH8:C1_02380C_A:C1_02450C_A:SNZ1:SNO1:HOM6:LYS2:PDE2:MIA40:C1_02900C_A:C1_02970W_A:TSR2:C1_03370W_A:C1_03540C_A:C1_03630W_A:C1_03790C_A:RPP1:C1_03830C_A:KTR4:C1_04040C_A:C1_04120C_A:ERB1:NOP4:C1_04510W_A:C1_04530C_A:C1_04710C_A:MRPL37:C1_04970W_A:RHD1:ARG2:RRP6:REI1:TRY2:ARO4:C1_05220C_A:C1_05270C_A:C1_05360C_A:C1_05380C_A:RSM22:RPS27A:C1_05630C_A:FAL1:C1_05650W_A:RAD10:C1_05990C_A:BUD16:C1_06530C_A:C1_06540C_A:NOP6:C1_06760C_A:RIA1:YMC1:C1_07340W_A:C1_07470C_A:LHP1:C1_07510W_A:C1_07660W_A:ADE4:GCS1:ADE5,7:MRPL3:ERD1:C1_07950C_A:C1_07960W_A:FUN12:DRG1:CAT8:MLT1:DIP2:SAM4:GCR3:UPC2:C1_08520C_A:GCD6:C1_08630W_A:MSS116:C1_08890C_A:CEF1:C1_09390W_A:GUA1:C1_09610W_A:C1_09710C_A:C1_09790C_A:BUD31:C1_09910C_A:PDC2:DBP3:MNN23:C1_10080W_A:C1_10200C_A:C1_10340W_A:FCY23:C1_10620W_A:RPA190:C1_10690W_A:C1_10920W_A:C1_10950C_A:C1_10970W_A:C1_11000C_A:PET127:FGR39:NEP1:KRR1:SAM2:C1_11610C_A:C1_11790W_A:MUP1:C1_11880W_A:C1_11900C_A:C1_11910W_A:SER33:TRP3:C1_12280C_A:CSI2:MRPS9:C1_12350W_A:WRS1:C1_12440W_A:C1_12570C_A:C1_12630C_A:C1_12670C_A:C1_12750C_A:C1_12760W_A:FEN1:C1_12820C_A:RPL14:C1_13060C_A:REX3:C1_13330C_A:RMP1:C1_13380W_A:AGC1:MCU1:C1_13820C_A:C1_14080W_A:C1_14330W_A:MPP10:FAV3:C2_00200W_A:C2_00280C_A:C2_00390C_A:C2_00410C_A:C2_00820W_A:C2_00840W_A:MXR1:C2_01070W_A:C2_01220W_A:C2_01420C_A:APT1:REX2:C2_01740C_A:C2_01820C_A:PRP39:C2_01860C_A:C2_01870C_A:ARO3:C2_02120W_A:C2_02270C_A:C2_02420C_A:UTP21:UTP22:PRS1:C2_02540W_A:C2_02630W_A:PDR17:SER2:C2_02930C_A:MET1:BNA31:SMP3:PRP3:C2_03560C_A:MNN42:GPI13:SCH9:C2_03950W_A:C2_04120C_A:TBF1:BAT21:SUV3:RIM2:C2_04570W_A:SNU114:C2_04700C_A:C2_04820W_A:PZF1:CKA2:C2_05050C_A:C2_05080C_A:MAK5:C2_05160C_A:CDC21:RPF2:C2_05270W_A:RPC40:KTI11:C2_05670C_A:C2_05750W_A:C2_05830C_A:C2_05840W_A:IDP1:LAS1:ECM17:ILV1:SER1:MAK16:C2_06480W_A:C2_06520C_A:C2_06530W_A:VAS1:C2_06650C_A:C2_06660W_A:RPL11:FHL1:C2_06850W_A:C2_06950C_A:SPE3:AAH1:NOC4:C2_07360W_A:C2_07410W_A:RCL1:C2_07680W_A:PHO86:PRS5:C2_07920W_A:NSA1:C2_08180C_A:EAF3:RRP8:C2_08530C_A:MSU1:UTP15:MFG1:ASN1:C2_09160W_A:C2_09180W_A:MED18:C2_09310C_A:PES1:TAZ1:RRP15:C2_09500W_A:C2_09660W_A:LEU42:SMM1:C2_09920W_A:CWC22:UBP8:GPD1:PUS7:HEM3:C2_10740C_A:RPC11:C2_10810W_A:RPS24:C3_00100W_A:ULP3:RHR2:UTP8:BUD21:SOF1:C3_00660W_A:C3_00830C_A:BMS1:ARP9:UTP20:NCE103:C3_01310W_A:URA3:MET18:RKI1:RPS7A:C3_01520C_A:C3_01560W_A:C3_02020W_A:C3_02040C_A:UTP4:C3_02180C_A:ILV2:C3_02350W_A:PHA2:C3_02670W_A:C3_02750W_A:C3_02840W_A:C3_028

						50C_A:MET13:C3_03070W_A:C3_03110W_A:C3_03330C_A:YAH1:C3_03560W_A:HBR3:RAD53:C3_04370C_A:C3_04380C_A:ADE2:MAK21:RPS15:RPP2B:ARV1:SFP1:NOP14:CEM1:C3_05140C_A:C3_05160C_A:RPL8B:C3_05280C_A:C3_05380W_A:GPI1:C3_05440C_A:QDR2:C3_05800W_A:C3_05860C_A:TRM12:C3_05900W_A:CTA4:NOG1:C3_06150W_A:C3_06240C_A:DOT1:ISW2:C3_06370C_A:NSA2:OPI3:PRP5:C3_06830C_A:RPS12:NOP13:C3_07400W_A:C3_07550C_A:C3_07570C_A:RIT1:RAD4:UTP9:C3_07800C_A:HIS4:MET15:DUR4:RLP24:NMD5:HIS5:C4_00690C_A:C4_00740W_A:C4_00800W_A:C4_00810C_A:C4_00880W_A:ARO1:C4_00940W_A:PTC8:RAT1:AAT22:C4_01280C_A:PGA53:ILV6:PWP1:NIP1:C4_01500W_A:TOA2:HSX11:TIM10:ZCF25:C4_02400C_A:SLX4:HIT1:SAS10:RPC53:HCA4:C4_02850W_A:ZUO1:C4_02880C_A:NAN1:C4_03140C_A:C4_03170W_A:DAO2:C4_03410W_A:RAD2:C4_03720C_A:C4_03730C_A:C4_03740W_A:NUP84:C4_03830W_A:RRP42:RAM1:ECM1:C4_04130W_A:DUO1:C4_04390W_A:C4_04520W_A:SSZ1:BAS1:C4_04810C_A:AGP3:RPL30:C4_05230C_A:C4_05260W_A:C4_05360C_A:OFD1:HOM3:C4_05650W_A:TRP5:C4_06210C_A:C4_06410W_A:NOP1:C4_06790W_A:C4_06950W_A:MET16:C4_07060W_A:TRP4:C4_07140W_A:C4_07150W_A:C5_00030W_A:C5_00260W_A:C5_00280C_A:DUS4:C5_00320W_A:MRP17:MET14:RMT2:SPE2:C5_00820W_A:C5_00920W_A:BUD23:CYC3:C5_01140C_A:PUS4:C5_01430C_A:FYV5:SPB4:C5_01610W_A:CCN1:C5_01700W_A:C5_01930W_A:C5_02010C_A:C5_02070C_A:RIX7:THR4:C5_02440C_A:TIF5:SPR28:UTP13:C5_02590C_A:BUR2:CKB2:MSW1:C5_02780W_A:C5_02820C_A:C5_03010W_A:RMS1:CAM1-1:C5_03290C_A:PCL1:RPO26:FUR1:C5_03400C_A:C5_03460C_A:C5_03530C_A:C5_03550W_A:MDJ1:C5_03840W_A:C5_03920C_A:C5_03970W_A:C5_04120C_A:SAH1:C5_04290C_A:C5_04340W_A:URA7:CSU57:MSM1:C5_04720C_A:HYS2:HAS1:C5_04840C_A:C5_04910W_A:GRF10:C5_05250C_A:SDH4:HIS1:C5_05340W_A:ILV3:NOP5:C6_00530C_A:C6_00560W_A:FCA1:C6_00640C_A:CDC14:TOP1:HAL22:C6_01040C_A:C6_01120C_A:CIC1:PMP1:TIF3:RAD18:HCH1:C6_01890C_A:C6_01980C_A:SWD3:C6_02230W_A:RPL10A:ALG11:C6_02280W_A:C6_02290C_A:C6_02300C_A:C6_02350C_A:C6_02370C_A:C6_02430W_A:PRP45:C6_02690C_A:MRT4:C6_02900C_A:POP4:C6_03210C_A:BMT4:C6_03320W_A:C6_03380W_A:C6_03390W_A:C6_03440W_A:NAM2:SPB1:C6_04240W_A:NOP8:ILS1:C6_04530C_A:C7_00160C_A:C7_00220W_A:C7_00330C_A:THG1:RPA135:NOP15:YML6:C7_01020C_A:C7_01030C_A:DBP7:C7_01210C_A:ASC1:C7_01270C_A:C7_01360C_A:C7_01400C_A:GIR2:PRT1:FLU1:C7_01570C_A:C7_01600W_A:C7_01950W_A:LIG1:C7_02340C_A:C7_02460C_A:C7_02660C_A:C7_02930C_A:IFM1:C7_03400C_A:ISY1:ENP2:ARG4:C7_03590C_A:ENP1:HIS7:MRP7:C7_03850W_A:C7_03880C_A:C7_04150W_A:UTP18:MIS12:CR_00430C_A:CR_00460C_A:MRPL6:ADE1:BUD22:PWP2:CR_00830W_A:TRM1:DAL81:CR_01260W_A:CR_01320C_A:CR_01410C_A:PRS:CR_01550C_A:TRP2:CR_01600C_A:MET6:CR

						_01670W_A:CDC60:CR_01700C_A:CR_01710W_A:CR_01780W_A:CR_01950W_A:CR_02030C_A:MCM6:MET2:ERG25:CR_02420W_A:CR_02430C_A:CHO2:CR_02550C_A:PAN6:RRP9:RPC31:CR_02890C_A:SRP40:ARC1:SMC5:CR_03110W_A:CR_03200C_A:CR_03230W_A:CR_03360W_A:KTI12:MTG1:CR_03400W_A:NCS2:YVH1:SMC1:PRP42:CR_03940W_A:MEX67:CR_04110W_A:CR_04160C_A:CR_04170W_A:QDR1:CR_04240C_A:CR_04300W_A:CR_04500C_A:CR_04710W_A:ADE6:RPB8:CR_04920W_A:NOC2:CR_05550C_A:RNR3:MTG2:DBP8:RFX1:RPL7:CR_06230W_A:FGR50:CR_06450W_A:RRN11:HSP60:CR_06680C_A:NMD3:CR_06840W_A:CR_06970C_A:CR_06980W_A:POL2:MIS11:CR_07030C_A:URA2:CR_07310W_A:PIF1:FUN30:CR_07640C_A:ARO2:CDC45:DBP6:IMP4:CR_08000C_A:NOP10:SSB1:RIO2:ATS1:CR_08330W_A:CYS3:RPP2A:GSH2:CR_08410W_A:TSR1:CR_08940W_A:MPS1:ELF1:SSF1:CR_09740W_A:MED21:CR_09800C_A:DPB2:SIK1:UTP5:CR_10260W_A:CR_10410C_A:CR_10470C_A:DRS1:PRO1:LTV1:POP3

2E+06	organic substance biosynthetic process	699 out of 1061 genes, 65.9%	2571 out of 6473 background genes, 39.7%	3.62E-76	0.00%	RIM8:LEU4:HBR1:C1_00510W_A:CNS1:RPO41:SPE1:NRM1:RRS1:TRI1:HMT1:C1_01150C_A:C1_01160C_A:TRM2:ABC1:C1_01530C_A:CYS4:C1_02090C_A:ABP140:CWH8:C1_02380C_A:C1_02450C_A:SNZ1:SNO1:HOM6:LYS2:PDE2:MIA40:C1_02900C_A:C1_02970W_A:TSR2:C1_03370W_A:C1_03540C_A:C1_03630W_A:C1_03790C_A:RPP1:C1_03830C_A:KTR4:C1_04040C_A:C1_04120C_A:ERB1:NOP4:C1_04510W_A:C1_04530C_A:C1_04710C_A:MRPL37:C1_04970W_A:RHD1:ARG2:RRP6:REI1:TRY2:ARO4:C1_05220C_A:C1_05270C_A:C1_05360C_A:C1_05380C_A:RSM22:RPS27A:C1_05630C_A:FAL1:C1_05650W_A:RAD10:C1_05990C_A:BUD16:C1_06530C_A:C1_06540C_A:NOP6:C1_06760C_A:RIA1:YMC1:C1_07340W_A:C1_07470C_A:LHP1:C1_07510W_A:C1_07660W_A:ADE4:GCS1:ADE5,7:MRPL3:ERD1:C1_07950C_A:C1_07960W_A:FUN12:DRG1:CAT8:MLT1:DIP2:SAM4:GCR3:UPC2:C1_08520C_A:GCD6:C1_08630W_A:MSS116:C1_08890C_A:CEF1:C1_09390W_A:GUA1:C1_09610W_A:C1_09710C_A:C1_09790C_A:BUD31:C1_09910C_A:PDC2:DBP3:MNN23:C1_10080W_A:C1_10200C_A:C1_10340W_A:FCY23:C1_10620W_A:RPA190:C1_10690W_A:C1_10920W_A:C1_10950C_A:C1_10970W_A:C1_11000C_A:PET127:FGR39:NEP1:KRR1:SAM2:C1_11610C_A:C1_11790W_A:MUP1:C1_11880W_A:C1_11900C_A:C1_11910W_A:SER33:TRP3:C1_12280C_A:CSI2:MRPS9:C1_12350W_A:WRS1:C1_12440W_A:C1_12570C_A:C1_12630C_A:C1_12670C_A:C1_12750C_A:C1_12760W_A:FEN1:C1_12820C_A:RPL14:C1_13060C_A:REX3:C1_13330C_A:RMP1:C1_13380W_A:MCU1:C1_13820C_A:C1_14080W_A:C1_14330W_A:MPP10:FAV3:C2_00200W_A:C2_00280C_A:C2_00390C_A:C2_00410C_A:C2_00820W_A:C2_00840W_A:MXR1:C2_01070W_A:C2_01220W_A:C2_01420C_A:APT1:REX2:C2_01740C_A:C2_01820C_A:PRP39:C2_01860C_A:C2_01870C_A:ARO3:C2_02120W_A:C2_02270C_A:C2_02420C_A:UTP21:UTP22:PRS1:C2_02540W_A:C2_02630W_A:PDR17:SER2:C2_02930C_A:MET1:BNA31:SMP3:PRP3:C2_03560C_A:MNN42:GPI13:SCH9:C2_03950W_A:C2_04120C_A:TBF1:BAT21:SUV3:RIM2:C2_04570W_A:SNU114:C2_04700C_A:C2_04820W_A:PZF1:CKA2:C2_05050C_A:C2_05080C_A:MAK5:C2_05160C_A:CDC21:RPF2:C2_05270W_A:RPC40:KTI11:C2_05670C_A:C2_05750W_A:C2_05830C_A:C2_05840W_A:IDP1:LAS1:ECM17:ILV1:SER1:MAK16:C2_06480W_A:C2_06520C_A:C2_06530W_A:VAS1:C2_06650C_A:C2_06660W_A:RPL11:FHL1:C2_06850W_A:C2_06950C_A:SPE3:AAH1:NOC4:C2_07360W_A:C2_07410W_A:RCL1:C2_07680W_A:PHO86:PRS5:C2_07920W_A:NSA1:C2_08180C_A:EAF3:RRP8:C2_08530C_A:MSU1:UTP15:MFG1:ASN1:C2_09160W_A:C2_09180W_A:MED18:C2_09310C_A:PES1:TAZ1:RRP15:C2_09500W_A:C2_09660W_A:LEU42:SMM1:C2_09920W_A:CWC22:UBP8:GPD1:PUS7:HEM3:C2_10740C_A:RPC11:C2_10810W_A:RPS24:C3_00100W_A:ULP3:RHR2:UTP8:BUD21:SOF1:C3_00660W_A:C3_00830C_A:BMS1:ARP9:UTP20:NCE103:C3_01310W_A:URA3:MET18:RKI1:RPS7A:C3_01520C_A:C3_01560W_A:C3_02020W_A:C3_02040C_A:UTP4:C3_02180C_A:ILV2:C3_02350W_A:PHA2:C3_02670W_A:C3_02750W_A:C3_02840W_A:C3_02850C_

						A:MET13:C3_03070W_A:C3_03110W_A:C3_03330C_A:YAH1:C3_03560W_A:HBR3:RAD53:C3_04370C_A:C3_04380C_A:ADE2:MAK21:RPS15:RPP2B:ARV1:SFP1:NOP14:CEM1:C3_05140C_A:C3_05160C_A:RPL8B:C3_05280C_A:C3_05380W_A:GPI1:C3_05440C_A:QDR2:C3_05800W_A:C3_05860C_A:TRM12:C3_05900W_A:CTA4:NOG1:C3_06150W_A:C3_06240C_A:DOT1:ISW2:C3_06370C_A:NSA2:OPI3:PRP5:C3_06830C_A:RPS12:NOP13:C3_07400W_A:C3_07550C_A:C3_07570C_A:RIT1:RAD4:UTP9:C3_07800C_A:HIS4:MET15:DUR4:RLP24:NMD5:HIS5:C4_00690C_A:C4_00740W_A:C4_00800W_A:C4_00810C_A:C4_00880W_A:ARO1:C4_00940W_A:PTC8:RAT1:C4_01280C_A:PGA53:ILV6:PWP1:NIP1:C4_01500W_A:TOA2:HSX11:TIM10:ZCF25:C4_02400C_A:SLX4:HIT1:SAS10:RPC53:HCA4:C4_02850W_A:ZUO1:C4_02880C_A:NAN1:C4_03140C_A:C4_03170W_A:DAO2:C4_03410W_A:RAD2:C4_03720C_A:C4_03730C_A:C4_03740W_A:NUP84:C4_03830W_A:RRP42:RAM1:ECM1:C4_04130W_A:DUO1:C4_04390W_A:C4_04520W_A:SSZ1:BAS1:C4_04810C_A:AGP3:RPL30:C4_05230C_A:C4_05260W_A:C4_05360C_A:OFD1:HOM3:C4_05650W_A:TRP5:C4_06210C_A:C4_06410W_A:NOP1:C4_06790W_A:C4_06950W_A:MET16:C4_07060W_A:TRP4:C4_07140W_A:C4_07150W_A:C5_00030W_A:C5_00260W_A:C5_00280C_A:DUS4:C5_00320W_A:MRP17:MET14:RMT2:SPE2:C5_00820W_A:C5_00920W_A:BUD23:CYC3:C5_01140C_A:PUS4:C5_01430C_A:FYV5:SPB4:C5_01610W_A:CCN1:C5_01700W_A:C5_01930W_A:C5_02010C_A:C5_02070C_A:RIX7:THR4:C5_02440C_A:TIF5:SPR28:UTP13:C5_02590C_A:BUR2:CKB2:MSW1:C5_02780W_A:C5_02820C_A:C5_03010W_A:RMS1:CAM1-1:C5_03290C_A:PCL1:RPO26:FUR1:C5_03400C_A:C5_03460C_A:C5_03530C_A:C5_03550W_A:MDJ1:C5_03840W_A:C5_03920C_A:C5_03970W_A:C5_04120C_A:SAH1:C5_04290C_A:C5_04340W_A:URA7:CSU57:MSM1:C5_04720C_A:HYS2:HAS1:C5_04840C_A:C5_04910W_A:GRF10:C5_05250C_A:SDH4:HIS1:C5_05340W_A:ILV3:NOP5:C6_00530C_A:C6_00560W_A:FCA1:C6_00640C_A:CDC14:TOP1:HAL22:C6_01040C_A:C6_01120C_A:CIC1:PMP1:TIF3:RAD18:HCH1:C6_01890C_A:C6_01980C_A:SWD3:C6_02230W_A:RPL10A:ALG11:C6_02280W_A:C6_02290C_A:C6_02300C_A:C6_02350C_A:C6_02370C_A:C6_02430W_A:PRP45:C6_02690C_A:MRT4:C6_02900C_A:POP4:C6_03210C_A:BMT4:C6_03380W_A:C6_03390W_A:C6_03440W_A:NAM2:SPB1:C6_04240W_A:NOP8:ILS1:C6_04530C_A:C7_00160C_A:C7_00220W_A:C7_00330C_A:THG1:RPA135:NOP15:YML6:C7_01020C_A:C7_01030C_A:DBP7:C7_01210C_A:ASC1:C7_01270C_A:C7_01360C_A:C7_01400C_A:GIR2:PRT1:FLU1:C7_01570C_A:C7_01600W_A:C7_01950W_A:LIG1:C7_02340C_A:C7_02460C_A:C7_02660C_A:C7_02930C_A:IFM1:C7_03400C_A:ISY1:ENP2:ARG4:C7_03590C_A:ENP1:HIS7:MRP7:C7_03850W_A:C7_03880C_A:C7_04150W_A:UTP18:MIS12:CR_00430C_A:CR_00460C_A:MRPL6:ADE1:BUD22:PWP2:CR_00830W_A:TRM1:DAL81:CR_01260W_A:CR_01320C_A:CR_01410C_A:PRS:CR_01550C_A:TRP2:CR_01600C_A:MET6:CR_01670W_A:CDC6

						0:CR_01700C_A:CR_01710W_A:CR_01780W_A:CR_01950W_A:CR_02030C_A:MCM6:MET2:ERG25:CR_02420W_A:CR_02430C_A:CHO2:CR_02550C_A:PAN6:RRP9:RPC31:CR_02890C_A:SRP40:ARC1:SMC5:CR_03110W_A:CR_03200C_A:CR_03230W_A:CR_03360W_A:KTI12:MTG1:CR_03400W_A:NCS2:YVH1:SMC1:PRP42:CR_03940W_A:MEX67:CR_04110W_A:CR_04160C_A:CR_04170W_A:QDR1:CR_04240C_A:CR_04300W_A:CR_04500C_A:CR_04710W_A:ADE6:RPB8:CR_04920W_A:NOC2:CR_05550C_A:RNR3:MTG2:DBP8:RFX1:RPL7:CR_06230W_A:FGR50:CR_06450W_A:RRN11:HSP60:CR_06680C_A:NMD3:CR_06840W_A:CR_06970C_A:CR_06980W_A:POL2:MIS11:CR_07030C_A:URA2:CR_07310W_A:PIF1:FUN30:CR_07640C_A:ARO2:CDC45:DBP6:IMP4:CR_08000C_A:NOP10:SSB1:RIO2:ATS1:CR_08330W_A:CYS3:RPP2A:GSH2:CR_08410W_A:TSR1:CR_08940W_A:MPS1:ELF1:SSF1:CR_09740W_A:MED21:CR_09800C_A:DPB2:SIK1:UTP5:CR_10260W_A:CR_10410C_A:CR_10470C_A:DRS1:PRO1:LTV1:POP3

9059	macromolecule biosynthetic process	619 out of 1061 genes, 58.3%	2190 out of 6473 background genes, 33.8%	5.33E-69	0.00%	RIM8:HBR1:CNS1:RPO41:NRM1:RRS1:TRI1:HMT1:C1_01150C_A:C1_01160C_A:TRM2:C1_01530C_A:C1_02090C_A:ABP140:CWH8:C1_02380C_A:C1_02450C_A:HOM6:PDE2:MIA40:C1_02900C_A:TSR2:C1_03370W_A:C1_03540C_A:C1_03630W_A:C1_03790C_A:RPP1:C1_03830C_A:KTR4:C1_04040C_A:C1_04120C_A:ERB1:NOP4:C1_04510W_A:C1_04530C_A:C1_04710C_A:MRPL37:C1_04970W_A:RRP6:REI1:TRY2:C1_05220C_A:C1_05270C_A:C1_05360C_A:C1_05380C_A:RSM22:RPS27A:C1_05630C_A:FAL1:C1_05650W_A:RAD10:C1_06530C_A:C1_06540C_A:NOP6:C1_06760C_A:RIA1:C1_07340W_A:C1_07470C_A:LHP1:C1_07510W_A:C1_07660W_A:MRPL3:ERD1:C1_07950C_A:C1_07960W_A:FUN12:DRG1:CAT8:MLT1:DIP2:GCR3:C1_08520C_A:GCD6:C1_08630W_A:MSS116:C1_08890C_A:CEF1:C1_09390W_A:C1_09710C_A:C1_09790C_A:BUD31:C1_09910C_A:PDC2:DBP3:MNN23:C1_10080W_A:C1_10200C_A:C1_10340W_A:FCY23:C1_10620W_A:RPA190:C1_10690W_A:C1_10920W_A:C1_10950C_A:C1_10970W_A:C1_11000C_A:PET127:FGR39:NEP1:KRR1:SAM2:MUP1:C1_11880W_A:C1_11900C_A:C1_11910W_A:SER33:TRP3:C1_12280C_A:CSI2:MRPS9:C1_12350W_A:WRS1:C1_12440W_A:C1_12570C_A:C1_12630C_A:C1_12670C_A:C1_12750C_A:C1_12760W_A:FEN1:C1_12820C_A:RPL14:C1_13060C_A:REX3:RMP1:C1_13380W_A:MCU1:C1_13820C_A:C1_14080W_A:C1_14330W_A:MPP10:FAV3:C2_00200W_A:C2_00280C_A:C2_00410C_A:C2_00820W_A:C2_00840W_A:C2_01070W_A:C2_01220W_A:C2_01420C_A:REX2:C2_01740C_A:C2_01820C_A:PRP39:C2_01860C_A:C2_01870C_A:C2_02120W_A:C2_02270C_A:C2_02420C_A:UTP21:UTP22:PRS1:C2_02540W_A:C2_02630W_A:PDR17:C2_02930C_A:MET1:SMP3:PRP3:C2_03560C_A:MNN42:GPI13:SCH9:C2_03950W_A:C2_04120C_A:TBF1:SUV3:RIM2:C2_04570W_A:SNU114:C2_04700C_A:C2_04820W_A:PZF1:CKA2:C2_05050C_A:C2_05080C_A:MAK5:C2_05160C_A:RPF2:C2_05270W_A:RPC40:KTI11:C2_05670C_A:C2_05750W_A:C2_05830C_A:C2_05840W_A:IDP1:LAS1:ECM17:SER1:MAK16:C2_06480W_A:C2_06520C_A:C2_06530W_A:VAS1:C2_06650C_A:C2_06660W_A:RPL11:FHL1:C2_06850W_A:C2_06950C_A:SPE3:NOC4:C2_07360W_A:RCL1:C2_07680W_A:PHO86:PRS5:C2_07920W_A:NSA1:C2_08180C_A:EAF3:RRP8:C2_08530C_A:MSU1:UTP15:MFG1:C2_09160W_A:C2_09180W_A:MED18:C2_09310C_A:PES1:TAZ1:RRP15:C2_09500W_A:C2_09660W_A:SMM1:C2_09920W_A:CWC22:UBP8:PUS7:HEM3:C2_10740C_A:RPC11:C2_10810W_A:RPS24:C3_00100W_A:ULP3:UTP8:BUD21:SOF1:C3_00660W_A:C3_00830C_A:BMS1:ARP9:UTP20:NCE103:C3_01310W_A:MET18:RPS7A:C3_01520C_A:C3_01560W_A:C3_02020W_A:C3_02040C_A:UTP4:C3_02180C_A:ILV2:C3_02350W_A:C3_02670W_A:C3_02750W_A:C3_02840W_A:C3_02850C_A:C3_03070W_A:C3_03110W_A:C3_03330C_A:C3_03560W_A:HBR3:C3_04370C_A:C3_04380C_A:MAK21:RPS15:RPP2B:ARV1:SFP1:NOP14:CEM1:C3_05140C_A:C3_05160C_A:RPL8B:C3_05280C_A:C3_05380W_A:GPI1:C3_05440C_A:QDR2:C3_05800W_A:C3_05860C_A:TRM12:C3_05900W_A:CTA4:NOG1:C3_06150W_A:C3_06240C_A:DOT1:ISW2:

						C3_06370C_A:NSA2:PRP5:C3_06830C_A:RPS12:NOP13:C3_07400W_A:C3_07550C_A:C3_07570C_A:RIT1:RAD4:UTP9:C3_07800C_A:DUR4:RLP24:NMD5:HIS5:C4_00690C_A:C4_00740W_A:C4_00800W_A:C4_00810C_A:C4_00880W_A:ARO1:C4_00940W_A:PTC8:RAT1:C4_01280C_A:PGA53:PWP1:NIP1:C4_01500W_A:TOA2:HSX11:TIM10:ZCF25:C4_02400C_A:SLX4:HIT1:SAS10:RPC53:HCA4:C4_02850W_A:ZUO1:C4_02880C_A:NAN1:C4_03140C_A:C4_03170W_A:DAO2:C4_03410W_A:RAD2:C4_03720C_A:C4_03730C_A:C4_03740W_A:NUP84:C4_03830W_A:RRP42:RAM1:ECM1:C4_04130W_A:DUO1:C4_04390W_A:C4_04520W_A:SSZ1:BAS1:C4_04810C_A:AGP3:RPL30:C4_05230C_A:C4_05260W_A:C4_05360C_A:OFD1:C4_05650W_A:C4_06210C_A:C4_06410W_A:NOP1:C4_06790W_A:C4_06950W_A:C4_07060W_A:C4_07140W_A:C4_07150W_A:C5_00030W_A:C5_00280C_A:DUS4:C5_00320W_A:MRP17:MET14:RMT2:C5_00820W_A:C5_00920W_A:BUD23:CYC3:C5_01140C_A:PUS4:C5_01430C_A:FYV5:SPB4:C5_01610W_A:CCN1:C5_01700W_A:C5_01930W_A:C5_02010C_A:C5_02070C_A:RIX7:C5_02440C_A:TIF5:SPR28:UTP13:C5_02590C_A:BUR2:CKB2:MSW1:C5_02780W_A:C5_02820C_A:C5_03010W_A:RMS1:CAM1-1:C5_03290C_A:PCL1:RPO26:FUR1:C5_03400C_A:C5_03460C_A:C5_03530C_A:C5_03550W_A:MDJ1:C5_03920C_A:C5_03970W_A:C5_04120C_A:C5_04290C_A:C5_04340W_A:CSU57:MSM1:HYS2:HAS1:C5_04840C_A:C5_04910W_A:GRF10:C5_05250C_A:SDH4:C5_05340W_A:NOP5:C6_00530C_A:C6_00560W_A:C6_00640C_A:CDC14:TOP1:C6_01040C_A:C6_01120C_A:CIC1:PMP1:TIF3:RAD18:HCH1:C6_01890C_A:C6_01980C_A:SWD3:C6_02230W_A:RPL10A:ALG11:C6_02280W_A:C6_02290C_A:C6_02300C_A:C6_02350C_A:C6_02370C_A:C6_02430W_A:PRP45:C6_02690C_A:MRT4:C6_02900C_A:POP4:C6_03210C_A:C6_03380W_A:C6_03390W_A:C6_03440W_A:NAM2:SPB1:C6_04240W_A:NOP8:ILS1:C6_04530C_A:C7_00160C_A:C7_00220W_A:C7_00330C_A:THG1:RPA135:NOP15:YML6:C7_01020C_A:C7_01030C_A:DBP7:C7_01210C_A:ASC1:C7_01270C_A:C7_01360C_A:C7_01400C_A:GIR2:PRT1:FLU1:C7_01570C_A:C7_01600W_A:C7_01950W_A:LIG1:C7_02340C_A:C7_02460C_A:C7_02660C_A:C7_02930C_A:IFM1:C7_03400C_A:ISY1:ENP2:C7_03590C_A:ENP1:HIS7:MRP7:C7_03850W_A:C7_03880C_A:C7_04150W_A:UTP18:CR_00430C_A:CR_00460C_A:MRPL6:ADE1:BUD22:PWP2:CR_00830W_A:TRM1:DAL81:CR_01260W_A:CR_01320C_A:CR_01410C_A:PRS:CR_01550C_A:CR_01600C_A:CR_01670W_A:CDC60:CR_01700C_A:CR_01710W_A:CR_01780W_A:CR_01950W_A:CR_02030C_A:MCM6:ERG25:CR_02420W_A:CR_02430C_A:CR_02550C_A:RRP9:RPC31:CR_02890C_A:SRP40:ARC1:SMC5:CR_03110W_A:CR_03200C_A:CR_03230W_A:CR_03360W_A:KTI12:MTG1:CR_03400W_A:NCS2:YVH1:SMC1:PRP42:CR_03940W_A:MEX67:CR_04110W_A:CR_04160C_A:CR_04170W_A:CR_04240C_A:CR_04300W_A:CR_04500C_A:CR_04710W_A:RPB8:CR_04920W_A:NOC2:CR_05550C_A:RNR3:MTG2:DBP8:RFX1:RPL7:CR_06230W_A:FGR50:CR_06450W_A:RRN11:HSP60:CR_06680C_A:NMD3:CR_06840W_A:CR_06

						970C_A:CR_06980W_A:POL2:CR_07030C_A:URA2:CR_07310W_A:PIF1:FUN30:CR_07640C_A:CDC45:DBP6:IMP4:CR_08000C_A:NOP10:SSB1:RIO2:ATS1:CR_08330W_A:RPP2A:GSH2:CR_08410W_A:TSR1:CR_08940W_A:MPS1:ELF1:SSF1:CR_09740W_A:MED21:CR_09800C_A:DPB2:SIK1:UTP5:CR_10260W_A:CR_10410C_A:CR_10470C_A:DRS1:LTV1:POP3

470	maturation of LSU-rRNA	212 out of 1061 genes, 20.0%	431 out of 6473 background genes, 6.7%	4.60E-58	0.00%	TRI1:C1_02090C_A:C1_02450C_A:PDE2:C1_02900C_A:RPP1:C1_03830C_A:C1_04120C_A:NOP4:C1_04510W_A:C1_04710C_A:C1_04970W_A:REI1:TRY2:C1_05220C_A:C1_05650W_A:C1_06540C_A:NOP6:RIA1:C1_07510W_A:C1_07660W_A:ERD1:C1_07960W_A:GCR3:C1_08630W_A:CEF1:C1_09390W_A:PDC2:DBP3:FCY23:C1_11000C_A:MUP1:C1_11900C_A:C1_11910W_A:TRP3:C1_12280C_A:CSI2:C1_12350W_A:C1_12440W_A:C1_12570C_A:C1_12630C_A:C1_12750C_A:C1_12760W_A:C1_12820C_A:RMP1:C1_13380W_A:MCU1:C2_00280C_A:C2_00820W_A:C2_01220W_A:C2_01820C_A:C2_01860C_A:C2_02120W_A:C2_02540W_A:C2_02630W_A:PRP3:SCH9:C2_04120C_A:SUV3:SNU114:C2_04700C_A:PZF1:CKA2:C2_05050C_A:MAK5:RPF2:C2_05750W_A:C2_05830C_A:LAS1:ECM17:MAK16:C2_06520C_A:C2_06530W_A:C2_06660W_A:C2_06850W_A:EAF3:RRP8:C2_08530C_A:MSU1:UTP15:C2_09160W_A:C2_09180W_A:RRP15:SMM1:C2_09920W_A:CWC22:C2_10740C_A:C3_00660W_A:C3_00830C_A:ARP9:NCE103:C3_01310W_A:C3_01560W_A:ILV2:C3_02670W_A:C3_02840W_A:C3_02850C_A:C3_03330C_A:C3_04370C_A:ARV1:NOP14:CEM1:C3_05160C_A:RPL8B:C3_05280C_A:C3_05380W_A:C3_05440C_A:QDR2:C3_05800W_A:C3_05860C_A:C3_05900W_A:NSA2:PRP5:C3_07550C_A:C3_07570C_A:DUR4:NMD5:C4_00740W_A:RAT1:C4_01280C_A:SLX4:HIT1:C4_02880C_A:DAO2:C4_03720C_A:NUP84:C4_03830W_A:RAM1:AGP3:C4_05260W_A:C4_05360C_A:C4_06210C_A:C4_06410W_A:C4_06790W_A:C4_06950W_A:C4_07060W_A:RMT2:C5_00920W_A:C5_01430C_A:SPB4:C5_01930W_A:C5_02010C_A:C5_02440C_A:SPR28:CKB2:C5_03010W_A:RMS1:C5_03920C_A:C5_04120C_A:HAS1:C5_04910W_A:GRF10:C6_01120C_A:CIC1:C6_01890C_A:SWD3:C6_02230W_A:ALG11:C6_02430W_A:C6_03210C_A:C6_03390W_A:C6_03440W_A:SPB1:C6_04240W_A:NOP8:NOP15:DBP7:C7_01360C_A:GIR2:FLU1:C7_01570C_A:LIG1:C7_02460C_A:C7_02660C_A:C7_02930C_A:C7_03400C_A:ISY1:C7_03590C_A:ENP1:C7_03850W_A:C7_03880C_A:C7_04150W_A:CR_00830W_A:DAL81:CR_01320C_A:CR_01550C_A:CR_01600C_A:CR_01670W_A:CR_01700C_A:CR_02030C_A:CR_02890C_A:CR_03200C_A:CR_03360W_A:PRP42:CR_03940W_A:CR_04170W_A:CR_04500C_A:CR_04710W_A:CR_05550C_A:RNR3:RPL7:CR_06230W_A:CR_06450W_A:RRN11:CR_06970C_A:CR_07640C_A:DBP6:CR_08000C_A:SSF1:CR_09740W_A:MED21:CR_10260W_A

463	maturation of LSU-rRNA from tricistronic rRNA transcript (SSU-rRNA, 5.8S rRNA, LSU-rRNA)	211 out of 1061 genes, 19.9%	429 out of 6473 background genes, 6.6%	9.53E-58	0.00%	TRI1:C1_02090C_A:C1_02450C_A:PDE2:C1_02900C_A:RPP1:C1_03830C_A:C1_04120C_A:NOP4:C1_04510W_A:C1_04710C_A:C1_04970W_A:REI1:TRY2:C1_05220C_A:C1_05650W_A:C1_06540C_A:NOP6:RIA1:C1_07510W_A:C1_07660W_A:ERD1:C1_07960W_A:GCR3:C1_08630W_A:CEF1:C1_09390W_A:PDC2:DBP3:FCY23:C1_11000C_A:MUP1:C1_11900C_A:C1_11910W_A:TRP3:C1_12280C_A:CSI2:C1_12350W_A:C1_12440W_A:C1_12570C_A:C1_12630C_A:C1_12750C_A:C1_12760W_A:C1_12820C_A:RMP1:C1_13380W_A:MCU1:C2_00280C_A:C2_00820W_A:C2_01220W_A:C2_01820C_A:C2_01860C_A:C2_02120W_A:C2_02540W_A:C2_02630W_A:PRP3:SCH9:C2_04120C_A:SUV3:SNU114:C2_04700C_A:PZF1:CKA2:C2_05050C_A:MAK5:RPF2:C2_05750W_A:C2_05830C_A:LAS1:ECM17:MAK16:C2_06520C_A:C2_06530W_A:C2_06660W_A:C2_06850W_A:EAF3:RRP8:C2_08530C_A:MSU1:UTP15:C2_09160W_A:C2_09180W_A:RRP15:SMM1:C2_09920W_A:CWC22:C2_10740C_A:C3_00660W_A:C3_00830C_A:ARP9:NCE103:C3_01310W_A:C3_01560W_A:ILV2:C3_02670W_A:C3_02840W_A:C3_02850C_A:C3_03330C_A:C3_04370C_A:ARV1:NOP14:CEM1:C3_05160C_A:C3_05280C_A:C3_05380W_A:C3_05440C_A:QDR2:C3_05800W_A:C3_05860C_A:C3_05900W_A:NSA2:PRP5:C3_07550C_A:C3_07570C_A:DUR4:NMD5:C4_00740W_A:RAT1:C4_01280C_A:SLX4:HIT1:C4_02880C_A:DAO2:C4_03720C_A:NUP84:C4_03830W_A:RAM1:AGP3:C4_05260W_A:C4_05360C_A:C4_06210C_A:C4_06410W_A:C4_06790W_A:C4_06950W_A:C4_07060W_A:RMT2:C5_00920W_A:C5_01430C_A:SPB4:C5_01930W_A:C5_02010C_A:C5_02440C_A:SPR28:CKB2:C5_03010W_A:RMS1:C5_03920C_A:C5_04120C_A:HAS1:C5_04910W_A:GRF10:C6_01120C_A:CIC1:C6_01890C_A:SWD3:C6_02230W_A:ALG11:C6_02430W_A:C6_03210C_A:C6_03390W_A:C6_03440W_A:SPB1:C6_04240W_A:NOP8:NOP15:DBP7:C7_01360C_A:GIR2:FLU1:C7_01570C_A:LIG1:C7_02460C_A:C7_02660C_A:C7_02930C_A:C7_03400C_A:ISY1:C7_03590C_A:ENP1:C7_03850W_A:C7_03880C_A:C7_04150W_A:CR_00830W_A:DAL81:CR_01320C_A:CR_01550C_A:CR_01600C_A:CR_01670W_A:CR_01700C_A:CR_02030C_A:CR_02890C_A:CR_03200C_A:CR_03360W_A:PRP42:CR_03940W_A:CR_04170W_A:CR_04500C_A:CR_04710W_A:CR_05550C_A:RNR3:RPL7:CR_06230W_A:CR_06450W_A:RRN11:CR_06970C_A:CR_07640C_A:DBP6:CR_08000C_A:SSF1:CR_09740W_A:MED21:CR_10260W_A

480	endonucleolytic cleavage in 5'-ETS of tricistronic rRNA transcript (SSU-rRNA, 5.8S rRNA, LSU-rRNA)	192 out of 1061 genes, 18.1%	390 out of 6473 background genes, 6.0%	5.13E-52	0.00%	HBR1:CNS1:RPO41:C1_02090C_A:C1_02900C_A:C1_03630W_A:RPP1:C1_03830C_A:C1_04040C_A:C1_04120C_A:NOP4:C1_04510W_A:REI1:TRY2:C1_05360C_A:C1_05380C_A:C1_05650W_A:C1_06540C_A:NOP6:RIA1:C1_07660W_A:C1_07960W_A:MLT1:DIP2:GCD6:C1_08630W_A:CEF1:C1_09390W_A:C1_09710C_A:C1_09790C_A:PDC2:MNN23:FCY23:C1_11000C_A:NEP1:C1_12350W_A:C1_12440W_A:C1_12570C_A:C1_12750C_A:C1_12820C_A:RMP1:C1_13820C_A:C1_14080W_A:MPP10:FAV3:C2_00200W_A:C2_00280C_A:C2_00410C_A:C2_00820W_A:C2_01220W_A:C2_02420C_A:C2_02540W_A:C2_02630W_A:MET1:PRP3:SCH9:C2_04120C_A:TBF1:PZF1:C2_05160C_A:C2_05830C_A:LAS1:C2_06520C_A:C2_06660W_A:FHL1:C2_06850W_A:NOC4:RCL1:PRS5:C2_07920W_A:NSA1:RRP8:C2_08530C_A:MSU1:RRP15:SMM1:CWC22:C2_10740C_A:C2_10810W_A:C3_00100W_A:BUD21:C3_00660W_A:BMS1:UTP20:NCE103:C3_01310W_A:C3_02020W_A:C3_02040C_A:C3_02670W_A:C3_02840W_A:C3_02850C_A:C3_03330C_A:C3_04370C_A:SFP1:NOP14:QDR2:C3_05800W_A:C3_06150W_A:ISW2:C3_06370C_A:PRP5:C3_07550C_A:RAD4:C3_07800C_A:DUR4:NMD5:C4_00690C_A:RAT1:C4_01280C_A:SLX4:HIT1:SAS10:RPC53:C4_02880C_A:NUP84:C4_03830W_A:C4_04520W_A:BAS1:C4_05260W_A:C4_05650W_A:C4_06790W_A:C4_07060W_A:C4_07150W_A:C5_01430C_A:SPB4:C5_02010C_A:C5_02070C_A:RIX7:C5_02440C_A:SPR28:UTP13:C5_03010W_A:RMS1:C5_03920C_A:C5_04120C_A:C5_04840C_A:C5_04910W_A:GRF10:C5_05340W_A:NOP5:C6_02230W_A:C6_02430W_A:C6_02900C_A:POP4:C6_03440W_A:C6_04240W_A:C7_00220W_A:C7_01360C_A:LIG1:C7_02460C_A:C7_02930C_A:C7_03400C_A:C7_03590C_A:C7_03850W_A:C7_04150W_A:UTP18:PWP2:DAL81:CR_01700C_A:CR_02030C_A:CR_02420W_A:RRP9:CR_02890C_A:SMC5:CR_03200C_A:CR_03360W_A:NCS2:YVH1:SMC1:CR_03940W_A:CR_04710W_A:CR_05550C_A:DBP8:RFX1:CR_06230W_A:CR_06450W_A:RRN11:CR_06680C_A:POL2:PIF1:FUN30:DBP6:IMP4:CR_08000C_A:RIO2:ATS1:CR_08330W_A:CR_08410W_A:MPS1:CR_09740W_A:CR_09800C_A:CR_10260W_A

6807	nitrogen compound metabolic process	785 out of 1061 genes, 74.0%	3438 out of 6473 background genes, 53.1%	4.11E-49	0.00%	RIM8:LEU4:HBR1:C1_00510W_A:CNS1:RPO41:SPE1:NRM1:RRS1:TRI1:NMA111:HMT1:C1_01150C_A:C1_01160C_A:TRM2:ABC1:C1_01530C_A:CYS4:C1_02090C_A:ABP140:CWH8:C1_02380C_A:C1_02390W_A:C1_02450C_A:SNZ1:SNO1:HOM6:LYS2:PDE2:MIA40:C1_02900C_A:C1_02970W_A:TSR2:C1_03260W_A:C1_03370W_A:C1_03540C_A:C1_03630W_A:C1_03790C_A:RPP1:C1_03830C_A:KTR4:C1_04040C_A:C1_04120C_A:ERB1:RPN4:NOP4:C1_04510W_A:C1_04530C_A:C1_04710C_A:MRPL37:C1_04970W_A:RHD1:ARG2:RRP6:REI1:TRY2:ARO4:C1_05220C_A:C1_05270C_A:C1_05360C_A:C1_05380C_A:RSM22:RPS27A:C1_05630C_A:FAL1:C1_05650W_A:RAD10:C1_05990C_A:BUD16:C1_06530C_A:C1_06540C_A:NOP6:C1_06760C_A:RIA1:YMC1:C1_07340W_A:C1_07470C_A:LHP1:C1_07510W_A:C1_07660W_A:ADE4:ECO1:C1_07850C_A:GCS1:ADE5,7:MRPL3:ERD1:C1_07950C_A:C1_07960W_A:FUN12:DRG1:CAT8:MLT1:DIP2:SAM4:GCR3:C1_08520C_A:GCD6:C1_08630W_A:MSS116:C1_08890C_A:C1_09040C_A:CEF1:C1_09390W_A:GUA1:C1_09610W_A:C1_09710C_A:C1_09790C_A:C1_09840C_A:BUD31:C1_09910C_A:PDC2:DBP3:MNN23:C1_10080W_A:C1_10200C_A:C1_10340W_A:FCY23:C1_10620W_A:APN2:RPA190:C1_10690W_A:NIT3:C1_10920W_A:C1_10950C_A:C1_10970W_A:C1_11000C_A:PET127:FGR39:NEP1:KRR1:SAM2:GAR1:C1_11610C_A:C1_11790W_A:MUP1:C1_11880W_A:C1_11900C_A:C1_11910W_A:SER33:TRP3:C1_12280C_A:CSI2:MRPS9:C1_12350W_A:WRS1:C1_12440W_A:C1_12570C_A:C1_12630C_A:C1_12640W_A:C1_12670C_A:C1_12750C_A:C1_12760W_A:FEN1:C1_12820C_A:RPL14:C1_13060C_A:REX3:C1_13330C_A:RMP1:C1_13380W_A:AGC1:KNS1:MCU1:C1_13560W_A:C1_13820C_A:MET3:YBP1:C1_14080W_A:C1_14160W_A:C1_14330W_A:MPP10:FAV3:C2_00200W_A:C2_00280C_A:C2_00390C_A:C2_00410C_A:C2_00620C_A:C2_00820W_A:C2_00840W_A:MXR1:C2_01060C_A:C2_01070W_A:C2_01220W_A:C2_01240C_A:ESC4:CLB2:C2_01420C_A:APT1:REX2:C2_01740C_A:C2_01820C_A:PRP39:C2_01860C_A:C2_01870C_A:ARO3:C2_02120W_A:SAM37:C2_02270C_A:C2_02420C_A:UTP21:UTP22:C2_02490C_A:PRS1:C2_02540W_A:C2_02630W_A:PDR17:SER2:C2_02930C_A:MET1:BNA31:SMP3:C2_03130W_A:PRP3:C2_03560C_A:C2_03700W_A:C2_03830W_A:GPI13:SCH9:C2_03950W_A:C2_04120C_A:UGA1:TBF1:BAT21:SUV3:RIM2:MED8:C2_04570W_A:SNU114:C2_04700C_A:C2_04820W_A:PZF1:CKA2:C2_04990W_A:C2_05050C_A:C2_05080C_A:MAK5:C2_05160C_A:CDC21:SGO1:RPF2:C2_05270W_A:RPC40:KTI11:C2_05670C_A:C2_05750W_A:C2_05830C_A:C2_05840W_A:IDP1:LAS1:CSM3:ECM17:ILV1:SER1:MAK16:C2_06480W_A:C2_06520C_A:C2_06530W_A:VAS1:C2_06650C_A:C2_06660W_A:AMO2:RPL11:FHL1:C2_06850W_A:C2_06950C_A:SPE3:AAH1:RCK2:C2_07270W_A:NOC4:C2_07360W_A:RCL1:C2_07680W_A:PHO86:C2_07860W_A:PRS5:C2_07920W_A:NSA1:C2_08180C_A:C2_08200W_A:EAF3:RRP8:C2_08530C_A:MSU1:UTP15:MFG1:APE3:CDC47:ASN1:C2_09160W_A:C2_09180W_A:MED18:C2_09310C_A:PES1:TAZ1:RRP15:C2_09500W_A:C2_09660W_A:LEU42:SMM1:C2_09920W_A:C

						WC22:UBP8:PUS7:HEM3:C2_10740C_A:RPC11:C2_10810W_A:RPS24:C3_00100W_A:ULP3:UTP8:BUD21:SOF1:C3_00660W_A:C3_00830C_A:BMS1:ARP9:UTP20:NCE103:C3_01310W_A:URA3:MET18:RKI1:RPS7A:C3_01520C_A:C3_01560W_A:PPT1:SCM3:C3_02020W_A:C3_02040C_A:UTP4:C3_02180C_A:ILV2:C3_02350W_A:PHA2:DDC1:C3_02670W_A:SMC3:C3_02750W_A:C3_02840W_A:C3_02850C_A:MET13:C3_03070W_A:C3_03110W_A:C3_03330C_A:YAH1:C3_03470W_A:ULP1:C3_03560W_A:HBR3:RAD53:C3_04370C_A:C3_04380C_A:ADE2:MAK21:RPS15:RPP2B:ARV1:SFP1:NOP14:CEM1:C3_05140C_A:C3_05160C_A:WOR2:RPL8B:C3_05280C_A:C3_05380W_A:GPI1:C3_05440C_A:C3_05510W_A:QDR2:BAT22:C3_05800W_A:C3_05860C_A:TRM12:C3_05900W_A:CTA4:NOG1:C3_06150W_A:MMS21:CYM1:C3_06240C_A:C3_06270C_A:DOT1:ISW2:C3_06370C_A:NSA2:OPI3:C3_06600C_A:FUN31:C3_06630W_A:PRP5:C3_06830C_A:RPS12:NOP13:C3_07400W_A:C3_07550C_A:C3_07570C_A:RIT1:RAD4:UTP9:C3_07800C_A:HIS4:MET15:DUR4:RLP24:NMD5:HIS5:C4_00690C_A:C4_00700C_A:C4_00740W_A:MLH1:C4_00800W_A:C4_00810C_A:C4_00880W_A:ARO1:C4_00940W_A:PTC8:RAT1:AAT22:C4_01280C_A:PGA53:ILV6:PWP1:C4_01470W_A:NIP1:C4_01500W_A:C4_01560C_A:TOA2:HSX11:TIM10:ZCF25:C4_02400C_A:SLX4:HIT1:C4_02770C_A:SAS10:RPC53:HCA4:C4_02850W_A:ZUO1:C4_02880C_A:NAN1:C4_03140C_A:C4_03170W_A:DAO2:C4_03410W_A:RAD2:C4_03720C_A:C4_03730C_A:C4_03740W_A:NUP84:C4_03830W_A:RRP42:C4_03870C_A:RAM1:FGR28:ECM1:C4_04130W_A:DUO1:C4_04390W_A:SAP10:C4_04520W_A:SSZ1:BAS1:C4_04810C_A:AGP3:RPL30:C4_05230C_A:C4_05260W_A:C4_05360C_A:OFD1:HOM3:C4_05650W_A:C4_05890W_A:TRP5:C4_06210C_A:C4_06410W_A:NOP1:C4_06790W_A:C4_06950W_A:MET16:C4_07060W_A:TRP4:C4_07140W_A:C4_07150W_A:C5_00030W_A:C5_00260W_A:C5_00280C_A:DUS4:C5_00320W_A:MRP17:MET14:IFG3:EXO1:RMT2:SPE2:C5_00820W_A:C5_00920W_A:BUD23:CYC3:C5_01140C_A:PUS4:C5_01430C_A:FYV5:SPB4:C5_01610W_A:CCN1:C5_01700W_A:C5_01930W_A:C5_02010C_A:C5_02070C_A:RIX7:THR4:PDE1:C5_02440C_A:TIF5:SPR28:UTP13:C5_02590C_A:BUR2:C5_02740W_A:CKB2:MSW1:C5_02780W_A:C5_02820C_A:C5_03010W_A:RMS1:CAM1-1:C5_03290C_A:PCL1:RPO26:FUR1:C5_03400C_A:C5_03460C_A:C5_03530C_A:MDJ1:C5_03640W_A:C5_03700C_A:C5_03840W_A:HAM1:C5_03920C_A:C5_03970W_A:C5_04120C_A:SAH1:C5_04290C_A:C5_04340W_A:URA7:CSU57:MSM1:C5_04720C_A:HYS2:HAS1:C5_04840C_A:C5_04910W_A:GRF10:C5_05250C_A:SDH4:HIS1:C5_05340W_A:ILV3:DNA2:NOP5:C6_00530C_A:C6_00560W_A:FCA1:C6_00640C_A:CDC14:C6_00760W_A:TOP1:C6_01040C_A:CIP1:C6_01120C_A:CIC1:PMP1:TIF3:RAD18:HCH1:C6_01890C_A:C6_01980C_A:SWD3:C6_02230W_A:RPL10A:ALG11:C6_02280W_A:C6_02290C_A:C6_02300C_A:C6_02350C_A:C6_02370C_A:C6_02430W_A:PRP45:C6_02690C_A:MRT4:C6_02900C_A:POP4:C6_03210C_A:ARP4:C6_03380W_A:C6

						_03390W_A:C6_03440W_A:NAM2:SPB1:C6_04240W_A:NOP8:ILS1:C6_04530C_A:C7_00160C_A:C7_00220W_A:C7_00330C_A:DCC1:PSF3:THG1:RPA135:NOP15:YML6:C7_01020C_A:C7_01030C_A:DBP7:C7_01210C_A:ASC1:C7_01270C_A:PSF1:C7_01360C_A:C7_01400C_A:GIR2:PRT1:FLU1:C7_01570C_A:SMC6:C7_01600W_A:C7_01950W_A:LIG1:C7_02340C_A:C7_02460C_A:C7_02660C_A:C7_02930C_A:IFM1:C7_03400C_A:ISY1:ENP2:ARG4:C7_03590C_A:ENP1:HIS7:MRP7:C7_03850W_A:C7_03880C_A:C7_04150W_A:UTP18:MIS12:MMS22:RTT109:CR_00430C_A:CR_00460C_A:MRPL6:ADE1:BUD22:PWP2:CR_00830W_A:TRM1:DAL81:CTF18:CR_01260W_A:CR_01320C_A:CR_01410C_A:PRS:CR_01550C_A:TRP2:CR_01600C_A:MET6:CR_01670W_A:CDC60:CR_01700C_A:CR_01710W_A:CR_01780W_A:CR_01950W_A:CR_02030C_A:MCM6:MET2:ERG25:CR_02420W_A:CR_02430C_A:CHO2:CR_02550C_A:PAN6:RRP9:RPC31:CR_02890C_A:SRP40:ARC1:SMC5:CR_03110W_A:CR_03200C_A:CR_03230W_A:CR_03340C_A:CR_03360W_A:KTI12:MTG1:CR_03400W_A:CR_03440W_A:NCS2:YVH1:SGD1:SMC1:PRP42:CR_03760W_A:CR_03940W_A:CR_04110W_A:CR_04160C_A:CR_04170W_A:CR_04240C_A:CR_04300W_A:CR_04500C_A:CR_04560C_A:CR_04710W_A:ADE6:RPB8:CR_04920W_A:NOC2:CR_05550C_A:RNR3:MTG2:DBP8:RFX1:RPL7:CR_06230W_A:FGR50:CR_06450W_A:RRN11:HSP60:CR_06680C_A:NMD3:CR_06840W_A:ATX1:CR_06970C_A:CR_06980W_A:POL2:MIS11:CR_07030C_A:URA2:CR_07310W_A:PIF1:CR_07480W_A:FUN30:CR_07640C_A:ARO2:CDC45:DBP6:ASF1:IMP4:CR_08000C_A:NOP10:SSB1:RIO2:ATS1:CR_08330W_A:CYS3:RPP2A:GSH2:CR_08410W_A:TSR1:CR_08940W_A:MPS1:CR_09010C_A:CR_09310W_A:SSF1:CR_09740W_A:MED21:CR_09800C_A:DPB2:SIK1:CR_09990W_A:CR_10180W_A:UTP5:CR_10260W_A:CR_10410C_A:CR_10470C_A:MCD1:DRS1:YHB4:PRO1:LTV1:POP3

71704	organic substance metabolic process	804 out of 1061 genes, 75.8%	3737 out of 6473 background genes, 57.7%	1.26E-37	0.00%	RIM8:LEU4:HBR1:C1_00510W_A:CNS1:RPO41:SPE1:NRM1:RRS1:TRI1:NMA111:HMT1:C1_01150C_A:C1_01160C_A:TRM2:ABC1:C1_01530C_A:CYS4:C1_02090C_A:ABP140:CWH8:C1_02380C_A:C1_02390W_A:C1_02450C_A:SNZ1:SNO1:HOM6:LYS2:PDE2:MIA40:C1_02900C_A:C1_02970W_A:TSR2:C1_03260W_A:C1_03370W_A:C1_03540C_A:C1_03630W_A:C1_03790C_A:RPP1:C1_03830C_A:KTR4:C1_04040C_A:C1_04120C_A:ERB1:RPN4:NOP4:C1_04510W_A:C1_04530C_A:C1_04710C_A:MRPL37:C1_04970W_A:RHD1:ARG2:RRP6:REI1:TRY2:ARO4:C1_05220C_A:C1_05270C_A:C1_05360C_A:C1_05380C_A:RSM22:RPS27A:C1_05630C_A:FAL1:C1_05650W_A:RAD10:C1_05930C_A:C1_05990C_A:BUD16:C1_06530C_A:C1_06540C_A:NOP6:C1_06760C_A:RIA1:YMC1:C1_07340W_A:C1_07470C_A:LHP1:C1_07510W_A:C1_07660W_A:ADE4:ECO1:C1_07850C_A:GCS1:ADE5,7:MRPL3:ERD1:C1_07950C_A:C1_07960W_A:FUN12:DRG1:CAT8:MLT1:DIP2:SAM4:GCR3:UPC2:C1_08520C_A:GCD6:C1_08630W_A:MSS116:C1_08890C_A:C1_09040C_A:CEF1:C1_09390W_A:GUA1:C1_09610W_A:C1_09710C_A:C1_09790C_A:C1_09840C_A:BUD31:C1_09910C_A:PDC2:DBP3:MNN23:C1_10080W_A:C1_10200C_A:C1_10340W_A:FCY23:C1_10620W_A:APN2:RPA190:C1_10690W_A:C1_10920W_A:C1_10950C_A:C1_10970W_A:C1_11000C_A:PET127:FGR39:NEP1:KRR1:SAM2:GAR1:C1_11610C_A:C1_11790W_A:MUP1:C1_11880W_A:C1_11900C_A:C1_11910W_A:SER33:TRP3:C1_12280C_A:CSI2:MRPS9:C1_12350W_A:WRS1:C1_12440W_A:C1_12570C_A:C1_12630C_A:C1_12640W_A:C1_12670C_A:C1_12750C_A:C1_12760W_A:FEN1:C1_12820C_A:RPL14:C1_13060C_A:REX3:C1_13330C_A:RMP1:C1_13380W_A:KNS1:MCU1:C1_13560W_A:C1_13820C_A:MET3:YBP1:C1_14080W_A:C1_14160W_A:C1_14330W_A:MPP10:FAV3:C2_00200W_A:C2_00280C_A:C2_00390C_A:C2_00410C_A:C2_00620C_A:C2_00820W_A:C2_00840W_A:MXR1:C2_01060C_A:C2_01070W_A:C2_01220W_A:C2_01240C_A:ESC4:CLB2:C2_01420C_A:APT1:REX2:C2_01740C_A:C2_01820C_A:PRP39:C2_01860C_A:C2_01870C_A:ARO3:C2_02120W_A:SAM37:C2_02270C_A:C2_02420C_A:UTP21:UTP22:C2_02490C_A:PRS1:C2_02540W_A:C2_02630W_A:PDR17:SER2:C2_02930C_A:MET1:BNA31:SMP3:C2_03130W_A:PRP3:C2_03560C_A:MNN42:C2_03700W_A:C2_03830W_A:GPI13:SCH9:C2_03950W_A:C2_04120C_A:UGA1:TBF1:BAT21:C2_04340C_A:SUV3:RIM2:MED8:C2_04570W_A:SNU114:C2_04700C_A:C2_04820W_A:PZF1:CKA2:C2_04990W_A:C2_05050C_A:C2_05080C_A:MAK5:C2_05160C_A:CDC21:SGO1:RPF2:C2_05270W_A:RPC40:KTI11:C2_05670C_A:C2_05750W_A:C2_05830C_A:C2_05840W_A:IDP1:C2_05980C_A:LAS1:CSM3:ECM17:ILV1:SER1:MAK16:C2_06480W_A:C2_06520C_A:C2_06530W_A:VAS1:C2_06650C_A:C2_06660W_A:AMO2:RPL11:FHL1:C2_06850W_A:C2_06950C_A:SPE3:AAH1:RCK2:DQD1:C2_07270W_A:NOC4:C2_07360W_A:C2_07410W_A:RCL1:C2_07680W_A:PHO86:C2_07860W_A:PRS5:C2_07920W_A:NSA1:C2_08180C_A:C2_08200W_A:EAF3:RRP8:C2_08530C_A:MSU1:UTP15:MFG1:APE3:CDC47:ASN1:C2_09160W_A:C2_09180W_A:MED18:C2_09310C_A:PES1:TAZ1:RR

						P15:C2_09500W_A:C2_09660W_A:LEU42:SMM1:C2_09920W_A:CWC22:UBP8:GPD1:PUS7:HEM3:C2_10740C_A:RPC11:C2_10810W_A:RPS24:C3_00100W_A:ULP3:RHR2:UTP8:BUD21:SOF1:C3_00660W_A:C3_00830C_A:BMS1:ARP9:UTP20:NCE103:C3_01310W_A:URA3:MET18:RKI1:RPS7A:C3_01520C_A:C3_01560W_A:PPT1:SCM3:C3_02020W_A:C3_02040C_A:UTP4:C3_02180C_A:ILV2:C3_02350W_A:PHA2:DDC1:C3_02670W_A:SMC3:C3_02750W_A:C3_02840W_A:C3_02850C_A:MET13:C3_03070W_A:C3_03110W_A:C3_03330C_A:YAH1:C3_03470W_A:ULP1:C3_03560W_A:HBR3:RAD53:ATF1:C3_04370C_A:C3_04380C_A:ADE2:MAK21:RPS15:RPP2B:ARV1:SFP1:NOP14:CEM1:C3_05140C_A:C3_05160C_A:WOR2:RPL8B:C3_05280C_A:C3_05380W_A:GPI1:C3_05440C_A:C3_05510W_A:QDR2:BAT22:C3_05800W_A:C3_05860C_A:TRM12:C3_05900W_A:CTA4:NOG1:C3_06150W_A:MMS21:CYM1:C3_06240C_A:C3_06270C_A:DOT1:ISW2:C3_06370C_A:NSA2:OPI3:C3_06600C_A:FUN31:C3_06630W_A:PRP5:C3_06830C_A:RPS12:NOP13:C3_07400W_A:C3_07550C_A:C3_07570C_A:RIT1:RAD4:UTP9:C3_07800C_A:HIS4:MET15:DUR4:RLP24:NMD5:HIS5:C4_00690C_A:C4_00700C_A:C4_00740W_A:MLH1:C4_00800W_A:C4_00810C_A:C4_00880W_A:ARO1:C4_00940W_A:PTC8:RAT1:AAT22:C4_01280C_A:PGA53:ILV6:PWP1:C4_01470W_A:NIP1:C4_01500W_A:C4_01560C_A:TOA2:HSX11:TIM10:ZCF25:C4_02400C_A:SLX4:HIT1:C4_02770C_A:SAS10:RPC53:HCA4:C4_02850W_A:ZUO1:C4_02880C_A:NAN1:C4_03140C_A:C4_03170W_A:DAO2:C4_03410W_A:RAD2:C4_03720C_A:C4_03730C_A:C4_03740W_A:NUP84:C4_03830W_A:RRP42:C4_03870C_A:RAM1:FGR28:ECM1:C4_04130W_A:DUO1:C4_04390W_A:SAP10:C4_04520W_A:SSZ1:BAS1:C4_04810C_A:AGP3:RPL30:C4_05230C_A:C4_05260W_A:C4_05360C_A:OFD1:HOM3:C4_05650W_A:C4_05890W_A:TRP5:C4_06210C_A:C4_06410W_A:NOP1:C4_06790W_A:C4_06950W_A:MET16:C4_07060W_A:TRP4:C4_07140W_A:C4_07150W_A:C5_00030W_A:C5_00260W_A:C5_00280C_A:DUS4:C5_00320W_A:MRP17:MET14:IFG3:EXO1:RMT2:SPE2:C5_00820W_A:C5_00920W_A:BUD23:CYC3:C5_01140C_A:PUS4:C5_01430C_A:FYV5:SPB4:C5_01610W_A:CCN1:C5_01700W_A:C5_01930W_A:C5_02010C_A:C5_02070C_A:RIX7:THR4:PDE1:C5_02440C_A:TIF5:SPR28:UTP13:C5_02590C_A:BUR2:C5_02740W_A:CKB2:MSW1:C5_02780W_A:C5_02820C_A:C5_03010W_A:RMS1:CAM1-1:C5_03290C_A:PCL1:RPO26:FUR1:C5_03400C_A:C5_03460C_A:C5_03530C_A:C5_03550W_A:MDJ1:C5_03640W_A:C5_03700C_A:C5_03840W_A:HAM1:C5_03920C_A:C5_03970W_A:C5_04120C_A:SAH1:C5_04290C_A:C5_04340W_A:URA7:CSU57:MSM1:C5_04720C_A:HYS2:HAS1:C5_04840C_A:C5_04910W_A:C5_05000C_A:GRF10:C5_05250C_A:SDH4:HIS1:C5_05340W_A:ILV3:DNA2:NOP5:C6_00530C_A:C6_00560W_A:FCA1:C6_00640C_A:CDC14:C6_00760W_A:TOP1:HAL22:C6_01040C_A:CIP1:C6_01120C_A:CIC1:EBP1:C6_01300W_A:PMP1:TIF3:RAD18:HCH1:C6_01890C_A:C6_01980C_A:SWD3:C6_02230W_A:RPL10A:ALG11:C6_02280W_A:C6_02290C_A:C6_02

						300C_A:C6_02350C_A:C6_02370C_A:C6_02420W_A:C6_02430W_A:PRP45:C6_02690C_A:MRT4:C6_02900C_A:POP4:C6_03210C_A:BMT4:ARP4:C6_03380W_A:C6_03390W_A:C6_03440W_A:NAM2:PAD1:SPB1:C6_04240W_A:NOP8:ILS1:C6_04530C_A:C7_00160C_A:C7_00220W_A:C7_00330C_A:DCC1:PSF3:THG1:RPA135:NOP15:YML6:C7_01020C_A:C7_01030C_A:DBP7:C7_01210C_A:ASC1:C7_01270C_A:PSF1:C7_01360C_A:C7_01400C_A:GIR2:PRT1:FLU1:C7_01570C_A:SMC6:C7_01600W_A:C7_01950W_A:LIG1:C7_02340C_A:C7_02460C_A:C7_02660C_A:C7_02930C_A:IFM1:C7_03400C_A:ISY1:ENP2:ARG4:C7_03590C_A:ENP1:HIS7:MRP7:C7_03850W_A:C7_03880C_A:C7_04150W_A:UTP18:MIS12:MMS22:RTT109:CR_00430C_A:CR_00460C_A:MRPL6:ADE1:BUD22:PWP2:CR_00830W_A:TRM1:DAL81:CTF18:CR_01260W_A:CR_01320C_A:CR_01410C_A:PRS:CR_01550C_A:TRP2:CR_01600C_A:MET6:CR_01670W_A:CDC60:CR_01700C_A:CR_01710W_A:CR_01780W_A:CR_01950W_A:CR_02030C_A:MCM6:MET2:ERG25:CR_02420W_A:CR_02430C_A:CHO2:CR_02550C_A:PAN6:RRP9:RPC31:CR_02890C_A:SRP40:ARC1:SMC5:CR_03110W_A:CR_03200C_A:CR_03230W_A:CR_03340C_A:CR_03360W_A:KTI12:MTG1:CR_03400W_A:CR_03440W_A:NCS2:YVH1:SGD1:SMC1:PRP42:CR_03760W_A:CR_03940W_A:MEX67:CR_04110W_A:CR_04160C_A:CR_04170W_A:QDR1:CR_04240C_A:CR_04300W_A:CR_04500C_A:CR_04560C_A:CR_04710W_A:ADE6:RPB8:CR_04920W_A:NOC2:CR_05550C_A:RNR3:MTG2:DBP8:RFX1:RPL7:CR_06230W_A:FGR50:CR_06450W_A:RRN11:HSP60:CR_06680C_A:NMD3:CR_06840W_A:ATX1:CR_06970C_A:CR_06980W_A:POL2:MIS11:CR_07030C_A:URA2:CR_07310W_A:PIF1:CR_07480W_A:FUN30:CR_07640C_A:ARO2:CDC45:DBP6:ASF1:IMP4:CR_08000C_A:NOP10:SSB1:RIO2:ATS1:CR_08330W_A:CYS3:RPP2A:GSH2:CR_08410W_A:TSR1:CR_08940W_A:MPS1:CR_09010C_A:CR_09310W_A:ELF1:SSF1:CR_09740W_A:MED21:CR_09800C_A:DPB2:SIK1:CR_09990W_A:CR_10180W_A:UTP5:CR_10260W_A:CR_10410C_A:CR_10470C_A:MCD1:DRS1:YHB4:PRO1:LTV1:POP3

71840	cellular component organization or biogenesis	561 out of 1061 genes, 52.9%	2243 out of 6473 background genes, 34.7%	1.50E-37	0.00%	HBR1:CNS1:RPO41:NRM1:RRS1:TRI1:C1_01160C_A:C1_02090C_A:ABP140:C1_02380C_A:C1_02450C_A:PDE2:MIA40:C1_02900C_A:TSR2:ARX1:C1_03260W_A:C1_03540C_A:C1_03630W_A:C1_03790C_A:RPP1:C1_03830C_A:C1_04040C_A:C1_04120C_A:ERB1:C1_04340C_A:NOP4:C1_04510W_A:C1_04710C_A:C1_04970W_A:RRP6:REI1:TRY2:C1_05210C_A:C1_05220C_A:C1_05270C_A:C1_05360C_A:C1_05380C_A:MTM1:RSM22:RPS27A:FAL1:C1_05650W_A:C1_06530C_A:C1_06540C_A:NOP6:C1_06760C_A:MEF2:RIA1:C1_07340W_A:C1_07470C_A:C1_07510W_A:C1_07660W_A:ECO1:ERD1:C1_07950C_A:C1_07960W_A:FUN12:CAT8:MLT1:DIP2:GCR3:GCD6:C1_08630W_A:MSS116:JIP5:NAR1:C1_09040C_A:CEF1:C1_09390W_A:YTM1:C1_09620C_A:C1_09710C_A:C1_09790C_A:C1_09840C_A:PDC2:DBP3:MNN23:FCY23:C1_10620W_A:RPA190:C1_10920W_A:C1_10950C_A:C1_10970W_A:C1_11000C_A:NEP1:KRR1:GAR1:MUP1:C1_11900C_A:C1_11910W_A:TRP3:C1_12280C_A:CSI2:C1_12350W_A:C1_12440W_A:C1_12570C_A:C1_12630C_A:C1_12640W_A:C1_12680W_A:C1_12750C_A:C1_12760W_A:FEN1:C1_12820C_A:C1_13010W_A:REX3:RMP1:C1_13370W_A:C1_13380W_A:AGC1:COX19:KNS1:MCU1:C1_13560W_A:C1_13820C_A:C1_14080W_A:C1_14330W_A:MPP10:FAV3:C2_00200W_A:C2_00280C_A:C2_00410C_A:C2_00810C_A:C2_00820W_A:C2_00840W_A:C2_01070W_A:C2_01220W_A:C2_01240C_A:CLB2:C2_01420C_A:REX2:C2_01820C_A:PRP39:C2_01860C_A:C2_02120W_A:SAM37:C2_02420C_A:UTP21:UTP22:PRS1:C2_02540W_A:C2_02630W_A:PDR17:C2_02930C_A:MET1:SMP3:PRP3:MNN42:SCH9:C2_04120C_A:TBF1:SUV3:RIM2:C2_04570W_A:SNU114:C2_04700C_A:C2_04820W_A:PZF1:CKA2:C2_05050C_A:C2_05080C_A:MAK5:C2_05160C_A:SGO1:RPF2:C2_05270W_A:C2_05670C_A:C2_05750W_A:C2_05830C_A:C2_05840W_A:LAS1:CSM3:ECM17:MAK16:RTA3:C2_06520C_A:C2_06530W_A:C2_06650C_A:C2_06660W_A:RPL11:FHL1:C2_06850W_A:C2_06950C_A:RCK2:NOC4:C2_07360W_A:RCL1:PRS5:C2_07920W_A:NSA1:KRE30:RPS10:C2_08180C_A:EAF3:RRP8:C2_08530C_A:MSU1:SMC2:UTP15:MFG1:APE3:CDC47:C2_09160W_A:C2_09180W_A:C2_09290W_A:MED18:C2_09310C_A:PES1:TAZ1:RRP15:C2_09660W_A:SMM1:TIM23:C2_09920W_A:CWC22:GPD1:PUS7:C2_10740C_A:C2_10810W_A:RPS24:C3_00100W_A:ULP3:UTP8:BUD21:SOF1:C3_00660W_A:C3_00830C_A:BMS1:ARP9:UTP20:NCE103:C3_01310W_A:RPS7A:C3_01560W_A:SCM3:C3_02020W_A:C3_02040C_A:UTP4:ILV2:C3_02350W_A:DDC1:C3_02670W_A:SMC3:C3_02840W_A:C3_02850C_A:C3_03070W_A:C3_03110W_A:C3_03330C_A:YAH1:C3_03470W_A:HBR3:C3_04370C_A:C3_04380C_A:MAK21:RPS15:ARV1:SFP1:NOP14:CEM1:C3_05160C_A:WOR2:RPL8B:C3_05280C_A:C3_05380W_A:C3_05440C_A:QDR2:SAM50:C3_05800W_A:C3_05860C_A:C3_05900W_A:NOG1:C3_06150W_A:DOT1:ISW2:C3_06370C_A:NSA2:C3_06600C_A:C3_06630W_A:PRP5:NOP13:C3_07550C_A:C3_07570C_A:RAD4:UTP9:C3_07800C_A:DUR4:RLP24:NMD5:C4_00690C_A:C4_00700C_A:C4_00740W_A:MLH1:C4_00800W_A:C4_00810C_A:C4_00880W_A:RAT1:C4_0113

						0C_A:C4_01280C_A:PWP1:C4_01500W_A:C4_01560C_A:TOA2:TIM10:C4_02400C_A:SLX4:C4_02720C_A:HIT1:C4_02770C_A:SAS10:RPC53:HCA4:ZUO1:C4_02880C_A:NAN1:C4_03140C_A:C4_03170W_A:DAO2:C4_03720C_A:C4_03740W_A:NUP84:C4_03830W_A:RRP42:C4_03870C_A:RAM1:ECM1:C4_04130W_A:DUO1:SAP10:C4_04520W_A:SSZ1:BAS1:C4_04820C_A:AGP3:RPL30:C4_05010W_A:C4_05230C_A:C4_05260W_A:C4_05360C_A:OFD1:TUB4:C4_05650W_A:C4_06210C_A:C4_06410W_A:C4_06450W_A:NOP1:C4_06790W_A:C4_06950W_A:C4_07060W_A:C4_07140W_A:C4_07150W_A:C5_00260W_A:C5_00280C_A:MRP17:EXO1:RMT2:C5_00920W_A:BUD23:CLN3:CYC3:C5_01140C_A:PUS4:C5_01430C_A:FYV5:SPB4:C5_01930W_A:C5_02010C_A:C5_02070C_A:RIX7:PDE1:C5_02440C_A:TIF5:SPR28:UTP13:BUR2:C5_02740W_A:CKB2:C5_02780W_A:C5_03010W_A:RMS1:PCL1:C5_03400C_A:C5_03550W_A:MDJ1:C5_03920C_A:C5_03970W_A:C5_04120C_A:C5_04720C_A:HAS1:C5_04840C_A:C5_04910W_A:C5_05010W_A:GRF10:SDH4:C5_05340W_A:DNA2:NOP5:C6_00560W_A:CDC14:TOP1:C6_01040C_A:C6_01120C_A:CIC1:QDR3:TIF3:C6_01890C_A:C6_02150C_A:SWD3:C6_02230W_A:RPL10A:ALG11:C6_02290C_A:C6_02430W_A:PRP45:C6_02690C_A:MRT4:C6_02900C_A:POP4:C6_02970C_A:C6_03210C_A:ARP4:C6_03380W_A:C6_03390W_A:C6_03440W_A:NAM2:NOG2:SPB1:C6_04240W_A:NOP8:C7_00160C_A:C7_00220W_A:C7_00330C_A:DCC1:THG1:RPA135:NOP15:C7_01030C_A:DBP7:C7_01210C_A:C7_01270C_A:C7_01360C_A:C7_01400C_A:GIR2:FLU1:C7_01570C_A:C7_01600W_A:C7_01950W_A:LIG1:C7_02460C_A:C7_02660C_A:C7_02930C_A:C7_03400C_A:ISY1:ENP2:C7_03590C_A:ENP1:C7_03850W_A:C7_03880C_A:MAM33:C7_04150W_A:UTP18:RTT109:CR_00430C_A:CR_00460C_A:BUD22:PWP2:CR_00830W_A:DAL81:CTF18:CR_01320C_A:CR_01410C_A:CR_01550C_A:CR_01600C_A:CR_01670W_A:CR_01700C_A:CR_01710W_A:CR_01780W_A:CR_02030C_A:MCM6:CR_02420W_A:CR_02550C_A:RRP9:CR_02890C_A:SRP40:ARC1:SMC5:CR_03200C_A:CR_03230W_A:CR_03340C_A:CR_03360W_A:KTI12:NCS2:YVH1:SGD1:SMC1:PRP42:CR_03760W_A:CR_03940W_A:MEX67:CR_04110W_A:CR_04170W_A:QDR1:CR_04240C_A:CR_04500C_A:CR_04560C_A:CR_04710W_A:CR_04920W_A:BIR1:NOC2:CR_05550C_A:RNR3:MTG2:DBP8:SDA1:RFX1:RPL7:CR_06230W_A:FGR50:CR_06450W_A:RRN11:HSP60:CR_06680C_A:NMD3:CR_06740W_A:CR_06840W_A:CR_06970C_A:POL2:CR_07030C_A:CR_07310W_A:PIF1:FUN30:CR_07640C_A:CDC45:DBP6:ASF1:IMP4:CR_08000C_A:NOP10:SSB1:RIO2:ATS1:CR_08330W_A:CR_08410W_A:TSR1:CR_08500W_A:MPS1:ELF1:SSF1:CR_09740W_A:MED21:CR_09800C_A:DPB2:SIK1:CR_10180W_A:UTP5:CR_10260W_A:CR_10410C_A:CR_10470C_A:MCD1:DRS1:LTV1:POP3

22618	protein-RNA complex assembly	110 out of 1061 genes, 10.4%	193 out of 6473 background genes, 3.0%	8.24E-36	0.00%	C1_02900C_A:C1_04040C_A:C1_04120C_A:ERB1:NOP4:C1_04710C_A:C1_04970W_A:RRP6:C1_05360C_A:RPS27A:C1_06760C_A:ERD1:FUN12:JIP5:C1_09040C_A:CEF1:C1_09710C_A:RPA190:C1_10970W_A:TRP3:CSI2:C1_12440W_A:C1_12750C_A:C1_12760W_A:C1_14080W_A:C2_00200W_A:C2_00280C_A:C2_01070W_A:PRP39:UTP22:C2_02540W_A:PRP3:SNU114:MAK5:C2_05160C_A:RPF2:C2_05750W_A:C2_05830C_A:C2_06650C_A:RPL11:C2_09180W_A:C2_10740C_A:C3_00100W_A:C3_00660W_A:BMS1:ARP9:C3_01560W_A:C3_02020W_A:C3_02040C_A:C3_02350W_A:C3_02670W_A:SMC3:C3_03330C_A:C3_04370C_A:MAK21:ARV1:C3_05160C_A:QDR2:NOG1:C3_06370C_A:PRP5:RLP24:HIT1:C4_05010W_A:C4_05230C_A:C4_06410W_A:RMT2:SPB4:C5_01930W_A:C5_02010C_A:C5_02070C_A:TIF5:C5_04720C_A:HAS1:C5_05340W_A:CIC1:TIF3:C6_02230W_A:PRP45:SPB1:RPA135:DBP7:FLU1:C7_03400C_A:ISY1:ENP2:UTP18:BUD22:PWP2:CR_00830W_A:CR_01410C_A:CR_01550C_A:CR_01700C_A:ARC1:YVH1:SGD1:CR_03940W_A:CR_04110W_A:CR_04170W_A:NOC2:CR_05550C_A:SDA1:CR_06680C_A:TSR1:ELF1:SSF1:CR_09800C_A:CR_10470C_A:DRS1:LTV1

43170	macromolecule metabolic process	716 out of 1061 genes, 67.5%	3194 out of 6473 background genes, 49.3%	1.05E-35	0.00%	RIM8:LEU4:HBR1:CNS1:RPO41:NRM1:RRS1:TRI1:NMA111:HMT1:C1_01150C_A:C1_01160C_A:TRM2:ABC1:C1_01530C_A:C1_02090C_A:ABP140:CWH8:C1_02380C_A:C1_02390W_A:C1_02450C_A:HOM6:PDE2:MIA40:C1_02900C_A:TSR2:C1_03260W_A:C1_03370W_A:C1_03540C_A:C1_03630W_A:C1_03790C_A:RPP1:C1_03830C_A:KTR4:C1_04040C_A:C1_04120C_A:ERB1:RPN4:NOP4:C1_04510W_A:C1_04530C_A:C1_04710C_A:MRPL37:C1_04970W_A:RHD1:RRP6:REI1:TRY2:C1_05220C_A:C1_05270C_A:C1_05360C_A:C1_05380C_A:RSM22:RPS27A:C1_05630C_A:FAL1:C1_05650W_A:RAD10:C1_06530C_A:C1_06540C_A:NOP6:C1_06760C_A:RIA1:C1_07340W_A:C1_07470C_A:LHP1:C1_07510W_A:C1_07660W_A:ECO1:C1_07850C_A:GCS1:MRPL3:ERD1:C1_07950C_A:C1_07960W_A:FUN12:DRG1:CAT8:MLT1:DIP2:GCR3:C1_08520C_A:GCD6:C1_08630W_A:MSS116:C1_08890C_A:C1_09040C_A:CEF1:C1_09390W_A:C1_09610W_A:C1_09710C_A:C1_09790C_A:C1_09840C_A:BUD31:C1_09910C_A:PDC2:DBP3:MNN23:C1_10080W_A:C1_10200C_A:C1_10340W_A:FCY23:C1_10620W_A:APN2:RPA190:C1_10690W_A:C1_10920W_A:C1_10950C_A:C1_10970W_A:C1_11000C_A:PET127:FGR39:NEP1:KRR1:SAM2:GAR1:MUP1:C1_11880W_A:C1_11900C_A:C1_11910W_A:SER33:TRP3:C1_12280C_A:CSI2:MRPS9:C1_12350W_A:WRS1:C1_12440W_A:C1_12570C_A:C1_12630C_A:C1_12640W_A:C1_12670C_A:C1_12750C_A:C1_12760W_A:FEN1:C1_12820C_A:RPL14:C1_13060C_A:REX3:RMP1:C1_13380W_A:KNS1:MCU1:C1_13560W_A:C1_13820C_A:YBP1:C1_14080W_A:C1_14160W_A:C1_14330W_A:MPP10:FAV3:C2_00200W_A:C2_00280C_A:C2_00390C_A:C2_00410C_A:C2_00620C_A:C2_00820W_A:C2_00840W_A:MXR1:C2_01060C_A:C2_01070W_A:C2_01220W_A:C2_01240C_A:ESC4:CLB2:C2_01420C_A:REX2:C2_01740C_A:C2_01820C_A:PRP39:C2_01860C_A:C2_01870C_A:C2_02120W_A:SAM37:C2_02270C_A:C2_02420C_A:UTP21:UTP22:C2_02490C_A:PRS1:C2_02540W_A:C2_02630W_A:PDR17:C2_02930C_A:MET1:SMP3:C2_03130W_A:PRP3:C2_03560C_A:MNN42:C2_03700W_A:C2_03830W_A:GPI13:SCH9:C2_03950W_A:C2_04120C_A:TBF1:BAT21:SUV3:RIM2:MED8:C2_04570W_A:SNU114:C2_04700C_A:C2_04820W_A:PZF1:CKA2:C2_04990W_A:C2_05050C_A:C2_05080C_A:MAK5:C2_05160C_A:CDC21:SGO1:RPF2:C2_05270W_A:RPC40:KTI11:C2_05670C_A:C2_05750W_A:C2_05830C_A:C2_05840W_A:IDP1:LAS1:CSM3:ECM17:SER1:MAK16:C2_06480W_A:C2_06520C_A:C2_06530W_A:VAS1:C2_06650C_A:C2_06660W_A:RPL11:FHL1:C2_06850W_A:C2_06950C_A:SPE3:RCK2:C2_07270W_A:NOC4:C2_07360W_A:RCL1:C2_07680W_A:PHO86:C2_07860W_A:PRS5:C2_07920W_A:NSA1:C2_08180C_A:C2_08200W_A:EAF3:RRP8:C2_08530C_A:MSU1:UTP15:MFG1:APE3:CDC47:C2_09160W_A:C2_09180W_A:MED18:C2_09310C_A:PES1:TAZ1:RRP15:C2_09500W_A:C2_09660W_A:SMM1:C2_09920W_A:CWC22:UBP8:PUS7:HEM3:C2_10740C_A:RPC11:C2_10810W_A:RPS24:C3_00100W_A:ULP3:UTP8:BUD21:SOF1:C3_00660W_A:C3_00830C_A:BMS1:ARP9:UTP20:NCE103:C3_01310W_A:MET18:RPS7A:C3_01520C_A:C3_01560W_A:PPT1:SCM

						3:C3_02020W_A:C3_02040C_A:UTP4:C3_02180C_A:ILV2:C3_02350W_A:DDC1:C3_02670W_A:SMC3:C3_02750W_A:C3_02840W_A:C3_02850C_A:C3_03070W_A:C3_03110W_A:C3_03330C_A:ULP1:C3_03560W_A:HBR3:RAD53:C3_04370C_A:C3_04380C_A:MAK21:RPS15:RPP2B:ARV1:SFP1:NOP14:CEM1:C3_05140C_A:C3_05160C_A:WOR2:RPL8B:C3_05280C_A:C3_05380W_A:GPI1:C3_05440C_A:C3_05510W_A:QDR2:C3_05800W_A:C3_05860C_A:TRM12:C3_05900W_A:CTA4:NOG1:C3_06150W_A:MMS21:CYM1:C3_06240C_A:C3_06270C_A:DOT1:ISW2:C3_06370C_A:NSA2:C3_06600C_A:FUN31:C3_06630W_A:PRP5:C3_06830C_A:RPS12:NOP13:C3_07400W_A:C3_07550C_A:C3_07570C_A:RIT1:RAD4:UTP9:C3_07800C_A:DUR4:RLP24:NMD5:HIS5:C4_00690C_A:C4_00700C_A:C4_00740W_A:MLH1:C4_00800W_A:C4_00810C_A:C4_00880W_A:ARO1:C4_00940W_A:PTC8:RAT1:C4_01280C_A:PGA53:PWP1:C4_01470W_A:NIP1:C4_01500W_A:C4_01560C_A:TOA2:HSX11:TIM10:ZCF25:C4_02400C_A:SLX4:HIT1:C4_02770C_A:SAS10:RPC53:HCA4:C4_02850W_A:ZUO1:C4_02880C_A:NAN1:C4_03140C_A:C4_03170W_A:DAO2:C4_03410W_A:RAD2:C4_03720C_A:C4_03730C_A:C4_03740W_A:NUP84:C4_03830W_A:RRP42:C4_03870C_A:RAM1:FGR28:ECM1:C4_04130W_A:DUO1:C4_04390W_A:SAP10:C4_04520W_A:SSZ1:BAS1:C4_04810C_A:AGP3:RPL30:C4_05230C_A:C4_05260W_A:C4_05360C_A:OFD1:C4_05650W_A:C4_05890W_A:C4_06210C_A:C4_06410W_A:NOP1:C4_06790W_A:C4_06950W_A:C4_07060W_A:C4_07140W_A:C4_07150W_A:C5_00030W_A:C5_00280C_A:DUS4:C5_00320W_A:MRP17:MET14:EXO1:RMT2:C5_00820W_A:C5_00920W_A:BUD23:CYC3:C5_01140C_A:PUS4:C5_01430C_A:FYV5:SPB4:C5_01610W_A:CCN1:C5_01700W_A:C5_01930W_A:C5_02010C_A:C5_02070C_A:RIX7:C5_02440C_A:TIF5:SPR28:UTP13:C5_02590C_A:BUR2:C5_02740W_A:CKB2:MSW1:C5_02780W_A:C5_02820C_A:C5_03010W_A:RMS1:CAM1-1:C5_03290C_A:PCL1:RPO26:FUR1:C5_03400C_A:C5_03460C_A:C5_03530C_A:C5_03550W_A:MDJ1:C5_03640W_A:C5_03700C_A:C5_03920C_A:C5_03970W_A:C5_04120C_A:C5_04290C_A:C5_04340W_A:CSU57:MSM1:HYS2:HAS1:C5_04840C_A:C5_04910W_A:GRF10:C5_05250C_A:SDH4:C5_05340W_A:DNA2:NOP5:C6_00530C_A:C6_00560W_A:C6_00640C_A:CDC14:C6_00760W_A:TOP1:C6_01040C_A:CIP1:C6_01120C_A:CIC1:PMP1:TIF3:RAD18:HCH1:C6_01890C_A:C6_01980C_A:SWD3:C6_02230W_A:RPL10A:ALG11:C6_02280W_A:C6_02290C_A:C6_02300C_A:C6_02350C_A:C6_02370C_A:C6_02430W_A:PRP45:C6_02690C_A:MRT4:C6_02900C_A:POP4:C6_03210C_A:BMT4:ARP4:C6_03380W_A:C6_03390W_A:C6_03440W_A:NAM2:SPB1:C6_04240W_A:NOP8:ILS1:C6_04530C_A:C7_00160C_A:C7_00220W_A:C7_00330C_A:DCC1:PSF3:THG1:RPA135:NOP15:YML6:C7_01020C_A:C7_01030C_A:DBP7:C7_01210C_A:ASC1:C7_01270C_A:PSF1:C7_01360C_A:C7_01400C_A:GIR2:PRT1:FLU1:C7_01570C_A:SMC6:C7_01600W_A:C7_01950W_A:LIG1:C7_02340C_A:C7_02460C_A:C7_02660C_A:C7_02930C_A:IFM1:C7_03400C_A:ISY1:ENP2:C7_03590C_A:ENP1:HIS7:M

						RP7:C7_03850W_A:C7_03880C_A:C7_04150W_A:UTP18:MMS22:RTT109:CR_00430C_A:CR_00460C_A:MRPL6:ADE1:BUD22:PWP2:CR_00830W_A:TRM1:DAL81:CTF18:CR_01260W_A:CR_01320C_A:CR_01410C_A:PRS:CR_01550C_A:CR_01600C_A:CR_01670W_A:CDC60:CR_01700C_A:CR_01710W_A:CR_01780W_A:CR_01950W_A:CR_02030C_A:MCM6:ERG25:CR_02420W_A:CR_02430C_A:CR_02550C_A:RRP9:RPC31:CR_02890C_A:SRP40:ARC1:SMC5:CR_03110W_A:CR_03200C_A:CR_03230W_A:CR_03340C_A:CR_03360W_A:KTI12:MTG1:CR_03400W_A:CR_03440W_A:NCS2:YVH1:SGD1:SMC1:PRP42:CR_03760W_A:CR_03940W_A:MEX67:CR_04110W_A:CR_04160C_A:CR_04170W_A:CR_04240C_A:CR_04300W_A:CR_04500C_A:CR_04560C_A:CR_04710W_A:RPB8:CR_04920W_A:NOC2:CR_05550C_A:RNR3:MTG2:DBP8:RFX1:RPL7:CR_06230W_A:FGR50:CR_06450W_A:RRN11:HSP60:CR_06680C_A:NMD3:CR_06840W_A:ATX1:CR_06970C_A:CR_06980W_A:POL2:CR_07030C_A:URA2:CR_07310W_A:PIF1:CR_07480W_A:FUN30:CR_07640C_A:CDC45:DBP6:ASF1:IMP4:CR_08000C_A:NOP10:SSB1:RIO2:ATS1:CR_08330W_A:RPP2A:GSH2:CR_08410W_A:TSR1:CR_08940W_A:MPS1:CR_09310W_A:ELF1:SSF1:CR_09740W_A:MED21:CR_09800C_A:DPB2:SIK1:CR_09990W_A:CR_10180W_A:UTP5:CR_10260W_A:CR_10410C_A:CR_10470C_A:MCD1:DRS1:YHB4:PRO1:LTV1:POP3

27	ribosomal large subunit assembly	77 out of 1061 genes, 7.3%	105 out of 6473 background genes, 1.6%	1.25E-35	0.00%	C1_04040C_A:ERB1:NOP4:C1_04710C_A:C1_04970W_A:RRP6:C1_05360C_A:C1_06760C_A:ERD1:JIP5:C1_09040C_A:C1_09710C_A:RPA190:C1_10970W_A:TRP3:CSI2:C1_12440W_A:C1_12760W_A:C1_14080W_A:C2_01070W_A:UTP22:C2_02540W_A:MAK5:C2_05160C_A:RPF2:C2_05750W_A:RPL11:C2_09180W_A:C2_10740C_A:BMS1:ARP9:C3_01560W_A:C3_02040C_A:C3_02350W_A:C3_02670W_A:SMC3:C3_04370C_A:MAK21:ARV1:C3_05160C_A:QDR2:C3_06370C_A:C4_05010W_A:C4_05230C_A:RMT2:SPB4:C5_04720C_A:HAS1:C5_05340W_A:CIC1:C6_02230W_A:SPB1:RPA135:DBP7:FLU1:C7_03400C_A:ENP2:UTP18:BUD22:PWP2:CR_01410C_A:CR_01550C_A:YVH1:SGD1:CR_03940W_A:CR_04170W_A:NOC2:CR_05550C_A:SDA1:CR_06680C_A:TSR1:ELF1:SSF1:CR_09800C_A:CR_10470C_A:DRS1:LTV1

42255	ribosome assembly	85 out of 1061 genes, 8.0%	125 out of 6473 background genes, 1.9%	1.84E-35	0.00%	RRS1:C1_04040C_A:ERB1:NOP4:C1_04710C_A:C1_04970W_A:RRP6:C1_05360C_A:RPS27A:C1_06760C_A:RIA1:ERD1:FUN12:JIP5:C1_09040C_A:C1_09710C_A:RPA190:C1_10970W_A:TRP3:CSI2:C1_12440W_A:C1_12760W_A:C1_14080W_A:C2_01070W_A:UTP22:C2_02540W_A:MAK5:C2_05160C_A:RPF2:C2_05750W_A:RPL11:C2_09180W_A:C2_10740C_A:BMS1:ARP9:C3_01560W_A:C3_02020W_A:C3_02040C_A:C3_02350W_A:C3_02670W_A:SMC3:C3_04370C_A:MAK21:ARV1:C3_05160C_A:QDR2:C3_06370C_A:C4_02880C_A:C4_05010W_A:C4_05230C_A:RMT2:SPB4:C5_02070C_A:TIF5:C5_04720C_A:HAS1:C5_05340W_A:CIC1:C6_02230W_A:SPB1:RPA135:DBP7:FLU1:C7_03400C_A:ENP2:UTP18:BUD22:PWP2:CR_01410C_A:CR_01550C_A:YVH1:SGD1:CR_03940W_A:CR_04170W_A:NOC2:CR_05550C_A:SDA1:CR_06680C_A:TSR1:ELF1:SSF1:CR_09800C_A:CR_10470C_A:DRS1:LTV1

44238	primary metabolic process	781 out of 1061 genes, 73.6%	3618 out of 6473 background genes, 55.9%	1.84E-35	0.00%	RIM8:LEU4:HBR1:CNS1:RPO41:NRM1:RRS1:TRI1:NMA111:HMT1:C1_01150C_A:C1_01160C_A:TRM2:ABC1:C1_01530C_A:CYS4:C1_02090C_A:ABP140:CWH8:C1_02380C_A:C1_02390W_A:C1_02450C_A:SNZ1:HOM6:LYS2:PDE2:MIA40:C1_02900C_A:TSR2:C1_03260W_A:C1_03370W_A:C1_03540C_A:C1_03630W_A:C1_03790C_A:RPP1:C1_03830C_A:KTR4:C1_04040C_A:C1_04120C_A:ERB1:RPN4:NOP4:C1_04510W_A:C1_04530C_A:C1_04710C_A:MRPL37:C1_04970W_A:RHD1:ARG2:RRP6:REI1:TRY2:ARO4:C1_05220C_A:C1_05270C_A:C1_05360C_A:C1_05380C_A:RSM22:RPS27A:C1_05630C_A:FAL1:C1_05650W_A:RAD10:C1_06530C_A:C1_06540C_A:NOP6:C1_06760C_A:RIA1:C1_07340W_A:C1_07470C_A:LHP1:C1_07510W_A:C1_07660W_A:ADE4:ECO1:C1_07850C_A:GCS1:ADE5,7:MRPL3:ERD1:C1_07950C_A:C1_07960W_A:FUN12:DRG1:CAT8:MLT1:DIP2:SAM4:GCR3:UPC2:C1_08520C_A:GCD6:C1_08630W_A:MSS116:C1_08890C_A:C1_09040C_A:CEF1:C1_09390W_A:GUA1:C1_09610W_A:C1_09710C_A:C1_09790C_A:C1_09840C_A:BUD31:C1_09910C_A:PDC2:DBP3:MNN23:C1_10080W_A:C1_10200C_A:C1_10340W_A:FCY23:C1_10620W_A:APN2:RPA190:C1_10690W_A:C1_10920W_A:C1_10950C_A:C1_10970W_A:C1_11000C_A:PET127:FGR39:NEP1:KRR1:SAM2:GAR1:C1_11610C_A:C1_11790W_A:MUP1:C1_11880W_A:C1_11900C_A:C1_11910W_A:SER33:TRP3:C1_12280C_A:CSI2:MRPS9:C1_12350W_A:WRS1:C1_12440W_A:C1_12570C_A:C1_12630C_A:C1_12640W_A:C1_12670C_A:C1_12750C_A:C1_12760W_A:FEN1:C1_12820C_A:RPL14:C1_13060C_A:REX3:RMP1:C1_13380W_A:KNS1:MCU1:C1_13560W_A:C1_13820C_A:YBP1:C1_14080W_A:C1_14160W_A:C1_14330W_A:MPP10:FAV3:C2_00200W_A:C2_00280C_A:C2_00390C_A:C2_00410C_A:C2_00620C_A:C2_00820W_A:C2_00840W_A:MXR1:C2_01060C_A:C2_01070W_A:C2_01220W_A:C2_01240C_A:ESC4:CLB2:C2_01420C_A:APT1:REX2:C2_01740C_A:C2_01820C_A:PRP39:C2_01860C_A:C2_01870C_A:ARO3:C2_02120W_A:SAM37:C2_02270C_A:C2_02420C_A:UTP21:UTP22:C2_02490C_A:PRS1:C2_02540W_A:C2_02630W_A:PDR17:SER2:C2_02930C_A:MET1:BNA31:SMP3:C2_03130W_A:PRP3:C2_03560C_A:MNN42:C2_03700W_A:C2_03830W_A:GPI13:SCH9:C2_03950W_A:C2_04120C_A:UGA1:TBF1:BAT21:C2_04340C_A:SUV3:RIM2:MED8:C2_04570W_A:SNU114:C2_04700C_A:C2_04820W_A:PZF1:CKA2:C2_04990W_A:C2_05050C_A:C2_05080C_A:MAK5:C2_05160C_A:CDC21:SGO1:RPF2:C2_05270W_A:RPC40:KTI11:C2_05670C_A:C2_05750W_A:C2_05830C_A:C2_05840W_A:IDP1:C2_05980C_A:LAS1:CSM3:ECM17:ILV1:SER1:MAK16:C2_06480W_A:C2_06520C_A:C2_06530W_A:VAS1:C2_06650C_A:C2_06660W_A:RPL11:FHL1:C2_06850W_A:C2_06950C_A:SPE3:AAH1:RCK2:C2_07270W_A:NOC4:C2_07360W_A:C2_07410W_A:RCL1:C2_07680W_A:PHO86:C2_07860W_A:PRS5:C2_07920W_A:NSA1:C2_08180C_A:C2_08200W_A:EAF3:RRP8:C2_08530C_A:MSU1:UTP15:MFG1:APE3:CDC47:ASN1:C2_09160W_A:C2_09180W_A:MED18:C2_09310C_A:PES1:TAZ1:RRP15:C2_09500W_A:C2_09660W_A:LEU42:SMM1:C2_09920W_A:CWC22:UBP8:GPD1:PUS7:HEM3:C2_10740C_A:RPC11:

						C2_10810W_A:RPS24:C3_00100W_A:ULP3:RHR2:UTP8:BUD21:SOF1:C3_00660W_A:C3_00830C_A:BMS1:ARP9:UTP20:NCE103:C3_01310W_A:URA3:MET18:RKI1:RPS7A:C3_01520C_A:C3_01560W_A:PPT1:SCM3:C3_02020W_A:C3_02040C_A:UTP4:C3_02180C_A:ILV2:C3_02350W_A:PHA2:DDC1:C3_02670W_A:SMC3:C3_02750W_A:C3_02840W_A:C3_02850C_A:MET13:C3_03070W_A:C3_03110W_A:C3_03330C_A:C3_03470W_A:ULP1:C3_03560W_A:HBR3:RAD53:C3_04370C_A:C3_04380C_A:ADE2:MAK21:RPS15:RPP2B:ARV1:SFP1:NOP14:CEM1:C3_05140C_A:C3_05160C_A:WOR2:RPL8B:C3_05280C_A:C3_05380W_A:GPI1:C3_05440C_A:C3_05510W_A:QDR2:BAT22:C3_05800W_A:C3_05860C_A:TRM12:C3_05900W_A:CTA4:NOG1:C3_06150W_A:MMS21:CYM1:C3_06240C_A:C3_06270C_A:DOT1:ISW2:C3_06370C_A:NSA2:OPI3:C3_06600C_A:FUN31:C3_06630W_A:PRP5:C3_06830C_A:RPS12:NOP13:C3_07400W_A:C3_07550C_A:C3_07570C_A:RIT1:RAD4:UTP9:C3_07800C_A:HIS4:MET15:DUR4:RLP24:NMD5:HIS5:C4_00690C_A:C4_00700C_A:C4_00740W_A:MLH1:C4_00800W_A:C4_00810C_A:C4_00880W_A:ARO1:C4_00940W_A:PTC8:RAT1:AAT22:C4_01280C_A:PGA53:ILV6:PWP1:C4_01470W_A:NIP1:C4_01500W_A:C4_01560C_A:TOA2:HSX11:TIM10:ZCF25:C4_02400C_A:SLX4:HIT1:C4_02770C_A:SAS10:RPC53:HCA4:C4_02850W_A:ZUO1:C4_02880C_A:NAN1:C4_03140C_A:C4_03170W_A:DAO2:C4_03410W_A:RAD2:C4_03720C_A:C4_03730C_A:C4_03740W_A:NUP84:C4_03830W_A:RRP42:C4_03870C_A:RAM1:FGR28:ECM1:C4_04130W_A:DUO1:C4_04390W_A:SAP10:C4_04520W_A:SSZ1:BAS1:C4_04810C_A:AGP3:RPL30:C4_05230C_A:C4_05260W_A:C4_05360C_A:OFD1:HOM3:C4_05650W_A:C4_05890W_A:TRP5:C4_06210C_A:C4_06410W_A:NOP1:C4_06790W_A:C4_06950W_A:MET16:C4_07060W_A:TRP4:C4_07140W_A:C4_07150W_A:C5_00030W_A:C5_00260W_A:C5_00280C_A:DUS4:C5_00320W_A:MRP17:MET14:IFG3:EXO1:RMT2:C5_00820W_A:C5_00920W_A:BUD23:CYC3:C5_01140C_A:PUS4:C5_01430C_A:FYV5:SPB4:C5_01610W_A:CCN1:C5_01700W_A:C5_01930W_A:C5_02010C_A:C5_02070C_A:RIX7:THR4:PDE1:C5_02440C_A:TIF5:SPR28:UTP13:C5_02590C_A:BUR2:C5_02740W_A:CKB2:MSW1:C5_02780W_A:C5_02820C_A:C5_03010W_A:RMS1:CAM1-1:C5_03290C_A:PCL1:RPO26:FUR1:C5_03400C_A:C5_03460C_A:C5_03530C_A:MDJ1:C5_03640W_A:C5_03700C_A:HAM1:C5_03920C_A:C5_03970W_A:C5_04120C_A:SAH1:C5_04290C_A:C5_04340W_A:URA7:CSU57:MSM1:HYS2:HAS1:C5_04840C_A:C5_04910W_A:C5_05000C_A:GRF10:C5_05250C_A:SDH4:HIS1:C5_05340W_A:ILV3:DNA2:NOP5:C6_00530C_A:C6_00560W_A:FCA1:C6_00640C_A:CDC14:C6_00760W_A:TOP1:HAL22:C6_01040C_A:CIP1:C6_01120C_A:CIC1:EBP1:C6_01300W_A:PMP1:TIF3:RAD18:HCH1:C6_01890C_A:C6_01980C_A:SWD3:C6_02230W_A:RPL10A:ALG11:C6_02280W_A:C6_02290C_A:C6_02300C_A:C6_02350C_A:C6_02370C_A:C6_02420W_A:C6_02430W_A:PRP45:C6_02690C_A:MRT4:C6_02900C_A:POP4:C6_03210C_A:BMT4:ARP4:C6_03380W_A:C6_03390W_A:C6_03440W_A:NAM2:SPB1:C6_04240

						W_A:NOP8:ILS1:C6_04530C_A:C7_00160C_A:C7_00220W_A:C7_00330C_A:DCC1:PSF3:THG1:RPA135:NOP15:YML6:C7_01020C_A:C7_01030C_A:DBP7:C7_01210C_A:ASC1:C7_01270C_A:PSF1:C7_01360C_A:C7_01400C_A:GIR2:PRT1:FLU1:C7_01570C_A:SMC6:C7_01600W_A:C7_01950W_A:LIG1:C7_02340C_A:C7_02460C_A:C7_02660C_A:C7_02930C_A:IFM1:C7_03400C_A:ISY1:ENP2:ARG4:C7_03590C_A:ENP1:HIS7:MRP7:C7_03850W_A:C7_03880C_A:C7_04150W_A:UTP18:MMS22:RTT109:CR_00430C_A:CR_00460C_A:MRPL6:ADE1:BUD22:PWP2:CR_00830W_A:TRM1:DAL81:CTF18:CR_01260W_A:CR_01320C_A:CR_01410C_A:PRS:CR_01550C_A:TRP2:CR_01600C_A:MET6:CR_01670W_A:CDC60:CR_01700C_A:CR_01710W_A:CR_01780W_A:CR_01950W_A:CR_02030C_A:MCM6:MET2:ERG25:CR_02420W_A:CR_02430C_A:CHO2:CR_02550C_A:RRP9:RPC31:CR_02890C_A:SRP40:ARC1:SMC5:CR_03110W_A:CR_03200C_A:CR_03230W_A:CR_03340C_A:CR_03360W_A:KTI12:MTG1:CR_03400W_A:CR_03440W_A:NCS2:YVH1:SGD1:SMC1:PRP42:CR_03760W_A:CR_03940W_A:CR_04110W_A:CR_04160C_A:CR_04170W_A:QDR1:CR_04240C_A:CR_04300W_A:CR_04500C_A:CR_04560C_A:CR_04710W_A:ADE6:RPB8:CR_04920W_A:NOC2:CR_05550C_A:RNR3:MTG2:DBP8:RFX1:RPL7:CR_06230W_A:FGR50:CR_06450W_A:RRN11:HSP60:CR_06680C_A:NMD3:CR_06840W_A:ATX1:CR_06970C_A:CR_06980W_A:POL2:MIS11:CR_07030C_A:URA2:CR_07310W_A:PIF1:CR_07480W_A:FUN30:CR_07640C_A:ARO2:CDC45:DBP6:ASF1:IMP4:CR_08000C_A:NOP10:SSB1:RIO2:ATS1:CR_08330W_A:CYS3:RPP2A:GSH2:CR_08410W_A:TSR1:CR_08940W_A:MPS1:CR_09010C_A:CR_09310W_A:SSF1:CR_09740W_A:MED21:CR_09800C_A:DPB2:SIK1:CR_09990W_A:CR_10180W_A:UTP5:CR_10260W_A:CR_10410C_A:CR_10470C_A:MCD1:DRS1:YHB4:PRO1:LTV1:POP3

71826	protein-RNA complex organization	111 out of 1061 genes, 10.5%	198 out of 6473 background genes, 3.1%	3.17E-35	0.00%	C1_02900C_A:C1_04040C_A:C1_04120C_A:ERB1:NOP4:C1_04710C_A:C1_04970W_A:RRP6:C1_05360C_A:RPS27A:C1_06760C_A:ERD1:FUN12:JIP5:C1_09040C_A:CEF1:YTM1:C1_09710C_A:RPA190:C1_10970W_A:TRP3:CSI2:C1_12440W_A:C1_12750C_A:C1_12760W_A:C1_14080W_A:C2_00200W_A:C2_00280C_A:C2_01070W_A:PRP39:UTP22:C2_02540W_A:PRP3:SNU114:MAK5:C2_05160C_A:RPF2:C2_05750W_A:C2_05830C_A:C2_06650C_A:RPL11:C2_09180W_A:C2_10740C_A:C3_00100W_A:C3_00660W_A:BMS1:ARP9:C3_01560W_A:C3_02020W_A:C3_02040C_A:C3_02350W_A:C3_02670W_A:SMC3:C3_03330C_A:C3_04370C_A:MAK21:ARV1:C3_05160C_A:QDR2:NOG1:C3_06370C_A:PRP5:RLP24:HIT1:C4_05010W_A:C4_05230C_A:C4_06410W_A:RMT2:SPB4:C5_01930W_A:C5_02010C_A:C5_02070C_A:TIF5:C5_04720C_A:HAS1:C5_05340W_A:CIC1:TIF3:C6_02230W_A:PRP45:SPB1:RPA135:DBP7:FLU1:C7_03400C_A:ISY1:ENP2:UTP18:BUD22:PWP2:CR_00830W_A:CR_01410C_A:CR_01550C_A:CR_01700C_A:ARC1:YVH1:SGD1:CR_03940W_A:CR_04110W_A:CR_04170W_A:NOC2:CR_05550C_A:SDA1:CR_06680C_A:TSR1:ELF1:SSF1:CR_09800C_A:CR_10470C_A:DRS1:LTV1

460	maturation of 5.8S rRNA	91 out of 1061 genes, 8.6%	144 out of 6473 background genes, 2.2%	3.32E-34	0.00%	RRS1:C1_01160C_A:RPP1:C1_03830C_A:C1_04040C_A:RRP6:C1_06540C_A:C1_07660W_A:C1_07960W_A:DIP2:C1_09710C_A:DBP3:NEP1:KRR1:CSI2:C1_12350W_A:REX3:RMP1:C1_13820C_A:C1_14080W_A:MPP10:FAV3:C2_00200W_A:C2_00410C_A:C2_00820W_A:REX2:C2_02540W_A:C2_02630W_A:TBF1:MAK5:RPF2:LAS1:MAK16:C2_06660W_A:FHL1:NOC4:RCL1:C2_09160W_A:RRP15:BUD21:UTP20:C3_01560W_A:C3_02020W_A:C3_02040C_A:C3_02350W_A:C3_02840W_A:NOP14:C3_05160C_A:ISW2:C3_06370C_A:NSA2:RAD4:C3_07800C_A:DUR4:C4_00690C_A:RAT1:HIT1:SAS10:C4_03740W_A:RRP42:C4_04520W_A:BAS1:BUD23:C5_02070C_A:UTP13:C5_03920C_A:C5_04840C_A:NOP5:CIC1:SPB1:C7_03400C_A:ENP1:C7_03850W_A:UTP18:PWP2:CR_02420W_A:CR_02550C_A:RRP9:CR_03200C_A:CR_03360W_A:DBP8:RPL7:CR_06450W_A:PIF1:DBP6:ATS1:CR_08330W_A:MPS1:CR_09800C_A:CR_10260W_A:CR_10410C_A

466	maturation of 5.8S rRNA from tricistronic rRNA transcript (SSU-rRNA, 5.8S rRNA, LSU-rRNA)	91 out of 1061 genes, 8.6%	144 out of 6473 background genes, 2.2%	3.32E-34	0.00%	RRS1:C1_01160C_A:RPP1:C1_03830C_A:C1_04040C_A:RRP6:C1_06540C_A:C1_07660W_A:C1_07960W_A:DIP2:C1_09710C_A:DBP3:NEP1:KRR1:CSI2:C1_12350W_A:REX3:RMP1:C1_13820C_A:C1_14080W_A:MPP10:FAV3:C2_00200W_A:C2_00410C_A:C2_00820W_A:REX2:C2_02540W_A:C2_02630W_A:TBF1:MAK5:RPF2:LAS1:MAK16:C2_06660W_A:FHL1:NOC4:RCL1:C2_09160W_A:RRP15:BUD21:UTP20:C3_01560W_A:C3_02020W_A:C3_02040C_A:C3_02350W_A:C3_02840W_A:NOP14:C3_05160C_A:ISW2:C3_06370C_A:NSA2:RAD4:C3_07800C_A:DUR4:C4_00690C_A:RAT1:HIT1:SAS10:C4_03740W_A:RRP42:C4_04520W_A:BAS1:BUD23:C5_02070C_A:UTP13:C5_03920C_A:C5_04840C_A:NOP5:CIC1:SPB1:C7_03400C_A:ENP1:C7_03850W_A:UTP18:PWP2:CR_02420W_A:CR_02550C_A:RRP9:CR_03200C_A:CR_03360W_A:DBP8:RPL7:CR_06450W_A:PIF1:DBP6:ATS1:CR_08330W_A:MPS1:CR_09800C_A:CR_10260W_A:CR_10410C_A

44237	cellular metabolic process	781 out of 1061 genes, 73.6%	3715 out of 6473 background genes, 57.4%	8.92E-30	0.00%	RIM8:LEU4:HBR1:C1_00510W_A:C1_00520W_A:CNS1:RPO41:SPE1:NRM1:RRS1:TRI1:NMA111:HMT1:C1_01150C_A:C1_01160C_A:TRM2:ABC1:C1_01530C_A:CYS4:C1_02090C_A:ABP140:CWH8:C1_02380C_A:C1_02390W_A:C1_02450C_A:SNZ1:SNO1:HOM6:LYS2:PDE2:MIA40:C1_02900C_A:C1_02970W_A:TSR2:C1_03260W_A:C1_03370W_A:C1_03540C_A:C1_03630W_A:C1_03790C_A:RPP1:C1_03830C_A:KTR4:C1_04040C_A:C1_04120C_A:ERB1:RPN4:NOP4:C1_04510W_A:C1_04530C_A:C1_04710C_A:MRPL37:C1_04970W_A:RHD1:ARG2:RRP6:REI1:TRY2:ARO4:C1_05220C_A:C1_05270C_A:C1_05360C_A:C1_05380C_A:RSM22:RPS27A:C1_05630C_A:FAL1:C1_05650W_A:RAD10:C1_05990C_A:BUD16:C1_06530C_A:C1_06540C_A:NOP6:C1_06760C_A:RIA1:YMC1:C1_07340W_A:C1_07470C_A:LHP1:C1_07510W_A:C1_07660W_A:ADE4:ECO1:GCS1:ADE5,7:MRPL3:ERD1:C1_07950C_A:C1_07960W_A:FUN12:DRG1:CAT8:MLT1:DIP2:SAM4:GCR3:UPC2:C1_08520C_A:GCD6:C1_08630W_A:MSS116:C1_08890C_A:NAR1:CEF1:C1_09390W_A:GUA1:C1_09610W_A:C1_09620C_A:C1_09710C_A:C1_09790C_A:C1_09840C_A:BUD31:C1_09910C_A:PDC2:DBP3:MNN23:C1_10080W_A:C1_10200C_A:C1_10340W_A:FCY23:C1_10620W_A:APN2:RPA190:C1_10690W_A:C1_10920W_A:C1_10950C_A:C1_10970W_A:C1_11000C_A:PET127:FGR39:NEP1:KRR1:SAM2:GAR1:C1_11610C_A:C1_11790W_A:MUP1:C1_11880W_A:C1_11900C_A:C1_11910W_A:SER33:TRP3:C1_12280C_A:CSI2:MRPS9:C1_12350W_A:WRS1:C1_12440W_A:C1_12570C_A:C1_12630C_A:C1_12670C_A:C1_12750C_A:C1_12760W_A:FEN1:C1_12820C_A:RPL14:C1_13060C_A:REX3:C1_13330C_A:RMP1:C1_13380W_A:AGC1:KNS1:MCU1:C1_13560W_A:C1_13820C_A:MET3:C1_14080W_A:C1_14330W_A:MPP10:FAV3:C2_00200W_A:C2_00280C_A:C2_00390C_A:C2_00410C_A:C2_00810C_A:C2_00820W_A:C2_00840W_A:MXR1:C2_01070W_A:C2_01220W_A:C2_01240C_A:ESC4:C2_01420C_A:APT1:REX2:C2_01740C_A:C2_01820C_A:PRP39:C2_01860C_A:C2_01870C_A:ARO3:C2_02120W_A:C2_02270C_A:C2_02420C_A:UTP21:UTP22:PRS1:C2_02540W_A:C2_02630W_A:PDR17:SER2:C2_02930C_A:MET1:BNA31:SMP3:PRP3:C2_03560C_A:MNN42:GPI13:SCH9:C2_03950W_A:C2_04120C_A:UGA1:TBF1:BAT21:C2_04340C_A:SUV3:RIM2:C2_04570W_A:SNU114:C2_04700C_A:C2_04820W_A:PZF1:CKA2:C2_04990W_A:C2_05050C_A:C2_05080C_A:MAK5:C2_05160C_A:CDC21:RPF2:C2_05270W_A:RPC40:KTI11:C2_05670C_A:C2_05750W_A:C2_05830C_A:C2_05840W_A:C2_05860C_A:IDP1:C2_05980C_A:LAS1:CSM3:ECM17:ILV1:SER1:MAK16:C2_06480W_A:C2_06520C_A:C2_06530W_A:VAS1:C2_06650C_A:C2_06660W_A:RPL11:FHL1:C2_06850W_A:C2_06950C_A:SPE3:AAH1:RCK2:DQD1:NOC4:C2_07360W_A:C2_07410W_A:RCL1:C2_07680W_A:PHO86:PRS5:C2_07920W_A:NSA1:C2_08180C_A:EAF3:RRP8:C2_08530C_A:MSU1:UTP15:MFG1:APE3:CDC47:ASN1:MET10:C2_09160W_A:C2_09180W_A:MED18:C2_09310C_A:PES1:TAZ1:RRP15:C2_09500W_A:C2_09660W_A:LEU42:COE1:SMM1:C2_09920W_A:CWC22:UBP8:GPD1:PUS7:HEM3:C2_10740C_A:RPC11:C2_10810W_A:RPS24:C3_00100W_A:ULP3:

						UTP8:BUD21:SOF1:C3_00660W_A:C3_00830C_A:BMS1:ARP9:UTP20:NCE103:C3_01310W_A:URA3:MET18:RKI1:RPS7A:C3_01520C_A:C3_01560W_A:PPT1:SCM3:C3_01850W_A:C3_02020W_A:C3_02040C_A:UTP4:C3_02180C_A:ILV2:C3_02350W_A:PHA2:DDC1:C3_02670W_A:SMC3:C3_02750W_A:C3_02840W_A:C3_02850C_A:MET13:C3_03070W_A:C3_03110W_A:C3_03330C_A:YAH1:C3_03470W_A:C3_03560W_A:HBR3:RAD53:C3_04370C_A:C3_04380C_A:ADE2:MAK21:RPS15:RPP2B:ARV1:SFP1:NOP14:CEM1:C3_05140C_A:C3_05160C_A:WOR2:RPL8B:C3_05280C_A:C3_05380W_A:GPI1:C3_05440C_A:C3_05510W_A:QDR2:BAT22:C3_05800W_A:C3_05860C_A:TRM12:C3_05900W_A:CTA4:NOG1:C3_06150W_A:MMS21:C3_06240C_A:C3_06270C_A:DOT1:ISW2:C3_06370C_A:NSA2:OPI3:FUN31:C3_06630W_A:PRP5:C3_06830C_A:RPS12:NOP13:C3_07400W_A:C3_07550C_A:C3_07570C_A:RIT1:RAD4:UTP9:C3_07800C_A:HIS4:MET15:DUR4:RLP24:NMD5:HIS5:C4_00690C_A:C4_00700C_A:C4_00740W_A:MLH1:C4_00800W_A:C4_00810C_A:C4_00880W_A:ARO1:C4_00940W_A:PTC8:RAT1:AAT22:C4_01280C_A:PGA53:ILV6:PWP1:NIP1:C4_01500W_A:TOA2:HSX11:TIM10:ZCF25:C4_02400C_A:SLX4:HIT1:C4_02770C_A:SAS10:RPC53:HCA4:C4_02850W_A:ZUO1:C4_02880C_A:NAN1:C4_03140C_A:C4_03170W_A:DAO2:C4_03410W_A:RAD2:C4_03720C_A:C4_03730C_A:C4_03740W_A:NUP84:C4_03830W_A:RRP42:C4_03870C_A:RAM1:ECM1:C4_04130W_A:DUO1:C4_04390W_A:C4_04520W_A:SSZ1:BAS1:C4_04810C_A:AGP3:RPL30:C4_05230C_A:C4_05260W_A:C4_05360C_A:OFD1:HOM3:C4_05650W_A:TRP5:C4_06210C_A:C4_06410W_A:NOP1:C4_06790W_A:C4_06950W_A:MET16:C4_07060W_A:TRP4:C4_07140W_A:C4_07150W_A:C5_00030W_A:C5_00260W_A:C5_00280C_A:DUS4:C5_00320W_A:MRP17:MET14:IFG3:EXO1:RMT2:SPE2:C5_00820W_A:C5_00920W_A:BUD23:CYC3:C5_01140C_A:PUS4:C5_01430C_A:FYV5:SPB4:C5_01610W_A:CCN1:C5_01700W_A:C5_01930W_A:C5_02010C_A:C5_02070C_A:RIX7:THR4:PDE1:C5_02440C_A:TIF5:SPR28:UTP13:C5_02590C_A:BUR2:CKB2:MSW1:C5_02780W_A:C5_02820C_A:C5_03010W_A:RMS1:CAM1-1:C5_03290C_A:PCL1:RPO26:FUR1:C5_03400C_A:C5_03460C_A:C5_03530C_A:C5_03550W_A:MDJ1:C5_03840W_A:HAM1:C5_03920C_A:C5_03970W_A:C5_04120C_A:SAH1:C5_04290C_A:C5_04340W_A:URA7:CSU57:MSM1:C5_04720C_A:HYS2:HAS1:C5_04840C_A:C5_04910W_A:C5_05000C_A:GRF10:PCL5:C5_05250C_A:SDH4:HIS1:C5_05340W_A:ILV3:DNA2:C6_00360C_A:NOP5:C6_00530C_A:C6_00560W_A:FCA1:C6_00640C_A:CDC14:TOP1:HAL22:C6_01040C_A:CIP1:C6_01120C_A:CIC1:C6_01300W_A:PMP1:TIF3:RAD18:HCH1:C6_01890C_A:C6_01980C_A:C6_02150C_A:SWD3:C6_02230W_A:RPL10A:ALG11:C6_02280W_A:C6_02290C_A:C6_02300C_A:C6_02350C_A:C6_02370C_A:C6_02420W_A:C6_02430W_A:PRP45:C6_02690C_A:MRT4:C6_02900C_A:POP4:C6_03210C_A:BMT4:C6_03320W_A:C6_03330C_A:ARP4:C6_03380W_A:C6_03390W_A:C6_03440W_A:NAM2:PAD1:SPB1:C6_04240W_A:NOP8:ILS1:C6_04530C_A:C7_00160C_A:C7_00220W_A:C7_00330C_A:DCC1:PSF3:THG1:RPA1

						35:NOP15:YML6:C7_01020C_A:C7_01030C_A:DBP7:C7_01210C_A:ASC1:C7_01270C_A:PSF1:C7_01360C_A:QCE1:C7_01400C_A:GIR2:PRT1:FLU1:C7_01570C_A:SMC6:C7_01600W_A:C7_01950W_A:LIG1:C7_02340C_A:C7_02460C_A:C7_02660C_A:C7_02930C_A:IFM1:C7_03400C_A:ISY1:ENP2:ARG4:C7_03590C_A:ENP1:HIS7:MRP7:C7_03850W_A:C7_03880C_A:MAM33:C7_04150W_A:UTP18:MIS12:MMS22:RTT109:CR_00430C_A:CR_00460C_A:MRPL6:ADE1:BUD22:PWP2:CR_00830W_A:TRM1:DAL81:CTF18:CR_01260W_A:CR_01320C_A:CR_01410C_A:PRS:CR_01550C_A:TRP2:CR_01600C_A:MET6:CR_01670W_A:CDC60:CR_01700C_A:CR_01710W_A:CR_01780W_A:CR_01950W_A:CR_02030C_A:MCM6:MET2:ERG25:CR_02420W_A:CR_02430C_A:CHO2:CR_02550C_A:PAN6:NUE1:RRP9:RPC31:CR_02890C_A:SRP40:ARC1:SMC5:CR_03110W_A:CR_03200C_A:CR_03230W_A:CR_03340C_A:CR_03360W_A:KTI12:MTG1:CR_03400W_A:CR_03440W_A:NCS2:YVH1:SGD1:SMC1:PRP42:CR_03940W_A:MEX67:CR_04110W_A:CR_04160C_A:CR_04170W_A:QDR1:CR_04240C_A:CR_04300W_A:CR_04500C_A:CR_04560C_A:CR_04710W_A:ADE6:RPB8:CR_04920W_A:NOC2:CR_05550C_A:RNR3:MTG2:DBP8:RFX1:RPL7:CR_06230W_A:FGR50:CR_06450W_A:RRN11:HSP60:CR_06680C_A:NMD3:CR_06840W_A:CR_06970C_A:CR_06980W_A:POL2:MIS11:CR_07030C_A:URA2:CR_07310W_A:PIF1:FUN30:CR_07640C_A:ARO2:CDC45:DBP6:IMP4:CR_08000C_A:NOP10:SSB1:RIO2:ATS1:CR_08330W_A:CYS3:RPP2A:GSH2:CR_08410W_A:TSR1:CR_08940W_A:MPS1:CR_09010C_A:ELF1:SSF1:CR_09740W_A:MED21:CR_09800C_A:DPB2:SIK1:CR_09990W_A:CR_10180W_A:CR_10190C_A:UTP5:CR_10260W_A:CR_10410C_A:CR_10470C_A:MCD1:DRS1:PRO1:LTV1:POP3

6399	tRNA metabolic process	94 out of 1061 genes, 8.9%	168 out of 6473 background genes, 2.6%	2.77E-29	0.00%	C1_01150C_A:C1_01160C_A:TRM2:C1_01530C_A:ABP140:RPP1:C1_03830C_A:C1_04530C_A:RRP6:C1_05360C_A:C1_06540C_A:LHP1:C1_09390W_A:C1_09790C_A:C1_12350W_A:WRS1:C1_12570C_A:C1_12820C_A:C1_14330W_A:C2_00820W_A:C2_02420C_A:RPC40:KTI11:C2_05670C_A:C2_06480W_A:VAS1:C2_06660W_A:C2_07360W_A:C2_09160W_A:C2_09310C_A:C2_09500W_A:SMM1:PUS7:RPC11:C3_02840W_A:C3_05140C_A:C3_05800W_A:TRM12:C3_07400W_A:RIT1:C4_00740W_A:C4_00810C_A:C4_00880W_A:C4_00940W_A:C4_01500W_A:RPC53:C4_02850W_A:C4_03140C_A:C4_03730C_A:C4_03740W_A:C4_03830W_A:RRP42:C4_04810C_A:C4_05260W_A:NOP1:C4_07060W_A:DUS4:PUS4:C5_01610W_A:MSW1:RPO26:MSM1:C6_02290C_A:C6_02350C_A:POP4:C6_03210C_A:NAM2:ILS1:C6_04530C_A:C7_00330C_A:THG1:C7_01210C_A:LIG1:C7_02340C_A:C7_03400C_A:TRM1:PRS:CDC60:CR_02550C_A:RPC31:CR_02890C_A:ARC1:CR_03110W_A:CR_03200C_A:KTI12:CR_03400W_A:NCS2:CR_04160C_A:CR_04300W_A:RPB8:CR_05550C_A:ATS1:CR_08940W_A:POP3

9451	RNA modification	64 out of 1061 genes, 6.0%	97 out of 6473 background genes, 1.5%	6.39E-25	0.00%	C1_01150C_A:TRM2:ABP140:C1_04530C_A:C1_05360C_A:C1_09390W_A:C1_09790C_A:C1_10620W_A:NEP1:GAR1:C1_12570C_A:C1_14330W_A:C2_00820W_A:C2_02120W_A:C2_02420C_A:C2_04820W_A:KTI11:C2_06480W_A:C2_07360W_A:RRP8:C2_09500W_A:SMM1:PUS7:C3_05140C_A:C3_05860C_A:TRM12:C3_07400W_A:RIT1:C4_00810C_A:C4_00940W_A:C4_01500W_A:C4_02850W_A:C4_03140C_A:C4_03730C_A:C4_03830W_A:C4_04810C_A:C4_05260W_A:NOP1:C4_06950W_A:DUS4:BUD23:PUS4:C5_01610W_A:C6_02290C_A:C6_02350C_A:SPB1:C6_04530C_A:C7_00330C_A:THG1:C7_02340C_A:TRM1:CR_01780W_A:CR_02030C_A:KTI12:NCS2:CR_04110W_A:CR_04160C_A:CR_04170W_A:CR_04240C_A:CR_04300W_A:NOP10:ATS1:CR_08940W_A:CR_10260W_A

1E+05	non-membrane-bounded organelle assembly	91 out of 1061 genes, 8.6%	180 out of 6473 background genes, 2.8%	8.32E-24	0.00%	RRS1:C1_04040C_A:ERB1:NOP4:C1_04710C_A:C1_04970W_A:RRP6:C1_05360C_A:RPS27A:C1_06760C_A:RIA1:ERD1:FUN12:JIP5:C1_09040C_A:C1_09710C_A:RPA190:C1_10970W_A:TRP3:CSI2:C1_12440W_A:C1_12760W_A:C1_14080W_A:C2_01070W_A:UTP22:C2_02540W_A:MAK5:C2_05160C_A:RPF2:C2_05750W_A:RPL11:C2_09180W_A:C2_10740C_A:BMS1:ARP9:C3_01560W_A:SCM3:C3_02020W_A:C3_02040C_A:C3_02350W_A:C3_02670W_A:SMC3:C3_04370C_A:MAK21:ARV1:C3_05160C_A:QDR2:C3_06370C_A:C4_02880C_A:C4_05010W_A:C4_05230C_A:TUB4:RMT2:SPB4:C5_02070C_A:TIF5:C5_04720C_A:HAS1:C5_05340W_A:CIC1:C6_02230W_A:ARP4:SPB1:RPA135:DBP7:FLU1:C7_03400C_A:ENP2:UTP18:BUD22:PWP2:CR_01410C_A:CR_01550C_A:YVH1:SGD1:CR_03940W_A:CR_04170W_A:NOC2:CR_05550C_A:SDA1:CR_06680C_A:CR_06740W_A:TSR1:MPS1:ELF1:SSF1:CR_09800C_A:CR_10180W_A:CR_10470C_A:DRS1:LTV1

8152	metabolic process	832 out of 1061 genes, 78.4%	4250 out of 6473 background genes, 65.7%	6.34E-20	0.00%	RIM8:LEU4:HBR1:C1_00510W_A:C1_00520W_A:CNS1:RPO41:SPE1:NRM1:RRS1:TRI1:NMA111:HMT1:C1_01150C_A:C1_01160C_A:TRM2:ABC1:C1_01530C_A:CYS4:C1_02090C_A:ABP140:CWH8:C1_02380C_A:C1_02390W_A:C1_02450C_A:SNZ1:SNO1:HOM6:LYS2:PDE2:MIA40:C1_02900C_A:C1_02970W_A:TSR2:C1_03260W_A:C1_03370W_A:C1_03540C_A:C1_03630W_A:C1_03790C_A:RPP1:C1_03830C_A:KTR4:C1_04040C_A:C1_04120C_A:ERB1:RPN4:NOP4:C1_04510W_A:C1_04530C_A:C1_04710C_A:MRPL37:C1_04970W_A:RHD1:ARG2:RRP6:REI1:TRY2:ARO4:C1_05220C_A:C1_05270C_A:C1_05330C_A:C1_05360C_A:C1_05380C_A:RSM22:RPS27A:C1_05630C_A:FAL1:C1_05650W_A:RAD10:C1_05930C_A:C1_05990C_A:BUD16:C1_06530C_A:C1_06540C_A:NOP6:C1_06760C_A:RIA1:YMC1:C1_07340W_A:C1_07470C_A:LHP1:C1_07510W_A:C1_07660W_A:ADE4:ECO1:C1_07850C_A:GCS1:ADE5,7:MRPL3:ERD1:C1_07950C_A:C1_07960W_A:FUN12:DRG1:CAT8:MLT1:DIP2:SAM4:GCR3:UPC2:C1_08520C_A:GCD6:C1_08630W_A:YMC2:MSS116:C1_08890C_A:NAR1:C1_09040C_A:CEF1:C1_09390W_A:GUA1:C1_09610W_A:C1_09620C_A:C1_09710C_A:C1_09790C_A:C1_09840C_A:BUD31:C1_09910C_A:PDC2:DBP3:MNN23:C1_10080W_A:C1_10200C_A:C1_10340W_A:FCY23:C1_10620W_A:APN2:RPA190:C1_10690W_A:NIT3:C1_10920W_A:C1_10950C_A:C1_10970W_A:C1_11000C_A:PET127:FGR39:NEP1:KRR1:SAM2:GAR1:C1_11610C_A:C1_11790W_A:MUP1:C1_11880W_A:C1_11900C_A:C1_11910W_A:SER33:TRP3:C1_12280C_A:CSI2:MRPS9:C1_12350W_A:WRS1:C1_12440W_A:C1_12570C_A:C1_12630C_A:C1_12640W_A:C1_12670C_A:C1_12750C_A:C1_12760W_A:FEN1:C1_12820C_A:RPL14:C1_13060C_A:REX3:C1_13330C_A:RMP1:C1_13380W_A:AGC1:KNS1:MCU1:C1_13560W_A:C1_13820C_A:MET3:YBP1:C1_14080W_A:C1_14160W_A:C1_14320C_A:C1_14330W_A:MPP10:FAV3:C2_00200W_A:C2_00280C_A:C2_00390C_A:C2_00410C_A:C2_00620C_A:C2_00810C_A:C2_00820W_A:C2_00840W_A:MXR1:C2_01060C_A:C2_01070W_A:C2_01220W_A:C2_01240C_A:ESC4:CLB2:C2_01420C_A:APT1:REX2:C2_01740C_A:C2_01820C_A:PRP39:C2_01860C_A:C2_01870C_A:ARO3:C2_02120W_A:SAM37:C2_02270C_A:C2_02420C_A:UTP21:UTP22:C2_02490C_A:PRS1:C2_02540W_A:C2_02630W_A:PDR17:SER2:C2_02930C_A:MET1:BNA31:SMP3:C2_03130W_A:PRP3:C2_03560C_A:MNN42:C2_03700W_A:C2_03830W_A:GPI13:SCH9:C2_03950W_A:C2_04120C_A:UGA1:TBF1:BAT21:C2_04340C_A:SUV3:RIM2:MED8:C2_04570W_A:SNU114:C2_04700C_A:C2_04820W_A:PZF1:CKA2:C2_04990W_A:C2_05050C_A:C2_05080C_A:MAK5:C2_05160C_A:CDC21:SGO1:RPF2:C2_05270W_A:RPC40:KTI11:C2_05670C_A:C2_05750W_A:C2_05830C_A:C2_05840W_A:C2_05860C_A:IDP1:C2_05980C_A:LAS1:CSM3:ECM17:ILV1:SER1:MAK16:C2_06480W_A:C2_06520C_A:C2_06530W_A:VAS1:C2_06650C_A:C2_06660W_A:AMO2:RPL11:FHL1:C2_06850W_A:C2_06950C_A:SPE3:AAH1:RCK2:DQD1:C2_07270W_A:NOC4:C2_07360W_A:C2_07410W_A:RCL1:C2_07680W_A:PHO86:C2_07860W_A:PRS5:C2_07920W_A:NSA1:C2_08180C_A:C2_08200W_A:EAF3:RRP8:C2_0853

						0C_A:MSU1:UTP15:MFG1:APE3:CDC47:ASN1:MET10:C2_09160W_A:C2_09180W_A:MED18:C2_09310C_A:PES1:TAZ1:RRP15:C2_09500W_A:C2_09660W_A:LEU42:COE1:SMM1:C2_09920W_A:CWC22:UBP8:GPD1:PUS7:HEM3:C2_10740C_A:RPC11:C2_10810W_A:RPS24:C3_00100W_A:ULP3:RHR2:UTP8:BUD21:SOF1:C3_00660W_A:C3_00830C_A:BMS1:ARP9:UTP20:NCE103:C3_01310W_A:URA3:MET18:RKI1:RPS7A:C3_01520C_A:C3_01560W_A:PPT1:SCM3:C3_01850W_A:C3_02020W_A:C3_02040C_A:UTP4:C3_02180C_A:ILV2:C3_02350W_A:PHA2:DDC1:C3_02670W_A:SMC3:C3_02750W_A:C3_02840W_A:C3_02850C_A:MET13:C3_03070W_A:C3_03110W_A:C3_03330C_A:YAH1:C3_03470W_A:ULP1:C3_03560W_A:HBR3:RAD53:ATF1:C3_04370C_A:C3_04380C_A:ADE2:MAK21:RPS15:RPP2B:ARV1:SFP1:NOP14:CEM1:C3_05140C_A:C3_05160C_A:WOR2:RPL8B:C3_05280C_A:C3_05380W_A:GPI1:C3_05440C_A:C3_05510W_A:QDR2:BAT22:C3_05800W_A:C3_05860C_A:TRM12:C3_05900W_A:CTA4:FAA2:NOG1:C3_06150W_A:MMS21:CYM1:C3_06240C_A:C3_06270C_A:DOT1:ISW2:C3_06370C_A:NSA2:OPI3:C3_06600C_A:FUN31:C3_06630W_A:PRP5:C3_06830C_A:RPS12:NOP13:C3_07400W_A:C3_07550C_A:C3_07570C_A:RIT1:RAD4:UTP9:C3_07800C_A:HIS4:MET15:DUR4:RLP24:NMD5:HIS5:C4_00690C_A:C4_00700C_A:C4_00740W_A:MLH1:C4_00800W_A:C4_00810C_A:C4_00880W_A:ARO1:C4_00940W_A:PTC8:RAT1:AAT22:C4_01280C_A:PGA53:ILV6:PWP1:C4_01470W_A:NIP1:C4_01500W_A:C4_01560C_A:TOA2:HSX11:TIM10:ZCF25:C4_02400C_A:SLX4:SUL2:HIT1:C4_02770C_A:SAS10:RPC53:HCA4:C4_02850W_A:ZUO1:C4_02880C_A:NAN1:C4_03140C_A:C4_03170W_A:DAO2:C4_03410W_A:RAD2:C4_03720C_A:C4_03730C_A:C4_03740W_A:NUP84:C4_03830W_A:RRP42:C4_03870C_A:RAM1:FGR28:ECM1:C4_04130W_A:DUO1:C4_04390W_A:SAP10:C4_04520W_A:SSZ1:BAS1:C4_04810C_A:AGP3:RPL30:C4_05230C_A:C4_05260W_A:C4_05360C_A:OFD1:HOM3:C4_05650W_A:C4_05890W_A:TRP5:C4_06210C_A:C4_06410W_A:NOP1:C4_06790W_A:C4_06950W_A:MET16:C4_07060W_A:TRP4:C4_07140W_A:C4_07150W_A:C5_00030W_A:C5_00260W_A:C5_00280C_A:DUS4:C5_00320W_A:MRP17:MET14:IFG3:EXO1:RMT2:SPE2:C5_00820W_A:C5_00920W_A:BUD23:CYC3:C5_01140C_A:PUS4:C5_01430C_A:FYV5:SPB4:C5_01610W_A:CCN1:C5_01700W_A:C5_01930W_A:C5_02010C_A:C5_02070C_A:RIX7:THR4:PDE1:C5_02440C_A:TIF5:SPR28:UTP13:C5_02590C_A:BUR2:C5_02740W_A:CKB2:MSW1:C5_02780W_A:C5_02820C_A:C5_03010W_A:RMS1:CAM1-1:C5_03290C_A:PCL1:RPO26:FUR1:C5_03400C_A:C5_03460C_A:C5_03530C_A:C5_03550W_A:MDJ1:C5_03640W_A:C5_03700C_A:C5_03840W_A:HAM1:C5_03920C_A:C5_03970W_A:C5_04120C_A:SAH1:C5_04290C_A:C5_04340W_A:URA7:CSU57:MSM1:C5_04720C_A:HYS2:HAS1:C5_04840C_A:C5_04910W_A:C5_05000C_A:GRF10:PCL5:C5_05250C_A:SDH4:HIS1:C5_05340W_A:ILV3:DNA2:C6_00360C_A:NOP5:C6_00530C_A:C6_00560W_A:FCA1:C6_00640C_A:CDC14:C6_00760W_A:TOP1:HAL22:C6_01040C_A:CIP1:C6_01120C_A:CIC1:EBP1:QDR3:C6_01300W_A:PMP1:TIF3:RAD18

						:HCH1:C6_01890C_A:C6_01980C_A:C6_02150C_A:SWD3:C6_02230W_A:RPL10A:ALG11:C6_02280W_A:C6_02290C_A:C6_02300C_A:C6_02350C_A:C6_02370C_A:C6_02420W_A:C6_02430W_A:PRP45:C6_02690C_A:MRT4:C6_02900C_A:POP4:C6_03210C_A:BMT4:C6_03320W_A:C6_03330C_A:ARP4:C6_03380W_A:C6_03390W_A:C6_03440W_A:NAM2:PAD1:SPB1:C6_04240W_A:NOP8:ILS1:C6_04530C_A:C7_00160C_A:C7_00220W_A:C7_00330C_A:DCC1:PSF3:THG1:RPA135:NOP15:YML6:C7_01020C_A:C7_01030C_A:C7_01170C_A:DBP7:C7_01210C_A:ASC1:C7_01270C_A:PSF1:C7_01360C_A:QCE1:C7_01400C_A:GIR2:PRT1:FLU1:C7_01570C_A:SMC6:C7_01600W_A:C7_01950W_A:LIG1:C7_02340C_A:C7_02460C_A:C7_02660C_A:C7_02930C_A:IFM1:C7_03400C_A:ISY1:ENP2:ARG4:C7_03590C_A:ENP1:HIS7:MRP7:C7_03850W_A:C7_03880C_A:MAM33:C7_04150W_A:UTP18:MIS12:MMS22:RTT109:CR_00430C_A:CR_00460C_A:MRPL6:ADE1:BUD22:PWP2:CR_00830W_A:TRM1:DAL81:CTF18:CR_01260W_A:CR_01320C_A:CR_01410C_A:PRS:CR_01550C_A:TRP2:CR_01600C_A:MET6:CR_01670W_A:CDC60:CR_01700C_A:CR_01710W_A:CR_01780W_A:CR_01950W_A:CR_02030C_A:MCM6:MET2:ERG25:CR_02420W_A:CR_02430C_A:CHO2:CR_02550C_A:PAN6:NUE1:RRP9:RPC31:CR_02890C_A:SRP40:ARC1:SMC5:CR_03110W_A:CR_03200C_A:CR_03230W_A:CR_03340C_A:CR_03360W_A:KTI12:MTG1:CR_03400W_A:CR_03440W_A:NCS2:YVH1:SGD1:SMC1:PRP42:CR_03760W_A:CR_03940W_A:MEX67:CR_04110W_A:CR_04160C_A:CR_04170W_A:QDR1:CR_04240C_A:CR_04300W_A:CR_04500C_A:CR_04560C_A:CR_04710W_A:ADE6:RPB8:CR_04920W_A:CR_05480W_A:NOC2:CR_05550C_A:RNR3:MTG2:DBP8:RFX1:RPL7:CR_06230W_A:FGR50:CR_06450W_A:RRN11:HSP60:CR_06680C_A:NMD3:CR_06840W_A:ATX1:CR_06970C_A:CR_06980W_A:POL2:MIS11:CR_07030C_A:URA2:CR_07310W_A:PIF1:CR_07480W_A:FUN30:CR_07640C_A:ARO2:CDC45:DBP6:ASF1:CR_07940W_A:IMP4:CR_08000C_A:NOP10:SSB1:RIO2:ATS1:CR_08330W_A:CYS3:RPP2A:GSH2:CR_08410W_A:TSR1:CR_08940W_A:MPS1:CR_09010C_A:CR_09310W_A:ELF1:SSF1:CR_09740W_A:MED21:CR_09800C_A:DPB2:SIK1:CR_09990W_A:CR_10180W_A:CR_10190C_A:UTP5:CR_10260W_A:CR_10410C_A:CR_10470C_A:MCD1:DRS1:YHB4:PRO1:LTV1:POP3

70925	organelle assembly	91 out of 1061 genes, 8.6%	203 out of 6473 background genes, 3.1%	5.03E-19	0.00%	RRS1:C1_04040C_A:ERB1:NOP4:C1_04710C_A:C1_04970W_A:RRP6:C1_05360C_A:RPS27A:C1_06760C_A:RIA1:ERD1:FUN12:JIP5:C1_09040C_A:C1_09710C_A:RPA190:C1_10970W_A:TRP3:CSI2:C1_12440W_A:C1_12760W_A:C1_14080W_A:C2_01070W_A:UTP22:C2_02540W_A:MAK5:C2_05160C_A:RPF2:C2_05750W_A:RPL11:C2_09180W_A:C2_10740C_A:BMS1:ARP9:C3_01560W_A:SCM3:C3_02020W_A:C3_02040C_A:C3_02350W_A:C3_02670W_A:SMC3:C3_04370C_A:MAK21:ARV1:C3_05160C_A:QDR2:C3_06370C_A:C4_02880C_A:C4_05010W_A:C4_05230C_A:TUB4:RMT2:SPB4:C5_02070C_A:TIF5:C5_04720C_A:HAS1:C5_05340W_A:CIC1:C6_02230W_A:ARP4:SPB1:RPA135:DBP7:FLU1:C7_03400C_A:ENP2:UTP18:BUD22:PWP2:CR_01410C_A:CR_01550C_A:YVH1:SGD1:CR_03940W_A:CR_04170W_A:NOC2:CR_05550C_A:SDA1:CR_06680C_A:CR_06740W_A:TSR1:MPS1:ELF1:SSF1:CR_09800C_A:CR_10180W_A:CR_10470C_A:DRS1:LTV1

8033	tRNA processing	58 out of 1061 genes, 5.5%	99 out of 6473 background genes, 1.5%	1.54E-18	0.00%	C1_01150C_A:TRM2:ABP140:RPP1:C1_04530C_A:C1_05360C_A:LHP1:C1_09390W_A:C1_09790C_A:C1_12570C_A:C1_14330W_A:C2_00820W_A:C2_02420C_A:KTI11:C2_05670C_A:C2_06480W_A:C2_07360W_A:C2_09500W_A:SMM1:PUS7:C3_02840W_A:C3_05140C_A:C3_05800W_A:TRM12:C3_07400W_A:RIT1:C4_00740W_A:C4_00810C_A:C4_00880W_A:C4_00940W_A:C4_01500W_A:C4_02850W_A:C4_03140C_A:C4_03730C_A:C4_03830W_A:C4_04810C_A:C4_05260W_A:NOP1:DUS4:PUS4:C5_01610W_A:C6_02290C_A:C6_02350C_A:POP4:C6_04530C_A:C7_00330C_A:THG1:LIG1:C7_02340C_A:TRM1:CR_03200C_A:KTI12:NCS2:CR_04160C_A:CR_04300W_A:ATS1:CR_08940W_A:POP3

447	endonucleolytic cleavage in ITS1 to separate SSU-rRNA from 5.8S rRNA and LSU-rRNA from tricistronic rRNA transcript (SSU-rRNA, 5.8S rRNA, LSU-rRNA)	59 out of 1061 genes, 5.6%	103 out of 6473 background genes, 1.6%	3.32E-18	0.00%	RRS1:RPP1:C1_04040C_A:DIP2:C1_09710C_A:NEP1:KRR1:C1_13820C_A:C1_14080W_A:MPP10:FAV3:C2_00200W_A:C2_00410C_A:C2_00820W_A:C2_02540W_A:C2_02630W_A:TBF1:FHL1:NOC4:RCL1:BUD21:UTP20:C3_02020W_A:C3_02040C_A:C3_02350W_A:C3_02840W_A:NOP14:ISW2:C3_06370C_A:RAD4:C3_07800C_A:DUR4:C4_00690C_A:HIT1:SAS10:C4_04520W_A:BAS1:BUD23:C5_02070C_A:UTP13:C5_03920C_A:C5_04840C_A:NOP5:ENP1:C7_03850W_A:UTP18:PWP2:CR_02420W_A:RRP9:CR_03200C_A:CR_03360W_A:DBP8:PIF1:ATS1:CR_08330W_A:MPS1:CR_09800C_A:CR_10260W_A:CR_10410C_A

9987	cellular process	879 out of 1061 genes, 82.8%	4625 out of 6473 background genes, 71.5%	9.44E-18	0.00%	RIM8:C1_00160C_A:LEU4:HBR1:C1_00510W_A:C1_00520W_A:CNS1:RPO41:SPE1:NRM1:RRS1:TRI1:NMA111:HMT1:ZPR1:C1_01150C_A:C1_01160C_A:TRM2:ABC1:C1_01530C_A:C1_01750W_A:CYS4:C1_02090C_A:ABP140:CWH8:C1_02380C_A:C1_02390W_A:C1_02450C_A:SNZ1:SNO1:HOM6:LYS2:PDE2:MIA40:C1_02900C_A:C1_02970W_A:TSR2:ARX1:C1_03260W_A:C1_03370W_A:C1_03540C_A:C1_03630W_A:C1_03790C_A:RPP1:C1_03830C_A:C1_03870C_A:KTR4:C1_04040C_A:C1_04120C_A:ERB1:RPN4:C1_04340C_A:NOP4:C1_04510W_A:C1_04530C_A:C1_04710C_A:MRPL37:C1_04970W_A:RHD1:C1_05010C_A:ARG2:RRP6:REI1:TRY2:ARO4:C1_05210C_A:C1_05220C_A:C1_05270C_A:C1_05330C_A:C1_05360C_A:C1_05380C_A:MTM1:RSM22:RPS27A:C1_05630C_A:FAL1:C1_05650W_A:RAD10:C1_05930C_A:C1_05990C_A:BUD16:C1_06510C_A:C1_06530C_A:C1_06540C_A:NOP6:C1_06760C_A:MEF2:RIA1:YMC1:C1_07340W_A:C1_07470C_A:LHP1:C1_07510W_A:C1_07660W_A:C1_07690C_A:ADE4:ECO1:GCS1:ADE5,7:MRPL3:ERD1:C1_07950C_A:C1_07960W_A:FUN12:DRG1:CAT8:MLT1:DIP2:SAM4:GCR3:UPC2:C1_08520C_A:GCD6:C1_08630W_A:SWI6:YMC2:MSS116:JIP5:C1_08890C_A:NAR1:C1_09040C_A:CTA8:CEF1:C1_09390W_A:GUA1:YTM1:C1_09610W_A:C1_09620C_A:C1_09710C_A:C1_09790C_A:C1_09840C_A:BUD31:C1_09910C_A:PDC2:DBP3:MNN23:C1_10080W_A:C1_10200C_A:C1_10340W_A:FCY23:C1_10620W_A:PEA2:APN2:RPA190:C1_10690W_A:ALP1:C1_10920W_A:C1_10950C_A:C1_10970W_A:C1_11000C_A:PET127:FGR39:NEP1:KRR1:SAM2:GAR1:C1_11610C_A:C1_11790W_A:MUP1:C1_11880W_A:C1_11900C_A:C1_11910W_A:SER33:TRP3:C1_12280C_A:CSI2:MRPS9:C1_12350W_A:WRS1:C1_12440W_A:C1_12570C_A:C1_12630C_A:C1_12640W_A:C1_12670C_A:C1_12680W_A:C1_12750C_A:C1_12760W_A:FEN1:C1_12820C_A:C1_13010W_A:RPL14:C1_13060C_A:C1_13130C_A:REX3:C1_13330C_A:RMP1:C1_13370W_A:C1_13380W_A:AGC1:COX19:KNS1:MCU1:C1_13560W_A:C1_13820C_A:SMF13:MET3:YBP1:C1_14080W_A:C1_14330W_A:MPP10:FAV3:C2_00200W_A:C2_00280C_A:C2_00390C_A:C2_00410C_A:C2_00810C_A:C2_00820W_A:C2_00840W_A:MXR1:HGT8:C2_01070W_A:C2_01220W_A:C2_01240C_A:ESC4:CLB2:C2_01420C_A:APT1:REX2:C2_01740C_A:C2_01820C_A:PRP39:C2_01860C_A:C2_01870C_A:ARO3:C2_02120W_A:SAM37:C2_02270C_A:ECM3:C2_02420C_A:UTP21:UTP22:PRS1:C2_02540W_A:C2_02580W_A:C2_02630W_A:PDR17:SER2:C2_02930C_A:MET1:BNA31:SMP3:PRP3:C2_03560C_A:MNN42:GPI13:SCH9:C2_03950W_A:C2_04120C_A:UGA1:TBF1:BAT21:C2_04340C_A:SUV3:RIM2:C2_04570W_A:SNU114:C2_04700C_A:C2_04820W_A:PZF1:CKA2:C2_04990W_A:C2_05050C_A:FRE9:C2_05080C_A:MAK5:C2_05160C_A:CDC21:SGO1:RPF2:C2_05270W_A:RPC40:KTI11:C2_05670C_A:C2_05750W_A:C2_05830C_A:C2_05840W_A:C2_05860C_A:IDP1:C2_05980C_A:LAS1:CSM3:ECM17:ILV1:SER1:MAK16:RTA3:C2_06480W_A:C2_06520C_A:C2_06530W_A:VAS1:C2_06650C_A:C2_06660W_A:RPL11:FHL1:C2_06850W_A:C2_06950C_A:SPE3:AAH1:RCK2:SMF12:DQD1:NOC4:C2_07360W_A:

						C2_07410W_A:RCL1:C2_07680W_A:PHO86:PRS5:C2_07920W_A:NSA1:KRE30:RPS10:SIT1:C2_08180C_A:EAF3:RRP8:C2_08530C_A:MSU1:SMC2:UTP15:MFG1:APE3:CDC47:ASN1:MET10:C2_09160W_A:C2_09180W_A:C2_09290W_A:MED18:C2_09310C_A:PES1:TAZ1:RRP15:C2_09500W_A:C2_09660W_A:LEU42:COE1:SMM1:TIM23:C2_09920W_A:CWC22:UBP8:GPD1:PUS7:HEM3:C2_10740C_A:RPC11:C2_10810W_A:RPS24:C3_00100W_A:ULP3:RHR2:UTP8:BUD21:SOF1:C3_00660W_A:C3_00830C_A:BMS1:ARP9:UTP20:NCE103:C3_01310W_A:URA3:MET18:RKI1:RPS7A:C3_01520C_A:C3_01560W_A:C3_01680C_A:PPT1:SCM3:C3_01850W_A:C3_02020W_A:C3_02040C_A:UTP4:C3_02180C_A:ILV2:C3_02350W_A:ALR1:PHA2:DDC1:C3_02670W_A:SMC3:C3_02750W_A:C3_02760C_A:C3_02840W_A:C3_02850C_A:C3_02870C_A:MET13:C3_03070W_A:C3_03110W_A:C3_03330C_A:YAH1:C3_03470W_A:C3_03560W_A:HBR3:RAD53:EGD2:CTP1:C3_04370C_A:C3_04380C_A:ADE2:MAK21:RPS15:RPP2B:ARV1:SFP1:NOP14:CEM1:C3_05140C_A:C3_05160C_A:WOR2:RPL8B:C3_05280C_A:C3_05380W_A:GPI1:C3_05440C_A:C3_05510W_A:QDR2:BAT22:SAM50:C3_05800W_A:C3_05860C_A:TRM12:C3_05900W_A:CTA4:NOG1:C3_06150W_A:MMS21:OAC1:C3_06240C_A:C3_06270C_A:DOT1:ISW2:C3_06370C_A:NSA2:OPI3:C3_06600C_A:FUN31:C3_06630W_A:PRP5:C3_06830C_A:RPS12:NOP13:C3_07400W_A:C3_07550C_A:C3_07570C_A:RIT1:RAD4:UTP9:C3_07800C_A:HIS4:MET15:DUR4:RLP24:NMD5:HIS5:C4_00690C_A:C4_00700C_A:C4_00740W_A:MLH1:C4_00800W_A:C4_00810C_A:C4_00880W_A:ARO1:C4_00940W_A:PTC8:RAT1:C4_01130C_A:AAT22:C4_01280C_A:PGA53:ILV6:PWP1:C4_01470W_A:NIP1:C4_01500W_A:C4_01560C_A:TOA2:HSX11:TIM10:ZCF25:C4_02260C_A:C4_02400C_A:SLX4:SUL2:C4_02720C_A:HIT1:C4_02770C_A:SAS10:RPC53:HCA4:C4_02850W_A:ZUO1:C4_02880C_A:GST2:NAN1:C4_03140C_A:C4_03170W_A:DAO2:C4_03410W_A:RAD2:C4_03700W_A:C4_03720C_A:C4_03730C_A:C4_03740W_A:NUP84:C4_03830W_A:RRP42:C4_03870C_A:RAM1:FGR28:ECM1:C4_04130W_A:DUO1:C4_04390W_A:SAP10:C4_04520W_A:SSZ1:BAS1:C4_04810C_A:C4_04820C_A:AGP3:FGR10:RPL30:C4_05010W_A:C4_05230C_A:C4_05260W_A:C4_05360C_A:OFD1:HOM3:TUB4:C4_05650W_A:C4_05890W_A:TRP5:C4_06210C_A:C4_06410W_A:C4_06450W_A:NOP1:C4_06790W_A:C4_06850C_A:C4_06950W_A:MET16:C4_07060W_A:TRP4:C4_07140W_A:C4_07150W_A:C5_00030W_A:C5_00250C_A:C5_00260W_A:C5_00280C_A:DUS4:C5_00320W_A:MRP17:MET14:IFG3:EXO1:RMT2:SPE2:C5_00820W_A:C5_00920W_A:BUD23:CLN3:CYC3:C5_01140C_A:PUS4:C5_01430C_A:FYV5:SPB4:C5_01610W_A:CCN1:C5_01700W_A:C5_01930W_A:C5_02010C_A:C5_02070C_A:RIX7:THR4:PDE1:C5_02440C_A:TIF5:SPR28:UTP13:C5_02590C_A:BUR2:C5_02740W_A:CKB2:MSW1:C5_02780W_A:C5_02820C_A:C5_03010W_A:RMS1:CAM1-1:C5_03290C_A:PCL1:RPO26:FUR1:C5_03400C_A:C5_03460C_A:C5_03470C_A:C5_03530C_A:C5_03550W_A:MDJ1:C5_03840W_A:HAM1:C5_03920C_A:C5_03970W_

						A:C5_04120C_A:SAH1:C5_04290C_A:C5_04340W_A:URA7:CSU57:MSM1:C5_04720C_A:HYS2:HAS1:C5_04840C_A:C5_04910W_A:C5_05000C_A:C5_05010W_A:GRF10:PCL5:C5_05250C_A:SDH4:HIS1:C5_05340W_A:ILV3:DNA2:C6_00360C_A:NOP5:C6_00530C_A:C6_00560W_A:FCA1:C6_00640C_A:C6_00660C_A:CDC14:C6_00760W_A:TOP1:HAL22:C6_01040C_A:CIP1:C6_01120C_A:CIC1:QDR3:C6_01300W_A:PMP1:TIF3:RAD18:HCH1:C6_01890C_A:C6_01980C_A:C6_02150C_A:SWD3:C6_02230W_A:RPL10A:ALG11:C6_02280W_A:C6_02290C_A:C6_02300C_A:C6_02350C_A:C6_02370C_A:C6_02420W_A:C6_02430W_A:PRP45:C6_02690C_A:MRT4:C6_02900C_A:POP4:C6_02970C_A:C6_03210C_A:BMT4:C6_03320W_A:C6_03330C_A:ARP4:C6_03380W_A:C6_03390W_A:C6_03440W_A:NAM2:C6_03540W_A:PAD1:NOG2:OPT8:SPB1:C6_04240W_A:NOP8:ILS1:C6_04530C_A:C7_00160C_A:C7_00220W_A:C7_00330C_A:DCC1:PSF3:THG1:RPA135:NOP15:YML6:C7_01020C_A:C7_01030C_A:DBP7:C7_01210C_A:ASC1:C7_01270C_A:PSF1:C7_01360C_A:QCE1:C7_01400C_A:GIR2:PRT1:FLU1:C7_01570C_A:SMC6:C7_01600W_A:C7_01660C_A:C7_01950W_A:LIG1:C7_02340C_A:C7_02460C_A:C7_02660C_A:C7_02930C_A:IFM1:C7_03400C_A:ISY1:ENP2:ARG4:C7_03590C_A:ENP1:HIS7:MRP7:C7_03850W_A:C7_03880C_A:MAM33:C7_04150W_A:UTP18:NRG1:MIS12:MMS22:RTT109:CR_00430C_A:CR_00460C_A:MRPL6:ADE1:BUD22:PWP2:CR_00830W_A:TRM1:DAL81:CTF18:CR_01260W_A:CR_01320C_A:CR_01410C_A:PRS:CR_01550C_A:TRP2:CR_01600C_A:MET6:CR_01670W_A:CDC60:CR_01700C_A:CR_01710W_A:CR_01780W_A:CR_01950W_A:MRS2:CR_02030C_A:MCM6:MET2:ERG25:CR_02420W_A:CR_02430C_A:CHO2:CR_02550C_A:PAN6:NUE1:RRP9:RPC31:CR_02890C_A:SRP40:ARC1:SMC5:CR_03110W_A:CR_03200C_A:CR_03230W_A:CR_03340C_A:CR_03360W_A:KTI12:MTG1:CR_03400W_A:CR_03440W_A:NCS2:YVH1:SGD1:SMC1:PRP42:CR_03760W_A:CR_03940W_A:MEX67:CR_04110W_A:CR_04160C_A:CR_04170W_A:QDR1:CR_04240C_A:CR_04300W_A:CR_04500C_A:CR_04560C_A:CR_04710W_A:ADE6:RPB8:CR_04920W_A:BIR1:CR_05480W_A:NOC2:CR_05550C_A:RNR3:MTG2:DBP8:SDA1:RFX1:RPL7:CR_06230W_A:FGR50:CR_06450W_A:RRN11:HSP60:CR_06680C_A:NMD3:CR_06740W_A:CR_06840W_A:BUD5:ATX1:CR_06970C_A:CR_06980W_A:POL2:MIS11:CR_07030C_A:URA2:CR_07310W_A:PIF1:CR_07480W_A:FUN30:CR_07640C_A:ARO2:CDC45:DBP6:ASF1:CR_07940W_A:IMP4:CR_08000C_A:NOP10:SSB1:RIO2:ATS1:CR_08330W_A:CYS3:RPP2A:GSH2:CR_08410W_A:TSR1:CR_08500W_A:CR_08940W_A:MPS1:CR_09010C_A:ELF1:SSF1:CR_09740W_A:MED21:CR_09800C_A:DPB2:SIK1:CR_09990W_A:CR_10180W_A:CR_10190C_A:UTP5:CR_10260W_A:CR_10410C_A:CR_10470C_A:MCD1:DRS1:PRO1:LTV1:POP3

6400	tRNA modification	45 out of 1061 genes, 4.2%	71 out of 6473 background genes, 1.1%	7.66E-16	0.00%	C1_01150C_A:TRM2:ABP140:C1_04530C_A:C1_05360C_A:C1_09390W_A:C1_09790C_A:C1_12570C_A:C1_14330W_A:C2_00820W_A:C2_02420C_A:KTI11:C2_06480W_A:C2_09500W_A:SMM1:PUS7:C3_05140C_A:TRM12:C3_07400W_A:RIT1:C4_00810C_A:C4_00940W_A:C4_01500W_A:C4_02850W_A:C4_03140C_A:C4_03730C_A:C4_03830W_A:C4_04810C_A:C4_05260W_A:DUS4:PUS4:C5_01610W_A:C6_02290C_A:C6_02350C_A:C6_04530C_A:C7_00330C_A:THG1:C7_02340C_A:TRM1:KTI12:NCS2:CR_04160C_A:CR_04300W_A:ATS1:CR_08940W_A

65003	protein-containing complex assembly	145 out of 1061 genes, 13.7%	444 out of 6473 background genes, 6.9%	2.19E-15	0.00%	C1_02380C_A:C1_02900C_A:C1_03790C_A:C1_04040C_A:C1_04120C_A:ERB1:NOP4:C1_04710C_A:C1_04970W_A:RRP6:C1_05360C_A:RPS27A:C1_06760C_A:ERD1:FUN12:JIP5:C1_09040C_A:CEF1:C1_09620C_A:C1_09710C_A:RPA190:C1_10970W_A:TRP3:CSI2:C1_12440W_A:C1_12640W_A:C1_12750C_A:C1_12760W_A:C1_13010W_A:COX19:C1_14080W_A:C2_00200W_A:C2_00280C_A:C2_01070W_A:PRP39:SAM37:UTP22:C2_02540W_A:PRP3:SNU114:MAK5:C2_05160C_A:SGO1:RPF2:C2_05750W_A:C2_05830C_A:C2_06530W_A:C2_06650C_A:RPL11:C2_06950C_A:CDC47:C2_09180W_A:C2_09290W_A:MED18:TAZ1:C2_10740C_A:C3_00100W_A:C3_00660W_A:BMS1:ARP9:C3_01560W_A:SCM3:C3_02020W_A:C3_02040C_A:C3_02350W_A:C3_02670W_A:SMC3:C3_03330C_A:HBR3:C3_04370C_A:MAK21:ARV1:C3_05160C_A:QDR2:NOG1:DOT1:C3_06370C_A:C3_06600C_A:PRP5:RLP24:TOA2:HIT1:C4_02770C_A:C4_04130W_A:C4_05010W_A:C4_05230C_A:TUB4:C4_06410W_A:RMT2:CYC3:SPB4:C5_01930W_A:C5_02010C_A:C5_02070C_A:TIF5:C5_02740W_A:C5_04720C_A:HAS1:C5_05340W_A:CIC1:TIF3:C6_02230W_A:PRP45:ARP4:SPB1:RPA135:DBP7:FLU1:C7_01600W_A:C7_03400C_A:ISY1:ENP2:C7_03850W_A:UTP18:BUD22:PWP2:CR_00830W_A:DAL81:CR_01410C_A:CR_01550C_A:CR_01700C_A:MCM6:ARC1:CR_03340C_A:YVH1:SGD1:CR_03940W_A:CR_04110W_A:CR_04170W_A:NOC2:CR_05550C_A:SDA1:HSP60:CR_06680C_A:CR_06740W_A:CDC45:TSR1:ELF1:SSF1:CR_09800C_A:CR_10180W_A:CR_10410C_A:CR_10470C_A:DRS1:LTV1

1510	RNA methylation	27 out of 1061 genes, 2.5%	35 out of 6473 background genes, 0.5%	4.83E-12	0.00%	C1_01150C_A:ABP140:C1_04530C_A:NEP1:C2_06480W_A:C2_07360W_A:RRP8:C3_05140C_A:C3_05860C_A:TRM12:C3_07400W_A:C4_03730C_A:C4_03830W_A:C4_04810C_A:NOP1:BUD23:SPB1:C6_04530C_A:C7_02340C_A:TRM1:CR_01780W_A:CR_02030C_A:CR_04160C_A:CR_04170W_A:CR_04300W_A:CR_08940W_A:CR_10260W_A
6520	amino acid metabolic process	73 out of 1061 genes, 6.9%	191 out of 6473 background genes, 3.0%	2.41E-10	0.00%	LEU4:C1_01530C_A:CYS4:SNZ1:HOM6:LYS2:ARG2:ARO4:SAM4:SAM2:C1_11610C_A:SER33:TRP3:WRS1:C2_00390C_A:MXR1:C2_01860C_A:ARO3:PRS1:SER2:MET1:BNA31:UGA1:BAT21:IDP1:ILV1:SER1:VAS1:C2_08530C_A:ASN1:C2_09310C_A:LEU42:ILV2:PHA2:MET13:C3_03470W_A:BAT22:HIS4:MET15:HIS5:ARO1:AAT22:ILV6:HOM3:TRP5:MET16:TRP4:IFG3:THR4:MSW1:C5_02820C_A:SAH1:MSM1:HIS1:ILV3:NAM2:ILS1:ARG4:HIS7:PRS:TRP2:MET6:CDC60:MET2:ARC1:CR_03110W_A:CR_03400W_A:MIS11:URA2:ARO2:CYS3:CR_09010C_A:PRO1

16053	organic acid biosynthetic process	65 out of 1061 genes, 6.1%	164 out of 6473 background genes, 2.5%	7.79E-10	0.00%	RIM8:LEU4:C1_00510W_A:SPE1:HMT1:CYS4:SNZ1:HOM6:LYS2:C1_02970W_A:ARG2:ARO4:C1_05990C_A:SAM4:C1_11610C_A:SER33:TRP3:FEN1:C1_13330C_A:C2_00390C_A:MXR1:C2_01860C_A:ARO3:PRS1:SER2:MET1:BNA31:BAT21:IDP1:ECM17:ILV1:SER1:SPE3:C2_07410W_A:C2_08530C_A:ASN1:LEU42:ILV2:PHA2:MET13:HIS4:MET15:HIS5:ARO1:ILV6:HOM3:TRP5:MET16:TRP4:SPE2:THR4:C5_02820C_A:HIS1:ILV3:ARG4:HIS7:TRP2:MET6:MET2:ERG25:PAN6:MIS11:ARO2:CYS3:PRO1
46394	carboxylic acid biosynthetic process	65 out of 1061 genes, 6.1%	164 out of 6473 background genes, 2.5%	7.79E-10	0.00%	RIM8:LEU4:C1_00510W_A:SPE1:HMT1:CYS4:SNZ1:HOM6:LYS2:C1_02970W_A:ARG2:ARO4:C1_05990C_A:SAM4:C1_11610C_A:SER33:TRP3:FEN1:C1_13330C_A:C2_00390C_A:MXR1:C2_01860C_A:ARO3:PRS1:SER2:MET1:BNA31:BAT21:IDP1:ECM17:ILV1:SER1:SPE3:C2_07410W_A:C2_08530C_A:ASN1:LEU42:ILV2:PHA2:MET13:HIS4:MET15:HIS5:ARO1:ILV6:HOM3:TRP5:MET16:TRP4:SPE2:THR4:C5_02820C_A:HIS1:ILV3:ARG4:HIS7:TRP2:MET6:MET2:ERG25:PAN6:MIS11:ARO2:CYS3:PRO1

43414	macromolecule methylation	40 out of 1061 genes, 3.8%	77 out of 6473 background genes, 1.2%	8.77E-10	0.00%	HMT1:C1_01150C_A:ABP140:C1_04530C_A:C1_09040C_A:NEP1:C1_14160W_A:C2_00620C_A:C2_03130W_A:C2_06480W_A:C2_07360W_A:RRP8:UBP8:C3_05140C_A:C3_05860C_A:TRM12:DOT1:C3_07400W_A:C4_03730C_A:C4_03830W_A:C4_04810C_A:NOP1:RMT2:BUD23:RMS1:C6_01120C_A:SWD3:SPB1:C6_04530C_A:C7_02340C_A:TRM1:CR_01780W_A:CR_02030C_A:CR_03760W_A:CR_04160C_A:CR_04170W_A:CR_04300W_A:CR_08940W_A:CR_09310W_A:CR_10260W_A
32259	methylation	40 out of 1061 genes, 3.8%	78 out of 6473 background genes, 1.2%	1.52E-09	0.00%	HMT1:C1_01150C_A:ABP140:C1_04530C_A:C1_09040C_A:NEP1:C1_14160W_A:C2_00620C_A:C2_03130W_A:C2_06480W_A:C2_07360W_A:RRP8:UBP8:C3_05140C_A:C3_05860C_A:TRM12:DOT1:C3_07400W_A:C4_03730C_A:C4_03830W_A:C4_04810C_A:NOP1:RMT2:BUD23:RMS1:C6_01120C_A:SWD3:SPB1:C6_04530C_A:C7_02340C_A:TRM1:CR_01780W_A:CR_02030C_A:CR_03760W_A:CR_04160C_A:CR_04170W_A:CR_04300W_A:CR_08940W_A:CR_09310W_A:CR_10260W_A

8380	RNA splicing	50 out of 1061 genes, 4.7%	119 out of 6473 background genes, 1.8%	3.32E-08	0.00%	C1_02090C_A:C1_02900C_A:C1_04120C_A:TRY2:GCR3:MSS116:C1_08890C_A:CEF1:BUD31:C1_10080W_A:C1_12630C_A:C1_12670C_A:C1_12750C_A:C1_13380W_A:C2_00200W_A:C2_00280C_A:C2_01820C_A:PRP39:PRP3:SUV3:SNU114:C2_05670C_A:C2_06650C_A:CWC22:UBP8:C2_10810W_A:C3_00100W_A:C3_00660W_A:C3_01560W_A:C3_03330C_A:C3_04380C_A:C3_05800W_A:PRP5:C4_01280C_A:C4_05360C_A:C4_06410W_A:C5_00920W_A:C5_01930W_A:C5_03010W_A:C5_03400C_A:C6_00530C_A:PRP45:C6_02690C_A:C6_03440W_A:NAM2:LIG1:ISY1:CR_01700C_A:CR_03230W_A:PRP42
8652	amino acid biosynthetic process	43 out of 1061 genes, 4.1%	97 out of 6473 background genes, 1.5%	1.07E-07	0.00%	LEU4:CYS4:SNZ1:HOM6:LYS2:ARG2:SAM4:C1_11610C_A:SER33:TRP3:C2_00390C_A:MXR1:C2_01860C_A:PRS1:SER2:MET1:BAT21:IDP1:ILV1:SER1:C2_08530C_A:ASN1:LEU42:ILV2:PHA2:MET13:HIS4:MET15:HIS5:HOM3:TRP5:MET16:TRP4:THR4:C5_02820C_A:HIS1:ARG4:HIS7:TRP2:MET6:MET2:CYS3:PRO1

71035	nuclear polyadenylation-dependent rRNA catabolic process	14 out of 1061 genes, 1.3%	15 out of 6473 background genes, 0.2%	2.00E-07	0.00%	C1_01160C_A:C1_03830C_A:RRP6:C1_05360C_A:C1_06540C_A:C1_07960W_A:C1_09790C_A:C1_12350W_A:C2_06660W_A:C2_09160W_A:RAT1:C4_03740W_A:C7_03400C_A:CR_02550C_A
154	rRNA modification	17 out of 1061 genes, 1.6%	21 out of 6473 background genes, 0.3%	2.06E-07	0.00%	C1_10620W_A:NEP1:C2_02120W_A:C2_04820W_A:RRP8:PUS7:C3_05860C_A:NOP1:C4_06950W_A:BUD23:SPB1:CR_01780W_A:CR_02030C_A:CR_04170W_A:CR_04240C_A:NOP10:CR_10260W_A
43633	polyadenylation-dependent RNA catabolic process	15 out of 1061 genes, 1.4%	17 out of 6473 background genes, 0.3%	2.49E-07	0.00%	C1_01160C_A:C1_03830C_A:RRP6:C1_05360C_A:C1_06540C_A:C1_07960W_A:C1_09790C_A:C1_12350W_A:C2_06660W_A:C2_09160W_A:RAT1:C4_03740W_A:RRP42:C7_03400C_A:CR_02550C_A
43634	polyadenylation-dependent ncRNA catabolic process	15 out of 1061 genes, 1.4%	17 out of 6473 background genes, 0.3%	2.49E-07	0.00%	C1_01160C_A:C1_03830C_A:RRP6:C1_05360C_A:C1_06540C_A:C1_07960W_A:C1_09790C_A:C1_12350W_A:C2_06660W_A:C2_09160W_A:RAT1:C4_03740W_A:RRP42:C7_03400C_A:CR_02550C_A

71029	nuclear ncRNA surveillance	15 out of 1061 genes, 1.4%	17 out of 6473 background genes, 0.3%	2.49E-07	0.00%	C1_01160C_A:C1_03830C_A:RRP6:C1_05360C_A:C1_06540C_A:C1_07960W_A:C1_09790C_A:C1_12350W_A:C2_06660W_A:C2_09160W_A:RAT1:C4_03740W_A:RRP42:C7_03400C_A:CR_02550C_A
71046	nuclear polyadenylation-dependent ncRNA catabolic process	15 out of 1061 genes, 1.4%	17 out of 6473 background genes, 0.3%	2.49E-07	0.00%	C1_01160C_A:C1_03830C_A:RRP6:C1_05360C_A:C1_06540C_A:C1_07960W_A:C1_09790C_A:C1_12350W_A:C2_06660W_A:C2_09160W_A:RAT1:C4_03740W_A:RRP42:C7_03400C_A:CR_02550C_A
375	RNA splicing, via transesterification reactions	45 out of 1061 genes, 4.2%	107 out of 6473 background genes, 1.7%	3.30E-07	0.00%	C1_02090C_A:C1_02900C_A:C1_04120C_A:TRY2:GCR3:MSS116:C1_08890C_A:CEF1:BUD31:C1_10080W_A:C1_12630C_A:C1_12670C_A:C1_12750C_A:C1_13380W_A:C2_00200W_A:C2_00280C_A:C2_01820C_A:PRP39:PRP3:SUV3:SNU114:C2_06650C_A:CWC22:C2_10810W_A:C3_00100W_A:C3_00660W_A:C3_01560W_A:C3_03330C_A:C3_04380C_A:PRP5:C4_01280C_A:C4_05360C_A:C4_06410W_A:C5_00920W_A:C5_01930W_A:C5_03010W_A:C5_03400C_A:C6_00530C_A:PRP45:C6_02690C_A:NAM2:ISY1:CR_01700C_A:CR_03230W_A:PRP42

43933	protein-containing complex organization	169 out of 1061 genes, 15.9%	665 out of 6473 background genes, 10.3%	3.48E-07	0.00%	C1_02380C_A:C1_02900C_A:C1_03630W_A:C1_03790C_A:C1_04040C_A:C1_04120C_A:ERB1:NOP4:C1_04710C_A:C1_04970W_A:RRP6:C1_05360C_A:C1_05380C_A:RPS27A:C1_06760C_A:C1_07340W_A:ERD1:FUN12:JIP5:C1_09040C_A:CEF1:YTM1:C1_09620C_A:C1_09710C_A:RPA190:C1_10970W_A:TRP3:CSI2:C1_12440W_A:C1_12640W_A:C1_12750C_A:C1_12760W_A:C1_13010W_A:COX19:C1_14080W_A:C2_00200W_A:C2_00280C_A:C2_01070W_A:C2_01220W_A:PRP39:SAM37:UTP22:C2_02540W_A:C2_02930C_A:PRP3:SNU114:MAK5:C2_05160C_A:SGO1:RPF2:C2_05750W_A:C2_05830C_A:C2_06530W_A:C2_06650C_A:RPL11:C2_06950C_A:SMC2:CDC47:C2_09180W_A:C2_09290W_A:MED18:TAZ1:C2_10740C_A:C3_00100W_A:C3_00660W_A:BMS1:ARP9:C3_01560W_A:SCM3:C3_02020W_A:C3_02040C_A:C3_02350W_A:C3_02670W_A:SMC3:C3_03330C_A:HBR3:C3_04370C_A:MAK21:ARV1:C3_05160C_A:WOR2:QDR2:NOG1:DOT1:ISW2:C3_06370C_A:C3_06600C_A:PRP5:RLP24:C4_01130C_A:TOA2:HIT1:C4_02770C_A:NUP84:C4_04130W_A:C4_05010W_A:C4_05230C_A:OFD1:TUB4:C4_06410W_A:C4_06450W_A:RMT2:CYC3:SPB4:C5_01930W_A:C5_02010C_A:C5_02070C_A:TIF5:C5_02740W_A:C5_02780W_A:C5_03550W_A:C5_04720C_A:HAS1:GRF10:C5_05340W_A:C6_00560W_A:TOP1:CIC1:TIF3:C6_02230W_A:PRP45:C6_02690C_A:ARP4:SPB1:RPA135:DBP7:C7_01400C_A:FLU1:C7_01600W_A:C7_03400C_A:ISY1:ENP2:C7_03850W_A:UTP18:BUD22:PWP2:CR_00830W_A:DAL81:CR_01410C_A:CR_01550C_A:CR_01700C_A:MCM6:ARC1:CR_03340C_A:YVH1:SGD1:CR_03760W_A:CR_03940W_A:CR_04110W_A:CR_04170W_A:NOC2:CR_05550C_A:SDA1:HSP60:CR_06680C_A:CR_06740W_A:POL2:FUN30:CDC45:ASF1:TSR1:ELF1:SSF1:CR_09800C_A:CR_10180W_A:CR_10410C_A:CR_10470C_A:DRS1:LTV1

2E+06	alpha-amino acid biosynthetic process	39 out of 1061 genes, 3.7%	87 out of 6473 background genes, 1.3%	5.58E-07	0.00%	LEU4:CYS4:SNZ1:HOM6:LYS2:ARG2:SAM4:C1_11610C_A:SER33:TRP3:C2_00390C_A:MXR1:C2_01860C_A:PRS1:SER2:MET1:BAT21:IDP1:ILV1:SER1:C2_08530C_A:ASN1:LEU42:ILV2:PHA2:MET13:MET15:HOM3:TRP5:MET16:TRP4:THR4:C5_02820C_A:ARG4:TRP2:MET6:MET2:CYS3:PRO1
96	sulfur amino acid metabolic process	23 out of 1061 genes, 2.2%	37 out of 6473 background genes, 0.6%	6.92E-07	0.00%	CYS4:SNZ1:HOM6:C1_02970W_A:SAM4:SAM2:C1_11610C_A:MET3:C2_00390C_A:MXR1:C2_01860C_A:MET1:ECM17:MET13:MET15:HOM3:MET16:MET14:C5_02820C_A:MET6:MET2:CYS3:CR_09010C_A
1E+05	tRNA surveillance	13 out of 1061 genes, 1.2%	14 out of 6473 background genes, 0.2%	1.15E-06	0.00%	C1_01160C_A:C1_03830C_A:RRP6:C1_05360C_A:C1_06540C_A:C1_09790C_A:C1_12350W_A:C2_06660W_A:C2_09160W_A:C4_03740W_A:RRP42:C7_03400C_A:CR_02550C_A
71038	nuclear polyadenylation-dependent tRNA catabolic process	13 out of 1061 genes, 1.2%	14 out of 6473 background genes, 0.2%	1.15E-06	0.00%	C1_01160C_A:C1_03830C_A:RRP6:C1_05360C_A:C1_06540C_A:C1_09790C_A:C1_12350W_A:C2_06660W_A:C2_09160W_A:C4_03740W_A:RRP42:C7_03400C_A:CR_02550C_A

97	sulfur amino acid biosynthetic process	19 out of 1061 genes, 1.8%	28 out of 6473 background genes, 0.4%	2.69E-06	0.00%	CYS4:SNZ1:HOM6:C1_02970W_A:SAM4:C1_11610C_A:C2_00390C_A:MXR1:C2_01860C_A:MET1:ECM17:MET13:MET15:HOM3:MET16:C5_02820C_A:MET6:MET2:CYS3
377	RNA splicing, via transesterification reactions with bulged adenosine as nucleophile	42 out of 1061 genes, 4.0%	102 out of 6473 background genes, 1.6%	2.92E-06	0.00%	C1_02090C_A:C1_02900C_A:C1_04120C_A:TRY2:GCR3:MSS116:C1_08890C_A:CEF1:BUD31:C1_10080W_A:C1_12630C_A:C1_12670C_A:C1_12750C_A:C1_13380W_A:C2_00200W_A:C2_00280C_A:C2_01820C_A:PRP39:PRP3:SNU114:C2_06650C_A:CWC22:C2_10810W_A:C3_00100W_A:C3_00660W_A:C3_01560W_A:C3_03330C_A:C3_04380C_A:PRP5:C4_01280C_A:C4_06410W_A:C5_00920W_A:C5_01930W_A:C5_03010W_A:C5_03400C_A:C6_00530C_A:PRP45:C6_02690C_A:ISY1:CR_01700C_A:CR_03230W_A:PRP42

2E+06	alpha-amino acid metabolic process	48 out of 1061 genes, 4.5%	125 out of 6473 background genes, 1.9%	3.36E-06	0.00%	LEU4:CYS4:SNZ1:HOM6:LYS2:ARG2:SAM4:SAM2:C1_11610C_A:SER33:TRP3:C2_00390C_A:MXR1:C2_01860C_A:PRS1:SER2:MET1:BNA31:UGA1:BAT21:IDP1:ILV1:SER1:C2_08530C_A:ASN1:LEU42:ILV2:PHA2:MET13:C3_03470W_A:MET15:HOM3:TRP5:MET16:TRP4:IFG3:THR4:C5_02820C_A:SAH1:ARG4:TRP2:MET6:MET2:MIS11:URA2:CYS3:CR_09010C_A:PRO1

22607	cellular component assembly	157 out of 1061 genes, 14.8%	623 out of 6473 background genes, 9.6%	3.37E-06	0.00%	RRS1:C1_02380C_A:C1_02900C_A:C1_03790C_A:C1_04040C_A:C1_04120C_A:ERB1:NOP4:C1_04710C_A:C1_04970W_A:RRP6:C1_05360C_A:RPS27A:C1_06760C_A:RIA1:ERD1:FUN12:JIP5:NAR1:C1_09040C_A:CEF1:C1_09620C_A:C1_09710C_A:RPA190:C1_10970W_A:TRP3:CSI2:C1_12440W_A:C1_12640W_A:C1_12750C_A:C1_12760W_A:C1_13010W_A:COX19:C1_14080W_A:C2_00200W_A:C2_00280C_A:C2_00810C_A:C2_00840W_A:C2_01070W_A:PRP39:SAM37:UTP22:C2_02540W_A:PRP3:SNU114:MAK5:C2_05160C_A:SGO1:RPF2:C2_05750W_A:C2_05830C_A:C2_06530W_A:C2_06650C_A:RPL11:C2_06950C_A:CDC47:C2_09180W_A:C2_09290W_A:MED18:TAZ1:C2_10740C_A:C3_00100W_A:C3_00660W_A:BMS1:ARP9:C3_01560W_A:SCM3:C3_02020W_A:C3_02040C_A:C3_02350W_A:C3_02670W_A:SMC3:C3_03330C_A:YAH1:HBR3:C3_04370C_A:MAK21:ARV1:C3_05160C_A:QDR2:NOG1:DOT1:C3_06370C_A:C3_06600C_A:PRP5:RLP24:TOA2:C4_02400C_A:HIT1:C4_02770C_A:C4_02880C_A:C4_04130W_A:C4_05010W_A:C4_05230C_A:TUB4:C4_06410W_A:RMT2:CYC3:SPB4:C5_01930W_A:C5_02010C_A:C5_02070C_A:TIF5:C5_02740W_A:C5_04720C_A:HAS1:C5_05340W_A:C6_00560W_A:CIC1:QDR3:TIF3:C6_02230W_A:PRP45:ARP4:SPB1:RPA135:DBP7:FLU1:C7_01600W_A:C7_03400C_A:ISY1:ENP2:C7_03850W_A:UTP18:BUD22:PWP2:CR_00830W_A:DAL81:CR_01410C_A:CR_01550C_A:CR_01700C_A:MCM6:ARC1:CR_03340C_A:YVH1:SGD1:CR_03940W_A:CR_04110W_A:CR_04170W_A:QDR1:NOC2:CR_05550C_A:SDA1:HSP60:CR_06680C_A:CR_06740W_A:CDC45:TSR1:MPS1:ELF1:SSF1:CR_09800C_A:CR_10180W_A:CR_10410C_A:CR_10470C_A:DRS1:LTV1

30488	tRNA methylation	16 out of 1061 genes, 1.5%	21 out of 6473 background genes, 0.3%	3.67E-06	0.00%	C1_01150C_A:ABP140:C1_04530C_A:C2_06480W_A:C3_05140C_A:TRM12:C3_07400W_A:C4_03730C_A:C4_03830W_A:C4_04810C_A:C6_04530C_A:C7_02340C_A:TRM1:CR_04160C_A:CR_04300W_A:CR_08940W_A		
33750	ribosome localization	33 out of 1061 genes, 3.1%	72 out of 6473 background genes, 1.1%	6.48E-06	0.00%	RRS1:C1_02450C_A:ARX1:C1_03630W_A:C1_04040C_A:C1_06530C_A:C1_12760W_A:C2_01220W_A:C2_05750W_A:C2_06850W_A:KRE30:MFG1:C3_02040C_A:SFP1:NOG1:C3_06150W_A:ZUO1:ECM1:C4_06210C_A:BUD23:RIX7:C6_02230W_A:RPL10A:MRT4:NOG2:C7_04150W_A:YVH1:MEX67:SDA1:RFX1:NMD3:SSB1:LTV1		

54	ribosomal subunit export from nucleus	33 out of 1061 genes, 3.1%	72 out of 6473 background genes, 1.1%	6.48E-06	0.00%	RRS1:C1_02450C_A:ARX1:C1_03630W_A:C1_04040C_A:C1_06530C_A:C1_12760W_A:C2_01220W_A:C2_05750W_A:C2_06850W_A:KRE30:MFG1:C3_02040C_A:SFP1:NOG1:C3_06150W_A:ZUO1:ECM1:C4_06210C_A:BUD23:RIX7:C6_02230W_A:RPL10A:MRT4:NOG2:C7_04150W_A:YVH1:MEX67:SDA1:RFX1:NMD3:SSB1:LTV1
467	exonucleolytic trimming to generate mature 3'-end of 5.8S rRNA from tricistronic rRNA transcript (SSU-rRNA, 5.8S rRNA, LSU-rRNA)	14 out of 1061 genes, 1.3%	17 out of 6473 background genes, 0.3%	6.53E-06	0.00%	C1_01160C_A:C1_03830C_A:RRP6:C1_06540C_A:C1_07960W_A:C1_12350W_A:REX3:REX2:C2_06660W_A:C2_09160W_A:C4_03740W_A:RRP42:C7_03400C_A:CR_02550C_A

16071	mRNA metabolic process	73 out of 1061 genes, 6.9%	231 out of 6473 background genes, 3.6%	7.20E-06	0.00%	C1_02090C_A:C1_02900C_A:C1_03370W_A:RPP1:C1_03830C_A:C1_04120C_A:RRP6:TRY2:C1_05360C_A:C1_06540C_A:C1_07960W_A:GCR3:C1_08630W_A:C1_08890C_A:CEF1:C1_09790C_A:BUD31:C1_10080W_A:C1_10620W_A:C1_11900C_A:C1_11910W_A:C1_12350W_A:C1_12630C_A:C1_12670C_A:C1_12750C_A:RMP1:C1_13380W_A:C2_00200W_A:C2_00280C_A:C2_01820C_A:PRP39:PRP3:SNU114:C2_06650C_A:C2_06660W_A:CWC22:PUS7:C2_10810W_A:C3_00100W_A:C3_00660W_A:C3_01560W_A:C3_02840W_A:C3_03330C_A:C3_04380C_A:PRP5:C4_00740W_A:RAT1:C4_01280C_A:C4_03740W_A:C4_05360C_A:OFD1:C4_06410W_A:C5_00920W_A:PUS4:C5_01610W_A:C5_01930W_A:C5_03010W_A:C5_03400C_A:C6_00530C_A:C6_02350C_A:PRP45:C6_02690C_A:MRT4:POP4:C7_03400C_A:ISY1:CR_01700C_A:CR_02550C_A:CR_03200C_A:CR_03230W_A:PRP42:CR_04240C_A:POP3
16078	tRNA catabolic process	13 out of 1061 genes, 1.2%	15 out of 6473 background genes, 0.2%	7.34E-06	0.00%	C1_01160C_A:C1_03830C_A:RRP6:C1_05360C_A:C1_06540C_A:C1_09790C_A:C1_12350W_A:C2_06660W_A:C2_09160W_A:C4_03740W_A:RRP42:C7_03400C_A:CR_02550C_A

398	mRNA splicing, via spliceosome	41 out of 1061 genes, 3.9%	101 out of 6473 background genes, 1.6%	7.59E-06	0.00%	C1_02090C_A:C1_02900C_A:C1_04120C_A:TRY2:GCR3:C1_08890C_A:CEF1:BUD31:C1_10080W_A:C1_12630C_A:C1_12670C_A:C1_12750C_A:C1_13380W_A:C2_00200W_A:C2_00280C_A:C2_01820C_A:PRP39:PRP3:SNU114:C2_06650C_A:CWC22:C2_10810W_A:C3_00100W_A:C3_00660W_A:C3_01560W_A:C3_03330C_A:C3_04380C_A:PRP5:C4_01280C_A:C4_06410W_A:C5_00920W_A:C5_01930W_A:C5_03010W_A:C5_03400C_A:C6_00530C_A:PRP45:C6_02690C_A:ISY1:CR_01700C_A:CR_03230W_A:PRP42
71027	nuclear RNA surveillance	16 out of 1061 genes, 1.5%	22 out of 6473 background genes, 0.3%	1.14E-05	0.00%	C1_01160C_A:C1_03830C_A:RRP6:C1_05360C_A:C1_06540C_A:C1_07960W_A:C1_09790C_A:C1_12350W_A:C2_06660W_A:C2_09160W_A:RAT1:C4_03740W_A:RRP42:C5_04910W_A:C7_03400C_A:CR_02550C_A
31125	rRNA 3'-end processing	15 out of 1061 genes, 1.4%	20 out of 6473 background genes, 0.3%	1.73E-05	0.00%	C1_01160C_A:C1_03830C_A:RRP6:C1_06540C_A:C1_07960W_A:C1_12350W_A:REX3:REX2:C2_06660W_A:C2_09160W_A:C4_03740W_A:RRP42:C7_03400C_A:CR_02550C_A:TSR1

71025	RNA surveillance	16 out of 1061 genes, 1.5%	23 out of 6473 background genes, 0.4%	3.18E-05	0.00%	C1_01160C_A:C1_03830C_A:RRP6:C1_05360C_A:C1_06540C_A:C1_07960W_A:C1_09790C_A:C1_12350W_A:C2_06660W_A:C2_09160W_A:RAT1:C4_03740W_A:RRP42:C5_04910W_A:C7_03400C_A:CR_02550C_A
6555	methionine metabolic process	17 out of 1061 genes, 1.6%	26 out of 6473 background genes, 0.4%	4.70E-05	0.00%	CYS4:HOM6:SAM4:SAM2:C1_11610C_A:C2_00390C_A:MXR1:C2_01860C_A:MET1:MET13:MET15:HOM3:MET16:C5_02820C_A:MET6:MET2:CYS3
9086	methionine biosynthetic process	16 out of 1061 genes, 1.5%	24 out of 6473 background genes, 0.4%	8.11E-05	0.00%	CYS4:HOM6:SAM4:C1_11610C_A:C2_00390C_A:MXR1:C2_01860C_A:MET1:MET13:MET15:HOM3:MET16:C5_02820C_A:MET6:MET2:CYS3

19752	carboxylic acid metabolic process	95 out of 1061 genes, 9.0%	349 out of 6473 background genes, 5.4%	0.00016	0.00%	RIM8:LEU4:C1_00510W_A:SPE1:HMT1:C1_01530C_A:CYS4:SNZ1:HOM6:LYS2:C1_02970W_A:ARG2:ARO4:C1_05990C_A:CAT8:SAM4:SAM2:C1_11610C_A:SER33:TRP3:WRS1:FEN1:C1_13330C_A:MET3:C2_00390C_A:MXR1:C2_01860C_A:ARO3:PRS1:SER2:MET1:BNA31:UGA1:BAT21:C2_04340C_A:IDP1:C2_05980C_A:ECM17:ILV1:SER1:VAS1:SPE3:DQD1:C2_07410W_A:C2_08530C_A:ASN1:C2_09310C_A:LEU42:ILV2:PHA2:MET13:C3_03470W_A:BAT22:HIS4:MET15:HIS5:ARO1:AAT22:ILV6:HOM3:TRP5:MET16:TRP4:MET14:IFG3:SPE2:THR4:MSW1:C5_02820C_A:SAH1:MSM1:HIS1:ILV3:NAM2:PAD1:ILS1:ARG4:HIS7:ADE1:PRS:TRP2:MET6:CDC60:MET2:ERG25:PAN6:ARC1:CR_03110W_A:CR_03400W_A:MIS11:URA2:ARO2:CYS3:CR_09010C_A:PRO1
46112	nucleobase biosynthetic process	15 out of 1061 genes, 1.4%	23 out of 6473 background genes, 0.4%	0.00033	0.00%	ADE4:ADE5,7:APT1:PRS1:SER1:AAH1:URA3:ADE2:BAS1:URA7:GRF10:ADE1:MET6:MIS11:URA2

16073	snRNA metabolic process	16 out of 1061 genes, 1.5%	26 out of 6473 background genes, 0.4%	0.00042	0.00%	RRP6:C1_05360C_A:C1_07960W_A:C1_09790C_A:C1_10620W_A:GAR1:REX3:REX2:C2_09160W_A:PUS7:C4_03740W_A:C6_02350C_A:C7_03400C_A:CR_02550C_A:CR_04110W_A:NOP10
31503	protein-containing complex localization	35 out of 1061 genes, 3.3%	92 out of 6473 background genes, 1.4%	0.00068	0.00%	RRS1:C1_02450C_A:ARX1:C1_03630W_A:C1_04040C_A:C1_06530C_A:C1_09840C_A:C1_12760W_A:C2_01220W_A:C2_05750W_A:C2_06850W_A:KRE30:MFG1:C3_02040C_A:SFP1:NOG1:C3_06150W_A:ZUO1:ECM1:C4_06210C_A:BUD23:RIX7:CDC14:C6_02230W_A:RPL10A:MRT4:NOG2:C7_04150W_A:YVH1:MEX67:SDA1:RFX1:NMD3:SSB1:LTV1

6397	mRNA processing	49 out of 1061 genes, 4.6%	149 out of 6473 background genes, 2.3%	0.00071	0.00%	C1_02090C_A:C1_02900C_A:C1_03370W_A:C1_03830C_A:C1_04120C_A:TRY2:C1_06540C_A:GCR3:C1_08630W_A:C1_08890C_A:CEF1:BUD31:C1_10080W_A:C1_11910W_A:C1_12350W_A:C1_12630C_A:C1_12670C_A:C1_12750C_A:C1_13380W_A:C2_00200W_A:C2_00280C_A:C2_01820C_A:PRP39:PRP3:SNU114:C2_06650C_A:CWC22:C2_10810W_A:C3_00100W_A:C3_00660W_A:C3_01560W_A:C3_03330C_A:C3_04380C_A:PRP5:C4_01280C_A:C4_05360C_A:C4_06410W_A:C5_00920W_A:C5_01930W_A:C5_03010W_A:C5_03400C_A:C6_00530C_A:PRP45:C6_02690C_A:C7_03400C_A:ISY1:CR_01700C_A:CR_03230W_A:PRP42
9067	aspartate family amino acid biosynthetic process	20 out of 1061 genes, 1.9%	39 out of 6473 background genes, 0.6%	0.00082	0.00%	CYS4:HOM6:LYS2:SAM4:C1_11610C_A:C2_00390C_A:MXR1:C2_01860C_A:MET1:C2_08530C_A:ASN1:MET13:MET15:HOM3:MET16:THR4:C5_02820C_A:MET6:MET2:CYS3
9113	purine nucleobase biosynthetic process	11 out of 1061 genes, 1.0%	14 out of 6473 background genes, 0.2%	0.00082	0.00%	ADE4:ADE5,7:APT1:SER1:AAH1:ADE2:BAS1:GRF10:ADE1:MET6:MIS11

43436	oxoacid metabolic process	95 out of 1061 genes, 9.0%	361 out of 6473 background genes, 5.6%	0.00091	0.00%	RIM8:LEU4:C1_00510W_A:SPE1:HMT1:C1_01530C_A:CYS4:SNZ1:HOM6:LYS2:C1_02970W_A:ARG2:ARO4:C1_05990C_A:CAT8:SAM4:SAM2:C1_11610C_A:SER33:TRP3:WRS1:FEN1:C1_13330C_A:MET3:C2_00390C_A:MXR1:C2_01860C_A:ARO3:PRS1:SER2:MET1:BNA31:UGA1:BAT21:C2_04340C_A:IDP1:C2_05980C_A:ECM17:ILV1:SER1:VAS1:SPE3:DQD1:C2_07410W_A:C2_08530C_A:ASN1:C2_09310C_A:LEU42:ILV2:PHA2:MET13:C3_03470W_A:BAT22:HIS4:MET15:HIS5:ARO1:AAT22:ILV6:HOM3:TRP5:MET16:TRP4:MET14:IFG3:SPE2:THR4:MSW1:C5_02820C_A:SAH1:MSM1:HIS1:ILV3:NAM2:PAD1:ILS1:ARG4:HIS7:ADE1:PRS:TRP2:MET6:CDC60:MET2:ERG25:PAN6:ARC1:CR_03110W_A:CR_03400W_A:MIS11:URA2:ARO2:CYS3:CR_09010C_A:PRO1

6082	organic acid metabolic process	95 out of 1061 genes, 9.0%	362 out of 6473 background genes, 5.6%	0.00104	0.00%	RIM8:LEU4:C1_00510W_A:SPE1:HMT1:C1_01530C_A:CYS4:SNZ1:HOM6:LYS2:C1_02970W_A:ARG2:ARO4:C1_05990C_A:CAT8:SAM4:SAM2:C1_11610C_A:SER33:TRP3:WRS1:FEN1:C1_13330C_A:MET3:C2_00390C_A:MXR1:C2_01860C_A:ARO3:PRS1:SER2:MET1:BNA31:UGA1:BAT21:C2_04340C_A:IDP1:C2_05980C_A:ECM17:ILV1:SER1:VAS1:SPE3:DQD1:C2_07410W_A:C2_08530C_A:ASN1:C2_09310C_A:LEU42:ILV2:PHA2:MET13:C3_03470W_A:BAT22:HIS4:MET15:HIS5:ARO1:AAT22:ILV6:HOM3:TRP5:MET16:TRP4:MET14:IFG3:SPE2:THR4:MSW1:C5_02820C_A:SAH1:MSM1:HIS1:ILV3:NAM2:PAD1:ILS1:ARG4:HIS7:ADE1:PRS:TRP2:MET6:CDC60:MET2:ERG25:PAN6:ARC1:CR_03110W_A:CR_03400W_A:MIS11:URA2:ARO2:CYS3:CR_09010C_A:PRO1
9066	aspartate family amino acid metabolic process	22 out of 1061 genes, 2.1%	46 out of 6473 background genes, 0.7%	0.00105	0.00%	CYS4:HOM6:LYS2:SAM4:SAM2:C1_11610C_A:C2_00390C_A:MXR1:C2_01860C_A:MET1:ILV1:C2_08530C_A:ASN1:MET13:MET15:HOM3:MET16:THR4:C5_02820C_A:MET6:MET2:CYS3

31167	rRNA methylation	10 out of 1061 genes, 0.9%	12 out of 6473 background genes, 0.2%	0.00108	0.00%	NEP1:RRP8:C3_05860C_A:NOP1:BUD23:SPB1:CR_01780W_A:CR_02030C_A:CR_04170W_A:CR_10260W_A
42401	biogenic amine biosynthetic process	9 out of 1061 genes, 0.8%	10 out of 6473 background genes, 0.2%	0.00118	0.00%	SPE1:TRP3:C1_13330C_A:PRS1:SPE3:TRP5:TRP4:SPE2:TRP2
46083	adenine metabolic process	9 out of 1061 genes, 0.8%	10 out of 6473 background genes, 0.2%	0.00118	0.00%	ADE4:ADE5,7:APT1:AAH1:ADE2:BAS1:GRF10:ADE1:MET6
9309	amine biosynthetic process	9 out of 1061 genes, 0.8%	10 out of 6473 background genes, 0.2%	0.00118	0.00%	SPE1:TRP3:C1_13330C_A:PRS1:SPE3:TRP5:TRP4:SPE2:TRP2
16074	sno(s)RNA metabolic process	19 out of 1061 genes, 1.8%	37 out of 6473 background genes, 0.6%	0.00155	0.00%	RPP1:RRP6:C1_05360C_A:C1_07960W_A:C1_09790C_A:C1_10620W_A:C2_00280C_A:TBF1:C3_02840W_A:C4_00740W_A:RAT1:C4_03740W_A:C4_04520W_A:NOP1:POP4:C7_03400C_A:CR_02550C_A:CR_03200C_A:POP3

98781	ncRNA transcription	25 out of 1061 genes, 2.4%	58 out of 6473 background genes, 0.9%	0.00213	0.00%	RPA190:C1_10920W_A:C1_12820C_A:C2_01070W_A:C2_02630W_A:TBF1:PZF1:RPC40:FHL1:RPC11:RPC53:C4_07060W_A:RPO26:TOP1:C6_03210C_A:RPA135:C7_01210C_A:C7_01400C_A:CR_01950W_A:RPC31:CR_02890C_A:RPB8:CR_05550C_A:RRN11:PIF1
55	ribosomal large subunit export from nucleus	24 out of 1061 genes, 2.3%	55 out of 6473 background genes, 0.8%	0.00265	0.00%	RRS1:C1_02450C_A:ARX1:C1_03630W_A:C1_06530C_A:C1_12760W_A:C2_01220W_A:C2_05750W_A:MFG1:C3_02040C_A:SFP1:C3_06150W_A:ECM1:C4_06210C_A:RIX7:RPL10A:MRT4:NOG2:C7_04150W_A:YVH1:MEX67:SDA1:RFX1:NMD3
43144	sno(s)RNA processing	17 out of 1061 genes, 1.6%	32 out of 6473 background genes, 0.5%	0.00317	0.00%	RPP1:RRP6:C1_05360C_A:C1_07960W_A:C1_10620W_A:C2_00280C_A:C3_02840W_A:C4_00740W_A:RAT1:C4_03740W_A:C4_04520W_A:NOP1:POP4:C7_03400C_A:CR_02550C_A:CR_03200C_A:POP3
42797	tRNA transcription by RNA polymerase III	12 out of 1061 genes, 1.1%	18 out of 6473 background genes, 0.3%	0.00419	0.00%	C1_12820C_A:RPC40:RPC11:RPC53:C4_07060W_A:RPO26:C6_03210C_A:C7_01210C_A:RPC31:CR_02890C_A:RPB8:CR_05550C_A

9304	tRNA transcription	12 out of 1061 genes, 1.1%	18 out of 6473 background genes, 0.3%	0.00419	0.00%	C1_12820C_A:RPC40:RPC11:RPC53:C4_07060W_A:RPO26:C6_03210C_A:C7_01210C_A:RPC31:CR_02890C_A:RPB8:CR_05550C_A
18202	peptidyl-histidine modification	9 out of 1061 genes, 0.8%	11 out of 6473 background genes, 0.2%	0.00556	0.00%	C1_09040C_A:C2_01060C_A:C2_03830W_A:KTI11:C2_05840W_A:C2_07920W_A:C4_00690C_A:C5_03640W_A:C5_03700C_A
16075	rRNA catabolic process	15 out of 1061 genes, 1.4%	27 out of 6473 background genes, 0.4%	0.00618	0.02%	C1_01160C_A:C1_03830C_A:RRP6:C1_05360C_A:C1_06540C_A:C1_07960W_A:C1_09790C_A:C1_12350W_A:C2_06660W_A:C2_09160W_A:RAT1:C4_03740W_A:RRP42:C7_03400C_A:CR_02550C_A
17183	protein histidyl modification to diphthamide	8 out of 1061 genes, 0.8%	9 out of 6473 background genes, 0.1%	0.00655	0.02%	C2_01060C_A:C2_03830W_A:KTI11:C2_05840W_A:C2_07920W_A:C4_00690C_A:C5_03640W_A:C5_03700C_A
9112	nucleobase metabolic process	16 out of 1061 genes, 1.5%	31 out of 6473 background genes, 0.5%	0.0105	0.02%	ADE4:ADE5,7:APT1:PRS1:SER1:AAH1:URA3:ADE2:BAS1:URA7:GRF10:FCA1:ADE1:MET6:MIS11:URA2

51168	nuclear export	46 out of 1061 genes, 4.3%	150 out of 6473 background genes, 2.3%	0.01375	0.02%	RRS1:HMT1:C1_02450C_A:ARX1:C1_03630W_A:C1_04040C_A:C1_04120C_A:C1_06530C_A:C1_07690C_A:C1_11910W_A:C1_12760W_A:C2_01220W_A:UTP22:C2_05750W_A:C2_06850W_A:KRE30:RPS10:MFG1:UTP8:C3_01520C_A:C3_02040C_A:RPS15:SFP1:NOG1:C3_06150W_A:ZUO1:NUP84:ECM1:C4_06210C_A:BUD23:RIX7:C5_03550W_A:C6_02230W_A:RPL10A:MRT4:NOG2:ENP1:C7_04150W_A:YVH1:MEX67:SDA1:RFX1:NMD3:SSB1:ELF1:LTV1
6401	RNA catabolic process	36 out of 1061 genes, 3.4%	108 out of 6473 background genes, 1.7%	0.01722	0.02%	C1_01160C_A:RPP1:C1_03830C_A:RRP6:C1_05360C_A:C1_06540C_A:C1_07960W_A:GCR3:C1_09790C_A:PET127:C1_11900C_A:C1_12350W_A:RMP1:SUV3:C2_06660W_A:MSU1:C2_09160W_A:C2_09180W_A:C3_02840W_A:C4_00740W_A:RAT1:C4_03740W_A:RRP42:OFD1:C5_00280C_A:C5_03400C_A:HYS2:C5_04910W_A:MRT4:POP4:C7_03400C_A:CR_02550C_A:CR_03200C_A:CR_04560C_A:PIF1:POP3

34655	nucleobase-containing compound catabolic process	40 out of 1061 genes, 3.8%	125 out of 6473 background genes, 1.9%	0.0173	0.02%	C1_01160C_A:RPP1:C1_03830C_A:RRP6:C1_05360C_A:C1_06540C_A:C1_07960W_A:GCR3:C1_09790C_A:PET127:C1_11900C_A:C1_12350W_A:RMP1:SUV3:C2_06660W_A:MSU1:C2_09160W_A:C2_09180W_A:C3_02840W_A:C4_00740W_A:RAT1:C4_03740W_A:RRP42:OFD1:C5_00280C_A:PDE1:C5_03400C_A:HAM1:SAH1:HYS2:C5_04910W_A:MRT4:POP4:C7_03400C_A:CR_01550C_A:CR_02550C_A:CR_03200C_A:CR_04560C_A:PIF1:POP3
1522	pseudouridine synthesis	9 out of 1061 genes, 0.8%	12 out of 6473 background genes, 0.2%	0.019	0.03%	C1_10620W_A:GAR1:PUS7:C4_06950W_A:PUS4:C5_01610W_A:C6_02350C_A:CR_04110W_A:NOP10
9092	homoserine metabolic process	9 out of 1061 genes, 0.8%	12 out of 6473 background genes, 0.2%	0.019	0.03%	CYS4:HOM6:C2_00390C_A:MET15:HOM3:MET6:MET2:CYS3:CR_09010C_A
33967	box C/D RNA metabolic process	8 out of 1061 genes, 0.8%	10 out of 6473 background genes, 0.2%	0.02805	0.03%	RPP1:C3_02840W_A:C4_00740W_A:C4_04520W_A:NOP1:POP4:CR_03200C_A:POP3

34963	box C/D RNA processing	8 out of 1061 genes, 0.8%	10 out of 6473 background genes, 0.2%	0.02805	0.03%	RPP1:C3_02840W_A:C4_00740W_A:C4_04520W_A:NOP1:POP4:CR_03200C_A:POP3
34661	ncRNA catabolic process	15 out of 1061 genes, 1.4%	30 out of 6473 background genes, 0.5%	0.03381	0.03%	C1_01160C_A:C1_03830C_A:RRP6:C1_05360C_A:C1_06540C_A:C1_07960W_A:C1_09790C_A:C1_12350W_A:C2_06660W_A:C2_09160W_A:RAT1:C4_03740W_A:RRP42:C7_03400C_A:CR_02550C_A
294	nuclear-transcribed mRNA catabolic process, RNase MRP-dependent	7 out of 1061 genes, 0.7%	8 out of 6473 background genes, 0.1%	0.03585	0.03%	RPP1:RMP1:C3_02840W_A:C4_00740W_A:POP4:CR_03200C_A:POP3
46084	adenine biosynthetic process	7 out of 1061 genes, 0.7%	8 out of 6473 background genes, 0.1%	0.03585	0.03%	ADE5,7:APT1:ADE2:BAS1:GRF10:ADE1:MET6
9069	serine family amino acid metabolic process	16 out of 1061 genes, 1.5%	34 out of 6473 background genes, 0.5%	0.04716	0.05%	CYS4:SNZ1:HOM6:C1_11610C_A:SER33:C2_00390C_A:SER2:SER1:MET15:HOM3:MET16:MET6:MET2:MIS11:CYS3:CR_09010C_A

71265	L-methionine biosynthetic process	9 out of 1061 genes, 0.8%	13 out of 6473 background genes, 0.2%	0.05275	0.05%	CYS4:SAM4:C2_00390C_A:MXR1:C2_01860C_A:MET15:C5_02820C_A:MET6:MET2
963	mitochondrial RNA processing	9 out of 1061 genes, 0.8%	13 out of 6473 background genes, 0.2%	0.05275	0.05%	MSS116:PET127:C1_14330W_A:C2_00820W_A:SUV3:C4_00810C_A:C4_03730C_A:C4_05360C_A:C6_03440W_A
44272	sulfur compound biosynthetic process	23 out of 1061 genes, 2.2%	60 out of 6473 background genes, 0.9%	0.05774	0.05%	RIM8:CYS4:SNZ1:HOM6:C1_02970W_A:SAM4:SAM2:C1_11610C_A:C2_00390C_A:MXR1:C2_01860C_A:MET1:ECM17:MET13:MET15:HOM3:MET16:C5_02820C_A:C5_03840W_A:C7_03590C_A:MET6:MET2:CYS3

51169	nuclear transport	54 out of 1061 genes, 5.1%	195 out of 6473 background genes, 3.0%	0.06191	0.05%	RRS1:HMT1:C1_02450C_A:ARX1:C1_03630W_A:C1_04040C_A:C1_04120C_A:REI1:C1_05630C_A:C1_06530C_A:C1_07690C_A:C1_11910W_A:C1_12760W_A:C2_01220W_A:UTP22:C2_05270W_A:C2_05750W_A:C2_06850W_A:KRE30:RPS10:MFG1:UTP8:C3_01520C_A:C3_02040C_A:RPS15:SFP1:NOG1:C3_06150W_A:NMD5:ZUO1:NUP84:ECM1:C4_06210C_A:C4_06790W_A:BUD23:RIX7:C5_03550W_A:C6_02230W_A:RPL10A:MRT4:NOG2:C7_02460C_A:ENP1:C7_04150W_A:YVH1:MEX67:SDA1:RFX1:NMD3:SSB1:CR_08330W_A:CR_08500W_A:ELF1:LTV1

6913	nucleocytoplasmic transport	54 out of 1061 genes, 5.1%	195 out of 6473 background genes, 3.0%	0.06191	0.05%	RRS1:HMT1:C1_02450C_A:ARX1:C1_03630W_A:C1_04040C_A:C1_04120C_A:REI1:C1_05630C_A:C1_06530C_A:C1_07690C_A:C1_11910W_A:C1_12760W_A:C2_01220W_A:UTP22:C2_05270W_A:C2_05750W_A:C2_06850W_A:KRE30:RPS10:MFG1:UTP8:C3_01520C_A:C3_02040C_A:RPS15:SFP1:NOG1:C3_06150W_A:NMD5:ZUO1:NUP84:ECM1:C4_06210C_A:C4_06790W_A:BUD23:RIX7:C5_03550W_A:C6_02230W_A:RPL10A:MRT4:NOG2:C7_02460C_A:ENP1:C7_04150W_A:YVH1:MEX67:SDA1:RFX1:NMD3:SSB1:CR_08330W_A:CR_08500W_A:ELF1:LTV1
959	mitochondrial RNA metabolic process	13 out of 1061 genes, 1.2%	25 out of 6473 background genes, 0.4%	0.07173	0.06%	RPO41:MSS116:PET127:C1_14330W_A:C2_00820W_A:C2_02120W_A:SUV3:MSU1:C4_00810C_A:C4_03730C_A:C4_05360C_A:MSW1:C6_03440W_A
9070	serine family amino acid biosynthetic process	11 out of 1061 genes, 1.0%	19 out of 6473 background genes, 0.3%	0.07683	0.06%	CYS4:SNZ1:HOM6:C1_11610C_A:SER33:SER2:SER1:MET15:HOM3:MET16:CYS3

34475	U4 snRNA 3'-end processing	8 out of 1061 genes, 0.8%	11 out of 6473 background genes, 0.2%	0.08803	0.06%	RRP6:C1_05360C_A:C1_07960W_A:REX2:C2_09160W_A:C4_03740W_A:C7_03400C_A:CR_02550C_A
6534	cysteine metabolic process	8 out of 1061 genes, 0.8%	11 out of 6473 background genes, 0.2%	0.08803	0.06%	CYS4:SNZ1:C1_11610C_A:C2_00390C_A:MET15:MET16:CYS3:CR_09010C_A

2E+06	organonitrogen compound biosynthetic process	253 out of 1061 genes, 23.8%	1258 out of 6473 background genes, 19.4%	0.09736	0.06%	RIM8:LEU4:C1_00510W_A:SPE1:NRM1:HMT1:C1_01150C_A:C1_01530C_A:CYS4:CWH8:SNZ1:SNO1:HOM6:LYS2:MIA40:C1_02970W_A:C1_03540C_A:KTR4:MRPL37:RHD1:ARG2:ARO4:C1_05220C_A:C1_05270C_A:RSM22:RPS27A:C1_05630C_A:C1_05990C_A:BUD16:YMC1:C1_07340W_A:C1_07470C_A:ADE4:GCS1:ADE5,7:MRPL3:ERD1:FUN12:DRG1:SAM4:C1_08520C_A:GCD6:C1_08630W_A:C1_08890C_A:GUA1:C1_09910C_A:MNN23:C1_10080W_A:C1_10200C_A:C1_10340W_A:C1_10690W_A:FGR39:SAM2:C1_11610C_A:C1_11790W_A:MUP1:C1_11880W_A:C1_11910W_A:SER33:TRP3:MRPS9:WRS1:FEN1:RPL14:C1_13060C_A:C1_13330C_A:C1_14330W_A:C2_00390C_A:MXR1:APT1:C2_01740C_A:C2_01860C_A:ARO3:C2_02270C_A:PRS1:SER2:C2_02930C_A:MET1:BNA31:SMP3:C2_03560C_A:GPI13:C2_03950W_A:BAT21:RIM2:C2_04700C_A:C2_04820W_A:C2_05050C_A:CDC21:C2_05270W_A:IDP1:ECM17:ILV1:SER1:VAS1:RPL11:C2_06950C_A:SPE3:AAH1:C2_07680W_A:C2_08530C_A:ASN1:TAZ1:LEU42:HEM3:RPS24:URA3:RKI1:RPS7A:C3_01520C_A:ILV2:PHA2:MET13:YAH1:C3_04380C_A:ADE2:RPS15:RPP2B:ARV1:C3_05280C_A:C3_05380W_A:GPI1:C3_05440C_A:TRM12:C3_06240C_A:OPI3:C3_06830C_A:RPS12:C3_07400W_A:HIS4:MET15:HIS5:C4_00810C_A:C4_00880W_A:ARO1:PTC8:PGA53:ILV6:NIP1:HSX11:TIM10:ZUO1:C4_03410W_A:C4_04130W_A:DUO1:C4_04390W_A:SSZ1:BAS1:RPL30:C4_05360C_A:OFD1:HOM3:TRP5:MET16:TRP4:C4_07140W_A:C5_00030W_A:C5_00280C_A:C5_00320W_A:MRP17:MET14:SPE2:C5_00820W_A:C5_01140C_A:CCN1:C5_01700W_A:C5_02010C_A:THR4:TIF5:C5_02590C_A:MSW1:C5_02780W_A:C5_02820C_A:CAM1-1:C5_03290C_A:PCL1:FUR1:C5_03460C_A:C5_03530C_A:C5_03840W_A:C5_03970W_A:SAH1:C5_04290C_A:URA7:CSU57:MSM1:C5_04720C_A:GRF10:C5_05250C_A:SDH4:HIS1:ILV3:C6_00530C_A:C6_00560W_A:C6_01120C_A:CIC1:PMP1:TIF3:C6_01980C_A:RPL10A:ALG11:C6_02280W_A:C6_02290C_A:C6_02370C_A:NAM2:ILS1:YML6:C7_01020C_A:ASC1:GIR2:PRT1:C7_01570C_A:C7_01600W_A:C7_01950W_A:IFM1:ARG4:HIS7:MRP7:MIS12:CR_00430C_A:MRPL6:ADE1:CR_01260W_A:CR_01320C_A:PRS:TRP2:MET6:CDC60:MCM6:MET2:ERG25:CR_02430C_A:CHO2:PAN6:ARC1:CR_03110W_A:MTG1:CR_03400W_A:CR_04160C_A:ADE6:CR_04920W_A:MTG2:MIS11:URA2:CR_07310W_A:ARO2:CDC45:NOP10:SSB1:CYS3:RPP2A:GSH2:PRO1
**S45 vs B45 Upregulated genes**						
GOID	GO_term	Cluster frequency	Background frequency	Corrected P-value	False discovery rate	Gene(s) annotated to the term

34470	ncRNA processing	306 out of 804 genes, 38.1%	860 out of 6473 background genes, 13.3%	2.18E-80	0.00%	HBR1:CNS1:RRS1:C1_01150C_A:C1_01160C_A:C1_01470W_A:C1_01900C_A:C1_02090C_A:ABP140:C1_02450C_A:PDE2:TSR2:ARO80:C1_03830C_A:C1_04040C_A:C1_04120C_A:ERB1:NOP4:RRP6:C1_05220C_A:C1_05230W_A:C1_05360C_A:RPS27A:FAL1:C1_05650W_A:C1_06540C_A:NOP6:C1_06760C_A:C1_06770W_A:C1_06800W_A:C1_07090C_A:RIA1:YPD1:PEX3:LHP1:C1_07510W_A:C1_07570C_A:C1_07950C_A:FUN12:GCD6:C1_08630W_A:MSS116:C1_09390W_A:C1_09710C_A:DBP3:TPK1:C1_10620W_A:C1_10970W_A:C1_11000C_A:KRR1:C1_11560C_A:C1_11900C_A:C1_11910W_A:CSI2:C1_12350W_A:C1_12440W_A:C1_12570C_A:CHR1:C1_12760W_A:C1_14080W_A:MPP10:FAV3:NDT80:HMA1:C2_00280C_A:C2_00410C_A:C2_01070W_A:C2_01510C_A:C2_02420C_A:PRS1:C2_02540W_A:C2_02710C_A:PRP3:SCH9:C2_04120C_A:C2_04570W_A:SNU114:C2_04820W_A:C2_05080C_A:MAK5:C2_05160C_A:RPF2:C2_05520W_A:KTI11:TES1:LAS1:CNT:ECM17:RTA2:C2_06480W_A:C2_06850W_A:NOC4:C2_07360W_A:RCL1:PRS5:C2_07920W_A:NSA1:RRP8:C2_08840W_A:PES1:RRP15:C2_09500W_A:C2_09660W_A:CWC22:OCA1:PUS7:C2_10740C_A:C2_10810W_A:C3_00100W_A:UTP8:BUD21:SOF1:C3_00950C_A:BMS1:C3_01560W_A:C3_02020W_A:C3_02040C_A:UTP4:C3_02350W_A:C3_03070W_A:C3_03330C_A:HBR3:URK1:C3_04370C_A:C3_04380C_A:MAK21:SFP1:CEM1:C3_05140C_A:C3_05160C_A:C3_05380W_A:QDR2:C3_05800W_A:C3_05860C_A:NOG1:ELP3:DED1:C3_06150W_A:C3_06370C_A:NSA2:PRP5:C3_06760W_A:NOP13:C3_07400W_A:C3_07480W_A:C3_07550C_A:RIT1:UTP9:DUR4:C4_00490W_A:RLP24:NMD5:C4_00690C_A:C4_00810C_A:C4_00940W_A:MDN1:RAT1:C4_01280C_A:PWP1:C4_01670C_A:C4_02850W_A:ZUO1:C4_02880C_A:C4_03040W_A:C4_03150W_A:C4_03720C_A:C4_03730C_A:C4_03740W_A:C4_03830W_A:RRP42:RAM1:ECM1:C4_04510W_A:SSZ1:C4_04810C_A:AGP3:NBP35:C4_05260W_A:C4_05650W_A:C4_06210C_A:C4_06410W_A:NOP1:C4_06790W_A:C4_07060W_A:C4_07100C_A:DUS4:RMT2:C5_00920W_A:C5_00950C_A:BUD23:PUS4:C5_01430C_A:FYV5:SPB4:C5_01610W_A:C5_01930W_A:RIB2:C5_02010C_A:C5_02070C_A:RIX7:UTP13:C5_02730C_A:C5_03010W_A:RMS1:C5_03670C_A:C5_03920C_A:C5_04610W_A:HAS1:C5_04990W_A:C5_05340W_A:NOP5:C6_01040C_A:RCN1:CIC1:C6_01890C_A:C6_02230W_A:C6_02350C_A:NIP7:MRT4:POP4:C6_03210C_A:C6_03390W_A:ALS5:SNQ2:SPB1:C6_04530C_A:C7_00160C_A:C7_00330C_A:NOP15:C7_01030C_A:DBP7:C7_01360C_A:FLU1:C7_02100W_A:C7_02340C_A:C7_02460C_A:C7_02930C_A:ISY1:ENP2:C7_03990C_A:C7_04140C_A:UTP18:CR_00460C_A:CR_00670C_A:BUD22:PWP2:TRM1:CR_01410C_A:CR_01700C_A:CR_01780W_A:CR_02030C_A:CR_02420W_A:RPC19:DBP2:CR_02890C_A:SRP40:CR_03230W_A:CR_03360W_A:KTI12:NCS2:YVH1:IFF3:PRP42:CR_03940W_A:CR_04110W_A:CR_04160C_A:CR_04170W_A:CR_04240C_A:CR_04300W_A:NHP2:NOC2:CR_05550C_A:RNR3:MTG2:DBP8:VPS75:SFL1:RPL7:FGR50:CR_06680C_A:NMD3:CR_06840W_A:CR_07030C_A:CR_07080W_A:CR_07640C_A:DBP6:IMP4:CR_08000C_A:N

						OP10:SSB1:RIO2:TRM9:ATS1:CR_08330W_A:TSR1:PRP8:CR_08940W_A:CR_09110C_A:SSF1:CR_09800C_A:SIK1:UTP5:CR_10410C_A:CR_10470C_A:DRS1:POP3

34660	ncRNA metabolic process	319 out of 804 genes, 39.7%	935 out of 6473 background genes, 14.4%	4.19E-79	0.00%	HBR1:CNS1:RRS1:C1_01150C_A:C1_01160C_A:C1_01470W_A:C1_01530C_A:C1_01900C_A:C1_02090C_A:ABP140:C1_02450C_A:PDE2:TSR2:ARO80:C1_03830C_A:C1_04040C_A:C1_04120C_A:ERB1:NOP4:RRP6:C1_05220C_A:C1_05230W_A:C1_05360C_A:RPS27A:FAL1:C1_05650W_A:NPL4:C1_06540C_A:NOP6:C1_06760C_A:C1_06770W_A:C1_06800W_A:C1_07090C_A:RIA1:YPD1:PEX3:LHP1:C1_07510W_A:C1_07570C_A:C1_07950C_A:FUN12:GCD6:C1_08630W_A:MSS116:C1_09390W_A:C1_09710C_A:DBP3:TPK1:C1_10620W_A:C1_10970W_A:C1_11000C_A:KRR1:GAR1:C1_11560C_A:C1_11900C_A:C1_11910W_A:CSI2:C1_12350W_A:C1_12440W_A:C1_12570C_A:CHR1:C1_12760W_A:C1_14080W_A:MPP10:FAV3:NDT80:HMA1:C2_00280C_A:C2_00410C_A:C2_01070W_A:C2_01510C_A:C2_02420C_A:PRS1:C2_02540W_A:C2_02710C_A:PRP3:SCH9:C2_04120C_A:C2_04570W_A:SNU114:C2_04820W_A:C2_05080C_A:MAK5:C2_05160C_A:RPF2:RPC40:C2_05520W_A:KTI11:TES1:LAS1:CNT:ECM17:RTA2:C2_06480W_A:C2_06850W_A:RPA12:NOC4:C2_07360W_A:RCL1:PRS5:C2_07920W_A:NSA1:RRP8:RPT6:C2_08840W_A:PES1:RRP15:C2_09500W_A:C2_09660W_A:CWC22:OCA1:PUS7:C2_10740C_A:RPC11:C2_10810W_A:C3_00100W_A:UTP8:BUD21:SOF1:C3_00950C_A:BMS1:C3_01560W_A:C3_02020W_A:C3_02040C_A:UTP4:C3_02350W_A:C3_03070W_A:C3_03330C_A:HBR3:URK1:C3_04370C_A:C3_04380C_A:MAK21:SFP1:CEM1:C3_05140C_A:C3_05160C_A:C3_05380W_A:QDR2:C3_05800W_A:C3_05860C_A:NOG1:ELP3:DED1:C3_06150W_A:C3_06370C_A:NSA2:PRP5:C3_06760W_A:NOP13:C3_07400W_A:C3_07480W_A:C3_07550C_A:RIT1:UTP9:DUR4:C4_00490W_A:RLP24:NMD5:C4_00690C_A:C4_00810C_A:C4_00940W_A:MDN1:RAT1:C4_01280C_A:PWP1:C4_01670C_A:C4_02850W_A:ZUO1:C4_02880C_A:C4_03040W_A:C4_03150W_A:C4_03720C_A:C4_03730C_A:C4_03740W_A:C4_03830W_A:RRP42:RAM1:ECM1:C4_04510W_A:SSZ1:C4_04810C_A:AGP3:NBP35:C4_05260W_A:C4_05650W_A:C4_06210C_A:C4_06410W_A:NOP1:C4_06790W_A:C4_07060W_A:C4_07100C_A:DUS4:RMT2:C5_00920W_A:C5_00950C_A:BUD23:PUS4:C5_01430C_A:FYV5:SPB4:C5_01610W_A:C5_01930W_A:RIB2:C5_02010C_A:C5_02070C_A:RIX7:UTP13:C5_02730C_A:C5_03010W_A:RMS1:RPO26:C5_03670C_A:C5_03920C_A:C5_04610W_A:HAS1:C5_04990W_A:C5_05340W_A:NOP5:C6_01040C_A:RCN1:CIC1:C6_01890C_A:C6_02230W_A:C6_02350C_A:NIP7:MRT4:POP4:C6_03210C_A:C6_03390W_A:ALS5:SNQ2:SPB1:C6_04530C_A:C7_00160C_A:C7_00330C_A:RPA135:NOP15:C7_01030C_A:DBP7:C7_01360C_A:FLU1:C7_02100W_A:C7_02340C_A:C7_02460C_A:C7_02930C_A:ISY1:ENP2:C7_03990C_A:C7_04140C_A:UTP18:CR_00460C_A:CR_00670C_A:BUD22:PWP2:TRM1:CR_01410C_A:CR_01700C_A:CR_01780W_A:CR_01950W_A:CR_02030C_A:CR_02420W_A:RPC19:DBP2:RPC31:CR_02890C_A:SRP40:CR_03110W_A:CR_03230W_A:CR_03360W_A:KTI12:NCS2:YVH1:IFF3:PRP42:CR_03940W_A:CR_04110W_A:CR_04160C_A:CR_04170W_A:CR_04240C_A:CR_04300W_A:NHP2:RPB8:NOC2:CR_05550C_A:RNR3:MTG2:DBP8:VPS75:SFL1:RPL7:F

						GR50:CR_06680C_A:NMD3:CR_06840W_A:CR_07030C_A:CR_07080W_A:CR_07640C_A:DBP6:IMP4:CR_08000C_A:NOP10:SSB1:RIO2:TRM9:ATS1:CR_08330W_A:TSR1:PRP8:CR_08940W_A:CR_09110C_A:SSF1:CR_09800C_A:SIK1:UTP5:CR_10410C_A:CR_10470C_A:DRS1:POP3

6396	RNA processing	311 out of 804 genes, 38.7%	931 out of 6473 background genes, 14.4%	6.97E-74	0.00%	HBR1:CNS1:RRS1:C1_01150C_A:C1_01160C_A:C1_01470W_A:C1_01900C_A:C1_02090C_A:ABP140:C1_02450C_A:PDE2:TSR2:ARO80:C1_03370W_A:C1_03830C_A:C1_04040C_A:C1_04120C_A:ERB1:NOP4:C1_04990C_A:RRP6:C1_05220C_A:C1_05230W_A:C1_05360C_A:RPS27A:FAL1:C1_05650W_A:C1_06540C_A:NOP6:C1_06760C_A:C1_06770W_A:C1_06800W_A:C1_07090C_A:RIA1:YPD1:PEX3:LHP1:C1_07510W_A:C1_07570C_A:C1_07950C_A:FUN12:GCD6:C1_08630W_A:MSS116:C1_09390W_A:C1_09710C_A:DBP3:TPK1:C1_10620W_A:C1_10970W_A:C1_11000C_A:KRR1:C1_11560C_A:C1_11900C_A:C1_11910W_A:CSI2:C1_12350W_A:C1_12440W_A:C1_12570C_A:CHR1:C1_12670C_A:C1_12760W_A:C1_14080W_A:MPP10:FAV3:NDT80:HMA1:C2_00280C_A:C2_00410C_A:C2_01070W_A:C2_01510C_A:C2_02420C_A:PRS1:C2_02540W_A:C2_02710C_A:PRP3:SCH9:C2_04120C_A:C2_04570W_A:SNU114:C2_04820W_A:C2_05080C_A:MAK5:C2_05160C_A:RPF2:C2_05520W_A:KTI11:TES1:LAS1:CNT:ECM17:RTA2:C2_06480W_A:C2_06850W_A:NOC4:C2_07360W_A:RCL1:PRS5:C2_07920W_A:NSA1:RRP8:C2_08840W_A:PES1:RRP15:C2_09500W_A:C2_09660W_A:CWC22:OCA1:PUS7:C2_10740C_A:C2_10810W_A:C3_00100W_A:UTP8:BUD21:SOF1:C3_00950C_A:BMS1:C3_01560W_A:C3_02020W_A:C3_02040C_A:UTP4:C3_02350W_A:C3_03070W_A:C3_03330C_A:HBR3:URK1:C3_04370C_A:C3_04380C_A:MAK21:SFP1:CEM1:C3_05140C_A:C3_05160C_A:C3_05380W_A:QDR2:C3_05800W_A:C3_05860C_A:NOG1:ELP3:DED1:C3_06150W_A:C3_06370C_A:NSA2:PRP5:C3_06760W_A:NOP13:C3_07400W_A:C3_07480W_A:C3_07550C_A:RIT1:UTP9:DUR4:C4_00490W_A:RLP24:NMD5:C4_00690C_A:C4_00810C_A:C4_00940W_A:MDN1:RAT1:C4_01280C_A:PWP1:C4_01670C_A:C4_02850W_A:ZUO1:C4_02880C_A:C4_03040W_A:C4_03150W_A:C4_03720C_A:C4_03730C_A:C4_03740W_A:C4_03830W_A:RRP42:RAM1:ECM1:C4_04510W_A:SSZ1:C4_04810C_A:AGP3:NBP35:C4_05260W_A:C4_05650W_A:C4_06210C_A:C4_06410W_A:NOP1:C4_06790W_A:C4_07060W_A:C4_07100C_A:DUS4:RMT2:C5_00920W_A:C5_00950C_A:BUD23:PUS4:C5_01430C_A:FYV5:SPB4:C5_01610W_A:C5_01930W_A:RIB2:C5_02010C_A:C5_02070C_A:RIX7:UTP13:C5_02730C_A:C5_03010W_A:RMS1:C5_03670C_A:C5_03920C_A:C5_04610W_A:HAS1:C5_04990W_A:C5_05340W_A:NOP5:C6_00530C_A:C6_01040C_A:RCN1:CIC1:C6_01890C_A:C6_02230W_A:C6_02350C_A:NIP7:MRT4:POP4:C6_03210C_A:C6_03390W_A:ALS5:SNQ2:SPB1:C6_04530C_A:C7_00160C_A:C7_00330C_A:NOP15:C7_01030C_A:DBP7:C7_01360C_A:FLU1:C7_02100W_A:C7_02340C_A:C7_02460C_A:C7_02930C_A:ISY1:ENP2:C7_03990C_A:C7_04140C_A:UTP18:CR_00460C_A:CR_00670C_A:BUD22:PWP2:TRM1:CR_01410C_A:CR_01700C_A:CR_01780W_A:CR_02030C_A:CR_02420W_A:RPC19:DBP2:CR_02890C_A:SRP40:CR_03230W_A:CR_03360W_A:KTI12:NCS2:YVH1:IFF3:PRP42:CR_03940W_A:CR_04110W_A:CR_04160C_A:CR_04170W_A:CR_04240C_A:CR_04300W_A:NHP2:NOC2:CR_05550C_A:RNR3:MTG2:DBP8:VPS75:SFL1:RPL7:FGR50:CR_06680C_A:NMD3:CR_06840W_A:CR_07030C_A:CR

						_07080W_A:CR_07640C_A:DBP6:IMP4:CR_08000C_A:NOP10:SSB1:RIO2:TRM9:ATS1:CR_08330W_A:TSR1:PRP8:CR_08940W_A:CR_09110C_A:SSF1:CR_09800C_A:SIK1:UTP5:CR_10410C_A:CR_10430C_A:CR_10470C_A:DRS1:POP3

42254	ribosome biogenesis	297 out of 804 genes, 36.9%	886 out of 6473 background genes, 13.7%	4.23E-70	0.00%	HBR1:CNS1:RRS1:C1_01160C_A:C1_01470W_A:C1_01900C_A:C1_02090C_A:ABP140:C1_02450C_A:PDE2:TSR2:RPL6:ARO80:ARX1:C1_03830C_A:C1_04040C_A:C1_04120C_A:ERB1:NOP4:RRP6:C1_05220C_A:C1_05230W_A:C1_05360C_A:RPS27A:FAL1:C1_05650W_A:C1_06540C_A:NOP6:C1_06760C_A:C1_06770W_A:C1_06800W_A:C1_07090C_A:RIA1:YPD1:PEX3:C1_07510W_A:C1_07570C_A:C1_07950C_A:FUN12:GCD6:C1_08630W_A:MSS116:JIP5:C1_09390W_A:YTM1:C1_09710C_A:DBP3:TPK1:C1_10620W_A:C1_10970W_A:C1_11000C_A:KRR1:GAR1:C1_11560C_A:C1_11900C_A:C1_11910W_A:CSI2:C1_12350W_A:C1_12440W_A:C1_12570C_A:CHR1:C1_12680W_A:C1_12760W_A:C1_13370W_A:C1_14080W_A:MPP10:FAV3:NDT80:C2_00280C_A:C2_00410C_A:C2_01070W_A:C2_01510C_A:C2_02420C_A:PRS1:C2_02540W_A:C2_02710C_A:PRP3:SCH9:C2_04120C_A:C2_04570W_A:SNU114:C2_04820W_A:C2_05080C_A:MAK5:C2_05160C_A:RPF2:C2_05520W_A:TES1:LAS1:CNT:ECM17:RTA2:RPL11:C2_06850W_A:NOC4:C2_07360W_A:RCL1:PRS5:C2_07920W_A:NSA1:KRE30:RRP8:TIF4631:C2_08840W_A:PES1:RRP15:C2_09660W_A:CWC22:OCA1:PUS7:MTR2:C2_10740C_A:C2_10810W_A:C3_00100W_A:UTP8:BUD21:SOF1:C3_00950C_A:BMS1:C3_01560W_A:C3_02020W_A:C3_02040C_A:UTP4:C3_02350W_A:C3_03070W_A:C3_03330C_A:HBR3:URK1:C3_04370C_A:C3_04380C_A:MAK21:SFP1:CEM1:C3_05160C_A:C3_05380W_A:QDR2:C3_05800W_A:C3_05860C_A:NOG1:DED1:C3_06150W_A:C3_06370C_A:NSA2:PRP5:C3_06760W_A:NOP13:C3_07480W_A:C3_07550C_A:UTP9:DUR4:C4_00490W_A:RLP24:NMD5:C4_00690C_A:C4_00810C_A:MDN1:RAT1:C4_01280C_A:PWP1:C4_01670C_A:ZUO1:C4_02880C_A:C4_03040W_A:C4_03150W_A:C4_03720C_A:C4_03740W_A:C4_03830W_A:RRP42:RAM1:ECM1:C4_04510W_A:SSZ1:AGP3:C4_05010W_A:C4_05260W_A:C4_05650W_A:C4_06210C_A:C4_06410W_A:NOP1:C4_06790W_A:C4_07060W_A:C4_07100C_A:RMT2:C5_00920W_A:C5_00950C_A:BUD23:PUS4:C5_01430C_A:FYV5:SPB4:C5_01930W_A:RIB2:C5_02010C_A:C5_02070C_A:RIX7:TIF5:UTP13:C5_03010W_A:RMS1:C5_03670C_A:C5_03920C_A:C5_04610W_A:HAS1:C5_04990W_A:C5_05340W_A:NOP5:C6_01040C_A:RCN1:CIC1:C6_01890C_A:C6_02230W_A:NIP7:MRT4:POP4:C6_03210C_A:C6_03390W_A:ALS5:SNQ2:SPB1:C7_00160C_A:C7_00330C_A:RPA135:NOP15:C7_01030C_A:DBP7:C7_01360C_A:FLU1:C7_02100W_A:C7_02460C_A:C7_02930C_A:ISY1:ENP2:C7_04140C_A:UTP18:CR_00460C_A:BUD22:PWP2:CR_01410C_A:CR_01700C_A:CR_01780W_A:CR_02030C_A:CR_02420W_A:RPC19:DBP2:CR_02890C_A:SRP40:CR_03230W_A:CR_03360W_A:KTI12:NCS2:YVH1:IFF3:SGD1:PRP42:CR_03940W_A:MEX67:CR_04110W_A:CR_04170W_A:CR_04240C_A:NHP2:NOC2:CR_05550C_A:RNR3:MTG2:DBP8:SDA1:VPS75:SFL1:RPL7:FGR50:CR_06680C_A:NMD3:CR_06840W_A:CR_07030C_A:CR_07080W_A:CR_07640C_A:DBP6:IMP4:CR_08000C_A:NOP10:SSB1:RIO2:ATS1:CR_08330W_A:TSR1:CR_08500W_A:PRP8:CR_09110C_A:ELF1:SSF1:CR_09800C_A:SIK1:UTP5:CR_10410C_A:CR_10470C_A:DRS1:POP3

22613	ribonucleoprotein complex biogenesis	300 out of 804 genes, 37.3%	915 out of 6473 background genes, 14.1%	2.58E-68	0.00%	HBR1:CNS1:RRS1:C1_01160C_A:C1_01470W_A:C1_01900C_A:C1_02090C_A:ABP140:C1_02450C_A:PDE2:TSR2:RPL6:ARO80:ARX1:C1_03830C_A:C1_04040C_A:C1_04120C_A:ERB1:NOP4:RRP6:C1_05220C_A:C1_05230W_A:C1_05360C_A:RPS27A:FAL1:C1_05650W_A:C1_06540C_A:NOP6:C1_06760C_A:C1_06770W_A:C1_06800W_A:SUI2:C1_07090C_A:RIA1:YPD1:PEX3:C1_07510W_A:C1_07570C_A:C1_07950C_A:FUN12:GCD6:C1_08630W_A:MSS116:JIP5:C1_09390W_A:YTM1:C1_09710C_A:DBP3:TPK1:C1_10620W_A:C1_10970W_A:C1_11000C_A:KRR1:GAR1:C1_11560C_A:C1_11900C_A:C1_11910W_A:CSI2:C1_12350W_A:C1_12440W_A:C1_12570C_A:CHR1:C1_12680W_A:C1_12760W_A:C1_13370W_A:C1_14080W_A:MPP10:FAV3:NDT80:C2_00280C_A:C2_00410C_A:C2_01070W_A:C2_01510C_A:C2_02420C_A:PRS1:C2_02540W_A:C2_02710C_A:PRP3:SCH9:C2_04120C_A:C2_04570W_A:SNU114:C2_04820W_A:C2_05080C_A:MAK5:C2_05160C_A:RPF2:C2_05520W_A:TES1:LAS1:CNT:ECM17:RTA2:RPL11:C2_06850W_A:NOC4:C2_07360W_A:RCL1:PRS5:C2_07920W_A:NSA1:KRE30:RRP8:TIF4631:C2_08840W_A:PES1:RRP15:C2_09660W_A:CWC22:OCA1:PUS7:MTR2:TIF11:C2_10740C_A:C2_10810W_A:C3_00100W_A:UTP8:BUD21:SOF1:C3_00950C_A:BMS1:C3_01560W_A:C3_02020W_A:C3_02040C_A:UTP4:C3_02350W_A:C3_03070W_A:C3_03330C_A:HBR3:URK1:C3_04370C_A:C3_04380C_A:MAK21:SFP1:CEM1:C3_05160C_A:C3_05380W_A:QDR2:C3_05800W_A:C3_05860C_A:NOG1:DED1:C3_06150W_A:C3_06370C_A:NSA2:PRP5:C3_06760W_A:NOP13:C3_07480W_A:C3_07550C_A:UTP9:DUR4:C4_00490W_A:RLP24:NMD5:C4_00690C_A:C4_00810C_A:MDN1:RAT1:C4_01280C_A:PWP1:C4_01670C_A:ZUO1:C4_02880C_A:C4_03040W_A:C4_03150W_A:C4_03720C_A:C4_03740W_A:C4_03830W_A:RRP42:RAM1:ECM1:C4_04510W_A:SSZ1:AGP3:C4_05010W_A:C4_05260W_A:C4_05650W_A:C4_06210C_A:C4_06410W_A:NOP1:C4_06790W_A:C4_07060W_A:C4_07100C_A:RMT2:C5_00920W_A:C5_00950C_A:BUD23:PUS4:C5_01430C_A:FYV5:SPB4:C5_01930W_A:RIB2:C5_02010C_A:C5_02070C_A:RIX7:GCD11:TIF5:UTP13:C5_03010W_A:RMS1:C5_03670C_A:C5_03920C_A:C5_04610W_A:HAS1:C5_04990W_A:C5_05340W_A:NOP5:C6_01040C_A:RCN1:CIC1:C6_01890C_A:C6_02230W_A:NIP7:MRT4:POP4:C6_03210C_A:C6_03390W_A:ALS5:SNQ2:SPB1:C7_00160C_A:C7_00330C_A:RPA135:NOP15:C7_01030C_A:DBP7:C7_01360C_A:FLU1:C7_02100W_A:C7_02460C_A:C7_02930C_A:ISY1:ENP2:C7_04140C_A:UTP18:CR_00460C_A:BUD22:PWP2:CR_01410C_A:CR_01700C_A:CR_01780W_A:CR_02030C_A:CR_02420W_A:RPC19:DBP2:CR_02890C_A:SRP40:CR_03230W_A:CR_03360W_A:KTI12:NCS2:YVH1:IFF3:SGD1:PRP42:CR_03940W_A:MEX67:CR_04110W_A:CR_04170W_A:CR_04240C_A:NHP2:NOC2:CR_05550C_A:RNR3:MTG2:DBP8:SDA1:VPS75:SFL1:RPL7:FGR50:CR_06680C_A:NMD3:CR_06840W_A:CR_07030C_A:CR_07080W_A:CR_07640C_A:DBP6:IMP4:CR_08000C_A:NOP10:SSB1:RIO2:ATS1:CR_08330W_A:TSR1:CR_08500W_A:PRP8:CR_09110C_A:ELF1:SSF1:CR_09800C_A:SIK1:UTP5:CR_10410C_A:CR_10470C_A:DRS1:POP3

6364	rRNA processing	278 out of 804 genes, 34.6%	806 out of 6473 background genes, 12.5%	9.64E-68	0.00%	HBR1:CNS1:RRS1:C1_01160C_A:C1_01470W_A:C1_01900C_A:C1_02090C_A:ABP140:C1_02450C_A:PDE2:TSR2:ARO80:C1_03830C_A:C1_04040C_A:C1_04120C_A:ERB1:NOP4:RRP6:C1_05220C_A:C1_05230W_A:C1_05360C_A:RPS27A:FAL1:C1_05650W_A:C1_06540C_A:NOP6:C1_06760C_A:C1_06770W_A:C1_06800W_A:C1_07090C_A:RIA1:YPD1:PEX3:C1_07510W_A:C1_07570C_A:C1_07950C_A:FUN12:GCD6:C1_08630W_A:MSS116:C1_09390W_A:C1_09710C_A:DBP3:TPK1:C1_10620W_A:C1_10970W_A:C1_11000C_A:KRR1:C1_11560C_A:C1_11900C_A:C1_11910W_A:CSI2:C1_12350W_A:C1_12440W_A:C1_12570C_A:CHR1:C1_12760W_A:C1_14080W_A:MPP10:FAV3:NDT80:C2_00280C_A:C2_00410C_A:C2_01070W_A:C2_01510C_A:C2_02420C_A:PRS1:C2_02540W_A:C2_02710C_A:PRP3:SCH9:C2_04120C_A:C2_04570W_A:SNU114:C2_04820W_A:C2_05080C_A:MAK5:C2_05160C_A:RPF2:C2_05520W_A:TES1:LAS1:CNT:ECM17:RTA2:C2_06850W_A:NOC4:C2_07360W_A:RCL1:PRS5:C2_07920W_A:NSA1:RRP8:C2_08840W_A:PES1:RRP15:C2_09660W_A:CWC22:OCA1:PUS7:C2_10740C_A:C2_10810W_A:C3_00100W_A:UTP8:BUD21:SOF1:C3_00950C_A:BMS1:C3_01560W_A:C3_02020W_A:C3_02040C_A:UTP4:C3_02350W_A:C3_03070W_A:C3_03330C_A:HBR3:URK1:C3_04370C_A:C3_04380C_A:MAK21:SFP1:CEM1:C3_05160C_A:C3_05380W_A:QDR2:C3_05800W_A:C3_05860C_A:NOG1:DED1:C3_06150W_A:C3_06370C_A:NSA2:PRP5:C3_06760W_A:NOP13:C3_07480W_A:C3_07550C_A:UTP9:DUR4:C4_00490W_A:RLP24:NMD5:C4_00690C_A:C4_00810C_A:MDN1:RAT1:C4_01280C_A:PWP1:C4_01670C_A:ZUO1:C4_02880C_A:C4_03040W_A:C4_03150W_A:C4_03720C_A:C4_03740W_A:C4_03830W_A:RRP42:RAM1:ECM1:C4_04510W_A:SSZ1:AGP3:C4_05260W_A:C4_05650W_A:C4_06210C_A:C4_06410W_A:NOP1:C4_06790W_A:C4_07060W_A:C4_07100C_A:RMT2:C5_00920W_A:C5_00950C_A:BUD23:PUS4:C5_01430C_A:FYV5:SPB4:C5_01930W_A:RIB2:C5_02010C_A:C5_02070C_A:RIX7:UTP13:C5_03010W_A:RMS1:C5_03670C_A:C5_03920C_A:C5_04610W_A:HAS1:C5_04990W_A:C5_05340W_A:NOP5:C6_01040C_A:RCN1:CIC1:C6_01890C_A:C6_02230W_A:NIP7:MRT4:POP4:C6_03210C_A:C6_03390W_A:ALS5:SNQ2:SPB1:C7_00160C_A:C7_00330C_A:NOP15:C7_01030C_A:DBP7:C7_01360C_A:FLU1:C7_02100W_A:C7_02460C_A:C7_02930C_A:ISY1:ENP2:C7_04140C_A:UTP18:CR_00460C_A:BUD22:PWP2:CR_01410C_A:CR_01700C_A:CR_01780W_A:CR_02030C_A:CR_02420W_A:RPC19:DBP2:CR_02890C_A:SRP40:CR_03230W_A:CR_03360W_A:KTI12:NCS2:YVH1:IFF3:PRP42:CR_03940W_A:CR_04110W_A:CR_04170W_A:CR_04240C_A:NHP2:NOC2:CR_05550C_A:RNR3:MTG2:DBP8:VPS75:SFL1:RPL7:FGR50:CR_06680C_A:NMD3:CR_06840W_A:CR_07030C_A:CR_07080W_A:CR_07640C_A:DBP6:IMP4:CR_08000C_A:NOP10:SSB1:RIO2:ATS1:CR_08330W_A:TSR1:PRP8:CR_09110C_A:SSF1:CR_09800C_A:SIK1:UTP5:CR_10410C_A:CR_10470C_A:DRS1:POP3

16072	rRNA metabolic process	283 out of 804 genes, 35.2%	835 out of 6473 background genes, 12.9%	3.12E-67	0.00%	HBR1:CNS1:RRS1:C1_01160C_A:C1_01470W_A:C1_01900C_A:C1_02090C_A:ABP140:C1_02450C_A:PDE2:TSR2:ARO80:C1_03830C_A:C1_04040C_A:C1_04120C_A:ERB1:NOP4:RRP6:C1_05220C_A:C1_05230W_A:C1_05360C_A:RPS27A:FAL1:C1_05650W_A:NPL4:C1_06540C_A:NOP6:C1_06760C_A:C1_06770W_A:C1_06800W_A:C1_07090C_A:RIA1:YPD1:PEX3:C1_07510W_A:C1_07570C_A:C1_07950C_A:FUN12:GCD6:C1_08630W_A:MSS116:C1_09390W_A:C1_09710C_A:DBP3:TPK1:C1_10620W_A:C1_10970W_A:C1_11000C_A:KRR1:C1_11560C_A:C1_11900C_A:C1_11910W_A:CSI2:C1_12350W_A:C1_12440W_A:C1_12570C_A:CHR1:C1_12760W_A:C1_14080W_A:MPP10:FAV3:NDT80:C2_00280C_A:C2_00410C_A:C2_01070W_A:C2_01510C_A:C2_02420C_A:PRS1:C2_02540W_A:C2_02710C_A:PRP3:SCH9:C2_04120C_A:C2_04570W_A:SNU114:C2_04820W_A:C2_05080C_A:MAK5:C2_05160C_A:RPF2:C2_05520W_A:TES1:LAS1:CNT:ECM17:RTA2:C2_06850W_A:RPA12:NOC4:C2_07360W_A:RCL1:PRS5:C2_07920W_A:NSA1:RRP8:RPT6:C2_08840W_A:PES1:RRP15:C2_09660W_A:CWC22:OCA1:PUS7:C2_10740C_A:C2_10810W_A:C3_00100W_A:UTP8:BUD21:SOF1:C3_00950C_A:BMS1:C3_01560W_A:C3_02020W_A:C3_02040C_A:UTP4:C3_02350W_A:C3_03070W_A:C3_03330C_A:HBR3:URK1:C3_04370C_A:C3_04380C_A:MAK21:SFP1:CEM1:C3_05160C_A:C3_05380W_A:QDR2:C3_05800W_A:C3_05860C_A:NOG1:DED1:C3_06150W_A:C3_06370C_A:NSA2:PRP5:C3_06760W_A:NOP13:C3_07480W_A:C3_07550C_A:UTP9:DUR4:C4_00490W_A:RLP24:NMD5:C4_00690C_A:C4_00810C_A:MDN1:RAT1:C4_01280C_A:PWP1:C4_01670C_A:ZUO1:C4_02880C_A:C4_03040W_A:C4_03150W_A:C4_03720C_A:C4_03740W_A:C4_03830W_A:RRP42:RAM1:ECM1:C4_04510W_A:SSZ1:AGP3:C4_05260W_A:C4_05650W_A:C4_06210C_A:C4_06410W_A:NOP1:C4_06790W_A:C4_07060W_A:C4_07100C_A:RMT2:C5_00920W_A:C5_00950C_A:BUD23:PUS4:C5_01430C_A:FYV5:SPB4:C5_01930W_A:RIB2:C5_02010C_A:C5_02070C_A:RIX7:UTP13:C5_03010W_A:RMS1:C5_03670C_A:C5_03920C_A:C5_04610W_A:HAS1:C5_04990W_A:C5_05340W_A:NOP5:C6_01040C_A:RCN1:CIC1:C6_01890C_A:C6_02230W_A:NIP7:MRT4:POP4:C6_03210C_A:C6_03390W_A:ALS5:SNQ2:SPB1:C7_00160C_A:C7_00330C_A:RPA135:NOP15:C7_01030C_A:DBP7:C7_01360C_A:FLU1:C7_02100W_A:C7_02460C_A:C7_02930C_A:ISY1:ENP2:C7_04140C_A:UTP18:CR_00460C_A:BUD22:PWP2:CR_01410C_A:CR_01700C_A:CR_01780W_A:CR_01950W_A:CR_02030C_A:CR_02420W_A:RPC19:DBP2:CR_02890C_A:SRP40:CR_03230W_A:CR_03360W_A:KTI12:NCS2:YVH1:IFF3:PRP42:CR_03940W_A:CR_04110W_A:CR_04170W_A:CR_04240C_A:NHP2:NOC2:CR_05550C_A:RNR3:MTG2:DBP8:VPS75:SFL1:RPL7:FGR50:CR_06680C_A:NMD3:CR_06840W_A:CR_07030C_A:CR_07080W_A:CR_07640C_A:DBP6:IMP4:CR_08000C_A:NOP10:SSB1:RIO2:ATS1:CR_08330W_A:TSR1:PRP8:CR_09110C_A:SSF1:CR_09800C_A:SIK1:UTP5:CR_10410C_A:CR_10470C_A:DRS1:POP3

16070	RNA metabolic process	339 out of 804 genes, 42.2%	1245 out of 6473 background genes, 19.2%	7.75E-56	0.00%	HBR1:CNS1:RRS1:C1_01150C_A:C1_01160C_A:C1_01470W_A:C1_01530C_A:C1_01900C_A:C1_02090C_A:ABP140:C1_02450C_A:PDE2:TSR2:ARO80:C1_03370W_A:C1_03830C_A:C1_04040C_A:C1_04120C_A:ERB1:NOP4:C1_04990C_A:RRP6:C1_05220C_A:C1_05230W_A:C1_05360C_A:RPS27A:FAL1:C1_05650W_A:NPL4:C1_06540C_A:NOP6:C1_06760C_A:C1_06770W_A:C1_06800W_A:C1_07090C_A:RIA1:YPD1:PEX3:LHP1:C1_07510W_A:C1_07570C_A:C1_07950C_A:FUN12:GCD6:C1_08630W_A:MSS116:C1_09390W_A:C1_09710C_A:DBP3:TPK1:C1_10620W_A:C1_10970W_A:C1_11000C_A:KRR1:GAR1:C1_11560C_A:C1_11900C_A:C1_11910W_A:CSI2:C1_12350W_A:C1_12440W_A:C1_12570C_A:CHR1:C1_12670C_A:C1_12760W_A:C1_14080W_A:MPP10:FAV3:NDT80:HMA1:C2_00220C_A:C2_00280C_A:C2_00410C_A:C2_01070W_A:C2_01510C_A:C2_01870C_A:C2_02420C_A:PRS1:C2_02540W_A:C2_02710C_A:PRP3:C2_03550C_A:SCH9:C2_04120C_A:C2_04570W_A:SNU114:C2_04820W_A:CRK1:C2_05080C_A:MAK5:ERF1:C2_05160C_A:RPF2:RPC40:C2_05520W_A:KTI11:TES1:LAS1:CNT:ECM17:RTA2:C2_06480W_A:C2_06850W_A:RPA12:NOC4:C2_07360W_A:RCL1:PRS5:C2_07920W_A:NSA1:RRP8:TIF4631:RPT6:C2_08840W_A:PES1:RRP15:C2_09500W_A:C2_09660W_A:CWC22:OCA1:PUS7:C2_10740C_A:RPC11:C2_10810W_A:C3_00100W_A:UTP8:BUD21:SOF1:C3_00950C_A:BMS1:C3_01560W_A:C3_02020W_A:C3_02040C_A:UTP4:C3_02350W_A:C3_03070W_A:C3_03330C_A:HBR3:URK1:C3_04370C_A:C3_04380C_A:MAK21:SFP1:CEM1:C3_05140C_A:C3_05160C_A:C3_05380W_A:QDR2:C3_05800W_A:C3_05860C_A:CTA4:NOG1:ELP3:DED1:C3_06150W_A:C3_06370C_A:NSA2:PRP5:C3_06760W_A:NOP13:C3_07400W_A:C3_07480W_A:C3_07550C_A:RIT1:UTP9:DUR4:C4_00490W_A:RLP24:NMD5:C4_00690C_A:C4_00810C_A:C4_00940W_A:MDN1:RAT1:C4_01280C_A:PWP1:C4_01670C_A:TOA2:C4_02850W_A:ZUO1:C4_02880C_A:C4_03040W_A:C4_03150W_A:C4_03720C_A:C4_03730C_A:C4_03740W_A:C4_03830W_A:RRP42:RAM1:ECM1:C4_04510W_A:SSZ1:C4_04810C_A:AGP3:NBP35:C4_05260W_A:C4_05650W_A:C4_06210C_A:C4_06410W_A:NOP1:C4_06790W_A:C4_07060W_A:C4_07100C_A:C5_00280C_A:DUS4:RMT2:C5_00920W_A:C5_00950C_A:BUD23:PUS4:C5_01430C_A:FYV5:SPB4:C5_01610W_A:C5_01930W_A:RIB2:C5_02010C_A:C5_02070C_A:RIX7:TIF5:UTP13:C5_02730C_A:C5_03010W_A:RMS1:RPO26:C5_03670C_A:FGR27:C5_03920C_A:C5_04610W_A:HAS1:C5_04990W_A:C5_05340W_A:NOP5:C6_00530C_A:C6_00640C_A:C6_01040C_A:RCN1:CIC1:C6_01890C_A:C6_02230W_A:C6_02350C_A:NIP7:MRT4:POP4:C6_03210C_A:C6_03390W_A:FGR17:ALS5:SNQ2:SPB1:C6_04530C_A:C7_00160C_A:C7_00330C_A:RPA135:NOP15:C7_01030C_A:DBP7:C7_01360C_A:FLU1:C7_02100W_A:C7_02340C_A:C7_02460C_A:C7_02930C_A:ZCF29:ISY1:ENP2:C7_03990C_A:C7_04140C_A:UTP18:CR_00460C_A:CR_00670C_A:BUD22:PWP2:TRM1:CR_01410C_A:CR_01700C_A:CR_01780W_A:CR_01950W_A:CR_02030C_A:CR_02420W_A:RPC19:DBP2:RPC31:CR_02890C_A:SRP40:CR_03110W_A:CR_03230W_A:CR_03360W_A:KTI12:N

						CS2:YVH1:IFF3:SGD1:PRP42:CR_03940W_A:CR_04110W_A:CR_04160C_A:CR_04170W_A:CR_04240C_A:CR_04300W_A:NHP2:RPB8:NOC2:CR_05550C_A:RNR3:MTG2:DBP8:VPS75:SFL1:RPL7:FGR50:CR_06680C_A:NMD3:CR_06840W_A:CR_07030C_A:CR_07080W_A:CR_07640C_A:DBP6:IMP4:CR_08000C_A:NOP10:SSB1:RIO2:TRM9:ATS1:CR_08330W_A:TSR1:PRP8:CR_08940W_A:CR_09110C_A:SSF1:CR_09800C_A:SIK1:UTP5:CR_10410C_A:CR_10430C_A:CR_10470C_A:DRS1:POP3

42274	ribosomal small subunit biogenesis	225 out of 804 genes, 28.0%	636 out of 6473 background genes, 9.8%	1.09E-54	0.00%	HBR1:CNS1:RRS1:C1_01160C_A:C1_01470W_A:C1_02090C_A:ABP140:C1_02450C_A:TSR2:ARO80:C1_03830C_A:C1_04040C_A:C1_04120C_A:NOP4:C1_05220C_A:C1_05230W_A:C1_05360C_A:RPS27A:FAL1:C1_05650W_A:C1_06540C_A:NOP6:C1_06770W_A:C1_06800W_A:C1_07090C_A:RIA1:C1_07510W_A:C1_07570C_A:FUN12:GCD6:C1_08630W_A:MSS116:C1_09390W_A:C1_09710C_A:C1_11000C_A:KRR1:C1_11900C_A:C1_12350W_A:C1_12440W_A:C1_12570C_A:CHR1:C1_14080W_A:MPP10:FAV3:NDT80:C2_00280C_A:C2_00410C_A:C2_01070W_A:C2_01510C_A:C2_02420C_A:PRS1:C2_02540W_A:C2_02710C_A:PRP3:SCH9:C2_04120C_A:C2_04570W_A:C2_04820W_A:C2_05080C_A:MAK5:C2_05160C_A:C2_05520W_A:LAS1:RTA2:C2_06850W_A:NOC4:C2_07360W_A:RCL1:PRS5:C2_07920W_A:NSA1:RRP8:C2_08840W_A:PES1:RRP15:C2_09660W_A:CWC22:C2_10740C_A:C2_10810W_A:C3_00100W_A:UTP8:BUD21:SOF1:C3_00950C_A:BMS1:C3_01560W_A:C3_02020W_A:C3_02040C_A:UTP4:C3_02350W_A:C3_03070W_A:C3_03330C_A:HBR3:URK1:C3_04370C_A:C3_04380C_A:MAK21:SFP1:CEM1:C3_05160C_A:QDR2:C3_05800W_A:DED1:C3_06150W_A:C3_06370C_A:NSA2:PRP5:C3_06760W_A:NOP13:C3_07480W_A:C3_07550C_A:UTP9:DUR4:C4_00490W_A:RLP24:NMD5:C4_00690C_A:C4_00810C_A:MDN1:RAT1:C4_01280C_A:C4_02880C_A:C4_03040W_A:C4_03150W_A:C4_03720C_A:C4_03740W_A:C4_03830W_A:ECM1:C4_04510W_A:C4_05260W_A:C4_05650W_A:C4_06790W_A:C4_07060W_A:C4_07100C_A:C5_00920W_A:C5_00950C_A:BUD23:PUS4:C5_01430C_A:FYV5:SPB4:RIB2:C5_02010C_A:C5_02070C_A:RIX7:UTP13:C5_03010W_A:RMS1:C5_03670C_A:C5_03920C_A:HAS1:C5_04990W_A:C5_05340W_A:NOP5:C6_01040C_A:CIC1:C6_01890C_A:C6_02230W_A:NIP7:POP4:C6_03210C_A:ALS5:SNQ2:C7_00160C_A:C7_00330C_A:NOP15:C7_01030C_A:DBP7:C7_01360C_A:C7_02100W_A:C7_02460C_A:C7_02930C_A:ISY1:ENP2:C7_04140C_A:UTP18:CR_00460C_A:BUD22:PWP2:CR_01410C_A:CR_01700C_A:CR_01780W_A:CR_02030C_A:CR_02420W_A:RPC19:CR_02890C_A:SRP40:CR_03230W_A:CR_03360W_A:KTI12:NCS2:YVH1:IFF3:SGD1:CR_03940W_A:CR_04110W_A:CR_04240C_A:NOC2:CR_05550C_A:RNR3:MTG2:DBP8:VPS75:SFL1:FGR50:CR_06680C_A:NMD3:CR_06840W_A:CR_07030C_A:CR_07080W_A:DBP6:IMP4:CR_08000C_A:RIO2:ATS1:CR_08330W_A:TSR1:PRP8:ELF1:CR_09800C_A:UTP5:CR_10410C_A:CR_10470C_A:DRS1:POP3

30490	maturation of SSU-rRNA	222 out of 804 genes, 27.6%	627 out of 6473 background genes, 9.7%	6.79E-54	0.00%	HBR1:CNS1:RRS1:C1_01160C_A:C1_01470W_A:C1_02090C_A:ABP140:C1_02450C_A:TSR2:ARO80:C1_03830C_A:C1_04040C_A:C1_04120C_A:NOP4:C1_05220C_A:C1_05230W_A:C1_05360C_A:RPS27A:FAL1:C1_05650W_A:C1_06540C_A:NOP6:C1_06770W_A:C1_06800W_A:C1_07090C_A:RIA1:C1_07510W_A:C1_07570C_A:FUN12:GCD6:C1_08630W_A:MSS116:C1_09390W_A:C1_09710C_A:C1_11000C_A:KRR1:C1_11900C_A:C1_12350W_A:C1_12440W_A:C1_12570C_A:CHR1:C1_14080W_A:MPP10:FAV3:NDT80:C2_00280C_A:C2_00410C_A:C2_01070W_A:C2_01510C_A:C2_02420C_A:PRS1:C2_02540W_A:PRP3:SCH9:C2_04120C_A:C2_04570W_A:C2_04820W_A:C2_05080C_A:MAK5:C2_05160C_A:C2_05520W_A:LAS1:RTA2:C2_06850W_A:NOC4:C2_07360W_A:RCL1:PRS5:C2_07920W_A:NSA1:RRP8:C2_08840W_A:PES1:RRP15:C2_09660W_A:CWC22:C2_10740C_A:C2_10810W_A:C3_00100W_A:UTP8:BUD21:SOF1:C3_00950C_A:BMS1:C3_01560W_A:C3_02020W_A:C3_02040C_A:UTP4:C3_02350W_A:C3_03070W_A:C3_03330C_A:HBR3:URK1:C3_04370C_A:C3_04380C_A:MAK21:SFP1:CEM1:C3_05160C_A:QDR2:C3_05800W_A:DED1:C3_06150W_A:C3_06370C_A:NSA2:PRP5:C3_06760W_A:NOP13:C3_07480W_A:C3_07550C_A:UTP9:DUR4:C4_00490W_A:RLP24:NMD5:C4_00690C_A:C4_00810C_A:MDN1:RAT1:C4_01280C_A:C4_02880C_A:C4_03040W_A:C4_03150W_A:C4_03720C_A:C4_03740W_A:C4_03830W_A:ECM1:C4_04510W_A:C4_05260W_A:C4_05650W_A:C4_06790W_A:C4_07060W_A:C4_07100C_A:C5_00920W_A:C5_00950C_A:BUD23:PUS4:C5_01430C_A:FYV5:SPB4:RIB2:C5_02010C_A:C5_02070C_A:RIX7:UTP13:C5_03010W_A:RMS1:C5_03670C_A:C5_03920C_A:HAS1:C5_04990W_A:C5_05340W_A:NOP5:C6_01040C_A:CIC1:C6_01890C_A:C6_02230W_A:NIP7:POP4:C6_03210C_A:ALS5:SNQ2:C7_00160C_A:C7_00330C_A:NOP15:C7_01030C_A:DBP7:C7_01360C_A:C7_02100W_A:C7_02460C_A:C7_02930C_A:ISY1:ENP2:C7_04140C_A:UTP18:CR_00460C_A:BUD22:PWP2:CR_01410C_A:CR_01700C_A:CR_01780W_A:CR_02030C_A:CR_02420W_A:RPC19:CR_02890C_A:SRP40:CR_03230W_A:CR_03360W_A:KTI12:NCS2:YVH1:IFF3:CR_03940W_A:CR_04110W_A:CR_04240C_A:NOC2:CR_05550C_A:RNR3:MTG2:DBP8:VPS75:SFL1:FGR50:CR_06680C_A:NMD3:CR_06840W_A:CR_07030C_A:CR_07080W_A:DBP6:IMP4:CR_08000C_A:RIO2:ATS1:CR_08330W_A:TSR1:PRP8:CR_09800C_A:UTP5:CR_10410C_A:CR_10470C_A:DRS1:POP3

462	maturation of SSU-rRNA from tricistronic rRNA transcript (SSU-rRNA, 5.8S rRNA, LSU-rRNA)	219 out of 804 genes, 27.2%	620 out of 6473 background genes, 9.6%	8.08E-53	0.00%	HBR1:CNS1:RRS1:C1_01160C_A:C1_01470W_A:C1_02090C_A:ABP140:C1_02450C_A:TSR2:ARO80:C1_03830C_A:C1_04040C_A:C1_04120C_A:NOP4:C1_05220C_A:C1_05230W_A:C1_05360C_A:RPS27A:FAL1:C1_05650W_A:C1_06540C_A:NOP6:C1_06770W_A:C1_06800W_A:C1_07090C_A:RIA1:C1_07510W_A:C1_07570C_A:FUN12:GCD6:C1_08630W_A:MSS116:C1_09390W_A:C1_09710C_A:C1_11000C_A:KRR1:C1_11900C_A:C1_12350W_A:C1_12440W_A:C1_12570C_A:CHR1:C1_14080W_A:MPP10:FAV3:NDT80:C2_00280C_A:C2_00410C_A:C2_01070W_A:C2_01510C_A:C2_02420C_A:PRS1:C2_02540W_A:PRP3:SCH9:C2_04120C_A:C2_04570W_A:C2_04820W_A:C2_05080C_A:MAK5:C2_05160C_A:C2_05520W_A:LAS1:RTA2:C2_06850W_A:NOC4:C2_07360W_A:RCL1:PRS5:C2_07920W_A:NSA1:RRP8:PES1:RRP15:C2_09660W_A:CWC22:C2_10740C_A:C2_10810W_A:C3_00100W_A:UTP8:BUD21:SOF1:C3_00950C_A:BMS1:C3_01560W_A:C3_02020W_A:C3_02040C_A:UTP4:C3_02350W_A:C3_03070W_A:C3_03330C_A:HBR3:URK1:C3_04370C_A:C3_04380C_A:MAK21:SFP1:CEM1:C3_05160C_A:QDR2:C3_05800W_A:DED1:C3_06150W_A:C3_06370C_A:NSA2:PRP5:C3_06760W_A:NOP13:C3_07480W_A:C3_07550C_A:UTP9:DUR4:C4_00490W_A:RLP24:NMD5:C4_00690C_A:C4_00810C_A:MDN1:RAT1:C4_01280C_A:C4_02880C_A:C4_03040W_A:C4_03150W_A:C4_03720C_A:C4_03740W_A:C4_03830W_A:ECM1:C4_04510W_A:C4_05260W_A:C4_05650W_A:C4_06790W_A:C4_07060W_A:C4_07100C_A:C5_00920W_A:C5_00950C_A:BUD23:PUS4:C5_01430C_A:FYV5:SPB4:RIB2:C5_02010C_A:C5_02070C_A:RIX7:UTP13:C5_03010W_A:RMS1:C5_03670C_A:C5_03920C_A:HAS1:C5_04990W_A:C5_05340W_A:NOP5:C6_01040C_A:CIC1:C6_01890C_A:C6_02230W_A:NIP7:POP4:C6_03210C_A:ALS5:SNQ2:C7_00160C_A:C7_00330C_A:NOP15:C7_01030C_A:DBP7:C7_01360C_A:C7_02100W_A:C7_02460C_A:C7_02930C_A:ISY1:ENP2:C7_04140C_A:UTP18:CR_00460C_A:BUD22:PWP2:CR_01410C_A:CR_01700C_A:CR_01780W_A:CR_02030C_A:CR_02420W_A:RPC19:CR_02890C_A:SRP40:CR_03360W_A:KTI12:NCS2:YVH1:IFF3:CR_03940W_A:CR_04110W_A:NOC2:CR_05550C_A:RNR3:MTG2:DBP8:VPS75:SFL1:FGR50:CR_06680C_A:NMD3:CR_06840W_A:CR_07030C_A:CR_07080W_A:DBP6:IMP4:CR_08000C_A:RIO2:ATS1:CR_08330W_A:TSR1:PRP8:CR_09800C_A:UTP5:CR_10410C_A:CR_10470C_A:DRS1:POP3

6139	nucleobase-containing compound metabolic process	377 out of 804 genes, 46.9%	1659 out of 6473 background genes, 25.6%	2.04E-41	0.00%	HBR1:CNS1:RRS1:C1_01150C_A:C1_01160C_A:C1_01470W_A:C1_01530C_A:C1_01900C_A:C1_02090C_A:ABP140:C1_02310C_A:C1_02440C_A:C1_02450C_A:PDE2:TSR2:ARO80:C1_03370W_A:C1_03830C_A:C1_04040C_A:C1_04120C_A:ERB1:NOP4:C1_04990C_A:RRP6:C1_05220C_A:C1_05230W_A:C1_05360C_A:RPS27A:FAL1:C1_05650W_A:NPL4:C1_06120C_A:C1_06540C_A:NOP6:C1_06760C_A:C1_06770W_A:C1_06800W_A:C1_07090C_A:RIA1:YPD1:PEX3:LHP1:C1_07510W_A:C1_07570C_A:ADE4:ADE5,7:C1_07950C_A:FUN12:GCD6:C1_08630W_A:MSS116:C1_09390W_A:GUA1:C1_09710C_A:DBP3:TPK1:C1_10620W_A:HNT1:C1_10970W_A:C1_11000C_A:C1_11370C_A:KRR1:GAR1:C1_11560C_A:C1_11900C_A:C1_11910W_A:CSI2:C1_12350W_A:C1_12440W_A:C1_12570C_A:CHR1:C1_12670C_A:C1_12760W_A:C1_13270W_A:C1_14080W_A:MPP10:FAV3:NDT80:HMA1:C2_00220C_A:C2_00280C_A:C2_00410C_A:C2_01070W_A:C2_01510C_A:C2_01870C_A:C2_02420C_A:PRS1:C2_02540W_A:C2_02710C_A:HPT1:LIG4:ADE8:PRP3:C2_03360W_A:C2_03550C_A:SCH9:C2_04120C_A:C2_04570W_A:SNU114:C2_04820W_A:CRK1:C2_05080C_A:MAK5:ERF1:C2_05160C_A:RPF2:RPC40:C2_05520W_A:KTI11:TES1:LAS1:CNT:ECM17:IMH3:RTA2:C2_06480W_A:C2_06850W_A:RPA12:NOC4:C2_07360W_A:RCL1:RNR22:PRS5:C2_07920W_A:NSA1:ORF298:C2_08460C_A:RRP8:TIF4631:RPT6:SPO11:C2_08840W_A:PES1:RRP15:C2_09500W_A:C2_09660W_A:FCY21:CWC22:OCA1:PUS7:C2_10740C_A:RPC11:C2_10810W_A:C3_00100W_A:UTP8:BUD21:SOF1:C3_00950C_A:BMS1:URA3:C3_01560W_A:C3_02020W_A:C3_02040C_A:UTP4:C3_02350W_A:C3_03070W_A:C3_03330C_A:HBR3:URK1:C3_04370C_A:C3_04380C_A:ADE2:MAK21:SFP1:CEM1:C3_05140C_A:C3_05160C_A:WOR2:C3_05380W_A:QDR2:C3_05800W_A:C3_05860C_A:CTA4:NOG1:ELP3:DED1:C3_06150W_A:HNT2:C3_06370C_A:NSA2:PRP5:C3_06760W_A:C3_06940W_A:NOP13:C3_07400W_A:C3_07480W_A:C3_07550C_A:RIT1:UTP9:DUR4:C4_00490W_A:RLP24:NMD5:C4_00690C_A:C4_00700C_A:C4_00810C_A:C4_00940W_A:MDN1:RAT1:C4_01280C_A:PWP1:C4_01670C_A:TOA2:C4_02850W_A:ZUO1:C4_02880C_A:C4_03040W_A:C4_03150W_A:C4_03720C_A:C4_03730C_A:C4_03740W_A:C4_03830W_A:RRP42:RAM1:ECM1:C4_04510W_A:SSZ1:C4_04720W_A:C4_04810C_A:AGP3:NBP35:C4_05260W_A:C4_05650W_A:C4_06210C_A:C4_06410W_A:NOP1:C4_06790W_A:C4_07060W_A:C4_07100C_A:C5_00260W_A:C5_00280C_A:DUS4:RMT2:URA4:C5_00920W_A:C5_00950C_A:BUD23:PUS4:C5_01430C_A:FYV5:SPB4:C5_01610W_A:C5_01930W_A:RIB2:C5_02010C_A:C5_02070C_A:RIX7:TIF5:UTP13:C5_02730C_A:C5_03010W_A:RMS1:RPO26:FUR1:C5_03670C_A:FGR27:HAM1:C5_03920C_A:URA7:C5_04610W_A:HAS1:C5_04990W_A:C5_05340W_A:NOP5:C6_00530C_A:C6_00640C_A:C6_01040C_A:RCN1:CIC1:C6_01890C_A:C6_02230W_A:C6_02350C_A:NIP7:C6_02410W_A:C6_02560W_A:MRT4:POP4:C6_03210C_A:C6_03390W_A:FGR17:ALS5:SBA1:SNQ2:SPB1:C6_04530C_A:C7_00160C_A:C7_00330C_A:RPA135:NOP15:C7_01030C_A:DBP7:C7_01360C_A:FLU1:RAD14:C7_02100

						W_A:C7_02340C_A:C7_02460C_A:C7_02930C_A:ZCF29:ISY1:ENP2:C7_03990C_A:C7_04140C_A:UTP18:CR_00460C_A:CR_00670C_A:BUD22:PWP2:TRM1:CAB3:CR_01410C_A:CR_01700C_A:CR_01780W_A:CR_01950W_A:CR_02030C_A:CR_02420W_A:RPC19:DBP2:RPC31:CR_02890C_A:SRP40:CR_03110W_A:CR_03230W_A:CR_03360W_A:KTI12:NCS2:YVH1:IFF3:SGD1:PRP42:CR_03940W_A:CR_04110W_A:CR_04160C_A:CR_04170W_A:CR_04240C_A:CR_04300W_A:NHP2:ADE6:RPB8:NOC2:CR_05550C_A:RNR3:MTG2:DBP8:VPS75:SFL1:RPL7:FGR50:CR_06680C_A:NMD3:CR_06840W_A:CR_07030C_A:CR_07080W_A:CR_07640C_A:DBP6:IMP4:CR_08000C_A:NOP10:SSB1:RIO2:TRM9:ATS1:CR_08330W_A:TSR1:PRP8:CR_08940W_A:CR_09110C_A:CR_09140C_A:SSF1:CR_09800C_A:SIK1:UTP5:CR_10410C_A:CR_10430C_A:CR_10470C_A:DRS1:POP3

90304	nucleic acid metabolic process	352 out of 804 genes, 43.8%	1509 out of 6473 background genes, 23.3%	2.73E-40	0.00%	HBR1:CNS1:RRS1:C1_01150C_A:C1_01160C_A:C1_01470W_A:C1_01530C_A:C1_01900C_A:C1_02090C_A:ABP140:C1_02310C_A:C1_02440C_A:C1_02450C_A:PDE2:TSR2:ARO80:C1_03370W_A:C1_03830C_A:C1_04040C_A:C1_04120C_A:ERB1:NOP4:C1_04990C_A:RRP6:C1_05220C_A:C1_05230W_A:C1_05360C_A:RPS27A:FAL1:C1_05650W_A:NPL4:C1_06120C_A:C1_06540C_A:NOP6:C1_06760C_A:C1_06770W_A:C1_06800W_A:C1_07090C_A:RIA1:YPD1:PEX3:LHP1:C1_07510W_A:C1_07570C_A:C1_07950C_A:FUN12:GCD6:C1_08630W_A:MSS116:C1_09390W_A:C1_09710C_A:DBP3:TPK1:C1_10620W_A:C1_10970W_A:C1_11000C_A:C1_11370C_A:KRR1:GAR1:C1_11560C_A:C1_11900C_A:C1_11910W_A:CSI2:C1_12350W_A:C1_12440W_A:C1_12570C_A:CHR1:C1_12670C_A:C1_12760W_A:C1_14080W_A:MPP10:FAV3:NDT80:HMA1:C2_00220C_A:C2_00280C_A:C2_00410C_A:C2_01070W_A:C2_01510C_A:C2_01870C_A:C2_02420C_A:PRS1:C2_02540W_A:C2_02710C_A:LIG4:PRP3:C2_03550C_A:SCH9:C2_04120C_A:C2_04570W_A:SNU114:C2_04820W_A:CRK1:C2_05080C_A:MAK5:ERF1:C2_05160C_A:RPF2:RPC40:C2_05520W_A:KTI11:TES1:LAS1:CNT:ECM17:RTA2:C2_06480W_A:C2_06850W_A:RPA12:NOC4:C2_07360W_A:RCL1:PRS5:C2_07920W_A:NSA1:ORF298:RRP8:TIF4631:RPT6:SPO11:C2_08840W_A:PES1:RRP15:C2_09500W_A:C2_09660W_A:CWC22:OCA1:PUS7:C2_10740C_A:RPC11:C2_10810W_A:C3_00100W_A:UTP8:BUD21:SOF1:C3_00950C_A:BMS1:C3_01560W_A:C3_02020W_A:C3_02040C_A:UTP4:C3_02350W_A:C3_03070W_A:C3_03330C_A:HBR3:URK1:C3_04370C_A:C3_04380C_A:MAK21:SFP1:CEM1:C3_05140C_A:C3_05160C_A:WOR2:C3_05380W_A:QDR2:C3_05800W_A:C3_05860C_A:CTA4:NOG1:ELP3:DED1:C3_06150W_A:C3_06370C_A:NSA2:PRP5:C3_06760W_A:C3_06940W_A:NOP13:C3_07400W_A:C3_07480W_A:C3_07550C_A:RIT1:UTP9:DUR4:C4_00490W_A:RLP24:NMD5:C4_00690C_A:C4_00700C_A:C4_00810C_A:C4_00940W_A:MDN1:RAT1:C4_01280C_A:PWP1:C4_01670C_A:TOA2:C4_02850W_A:ZUO1:C4_02880C_A:C4_03040W_A:C4_03150W_A:C4_03720C_A:C4_03730C_A:C4_03740W_A:C4_03830W_A:RRP42:RAM1:ECM1:C4_04510W_A:SSZ1:C4_04810C_A:AGP3:NBP35:C4_05260W_A:C4_05650W_A:C4_06210C_A:C4_06410W_A:NOP1:C4_06790W_A:C4_07060W_A:C4_07100C_A:C5_00280C_A:DUS4:RMT2:C5_00920W_A:C5_00950C_A:BUD23:PUS4:C5_01430C_A:FYV5:SPB4:C5_01610W_A:C5_01930W_A:RIB2:C5_02010C_A:C5_02070C_A:RIX7:TIF5:UTP13:C5_02730C_A:C5_03010W_A:RMS1:RPO26:C5_03670C_A:FGR27:C5_03920C_A:C5_04610W_A:HAS1:C5_04990W_A:C5_05340W_A:NOP5:C6_00530C_A:C6_00640C_A:C6_01040C_A:RCN1:CIC1:C6_01890C_A:C6_02230W_A:C6_02350C_A:NIP7:MRT4:POP4:C6_03210C_A:C6_03390W_A:FGR17:ALS5:SBA1:SNQ2:SPB1:C6_04530C_A:C7_00160C_A:C7_00330C_A:RPA135:NOP15:C7_01030C_A:DBP7:C7_01360C_A:FLU1:RAD14:C7_02100W_A:C7_02340C_A:C7_02460C_A:C7_02930C_A:ZCF29:ISY1:ENP2:C7_03990C_A:C7_04140C_A:UTP18:CR_00460C_A:CR_00670C_A:BUD22:PWP2:TRM1:CR_01410C_A:CR_01700C_A:CR_01780W_A:CR_0195

						0W_A:CR_02030C_A:CR_02420W_A:RPC19:DBP2:RPC31:CR_02890C_A:SRP40:CR_03110W_A:CR_03230W_A:CR_03360W_A:KTI12:NCS2:YVH1:IFF3:SGD1:PRP42:CR_03940W_A:CR_04110W_A:CR_04160C_A:CR_04170W_A:CR_04240C_A:CR_04300W_A:NHP2:RPB8:NOC2:CR_05550C_A:RNR3:MTG2:DBP8:VPS75:SFL1:RPL7:FGR50:CR_06680C_A:NMD3:CR_06840W_A:CR_07030C_A:CR_07080W_A:CR_07640C_A:DBP6:IMP4:CR_08000C_A:NOP10:SSB1:RIO2:TRM9:ATS1:CR_08330W_A:TSR1:PRP8:CR_08940W_A:CR_09110C_A:CR_09140C_A:SSF1:CR_09800C_A:SIK1:UTP5:CR_10410C_A:CR_10430C_A:CR_10470C_A:DRS1:POP3

42273	ribosomal large subunit biogenesis	185 out of 804 genes, 23.0%	546 out of 6473 background genes, 8.4%	2.81E-40	0.00%	RRS1:C1_02090C_A:C1_02450C_A:PDE2:RPL6:C1_03830C_A:C1_04040C_A:C1_04120C_A:ERB1:NOP4:RRP6:C1_05220C_A:C1_05360C_A:C1_05650W_A:C1_06540C_A:NOP6:C1_06760C_A:C1_06800W_A:C1_07090C_A:RIA1:YPD1:PEX3:C1_07510W_A:C1_07570C_A:C1_08630W_A:JIP5:C1_09390W_A:YTM1:C1_09710C_A:DBP3:TPK1:C1_10970W_A:C1_11000C_A:C1_11560C_A:C1_11900C_A:C1_11910W_A:CSI2:C1_12350W_A:C1_12440W_A:C1_12570C_A:CHR1:C1_12760W_A:C1_13370W_A:C1_14080W_A:C2_00280C_A:C2_01070W_A:C2_02540W_A:PRP3:SCH9:C2_04120C_A:C2_04570W_A:SNU114:MAK5:C2_05160C_A:RPF2:TES1:LAS1:CNT:ECM17:RPL11:C2_06850W_A:NSA1:RRP8:TIF4631:C2_08840W_A:PES1:RRP15:C2_09660W_A:CWC22:OCA1:C2_10740C_A:C3_00950C_A:BMS1:C3_01560W_A:C3_02040C_A:C3_02350W_A:C3_03330C_A:URK1:C3_04370C_A:MAK21:SFP1:CEM1:C3_05160C_A:C3_05380W_A:QDR2:C3_05800W_A:C3_05860C_A:NOG1:C3_06150W_A:C3_06370C_A:NSA2:PRP5:C3_07550C_A:DUR4:RLP24:NMD5:MDN1:RAT1:C4_01280C_A:C4_01670C_A:C4_02880C_A:C4_03150W_A:C4_03720C_A:C4_03830W_A:RAM1:AGP3:C4_05010W_A:C4_05260W_A:C4_06210C_A:C4_06410W_A:C4_06790W_A:C4_07060W_A:C4_07100C_A:RMT2:C5_00920W_A:C5_01430C_A:SPB4:C5_01930W_A:C5_02010C_A:RIX7:C5_03010W_A:RMS1:C5_03670C_A:C5_03920C_A:C5_04610W_A:HAS1:C5_04990W_A:C5_05340W_A:RCN1:CIC1:C6_01890C_A:C6_02230W_A:NIP7:MRT4:C6_03210C_A:C6_03390W_A:ALS5:SNQ2:SPB1:C7_00160C_A:RPA135:NOP15:DBP7:C7_01360C_A:FLU1:C7_02460C_A:C7_02930C_A:ISY1:ENP2:UTP18:BUD22:PWP2:CR_01410C_A:CR_01700C_A:CR_02030C_A:RPC19:CR_02890C_A:CR_03360W_A:YVH1:SGD1:PRP42:CR_03940W_A:CR_04170W_A:NOC2:CR_05550C_A:RNR3:SDA1:VPS75:SFL1:RPL7:CR_06680C_A:CR_07080W_A:CR_07640C_A:DBP6:CR_08000C_A:CR_08330W_A:TSR1:CR_08500W_A:PRP8:CR_09110C_A:ELF1:SSF1:CR_09800C_A:CR_10470C_A:DRS1

46483	heterocycle metabolic process	385 out of 804 genes, 47.9%	1733 out of 6473 background genes, 26.8%	5.20E-40	0.00%	RIM8:HBR1:CNS1:RRS1:HMT1:C1_01150C_A:C1_01160C_A:C1_01470W_A:C1_01530C_A:C1_01900C_A:C1_02090C_A:ABP140:C1_02310C_A:C1_02440C_A:C1_02450C_A:PDE2:TSR2:ARO80:C1_03370W_A:C1_03830C_A:C1_04040C_A:C1_04120C_A:ERB1:NOP4:C1_04990C_A:RRP6:C1_05220C_A:C1_05230W_A:C1_05360C_A:RPS27A:RIB4:FAL1:C1_05650W_A:NPL4:C1_06120C_A:C1_06540C_A:NOP6:C1_06760C_A:C1_06770W_A:C1_06800W_A:C1_07090C_A:RIA1:YMC1:YPD1:PEX3:LHP1:C1_07510W_A:C1_07570C_A:ADE4:ADE5,7:C1_07950C_A:FUN12:GCD6:C1_08630W_A:MSS116:C1_09390W_A:GUA1:C1_09710C_A:DBP3:TPK1:C1_10620W_A:HNT1:C1_10970W_A:C1_11000C_A:C1_11370C_A:KRR1:GAR1:C1_11560C_A:C1_11900C_A:C1_11910W_A:CSI2:C1_12350W_A:C1_12440W_A:C1_12570C_A:CHR1:C1_12670C_A:C1_12760W_A:C1_13270W_A:C1_14080W_A:MPP10:FAV3:NDT80:HMA1:C2_00220C_A:C2_00280C_A:C2_00410C_A:C2_01070W_A:C2_01510C_A:C2_01870C_A:C2_02420C_A:PRS1:C2_02540W_A:C2_02710C_A:HPT1:LIG4:ADE8:PRP3:C2_03360W_A:C2_03550C_A:SCH9:C2_04120C_A:C2_04570W_A:SNU114:C2_04820W_A:CRK1:C2_05080C_A:MAK5:ERF1:C2_05160C_A:RPF2:RPC40:C2_05520W_A:KTI11:TES1:LAS1:CNT:ECM17:IMH3:RTA2:C2_06480W_A:C2_06850W_A:RPA12:NOC4:C2_07360W_A:RCL1:RNR22:PRS5:C2_07920W_A:NSA1:ORF298:C2_08460C_A:RRP8:TIF4631:RPT6:SPO11:C2_08840W_A:PES1:RRP15:C2_09500W_A:C2_09660W_A:FCY21:CWC22:OCA1:PUS7:C2_10740C_A:RPC11:C2_10810W_A:C3_00100W_A:UTP8:BUD21:SOF1:C3_00950C_A:BMS1:URA3:C3_01560W_A:C3_02020W_A:C3_02040C_A:UTP4:C3_02350W_A:C3_03070W_A:C3_03330C_A:HBR3:URK1:C3_04370C_A:C3_04380C_A:ADE2:MAK21:SFP1:CEM1:C3_05140C_A:C3_05160C_A:WOR2:C3_05380W_A:QDR2:C3_05800W_A:C3_05860C_A:CTA4:NOG1:ELP3:DED1:C3_06150W_A:HNT2:C3_06370C_A:NSA2:PRP5:C3_06760W_A:C3_06940W_A:NOP13:C3_07400W_A:C3_07480W_A:C3_07550C_A:RIT1:UTP9:DUR4:C4_00490W_A:RLP24:NMD5:C4_00690C_A:C4_00700C_A:C4_00810C_A:C4_00940W_A:MDN1:RAT1:C4_01280C_A:PWP1:C4_01670C_A:TOA2:C4_02850W_A:ZUO1:C4_02880C_A:C4_03040W_A:C4_03150W_A:C4_03720C_A:C4_03730C_A:C4_03740W_A:C4_03830W_A:RRP42:RAM1:ECM1:C4_04270W_A:C4_04510W_A:SSZ1:C4_04720W_A:C4_04810C_A:AGP3:NBP35:C4_05260W_A:C4_05650W_A:C4_06210C_A:C4_06410W_A:NOP1:C4_06790W_A:C4_07060W_A:C4_07100C_A:C5_00260W_A:C5_00280C_A:DUS4:RMT2:URA4:C5_00920W_A:C5_00950C_A:BUD23:PUS4:C5_01430C_A:FYV5:SPB4:C5_01610W_A:C5_01930W_A:RIB2:C5_02010C_A:C5_02070C_A:RIX7:TIF5:UTP13:C5_02730C_A:C5_03010W_A:RMS1:RPO26:FUR1:C5_03670C_A:FGR27:HAM1:C5_03920C_A:URA7:C5_04610W_A:HAS1:C5_04990W_A:C5_05340W_A:NOP5:C6_00530C_A:C6_00640C_A:C6_01040C_A:RCN1:CIC1:C6_01890C_A:C6_02230W_A:C6_02350C_A:NIP7:C6_02410W_A:C6_02560W_A:MRT4:POP4:C6_03210C_A:C6_03390W_A:FGR17:ALS5:SBA1:SHM2:SNQ2:SPB1:C6_04530C_A:C7_00160C_A:C7_00330C_A:RPA135:NOP15:C7_01030C_A:D

						BP7:C7_01360C_A:FLU1:RAD14:C7_02100W_A:C7_02340C_A:C7_02460C_A:C7_02930C_A:ZCF29:ISY1:ENP2:C7_03990C_A:C7_04140C_A:UTP18:MIS12:CR_00460C_A:CR_00670C_A:BUD22:PWP2:TRM1:CAB3:CR_01410C_A:CR_01700C_A:CR_01780W_A:CR_01950W_A:CR_02030C_A:CR_02420W_A:RPC19:DBP2:RPC31:CR_02890C_A:SRP40:CR_03110W_A:CR_03230W_A:CR_03360W_A:KTI12:NCS2:YVH1:IFF3:SGD1:PRP42:CR_03940W_A:CR_04110W_A:CR_04160C_A:CR_04170W_A:CR_04240C_A:CR_04300W_A:NHP2:ADE6:RPB8:CR_04920W_A:NOC2:CR_05550C_A:RNR3:MTG2:DBP8:VPS75:SFL1:RPL7:FGR50:CR_06680C_A:NMD3:CR_06840W_A:CR_07030C_A:CR_07080W_A:CR_07640C_A:DBP6:IMP4:CR_08000C_A:NOP10:SSB1:RIO2:TRM9:ATS1:CR_08330W_A:TSR1:PRP8:CR_08940W_A:CR_09110C_A:CR_09140C_A:SSF1:CR_09800C_A:SIK1:UTP5:CR_10410C_A:CR_10430C_A:CR_10470C_A:DRS1:POP3

6725	cellular aromatic compound metabolic process	384 out of 804 genes, 47.8%	1727 out of 6473 background genes, 26.7%	5.91E-40	0.00%	HBR1:CNS1:RRS1:HMT1:C1_01150C_A:C1_01160C_A:C1_01470W_A:C1_01530C_A:C1_01900C_A:C1_02090C_A:ABP140:C1_02310C_A:C1_02440C_A:C1_02450C_A:PDE2:TSR2:ARO80:C1_03370W_A:C1_03830C_A:C1_04040C_A:C1_04120C_A:ERB1:NOP4:C1_04990C_A:RRP6:C1_05220C_A:C1_05230W_A:C1_05360C_A:RPS27A:FAL1:C1_05650W_A:NPL4:C1_06120C_A:C1_06540C_A:NOP6:C1_06760C_A:C1_06770W_A:C1_06800W_A:C1_07090C_A:RIA1:YMC1:YPD1:PEX3:LHP1:C1_07510W_A:C1_07570C_A:ADE4:ADE5,7:C1_07950C_A:FUN12:GCD6:C1_08630W_A:MSS116:C1_09390W_A:GUA1:C1_09710C_A:DBP3:TPK1:C1_10620W_A:HNT1:C1_10970W_A:C1_11000C_A:C1_11370C_A:KRR1:GAR1:C1_11560C_A:C1_11900C_A:C1_11910W_A:CSI2:C1_12350W_A:C1_12440W_A:C1_12570C_A:CHR1:C1_12670C_A:C1_12760W_A:C1_13270W_A:C1_14080W_A:MPP10:FAV3:NDT80:HMA1:C2_00220C_A:C2_00280C_A:C2_00410C_A:C2_01070W_A:C2_01510C_A:C2_01870C_A:C2_02420C_A:PRS1:C2_02540W_A:C2_02710C_A:HPT1:LIG4:ADE8:PRP3:C2_03360W_A:C2_03550C_A:SCH9:C2_04120C_A:C2_04570W_A:SNU114:C2_04820W_A:CRK1:C2_05080C_A:MAK5:ERF1:C2_05160C_A:RPF2:RPC40:C2_05520W_A:KTI11:TES1:LAS1:CNT:ECM17:IMH3:RTA2:C2_06480W_A:C2_06850W_A:RPA12:NOC4:C2_07360W_A:RCL1:RNR22:PRS5:C2_07920W_A:NSA1:ORF298:C2_08460C_A:RRP8:TIF4631:RPT6:SPO11:C2_08840W_A:PES1:RRP15:C2_09500W_A:C2_09660W_A:FCY21:CWC22:OCA1:PUS7:C2_10740C_A:RPC11:C2_10810W_A:C3_00100W_A:UTP8:BUD21:SOF1:C3_00950C_A:BMS1:URA3:C3_01560W_A:C3_02020W_A:C3_02040C_A:UTP4:C3_02350W_A:PHA2:C3_03070W_A:C3_03330C_A:HBR3:URK1:C3_04370C_A:C3_04380C_A:ADE2:MAK21:SFP1:CEM1:C3_05140C_A:C3_05160C_A:WOR2:C3_05380W_A:QDR2:C3_05800W_A:C3_05860C_A:CTA4:NOG1:ELP3:DED1:C3_06150W_A:HNT2:C3_06370C_A:NSA2:PRP5:C3_06760W_A:C3_06940W_A:NOP13:C3_07400W_A:C3_07480W_A:C3_07550C_A:RIT1:UTP9:DUR4:C4_00490W_A:RLP24:NMD5:C4_00690C_A:C4_00700C_A:C4_00810C_A:C4_00940W_A:MDN1:RAT1:C4_01280C_A:PWP1:C4_01670C_A:TOA2:C4_02850W_A:ZUO1:C4_02880C_A:C4_03040W_A:C4_03150W_A:C4_03720C_A:C4_03730C_A:C4_03740W_A:C4_03830W_A:RRP42:RAM1:ECM1:C4_04510W_A:SSZ1:C4_04720W_A:C4_04810C_A:AGP3:NBP35:C4_05260W_A:C4_05650W_A:C4_06210C_A:C4_06410W_A:NOP1:C4_06790W_A:C4_07060W_A:C4_07100C_A:C5_00260W_A:C5_00280C_A:DUS4:RMT2:URA4:C5_00920W_A:C5_00950C_A:BUD23:PUS4:C5_01430C_A:FYV5:SPB4:C5_01610W_A:C5_01930W_A:RIB2:C5_02010C_A:C5_02070C_A:RIX7:TIF5:UTP13:C5_02730C_A:C5_03010W_A:RMS1:RPO26:FUR1:C5_03670C_A:FGR27:HAM1:C5_03920C_A:URA7:C5_04610W_A:HAS1:C5_04990W_A:C5_05340W_A:NOP5:C6_00530C_A:C6_00640C_A:C6_01040C_A:RCN1:CIC1:C6_01890C_A:C6_02230W_A:C6_02350C_A:NIP7:C6_02410W_A:C6_02560W_A:MRT4:POP4:C6_03210C_A:C6_03390W_A:FGR17:PAD1:ALS5:SBA1:SHM2:SNQ2:SPB1:C6_04530C_A:C7_00160C_A:C7_00330C_A:RPA135:NOP15:C7_01030C_A:DBP7:C7_

						01360C_A:FLU1:RAD14:C7_02100W_A:C7_02340C_A:C7_02460C_A:C7_02930C_A:ZCF29:ISY1:ENP2:C7_03990C_A:C7_04140C_A:UTP18:MIS12:CR_00460C_A:CR_00670C_A:BUD22:PWP2:TRM1:CAB3:CR_01410C_A:CR_01700C_A:CR_01780W_A:CR_01950W_A:CR_02030C_A:CR_02420W_A:RPC19:DBP2:RPC31:CR_02890C_A:SRP40:CR_03110W_A:CR_03230W_A:CR_03360W_A:KTI12:NCS2:YVH1:IFF3:SGD1:PRP42:CR_03940W_A:CR_04110W_A:CR_04160C_A:CR_04170W_A:CR_04240C_A:CR_04300W_A:NHP2:ADE6:RPB8:CR_04920W_A:NOC2:CR_05550C_A:RNR3:MTG2:DBP8:VPS75:SFL1:RPL7:FGR50:CR_06680C_A:NMD3:CR_06840W_A:CR_07030C_A:CR_07080W_A:CR_07640C_A:DBP6:IMP4:CR_08000C_A:NOP10:SSB1:RIO2:TRM9:ATS1:CR_08330W_A:TSR1:PRP8:CR_08940W_A:CR_09110C_A:CR_09140C_A:SSF1:CR_09800C_A:SIK1:UTP5:CR_10410C_A:CR_10430C_A:CR_10470C_A:DRS1:POP3

967	rRNA 5'-end processing	156 out of 804 genes, 19.4%	419 out of 6473 background genes, 6.5%	6.91E-39	0.00%	HBR1:CNS1:C1_02090C_A:C1_03830C_A:C1_04040C_A:C1_04120C_A:NOP4:C1_05360C_A:C1_05650W_A:C1_06540C_A:NOP6:C1_06770W_A:C1_06800W_A:C1_07090C_A:RIA1:C1_07570C_A:C1_08630W_A:MSS116:C1_09390W_A:C1_09710C_A:C1_11000C_A:C1_11900C_A:CSI2:C1_12350W_A:C1_12440W_A:C1_12570C_A:CHR1:C1_14080W_A:MPP10:FAV3:NDT80:C2_00280C_A:C2_00410C_A:C2_01510C_A:C2_02420C_A:C2_02540W_A:PRP3:SCH9:C2_04120C_A:C2_05080C_A:MAK5:C2_05160C_A:LAS1:RTA2:C2_06850W_A:NOC4:RCL1:PRS5:C2_07920W_A:NSA1:RRP8:RRP15:CWC22:C2_10740C_A:C2_10810W_A:C3_00100W_A:BUD21:BMS1:C3_02020W_A:C3_02040C_A:C3_03330C_A:HBR3:URK1:C3_04370C_A:MAK21:SFP1:CEM1:C3_05160C_A:C3_05800W_A:C3_06150W_A:C3_06370C_A:NSA2:PRP5:C3_07480W_A:C3_07550C_A:DUR4:C4_00490W_A:RLP24:NMD5:C4_00690C_A:MDN1:RAT1:C4_01280C_A:C4_02880C_A:C4_03150W_A:C4_03720C_A:C4_03830W_A:ECM1:C4_04510W_A:C4_05260W_A:C4_05650W_A:C4_06790W_A:C4_07060W_A:C4_07100C_A:C5_00920W_A:C5_00950C_A:C5_01430C_A:FYV5:SPB4:RIB2:C5_02010C_A:C5_02070C_A:RIX7:UTP13:C5_03010W_A:RMS1:C5_03670C_A:C5_03920C_A:C5_04990W_A:C5_05340W_A:NOP5:C6_02230W_A:POP4:ALS5:SNQ2:C7_00160C_A:C7_00330C_A:DBP7:C7_01360C_A:C7_02460C_A:C7_02930C_A:ISY1:C7_04140C_A:UTP18:CR_00460C_A:PWP2:CR_01410C_A:CR_01700C_A:CR_02030C_A:CR_02420W_A:CR_02890C_A:SRP40:CR_03360W_A:NCS2:YVH1:CR_03940W_A:CR_04110W_A:NOC2:CR_05550C_A:RNR3:DBP8:VPS75:SFL1:RPL7:FGR50:DBP6:IMP4:CR_08000C_A:RIO2:ATS1:CR_08330W_A:PRP8:CR_09800C_A:CR_10410C_A:DRS1:POP3

34471	ncRNA 5'-end processing	156 out of 804 genes, 19.4%	420 out of 6473 background genes, 6.5%	9.82E-39	0.00%	HBR1:CNS1:C1_02090C_A:C1_03830C_A:C1_04040C_A:C1_04120C_A:NOP4:C1_05360C_A:C1_05650W_A:C1_06540C_A:NOP6:C1_06770W_A:C1_06800W_A:C1_07090C_A:RIA1:C1_07570C_A:C1_08630W_A:MSS116:C1_09390W_A:C1_09710C_A:C1_11000C_A:C1_11900C_A:CSI2:C1_12350W_A:C1_12440W_A:C1_12570C_A:CHR1:C1_14080W_A:MPP10:FAV3:NDT80:C2_00280C_A:C2_00410C_A:C2_01510C_A:C2_02420C_A:C2_02540W_A:PRP3:SCH9:C2_04120C_A:C2_05080C_A:MAK5:C2_05160C_A:LAS1:RTA2:C2_06850W_A:NOC4:RCL1:PRS5:C2_07920W_A:NSA1:RRP8:RRP15:CWC22:C2_10740C_A:C2_10810W_A:C3_00100W_A:BUD21:BMS1:C3_02020W_A:C3_02040C_A:C3_03330C_A:HBR3:URK1:C3_04370C_A:MAK21:SFP1:CEM1:C3_05160C_A:C3_05800W_A:C3_06150W_A:C3_06370C_A:NSA2:PRP5:C3_07480W_A:C3_07550C_A:DUR4:C4_00490W_A:RLP24:NMD5:C4_00690C_A:MDN1:RAT1:C4_01280C_A:C4_02880C_A:C4_03150W_A:C4_03720C_A:C4_03830W_A:ECM1:C4_04510W_A:C4_05260W_A:C4_05650W_A:C4_06790W_A:C4_07060W_A:C4_07100C_A:C5_00920W_A:C5_00950C_A:C5_01430C_A:FYV5:SPB4:RIB2:C5_02010C_A:C5_02070C_A:RIX7:UTP13:C5_03010W_A:RMS1:C5_03670C_A:C5_03920C_A:C5_04990W_A:C5_05340W_A:NOP5:C6_02230W_A:POP4:ALS5:SNQ2:C7_00160C_A:C7_00330C_A:DBP7:C7_01360C_A:C7_02460C_A:C7_02930C_A:ISY1:C7_04140C_A:UTP18:CR_00460C_A:PWP2:CR_01410C_A:CR_01700C_A:CR_02030C_A:CR_02420W_A:CR_02890C_A:SRP40:CR_03360W_A:NCS2:YVH1:CR_03940W_A:CR_04110W_A:NOC2:CR_05550C_A:RNR3:DBP8:VPS75:SFL1:RPL7:FGR50:DBP6:IMP4:CR_08000C_A:RIO2:ATS1:CR_08330W_A:PRP8:CR_09800C_A:CR_10410C_A:DRS1:POP3

966	RNA 5'-end processing	156 out of 804 genes, 19.4%	421 out of 6473 background genes, 6.5%	1.39E-38	0.00%	HBR1:CNS1:C1_02090C_A:C1_03830C_A:C1_04040C_A:C1_04120C_A:NOP4:C1_05360C_A:C1_05650W_A:C1_06540C_A:NOP6:C1_06770W_A:C1_06800W_A:C1_07090C_A:RIA1:C1_07570C_A:C1_08630W_A:MSS116:C1_09390W_A:C1_09710C_A:C1_11000C_A:C1_11900C_A:CSI2:C1_12350W_A:C1_12440W_A:C1_12570C_A:CHR1:C1_14080W_A:MPP10:FAV3:NDT80:C2_00280C_A:C2_00410C_A:C2_01510C_A:C2_02420C_A:C2_02540W_A:PRP3:SCH9:C2_04120C_A:C2_05080C_A:MAK5:C2_05160C_A:LAS1:RTA2:C2_06850W_A:NOC4:RCL1:PRS5:C2_07920W_A:NSA1:RRP8:RRP15:CWC22:C2_10740C_A:C2_10810W_A:C3_00100W_A:BUD21:BMS1:C3_02020W_A:C3_02040C_A:C3_03330C_A:HBR3:URK1:C3_04370C_A:MAK21:SFP1:CEM1:C3_05160C_A:C3_05800W_A:C3_06150W_A:C3_06370C_A:NSA2:PRP5:C3_07480W_A:C3_07550C_A:DUR4:C4_00490W_A:RLP24:NMD5:C4_00690C_A:MDN1:RAT1:C4_01280C_A:C4_02880C_A:C4_03150W_A:C4_03720C_A:C4_03830W_A:ECM1:C4_04510W_A:C4_05260W_A:C4_05650W_A:C4_06790W_A:C4_07060W_A:C4_07100C_A:C5_00920W_A:C5_00950C_A:C5_01430C_A:FYV5:SPB4:RIB2:C5_02010C_A:C5_02070C_A:RIX7:UTP13:C5_03010W_A:RMS1:C5_03670C_A:C5_03920C_A:C5_04990W_A:C5_05340W_A:NOP5:C6_02230W_A:POP4:ALS5:SNQ2:C7_00160C_A:C7_00330C_A:DBP7:C7_01360C_A:C7_02460C_A:C7_02930C_A:ISY1:C7_04140C_A:UTP18:CR_00460C_A:PWP2:CR_01410C_A:CR_01700C_A:CR_02030C_A:CR_02420W_A:CR_02890C_A:SRP40:CR_03360W_A:NCS2:YVH1:CR_03940W_A:CR_04110W_A:NOC2:CR_05550C_A:RNR3:DBP8:VPS75:SFL1:RPL7:FGR50:DBP6:IMP4:CR_08000C_A:RIO2:ATS1:CR_08330W_A:PRP8:CR_09800C_A:CR_10410C_A:DRS1:POP3

479	endonucleolytic cleavage of tricistronic rRNA transcript (SSU-rRNA, 5.8S rRNA, LSU-rRNA)	167 out of 804 genes, 20.8%	473 out of 6473 background genes, 7.3%	1.92E-38	0.00%	HBR1:CNS1:RRS1:C1_02090C_A:ARO80:C1_03830C_A:C1_04040C_A:C1_04120C_A:NOP4:C1_05360C_A:C1_05650W_A:C1_06540C_A:NOP6:C1_06770W_A:C1_06800W_A:C1_07090C_A:RIA1:C1_07570C_A:GCD6:C1_08630W_A:MSS116:C1_09390W_A:C1_09710C_A:DBP3:C1_11000C_A:KRR1:C1_11900C_A:CSI2:C1_12350W_A:C1_12440W_A:C1_12570C_A:CHR1:C1_14080W_A:MPP10:FAV3:NDT80:C2_00280C_A:C2_00410C_A:C2_01510C_A:C2_02420C_A:C2_02540W_A:PRP3:SCH9:C2_04120C_A:C2_05080C_A:MAK5:C2_05160C_A:LAS1:RTA2:C2_06850W_A:NOC4:RCL1:PRS5:C2_07920W_A:NSA1:RRP8:RRP15:CWC22:C2_10740C_A:C2_10810W_A:C3_00100W_A:BUD21:BMS1:C3_02020W_A:C3_02040C_A:C3_02350W_A:C3_03330C_A:HBR3:URK1:C3_04370C_A:MAK21:SFP1:CEM1:C3_05160C_A:QDR2:C3_05800W_A:DED1:C3_06150W_A:C3_06370C_A:NSA2:PRP5:C3_07480W_A:C3_07550C_A:DUR4:C4_00490W_A:RLP24:NMD5:C4_00690C_A:MDN1:RAT1:C4_01280C_A:C4_02880C_A:C4_03150W_A:C4_03720C_A:C4_03830W_A:ECM1:C4_04510W_A:C4_05260W_A:C4_05650W_A:C4_06790W_A:C4_07060W_A:C4_07100C_A:C5_00920W_A:C5_00950C_A:BUD23:C5_01430C_A:FYV5:SPB4:RIB2:C5_02010C_A:C5_02070C_A:RIX7:UTP13:C5_03010W_A:RMS1:C5_03670C_A:C5_03920C_A:C5_04990W_A:C5_05340W_A:NOP5:C6_02230W_A:POP4:ALS5:SNQ2:C7_00160C_A:C7_00330C_A:DBP7:C7_01360C_A:C7_02460C_A:C7_02930C_A:ISY1:C7_04140C_A:UTP18:CR_00460C_A:PWP2:CR_01410C_A:CR_01700C_A:CR_02030C_A:CR_02420W_A:CR_02890C_A:SRP40:CR_03360W_A:NCS2:YVH1:IFF3:CR_03940W_A:CR_04110W_A:NOC2:CR_05550C_A:RNR3:DBP8:VPS75:SFL1:FGR50:CR_06680C_A:DBP6:IMP4:CR_08000C_A:RIO2:ATS1:CR_08330W_A:TSR1:PRP8:CR_09800C_A:CR_10410C_A:DRS1:POP3

36260	RNA capping	157 out of 804 genes, 19.5%	427 out of 6473 background genes, 6.6%	2.29E-38	0.00%	HBR1:CNS1:C1_02090C_A:C1_03830C_A:C1_04040C_A:C1_04120C_A:NOP4:C1_05360C_A:C1_05650W_A:C1_06540C_A:NOP6:C1_06770W_A:C1_06800W_A:C1_07090C_A:RIA1:C1_07570C_A:C1_08630W_A:MSS116:C1_09390W_A:C1_09710C_A:C1_11000C_A:C1_11900C_A:CSI2:C1_12350W_A:C1_12440W_A:C1_12570C_A:CHR1:C1_14080W_A:MPP10:FAV3:NDT80:C2_00280C_A:C2_00410C_A:C2_01510C_A:C2_02420C_A:C2_02540W_A:PRP3:SCH9:C2_04120C_A:C2_05080C_A:MAK5:C2_05160C_A:LAS1:RTA2:C2_06850W_A:NOC4:C2_07360W_A:RCL1:PRS5:C2_07920W_A:NSA1:RRP8:RRP15:CWC22:C2_10740C_A:C2_10810W_A:C3_00100W_A:BUD21:BMS1:C3_02020W_A:C3_02040C_A:C3_03330C_A:HBR3:URK1:C3_04370C_A:MAK21:SFP1:CEM1:C3_05160C_A:C3_05800W_A:C3_06150W_A:C3_06370C_A:NSA2:PRP5:C3_07480W_A:C3_07550C_A:DUR4:C4_00490W_A:RLP24:NMD5:C4_00690C_A:MDN1:RAT1:C4_01280C_A:C4_02880C_A:C4_03150W_A:C4_03720C_A:C4_03830W_A:ECM1:C4_04510W_A:C4_05260W_A:C4_05650W_A:C4_06790W_A:C4_07060W_A:C4_07100C_A:C5_00920W_A:C5_00950C_A:C5_01430C_A:FYV5:SPB4:RIB2:C5_02010C_A:C5_02070C_A:RIX7:UTP13:C5_03010W_A:RMS1:C5_03670C_A:C5_03920C_A:C5_04990W_A:C5_05340W_A:NOP5:C6_02230W_A:POP4:ALS5:SNQ2:C7_00160C_A:C7_00330C_A:DBP7:C7_01360C_A:C7_02460C_A:C7_02930C_A:ISY1:C7_04140C_A:UTP18:CR_00460C_A:PWP2:CR_01410C_A:CR_01700C_A:CR_02030C_A:CR_02420W_A:CR_02890C_A:SRP40:CR_03360W_A:NCS2:YVH1:CR_03940W_A:CR_04110W_A:NOC2:CR_05550C_A:RNR3:DBP8:VPS75:SFL1:RPL7:FGR50:DBP6:IMP4:CR_08000C_A:RIO2:ATS1:CR_08330W_A:PRP8:CR_09800C_A:CR_10410C_A:DRS1:POP3

472	endonucleolytic cleavage to generate mature 5'-end of SSU-rRNA from (SSU-rRNA, 5.8S rRNA, LSU-rRNA)	154 out of 804 genes, 19.2%	417 out of 6473 background genes, 6.4%	8.32E-38	0.00%	HBR1:CNS1:C1_02090C_A:C1_03830C_A:C1_04040C_A:C1_04120C_A:NOP4:C1_05360C_A:C1_05650W_A:C1_06540C_A:NOP6:C1_06770W_A:C1_06800W_A:C1_07090C_A:RIA1:C1_07570C_A:C1_08630W_A:MSS116:C1_09390W_A:C1_09710C_A:C1_11000C_A:C1_11900C_A:C1_12350W_A:C1_12440W_A:C1_12570C_A:CHR1:C1_14080W_A:MPP10:FAV3:NDT80:C2_00280C_A:C2_00410C_A:C2_01510C_A:C2_02420C_A:C2_02540W_A:PRP3:SCH9:C2_04120C_A:C2_05080C_A:MAK5:C2_05160C_A:LAS1:RTA2:C2_06850W_A:NOC4:RCL1:PRS5:C2_07920W_A:NSA1:RRP8:RRP15:CWC22:C2_10740C_A:C2_10810W_A:C3_00100W_A:BUD21:BMS1:C3_02020W_A:C3_02040C_A:C3_03330C_A:HBR3:URK1:C3_04370C_A:MAK21:SFP1:CEM1:C3_05160C_A:C3_05800W_A:C3_06150W_A:C3_06370C_A:NSA2:PRP5:C3_07480W_A:C3_07550C_A:DUR4:C4_00490W_A:RLP24:NMD5:C4_00690C_A:MDN1:RAT1:C4_01280C_A:C4_02880C_A:C4_03150W_A:C4_03720C_A:C4_03830W_A:ECM1:C4_04510W_A:C4_05260W_A:C4_05650W_A:C4_06790W_A:C4_07060W_A:C4_07100C_A:C5_00920W_A:C5_00950C_A:C5_01430C_A:FYV5:SPB4:RIB2:C5_02010C_A:C5_02070C_A:RIX7:UTP13:C5_03010W_A:RMS1:C5_03670C_A:C5_03920C_A:C5_04990W_A:C5_05340W_A:NOP5:C6_02230W_A:POP4:ALS5:SNQ2:C7_00160C_A:C7_00330C_A:DBP7:C7_01360C_A:C7_02460C_A:C7_02930C_A:ISY1:C7_04140C_A:UTP18:CR_00460C_A:PWP2:CR_01410C_A:CR_01700C_A:CR_02030C_A:CR_02420W_A:CR_02890C_A:SRP40:CR_03360W_A:NCS2:YVH1:CR_03940W_A:CR_04110W_A:NOC2:CR_05550C_A:RNR3:DBP8:VPS75:SFL1:FGR50:DBP6:IMP4:CR_08000C_A:RIO2:ATS1:CR_08330W_A:PRP8:CR_09800C_A:CR_10410C_A:DRS1:POP3

44085	cellular component biogenesis	316 out of 804 genes, 39.3%	1340 out of 6473 background genes, 20.7%	1.27E-35	0.00%	PHR2:HBR1:CNS1:RRS1:C1_01160C_A:C1_01470W_A:C1_01900C_A:C1_02090C_A:ABP140:C1_02450C_A:PDE2:ATG17:TSR2:RPL6:ARO80:ARX1:C1_03790C_A:C1_03830C_A:C1_04040C_A:C1_04120C_A:ERB1:NOP4:RRP6:C1_05220C_A:C1_05230W_A:C1_05360C_A:RPS27A:FAL1:C1_05650W_A:C1_06540C_A:NOP6:C1_06760C_A:C1_06770W_A:C1_06800W_A:SUI2:C1_07090C_A:RIA1:YPD1:PEX3:C1_07510W_A:C1_07570C_A:C1_07950C_A:FUN12:GCD6:C1_08630W_A:MSS116:JIP5:C1_09390W_A:YTM1:C1_09710C_A:DBP3:TPK1:C1_10620W_A:C1_10970W_A:C1_11000C_A:KRR1:GAR1:C1_11560C_A:C1_11900C_A:C1_11910W_A:CSI2:C1_12350W_A:C1_12440W_A:C1_12570C_A:CHR1:C1_12680W_A:C1_12760W_A:C1_13370W_A:SIM1:C1_14080W_A:SAM35:MPP10:FAV3:NDT80:C2_00280C_A:C2_00410C_A:C2_00810C_A:C2_01070W_A:C2_01510C_A:C2_02420C_A:PRS1:C2_02540W_A:C2_02710C_A:PRP3:MNN42:SCH9:C2_04120C_A:C2_04570W_A:SNU114:C2_04820W_A:C2_05080C_A:MAK5:C2_05160C_A:RPF2:C2_05520W_A:TES1:LAS1:CNT:ECM17:RTA2:RPL11:C2_06850W_A:NOC4:C2_07360W_A:RCL1:PRS5:C2_07920W_A:NSA1:KRE30:RRP8:TIF4631:RPT6:C2_08840W_A:PES1:RRP15:C2_09660W_A:CWC22:OCA1:PUS7:MTR2:TIF11:C2_10740C_A:C2_10810W_A:C3_00100W_A:UTP8:BUD21:SOF1:C3_00950C_A:BMS1:C3_01560W_A:C3_02020W_A:C3_02040C_A:UTP4:C3_02350W_A:C3_03070W_A:C3_03330C_A:HBR3:URK1:C3_04370C_A:C3_04380C_A:MAK21:SFP1:CEM1:C3_05160C_A:C3_05380W_A:QDR2:C3_05800W_A:C3_05860C_A:NOG1:DED1:C3_06150W_A:C3_06370C_A:NSA2:PRP5:C3_06760W_A:NOP13:C3_07480W_A:C3_07550C_A:UTP9:DUR4:C4_00490W_A:RLP24:NMD5:C4_00690C_A:C4_00810C_A:MDN1:RAT1:C4_01280C_A:PWP1:C4_01670C_A:TOA2:ZUO1:C4_02880C_A:C4_03040W_A:C4_03150W_A:C4_03720C_A:C4_03740W_A:C4_03830W_A:RRP42:RAM1:ECM1:C4_04510W_A:SSZ1:AGP3:C4_05010W_A:MYO5:NBP35:C4_05260W_A:C4_05650W_A:C4_06210C_A:C4_06410W_A:NOP1:C4_06790W_A:MNN46:C4_07060W_A:C4_07100C_A:RMT2:C5_00920W_A:C5_00950C_A:BUD23:PUS4:C5_01430C_A:FYV5:SPB4:C5_01930W_A:RIB2:C5_02010C_A:C5_02070C_A:RIX7:GCD11:TIF5:UTP13:C5_03010W_A:RMS1:C5_03670C_A:C5_03920C_A:C5_04610W_A:HAS1:C5_04990W_A:C5_05340W_A:NOP5:C6_01040C_A:RCN1:CIC1:C6_01890C_A:C6_02230W_A:NIP7:MOB1:SAC6:MRT4:POP4:C6_03210C_A:C6_03390W_A:ALS5:SNQ2:SPB1:C7_00160C_A:C7_00330C_A:RPA135:NOP15:C7_01030C_A:DBP7:C7_01360C_A:FLU1:C7_02100W_A:C7_02460C_A:C7_02930C_A:ISY1:ENP2:C7_04140C_A:UTP18:CR_00460C_A:BUD22:BMT3:PWP2:CR_01410C_A:CR_01700C_A:CR_01780W_A:CR_02030C_A:CR_02420W_A:RPC19:DBP2:CR_02890C_A:SRP40:CR_03230W_A:CR_03360W_A:KTI12:NCS2:YVH1:IFF3:SGD1:PRP42:CR_03940W_A:MEX67:CR_04110W_A:CR_04170W_A:CR_04240C_A:NHP2:NOC2:CR_05550C_A:RNR3:MTG2:DBP8:SDA1:VPS75:SFL1:RPL7:FGR50:CR_06680C_A:NMD3:CR_06840W_A:CR_07030C_A:CR_07080W_A:CR_07640C_A:DBP6:IMP4:CR_08000C_A:NOP10:SSB1:RIO2:CR_08130W_A:ATS1:CR_08

						330W_A:TSR1:CR_08500W_A:PRP8:CR_09110C_A:ELF1:SSF1:CR_09800C_A:SIK1:UTP5:CR_10410C_A:CR_10470C_A:DRS1:POP3

71826	protein-RNA complex organization	95 out of 804 genes, 11.8%	198 out of 6473 background genes, 3.1%	8.06E-33	0.00%	RPL6:C1_04040C_A:C1_04120C_A:ERB1:NOP4:RRP6:C1_05360C_A:RPS27A:C1_06760C_A:SUI2:FUN12:JIP5:YTM1:C1_09710C_A:C1_10970W_A:CSI2:C1_12440W_A:C1_12760W_A:C1_14080W_A:C2_00280C_A:C2_01070W_A:C2_02540W_A:C2_02710C_A:PRP3:SNU114:MAK5:C2_05160C_A:RPF2:RPL11:TIF11:C2_10740C_A:C3_00100W_A:BMS1:C3_01560W_A:C3_02020W_A:C3_02040C_A:C3_02350W_A:C3_03330C_A:C3_04370C_A:MAK21:C3_05160C_A:QDR2:NOG1:DED1:C3_06370C_A:PRP5:C3_07480W_A:RLP24:MDN1:C4_03150W_A:C4_05010W_A:C4_06410W_A:C4_07100C_A:RMT2:SPB4:C5_01930W_A:C5_02010C_A:C5_02070C_A:GCD11:TIF5:HAS1:C5_05340W_A:CIC1:C6_02230W_A:NIP7:SPB1:RPA135:DBP7:FLU1:ISY1:ENP2:UTP18:BUD22:PWP2:CR_01410C_A:CR_01700C_A:DBP2:YVH1:SGD1:CR_03940W_A:CR_04110W_A:CR_04170W_A:NOC2:CR_05550C_A:SDA1:CR_06680C_A:CR_07080W_A:HSP104:TSR1:PRP8:ELF1:SSF1:CR_09800C_A:CR_10470C_A:DRS1

10467	gene expression	400 out of 804 genes, 49.8%	1951 out of 6473 background genes, 30.1%	9.56E-33	0.00%	RIM8:HBR1:CNS1:RRS1:HMT1:C1_01150C_A:C1_01160C_A:C1_01470W_A:C1_01530C_A:C1_01900C_A:C1_02090C_A:ABP140:C1_02310C_A:C1_02430C_A:C1_02450C_A:HOM6:PDE2:MIA40:TSR2:RPL6:ARO80:MAS1:C1_03370W_A:C1_03790C_A:C1_03830C_A:C1_04040C_A:C1_04120C_A:ERB1:NOP4:C1_04990C_A:RRP6:C1_05220C_A:C1_05230W_A:C1_05360C_A:RPS27A:C1_05630C_A:FAL1:C1_05650W_A:C1_06540C_A:NOP6:C1_06760C_A:C1_06770W_A:MRPL19:C1_06800W_A:SUI2:C1_07090C_A:RIA1:YPD1:PEX3:LHP1:C1_07510W_A:C1_07570C_A:C1_07950C_A:FUN12:DRG1:GCD6:C1_08630W_A:MSS116:C1_09390W_A:C1_09710C_A:DBP3:TPK1:C1_10620W_A:C1_10970W_A:C1_11000C_A:KRR1:C1_11560C_A:C1_11900C_A:C1_11910W_A:CSI2:C1_12350W_A:C1_12440W_A:C1_12570C_A:CHR1:C1_12670C_A:C1_12760W_A:C1_13260W_A:HSP70:C1_14080W_A:MPP10:FAV3:NDT80:HMA1:C2_00220C_A:C2_00280C_A:C2_00410C_A:C2_00880W_A:C2_01070W_A:C2_01510C_A:C2_01870C_A:C2_02420C_A:PRS1:C2_02540W_A:C2_02710C_A:PRP3:SCH9:GCD7:C2_04120C_A:C2_04570W_A:SNU114:C2_04820W_A:CRK1:C2_05080C_A:MAK5:ERF1:C2_05160C_A:RPF2:RPC40:C2_05520W_A:KTI11:TES1:LAS1:CNT:ECM17:RTA2:C2_06480W_A:RPL11:C2_06850W_A:SPE3:RPA12:NOC4:C2_07360W_A:SSC1:RCL1:C2_07610C_A:PRS5:C2_07920W_A:NSA1:RRP8:TIF4631:C2_08840W_A:SLP2:PES1:RRP15:C2_09500W_A:C2_09660W_A:FCY21:CWC22:OCA1:PUS7:MTR2:TIF11:C2_10740C_A:RPC11:C2_10810W_A:C3_00100W_A:UTP8:BUD21:SOF1:C3_00950C_A:BMS1:C3_01520C_A:C3_01560W_A:C3_02020W_A:C3_02030W_A:C3_02040C_A:UTP4:C3_02180C_A:C3_02350W_A:C3_03070W_A:C3_03330C_A:HBR3:SAP9:URK1:C3_04370C_A:C3_04380C_A:MAK21:RPP2B:SFP1:CEM1:C3_05140C_A:C3_05160C_A:SAP3:C3_05380W_A:QDR2:C3_05800W_A:C3_05860C_A:CTA4:NOG1:ELP3:DED1:C3_06150W_A:C3_06370C_A:NSA2:PRP5:C3_06760W_A:C3_06830C_A:NOP13:C3_07400W_A:C3_07480W_A:C3_07550C_A:RIT1:UTP9:DUR4:C4_00490W_A:RLP24:NMD5:C4_00690C_A:C4_00810C_A:C4_00940W_A:MDN1:DAG7:RAT1:C4_01280C_A:PWP1:NIP1:C4_01670C_A:TOA2:HGH1:C4_02850W_A:ZUO1:C4_02880C_A:C4_03040W_A:C4_03150W_A:C4_03720C_A:C4_03730C_A:C4_03740W_A:C4_03830W_A:RRP42:RAM1:C4_04090C_A:ECM1:C4_04510W_A:SSZ1:C4_04720W_A:C4_04810C_A:AGP3:NBP35:C4_05260W_A:C4_05650W_A:C4_06210C_A:C4_06410W_A:NOP1:C4_06790W_A:C4_07060W_A:C4_07100C_A:C4_07140W_A:C5_00280C_A:DUS4:RMT2:C5_00920W_A:C5_00950C_A:BUD23:PUS4:C5_01430C_A:FYV5:SPB4:C5_01610W_A:MAS2:GIS2:C5_01930W_A:RIB2:C5_02010C_A:C5_02070C_A:RIX7:GCD11:TIF5:UTP13:C5_02730C_A:C5_03010W_A:RMS1:RPO26:FUR1:C5_03530C_A:C5_03550W_A:C5_03670C_A:FGR27:C5_03920C_A:CSU57:C5_04610W_A:HAS1:C5_04990W_A:C5_05340W_A:NOP5:FET31:C6_00530C_A:C6_00640C_A:C6_01040C_A:RCN1:CIC1:C6_01890C_A:C6_01980C_A:C6_02230W_A:C6_02350C_A:NIP7:MRT4:POP4:EMF1:C6_03210C_A:C6_03390W_A:FGR17:ALS5:SBA1:SHM2:SNQ2:SPB1:C6_04530C_A:C7_00

						160C_A:C7_00330C_A:C7_00380W_A:C7_00490C_A:RPA135:NOP15:C7_01030C_A:DBP7:GCN3:C7_01360C_A:FLU1:C7_02100W_A:C7_02340C_A:C7_02460C_A:C7_02930C_A:ZCF29:CPY1:ISY1:ENP2:C7_03990C_A:C7_04140C_A:UTP18:CR_00460C_A:CR_00670C_A:BUD22:PWP2:TRM1:CR_01410C_A:CR_01700C_A:CR_01780W_A:CR_01950W_A:CR_02030C_A:CR_02420W_A:CR_02430C_A:RPC19:DBP2:RPC31:CR_02890C_A:SRP40:CR_03110W_A:CR_03230W_A:CR_03360W_A:KTI12:NCS2:YVH1:IFF3:PRP42:CR_03940W_A:GCD1:MEX67:CR_04110W_A:CR_04160C_A:CR_04170W_A:CR_04240C_A:CR_04300W_A:NHP2:RPB8:CR_04920W_A:NOC2:CR_05550C_A:RNR3:MTG2:DBP8:VPS75:SFL1:RPL7:FGR50:CR_06680C_A:NMD3:CR_06840W_A:CR_06980W_A:CR_07030C_A:CR_07080W_A:CR_07470W_A:CR_07640C_A:DBP6:IMP4:CR_08000C_A:NOP10:SSB1:RIO2:CR_08130W_A:TRM9:HSP104:ATS1:CR_08330W_A:GSH2:TSR1:PRP8:CR_08940W_A:CR_09110C_A:ELF1:SSF1:CR_09800C_A:SIK1:UTP5:AHA1:CR_10400W_A:CR_10410C_A:CR_10430C_A:CR_10470C_A:DRS1:POP3

2E+06	organic cyclic compound metabolic process	393 out of 804 genes, 48.9%	1903 out of 6473 background genes, 29.4%	1.19E-32	0.00%	RIM8:HBR1:CNS1:RRS1:HMT1:C1_01150C_A:C1_01160C_A:C1_01470W_A:C1_01530C_A:C1_01900C_A:C1_02090C_A:ABP140:C1_02310C_A:C1_02440C_A:C1_02450C_A:PDE2:TSR2:ARO80:C1_03370W_A:C1_03830C_A:C1_04040C_A:C1_04120C_A:ERB1:NOP4:C1_04990C_A:RRP6:C1_05220C_A:C1_05230W_A:C1_05360C_A:RPS27A:RIB4:FAL1:C1_05650W_A:NPL4:C1_06120C_A:C1_06540C_A:NOP6:C1_06760C_A:C1_06770W_A:C1_06800W_A:C1_07090C_A:RIA1:YMC1:YPD1:PEX3:LHP1:C1_07510W_A:C1_07570C_A:ADE4:ADE5,7:C1_07950C_A:FUN12:GCD6:C1_08630W_A:MSS116:C1_09390W_A:GUA1:C1_09610W_A:C1_09710C_A:DBP3:TPK1:C1_10620W_A:HNT1:C1_10970W_A:C1_11000C_A:C1_11370C_A:KRR1:GAR1:C1_11560C_A:C1_11900C_A:C1_11910W_A:CSI2:C1_12350W_A:C1_12440W_A:C1_12570C_A:CHR1:C1_12670C_A:C1_12760W_A:C1_13270W_A:C1_14080W_A:MPP10:FAV3:NDT80:HMA1:C2_00220C_A:C2_00280C_A:C2_00410C_A:C2_01070W_A:C2_01510C_A:C2_01870C_A:C2_02420C_A:PRS1:C2_02540W_A:C2_02710C_A:HPT1:LIG4:ADE8:PRP3:C2_03360W_A:C2_03550C_A:SCH9:C2_04120C_A:C2_04570W_A:SNU114:C2_04820W_A:CRK1:C2_05080C_A:MAK5:ERF1:C2_05160C_A:RPF2:RPC40:C2_05520W_A:KTI11:TES1:LAS1:CNT:ECM17:IMH3:RTA2:C2_06480W_A:C2_06850W_A:RPA12:NOC4:C2_07360W_A:RCL1:RNR22:PRS5:C2_07920W_A:NSA1:ORF298:C2_08460C_A:RRP8:TIF4631:RPT6:SPO11:C2_08840W_A:PES1:RRP15:C2_09500W_A:C2_09660W_A:FCY21:CWC22:OCA1:PUS7:C2_10740C_A:RPC11:C2_10810W_A:C3_00100W_A:UTP8:BUD21:SOF1:C3_00950C_A:BMS1:URA3:C3_01560W_A:C3_02020W_A:C3_02040C_A:UTP4:C3_02350W_A:PHA2:C3_03070W_A:C3_03330C_A:HBR3:URK1:C3_04370C_A:C3_04380C_A:ADE2:MAK21:SFP1:CEM1:C3_05140C_A:C3_05160C_A:WOR2:SAP3:C3_05380W_A:QDR2:C3_05800W_A:C3_05860C_A:CTA4:NOG1:ELP3:DED1:C3_06150W_A:HNT2:C3_06370C_A:NSA2:PRP5:C3_06760W_A:C3_06940W_A:NOP13:C3_07400W_A:C3_07480W_A:C3_07550C_A:RIT1:C3_07670W_A:UTP9:DUR4:C4_00490W_A:RLP24:NMD5:C4_00690C_A:C4_00700C_A:C4_00810C_A:C4_00940W_A:MDN1:RAT1:C4_01280C_A:PWP1:C4_01670C_A:TOA2:C4_02850W_A:ZUO1:C4_02880C_A:C4_03040W_A:C4_03150W_A:C4_03720C_A:C4_03730C_A:C4_03740W_A:C4_03830W_A:RRP42:RAM1:ECM1:C4_04270W_A:C4_04510W_A:SSZ1:C4_04720W_A:C4_04810C_A:AGP3:NBP35:C4_05260W_A:C4_05650W_A:C4_06210C_A:C4_06410W_A:NOP1:C4_06790W_A:C4_07060W_A:C4_07100C_A:C5_00260W_A:C5_00280C_A:DUS4:RMT2:URA4:C5_00920W_A:C5_00950C_A:BUD23:PUS4:C5_01430C_A:FYV5:SPB4:C5_01610W_A:C5_01930W_A:RIB2:C5_02010C_A:C5_02070C_A:RIX7:TIF5:UTP13:C5_02730C_A:C5_03010W_A:RMS1:RPO26:FUR1:C5_03670C_A:FGR27:HAM1:C5_03920C_A:URA7:C5_04610W_A:HAS1:C5_04990W_A:C5_05340W_A:NOP5:C6_00530C_A:C6_00640C_A:C6_01040C_A:RCN1:CIC1:EBP1:ACF2:C6_01890C_A:C6_02230W_A:C6_02350C_A:NIP7:C6_02410W_A:C6_02560W_A:MRT4:POP4:C6_03210C_A:BMT4:C6_03390W_A:FGR17:PAD1:ALS5:SBA1:SHM2:SNQ2:SPB1:C6_

						04530C_A:C7_00160C_A:C7_00330C_A:RPA135:NOP15:C7_01030C_A:DBP7:C7_01360C_A:FLU1:RAD14:C7_02100W_A:C7_02340C_A:C7_02460C_A:C7_02930C_A:ZCF29:ISY1:ENP2:C7_03990C_A:C7_04140C_A:UTP18:MIS12:CR_00460C_A:CR_00670C_A:BUD22:PWP2:TRM1:CAB3:CR_01410C_A:CR_01700C_A:CR_01780W_A:CR_01950W_A:CR_02030C_A:CR_02420W_A:RPC19:DBP2:RPC31:CR_02890C_A:SRP40:CR_03110W_A:CR_03230W_A:CR_03360W_A:KTI12:NCS2:YVH1:IFF3:SGD1:PRP42:CR_03940W_A:CR_04110W_A:CR_04160C_A:CR_04170W_A:CR_04240C_A:CR_04300W_A:NHP2:ADE6:RPB8:CR_04920W_A:NOC2:CR_05550C_A:RNR3:MTG2:DBP8:VPS75:SFL1:RPL7:FGR50:CR_06680C_A:NMD3:CR_06840W_A:CR_07030C_A:CR_07080W_A:CR_07640C_A:DBP6:IMP4:CR_08000C_A:NOP10:SSB1:RIO2:TRM9:ATS1:CR_08330W_A:TSR1:PRP8:CR_08940W_A:CR_09110C_A:CR_09140C_A:SSF1:CR_09800C_A:SIK1:UTP5:CR_10410C_A:CR_10430C_A:CR_10470C_A:DRS1:POP3

22618	protein-RNA complex assembly	92 out of 804 genes, 11.4%	193 out of 6473 background genes, 3.0%	2.13E-31	0.00%	RPL6:C1_04040C_A:C1_04120C_A:ERB1:NOP4:RRP6:C1_05360C_A:RPS27A:C1_06760C_A:SUI2:FUN12:JIP5:C1_09710C_A:C1_10970W_A:CSI2:C1_12440W_A:C1_12760W_A:C1_14080W_A:C2_00280C_A:C2_01070W_A:C2_02540W_A:C2_02710C_A:PRP3:SNU114:MAK5:C2_05160C_A:RPF2:RPL11:TIF11:C2_10740C_A:C3_00100W_A:BMS1:C3_01560W_A:C3_02020W_A:C3_02040C_A:C3_02350W_A:C3_03330C_A:C3_04370C_A:MAK21:C3_05160C_A:QDR2:NOG1:C3_06370C_A:PRP5:C3_07480W_A:RLP24:MDN1:C4_03150W_A:C4_05010W_A:C4_06410W_A:C4_07100C_A:RMT2:SPB4:C5_01930W_A:C5_02010C_A:C5_02070C_A:GCD11:TIF5:HAS1:C5_05340W_A:CIC1:C6_02230W_A:NIP7:SPB1:RPA135:DBP7:FLU1:ISY1:ENP2:UTP18:BUD22:PWP2:CR_01410C_A:CR_01700C_A:DBP2:YVH1:SGD1:CR_03940W_A:CR_04110W_A:CR_04170W_A:NOC2:CR_05550C_A:SDA1:CR_06680C_A:CR_07080W_A:TSR1:PRP8:ELF1:SSF1:CR_09800C_A:CR_10470C_A:DRS1

42255	ribosome assembly	72 out of 804 genes, 9.0%	125 out of 6473 background genes, 1.9%	5.74E-31	0.00%	RRS1:RPL6:C1_04040C_A:ERB1:NOP4:RRP6:C1_05360C_A:RPS27A:C1_06760C_A:RIA1:FUN12:JIP5:C1_09710C_A:C1_10970W_A:CSI2:C1_12440W_A:C1_12760W_A:C1_14080W_A:C2_01070W_A:C2_02540W_A:C2_02710C_A:MAK5:C2_05160C_A:RPF2:RPL11:C2_10740C_A:BMS1:C3_01560W_A:C3_02020W_A:C3_02040C_A:C3_02350W_A:C3_04370C_A:MAK21:C3_05160C_A:QDR2:C3_06370C_A:MDN1:C4_02880C_A:C4_05010W_A:RMT2:SPB4:C5_02070C_A:TIF5:HAS1:C5_05340W_A:CIC1:C6_02230W_A:NIP7:SPB1:RPA135:DBP7:FLU1:ENP2:UTP18:BUD22:PWP2:CR_01410C_A:YVH1:SGD1:CR_03940W_A:CR_04170W_A:NOC2:CR_05550C_A:SDA1:CR_06680C_A:CR_07080W_A:TSR1:ELF1:SSF1:CR_09800C_A:CR_10470C_A:DRS1

9451	RNA modification	61 out of 804 genes, 7.6%	97 out of 6473 background genes, 1.5%	6.33E-29	0.00%	C1_01150C_A:ABP140:C1_05360C_A:C1_07570C_A:C1_09390W_A:C1_10620W_A:GAR1:C1_12570C_A:HMA1:C2_01510C_A:C2_02420C_A:C2_04820W_A:KTI11:C2_06480W_A:C2_07360W_A:RRP8:C2_08840W_A:C2_09500W_A:PUS7:C3_05140C_A:C3_05860C_A:ELP3:C3_07400W_A:RIT1:C4_00810C_A:C4_00940W_A:C4_02850W_A:C4_03730C_A:C4_03830W_A:C4_04810C_A:NBP35:C4_05260W_A:NOP1:DUS4:BUD23:PUS4:C5_01610W_A:RIB2:C5_02730C_A:C6_02350C_A:SPB1:C6_04530C_A:C7_00330C_A:C7_02340C_A:C7_03990C_A:CR_00670C_A:TRM1:CR_01780W_A:CR_02030C_A:KTI12:NCS2:CR_04110W_A:CR_04160C_A:CR_04170W_A:CR_04240C_A:CR_04300W_A:NHP2:NOP10:TRM9:ATS1:CR_08940W_A

480	endonucleolytic cleavage in 5'-ETS of tricistronic rRNA transcript (SSU-rRNA, 5.8S rRNA, LSU-rRNA)	132 out of 804 genes, 16.4%	390 out of 6473 background genes, 6.0%	3.34E-27	0.00%	HBR1:CNS1:C1_02090C_A:ARO80:C1_03830C_A:C1_04040C_A:C1_04120C_A:NOP4:C1_05360C_A:C1_05650W_A:C1_06540C_A:NOP6:C1_06770W_A:C1_06800W_A:C1_07090C_A:RIA1:C1_07570C_A:GCD6:C1_08630W_A:C1_09390W_A:C1_09710C_A:C1_11000C_A:C1_12350W_A:C1_12440W_A:C1_12570C_A:CHR1:C1_14080W_A:MPP10:FAV3:NDT80:C2_00280C_A:C2_00410C_A:C2_01510C_A:C2_02420C_A:C2_02540W_A:PRP3:SCH9:C2_04120C_A:C2_05160C_A:LAS1:RTA2:C2_06850W_A:NOC4:RCL1:PRS5:C2_07920W_A:NSA1:RRP8:RRP15:CWC22:C2_10740C_A:C2_10810W_A:C3_00100W_A:BUD21:BMS1:C3_02020W_A:C3_02040C_A:C3_03330C_A:URK1:C3_04370C_A:SFP1:QDR2:C3_05800W_A:DED1:C3_06150W_A:C3_06370C_A:PRP5:C3_07480W_A:C3_07550C_A:DUR4:C4_00490W_A:NMD5:C4_00690C_A:MDN1:RAT1:C4_01280C_A:C4_02880C_A:C4_03150W_A:C4_03830W_A:C4_04510W_A:C4_05260W_A:C4_05650W_A:C4_06790W_A:C4_07060W_A:C4_07100C_A:C5_00950C_A:C5_01430C_A:SPB4:RIB2:C5_02010C_A:C5_02070C_A:RIX7:UTP13:C5_03010W_A:RMS1:C5_03670C_A:C5_03920C_A:C5_04990W_A:C5_05340W_A:NOP5:C6_02230W_A:POP4:ALS5:SNQ2:C7_01360C_A:C7_02460C_A:C7_02930C_A:C7_04140C_A:UTP18:PWP2:CR_01700C_A:CR_02030C_A:CR_02420W_A:CR_02890C_A:CR_03360W_A:NCS2:YVH1:IFF3:CR_03940W_A:CR_05550C_A:DBP8:VPS75:SFL1:CR_06680C_A:DBP6:IMP4:CR_08000C_A:RIO2:ATS1:CR_08330W_A:PRP8:CR_09800C_A

27	ribosomal large subunit assembly	61 out of 804 genes, 7.6%	105 out of 6473 background genes, 1.6%	3.76E-26	0.00%	RPL6:C1_04040C_A:ERB1:NOP4:RRP6:C1_05360C_A:C1_06760C_A:JIP5:C1_09710C_A:C1_10970W_A:CSI2:C1_12440W_A:C1_12760W_A:C1_14080W_A:C2_01070W_A:C2_02540W_A:MAK5:C2_05160C_A:RPF2:RPL11:C2_10740C_A:BMS1:C3_01560W_A:C3_02040C_A:C3_02350W_A:C3_04370C_A:MAK21:C3_05160C_A:QDR2:C3_06370C_A:MDN1:C4_05010W_A:RMT2:SPB4:HAS1:C5_05340W_A:CIC1:C6_02230W_A:SPB1:RPA135:DBP7:FLU1:ENP2:UTP18:BUD22:PWP2:CR_01410C_A:YVH1:SGD1:CR_03940W_A:CR_04170W_A:NOC2:CR_05550C_A:SDA1:CR_06680C_A:TSR1:ELF1:SSF1:CR_09800C_A:CR_10470C_A:DRS1

34641	cellular nitrogen compound metabolic process	426 out of 804 genes, 53.0%	2297 out of 6473 background genes, 35.5%	2.00E-24	0.00%	RIM8:HBR1:C1_00510W_A:CNS1:RRS1:HMT1:C1_01150C_A:C1_01160C_A:C1_01470W_A:C1_01530C_A:C1_01900C_A:C1_02090C_A:ABP140:C1_02310C_A:C1_02430C_A:C1_02440C_A:C1_02450C_A:HOM6:PDE2:MIA40:TSR2:RPL6:ARO80:C1_03370W_A:C1_03830C_A:C1_04040C_A:C1_04120C_A:ERB1:NOP4:C1_04990C_A:RRP6:C1_05220C_A:C1_05230W_A:C1_05360C_A:RPS27A:RIB4:C1_05630C_A:FAL1:C1_05650W_A:NPL4:C1_06120C_A:C1_06540C_A:NOP6:C1_06760C_A:C1_06770W_A:MRPL19:C1_06800W_A:SUI2:C1_07090C_A:RIA1:YMC1:YPD1:PEX3:LHP1:C1_07510W_A:C1_07570C_A:ADE4:GCS1:ADE5,7:C1_07950C_A:FUN12:DRG1:GCD6:C1_08630W_A:MSS116:C1_09390W_A:GUA1:C1_09710C_A:DBP3:TPK1:C1_10620W_A:HNT1:C1_10970W_A:C1_11000C_A:C1_11370C_A:KRR1:GAR1:C1_11560C_A:C1_11900C_A:C1_11910W_A:CSI2:C1_12350W_A:C1_12440W_A:C1_12570C_A:CHR1:C1_12670C_A:C1_12760W_A:C1_13270W_A:C1_13330C_A:AGC1:C1_14080W_A:MPP10:FAV3:NDT80:HMA1:C2_00220C_A:C2_00280C_A:C2_00410C_A:C2_00880W_A:C2_01070W_A:C2_01510C_A:C2_01870C_A:C2_02420C_A:PRS1:C2_02540W_A:C2_02710C_A:HPT1:LIG4:ADE8:PRP3:C2_03360W_A:C2_03550C_A:SCH9:GCD7:C2_04120C_A:C2_04570W_A:SNU114:C2_04820W_A:CRK1:C2_05080C_A:MAK5:ERF1:C2_05160C_A:RPF2:RPC40:C2_05520W_A:KTI11:TES1:LAS1:CNT:ECM17:IMH3:RTA2:C2_06480W_A:RPL11:C2_06850W_A:SPE3:RPA12:NOC4:C2_07360W_A:RCL1:RNR22:C2_07610C_A:PRS5:C2_07920W_A:NSA1:ORF298:C2_08460C_A:RRP8:TIF4631:RPT6:SPO11:C2_08840W_A:SLP2:PES1:RRP15:C2_09500W_A:C2_09660W_A:FCY21:CWC22:OCA1:PUS7:TIF11:C2_10740C_A:RPC11:C2_10810W_A:C3_00100W_A:UTP8:BUD21:SOF1:C3_00950C_A:BMS1:URA3:C3_01520C_A:C3_01560W_A:C3_02020W_A:C3_02040C_A:UTP4:C3_02350W_A:C3_03070W_A:C3_03330C_A:HBR3:URK1:C3_04370C_A:C3_04380C_A:ADE2:MAK21:RPP2B:SFP1:CEM1:C3_05140C_A:C3_05160C_A:WOR2:C3_05380W_A:QDR2:C3_05800W_A:C3_05860C_A:CTA4:NOG1:ELP3:DED1:C3_06150W_A:HNT2:C3_06370C_A:NSA2:PRP5:C3_06760W_A:C3_06830C_A:C3_06940W_A:NOP13:C3_07400W_A:C3_07480W_A:C3_07550C_A:RIT1:UTP9:DUR4:C4_00490W_A:RLP24:NMD5:C4_00690C_A:C4_00700C_A:C4_00810C_A:C4_00940W_A:MDN1:DAG7:RAT1:C4_01280C_A:PWP1:NIP1:C4_01670C_A:TOA2:C4_02850W_A:ZUO1:C4_02880C_A:C4_03040W_A:C4_03150W_A:C4_03720C_A:C4_03730C_A:C4_03740W_A:C4_03830W_A:RRP42:RAM1:ECM1:C4_04510W_A:SSZ1:C4_04720W_A:C4_04810C_A:AGP3:NBP35:C4_05260W_A:C4_05650W_A:C4_06210C_A:C4_06410W_A:NOP1:C4_06790W_A:YDC1:C4_07060W_A:C4_07100C_A:C4_07140W_A:C5_00260W_A:C5_00280C_A:DUS4:RMT2:URA4:C5_00920W_A:C5_00950C_A:BUD23:PUS4:C5_01430C_A:FYV5:SPB4:C5_01610W_A:MAS2:GIS2:C5_01930W_A:RIB2:C5_02010C_A:C5_02070C_A:RIX7:GCD11:TIF5:UTP13:C5_02730C_A:C5_03010W_A:RMS1:RPO26:FUR1:C5_03530C_A:C5_03670C_A:FGR27:HAM1:C5_03920C_A:URA7:CSU57:C5_04610W_A:HAS1:C5_04990W_A:C5_05340W_A:NOP5:FET31:C6_005

						30C_A:C6_00640C_A:C6_01040C_A:RCN1:CIC1:C6_01890C_A:C6_01980C_A:C6_02230W_A:C6_02350C_A:NIP7:C6_02410W_A:C6_02560W_A:MRT4:POP4:C6_03210C_A:C6_03390W_A:FGR17:ALS5:SBA1:SHM2:SNQ2:SPB1:C6_04530C_A:C7_00160C_A:C7_00330C_A:C7_00380W_A:C7_00490C_A:RPA135:NOP15:C7_01030C_A:DBP7:GCN3:C7_01360C_A:FLU1:RAD14:C7_02100W_A:C7_02340C_A:C7_02460C_A:C7_02930C_A:ZCF29:CPY1:ISY1:ENP2:C7_03990C_A:C7_04140C_A:UTP18:MIS12:CR_00460C_A:CR_00670C_A:BUD22:PWP2:TRM1:CAB3:CR_01410C_A:CR_01700C_A:CR_01780W_A:CR_01950W_A:CR_02030C_A:CR_02420W_A:CR_02430C_A:RPC19:DBP2:RPC31:CR_02890C_A:SRP40:CR_03110W_A:CR_03230W_A:CR_03360W_A:KTI12:NCS2:YVH1:IFF3:SGD1:PRP42:CR_03940W_A:GCD1:CR_04110W_A:CR_04160C_A:CR_04170W_A:CR_04240C_A:CR_04300W_A:NHP2:ADE6:RPB8:CR_04920W_A:NOC2:CR_05550C_A:RNR3:MTG2:DBP8:VPS75:SFL1:RPL7:FGR50:CR_06680C_A:NMD3:CR_06840W_A:CR_07030C_A:CR_07080W_A:CR_07470W_A:CR_07640C_A:DBP6:IMP4:CR_08000C_A:NOP10:SSB1:RIO2:CR_08130W_A:TRM9:ATS1:CR_08330W_A:GSH2:TSR1:PRP8:CR_08940W_A:CR_09110C_A:CR_09140C_A:SSF1:CR_09800C_A:SIK1:UTP5:CR_10410C_A:CR_10430C_A:CR_10470C_A:DRS1:POP3

9059	macromolecule biosynthetic process	411 out of 804 genes, 51.1%	2190 out of 6473 background genes, 33.8%	3.45E-24	0.00%	RIM8:PHR2:HBR1:CNS1:RRS1:HMT1:C1_01150C_A:C1_01160C_A:C1_01470W_A:C1_01530C_A:C1_01900C_A:C1_02090C_A:ABP140:C1_02310C_A:C1_02430C_A:C1_02450C_A:HOM6:PDE2:MIA40:TSR2:RPL6:ARO80:MAS1:C1_03370W_A:C1_03790C_A:C1_03830C_A:C1_04040C_A:C1_04120C_A:ERB1:NOP4:C1_04990C_A:RRP6:C1_05220C_A:C1_05230W_A:C1_05360C_A:RPS27A:C1_05630C_A:FAL1:C1_05650W_A:C1_06540C_A:NOP6:C1_06760C_A:C1_06770W_A:MRPL19:C1_06800W_A:SUI2:C1_07090C_A:RIA1:YPD1:PEX3:LHP1:C1_07510W_A:C1_07570C_A:C1_07950C_A:FUN12:DRG1:GCD6:C1_08630W_A:MSS116:C1_09390W_A:C1_09710C_A:DBP3:TPK1:C1_10340W_A:C1_10620W_A:C1_10970W_A:C1_11000C_A:C1_11370C_A:KRR1:C1_11560C_A:C1_11900C_A:C1_11910W_A:CSI2:C1_12350W_A:C1_12440W_A:C1_12570C_A:CHR1:C1_12670C_A:C1_12760W_A:C1_13260W_A:HSP70:C1_14080W_A:FTR1:MPP10:FAV3:NDT80:HMA1:C2_00220C_A:C2_00280C_A:C2_00410C_A:C2_00880W_A:C2_01070W_A:C2_01510C_A:C2_01870C_A:C2_02420C_A:PRS1:C2_02540W_A:C2_02710C_A:LIG4:PRP3:MNN42:SCH9:GCD7:C2_04120C_A:C2_04570W_A:SNU114:C2_04820W_A:CRK1:C2_05080C_A:MAK5:ERF1:C2_05160C_A:RPF2:RPC40:C2_05520W_A:KTI11:TES1:LAS1:CNT:ECM17:RTA2:C2_06480W_A:RPL11:C2_06850W_A:SPE3:RPA12:NOC4:C2_07360W_A:SSC1:RCL1:C2_07610C_A:PRS5:C2_07920W_A:NSA1:ORF298:RRP8:TIF4631:C2_08840W_A:SLP2:PES1:RRP15:C2_09500W_A:C2_09660W_A:FCY21:CWC22:OCA1:PUS7:MTR2:TIF11:C2_10740C_A:RPC11:C2_10810W_A:C3_00100W_A:UTP8:BUD21:SOF1:C3_00950C_A:BMS1:C3_01520C_A:C3_01560W_A:C3_02020W_A:C3_02030W_A:C3_02040C_A:UTP4:C3_02180C_A:C3_02350W_A:C3_03070W_A:C3_03330C_A:HBR3:SAP9:URK1:C3_04370C_A:C3_04380C_A:MAK21:RPP2B:SFP1:CEM1:C3_05140C_A:C3_05160C_A:SAP3:C3_05380W_A:QDR2:C3_05800W_A:C3_05860C_A:CTA4:NOG1:ELP3:DED1:C3_06150W_A:C3_06370C_A:NSA2:PRP5:C3_06760W_A:C3_06830C_A:NOP13:C3_07400W_A:C3_07480W_A:C3_07550C_A:RIT1:UTP9:DUR4:C4_00490W_A:RLP24:NMD5:C4_00690C_A:C4_00810C_A:C4_00940W_A:MDN1:DAG7:RAT1:C4_01280C_A:PWP1:NIP1:C4_01670C_A:TOA2:HGH1:C4_02850W_A:ZUO1:C4_02880C_A:C4_03040W_A:C4_03150W_A:C4_03720C_A:C4_03730C_A:C4_03740W_A:C4_03830W_A:RRP42:RAM1:C4_04090C_A:ECM1:C4_04510W_A:SSZ1:C4_04720W_A:C4_04810C_A:AGP3:NBP35:C4_05260W_A:C4_05650W_A:C4_06210C_A:C4_06410W_A:MNN4:NOP1:C4_06790W_A:MNN46:C4_07060W_A:C4_07100C_A:C4_07140W_A:C5_00280C_A:DUS4:RMT2:C5_00920W_A:C5_00950C_A:BUD23:PUS4:C5_01430C_A:FYV5:SPB4:C5_01610W_A:MAS2:GIS2:C5_01930W_A:RIB2:C5_02010C_A:C5_02070C_A:RIX7:GCD11:TIF5:UTP13:C5_02730C_A:C5_03010W_A:RMS1:RPO26:FUR1:C5_03530C_A:C5_03550W_A:C5_03670C_A:FGR27:C5_03920C_A:C5_03970W_A:CSU57:C5_04610W_A:HAS1:C5_04990W_A:C5_05340W_A:NOP5:FET31:C6_00530C_A:C6_00640C_A:C6_01040C_A:RCN1:CIC1:C6_01890C_A:C6_01980C_A:C6_02230W_A:C6_02350C_A:NIP7:MRT4:POP4:E

						MF1:C6_03210C_A:C6_03390W_A:FGR17:ALS5:SBA1:SHM2:SNQ2:SPB1:C6_04530C_A:C7_00160C_A:C7_00330C_A:C7_00380W_A:C7_00490C_A:RPA135:NOP15:C7_01030C_A:DBP7:GCN3:C7_01360C_A:FLU1:C7_02100W_A:C7_02340C_A:C7_02460C_A:C7_02930C_A:ZCF29:CPY1:ISY1:ENP2:C7_03990C_A:C7_04140C_A:UTP18:CR_00460C_A:CR_00670C_A:BUD22:BMT3:PWP2:TRM1:CR_01410C_A:CR_01700C_A:CR_01780W_A:CR_01950W_A:CR_02030C_A:CR_02420W_A:CR_02430C_A:RPC19:DBP2:RPC31:CR_02890C_A:SRP40:CR_03110W_A:CR_03230W_A:CR_03360W_A:KTI12:NCS2:YVH1:IFF3:PRP42:CR_03940W_A:GCD1:MEX67:CR_04110W_A:CR_04160C_A:CR_04170W_A:CR_04240C_A:CR_04300W_A:NHP2:RPB8:CR_04920W_A:NOC2:CR_05550C_A:RNR3:MTG2:DBP8:VPS75:SFL1:RPL7:FGR50:CR_06680C_A:NMD3:CR_06840W_A:CR_06980W_A:CR_07030C_A:CR_07080W_A:CR_07470W_A:CR_07640C_A:DBP6:IMP4:CR_08000C_A:NOP10:SSB1:RIO2:CR_08130W_A:TRM9:HSP104:ATS1:CR_08330W_A:GSH2:TSR1:PRP8:CR_08940W_A:CR_09110C_A:ELF1:SSF1:CR_09800C_A:SIK1:UTP5:AHA1:CR_10400W_A:CR_10410C_A:CR_10430C_A:CR_10470C_A:DRS1:POP3

44249	cellular biosynthetic process	446 out of 804 genes, 55.5%	2485 out of 6473 background genes, 38.4%	9.82E-23	0.00%	RIM8:PHR2:HBR1:C1_00510W_A:CNS1:RRS1:HMT1:C1_01150C_A:C1_01160C_A:C1_01470W_A:C1_01530C_A:C1_01900C_A:C1_02090C_A:ABP140:C1_02310C_A:C1_02430C_A:C1_02450C_A:HOM6:PDE2:MIA40:TSR2:RPL6:ARO80:MAS1:C1_03370W_A:EHT1:C1_03790C_A:C1_03830C_A:C1_04040C_A:C1_04120C_A:ERB1:NOP4:C1_04990C_A:RHD1:RRP6:C1_05220C_A:C1_05230W_A:C1_05360C_A:RPS27A:RIB4:C1_05630C_A:FAL1:C1_05650W_A:C1_06540C_A:NOP6:C1_06760C_A:C1_06770W_A:MRPL19:C1_06800W_A:SUI2:C1_07090C_A:RIA1:YMC1:YPD1:PEX3:LHP1:C1_07510W_A:C1_07570C_A:ADE4:GCS1:ADE5,7:C1_07950C_A:FUN12:DRG1:GCD6:C1_08630W_A:MSS116:C1_09390W_A:GUA1:C1_09710C_A:DBP3:TPK1:C1_10340W_A:C1_10620W_A:C1_10970W_A:C1_11000C_A:C1_11370C_A:KRR1:C1_11560C_A:C1_11900C_A:C1_11910W_A:CSI2:C1_12350W_A:C1_12440W_A:C1_12570C_A:CHR1:C1_12670C_A:C1_12760W_A:C1_13260W_A:C1_13270W_A:C1_13330C_A:AGC1:HSP70:C1_14080W_A:FTR1:MPP10:FAV3:NDT80:HMA1:C2_00220C_A:C2_00280C_A:C2_00410C_A:C2_00880W_A:MXR1:C2_01070W_A:C2_01510C_A:C2_01870C_A:C2_02420C_A:PRS1:C2_02540W_A:SER2:C2_02710C_A:HPT1:LIG4:ADE8:PRP3:C2_03360W_A:MNN42:SCH9:GCD7:C2_04120C_A:BAT21:C2_04570W_A:SNU114:C2_04820W_A:CRK1:C2_05080C_A:MAK5:ERF1:C2_05160C_A:RPF2:RPC40:C2_05520W_A:KTI11:TES1:LAS1:CNT:ECM17:IMH3:RTA2:C2_06480W_A:RPL11:C2_06850W_A:SPE3:RPA12:NOC4:C2_07360W_A:SSC1:C2_07410W_A:RCL1:RNR22:C2_07610C_A:PRS5:C2_07920W_A:NSA1:ORF298:RRP8:TIF4631:C2_08840W_A:SLP2:PES1:RRP15:C2_09500W_A:C2_09660W_A:LEU42:FCY21:CWC22:OCA1:PUS7:MTR2:TIF11:C2_10740C_A:RPC11:C2_10810W_A:C3_00100W_A:UTP8:BUD21:SOF1:C3_00950C_A:BMS1:URA3:C3_01520C_A:C3_01560W_A:C3_02020W_A:C3_02030W_A:C3_02040C_A:UTP4:C3_02180C_A:C3_02350W_A:PHA2:C3_03070W_A:C3_03330C_A:HBR3:SAP9:URK1:C3_04370C_A:C3_04380C_A:ADE2:MAK21:RPP2B:SFP1:CEM1:C3_05140C_A:C3_05160C_A:SAP3:C3_05380W_A:QDR2:C3_05800W_A:C3_05860C_A:CTA4:NOG1:ELP3:DED1:C3_06150W_A:C3_06370C_A:NSA2:PRP5:C3_06760W_A:C3_06830C_A:NOP13:C3_07400W_A:C3_07480W_A:C3_07550C_A:RIT1:UTP9:DUR4:C4_00490W_A:RLP24:NMD5:C4_00690C_A:C4_00810C_A:C4_00940W_A:MDN1:DAG7:RAT1:C4_01280C_A:PWP1:NIP1:C4_01670C_A:TOA2:HGH1:C4_02850W_A:ZUO1:C4_02880C_A:C4_03040W_A:C4_03150W_A:C4_03720C_A:C4_03730C_A:C4_03740W_A:C4_03830W_A:RRP42:RAM1:C4_04090C_A:ECM1:C4_04510W_A:SSZ1:C4_04720W_A:C4_04810C_A:AGP3:NBP35:C4_05260W_A:C4_05650W_A:C4_06210C_A:C4_06410W_A:MNN4:NOP1:C4_06790W_A:MNN46:MET16:YDC1:C4_07060W_A:C4_07100C_A:C4_07140W_A:C5_00260W_A:C5_00280C_A:DUS4:RMT2:URA4:C5_00920W_A:C5_00950C_A:BUD23:PUS4:C5_01430C_A:FYV5:SPB4:C5_01610W_A:MAS2:GIS2:C5_01930W_A:RIB2:C5_02010C_A:C5_02070C_A:RIX7:GCD11:TIF5:UTP13:C5_02730C_A:C5_03010W_A:RMS1:RPO26:FUR1:C5_03530C_A:C5_03550W_A:C5_03670C_A:FGR27

						:C5_03920C_A:C5_03970W_A:URA7:CSU57:C5_04610W_A:HAS1:C5_04990W_A:C5_05340W_A:NOP5:FET31:C6_00530C_A:C6_00640C_A:HAL21:C6_01040C_A:RCN1:CIC1:C6_01890C_A:C6_01980C_A:C6_02230W_A:C6_02350C_A:NIP7:MRT4:POP4:EMF1:C6_03210C_A:C6_03390W_A:FGR17:ALS5:SBA1:SHM2:SNQ2:SPB1:C6_04530C_A:C7_00160C_A:C7_00330C_A:C7_00380W_A:C7_00490C_A:RPA135:NOP15:C7_01030C_A:DBP7:GCN3:C7_01360C_A:FLU1:C7_02100W_A:C7_02340C_A:C7_02460C_A:C7_02930C_A:ZCF29:CPY1:ISY1:ENP2:C7_03990C_A:C7_04140C_A:UTP18:MIS12:CR_00460C_A:CR_00670C_A:BUD22:BMT3:PWP2:TRM1:CAB3:CR_01410C_A:CR_01700C_A:CR_01780W_A:CR_01950W_A:CR_02030C_A:CR_02420W_A:CR_02430C_A:RPC19:DBP2:RPC31:CR_02890C_A:SRP40:CR_03110W_A:CR_03230W_A:CR_03360W_A:KTI12:NCS2:YVH1:IFF3:PRP42:CR_03940W_A:GCD1:MEX67:CR_04110W_A:CR_04160C_A:CR_04170W_A:CR_04240C_A:CR_04300W_A:NHP2:ADE6:RPB8:CR_04920W_A:NOC2:CR_05550C_A:RNR3:MTG2:DBP8:VPS75:SFL1:RPL7:FGR50:CR_06680C_A:NMD3:CR_06840W_A:CR_06980W_A:CR_07030C_A:CR_07080W_A:CR_07470W_A:CR_07640C_A:DBP6:IMP4:CR_08000C_A:NOP10:SSB1:RIO2:CR_08130W_A:TRM9:HSP104:ATS1:CR_08330W_A:CYS3:GSH2:TSR1:PRP8:CR_08940W_A:CR_09110C_A:ELF1:SSF1:CR_09800C_A:SIK1:UTP5:AHA1:CR_10400W_A:CR_10410C_A:CR_10430C_A:CR_10470C_A:DRS1:POP3

470	maturation of LSU-rRNA	131 out of 804 genes, 16.3%	431 out of 6473 background genes, 6.7%	8.13E-22	0.00%	C1_02090C_A:C1_02450C_A:PDE2:C1_03830C_A:C1_04120C_A:NOP4:C1_05220C_A:C1_05650W_A:C1_06540C_A:NOP6:C1_06800W_A:C1_07090C_A:RIA1:YPD1:PEX3:C1_07510W_A:C1_07570C_A:C1_08630W_A:C1_09390W_A:DBP3:TPK1:C1_11000C_A:C1_11560C_A:C1_11900C_A:C1_11910W_A:CSI2:C1_12350W_A:C1_12440W_A:C1_12570C_A:CHR1:C1_12760W_A:C2_00280C_A:C2_02540W_A:PRP3:SCH9:C2_04120C_A:SNU114:MAK5:RPF2:TES1:LAS1:CNT:ECM17:C2_06850W_A:RRP8:C2_08840W_A:RRP15:CWC22:OCA1:C2_10740C_A:C3_00950C_A:C3_01560W_A:C3_03330C_A:URK1:C3_04370C_A:CEM1:C3_05160C_A:C3_05380W_A:QDR2:C3_05800W_A:C3_05860C_A:NSA2:PRP5:C3_07550C_A:DUR4:NMD5:MDN1:RAT1:C4_01280C_A:C4_01670C_A:C4_02880C_A:C4_03150W_A:C4_03720C_A:C4_03830W_A:RAM1:AGP3:C4_05260W_A:C4_06210C_A:C4_06410W_A:C4_06790W_A:C4_07060W_A:C4_07100C_A:RMT2:C5_00920W_A:C5_01430C_A:SPB4:C5_01930W_A:C5_02010C_A:C5_03010W_A:RMS1:C5_03920C_A:C5_04610W_A:HAS1:C5_04990W_A:RCN1:CIC1:C6_01890C_A:C6_02230W_A:NIP7:C6_03210C_A:C6_03390W_A:ALS5:SNQ2:SPB1:NOP15:DBP7:C7_01360C_A:FLU1:C7_02460C_A:C7_02930C_A:ISY1:CR_01700C_A:CR_02030C_A:RPC19:CR_02890C_A:CR_03360W_A:PRP42:CR_03940W_A:CR_04170W_A:CR_05550C_A:RNR3:VPS75:SFL1:RPL7:CR_07080W_A:CR_07640C_A:DBP6:CR_08000C_A:PRP8:CR_09110C_A:SSF1

463	maturation of LSU-rRNA from tricistronic rRNA transcript (SSU-rRNA, 5.8S rRNA, LSU-rRNA)	130 out of 804 genes, 16.2%	429 out of 6473 background genes, 6.6%	1.74E-21	0.00%	C1_02090C_A:C1_02450C_A:PDE2:C1_03830C_A:C1_04120C_A:NOP4:C1_05220C_A:C1_05650W_A:C1_06540C_A:NOP6:C1_06800W_A:C1_07090C_A:RIA1:YPD1:PEX3:C1_07510W_A:C1_07570C_A:C1_08630W_A:C1_09390W_A:DBP3:TPK1:C1_11000C_A:C1_11560C_A:C1_11900C_A:C1_11910W_A:CSI2:C1_12350W_A:C1_12440W_A:C1_12570C_A:CHR1:C1_12760W_A:C2_00280C_A:C2_02540W_A:PRP3:SCH9:C2_04120C_A:SNU114:MAK5:RPF2:TES1:LAS1:CNT:ECM17:C2_06850W_A:RRP8:RRP15:CWC22:OCA1:C2_10740C_A:C3_00950C_A:C3_01560W_A:C3_03330C_A:URK1:C3_04370C_A:CEM1:C3_05160C_A:C3_05380W_A:QDR2:C3_05800W_A:C3_05860C_A:NSA2:PRP5:C3_07550C_A:DUR4:NMD5:MDN1:RAT1:C4_01280C_A:C4_01670C_A:C4_02880C_A:C4_03150W_A:C4_03720C_A:C4_03830W_A:RAM1:AGP3:C4_05260W_A:C4_06210C_A:C4_06410W_A:C4_06790W_A:C4_07060W_A:C4_07100C_A:RMT2:C5_00920W_A:C5_01430C_A:SPB4:C5_01930W_A:C5_02010C_A:C5_03010W_A:RMS1:C5_03920C_A:C5_04610W_A:HAS1:C5_04990W_A:RCN1:CIC1:C6_01890C_A:C6_02230W_A:NIP7:C6_03210C_A:C6_03390W_A:ALS5:SNQ2:SPB1:NOP15:DBP7:C7_01360C_A:FLU1:C7_02460C_A:C7_02930C_A:ISY1:CR_01700C_A:CR_02030C_A:RPC19:CR_02890C_A:CR_03360W_A:PRP42:CR_03940W_A:CR_04170W_A:CR_05550C_A:RNR3:VPS75:SFL1:RPL7:CR_07080W_A:CR_07640C_A:DBP6:CR_08000C_A:PRP8:CR_09110C_A:SSF1

9058	biosynthetic process	454 out of 804 genes, 56.5%	2586 out of 6473 background genes, 40.0%	5.33E-21	0.00%	RIM8:PHR2:HBR1:C1_00510W_A:CNS1:RRS1:HMT1:C1_01150C_A:C1_01160C_A:C1_01470W_A:C1_01530C_A:C1_01900C_A:C1_02090C_A:ABP140:C1_02310C_A:C1_02430C_A:C1_02450C_A:HOM6:PDE2:MIA40:TSR2:RPL6:ARO80:MAS1:C1_03370W_A:EHT1:C1_03790C_A:C1_03830C_A:C1_04040C_A:C1_04120C_A:ERB1:NOP4:C1_04990C_A:RHD1:RRP6:C1_05220C_A:C1_05230W_A:C1_05360C_A:RPS27A:RIB4:C1_05630C_A:FAL1:C1_05650W_A:C1_06540C_A:NOP6:C1_06760C_A:C1_06770W_A:MRPL19:C1_06800W_A:SUI2:C1_07090C_A:RIA1:YMC1:YPD1:PEX3:LHP1:C1_07510W_A:C1_07570C_A:ADE4:GCS1:ADE5,7:C1_07950C_A:FUN12:DRG1:GCD6:C1_08630W_A:MSS116:C1_09390W_A:GUA1:C1_09610W_A:C1_09710C_A:DBP3:TPK1:C1_10340W_A:C1_10620W_A:C1_10970W_A:C1_11000C_A:C1_11370C_A:KRR1:C1_11560C_A:C1_11900C_A:C1_11910W_A:CSI2:C1_12350W_A:C1_12440W_A:C1_12570C_A:CHR1:C1_12670C_A:C1_12760W_A:PSA2:C1_13260W_A:C1_13270W_A:C1_13330C_A:AGC1:HSP70:C1_14080W_A:FTR1:MPP10:FAV3:NDT80:HMA1:C2_00220C_A:C2_00280C_A:C2_00410C_A:C2_00880W_A:MXR1:C2_01070W_A:C2_01510C_A:C2_01870C_A:C2_02420C_A:PRS1:C2_02540W_A:SER2:C2_02710C_A:HPT1:LIG4:ADE8:PRP3:C2_03360W_A:MNN42:SCH9:GCD7:C2_04120C_A:BAT21:C2_04570W_A:SNU114:C2_04820W_A:CRK1:C2_05080C_A:MAK5:ERF1:C2_05160C_A:RPF2:RPC40:C2_05520W_A:KTI11:TES1:LAS1:CNT:ECM17:IMH3:RTA2:C2_06480W_A:RPL11:C2_06850W_A:SPE3:RPA12:NOC4:C2_07360W_A:SSC1:C2_07410W_A:RCL1:RNR22:C2_07610C_A:PRS5:C2_07920W_A:NSA1:ORF298:RRP8:TIF4631:C2_08840W_A:SLP2:PES1:RRP15:C2_09500W_A:C2_09660W_A:LEU42:FCY21:YOR1:CWC22:OCA1:GPD1:PUS7:MTR2:TIF11:C2_10740C_A:RPC11:C2_10810W_A:C3_00100W_A:UTP8:BUD21:SOF1:C3_00950C_A:BMS1:URA3:C3_01520C_A:C3_01560W_A:C3_02020W_A:C3_02030W_A:C3_02040C_A:UTP4:C3_02180C_A:C3_02350W_A:PHA2:C3_03070W_A:C3_03330C_A:HBR3:SAP9:URK1:C3_04370C_A:C3_04380C_A:ADE2:MAK21:RPP2B:SFP1:CEM1:C3_05140C_A:C3_05160C_A:SAP3:C3_05380W_A:QDR2:C3_05800W_A:C3_05860C_A:CTA4:NOG1:ELP3:DED1:C3_06150W_A:C3_06370C_A:NSA2:PRP5:C3_06760W_A:C3_06830C_A:NOP13:C3_07400W_A:C3_07480W_A:C3_07550C_A:RIT1:C3_07670W_A:UTP9:DUR4:C4_00490W_A:RLP24:NMD5:C4_00690C_A:C4_00810C_A:C4_00940W_A:MDN1:DAG7:RAT1:C4_01280C_A:PWP1:NIP1:C4_01670C_A:TOA2:HGH1:C4_02850W_A:ZUO1:C4_02880C_A:C4_03040W_A:C4_03150W_A:C4_03720C_A:C4_03730C_A:C4_03740W_A:C4_03830W_A:RRP42:RAM1:C4_04090C_A:ECM1:C4_04510W_A:SSZ1:C4_04720W_A:C4_04810C_A:AGP3:NBP35:C4_05260W_A:C4_05650W_A:C4_06210C_A:C4_06410W_A:MNN4:NOP1:C4_06790W_A:MNN46:MET16:YDC1:C4_07060W_A:C4_07100C_A:C4_07140W_A:VIP1:C5_00260W_A:C5_00280C_A:DUS4:RMT2:URA4:C5_00920W_A:C5_00950C_A:BUD23:PUS4:C5_01430C_A:FYV5:SPB4:C5_01610W_A:MAS2:GIS2:C5_01930W_A:RIB2:C5_02010C_A:C5_02070C_A:RIX7:GCD11:TIF5:UTP13:C5_02730C_A:C5_03010W_A:RMS1:RPO26:F

						UR1:C5_03530C_A:C5_03550W_A:C5_03670C_A:FGR27:C5_03920C_A:C5_03970W_A:URA7:CSU57:C5_04610W_A:HAS1:C5_04990W_A:C5_05340W_A:NOP5:FET31:C6_00530C_A:C6_00640C_A:HAL21:C6_01040C_A:RCN1:CIC1:ACF2:C6_01890C_A:C6_01980C_A:C6_02230W_A:C6_02350C_A:NIP7:MRT4:POP4:EMF1:C6_03210C_A:BMT4:C6_03390W_A:FGR17:ALS5:SBA1:SHM2:SNQ2:SPB1:C6_04530C_A:C7_00160C_A:C7_00330C_A:C7_00380W_A:C7_00490C_A:RPA135:NOP15:C7_01030C_A:DBP7:GCN3:C7_01360C_A:FLU1:C7_02100W_A:C7_02340C_A:C7_02460C_A:C7_02930C_A:ZCF29:CPY1:ISY1:ENP2:C7_03990C_A:C7_04140C_A:UTP18:MIS12:CR_00460C_A:CR_00670C_A:BUD22:BMT3:PWP2:TRM1:CAB3:CR_01410C_A:CR_01700C_A:CR_01780W_A:CR_01950W_A:CR_02030C_A:CR_02420W_A:CR_02430C_A:RPC19:DBP2:RPC31:CR_02890C_A:SRP40:CR_03110W_A:CR_03230W_A:CR_03360W_A:KTI12:NCS2:YVH1:IFF3:PRP42:CR_03940W_A:GCD1:MEX67:CR_04110W_A:CR_04160C_A:CR_04170W_A:CR_04240C_A:CR_04300W_A:NHP2:ADE6:RPB8:CR_04920W_A:NOC2:CR_05550C_A:RNR3:MTG2:DBP8:VPS75:SFL1:RPL7:FGR50:CR_06680C_A:NMD3:CR_06840W_A:CR_06980W_A:CR_07030C_A:CR_07080W_A:CR_07470W_A:CR_07640C_A:DBP6:IMP4:CR_08000C_A:NOP10:SSB1:RIO2:CR_08130W_A:TRM9:HSP104:ATS1:CR_08330W_A:CYS3:GSH2:TSR1:PRP8:CR_08940W_A:CR_09110C_A:ELF1:SSF1:CR_09800C_A:SIK1:UTP5:AHA1:CR_10400W_A:CR_10410C_A:CR_10430C_A:CR_10470C_A:DRS1:POP3

2E+06	organic substance biosynthetic process	451 out of 804 genes, 56.1%	2571 out of 6473 background genes, 39.7%	1.20E-20	0.00%	RIM8:PHR2:HBR1:C1_00510W_A:CNS1:RRS1:HMT1:C1_01150C_A:C1_01160C_A:C1_01470W_A:C1_01530C_A:C1_01900C_A:C1_02090C_A:ABP140:C1_02310C_A:C1_02430C_A:C1_02450C_A:HOM6:PDE2:MIA40:TSR2:RPL6:ARO80:MAS1:C1_03370W_A:EHT1:C1_03790C_A:C1_03830C_A:C1_04040C_A:C1_04120C_A:ERB1:NOP4:C1_04990C_A:RHD1:RRP6:C1_05220C_A:C1_05230W_A:C1_05360C_A:RPS27A:RIB4:C1_05630C_A:FAL1:C1_05650W_A:C1_06540C_A:NOP6:C1_06760C_A:C1_06770W_A:MRPL19:C1_06800W_A:SUI2:C1_07090C_A:RIA1:YMC1:YPD1:PEX3:LHP1:C1_07510W_A:C1_07570C_A:ADE4:GCS1:ADE5,7:C1_07950C_A:FUN12:DRG1:GCD6:C1_08630W_A:MSS116:C1_09390W_A:GUA1:C1_09610W_A:C1_09710C_A:DBP3:TPK1:C1_10340W_A:C1_10620W_A:C1_10970W_A:C1_11000C_A:C1_11370C_A:KRR1:C1_11560C_A:C1_11900C_A:C1_11910W_A:CSI2:C1_12350W_A:C1_12440W_A:C1_12570C_A:CHR1:C1_12670C_A:C1_12760W_A:C1_13260W_A:C1_13270W_A:C1_13330C_A:HSP70:C1_14080W_A:FTR1:MPP10:FAV3:NDT80:HMA1:C2_00220C_A:C2_00280C_A:C2_00410C_A:C2_00880W_A:MXR1:C2_01070W_A:C2_01510C_A:C2_01870C_A:C2_02420C_A:PRS1:C2_02540W_A:SER2:C2_02710C_A:HPT1:LIG4:ADE8:PRP3:C2_03360W_A:MNN42:SCH9:GCD7:C2_04120C_A:BAT21:C2_04570W_A:SNU114:C2_04820W_A:CRK1:C2_05080C_A:MAK5:ERF1:C2_05160C_A:RPF2:RPC40:C2_05520W_A:KTI11:TES1:LAS1:CNT:ECM17:IMH3:RTA2:C2_06480W_A:RPL11:C2_06850W_A:SPE3:RPA12:NOC4:C2_07360W_A:SSC1:C2_07410W_A:RCL1:RNR22:C2_07610C_A:PRS5:C2_07920W_A:NSA1:ORF298:RRP8:TIF4631:C2_08840W_A:SLP2:PES1:RRP15:C2_09500W_A:C2_09660W_A:LEU42:FCY21:CWC22:OCA1:GPD1:PUS7:MTR2:TIF11:C2_10740C_A:RPC11:C2_10810W_A:C3_00100W_A:UTP8:BUD21:SOF1:C3_00950C_A:BMS1:URA3:C3_01520C_A:C3_01560W_A:C3_02020W_A:C3_02030W_A:C3_02040C_A:UTP4:C3_02180C_A:C3_02350W_A:PHA2:C3_03070W_A:C3_03330C_A:HBR3:SAP9:URK1:C3_04370C_A:C3_04380C_A:ADE2:MAK21:RPP2B:SFP1:CEM1:C3_05140C_A:C3_05160C_A:SAP3:C3_05380W_A:QDR2:C3_05800W_A:C3_05860C_A:CTA4:NOG1:ELP3:DED1:C3_06150W_A:C3_06370C_A:NSA2:PRP5:C3_06760W_A:C3_06830C_A:NOP13:C3_07400W_A:C3_07480W_A:C3_07550C_A:RIT1:C3_07670W_A:UTP9:DUR4:C4_00490W_A:RLP24:NMD5:C4_00690C_A:C4_00810C_A:C4_00940W_A:MDN1:DAG7:RAT1:C4_01280C_A:PWP1:NIP1:C4_01670C_A:TOA2:HGH1:C4_02850W_A:ZUO1:C4_02880C_A:C4_03040W_A:C4_03150W_A:C4_03720C_A:C4_03730C_A:C4_03740W_A:C4_03830W_A:RRP42:RAM1:C4_04090C_A:ECM1:C4_04510W_A:SSZ1:C4_04720W_A:C4_04810C_A:AGP3:NBP35:C4_05260W_A:C4_05650W_A:C4_06210C_A:C4_06410W_A:MNN4:NOP1:C4_06790W_A:MNN46:MET16:YDC1:C4_07060W_A:C4_07100C_A:C4_07140W_A:VIP1:C5_00260W_A:C5_00280C_A:DUS4:RMT2:URA4:C5_00920W_A:C5_00950C_A:BUD23:PUS4:C5_01430C_A:FYV5:SPB4:C5_01610W_A:MAS2:GIS2:C5_01930W_A:RIB2:C5_02010C_A:C5_02070C_A:RIX7:GCD11:TIF5:UTP13:C5_02730C_A:C5_03010W_A:RMS1:RPO26:FUR1:C5_03530C_

						A:C5_03550W_A:C5_03670C_A:FGR27:C5_03920C_A:C5_03970W_A:URA7:CSU57:C5_04610W_A:HAS1:C5_04990W_A:C5_05340W_A:NOP5:FET31:C6_00530C_A:C6_00640C_A:HAL21:C6_01040C_A:RCN1:CIC1:ACF2:C6_01890C_A:C6_01980C_A:C6_02230W_A:C6_02350C_A:NIP7:MRT4:POP4:EMF1:C6_03210C_A:BMT4:C6_03390W_A:FGR17:ALS5:SBA1:SHM2:SNQ2:SPB1:C6_04530C_A:C7_00160C_A:C7_00330C_A:C7_00380W_A:C7_00490C_A:RPA135:NOP15:C7_01030C_A:DBP7:GCN3:C7_01360C_A:FLU1:C7_02100W_A:C7_02340C_A:C7_02460C_A:C7_02930C_A:ZCF29:CPY1:ISY1:ENP2:C7_03990C_A:C7_04140C_A:UTP18:MIS12:CR_00460C_A:CR_00670C_A:BUD22:BMT3:PWP2:TRM1:CAB3:CR_01410C_A:CR_01700C_A:CR_01780W_A:CR_01950W_A:CR_02030C_A:CR_02420W_A:CR_02430C_A:RPC19:DBP2:RPC31:CR_02890C_A:SRP40:CR_03110W_A:CR_03230W_A:CR_03360W_A:KTI12:NCS2:YVH1:IFF3:PRP42:CR_03940W_A:GCD1:MEX67:CR_04110W_A:CR_04160C_A:CR_04170W_A:CR_04240C_A:CR_04300W_A:NHP2:ADE6:RPB8:CR_04920W_A:NOC2:CR_05550C_A:RNR3:MTG2:DBP8:VPS75:SFL1:RPL7:FGR50:CR_06680C_A:NMD3:CR_06840W_A:CR_06980W_A:CR_07030C_A:CR_07080W_A:CR_07470W_A:CR_07640C_A:DBP6:IMP4:CR_08000C_A:NOP10:SSB1:RIO2:CR_08130W_A:TRM9:HSP104:ATS1:CR_08330W_A:CYS3:GSH2:TSR1:PRP8:CR_08940W_A:CR_09110C_A:ELF1:SSF1:CR_09800C_A:SIK1:UTP5:AHA1:CR_10400W_A:CR_10410C_A:CR_10430C_A:CR_10470C_A:DRS1:POP3

6399	tRNA metabolic process	72 out of 804 genes, 9.0%	168 out of 6473 background genes, 2.6%	1.83E-20	0.00%	C1_01150C_A:C1_01160C_A:C1_01530C_A:ABP140:C1_03830C_A:RRP6:C1_05230W_A:C1_05360C_A:C1_06540C_A:LHP1:C1_09390W_A:C1_12350W_A:C1_12570C_A:HMA1:C2_01510C_A:C2_02420C_A:RPC40:KTI11:C2_06480W_A:C2_07360W_A:C2_08840W_A:C2_09500W_A:PUS7:RPC11:C3_05140C_A:C3_05800W_A:ELP3:C3_07400W_A:RIT1:C4_00810C_A:C4_00940W_A:C4_02850W_A:C4_03040W_A:C4_03730C_A:C4_03740W_A:C4_03830W_A:RRP42:C4_04810C_A:NBP35:C4_05260W_A:NOP1:C4_07060W_A:DUS4:PUS4:C5_01610W_A:RIB2:C5_02730C_A:RPO26:C5_04990W_A:C6_02350C_A:POP4:C6_03210C_A:C6_04530C_A:C7_00330C_A:C7_02340C_A:C7_03990C_A:CR_00670C_A:TRM1:RPC19:RPC31:CR_02890C_A:CR_03110W_A:KTI12:NCS2:CR_04160C_A:CR_04300W_A:RPB8:CR_05550C_A:TRM9:ATS1:CR_08940W_A:POP3

6400	tRNA modification	44 out of 804 genes, 5.5%	71 out of 6473 background genes, 1.1%	7.09E-20	0.00%	C1_01150C_A:ABP140:C1_05360C_A:C1_09390W_A:C1_12570C_A:HMA1:C2_01510C_A:C2_02420C_A:KTI11:C2_06480W_A:C2_08840W_A:C2_09500W_A:PUS7:C3_05140C_A:ELP3:C3_07400W_A:RIT1:C4_00810C_A:C4_00940W_A:C4_02850W_A:C4_03730C_A:C4_03830W_A:C4_04810C_A:NBP35:C4_05260W_A:DUS4:PUS4:C5_01610W_A:RIB2:C5_02730C_A:C6_02350C_A:C6_04530C_A:C7_00330C_A:C7_02340C_A:C7_03990C_A:CR_00670C_A:TRM1:KTI12:NCS2:CR_04160C_A:CR_04300W_A:TRM9:ATS1:CR_08940W_A

1E+05	non-membrane-bounded organelle assembly	74 out of 804 genes, 9.2%	180 out of 6473 background genes, 2.8%	9.33E-20	0.00%	RRS1:RPL6:C1_04040C_A:ERB1:NOP4:RRP6:C1_05360C_A:RPS27A:C1_06760C_A:RIA1:FUN12:JIP5:C1_09710C_A:C1_10970W_A:CSI2:C1_12440W_A:C1_12760W_A:C1_14080W_A:C2_01070W_A:C2_02540W_A:C2_02710C_A:MAK5:C2_05160C_A:RPF2:RPL11:TIF4631:C2_10740C_A:BMS1:C3_01560W_A:C3_02020W_A:C3_02040C_A:C3_02350W_A:C3_04370C_A:MAK21:C3_05160C_A:QDR2:C3_06370C_A:MDN1:C4_02880C_A:C4_05010W_A:RMT2:SPB4:C5_02070C_A:TIF5:HAS1:C5_05340W_A:CIC1:C6_02230W_A:NIP7:MOB1:SPB1:RPA135:DBP7:FLU1:ENP2:UTP18:BUD22:PWP2:CR_01410C_A:YVH1:SGD1:CR_03940W_A:CR_04170W_A:NOC2:CR_05550C_A:SDA1:CR_06680C_A:CR_07080W_A:TSR1:ELF1:SSF1:CR_09800C_A:CR_10470C_A:DRS1

8033	tRNA processing	50 out of 804 genes, 6.2%	99 out of 6473 background genes, 1.5%	2.22E-17	0.00%	C1_01150C_A:ABP140:C1_05360C_A:LHP1:C1_09390W_A:C1_12570C_A:HMA1:C2_01510C_A:C2_02420C_A:KTI11:C2_06480W_A:C2_07360W_A:C2_08840W_A:C2_09500W_A:PUS7:C3_05140C_A:C3_05800W_A:ELP3:C3_07400W_A:RIT1:C4_00810C_A:C4_00940W_A:C4_02850W_A:C4_03730C_A:C4_03830W_A:C4_04810C_A:NBP35:C4_05260W_A:NOP1:DUS4:PUS4:C5_01610W_A:RIB2:C5_02730C_A:C6_02350C_A:POP4:C6_04530C_A:C7_00330C_A:C7_02340C_A:C7_03990C_A:CR_00670C_A:TRM1:KTI12:NCS2:CR_04160C_A:CR_04300W_A:TRM9:ATS1:CR_08940W_A:POP3

70925	organelle assembly	75 out of 804 genes, 9.3%	203 out of 6473 background genes, 3.1%	9.62E-17	0.00%	RRS1:ATG17:RPL6:C1_04040C_A:ERB1:NOP4:RRP6:C1_05360C_A:RPS27A:C1_06760C_A:RIA1:FUN12:JIP5:C1_09710C_A:C1_10970W_A:CSI2:C1_12440W_A:C1_12760W_A:C1_14080W_A:C2_01070W_A:C2_02540W_A:C2_02710C_A:MAK5:C2_05160C_A:RPF2:RPL11:TIF4631:C2_10740C_A:BMS1:C3_01560W_A:C3_02020W_A:C3_02040C_A:C3_02350W_A:C3_04370C_A:MAK21:C3_05160C_A:QDR2:C3_06370C_A:MDN1:C4_02880C_A:C4_05010W_A:RMT2:SPB4:C5_02070C_A:TIF5:HAS1:C5_05340W_A:CIC1:C6_02230W_A:NIP7:MOB1:SPB1:RPA135:DBP7:FLU1:ENP2:UTP18:BUD22:PWP2:CR_01410C_A:YVH1:SGD1:CR_03940W_A:CR_04170W_A:NOC2:CR_05550C_A:SDA1:CR_06680C_A:CR_07080W_A:TSR1:ELF1:SSF1:CR_09800C_A:CR_10470C_A:DRS1

460	maturation of 5.8S rRNA	60 out of 804 genes, 7.5%	144 out of 6473 background genes, 2.2%	6.13E-16	0.00%	RRS1:C1_01160C_A:C1_03830C_A:C1_04040C_A:RRP6:C1_06540C_A:C1_06800W_A:C1_07090C_A:C1_09710C_A:DBP3:KRR1:CSI2:C1_12350W_A:CHR1:C1_14080W_A:MPP10:FAV3:C2_00410C_A:C2_02540W_A:MAK5:RPF2:LAS1:NOC4:RCL1:RRP15:BUD21:C3_01560W_A:C3_02020W_A:C3_02040C_A:C3_02350W_A:C3_05160C_A:C3_06370C_A:NSA2:DUR4:C4_00490W_A:C4_00690C_A:RAT1:C4_03740W_A:RRP42:BUD23:C5_02070C_A:UTP13:C5_03920C_A:NOP5:CIC1:ALS5:SPB1:UTP18:PWP2:CR_02420W_A:CR_03360W_A:DBP8:RPL7:CR_07080W_A:DBP6:ATS1:CR_08330W_A:PRP8:CR_09800C_A:CR_10410C_A

466	maturation of 5.8S rRNA from tricistronic rRNA transcript (SSU-rRNA, 5.8S rRNA, LSU-rRNA)	60 out of 804 genes, 7.5%	144 out of 6473 background genes, 2.2%	6.13E-16	0.00%	RRS1:C1_01160C_A:C1_03830C_A:C1_04040C_A:RRP6:C1_06540C_A:C1_06800W_A:C1_07090C_A:C1_09710C_A:DBP3:KRR1:CSI2:C1_12350W_A:CHR1:C1_14080W_A:MPP10:FAV3:C2_00410C_A:C2_02540W_A:MAK5:RPF2:LAS1:NOC4:RCL1:RRP15:BUD21:C3_01560W_A:C3_02020W_A:C3_02040C_A:C3_02350W_A:C3_05160C_A:C3_06370C_A:NSA2:DUR4:C4_00490W_A:C4_00690C_A:RAT1:C4_03740W_A:RRP42:BUD23:C5_02070C_A:UTP13:C5_03920C_A:NOP5:CIC1:ALS5:SPB1:UTP18:PWP2:CR_02420W_A:CR_03360W_A:DBP8:RPL7:CR_07080W_A:DBP6:ATS1:CR_08330W_A:PRP8:CR_09800C_A:CR_10410C_A
1510	RNA methylation	26 out of 804 genes, 3.2%	35 out of 6473 background genes, 0.5%	6.27E-14	0.00%	C1_01150C_A:ABP140:C2_06480W_A:C2_07360W_A:RRP8:C2_08840W_A:C3_05140C_A:C3_05860C_A:C3_07400W_A:C4_03730C_A:C4_03830W_A:C4_04810C_A:NOP1:BUD23:SPB1:C6_04530C_A:C7_02340C_A:CR_00670C_A:TRM1:CR_01780W_A:CR_02030C_A:CR_04160C_A:CR_04170W_A:CR_04300W_A:TRM9:CR_08940W_A

43170	macromolecule metabolic process	507 out of 804 genes, 63.1%	3194 out of 6473 background genes, 49.3%	6.49E-14	0.00%	RIM8:PHR2:HBR1:CNS1:RRS1:NMA111:HMT1:C1_01130W_A:C1_01150C_A:C1_01160C_A:C1_01470W_A:C1_01530C_A:C1_01900C_A:C1_02090C_A:ABP140:C1_02310C_A:C1_02430C_A:C1_02440C_A:C1_02450C_A:HOM6:PDE2:MIA40:ATG17:TSR2:RPL6:ARO80:MAS1:C1_03370W_A:C1_03430W_A:C1_03790C_A:C1_03830C_A:C1_04040C_A:C1_04120C_A:ERB1:RPN4:NOP4:C1_04990C_A:RHD1:RRP6:C1_05220C_A:C1_05230W_A:C1_05360C_A:RPS27A:RIB4:C1_05630C_A:FAL1:C1_05650W_A:NPL4:C1_06120C_A:C1_06540C_A:NOP6:C1_06760C_A:C1_06770W_A:MRPL19:C1_06800W_A:CAT1:C1_06910C_A:SUI2:C1_07090C_A:RIA1:YPD1:RME1:C1_07390W_A:PEX3:LHP1:C1_07510W_A:C1_07570C_A:GCS1:C1_07950C_A:FUN12:DRG1:GCD6:C1_08630W_A:MSS116:C1_09390W_A:C1_09610W_A:C1_09710C_A:DBP3:TPK1:C1_10340W_A:C1_10410W_A:C1_10620W_A:C1_10970W_A:C1_11000C_A:C1_11370C_A:KRR1:GAR1:C1_11560C_A:GAD1:C1_11900C_A:C1_11910W_A:CSI2:C1_12350W_A:PKH2:C1_12440W_A:C1_12570C_A:CHR1:C1_12670C_A:C1_12760W_A:C1_13260W_A:C1_13270W_A:KNS1:HSP70:MODF:C1_14080W_A:FTR1:C1_14160W_A:SAM35:MPP10:FAV3:NDT80:HMA1:C2_00220C_A:C2_00280C_A:C2_00410C_A:C2_00620C_A:C2_00880W_A:MXR1:C2_01060C_A:C2_01070W_A:CLB2:C2_01510C_A:C2_01870C_A:ZCF6:C2_02420C_A:PRS1:C2_02540W_A:C2_02710C_A:C2_02960C_A:LIG4:C2_03130W_A:PRP3:C2_03550C_A:MNN42:C2_03700W_A:C2_03830W_A:C2_03910C_A:SCH9:GCD7:C2_04120C_A:BAT21:C2_04570W_A:SNU114:C2_04820W_A:CRK1:C2_05080C_A:MAK5:ERF1:PEX2:C2_05160C_A:RPF2:RPC40:PEX6:C2_05520W_A:KTI11:PNG2:TES1:LAS1:CNT:ECM17:RTA2:C2_06480W_A:RPL11:C2_06850W_A:C2_06890C_A:SPE3:C2_07070W_A:RCK2:C2_07140W_A:RPA12:NOC4:C2_07360W_A:SSC1:RCL1:RNR22:C2_07610C_A:PRS5:C2_07920W_A:NSA1:C2_08200W_A:ORF298:RRP8:C2_08620W_A:TIF4631:RPT6:SPO11:C2_08840W_A:FPK1:SLP2:GLO2:PES1:RRP15:C2_09500W_A:C2_09660W_A:C2_09930W_A:FCY21:YOR1:CWC22:OCA1:PUS7:MTR2:TIF11:C2_10740C_A:RPC11:C2_10810W_A:C3_00100W_A:UTP8:BUD21:SOF1:MIH1:C3_00950C_A:BMS1:C3_01520C_A:C3_01560W_A:C3_02020W_A:C3_02030W_A:C3_02040C_A:UTP4:C3_02180C_A:C3_02350W_A:C3_03070W_A:C3_03330C_A:ULP1:HBR3:SAP9:URK1:C3_04370C_A:C3_04380C_A:MAK21:RPP2B:SFP1:CEM1:C3_05140C_A:C3_05160C_A:WOR2:SAP3:C3_05380W_A:C3_05510W_A:QDR2:C3_05790C_A:C3_05800W_A:C3_05860C_A:CTA4:NOG1:ELP3:DED1:C3_06150W_A:CYM1:HNT2:C3_06370C_A:NSA2:PRP5:C3_06760W_A:C3_06830C_A:C3_06860C_A:C3_06940W_A:NOP13:C3_07380W_A:C3_07400W_A:C3_07480W_A:C3_07550C_A:RIT1:UTP9:DUR4:C4_00490W_A:RLP24:NMD5:C4_00690C_A:C4_00700C_A:C4_00810C_A:C4_00940W_A:MDN1:DAG7:RAT1:C4_01280C_A:PWP1:C4_01470W_A:NIP1:C4_01670C_A:TOA2:HGH1:HOS3:C4_02850W_A:ZUO1:C4_02880C_A:C4_03040W_A:C4_03050C_A:C4_03150W_A:C4_03720C_A:C4_03730C_A:C4_03740W_A:C4_03830W_A:RRP42:RAM1:C4_04090C_A:ECM1:SAP10:C4_04500C_A:C4_0451

						0W_A:OMA1:SSZ1:C4_04720W_A:C4_04810C_A:AGP3:NBP35:C4_05260W_A:C4_05650W_A:C4_06210C_A:SOU1:C4_06410W_A:MNN4:NOP1:C4_06790W_A:MNN46:C4_07060W_A:C4_07100C_A:C4_07140W_A:C5_00280C_A:DUS4:RMT2:C5_00920W_A:C5_00950C_A:BUD23:PUS4:C5_01430C_A:FYV5:GLR1:SPB4:C5_01610W_A:MAS2:GIS2:C5_01930W_A:RIB2:C5_02010C_A:C5_02070C_A:RIX7:GCD11:TIF5:UTP13:C5_02730C_A:C5_03010W_A:RMS1:RPO26:FUR1:C5_03530C_A:C5_03550W_A:C5_03640W_A:C5_03670C_A:FGR27:C5_03700C_A:C5_03920C_A:C5_03970W_A:CSU57:C5_04610W_A:HAS1:C5_04990W_A:C5_05340W_A:PEX4:NOP5:FET31:C6_00530C_A:C6_00640C_A:C6_00760W_A:C6_01040C_A:RCN1:CIC1:ACF2:C6_01870C_A:C6_01890C_A:C6_01980C_A:C6_02230W_A:C6_02350C_A:NIP7:C6_02410W_A:MOB1:MRT4:POP4:EMF1:C6_03210C_A:BMT4:C6_03390W_A:FGR17:ALS5:SBA1:SHM2:SNQ2:SPB1:C6_04530C_A:C7_00160C_A:C7_00330C_A:C7_00380W_A:C7_00490C_A:RPA135:NOP15:C7_01030C_A:DBP7:GCN3:C7_01360C_A:FLU1:RAD14:C7_02100W_A:C7_02340C_A:C7_02460C_A:PEX1:C7_02930C_A:ZCF29:CPY1:ISY1:ENP2:C7_03990C_A:C7_04140C_A:UTP18:CR_00460C_A:PHO81:CR_00670C_A:BUD22:BMT3:PWP2:TRM1:CAB3:CR_01410C_A:CR_01700C_A:CR_01780W_A:CR_01950W_A:CR_02030C_A:CR_02420W_A:CR_02430C_A:CR_02460W_A:RPC19:DBP2:RPC31:CR_02890C_A:AQY1:SRP40:CR_03110W_A:CR_03230W_A:CR_03360W_A:KTI12:NCS2:YVH1:IFF3:SGD1:PRP42:CR_03760W_A:CR_03940W_A:GCD1:MEX67:CR_04110W_A:CR_04160C_A:CR_04170W_A:CR_04240C_A:CR_04300W_A:NHP2:RPB8:CR_04920W_A:YCP4:NOC2:CR_05550C_A:RNR3:MTG2:DBP8:VPS75:SFL1:SOK1:RPL7:FGR50:CR_06680C_A:NMD3:CR_06840W_A:CR_06980W_A:CR_07030C_A:CR_07080W_A:CR_07470W_A:CR_07480W_A:CR_07640C_A:DBP6:IMP4:CR_08000C_A:NOP10:SSB1:RIO2:CR_08130W_A:TRM9:HSP104:ATS1:CR_08330W_A:GSH2:TSR1:PRP8:CR_08940W_A:CR_09110C_A:CR_09140C_A:CR_09310W_A:ELF1:SSF1:CR_09800C_A:SIK1:UGA3:CR_09990W_A:UTP5:AHA1:CR_10400W_A:CR_10410C_A:CR_10430C_A:CR_10470C_A:DRS1:POP3

6807	nitrogen compound metabolic process	536 out of 804 genes, 66.7%	3438 out of 6473 background genes, 53.1%	8.69E-14	0.00%	RIM8:HBR1:C1_00510W_A:CNS1:RRS1:NMA111:HMT1:C1_01130W_A:C1_01150C_A:C1_01160C_A:C1_01470W_A:C1_01530C_A:C1_01900C_A:C1_02090C_A:ABP140:C1_02310C_A:C1_02430C_A:C1_02440C_A:C1_02450C_A:HOM6:PDE2:MIA40:ATG17:TSR2:RPL6:ARO80:MAS1:C1_03370W_A:C1_03430W_A:C1_03790C_A:C1_03830C_A:C1_04040C_A:C1_04120C_A:ERB1:RPN4:NOP4:C1_04990C_A:RHD1:RRP6:C1_05220C_A:C1_05230W_A:C1_05360C_A:RPS27A:RIB4:C1_05630C_A:FAL1:C1_05650W_A:NPL4:C1_06120C_A:C1_06540C_A:NOP6:C1_06760C_A:C1_06770W_A:MRPL19:C1_06800W_A:CAT1:C1_06910C_A:SUI2:C1_07090C_A:RIA1:YMC1:YPD1:RME1:C1_07390W_A:PEX3:LHP1:C1_07510W_A:C1_07570C_A:ADE4:GCS1:ADE5,7:C1_07950C_A:FUN12:DRG1:GCD6:C1_08630W_A:MSS116:C1_09390W_A:GUA1:C1_09610W_A:C1_09710C_A:DBP3:TPK1:C1_10340W_A:C1_10410W_A:GLY1:C1_10620W_A:HNT1:C1_10970W_A:C1_11000C_A:C1_11370C_A:KRR1:GAR1:C1_11560C_A:GAD1:C1_11900C_A:C1_11910W_A:CSI2:C1_12350W_A:PKH2:C1_12440W_A:C1_12570C_A:CHR1:C1_12670C_A:C1_12760W_A:C1_13260W_A:C1_13270W_A:C1_13330C_A:AGC1:KNS1:HSP70:MET3:MODF:C1_14080W_A:FTR1:C1_14160W_A:SAM35:MPP10:FAV3:NDT80:HMA1:C2_00220C_A:C2_00280C_A:C2_00410C_A:C2_00620C_A:C2_00880W_A:MXR1:C2_01060C_A:C2_01070W_A:CLB2:C2_01510C_A:C2_01870C_A:ZCF6:C2_02420C_A:PRS1:C2_02540W_A:SER2:C2_02710C_A:HPT1:C2_02960C_A:LIG4:ADE8:C2_03130W_A:PRP3:C2_03360W_A:C2_03550C_A:C2_03700W_A:C2_03830W_A:C2_03910C_A:SCH9:GCD7:C2_04120C_A:UGA1:BAT21:C2_04570W_A:SNU114:C2_04820W_A:CRK1:C2_05080C_A:MAK5:ERF1:PEX2:C2_05160C_A:RPF2:RPC40:C2_05520W_A:KTI11:PNG2:TES1:LAS1:CNT:ECM17:IMH3:RTA2:C2_06480W_A:AMO2:RPL11:C2_06850W_A:C2_06890C_A:SPE3:C2_07070W_A:RCK2:C2_07140W_A:RPA12:NOC4:C2_07360W_A:SSC1:RCL1:RNR22:C2_07610C_A:PRS5:C2_07920W_A:NSA1:C2_08200W_A:ORF298:C2_08460C_A:RRP8:C2_08620W_A:TIF4631:RPT6:SPO11:C2_08840W_A:FPK1:SLP2:GLO2:PES1:RRP15:C2_09500W_A:C2_09660W_A:LEU42:C2_09930W_A:FCY21:YOR1:CWC22:OCA1:PUS7:TIF11:C2_10740C_A:RPC11:C2_10810W_A:C3_00100W_A:UTP8:BUD21:SOF1:MIH1:C3_00950C_A:BMS1:URA3:C3_01520C_A:C3_01560W_A:C3_02020W_A:C3_02030W_A:C3_02040C_A:UTP4:C3_02180C_A:C3_02350W_A:PHA2:C3_03070W_A:C3_03330C_A:C3_03470W_A:ULP1:HBR3:C3_03680W_A:SAP9:AGT1:URK1:C3_04370C_A:C3_04380C_A:ADE2:MAK21:RPP2B:SFP1:CEM1:C3_05140C_A:C3_05160C_A:WOR2:SAP3:C3_05380W_A:C3_05510W_A:QDR2:BAT22:C3_05790C_A:C3_05800W_A:C3_05860C_A:CTA4:NOG1:ELP3:DED1:C3_06150W_A:CYM1:HNT2:C3_06370C_A:NSA2:PRP5:C3_06760W_A:C3_06830C_A:C3_06860C_A:C3_06940W_A:NOP13:C3_07380W_A:C3_07400W_A:C3_07480W_A:C3_07550C_A:RIT1:UTP9:CAR2:DUR4:C4_00490W_A:RLP24:NMD5:C4_00690C_A:C4_00700C_A:C4_00810C_A:C4_00940W_A:MDN1:DAG7:RAT1:C4_01280C_A:PWP1:C4_01470W_A:NIP1:C4_01670C_A:TOA2:HGH1:HOS3:C4_02850W_A:ZU

						O1:C4_02880C_A:C4_03040W_A:C4_03050C_A:C4_03150W_A:C4_03720C_A:C4_03730C_A:C4_03740W_A:C4_03830W_A:RRP42:RAM1:C4_04090C_A:ECM1:C4_04270W_A:SAP10:C4_04500C_A:C4_04510W_A:OMA1:SSZ1:C4_04720W_A:C4_04810C_A:AGP3:NBP35:C4_05260W_A:C4_05650W_A:C4_06210C_A:SOU1:C4_06410W_A:MNN4:NOP1:C4_06790W_A:MET16:YDC1:C4_07060W_A:C4_07100C_A:C4_07140W_A:C5_00260W_A:C5_00280C_A:DUS4:RMT2:URA4:C5_00920W_A:C5_00950C_A:BUD23:PUS4:C5_01430C_A:FYV5:GLR1:SPB4:C5_01610W_A:MAS2:GIS2:C5_01930W_A:RIB2:C5_02010C_A:C5_02070C_A:RIX7:GCD11:TIF5:UTP13:C5_02730C_A:C5_03010W_A:RMS1:RPO26:FUR1:C5_03530C_A:C5_03640W_A:C5_03670C_A:FGR27:C5_03700C_A:HAM1:C5_03920C_A:C5_03970W_A:URA7:CSU57:C5_04610W_A:HAS1:C5_04990W_A:C5_05340W_A:PEX4:NOP5:FET31:C6_00530C_A:C6_00640C_A:C6_00760W_A:HAL21:C6_01040C_A:RCN1:CIC1:C6_01870C_A:C6_01890C_A:C6_01980C_A:C6_02230W_A:C6_02350C_A:NIP7:C6_02410W_A:GCV1:C6_02560W_A:MOB1:MRT4:POP4:C6_03210C_A:C6_03390W_A:FGR17:ALS5:SBA1:SHM2:SNQ2:SPB1:C6_04530C_A:C7_00160C_A:C7_00330C_A:C7_00380W_A:C7_00490C_A:RPA135:NOP15:C7_01030C_A:DBP7:GCN3:C7_01360C_A:FLU1:RAD14:C7_02100W_A:C7_02340C_A:C7_02460C_A:C7_02930C_A:ZCF29:CPY1:ISY1:ENP2:C7_03990C_A:C7_04140C_A:UTP18:MIS12:CR_00460C_A:PHO81:CR_00670C_A:BUD22:BMT3:PWP2:TRM1:CAB3:CR_01410C_A:CR_01700C_A:CR_01780W_A:CR_01950W_A:CR_02030C_A:CR_02420W_A:CR_02430C_A:CR_02460W_A:RPC19:DBP2:RPC31:CR_02890C_A:AQY1:SRP40:CR_03110W_A:CR_03230W_A:CR_03360W_A:KTI12:NCS2:YVH1:IFF3:SGD1:PRP42:CR_03760W_A:CR_03940W_A:GCD1:CR_04110W_A:CR_04160C_A:CR_04170W_A:CR_04240C_A:CR_04300W_A:NHP2:ADE6:RPB8:CR_04920W_A:YCP4:NOC2:CR_05550C_A:RNR3:MTG2:DBP8:VPS75:SFL1:SOK1:RPL7:FGR50:CR_06680C_A:NMD3:CR_06840W_A:CR_06980W_A:CR_07030C_A:CR_07080W_A:CR_07470W_A:CR_07480W_A:CR_07640C_A:DBP6:IMP4:CR_08000C_A:NOP10:SSB1:RIO2:CR_08130W_A:TRM9:HSP104:ATS1:CR_08330W_A:CYS3:GSH2:TSR1:PRP8:CR_08940W_A:CR_09110C_A:CR_09140C_A:CR_09310W_A:SSF1:CR_09800C_A:SIK1:UGA3:CR_09990W_A:CR_10170C_A:UTP5:AHA1:CR_10400W_A:CR_10410C_A:CR_10430C_A:CR_10470C_A:DRS1:POP3

71704	organic substance metabolic process	571 out of 804 genes, 71.0%	3737 out of 6473 background genes, 57.7%	1.01E-13	0.00%	RIM8:PHR2:HBR1:GLO1:C1_00510W_A:CIS2:CNS1:RRS1:NMA111:HMT1:C1_01130W_A:C1_01150C_A:C1_01160C_A:C1_01470W_A:C1_01530C_A:C1_01900C_A:C1_02090C_A:ABP140:C1_02310C_A:C1_02430C_A:C1_02440C_A:C1_02450C_A:HOM6:PDE2:MIA40:ATG17:TSR2:RPL6:ARO80:MAS1:C1_03370W_A:EHT1:C1_03430W_A:C1_03790C_A:C1_03830C_A:C1_04040C_A:C1_04120C_A:ERB1:RPN4:NOP4:C1_04990C_A:RHD1:RRP6:C1_05220C_A:C1_05230W_A:C1_05360C_A:RPS27A:RIB4:C1_05630C_A:FAL1:C1_05650W_A:C1_05930C_A:NPL4:C1_06120C_A:C1_06540C_A:ANT1:NOP6:C1_06760C_A:C1_06770W_A:MRPL19:C1_06800W_A:CAT1:C1_06910C_A:SUI2:C1_07090C_A:RIA1:YMC1:YPD1:RME1:C1_07390W_A:PEX3:LHP1:C1_07510W_A:C1_07570C_A:ADE4:GCS1:ADE5,7:C1_07950C_A:FUN12:DRG1:GCD6:C1_08630W_A:MSS116:C1_09390W_A:FTH1:GUA1:C1_09610W_A:C1_09710C_A:DBP3:TPK1:C1_10340W_A:C1_10410W_A:GLY1:C1_10620W_A:HNT1:C1_10970W_A:C1_11000C_A:C1_11370C_A:KRR1:GAR1:C1_11560C_A:GAD1:C1_11900C_A:C1_11910W_A:CSI2:C1_12350W_A:PKH2:C1_12440W_A:C1_12570C_A:CHR1:C1_12670C_A:C1_12760W_A:RBK1:C1_13260W_A:C1_13270W_A:C1_13330C_A:KNS1:HSP70:MET3:MODF:C1_14080W_A:FTR1:C1_14160W_A:SAM35:MPP10:FAV3:NDT80:HMA1:C2_00220C_A:C2_00280C_A:C2_00410C_A:C2_00620C_A:C2_00880W_A:MXR1:C2_01060C_A:C2_01070W_A:CLB2:C2_01510C_A:C2_01870C_A:ZCF6:C2_01920C_A:C2_02420C_A:PRS1:C2_02540W_A:FGR22:SER2:C2_02710C_A:HPT1:C2_02960C_A:LIG4:PLC2:ADE8:C2_03130W_A:PRP3:C2_03360W_A:C2_03550C_A:MNN42:C2_03700W_A:C2_03830W_A:C2_03910C_A:SCH9:GCD7:C2_04120C_A:UGA1:BAT21:C2_04570W_A:SNU114:C2_04820W_A:CRK1:C2_05080C_A:MAK5:ERF1:PEX2:C2_05160C_A:RPF2:RPC40:PEX6:C2_05520W_A:KTI11:PNG2:TES1:LAS1:CNT:ECM17:IMH3:RTA2:C2_06480W_A:AMO2:RPL11:C2_06850W_A:C2_06890C_A:SPE3:C2_07070W_A:RCK2:C2_07140W_A:RPA12:NOC4:C2_07360W_A:SSC1:C2_07410W_A:RCL1:RNR22:C2_07610C_A:PRS5:C2_07920W_A:NSA1:C2_08200W_A:ORF298:C2_08460C_A:RRP8:C2_08620W_A:TIF4631:RPT6:SPO11:C2_08840W_A:FPK1:SLP2:GLO2:PES1:RRP15:C2_09500W_A:C2_09660W_A:LEU42:C2_09930W_A:FCY21:YOR1:CWC22:OCA1:GPD1:PUS7:MTR2:TIF11:C2_10740C_A:RPC11:C2_10810W_A:C3_00100W_A:UTP8:BUD21:SOF1:MIH1:C3_00950C_A:BMS1:URA3:C3_01520C_A:C3_01560W_A:C3_02020W_A:C3_02030W_A:C3_02040C_A:UTP4:C3_02180C_A:C3_02350W_A:PHA2:C3_03070W_A:C3_03330C_A:C3_03470W_A:ULP1:HBR3:C3_03680W_A:SAP9:AGT1:URK1:C3_04370C_A:C3_04380C_A:ADE2:MAK21:RPP2B:SFP1:CEM1:C3_05140C_A:C3_05160C_A:WOR2:SAP3:C3_05380W_A:C3_05510W_A:QDR2:BAT22:C3_05790C_A:C3_05800W_A:C3_05860C_A:CTA4:NOG1:ELP3:DED1:C3_06150W_A:CYM1:HNT2:C3_06370C_A:NSA2:PRP5:C3_06760W_A:C3_06830C_A:C3_06860C_A:C3_06940W_A:NOP13:GCY1:C3_07380W_A:C3_07400W_A:C3_07480W_A:C3_07550C_A:RIT1:C3_07670W_A:UTP9:CAR2:DUR4:C4_00490W_A:RLP24:NMD5:C4_00690C_A:C4_00700C_A:C4_0

						0810C_A:C4_00940W_A:MDN1:DAG7:RAT1:C4_01280C_A:PWP1:C4_01470W_A:NIP1:C4_01670C_A:TOA2:HGH1:HOS3:C4_02620C_A:C4_02850W_A:ZUO1:C4_02880C_A:C4_03040W_A:C4_03050C_A:C4_03150W_A:C4_03720C_A:C4_03730C_A:C4_03740W_A:C4_03830W_A:RRP42:RAM1:C4_04090C_A:ECM1:C4_04270W_A:SAP10:C4_04500C_A:C4_04510W_A:OMA1:SSZ1:C4_04720W_A:C4_04810C_A:AGP3:NBP35:C4_05260W_A:C4_05650W_A:C4_06210C_A:SOU1:C4_06410W_A:MNN4:NOP1:C4_06790W_A:MNN46:MET16:YDC1:C4_07060W_A:C4_07100C_A:C4_07140W_A:VIP1:C5_00260W_A:C5_00280C_A:DUS4:RMT2:URA4:C5_00920W_A:C5_00950C_A:BUD23:PUS4:C5_01430C_A:FYV5:GLR1:SPB4:C5_01610W_A:MAS2:GIS2:C5_01930W_A:RIB2:C5_02010C_A:C5_02070C_A:RIX7:GCD11:TIF5:UTP13:C5_02730C_A:C5_03010W_A:RMS1:RPO26:FUR1:C5_03530C_A:C5_03550W_A:C5_03640W_A:C5_03670C_A:FGR27:C5_03700C_A:HAM1:C5_03920C_A:C5_03970W_A:URA7:CSU57:C5_04610W_A:HAS1:C5_04990W_A:C5_05340W_A:PEX4:NOP5:FET31:C6_00530C_A:C6_00640C_A:C6_00760W_A:HAL21:C6_01040C_A:RCN1:CIC1:EBP1:C6_01300W_A:ACF2:C6_01870C_A:C6_01890C_A:C6_01980C_A:C6_02230W_A:C6_02350C_A:NIP7:C6_02410W_A:C6_02420W_A:C6_02480W_A:GCV1:C6_02560W_A:MOB1:MRT4:POP4:EMF1:C6_03210C_A:BMT4:C6_03390W_A:FGR17:PAD1:ALS5:SBA1:SHM2:SNQ2:SPB1:C6_04530C_A:NAG1:C7_00160C_A:C7_00330C_A:C7_00380W_A:C7_00490C_A:RPA135:NOP15:C7_01030C_A:DBP7:GCN3:C7_01360C_A:FLU1:RAD14:C7_02100W_A:YCF1:C7_02340C_A:C7_02460C_A:PEX1:C7_02930C_A:ZCF29:CPY1:ISY1:ENP2:C7_03990C_A:C7_04140C_A:UTP18:MIS12:CR_00460C_A:PHO81:CR_00670C_A:BUD22:BMT3:PWP2:TRM1:CAB3:CR_01410C_A:CR_01700C_A:CR_01780W_A:CR_01950W_A:CR_02030C_A:CR_02420W_A:CR_02430C_A:CR_02460W_A:RPC19:DBP2:RPC31:CR_02890C_A:AQY1:SRP40:CR_03110W_A:CR_03230W_A:CR_03360W_A:KTI12:NCS2:YVH1:IFF3:SGD1:PRP42:CR_03760W_A:CR_03940W_A:GCD1:MEX67:CR_04110W_A:CR_04160C_A:CR_04170W_A:CR_04240C_A:CR_04300W_A:NHP2:ADE6:RPB8:CR_04920W_A:YCP4:NOC2:CR_05550C_A:RNR3:MTG2:DBP8:VPS75:SFL1:SOK1:RPL7:FGR50:CR_06680C_A:NMD3:CR_06840W_A:CR_06980W_A:CR_07030C_A:CR_07080W_A:CR_07470W_A:CR_07480W_A:CR_07640C_A:DBP6:IMP4:CR_08000C_A:NOP10:SSB1:RIO2:CR_08130W_A:TRM9:HSP104:ATS1:CR_08330W_A:CYS3:GSH2:TSR1:PRP8:CR_08940W_A:CR_09110C_A:CR_09140C_A:CR_09310W_A:ELF1:CR_09670C_A:SSF1:CR_09800C_A:SIK1:UGA3:CR_09990W_A:CR_10170C_A:UTP5:AHA1:CR_10400W_A:CR_10410C_A:CR_10430C_A:CR_10470C_A:DRS1:POP3

43414	macromolecule methylation	39 out of 804 genes, 4.9%	77 out of 6473 background genes, 1.2%	3.56E-13	0.00%	HMT1:C1_01150C_A:ABP140:C1_14160W_A:C2_00620C_A:C2_02960C_A:C2_03130W_A:C2_05520W_A:C2_06480W_A:C2_07360W_A:RRP8:C2_08840W_A:C3_05140C_A:C3_05860C_A:C3_07400W_A:C4_03730C_A:C4_03830W_A:C4_04500C_A:C4_04810C_A:NOP1:RMT2:C5_00950C_A:BUD23:RMS1:SPB1:C6_04530C_A:C7_00490C_A:C7_02340C_A:CR_00670C_A:TRM1:CR_01780W_A:CR_02030C_A:CR_03760W_A:CR_04160C_A:CR_04170W_A:CR_04300W_A:TRM9:CR_08940W_A:CR_09310W_A
32259	methylation	39 out of 804 genes, 4.9%	78 out of 6473 background genes, 1.2%	6.30E-13	0.00%	HMT1:C1_01150C_A:ABP140:C1_14160W_A:C2_00620C_A:C2_02960C_A:C2_03130W_A:C2_05520W_A:C2_06480W_A:C2_07360W_A:RRP8:C2_08840W_A:C3_05140C_A:C3_05860C_A:C3_07400W_A:C4_03730C_A:C4_03830W_A:C4_04500C_A:C4_04810C_A:NOP1:RMT2:C5_00950C_A:BUD23:RMS1:SPB1:C6_04530C_A:C7_00490C_A:C7_02340C_A:CR_00670C_A:TRM1:CR_01780W_A:CR_02030C_A:CR_03760W_A:CR_04160C_A:CR_04170W_A:CR_04300W_A:TRM9:CR_08940W_A:CR_09310W_A

44238	primary metabolic process	550 out of 804 genes, 68.4%	3618 out of 6473 background genes, 55.9%	9.25E-12	0.00%	RIM8:PHR2:HBR1:CNS1:RRS1:NMA111:HMT1:C1_01130W_A:C1_01150C_A:C1_01160C_A:C1_01470W_A:C1_01530C_A:C1_01900C_A:C1_02090C_A:ABP140:C1_02310C_A:C1_02430C_A:C1_02440C_A:C1_02450C_A:HOM6:PDE2:MIA40:ATG17:TSR2:RPL6:ARO80:MAS1:C1_03370W_A:EHT1:C1_03430W_A:C1_03790C_A:C1_03830C_A:C1_04040C_A:C1_04120C_A:ERB1:RPN4:NOP4:C1_04990C_A:RHD1:RRP6:C1_05220C_A:C1_05230W_A:C1_05360C_A:RPS27A:RIB4:C1_05630C_A:FAL1:C1_05650W_A:NPL4:C1_06120C_A:C1_06540C_A:ANT1:NOP6:C1_06760C_A:C1_06770W_A:MRPL19:C1_06800W_A:CAT1:C1_06910C_A:SUI2:C1_07090C_A:RIA1:YPD1:RME1:C1_07390W_A:PEX3:LHP1:C1_07510W_A:C1_07570C_A:ADE4:GCS1:ADE5,7:C1_07950C_A:FUN12:DRG1:GCD6:C1_08630W_A:MSS116:C1_09390W_A:FTH1:GUA1:C1_09610W_A:C1_09710C_A:DBP3:TPK1:C1_10340W_A:C1_10410W_A:GLY1:C1_10620W_A:HNT1:C1_10970W_A:C1_11000C_A:C1_11370C_A:KRR1:GAR1:C1_11560C_A:GAD1:C1_11900C_A:C1_11910W_A:CSI2:C1_12350W_A:PKH2:C1_12440W_A:C1_12570C_A:CHR1:C1_12670C_A:C1_12760W_A:RBK1:C1_13260W_A:C1_13270W_A:KNS1:HSP70:MODF:C1_14080W_A:FTR1:C1_14160W_A:SAM35:MPP10:FAV3:NDT80:HMA1:C2_00220C_A:C2_00280C_A:C2_00410C_A:C2_00620C_A:C2_00880W_A:MXR1:C2_01060C_A:C2_01070W_A:CLB2:C2_01510C_A:C2_01870C_A:ZCF6:C2_01920C_A:C2_02420C_A:PRS1:C2_02540W_A:FGR22:SER2:C2_02710C_A:HPT1:C2_02960C_A:LIG4:PLC2:ADE8:C2_03130W_A:PRP3:C2_03360W_A:C2_03550C_A:MNN42:C2_03700W_A:C2_03830W_A:C2_03910C_A:SCH9:GCD7:C2_04120C_A:UGA1:BAT21:C2_04570W_A:SNU114:C2_04820W_A:CRK1:C2_05080C_A:MAK5:ERF1:PEX2:C2_05160C_A:RPF2:RPC40:PEX6:C2_05520W_A:KTI11:PNG2:TES1:LAS1:CNT:ECM17:IMH3:RTA2:C2_06480W_A:RPL11:C2_06850W_A:C2_06890C_A:SPE3:C2_07070W_A:RCK2:C2_07140W_A:RPA12:NOC4:C2_07360W_A:SSC1:C2_07410W_A:RCL1:RNR22:C2_07610C_A:PRS5:C2_07920W_A:NSA1:C2_08200W_A:ORF298:C2_08460C_A:RRP8:C2_08620W_A:TIF4631:RPT6:SPO11:C2_08840W_A:FPK1:SLP2:GLO2:PES1:RRP15:C2_09500W_A:C2_09660W_A:LEU42:C2_09930W_A:FCY21:YOR1:CWC22:OCA1:GPD1:PUS7:TIF11:C2_10740C_A:RPC11:C2_10810W_A:C3_00100W_A:UTP8:BUD21:SOF1:MIH1:C3_00950C_A:BMS1:URA3:C3_01520C_A:C3_01560W_A:C3_02020W_A:C3_02030W_A:C3_02040C_A:UTP4:C3_02180C_A:C3_02350W_A:PHA2:C3_03070W_A:C3_03330C_A:C3_03470W_A:ULP1:HBR3:C3_03680W_A:SAP9:URK1:C3_04370C_A:C3_04380C_A:ADE2:MAK21:RPP2B:SFP1:CEM1:C3_05140C_A:C3_05160C_A:WOR2:SAP3:C3_05380W_A:C3_05510W_A:QDR2:BAT22:C3_05790C_A:C3_05800W_A:C3_05860C_A:CTA4:NOG1:ELP3:DED1:C3_06150W_A:CYM1:HNT2:C3_06370C_A:NSA2:PRP5:C3_06760W_A:C3_06830C_A:C3_06860C_A:C3_06940W_A:NOP13:GCY1:C3_07380W_A:C3_07400W_A:C3_07480W_A:C3_07550C_A:RIT1:C3_07670W_A:UTP9:CAR2:DUR4:C4_00490W_A:RLP24:NMD5:C4_00690C_A:C4_00700C_A:C4_00810C_A:C4_00940W_A:MDN1:DAG7:RAT1:C4_01280C_A:PWP1:C4_01470W_A:NI

						P1:C4_01670C_A:TOA2:HGH1:HOS3:C4_02620C_A:C4_02850W_A:ZUO1:C4_02880C_A:C4_03040W_A:C4_03050C_A:C4_03150W_A:C4_03720C_A:C4_03730C_A:C4_03740W_A:C4_03830W_A:RRP42:RAM1:C4_04090C_A:ECM1:SAP10:C4_04500C_A:C4_04510W_A:OMA1:SSZ1:C4_04720W_A:C4_04810C_A:AGP3:NBP35:C4_05260W_A:C4_05650W_A:C4_06210C_A:SOU1:C4_06410W_A:MNN4:NOP1:C4_06790W_A:MNN46:MET16:YDC1:C4_07060W_A:C4_07100C_A:C4_07140W_A:C5_00260W_A:C5_00280C_A:DUS4:RMT2:URA4:C5_00920W_A:C5_00950C_A:BUD23:PUS4:C5_01430C_A:FYV5:GLR1:SPB4:C5_01610W_A:MAS2:GIS2:C5_01930W_A:RIB2:C5_02010C_A:C5_02070C_A:RIX7:GCD11:TIF5:UTP13:C5_02730C_A:C5_03010W_A:RMS1:RPO26:FUR1:C5_03530C_A:C5_03640W_A:C5_03670C_A:FGR27:C5_03700C_A:HAM1:C5_03920C_A:C5_03970W_A:URA7:CSU57:C5_04610W_A:HAS1:C5_04990W_A:C5_05340W_A:PEX4:NOP5:FET31:C6_00530C_A:C6_00640C_A:C6_00760W_A:HAL21:C6_01040C_A:RCN1:CIC1:EBP1:C6_01300W_A:ACF2:C6_01870C_A:C6_01890C_A:C6_01980C_A:C6_02230W_A:C6_02350C_A:NIP7:C6_02410W_A:C6_02420W_A:GCV1:C6_02560W_A:MOB1:MRT4:POP4:C6_03210C_A:BMT4:C6_03390W_A:FGR17:ALS5:SBA1:SHM2:SNQ2:SPB1:C6_04530C_A:NAG1:C7_00160C_A:C7_00330C_A:C7_00380W_A:C7_00490C_A:RPA135:NOP15:C7_01030C_A:DBP7:GCN3:C7_01360C_A:FLU1:RAD14:C7_02100W_A:C7_02340C_A:C7_02460C_A:PEX1:C7_02930C_A:ZCF29:CPY1:ISY1:ENP2:C7_03990C_A:C7_04140C_A:UTP18:CR_00460C_A:PHO81:CR_00670C_A:BUD22:BMT3:PWP2:TRM1:CAB3:CR_01410C_A:CR_01700C_A:CR_01780W_A:CR_01950W_A:CR_02030C_A:CR_02420W_A:CR_02430C_A:CR_02460W_A:RPC19:DBP2:RPC31:CR_02890C_A:AQY1:SRP40:CR_03110W_A:CR_03230W_A:CR_03360W_A:KTI12:NCS2:YVH1:IFF3:SGD1:PRP42:CR_03760W_A:CR_03940W_A:GCD1:CR_04110W_A:CR_04160C_A:CR_04170W_A:CR_04240C_A:CR_04300W_A:NHP2:ADE6:RPB8:CR_04920W_A:YCP4:NOC2:CR_05550C_A:RNR3:MTG2:DBP8:VPS75:SFL1:SOK1:RPL7:FGR50:CR_06680C_A:NMD3:CR_06840W_A:CR_06980W_A:CR_07030C_A:CR_07080W_A:CR_07470W_A:CR_07480W_A:CR_07640C_A:DBP6:IMP4:CR_08000C_A:NOP10:SSB1:RIO2:CR_08130W_A:TRM9:HSP104:ATS1:CR_08330W_A:CYS3:GSH2:TSR1:PRP8:CR_08940W_A:CR_09110C_A:CR_09140C_A:CR_09310W_A:SSF1:CR_09800C_A:SIK1:UGA3:CR_09990W_A:CR_10170C_A:UTP5:AHA1:CR_10400W_A:CR_10410C_A:CR_10430C_A:CR_10470C_A:DRS1:POP3

71840	cellular component organization or biogenesis	373 out of 804 genes, 46.4%	2243 out of 6473 background genes, 34.7%	1.84E-10	0.00%	PHR2:HBR1:CNS1:RRS1:CCH1:C1_01160C_A:C1_01470W_A:C1_01900C_A:C1_02090C_A:ABP140:C1_02310C_A:C1_02450C_A:PDE2:MIA40:ATG17:TSR2:RPL6:ARO80:ARX1:MAS1:C1_03790C_A:C1_03830C_A:C1_04040C_A:C1_04120C_A:ERB1:C1_04340C_A:NOP4:RRP6:C1_05210C_A:C1_05220C_A:C1_05230W_A:C1_05360C_A:RPS27A:FAL1:C1_05650W_A:NPL4:C1_06540C_A:ANT1:NOP6:C1_06760C_A:C1_06770W_A:C1_06800W_A:SUI2:C1_07090C_A:RIA1:YPD1:PEX3:C1_07510W_A:C1_07570C_A:C1_07950C_A:FUN12:GCD6:C1_08630W_A:MSS116:JIP5:C1_09060C_A:C1_09390W_A:YTM1:C1_09710C_A:DBP3:TPK1:C1_10620W_A:C1_10970W_A:C1_11000C_A:C1_11010C_A:TIM50:C1_11370C_A:KRR1:GAR1:C1_11560C_A:C1_11900C_A:C1_11910W_A:CSI2:C1_12350W_A:C1_12440W_A:C1_12570C_A:CHR1:C1_12680W_A:C1_12760W_A:C1_13270W_A:C1_13370W_A:AGC1:KNS1:SIM1:C1_14080W_A:SAM35:MPP10:FAV3:NDT80:C2_00280C_A:C2_00410C_A:C2_00810C_A:C2_01070W_A:CLB2:C2_01510C_A:C2_02420C_A:PRS1:C2_02540W_A:C2_02710C_A:PRP3:MNN42:SCH9:C2_04120C_A:C2_04570W_A:SNU114:C2_04820W_A:C2_05080C_A:MAK5:ERF1:PEX2:C2_05160C_A:RPF2:PEX6:C2_05520W_A:TES1:LAS1:CNT:ECM17:RTA3:RTA2:RPL11:C2_06850W_A:RCK2:NOC4:C2_07360W_A:SSC1:RCL1:PRS5:C2_07920W_A:NSA1:KRE30:RRP8:TIF4631:RPT6:SPO11:C2_08840W_A:FPK1:C2_09070C_A:PES1:RRP15:C2_09660W_A:TIM23:CWC22:OCA1:GPD1:PUS7:MTR2:TIF11:C2_10740C_A:C2_10810W_A:C3_00100W_A:UTP8:BUD21:SOF1:C3_00950C_A:BMS1:C3_01560W_A:C3_02020W_A:C3_02040C_A:UTP4:C3_02350W_A:C3_03070W_A:C3_03330C_A:C3_03470W_A:HBR3:SAP9:URK1:C3_04370C_A:C3_04380C_A:MAK21:SFP1:CEM1:C3_05160C_A:WOR2:C3_05380W_A:QDR2:C3_05800W_A:C3_05860C_A:NOG1:DED1:C3_06150W_A:C3_06370C_A:NSA2:PRP5:C3_06760W_A:C3_07100C_A:NOP13:C3_07480W_A:C3_07550C_A:UTP9:DUR4:C4_00490W_A:RLP24:NMD5:C4_00690C_A:C4_00700C_A:C4_00810C_A:MDN1:RAT1:C4_01130C_A:C4_01280C_A:PWP1:C4_01670C_A:TOA2:C4_02720C_A:ZUO1:C4_02880C_A:C4_03040W_A:DNF3:C4_03150W_A:C4_03720C_A:C4_03740W_A:C4_03830W_A:RRP42:RAM1:ECM1:SAP10:C4_04510W_A:OMA1:SSZ1:AGP3:C4_05010W_A:MYO5:NBP35:C4_05260W_A:C4_05650W_A:C4_06210C_A:C4_06410W_A:NOP1:C4_06790W_A:MNN46:C4_07060W_A:C4_07100C_A:C4_07140W_A:C5_00260W_A:C5_00280C_A:RMT2:C5_00920W_A:C5_00950C_A:BUD23:PUS4:C5_01430C_A:FYV5:SPB4:MAS2:C5_01930W_A:RIB2:C5_02010C_A:C5_02070C_A:RIX7:GCD11:TIF5:UTP13:C5_03010W_A:RMS1:C5_03550W_A:C5_03670C_A:FGR27:C5_03920C_A:C5_03970W_A:C5_04610W_A:HAS1:C5_04990W_A:C5_05340W_A:PEX4:NOP5:C6_01040C_A:RCN1:CIC1:ACF2:C6_01890C_A:C6_02230W_A:NIP7:MOB1:SAC6:MRT4:POP4:C6_03210C_A:C6_03390W_A:ALS5:SNQ2:SPB1:C7_00160C_A:C7_00330C_A:RPA135:NOP15:C7_01030C_A:DBP7:C7_01360C_A:FLU1:RAD14:C7_02100W_A:FMP45:YCF1:C7_02460C_A:PEX1:C7_02930C_A:ISY1:ENP2:MAM33:C7_04140C_A:UTP18:CR_00460C_A:BUD22:BMT3:PWP2:CR_0

						1410C_A:CR_01700C_A:CR_01780W_A:CR_02030C_A:CR_02420W_A:RPC19:DBP2:CR_02890C_A:SRP40:CR_03230W_A:CR_03360W_A:KTI12:NCS2:YVH1:IFF3:SGD1:PRP42:CR_03760W_A:CR_03940W_A:MEX67:CR_04110W_A:CR_04170W_A:CR_04240C_A:NHP2:RBR2:CR_04920W_A:NOC2:CR_05550C_A:RNR3:MTG2:DBP8:SDA1:VPS75:SFL1:RPL7:FGR50:RGS2:CR_06680C_A:NMD3:CR_06840W_A:CR_07030C_A:CR_07080W_A:CR_07640C_A:DBP6:IMP4:CR_08000C_A:NOP10:SSB1:RIO2:CR_08130W_A:HSP104:ATS1:CR_08330W_A:TSR1:CR_08500W_A:PRP8:CR_09110C_A:CR_09140C_A:ELF1:SSF1:CR_09800C_A:SIK1:UTP5:CR_10410C_A:CR_10470C_A:DRS1:POP3
30488	tRNA methylation	17 out of 804 genes, 2.1%	21 out of 6473 background genes, 0.3%	1.73E-09	0.00%	C1_01150C_A:ABP140:C2_06480W_A:C2_08840W_A:C3_05140C_A:C3_07400W_A:C4_03730C_A:C4_03830W_A:C4_04810C_A:C6_04530C_A:C7_02340C_A:CR_00670C_A:TRM1:CR_04160C_A:CR_04300W_A:TRM9:CR_08940W_A

44237	cellular metabolic process	544 out of 804 genes, 67.7%	3715 out of 6473 background genes, 57.4%	1.68E-07	0.00%	RIM8:PHR2:HBR1:GLO1:C1_00510W_A:C1_00520W_A:CIS2:CNS1:RRS1:NMA111:HMT1:C1_01150C_A:C1_01160C_A:C1_01470W_A:SOD2:C1_01530C_A:C1_01900C_A:C1_02090C_A:ABP140:C1_02310C_A:C1_02430C_A:C1_02440C_A:C1_02450C_A:HOM6:PDE2:MIA40:ATG17:TSR2:RPL6:ARO80:MAS1:C1_03370W_A:EHT1:C1_03430W_A:C1_03790C_A:C1_03830C_A:C1_04040C_A:C1_04120C_A:ERB1:RPN4:NOP4:C1_04990C_A:RHD1:RRP6:C1_05220C_A:C1_05230W_A:C1_05360C_A:RPS27A:RIB4:C1_05630C_A:FAL1:C1_05650W_A:NPL4:C1_06120C_A:C1_06540C_A:ANT1:NOP6:C1_06760C_A:C1_06770W_A:MRPL19:C1_06800W_A:CAT1:C1_06910C_A:SUI2:C1_07090C_A:RIA1:YMC1:YPD1:PEX3:LHP1:C1_07510W_A:C1_07570C_A:ADE4:GCS1:ADE5,7:C1_07950C_A:FUN12:DRG1:GCD6:C1_08630W_A:MSS116:C1_09060C_A:C1_09390W_A:FTH1:C1_09440W_A:GUA1:C1_09610W_A:C1_09710C_A:DBP3:TPK1:C1_10340W_A:GLY1:C1_10620W_A:HNT1:C1_10970W_A:C1_11000C_A:C1_11010C_A:C1_11370C_A:KRR1:GAR1:C1_11560C_A:GAD1:MRF1:C1_11900C_A:C1_11910W_A:CSI2:C1_12350W_A:PKH2:C1_12440W_A:C1_12570C_A:CHR1:C1_12670C_A:C1_12760W_A:C1_13260W_A:C1_13270W_A:C1_13330C_A:AGC1:KNS1:HSP70:MET3:MODF:SIM1:C1_14080W_A:FTR1:MPP10:FAV3:NDT80:HMA1:C2_00220C_A:C2_00280C_A:C2_00410C_A:C2_00810C_A:C2_00880W_A:MXR1:C2_01070W_A:C2_01510C_A:C2_01870C_A:ZCF6:C2_01920C_A:C2_02420C_A:PRS1:C2_02540W_A:SER2:C2_02710C_A:HPT1:LIG4:ADE8:PRP3:C2_03360W_A:C2_03550C_A:MNN42:C2_03910C_A:SCH9:GCD7:C2_04120C_A:UGA1:BAT21:C2_04570W_A:SNU114:C2_04820W_A:CRK1:C2_05080C_A:MAK5:ERF1:PEX2:C2_05160C_A:RPF2:RPC40:PEX6:C2_05520W_A:KTI11:TES1:LAS1:CNT:ECM17:IMH3:RTA2:C2_06480W_A:RPL11:C2_06850W_A:SPE3:RCK2:C2_07140W_A:RPA12:NOC4:C2_07360W_A:SSC1:C2_07410W_A:RCL1:RNR22:C2_07610C_A:PRS5:C2_07920W_A:NSA1:ORF298:C2_08460C_A:RRP8:TIF4631:RPT6:SPO11:C2_08840W_A:FPK1:C2_09070C_A:MET10:SLP2:GLO2:PES1:RRP15:C2_09500W_A:C2_09660W_A:LEU42:FCY21:YOR1:CWC22:OCA1:GPD1:PUS7:MTR2:TIF11:C2_10740C_A:RPC11:C2_10810W_A:C3_00100W_A:UTP8:BUD21:DOT5:SOF1:MIH1:C3_00950C_A:BMS1:URA3:C3_01520C_A:C3_01560W_A:C3_02020W_A:C3_02030W_A:C3_02040C_A:UTP4:C3_02180C_A:C3_02350W_A:PHA2:C3_03070W_A:C3_03330C_A:BMT7:C3_03470W_A:HBR3:C3_03680W_A:SAP9:CSC25:AGT1:URK1:C3_04370C_A:C3_04380C_A:ADE2:MAK21:RPP2B:SFP1:CEM1:C3_05140C_A:C3_05160C_A:WOR2:SAP3:C3_05380W_A:C3_05510W_A:QDR2:BAT22:C3_05800W_A:C3_05860C_A:CTA4:NOG1:ELP3:DED1:C3_06150W_A:HNT2:C3_06370C_A:NSA2:PRP5:C3_06760W_A:C3_06830C_A:C3_06940W_A:C3_07100C_A:NOP13:C3_07400W_A:C3_07430W_A:C3_07480W_A:C3_07550C_A:RIT1:C3_07670W_A:UTP9:CAR2:DUR4:C4_00490W_A:RLP24:NMD5:C4_00690C_A:C4_00700C_A:C4_00810C_A:C4_00940W_A:MDN1:DAG7:RAT1:C4_01280C_A:PWP1:NIP1:C4_01670C_A:TOA2:HGH1:C4_02850W_A:ZUO1:C4_02880C_A:C4_03040W_A:C4_03150W_A:C4_03720C_A:C

						4_03730C_A:C4_03740W_A:C4_03830W_A:RRP42:RAM1:C4_04090C_A:ECM1:C4_04270W_A:C4_04510W_A:SSZ1:C4_04720W_A:C4_04810C_A:AGP3:MYO5:NBP35:C4_05260W_A:C4_05650W_A:C4_06210C_A:C4_06410W_A:MNN4:NOP1:C4_06790W_A:MNN46:MET16:YDC1:C4_07060W_A:C4_07100C_A:C4_07140W_A:VIP1:C5_00260W_A:C5_00280C_A:DUS4:RMT2:URA4:C5_00920W_A:C5_00950C_A:BUD23:PUS4:C5_01430C_A:FYV5:SPB4:C5_01610W_A:MAS2:GIS2:C5_01930W_A:RIB2:C5_02010C_A:C5_02070C_A:RIX7:GCD11:ZCF22:TIF5:UTP13:C5_02730C_A:C5_03010W_A:RMS1:RPO26:FUR1:C5_03530C_A:C5_03550W_A:C5_03670C_A:FGR27:HAM1:C5_03920C_A:C5_03970W_A:URA7:CSU57:C5_04610W_A:HAS1:C5_04990W_A:PCL5:C5_05340W_A:NOP5:FET31:C6_00530C_A:C6_00640C_A:HAL21:AEP1:C6_01040C_A:RCN1:CIC1:C6_01300W_A:ACF2:C6_01890C_A:C6_01980C_A:C6_02230W_A:C6_02350C_A:NIP7:C6_02410W_A:C6_02420W_A:GCV1:C6_02560W_A:MOB1:SAC6:MRT4:POP4:EMF1:C6_03210C_A:BMT4:C6_03330C_A:C6_03390W_A:FGR17:PAD1:ALS5:SBA1:SHM2:SNQ2:SPB1:C6_04530C_A:C7_00160C_A:C7_00330C_A:C7_00380W_A:C7_00490C_A:RPA135:NOP15:C7_01030C_A:DBP7:GCN3:C7_01360C_A:FLU1:RAD14:C7_02100W_A:C7_02340C_A:C7_02460C_A:PEX1:C7_02930C_A:ZCF29:CPY1:ISY1:ENP2:MAM33:C7_03990C_A:C7_04140C_A:UTP18:MIS12:CR_00460C_A:PHO81:CR_00670C_A:BUD22:BMT3:PWP2:TRM1:CAB3:CR_01410C_A:CR_01700C_A:CR_01780W_A:CR_01950W_A:CR_02030C_A:CR_02420W_A:CR_02430C_A:CR_02460W_A:CR_02510W_A:RPC19:DBP2:RPC31:CR_02890C_A:SRP40:CR_03110W_A:CR_03230W_A:CR_03360W_A:KTI12:NCS2:YVH1:IFF3:SGD1:PRP42:CR_03940W_A:GCD1:MEX67:CR_04110W_A:CR_04160C_A:CR_04170W_A:CR_04240C_A:CR_04300W_A:NHP2:ADE6:RPB8:CR_04920W_A:NOC2:CR_05550C_A:RNR3:MTG2:DBP8:VPS75:SFL1:SOK1:RPL7:FGR50:RGS2:CR_06680C_A:NMD3:CR_06840W_A:CR_06980W_A:CR_07030C_A:CR_07080W_A:CR_07470W_A:CR_07640C_A:DBP6:CR_07780W_A:IMP4:CR_08000C_A:NOP10:SSB1:RIO2:CR_08130W_A:TRM9:HSP104:ATS1:CR_08330W_A:CYS3:GSH2:TSR1:PRP8:CR_08940W_A:CR_09110C_A:CR_09140C_A:ELF1:CR_09670C_A:SSF1:CR_09800C_A:SIK1:UGA3:CR_09990W_A:CR_10170C_A:UTP5:AHA1:CR_10400W_A:CR_10410C_A:CR_10430C_A:CR_10470C_A:DRS1:POP3

65003	protein-containing complex assembly	101 out of 804 genes, 12.6%	444 out of 6473 background genes, 6.9%	3.24E-07	0.00%	RPL6:C1_03790C_A:C1_04040C_A:C1_04120C_A:ERB1:NOP4:RRP6:C1_05360C_A:RPS27A:C1_06760C_A:SUI2:FUN12:JIP5:C1_09710C_A:C1_10970W_A:CSI2:C1_12440W_A:C1_12760W_A:C1_14080W_A:SAM35:C2_00280C_A:C2_01070W_A:C2_02540W_A:C2_02710C_A:PRP3:SNU114:MAK5:C2_05160C_A:RPF2:RPL11:RPT6:TIF11:C2_10740C_A:C3_00100W_A:BMS1:C3_01560W_A:C3_02020W_A:C3_02040C_A:C3_02350W_A:C3_03330C_A:HBR3:C3_04370C_A:MAK21:C3_05160C_A:QDR2:NOG1:C3_06370C_A:PRP5:C3_07480W_A:RLP24:MDN1:TOA2:C4_03150W_A:C4_05010W_A:C4_06410W_A:C4_07100C_A:RMT2:SPB4:C5_01930W_A:C5_02010C_A:C5_02070C_A:GCD11:TIF5:HAS1:C5_05340W_A:CIC1:C6_02230W_A:NIP7:MOB1:SPB1:RPA135:DBP7:FLU1:ISY1:ENP2:UTP18:BUD22:PWP2:CR_01410C_A:CR_01700C_A:DBP2:YVH1:SGD1:CR_03940W_A:CR_04110W_A:CR_04170W_A:NOC2:CR_05550C_A:SDA1:VPS75:CR_06680C_A:CR_07080W_A:CR_08130W_A:TSR1:PRP8:ELF1:SSF1:CR_09800C_A:CR_10410C_A:CR_10470C_A:DRS1

8152	metabolic process	604 out of 804 genes, 75.1%	4250 out of 6473 background genes, 65.7%	5.81E-07	0.00%	RIM8:PHR2:HBR1:GLO1:C1_00510W_A:C1_00520W_A:CIS2:CNS1:RRS1:NMA111:HMT1:C1_01130W_A:C1_01150C_A:C1_01160C_A:C1_01470W_A:SOD2:C1_01530C_A:C1_01900C_A:C1_02090C_A:ABP140:C1_02310C_A:C1_02430C_A:C1_02440C_A:C1_02450C_A:HOM6:PDE2:MIA40:ATG17:TSR2:RPL6:ARO80:MAS1:C1_03370W_A:EHT1:C1_03430W_A:C1_03790C_A:C1_03830C_A:C1_04040C_A:C1_04120C_A:ERB1:RPN4:NOP4:C1_04990C_A:RHD1:RRP6:C1_05220C_A:C1_05230W_A:C1_05330C_A:C1_05360C_A:RPS27A:RIB4:C1_05630C_A:FAL1:C1_05650W_A:C1_05930C_A:NPL4:C1_06120C_A:C1_06540C_A:ANT1:NOP6:C1_06760C_A:C1_06770W_A:MRPL19:C1_06800W_A:CAT1:C1_06910C_A:SUI2:C1_07090C_A:RIA1:YMC1:YPD1:RME1:C1_07390W_A:PEX3:LHP1:C1_07510W_A:C1_07570C_A:ADE4:GCS1:ADE5,7:C1_07950C_A:FUN12:DRG1:GCD6:C1_08630W_A:MSS116:C1_09060C_A:C1_09390W_A:FTH1:C1_09440W_A:GUA1:C1_09610W_A:C1_09710C_A:DBP3:TPK1:C1_10340W_A:C1_10410W_A:GLY1:C1_10620W_A:HNT1:C1_10970W_A:C1_11000C_A:C1_11010C_A:C1_11370C_A:KRR1:GAR1:C1_11560C_A:GAD1:MRF1:C1_11900C_A:C1_11910W_A:CSI2:C1_12350W_A:PKH2:C1_12440W_A:C1_12570C_A:CHR1:C1_12670C_A:C1_12760W_A:RBK1:PSA2:C1_13260W_A:C1_13270W_A:C1_13330C_A:AGC1:KNS1:HSP70:MET3:MODF:SIM1:C1_14080W_A:FTR1:C1_14160W_A:SAM35:MPP10:FAV3:NDT80:HMA1:C2_00220C_A:C2_00280C_A:C2_00410C_A:C2_00620C_A:C2_00810C_A:C2_00880W_A:MXR1:C2_01060C_A:C2_01070W_A:CLB2:C2_01510C_A:C2_01870C_A:ZCF6:C2_01920C_A:C2_02420C_A:PRS1:C2_02540W_A:FGR22:SER2:C2_02710C_A:HPT1:C2_02960C_A:LIG4:PLC2:ADE8:C2_03130W_A:PRP3:C2_03360W_A:C2_03550C_A:MNN42:C2_03700W_A:C2_03830W_A:C2_03910C_A:SCH9:GCD7:C2_04120C_A:UGA1:BAT21:C2_04570W_A:SNU114:C2_04820W_A:CRK1:C2_05080C_A:MAK5:ERF1:PEX2:C2_05160C_A:RPF2:RPC40:PEX6:C2_05520W_A:KTI11:PNG2:TES1:LAS1:CNT:FAA2-1:ECM17:IMH3:RTA2:C2_06480W_A:AMO2:RPL11:C2_06850W_A:C2_06890C_A:SPE3:C2_07070W_A:RCK2:C2_07140W_A:RPA12:NOC4:C2_07360W_A:SSC1:C2_07410W_A:RCL1:RNR22:C2_07610C_A:PRS5:C2_07920W_A:NSA1:C2_08200W_A:ORF298:C2_08460C_A:RRP8:C2_08620W_A:TIF4631:RPT6:SPO11:C2_08840W_A:FPK1:C2_09070C_A:MET10:SLP2:GLO2:PES1:RRP15:C2_09500W_A:C2_09660W_A:LEU42:C2_09930W_A:FCY21:YOR1:CWC22:OCA1:GPD1:PUS7:MTR2:TIF11:C2_10740C_A:RPC11:C2_10810W_A:C3_00100W_A:UTP8:BUD21:DOT5:SOF1:MIH1:C3_00950C_A:BMS1:URA3:C3_01520C_A:C3_01560W_A:C3_02020W_A:C3_02030W_A:C3_02040C_A:UTP4:C3_02180C_A:C3_02350W_A:PHA2:C3_03070W_A:C3_03330C_A:BMT7:C3_03470W_A:ULP1:HBR3:C3_03680W_A:SAP9:CSC25:AGT1:URK1:C3_04370C_A:C3_04380C_A:ADE2:MAK21:RPP2B:SFP1:CEM1:C3_05140C_A:C3_05160C_A:WOR2:SAP3:C3_05380W_A:C3_05510W_A:QDR2:BAT22:C3_05790C_A:C3_05800W_A:C3_05860C_A:CTA4:FAA2:NOG1:ELP3:DED1:C3_06150W_A:CYM1:HNT2:C3_06370C_A:NS

						A2:PRP5:C3_06760W_A:C3_06830C_A:C3_06860C_A:C3_06940W_A:C3_07100C_A:NOP13:GCY1:C3_07380W_A:C3_07400W_A:C3_07430W_A:C3_07480W_A:C3_07550C_A:RIT1:C3_07670W_A:UTP9:CAR2:DUR4:C4_00490W_A:RLP24:NMD5:C4_00690C_A:C4_00700C_A:C4_00810C_A:C4_00940W_A:MDN1:DAG7:RAT1:C4_01280C_A:PWP1:C4_01470W_A:NIP1:C4_01670C_A:TOA2:HGH1:HOS3:C4_02620C_A:C4_02850W_A:ZUO1:C4_02880C_A:C4_03040W_A:C4_03050C_A:C4_03150W_A:C4_03720C_A:C4_03730C_A:C4_03740W_A:C4_03830W_A:RRP42:RAM1:C4_04090C_A:ECM1:C4_04270W_A:SAP10:C4_04500C_A:C4_04510W_A:OMA1:SSZ1:C4_04720W_A:C4_04810C_A:AGP3:MYO5:NBP35:C4_05260W_A:C4_05650W_A:C4_06210C_A:SOU1:C4_06410W_A:MNN4:NOP1:C4_06790W_A:MNN46:MET16:YDC1:C4_07060W_A:C4_07100C_A:C4_07140W_A:VIP1:C5_00260W_A:C5_00280C_A:DUS4:RMT2:URA4:TRX2:C5_00920W_A:C5_00950C_A:BUD23:PUS4:C5_01430C_A:FYV5:GLR1:SPB4:C5_01610W_A:MAS2:GIS2:C5_01930W_A:RIB2:C5_02010C_A:C5_02070C_A:RIX7:GCD11:ZCF22:TIF5:UTP13:C5_02730C_A:C5_03010W_A:RMS1:RPO26:FUR1:C5_03530C_A:C5_03550W_A:C5_03640W_A:C5_03670C_A:FGR27:C5_03700C_A:HAM1:C5_03920C_A:C5_03970W_A:URA7:CSU57:C5_04610W_A:HAS1:C5_04990W_A:PCL5:C5_05340W_A:PEX4:NOP5:FET31:C6_00530C_A:C6_00640C_A:C6_00760W_A:HAL21:AEP1:C6_01040C_A:RCN1:CIC1:EBP1:C6_01300W_A:ACF2:C6_01870C_A:C6_01890C_A:C6_01980C_A:C6_02230W_A:C6_02350C_A:NIP7:C6_02410W_A:C6_02420W_A:C6_02480W_A:GCV1:C6_02560W_A:MOB1:SAC6:MRT4:POP4:EMF1:C6_03210C_A:BMT4:C6_03330C_A:C6_03390W_A:FGR17:PAD1:ALS5:SBA1:SHM2:SNQ2:SPB1:C6_04530C_A:NAG1:C7_00160C_A:C7_00330C_A:C7_00380W_A:C7_00490C_A:RPA135:NOP15:C7_01030C_A:DBP7:GCN3:C7_01360C_A:FLU1:RAD14:C7_02100W_A:YCF1:C7_02340C_A:C7_02460C_A:PEX1:C7_02930C_A:ZCF29:CPY1:ISY1:ENP2:MAM33:C7_03990C_A:C7_04140C_A:UTP18:MIS12:CR_00460C_A:PHO81:CR_00670C_A:BUD22:BMT3:PWP2:TRM1:CAB3:CR_01410C_A:CR_01700C_A:CR_01780W_A:CR_01950W_A:CR_02030C_A:CR_02420W_A:CR_02430C_A:CR_02460W_A:CR_02510W_A:RPC19:DBP2:RPC31:CR_02890C_A:AQY1:SRP40:CR_03110W_A:CR_03230W_A:CR_03360W_A:KTI12:NCS2:YVH1:IFF3:SGD1:PRP42:CR_03760W_A:CR_03940W_A:GCD1:MEX67:CR_04110W_A:CR_04160C_A:CR_04170W_A:CR_04240C_A:CR_04300W_A:NHP2:RBR2:ADE6:RPB8:CR_04920W_A:YCP4:NOC2:CR_05550C_A:RNR3:MTG2:DBP8:VPS75:SFL1:SOK1:RPL7:FGR50:RGS2:CR_06680C_A:NMD3:CR_06840W_A:CR_06980W_A:CR_07030C_A:CR_07080W_A:CR_07470W_A:CR_07480W_A:CR_07640C_A:DBP6:CR_07780W_A:IMP4:CR_08000C_A:NOP10:SSB1:RIO2:CR_08130W_A:TRM9:HSP104:ATS1:CR_08330W_A:CYS3:GSH2:TSR1:PRP8:CR_08920W_A:CR_08940W_A:CR_09110C_A:CR_09140C_A:CR_09310W_A:ELF1:CR_09670C_A:SSF1:CR_09800C_A:SIK1:UGA3:CR_09990W_A:CR_10170C_A:UTP5:AHA1:CR_10400W_A:CR_10410C_A:CR_10430C_A:CR_10470C_A:DRS1:POP3

154	rRNA modification	15 out of 804 genes, 1.9%	21 out of 6473 background genes, 0.3%	8.30E-07	0.00%	C1_10620W_A:C2_04820W_A:RRP8:C2_08840W_A:PUS7:C3_05860C_A:NOP1:BUD23:SPB1:CR_01780W_A:CR_02030C_A:CR_04170W_A:CR_04240C_A:NHP2:NOP10
1522	pseudouridine synthesis	11 out of 804 genes, 1.4%	12 out of 6473 background genes, 0.2%	1.50E-06	0.00%	C1_07570C_A:C1_10620W_A:GAR1:PUS7:PUS4:C5_01610W_A:RIB2:C6_02350C_A:CR_04110W_A:NHP2:NOP10
447	endonucleolytic cleavage in ITS1 to separate SSU-rRNA from 5.8S rRNA and LSU-rRNA from tricistronic rRNA transcript (SSU-rRNA, 5.8S rRNA, LSU-rRNA)	36 out of 804 genes, 4.5%	103 out of 6473 background genes, 1.6%	3.11E-06	0.00%	RRS1:C1_04040C_A:C1_09710C_A:KRR1:CHR1:C1_14080W_A:MPP10:FAV3:C2_00410C_A:C2_02540W_A:NOC4:RCL1:BUD21:C3_02020W_A:C3_02040C_A:C3_02350W_A:C3_06370C_A:DUR4:C4_00490W_A:C4_00690C_A:BUD23:C5_02070C_A:UTP13:C5_03920C_A:NOP5:ALS5:UTP18:PWP2:CR_02420W_A:CR_03360W_A:DBP8:ATS1:CR_08330W_A:PRP8:CR_09800C_A:CR_10410C_A

42797	tRNA transcription by RNA polymerase III	13 out of 804 genes, 1.6%	18 out of 6473 background genes, 0.3%	9.93E-06	0.00%	C1_05230W_A:RPC40:RPC11:C4_03040W_A:C4_07060W_A:RPO26:C5_04990W_A:C6_03210C_A:RPC19:RPC31:CR_02890C_A:RPB8:CR_05550C_A
9304	tRNA transcription	13 out of 804 genes, 1.6%	18 out of 6473 background genes, 0.3%	9.93E-06	0.00%	C1_05230W_A:RPC40:RPC11:C4_03040W_A:C4_07060W_A:RPO26:C5_04990W_A:C6_03210C_A:RPC19:RPC31:CR_02890C_A:RPB8:CR_05550C_A
33750	ribosome localization	28 out of 804 genes, 3.5%	72 out of 6473 background genes, 1.1%	1.29E-05	0.00%	RRS1:C1_02450C_A:ARX1:C1_04040C_A:C1_12760W_A:C2_06850W_A:KRE30:MTR2:C3_02040C_A:SFP1:NOG1:C3_06150W_A:ZUO1:ECM1:C4_04510W_A:C4_06210C_A:C4_07100C_A:BUD23:RIX7:C6_02230W_A:MRT4:C7_04140C_A:YVH1:MEX67:SDA1:NMD3:CR_07080W_A:SSB1
54	ribosomal subunit export from nucleus	28 out of 804 genes, 3.5%	72 out of 6473 background genes, 1.1%	1.29E-05	0.00%	RRS1:C1_02450C_A:ARX1:C1_04040C_A:C1_12760W_A:C2_06850W_A:KRE30:MTR2:C3_02040C_A:SFP1:NOG1:C3_06150W_A:ZUO1:ECM1:C4_04510W_A:C4_06210C_A:C4_07100C_A:BUD23:RIX7:C6_02230W_A:MRT4:C7_04140C_A:YVH1:MEX67:SDA1:NMD3:CR_07080W_A:SSB1

2E+06	assembly of large subunit precursor of preribosome	8 out of 804 genes, 1.0%	8 out of 6473 background genes, 0.1%	7.57E-05	0.00%	RPF2:C3_05160C_A:NOG1:RLP24:SPB4:NIP7:CR_04170W_A:CR_07080W_A
377	RNA splicing, via transesterification reactions with bulged adenosine as nucleophile	32 out of 804 genes, 4.0%	102 out of 6473 background genes, 1.6%	0.00043	0.00%	C1_02090C_A:C1_04120C_A:C1_04990C_A:MSS116:C1_12670C_A:C2_00280C_A:PRP3:SNU114:CWC22:C2_10810W_A:C3_00100W_A:C3_01560W_A:C3_03330C_A:C3_04380C_A:DED1:PRP5:C3_07480W_A:C4_01280C_A:C4_03150W_A:C4_06410W_A:C4_07100C_A:C5_00920W_A:C5_01930W_A:C5_03010W_A:C5_04610W_A:C6_00530C_A:ISY1:CR_01700C_A:CR_03230W_A:PRP42:PRP8:CR_10430C_A
17183	protein histidyl modification to diphthamide	8 out of 804 genes, 1.0%	9 out of 6473 background genes, 0.1%	0.0006	0.00%	C2_01060C_A:C2_03830W_A:KTI11:C2_07920W_A:C4_00490W_A:C4_00690C_A:C5_03640W_A:C5_03700C_A
31120	snRNA pseudouridine synthesis	7 out of 804 genes, 0.9%	7 out of 6473 background genes, 0.1%	0.00061	0.00%	C1_10620W_A:GAR1:PUS7:C6_02350C_A:CR_04110W_A:NHP2:NOP10

398	mRNA splicing, via spliceosome	31 out of 804 genes, 3.9%	101 out of 6473 background genes, 1.6%	0.00114	0.00%	C1_02090C_A:C1_04120C_A:C1_04990C_A:C1_12670C_A:C2_00280C_A:PRP3:SNU114:CWC22:C2_10810W_A:C3_00100W_A:C3_01560W_A:C3_03330C_A:C3_04380C_A:DED1:PRP5:C3_07480W_A:C4_01280C_A:C4_03150W_A:C4_06410W_A:C4_07100C_A:C5_00920W_A:C5_01930W_A:C5_03010W_A:C5_04610W_A:C6_00530C_A:ISY1:CR_01700C_A:CR_03230W_A:PRP42:PRP8:CR_10430C_A
16071	mRNA metabolic process	55 out of 804 genes, 6.8%	231 out of 6473 background genes, 3.6%	0.00115	0.00%	C1_01470W_A:C1_02090C_A:C1_03370W_A:C1_03830C_A:C1_04120C_A:C1_04990C_A:RRP6:C1_05360C_A:C1_06540C_A:C1_06800W_A:C1_08630W_A:C1_10620W_A:C1_11900C_A:C1_11910W_A:C1_12350W_A:C1_12670C_A:C2_00280C_A:PRP3:C2_03550C_A:SNU114:CWC22:PUS7:C2_10810W_A:C3_00100W_A:C3_01560W_A:C3_03330C_A:C3_04380C_A:DED1:PRP5:C3_07480W_A:RAT1:C4_01280C_A:C4_03150W_A:C4_03740W_A:C4_06410W_A:C4_07100C_A:C5_00920W_A:PUS4:C5_01610W_A:C5_01930W_A:C5_03010W_A:C5_04610W_A:C6_00530C_A:C6_02350C_A:MRT4:POP4:ISY1:CR_01700C_A:DBP2:CR_03230W_A:PRP42:CR_04240C_A:PRP8:CR_10430C_A:POP3

31167	rRNA methylation	9 out of 804 genes, 1.1%	12 out of 6473 background genes, 0.2%	0.00144	0.00%	RRP8:C2_08840W_A:C3_05860C_A:NOP1:BUD23:SPB1:CR_01780W_A:CR_02030C_A:CR_04170W_A
375	RNA splicing, via transesterification reactions	32 out of 804 genes, 4.0%	107 out of 6473 background genes, 1.7%	0.00145	0.00%	C1_02090C_A:C1_04120C_A:C1_04990C_A:MSS116:C1_12670C_A:C2_00280C_A:PRP3:SNU114:CWC22:C2_10810W_A:C3_00100W_A:C3_01560W_A:C3_03330C_A:C3_04380C_A:DED1:PRP5:C3_07480W_A:C4_01280C_A:C4_03150W_A:C4_06410W_A:C4_07100C_A:C5_00920W_A:C5_01930W_A:C5_03010W_A:C5_04610W_A:C6_00530C_A:ISY1:CR_01700C_A:CR_03230W_A:PRP42:PRP8:CR_10430C_A
40031	snRNA modification	7 out of 804 genes, 0.9%	8 out of 6473 background genes, 0.1%	0.00438	0.00%	C1_10620W_A:GAR1:PUS7:C6_02350C_A:CR_04110W_A:NHP2:NOP10
31503	protein-containing complex localization	28 out of 804 genes, 3.5%	92 out of 6473 background genes, 1.4%	0.00469	0.00%	RRS1:C1_02450C_A:ARX1:C1_04040C_A:C1_12760W_A:C2_06850W_A:KRE30:MTR2:C3_02040C_A:SFP1:NOG1:C3_06150W_A:ZUO1:ECM1:C4_04510W_A:C4_06210C_A:C4_07100C_A:BUD23:RIX7:C6_02230W_A:MRT4:C7_04140C_A:YVH1:MEX67:SDA1:NMD3:CR_07080W_A:SSB1

8380	RNA splicing	33 out of 804 genes, 4.1%	119 out of 6473 background genes, 1.8%	0.00632	0.00%	C1_02090C_A:C1_04120C_A:C1_04990C_A:MSS116:C1_12670C_A:C2_00280C_A:PRP3:SNU114:CWC22:C2_10810W_A:C3_00100W_A:C3_01560W_A:C3_03330C_A:C3_04380C_A:C3_05800W_A:DED1:PRP5:C3_07480W_A:C4_01280C_A:C4_03150W_A:C4_06410W_A:C4_07100C_A:C5_00920W_A:C5_01930W_A:C5_03010W_A:C5_04610W_A:C6_00530C_A:ISY1:CR_01700C_A:CR_03230W_A:PRP42:PRP8:CR_10430C_A

9987	cellular process	627 out of 804 genes, 78.0%	4625 out of 6473 background genes, 71.5%	0.00635	0.00%	RIM8:C1_00160C_A:PHR2:HBR1:GLO1:C1_00510W_A:C1_00520W_A:CIS2:CNS1:RRS1:NMA111:HMT1:CCH1:ZPR1:C1_01130W_A:C1_01150C_A:C1_01160C_A:C1_01470W_A:SOD2:C1_01530C_A:C1_01750W_A:C1_01900C_A:C1_02090C_A:ABP140:C1_02310C_A:C1_02430C_A:C1_02440C_A:C1_02450C_A:HOM6:PDE2:MIA40:ATG17:TSR2:RPL6:ARO80:ARX1:MAS1:C1_03370W_A:EHT1:C1_03430W_A:C1_03790C_A:C1_03830C_A:C1_04040C_A:C1_04120C_A:ERB1:IFD6:RPN4:C1_04340C_A:NOP4:C1_04990C_A:RHD1:RRP6:C1_05210C_A:C1_05220C_A:C1_05230W_A:C1_05330C_A:C1_05360C_A:RPS27A:RIB4:C1_05630C_A:FAL1:C1_05650W_A:C1_05930C_A:NPL4:C1_06120C_A:C1_06510C_A:C1_06540C_A:ANT1:NOP6:C1_06760C_A:C1_06770W_A:MRPL19:C1_06800W_A:CAT1:C1_06910C_A:SUI2:C1_07090C_A:RIA1:YMC1:YPD1:RME1:C1_07390W_A:PEX3:LHP1:C1_07510W_A:C1_07570C_A:C1_07690C_A:ADE4:GCS1:ADE5,7:C1_07950C_A:CDR4:FUN12:DRG1:GCD6:C1_08630W_A:MSS116:JIP5:C1_09060C_A:CTA8:C1_09390W_A:FTH1:C1_09440W_A:GUA1:YTM1:C1_09610W_A:C1_09710C_A:DBP3:TPK1:C1_10340W_A:GLY1:C1_10620W_A:HNT1:C1_10970W_A:C1_11000C_A:C1_11010C_A:TIM50:C1_11370C_A:KRR1:GAR1:C1_11560C_A:GAD1:MRF1:C1_11900C_A:C1_11910W_A:CSI2:C1_12350W_A:PKH2:C1_12440W_A:C1_12570C_A:CHR1:C1_12670C_A:C1_12680W_A:C1_12760W_A:C1_13130C_A:C1_13260W_A:C1_13270W_A:C1_13330C_A:C1_13370W_A:AGC1:KNS1:HSP70:MET3:MODF:SIM1:C1_14080W_A:FTR1:SAM35:MPP10:FAV3:NDT80:HMA1:C2_00220C_A:C2_00280C_A:C2_00410C_A:C2_00810C_A:C2_00880W_A:MXR1:HGT8:C2_01070W_A:CLB2:C2_01510C_A:C2_01870C_A:ZCF6:C2_01920C_A:C2_02420C_A:PRS1:C2_02540W_A:FGR22:SER2:C2_02710C_A:HPT1:LIG4:ADE8:PRP3:C2_03360W_A:C2_03550C_A:MNN42:C2_03910C_A:SCH9:GCD7:C2_04120C_A:UGA1:BAT21:C2_04570W_A:SNU114:C2_04820W_A:CRK1:FRE9:C2_05080C_A:MAK5:ERF1:PEX2:C2_05160C_A:RPF2:RPC40:PEX6:C2_05520W_A:KTI11:C2_05810W_A:TES1:LAS1:CNT:ECM17:IMH3:RTA3:RTA2:C2_06480W_A:RPL11:C2_06850W_A:SPE3:C2_07070W_A:RCK2:C2_07140W_A:RPA12:NOC4:C2_07360W_A:SSC1:C2_07410W_A:RCL1:RNR22:C2_07610C_A:PRS5:C2_07920W_A:NSA1:KRE30:SIT1:ORF298:C2_08460C_A:RRP8:TIF4631:RPT6:SPO11:C2_08840W_A:FPK1:C2_09070C_A:MET10:SLP2:GLO2:PES1:RRP15:C2_09500W_A:C2_09660W_A:LEU42:TIM23:C2_09930W_A:FCY21:YOR1:CWC22:OCA1:GPD1:PUS7:MTR2:TIF11:C2_10740C_A:RPC11:C2_10810W_A:C3_00100W_A:UTP8:BUD21:DOT5:SOF1:MIH1:C3_00950C_A:BMS1:C3_01140W_A:URA3:C3_01520C_A:C3_01560W_A:C3_01680C_A:C3_02020W_A:C3_02030W_A:C3_02040C_A:UTP4:C3_02180C_A:C3_02350W_A:PHA2:C3_02870C_A:C3_03070W_A:C3_03330C_A:BMT7:C3_03470W_A:HBR3:C3_03680W_A:SAP9:CSC25:AGT1:URK1:C3_04370C_A:C3_04380C_A:RAS2:ADE2:MAK21:RPP2B:SFP1:CEM1:C3_05140C_A:C3_05160C_A:WOR2:SAP3:C3_05380W_A:C3_05510W_A:QDR2:BAT22:C3_05790C_A:C3_05800W_A:C3_05860C_A:CTA4:NOG1:ELP3:DED1:C3_06150W_A:HNT2:C3_06370C_A:NS

						A2:PRP5:C3_06760W_A:C3_06830C_A:FCR1:C3_06860C_A:C3_06940W_A:C3_07100C_A:NOP13:GCY1:C3_07400W_A:C3_07430W_A:C3_07480W_A:C3_07550C_A:RIT1:C3_07670W_A:UTP9:CAR2:DUR4:C4_00490W_A:RLP24:NMD5:C4_00690C_A:C4_00700C_A:C4_00810C_A:C4_00940W_A:MDN1:DAG7:RAT1:C4_01130C_A:C4_01280C_A:PWP1:C4_01470W_A:NIP1:C4_01670C_A:TOA2:HGH1:C4_02260C_A:HOS3:C4_02720C_A:C4_02850W_A:ZUO1:C4_02880C_A:GST2:C4_03040W_A:DNF3:C4_03150W_A:C4_03720C_A:C4_03730C_A:C4_03740W_A:C4_03830W_A:RRP42:RAM1:C4_04090C_A:ECM1:C4_04270W_A:SAP10:C4_04510W_A:OMA1:SSZ1:C4_04720W_A:C4_04810C_A:AGP3:FGR10:C4_05010W_A:MYO5:NBP35:C4_05260W_A:C4_05650W_A:CFL1:C4_06210C_A:C4_06410W_A:MNN4:NOP1:C4_06790W_A:C4_06850C_A:MNN46:MET16:YDC1:C4_07060W_A:C4_07100C_A:C4_07140W_A:VIP1:C5_00260W_A:C5_00280C_A:DUS4:RMT2:URA4:TRX2:C5_00920W_A:C5_00950C_A:BUD23:PUS4:C5_01430C_A:FYV5:GLR1:SPB4:C5_01610W_A:MAS2:GIS2:C5_01930W_A:RIB2:C5_02010C_A:C5_02070C_A:RIX7:GCD11:ZCF22:TIF5:UTP13:C5_02730C_A:C5_03010W_A:RMS1:RPO26:FUR1:C5_03470C_A:C5_03530C_A:C5_03550W_A:C5_03670C_A:FGR27:HAM1:C5_03920C_A:C5_03970W_A:URA7:CSU57:C5_04610W_A:HAS1:C5_04990W_A:PCL5:C5_05340W_A:PEX4:NOP5:FET31:C6_00530C_A:SLY41:C6_00640C_A:C6_00660C_A:C6_00760W_A:HAL21:AEP1:C6_01040C_A:RCN1:CIC1:C6_01300W_A:ACF2:C6_01870C_A:C6_01890C_A:C6_01980C_A:C6_02230W_A:C6_02350C_A:NIP7:C6_02410W_A:C6_02420W_A:GCV1:C6_02560W_A:MOB1:SAC6:MRT4:POP4:C6_03180C_A:EMF1:C6_03210C_A:BMT4:C6_03330C_A:C6_03390W_A:FGR17:PAD1:ALS5:ALS9:SBA1:SHM2:SNQ2:ALS4:SPB1:ALS2:C6_04530C_A:NAG1:C7_00160C_A:C7_00330C_A:C7_00380W_A:C7_00490C_A:RPA135:NOP15:C7_01030C_A:DBP7:GCN3:C7_01360C_A:FLU1:C7_01940C_A:RAD14:C7_02100W_A:FMP45:YCF1:C7_02340C_A:C7_02460C_A:PEX1:C7_02930C_A:ZCF29:CPY1:ISY1:ENP2:MAM33:C7_03990C_A:C7_04140C_A:UTP18:NRG1:MIS12:CR_00460C_A:PHO81:CR_00670C_A:BUD22:BMT3:PWP2:TRM1:CAB3:CR_01410C_A:CR_01700C_A:CR_01780W_A:CR_01950W_A:CR_02030C_A:CR_02420W_A:CR_02430C_A:CR_02460W_A:CR_02510W_A:RPC19:DBP2:RPC31:CR_02890C_A:AQY1:SRP40:CR_03110W_A:CR_03230W_A:CR_03360W_A:KTI12:NCS2:YVH1:IFF3:SGD1:PRP42:CR_03760W_A:CR_03940W_A:GCD1:MEX67:CR_04110W_A:CR_04160C_A:CR_04170W_A:CR_04240C_A:CR_04300W_A:NHP2:RBR2:ADE6:RPB8:CR_04920W_A:YCP4:PST3:NOC2:CR_05550C_A:RNR3:MTG2:DBP8:SDA1:VPS75:SFL1:SOK1:RPL7:FGR50:RGS2:CR_06680C_A:NMD3:CR_06840W_A:CR_06980W_A:CR_07030C_A:CRZ2:CR_07080W_A:CR_07160C_A:CR_07470W_A:CR_07480W_A:CR_07640C_A:DBP6:CR_07780W_A:IMP4:CR_08000C_A:NOP10:SSB1:RIO2:CR_08130W_A:TRM9:HSP104:ATS1:CR_08330W_A:CYS3:GSH2:TSR1:CR_08500W_A:PRP8:CR_08940W_A:CR_09110C_A:CR_09140C_A:ELF1:CR_09670C

						_A:SSF1:CR_09800C_A:SIK1:UGA3:CR_09990W_A:CR_10170C_A:UTP5:AHA1:CR_10400W_A:CR_10410C_A:CR_10430C_A:CR_10470C_A:DRS1:POP3
18202	peptidyl-histidine modification	8 out of 804 genes, 1.0%	11 out of 6473 background genes, 0.2%	0.00882	0.00%	C2_01060C_A:C2_03830W_A:KTI11:C2_07920W_A:C4_00490W_A:C4_00690C_A:C5_03640W_A:C5_03700C_A

6383	transcription by RNA polymerase III	14 out of 804 genes, 1.7%	31 out of 6473 background genes, 0.5%	0.00883	0.00%	C1_05230W_A:RPC40:RPC11:C4_03040W_A:C4_04510W_A:C4_07060W_A:RPO26:C5_04990W_A:C6_03210C_A:RPC19:RPC31:CR_02890C_A:RPB8:CR_05550C_A
43633	polyadenylation-dependent RNA catabolic process	10 out of 804 genes, 1.2%	17 out of 6473 background genes, 0.3%	0.00975	0.00%	C1_01160C_A:C1_03830C_A:RRP6:C1_05360C_A:C1_06540C_A:C1_06800W_A:C1_12350W_A:RAT1:C4_03740W_A:RRP42
43634	polyadenylation-dependent ncRNA catabolic process	10 out of 804 genes, 1.2%	17 out of 6473 background genes, 0.3%	0.00975	0.00%	C1_01160C_A:C1_03830C_A:RRP6:C1_05360C_A:C1_06540C_A:C1_06800W_A:C1_12350W_A:RAT1:C4_03740W_A:RRP42
71029	nuclear ncRNA surveillance	10 out of 804 genes, 1.2%	17 out of 6473 background genes, 0.3%	0.00975	0.00%	C1_01160C_A:C1_03830C_A:RRP6:C1_05360C_A:C1_06540C_A:C1_06800W_A:C1_12350W_A:RAT1:C4_03740W_A:RRP42
71046	nuclear polyadenylation-dependent ncRNA catabolic process	10 out of 804 genes, 1.2%	17 out of 6473 background genes, 0.3%	0.00975	0.00%	C1_01160C_A:C1_03830C_A:RRP6:C1_05360C_A:C1_06540C_A:C1_06800W_A:C1_12350W_A:RAT1:C4_03740W_A:RRP42

6397	mRNA processing	38 out of 804 genes, 4.7%	149 out of 6473 background genes, 2.3%	0.01093	0.00%	C1_01470W_A:C1_02090C_A:C1_03370W_A:C1_03830C_A:C1_04120C_A:C1_04990C_A:C1_06540C_A:C1_08630W_A:C1_11910W_A:C1_12350W_A:C1_12670C_A:C2_00280C_A:PRP3:SNU114:CWC22:C2_10810W_A:C3_00100W_A:C3_01560W_A:C3_03330C_A:C3_04380C_A:DED1:PRP5:C3_07480W_A:C4_01280C_A:C4_03150W_A:C4_06410W_A:C4_07100C_A:C5_00920W_A:C5_01930W_A:C5_03010W_A:C5_04610W_A:C6_00530C_A:ISY1:CR_01700C_A:CR_03230W_A:PRP42:PRP8:CR_10430C_A
2097	tRNA wobble base modification	13 out of 804 genes, 1.6%	28 out of 6473 background genes, 0.4%	0.01307	0.00%	C1_12570C_A:KTI11:ELP3:C4_00940W_A:C4_02850W_A:NBP35:C4_05260W_A:C5_02730C_A:KTI12:NCS2:CR_04300W_A:TRM9:ATS1
6413	translational initiation	16 out of 804 genes, 2.0%	41 out of 6473 background genes, 0.6%	0.01918	0.00%	C1_01900C_A:C1_02430C_A:SUI2:FUN12:GCD6:GCD7:TIF4631:TIF11:DED1:NIP1:C5_02010C_A:GCD11:TIF5:GCN3:GCD1:CR_04160C_A

9156	ribonucleoside monophosphate biosynthetic process	10 out of 804 genes, 1.2%	18 out of 6473 background genes, 0.3%	0.01951	0.00%	ADE4:ADE5,7:GUA1:HPT1:ADE8:C2_03360W_A:IMH3:URA3:ADE2:ADE6
9161	ribonucleoside monophosphate metabolic process	10 out of 804 genes, 1.2%	18 out of 6473 background genes, 0.3%	0.01951	0.00%	ADE4:ADE5,7:GUA1:HPT1:ADE8:C2_03360W_A:IMH3:URA3:ADE2:ADE6
71035	nuclear polyadenylation-dependent rRNA catabolic process	9 out of 804 genes, 1.1%	15 out of 6473 background genes, 0.2%	0.02312	0.00%	C1_01160C_A:C1_03830C_A:RRP6:C1_05360C_A:C1_06540C_A:C1_06800W_A:C1_12350W_A:RAT1:C4_03740W_A
9126	purine nucleoside monophosphate metabolic process	9 out of 804 genes, 1.1%	15 out of 6473 background genes, 0.2%	0.02312	0.00%	ADE4:ADE5,7:GUA1:HPT1:ADE8:C2_03360W_A:IMH3:ADE2:ADE6
9127	purine nucleoside monophosphate biosynthetic process	9 out of 804 genes, 1.1%	15 out of 6473 background genes, 0.2%	0.02312	0.00%	ADE4:ADE5,7:GUA1:HPT1:ADE8:C2_03360W_A:IMH3:ADE2:ADE6

9167	purine ribonucleoside monophosphate metabolic process	9 out of 804 genes, 1.1%	15 out of 6473 background genes, 0.2%	0.02312	0.00%	ADE4:ADE5,7:GUA1:HPT1:ADE8:C2_03360W_A:IMH3:ADE2:ADE6
9168	purine ribonucleoside monophosphate biosynthetic process	9 out of 804 genes, 1.1%	15 out of 6473 background genes, 0.2%	0.02312	0.00%	ADE4:ADE5,7:GUA1:HPT1:ADE8:C2_03360W_A:IMH3:ADE2:ADE6
70475	rRNA base methylation	6 out of 804 genes, 0.7%	7 out of 6473 background genes, 0.1%	0.03115	0.00%	C2_08840W_A:C3_05860C_A:BUD23:CR_01780W_A:CR_02030C_A:CR_04170W_A
2098	tRNA wobble uridine modification	12 out of 804 genes, 1.5%	26 out of 6473 background genes, 0.4%	0.03131	0.00%	C1_12570C_A:KTI11:ELP3:C4_00940W_A:C4_02850W_A:NBP35:C4_05260W_A:C5_02730C_A:KTI12:NCS2:TRM9:ATS1

51168	nuclear export	37 out of 804 genes, 4.6%	150 out of 6473 background genes, 2.3%	0.03226	0.00%	RRS1:HMT1:C1_01470W_A:C1_02450C_A:ARX1:C1_04040C_A:C1_04120C_A:C1_07690C_A:C1_11910W_A:C1_12760W_A:C2_06850W_A:KRE30:MTR2:UTP8:C3_01520C_A:C3_02040C_A:SFP1:NOG1:C3_06150W_A:ZUO1:ECM1:C4_04510W_A:C4_06210C_A:C4_07100C_A:BUD23:RIX7:C5_03550W_A:C6_02230W_A:MRT4:C7_04140C_A:YVH1:MEX67:SDA1:NMD3:CR_07080W_A:SSB1:ELF1
2E+06	mRNA pseudouridine synthesis	5 out of 804 genes, 0.6%	5 out of 6473 background genes, 0.1%	0.04029	0.00%	C1_10620W_A:PUS7:PUS4:C5_01610W_A:C6_02350C_A
42256	cytosolic ribosome assembly	5 out of 804 genes, 0.6%	5 out of 6473 background genes, 0.1%	0.04029	0.00%	RIA1:FUN12:C4_02880C_A:TIF5:CR_07080W_A
2183	cytoplasmic translational initiation	11 out of 804 genes, 1.4%	23 out of 6473 background genes, 0.4%	0.04578	0.00%	SUI2:FUN12:GCD6:GCD7:TIF11:DED1:C5_02010C_A:GCD11:TIF5:GCN3:GCD1

16075	rRNA catabolic process	12 out of 804 genes, 1.5%	27 out of 6473 background genes, 0.4%	0.05005	0.00%	C1_01160C_A:C1_03830C_A:RRP6:C1_05360C_A:NPL4:C1_06540C_A:C1_06800W_A:C1_12350W_A:RPT6:RAT1:C4_03740W_A:RRP42
46040	IMP metabolic process	7 out of 804 genes, 0.9%	10 out of 6473 background genes, 0.2%	0.05237	0.00%	ADE4:ADE5,7:HPT1:ADE8:C2_03360W_A:ADE2:ADE6
6188	IMP biosynthetic process	7 out of 804 genes, 0.9%	10 out of 6473 background genes, 0.2%	0.05237	0.00%	ADE4:ADE5,7:HPT1:ADE8:C2_03360W_A:ADE2:ADE6

22607	cellular component assembly	110 out of 804 genes, 13.7%	623 out of 6473 background genes, 9.6%	0.05813	0.00%	RRS1:ATG17:RPL6:C1_03790C_A:C1_04040C_A:C1_04120C_A:ERB1:NOP4:RRP6:C1_05360C_A:RPS27A:C1_06760C_A:SUI2:RIA1:FUN12:JIP5:C1_09710C_A:C1_10970W_A:CSI2:C1_12440W_A:C1_12760W_A:C1_14080W_A:SAM35:C2_00280C_A:C2_00810C_A:C2_01070W_A:C2_02540W_A:C2_02710C_A:PRP3:SNU114:MAK5:C2_05160C_A:RPF2:RPL11:TIF4631:RPT6:TIF11:C2_10740C_A:C3_00100W_A:BMS1:C3_01560W_A:C3_02020W_A:C3_02040C_A:C3_02350W_A:C3_03330C_A:HBR3:C3_04370C_A:MAK21:C3_05160C_A:QDR2:NOG1:C3_06370C_A:PRP5:C3_07480W_A:RLP24:MDN1:TOA2:C4_02880C_A:C4_03150W_A:C4_05010W_A:MYO5:NBP35:C4_06410W_A:C4_07100C_A:RMT2:SPB4:C5_01930W_A:C5_02010C_A:C5_02070C_A:GCD11:TIF5:HAS1:C5_05340W_A:CIC1:C6_02230W_A:NIP7:MOB1:SAC6:SPB1:RPA135:DBP7:FLU1:ISY1:ENP2:UTP18:BUD22:PWP2:CR_01410C_A:CR_01700C_A:DBP2:YVH1:SGD1:CR_03940W_A:CR_04110W_A:CR_04170W_A:NOC2:CR_05550C_A:SDA1:VPS75:CR_06680C_A:CR_07080W_A:CR_08130W_A:TSR1:PRP8:ELF1:SSF1:CR_09800C_A:CR_10410C_A:CR_10470C_A:DRS1
55	ribosomal large subunit export from nucleus	18 out of 804 genes, 2.2%	55 out of 6473 background genes, 0.8%	0.09129	0.00%	RRS1:C1_02450C_A:ARX1:C1_12760W_A:MTR2:C3_02040C_A:SFP1:C3_06150W_A:ECM1:C4_04510W_A:C4_06210C_A:C4_07100C_A:RIX7:MRT4:YVH1:MEX67:SDA1:NMD3

S120 vs B120 Upregulated genes						
GOID	GO_term	Cluster frequency	Background frequency	Corrected P-value	False discovery rate	Gene(s) annotated to the term
34470	ncRNA processing	157 out of 537 genes, 29.2%	860 out of 6473 background genes, 13.3%	1.42E-21	0.00%	CNS1:C1_01150C_A:C1_01160C_A:C1_02090C_A:PDE2:C1_02850W_A:C1_03830C_A:SEN15:C1_04040C_A:RRP6:REI1:NOP6:C1_06760C_A:C1_06800W_A:C1_07950C_A:C1_07960W_A:DIP2:MSS116:C1_09710C_A:C1_10880W_A:C1_10950C_A:C1_10970W_A:NEP1:KRR1:C1_13820C_A:FAV3:NDT80:C2_00410C_A:C2_00820W_A:C2_00840W_A:REX2:UTP21:C2_02540W_A:C2_02710C_A:C2_04120C_A:RIM2:C2_04570W_A:C2_04820W_A:MAK5:C2_05160C_A:LAS1:MAK16:RTA2:C2_06480W_A:C2_06850W_A:NOC4:RCL1:C2_07520C_A:C2_07920W_A:NSA1:RRP8:UTP15:C2_09160W_A:RRP15:C2_09500W_A:C2_09510C_A:C2_09660W_A:C2_09920W_A:PUS7:C3_00100W_A:BUD21:C3_00420W_A:BMS1:C3_01560W_A:C3_02040C_A:UTP4:ILV2:C3_02670W_A:HBR3:CCC1:C3_04370C_A:MAK21:SFP1:CEM1:C3_05120C_A:C3_05140C_A:C3_05380W_A:QDR2:C3_05800W_A:NSA2:C3_06760W_A:NOP13:C3_07550C_A:RAD4:RLP24:C4_00690C_A:RAT1:C4_01730C_A:TIM12:SAS10:HCA4:ZUO1:C4_02880C_A:NAN1:RAM1:C4_04810C_A:AGP3:C4_05230C_A:C4_06210C_A:C4_06790W_A:C5_01430C_A:C5_02010C_A:RIX7:C5_02730C_A:C5_03920C_A:C5_05340W_A:C6_01040C_A:C6_01890C_A:C6_02230W_A:C6_02430W_A:C6_04240W_A:C6_04530C_A:NOP15:C7_01030C_A:DBP7:C7_02100W_A:C7_02340C_A:TIM54:C7_03400C_A:ISY1:ENP2:TIM9:C7_03850W_A:C7_03880C_A:UTP18:CR_00670C_A:BUD22:CR_01410C_A:CR_02030C_A:CR_02420W_A:RRP9:CR_03200C_A:CR_03240C_A:KTI12:PRP42:CR_04110W_A:CR_04170W_A:CR_04240C_A:MTG2:DBP8:SFL1:FGR50:NMD3:CR_07030C_A:CR_07640C_A:IMP4:CR_08000C_A:CR_08330W_A:CR_08410W_A:TSR1:SKI2:CR_08940W_A:CR_09800C_A:UTP5:CR_10410C_A:CR_10470C_A:DRS1

42274	ribosomal small subunit biogenesis	129 out of 537 genes, 24.0%	636 out of 6473 background genes, 9.8%	3.73E-21	0.00%	CNS1:C1_01160C_A:C1_02090C_A:C1_02850W_A:C1_03830C_A:SEN15:C1_04040C_A:REI1:NOP6:C1_06800W_A:C1_07960W_A:DIP2:MSS116:C1_09710C_A:C1_10880W_A:C1_10950C_A:NEP1:KRR1:C1_13820C_A:FAV3:NDT80:C2_00410C_A:C2_00820W_A:C2_00840W_A:C2_02540W_A:C2_02710C_A:C2_04120C_A:RIM2:C2_04570W_A:C2_04820W_A:MAK5:C2_05160C_A:LAS1:MAK16:RTA2:C2_06850W_A:NOC4:RCL1:C2_07520C_A:C2_07920W_A:NSA1:RRP8:UTP15:C2_09160W_A:RRP15:C2_09510C_A:C2_09660W_A:C2_09920W_A:C3_00100W_A:BUD21:C3_00420W_A:BMS1:C3_01560W_A:C3_02040C_A:UTP4:C3_02670W_A:HBR3:CCC1:C3_04370C_A:MAK21:SFP1:CEM1:C3_05120C_A:QDR2:C3_05800W_A:NSA2:C3_06760W_A:NOP13:C3_07550C_A:RAD4:RLP24:C4_00690C_A:RAT1:C4_01730C_A:SAS10:HCA4:C4_02880C_A:NAN1:C4_06790W_A:C5_01430C_A:C5_02010C_A:RIX7:C5_03920C_A:C5_05340W_A:C6_01040C_A:C6_01890C_A:C6_02230W_A:C6_02430W_A:C6_04240W_A:NOP15:C7_01030C_A:DBP7:C7_02100W_A:TIM54:C7_03400C_A:ISY1:ENP2:TIM9:C7_03850W_A:C7_03880C_A:UTP18:BUD22:CR_01410C_A:CR_02030C_A:CR_02420W_A:RRP9:CR_03200C_A:KTI12:SGD1:CR_04110W_A:CR_04240C_A:MTG2:DBP8:SFL1:FGR50:NMD3:CR_07030C_A:IMP4:CR_08000C_A:CR_08330W_A:CR_08410W_A:TSR1:SKI2:ELF1:CR_09800C_A:UTP5:CR_10410C_A:CR_10470C_A:DRS1

42254	ribosome biogenesis	158 out of 537 genes, 29.4%	886 out of 6473 background genes, 13.7%	1.35E-20	0.00%	BFA1:CNS1:C1_01160C_A:RPS21:C1_02090C_A:PDE2:C1_02850W_A:C1_03830C_A:SEN15:C1_04040C_A:RRP6:REI1:NOP6:C1_06760C_A:C1_06800W_A:C1_07950C_A:C1_07960W_A:DIP2:MSS116:JIP5:C1_09710C_A:C1_10880W_A:C1_10950C_A:C1_10970W_A:NEP1:KRR1:C1_13820C_A:FAV3:NDT80:C2_00410C_A:C2_00820W_A:C2_00840W_A:REX2:UTP21:C2_02540W_A:C2_02710C_A:C2_04120C_A:RIM2:C2_04570W_A:C2_04820W_A:MAK5:C2_05160C_A:LAS1:MAK16:RTA2:C2_06850W_A:NOC4:RCL1:C2_07520C_A:C2_07920W_A:NSA1:KRE30:RRP8:UTP15:C2_09160W_A:RRP15:C2_09510C_A:C2_09660W_A:C2_09920W_A:PUS7:MTR2:C3_00100W_A:BUD21:C3_00420W_A:BMS1:C3_01560W_A:C3_02040C_A:UTP4:ILV2:C3_02670W_A:HBR3:CCC1:C3_04370C_A:MAK21:SFP1:CEM1:C3_05120C_A:C3_05380W_A:QDR2:C3_05800W_A:NSA2:C3_06760W_A:NOP13:C3_07550C_A:RAD4:RLP24:C4_00690C_A:RAT1:C4_01730C_A:TIM12:SAS10:HCA4:ZUO1:C4_02880C_A:NAN1:RAM1:C4_04820C_A:AGP3:C4_05010W_A:C4_05230C_A:C4_06210C_A:C4_06790W_A:C5_01430C_A:C5_02010C_A:RIX7:C5_03920C_A:C5_05340W_A:C6_01040C_A:C6_01890C_A:C6_02230W_A:C6_02430W_A:C6_04240W_A:NOP15:C7_01030C_A:DBP7:C7_02100W_A:TIM54:C7_03400C_A:ISY1:ENP2:TIM9:C7_03850W_A:C7_03880C_A:UTP18:BUD22:CR_01410C_A:CR_02030C_A:CR_02420W_A:RRP9:CR_03200C_A:CR_03240C_A:KTI12:SGD1:PRP42:CR_04110W_A:CR_04170W_A:CR_04240C_A:RPS3:MTG2:DBP8:SFL1:FGR50:NMD3:CR_07030C_A:CR_07640C_A:IMP4:CR_08000C_A:CR_08330W_A:CR_08410W_A:TSR1:CR_08500W_A:SKI2:ELF1:CR_09800C_A:UTP5:CR_10410C_A:CR_10470C_A:DRS1

30490	maturation of SSU-rRNA	126 out of 537 genes, 23.5%	627 out of 6473 background genes, 9.7%	3.62E-20	0.00%	CNS1:C1_01160C_A:C1_02090C_A:C1_02850W_A:C1_03830C_A:SEN15:C1_04040C_A:REI1:NOP6:C1_06800W_A:C1_07960W_A:DIP2:MSS116:C1_09710C_A:C1_10880W_A:C1_10950C_A:NEP1:KRR1:C1_13820C_A:FAV3:NDT80:C2_00410C_A:C2_00820W_A:C2_00840W_A:C2_02540W_A:C2_04120C_A:RIM2:C2_04570W_A:C2_04820W_A:MAK5:C2_05160C_A:LAS1:MAK16:RTA2:C2_06850W_A:NOC4:RCL1:C2_07520C_A:C2_07920W_A:NSA1:RRP8:UTP15:C2_09160W_A:RRP15:C2_09510C_A:C2_09660W_A:C2_09920W_A:C3_00100W_A:BUD21:C3_00420W_A:BMS1:C3_01560W_A:C3_02040C_A:UTP4:C3_02670W_A:HBR3:CCC1:C3_04370C_A:MAK21:SFP1:CEM1:C3_05120C_A:QDR2:C3_05800W_A:NSA2:C3_06760W_A:NOP13:C3_07550C_A:RAD4:RLP24:C4_00690C_A:RAT1:C4_01730C_A:SAS10:HCA4:C4_02880C_A:NAN1:C4_06790W_A:C5_01430C_A:C5_02010C_A:RIX7:C5_03920C_A:C5_05340W_A:C6_01040C_A:C6_01890C_A:C6_02230W_A:C6_02430W_A:C6_04240W_A:NOP15:C7_01030C_A:DBP7:C7_02100W_A:TIM54:C7_03400C_A:ISY1:ENP2:TIM9:C7_03850W_A:C7_03880C_A:UTP18:BUD22:CR_01410C_A:CR_02030C_A:CR_02420W_A:RRP9:CR_03200C_A:KTI12:CR_04110W_A:CR_04240C_A:MTG2:DBP8:SFL1:FGR50:NMD3:CR_07030C_A:IMP4:CR_08000C_A:CR_08330W_A:CR_08410W_A:TSR1:SKI2:CR_09800C_A:UTP5:CR_10410C_A:CR_10470C_A:DRS1

462	maturation of SSU-rRNA from tricistronic rRNA transcript (SSU-rRNA, 5.8S rRNA, LSU-rRNA)	125 out of 537 genes, 23.3%	620 out of 6473 background genes, 9.6%	4.17E-20	0.00%	CNS1:C1_01160C_A:C1_02090C_A:C1_02850W_A:C1_03830C_A:SEN15:C1_04040C_A:REI1:NOP6:C1_06800W_A:C1_07960W_A:DIP2:MSS116:C1_09710C_A:C1_10880W_A:C1_10950C_A:NEP1:KRR1:C1_13820C_A:FAV3:NDT80:C2_00410C_A:C2_00820W_A:C2_00840W_A:C2_02540W_A:C2_04120C_A:RIM2:C2_04570W_A:C2_04820W_A:MAK5:C2_05160C_A:LAS1:MAK16:RTA2:C2_06850W_A:NOC4:RCL1:C2_07520C_A:C2_07920W_A:NSA1:RRP8:UTP15:C2_09160W_A:RRP15:C2_09510C_A:C2_09660W_A:C2_09920W_A:C3_00100W_A:BUD21:C3_00420W_A:BMS1:C3_01560W_A:C3_02040C_A:UTP4:C3_02670W_A:HBR3:CCC1:C3_04370C_A:MAK21:SFP1:CEM1:C3_05120C_A:QDR2:C3_05800W_A:NSA2:C3_06760W_A:NOP13:C3_07550C_A:RAD4:RLP24:C4_00690C_A:RAT1:C4_01730C_A:SAS10:HCA4:C4_02880C_A:NAN1:C4_06790W_A:C5_01430C_A:C5_02010C_A:RIX7:C5_03920C_A:C5_05340W_A:C6_01040C_A:C6_01890C_A:C6_02230W_A:C6_02430W_A:C6_04240W_A:NOP15:C7_01030C_A:DBP7:C7_02100W_A:TIM54:C7_03400C_A:ISY1:ENP2:TIM9:C7_03850W_A:C7_03880C_A:UTP18:BUD22:CR_01410C_A:CR_02030C_A:CR_02420W_A:RRP9:CR_03200C_A:KTI12:CR_04110W_A:MTG2:DBP8:SFL1:FGR50:NMD3:CR_07030C_A:IMP4:CR_08000C_A:CR_08330W_A:CR_08410W_A:TSR1:SKI2:CR_09800C_A:UTP5:CR_10410C_A:CR_10470C_A:DRS1

34660	ncRNA metabolic process	162 out of 537 genes, 30.2%	935 out of 6473 background genes, 14.4%	7.65E-20	0.00%	CNS1:C1_01150C_A:C1_01160C_A:C1_02090C_A:PDE2:C1_02850W_A:C1_03830C_A:SEN15:C1_04040C_A:RRP6:REI1:NOP6:C1_06760C_A:C1_06800W_A:C1_07950C_A:C1_07960W_A:DIP2:MSS116:C1_09710C_A:C1_10880W_A:C1_10950C_A:C1_10970W_A:NEP1:KRR1:C1_13820C_A:FAV3:NDT80:C2_00410C_A:C2_00820W_A:C2_00840W_A:REX2:UTP21:C2_02540W_A:C2_02710C_A:C2_04120C_A:RIM2:C2_04570W_A:C2_04820W_A:MAK5:C2_05160C_A:RPC40:LAS1:MAK16:RTA2:C2_06480W_A:C2_06850W_A:RPA12:NOC4:RCL1:C2_07520C_A:C2_07920W_A:NSA1:RRP8:UTP15:C2_09160W_A:RRP15:C2_09500W_A:C2_09510C_A:C2_09660W_A:C2_09920W_A:PUS7:C3_00100W_A:BUD21:C3_00420W_A:BMS1:C3_01560W_A:C3_02040C_A:UTP4:ILV2:C3_02670W_A:HBR3:CCC1:C3_04370C_A:MAK21:SFP1:CEM1:C3_05120C_A:C3_05140C_A:C3_05380W_A:QDR2:C3_05800W_A:NSA2:C3_06760W_A:NOP13:C3_07550C_A:RAD4:RLP24:C4_00690C_A:RAT1:C4_01730C_A:TIM12:SAS10:HCA4:ZUO1:C4_02880C_A:NAN1:RAM1:C4_04810C_A:AGP3:C4_05230C_A:C4_06210C_A:C4_06790W_A:C5_01430C_A:C5_02010C_A:RIX7:C5_02730C_A:C5_03920C_A:MSM1:C5_05340W_A:C6_01040C_A:C6_01890C_A:C6_02230W_A:C6_02430W_A:C6_04240W_A:C6_04530C_A:NOP15:C7_01030C_A:DBP7:C7_02100W_A:C7_02340C_A:TIM54:C7_03400C_A:ISY1:ENP2:TIM9:C7_03850W_A:C7_03880C_A:UTP18:CR_00670C_A:BUD22:CR_01410C_A:CR_01950W_A:CR_02030C_A:CR_02420W_A:RRP9:DOM34:CR_03200C_A:CR_03240C_A:KTI12:PRP42:CR_04110W_A:CR_04170W_A:CR_04240C_A:MTG2:DBP8:SFL1:FGR50:NMD3:CR_07030C_A:CR_07640C_A:IMP4:CR_08000C_A:CR_08330W_A:CR_08410W_A:TSR1:SKI2:CR_08940W_A:CR_09800C_A:UTP5:CR_10410C_A:CR_10470C_A:DRS1

6364	rRNA processing	147 out of 537 genes, 27.4%	806 out of 6473 background genes, 12.5%	8.65E-20	0.00%	CNS1:C1_01160C_A:C1_02090C_A:PDE2:C1_02850W_A:C1_03830C_A:SEN15:C1_04040C_A:RRP6:REI1:NOP6:C1_06760C_A:C1_06800W_A:C1_07950C_A:C1_07960W_A:DIP2:MSS116:C1_09710C_A:C1_10880W_A:C1_10950C_A:C1_10970W_A:NEP1:KRR1:C1_13820C_A:FAV3:NDT80:C2_00410C_A:C2_00820W_A:C2_00840W_A:REX2:UTP21:C2_02540W_A:C2_02710C_A:C2_04120C_A:RIM2:C2_04570W_A:C2_04820W_A:MAK5:C2_05160C_A:LAS1:MAK16:RTA2:C2_06850W_A:NOC4:RCL1:C2_07520C_A:C2_07920W_A:NSA1:RRP8:UTP15:C2_09160W_A:RRP15:C2_09510C_A:C2_09660W_A:C2_09920W_A:PUS7:C3_00100W_A:BUD21:C3_00420W_A:BMS1:C3_01560W_A:C3_02040C_A:UTP4:ILV2:C3_02670W_A:HBR3:CCC1:C3_04370C_A:MAK21:SFP1:CEM1:C3_05120C_A:C3_05380W_A:QDR2:C3_05800W_A:NSA2:C3_06760W_A:NOP13:C3_07550C_A:RAD4:RLP24:C4_00690C_A:RAT1:C4_01730C_A:TIM12:SAS10:HCA4:ZUO1:C4_02880C_A:NAN1:RAM1:AGP3:C4_05230C_A:C4_06210C_A:C4_06790W_A:C5_01430C_A:C5_02010C_A:RIX7:C5_03920C_A:C5_05340W_A:C6_01040C_A:C6_01890C_A:C6_02230W_A:C6_02430W_A:C6_04240W_A:NOP15:C7_01030C_A:DBP7:C7_02100W_A:TIM54:C7_03400C_A:ISY1:ENP2:TIM9:C7_03850W_A:C7_03880C_A:UTP18:BUD22:CR_01410C_A:CR_02030C_A:CR_02420W_A:RRP9:CR_03200C_A:CR_03240C_A:KTI12:PRP42:CR_04110W_A:CR_04170W_A:CR_04240C_A:MTG2:DBP8:SFL1:FGR50:NMD3:CR_07030C_A:CR_07640C_A:IMP4:CR_08000C_A:CR_08330W_A:CR_08410W_A:TSR1:SKI2:CR_09800C_A:UTP5:CR_10410C_A:CR_10470C_A:DRS1

16072	rRNA metabolic process	150 out of 537 genes, 27.9%	835 out of 6473 background genes, 12.9%	1.33E-19	0.00%	CNS1:C1_01160C_A:C1_02090C_A:PDE2:C1_02850W_A:C1_03830C_A:SEN15:C1_04040C_A:RRP6:REI1:NOP6:C1_06760C_A:C1_06800W_A:C1_07950C_A:C1_07960W_A:DIP2:MSS116:C1_09710C_A:C1_10880W_A:C1_10950C_A:C1_10970W_A:NEP1:KRR1:C1_13820C_A:FAV3:NDT80:C2_00410C_A:C2_00820W_A:C2_00840W_A:REX2:UTP21:C2_02540W_A:C2_02710C_A:C2_04120C_A:RIM2:C2_04570W_A:C2_04820W_A:MAK5:C2_05160C_A:LAS1:MAK16:RTA2:C2_06850W_A:RPA12:NOC4:RCL1:C2_07520C_A:C2_07920W_A:NSA1:RRP8:UTP15:C2_09160W_A:RRP15:C2_09510C_A:C2_09660W_A:C2_09920W_A:PUS7:C3_00100W_A:BUD21:C3_00420W_A:BMS1:C3_01560W_A:C3_02040C_A:UTP4:ILV2:C3_02670W_A:HBR3:CCC1:C3_04370C_A:MAK21:SFP1:CEM1:C3_05120C_A:C3_05380W_A:QDR2:C3_05800W_A:NSA2:C3_06760W_A:NOP13:C3_07550C_A:RAD4:RLP24:C4_00690C_A:RAT1:C4_01730C_A:TIM12:SAS10:HCA4:ZUO1:C4_02880C_A:NAN1:RAM1:AGP3:C4_05230C_A:C4_06210C_A:C4_06790W_A:C5_01430C_A:C5_02010C_A:RIX7:C5_03920C_A:C5_05340W_A:C6_01040C_A:C6_01890C_A:C6_02230W_A:C6_02430W_A:C6_04240W_A:NOP15:C7_01030C_A:DBP7:C7_02100W_A:TIM54:C7_03400C_A:ISY1:ENP2:TIM9:C7_03850W_A:C7_03880C_A:UTP18:BUD22:CR_01410C_A:CR_01950W_A:CR_02030C_A:CR_02420W_A:RRP9:DOM34:CR_03200C_A:CR_03240C_A:KTI12:PRP42:CR_04110W_A:CR_04170W_A:CR_04240C_A:MTG2:DBP8:SFL1:FGR50:NMD3:CR_07030C_A:CR_07640C_A:IMP4:CR_08000C_A:CR_08330W_A:CR_08410W_A:TSR1:SKI2:CR_09800C_A:UTP5:CR_10410C_A:CR_10470C_A:DRS1

22613	ribonucleoprotein complex biogenesis	158 out of 537 genes, 29.4%	915 out of 6473 background genes, 14.1%	4.80E-19	0.00%	BFA1:CNS1:C1_01160C_A:RPS21:C1_02090C_A:PDE2:C1_02850W_A:C1_03830C_A:SEN15:C1_04040C_A:RRP6:REI1:NOP6:C1_06760C_A:C1_06800W_A:C1_07950C_A:C1_07960W_A:DIP2:MSS116:JIP5:C1_09710C_A:C1_10880W_A:C1_10950C_A:C1_10970W_A:NEP1:KRR1:C1_13820C_A:FAV3:NDT80:C2_00410C_A:C2_00820W_A:C2_00840W_A:REX2:UTP21:C2_02540W_A:C2_02710C_A:C2_04120C_A:RIM2:C2_04570W_A:C2_04820W_A:MAK5:C2_05160C_A:LAS1:MAK16:RTA2:C2_06850W_A:NOC4:RCL1:C2_07520C_A:C2_07920W_A:NSA1:KRE30:RRP8:UTP15:C2_09160W_A:RRP15:C2_09510C_A:C2_09660W_A:C2_09920W_A:PUS7:MTR2:C3_00100W_A:BUD21:C3_00420W_A:BMS1:C3_01560W_A:C3_02040C_A:UTP4:ILV2:C3_02670W_A:HBR3:CCC1:C3_04370C_A:MAK21:SFP1:CEM1:C3_05120C_A:C3_05380W_A:QDR2:C3_05800W_A:NSA2:C3_06760W_A:NOP13:C3_07550C_A:RAD4:RLP24:C4_00690C_A:RAT1:C4_01730C_A:TIM12:SAS10:HCA4:ZUO1:C4_02880C_A:NAN1:RAM1:C4_04820C_A:AGP3:C4_05010W_A:C4_05230C_A:C4_06210C_A:C4_06790W_A:C5_01430C_A:C5_02010C_A:RIX7:C5_03920C_A:C5_05340W_A:C6_01040C_A:C6_01890C_A:C6_02230W_A:C6_02430W_A:C6_04240W_A:NOP15:C7_01030C_A:DBP7:C7_02100W_A:TIM54:C7_03400C_A:ISY1:ENP2:TIM9:C7_03850W_A:C7_03880C_A:UTP18:BUD22:CR_01410C_A:CR_02030C_A:CR_02420W_A:RRP9:CR_03200C_A:CR_03240C_A:KTI12:SGD1:PRP42:CR_04110W_A:CR_04170W_A:CR_04240C_A:RPS3:MTG2:DBP8:SFL1:FGR50:NMD3:CR_07030C_A:CR_07640C_A:IMP4:CR_08000C_A:CR_08330W_A:CR_08410W_A:TSR1:CR_08500W_A:SKI2:ELF1:CR_09800C_A:UTP5:CR_10410C_A:CR_10470C_A:DRS1

6396	RNA processing	159 out of 537 genes, 29.6%	931 out of 6473 background genes, 14.4%	1.12E-18	0.00%	CNS1:C1_01150C_A:C1_01160C_A:C1_02090C_A:PDE2:C1_02850W_A:C1_03830C_A:SEN15:C1_04040C_A:RRP6:REI1:NOP6:C1_06760C_A:C1_06800W_A:C1_07950C_A:C1_07960W_A:DIP2:MSS116:C1_08890C_A:C1_09710C_A:C1_10880W_A:C1_10950C_A:C1_10970W_A:PET127:NEP1:KRR1:C1_13820C_A:FAV3:NDT80:C2_00410C_A:C2_00820W_A:C2_00840W_A:REX2:UTP21:C2_02540W_A:C2_02710C_A:C2_04120C_A:RIM2:C2_04570W_A:C2_04820W_A:MAK5:C2_05160C_A:LAS1:MAK16:RTA2:C2_06480W_A:C2_06850W_A:NOC4:RCL1:C2_07520C_A:C2_07920W_A:NSA1:RRP8:UTP15:C2_09160W_A:RRP15:C2_09500W_A:C2_09510C_A:C2_09660W_A:C2_09920W_A:PUS7:C3_00100W_A:BUD21:C3_00420W_A:BMS1:C3_01560W_A:C3_02040C_A:UTP4:ILV2:C3_02670W_A:HBR3:CCC1:C3_04370C_A:MAK21:SFP1:CEM1:C3_05120C_A:C3_05140C_A:C3_05380W_A:QDR2:C3_05800W_A:NSA2:C3_06760W_A:NOP13:C3_07550C_A:RAD4:RLP24:C4_00690C_A:RAT1:C4_01730C_A:TIM12:SAS10:HCA4:ZUO1:C4_02880C_A:NAN1:RAM1:C4_04810C_A:AGP3:C4_05230C_A:C4_06210C_A:C4_06790W_A:C5_01430C_A:C5_02010C_A:RIX7:C5_02730C_A:C5_03920C_A:C5_05340W_A:C6_01040C_A:C6_01890C_A:C6_02230W_A:C6_02430W_A:C6_04240W_A:C6_04530C_A:NOP15:C7_01030C_A:DBP7:C7_02100W_A:C7_02340C_A:TIM54:C7_03400C_A:ISY1:ENP2:TIM9:C7_03850W_A:C7_03880C_A:UTP18:CR_00670C_A:BUD22:CR_01410C_A:CR_02030C_A:CR_02420W_A:RRP9:CR_03200C_A:CR_03240C_A:KTI12:PRP42:CR_04110W_A:CR_04170W_A:CR_04240C_A:MTG2:DBP8:SFL1:FGR50:NMD3:CR_07030C_A:CR_07640C_A:IMP4:CR_08000C_A:CR_08330W_A:CR_08410W_A:TSR1:SKI2:CR_08940W_A:CR_09800C_A:UTP5:CR_10410C_A:CR_10470C_A:DRS1

966	RNA 5'-end processing	91 out of 537 genes, 16.9%	421 out of 6473 background genes, 6.5%	9.31E-16	0.00%	CNS1:C1_02090C_A:C1_02850W_A:C1_03830C_A:SEN15:C1_04040C_A:REI1:NOP6:C1_06800W_A:C1_07960W_A:DIP2:MSS116:C1_09710C_A:C1_10880W_A:C1_10950C_A:PET127:NEP1:C1_13820C_A:FAV3:NDT80:C2_00410C_A:C2_00820W_A:C2_02540W_A:C2_04120C_A:MAK5:C2_05160C_A:LAS1:MAK16:RTA2:C2_06850W_A:NOC4:RCL1:C2_07920W_A:NSA1:RRP8:RRP15:C2_09510C_A:C2_09920W_A:C3_00100W_A:BUD21:C3_00420W_A:BMS1:C3_02040C_A:C3_02670W_A:HBR3:C3_04370C_A:MAK21:SFP1:CEM1:C3_05800W_A:NSA2:C3_07550C_A:RAD4:RLP24:C4_00690C_A:RAT1:C4_01730C_A:SAS10:HCA4:C4_02880C_A:C4_06790W_A:C5_01430C_A:C5_02010C_A:RIX7:C5_03920C_A:C5_05340W_A:C6_02230W_A:C6_02430W_A:C6_04240W_A:DBP7:C7_03400C_A:ISY1:C7_03850W_A:C7_03880C_A:UTP18:CR_01410C_A:CR_02030C_A:CR_02420W_A:RRP9:CR_03200C_A:CR_04110W_A:DBP8:SFL1:FGR50:IMP4:CR_08000C_A:CR_08330W_A:CR_08410W_A:CR_09800C_A:CR_10410C_A:DRS1

472	endonucleolytic cleavage to generate mature 5'-end of SSU-rRNA from (SSU-rRNA, 5.8S rRNA, LSU-rRNA)	90 out of 537 genes, 16.8%	417 out of 6473 background genes, 6.4%	1.66E-15	0.00%	CNS1:C1_02090C_A:C1_02850W_A:C1_03830C_A:SEN15:C1_04040C_A:REI1:NOP6:C1_06800W_A:C1_07960W_A:DIP2:MSS116:C1_09710C_A:C1_10880W_A:C1_10950C_A:NEP1:C1_13820C_A:FAV3:NDT80:C2_00410C_A:C2_00820W_A:C2_02540W_A:C2_04120C_A:MAK5:C2_05160C_A:LAS1:MAK16:RTA2:C2_06850W_A:NOC4:RCL1:C2_07920W_A:NSA1:RRP8:RRP15:C2_09510C_A:C2_09920W_A:C3_00100W_A:BUD21:C3_00420W_A:BMS1:C3_02040C_A:C3_02670W_A:HBR3:C3_04370C_A:MAK21:SFP1:CEM1:C3_05800W_A:NSA2:C3_07550C_A:RAD4:RLP24:C4_00690C_A:RAT1:C4_01730C_A:SAS10:HCA4:C4_02880C_A:C4_06790W_A:C5_01430C_A:C5_02010C_A:RIX7:C5_03920C_A:C5_05340W_A:C6_02230W_A:C6_02430W_A:C6_04240W_A:DBP7:C7_03400C_A:ISY1:C7_03850W_A:C7_03880C_A:UTP18:CR_01410C_A:CR_02030C_A:CR_02420W_A:RRP9:CR_03200C_A:CR_04110W_A:DBP8:SFL1:FGR50:IMP4:CR_08000C_A:CR_08330W_A:CR_08410W_A:CR_09800C_A:CR_10410C_A:DRS1

967	rRNA 5'-end processing	90 out of 537 genes, 16.8%	419 out of 6473 background genes, 6.5%	2.32E-15	0.00%	CNS1:C1_02090C_A:C1_02850W_A:C1_03830C_A:SEN15:C1_04040C_A:REI1:NOP6:C1_06800W_A:C1_07960W_A:DIP2:MSS116:C1_09710C_A:C1_10880W_A:C1_10950C_A:NEP1:C1_13820C_A:FAV3:NDT80:C2_00410C_A:C2_00820W_A:C2_02540W_A:C2_04120C_A:MAK5:C2_05160C_A:LAS1:MAK16:RTA2:C2_06850W_A:NOC4:RCL1:C2_07920W_A:NSA1:RRP8:RRP15:C2_09510C_A:C2_09920W_A:C3_00100W_A:BUD21:C3_00420W_A:BMS1:C3_02040C_A:C3_02670W_A:HBR3:C3_04370C_A:MAK21:SFP1:CEM1:C3_05800W_A:NSA2:C3_07550C_A:RAD4:RLP24:C4_00690C_A:RAT1:C4_01730C_A:SAS10:HCA4:C4_02880C_A:C4_06790W_A:C5_01430C_A:C5_02010C_A:RIX7:C5_03920C_A:C5_05340W_A:C6_02230W_A:C6_02430W_A:C6_04240W_A:DBP7:C7_03400C_A:ISY1:C7_03850W_A:C7_03880C_A:UTP18:CR_01410C_A:CR_02030C_A:CR_02420W_A:RRP9:CR_03200C_A:CR_04110W_A:DBP8:SFL1:FGR50:IMP4:CR_08000C_A:CR_08330W_A:CR_08410W_A:CR_09800C_A:CR_10410C_A:DRS1

34471	ncRNA 5'-end processing	90 out of 537 genes, 16.8%	420 out of 6473 background genes, 6.5%	2.74E-15	0.00%	CNS1:C1_02090C_A:C1_02850W_A:C1_03830C_A:SEN15:C1_04040C_A:REI1:NOP6:C1_06800W_A:C1_07960W_A:DIP2:MSS116:C1_09710C_A:C1_10880W_A:C1_10950C_A:NEP1:C1_13820C_A:FAV3:NDT80:C2_00410C_A:C2_00820W_A:C2_02540W_A:C2_04120C_A:MAK5:C2_05160C_A:LAS1:MAK16:RTA2:C2_06850W_A:NOC4:RCL1:C2_07920W_A:NSA1:RRP8:RRP15:C2_09510C_A:C2_09920W_A:C3_00100W_A:BUD21:C3_00420W_A:BMS1:C3_02040C_A:C3_02670W_A:HBR3:C3_04370C_A:MAK21:SFP1:CEM1:C3_05800W_A:NSA2:C3_07550C_A:RAD4:RLP24:C4_00690C_A:RAT1:C4_01730C_A:SAS10:HCA4:C4_02880C_A:C4_06790W_A:C5_01430C_A:C5_02010C_A:RIX7:C5_03920C_A:C5_05340W_A:C6_02230W_A:C6_02430W_A:C6_04240W_A:DBP7:C7_03400C_A:ISY1:C7_03850W_A:C7_03880C_A:UTP18:CR_01410C_A:CR_02030C_A:CR_02420W_A:RRP9:CR_03200C_A:CR_04110W_A:DBP8:SFL1:FGR50:IMP4:CR_08000C_A:CR_08330W_A:CR_08410W_A:CR_09800C_A:CR_10410C_A:DRS1

36260	RNA capping	90 out of 537 genes, 16.8%	427 out of 6473 background genes, 6.6%	8.58E-15	0.00%	CNS1:C1_02090C_A:C1_02850W_A:C1_03830C_A:SEN15:C1_04040C_A:REI1:NOP6:C1_06800W_A:C1_07960W_A:DIP2:MSS116:C1_09710C_A:C1_10880W_A:C1_10950C_A:NEP1:C1_13820C_A:FAV3:NDT80:C2_00410C_A:C2_00820W_A:C2_02540W_A:C2_04120C_A:MAK5:C2_05160C_A:LAS1:MAK16:RTA2:C2_06850W_A:NOC4:RCL1:C2_07920W_A:NSA1:RRP8:RRP15:C2_09510C_A:C2_09920W_A:C3_00100W_A:BUD21:C3_00420W_A:BMS1:C3_02040C_A:C3_02670W_A:HBR3:C3_04370C_A:MAK21:SFP1:CEM1:C3_05800W_A:NSA2:C3_07550C_A:RAD4:RLP24:C4_00690C_A:RAT1:C4_01730C_A:SAS10:HCA4:C4_02880C_A:C4_06790W_A:C5_01430C_A:C5_02010C_A:RIX7:C5_03920C_A:C5_05340W_A:C6_02230W_A:C6_02430W_A:C6_04240W_A:DBP7:C7_03400C_A:ISY1:C7_03850W_A:C7_03880C_A:UTP18:CR_01410C_A:CR_02030C_A:CR_02420W_A:RRP9:CR_03200C_A:CR_04110W_A:DBP8:SFL1:FGR50:IMP4:CR_08000C_A:CR_08330W_A:CR_08410W_A:CR_09800C_A:CR_10410C_A:DRS1

479	endonucleolytic cleavage of tricistronic rRNA transcript (SSU-rRNA, 5.8S rRNA, LSU-rRNA)	95 out of 537 genes, 17.7%	473 out of 6473 background genes, 7.3%	2.76E-14	0.00%	CNS1:C1_02090C_A:C1_02850W_A:C1_03830C_A:SEN15:C1_04040C_A:REI1:NOP6:C1_06800W_A:C1_07960W_A:DIP2:MSS116:C1_09710C_A:C1_10880W_A:C1_10950C_A:NEP1:KRR1:C1_13820C_A:FAV3:NDT80:C2_00410C_A:C2_00820W_A:C2_02540W_A:C2_04120C_A:MAK5:C2_05160C_A:LAS1:MAK16:RTA2:C2_06850W_A:NOC4:RCL1:C2_07920W_A:NSA1:RRP8:RRP15:C2_09510C_A:C2_09920W_A:C3_00100W_A:BUD21:C3_00420W_A:BMS1:C3_02040C_A:C3_02670W_A:HBR3:CCC1:C3_04370C_A:MAK21:SFP1:CEM1:QDR2:C3_05800W_A:NSA2:C3_07550C_A:RAD4:RLP24:C4_00690C_A:RAT1:C4_01730C_A:SAS10:HCA4:C4_02880C_A:C4_06790W_A:C5_01430C_A:C5_02010C_A:RIX7:C5_03920C_A:C5_05340W_A:C6_02230W_A:C6_02430W_A:C6_04240W_A:DBP7:C7_03400C_A:ISY1:C7_03850W_A:C7_03880C_A:UTP18:CR_01410C_A:CR_02030C_A:CR_02420W_A:RRP9:CR_03200C_A:CR_04110W_A:DBP8:SFL1:FGR50:IMP4:CR_08000C_A:CR_08330W_A:CR_08410W_A:TSR1:SKI2:CR_09800C_A:CR_10410C_A:DRS1

16070	RNA metabolic process	176 out of 537 genes, 32.8%	1245 out of 6473 background genes, 19.2%	4.14E-12	0.00%	BFA1:CNS1:C1_01150C_A:C1_01160C_A:C1_02090C_A:PDE2:C1_02850W_A:C1_03830C_A:SEN15:C1_04040C_A:PSF2:RRP6:REI1:NOP6:C1_06760C_A:C1_06800W_A:C1_07950C_A:C1_07960W_A:DIP2:MSS116:C1_08890C_A:C1_09710C_A:C1_10880W_A:C1_10950C_A:C1_10970W_A:PET127:NEP1:KRR1:C1_13820C_A:FAV3:NDT80:C2_00410C_A:C2_00820W_A:C2_00840W_A:REX2:C2_01870C_A:UTP21:C2_02540W_A:C2_02710C_A:C2_04120C_A:RIM2:C2_04570W_A:C2_04820W_A:CRK1:MAK5:C2_05160C_A:RPC40:LAS1:MAK16:RTA2:C2_06480W_A:C2_06850W_A:RPA12:NOC4:RCL1:C2_07520C_A:C2_07630C_A:C2_07920W_A:NSA1:RRP8:UTP15:C2_09160W_A:RRP15:C2_09500W_A:C2_09510C_A:C2_09660W_A:C2_09920W_A:PUS7:C3_00100W_A:BUD21:C3_00420W_A:BMS1:C3_01560W_A:C3_02040C_A:UTP4:ILV2:C3_02670W_A:HBR3:CCC1:C3_04370C_A:MAK21:SFP1:CEM1:C3_05120C_A:C3_05140C_A:C3_05380W_A:QDR2:C3_05800W_A:CTA4:C3_06350W_A:NSA2:C3_06760W_A:NOP13:C3_07550C_A:RAD4:RLP24:C4_00690C_A:RAT1:C4_01730C_A:TIM12:ZCF25:SAS10:HCA4:ZUO1:C4_02880C_A:NAN1:RAM1:C4_04810C_A:AGP3:C4_05230C_A:OFD1:C4_06210C_A:C4_06790W_A:C5_01430C_A:C5_02010C_A:RIX7:C5_02730C_A:C5_03920C_A:MSM1:C5_05340W_A:C6_00640C_A:C6_01040C_A:C6_01890C_A:C6_02230W_A:C6_02430W_A:FGR17:C6_04240W_A:C6_04530C_A:NOP15:C7_01030C_A:DBP7:C7_02100W_A:C7_02340C_A:TIM54:C7_03400C_A:ISY1:ENP2:TIM9:C7_03850W_A:C7_03880C_A:UTP18:CR_00670C_A:BUD22:CR_01410C_A:CR_01950W_A:CR_02030C_A:CR_02420W_A:RRP9:DOM34:CR_03200C_A:CR_03240C_A:KTI12:SGD1:PRP42:CR_04110W_A:CR_04170W_A:CR_04240C_A:MTG2:DBP8:SFL1:FGR50:NMD3:CR_07030C_A:CR_07640C_A:IMP4:CR_08000C_A:CR_08330W_A:CR_08410W_A:TSR1:SKI2:CR_08940W_A:CR_09800C_A:UTP5:CR_10410C_A:CR_10470C_A:DRS1

460	maturation of 5.8S rRNA	43 out of 537 genes, 8.0%	144 out of 6473 background genes, 2.2%	2.35E-11	0.00%	C1_01160C_A:C1_03830C_A:C1_04040C_A:RRP6:C1_06800W_A:C1_07960W_A:DIP2:C1_09710C_A:C1_10880W_A:NEP1:KRR1:C1_13820C_A:FAV3:C2_00410C_A:C2_00820W_A:REX2:C2_02540W_A:MAK5:LAS1:MAK16:NOC4:RCL1:C2_09160W_A:RRP15:BUD21:C3_01560W_A:C3_02040C_A:NSA2:RAD4:C4_00690C_A:RAT1:SAS10:C5_03920C_A:C7_03400C_A:C7_03850W_A:UTP18:CR_02420W_A:RRP9:CR_03200C_A:DBP8:CR_08330W_A:CR_09800C_A:CR_10410C_A

466	maturation of 5.8S rRNA from tricistronic rRNA transcript (SSU-rRNA, 5.8S rRNA, LSU-rRNA)	43 out of 537 genes, 8.0%	144 out of 6473 background genes, 2.2%	2.35E-11	0.00%	C1_01160C_A:C1_03830C_A:C1_04040C_A:RRP6:C1_06800W_A:C1_07960W_A:DIP2:C1_09710C_A:C1_10880W_A:NEP1:KRR1:C1_13820C_A:FAV3:C2_00410C_A:C2_00820W_A:REX2:C2_02540W_A:MAK5:LAS1:MAK16:NOC4:RCL1:C2_09160W_A:RRP15:BUD21:C3_01560W_A:C3_02040C_A:NSA2:RAD4:C4_00690C_A:RAT1:SAS10:C5_03920C_A:C7_03400C_A:C7_03850W_A:UTP18:CR_02420W_A:RRP9:CR_03200C_A:DBP8:CR_08330W_A:CR_09800C_A:CR_10410C_A

42273	ribosomal large subunit biogenesis	97 out of 537 genes, 18.1%	546 out of 6473 background genes, 8.4%	5.42E-11	0.00%	BFA1:C1_02090C_A:PDE2:C1_02850W_A:C1_03830C_A:SEN15:C1_04040C_A:RRP6:REI1:NOP6:C1_06760C_A:C1_06800W_A:C1_07960W_A:JIP5:C1_09710C_A:C1_10970W_A:C2_00820W_A:C2_02540W_A:C2_04120C_A:C2_04570W_A:MAK5:C2_05160C_A:LAS1:MAK16:C2_06850W_A:NSA1:RRP8:UTP15:C2_09160W_A:RRP15:C2_09660W_A:C2_09920W_A:C3_00420W_A:BMS1:C3_01560W_A:C3_02040C_A:ILV2:C3_02670W_A:C3_04370C_A:MAK21:SFP1:CEM1:C3_05120C_A:C3_05380W_A:QDR2:C3_05800W_A:NSA2:C3_07550C_A:RAD4:RLP24:RAT1:C4_01730C_A:TIM12:C4_02880C_A:RAM1:AGP3:C4_05010W_A:C4_05230C_A:C4_06210C_A:C4_06790W_A:C5_01430C_A:C5_02010C_A:RIX7:C5_03920C_A:C5_05340W_A:C6_01890C_A:C6_02230W_A:C6_02430W_A:C6_04240W_A:NOP15:DBP7:C7_03400C_A:ISY1:ENP2:TIM9:C7_03850W_A:C7_03880C_A:UTP18:BUD22:CR_01410C_A:CR_02030C_A:CR_03200C_A:CR_03240C_A:SGD1:PRP42:CR_04170W_A:SFL1:CR_07640C_A:CR_08000C_A:CR_08330W_A:CR_08410W_A:TSR1:CR_08500W_A:ELF1:CR_09800C_A:CR_10470C_A:DRS1

480	endonucleolytic cleavage in 5'-ETS of tricistronic rRNA transcript (SSU-rRNA, 5.8S rRNA, LSU-rRNA)	74 out of 537 genes, 13.8%	390 out of 6473 background genes, 6.0%	3.12E-09	0.00%	CNS1:C1_02090C_A:C1_02850W_A:C1_03830C_A:SEN15:C1_04040C_A:REI1:NOP6:C1_06800W_A:C1_07960W_A:DIP2:C1_09710C_A:C1_10880W_A:NEP1:C1_13820C_A:FAV3:NDT80:C2_00410C_A:C2_00820W_A:C2_02540W_A:C2_04120C_A:C2_05160C_A:LAS1:RTA2:C2_06850W_A:NOC4:RCL1:C2_07920W_A:NSA1:RRP8:RRP15:C2_09510C_A:C3_00100W_A:BUD21:C3_00420W_A:BMS1:C3_02040C_A:C3_02670W_A:CCC1:C3_04370C_A:SFP1:QDR2:C3_05800W_A:C3_07550C_A:RAD4:C4_00690C_A:RAT1:C4_01730C_A:SAS10:C4_02880C_A:C4_06790W_A:C5_01430C_A:C5_02010C_A:RIX7:C5_03920C_A:C5_05340W_A:C6_02230W_A:C6_02430W_A:C6_04240W_A:C7_03400C_A:C7_03850W_A:UTP18:CR_02030C_A:CR_02420W_A:RRP9:CR_03200C_A:DBP8:SFL1:IMP4:CR_08000C_A:CR_08330W_A:CR_08410W_A:SKI2:CR_09800C_A

27	ribosomal large subunit assembly	33 out of 537 genes, 6.1%	105 out of 6473 background genes, 1.6%	5.71E-09	0.00%	C1_04040C_A:RRP6:C1_06760C_A:JIP5:C1_09710C_A:C1_10970W_A:C2_02540W_A:MAK5:C2_05160C_A:BMS1:C3_01560W_A:C3_02040C_A:C3_02670W_A:C3_04370C_A:MAK21:QDR2:C4_05010W_A:C4_05230C_A:C5_05340W_A:C6_02230W_A:DBP7:C7_03400C_A:ENP2:UTP18:BUD22:CR_01410C_A:SGD1:CR_04170W_A:TSR1:ELF1:CR_09800C_A:CR_10470C_A:DRS1
42255	ribosome assembly	35 out of 537 genes, 6.5%	125 out of 6473 background genes, 1.9%	5.10E-08	0.00%	C1_04040C_A:RRP6:C1_06760C_A:JIP5:C1_09710C_A:C1_10970W_A:C2_02540W_A:C2_02710C_A:MAK5:C2_05160C_A:BMS1:C3_01560W_A:C3_02040C_A:C3_02670W_A:C3_04370C_A:MAK21:QDR2:C4_02880C_A:C4_05010W_A:C4_05230C_A:C5_05340W_A:C6_02230W_A:DBP7:C7_03400C_A:ENP2:UTP18:BUD22:CR_01410C_A:SGD1:CR_04170W_A:TSR1:ELF1:CR_09800C_A:CR_10470C_A:DRS1

6139	nucleobase-containing compound metabolic process	200 out of 537 genes, 37.2%	1659 out of 6473 background genes, 25.6%	3.57E-07	0.00%	BFA1:CNS1:C1_01150C_A:C1_01160C_A:C1_02090C_A:RAD16:PDE2:C1_02850W_A:C1_03830C_A:SEN15:C1_04040C_A:PSF2:RRP6:REI1:C1_05270C_A:RAD10:C1_06120C_A:NOP6:C1_06760C_A:C1_06800W_A:C1_07950C_A:C1_07960W_A:DIP2:MSS116:C1_08890C_A:C1_09710C_A:URA1:HNT1:C1_10880W_A:C1_10950C_A:C1_10970W_A:PET127:NEP1:KRR1:OSM2:C1_13820C_A:FAV3:NDT80:C2_00180C_A:C2_00410C_A:C2_00820W_A:C2_00840W_A:REX2:C2_01870C_A:UTP21:C2_02540W_A:C2_02710C_A:C2_04120C_A:RIM2:C2_04570W_A:C2_04820W_A:CRK1:MAK5:C2_05160C_A:RPC40:LAS1:MAK16:RTA2:C2_06480W_A:C2_06850W_A:AAH1:RPA12:NOC4:RCL1:C2_07520C_A:RNR22:C2_07630C_A:C2_07920W_A:NSA1:ORF298:C2_08460C_A:RRP8:UTP15:C2_09160W_A:RRP15:C2_09500W_A:C2_09510C_A:C2_09660W_A:C2_09920W_A:PUS7:C3_00100W_A:BUD21:C3_00420W_A:BMS1:C3_01560W_A:C3_02040C_A:UTP4:ILV2:C3_02670W_A:HBR3:CCC1:C3_04370C_A:MAK21:SFP1:CEM1:C3_05120C_A:C3_05140C_A:C3_05380W_A:QDR2:C3_05800W_A:CTA4:C3_06350W_A:NSA2:C3_06760W_A:NOP13:C3_07550C_A:RAD4:RLP24:C4_00690C_A:C4_00700C_A:RAT1:C4_01730C_A:TIM12:ZCF25:SAS10:HCA4:ZUO1:C4_02880C_A:NAN1:RAM1:C4_04810C_A:AGP3:C4_05230C_A:OFD1:C4_06210C_A:C4_06790W_A:C5_00800C_A:C5_01140C_A:C5_01430C_A:C5_02010C_A:RIX7:C5_02730C_A:FUR1:C5_03920C_A:C5_04360C_A:MSM1:C5_05130C_A:C5_05340W_A:C6_00640C_A:C6_01040C_A:C6_01890C_A:C6_02230W_A:C6_02410W_A:C6_02430W_A:C6_02560W_A:FGR17:C6_04240W_A:C6_04530C_A:NOP15:C7_01030C_A:DBP7:C7_02100W_A:C7_02340C_A:TIM54:DFR1:C7_03400C_A:PNC1:ISY1:ENP2:TIM9:C7_03850W_A:C7_03880C_A:UTP18:CR_00670C_A:BUD22:CR_01410C_A:CR_01950W_A:CR_02030C_A:CR_02420W_A:RRP9:DOM34:CR_03200C_A:CR_03240C_A:KTI12:SGD1:PRP42:CR_04110W_A:CR_04170W_A:CR_04240C_A:MTG2:DBP8:SFL1:FGR50:NMD3:CR_07030C_A:URA2:CR_07640C_A:IMP4:CR_08000C_A:CR_08330W_A:CR_08410W_A:TSR1:SKI2:CR_08940W_A:CR_09140C_A:CR_09800C_A:UTP5:CR_10410C_A:CR_10470C_A:DRS1

46483	heterocycle metabolic process	205 out of 537 genes, 38.2%	1733 out of 6473 background genes, 26.8%	1.09E-06	0.00%	BFA1:CNS1:C1_01150C_A:C1_01160C_A:C1_02090C_A:RAD16:PDE2:C1_02850W_A:C1_03180W_A:C1_03830C_A:SEN15:C1_04040C_A:PSF2:RRP6:REI1:C1_05270C_A:RAD10:C1_06120C_A:NOP6:C1_06760C_A:C1_06800W_A:C1_07950C_A:C1_07960W_A:DIP2:MSS116:C1_08890C_A:C1_09710C_A:URA1:HNT1:C1_10880W_A:C1_10950C_A:C1_10970W_A:PET127:NEP1:KRR1:RIB3:OSM2:C1_13820C_A:FAV3:NDT80:C2_00180C_A:C2_00410C_A:PHHB:C2_00820W_A:C2_00840W_A:REX2:C2_01870C_A:UTP21:C2_02540W_A:C2_02710C_A:C2_04120C_A:RIM2:C2_04570W_A:C2_04820W_A:CRK1:MAK5:C2_05160C_A:RPC40:LAS1:MAK16:RTA2:C2_06480W_A:C2_06850W_A:AAH1:RPA12:NOC4:RCL1:C2_07520C_A:RNR22:C2_07630C_A:C2_07920W_A:NSA1:ORF298:C2_08460C_A:RRP8:UTP15:C2_09160W_A:RRP15:C2_09500W_A:C2_09510C_A:C2_09660W_A:C2_09920W_A:PUS7:C3_00100W_A:BUD21:C3_00420W_A:BMS1:C3_01560W_A:C3_02040C_A:UTP4:ILV2:C3_02670W_A:DAL4:YAH1:HBR3:CCC1:C3_04370C_A:MAK21:SFP1:CEM1:C3_05120C_A:C3_05140C_A:C3_05380W_A:QDR2:C3_05800W_A:CTA4:C3_06350W_A:NSA2:C3_06760W_A:NOP13:C3_07550C_A:RAD4:RLP24:C4_00690C_A:C4_00700C_A:RAT1:C4_01730C_A:TIM12:ZCF25:SAS10:HCA4:ZUO1:C4_02880C_A:NAN1:RAM1:C4_04810C_A:AGP3:C4_05230C_A:OFD1:C4_06210C_A:C4_06790W_A:C5_00800C_A:C5_01140C_A:C5_01430C_A:C5_02010C_A:RIX7:C5_02730C_A:FUR1:C5_03920C_A:C5_04360C_A:MSM1:C5_05130C_A:C5_05340W_A:C6_00640C_A:C6_01040C_A:C6_01890C_A:C6_02230W_A:C6_02410W_A:C6_02430W_A:C6_02560W_A:FGR17:C6_04240W_A:C6_04530C_A:NOP15:C7_01030C_A:DBP7:C7_02100W_A:C7_02340C_A:TIM54:DFR1:C7_03400C_A:PNC1:ISY1:ENP2:TIM9:C7_03850W_A:C7_03880C_A:UTP18:CR_00670C_A:BUD22:CR_01410C_A:CR_01950W_A:CR_02030C_A:CR_02420W_A:RRP9:DOM34:CR_03200C_A:CR_03240C_A:KTI12:SGD1:PRP42:CR_04110W_A:CR_04170W_A:CR_04240C_A:MTG2:DBP8:SFL1:FGR50:NMD3:CR_07030C_A:URA2:CR_07640C_A:IMP4:CR_08000C_A:CR_08330W_A:CR_08410W_A:TSR1:SKI2:CR_08940W_A:CR_09140C_A:CR_09800C_A:UTP5:CR_10410C_A:CR_10470C_A:DRS1

90304	nucleic acid metabolic process	184 out of 537 genes, 34.3%	1509 out of 6473 background genes, 23.3%	1.15E-06	0.00%	BFA1:CNS1:C1_01150C_A:C1_01160C_A:C1_02090C_A:RAD16:PDE2:C1_02850W_A:C1_03830C_A:SEN15:C1_04040C_A:PSF2:RRP6:REI1:C1_05270C_A:RAD10:C1_06120C_A:NOP6:C1_06760C_A:C1_06800W_A:C1_07950C_A:C1_07960W_A:DIP2:MSS116:C1_08890C_A:C1_09710C_A:C1_10880W_A:C1_10950C_A:C1_10970W_A:PET127:NEP1:KRR1:C1_13820C_A:FAV3:NDT80:C2_00410C_A:C2_00820W_A:C2_00840W_A:REX2:C2_01870C_A:UTP21:C2_02540W_A:C2_02710C_A:C2_04120C_A:RIM2:C2_04570W_A:C2_04820W_A:CRK1:MAK5:C2_05160C_A:RPC40:LAS1:MAK16:RTA2:C2_06480W_A:C2_06850W_A:RPA12:NOC4:RCL1:C2_07520C_A:C2_07630C_A:C2_07920W_A:NSA1:ORF298:RRP8:UTP15:C2_09160W_A:RRP15:C2_09500W_A:C2_09510C_A:C2_09660W_A:C2_09920W_A:PUS7:C3_00100W_A:BUD21:C3_00420W_A:BMS1:C3_01560W_A:C3_02040C_A:UTP4:ILV2:C3_02670W_A:HBR3:CCC1:C3_04370C_A:MAK21:SFP1:CEM1:C3_05120C_A:C3_05140C_A:C3_05380W_A:QDR2:C3_05800W_A:CTA4:C3_06350W_A:NSA2:C3_06760W_A:NOP13:C3_07550C_A:RAD4:RLP24:C4_00690C_A:C4_00700C_A:RAT1:C4_01730C_A:TIM12:ZCF25:SAS10:HCA4:ZUO1:C4_02880C_A:NAN1:RAM1:C4_04810C_A:AGP3:C4_05230C_A:OFD1:C4_06210C_A:C4_06790W_A:C5_01140C_A:C5_01430C_A:C5_02010C_A:RIX7:C5_02730C_A:C5_03920C_A:MSM1:C5_05340W_A:C6_00640C_A:C6_01040C_A:C6_01890C_A:C6_02230W_A:C6_02430W_A:FGR17:C6_04240W_A:C6_04530C_A:NOP15:C7_01030C_A:DBP7:C7_02100W_A:C7_02340C_A:TIM54:C7_03400C_A:ISY1:ENP2:TIM9:C7_03850W_A:C7_03880C_A:UTP18:CR_00670C_A:BUD22:CR_01410C_A:CR_01950W_A:CR_02030C_A:CR_02420W_A:RRP9:DOM34:CR_03200C_A:CR_03240C_A:KTI12:SGD1:PRP42:CR_04110W_A:CR_04170W_A:CR_04240C_A:MTG2:DBP8:SFL1:FGR50:NMD3:CR_07030C_A:CR_07640C_A:IMP4:CR_08000C_A:CR_08330W_A:CR_08410W_A:TSR1:SKI2:CR_08940W_A:CR_09140C_A:CR_09800C_A:UTP5:CR_10410C_A:CR_10470C_A:DRS1

6725	cellular aromatic compound metabolic process	203 out of 537 genes, 37.8%	1727 out of 6473 background genes, 26.7%	2.53E-06	0.00%	BFA1:CNS1:C1_01150C_A:C1_01160C_A:C1_02090C_A:RAD16:PDE2:C1_02850W_A:C1_03830C_A:SEN15:C1_04040C_A:PSF2:RRP6:REI1:C1_05270C_A:RAD10:C1_06120C_A:NOP6:C1_06760C_A:C1_06800W_A:C1_07950C_A:C1_07960W_A:DIP2:MSS116:C1_08890C_A:C1_09710C_A:URA1:HNT1:C1_10880W_A:C1_10950C_A:C1_10970W_A:PET127:NEP1:KRR1:OSM2:C1_13820C_A:FAV3:NDT80:C2_00180C_A:C2_00410C_A:PHHB:C2_00820W_A:C2_00840W_A:REX2:C2_01870C_A:UTP21:C2_02540W_A:C2_02710C_A:C2_04120C_A:RIM2:C2_04570W_A:C2_04820W_A:CRK1:MAK5:C2_05160C_A:RPC40:LAS1:MAK16:RTA2:C2_06480W_A:C2_06850W_A:AAH1:RPA12:NOC4:RCL1:C2_07520C_A:RNR22:C2_07630C_A:C2_07920W_A:NSA1:ORF298:C2_08460C_A:RRP8:UTP15:C2_09160W_A:RRP15:C2_09500W_A:C2_09510C_A:C2_09660W_A:C2_09920W_A:PUS7:C3_00100W_A:BUD21:C3_00420W_A:BMS1:C3_01560W_A:C3_02040C_A:UTP4:ILV2:C3_02670W_A:YAH1:HBR3:CCC1:C3_04370C_A:MAK21:SFP1:CEM1:C3_05120C_A:C3_05140C_A:C3_05380W_A:QDR2:C3_05800W_A:CTA4:C3_06350W_A:NSA2:C3_06760W_A:NOP13:C3_07550C_A:RAD4:RLP24:C4_00690C_A:C4_00700C_A:RAT1:C4_01730C_A:TIM12:ZCF25:SAS10:HCA4:ZUO1:C4_02880C_A:NAN1:RAM1:C4_04810C_A:AGP3:C4_05230C_A:OFD1:C4_06210C_A:C4_06790W_A:C5_00800C_A:C5_01140C_A:C5_01430C_A:C5_02010C_A:RIX7:C5_02730C_A:FUR1:C5_03920C_A:C5_04360C_A:MSM1:C5_05130C_A:C5_05340W_A:C6_00640C_A:C6_01040C_A:C6_01890C_A:C6_02230W_A:C6_02410W_A:C6_02430W_A:C6_02560W_A:FGR17:PAD1:C6_04240W_A:C6_04530C_A:NOP15:C7_01030C_A:DBP7:C7_02100W_A:C7_02340C_A:TIM54:DFR1:C7_03400C_A:PNC1:ISY1:ENP2:TIM9:C7_03850W_A:C7_03880C_A:UTP18:CR_00670C_A:BUD22:CR_01410C_A:CR_01950W_A:CR_02030C_A:CR_02420W_A:RRP9:DOM34:CR_03200C_A:CR_03240C_A:KTI12:SGD1:PRP42:CR_04110W_A:CR_04170W_A:CR_04240C_A:MTG2:DBP8:SFL1:FGR50:NMD3:CR_07030C_A:URA2:CR_07640C_A:IMP4:CR_08000C_A:CR_08330W_A:CR_08410W_A:TSR1:SKI2:CR_08940W_A:CR_09140C_A:CR_09800C_A:UTP5:CR_10410C_A:CR_10470C_A:DRS1

44085	cellular component biogenesis	165 out of 537 genes, 30.7%	1340 out of 6473 background genes, 20.7%	6.72E-06	0.00%	BFA1:CNS1:C1_01160C_A:RPS21:C1_02090C_A:PDE2:C1_02850W_A:C1_03830C_A:SEN15:C1_04040C_A:RRP6:REI1:NOP6:C1_06760C_A:C1_06800W_A:C1_07950C_A:C1_07960W_A:DIP2:MSS116:JIP5:C1_09710C_A:C1_10880W_A:C1_10950C_A:C1_10970W_A:NEP1:KRR1:C1_13820C_A:SIM1:FAV3:NDT80:C2_00410C_A:C2_00820W_A:C2_00840W_A:REX2:UTP21:C2_02540W_A:C2_02710C_A:MNN42:C2_04120C_A:RIM2:C2_04570W_A:C2_04820W_A:MAK5:C2_05160C_A:LAS1:MAK16:RTA2:C2_06850W_A:NOC4:RCL1:C2_07520C_A:C2_07920W_A:NSA1:KRE30:RRP8:UTP15:C2_09160W_A:RRP15:C2_09510C_A:C2_09660W_A:C2_09920W_A:PUS7:MTR2:C3_00100W_A:BUD21:C3_00420W_A:NDU1:BMS1:C3_01560W_A:C3_02040C_A:UTP4:ILV2:C3_02670W_A:YAH1:HBR3:CCC1:C3_04370C_A:MAK21:SFP1:CEM1:C3_05120C_A:C3_05380W_A:QDR2:C3_05800W_A:C3_06350W_A:NSA2:C3_06760W_A:NOP13:C3_07550C_A:RAD4:RLP24:C4_00690C_A:RAT1:C4_01730C_A:TIM12:SAS10:HCA4:ZUO1:C4_02880C_A:NAN1:RAM1:C4_04820C_A:AGP3:C4_05010W_A:C4_05230C_A:C4_06210C_A:C4_06790W_A:C5_01430C_A:C5_02010C_A:RIX7:C5_03800W_A:C5_03920C_A:C5_05340W_A:C6_01040C_A:C6_01890C_A:C6_02230W_A:C6_02430W_A:C6_04240W_A:NOP15:C7_01030C_A:DBP7:C7_02100W_A:TIM54:C7_03400C_A:ISY1:ENP2:TIM9:C7_03850W_A:C7_03880C_A:UTP18:BUD22:CR_01410C_A:CR_02030C_A:CR_02420W_A:RRP9:CR_03200C_A:CR_03240C_A:KTI12:SGD1:PRP42:CR_04110W_A:CR_04170W_A:QDR1:CR_04240C_A:RPS3:MTG2:DBP8:SFL1:FGR50:NMD3:CR_07030C_A:CR_07640C_A:IMP4:CR_08000C_A:CR_08330W_A:CR_08410W_A:TSR1:CR_08500W_A:SKI2:ELF1:CR_09800C_A:UTP5:CR_10410C_A:CR_10470C_A:DRS1

447	endonucleolytic cleavage in ITS1 to separate SSU-rRNA from 5.8S rRNA and LSU-rRNA from tricistronic rRNA transcript (SSU-rRNA, 5.8S rRNA, LSU-rRNA)	28 out of 537 genes, 5.2%	103 out of 6473 background genes, 1.6%	9.45E-06	0.00%	C1_04040C_A:DIP2:C1_09710C_A:C1_10880W_A:NEP1:KRR1:C1_13820C_A:FAV3:C2_00410C_A:C2_00820W_A:C2_02540W_A:NOC4:RCL1:BUD21:C3_02040C_A:RAD4:C4_00690C_A:SAS10:C5_03920C_A:C7_03850W_A:UTP18:CR_02420W_A:RRP9:CR_03200C_A:DBP8:CR_08330W_A:CR_09800C_A:CR_10410C_A

22618	protein-RNA complex assembly	40 out of 537 genes, 7.4%	193 out of 6473 background genes, 3.0%	3.47E-05	0.00%	C1_04040C_A:RRP6:C1_06760C_A:JIP5:C1_09710C_A:C1_10970W_A:C2_02540W_A:C2_02710C_A:MAK5:C2_05160C_A:C2_07520C_A:C3_00100W_A:BMS1:C3_01560W_A:C3_02040C_A:C3_02670W_A:C3_04370C_A:MAK21:QDR2:RLP24:C4_05010W_A:C4_05230C_A:C5_02010C_A:C5_05340W_A:C6_02230W_A:DBP7:C7_03400C_A:ISY1:ENP2:UTP18:BUD22:CR_01410C_A:SGD1:CR_04110W_A:CR_04170W_A:TSR1:ELF1:CR_09800C_A:CR_10470C_A:DRS1

2E+06	organic cyclic compound metabolic process	213 out of 537 genes, 39.7%	1903 out of 6473 background genes, 29.4%	6.76E-05	0.00%	BFA1:CNS1:C1_01150C_A:C1_01160C_A:C1_02090C_A:RAD16:PDE2:C1_02850W_A:C1_03180W_A:C1_03830C_A:SEN15:C1_04040C_A:PSF2:RRP6:REI1:C1_05270C_A:RAD10:C1_06120C_A:NOP6:C1_06760C_A:C1_06800W_A:C1_07950C_A:C1_07960W_A:DIP2:MSS116:C1_08890C_A:LIP6:C1_09610W_A:C1_09710C_A:URA1:HNT1:C1_10880W_A:C1_10950C_A:C1_10970W_A:PET127:NEP1:KRR1:RIB3:OSM2:C1_13820C_A:FAV3:NDT80:C2_00180C_A:C2_00410C_A:PHHB:C2_00820W_A:C2_00840W_A:REX2:C2_01870C_A:UTP21:C2_02540W_A:C2_02710C_A:C2_04120C_A:RIM2:C2_04570W_A:C2_04820W_A:CRK1:MAK5:C2_05160C_A:RPC40:LAS1:MAK16:RTA2:C2_06480W_A:C2_06850W_A:AAH1:RPA12:NOC4:RCL1:C2_07520C_A:RNR22:C2_07630C_A:C2_07920W_A:NSA1:C2_08170W_A:ORF298:C2_08460C_A:RRP8:UTP15:C2_09160W_A:RRP15:C2_09500W_A:C2_09510C_A:C2_09660W_A:C2_09920W_A:PUS7:C3_00100W_A:BUD21:C3_00420W_A:BMS1:C3_01560W_A:C3_02040C_A:UTP4:ILV2:C3_02670W_A:DAL4:YAH1:HBR3:CCC1:PTR22:C3_04370C_A:MAK21:SFP1:CEM1:C3_05120C_A:C3_05140C_A:C3_05380W_A:QDR2:C3_05800W_A:CTA4:C3_06350W_A:NSA2:C3_06760W_A:NOP13:C3_07550C_A:RAD4:RLP24:C4_00690C_A:C4_00700C_A:RAT1:C4_01730C_A:TIM12:ZCF25:SAS10:HCA4:ZUO1:C4_02880C_A:NAN1:RAM1:C4_04810C_A:AGP3:C4_05230C_A:OFD1:C4_06210C_A:C4_06790W_A:C5_00800C_A:C5_01140C_A:C5_01430C_A:C5_02010C_A:RIX7:C5_02730C_A:FUR1:C5_03920C_A:C5_04360C_A:MSM1:C5_05130C_A:C5_05340W_A:C6_00640C_A:C6_01040C_A:EBP1:C6_01890C_A:C6_02230W_A:C6_02410W_A:C6_02430W_A:C6_02560W_A:BMT4:FGR17:PAD1:C6_04240W_A:C6_04530C_A:NOP15:C7_01030C_A:DBP7:C7_02100W_A:C7_02340C_A:TIM54:DFR1:C7_03400C_A:PNC1:ISY1:ENP2:TIM9:C7_03850W_A:C7_03880C_A:UTP18:CR_00670C_A:BUD22:CR_01410C_A:CR_01950W_A:CR_02030C_A:CR_02420W_A:RRP9:DOM34:CR_03200C_A:CR_03240C_A:KTI12:SGD1:PRP42:CR_04110W_A:CR_04170W_A:QDR1:CR_04240C_A:MTG2:DBP8:SFL1:FGR50:NMD3:CR_07030C_A:URA2:CR_07640C_A:IMP4:CR_08000C_A:CR_08330W_A:CR_08410W_A:TSR1:SKI2:CR_08940W_A:CR_09140C_A:CR_09800C_A:UTP5:CR_10410C_A:CR_10470C_A:DRS1

71826	protein-RNA complex organization	40 out of 537 genes, 7.4%	198 out of 6473 background genes, 3.1%	7.30E-05	0.00%	C1_04040C_A:RRP6:C1_06760C_A:JIP5:C1_09710C_A:C1_10970W_A:C2_02540W_A:C2_02710C_A:MAK5:C2_05160C_A:C2_07520C_A:C3_00100W_A:BMS1:C3_01560W_A:C3_02040C_A:C3_02670W_A:C3_04370C_A:MAK21:QDR2:RLP24:C4_05010W_A:C4_05230C_A:C5_02010C_A:C5_05340W_A:C6_02230W_A:DBP7:C7_03400C_A:ISY1:ENP2:UTP18:BUD22:CR_01410C_A:SGD1:CR_04110W_A:CR_04170W_A:TSR1:ELF1:CR_09800C_A:CR_10470C_A:DRS1

10467	gene expression	217 out of 537 genes, 40.4%	1951 out of 6473 background genes, 30.1%	7.93E-05	0.00%	BFA1:CNS1:TUF1:C1_01150C_A:C1_01160C_A:RPS21:C1_02090C_A:HOM6:PDE2:C1_02850W_A:MIA40:C1_03180W_A:C1_03830C_A:SEN15:C1_04040C_A:PSF2:RRP6:REI1:C1_05270C_A:NOP6:C1_06760C_A:C1_06800W_A:C1_07950C_A:C1_07960W_A:LTP1:DIP2:MSS116:C1_08890C_A:C1_09710C_A:C1_09910C_A:C1_10880W_A:C1_10950C_A:C1_10970W_A:PET127:NEP1:KRR1:C1_13060C_A:C1_13260W_A:OSM2:C1_13820C_A:RBE1:FAV3:NDT80:C2_00410C_A:C2_00820W_A:C2_00840W_A:C2_00880W_A:REX2:C2_01740C_A:C2_01870C_A:UTP21:C2_02540W_A:C2_02710C_A:C2_03560C_A:C2_04120C_A:RIM2:C2_04570W_A:C2_04820W_A:CRK1:MAK5:C2_05160C_A:RPC40:IDP1:LAS1:MAK16:RTA2:C2_06480W_A:C2_06850W_A:RPA12:NOC4:RCL1:C2_07520C_A:C2_07920W_A:NSA1:RRP8:UTP15:C2_09160W_A:SLP2:RRP15:C2_09500W_A:C2_09510C_A:C2_09660W_A:C2_09920W_A:PUS7:MTR2:C3_00100W_A:BUD21:C3_00420W_A:BMS1:C3_01560W_A:C3_02040C_A:UTP4:ILV2:C3_02670W_A:HBR3:CCC1:SAP9:C3_04370C_A:MAK21:SFP1:CEM1:C3_05120C_A:C3_05140C_A:C3_05380W_A:QDR2:C3_05800W_A:CTA4:C3_06350W_A:NSA2:C3_06760W_A:C3_06830C_A:NOP13:C3_07550C_A:RAD4:RLP24:C4_00690C_A:DAG7:RAT1:C4_01730C_A:TIM12:HGH1:TIM10:ZCF25:SAS10:HCA4:ZUO1:C4_02880C_A:NAN1:RAM1:C4_04810C_A:AGP3:C4_05230C_A:OFD1:C4_06210C_A:C4_06790W_A:C5_00030W_A:MET14:C5_01140C_A:C5_01430C_A:GIS2:C5_02010C_A:RIX7:C5_02730C_A:FUR1:C5_03530C_A:C5_03920C_A:C5_04290C_A:CSU57:MSM1:SDH4:C5_05340W_A:FET31:C6_00640C_A:C6_01040C_A:C6_01890C_A:C6_01980C_A:C6_02230W_A:C6_02430W_A:FGR17:C6_04240W_A:C6_04530C_A:NAG3:NOP15:YML6:C7_01030C_A:DBP7:TOM40:C7_02100W_A:C7_02340C_A:TIM54:C7_03400C_A:ISY1:ENP2:TIM9:C7_03850W_A:C7_03880C_A:UTP18:CR_00670C_A:BUD22:CR_01410C_A:CR_01950W_A:CR_02030C_A:CR_02420W_A:CR_02690W_A:RRP9:DOM34:CR_03200C_A:CR_03240C_A:KTI12:PRP42:CR_04110W_A:CR_04170W_A:CR_04240C_A:RPS3:MTG2:DBP8:ACO2:SFL1:FGR50:NMD3:CR_07030C_A:URA2:CR_07640C_A:IMP4:CR_08000C_A:CR_08330W_A:CR_08410W_A:TSR1:SKI2:CR_08940W_A:ELF1:CR_09800C_A:UTP5:CR_10400W_A:CR_10410C_A:CR_10470C_A:DRS1
9082	branched-chain amino acid biosynthetic process	8 out of 537 genes, 1.5%	12 out of 6473 background genes, 0.2%	0.00078	0.00%	LEU4:BAT21:LEU42:ILV2:ILV6:ILV3:ILV5:LEU1

1E+05	non-membrane-bounded organelle assembly	35 out of 537 genes, 6.5%	180 out of 6473 background genes, 2.8%	0.00127	0.00%	C1_04040C_A:RRP6:C1_06760C_A:JIP5:C1_09710C_A:C1_10970W_A:C2_02540W_A:C2_02710C_A:MAK5:C2_05160C_A:BMS1:C3_01560W_A:C3_02040C_A:C3_02670W_A:C3_04370C_A:MAK21:QDR2:C4_02880C_A:C4_05010W_A:C4_05230C_A:C5_05340W_A:C6_02230W_A:DBP7:C7_03400C_A:ENP2:UTP18:BUD22:CR_01410C_A:SGD1:CR_04170W_A:TSR1:ELF1:CR_09800C_A:CR_10470C_A:DRS1

34641	cellular nitrogen compound metabolic process	240 out of 537 genes, 44.7%	2297 out of 6473 background genes, 35.5%	0.00273	0.00%	BFA1:CNS1:TUF1:C1_01150C_A:C1_01160C_A:RPS21:C1_02090C_A:HOM6:RAD16:PDE2:C1_02850W_A:MIA40:C1_03830C_A:SEN15:C1_04040C_A:PSF2:RRP6:REI1:C1_05270C_A:RAD10:C1_05990C_A:C1_06120C_A:GLT1:NOP6:C1_06760C_A:C1_06800W_A:GCS1:C1_07950C_A:C1_07960W_A:LTP1:DIP2:MSS116:C1_08890C_A:C1_09710C_A:URA1:C1_09910C_A:HNT1:C1_10880W_A:C1_10950C_A:C1_10970W_A:PET127:NEP1:KRR1:RIB3:C1_13060C_A:C1_13330C_A:OSM2:C1_13820C_A:RBE1:FAV3:NDT80:C2_00180C_A:C2_00410C_A:PHHB:C2_00820W_A:C2_00840W_A:C2_00880W_A:REX2:C2_01740C_A:C2_01870C_A:UTP21:C2_02540W_A:C2_02710C_A:C2_03560C_A:C2_04120C_A:RIM2:C2_04570W_A:C2_04820W_A:CRK1:MAK5:C2_05160C_A:RPC40:IDP1:LAS1:MAK16:RTA2:C2_06480W_A:C2_06850W_A:AAH1:RPA12:NOC4:RCL1:C2_07520C_A:RNR22:C2_07630C_A:C2_07920W_A:NSA1:ORF298:C2_08460C_A:RRP8:UTP15:C2_09160W_A:SLP2:RRP15:C2_09500W_A:C2_09510C_A:C2_09660W_A:C2_09920W_A:PUS7:C3_00100W_A:BUD21:C3_00420W_A:BMS1:C3_01560W_A:C3_02040C_A:UTP4:ILV2:C3_02670W_A:YAH1:HBR3:CCC1:C3_04370C_A:MAK21:SFP1:CEM1:C3_05120C_A:C3_05140C_A:C3_05380W_A:QDR2:C3_05800W_A:CTA4:C3_06350W_A:NSA2:C3_06760W_A:C3_06830C_A:NOP13:C3_07550C_A:RAD4:RLP24:C4_00690C_A:C4_00700C_A:DAG7:RAT1:C4_01730C_A:TIM12:TIM10:ZCF25:SAS10:HCA4:ZUO1:C4_02880C_A:NAN1:RAM1:C4_04810C_A:AGP3:C4_05230C_A:OFD1:GDH3:C4_06210C_A:C4_06790W_A:YDC1:C5_00030W_A:MET14:C5_00800C_A:C5_01140C_A:C5_01430C_A:GIS2:C5_02010C_A:RIX7:C5_02730C_A:FUR1:C5_03530C_A:C5_03920C_A:C5_04290C_A:C5_04360C_A:CSU57:MSM1:C5_05130C_A:SDH4:C5_05340W_A:FET31:C6_00640C_A:C6_01040C_A:C6_01890C_A:C6_01980C_A:C6_02230W_A:C6_02410W_A:C6_02430W_A:C6_02560W_A:FGR17:C6_04240W_A:C6_04530C_A:NAG3:NOP15:YML6:C7_01030C_A:DBP7:TOM40:C7_02100W_A:C7_02340C_A:TIM54:DFR1:C7_03400C_A:PNC1:ISY1:ENP2:TIM9:C7_03850W_A:C7_03880C_A:UTP18:CR_00670C_A:BUD22:CR_01410C_A:CR_01950W_A:CR_02030C_A:CR_02420W_A:RRP9:DOM34:CR_03200C_A:CR_03240C_A:KTI12:SGD1:PRP42:CR_04110W_A:CR_04170W_A:CR_04240C_A:RPS3:CR_04880W_A:MTG2:DBP8:ACO2:SFL1:FGR50:NMD3:CR_07030C_A:URA2:CR_07640C_A:IMP4:CR_08000C_A:CR_08330W_A:CR_08410W_A:TSR1:SKI2:CR_08940W_A:CR_09140C_A:CR_09800C_A:UTP5:CR_10410C_A:CR_10470C_A:DRS1

463	maturation of LSU-rRNA from tricistronic rRNA transcript (SSU-rRNA, 5.8S rRNA, LSU-rRNA)	63 out of 537 genes, 11.7%	429 out of 6473 background genes, 6.6%	0.00346	0.00%	C1_02090C_A:PDE2:C1_02850W_A:C1_03830C_A:SEN15:REI1:NOP6:C1_06800W_A:C1_07960W_A:C2_00820W_A:C2_02540W_A:C2_04120C_A:MAK5:LAS1:MAK16:C2_06850W_A:RRP8:UTP15:C2_09160W_A:RRP15:C2_09920W_A:C3_00420W_A:C3_01560W_A:ILV2:C3_02670W_A:C3_04370C_A:CEM1:C3_05120C_A:C3_05380W_A:QDR2:C3_05800W_A:NSA2:C3_07550C_A:RAT1:C4_01730C_A:TIM12:C4_02880C_A:RAM1:AGP3:C4_06210C_A:C4_06790W_A:C5_01430C_A:C5_02010C_A:C5_03920C_A:C6_01890C_A:C6_02230W_A:C6_02430W_A:C6_04240W_A:NOP15:DBP7:C7_03400C_A:ISY1:TIM9:C7_03850W_A:C7_03880C_A:CR_02030C_A:CR_03200C_A:CR_03240C_A:PRP42:CR_04170W_A:SFL1:CR_07640C_A:CR_08000C_A

470	maturation of LSU-rRNA	63 out of 537 genes, 11.7%	431 out of 6473 background genes, 6.7%	0.00406	0.00%	C1_02090C_A:PDE2:C1_02850W_A:C1_03830C_A:SEN15:REI1:NOP6:C1_06800W_A:C1_07960W_A:C2_00820W_A:C2_02540W_A:C2_04120C_A:MAK5:LAS1:MAK16:C2_06850W_A:RRP8:UTP15:C2_09160W_A:RRP15:C2_09920W_A:C3_00420W_A:C3_01560W_A:ILV2:C3_02670W_A:C3_04370C_A:CEM1:C3_05120C_A:C3_05380W_A:QDR2:C3_05800W_A:NSA2:C3_07550C_A:RAT1:C4_01730C_A:TIM12:C4_02880C_A:RAM1:AGP3:C4_06210C_A:C4_06790W_A:C5_01430C_A:C5_02010C_A:C5_03920C_A:C6_01890C_A:C6_02230W_A:C6_02430W_A:C6_04240W_A:NOP15:DBP7:C7_03400C_A:ISY1:TIM9:C7_03850W_A:C7_03880C_A:CR_02030C_A:CR_03200C_A:CR_03240C_A:PRP42:CR_04170W_A:SFL1:CR_07640C_A:CR_08000C_A

44249	cellular biosynthetic process	255 out of 537 genes, 47.5%	2485 out of 6473 background genes, 38.4%	0.00463	0.00%	LEU4:BFA1:CNS1:TUF1:C1_01150C_A:C1_01160C_A:RPS21:C1_02090C_A:HOM6:LYS2:PDE2:C1_02850W_A:MIA40:C1_03180W_A:C1_03830C_A:SEN15:C1_04040C_A:RHD1:PSF2:RRP6:REI1:C1_05270C_A:RAD10:C1_05990C_A:GLT1:NOP6:C1_06760C_A:C1_06800W_A:GCS1:C1_07950C_A:C1_07960W_A:LTP1:DIP2:MSS116:C1_08890C_A:LIP6:C1_09710C_A:URA1:C1_09910C_A:C1_10880W_A:C1_10950C_A:C1_10970W_A:PET127:NEP1:KRR1:RIB3:C1_13060C_A:C1_13260W_A:C1_13330C_A:OSM2:C1_13820C_A:RBE1:FAV3:NDT80:C2_00410C_A:PHHB:C2_00820W_A:C2_00840W_A:C2_00880W_A:REX2:C2_01740C_A:C2_01870C_A:UTP21:C2_02540W_A:C2_02710C_A:C2_03560C_A:MNN42:C2_04120C_A:BAT21:LYS22:RIM2:C2_04570W_A:C2_04820W_A:CRK1:MAK5:C2_05160C_A:RPC40:IDP1:LAS1:MAK16:RTA2:C2_06480W_A:C2_06850W_A:AAH1:RPA12:NOC4:RCL1:C2_07520C_A:RNR22:C2_07920W_A:NSA1:ORF298:RRP8:UTP15:C2_09160W_A:SLP2:RRP15:C2_09500W_A:C2_09510C_A:C2_09660W_A:LEU42:C2_09920W_A:PUS7:MTR2:C3_00100W_A:BUD21:C3_00420W_A:BMS1:C3_01560W_A:C3_02040C_A:UTP4:ILV2:C3_02670W_A:MET13:YAH1:HBR3:CCC1:SAP9:C3_04370C_A:MAK21:SFP1:CEM1:C3_05120C_A:C3_05140C_A:C3_05380W_A:QDR2:C3_05800W_A:CTA4:C3_06350W_A:NSA2:C3_06760W_A:C3_06830C_A:NOP13:C3_07550C_A:RAD4:RLP24:C4_00690C_A:DAG7:RAT1:ILV6:C4_01730C_A:TIM12:HGH1:TIM10:ZCF25:SAS10:HCA4:ZUO1:C4_02880C_A:NAN1:RAM1:C4_04810C_A:AGP3:C4_05230C_A:OFD1:HOM3:GDH3:C4_06210C_A:C4_06790W_A:MET16:YDC1:C5_00030W_A:MET14:C5_00800C_A:C5_01140C_A:C5_01430C_A:GIS2:C5_02010C_A:RIX7:THR4:C5_02730C_A:FUR1:C5_03530C_A:C5_03920C_A:C5_04290C_A:CSU57:MSM1:C5_05130C_A:SDH4:C5_05340W_A:ILV3:FET31:C6_00640C_A:ILV5:C6_01040C_A:C6_01890C_A:C6_01980C_A:C6_02230W_A:C6_02430W_A:FGR17:C6_04240W_A:C6_04530C_A:NAG3:NOP15:YML6:C7_01030C_A:DBP7:TOM40:C7_02100W_A:C7_02340C_A:TIM54:DFR1:C7_03400C_A:PNC1:ISY1:ENP2:TIM9:C7_03850W_A:C7_03880C_A:UTP18:C7_04210C_A:LEU1:CR_00570W_A:CR_00670C_A:BUD22:LYS12:CR_01410C_A:CR_01950W_A:CR_02030C_A:CR_02420W_A:CR_02690W_A:RRP9:DOM34:CR_03200C_A:CR_03240C_A:KTI12:PRP42:CR_04110W_A:CR_04170W_A:CR_04240C_A:RPS3:CR_04880W_A:MTG2:DBP8:ACO2:SFL1:FGR50:NMD3:CR_07030C_A:URA2:CR_07640C_A:IMP4:CR_08000C_A:CR_08330W_A:CR_08410W_A:TSR1:SKI2:CR_08940W_A:ELF1:CR_09800C_A:UTP5:CR_10400W_A:CR_10410C_A:CR_10470C_A:DRS1

9058	biosynthetic process	263 out of 537 genes, 49.0%	2586 out of 6473 background genes, 40.0%	0.00605	0.00%	LEU4:BFA1:CNS1:TUF1:C1_01150C_A:C1_01160C_A:RPS21:C1_02090C_A:HOM6:LYS2:PDE2:C1_02850W_A:MIA40:C1_03180W_A:C1_03830C_A:SEN15:C1_04040C_A:RHD1:PSF2:RRP6:REI1:C1_05270C_A:RAD10:C1_05990C_A:GLT1:NOP6:C1_06760C_A:C1_06800W_A:GCS1:C1_07950C_A:C1_07960W_A:LTP1:DIP2:MSS116:C1_08890C_A:LIP6:C1_09610W_A:C1_09710C_A:URA1:C1_09910C_A:C1_10880W_A:C1_10950C_A:C1_10970W_A:PET127:NEP1:KRR1:RIB3:C1_13060C_A:C1_13260W_A:C1_13330C_A:OSM2:C1_13820C_A:RBE1:FAV3:NDT80:C2_00410C_A:PHHB:C2_00820W_A:C2_00840W_A:C2_00880W_A:REX2:C2_01740C_A:C2_01870C_A:UTP21:C2_02540W_A:BNA32:C2_02710C_A:C2_03560C_A:MNN42:C2_04120C_A:BAT21:LYS22:RIM2:C2_04570W_A:C2_04820W_A:CRK1:MAK5:C2_05160C_A:RPC40:IDP1:LAS1:MAK16:RTA2:C2_06480W_A:C2_06850W_A:AAH1:RPA12:NOC4:RCL1:C2_07520C_A:RNR22:C2_07920W_A:NSA1:ORF298:RRP8:UTP15:C2_09160W_A:SLP2:RRP15:C2_09500W_A:C2_09510C_A:C2_09660W_A:LEU42:C2_09920W_A:GPD1:PUS7:MTR2:C3_00100W_A:RHR2:BUD21:C3_00420W_A:NDU1:BMS1:C3_01560W_A:C3_02040C_A:UTP4:ILV2:C3_02670W_A:MET13:YAH1:HBR3:CCC1:PTR22:SAP9:C3_04370C_A:MAK21:SFP1:CEM1:C3_05120C_A:C3_05140C_A:C3_05380W_A:QDR2:C3_05800W_A:CTA4:C3_06350W_A:NSA2:C3_06760W_A:C3_06830C_A:NOP13:C3_07550C_A:RAD4:RLP24:C4_00690C_A:DAG7:RAT1:ILV6:C4_01730C_A:TIM12:HGH1:TIM10:ZCF25:SAS10:HCA4:ZUO1:C4_02880C_A:NAN1:RAM1:C4_04810C_A:AGP3:C4_05230C_A:OFD1:HOM3:GDH3:C4_06210C_A:C4_06790W_A:MET16:YDC1:C5_00030W_A:MET14:C5_00800C_A:C5_01140C_A:C5_01430C_A:GIS2:C5_02010C_A:RIX7:THR4:C5_02730C_A:FUR1:C5_03530C_A:C5_03920C_A:C5_04290C_A:CSU57:MSM1:C5_05130C_A:SDH4:C5_05340W_A:ILV3:FET31:C6_00640C_A:ILV5:C6_01040C_A:C6_01890C_A:C6_01980C_A:C6_02230W_A:C6_02430W_A:BMT4:FGR17:C6_04240W_A:C6_04530C_A:NAG3:NOP15:YML6:C7_01030C_A:DBP7:TOM40:C7_02100W_A:C7_02340C_A:TIM54:DFR1:C7_03400C_A:PNC1:ISY1:ENP2:TIM9:C7_03850W_A:C7_03880C_A:UTP18:C7_04210C_A:LEU1:CR_00570W_A:CR_00670C_A:BUD22:LYS12:CR_01410C_A:CR_01950W_A:CR_02030C_A:CR_02420W_A:CR_02690W_A:RRP9:DOM34:CR_03200C_A:CR_03240C_A:KTI12:PRP42:CR_04110W_A:CR_04170W_A:QDR1:CR_04240C_A:RPS3:CR_04880W_A:MTG2:DBP8:ACO2:SFL1:FGR50:NMD3:CR_07030C_A:URA2:CR_07640C_A:IMP4:CR_08000C_A:CR_08330W_A:CR_08410W_A:TSR1:SKI2:CR_08940W_A:ELF1:CR_09800C_A:UTP5:CR_10400W_A:CR_10410C_A:CR_10470C_A:DRS1

2E+06	organic substance biosynthetic process	261 out of 537 genes, 48.6%	2571 out of 6473 background genes, 39.7%	0.00816	0.00%	LEU4:BFA1:CNS1:TUF1:C1_01150C_A:C1_01160C_A:RPS21:C1_02090C_A:HOM6:LYS2:PDE2:C1_02850W_A:MIA40:C1_03180W_A:C1_03830C_A:SEN15:C1_04040C_A:RHD1:PSF2:RRP6:REI1:C1_05270C_A:RAD10:C1_05990C_A:GLT1:NOP6:C1_06760C_A:C1_06800W_A:GCS1:C1_07950C_A:C1_07960W_A:LTP1:DIP2:MSS116:C1_08890C_A:LIP6:C1_09610W_A:C1_09710C_A:URA1:C1_09910C_A:C1_10880W_A:C1_10950C_A:C1_10970W_A:PET127:NEP1:KRR1:RIB3:C1_13060C_A:C1_13260W_A:C1_13330C_A:OSM2:C1_13820C_A:RBE1:FAV3:NDT80:C2_00410C_A:PHHB:C2_00820W_A:C2_00840W_A:C2_00880W_A:REX2:C2_01740C_A:C2_01870C_A:UTP21:C2_02540W_A:C2_02710C_A:C2_03560C_A:MNN42:C2_04120C_A:BAT21:LYS22:RIM2:C2_04570W_A:C2_04820W_A:CRK1:MAK5:C2_05160C_A:RPC40:IDP1:LAS1:MAK16:RTA2:C2_06480W_A:C2_06850W_A:AAH1:RPA12:NOC4:RCL1:C2_07520C_A:RNR22:C2_07920W_A:NSA1:ORF298:RRP8:UTP15:C2_09160W_A:SLP2:RRP15:C2_09500W_A:C2_09510C_A:C2_09660W_A:LEU42:C2_09920W_A:GPD1:PUS7:MTR2:C3_00100W_A:RHR2:BUD21:C3_00420W_A:BMS1:C3_01560W_A:C3_02040C_A:UTP4:ILV2:C3_02670W_A:MET13:YAH1:HBR3:CCC1:PTR22:SAP9:C3_04370C_A:MAK21:SFP1:CEM1:C3_05120C_A:C3_05140C_A:C3_05380W_A:QDR2:C3_05800W_A:CTA4:C3_06350W_A:NSA2:C3_06760W_A:C3_06830C_A:NOP13:C3_07550C_A:RAD4:RLP24:C4_00690C_A:DAG7:RAT1:ILV6:C4_01730C_A:TIM12:HGH1:TIM10:ZCF25:SAS10:HCA4:ZUO1:C4_02880C_A:NAN1:RAM1:C4_04810C_A:AGP3:C4_05230C_A:OFD1:HOM3:GDH3:C4_06210C_A:C4_06790W_A:MET16:YDC1:C5_00030W_A:MET14:C5_00800C_A:C5_01140C_A:C5_01430C_A:GIS2:C5_02010C_A:RIX7:THR4:C5_02730C_A:FUR1:C5_03530C_A:C5_03920C_A:C5_04290C_A:CSU57:MSM1:C5_05130C_A:SDH4:C5_05340W_A:ILV3:FET31:C6_00640C_A:ILV5:C6_01040C_A:C6_01890C_A:C6_01980C_A:C6_02230W_A:C6_02430W_A:BMT4:FGR17:C6_04240W_A:C6_04530C_A:NAG3:NOP15:YML6:C7_01030C_A:DBP7:TOM40:C7_02100W_A:C7_02340C_A:TIM54:DFR1:C7_03400C_A:PNC1:ISY1:ENP2:TIM9:C7_03850W_A:C7_03880C_A:UTP18:C7_04210C_A:LEU1:CR_00570W_A:CR_00670C_A:BUD22:LYS12:CR_01410C_A:CR_01950W_A:CR_02030C_A:CR_02420W_A:CR_02690W_A:RRP9:DOM34:CR_03200C_A:CR_03240C_A:KTI12:PRP42:CR_04110W_A:CR_04170W_A:QDR1:CR_04240C_A:RPS3:CR_04880W_A:MTG2:DBP8:ACO2:SFL1:FGR50:NMD3:CR_07030C_A:URA2:CR_07640C_A:IMP4:CR_08000C_A:CR_08330W_A:CR_08410W_A:TSR1:SKI2:CR_08940W_A:ELF1:CR_09800C_A:UTP5:CR_10400W_A:CR_10410C_A:CR_10470C_A:DRS1

71035	nuclear polyadenylation-dependent rRNA catabolic process	8 out of 537 genes, 1.5%	15 out of 6473 background genes, 0.2%	0.00817	0.00%	C1_01160C_A:C1_03830C_A:RRP6:C1_06800W_A:C1_07960W_A:C2_09160W_A:RAT1:C7_03400C_A
1510	RNA methylation	12 out of 537 genes, 2.2%	35 out of 6473 background genes, 0.5%	0.01336	0.00%	C1_01150C_A:NEP1:C2_06480W_A:RRP8:C3_05140C_A:C4_04810C_A:C6_04530C_A:C7_02340C_A:CR_00670C_A:CR_02030C_A:CR_04170W_A:CR_08940W_A
44282	small molecule catabolic process	23 out of 537 genes, 4.3%	108 out of 6473 background genes, 1.7%	0.01893	0.00%	GOR1:DUR1,2:ANT1:MLS1:BAT21:GLO2:FOX2:POX1-3:C3_06860C_A:DLD2:CAR2:SOU1:C5_00800C_A:CSU57:GCV1:PAD1:POT1:CRC1:CR_04880W_A:URA2:ECI1:CR_09670C_A:UGA3
70925	organelle assembly	35 out of 537 genes, 6.5%	203 out of 6473 background genes, 3.1%	0.02103	0.00%	C1_04040C_A:RRP6:C1_06760C_A:JIP5:C1_09710C_A:C1_10970W_A:C2_02540W_A:C2_02710C_A:MAK5:C2_05160C_A:BMS1:C3_01560W_A:C3_02040C_A:C3_02670W_A:C3_04370C_A:MAK21:QDR2:C4_02880C_A:C4_05010W_A:C4_05230C_A:C5_05340W_A:C6_02230W_A:DBP7:C7_03400C_A:ENP2:UTP18:BUD22:CR_01410C_A:SGD1:CR_04170W_A:TSR1:ELF1:CR_09800C_A:CR_10470C_A:DRS1

43633	polyadenylation-dependent RNA catabolic process	8 out of 537 genes, 1.5%	17 out of 6473 background genes, 0.3%	0.02659	0.00%	C1_01160C_A:C1_03830C_A:RRP6:C1_06800W_A:C1_07960W_A:C2_09160W_A:RAT1:C7_03400C_A
43634	polyadenylation-dependent ncRNA catabolic process	8 out of 537 genes, 1.5%	17 out of 6473 background genes, 0.3%	0.02659	0.00%	C1_01160C_A:C1_03830C_A:RRP6:C1_06800W_A:C1_07960W_A:C2_09160W_A:RAT1:C7_03400C_A
71029	nuclear ncRNA surveillance	8 out of 537 genes, 1.5%	17 out of 6473 background genes, 0.3%	0.02659	0.00%	C1_01160C_A:C1_03830C_A:RRP6:C1_06800W_A:C1_07960W_A:C2_09160W_A:RAT1:C7_03400C_A
71046	nuclear polyadenylation-dependent ncRNA catabolic process	8 out of 537 genes, 1.5%	17 out of 6473 background genes, 0.3%	0.02659	0.00%	C1_01160C_A:C1_03830C_A:RRP6:C1_06800W_A:C1_07960W_A:C2_09160W_A:RAT1:C7_03400C_A
9081	branched-chain amino acid metabolic process	8 out of 537 genes, 1.5%	17 out of 6473 background genes, 0.3%	0.02659	0.00%	LEU4:BAT21:LEU42:ILV2:ILV6:ILV3:ILV5:LEU1

71025	RNA surveillance	9 out of 537 genes, 1.7%	23 out of 6473 background genes, 0.4%	0.04968	0.04%	C1_01160C_A:C1_03830C_A:RRP6:C1_06800W_A:C1_07960W_A:C2_09160W_A:RAT1:C7_03400C_A:DOM34
6807	nitrogen compound metabolic process	328 out of 537 genes, 61.1%	3438 out of 6473 background genes, 53.1%	0.06298	0.08%	LEU4:BFA1:CNS1:TUF1:C1_01150C_A:C1_01160C_A:RPS21:C1_02090C_A:HOM6:RAD16:LYS2:PDE2:C1_02850W_A:MIA40:GOR1:C1_03180W_A:C1_03430W_A:C1_03830C_A:SEN15:C1_04040C_A:GPM2:DUR1,2:RHD1:PSF2:RRP6:REI1:C1_05270C_A:RAD10:C1_05990C_A:C1_06120C_A:GLT1:NOP6:C1_06760C_A:NPR1:C1_06800W_A:CAT1:RME1:GCS1:C1_07950C_A:C1_07960W_A:LTP1:DIP2:CAF16:MSS116:C1_08890C_A:AOX1:DAK2:LIP6:C1_09610W_A:MLS1:C1_09710C_A:URA1:C1_09910C_A:HNT1:C1_10880W_A:C1_10950C_A:C1_10970W_A:PET127:NEP1:KRR1:RIB3:C1_13060C_A:C1_13260W_A:C1_13330C_A:OSM2:C1_13820C_A:MET3:RBE1:FAV3:NDT80:C2_00180C_A:C2_00410C_A:PHHB:C2_00820W_A:C2_00840W_A:C2_00880W_A:REX2:C2_01740C_A:C2_01870C_A:UTP21:C2_02490C_A:C2_02540W_A:C2_02710C_A:AMO1:C2_03130W_A:C2_03560C_A:C2_03700W_A:C2_03830W_A:C2_04120C_A:BAT21:LYS22:RIM2:C2_04570W_A:C2_04820W_A:CRK1:C2_05060C_A:MAK5:C2_05160C_A:RPC40:IDP1:LAS1:MAK16:RTA2:C2_06480W_A:C2_06850W_A:AAH1:C2_07070W_A:RCK2:RPA12:NOC4:RCL1:C2_07520C_A:RNR22:C2_07630C_A:C2_07920W_A:NSA1:ARA1:C2_08200W_A:ORF298:C2_08460C_A:RRP8:UTP15:C2_09160W_A:SLP2:GLO2:C2_09280C_A:RRP15:C2_09500W_A:C2_09510C_A:C2_09660W_A:LEU42:C2_09920W_A:PUS7:C3_00100W_A:BUD21:C3_00420W_A:BMS1:C3_01560W_A:C3_02040C_A:UTP4:C3_02140C_A:ILV2:CCP1:C3_02670W_A:MET13:DAL4:YAH1:HBR3:CCC1:SAP9:AGT1:C3_04370C_A:MAK21:SFP1:CEM1:C3_05120C_A:C3_05140C_A:C3_05380W_A:QDR2:C3_05800W_A:CTA4:CYM1:C3_06350W_A:NSA2:C3_06760W_A:C3_06830C_A:C3_06860C_A:NOP13:C3_07550C_A:RAD4:CAR2:RLP24:C4_00690C_A:C4_00700C_A:DAG7:RAT1:BMT9:ILV6:C4_01470W_A:C4_01730C_A:TIM12:C4_01830C_A:HGH1:TIM10:ZCF25:HOS3:AHP1:SAS10:HCA4:ZUO1:C4_02880C_A:NAN1:C4_03050C_A:RAM1:SAP10:C4_04500C_A:C4_04810C_A:AGP3:C4_05230C_A:OFD1:HOM3:GDH3:C4_06210C_A:SOU2:SOU1:C4_06790W_A:MET16:YDC1:C5_00030W_A:MET14:C5_00800C_A:TFS1:C5_01140C_A:C5_01430C_A:GLR1:GIS2:C5_02010C_A:HSP12:C5_02110W_A:RIX7:THR4:C5_02690W_A:C5_02730C_A:FUR1:C5_03530C_A:C5_03920C_A:C5_04290C_A:C5_04360C_A:CSU57:MSM1:C5_05130C_A:SDH4:C5_05340W_A:ILV3:C5_05440C_A:FET31:C6_00640C_A:C6_00760W_A:ILV5:KAR5:C6_01040C_A:C6_01890C_A:C6_01980C_A:C6_02230W_A:C6_02410W_A:C6_02430W_A:GCV1:C6_02560W_A:FGR17:C6_04240W_A:C6_04530C_A:NAG3:NOP15:YML6:C

						7_01030C_A:DBP7:TOM40:C7_02100W_A:C7_02340C_A:C7_03030W_A:TIM54:DFR1:C7_03400C_A:PNC1:ISY1:ENP2:TIM9:C7_03850W_A:C7_03880C_A:UTP18:C7_04210C_A:LEU1:CR_00670C_A:BUD22:LYS12:CR_01410C_A:CSP37:CR_01950W_A:CRC1:CR_02030C_A:CR_02420W_A:CR_02690W_A:RRP9:ERV1:DOM34:CR_03200C_A:CR_03240C_A:IFR2:KTI12:SGD1:PRP42:CR_03760W_A:CR_04110W_A:CR_04170W_A:CR_04240C_A:RPS3:CR_04880W_A:YCP4:MTG2:DBP8:ACO2:SFL1:SOK1:FGR50:NMD3:CR_07030C_A:URA2:CR_07480W_A:CR_07640C_A:IMP4:CR_08000C_A:CR_08330W_A:CR_08410W_A:TSR1:SKI2:NIT2:PDK2:CR_08940W_A:SLP3:CR_09140C_A:CR_09310W_A:CR_09800C_A:UGA3:UTP5:CR_10400W_A:CR_10410C_A:CR_10470C_A:DRS1

71704	organic substance metabolic process	352 out of 537 genes, 65.5%	3737 out of 6473 background genes, 57.7%	0.06787	0.11%	LEU4:BFA1:CNS1:TUF1:C1_01150C_A:C1_01160C_A:RPS21:C1_02090C_A:HOM6:RAD16:LYS2:PDE2:C1_02850W_A:MIA40:GOR1:C1_03180W_A:C1_03430W_A:C1_03830C_A:SEN15:C1_04040C_A:GPM2:DUR1,2:RHD1:PSF2:RRP6:REI1:C1_05270C_A:RAD10:C1_05990C_A:C1_06120C_A:GLT1:ANT1:NOP6:C1_06760C_A:NPR1:C1_06800W_A:CAT1:RME1:GCS1:C1_07950C_A:C1_07960W_A:LTP1:DIP2:CAF16:MSS116:C1_08890C_A:AOX1:DAK2:LIP6:C1_09610W_A:MLS1:C1_09710C_A:URA1:C1_09910C_A:HNT1:C1_10880W_A:C1_10950C_A:C1_10970W_A:PET127:NEP1:KRR1:RIB3:C1_13060C_A:C1_13260W_A:C1_13330C_A:CYB2:OSM2:C1_13820C_A:MET3:RBE1:FAV3:NDT80:C2_00180C_A:C2_00410C_A:PHHB:C2_00820W_A:C2_00840W_A:C2_00880W_A:REX2:C2_01740C_A:C2_01870C_A:UTP21:C2_02490C_A:C2_02540W_A:C2_02710C_A:AMO1:C2_03130W_A:C2_03560C_A:MNN42:C2_03700W_A:C2_03830W_A:C2_04120C_A:BAT21:LYS22:RIM2:C2_04570W_A:C2_04820W_A:CRK1:C2_05060C_A:MAK5:C2_05160C_A:RPC40:IDP1:LAS1:MAK16:RTA2:C2_06480W_A:C2_06850W_A:AAH1:C2_07070W_A:RCK2:RPA12:NOC4:RCL1:C2_07520C_A:RNR22:C2_07630C_A:C2_07920W_A:NSA1:ARA1:C2_08170W_A:C2_08200W_A:ORF298:C2_08460C_A:RRP8:UTP15:C2_09160W_A:SLP2:GLO2:C2_09280C_A:RRP15:C2_09500W_A:C2_09510C_A:C2_09660W_A:LEU42:C2_09920W_A:GPD1:PUS7:MTR2:C3_00100W_A:RHR2:BUD21:C3_00420W_A:FOX2:BMS1:C3_01560W_A:POX1-3:C3_02040C_A:UTP4:C3_02140C_A:ILV2:CCP1:C3_02670W_A:FAA21:MET13:DAL4:YAH1:HBR3:CCC1:PTR22:SAP9:AGT1:C3_04370C_A:MAK21:SFP1:CEM1:C3_05120C_A:C3_05140C_A:C3_05380W_A:QDR2:C3_05800W_A:CTA4:CYM1:C3_06350W_A:NSA2:C3_06760W_A:C3_06830C_A:C3_06860C_A:YSA1:NOP13:DLD2:C3_07550C_A:RAD4:CAR2:RLP24:C4_00690C_A:C4_00700C_A:DAG7:RAT1:BMT9:ILV6:C4_01470W_A:C4_01730C_A:TIM12:C4_01830C_A:HGH1:TIM10:ZCF25:HOS3:AHP1:C4_02620C_A:SAS10:HCA4:ZUO1:C4_02880C_A:NAN1:C4_03050C_A:RAM1:SAP10:C4_04500C_A:C4_04810C_A:AGP3:C4_05230C_A:OFD1:HOM3:GDH3:C4_06210C_A:SOU2:SOU1:C4_06790W_A:MET16:YDC1:C5_00030W_A:MET14:C5_00800C_A:TFS1:C5_01140C_A:C5_01430C_A:GLR1:GIS2:C5_02010C_A:HSP12:C5_02110W_A:RIX7:THR4:C5_02690W_A:C5_02730C_A:FUR1:C5_03530C_A:C5_03920C_A:C5_04290C_A:C5_04360C_A:CSU57:MSM1:C5_05130C_A:SDH4:C5_05340W_A:ILV3:C5_05440C_A:FET31:C6_00640C_A:C6_00760W_A:ILV5:KAR5:C6_01040C_A:EBP1:C6_01890C_A:C6_01980C_A:C6_02230W_A:C6_02410W_A:C6_02430W_A:GCV1:C6_02560W_A:BMT4:C6_03470W_A:FGR17:PAD1:C6_04240W_A:PEX11:C6_04530C_A:NAG3:NOP15:YML6:C7_01030C_A:DBP7:TOM40:C7_02100W_A:C7_02340C_A:C7_03030W_A:TIM54:DFR1:C7_03400C_A:PNC1:ISY1:ENP2:TIM9:C7_03850W_A:C7_03880C_A:UTP18:C7_04210C_A:POT1:LEU1:CR_00570W_A:CR_00670C_A:BUD22:LYS12:CR_01410C_A:CSP37:CR_01950W_A:CRC1:CR_02030C_A:CR_02420W_A:CR_02690W_A:RRP9:ERV1:DOM34:CR_03200C_A:CR_03240C_A:IFR2:KTI12:SGD1:PRP42:CR_03760W_A:CR_

						04110W_A:CR_04170W_A:QDR1:CR_04240C_A:RPS3:CR_04880W_A:YCP4:MTG2:DBP8:ACO2:SFL1:SOK1:FGR50:NMD3:CR_07030C_A:URA2:CR_07480W_A:CR_07640C_A:IMP4:CR_08000C_A:CR_08330W_A:CR_08410W_A:TSR1:SKI2:ECI1:PDK2:CR_08940W_A:SLP3:CR_09140C_A:CR_09310W_A:ELF1:CR_09670C_A:CR_09800C_A:UGA3:UTP5:CR_10400W_A:CR_10410C_A:CR_10470C_A:DRS1
2E+06	alpha-amino acid biosynthetic process	19 out of 537 genes, 3.5%	87 out of 6473 background genes, 1.3%	0.06974	0.11%	LEU4:HOM6:LYS2:GLT1:BAT21:LYS22:IDP1:LEU42:ILV2:MET13:HOM3:GDH3:MET16:THR4:DFR1:C7_04210C_A:LEU1:LYS12:CR_04880W_A

19752	carboxylic acid metabolic process	50 out of 537 genes, 9.3%	349 out of 6473 background genes, 5.4%	0.07323	0.11%	LEU4:HOM6:LYS2:GOR1:C1_03180W_A:GPM2:C1_05990C_A:GLT1:ANT1:MLS1:C1_13330C_A:CYB2:MET3:BAT21:LYS22:IDP1:ARA1:GLO2:LEU42:FOX2:POX1-3:ILV2:FAA21:MET13:CCC1:DLD2:CAR2:ILV6:HOM3:GDH3:MET16:MET14:C5_00800C_A:THR4:MSM1:ILV3:ILV5:GCV1:PAD1:PEX11:DFR1:C7_04210C_A:POT1:LEU1:LYS12:CRC1:CR_04880W_A:URA2:ECI1:UGA3
**S20 vs B20 Downregulated genes**						
GOID	GO_term	Cluster frequency	Background frequency	Corrected P-value	False discovery rate	Gene(s) annotated to the term
8643	carbohydrate transport	55 out of 182 genes, 30.2%	585 out of 6473 background genes, 9.0%	7.26E-14	0.00%	POL93:HGT2:GAL1:GAL10:EHD3:ICL1:C1_05160C_A:C1_06000W_A:C1_07980C_A:PFY1:MLS1:GCA1:AMO1:HSP21:DCK1:C2_05130W_A:RHO2:GDH2:DDR48:IRO1:GFA1:C3_02290W_A:FAA21:AAP1:CDR11:C3_04630W_A:C3_05550C_A:RCT1:AHR1:FBP1:C4_00860C_A:CAT2:AMS1:RBT1:HWP1:FAV1:CSR1:HAP41:ZRT101:GPR1:PGA37:C5_04470C_A:CUP9:OPT1:ADH5:IDP2:WOR3:LAP3:FDH1:FRE7:ECI1:SLP3:CHS2:CDG1:PLB3

42710	biofilm formation	16 out of 182 genes, 8.8%	155 out of 6473 background genes, 2.4%	0.00372	0.00%	BRG1:GCA1:YVC1:RAS1:TEC1:ZCF31:AHR1:PGA10:CAT2:HWP1:PHR1:CSR1:MRV8:ALS1:ADH5:WOR3
98630	aggregation of unicellular organisms	16 out of 182 genes, 8.8%	155 out of 6473 background genes, 2.4%	0.00372	0.00%	BRG1:GCA1:YVC1:RAS1:TEC1:ZCF31:AHR1:PGA10:CAT2:HWP1:PHR1:CSR1:MRV8:ALS1:ADH5:WOR3
98743	cell aggregation	16 out of 182 genes, 8.8%	155 out of 6473 background genes, 2.4%	0.00372	0.00%	BRG1:GCA1:YVC1:RAS1:TEC1:ZCF31:AHR1:PGA10:CAT2:HWP1:PHR1:CSR1:MRV8:ALS1:ADH5:WOR3
71702	organic substance transport	66 out of 182 genes, 36.3%	1458 out of 6473 background genes, 22.5%	0.00838	0.00%	POL93:HGT2:GAL1:GAL10:EHD3:POR1:ICL1:C1_05160C_A:C1_06000W_A:C1_07980C_A:PFY1:MLS1:ECM21:GCA1:DDI1:GDI1:AMO1:HSP21:DCK1:C2_05130W_A:RTA2:YVC1:RHO2:GDH2:DDR48:IRO1:GFA1:C3_02290W_A:FAA21:AAP1:CDR11:C3_04630W_A:ZCF31:NCE102:C3_05550C_A:RCT1:AHR1:FBP1:PEX5:PGA10:C4_00860C_A:CAT2:AMS1:RBT1:HWP1:OSH3:FAV1:CSR1:HAP41:ZRT101:GPR1:PGA37:C5_04470C_A:CUP9:OPT1:ADH5:IDP2:WOR3:LAP3:FDH1:FRE7:ECI1:SLP3:CHS2:CDG1:PLB3

44282	small molecule catabolic process	12 out of 182 genes, 6.6%	108 out of 6473 background genes, 1.7%	0.02713	0.00%	GAL1:GAL10:ICL1:MLS1:GDH2:PEX5:CAT2:AMS1:LAP3:FDH1:ECI1:CDG1
44242	cellular lipid catabolic process	7 out of 182 genes, 3.8%	36 out of 6473 background genes, 0.6%	0.03128	0.29%	ICL1:MLS1:PEX5:CAT2:GDE1:ECI1:PLB3
90609	single-species submerged biofilm formation	13 out of 182 genes, 7.1%	132 out of 6473 background genes, 2.0%	0.04672	0.50%	BRG1:GCA1:RAS1:TEC1:ZCF31:AHR1:PGA10:CAT2:HWP1:PHR1:CSR1:ALS1:ADH5
90605	submerged biofilm formation	13 out of 182 genes, 7.1%	135 out of 6473 background genes, 2.1%	0.05896	0.44%	BRG1:GCA1:RAS1:TEC1:ZCF31:AHR1:PGA10:CAT2:HWP1:PHR1:CSR1:ALS1:ADH5
9062	fatty acid catabolic process	5 out of 182 genes, 2.7%	18 out of 6473 background genes, 0.3%	0.0639	0.60%	ICL1:MLS1:PEX5:CAT2:ECI1

60256	regulation of flocculation	4 out of 182 genes, 2.2%	10 out of 6473 background genes, 0.2%	0.06717	0.55%	GAL10:RLM1:SFL2:ALS1
44010	single-species biofilm formation	13 out of 182 genes, 7.1%	138 out of 6473 background genes, 2.1%	0.07387	0.50%	BRG1:GCA1:RAS1:TEC1:ZCF31:AHR1:PGA10:CAT2:HWP1:PHR1:CSR1:ALS1:ADH5
44011	single-species biofilm formation on inanimate substrate	12 out of 182 genes, 6.6%	120 out of 6473 background genes, 1.9%	0.07646	0.46%	BRG1:GCA1:RAS1:TEC1:ZCF31:PGA10:CAT2:HWP1:PHR1:CSR1:ALS1:ADH5
						**S45 vs B45 Down**
GOID	GO_term	Cluster frequency	Background frequency	Corrected P-value	False discovery rate	Gene(s) annotated to the term

6302	double-strand break repair	30 out of 372 genes, 8.1%	120 out of 6473 background genes, 1.9%	3.29E-09	0.00%	CHL4:POL32:C1_06630W_A:CDC54:IRR1:RFA1:ESC4:CDC21:CDC46:RFA2:MCM3:PDS5:C2_08380C_A:SGS1:RDH54:POL1:C3_04740C_A:CAC1:C4_05790W_A:PRI2:EXO1:YBL053:CTF5:SMC6:MMS22:CTF18:RAD51:ALG5:SMC1:CDC28
724	double-strand break repair via homologous recombination	24 out of 372 genes, 6.5%	80 out of 6473 background genes, 1.2%	7.89E-09	0.00%	CHL4:POL32:C1_06630W_A:CDC54:IRR1:RFA1:CDC21:CDC46:RFA2:MCM3:PDS5:C2_08380C_A:SGS1:RDH54:C3_04740C_A:CAC1:C4_05790W_A:YBL053:CTF5:SMC6:MMS22:CTF18:RAD51:ALG5
727	double-strand break repair via break-induced replication	19 out of 372 genes, 5.1%	53 out of 6473 background genes, 0.8%	3.91E-08	0.00%	CHL4:POL32:C1_06630W_A:CDC54:IRR1:CDC21:CDC46:MCM3:PDS5:C2_08380C_A:C3_04740C_A:CAC1:C4_05790W_A:YBL053:CTF5:SMC6:MMS22:CTF18:ALG5
725	recombinational repair	24 out of 372 genes, 6.5%	87 out of 6473 background genes, 1.3%	5.73E-08	0.00%	CHL4:POL32:C1_06630W_A:CDC54:IRR1:RFA1:CDC21:CDC46:RFA2:MCM3:PDS5:C2_08380C_A:SGS1:RDH54:C3_04740C_A:CAC1:C4_05790W_A:YBL053:CTF5:SMC6:MMS22:CTF18:RAD51:ALG5

6310	DNA recombination	31 out of 372 genes, 8.3%	158 out of 6473 background genes, 2.4%	1.08E-06	0.00%	CHL4:POL32:C1_06630W_A:CDC54:RAD54:IRR1:RFA1:CDC21:CDC46:RFA2:MCM3:PDS5:C2_08380C_A:SGS1:RDH54:POL1:C3_04740C_A:CAC1:C4_05790W_A:MSH2:YBL053:C6_00770C_A:CTF5:SMC6:MMS22:CCE1:ATO10:CTF18:RAD51:ALG5:CDC28
6260	DNA replication	27 out of 372 genes, 7.3%	124 out of 6473 background genes, 1.9%	1.23E-06	0.00%	DUT1:POL32:C1_06630W_A:CDC6:CDC54:MSH6:RFA1:RNR1:CDC46:RFA2:MCM3:C2_08380C_A:SGS1:POL1:C3_04740C_A:POL30:PRI2:MSH2:YBL053:CAC2:RFC3:C5_05350W_A:RFC2:MMS22:CTF18:RAD51:RFC4
6261	DNA-templated DNA replication	25 out of 372 genes, 6.7%	112 out of 6473 background genes, 1.7%	3.04E-06	0.00%	POL32:C1_06630W_A:CDC6:CDC54:MSH6:RFA1:CDC46:RFA2:MCM3:C2_08380C_A:SGS1:POL1:C3_04740C_A:POL30:PRI2:MSH2:YBL053:CAC2:RFC3:C5_05350W_A:RFC2:MMS22:CTF18:RAD51:RFC4

8643	carbohydrate transport	68 out of 372 genes, 18.3%	585 out of 6473 background genes, 9.0%	7.34E-06	0.00%	POL93:HGC1:C1_00830W_A:HGT1:GAL1:GAL10:ALG9:CPH1:PLB5:C1_11140W_A:HYR1:C2_02570W_A:DCK1:ITR1:LSP1:PST1:RHO2:C3_00170C_A:ATO1:IRO1:C3_02880W_A:ALT1:CDR11:C3_05990C_A:AHR1:PGA7:OPT6:PDC12:RFX2:ECE1:RBT1:HWP1:AXL2:C4_04250W_A:FAV1:C4_05250W_A:C4_06770W_A:MSH2:GPR1:ECM331:SUT1:MAL31:SAP6:SAP5:C6_04410C_A:FRP2:HGT12:DFI1:C7_01510W_A:DPP3:C7_03500W_A:PCK1:CDA2:BIO3:ADH5:GEF2:IDP2:OPT4:WOR3:MCT1:CFL11:ALS3:CR_07220C_A:YHB5:TUB1:CDG1:YKE2:CR_10610C_A
6281	DNA repair	40 out of 372 genes, 10.8%	266 out of 6473 background genes, 4.1%	1.43E-05	0.00%	CHL4:POL32:C1_06630W_A:CDC54:MSH6:IRR1:RFA1:ESC4:CDC21:CDC46:RFA2:MCM3:PDS5:C2_08380C_A:SGS1:RDH54:POL1:C3_04740C_A:C3_06400C_A:POL30:CAC1:C4_05790W_A:PRI2:MSH2:C4_07200C_A:EXO1:YBL053:RFC3:C6_00770C_A:CTF5:RFC2:SMC6:MMS22:CTF18:RAD51:ALG5:SMC1:CDC28:CR_10000C_A:RFC4

6259	DNA metabolic process	51 out of 372 genes, 13.7%	408 out of 6473 background genes, 6.3%	7.59E-05	0.00%	POL93:CDC13:DUT1:CHL4:POL32:C1_06630W_A:CDC6:CDC54:MSH6:RAD54:IRR1:RFA1:ESC4:RNR1:CDC21:CDC46:RFA2:MCM3:PDS5:C2_08380C_A:SGS1:RDH54:POL1:C3_04740C_A:C3_06400C_A:POL30:CAC1:C4_05790W_A:PRI2:MSH2:C4_07200C_A:EXO1:CTF8:YBL053:CAC2:RFC3:C5_05350W_A:C6_00770C_A:CTF5:RFC2:SMC6:MMS22:CCE1:ATO10:CTF18:RAD51:ALG5:SMC1:CDC28:CR_10000C_A:RFC4
6974	DNA damage response	42 out of 372 genes, 11.3%	305 out of 6473 background genes, 4.7%	8.28E-05	0.00%	CHL4:POL32:C1_06630W_A:CDC54:MSH6:IRR1:RFA1:ESC4:CDC21:CDC46:RFA2:MCM3:PDS5:C2_08380C_A:SGS1:RDH54:POL1:C3_04740C_A:C3_06400C_A:POL30:RFX2:CAC1:C4_05790W_A:PRI2:MSH2:C4_07200C_A:EXO1:YBL053:YNK1:RFC3:C6_00770C_A:CTF5:RFC2:SMC6:MMS22:CTF18:RAD51:ALG5:SMC1:CDC28:CR_10000C_A:RFC4

22402	cell cycle process	55 out of 372 genes, 14.8%	465 out of 6473 background genes, 7.2%	0.00014	0.00%	TUB2:HGC1:CDC12:CHL4:POL32:C1_06630W_A:C1_10630C_A:GIN4:CDC6:RAX2:CDC54:MSH6:MYO2:IRR1:RFA1:ESC4:CDC46:RFA2:MCM3:PDS5:C2_08380C_A:SGS1:RDH54:POL1:MEA1:C3_04740C_A:POL30:AXL2:MSH2:CDC11:EXO1:CTF8:YBL053:C5_03370C_A:CAC2:PGA4:C6_00770C_A:CAN3:CTF5:RFC2:BEM2:SMC6:CDA2:ATO10:CTF18:RSR1:RAD51:RHO1:SMC1:CDC10:CDC28:CR_06740W_A:TPM2:TUB1:RFC4
7064	mitotic sister chromatid cohesion	12 out of 372 genes, 3.2%	34 out of 6473 background genes, 0.5%	0.00022	0.00%	CHL4:C1_06630W_A:C1_10630C_A:IRR1:PDS5:C3_04740C_A:POL30:CTF8:YBL053:CTF5:CTF18:SMC1
7062	sister chromatid cohesion	14 out of 372 genes, 3.8%	47 out of 6473 background genes, 0.7%	0.00023	0.00%	CHL4:C1_06630W_A:C1_10630C_A:IRR1:PDS5:C3_04740C_A:POL30:CTF8:YBL053:CTF5:RFC2:CTF18:SMC1:RFC4
51276	chromosome organization	34 out of 372 genes, 9.1%	233 out of 6473 background genes, 3.6%	0.0004	0.00%	CDC13:TUB2:C1_04490W_A:CHL4:C1_06630W_A:C1_10630C_A:CDC54:MSH6:RAD54:IRR1:RFA1:CDC46:RFA2:MCM3:PDS5:SGS1:RDH54:C3_04740C_A:POL30:MSH2:EXO1:CTF8:YBL053:CAC2:C6_00770C_A:CTF5:RFC2:CTF18:RAD51:SMC1:CDC28:CR_06740W_A:TUB1:RFC4

7049	cell cycle	56 out of 372 genes, 15.1%	493 out of 6473 backgroun d genes, 7.6%	0.00044	0.00%	TUB2:HGC1:CDC12:CHL4:POL32:C1_06630W_A:C1_10630C_A:GIN4:CDC6:RAX2:CDC54:MSH6:RAD54:MYO2:IRR1:RFA1:ESC4:CDC46:RFA2:MCM3:PDS5:C2_08380C_A:SGS1:RDH54:POL1:MEA1:C3_04740C_A:POL30:AXL2:MSH2:CDC11:EXO1:CTF8:YBL053:C5_03370C_A:CAC2:PGA4:C6_00770C_A:CAN3:CTF5:RFC2:BEM2:SMC6:CDA2:ATO10:CTF18:RSR1:RAD51:RHO1:SMC1:CDC10:CDC28:CR_06740W_A:TPM2:TUB1:RFC4
278	mitotic cell cycle	37 out of 372 genes, 9.9%	283 out of 6473 backgroun d genes, 4.4%	0.00193	0.00%	TUB2:CDC12:CHL4:C1_06630W_A:GIN4:CDC6:RAX2:CDC54:RAD54:MYO2:RFA1:MCM3:PDS5:C2_08380C_A:SGS1:POL1:MEA1:C3_04740C_A:POL30:AXL2:CDC11:EXO1:C5_03370C_A:CAC2:CAN3:CTF5:RFC2:BEM2:RSR1:RAD51:RHO1:SMC1:CDC10:CDC28:CR_06740W_A:TPM2:TUB1
33260	nuclear DNA replicatio n	13 out of 372 genes, 3.5%	49 out of 6473 backgroun d genes, 0.8%	0.00279	0.00%	POL32:C1_06630W_A:CDC6:CDC54:RFA1:CDC46:MCM3:C2_08380C_A:POL1:C3_04740C_A:POL30:CAC2:RAD51
44786	cell cycle DNA replicatio n	13 out of 372 genes, 3.5%	49 out of 6473 backgroun d genes, 0.8%	0.00279	0.00%	POL32:C1_06630W_A:CDC6:CDC54:RFA1:CDC46:MCM3:C2_08380C_A:POL1:C3_04740C_A:POL30:CAC2:RAD51

2E+06	mitotic DNA replication	10 out of 372 genes, 2.7%	29 out of 6473 background genes, 0.4%	0.00304	0.00%	C1_06630W_A:CDC54:RFA1:MCM3:C2_08380C_A:POL1:C3_04740C_A:POL30:CAC2:RAD51
71103	DNA conformation change	10 out of 372 genes, 2.7%	29 out of 6473 background genes, 0.4%	0.00304	0.00%	CDC54:RAD54:RFA1:CDC46:RFA2:MCM3:SGS1:RDH54:C3_04740C_A:C6_00770C_A
2E+06	mitotic cell cycle process	36 out of 372 genes, 9.7%	278 out of 6473 background genes, 4.3%	0.00338	0.00%	TUB2:CDC12:CHL4:C1_06630W_A:GIN4:CDC6:RAX2:CDC54:MYO2:RFA1:MCM3:PDS5:C2_08380C_A:SGS1:POL1:MEA1:C3_04740C_A:POL30:AXL2:CDC11:EXO1:C5_03370C_A:CAC2:CAN3:CTF5:RFC2:BEM2:RSR1:RAD51:RHO1:SMC1:CDC10:CDC28:CR_06740W_A:TPM2:TUB1
30491	heteroduplex formation	5 out of 372 genes, 1.3%	6 out of 6473 background genes, 0.1%	0.00407	0.00%	RAD54:RFA1:RFA2:RDH54:RAD51
42710	biofilm formation	24 out of 372 genes, 6.5%	155 out of 6473 background genes, 2.4%	0.00808	0.08%	HGC1:ECM33:CPH1:HYR1:YVC1:EAP1:RAS1:CAS4:ERG6:TEC1:AHR1:PGA7:ECE1:HWP1:PHR1:MRV8:SUN41:SAP6:SAP5:ADH5:ATG13:WOR3:ALS3:SUC1

98630	aggregation of unicellular organisms	24 out of 372 genes, 6.5%	155 out of 6473 background genes, 2.4%	0.00808	0.08%	HGC1:ECM33:CPH1:HYR1:YVC1:EAP1:RAS1:CAS4:ERG6:TEC1:AHR1:PGA7:ECE1:HWP1:PHR1:MRV8:SUN41:SAP6:SAP5:ADH5:ATG13:WOR3:ALS3:SUC1
98743	cell aggregation	24 out of 372 genes, 6.5%	155 out of 6473 background genes, 2.4%	0.00808	0.08%	HGC1:ECM33:CPH1:HYR1:YVC1:EAP1:RAS1:CAS4:ERG6:TEC1:AHR1:PGA7:ECE1:HWP1:PHR1:MRV8:SUN41:SAP6:SAP5:ADH5:ATG13:WOR3:ALS3:SUC1
22414	reproductive process	37 out of 372 genes, 9.9%	303 out of 6473 background genes, 4.7%	0.00985	0.07%	CDC12:CPH1:CLA4:GIN4:CDC54:MSH6:MYO2:RFA1:RFA2:PDS5:C2_08380C_A:SGS1:RDH54:CAS4:POL1:TEC1:C3_04740C_A:AHR1:POL30:FAV1:MSH2:EXO1:YBL053:PGA4:C6_00770C_A:CTF5:SHE3:CDA2:ATO10:RSR1:RAD51:RHO1:WOR3:CDC10:CDC28:TPM2:TUB1

51716	cellular response to stimulus	97 out of 372 genes, 26.1%	1127 out of 6473 background genes, 17.4%	0.01193	0.07%	HGC1:HGT1:GAL1:GAL10:YPT7:ECM33:CHL4:GPI7:POL32:UME6:C1_06630W_A:RAC1:CPH1:KEX2:CLA4:GIN4:KIP4:CDC6:CDC54:MSH6:MYO2:IRR1:RFA1:SOD5:ESC4:MSB2:FAB1:RNR1:DCK1:FGR51:RHO3:CDC21:CDC46:LTE1:PST1:RFA2:MCM3:PDS5:RHO2:C2_08380C_A:C2_09650W_A:RAS1:FGR6-4:SGS1:RDH54:C3_00170C_A:ARF3:CAS4:POL1:TEC1:C3_04740C_A:RDI1:AHR1:C3_06400C_A:SEC61:RLM1:POL30:RFX2:PGA59:CAC1:PHR1:FGR6-10:C4_05790W_A:PRI2:MSH2:C4_07200C_A:EXO1:GPR1:YBL053:ACH1:YNK1:RFC3:SFL2:FET34:C6_00770C_A:SUN41:C6_01460C_A:CTF5:SHE3:HGT12:DFI1:RFC2:BEM2:SMC6:LMO1:MMS22:CTF18:RSR1:RAD51:ALG5:RHO1:SMC1:CDC28:YHB1:CR_10000C_A:RFC4:MAL2
32392	DNA geometric change	8 out of 372 genes, 2.2%	21 out of 6473 background genes, 0.3%	0.01352	0.07%	CDC54:RAD54:RFA1:CDC46:RFA2:MCM3:SGS1:RDH54

50896	response to stimulus	106 out of 372 genes, 28.5%	1272 out of 6473 background genes, 19.7%	0.01727	0.07%	HGC1:HGT1:GAL1:GAL10:YPT7:ECM33:CHL4:GPI7:POL32:UME6:C1_06630W_A:RAC1:CPH1:KEX2:CLA4:GIN4:KIP4:CDC6:CDC54:MSH6:HYR1:MYO2:IRR1:RFA1:SOD5:ESC4:MSB2:FAB1:C2_02570W_A:RNR1:DCK1:FGR51:RHO3:CDC21:CDC46:LSP1:LTE1:PST1:RFA2:MCM3:PDS5:RHO2:C2_08100W_A:C2_08380C_A:C2_09650W_A:RAS1:FGR6-4:SGS1:RDH54:C3_00170C_A:ARF3:CAS4:CDR11:POL1:TEC1:C3_04740C_A:RDI1:AHR1:C3_06400C_A:SEC61:RLM1:POL30:RFX2:PGA59:CAC1:PHR1:FGR6-10:C4_05790W_A:PRI2:MSH2:C4_07200C_A:EXO1:GPR1:YBL053:ACH1:YNK1:RFC3:SFL2:FET34:C6_00770C_A:SUN41:GPX2:C6_01460C_A:CTF5:SAP6:SHE3:HGT12:DFI1:RFC2:BEM2:SMC6:LMO1:PCK1:MMS22:CTF18:RSR1:RAD51:OPT4:ALG5:RHO1:SMC1:CDC28:YHB1:CR_10000C_A:RFC4:MAL2

33554	cellular response to stress	76 out of 372 genes, 20.4%	833 out of 6473 background genes, 12.9%	0.01942	0.06%	HGT1:GAL10:ECM33:CHL4:GPI7:POL32:UME6:C1_06630W_A:CPH1:KEX2:CLA4:KIP4:CDC54:MSH6:MYO2:IRR1:RFA1:SOD5:ESC4:MSB2:FAB1:RNR1:DCK1:FGR51:CDC21:CDC46:PST1:RFA2:MCM3:PDS5:C2_08380C_A:RAS1:FGR6-4:SGS1:RDH54:CAS4:POL1:TEC1:C3_04740C_A:RDI1:AHR1:C3_06400C_A:SEC61:RLM1:POL30:RFX2:CAC1:FGR6-10:C4_05790W_A:PRI2:MSH2:C4_07200C_A:EXO1:GPR1:YBL053:YNK1:RFC3:FET34:C6_00770C_A:C6_01460C_A:CTF5:SHE3:HGT12:RFC2:SMC6:MMS22:CTF18:RSR1:RAD51:ALG5:SMC1:CDC28:YHB1:CR_10000C_A:RFC4:MAL2
7163	establishment or maintenance of cell polarity	19 out of 372 genes, 5.1%	112 out of 6473 background genes, 1.7%	0.01988	0.06%	TUB2:CDC12:CLA4:RAX2:MYO2:MSB2:RHO3:RHO2:RAS1:CAS4:MEA1:AXL2:CDC11:C6_02090C_A:C7_03480W_A:RSR1:RHO1:CR_06740W_A:TUB1
3	reproduction	41 out of 372 genes, 11.0%	364 out of 6473 background genes, 5.6%	0.0232	0.06%	CDC12:CPH1:CLA4:GIN4:RAX2:CDC54:MSH6:MYO2:RFA1:RFA2:PDS5:C2_08380C_A:RAS1:SGS1:RDH54:CAS4:POL1:TEC1:C3_04740C_A:AHR1:POL30:AXL2:FAV1:MSH2:CDC11:EXO1:YBL053:PGA4:C6_00770C_A:CTF5:SHE3:CDA2:ATO10:RSR1:RAD51:RHO1:WOR3:CDC10:CDC28:TPM2:TUB1

6950	response to stress	79 out of 372 genes, 21.2%	882 out of 6473 background genes, 13.6%	0.02504	0.06%	HGT1:GAL10:ECM33:CHL4:GPI7:POL32:UME6:C1_06630W_A:CPH1:KEX2:CLA4:KIP4:CDC54:MSH6:MYO2:IRR1:RFA1:SOD5:ESC4:MSB2:FAB1:RNR1:DCK1:FGR51:CDC21:CDC46:LSP1:PST1:RFA2:MCM3:PDS5:C2_08100W_A:C2_08380C_A:RAS1:FGR6-4:SGS1:RDH54:CAS4:POL1:TEC1:C3_04740C_A:RDI1:AHR1:C3_06400C_A:SEC61:RLM1:POL30:RFX2:CAC1:FGR6-10:C4_05790W_A:PRI2:MSH2:C4_07200C_A:EXO1:GPR1:YBL053:YNK1:RFC3:FET34:C6_00770C_A:GPX2:C6_01460C_A:CTF5:SHE3:HGT12:RFC2:SMC6:MMS22:CTF18:RSR1:RAD51:ALG5:SMC1:CDC28:YHB1:CR_10000C_A:RFC4:MAL2
44182	filamentous growth of a population of unicellular organisms	49 out of 372 genes, 13.2%	470 out of 6473 background genes, 7.3%	0.02853	0.06%	HGC1:HGT1:GAL10:ECM33:GPI7:UME6:RAC1:CPH1:KEX2:CLA4:GIN4:KIP4:MYO2:ESC4:MSB2:FAB1:RNR1:DCK1:FGR51:YVC1:RAS1:FGR6-4:SGS1:CAS4:TEC1:RDI1:AHR1:RFX2:PGA59:PHR1:FGR6-10:CDC11:GPR1:SFL2:FET34:SUN41:SHE3:HGT12:DFI1:LMO1:CHT1:LAG1:RSR1:RAD51:CDC10:ALS3:YHB1:TPM2:MAL2

8360	regulation of cell shape	10 out of 372 genes, 2.7%	37 out of 6473 background genes, 0.6%	0.03494	0.17%	TUB2:MYO2:RHO3:RAS1:C3_00170C_A:CAS4:C7_03480W_A:RHO1:CR_06740W_A:TUB1
22616	DNA strand elongation	9 out of 372 genes, 2.4%	30 out of 6473 background genes, 0.5%	0.03521	0.16%	C1_06630W_A:CDC54:MCM3:SGS1:POL30:EXO1:RFC3:RFC2:RFC4
32508	DNA duplex unwinding	7 out of 372 genes, 1.9%	18 out of 6473 background genes, 0.3%	0.04183	0.21%	CDC54:RFA1:CDC46:RFA2:MCM3:SGS1:RDH54
7264	small GTPase mediated signal transduction	13 out of 372 genes, 3.5%	62 out of 6473 background genes, 1.0%	0.04462	0.21%	YPT7:RAC1:RHO3:LTE1:RHO2:C2_09650W_A:RAS1:C3_00170C_A:ARF3:RDI1:BEM2:RSR1:RHO1

40007	growth	61 out of 372 genes, 16.4%	641 out of 6473 background genes, 9.9%	0.04553	0.20%	HGC1:HGT1:GAL10:ECM33:CDC12:GPI7:POL32:UME6:RAC1:CPH1:KEX2:CLA4:GIN4:KIP4:RAX2:MYO2:ESC4:MSB2:FAB1:RNR1:DCK1:FGR51:RHO3:YVC1:RAS1:FGR6-4:SGS1:IRO1:CAS4:VAM3:TEC1:RDI1:AHR1:RFX2:PGA59:PHR1:FGR6-10:CDC11:GPR1:YNK1:SFL2:FET34:SUN41:SHE3:HGT12:DFI1:FGR6-1:LMO1:C7_03480W_A:CHT1:LAG1:RSR1:RAD51:RHO1:CDC10:CDC28:CFL11:ALS3:YHB1:TPM2:MAL2
48285	organelle fission	21 out of 372 genes, 5.6%	140 out of 6473 background genes, 2.2%	0.04975	0.20%	TUB2:CHL4:GIN4:RFA1:RFA2:PDS5:SGS1:RDH54:C3_04740C_A:CAC2:C6_00770C_A:CTF5:ATO10:RSR1:RAD51:SMC1:CDC10:CR_05730C_A:CDC28:CR_06740W_A:TUB1
51647	nucleus localization	8 out of 372 genes, 2.2%	25 out of 6473 background genes, 0.4%	0.05864	0.24%	TUB2:MYO2:CDC11:RSR1:CDC10:CR_06740W_A:CR_07220C_A:TUB1
7097	nuclear migration	8 out of 372 genes, 2.2%	25 out of 6473 background genes, 0.4%	0.05864	0.23%	TUB2:MYO2:CDC11:RSR1:CDC10:CR_06740W_A:CR_07220C_A:TUB1

30447	filamentous growth	60 out of 372 genes, 16.1%	633 out of 6473 background genes, 9.8%	0.0599	0.23%	HGC1:HGT1:GAL10:ECM33:CDC12:GPI7:POL32:UME6:RAC1:CPH1:KEX2:CLA4:GIN4:KIP4:RAX2:MYO2:ESC4:MSB2:FAB1:RNR1:DCK1:FGR51:RHO3:YVC1:RAS1:FGR6-4:SGS1:IRO1:CAS4:VAM3:TEC1:RDI1:AHR1:RFX2:PGA59:PHR1:FGR6-10:CDC11:GPR1:SFL2:FET34:SUN41:SHE3:HGT12:DFI1:FGR6-1:LMO1:C7_03480W_A:CHT1:LAG1:RSR1:RAD51:RHO1:CDC10:CDC28:CFL11:ALS3:YHB1:TPM2:MAL2
280	nuclear division	20 out of 372 genes, 5.4%	132 out of 6473 background genes, 2.0%	0.0654	0.22%	TUB2:CHL4:GIN4:RFA1:RFA2:PDS5:SGS1:RDH54:C3_04740C_A:CAC2:C6_00770C_A:CTF5:ATO10:RSR1:RAD51:SMC1:CDC10:CDC28:CR_06740W_A:TUB1
51701	biological process involved in interaction with host	20 out of 372 genes, 5.4%	132 out of 6473 background genes, 2.0%	0.0654	0.22%	ECM33:CPH1:KEX2:HYR1:SOD5:EAP1:RAS1:TEC1:RFX2:ECE1:HWP1:PHR1:GPR1:SUN41:SAP6:SAP5:SHE3:WOR3:CDC10:ALS3
22604	regulation of cell morphogenesis	10 out of 372 genes, 2.7%	40 out of 6473 background genes, 0.6%	0.07282	0.21%	TUB2:MYO2:RHO3:RAS1:C3_00170C_A:CAS4:C7_03480W_A:RHO1:CR_06740W_A:TUB1

44403	biological process involved in symbiotic interaction	21 out of 372 genes, 5.6%	144 out of 6473 background genes, 2.2%	0.07608	0.25%	ECM33:CPH1:KEX2:HYR1:SOD5:EAP1:RAS1:TEC1:AHR1:RFX2:ECE1:HWP1:PHR1:GPR1:SUN41:SAP6:SAP5:SHE3:WOR3:CDC10:ALS3
44419	biological process involved in interspecies interaction between organisms	38 out of 372 genes, 10.2%	347 out of 6473 background genes, 5.4%	0.0883	0.29%	HGC1:ECM33:GPI7:UME6:RBT4:CPH1:KEX2:CLA4:MCD4:HYR1:SOD5:PST1:YVC1:EAP1:RAS1:IRO1:TEC1:AHR1:PGA7:RFX2:ECE1:RBT1:HWP1:PHR1:CDC11:GPR1:SFL2:FET34:SUN41:SAP6:SAP5:SHE3:RSR1:WOR3:CDC10:CFL11:ALS3:YHB1
**S120 vs B120 Downregulated genes**						
GOID	GO_term	Cluster frequency	Background frequency	Corrected P-value	False discovery rate	Gene(s) annotated to the term

8643	carbohydrate transport	83 out of 356 genes, 23.3%	585 out of 6473 background genes, 9.0%	6.70E-14	0.00%	HMX1:HGC1:C1_00830W_A:TRS20:C1_01620C_A:HOL4:HGT1:CPH1:PLB5:HYR1:KAR9:FTR1:DCK1:FRP3:LSP1:PST1:RHO2:IRF1:DDR48:ATO1:IRO1:UTR2:GFA1:C3_02290W_A:CDR11:RAX1:C3_05990C_A:AHR1:EVP1:FRP1:PGA7:OPT6:RFX2:ECE1:RBT1:HWP1:AXL2:C4_04250W_A:FAV1:CSR1:C4_04980W_A:C4_05080C_A:C4_05250W_A:ARO9:C4_05980C_A:C4_06770W_A:C5_00230C_A:GPR1:C5_01440C_A:ECM331:PUT1:C5_04260W_A:MAL31:SAP6:SAP5:ARG3:C6_03260W_A:SAP4:CSA1:FRP2:C7_00230W_A:DFI1:C7_00630C_A:DPP3:PGA28:C7_03500W_A:ARG1:GEF2:IDP2:WOR3:RBR1:DIT2:CR_05540C_A:MCT1:ALS3:CR_07220C_A:YHB5:PGA13:CHS2:TUB1:YKE2:PLB3:CR_10610C_A
42710	biofilm formation	31 out of 356 genes, 8.7%	155 out of 6473 background genes, 2.4%	1.92E-07	0.00%	HGC1:ECM33:CPH1:IPT1:HYR1:CHK1:YVC1:EAP1:RAS1:CAS4:ERG6:AHR1:ZFU2:PGA7:RBT5:ECE1:HWP1:PHR1:CSR1:CLN3:CCN1:SUN41:SAP6:SAP5:CSA1:ATG13:WOR3:BCR1:ALS3:SUC1:DEF1

98630	aggregation of unicellular organisms	31 out of 356 genes, 8.7%	155 out of 6473 background genes, 2.4%	1.92E-07	0.00%	HGC1:ECM33:CPH1:IPT1:HYR1:CHK1:YVC1:EAP1:RAS1:CAS4:ERG6:AHR1:ZFU2:PGA7:RBT5:ECE1:HWP1:PHR1:CSR1:CLN3:CCN1:SUN41:SAP6:SAP5:CSA1:ATG13:WOR3:BCR1:ALS3:SUC1:DEF1
98743	cell aggregation	31 out of 356 genes, 8.7%	155 out of 6473 background genes, 2.4%	1.92E-07	0.00%	HGC1:ECM33:CPH1:IPT1:HYR1:CHK1:YVC1:EAP1:RAS1:CAS4:ERG6:AHR1:ZFU2:PGA7:RBT5:ECE1:HWP1:PHR1:CSR1:CLN3:CCN1:SUN41:SAP6:SAP5:CSA1:ATG13:WOR3:BCR1:ALS3:SUC1:DEF1
7163	establishment or maintenance of cell polarity	25 out of 356 genes, 7.0%	112 out of 6473 background genes, 1.7%	1.07E-06	0.00%	TUB2:CDC12:MSB1:CLA4:RAX2:MYO2:MSB2:RHO3:RHO2:RAS1:CAS4:RAX1:MEA1:AXL2:CDC11:CCN1:INT1:C5_03170C_A:C6_02090C_A:C7_03480W_A:RSR1:RHO1:CR_06740W_A:TUB1:CR_09520C_A
44010	single-species biofilm formation	28 out of 356 genes, 7.9%	138 out of 6473 background genes, 2.1%	1.07E-06	0.00%	HGC1:ECM33:CPH1:IPT1:HYR1:CHK1:EAP1:RAS1:CAS4:AHR1:ZFU2:PGA7:RBT5:ECE1:HWP1:PHR1:CSR1:CLN3:CCN1:SUN41:SAP6:SAP5:CSA1:ATG13:BCR1:ALS3:SUC1:DEF1

44419	biological process involved in interspecies interaction between organisms	48 out of 356 genes, 13.5%	347 out of 6473 background genes, 5.4%	1.50E-06	0.00%	HGC1:ECM33:MSB1:UME6:RBT4:CPH1:IPT1:CLA4:MCD4:CHS3:HYR1:FTR1:SOD5:CTF1:CHK1:PST1:YVC1:PHM7:EAP1:RAS1:IRO1:UTR2:YCK2:AHR1:PGA7:RFX2:SOD1:ECE1:RBT1:HWP1:PHR1:CDC11:GPR1:INT1:FET34:SUN41:SAP6:SAP5:SHE3:SAP4:CHS1:RSR1:WOR3:CDC10:BCR1:ALS3:YHB1:DEF1
51647	nucleus localization	12 out of 356 genes, 3.4%	25 out of 6473 background genes, 0.4%	1.79E-06	0.00%	TUB2:C1_11250W_A:KAR9:MYO2:KIP2:CDC11:MYO1:RSR1:CDC10:CR_06740W_A:CR_07220C_A:TUB1
7097	nuclear migration	12 out of 356 genes, 3.4%	25 out of 6473 background genes, 0.4%	1.79E-06	0.00%	TUB2:C1_11250W_A:KAR9:MYO2:KIP2:CDC11:MYO1:RSR1:CDC10:CR_06740W_A:CR_07220C_A:TUB1
51301	cell division	27 out of 356 genes, 7.6%	134 out of 6473 background genes, 2.1%	2.55E-06	0.00%	TEM1:CDC12:MSB1:CDC3:GIN4:RAX2:MYO2:CAS4:RAX1:MEA1:AXL2:CDC11:CLN3:MYO1:INT1:C5_03170C_A:FET34:SUN41:CTF5:IQG1:CHS1:RSR1:CR_02210W_A:RHO1:CDC10:TPM2:CHS2
910	cytokinesis	21 out of 356 genes, 5.9%	87 out of 6473 background genes, 1.3%	5.44E-06	0.00%	TEM1:CDC12:CDC3:GIN4:RAX2:MYO2:RAX1:MEA1:AXL2:CDC11:MYO1:INT1:C5_03170C_A:IQG1:CHS1:RSR1:CR_02210W_A:RHO1:CDC10:TPM2:CHS2

19954	asexual reproduction	21 out of 356 genes, 5.9%	95 out of 6473 background genes, 1.5%	2.92E-05	0.00%	CLA4:GIN4:RAX2:CHS3:MYO2:RAS1:CAS4:RAX1:AXL2:CDC11:CLN3:INT1:C5_03170C_A:ARG3:CHS1:RSR1:CR_02210W_A:RHO1:CDC10:DIT2:CHS2
51701	biological process involved in interaction with host	25 out of 356 genes, 7.0%	132 out of 6473 background genes, 2.0%	3.78E-05	0.00%	ECM33:MSB1:CPH1:HYR1:FTR1:SOD5:EAP1:RAS1:UTR2:YCK2:RFX2:ECE1:HWP1:PHR1:GPR1:INT1:SUN41:SAP6:SAP5:SHE3:SAP4:WOR3:CDC10:BCR1:ALS3
2E+06	organelle disassembly	37 out of 356 genes, 10.4%	256 out of 6473 background genes, 4.0%	4.23E-05	0.00%	TUB2:CDC3:SFH5:KAR9:C2_01340W_A:SRD1:DCK1:SPO7:LSP1:NGT1:IRO1:RAX1:C3_05990C_A:ZFU2:CSP2:C4_01220C_A:OPT6:ZCF27:RFX2:C4_03460C_A:FAV1:C4_04980W_A:C4_05080C_A:GPR1:C5_01440C_A:ZCF20:PUT1:GPX2:C6_01620W_A:LMO1:SNG3:CR_01280C_A:ATG13:CR_05540C_A:BCR1:CR_07220C_A:YKE2
44403	biological process involved in symbiotic interaction	26 out of 356 genes, 7.3%	144 out of 6473 background genes, 2.2%	5.57E-05	0.00%	ECM33:MSB1:CPH1:HYR1:FTR1:SOD5:EAP1:RAS1:UTR2:YCK2:AHR1:RFX2:ECE1:HWP1:PHR1:GPR1:INT1:SUN41:SAP6:SAP5:SHE3:SAP4:WOR3:CDC10:BCR1:ALS3

44011	single-species biofilm formation on inanimate substrate	23 out of 356 genes, 6.5%	120 out of 6473 background genes, 1.9%	0.0001	0.00%	HGC1:ECM33:CPH1:IPT1:HYR1:CHK1:EAP1:RAS1:CAS4:ZFU2:PGA7:RBT5:ECE1:HWP1:PHR1:CSR1:SUN41:SAP6:CSA1:BCR1:ALS3:SUC1:DEF1
281	mitotic cytokinesis	17 out of 356 genes, 4.8%	69 out of 6473 background genes, 1.1%	0.00011	0.00%	CDC12:CDC3:RAX2:MYO2:RAX1:MEA1:AXL2:CDC11:MYO1:INT1:C5_03170C_A:IQG1:CHS1:RSR1:RHO1:CDC10:TPM2
30447	filamentous growth	66 out of 356 genes, 18.5%	633 out of 6473 background genes, 9.8%	0.00014	0.00%	HGC1:HGT1:ECM33:CDC12:CDC3:C1_05980W_A:UME6:C1_07220W_A:CPH1:FGR6-3:IPT1:CLA4:GIN4:KIP4:RAX2:CHS3:MYO2:MSB2:CHK1:DCK1:RHO3:YVC1:DDR48:SSO2:RAS1:IRO1:CAS4:UTR2:GFA1:C3_02290W_A:VAM3:RDI1:YCK2:AHR1:ZFU2:ZCF27:RFX2:SOD1:PHR1:CSR1:FGR6-10:CDC11:CLN3:GPR1:CCN1:INT1:FET34:SUN41:STE23:SHE3:DFI1:CHS1:LMO1:C7_03480W_A:LAG1:RSR1:RHO1:RBR1:CDC10:BCR1:ALS3:YHB1:TPM2:PGA13:CHS2:DEF1

61640	cytoskeleton-dependent cytokinesis	17 out of 356 genes, 4.8%	70 out of 6473 background genes, 1.1%	0.00014	0.00%	CDC12:CDC3:RAX2:MYO2:RAX1:MEA1:AXL2:CDC11:MYO1:INT1:C5_03170C_A:IQG1:CHS1:RSR1:RHO1:CDC10:TPM2
90609	single-species submerged biofilm formation	24 out of 356 genes, 6.7%	132 out of 6473 background genes, 2.0%	0.00016	0.00%	HGC1:ECM33:CPH1:IPT1:HYR1:CHK1:EAP1:RAS1:CAS4:AHR1:ZFU2:PGA7:RBT5:ECE1:HWP1:PHR1:CSR1:SUN41:SAP6:CSA1:BCR1:ALS3:SUC1:DEF1
422	autophagy of mitochondrion	34 out of 356 genes, 9.6%	236 out of 6473 background genes, 3.6%	0.00016	0.00%	SFH5:KAR9:C2_01340W_A:SRD1:DCK1:LSP1:NGT1:IRO1:RAX1:C3_05990C_A:ZFU2:CSP2:C4_01220C_A:OPT6:ZCF27:RFX2:C4_03460C_A:FAV1:C4_04980W_A:C4_05080C_A:GPR1:C5_01440C_A:ZCF20:PUT1:GPX2:C6_01620W_A:LMO1:SNG3:CR_01280C_A:ATG13:CR_05540C_A:BCR1:CR_07220C_A:YKE2
61726	mitochondrion disassembly	34 out of 356 genes, 9.6%	236 out of 6473 background genes, 3.6%	0.00016	0.00%	SFH5:KAR9:C2_01340W_A:SRD1:DCK1:LSP1:NGT1:IRO1:RAX1:C3_05990C_A:ZFU2:CSP2:C4_01220C_A:OPT6:ZCF27:RFX2:C4_03460C_A:FAV1:C4_04980W_A:C4_05080C_A:GPR1:C5_01440C_A:ZCF20:PUT1:GPX2:C6_01620W_A:LMO1:SNG3:CR_01280C_A:ATG13:CR_05540C_A:BCR1:CR_07220C_A:YKE2

40007	growth	66 out of 356 genes, 18.5%	641 out of 6473 background genes, 9.9%	0.00022	0.00%	HGC1:HGT1:ECM33:CDC12:CDC3:C1_05980W_A:UME6:C1_07220W_A:CPH1:FGR6-3:IPT1:CLA4:GIN4:KIP4:RAX2:CHS3:MYO2:MSB2:CHK1:DCK1:RHO3:YVC1:DDR48:SSO2:RAS1:IRO1:CAS4:UTR2:GFA1:C3_02290W_A:VAM3:RDI1:YCK2:AHR1:ZFU2:ZCF27:RFX2:SOD1:PHR1:CSR1:FGR6-10:CDC11:CLN3:GPR1:CCN1:INT1:FET34:SUN41:STE23:SHE3:DFI1:CHS1:LMO1:C7_03480W_A:LAG1:RSR1:RHO1:RBR1:CDC10:BCR1:ALS3:YHB1:TPM2:PGA13:CHS2:DEF1
90605	submerged biofilm formation	24 out of 356 genes, 6.7%	135 out of 6473 background genes, 2.1%	0.00024	0.00%	HGC1:ECM33:CPH1:IPT1:HYR1:CHK1:EAP1:RAS1:CAS4:AHR1:ZFU2:PGA7:RBT5:ECE1:HWP1:PHR1:CSR1:SUN41:SAP6:CSA1:BCR1:ALS3:SUC1:DEF1
99111	microtubule-based transport	9 out of 356 genes, 2.5%	20 out of 6473 background genes, 0.3%	0.00042	0.00%	TUB2:C1_11250W_A:KAR9:KIP2:MYO1:CR_06740W_A:CR_07220C_A:TUB1:CR_09520C_A
7018	microtubule-based movement	10 out of 356 genes, 2.8%	26 out of 6473 background genes, 0.4%	0.00055	0.00%	TUB2:C1_11250W_A:KIP4:KAR9:KIP2:MYO1:CR_06740W_A:CR_07220C_A:TUB1:CR_09520C_A

44406	adhesion of symbiont to host	16 out of 356 genes, 4.5%	69 out of 6473 background genes, 1.1%	0.00068	0.00%	ECM33:MSB1:HYR1:EAP1:UTR2:RFX2:HWP1:PHR1:SUN41:SAP6:SAP5:SAP4:WOR3:CDC10:BCR1:ALS3
33212	iron import into cell	6 out of 356 genes, 1.7%	8 out of 6473 background genes, 0.1%	0.0007	0.00%	FTR1:FRP1:ZCF20:FET34:FRP2:ALS3
30705	cytoskeleton-dependent intracellular transport	9 out of 356 genes, 2.5%	21 out of 6473 background genes, 0.3%	0.0007	0.00%	TUB2:C1_11250W_A:KAR9:MYO2:KIP2:MYO1:CR_07220C_A:TUB1:CR_09520C_A
22411	cellular component disassembly	39 out of 356 genes, 11.0%	310 out of 6473 background genes, 4.8%	0.00076	0.00%	TUB2:CDC3:SFH5:C1_11250W_A:KAR9:C2_01340W_A:SRD1:DCK1:SPO7:LSP1:NGT1:IRO1:VAM3:RAX1:C3_05990C_A:ZFU2:CSP2:C4_01220C_A:OPT6:ZCF27:RFX2:C4_03460C_A:FAV1:C4_04980W_A:C4_05080C_A:GPR1:C5_01440C_A:ZCF20:PUT1:GPX2:C6_01620W_A:LMO1:SNG3:CR_01280C_A:ATG13:CR_05540C_A:BCR1:CR_07220C_A:YKE2

7010	cytoskeleton organization	28 out of 356 genes, 7.9%	188 out of 6473 background genes, 2.9%	0.00111	0.00%	TUB2:TEM1:CDC12:CDC3:HCM1:CLA4:C1_11250W_A:GIN4:KAR9:MYO2:RHO3:BUD14:RHO2:KIP2:RDI1:CDC11:MYO1:INT1:BEM2:IQG1:CHS1:RSR1:RHO1:CDC10:CR_06740W_A:TPM2:TUB1:CR_09520C_A
6879	intracellular iron ion homeostasis	13 out of 356 genes, 3.7%	50 out of 6473 background genes, 0.8%	0.00193	0.00%	HMX1:FTR1:PHO13:IRO1:FRP1:RBT5:C4_04760C_A:ZCF20:CCC2:FET34:CSA1:FRP2:ALS3
30448	hyphal growth	19 out of 356 genes, 5.3%	103 out of 6473 background genes, 1.6%	0.00259	0.00%	CDC12:CDC3:RAX2:CHS3:MYO2:RHO3:SSO2:GFA1:VAM3:RDI1:YCK2:CDC11:CLN3:CCN1:CHS1:RSR1:RHO1:CDC10:CHS2
8360	regulation of cell shape	11 out of 356 genes, 3.1%	37 out of 6473 background genes, 0.6%	0.00289	0.00%	TUB2:MYO2:RHO3:BUD14:RAS1:CAS4:C7_03480W_A:RHO1:CR_06740W_A:TUB1:CR_09520C_A

44182	filamentous growth of a population of unicellular organisms	50 out of 356 genes, 14.0%	470 out of 6473 background genes, 7.3%	0.00335	0.00%	HGC1:HGT1:ECM33:C1_05980W_A:UME6:CPH1:FGR6-3:IPT1:CLA4:GIN4:KIP4:MYO2:MSB2:CHK1:DCK1:YVC1:DDR48:RAS1:CAS4:UTR2:C3_02290W_A:RDI1:AHR1:ZFU2:ZCF27:RFX2:SOD1:PHR1:CSR1:FGR6-10:CDC11:CLN3:GPR1:CCN1:INT1:FET34:SUN41:STE23:SHE3:DFI1:LMO1:LAG1:RSR1:RBR1:CDC10:ALS3:YHB1:TPM2:PGA13:DEF1
98771	inorganic ion homeostasis	19 out of 356 genes, 5.3%	105 out of 6473 background genes, 1.6%	0.0035	0.00%	HMX1:CTR2:FTR1:YVC1:PHO13:IRO1:FRP1:RBT5:SOD1:C4_04760C_A:CSR1:C4_06770W_A:ZCF20:CCC2:FET34:CSA1:FRP2:GEF2:ALS3
10970	transport along microtubule	8 out of 356 genes, 2.2%	19 out of 6473 background genes, 0.3%	0.00354	0.00%	TUB2:C1_11250W_A:KAR9:KIP2:MYO1:CR_07220C_A:TUB1:CR_09520C_A
31109	microtubule polymerization or depolymerization	6 out of 356 genes, 1.7%	10 out of 6473 background genes, 0.2%	0.00477	0.00%	TUB2:C1_11250W_A:KIP2:CR_06740W_A:TUB1:CR_09520C_A

71702	organic substance transport	116 out of 356 genes, 32.6%	1458 out of 6473 background genes, 22.5%	0.00479	0.00%	HMX1:HGC1:C1_00830W_A:TRS20:C1_01620C_A:HOL4:HGT1:YPT7:TEM1:C1_05980W_A:CPH1:PLB5:CLA4:SFH5:MCD4:HYR1:KAR9:MYO2:FTR1:SOD5:C2_01340W_A:CHK1:DCK1:RHO3:FRP3:LSP1:PST1:C2_07220W_A:YVC1:RHO2:IRF1:DDR48:YEA4:SSO2:NGT1:ATO1:IRO1:ARF3:UTR2:GFA1:C3_02290W_A:CDR11:VAM3:RAX1:CDR1:C3_05990C_A:AHR1:EVP1:FRP1:PGA7:RBT5:SPC2:C4_01220C_A:OPT6:RFX2:ECE1:RBT1:HWP1:AXL2:C4_04250W_A:FAV1:CSR1:C4_04980W_A:C4_05080C_A:C4_05250W_A:ARO9:C4_05980C_A:C4_06770W_A:C5_00230C_A:GPR1:C5_01440C_A:ZCF20:ECM331:PUT1:C5_04260W_A:MAL31:C6_01620W_A:PTK2:C6_02160W_A:SAP6:SAP5:ARG3:C6_03260W_A:SAP4:CSA1:FRP2:C7_00230W_A:DFI1:C7_00630C_A:DPP3:PGA28:C7_03500W_A:ARG1:CR_01280C_A:GEF2:IDP2:RHO1:ATG13:WOR3:RBR1:DIT2:CR_05540C_A:MCT1:CR_06790C_A:ALS3:CR_07220C_A:YHB5:PGA13:CHS2:TUB1:SUC1:CR_09520C_A:CR_09570W_A:YKE2:PLB3:CR_10610C_A

22604	regulation of cell morphogenesis	11 out of 356 genes, 3.1%	40 out of 6473 background genes, 0.6%	0.00673	0.00%	TUB2:MYO2:RHO3:BUD14:RAS1:CAS4:C7_03480W_A:RHO1:CR_06740W_A:TUB1:CR_09520C_A
31106	septin ring organization	9 out of 356 genes, 2.5%	27 out of 6473 background genes, 0.4%	0.00833	0.00%	CDC12:CDC3:CLA4:GIN4:CDC11:INT1:RSR1:RHO1:CDC10
55082	intracellular chemical homeostasis	25 out of 356 genes, 7.0%	176 out of 6473 background genes, 2.7%	0.01012	0.00%	HMX1:CTR2:FTR1:FRP3:YVC1:PHO13:RAS1:IRO1:CDR1:YCK2:FRP1:RBT5:SOD1:C4_04760C_A:CSR1:C4_06770W_A:GPR1:ZCF20:CCC2:FET34:PTK2:CSA1:FRP2:GEF2:ALS3
32185	septin cytoskeleton organization	9 out of 356 genes, 2.5%	29 out of 6473 background genes, 0.4%	0.01614	0.00%	CDC12:CDC3:CLA4:GIN4:CDC11:INT1:RSR1:RHO1:CDC10

6873	intracellular monoatomic ion homeostasis	21 out of 356 genes, 5.9%	137 out of 6473 background genes, 2.1%	0.0163	0.00%	HMX1:CTR2:FTR1:YVC1:PHO13:IRO1:CDR1:FRP1:RBT5:SOD1:C4_04760C_A:CSR1:C4_06770W_A:ZCF20:CCC2:FET34:PTK2:CSA1:FRP2:GEF2:ALS3
44407	single-species biofilm formation in or on host organism	6 out of 356 genes, 1.7%	12 out of 6473 background genes, 0.2%	0.01911	0.00%	HYR1:EAP1:HWP1:PHR1:SUN41:BCR1

51179	localization	157 out of 356 genes, 44.1%	2189 out of 6473 background genes, 33.8%	0.0224	0.00%	HMX1:TUB2:HGC1:C1_00830W_A:TRS20:C1_01620C_A:HOL4:HGT1:EXG2:YPT7:TEM1:C1_05980W_A:CPH1:PLB5:CTR2:IPT1:CLA4:SFH5:C1_11250W_A:KIP4:MCD4:FEN1:CHS3:HYR1:KAR9:MYO2:FTR1:SOD5:C2_01340W_A:SRD1:CHK1:DCK1:RHO3:FRP3:LSP1:LTE1:PST1:C2_07220W_A:YVC1:RHO2:C2_08100W_A:IRF1:DDR48:EAP1:YEA4:SSO2:RAS1:KIP2:NGT1:ATO1:IRO1:ARF3:UTR2:C3_01940C_A:GFA1:C3_02290W_A:CDR11:VAM3:C3_04350C_A:RAX1:MEA1:CDR1:YCK2:C3_05990C_A:AHR1:EVP1:FRP1:PGA7:RBT5:SPC2:C4_01220C_A:OPT6:RFX2:ECE1:RBT1:HWP1:AXL2:C4_04250W_A:FAV1:CSR1:C4_04980W_A:C4_05080C_A:C4_05250W_A:ARO9:C4_05980C_A:C4_06770W_A:CDC11:C5_00230C_A:GIT2:AUR1:GPR1:C5_01440C_A:ZCF20:MYO1:ECM331:INT1:PUT1:CCC2:C5_03170C_A:C5_03930C_A:C5_04260W_A:MAL31:FET34:CAN3:C6_01620W_A:PTK2:CTF5:C6_02160W_A:SAP6:SAP5:SHE3:ARG3:C6_03260W_A:SAP4:CSA1:FRP2:C7_00230W_A:DFI1:C7_00630C_A:IQG1:DPP3:PGA28:SNG3:C7_03480W_A:C7_03500W_A:ARG1:CR_01280C_A:RSR1:GEF2:IDP2:RHO1:ATG13:WOR3:RBR1:CDC10:DIT2:CR_05540C_A:MCT1:BCR1:SEO1:CR_06740W_A:CR_06790C_A:ALS3:CR_07220C_A:YHB1:YHB5:TPM2:PGA13:CHS2:TUB1:SUC1:CR_09520C_A:CR_09570W_A:YKE2:PLB3:PTP3:CR_10610C_A

22603	regulation of anatomical structure morphogenesis	11 out of 356 genes, 3.1%	45 out of 6473 background genes, 0.7%	0.02306	0.00%	TUB2:MYO2:RHO3:BUD14:RAS1:CAS4:C7_03480W_A:RHO1:CR_06740W_A:TUB1:CR_09520C_A
30003	intracellular monoatomic cation homeostasis	20 out of 356 genes, 5.6%	130 out of 6473 background genes, 2.0%	0.02462	0.00%	HMX1:CTR2:FTR1:YVC1:PHO13:IRO1:CDR1:FRP1:RBT5:SOD1:C4_04760C_A:CSR1:C4_06770W_A:ZCF20:CCC2:FET34:CSA1:FRP2:GEF2:ALS3
10570	regulation of filamentous growth	30 out of 356 genes, 8.4%	244 out of 6473 background genes, 3.8%	0.02465	0.00%	HGC1:MSB1:C1_05980W_A:UME6:CPH1:IPT1:CLA4:GIN4:CDC6:MYO2:MSB2:CHK1:C2_05580W_A:IRF1:RAS1:C3_01800C_A:YCK2:AHR1:ZFU2:ZCF27:RFX2:CLN3:GPR1:CCN1:RSR1:CR_02210W_A:BCR1:CLG1:DEF1:PTP3

30473	nuclear migration along microtubule	7 out of 356 genes, 2.0%	18 out of 6473 background genes, 0.3%	0.02786	0.00%	TUB2:C1_11250W_A:KAR9:KIP2:MYO1:CR_07220C_A:TUB1
72384	organelle transport along microtubule	7 out of 356 genes, 2.0%	18 out of 6473 background genes, 0.3%	0.02786	0.00%	TUB2:C1_11250W_A:KAR9:KIP2:MYO1:CR_07220C_A:TUB1
40008	regulation of growth	31 out of 356 genes, 8.7%	258 out of 6473 background genes, 4.0%	0.02843	0.00%	HGC1:MSB1:C1_05980W_A:UME6:CPH1:IPT1:CLA4:GIN4:CDC6:MYO2:MSB2:CHK1:C2_05580W_A:IRF1:RAS1:C3_01800C_A:YCK2:AHR1:ZFU2:ZCF27:RFX2:CLN3:GPR1:CCN1:C7_03480W_A:RSR1:CR_02210W_A:BCR1:CLG1:DEF1:PTP3
50801	monoatomic ion homeostasis	21 out of 356 genes, 5.9%	142 out of 6473 background genes, 2.2%	0.0285	0.00%	HMX1:CTR2:FTR1:YVC1:PHO13:IRO1:CDR1:FRP1:RBT5:SOD1:C4_04760C_A:CSR1:C4_06770W_A:ZCF20:CCC2:FET34:PTK2:CSA1:FRP2:GEF2:ALS3
7120	axial cellular bud site selection	6 out of 356 genes, 1.7%	13 out of 6473 background genes, 0.2%	0.03386	0.00%	RAX2:RAX1:AXL2:INT1:C5_03170C_A:RSR1

7114	cell budding	11 out of 356 genes, 3.1%	47 out of 6473 background genes, 0.7%	0.03582	0.00%	GIN4:RAX2:MYO2:CAS4:RAX1:AXL2:CLN3:INT1:C5_03170C_A:RSR1:RHO1
55080	monoatomic cation homeostasis	20 out of 356 genes, 5.6%	134 out of 6473 background genes, 2.1%	0.03867	0.00%	HMX1:CTR2:FTR1:YVC1:PHO13:IRO1:CDR1:FRP1:RBT5:SOD1:C4_04760C_A:CSR1:C4_06770W_A:ZCF20:CCC2:FET34:CSA1:FRP2:GEF2:ALS3
33215	reductive iron assimilation	4 out of 356 genes, 1.1%	5 out of 6473 background genes, 0.1%	0.04452	0.00%	FTR1:FRP1:FET34:ALS3
2E+06	mitotic cell cycle process	32 out of 356 genes, 9.0%	278 out of 6473 background genes, 4.3%	0.04978	0.00%	TUB2:TEM1:CDC12:CDC3:C1_11250W_A:GIN4:CDC6:RAX2:KAR9:MYO2:RAX1:MEA1:AXL2:CDC11:CLN3:MYO1:CCN1:INT1:C5_03170C_A:CAN3:PTK2:CTF5:BEM2:IQG1:CHS1:RSR1:RHO1:CDC10:CR_06740W_A:TPM2:TUB1:CR_09520C_A
48878	chemical homeostasis	25 out of 356 genes, 7.0%	195 out of 6473 background genes, 3.0%	0.05989	0.03%	HMX1:CTR2:FTR1:FRP3:YVC1:PHO13:RAS1:IRO1:CDR1:YCK2:FRP1:RBT5:SOD1:C4_04760C_A:CSR1:C4_06770W_A:GPR1:ZCF20:CCC2:FET34:PTK2:CSA1:FRP2:GEF2:ALS3

71852	fungal-type cell wall organization or biogenesis	27 out of 356 genes, 7.6%	219 out of 6473 background genes, 3.4%	0.06065	0.03%	ECM33:CPH1:FEN1:CHS3:MSB2:CHK1:DCK1:YEA4:CAS4:UTR2:GFA1:EVP1:RLM1:DPM3:SOD1:RBT1:HWP1:PGA31:PHR1:SUN41:CHS1:LMO1:RHO1:CDC10:DIT2:PGA13:CHS2
71963	establishment or maintenance of cell polarity regulating cell shape	7 out of 356 genes, 2.0%	20 out of 6473 background genes, 0.3%	0.06165	0.03%	TUB2:MYO2:RHO3:RAS1:CR_06740W_A:TUB1:CR_09520C_A
31505	fungal-type cell wall organization	22 out of 356 genes, 6.2%	161 out of 6473 background genes, 2.5%	0.06383	0.03%	ECM33:CPH1:CHS3:MSB2:DCK1:CAS4:UTR2:EVP1:RLM1:SOD1:RBT1:HWP1:PGA31:PHR1:SUN41:CHS1:LMO1:RHO1:CDC10:DIT2:PGA13:CHS2
45229	external encapsulating structure organization	22 out of 356 genes, 6.2%	162 out of 6473 background genes, 2.5%	0.0702	0.03%	ECM33:CPH1:CHS3:MSB2:DCK1:CAS4:UTR2:EVP1:RLM1:SOD1:RBT1:HWP1:PGA31:PHR1:SUN41:CHS1:LMO1:RHO1:CDC10:DIT2:PGA13:CHS2
71555	cell wall organization	22 out of 356 genes, 6.2%	162 out of 6473 background genes, 2.5%	0.0702	0.03%	ECM33:CPH1:CHS3:MSB2:DCK1:CAS4:UTR2:EVP1:RLM1:SOD1:RBT1:HWP1:PGA31:PHR1:SUN41:CHS1:LMO1:RHO1:CDC10:DIT2:PGA13:CHS2

30010	establishment of cell polarity	12 out of 356 genes, 3.4%	59 out of 6473 background genes, 0.9%	0.07028	0.03%	MSB1:RAX2:MYO2:MSB2:RHO3:RAX1:MEA1:AXL2:INT1:C5_03170C_A:C7_03480W_A:RSR1
278	mitotic cell cycle	32 out of 356 genes, 9.0%	283 out of 6473 background genes, 4.4%	0.07063	0.03%	TUB2:TEM1:CDC12:CDC3:C1_11250W_A:GIN4:CDC6:RAX2:KAR9:MYO2:RAX1:MEA1:AXL2:CDC11:CLN3:MYO1:CCN1:INT1:C5_03170C_A:CAN3:PTK2:CTF5:BEM2:IQG1:CHS1:RSR1:RHO1:CDC10:CR_06740W_A:TPM2:TUB1:CR_09520C_A

6810	transport	144 out of 356 genes, 40.4%	2011 out of 6473 background genes, 31.1%	0.07508	0.03%	HMX1:TUB2:HGC1:C1_00830W_A:TRS20:C1_01620C_A:HOL4:HGT1:EXG2:YPT7:TEM1:C1_05980W_A:CPH1:PLB5:CTR2:CLA4:SFH5:C1_11250W_A:KIP4:MCD4:FEN1:HYR1:KAR9:MYO2:FTR1:SOD5:C2_01340W_A:SRD1:CHK1:DCK1:RHO3:FRP3:LSP1:LTE1:PST1:C2_07220W_A:YVC1:RHO2:IRF1:DDR48:EAP1:YEA4:SSO2:KIP2:NGT1:ATO1:IRO1:ARF3:UTR2:C3_01940C_A:GFA1:C3_02290W_A:CDR11:VAM3:C3_04350C_A:RAX1:CDR1:YCK2:C3_05990C_A:AHR1:EVP1:FRP1:PGA7:RBT5:SPC2:C4_01220C_A:OPT6:RFX2:ECE1:RBT1:HWP1:AXL2:C4_04250W_A:FAV1:CSR1:C4_04980W_A:C4_05080C_A:C4_05250W_A:ARO9:C4_05980C_A:C4_06770W_A:CDC11:C5_00230C_A:GIT2:AUR1:GPR1:C5_01440C_A:ZCF20:MYO1:ECM331:PUT1:CCC2:C5_03930C_A:C5_04260W_A:MAL31:FET34:CAN3:C6_01620W_A:PTK2:C6_02160W_A:SAP6:SAP5:SHE3:ARG3:C6_03260W_A:SAP4:CSA1:FRP2:C7_00230W_A:DFI1:C7_00630C_A:DPP3:PGA28:SNG3:C7_03500W_A:ARG1:CR_01280C_A:RSR1:GEF2:IDP2:RHO1:ATG13:WOR3:RBR1:CDC10:DIT2:CR_05540C_A:MCT1:SEO1:CR_06740W_A:CR_06790C_A:ALS3:CR_07220C_A:YHB1:YHB5:PGA13:CHS2:TUB1:SUC1:CR_09520C_A:CR_09570W_A:YKE2:PLB3:CR_10610C_A
