# Supplementary material for: Reprogramming in Candida albicans Gene Expression Network under Butanol Stress Abrogates Hyphal Development
Source: Int J Mol Sci. 2023 Dec 7;24(24):17227. doi: 10.3390/ijms242417227 (PMC10743114; doi:10.3390/ijms242417227)
Supplement: Supplementary file 1 [file ijms-24-17227-s001.zip › Table S4.pdf]

Table-S4: Genes with description containing term "stress" in the Serum vs. Serum with Butanol				
Gene ID	20 min	45 min	120 min	Common name
**Upregulated Genes**				
**C1_03870C_A**	**10.1**			
**C1_08070W_A**		**9.20**	**8.87**	CDR4
**C4_04100C_A**	**8.6**	**21.24**		ECM1
**C6_01180C_A**	**8.16**	**9.88**	**11.31**	EBP1
**C2_04080W_A**	**7.18**	**8.59**		
**C1_07990C_A**		**7.10**	**5.04**	
**CR_08340W_A**	**6.75**	**4.93**		CYS3
**C4_06710W_A**	**6.62**	**5.54**	**2.97**	
**C1_04330W_A**	**2.38**	**6.15**		RPN4
**C1_06810W_A**		**5.77**	**11.65**	CAT1
**C2_10240W_A**	**5.5**	**7.70**	**3.89**	GPD1
**C2_06170C_A**	**4.91**	**2.25**		ECM17
**CR_03710C_A**		**4.67**	**2.38**	
**C7_01670W_A**		**4.30**		
**C1_05840W_A**		**4.13**	**2.06**	PRN1
**CR_08890C_A**	**4.11**	**4.81**	**8.73**	ASR2
**C3_01300C_A**	**3.67**			NCE103
**C3_07100C_A**		**3.41**	**2.1**	
**C6_00290W_A**		**3.67**	**4.08**	
**C3_00320W_A**	**3.28**		**3.56**	RHR2
**C6_01870C_A**		**3.90**		
**C1_11700C_A**		**3.84**	**2.54**	MRF1
**C1_09170W_A**	**3.25**	**3.04**		CTA8
**C4_00960W_A**	**3.16**			PTC8
**C1_04740W_A**		**3.12**	**2.84**	LPG20
**CR_09670C_A**		**3.07**	**2.58**	

**C2_07070W_A**		**3.23**	**2.56**	
**C4_06780C_A**		**3.03**	**2.19**	OYE32
**C7_04090C_A**	**3.03**	**6.42**	**3.42**	
**C2_02880C_A**		**2.38**	**2.37**	EBP7
**C1_00840C_A**	**2.98**			NRM1
**C2_07130C_A**	**2.94**	**4.31**	**3.34**	RCK2
**CR_01550C_A**	**2.85**			
**C1_00500C_A**		**2.73**		GLO1
**C1_09010W_A**	**2.76**			NAR1
**C6_03750C_A**		**2.35**		SBA1
**CR_10270C_A**		**2.71**		AHA1
**C1_11910W_A**	**2.71**	**3.66**		
**C7_04230W_A**	**2.45**	**8.44**	**11.72**	NRG1
**C1_04330W_A**	**2.38**			RPN4
**C3_05930W_A**	**2.35**	**3.78**	**2.02**	CTA4
**C7_01940C_A**		**2.35**		
**C6_01160W_A**		**2.32**		RCN1
**CR_06490C_A**	**2.18**			HSP60
**C1_10220C_A**		**2.29**		TPK1
**C1_13960W_A**	**2.12**			YBP1
**C3_06310C_A**	**2.08**			ISW2
**C3_06620W_A**	**2.05**			FUN31
**C6_01070C_A**	**2.05**			CIP1
**C1_01520C_A**		**2.21**	**2.81**	SOD2
**C3_06270C_A**	**2.04**			
**CR_08370W_A**	**2.04**	**2.18**		GSH2
**C1_03300C_A**		**2.14**		MAS1
**C3_06340W_A**		**2.13**		HNT2
**CR_00980C_A**		**2.13**		
**C1_01130W_A**		**2.12**		

**C6_02480W_A**		**2.12**		
**C1_05540C_A**		**2.10**		
**C7_02330W_A**		**2.09**		YCF1
**CR_02510W_A**		**2.09**		
**C1_11660W_A**		**2.06**		GAD1
**C2_09880C_A**		**2.06**		
**CR_08250C_A**		**2.06**		HSP104
**C5_01520C_A**		**2.02**	**2.14**	GLR1
**C5_02080C_A**			**5.54**	HSP12
**C7_00110W_A**			**3.82**	SOD3
**C2_07630C_A**			**2.66**	
**C4_02410C_A**			**2.48**	AHP1
**CR_03470W_A**			**2.44**	
**CR_03280W_A**			**2.26**	IFR2
**C1_04320W_A**			**2.22**	GPM2
**C1_06780W_A**			**2.03**	NPR1
**C1_09690W_A**	**-3.15**		**2.63**	MLS1
**C2_06470W_A**	**-2.29**	**3.83**	**2.63**	RTA2
**C3_01200W_A**	**6.11**			UTP20
**C1_04040C_A**	**5.58**	**13.39**	**2.48**	
**CR_05520W_A**	**5.44**	**11.85**		NOC2
**C1_10970W_A**	**5.42**	**10.13**	**2.58**	
**C4_05010W_A**	**5.4**	**21.10**	**2.5**	
**CR_05630W_A**	**5.39**	**9.75**	**2.27**	DBP8
**CR_09800C_A**	**5.34**	**6.20**	**2.12**	
**C1_03230C_A**	**5.25**	**8.97**		ARX1
**C2_05090W_A**	**5.03**	**15.30**	**2.3**	MAK5
**C1_04390C_A**	**4.95**	**9.46**		NOP4
**C7_04190C_A**	**4.81**	**11.62**	**2.62**	UTP18
**CR_03650W_A**	**4.59**	**7.24**	**2.15**	SGD1

C5_02550C_A	4.36	8.79		UTP13
CR_02530W_A		4.35		DBP2
C4_00540C_A	4.32	3.59		NMD5
C4_02790C_A	4.05		2.54	SAS10
C2_07450C_A	3.99	5.16	2.76	RCL1
C2_08000C_A	3.92	5.66	2.44	KRE30
CR_06720W_A	3.88	8.75	2.43	NMD3
CR_10550W_A	3.88	8.31	2.37	DRS1
C1_08870C_A	3.87	4.66	2.37	JIP5
C4_01450W_A	3.84	5.60		PWP1
C4_02850W_A	3.22	5.91		
C2_00070C_A	3.17	6.78		MPP10
C1_00640C_A	3.06			RPO41
C2_06390C_A		3.31		IMH3
C2_10740C_A	3.06	5.75		
CR_00800C_A	2.97	3.66		PWP2
CR_00680W_A	2.85	6.33	2.07	BUD22
C1_00990C_A	2.81	3.20		NMA111
C1_07910C_A	2.24			MRPL3
C4_07130W_A	2.07			
C2_07890W_A	2.04	2.24		PRS5
Down regulated genes				
C6_02100W_A	-428.43		-7.66	
C6_02210W_A	-11.91	-4.35	-4.81	
C2_09220W_A	-5.71		-2.3	DDR48
C1_11270W_A	-4.62			
C7_00350C_A	-3.66			
C5_04980W_A	-3.62			
C1_11850W_A	-3.09			
CR_00780C_A	-2.52	-2.18		GSY1

**C4_03430W_A**	**-2.44**			MOH1
**C7_03440W_A**		**-3.37**	**-2.49**	SNG4
**C2_01780W_A**		**-3.14**	**-2.69**	MSB2
**C2_03790C_A**		**-2.70**	**-2.31**	ASR3
**C2_02860W_A**		**-2.43**		SUR2
**C1_10510W_A**		**-2.01**		
**C2_04010C_A**	**-7.44**			HSP21
**C2_00680C_A**		**-51.19**	**-52.42**	SOD5
