# Supplementary material for: Reprogramming in Candida albicans Gene Expression Network under Butanol Stress Abrogates Hyphal Development
Source: Int J Mol Sci. 2023 Dec 7;24(24):17227. doi: 10.3390/ijms242417227 (PMC10743114; doi:10.3390/ijms242417227)
Supplement: Supplementary file 1 [file ijms-24-17227-s001.zip › Table S5.pdf]

Table S5:Primers from selected genes of *Candida albicans* used in q Real Time PCR

S.No.	Gene Name	Primer Sequence(5'->3')	Primer Length
01.	ACT1	Forward primer ACTCTGTCTGGATTGGTGGT<br>Reverse primer TGTGGTGAACAATGGATGGAC	20<br>21
02.	ALS3	Forward primer TCGTCCTCATTACACCAAC<br>Reverse primer ACAGAAACCCAAGAACAAC	19<br>19
03.	ECE1	Forward primer CTCAAGCTGCCATCATCCA<br>Reverse primer TGCAGGAACAGTAGGTGCT	19<br>19
04.	HWP1	Forward Primer CTGCTCAACTTATTGCTATC<br>Reverse primer CATCACATGGTTCTTGATAG	20<br>20
05.	RME1	Forward primer CACCGGAATCTCCACACA<br>Reverse primer AGGTAGCAGAGCCCAAAG	18<br>18
06.	SAP5	Forward primer GGTGACGGTTCTTACGCT<br>Reverse primer GTCGTAATCAAACTCGGTAG	18<br>20
07.	UME6	Forward primer AGTGGTTCATCAATACCAGA<br>Reverse primer ACAATTTTGGAGCAATCACC	20<br>20
08.	WOR3	Forward primer AGCCCAAGGGAGATGTGTTC<br>Reverse primer CCTCGTGCAGGCTTTTCTTG	20<br>20
09.	YWP1	Forward primer CCAGGTACTTTGACACCGGAA<br>Reverse primer GGCAGCTTTACCAGAACCTGA	21<br>21
10.	C4_05010W_A	Forward primer GTTTGGGACACCACAACAAGA<br>Reverse primer TGAACCAGACCATTTAACACAA	21<br>22
11.	C5_03440W_A	Forward primer CAGCACCTCAAAACGATAAA<br>Reverse primer CAATGGAAGTTGCTTTTCAGT	20<br>21
