# Supplementary material for: Reprogramming in Candida albicans Gene Expression Network under Butanol Stress Abrogates Hyphal Development
Source: Int J Mol Sci. 2023 Dec 7;24(24):17227. doi: 10.3390/ijms242417227 (PMC10743114; doi:10.3390/ijms242417227)
Supplement: Supplementary file 1 [file ijms-24-17227-s001.zip › Table-S1.pdf]

Table S1: Differential gene expression when the serum and serum with butanol data were compared to 0 min gene expression data.

S20 vs Control

Name	Fold change	P-value	GeneID
ECE1	7958.741	3.01E-07	3646814
HWP1	3569.387	3.48E-18	3645372
C1_13100W_A	1651.271	1.6E-05	30515073
HGT12	1149.695	5.4E-05	3638048
CDC11	904.3553	6.89E-05	3647888
CRH11	522.4321	2.49E-11	3637905
MLC1	377.32	0.000548	30515387
DPM2	317.9686	0.000723	30515325
DPM1	297.1951	5.36E-10	3640700
C3_06670C_A	285.6126	0.000904	3642254
C6_02210W_A	278.3368	0.000963	3645864
RBT1	270.0715	6.43E-18	3645377
CDC10	255.1713	1.42E-09	3640947
C7_02280W_A	247.4458	0.001363	3636999
VMA10	243.1235	0.001297	3645215
HYR1	242.5002	1.33E-08	3636226
C4_05390W_A	237.0342	0.001371	3646951
C3_06750W_A	219.3537	0.001816	30515219
PXP2	219.3371	0.002496	3636558
UME6	217.4576	3.52E-09	3647522
PGA31	216.6754	0.001657	3641226
COG4	204.6403	0.001884	3645571
MCD4	202.9417	0.00191	3642036
C3_02530W_A	194.096	0.002059	30515189
C1_04180W_A	190.5388	0.00213	3644716
C5_03730W_A	185.6719	0.002261	3647238
CR_05310W_A	176.3992	0.002548	3648125
C6_02140W_A	153.7904	0.003248	3645886
ALI1	142.764	0.003987	3636635
FAD1	139.3284	0.003856	3634930
SAM51	134.2017	0.004167	3641582
PDI1	130.785	1.37E-26	3641367
MAL31	128.3534	2.28E-06	3636420
ECM331	125.8235	0.004567	3637731
SHE3	125.6725	7.01E-08	3639277
C4_02460W_A	125.5132	0.004676	3637844
ALS3	123.0369	1.58E-34	3647965
PMI1	121.8039	3.7E-11	3643795
DDR48	117.7729	2.68E-23	3644061
OST1	110.6985	1.89E-07	3643657

LDG3	108.009	0.006972	3637001
PMT2	105.905	3.21E-23	3638538
LYS22	104.883	2.03E-16	3645466
CR_06380C_A	101.6123	0.006929	3648139
DPP1	101.2769	0.00708	3644537
C1_03600W_A	100.4187	4.29E-07	3636880
FGR6-10	100.1884	0.007132	3641770
MRV8	98.45328	0.00739	3636450
LYS9	95.92124	4.18E-18	3642279
ERV46	95.47855	1.48E-10	3644129
C6_00400C_A	89.27913	0.008923	3646087
C4_04380C_A	88.59897	6.24E-07	30515266
C3_02620C_A	86.29842	0.00965	3638326
DOG1	83.70895	8.52E-07	3641597
C4_00250W_A	82.379	0.016774	3645035
C7_00240W_A	81.16817	0.010739	3637980
C6_02100W_A	81.13577	4.82E-18	3647554
C2_10010C_A	78.93747	0.010855	3643962
C3_07840C_A	77.6665	0.010909	3647465
PHR1	76.81544	1.36E-27	3641108
ERV29	76.43994	2.11E-16	3635672
SEC21	74.8635	8.45E-20	3644786
C1_10560C_A	74.64634	0.011727	3647196
THR1	74.28048	1.59E-06	3641572
PMM1	71.07043	2.47E-13	3636852
FRP2	70.09336	8.55E-13	3638008
AGM1	68.3892	2.9E-06	3636138
YNK1	67.09687	3.96E-17	3637746
SEC23	66.66096	1.39E-21	3641818
SOD5	65.97764	3.25E-11	3638886
ERV25	65.48139	2.76E-13	3642243
CR_01490C_A	65.40035	1.36E-11	30515376
CR_09330C_A	65.31349	7.66E-20	3641899
PMT4	64.11715	1.01E-19	3644096
CDC28	63.1867	5.36E-06	3644838
TUB2	60.88163	8.3E-11	3639329
HHT21	59.96082	6.24E-06	3644707
CWH43	59.89497	4.66E-11	3640389
CAR1	59.84384	7.8E-19	3636503
C6_02180W_A	59.81555	0.017052	3645861
HOM6	59.42902	6.09E-06	3636815
LSC1	58.7424	1.27E-17	3640199
ERG28	58.70052	0.017532	3638833
C6_01880W_A	58.6654	0.017604	3641599
C1_02760W_A	57.81004	1.33E-12	3636870
EMP24	56.48249	2.69E-08	3647247
C2_05720C_A	55.85911	8.22E-11	3645574

DUR3	55.44931	0.021498	3644760
CR_04650W_A	54.35216	0.021776	30515396
CR_00280C_A	52.45718	0.021276	3639719
CR_04490C_A	52.17075	0.02127	3641013
C6_00710W_A	52.16029	0.021305	3647073
TEM1	51.64514	0.021629	3636923
SEC22	51.61951	1.58E-05	30515391
C1_02100W_A	51.61404	0.021683	3644578
ARF3	51.28244	1.33E-05	3636628
C3_06730W_A	50.45784	0.022713	3638504
RET2	50.15279	6.92E-16	3637714
C7_02310C_A	48.71631	0.024178	3636936
C4_02600C_A	48.27653	0.024882	3637912
PTP3	48.22755	2.52E-23	3638940
HRT2	48.20499	1.66E-05	3635709
FGR6-3	48.01583	0.024434	3647954
C1_11950W_A	47.98688	0.024488	3642045
C5_03000C_A	47.4661	0.02485	3637818
C7_01100C_A	47.44126	0.025406	3637948
TUB1	47.32587	1.99E-15	3641930
ACP12	46.53032	2.06E-05	3637361
C2_05510C_A	46.28917	0.025874	3643420
ACO2	45.8809	1.14E-09	3646708
C1_07490C_A	45.85821	0.027986	3646666
HET1	45.18248	4.57E-19	3647897
GFA1	45.15758	1.55E-22	3636557
ERP5	44.86431	1.76E-11	30515046
TOS1	43.86055	3.45E-21	3636566
SNZ1	43.5031	8E-10	3636803
KAR3	42.69521	0.029395	3644078
C1_07920W_A	42.68658	0.029124	30515026
SSR1	42.4979	1.96E-15	3638055
TNA1	42.34661	0.032751	3637827
LYS12	41.92106	6.35E-18	3640134
DPM3	41.85542	1.07E-07	30515245
CEK1	41.68451	2.19E-09	3642789
CR_00990W_A	41.47561	3.8E-05	3640425
CR_05130C_A	41.42032	3.47E-05	3646401
CSR1	41.07076	2.59E-09	3641162
CR_09230C_A	41.02465	3.2E-09	3641918
PMT1	40.90119	4.79E-20	3641393
ATP18	40.4522	4.7E-05	30515084
C7_00680W_A	40.13458	0.032274	3637967
SEC13	39.95099	1.09E-15	3638425
SEF2	39.32218	1.44E-06	3647052
ATP19	38.92754	5.3E-05	30515061
C2_05290C_A	38.73497	9.05E-09	3643472

ATP1	38.52763	1.97E-21	3646134
SAR1	38.50827	1.45E-14	3645865
C7_03650W_A	37.97763	0.033919	3646224
PGA63	36.90428	2.67E-19	3639524
C5_05020C_A	36.65607	5E-11	3636486
DAG7	36.61615	5.21E-10	3635607
C5_01230C_A	36.54719	7.6E-13	3642618
C2_09590C_A	36.27805	0.036843	3643800
SRB1	36.09237	6.27E-20	3647454
C6_02190C_A	35.86242	4.03E-07	3645862
GDE1	35.71588	3.5E-20	3636505
C1_07260C_A	35.71094	7.89E-05	3644456
MNT1	35.29577	6.54E-20	3636585
LYS1	35.2358	1.02E-14	6335429
GUP1	34.88437	0.0399	3636171
SEC12	34.82935	1.43E-15	3641639
ARC18	34.42805	0.039704	3644056
CFL11	34.37996	1E-05	3646622
C3_07130W_A	34.35286	0.04006	3638440
ENA21	34.15509	1.03E-21	3641392
ACT1	33.98124	6.39E-21	3636195
CR_05800C_A	33.63931	8.91E-05	3646706
C1_06700W_A	33.52773	9.21E-05	3639511
C1_05100W_A	33.25602	0.041623	3647669
CR_10130W_A	33.22728	0.04214	3638970
C4_06140C_A	33.04314	9.62E-05	3642821
MTS1	32.54563	1.08E-18	3634873
RFA2	32.29695	0.000265	3643506
C5_03930C_A	32.24598	2.91E-08	3647447
C4_01140C_A	32.05701	0.043832	3635657
HIS1	31.84594	6.51E-12	3636501
ILV5	31.70479	7.1E-19	3644012
C1_06340W_A	31.60927	2.55E-08	3639499
C6_01460C_A	31.55086	9.58E-12	3641623
C7_00670C_A	31.45914	6.29E-12	3638024
CYT1	31.39317	3.4E-13	3643441
LYS4	31.08726	4.26E-11	3641180
AAP1	31.05959	1.57E-08	3647397
COF1	30.72591	1.99E-16	30515293
CDC12	30.66979	1.83E-15	3636809
C1_03150C_A	30.65878	0.049867	30514976
CR_04730W_A	30.4504	2.11E-08	3647305
OSH3	30.41898	6.05E-13	3645368
C1_03940W_A	30.31639	5.23E-06	3646415
CEX1	30.2589	1.27E-08	3644913
CR_09090C_A	30.05499	8.33E-11	3641927
GIG1	29.84201	0.049114	3644712

AMO2	29.25874	7.02E-08	3648021
C1_06050C_A	28.98462	0.000197	30515003
C5_00080C_A	28.77964	9.72E-14	3647886
CMP1	28.66111	4.72E-11	3639328
DOA1	28.59486	1.15E-14	3634871
CR_06170W_A	28.13533	0.00023	3644865
QCR2	28.01145	7.26E-14	3646352
C4_04980W_A	27.84593	3.7E-08	3641167
C1_01210W_A	27.83976	1.72E-09	3640209
BGL2	27.02286	1.64E-17	3635691
CR_03470W_A	27.00187	0.000251	3643198
C1_04780C_A	26.64033	1.3E-09	3644774
ERG6	26.63832	1.35E-09	3636655
POL30	26.52104	0.000791	3635583
MYO2	26.45777	5.76E-16	3636140
KAR2	26.33983	7.89E-18	3638830
BFR1	26.29979	1.84E-17	3635579
EXG2	26.24369	4.46E-07	3636816
CR_03000C_A	26.12117	0.000367	3642597
CHS2	25.96326	8.6E-19	3641893
SAC1	25.8608	1.55E-09	3634864
JEM1	25.7822	5.28E-06	3643876
ERG11	25.34444	1.21E-09	3641571
C2_10200W_A	25.31222	0.000416	3643928
MAL2	25.02093	4.81E-06	3638946
ERG26	24.84065	2.14E-07	3642803
C1_04430C_A	24.52721	0.000459	30514986
TSA1	24.20472	3.17E-16	3642269
C4_00710W_A	24.15143	6.47E-06	3645750
CLC1	24.03938	2.22E-13	3635662
C1_11860W_A	23.93868	5.81E-18	3642009
C5_02920W_A	23.93345	0.000597	3637751
SEC20	23.89999	7.52E-06	3639206
C2_05130W_A	23.68147	5.68E-09	3643458
STT3	23.55962	2.86E-14	3635743
ATO1	23.38155	1.09E-05	3647761
ATP3	22.85708	2.58E-18	3640387
MSB1	22.74738	1E-08	3648218
C2_07540W_A	22.63995	0.00082	3645222
LSC2	22.61663	1.08E-16	3646618
GDH3	22.48015	3.06E-13	3648086
LHS1	22.41795	2.69E-15	3643032
CBR1	22.31518	5.42E-10	3646961
SEC18	22.18444	3.19E-15	3636208
COX5	22.12744	4.73E-12	3634768
CR_03520C_A	21.90162	4.38E-10	3643203
CR_03330W_A	21.87483	0.000721	3645095

C1_04010C_A	21.87483	0.000723	3646426
TSA1B	21.77444	6.04E-15	30515214
QCR8	21.62783	5.7E-10	30515112
LAP41	21.56629	5.95E-12	3636653
CWH41	21.12078	9.11E-10	3646533
C1_06920C_A	20.99517	0.00083	3639525
CR_10530W_A	20.95872	0.000945	3639042
TVP18	20.94709	1.33E-06	3634844
ECM33	20.90905	3.32E-16	30514977
RBT4	20.63077	1.38E-09	3639534
TRP4	20.62597	2.19E-05	3642904
SPC3	20.57005	1.49E-07	3636121
MNS1	20.39878	4.11E-12	3644692
MDH1-1	20.3256	2.55E-15	3635636
C7_01710W_A	20.3115	3.91E-12	3637012
C6_04100W_A	20.03595	2.36E-12	3647836
C1_02500W_A	20.00657	0.001101	3636795
SEP7	19.98619	5.6E-08	3644596
HAT1	19.97685	2.38E-05	3644770
C2_07140W_A	19.9247	8.11E-10	3643508
POL1	19.78593	3.47E-08	3635144
SPC2	19.7489	2.93E-05	3645769
C3_00520W_A	19.72695	2.87E-05	3640513
CR_00130C_A	19.72235	1.11E-09	3639709
RGD1	19.68014	8.13E-07	3639868
ADK1	19.64284	7.69E-14	3644533
C2_03970W_A	19.41043	0.001318	3637419
C4_04440W_A	19.30303	1.6E-11	3641177
PGA10	19.28266	3.68E-05	3645009
FAV2	19.24943	0.001464	3647236
PYC2	19.13338	9.39E-09	3637323
SEC62	19.1106	9.34E-12	3636860
APL4	18.98066	1.91E-11	3642565
VMA2	18.97098	4.76E-16	3646710
C2_07650C_A	18.94856	3.34E-05	3642342
CYP1	18.87452	2.34E-17	3636978
ATP2	18.63229	4.52E-15	3645039
SSA2	18.60789	4.15E-14	3644711
APL2	18.50979	2.8E-11	3638343
C1_07590C_A	18.2851	1.82E-07	3646673
FRE7	18.06608	2.7E-15	3646024
C2_07010W_A	18.04905	1.73E-09	3643535
ASH2	18.04604	0.001603	3636445
MCM2	18.03735	0.00152	3643197
HTA2	18.03022	5.09E-07	3644715
PGA6	17.82562	0.001884	3634774
CR_05500C_A	17.73072	0.001557	3647598

DUR1,2	17.67577	9.88E-10	3644771
C7_04000W_A	17.65607	7.73E-09	3643114
PGA54	17.655	3.76E-10	3637929
C1_06480C_A	17.61812	1.14E-10	30515008
SEC61	17.57487	6.02E-15	3647462
C5_02210W_A	17.55785	1.42E-08	3637759
C1_14320C_A	17.52059	0.001704	3643316
C1_01190C_A	17.39343	7.37E-05	3640207
FRE30	17.15163	1.22E-15	3646025
C6_03130W_A	17.05031	6.27E-06	30515341
CR_03950W_A	17.00788	0.001798	3640960
C2_07420W_A	16.97778	8.72E-09	3645202
C3_02140C_A	16.95698	1.28E-05	3636656
PLB5	16.86549	5.63E-10	3640750
C2_09040W_A	16.59808	4.17E-08	3645792
SWI6	16.50497	0.001969	3634932
HGT1	16.40609	1.98E-13	3645420
C5_02380W_A	16.39438	3.02E-13	3637794
C3_07230W_A	16.35838	4.22E-11	3638531
ACB1	16.34404	4.97E-09	30515350
HIS5	16.25675	8.24E-05	3645756
CHO1	16.23072	1.36E-06	3644557
FMA1	16.21731	0.00413	3647986
TAL1	16.12997	8.4E-15	3643225
ASK1	16.06027	0.00229	3635656
CR_04080C_A	16.04207	4.65E-15	3641023
ACO1	15.9938	4.53E-11	3642090
GND1	15.88623	2.05E-13	3636131
DPP3	15.87757	2.05E-06	3636942
SGA1	15.86014	7.61E-06	3636652
TPM2	15.72683	9.01E-14	30515441
C1_09980C_A	15.71929	8.98E-05	3634863
CAR2	15.64847	7.34E-09	3645027
CUP5	15.61149	2.18E-12	3635174
C4_01510W_A	15.55003	1.04E-07	3635616
C4_00390W_A	15.4811	0.000142	3645016
ALD5	15.39468	8.26E-11	3643026
LAT1	15.38238	4.9E-15	3636986
GLO3	15.35756	1.58E-15	3640556
LMO1	15.33249	1.87E-13	3641403
CIT1	15.30049	2.08E-12	3643201
C4_05080C_A	15.20142	1.04E-07	3641129
C4_04860W_A	15.14513	5.11E-07	3641161
C2_00590W_A	15.14413	0.000179	3638851
C1_09020W_A	15.05182	9.45E-07	3634917
GRP1	15.01655	0.00016	3634899
CYB5	14.99762	0.000176	3637965

C4_00020W_A	14.95452	1.79E-11	3648053
C5_03120W_A	14.93044	0.003771	3637705
SKN1	14.82069	2.9E-13	3641755
MEP1	14.80247	3.22E-07	3636553
AMO1	14.79282	1.4E-07	3642994
POR1	14.79171	1.39E-13	3644698
SEC24	14.75237	9.58E-14	3634939
AAT1	14.68204	4.34E-11	3643468
HIS7	14.66037	2E-08	3647868
C2_02020W_A	14.5807	2.16E-09	3635769
C2_07550W_A	14.49301	4.23E-09	3645221
RFC5	14.49207	0.005284	3638890
RHO3	14.20279	5.68E-09	3643448
ACS2	14.19287	1.52E-12	3644710
CDC3	14.06181	4.3E-13	3644719
C1_00700W_A	13.9201	0.004592	3639330
INO1	13.905	1.04E-12	3638966
C4_05790W_A	13.80816	4.32E-08	3641784
C2_02410W_A	13.8035	6.24E-10	3635755
SAP6	13.77133	0.008125	3639229
C4_03290W_A	13.76819	9.32E-10	3637887
C1_05180C_A	13.73247	0.004534	30514990
BIO2	13.6076	0.0137	3640106
STE24	13.55659	2.7E-12	3645040
CAT2	13.50325	8.46E-13	3635659
ABG1	13.49835	5.77E-08	3647715
C2_06610C_A	13.45618	0.004838	30515140
ARC40	13.42288	6.57E-12	3644866
TEF2	13.40452	1.06E-13	3646502
MEU1	13.39284	5.08E-08	3645635
C2_09780C_A	13.38389	1.52E-09	3643805
SEC8	13.36916	1.35E-09	3647078
C4_05250W_A	13.2927	8.29E-05	3646158
C2_09050C_A	13.25232	3.97E-06	3645791
RIC1	13.23605	2.44E-05	3639331
SOU1	13.16254	0.00127	3642799
KEX2	13.13275	7.21E-13	3634914
C1_04200C_A	13.05469	3.2E-05	3644718
CR_01080W_A	13.02592	5.58E-12	3640381
SEC72	13.00199	5.97E-09	3645768
NUO1	12.99216	1.82E-07	3647100
CSA1	12.98604	5.48E-09	3638009
STV1	12.9296	4.77E-12	3646070
PBR1	12.85228	0.006774	3639560
SOL3	12.80496	4.33E-05	3646625
RFC2	12.75777	3.72E-05	3637939
RPS17B	12.71772	2.9E-10	30515044

CDC42	12.67011	1.71E-08	3646488
YPT31	12.66146	3.09E-11	3647999
PLC2	12.60502	4.78E-05	3642985
ALG8	12.58623	0.000461	3636562
CRD2	12.56596	0.009141	30515234
RNR1	12.56213	1.44E-07	3642989
MRP8	12.49068	2.18E-06	3641178
ALS1	12.47132	1.36E-13	3640280
PRD1	12.41334	1.58E-13	3642471
C1_03720C_A	12.40199	6.21E-09	3648216
HHO1	12.39312	4.22E-05	30515358
PSF2	12.37321	0.008791	3647534
SUB2	12.3585	3.21E-12	3645033
ASN1	12.33523	2.54E-12	3645790
C4_04970C_A	12.25647	6.23E-05	30515274
INP51	12.25504	8.67E-06	3643806
C2_03020C_A	12.24664	6.87E-10	3643019
ARP3	12.19585	2.33E-11	3643479
IDH1	12.12122	1.48E-12	3634893
C4_04600C_A	12.10018	0.000561	3641156
CR_00880W_A	12.06119	0.000526	3640397
HGT3	12.0585	0.01136	3643185
ARC35	12.0102	4.14E-07	3639823
CR_04860C_A	11.95563	0.008948	3647294
C4_06810C_A	11.9397	1.89E-11	3642916
ATP5	11.93821	1.41E-12	3640521
SMC1	11.93284	0.000894	3643221
YHB5	11.88682	3.21E-13	3647343
THS1	11.84494	1.1E-11	3647882
KGD2	11.81161	9.15E-14	3646036
C5_00060C_A	11.70026	1.3E-06	3647889
LEU2	11.69279	8.14E-05	3638034
CDC24	11.67287	1.78E-11	3643737
PHO15	11.63713	1.19E-06	3644459
C1_05320C_A	11.62825	1.79E-06	3642470
C3_04780C_A	11.53891	9.39E-09	3635214
SEC27	11.4205	2.53E-13	3645870
C2_03210W_A	11.41652	0.000801	30515098
TRX1	11.36682	8.72E-10	3638950
BMH1	11.36525	1.16E-11	3636810
C1_09670C_A	11.33281	0.012568	3634872
C6_02480W_A	11.31131	7.05E-05	3639170
CR_00870C_A	11.30916	0.009636	3640398
IDP1	11.30665	3.02E-07	3647474
SPE3	11.26307	3.94E-07	3643528
RDI1	11.24964	3.89E-11	3635167
MCM6	11.22829	0.000111	3640142

PFY1	11.17447	6.78E-11	3640703
CRN1	11.17353	5.21E-11	3637034
NUC2	11.14525	5.25E-09	3637030
C5_04950C_A	11.13336	1.05E-05	3636422
VPS1	11.12113	4.67E-12	3642656
CR_05360C_A	11.12038	0.009644	3648033
SGT2	11.11959	4.67E-12	3642978
C4_04800W_A	11.04392	1.63E-10	3641113
RHB1	11.0236	0.000832	3635232
C5_02520W_A	11.00126	1.69E-05	3637768
PRO3	11.00062	2.33E-08	3645036
TPO3	10.99874	6.68E-06	3634790
ERG10	10.9674	1.45E-10	3648275
UGA32	10.96668	0.00015	3639333
VRG4	10.9269	1.63E-09	3641768
C1_05490C_A	10.92572	4.75E-08	3642507
C3_01070C_A	10.81609	0.010648	3639832
SEC26	10.79646	2.53E-11	3641005
APM1	10.78206	3.71E-09	3636424
VMA8	10.77252	7.48E-08	3642798
EGD1	10.73046	6.15E-10	3645340
PST3	10.72074	2.98E-10	3648031
CDC46	10.71213	0.000384	3646912
SSH1	10.69614	1.07E-06	3642504
ERG2	10.65192	0.011067	3639347
RIP1	10.58452	1.71E-10	3635204
HHT2	10.57716	3.04E-05	3647326
MES1	10.5524	6.27E-11	3636516
PDB1	10.55141	1.06E-11	3641186
MSH6	10.54495	6.69E-05	3636161
C3_04630W_A	10.5276	1E-06	3635189
FGR34	10.51367	2.82E-09	3639033
ERG13	10.47535	1.32E-08	3641881
ATP4	10.40245	7.45E-12	3643418
IHD1	10.37733	1.75E-11	3640291
WBP1	10.33444	3.99E-11	3644398
NHP6A	10.30444	1.43E-09	30515244
GIN4	10.29277	7.09E-06	3644543
C3_01020W_A	10.26586	0.001196	3639828
MIS11	10.26187	2.97E-10	3645732
GIT2	10.26052	4.59E-09	3642633
C1_04030W_A	10.2562	0.000224	3646428
KRE5	10.24166	5.84E-10	3638384
FAS2	10.22601	6.27E-11	3635222
SPF1	10.20648	1.3E-11	3646573
CR_10440W_A	10.19634	0.012019	3638976
C1_02270C_A	10.15691	1.29E-05	3644566

CRC1	10.15395	1.06E-08	3640112
CR_10140W_A	10.15164	6.95E-09	3638972
CAS1	10.09778	0.000238	3645324
FAA21	10.09491	7.77E-09	3638418
FOX3	10.09341	3.5E-05	3636629
VMA5	10.04394	2.51E-07	3642316
C2_04450W_A	10.04096	0.012021	3645467
C4_06160W_A	10.03913	0.001245	3642819
FGR38	10.01864	3.24E-07	3645412
ATP17	10.01064	1.46E-10	30515364
ZWF1	9.991992	8.98E-10	3634913
C3_05880C_A	9.960498	7.16E-10	3641737
NDH51	9.960419	5.06E-09	3645444
HUT1	9.949748	0.001288	3638505
CR_04770C_A	9.948766	0.013767	3647302
CAC2	9.94329	0.001242	3646978
C3_06400C_A	9.941318	0.001229	3642260
C5_05230C_A	9.920051	1.6E-11	3636470
C6_02880W_A	9.915503	0.013226	3639238
MCR1	9.873577	6.28E-11	3647561
CPH1	9.862307	1.74E-05	3644468
C3_07820W_A	9.81133	1.03E-05	3647463
C3_01940C_A	9.753469	5.58E-07	3636600
C5_04550W_A	9.749609	0.000341	3636508
C1_11960C_A	9.746125	0.013417	30515060
C3_02820C_A	9.730132	1.15E-07	3638419
PET9	9.70834	2E-10	3641587
C2_06770W_A	9.696068	0.013731	3643515
C2_02260W_A	9.685127	2.07E-06	3635778
HXK2	9.640497	3.08E-10	3641015
DAP1	9.633401	0.000471	3641021
HGT2	9.627447	3.36E-11	3644579
PUP2	9.617688	5.32E-10	3646617
C1_00630W_A	9.609066	1.08E-05	3639380
APL5	9.58311	6.58E-09	3638377
ALS4	9.580137	5.21E-10	3647972
ALG6	9.577169	1.62E-05	3647316
C5_00840W_A	9.575042	0.001479	3644130
RPL24A	9.559655	7.1E-10	3641174
CR_08610W_A	9.526834	2.25E-05	3642109
FUM12	9.521428	1.04E-10	3638489
C3_06920W_A	9.501887	2.12E-06	3638511
CR_00910W_A	9.483033	1.27E-09	30515371
AGE3	9.46991	1.17E-08	3644565
C3_01720C_A	9.462328	7.76E-10	3636601
GEA2	9.449598	1.82E-09	3636646
AXL2	9.435373	1.19E-07	3641184

C1_12650C_A	9.431305	0.01453	3646836
QCR7	9.419272	3.12E-07	3639233
ASM3	9.400796	0.001758	3639332
C3_00470W_A	9.390268	1.58E-08	3640520
GLT1	9.377367	3.7E-05	3639519
C7_01740C_A	9.360896	8.82E-05	3637010
IPP1	9.342677	4.11E-11	3643874
CR_05760C_A	9.290329	0.002042	3646695
EAF6	9.27216	0.015644	3646494
C1_09060C_A	9.268396	0.015848	3634772
C2_05850C_A	9.259069	1.66E-06	3647472
C2_05800C_A	9.195934	8.26E-09	3645584
C2_10860C_A	9.176777	3E-10	3641233
SEC34	9.167711	2.83E-09	3644455
DSL1	9.165772	0.000428	3645718
NPL4	9.156918	1.63E-06	3639820
CR_00290W_A	9.12847	3.55E-11	3639717
HEM13	9.126206	1.07E-09	3647390
YVC1	9.114375	3.13E-10	3642303
DED81	9.060183	7.85E-11	3646227
C3_04970C_A	9.011876	5.48E-10	3635164
SNG3	9.002755	2.06E-06	3646715
STE23	8.998554	9.48E-11	3639236
C3_06630W_A	8.968933	0.017191	3642250
ARE2	8.965871	6.05E-09	3643526
KTR4	8.964802	0.017842	3646425
C4_02420C_A	8.959338	0.018478	3637849
TRS20	8.880191	0.002606	3640214
C7_02080W_A	8.84059	0.000163	3637025
NPT1	8.824424	1.49E-08	3643109
VMA11	8.809865	8.84E-05	3637054
C6_00910C_A	8.808543	4.36E-10	3644030
STE13	8.797481	7.06E-06	3647597
SUN41	8.79043	5.49E-10	3647067
SDH12	8.784664	1.2E-07	3642775
C3_05550C_A	8.778133	5.76E-07	3643659
CR_05010W_A	8.699483	0.002826	3646389
C2_02310W_A	8.687299	2.2E-06	3635809
HSL1	8.678862	2.63E-06	3637810
ZCF24	8.654991	0.00023	3645417
C7_01500W_A	8.64151	0.000642	3637050
ROB1	8.639477	1.66E-09	3636212
FRS2	8.637385	1.11E-10	3636824
C5_05040W_A	8.629831	9.43E-05	3636488
ARO8	8.623154	8.51E-11	3638828
C1_00210C_A	8.591643	4.61E-11	3639335
PPT2	8.591505	0.022926	3634762

ATP7	8.575508	7.7E-09	3646672
C5_01590W_A	8.569591	0.000302	3647041
BNR1	8.551751	1.34E-05	3639672
C1_10750C_A	8.547663	0.003332	30515043
DPB4	8.54757	0.000676	3638818
TFP1	8.547363	7.2E-10	3636662
CDC19	8.533853	2.27E-09	3643438
CR_08480C_A	8.521764	3.79E-05	30515442
C1_06270W_A	8.515179	2.98E-05	3647523
CAK1	8.510694	1.29E-05	3637352
HOM2	8.494869	8.41E-10	3635826
IDP2	8.471076	4.69E-09	3644934
PEX8	8.467099	0.000332	3647393
C1_04470C_A	8.465273	3.55E-05	3646142
HGC1	8.435457	1.79E-06	3639349
C1_12140W_A	8.43359	3.2E-05	3642050
C1_01290C_A	8.429466	0.003677	3640184
GLC3	8.420783	6.78E-10	3639200
RHO1	8.405783	4.47E-09	3642564
RAS1	8.390282	7.21E-11	3643927
MUM2	8.389647	1.67E-09	3636432
LEU1	8.383136	2.75E-06	3639685
SHP1	8.358712	3.75E-09	3640083
C2_09030W_A	8.355906	0.022302	3645793
C3_01610W_A	8.339814	9.63E-09	3636664
C7_01320W_A	8.325003	6.31E-07	3645896
C1_03170C_A	8.268318	0.026108	3636805
C6_00920W_A	8.266765	5.61E-08	3644031
TSC11	8.261844	0.003907	3645267
PDX1	8.244515	1.14E-08	3636128
C5_04410C_A	8.230299	0.0014	3636410
C6_04280W_A	8.20978	1.82E-06	3646279
CR_04850C_A	8.209415	0.025259	3647295
MET6	8.207207	1.49E-09	3640084
ATP14	8.203656	7.38E-09	30515134
C1_11760C_A	8.201156	0.000158	3645328
C2_01910W_A	8.197238	0.023287	3635790
C6_00770C_A	8.190559	0.023922	3647079
ARO3	8.188037	1.05E-07	3635770
HMG1	8.179404	3.56E-07	3644687
CR_09560C_A	8.165431	0.026365	3647099
C4_04280C_A	8.151744	0.000743	3646846
PEX11	8.149276	0.000993	3646288
CLA4	8.095031	1.23E-08	3634878
EGD2	8.049893	9.03E-09	3635284
ARO4	8.045915	1.52E-09	3647668
DCK1	8.02913	4.47E-09	3637383

HBR2	8.024514	1.45E-09	3646273
MON2	8.022149	5.71E-10	3636201
ARP1	8.014407	0.005852	3646349
GVP36	7.995841	4.63E-05	3641772
APM4	7.967126	0.000883	3642297
BEM2	7.941723	1.08E-08	3637044
FTR2	7.93441	1.9E-06	3643303
C2_09970C_A	7.872653	7.82E-10	3643820
C6_03960W_A	7.860531	1.9E-09	3640269
UBA2	7.84999	8.16E-06	3640701
TPD3	7.835594	7.75E-09	3638512
C2_09100C_A	7.833991	6.55E-05	3644113
CR_07350W_A	7.804532	7.22E-09	3646055
MNN12	7.799779	1.64E-09	3634904
C2_03340W_A	7.789965	8.18E-06	3637392
C4_01840C_A	7.776357	1.34E-05	3635592
C7_00150W_A	7.756562	1.52E-08	3637973
C3_02290W_A	7.728547	5.87E-07	3636556
RAD52	7.720498	0.000378	3647148
C3_02210C_A	7.702235	1.03E-05	3636641
OPT6	7.695194	4.76E-06	3635603
TOM20	7.6912	2.41E-06	3636817
C5_02050W_A	7.682001	3.78E-09	3643768
IFA4	7.659863	0.005965	3645454
C1_10540C_A	7.658558	0.000557	3647193
C5_05200C_A	7.657644	8.29E-05	3636467
PDA1	7.656961	3.39E-08	3642902
MRV2	7.636262	1.33E-05	3636545
NGT1	7.62659	0.039355	3640573
C6_03950C_A	7.619357	0.000394	3640268
C1_14530W_A	7.556085	7.38E-08	3643333
KSR1	7.535887	0.000453	3646052
SDH2	7.526129	4.75E-06	3646397
CDC21	7.486637	0.038748	3643463
GNA1	7.475239	3.48E-05	3637397
SFL2	7.467464	1.21E-07	3636434
MPT5	7.465693	2.83E-06	3636205
OPI3	7.424515	0.030975	3642277
SNL1	7.421496	5.6E-06	3647192
C5_04150C_A	7.421134	0.001784	3636525
C7_03800W_A	7.416783	0.001398	3643150
TYR1	7.328819	2.59E-05	3635639
RFA1	7.308874	7.25E-05	3638823
HHF1	7.28578	5.18E-06	3644705
C4_07080C_A	7.261085	0.008909	3642905
NIF3	7.256648	0.000117	3646527
SER33	7.238783	4.92E-09	3642030

APE3	7.229659	0.001654	3643875
TMA19	7.21013	3.53E-08	3640399
C1_01800W_A	7.199881	0.007338	3645407
FESUR1	7.186711	3.16E-07	3638514
RPP2A	7.177218	1.34E-07	30515439
HOM3	7.175343	0.000229	3641771
VMA7	7.172469	0.001831	3637332
THR4	7.172189	5.91E-09	3637711
C7_01460C_A	7.163717	0.001413	3636958
C4_00770C_A	7.15653	2.27E-05	3645760
RPS15	7.141312	2.61E-08	3635194
PMR1	7.126377	2.2E-08	3638043
C3_04080W_A	7.125874	6.02E-08	30515200
CR_02630C_A	7.123663	0.038627	3642568
SCP1	7.11328	0.037355	3646554
C2_07110C_A	7.097394	0.000174	3643505
C3_01590W_A	7.095726	0.000681	3636666
YBN5	7.085915	1.06E-08	3644781
AIP2	7.054604	2.49E-09	3638393
RTS1	7.034261	1.54E-09	3646557
C1_02970W_A	7.033437	1.32E-07	3636835
C4_03050C_A	7.032876	2.17E-05	3637930
C1_06630W_A	7.030902	0.001512	3639544
SNF5	7.026929	5.26E-05	3648297
C1_02290C_A	7.026187	0.000548	3644568
SOD1	6.990539	0.000347	30515248
BIO32	6.978721	0.001634	3643430
C1_02650W_A	6.972347	1.98E-06	3636818
C2_08930W_A	6.966829	1.3E-07	3645795
C1_10310W_A	6.957902	0.040324	3634906
MVB12	6.946159	0.000746	30515400
C2_04330C_A	6.934901	2.05E-06	3645453
C3_01510W_A	6.920664	8.3E-05	3636571
C1_00420W_A	6.8769	2.32E-06	3639286
C3_02570W_A	6.839876	1.13E-05	3638375
HEM15	6.815579	2.36E-08	3645211
C4_04920W_A	6.814817	0.000808	3641172
C7_03350C_A	6.804703	0.041586	3646722
C1_03490W_A	6.780699	8.37E-06	3636914
CR_00060C_A	6.766078	6.37E-07	3639673
CR_05200C_A	6.710771	0.000844	3648080
MP65	6.709756	1.73E-07	3643951
C1_00450C_A	6.694454	5.32E-05	30514955
C2_00730C_A	6.682478	1.91E-05	3638913
C2_09650W_A	6.673424	1.28E-08	3643794
C1_12180C_A	6.668903	0.002328	3642038
C7_03660C_A	6.665825	9.72E-09	3646226

C3_03160C_A	6.664993	0.010281	3638424
CDC50	6.658991	5.82E-06	3640302
DPP2	6.65729	0.010497	3645341
CR_01100C_A	6.656556	0.043722	3640378
CR_05860W_A	6.655793	3.95E-06	3646703
MNN24	6.647387	7.8E-08	3638789
PXA2	6.643611	1.19E-06	3642055
DUN1	6.63952	0.002404	3636494
LYS2	6.638822	2.38E-08	3636875
ACC1	6.632364	1.09E-07	3639688
RPL8B	6.630815	6.2E-08	3635198
C4_02930W_A	6.630559	0.011304	3637876
C1_13270W_A	6.624755	0.000299	3636144
C1_12400C_A	6.619204	0.000931	3642059
C2_10830W_A	6.612823	0.015088	30515170
RAD53	6.592654	0.012228	3645633
C1_10510W_A	6.590793	0.046773	3647189
ZRT2	6.588192	1.5E-05	3635861
C5_01410C_A	6.56759	6.47E-06	3647054
C1_11740W_A	6.549349	0.002301	3645317
FAT1	6.548952	1.06E-07	3647083
C6_00930C_A	6.522591	6.23E-05	3644032
CAM1-1	6.490954	1.16E-07	3646358
CLB4	6.488649	0.01117	3643102
C1_08730W_A	6.482059	0.002338	3634938
PMT5	6.444262	0.0005	3639011
C1_11280W_A	6.441377	1.45E-05	3644554
ARP2	6.416802	3.72E-08	3641915
CR_03600C_A	6.409498	2.21E-06	3643207
YBL053	6.392742	0.000944	3647045
END3	6.38121	4.32E-05	3636644
C4_02040W_A	6.374456	0.000276	3635649
C1_04280C_A	6.361466	0.003046	3644709
HEM2	6.358928	5.8E-08	3637428
C4_00380W_A	6.329201	0.000658	3645015
RCT1	6.309773	3.58E-07	3641727
MDH1	6.276794	3.73E-08	3639667
C2_05560W_A	6.251865	0.003526	3645561
C2_09910C_A	6.250591	0.015798	30515162
UAP1	6.247257	2.19E-08	3637769
C5_01960C_A	6.236381	6.63E-05	3643738
C7_04120W_A	6.227169	0.001565	3643132
C7_03640C_A	6.22652	0.014058	3646223
CDC7	6.221631	0.000283	3643424
C1_05670W_A	6.202349	1.99E-06	3639899
C1_12240C_A	6.200708	0.000377	3642023
EAP1	6.19157	6.21E-07	3643814

C7_00600C_A	6.189329	0.001103	3638031
C4_03400W_A	6.18544	0.020816	30515256
C6_01960W_A	6.181032	0.006659	3644559
C2_03450W_A	6.178047	0.001339	3637349
ARC19	6.17121	0.005128	3640418
ABP1	6.171145	1.49E-08	3637875
HTA1	6.144053	2.41E-05	3645658
PHO89	6.141092	1.84E-07	3635633
C2_01260W_A	6.137039	0.014463	3638792
HFL1	6.133442	0.016362	3636883
SAS2	6.130831	0.014099	3638817
SAM4	6.129074	1.3E-08	3646506
CR_09670C_A	6.127159	4.15E-05	3647091
PLD1	6.097278	5.91E-09	3645329
ERG12	6.086796	3.43E-05	3634859
C3_06610W_A	6.081559	7.24E-05	3642248
LSP1	6.063994	1.62E-07	3648025
GRS1	6.062999	4.4E-08	3642474
BUD7	6.059015	1.17E-07	3647228
RFX2	6.04692	0.002958	3635658
C3_00210C_A	6.041429	0.015039	3640560
CPY1	6.01905	8.02E-08	3646721
CTA2	6.012993	1.11E-05	3639372
CR_04880W_A	6.005429	0.001646	3647309
MYO1	5.967666	2.26E-05	3647248
C1_02910C_A	5.948469	0.018746	3636829
C1_07780W_A	5.938438	1.54E-06	3647955
C1_01250W_A	5.935509	1.29E-05	3640212
CAM1	5.924961	1.97E-08	3641734
DQD1	5.915274	0.000122	3643488
ADO1	5.911867	4.34E-07	3639274
PGA33	5.908886	8.23E-05	3637350
C1_08080C_A	5.905358	7.33E-05	30515029
HEM14	5.900407	0.000364	3634923
KGD1	5.895119	5.2E-07	3647752
C7_02670W_A	5.886337	0.000244	3647651
CTA7	5.87097	7.5E-07	3637776
C4_06460C_A	5.866254	0.016	3642791
RPL43A	5.862761	4.58E-07	30515319
C4_04090C_A	5.857652	0.000135	3641225
FGR51	5.857221	0.000165	3647796
PIL1	5.84721	6.47E-07	3644769
CHS8	5.826818	4.76E-06	3640532
POT1	5.810206	5.41E-05	3639707
C3_03510C_A	5.797564	0.000149	3638310
MNN10	5.783226	5.49E-05	3644997
C4_01460C_A	5.77187	0.016684	3635622

AYR1	5.770057	0.000802	3647754
C2_06320W_A	5.730532	8.03E-08	3646927
SRO77	5.71523	3.28E-08	3646088
C2_02870W_A	5.70995	0.000108	3642972
VMA13	5.704577	3.84E-07	30515402
C2_02220C_A	5.692921	0.047299	3635774
C2_09250W_A	5.683189	0.01704	3644065
C1_03240W_A	5.670858	0.006092	3636895
AHP2	5.658486	0.000175	3636977
CMK2	5.65581	3.21E-06	3643961
CDG1	5.647131	1.92E-06	3641883
CDC54	5.639042	0.006515	3647606
ERG8	5.637382	6.44E-06	3635640
CR_07110C_A	5.628829	0.018933	3648224
C3_04590W_A	5.615918	2.15E-07	30515204
C4_00910C_A	5.606538	0.00663	3635679
FMO2	5.602758	0.02435	3637440
IDH2	5.591224	6.18E-08	3642979
LAG1	5.590822	0.001433	3640383
CR_06070W_A	5.581562	8.39E-05	3644840
PRO2	5.573196	4.21E-06	3638532
FOX2	5.568507	0.000127	3648327
C5_05350W_A	5.558487	0.005569	3636401
RFC3	5.552059	0.006981	3647440
C1_10730W_A	5.539204	2.95E-05	3644404
CHT4	5.536635	0.00789	3635768
C1_12070C_A	5.5277	0.000878	3642025
C4_02470C_A	5.523827	1.77E-06	3637869
FLC2	5.515942	1.61E-07	3647962
CHC1	5.511545	7.76E-07	3647552
C1_11120C_A	5.504122	6.08E-07	3644412
PIKA	5.502814	8.46E-07	3643765
C3_00310C_A	5.450714	4.8E-05	3640523
MMS21	5.439869	0.024721	3642265
SMC5	5.424758	0.002748	3645103
RBD1	5.423033	2.69E-07	3642018
C1_02330C_A	5.420957	1.32E-05	30514969
CR_01070W_A	5.420097	0.023876	3640382
C3_04420W_A	5.409301	7.9E-06	3635180
CR_08670C_A	5.404523	0.007031	3642126
ADE1	5.38496	1.38E-05	3639722
CR_01280C_A	5.367358	0.00271	3640400
GYP1	5.361963	6.24E-07	3641138
MNN26	5.359427	4.15E-05	3646240
IRO1	5.345225	2.2E-07	3636648
C7_01480W_A	5.342689	0.000958	3636956
C1_12060C_A	5.337814	0.021485	3642026

CR_06270W_A	5.333475	0.021785	3644854
C4_06860C_A	5.332392	0.021566	30515286
C6_01750C_A	5.332104	0.022134	3641640
EFB1	5.329831	3.2E-07	3641125
C4_00330C_A	5.326494	5.92E-06	30515227
CMD1	5.324582	2.36E-06	3646520
SER1	5.319138	1.76E-05	3646909
C2_01320W_A	5.314683	0.000135	3638846
RBT5	5.308137	8.84E-05	3645007
CR_01320C_A	5.284511	0.000846	3640386
C4_04180C_A	5.279597	0.002933	3641183
C3_04460W_A	5.276477	1.35E-05	3635208
C2_03290W_A	5.276405	0.000299	3637429
C4_03860C_A	5.246291	0.023553	3647854
PDR17	5.246008	0.02402	3643007
C4_03530W_A	5.240982	0.025068	3645376
C2_08160C_A	5.240836	8.95E-07	30515152
IFR2	5.231447	7.79E-07	3645100
C2_01160W_A	5.230462	0.00164	3638861
BUB1	5.223309	0.000164	3637922
TOP2	5.221698	0.000509	3642777
CYK3	5.197671	0.011663	3639539
CAT1	5.196823	5.57E-05	3639495
C1_00920W_A	5.185805	6.36E-05	3639283
CR_01180W_A	5.183388	0.000156	3640427
IFK2	5.182736	0.00108	3637439
CR_06520C_A	5.152356	0.00108	3645263
C6_03470W_A	5.144351	6.37E-06	3640253
CDC13	5.143262	0.000301	3639293
C6_04560W_A	5.139907	2.76E-06	3645977
GAL4	5.137987	1.07E-06	3641244
PRN4	5.132655	0.003262	3639877
C2_07980W_A	5.120097	0.001965	3642312
TIP1	5.113228	0.001818	3636512
OCH1	5.106377	1.42E-06	3641751
SEC4	5.106139	7.85E-07	3640119
C2_09560C_A	5.104174	6.06E-05	3643813
CHS7	5.102603	7.3E-06	3639869
DUT1	5.07202	0.028242	3640188
C5_03950W_A	5.07037	0.001637	3647445
C2_09810C_A	5.068799	0.000177	3643802
ARF2	5.067558	2.47E-07	3635271
ANB1	5.063088	4.46E-07	3641648
CR_05450C_A	5.063032	0.001182	3648236
SMF3	5.059933	0.000104	3638852
GSP1	5.05313	6.4E-07	3646925
SMC2	5.053002	0.003966	3643918

RPS12	5.051544	7.31E-06	3638438
RNH1	5.047888	0.000163	3639237
CCC1	5.044599	0.003893	3645645
MNN9	5.042605	2.11E-06	3641735
C4_00190W_A	5.039678	0.032224	3645030
C3_07770C_A	5.033574	7.47E-05	3647457
C7_02100W_A	5.028475	5.57E-07	3637023
APG7	5.028206	0.002167	3646615
C2_02090W_A	5.0212	0.001124	3635854
CR_03530W_A	5.018348	6.68E-07	3643204
GPX2	5.017698	3.64E-07	3644009
MDL1	5.014136	1.72E-06	3640146
C1_07510W_A	5.010103	0.030482	3646664
ACS1	5.008528	9.45E-05	3643941
PMT6	5.002545	2.81E-06	3641116
ERG9	4.999515	6.9E-06	3643913
C5_03140C_A	4.998905	9.07E-07	3637798
C1_02430C_A	4.995544	2.66E-06	3636846
C1_06200W_A	4.995318	0.000111	3648300
CR_02620C_A	4.994553	0.000178	3642567
CR_05680C_A	4.990849	0.003798	3646684
CTP1	4.986583	0.014916	3635141
C3_03080W_A	4.982532	0.001985	3638397
C7_00120W_A	4.979193	0.000456	3637976
C4_04160W_A	4.964606	9.48E-06	3641185
C7_03160W_A	4.956961	6.66E-07	3641345
C6_03050C_A	4.952022	0.037097	3639270
CHT2	4.939901	3.37E-05	3636523
C4_00410W_A	4.925038	0.000342	3645019
PLC1	4.891512	0.000475	3647860
CR_09520C_A	4.891087	0.031024	3647103
C6_00270W_A	4.872954	0.000412	3646066
C5_04420W_A	4.867012	0.002032	3636411
TIP20	4.866093	8.31E-06	3636457
LAP3	4.863248	8.25E-05	3641012
TEF1	4.862724	2.55E-06	3648254
C4_05340W_A	4.839855	0.000218	3646947
HYU1	4.839775	6.19E-05	3643024
C1_08800W_A	4.837081	0.000581	3634791
MAE1	4.833762	9.82E-07	3641615
SPE1	4.812509	1.3E-06	3639327
C3_06410C_A	4.804233	0.01558	3642261
C1_00820W_A	4.803044	0.033147	3639345
C4_03770W_A	4.797895	0.03318	3645354
C6_03580W_A	4.795897	0.006037	3640250
C5_01440C_A	4.793985	0.006445	3647043
VMA4	4.781401	2.27E-06	3640111

C1_05900W_A	4.765009	3.49E-07	3639866
UGP1	4.759202	9.71E-06	3648144
C1_02890C_A	4.758756	0.040296	3636867
ADE6	4.758255	3.3E-05	3647304
FAS1	4.757399	1.36E-05	3641593
MET13	4.749108	0.000243	3638382
C1_01010W_A	4.747363	0.000213	3640181
ILV2	4.747117	5.83E-07	3636552
SRV2	4.738655	1.46E-06	3640938
C6_01370W_A	4.73421	0.00023	3641607
ORC3	4.723849	0.000244	3645639
CR_02210W_A	4.713933	5.95E-06	3644943
C7_00140C_A	4.685289	0.000136	3637974
C1_07440W_A	4.681546	2.95E-06	3644482
COX4	4.654408	1.54E-06	3635737
RHO2	4.650796	0.008031	30515143
C3_06690C_A	4.641896	0.003623	3642256
C7_03780C_A	4.620232	0.040584	3643119
KIP3	4.607211	0.008749	3641716
C2_05170W_A	4.604194	0.00229	3643462
C4_05380C_A	4.599028	0.019559	30515276
PAN3	4.563835	0.000138	3636464
SAH1	4.563812	1.41E-05	3636452
C6_02530C_A	4.555931	0.020054	3639175
CR_05900W_A	4.55461	0.00854	3646681
APN1	4.553958	0.00071	3642263
C2_09620W_A	4.54856	0.000358	3643797
C7_00520W_A	4.54809	1.31E-06	3638001
NDE1	4.547453	7.58E-07	3638354
C1_14170W_A	4.546765	2.43E-06	3643323
C2_08650W_A	4.545553	2.74E-05	3643908
REV3	4.536442	0.000841	3641749
MNN2	4.529074	0.000425	3644405
C6_02470W_A	4.521126	0.000154	3639169
RPS1	4.516565	2.5E-05	3636924
C1_04930C_A	4.511236	0.004777	3644792
ATP20	4.507289	0.000327	3647602
C6_02330W_A	4.502678	7.44E-07	3645877
PMS1	4.500903	0.02329	3647714
ERG4	4.500671	2.18E-06	3640511
C7_03050W_A	4.495374	0.001789	3641378
C1_14240W_A	4.484747	0.000107	3643306
SSO2	4.478966	2.81E-06	3643809
YPT72	4.473119	0.000666	3643523
C1_10630C_A	4.472668	0.009594	3647683
BNI4	4.469513	6.62E-05	3646406
C1_12740W_A	4.4691	4.23E-05	3646819

USO1	4.468454	1.77E-06	3648280
EIF4E	4.446408	2E-05	3638981
RPP1B	4.443729	7.56E-05	3643104
CR_09070C_A	4.440979	0.000243	3641926
CR_02570C_A	4.438214	0.006647	3647685
RPL19A	4.434934	2.9E-05	3635257
C3_05280C_A	4.426884	0.011227	3635202
C1_01950C_A	4.423125	0.021815	3645421
CBP1	4.419083	6.46E-05	3641920
C6_00200C_A	4.416972	0.000164	3646082
CYP5	4.413257	3.21E-06	3642257
C1_02510W_A	4.406937	0.003141	3636796
ADH2	4.402566	7.19E-07	3640751
C6_00110C_A	4.402356	0.004484	3647896
ARO2	4.391233	8.01E-06	3648190
HOG1	4.389342	7.96E-06	3637393
APS3	4.371817	0.004787	3646491
DNM1	4.365143	5.27E-05	3643673
RPS7A	4.360379	1E-05	3636573
MDH1-3	4.359076	9.09E-05	3641197
CR_08440W_A	4.359072	0.024287	3642132
C4_04330C_A	4.357524	8.61E-06	3646840
ZCF26	4.354832	0.048205	3635666
PEX6	4.345437	0.000279	3643436
MMD1	4.344995	5.84E-06	30515015
CR_08350W_A	4.334134	1.57E-05	3642082
MNN21	4.325369	8.59E-05	3648124
C3_01180C_A	4.323613	2.01E-05	3647960
C1_02780W_A	4.320088	0.000419	3636871
CDC73	4.319965	0.000239	3636830
NHX1	4.317297	4.56E-06	3647141
SHM1	4.304162	1.56E-05	3646724
YKT6	4.302491	9.41E-06	3636863
C2_07270W_A	4.294738	0.002459	3643474
TLO9	4.294055	0.000789	3648051
C7_04310C_A	4.291712	7.64E-05	3643141
C1_10090C_A	4.291117	1.2E-05	3634817
TLO1	4.289848	0.001446	3639677
C3_07450C_A	4.286121	0.016185	3638481
MSC7	4.282537	0.000299	3645214
PTC7	4.276904	4.59E-06	3645000
C3_04130W_A	4.275276	3.35E-05	3635283
TRP99	4.272305	0.000326	3642494
ATP16	4.269726	6E-06	3638963
LYS21	4.263147	4.28E-06	3644763
UBC15	4.248402	0.000874	3641260
C3_07630C_A	4.246181	1.34E-05	3638491

ENT3	4.246172	2.51E-06	3635813
YHM1	4.235623	0.000286	3644535
CR_08980C_A	4.227858	0.000491	3641890
IML2	4.227143	3.33E-06	3643302
GWT1	4.226826	0.002129	3645575
RPS8A	4.22088	6.09E-06	3645565
MSI3	4.214665	1.7E-05	3639821
PNP1	4.210877	7.43E-05	3638426
SCL1	4.208555	1.69E-05	3640510
PPH21	4.205571	1.91E-05	3636665
C3_02190C_A	4.204058	0.000412	3636642
PXA1	4.200036	1.56E-05	3639713
RPB11	4.181735	0.022952	30514957
C1_13280C_A	4.177465	0.001529	3636145
VPS51	4.17663	0.027806	3639242
ZCF32	4.175263	0.006824	3635213
NIT3	4.172969	0.000911	3644408
C3_01140W_A	4.166966	0.004326	3639902
TRP3	4.155172	0.001317	3642035
C1_02680C_A	4.151313	0.002566	3636822
CCT5	4.14806	6.91E-06	3643478
CPR3	4.139963	0.000244	3635812
KEL1	4.139093	4.8E-06	3639318
RPN7	4.139033	1.28E-06	3641909
C2_01170C_A	4.137076	1.42E-05	3638860
C2_06950C_A	4.118096	0.028705	3643527
VCX1	4.100945	0.003644	3646508
UBA1	4.100064	2.07E-06	3642286
CR_09590W_A	4.096411	0.00423	3647095
CR_09570W_A	4.092861	0.03733	3647098
CHT3	4.087898	0.024286	3638967
RPL20B	4.086463	2.46E-05	3635615
C1_00330C_A	4.078621	0.004594	3639296
HTA3	4.077191	0.018898	3638322
CHS5	4.076529	5.62E-06	3637333
C3_01210C_A	4.072588	8.79E-05	3636612
RFC4	4.057295	0.013769	3638994
ARO9	4.055468	0.007539	3641773
ARC15	4.05516	0.001319	3646043
HFL2	4.050918	0.006183	3637833
ERG251	4.021857	1.92E-05	3635716
RPS26A	4.020876	0.000123	3635787
C6_03990C_A	4.012999	0.008697	3640271
RPP1A	4.007548	0.000123	3636839
C1_08780W_A	4.00676	9.4E-05	3634789
C1_09430W_A	4.005924	3.44E-05	3634855
C1_04460C_A	4.005611	0.024041	3646140

YSA1	4.004955	0.004925	3638516
YHM2	4.004864	0.000109	3647137
HAC1	3.999477	4.61E-05	3639849
PGA38	3.995974	0.040161	3637846
MCA1	3.99438	9.8E-06	3635233
CR_07570W_A	3.97479	0.002638	3648004
COX6	3.968101	1.02E-05	30515099
TOM40	3.964615	1.06E-05	3636968
ERG27	3.959248	0.000698	3640432
GLO2	3.958029	0.006693	3644067
C6_02580W_A	3.949989	0.000279	3639207
C3_07740W_A	3.946588	5.37E-06	3638453
C3_01310W_A	3.939431	0.010136	3636620
CR_07170W_A	3.934186	0.000781	3645271
MKK2	3.932895	1.99E-05	3645580
MED1	3.932297	0.001252	3637418
C2_03910C_A	3.929919	0.001437	3637426
CSP1	3.918971	0.005894	3648242
C3_01420C_A	3.917771	0.010675	3636634
C2_10470C_A	3.917255	0.001168	3641196
CR_08900C_A	3.916721	0.00054	3641908
C3_03840C_A	3.91211	0.001382	3645628
C3_04810C_A	3.909933	4.79E-06	30515205
C2_07720C_A	3.909067	0.000991	3642304
DDI1	3.879424	2.07E-05	3643295
C2_01690W_A	3.873824	0.000137	3635728
RPL18	3.869799	6.61E-05	3635156
EMP70	3.852388	9.9E-06	3637919
C1_00830W_A	3.849186	0.028817	3639344
CDC68	3.849058	6.18E-06	3642787
ILV6	3.847807	0.000903	3635598
C3_00910W_A	3.847204	0.041876	3647755
CR_10020C_A	3.837983	0.005294	3639032
CCT2	3.837818	1.05E-05	3643815
C3_00170C_A	3.830113	0.000805	3640589
LIP8	3.829812	0.005473	3646727
SYS1	3.828367	0.002338	3643138
C4_06940C_A	3.824544	0.013519	3642928
EXO1	3.824469	0.047552	3641583
KAR9	3.820378	0.012541	3636136
ZCF7	3.816531	0.036992	3636667
SUC1	3.812734	0.002637	3641916
C7_04280C_A	3.806238	0.000315	3643173
GDB1	3.797586	2.02E-05	3647409
TOM22	3.790525	0.005079	3647650
SEC3	3.789551	0.000181	3642806
CR_07370W_A	3.779737	0.003348	3646053

RAD7	3.776123	0.003301	3635833
LEU4	3.772035	0.038523	3639339
UBC4	3.767128	3.26E-05	3638927
CR_05140W_A	3.762688	0.009213	3646400
TOM70	3.759882	5.35E-05	3647645
FRP3	3.756627	0.000816	3648102
C2_05220C_A	3.75624	0.000109	3643466
RPL28	3.747916	1.22E-05	30515386
C2_06920C_A	3.746453	0.014044	3643524
C7_03260C_A	3.745562	0.014463	3641366
C2_00180C_A	3.743811	0.015926	3638905
C1_04190C_A	3.718987	0.00805	3644717
RPL10A	3.718767	9.94E-05	3645868
C1_01050C_A	3.71751	0.001525	3640170
VPS52	3.717021	0.001204	3638996
CR_02260C_A	3.707917	0.03236	3644912
PCK1	3.705119	0.000265	3639701
DOT1	3.704418	0.003536	3642236
GAT1	3.702616	0.005218	3641794
CSH1	3.702384	0.000525	3646427
GPI7	3.701056	0.01265	3647672
RPN2	3.699942	9.27E-06	3642027
C1_07340W_A	3.691404	0.000475	3644472
CR_00380W_A	3.676387	0.031047	3639682
C5_01980C_A	3.67327	0.001585	3643775
C4_01020C_A	3.666797	5.4E-05	3635606
ABP2	3.653198	0.046791	3643443
C5_03490C_A	3.652206	6.86E-05	3646967
C1_13190W_A	3.650994	5.6E-05	3636163
C7_00230W_A	3.648588	0.027589	3637982
C4_01730C_A	3.644255	0.001161	3635588
PEX3	3.639587	0.00322	3644484
C6_02560W_A	3.638922	0.012735	3639205
C4_03130W_A	3.627604	2.26E-05	3637921
C3_05010C_A	3.624619	0.005536	3635169
C1_07820W_A	3.615764	0.0429	3640735
CR_09010C_A	3.610807	0.000309	3641892
CR_05880W_A	3.600767	0.028246	3646702
CDC48	3.598504	0.000358	3644440
CNB1	3.59516	0.006195	3636463
FAA2-1	3.590622	0.001892	3644100
HOC1	3.589869	0.002034	3641605
CR_02410W_A	3.581474	0.005914	3644928
MIR1	3.577297	0.000225	3634782
HGT7	3.571017	0.00039	3638876
RTA2	3.57001	7.24E-05	3646567
ZCF39	3.56962	5.46E-05	3639037

CYC1	3.56024	0.000789	3643937
COQ3	3.551798	0.006053	3641630
C1_11100W_A	3.55021	0.009537	3644414
MEA1	3.54812	0.033535	3635154
DAO1	3.545193	0.001306	3636885
ZCF5	3.543083	0.040147	3641819
C5_02400W_A	3.541218	0.000294	3637724
SYS3	3.537813	2.19E-05	3645119
YPT1	3.53434	8.95E-05	3636915
C2_01900C_A	3.530223	0.000183	3635789
POL3	3.52156	0.001745	3641359
C1_03440C_A	3.521544	0.000803	3636908
VPS35	3.520519	4.95E-05	3645567
C6_01050W_A	3.51517	0.00528	3644015
C1_12700W_A	3.514355	0.009678	3646831
RPS16A	3.511864	4.99E-05	30514974
C4_06020C_A	3.511864	0.000195	3646519
GTT11	3.503801	0.003401	3645644
C1_13030C_A	3.492924	1.62E-05	3636122
C7_01930C_A	3.491217	0.008134	3637027
NAM2	3.4893	0.010202	3640275
C4_07240W_A	3.487261	0.002158	3642945
C1_07840W_A	3.486648	0.000717	3640737
RPL17B	3.484666	0.00033	3645439
CR_07080W_A	3.472962	0.000173	3647964
C5_00340W_A	3.467975	0.012369	3641555
C1_09750W_A	3.466902	0.002219	3634822
C5_01550C_A	3.466429	0.000161	3647063
PKH3	3.458827	0.000425	3646075
MCI4	3.454564	0.002442	3640118
C1_14060W_A	3.448728	0.000572	3643344
VPS8	3.444698	0.011367	3640534
C1_04620W_A	3.442335	6.2E-05	3646135
C1_00320W_A	3.441664	0.009167	3639297
LRG1	3.438669	8.28E-05	3639727
RSR1	3.437553	0.000192	3640145
BTS1	3.43434	0.020717	3646982
CR_03460W_A	3.430462	0.00251	3643213
COQ5	3.42716	0.001145	3643416
ECI1	3.426011	0.001677	3642128
SEC10	3.419897	0.000313	3642961
SES1	3.419709	0.000112	3638415
ALG9	3.413484	0.001777	3644457
C1_04090C_A	3.411574	0.039734	3644697
URA6	3.403657	4.3E-05	3647988
PRE2	3.398572	5.87E-05	3643490
LRO1	3.398005	0.000973	3639356

ALA1	3.389175	8.58E-05	3640285
C5_01350W_A	3.38494	0.000907	3642640
C1_10460W_A	3.3726	0.00176	3647183
ANP1	3.368125	0.002939	3643917
C3_03760W_A	3.368012	0.001525	3645638
RNA1	3.357223	0.00041	3636595
GLR1	3.342682	6.3E-05	3647061
C6_04250W_A	3.336202	0.000106	30515345
PGA59	3.332264	0.000811	3637881
ECM39	3.330477	0.013278	3641386
RIB5	3.32941	0.001129	3636499
EXO84	3.320346	6.02E-05	3644022
ERV1	3.310173	0.042756	30515385
C4_00590C_A	3.308915	0.000176	3645772
C3_01950C_A	3.30878	0.000572	3636599
MUQ1	3.307829	0.000932	3638898
C4_00780C_A	3.307399	0.000114	3645759
C5_04900C_A	3.301183	0.003201	3636416
VPS33	3.300772	0.0014	3647477
MCM3	3.297824	0.002531	3645223
PHO88	3.291517	0.001456	3641898
C2_04490W_A	3.284771	0.000852	3645463
OBPA	3.269104	0.000262	3643764
PTC6	3.268295	0.008032	3647351
CR_04330W_A	3.264814	0.013796	3640997
BET2	3.245244	0.008246	3639025
RPS30	3.241223	0.000776	30515390
C1_04580C_A	3.239455	0.014415	3646132
C7_00210C_A	3.239076	0.001527	3637983
C1_05920W_A	3.23826	0.033224	3639864
C2_03530W_A	3.237403	0.000225	3637341
YPD1	3.234746	0.001532	3644458
C1_05390C_A	3.228363	0.001453	3642479
C5_02310C_A	3.227603	0.002335	3637787
C6_02030C_A	3.226426	0.007092	3647562
RPN8	3.22526	0.000136	3643770
SFH5	3.224225	0.016194	3634884
C5_04770W_A	3.219041	0.021948	3636444
PPE1	3.215594	0.049045	3635802
CR_06260W_A	3.21392	0.00331	3644853
SEC1	3.212323	0.000253	3636935
C2_01390W_A	3.212183	0.000211	3635726
C5_02500C_A	3.204558	0.000656	3637766
CFL5	3.203075	0.038153	3642612
PHB2	3.196235	0.000843	3635238
LCB2	3.193209	0.001209	3636181
C2_01930C_A	3.190275	0.003656	3635793

C7_02470C_A	3.190074	0.010622	3636945
C3_01900C_A	3.189436	0.031564	3636559
C5_01890W_A	3.188043	0.003721	3643750
C3_00790W_A	3.187887	0.024141	3648186
SHM2	3.180966	0.000501	3640306
AAT21	3.171396	0.000168	3647241
APR1	3.169739	0.000219	3645204
C7_03480W_A	3.168961	0.000155	3646230
CR_01050C_A	3.164468	0.001808	3640384
RPL35	3.163878	0.00252	30515207
CR_01300W_A	3.159535	0.014145	30515373
RPP0	3.157231	0.001283	3638023
NUO2	3.15524	0.000167	3638381
C1_08050W_A	3.153911	0.000822	3640709
CR_07230W_A	3.153193	0.012535	3646031
C7_00420C_A	3.152968	0.010372	3638032
CR_08050C_A	3.151544	0.001799	3644133
C1_11890W_A	3.149874	0.003068	3642006
C7_03200C_A	3.147333	0.015023	3641342
ILV1	3.145649	0.002879	3646918
C3_00050C_A	3.141758	0.019385	3640570
C4_05850C_A	3.139253	0.029473	3641791
PGA52	3.138234	0.000108	3647908
CAS4	3.136364	0.000479	3636568
RAD51	3.1344	0.005833	3644944
SSB1	3.130387	0.001167	3642095
C3_06970W_A	3.126409	0.00543	3638506
PHO85	3.126243	0.000679	3646148
ERG24	3.119446	0.038918	3647716
FEN12	3.118283	0.001712	3637404
RPS14B	3.115388	0.00056	30515006
ALS2	3.108051	0.000883	3645724
C2_07100W_A	3.097429	0.001161	3643504
C3_03340C_A	3.097071	0.012581	30515195
EFT2	3.090014	0.001543	3642998
FBA1	3.086315	0.001796	3635585
C1_14500C_A	3.081537	0.000547	3643331
C1_07100C_A	3.081267	0.04458	3639487
C1_01000C_A	3.080769	0.046675	3640180
CR_10600C_A	3.070685	0.000162	3639004
C1_06670W_A	3.070225	0.002542	3639541
HXT5	3.060491	0.033838	3643209
RAD16	3.057254	0.00021	3636874
CR_00090C_A	3.053579	0.000406	3639734
C1_02320C_A	3.052493	0.000237	3644572
C1_14030W_A	3.0486	0.002144	3643340
C1_07630W_A	3.046552	0.011966	3646660

C2_05590C_A	3.045937	0.007807	3645564
BIG1	3.043247	0.005373	3644434
HAT2	3.040821	0.001555	3645973
C6_00340C_A	3.03275	0.003284	3646074
C7_02560W_A	3.027451	0.002407	3637016
C2_10720C_A	3.0249	0.001026	3641209
CR_09510C_A	3.013919	0.002382	3647105
ERG5	3.012704	0.000155	3641400
PST1	3.004113	0.004671	3643520
C5_02750C_A	3.001568	0.002882	3637740
C2_10670W_A	2.998515	0.000318	3641251
CFL2	2.998077	0.040201	3641811
RPN1	2.993909	0.000542	3636147
CR_10590W_A	2.993536	0.003318	3639003
FRS1	2.990909	0.000688	3640121
ECM15	2.985888	0.011058	30515218
C5_04470C_A	2.985562	0.00048	3636414
C1_13490C_A	2.982605	0.020008	3636230
C7_03370C_A	2.979713	0.000207	3646720
C6_00080C_A	2.9792	0.018092	3647899
C1_09070W_A	2.978826	0.00211	3634773
POB3	2.978031	0.000445	3635827
C1_02160W_A	2.977932	0.02033	3644584
C4_03320W_A	2.976292	0.009262	3648092
GCN4	2.975472	0.000387	3643823
STP1	2.973826	0.023283	3635152
BEM3	2.972139	0.000211	3637885
C3_06680C_A	2.969209	0.047967	3642255
C7_00250C_A	2.968337	0.002763	3637979
CR_10290C_A	2.966737	0.000357	3638935
C6_03330C_A	2.965182	0.026171	3639199
C5_04710W_A	2.959707	0.031406	3636517
ERG7	2.958764	0.000279	3635761
CR_08000C_A	2.954169	0.000265	3644150
RIB4	2.951151	0.001181	30514995
TFC4	2.950086	0.011524	3638420
FUM11	2.948993	0.00245	3641016
C2_07610C_A	2.946371	0.000218	3645195
RIM1	2.942539	0.011662	3639898
PDS5	2.939677	0.010579	3642341
C1_08110W_A	2.936458	0.000492	3640716
C5_05440C_A	2.92545	0.000845	3636431
RFC1	2.923625	0.001715	3645582
C3_03210W_A	2.923005	0.035717	3638315
CHS6	2.914105	0.021667	3641377
C6_03820C_A	2.910948	0.006815	3640288
LPD1	2.910244	0.001082	3646037

C5_00040C_A	2.90745	0.000642	3647874
KRS1	2.902415	0.000844	3638486
SWC4	2.901764	0.005537	3639679
C2_04170C_A	2.895563	0.000444	30515111
CR_00470W_A	2.885032	0.000485	3639725
C1_08680C_A	2.883559	0.007721	3634933
CTA24	2.878725	0.002748	3636475
C7_00410C_A	2.871329	0.030295	3638033
GYP2	2.869192	0.001511	3641780
C6_04060W_A	2.866542	0.003825	3647828
C1_06190C_A	2.864881	0.006711	3648391
C1_02490C_A	2.856406	0.032734	3636853
PRC3	2.853337	0.002892	3639889
POL2	2.851164	0.010539	3645733
CR_02770C_A	2.851109	0.024039	3642581
C5_01420W_A	2.84678	0.017499	3647055
GPR1	2.842382	0.001048	3642616
SIM1	2.835401	0.009317	3636185
CR_03690W_A	2.83469	0.011572	3643222
PPZ1	2.831172	0.00469	3645265
ARP8	2.829629	0.000274	3640200
ARL3	2.825537	0.023834	3644397
APT1	2.823035	0.027171	3635842
TPI1	2.819501	0.001	3638482
TFS1	2.81871	0.001057	3642655
C2_04410W_A	2.811249	0.033017	3645455
TLG2	2.804966	0.024478	3635773
YCS4	2.802457	0.002712	3645196
RAD6	2.800565	0.016537	3643143
HHF22	2.800539	0.011789	3647327
PEP3	2.795462	0.02109	3639267
C2_02840C_A	2.790663	0.014798	3642976
SGT1	2.785465	0.000485	3644068
ALT1	2.784704	0.012629	3638307
VAN1	2.784665	0.000802	3638527
C6_02410W_A	2.778859	0.002798	3645884
CR_08560C_A	2.777187	0.00369	3642142
C1_08490W_A	2.775663	0.003658	3646492
ETR1	2.775257	0.000412	3640561
C7_01160C_A	2.775065	0.027346	30515351
C6_02810C_A	2.769963	0.003076	3639248
CDC55	2.768113	0.000436	3635785
ADP1	2.762542	0.000591	3642458
C2_07440C_A	2.762063	0.025517	3645200
CSH3	2.761052	0.000452	3646798
SYN8	2.753412	0.026446	3645120
RPL13	2.748658	0.001233	3636841

PGA49	2.747585	0.03008	3646525
C6_02680W_A	2.745854	0.017412	3639226
C2_10700C_A	2.745548	0.011722	3641207
PDC11	2.74543	0.004294	3642780
FGR24	2.743752	0.006392	3643288
C3_00610W_A	2.741268	0.041887	3640578
CR_01350C_A	2.737721	0.001217	3640166
C2_10630W_A	2.734481	0.009934	3641248
YST1	2.730951	0.001024	3643678
CR_04460C_A	2.728157	0.038899	3640988
C3_00850C_A	2.720648	0.003642	3647748
C1_14040W_A	2.708295	0.003532	3643342
SAL6	2.707242	0.000942	3640289
RPL37B	2.696582	0.003414	30515053
C2_06110W_A	2.689056	0.010358	3644094
C1_11250W_A	2.685831	0.020544	3644556
RPN6	2.684824	0.008281	3645352
VTC3	2.682125	0.000573	3643206
VAS1	2.680359	0.001979	3648340
C4_00170W_A	2.675472	0.0024	3645028
ZDS1	2.668906	0.002369	3641103
C5_00050W_A	2.664519	0.02453	3647875
PEX1	2.664446	0.006188	3636943
RPL5	2.663063	0.004708	3637057
AKR1	2.658719	0.000912	3636166
C1_09470C_A	2.65657	0.006451	3634761
AGO1	2.650296	0.00347	3642828
DPS1-1	2.649204	0.00245	3645134
GCF1	2.646993	0.003502	3646498
ADE5,7	2.646912	0.005433	3640719
MSK1	2.646783	0.030873	3637032
CR_08130W_A	2.645768	0.025447	3642070
C2_00400C_A	2.637896	0.006193	3638821
SUR7	2.637455	0.001984	3641610
C3_06490W_A	2.637018	0.009531	3642285
C1_14180W_A	2.632919	0.022925	3643324
POX1-3	2.627403	0.021021	3636598
C6_00190W_A	2.627343	0.020071	3646080
RPL6	2.626444	0.002115	30514975
C5_00750C_A	2.622795	0.005791	3644121
ACP1	2.621476	0.008855	3639825
URA4	2.615413	0.012625	3642632
MAC1	2.611606	0.032286	3637985
C4_02780W_A	2.607419	0.003238	3637902
ILV3	2.599883	0.005369	3636428
C1_13060C_A	2.598583	0.004164	3636123
C1_04980C_A	2.597589	0.006866	3647529

C5_00580W_A	2.595371	0.008415	3641588
RNR21	2.594976	0.008114	3643021
C3_01820W_A	2.593932	0.015303	3636584
MRP2	2.593906	0.02456	3636498
DAP2	2.587679	0.010822	3637815
RPD31	2.58137	0.007112	3638520
CST20	2.580029	0.018185	3637790
ARC1	2.579689	0.00125	3645108
C1_00660C_A	2.576965	0.001323	3639376
ARP9	2.575517	0.031686	3639829
RPL3	2.573651	0.006478	3647719
CDC47	2.565239	0.013768	3645780
RAC1	2.560721	0.001545	3639508
NUP159	2.557997	0.004657	3647458
MED16	2.557144	0.007772	3646725
SRD1	2.557004	0.029122	3635764
SLC1	2.552063	0.033172	3638324
C2_05580W_A	2.5496	0.003617	3645563
C1_14090W_A	2.547684	0.011685	30515079
C2_00060C_A	2.547452	0.000644	3647914
C7_00790W_A	2.545386	0.009811	3637943
C3_04360W_A	2.539839	0.012354	3635276
C1_12920C_A	2.539525	0.031273	3636155
C2_09930W_A	2.535502	0.024276	3643824
ALG7	2.533538	0.031237	3642291
C2_01340W_A	2.531677	0.002106	3638844
C1_11600W_A	2.526713	0.036845	3645346
GLO1	2.5266	0.012857	3639312
C1_02240W_A	2.522099	0.001326	3644597
CR_08620C_A	2.5192	0.039809	3642110
TIM44	2.518772	0.001413	3640529
ASR3	2.516094	0.002341	3637402
TUF1	2.515857	0.002456	3639360
TAF14	2.511776	0.009037	3637357
RPT4	2.511226	0.002027	3640966
C2_03900C_A	2.51117	0.002325	3637395
TRP5	2.510456	0.001549	3648087
C3_01910C_A	2.509564	0.016434	30515182
GBP2	2.508828	0.001745	3637992
ZCF31	2.506743	0.003085	3635191
C1_12430W_A	2.503684	0.012375	3642013
MSH2	2.496618	0.037832	3642939
PR26	2.495842	0.001891	3642981
C5_00790C_A	2.493876	0.006156	3644125
AVT4	2.493262	0.010725	3645316
C1_02700C_A	2.493246	0.008607	30514972
CR_01820W_A	2.486681	0.005165	3640164

PKH2	2.486447	0.006137	3642058
CR_00350W_A	2.486252	0.002839	3639686
C2_00570W_A	2.485359	0.004514	3638853
C1_12170C_A	2.484182	0.032664	3642039
C1_09360C_A	2.483945	0.001885	3634807
C1_05720W_A	2.481875	0.007863	30515000
SMT3	2.481254	0.003913	3644550
INT1	2.47867	0.004335	3637761
CR_09190C_A	2.476473	0.041326	3641885
C4_06770W_A	2.472649	0.01103	3642920
C1_12830C_A	2.471621	0.009871	3636176
C2_08270C_A	2.470841	0.019301	3642347
CR_03860C_A	2.466454	0.011641	3643186
RPN3	2.465335	0.002217	3636917
C2_00320W_A	2.463224	0.03058	3638798
UBI3	2.458014	0.010471	3642934
EBP7	2.457885	0.036105	3642971
CR_00490W_A	2.457503	0.010039	3639723
CR_09100C_A	2.454357	0.0156	3641928
C1_04860W_A	2.450997	0.003806	3644784
C3_05150W_A	2.450223	0.004831	3635162
SAC6	2.448943	0.008342	3639213
C1_07050C_A	2.444034	0.017096	3639532
C1_12660W_A	2.443348	0.044369	3646835
GDI1	2.44323	0.002406	3643298
LTE1	2.436734	0.016687	3643495
PEP1	2.432599	0.002108	3647612
NTH1	2.431692	0.004366	3639665
COQ6	2.430954	0.008512	3636878
TCP1	2.430817	0.007103	3646499
FDH3	2.425506	0.006981	3638923
C1_02040C_A	2.424704	0.009048	3644586
NMT1	2.424194	0.008929	3635624
ALG2	2.41914	0.03045	3647832
MKC1	2.419032	0.002569	3639710
RLM1	2.412727	0.004515	3635703
C5_02230W_A	2.408661	0.001979	3637708
C2_10460C_A	2.401298	0.016443	3641195
UBP13	2.40124	0.003194	3638879
CR_06560C_A	2.400257	0.018848	3648367
C1_11850W_A	2.400051	0.00986	3642010
GCV3	2.393073	0.003768	3636194
MID1	2.392555	0.03482	3647441
VPH1	2.386094	0.004831	3646157
C2_02000W_A	2.38584	0.002237	3635767
UBP6	2.383399	0.004237	3639317
C4_04720W_A	2.380394	0.026887	3641137

C1_08690W_A	2.380315	0.034208	3634934
RPS13	2.378904	0.005715	30515328
ECM4	2.378816	0.007525	3640144
FDH1	2.377671	0.029389	3646398
RPS5	2.375371	0.007896	3637700
HPC2	2.374576	0.032738	3636188
PSD2	2.373384	0.008722	3636515
C1_11140W_A	2.371379	0.021936	3644410
COX8	2.369149	0.004316	30515130
C2_07190C_A	2.368284	0.022191	3643513
SRP101	2.363755	0.006433	3636513
GDA1	2.360271	0.003074	3641762
CR_06930W_A	2.35925	0.048223	3648382
C3_02970C_A	2.358902	0.036756	3638385
C7_03010W_A	2.351981	0.002354	3641382
RPS6A	2.345161	0.008151	3635701
C3_02160C_A	2.342326	0.004675	3636654
C1_05780W_A	2.342054	0.00715	3639888
RPL2	2.33582	0.015428	30515049
TEC1	2.332873	0.015211	3635261
VAC8	2.328072	0.005166	3647410
RIM13	2.327598	0.015151	3636487
KEM1	2.324786	0.00532	3636222
YRB1	2.321083	0.003292	3639663
BCY1	2.320904	0.004146	3638832
SEC9	2.315414	0.006128	3644052
C4_04390W_A	2.314898	0.010557	30515267
C2_00830C_A	2.313391	0.018907	3638808
VPS17	2.308007	0.004111	3640218
CR_09530C_A	2.304961	0.00248	3647101
C7_02780W_A	2.300867	0.007046	3641361
FAD3	2.291838	0.014276	3636124
CR_08640C_A	2.286219	0.018401	3642112
PRE10	2.283328	0.005193	3636957
COX7	2.283093	0.021029	3638339
DBP5	2.280254	0.008376	3636564
RPS4A	2.280147	0.010738	3641247
C1_01300W_A	2.277731	0.01847	3640185
PUP3	2.275124	0.009735	3646718
C1_04640W_A	2.267745	0.025138	3644761
C3_02120W_A	2.258719	0.022929	3636658
HPA2	2.25868	0.021647	3647907
MRE11	2.258602	0.038593	3645898
KIN2	2.256507	0.009758	3639697
YBP1	2.25359	0.024229	3636187
RPL4B	2.252607	0.021008	3643347
SWI4	2.24906	0.031943	3645408

C5_04330W_A	2.248367	0.034588	30515314
RIM20	2.244668	0.013886	3634809
C6_00430C_A	2.242575	0.026102	3646089
RPS3	2.241197	0.013664	3647299
MGM101	2.239454	0.044646	3636820
C1_10820C_A	2.2361	0.010681	3644435
C3_00640W_A	2.228847	0.005676	3640574
C1_01400C_A	2.224377	0.02567	3640237
C1_04630C_A	2.223486	0.006377	3644762
GPD2	2.223253	0.02713	3644563
CR_02300C_A	2.219137	0.006627	3644938
C4_06690C_A	2.219114	0.024181	30515285
PEX7	2.213893	0.04455	3644027
PKC1	2.213257	0.012681	3635210
DTD2	2.192355	0.026001	3638390
ERF1	2.190653	0.014138	3643455
C3_07850W_A	2.188425	0.02016	3647466
C2_04370W_A	2.186244	0.026897	3645460
ISN1	2.186041	0.012845	3640196
ECM29	2.18266	0.012409	3638433
C5_04960W_A	2.18176	0.017513	3636423
SLA1	2.181373	0.010844	3635739
RPL25	2.181262	0.023229	30515338
MET15	2.178993	0.036881	3645031
C3_06350W_A	2.176358	0.044402	30515215
C5_02390C_A	2.170699	0.01366	3637795
MAM33	2.170545	0.035209	3643103
VTI1	2.161236	0.016731	3638352
C6_04110W_A	2.159243	0.011662	3647834
IRS4	2.153176	0.030593	3645650
SEC15	2.148918	0.010274	3646845
C3_06710W_A	2.146131	0.027401	3638448
NPL3	2.139368	0.018058	3643310
C1_03690W_A	2.132369	0.030038	3648426
C2_07220W_A	2.131508	0.021893	3643483
C4_00500W_A	2.121184	0.037106	3648379
PIN3	2.119033	0.015464	3635228
RPL32	2.119006	0.029863	30515335
STB5	2.116046	0.044705	3640208
C3_01800C_A	2.101643	0.039041	3636586
C3_05410W_A	2.0926	0.02111	3643665
PGA56	2.091706	0.032416	30515313
C1_10680C_A	2.09026	0.01899	3647678
YPT52	2.086862	0.02348	3643346
C4_01950W_A	2.077355	0.01997	3635665
C5_03980W_A	2.077315	0.027784	3647442
CDC53	2.075387	0.009402	3636603

RPS21B	2.075063	0.037494	30514961
SIT4	2.073284	0.02656	3647993
SRB9	2.073225	0.037668	3635845
SBP1	2.071011	0.016109	3635280
NCP1	2.067851	0.014001	3637839
MNT2	2.066764	0.040741	3636583
C2_02390W_A	2.064409	0.017968	3635829
PRE9	2.061414	0.021737	3638311
RPT5	2.055811	0.016123	3642911
CR_04720C_A	2.05559	0.008056	3648140
C5_02110W_A	2.054778	0.009876	3637732
SNF4	2.048692	0.010367	3640264
C5_02450W_A	2.048249	0.039284	3637729
C4_04750W_A	2.047662	0.028634	3641133
C2_05040C_A	2.045811	0.012691	3643449
ADE17	2.043642	0.019752	3641024
PUP1	2.039829	0.016712	3638936
RPL15A	2.037821	0.035568	3641025
CR_05480W_A	2.035631	0.040118	3648238
VRP1	2.035001	0.034131	3642293
C5_03170C_A	2.034493	0.0395	3637801
ATC1	2.032576	0.035082	3639570
RPS18	2.027343	0.041247	3637953
NRG1	-2.0018	0.013845	3643166
C2_06600W_A	-2.0023	0.021268	3646563
RIA1	-2.00571	0.021722	3644478
C4_02110W_A	-2.00675	0.039144	3635673
C3_04380C_A	-2.00871	0.044612	3635173
C2_02490C_A	-2.01263	0.010971	3635815
MBP1	-2.01315	0.009364	3635281
C5_00260W_A	-2.0141	0.00978	3641561
CR_08400C_A	-2.01583	0.045994	3642115
PSA2	-2.01674	0.005998	3636159
CR_07320C_A	-2.01886	0.018868	3646057
C1_04370C_A	-2.02286	0.026596	3647994
CDC37	-2.02965	0.022649	3639210
C2_10680W_A	-2.03064	0.0189	3641252
C1_06910C_A	-2.03454	0.022798	3639526
C6_03380W_A	-2.03476	0.01333	3639231
PAM16	-2.0405	0.029587	3643321
CR_00820C_A	-2.04081	0.029707	3640413
C3_05360C_A	-2.04093	0.031751	3643676
C4_02920W_A	-2.04132	0.02585	3637878
C4_07130W_A	-2.0447	0.022499	3642938
SFT2	-2.05564	0.028015	3644442
SET2	-2.05579	0.010782	3643923
C1_01710W_A	-2.05796	0.01231	3640201

TAF145	-2.06944	0.011005	3639486
CR_10730C_A	-2.06989	0.02251	3638998
C2_08240W_A	-2.07185	0.004012	3642344
C4_06420W_A	-2.07281	0.01419	3642795
C4_00880W_A	-2.07567	0.029069	3635681
C2_00740C_A	-2.07611	0.005079	3638912
C2_00230W_A	-2.07623	0.028667	3638901
PFK1	-2.08026	0.030307	3636448
DUG3	-2.08157	0.008565	3641381
ARO10	-2.08379	0.028291	3647320
PRS1	-2.08545	0.015758	3635817
FTR1	-2.08631	0.039249	3643318
WAL1	-2.09095	0.010492	3647093
C7_03270W_A	-2.0951	0.014857	3641365
C5_01090C_A	-2.09513	0.045761	3642625
C1_10910C_A	-2.0986	0.029368	3644445
OPY2	-2.1022	0.007803	3640765
ADE8	-2.10476	0.044447	3642999
PEX5	-2.10886	0.009146	3645026
ERB1	-2.10923	0.02442	3644702
C6_04290W_A	-2.11456	0.003762	3646280
ERG3	-2.12154	0.036528	3644776
VPS24	-2.12543	0.009285	3638892
GRX3	-2.12631	0.008256	3637858
C6_03370W_A	-2.12738	0.037423	3639230
MNL1	-2.12969	0.00794	3646050
C3_02630C_A	-2.13058	0.043358	3638328
C2_00490W_A	-2.13067	0.002543	3638870
VPS27	-2.1307	0.003039	3639326
C5_04180W_A	-2.13876	0.0449	3636544
SLR1	-2.13987	0.00683	3643957
C1_06070W_A	-2.1462	0.01612	3639824
URK1	-2.14785	0.005156	3635186
SEH1	-2.15224	0.01433	3642290
BMT5	-2.15268	0.002359	3635781
STI1	-2.15551	0.002474	3643758
C7_03280C_A	-2.15636	0.025764	3641353
MXR1	-2.15817	0.042579	3638881
IFF9	-2.15882	0.006252	3641008
C1_05120W_A	-2.16227	0.018887	3647667
XYL2	-2.16953	0.009664	3638961
SUI3	-2.17134	0.003738	3643130
MEF2	-2.17376	0.019698	3639564
C1_09540W_A	-2.17422	0.020103	3634885
NHP2	-2.1749	0.022135	3641003
PHO4	-2.17575	0.024248	3641817
CR_07250C_A	-2.18569	0.033876	3646028

C3_07070C_A	-2.18637	0.016746	3638446
C1_02900C_A	-2.1867	0.04555	3636869
C3_04790W_A	-2.18927	0.043629	3635215
SLU7	-2.18975	0.042475	3638501
DOT6	-2.19029	0.009251	3640078
C3_06440W_A	-2.19287	0.00337	3642281
HEM1	-2.19768	0.001781	3640149
SMC6	-2.19928	0.009043	3637039
BLP1	-2.20371	0.007076	30515065
C1_10140C_A	-2.20673	0.009261	3634780
RHD3	-2.20735	0.003244	3641212
C2_03760C_A	-2.21493	0.007912	3637371
C5_00390C_A	-2.21618	0.007393	3641552
CR_09480W_A	-2.21823	0.019748	3641939
C2_05770W_A	-2.22138	0.004993	3645579
C1_03400W_A	-2.2226	0.005097	3636904
C2_00770W_A	-2.22428	0.017891	3638909
C3_03330C_A	-2.2268	0.013261	3638364
LPG20	-2.22786	0.04991	3644780
SEF1	-2.22788	0.006172	3644945
CR_02460W_A	-2.22802	0.00993	3644922
C1_05540C_A	-2.23275	0.01934	3642503
C5_00820W_A	-2.23536	0.017124	3644128
KCS1	-2.24549	0.026172	3648120
BNA4	-2.24671	0.007279	3640528
C2_00170C_A	-2.24708	0.009062	3638906
C1_06470W_A	-2.26377	0.018817	30515007
HGT8	-2.26422	0.02121	3638875
CKA1	-2.26474	0.006037	3639018
SCT1	-2.2661	0.005167	3648346
ESA1	-2.26612	0.011141	3640518
IFF3	-2.26652	0.006262	3643189
C7_04140C_A	-2.26675	0.025266	3643129
ASR2	-2.26717	0.00238	3641907
USO6	-2.28013	0.003912	3643647
LIG1	-2.28379	0.001432	3636947
C3_04700W_A	-2.29371	0.045386	3635247
C2_08860W_A	-2.29374	0.002746	3645776
BUD14	-2.29562	0.005507	3643469
WOR4	-2.29993	0.002428	3638454
CWT1	-2.30421	0.004501	3647595
C3_07490W_A	-2.30434	0.001072	3638528
GLN3	-2.30486	0.004465	3636453
TPS1	-2.30578	0.000815	3646699
MTR2	-2.30678	0.021091	30515167
DES1	-2.31046	0.003605	3636529
CR_03110W_A	-2.31288	0.044998	3645124

C5_05140W_A	-2.31357	0.023541	3636460
C7_00260C_A	-2.31516	0.001476	3637978
C4_02090C_A	-2.31532	0.015604	3635642
C5_03150W_A	-2.31715	0.004168	3637799
C6_03910C_A	-2.32077	0.03581	3640263
SPL1	-2.32092	0.003789	3638035
CR_01370C_A	-2.32223	0.003294	3640168
SIS1	-2.32448	0.003876	3644841
C3_07940W_A	-2.32513	0.006611	3647453
C1_03120W_A	-2.32878	0.000697	3636926
STD1	-2.32966	0.005465	3647459
PHHB	-2.3305	0.010034	3638871
C4_00080C_A	-2.33537	0.016156	3648065
C2_07370W_A	-2.34011	0.024598	3645233
C2_07680W_A	-2.34322	0.009799	3642339
BRO1	-2.3435	0.006208	3636590
SAP10	-2.34569	0.007809	3641126
SHA3	-2.34616	0.017005	3644580
LPI9	-2.3524	0.005425	3637060
FTH2	-2.35334	0.0179	3640401
C1_14190C_A	-2.35406	0.013845	3643300
C2_01310W_A	-2.35825	0.01314	3638788
C1_10230C_A	-2.36481	0.004679	3634880
CR_01440C_A	-2.36679	0.046155	30515374
C2_01190C_A	-2.37319	0.036316	3638859
RTG3	-2.37325	0.003179	3644423
CR_04350C_A	-2.37412	0.003662	3641001
GAP4	-2.37805	0.003272	3644463
C6_01340C_A	-2.37839	0.026664	30515331
C2_10600W_A	-2.38024	0.012397	3641245
C1_03310W_A	-2.38498	0.002896	3636856
C3_03370C_A	-2.38727	0.001482	3638366
C1_01220C_A	-2.38737	0.033408	3640210
SVF1	-2.38969	0.00225	3639289
C5_03440W_A	-2.39013	0.041916	30515310
C1_09130W_A	-2.39313	0.001121	3634943
MLS1	-2.40402	0.007537	3634875
C2_03500W_A	-2.41337	0.001008	3637345
CNT	-2.42223	0.002722	3644071
IRE1	-2.42241	0.000624	3640726
PRB1	-2.43497	0.001819	3643521
ZCF9	-2.43667	0.006838	3638869
CR_08420W_A	-2.43763	0.001255	3642117
CR_05910W_A	-2.43968	0.016661	3646680
C3_00570C_A	-2.44146	0.004011	3640584
TOP1	-2.44779	0.002889	3644034
C3_04720C_A	-2.44834	0.009985	3635249

CDR11	-2.45367	0.001964	3647389
UTP15	-2.45422	0.013124	3643906
C1_01140C_A	-2.45497	0.002584	3640202
C3_07660W_A	-2.45703	0.016651	3638462
POP2	-2.45831	0.003713	3640301
CR_09920W_A	-2.46226	0.028491	3639028
C2_02960C_A	-2.47165	0.004318	3643011
C5_05000C_A	-2.47279	0.004906	3636539
C3_04920C_A	-2.47449	0.000272	3635268
BPH1	-2.47541	0.006918	3639547
PSP1	-2.4777	0.000884	3644551
FAB1	-2.47854	0.000338	3635766
CR_09610C_A	-2.484	0.004888	3647094
MSN4	-2.48455	0.000971	3634911
C1_06660W_A	-2.48474	0.001594	3639542
CAG1	-2.48569	0.042553	3636469
DAC1	-2.48602	0.0025	3645967
C4_04010W_A	-2.48845	0.047982	3641218
C1_04220C_A	-2.49046	0.017891	3644721
C7_00200W_A	-2.49525	0.004	3637968
HMT1	-2.49662	0.018565	3640182
CEK2	-2.50038	0.037994	3642459
C2_01820C_A	-2.50528	0.019462	3635831
MSS51	-2.51083	0.002053	3639031
C4_03850W_A	-2.5116	0.00169	3647853
CKS1	-2.51193	0.005777	3647732
CR_03910C_A	-2.51219	0.002959	3640954
DOT4	-2.51267	0.000273	3646810
C2_07640W_A	-2.51405	0.000356	3645192
C3_03300C_A	-2.51461	0.001793	3638361
C4_04350W_A	-2.51782	0.008565	3646852
NMA111	-2.51889	0.000726	3640179
NOP1	-2.51949	0.004487	3642947
C5_03960W_A	-2.52177	0.001594	3647444
C2_04850C_A	-2.52195	0.002136	3648114
C1_10120C_A	-2.52233	0.000182	3634779
CR_09740W_A	-2.53231	0.002569	3639008
CR_05640C_A	-2.53512	0.036741	3646690
GAP5	-2.53649	0.000716	3646954
MNN4-4	-2.53656	0.016129	3639250
PLB3	-2.5373	0.002017	3647089
C6_01450C_A	-2.53834	0.027904	3641624
TCC1	-2.54075	0.00094	3638524
C1_09650W_A	-2.54449	0.000841	3634870
SST2	-2.55103	0.030604	3637754
PNC1	-2.55209	0.004113	3646232
C4_01960C_A	-2.55413	0.001178	3635664

C3_00500C_A	-2.55533	0.008654	3640515
NBP2	-2.5555	0.003948	3636963
C2_02770W_A	-2.55943	0.000162	3643031
CR_08040W_A	-2.56299	0.000774	3644135
SHY1	-2.5635	0.000976	3634824
NMD5	-2.56836	0.001344	3645745
C1_00520W_A	-2.56923	0.007263	3639310
CDC34	-2.57316	0.002226	3637028
C1_11290W_A	-2.5779	0.01042	3644553
C2_06090W_A	-2.5804	0.007777	3644097
C3_07680W_A	-2.58099	0.001884	3638459
CCC2	-2.58158	0.000849	3637821
CR_10400W_A	-2.58159	0.00292	3638956
PAM18	-2.58431	0.003841	3645747
RAT1	-2.5855	0.001482	3635686
BRG1	-2.58765	0.005781	3647664
C6_00290W_A	-2.59301	0.008222	3646069
C5_03510C_A	-2.59601	0.002889	3646969
MNN14	-2.60094	0.001603	3643651
UBC8	-2.60651	0.002058	3645433
C4_02700W_A	-2.60686	0.0155	3637859
C6_03540W_A	-2.60781	0.024992	3640244
SPT5	-2.60792	0.001108	3635847
SOK1	-2.6109	0.002547	3642495
C4_02570C_A	-2.61354	0.014322	3637916
NCR1	-2.61545	0.000393	3643314
CR_06450W_A	-2.61568	0.019482	3645279
C4_06990W_A	-2.61784	0.010613	3642968
PRP45	-2.61786	0.038815	3639166
C3_06820C_A	-2.62188	0.00015	3638544
C6_01620W_A	-2.62842	0.004179	3641647
C5_01560C_A	-2.63106	0.017767	3647064
C1_05360C_A	-2.64751	0.000501	3642482
C5_03640W_A	-2.65064	0.002565	3646984
FET34	-2.65196	0.002208	3647131
NUP49	-2.65462	0.000666	3636173
C2_03000C_A	-2.65617	0.006965	3643022
CR_07480W_A	-2.65927	0.000702	3646047
CR_05540C_A	-2.66017	0.000433	3647594
C1_02850W_A	-2.66285	0.000357	3636862
C1_14580C_A	-2.66295	0.000511	3643290
C2_02120W_A	-2.66603	0.001757	3635746
CR_06010W_A	-2.67153	0.029191	3644870
YTH1	-2.67288	0.003988	3645572
ISU1	-2.67434	0.001131	3637007
C6_01980C_A	-2.67633	0.003592	3644560
CR_08330W_A	-2.67827	0.009692	3642080

C3_07290W_A	-2.68372	0.000311	3638430
CR_02890C_A	-2.68647	0.002955	3642585
C7_01280C_A	-2.68823	0.014763	3645909
C1_01070C_A	-2.68925	0.03524	3640172
SSY5	-2.68968	0.000176	3642140
C4_03000C_A	-2.70181	0.001966	3637935
ARG81	-2.70289	0.001013	3634775
C6_02930W_A	-2.70688	0.000396	3639243
MSN5	-2.7074	0.002665	3637889
C6_00700C_A	-2.70946	0.001741	3647074
MRPL27	-2.70952	0.004912	3636884
C1_13430C_A	-2.70981	0.046233	3636223
SPT14	-2.7129	0.000466	3641019
CET1	-2.71607	6.64E-05	3640140
GPM2	-2.71749	0.003548	3644713
C3_05140C_A	-2.72358	0.010724	3635161
VPS28	-2.72405	0.003128	3645794
OFD1	-2.7294	0.000318	3646962
NAG1	-2.7306	0.002794	3645966
HNM1	-2.7333	0.002526	3638857
OYE2	-2.73679	0.002434	3641603
C2_01070W_A	-2.73751	0.002767	3638834
C2_07020C_A	-2.73758	0.014698	3643497
C1_06540C_A	-2.73981	0.00134	3639520
C2_05980C_A	-2.74053	0.018104	3644075
C1_13560W_A	-2.74082	0.012612	3636174
DRE2	-2.74729	0.000482	3642571
DYN1	-2.75185	4.3E-05	3635236
HST2	-2.75368	0.008375	3640162
C1_09850C_A	-2.7541	0.000227	3634849
TOR1	-2.75526	0.000426	3643480
CR_10420W_A	-2.75592	0.001062	3638974
C2_07920W_A	-2.76079	0.025776	3642294
C3_05430W_A	-2.76419	0.008281	3643667
AUR1	-2.76493	0.00067	3642617
RIT1	-2.76716	0.01737	3638492
C6_02350C_A	-2.76858	0.002905	3645881
C6_00640C_A	-2.7738	0.011139	3647134
C2_10050W_A	-2.78306	0.000452	3643949
CR_05550C_A	-2.78567	0.000741	3647593
PDE1	-2.79259	0.005452	3637713
C1_14480W_A	-2.79695	0.006326	3643328
CR_02960W_A	-2.80152	0.041053	3642591
CR_10800C_A	-2.80169	0.000115	3638948
NOT5	-2.80271	0.000167	3640763
C1_07810C_A	-2.80366	0.016038	3640708
C4_04660C_A	-2.81234	0.000204	3641150

MSU1	-2.81676	0.019955	3643919
C5_00570W_A	-2.81954	0.000168	3641589
C7_03500W_A	-2.8198	0.00147	3646246
C7_03970C_A	-2.82397	0.001157	3643117
C6_03220W_A	-2.82732	0.022875	3639179
C1_03750W_A	-2.82734	0.004699	3644690
CR_07830C_A	-2.83569	0.00022	3647345
C3_00940W_A	-2.83685	4.85E-05	3639851
C2_02540W_A	-2.83778	0.000771	3635820
C1_07960W_A	-2.84744	0.040458	3640725
CR_09460C_A	-2.85322	0.000132	30515453
C1_05650W_A	-2.85894	0.008537	3639838
C2_00810C_A	-2.85935	0.02012	3638810
FET3	-2.86001	0.00675	3647153
C1_06970C_A	-2.86153	0.002072	3639567
IFM1	-2.86212	0.000789	3641389
CR_06650C_A	-2.86551	0.030294	3646620
MRPL40	-2.87014	0.000107	3640968
PTP1	-2.87254	0.001292	3642093
C6_02980C_A	-2.87928	1.67E-05	3639192
ZCF27	-2.88703	0.001255	3635631
DBP2	-2.89321	0.004391	3647692
C1_05250W_A	-2.89798	0.042817	3642462
C2_01370C_A	-2.90139	0.000284	3635723
CR_06230W_A	-2.90208	0.005029	3644867
C7_01940C_A	-2.90584	0.000229	3636971
C7_02550C_A	-2.91475	0.003137	3637018
PWP2	-2.91571	0.000637	3640416
C2_02110C_A	-2.91681	0.021209	3635745
CCS1	-2.91993	2.81E-05	3644476
MAS1	-2.91999	0.001315	3636855
EFG1	-2.9207	2.05E-05	3644145
CR_02270C_A	-2.92521	0.019137	3644911
MRPL37	-2.92748	0.015852	3644782
C1_04820C_A	-2.93409	0.008252	3644787
C2_07250C_A	-2.9385	0.039552	3643487
C3_02030W_A	-2.93939	0.0016	3636580
VPS13	-2.94097	0.000146	3646528
NEP1	-2.94577	0.00447	3644545
PES1	-2.94666	0.00056	3644105
C4_01500W_A	-2.94723	0.004151	3635617
BOI2	-2.94835	0.000175	3640404
HAL22	-2.94878	0.019885	3644051
C2_02420C_A	-2.9502	0.002797	3635756
ARG4	-2.95494	0.007965	3646237
MRPL36	-2.95864	0.001707	3643733
VPS21	-2.95899	7.17E-05	3642092

MET4	-2.96354	0.000947	3641219
CR_10280W_A	-2.96524	0.01031	3638934
C1_11900C_A	-2.96812	0.004342	3642005
C3_02420C_A	-2.9688	0.005437	3638342
C7_02110W_A	-2.97001	3.31E-05	3637022
C1_14250C_A	-2.97533	0.000578	3643307
C5_02280C_A	-2.97634	0.000803	3637712
ZPR1	-2.98046	0.000289	3640177
C1_02210W_A	-2.9861	0.000316	3644594
EXM2	-2.98802	0.002918	3642929
C2_07000W_A	-2.9885	0.003447	3643534
C2_05270W_A	-2.99257	0.000106	3643470
PTC5	-2.99341	0.00041	3642072
C3_07370W_A	-2.99834	0.000174	3638474
YCF1	-3.00733	0.00015	3636934
C2_00190C_A	-3.01684	0.000508	3638904
REG1	-3.01765	0.000135	3638858
FMT1	-3.02907	0.000908	3646530
C3_07430W_A	-3.03116	0.000295	3638484
CR_05440W_A	-3.03134	0.001075	3648245
GCA1	-3.0326	0.000724	3634903
C3_05790C_A	-3.03536	0.012843	3641753
CR_03120W_A	-3.03763	3.38E-05	3645123
C2_02920W_A	-3.04288	0.000276	3643016
NOP15	-3.04371	0.004366	3637966
C2_10170C_A	-3.04851	0.000146	3643931
CR_00430C_A	-3.04909	0.018762	30515366
C4_05650W_A	-3.05262	0.007611	3641814
UTP21	-3.05587	0.000706	3635757
RCA1	-3.06314	7.49E-05	3639303
C4_01280C_A	-3.06761	0.00296	3635700
MLH3	-3.07042	0.000521	3643294
REP1	-3.07202	0.025849	3639708
C3_02890C_A	-3.07526	0.032428	3638336
C2_06510W_A	-3.07639	0.000989	3646570
C2_07060W_A	-3.08233	0.001346	3643500
NOP5	-3.08587	0.001135	3646084
ATG15	-3.08649	1.38E-05	3641641
C4_01420W_A	-3.08716	0.000432	3635626
PGA55	-3.09433	0.000316	3645784
C5_03700C_A	-3.10371	0.004568	3646990
C2_06800C_A	-3.1052	0.002066	3643518
TRY2	-3.10671	0.001554	3647670
ULP2	-3.11037	0.006734	3643196
FCA1	-3.11593	0.000482	30515327
PSO2	-3.11632	0.012344	3641241
ALR1	-3.12237	0.000103	3636616

C7_02460C_A	-3.12542	0.000162	3636946
RTG1	-3.12825	0.000415	3634929
RMT2	-3.13422	0.002417	3641569
C7_01210C_A	-3.13558	0.000683	3645904
PHO86	-3.13755	0.002032	3642323
TCO89	-3.13879	0.000122	3644788
C2_10790C_A	-3.14976	0.000392	3641242
TUS1	-3.15282	3.83E-05	3646144
SLM2	-3.15457	0.000162	3647559
C7_01170C_A	-3.155	0.004992	3645914
CDR1	-3.15508	0.000797	3635237
STF2	-3.15649	0.00148	30515083
SDS24	-3.15689	0.000654	3640756
ZCF28	-3.15804	0.00305	3634776
CR_03410W_A	-3.16041	0.002352	3645136
C1_02380C_A	-3.16229	0.046799	3636790
MHP1	-3.16311	0.000143	3642460
RIM21	-3.17508	8.67E-05	3643739
RAD1	-3.17598	7.65E-05	3641192
CR_08190W_A	-3.17803	0.04488	3642084
C6_01550C_A	-3.18627	0.004479	3641653
C4_07100C_A	-3.18653	3.2E-05	3642903
C4_00490W_A	-3.19221	0.000315	3645013
C4_03190W_A	-3.19271	0.000921	3637838
CR_02970C_A	-3.19866	2.01E-06	3642603
CR_04170W_A	-3.20698	0.000921	3640977
SSU72	-3.20728	0.000368	3645129
C2_04820W_A	-3.2096	0.002729	3647776
DFG16	-3.21613	5.75E-05	3637413
DAL81	-3.22294	0.001559	3640419
STP2	-3.22489	9.59E-05	3636214
C1_01580W_A	-3.226	0.000321	3640222
CR_03260W_A	-3.23109	0.000134	30515389
C5_02010C_A	-3.2358	0.000158	3643772
CKA2	-3.24036	0.000248	3643444
NBN1	-3.24372	0.00027	3637410
TRM12	-3.2475	0.005736	3641738
C4_04460C_A	-3.25102	1.51E-05	3641127
C3_03110W_A	-3.2519	0.014905	3638400
MIT1	-3.25397	2.02E-06	3644089
CHT1	-3.25446	0.015512	3639704
CR_07670W_A	-3.25823	0.000989	3647255
C7_03380W_A	-3.25931	0.00332	30515359
C3_04620C_A	-3.26686	0.002488	3635155
C4_05010W_A	-3.26889	0.019141	3641148
BUL4	-3.26916	9.85E-05	3642037
C2_05080C_A	-3.2763	0.028824	3643453

C2_05400W_A	-3.27919	0.038295	3643435
MED3	-3.2813	0.007609	3646522
FGR13	-3.28196	0.004796	3648078
PCL5	-3.28527	1.69E-05	3636466
C4_07060W_A	-3.28624	1.33E-06	3642908
SPO7	-3.29722	0.000181	3645576
RRP6	-3.2977	0.000363	3647535
C2_02280W_A	-3.29784	5.12E-05	3635806
C3_05380W_A	-3.30398	0.014952	3643679
C6_00170C_A	-3.30769	5.52E-05	3647903
DUS4	-3.31066	0.000214	3641580
DCK2	-3.3149	0.00081	3637384
C4_06080W_A	-3.31572	0.049228	30515280
CR_01780W_A	-3.31706	0.01736	3640157
C2_03110W_A	-3.32821	0.04527	3642995
HOS3	-3.32909	2.8E-05	3637886
C6_03290W_A	-3.32964	6.17E-05	3639195
CR_06570C_A	-3.33395	0.002279	3648366
C4_06210C_A	-3.33559	0.00016	3642815
C2_10540W_A	-3.33772	4.65E-05	3641200
PWP1	-3.33915	0.00039	3635623
C7_01370W_A	-3.34192	0.021971	3645901
HNM3	-3.34779	0.012038	3640093
CR_03940W_A	-3.35787	0.001935	3648378
PRS5	-3.36263	1.83E-05	3642296
FZO1	-3.36829	8.89E-07	3646848
C1_11080W_A	-3.36842	2.66E-05	3644416
NPR2	-3.36887	0.000784	3638360
C1_13880C_A	-3.36946	9.9E-05	3636133
C2_10740C_A	-3.37397	1.5E-05	3641236
HRR25	-3.37423	1.21E-05	3645878
C5_04530W_A	-3.3754	0.000236	3636507
NAB3	-3.37794	4.18E-05	3639209
C7_02120C_A	-3.37902	0.013669	3637019
AQY1	-3.38151	0.004218	3642587
C3_03670W_A	-3.38337	3.28E-05	3645649
C4_01970W_A	-3.38604	0.000106	3635663
C4_06620C_A	-3.39862	0.006104	3642813
C3_06560W_A	-3.39903	1.77E-05	3642276
C4_04560C_A	-3.40062	6.57E-07	3641105
C1_11670W_A	-3.40576	2.54E-05	3645338
YAH1	-3.40818	0.001125	3638368
C5_00780C_A	-3.40937	0.000365	3644124
DUO1	-3.40958	0.031785	3646858
C1_09840C_A	-3.41001	8.32E-06	3634848
C7_01660C_A	-3.41472	0.000171	3636983
C2_07800W_A	-3.41662	0.015515	3642325

RIX7	-3.42439	3.98E-05	3637735
HRQ2	-3.43177	0.001865	3647850
C3_01130C_A	-3.43836	8.63E-05	3639901
C3_02180C_A	-3.4436	0.000127	3636643
PDX3	-3.44545	6.22E-05	3640949
C1_02960C_A	-3.45074	2.13E-05	3636834
SSK1	-3.45103	2.96E-05	3636184
AUT7	-3.45106	0.000124	30514999
PNG2	-3.45229	2.9E-05	3645568
C3_07800C_A	-3.46104	0.005819	3647461
C1_14380C_A	-3.46599	0.000307	3643285
C2_02820C_A	-3.4673	0.000173	3643027
C4_02520C_A	-3.46863	4.61E-05	3637863
UGT51C1	-3.47187	1.56E-05	3640147
C3_05250C_A	-3.47284	0.000137	3635199
C3_05460W_A	-3.47396	0.001406	3643670
C5_04290C_A	-3.48567	2.79E-05	3636455
CR_04240C_A	-3.48647	0.000204	3640989
C3_06760W_A	-3.48673	0.002773	3638502
C6_04300W_A	-3.48707	0.000309	3646286
C7_03210W_A	-3.48981	0.004799	3641371
CR_07940W_A	-3.49144	0.002304	3644139
C3_06420C_A	-3.49533	7.34E-05	3642262
C1_06820W_A	-3.50298	2.99E-05	3639493
C5_00800C_A	-3.51708	7.46E-07	3644126
CUP2	-3.52377	4.06E-05	3636189
CR_10750C_A	-3.531	0.003416	3638942
C1_01890C_A	-3.53343	1.87E-05	3645427
C1_08700W_A	-3.53492	1.94E-05	3634936
C1_11580W_A	-3.5356	1.48E-05	3645330
C5_03740W_A	-3.53676	0.001485	3647237
CR_08940W_A	-3.5383	0.000255	3641914
YME1	-3.5395	1.21E-05	3641816
RPA135	-3.54165	8.4E-05	3637996
CR_10060W_A	-3.54383	2.49E-05	3639035
IMP4	-3.54486	0.000762	3644138
TOK1	-3.54596	2.63E-05	3645754
C1_07950C_A	-3.54788	0.008408	3640724
C1_03140W_A	-3.54882	5.16E-05	3636929
CR_06060W_A	-3.55158	0.001528	6335358
C2_03570C_A	-3.5532	0.007372	3637336
C2_09000C_A	-3.56061	0.031765	3645782
MNN4	-3.56235	1.21E-05	3642784
SBA1	-3.57479	7.09E-06	3640305
C3_05900W_A	-3.57723	0.000119	3641739
C3_02900W_A	-3.57779	0.001373	3638337
ECM17	-3.57821	0.006882	3644077

C1_09390W_A	-3.59193	0.002219	3634851
C3_03000W_A	-3.59288	0.026697	3638389
ARG5,6	-3.59312	0.000288	3634830
RRP42	-3.59326	0.005025	3647852
C7_01150W_A	-3.59511	0.001625	3645913
C1_07130C_A	-3.59985	5.8E-05	3644462
C4_06850C_A	-3.59989	1.54E-05	3642912
C5_03410C_A	-3.59993	0.000969	3646347
C1_01630W_A	-3.60235	2.05E-05	3640194
GPD1	-3.60423	0.000597	3643924
PET127	-3.61239	7.89E-05	3644417
SNT1	-3.62102	1.92E-05	3642033
ADA2	-3.63829	6.3E-05	3644450
C7_01070C_A	-3.64275	1.46E-05	3637949
C2_04280W_A	-3.64335	0.001809	3637326
HSK3	-3.64351	0.003529	3635730
EHT1	-3.64426	0.000182	3636894
DAL5	-3.65005	0.006604	3638401
CR_03660C_A	-3.65445	0.000611	3643219
KNS1	-3.66471	8.55E-05	3636228
C6_00980C_A	-3.66703	0.000123	3644046
C7_00160C_A	-3.67141	5.95E-05	3637972
ATO10	-3.67708	0.03437	3640394
PFK26	-3.68104	5.26E-05	3634912
C1_00270W_A	-3.68301	1.93E-07	3639300
C1_00860W_A	-3.68601	0.000847	3639358
C6_04050W_A	-3.69094	0.000608	3647830
IFM3	-3.69723	0.003997	3642335
CR_08500W_A	-3.69773	0.000891	3642136
CR_06290C_A	-3.69874	3.39E-05	3644856
TPO4	-3.70296	3.52E-05	3640956
C1_10880W_A	-3.70388	0.000338	3644449
SOD6	-3.71412	0.000813	3638899
TRY4	-3.71588	0.035907	3635240
C7_01490W_A	-3.72369	0.000423	3637051
C7_03400C_A	-3.73171	8.17E-07	3646717
C6_02650C_A	-3.73409	0.000687	3639223
CRP1	-3.73728	3.22E-05	3634826
ZCF29	-3.73786	6.32E-06	3641370
MET10	-3.74192	1.17E-05	3644088
UTP13	-3.74298	0.000338	3637718
C2_01660C_A	-3.74508	3.2E-05	3635742
CEM1	-3.74571	0.008119	3635242
C6_03390W_A	-3.74845	0.000655	3639232
YTM1	-3.75106	9.65E-05	3634765
C2_04770W_A	-3.75207	0.003836	3647783
YPT53	-3.75654	5.5E-05	3642483

LEU3	-3.75708	9.53E-06	3637757
RPA190	-3.76538	2.26E-06	3647677
TYE7	-3.77144	0.002068	3636203
C4_03020W_A	-3.77999	8.74E-07	3637934
C3_05860C_A	-3.78135	0.000506	3641759
C7_02930C_A	-3.78411	3.17E-05	3641390
RLP24	-3.79086	0.000211	3645748
UTP8	-3.79891	1.91E-05	3640547
C1_13330C_A	-3.80102	3.59E-05	3636150
CTR2	-3.80688	1.63E-05	3648427
C2_00540W_A	-3.81089	1.79E-06	3638865
MLP1	-3.82043	2.76E-07	3635688
C2_08850C_A	-3.82526	6.12E-06	3643890
ZCF35	-3.83601	0.000786	3641740
C7_01030C_A	-3.83767	1.36E-05	3638019
GNP3	-3.84033	0.002646	3639027
C2_03550C_A	-3.84758	1.88E-06	3637338
C1_05270C_A	-3.84796	4.04E-05	3642476
C7_01360C_A	-3.85345	0.021148	3645900
NOC4	-3.85767	0.000327	3645224
RXT3	-3.8588	3.74E-06	3643431
C5_01610W_A	-3.85902	0.008159	3647251
C1_14080W_A	-3.86317	3.11E-05	3643345
PUS7	-3.86574	8.04E-05	3643960
PDE2	-3.87345	2.77E-08	3636877
C1_00160C_A	-3.87621	5.2E-05	3639341
CR_10040W_A	-3.87834	0.001044	3639034
C4_02850W_A	-3.88395	1.88E-07	3637910
ELP3	-3.88519	3.36E-06	3641747
C5_03460C_A	-3.88551	0.00496	3646345
RPF1	-3.88638	0.000424	3637890
OYE32	-3.89384	3.61E-06	3642919
CAN1	-3.89413	0.00042	3644035
CR_06330C_A	-3.89685	0.003691	3644846
C3_01680C_A	-3.90175	0.003205	3636605
C2_00090W_A	-3.90564	0.003667	3647910
RAD3	-3.9158	3.9E-06	3638014
C4_00460C_A	-3.9181	1.3E-06	3645010
CR_00460C_A	-3.91901	0.000897	3639726
TAZ1	-3.92032	0.00047	3644108
C1_01750W_A	-3.92051	0.01581	3645413
C6_04510C_A	-3.9213	2.35E-05	3645955
C7_00590W_A	-3.9225	2.93E-05	3637994
RCL1	-3.92606	3.15E-05	3645199
UGA3	-3.93135	2.31E-06	3638926
C4_00750C_A	-3.9334	0.005377	3645763
HCH1	-3.94591	0.004425	3641626

C2_04520C_A	-3.94749	0.014759	3645447
C6_03000C_A	-3.94887	0.022899	3639194
C1_04720W_A	-3.96917	0.000392	30514988
SPB1	-3.97736	2.95E-05	3648179
RPA34	-3.99767	5.23E-05	3634883
DBP3	-3.99903	0.000124	3634868
C4_00260W_A	-4.00349	1.23E-07	3645037
COX17	-4.00358	0.004604	30515088
UPC2	-4.00778	9.95E-06	3646489
KRR1	-4.0133	3.5E-05	3644542
ELF1	-4.01655	2.08E-07	3641903
CR_02100C_A	-4.02125	0.00016	3640141
C1_10440W_A	-4.02218	1.27E-05	3647180
CR_05150W_A	-4.02252	0.012403	30515403
CR_04160C_A	-4.03465	9.52E-05	3640976
PDR6	-4.03555	2.41E-06	3638824
C2_09790W_A	-4.04252	0.000208	3643804
C5_02680W_A	-4.06016	6.59E-07	3637774
NOC2	-4.06118	2.16E-05	3647596
ZCF30	-4.0709	2.6E-06	3642051
PUF3	-4.0724	0.000133	3646950
C6_04180W_A	-4.07543	3.62E-07	3648176
MSS116	-4.0791	3.92E-05	3634792
BUD5	-4.08287	6.77E-06	3647315
SPO11	-4.08484	0.01304	3643873
CR_03710C_A	-4.08718	1.34E-06	3643224
C2_03690C_A	-4.10225	0.000298	3637376
IML1	-4.10641	6.15E-07	3636813
C1_05790W_A	-4.10836	0.00255	30515002
BMT9	-4.1388	4.84E-06	3635654
C5_04910W_A	-4.146	0.005108	3636417
C6_03590C_A	-4.16086	0.002355	3640251
CR_03250C_A	-4.16779	8.21E-07	3645102
MTLA2	-4.17481	0.04499	3643766
C4_04510W_A	-4.20407	2.44E-06	3641120
C5_04170W_A	-4.20533	4.64E-07	3636543
URA7	-4.20824	7.11E-08	3636510
C6_00360C_A	-4.20878	0.000699	3646083
C1_06840C_A	-4.21718	1.76E-07	30515012
NOG1	-4.23265	4.16E-05	3641736
RAS2	-4.24752	2.88E-06	3635211
C7_03590C_A	-4.25322	7.96E-06	3646239
C4_02690W_A	-4.25856	8.06E-07	3637861
C1_08610C_A	-4.25879	0.001099	3646512
C3_06240C_A	-4.26229	0.026397	30515213
C1_11880W_A	-4.27516	0.001148	3642007
RPO41	-4.27759	1.59E-07	3639378

FAL1	-4.27811	1.39E-05	3639839
CR_03160W_A	-4.28018	0.000356	3645117
C2_02570W_A	-4.2815	0.001371	3635858
C4_02260C_A	-4.28158	0.002168	3635690
RPF2	-4.28816	0.000836	3643467
CR_02510W_A	-4.29192	6.6E-08	3647694
C2_02630W_A	-4.29504	0.000353	3635839
C1_13390W_A	-4.30636	0.000179	3636218
C7_02160W_A	-4.32351	0.000254	3636994
C1_08890C_A	-4.32738	0.011443	3634924
C3_00030C_A	-4.32787	3.8E-05	3640571
DEF1	-4.32938	1.8E-05	3639023
CR_08680C_A	-4.33019	7.47E-08	3642127
C3_05450C_A	-4.33468	5.94E-06	3643669
HPT1	-4.34149	1.01E-06	3643034
CIP1	-4.3474	0.002127	3644018
C1_11510C_A	-4.36975	2.32E-05	3645336
C3_06270C_A	-4.37063	8.97E-05	3642239
CEF1	-4.3803	1.76E-06	3634808
C7_01020C_A	-4.38828	3.29E-05	3638020
SUR2	-4.39857	1.55E-05	3642973
ROA1	-4.40303	7.27E-07	3645424
C1_10080W_A	-4.40337	0.000817	3634814
SUV3	-4.41035	0.000122	3645451
C7_01400C_A	-4.41259	0.000129	3645893
RIM101	-4.41554	1.8E-05	3643280
CR_10430C_A	-4.41808	0.010896	3638975
C1_07560W_A	-4.42422	0.000136	3646676
C1_06440C_A	-4.4295	2.83E-05	3639552
CSI2	-4.43918	0.005121	3642016
TRY3	-4.45181	3.27E-05	3642652
GZF3	-4.45206	5.88E-07	3642563
C2_01540W_A	-4.46245	6.26E-05	3635804
C2_09880C_A	-4.48003	2.2E-06	3643779
UTP20	-4.48153	6.47E-05	3647956
ZCF3	-4.48344	0.000122	3645335
C6_03460W_A	-4.49694	2.88E-09	3640252
C3_05850W_A	-4.49858	0.000105	3641758
C1_10250C_A	-4.50392	0.005427	3634882
C3_06700C_A	-4.50615	1.68E-06	3642247
MTG1	-4.51593	0.000626	3645139
C5_00100C_A	-4.52018	1.91E-07	3647883
DRS1	-4.52256	3.24E-05	3639045
C1_08770W_A	-4.52609	8.58E-05	3634788
HGT19	-4.5277	4.1E-08	3640558
C3_03740W_A	-4.53602	0.014506	3645640
IFH1	-4.53979	7.45E-09	3637770

CIC1	-4.54511	5.05E-05	3644059
BUB3	-4.55221	3.32E-08	3646362
PUT2	-4.55655	2.01E-05	3636439
C1_11680C_A	-4.56191	0.002364	30515056
CR_06840W_A	-4.56754	2.35E-06	3647322
PRP39	-4.57121	0.002777	3635832
C2_05810W_A	-4.57632	2.34E-07	3647481
CR_03200C_A	-4.57877	1.17E-06	3645131
NDT80	-4.58601	9.32E-08	3638840
HAP2	-4.60069	5.28E-07	3641800
LAS1	-4.61154	1.14E-06	3644073
PDR16	-4.61508	0.00038	3644679
SSF1	-4.62479	8.89E-07	3647085
C3_05160C_A	-4.63253	1.9E-06	3635229
YVH1	-4.63926	0.001084	3643218
RSN1	-4.64145	3.69E-06	3638308
C4_03830W_A	-4.65145	1.61E-05	3647851
C3_00620C_A	-4.67078	1.13E-06	30515171
OPT7	-4.67608	0.002747	3645008
C2_00280C_A	-4.6833	0.000119	3638802
C2_05820W_A	-4.68408	1.76E-06	3647482
C1_12570C_A	-4.68657	1.16E-06	3647604
MTG2	-4.70203	3.73E-05	3646693
SUA71	-4.70251	7.6E-08	3636879
C1_11620W_A	-4.71062	0.008458	3645344
C2_02270C_A	-4.71499	2.59E-05	3635805
C2_05640W_A	-4.71541	7.4E-08	3645566
C4_05230C_A	-4.74678	2.7E-05	3646156
C4_03740W_A	-4.76711	1.73E-05	3645357
C6_02290C_A	-4.76731	1.52E-06	3645873
CR_08410W_A	-4.77097	4.77E-08	3642116
HAS1	-4.80055	7.6E-07	3636443
C2_09980W_A	-4.80576	1.3E-07	3643965
STB3	-4.80722	8.6E-09	3645781
C4_04810C_A	-4.80752	6.01E-06	3641112
C2_08180C_A	-4.80845	2.31E-05	3642317
C1_14350W_A	-4.80904	2.42E-08	3643281
CPA2	-4.81353	7.3E-06	3640385
C2_02300W_A	-4.82513	4.97E-05	3635808
NMD3	-4.82678	1.81E-06	3646614
C4_00100C_A	-4.8336	1.09E-07	3645004
TRK1	-4.83565	4.2E-09	3644153
UGA1	-4.83581	0.00076	3637329
GYP7	-4.83697	1.22E-08	3646245
SEN15	-4.83914	0.000618	3646411
C1_13380W_A	-4.84395	0.000778	3636217
CR_02930W_A	-4.84423	2.44E-09	3642588

LIP1	-4.85864	0.001507	3634888
RGA2	-4.86453	6.82E-08	3635661
C1_06750W_A	-4.86747	0.002823	3639506
MPP10	-4.86772	6.64E-08	3647912
RRP9	-4.87029	1.27E-05	3642576
C5_04860C_A	-4.87402	0.004953	3636437
C4_02330C_A	-4.88452	0.00462	3637884
C5_04120C_A	-4.89099	8.46E-05	3636522
C1_05200C_A	-4.905	2.41E-06	3642466
IFF6	-4.90968	2.3E-08	3644087
C2_00650W_A	-4.9098	1E-06	3638889
ATS1	-4.9165	2.93E-05	3642079
C3_01150C_A	-4.92001	0.000103	3648401
CR_10410C_A	-4.92275	4.44E-06	3638957
C3_00100W_A	-4.92433	4.88E-05	3640567
SMM1	-4.92492	5.47E-07	3643778
SOF1	-4.92529	3.41E-08	3640585
NAR1	-4.92841	1.53E-07	3634916
C6_02020C_A	-4.93179	5.47E-05	3647563
C4_04990C_A	-4.93289	0.016644	3641166
BRE1	-4.95033	2.76E-09	3641568
C5_01020C_A	-4.95366	0.025725	30515297
RIO2	-4.95619	1.65E-06	3642066
RIM2	-4.96924	3.83E-05	3645448
C5_04080C_A	-4.97397	4.07E-07	3647731
OLE2	-4.97701	1.45E-08	3643503
C5_05240C_A	-4.98721	0.022755	3636471
FLO8	-4.99995	2.25E-10	3646292
NIP7	-5.008	0.000208	3645880
BMS1	-5.01604	4.16E-06	3639857
SIP5	-5.01685	5.06E-09	3639865
C4_06040W_A	-5.02064	0.00046	3646521
C1_03840W_A	-5.03051	0.043271	30514984
C3_01780C_A	-5.03078	0.048882	30515179
C4_06790W_A	-5.04663	7.77E-07	3642918
LEU42	-5.04686	1.44E-06	3643808
SRP40	-5.07181	7.88E-07	3642605
C3_02670W_A	-5.08471	0.000766	3638405
C5_02020C_A	-5.08612	5.19E-10	3643771
C4_01240C_A	-5.08764	1.03E-05	3635705
HGT18	-5.09597	0.000216	3645111
C5_01430C_A	-5.09778	1.25E-05	3647056
C2_08950W_A	-5.12889	2.17E-07	3645788
C7_04160W_A	-5.17147	0.000151	3643127
C3_02910W_A	-5.17738	0.000584	3638378
PUT1	-5.17828	4.06E-07	3637779
SDA1	-5.19089	1.98E-05	3646688

CR_07910C_A	-5.1972	1.59E-08	3644142
RPD3	-5.19825	9.78E-08	3642580
SPO72	-5.21268	1.12E-06	3644072
FRE10	-5.21907	1.04E-06	3646841
C3_02750W_A	-5.2316	0.002516	3638411
C1_07220W_A	-5.23582	1.23E-09	3644460
C7_04090C_A	-5.23918	1.16E-07	3643135
C1_08630W_A	-5.24194	6.65E-07	3634928
CR_10760C_A	-5.25395	0.000168	3638943
CR_03870W_A	-5.25479	0.000266	3643187
C1_12680W_A	-5.26112	3.58E-06	3646833
C1_07150W_A	-5.26738	0.003804	30515019
EAF3	-5.26827	0.000269	3648318
CR_04600W_A	-5.27024	3.15E-07	3640950
RPN4	-5.27178	8.26E-07	3644684
C6_04400W_A	-5.27257	0.002865	3645949
C1_09320C_A	-5.27267	2.81E-09	3634834
IFR1	-5.27339	0.004786	3643930
MDN1	-5.29219	1.78E-09	3635614
C6_03210C_A	-5.30077	5.23E-05	3639178
C6_02970C_A	-5.30299	5.25E-05	3639190
PGA37	-5.30598	1.98E-05	3636484
C3_07670W_A	-5.31483	9.16E-10	3638460
ITR1	-5.32064	1.76E-07	3643440
C2_08920W_A	-5.32961	8.82E-10	3645797
POP4	-5.33969	4.57E-05	3639241
HBR1	-5.34142	4.99E-06	3639295
JIP5	-5.34309	1.57E-07	3634922
C1_11610C_A	-5.35095	5.13E-07	3645345
C1_05440C_A	-5.35127	2.04E-10	3642489
C1_04510W_A	-5.35261	0.000914	3646147
NAG3	-5.35959	5.43E-05	3645969
C1_01160C_A	-5.38443	3.82E-06	3640204
CHR1	-5.3947	7.6E-07	3647601
PGA48	-5.39873	8.43E-08	3647905
CBF1	-5.41551	1.65E-06	3642779
C2_06740W_A	-5.41685	5.46E-08	3648024
CR_07750C_A	-5.43553	7.4E-07	3647350
CR_03360W_A	-5.44474	2.15E-05	3645141
PAN6	-5.44623	6.35E-07	3647684
ARX1	-5.45199	4.34E-06	3636811
C2_06570C_A	-5.46534	6.83E-09	3646561
C3_02920W_A	-5.48698	9.74E-09	3638379
DAL8	-5.48878	0.000127	3635181
C6_01040C_A	-5.49669	2.7E-07	3644014
C2_01240C_A	-5.50044	0.000825	3638794
C7_02660C_A	-5.50425	0.002503	3647652

C1_08840W_A	-5.50543	0.004486	3634919
CR_02550C_A	-5.51144	2.89E-07	3647689
NOP14	-5.51458	3.23E-07	3635266
C5_05340W_A	-5.53991	3.5E-08	3636400
C4_03350C_A	-5.5429	2.72E-06	3646794
C3_00410C_A	-5.54572	0.008949	3640552
BUD22	-5.5495	3.58E-06	3640391
C1_13010W_A	-5.55347	8.55E-09	3636120
RTA3	-5.57303	7.24E-06	3646566
OPT4	-5.57647	0.000451	3647697
C1_04040C_A	-5.5785	1.25E-05	3646429
MIF2	-5.58065	9.16E-12	3639220
C3_00150W_A	-5.59198	7.8E-06	3640595
MTM1	-5.60066	4.96E-07	3642487
SPB4	-5.61175	0.000422	3647252
EPL1	-5.61716	5.33E-09	3639732
C3_01110C_A	-5.66031	0.025078	3639835
CR_06970C_A	-5.66755	1.99E-06	3645736
C6_01890C_A	-5.66952	1.47E-07	3641598
ARO80	-5.67977	6.96E-06	3636808
C7_02340C_A	-5.69505	0.000226	3636933
ENP1	-5.70006	1.74E-05	3647861
C3_07650C_A	-5.71104	0.000169	3638488
C1_09040C_A	-5.72031	3.68E-06	3634769
MUP1	-5.74086	8.92E-11	3642008
C4_05590W_A	-5.74128	2.46E-09	3641776
C3_04690C_A	-5.74719	0.002292	3635196
RNR3	-5.74883	3.12E-07	3647591
MRT4	-5.7513	1.03E-05	3639219
C1_10950C_A	-5.76279	1.42E-07	3644428
C1_09780C_A	-5.7773	4.99E-10	3634842
LIP4	-5.7807	7.36E-06	3645987
C3_02350W_A	-5.78557	3.05E-09	3636618
C1_11320C_A	-5.78858	8.72E-08	30515052
LTV1	-5.79827	1.4E-05	3639016
C1_00090W_A	-5.80884	0.000487	3639304
C2_07580W_A	-5.81066	0.000562	3645216
C6_04240W_A	-5.81245	0.000677	3646276
CR_04220C_A	-5.82696	1.98E-06	3640943
C6_03800C_A	-5.84147	5.29E-09	3640310
C5_01730W_A	-5.86121	0.000806	3643730
CR_00310C_A	-5.88413	9.22E-06	3639715
C2_04110W_A	-5.89589	0.000696	3637378
STE11	-5.90135	8.4E-14	3637369
UTP5	-5.90261	1.2E-06	3638922
UTP4	-5.91799	8.84E-07	3636657
RRN3	-5.92601	3.84E-09	3647925

FAR1	-5.92929	1.94E-06	3637970
C1_03830C_A	-5.94456	1.26E-05	3644678
MDM34	-5.95436	1.71E-09	3647517
C4_01090C_A	-5.9549	0.002301	3635685
NAN1	-5.95687	1.51E-07	3637932
OYE23	-5.97384	0.000933	3641618
C3_05440C_A	-5.97502	0.000167	3643668
SFP1	-5.99508	2.91E-10	3635225
DBP8	-6.00226	3.3E-06	3646692
FHL1	-6.00796	2.04E-07	3643493
SSU81	-6.02013	2.51E-07	3634944
VTC4	-6.02273	1.37E-08	3646795
C6_04530C_A	-6.02795	2.81E-11	3645970
C3_06370C_A	-6.03667	5.74E-09	3642258
C4_03580W_A	-6.06655	0.001911	3645369
MDJ1	-6.07872	3.66E-10	3646980
ATG9	-6.08032	1.18E-05	3635239
C3_07550C_A	-6.08481	2.76E-09	3638525
C1_09330W_A	-6.10027	0.001317	3634802
C2_05830C_A	-6.11966	8.88E-07	3647483
FGR23	-6.12346	3.54E-08	3636555
C3_02840W_A	-6.12618	0.000295	3638331
SLY41	-6.12907	7.89E-09	3647139
C5_00310C_A	-6.14198	3.86E-06	3641576
CNS1	-6.14608	1.3E-07	3639365
WSC1	-6.15319	1.15E-09	3635138
COX19	-6.18619	3.61E-07	3636220
MRP17	-6.18731	1E-08	3641548
CR_08650C_A	-6.20852	1E-12	3642124
RNR22	-6.20954	2.38E-05	3645217
ISA2	-6.21081	6.38E-09	3638513
CYC3	-6.22675	1.61E-09	3642621
C5_02440C_A	-6.24611	7.34E-06	3637728
C5_01990W_A	-6.27409	0.004263	3643774
C6_02790C_A	-6.2776	8.85E-10	3639245
MAK21	-6.30487	1.69E-10	3635147
NCS2	-6.3225	2.72E-07	3643216
ATX1	-6.33018	0.000187	30515426
CPA1	-6.34297	1.93E-08	3635715
SWE1	-6.36502	6.12E-13	3634866
HAP42	-6.37554	1.86E-11	3635729
HNT1	-6.39602	2.99E-11	3644441
MET16	-6.42483	4.23E-06	3642964
CR_01630C_A	-6.47694	0.001036	3640085
RMP1	-6.47901	0.001566	3636215
TPS2	-6.49238	5.59E-08	3636892
TBF1	-6.49305	5.48E-11	3637327

NOP13	-6.49953	6.7E-08	3638469
C4_00690C_A	-6.51313	1.31E-07	3645752
ALS5	-6.53915	2.21E-10	3640277
DAL1	-6.55188	8.82E-06	3640564
SUL2	-6.55569	0.000669	3637835
NSA2	-6.56206	9.64E-08	3642259
C5_02070C_A	-6.5664	6.71E-11	3643746
ARR3	-6.57467	2.13E-06	3642910
BAS1	-6.59476	4.55E-08	3641136
C4_04020C_A	-6.60161	0.00797	3641215
C2_06480W_A	-6.61071	2.89E-06	3646568
C6_04200C_A	-6.62873	2.09E-05	30515344
C2_07280W_A	-6.63055	0.0021	3643475
C1_07530W_A	-6.63628	0.001734	30515025
HSP104	-6.66281	1.11E-07	3642097
ABC1	-6.66634	1.88E-14	3640239
C1_09790C_A	-6.66916	1.23E-10	3634843
C5_04850W_A	-6.69347	0.000111	3636436
C1_13130C_A	-6.69603	3.62E-07	3636202
C7_00340C_A	-6.72411	1.18E-10	3638040
C2_00200W_A	-6.73251	1.77E-10	3638903
C2_04080W_A	-6.73337	2.48E-07	3637381
PRP8	-6.7476	6.45E-14	3642125
C2_04570W_A	-6.75141	1.56E-06	3645441
ISY1	-6.75416	3.35E-05	3646233
NOG2	-6.7736	7.42E-08	3640299
CR_06980W_A	-6.78139	0.000686	3645735
C1_05370C_A	-6.79067	1.29E-09	3642481
DIP2	-6.81216	9.91E-11	3640762
C2_10810W_A	-6.82238	5.3E-09	3641243
HIP1	-6.82708	1.71E-10	3643761
C3_02980C_A	-6.84719	1.1E-05	3638386
C4_03230C_A	-6.84976	0.006842	3637836
DED1	-6.86886	2.94E-10	3641752
C3_07400W_A	-6.88034	9.24E-07	3638471
C6_02230W_A	-6.89768	1.05E-10	3645866
C6_00530C_A	-6.89915	0.000106	30515326
UTP18	-6.90076	1.01E-08	3643123
C4_02740W_A	-6.91502	4.85E-10	3637855
CR_08080W_A	-6.9452	0.000431	3642094
RPA12	-6.97388	7.9E-05	3643477
GAC1	-6.98813	9.32E-09	3638025
RPC10	-6.99221	8.92E-08	3643292
C3_02020W_A	-6.99421	1.86E-08	3636592
RRP8	-6.99431	6.27E-06	3643900
KAR4	-6.99871	2.59E-05	3644937
C4_07040W_A	-6.99966	0.000567	3642963

C1_06090C_A	-7.00681	7E-12	3639822
ICL1	-7.00721	8.16E-12	3646146
C5_03630C_A	-7.03356	0.000154	3646983
ECM22	-7.10015	7.92E-11	3648000
MRF1	-7.10034	4.96E-11	3645321
CR_04920W_A	-7.11106	4.42E-10	3648207
C2_10130W_A	-7.12272	0.003127	3643935
RRP15	-7.14049	1.04E-07	3648397
RRN11	-7.14683	1.59E-07	3645276
TSR1	-7.16341	4.38E-08	3642135
C2_05160C_A	-7.19927	1.18E-09	3643461
C1_01620C_A	-7.21248	3.75E-12	3640193
C1_13250W_A	-7.23586	1.91E-14	3636143
CR_08470W_A	-7.23886	1.26E-09	3642134
NOP8	-7.24282	1.83E-07	3646290
HSX11	-7.26821	0.000346	3635660
RSM22	-7.32644	3E-11	3642506
UTP9	-7.38702	5.02E-12	3638451
MET2	-7.41692	5.73E-13	3640070
ARG1	-7.44265	1.94E-10	3639691
C6_01590W_A	-7.47163	9.5E-13	3641650
DHH1	-7.4789	3.68E-07	3639490
C3_07470W_A	-7.48452	0.02648	3638479
DBP7	-7.50389	2.83E-10	3645903
GRF10	-7.55158	5.63E-14	3636492
FUN31	-7.55201	4.37E-12	3642249
CR_00810W_A	-7.57144	1.48E-05	3640415
C2_05060C_A	-7.59772	0.00065	3643451
C4_07170C_A	-7.59973	9.76E-05	30515289
C2_09610W_A	-7.62331	2.83E-09	3643798
CR_01410C_A	-7.63714	4.48E-06	3640136
WHI3	-7.65149	3.14E-11	3636991
MET3	-7.66063	9.42E-06	3636132
MAK5	-7.67015	1.64E-08	3643454
RFX1	-7.7108	5.83E-07	3644844
C1_10970W_A	-7.71941	4.08E-09	3644427
SAS10	-7.72373	4.35E-12	3637899
CR_02030C_A	-7.76872	1.09E-08	3640152
C4_05970W_A	-7.83818	1.72E-12	3646514
C2_02580W_A	-7.84469	2.49E-08	3635860
CR_07030C_A	-7.84653	9.73E-06	3645730
C2_09500W_A	-7.85019	6.07E-09	3643817
SLD1	-7.85149	1.61E-12	3638406
FTH1	-7.85767	4.35E-09	3634852
RAD18	-7.85969	0.000397	3641637
C6_03200W_A	-7.88488	4.63E-14	3639177
C7_00330C_A	-7.90078	6.4E-09	3638042

CTA8	-7.90116	1.19E-12	3634947
C2_04120C_A	-7.90423	1.59E-06	3637377
RGS2	-7.93419	7.88E-08	3646629
C2_05840W_A	-7.94775	1.01E-08	3647484
CUP9	-7.99484	2.93E-09	3636950
C2_09160W_A	-8.01512	2.22E-06	3644090
ARG8	-8.02797	1.37E-05	3641131
PHR3	-8.05879	2.74E-17	3645003
C1_11000C_A	-8.15786	1.11E-05	3644422
ATG1	-8.16109	8.06E-15	3641175
C1_14330W_A	-8.18882	3.68E-08	3643278
FEN1	-8.21829	1.15E-08	3646829
NBP35	-8.24348	1.07E-11	3647412
COX11	-8.25985	1.14E-13	3646842
CR_03980W_A	-8.27039	5.03E-08	3640964
C3_01560W_A	-8.27182	2.62E-08	3636669
JEN2	-8.33948	3.44E-05	3641214
C1_09520C_A	-8.36705	2.25E-16	3634766
ALS6	-8.37104	3.96E-09	3642266
C3_06160C_A	-8.43561	1.01E-08	3641766
C4_05160C_A	-8.46675	5.37E-14	3647411
C5_03920C_A	-8.4865	5.47E-08	3647448
SNQ2	-8.55478	2.29E-17	3640290
C2_07410W_A	-8.55535	6.02E-06	3645203
C1_09710C_A	-8.57645	6.02E-10	3634818
C3_07580W_A	-8.60595	3.1E-07	30515225
C1_11020W_A	-8.61352	2.86E-07	3644420
ENP2	-8.64923	1.15E-09	3646234
CR_08580C_A	-8.65853	2.4E-06	3642105
C2_04700C_A	-8.67013	8.72E-05	3647794
C3_01770C_A	-8.6894	2.36E-08	3636588
CR_06550C_A	-8.71063	2.79E-11	3645260
BUD21	-8.71952	3.34E-10	3640541
C5_03800W_A	-8.72835	1.14E-11	3647230
SSY1	-8.72861	4.45E-10	3637382
ALS9	-8.76378	1.26E-10	3640281
RMS1	-8.77622	3.63E-07	3646361
C1_01510W_A	-8.8363	3.49E-14	3640229
SCH9	-8.83899	4.26E-13	3637421
NSA1	-8.8439	3.66E-06	3642313
HGT17	-8.85672	0.000272	3635687
PRP5	-8.87469	3.57E-12	3638447
C2_08700C_A	-8.93455	1.68E-14	3643887
MFG1	-8.96621	7.2E-13	3643884
C1_06760C_A	-9.0209	2.47E-08	3639505
C3_05800W_A	-9.0724	1.01E-10	3641754
C6_02150C_A	-9.07534	0.000187	3645888

C4_02770C_A	-9.10428	4.09E-07	3637903
C6_01780C_A	-9.13343	1.97E-08	3641636
CR_00750C_A	-9.15881	4.74E-09	3640438
C1_06510C_A	-9.22105	5.07E-12	3639549
ECM1	-9.23715	3.9E-06	3641224
C6_00380C_A	-9.24347	2.4E-09	3646085
HEM3	-9.24701	1.18E-11	3643940
C3_02040C_A	-9.27884	7.98E-09	3636579
KTI12	-9.29603	6.24E-07	3645140
HSP78	-9.34757	8.36E-14	3637414
C4_00810C_A	-9.36184	1.05E-06	3645742
NOP4	-9.40279	6.55E-11	3647991
ELA1	-9.4188	2.62E-09	3644680
QDR3	-9.43295	8.11E-09	3644023
C2_05750W_A	-9.49453	6.87E-08	3645577
C7_01690W_A	-9.59781	1.52E-08	3636980
OPT8	-9.60615	5.96E-09	3640266
HIT1	-9.62211	2.02E-05	3637854
PGA57	-9.62257	4.04E-05	3635608
C1_12440W_A	-9.65442	0.001993	3642012
HCA4	-9.69214	5.08E-10	3637911
CR_03670W_A	-9.71746	0.009427	3643220
C6_03280W_A	-9.71746	0.009436	3639185
ZCF2	-9.73021	3.43E-20	3642484
WAR1	-9.75776	2.19E-09	3644691
C4_02230C_A	-9.82717	0.045006	3635693
C1_06870C_A	-9.82869	0.045008	30515013
NAG4	-9.82922	0.045001	3645978
ALK6	-9.82922	0.045001	3637045
CR_04610C_A	-9.82922	0.045001	3640951
C1_05150C_A	-9.82922	0.045001	3642469
C1_09300C_A	-9.82922	0.045001	3634831
C4_07020C_A	-9.82922	0.045001	3642965
CR_09140C_A	-9.88022	4.05E-20	3641879
C2_04090W_A	-9.90417	0.02205	3637380
C2_01060C_A	-9.92345	3.44E-07	3638835
C2_08030W_A	-9.92686	4.82E-08	3642309
GCA2	-9.95286	5.85E-12	3647194
IFE2	-10.044	3.44E-12	3648027
C5_04840C_A	-10.196	1.03E-11	3636435
C1_05160C_A	-10.2174	2.24E-14	3642468
C1_04710C_A	-10.2212	4.1E-09	3644764
CR_02420W_A	-10.2724	3.8E-09	3644926
C1_12750C_A	-10.2778	1.2E-09	3646818
PTC8	-10.3207	1.67E-13	3635674
PRN1	-10.3397	1.42E-06	3639882
SEN2	-10.4031	3.56E-11	3637832

MIH1	-10.444	1.96E-15	3648188
C3_06940W_A	-10.448	1.09E-16	3638509
FET31	-10.4727	2.15E-09	3647151
C1_05330C_A	-10.5328	9.39E-12	3642485
C2_09820W_A	-10.6522	1.45E-09	3643801
C5_02980C_A	-10.6605	7.17E-08	3637816
C2_10090C_A	-10.7222	6.39E-11	3643939
SGD1	-10.7521	3.18E-13	3643191
CR_01710W_A	-10.7931	2.34E-07	3640130
ARF1	-10.8256	5.95E-18	3642130
C5_02640W_A	-10.9062	1.15E-08	3637785
C2_09660W_A	-11.0011	2.61E-13	3643793
CR_10470C_A	-11.154	2.21E-12	3638979
GST2	-11.1961	2.43E-14	3637937
GCV2	-11.2643	4.01E-11	3646505
C1_04120C_A	-11.3081	2.64E-05	3644701
C2_06660W_A	-11.41	3.6E-06	3648418
CR_02800C_A	-11.4213	3.07E-11	3642559
CR_08830W_A	-11.4516	4.41E-14	30515450
HBR3	-11.5197	2.21E-12	3645652
MAK16	-11.5998	1.73E-11	3646920
RRS1	-11.6173	3.83E-08	3639352
REI1	-11.6273	1.64E-10	3647536
TPS3	-11.6341	3.25E-17	3641205
C6_00810C_A	-11.7135	2.12E-17	3647068
ALD6	-11.7834	1.25E-09	3647407
C5_02590C_A	-11.817	2.41E-10	3637723
C6_02950C_A	-11.8331	3.29E-07	3639188
C2_00410C_A	-11.9145	4.01E-17	3638820
C1_01150C_A	-11.932	1.53E-14	3640203
CR_09800C_A	-11.9519	2.84E-16	3638984
FGR46	-11.9524	0.005539	3638989
WOR2	-12.0815	2.47E-07	3635230
C1_09210C_A	-12.2688	1.07E-17	3634897
RCH1	-12.3116	1.24E-11	3645002
C7_02610C_A	-12.3493	1.21E-11	3648285
C3_03250W_A	-12.4092	4.22E-13	3638318
C1_05930C_A	-12.4534	9.87E-15	3639862
ALP1	-12.528	2.94E-10	3644437
C3_06950W_A	-12.6831	1.11E-11	3638507
C6_03360C_A	-12.7654	4.33E-07	3639203
SRR1	-12.8339	5.55E-12	3647589
CR_01600C_A	-13.099	3.81E-08	3640080
C2_00760C_A	-13.1147	1.35E-17	3638910
C2_04670W_A	-13.1297	2.21E-10	3647789
C4_05350W_A	-13.2501	2.83E-20	3646948
NOP6	-13.4434	3.1E-07	3639507

C1_09820C_A	-13.5429	3.8E-05	3634846
C1_07650W_A	-13.5964	5.74E-10	3646657
POP3	-13.6367	7.78E-07	3638993
HSP21	-13.6432	8.16E-13	3637364
C5_04380C_A	-13.8246	1.17E-05	3636485
FGR47	-13.8578	2.77E-16	3641651
C1_07660W_A	-13.9137	8.75E-08	3646656
C1_01610C_A	-13.9882	4.62E-09	3640225
C3_05840W_A	-14.0053	0.017099	3641757
C4_07000W_A	-14.0075	0.016343	3642967
CR_06870C_A	-14.0082	0.016645	3647318
C2_08580W_A	-14.2979	1.28E-05	3643916
TRY6	-14.3001	1.48E-05	3638499
C5_01710C_A	-14.3653	2.07E-10	3643732
RIM8	-14.5532	1.64E-21	3639342
C4_04300C_A	-14.6724	4.35E-08	3646843
CLG1	-14.7784	5.02E-21	3646030
IFA14	-14.8115	4.01E-08	3639012
C6_03180C_A	-14.8627	2.54E-22	3639261
QDR2	-15.3511	1.06E-23	3643661
C7_01910C_A	-15.7878	5.21E-14	3637029
C1_11200W_A	-15.8066	4.54E-11	3644396
C6_01870C_A	-15.8291	1.46E-12	3641625
C3_01540W_A	-15.8698	5.46E-06	3636567
C1_06980C_A	-16.0912	5.3E-12	3639565
C2_08530C_A	-16.1988	6.19E-08	3643921
SUT1	-16.3318	2.86E-13	3637706
C3_04730C_A	-16.4144	0.001295	3635250
ALS7	-16.4177	7.46E-23	3642234
C3_06660C_A	-16.4196	0.001207	3642253
C1_10360C_A	-16.6332	5.81E-21	3634836
C1_02450C_A	-16.7018	7.11E-08	3636849
GCS1	-16.8061	1.67E-22	3640742
PZF1	-16.8434	7.07E-10	3648113
C7_03850W_A	-16.9359	1.25E-10	3643145
CYS3	-16.9492	4.67E-21	3642081
UTP22	-16.9777	1.75E-22	3635760
TSR2	-17.0869	5.82E-12	3636845
C1_09770W_A	-17.5023	2.07E-16	3634825
C2_02190C_A	-17.5448	1.32E-12	3635753
CIRT4B	-17.8335	1.42E-28	3642560
CR_04110W_A	-17.9139	1.06E-14	3641026
C1_01130W_A	-18.0925	8.12E-23	3640178
C3_04870W_A	-18.1827	0.007122	3635226
FGR42	-18.1827	0.007187	3647438
C5_00810C_A	-18.1865	0.006808	3644127
C1_07040C_A	-18.1875	0.008427	3639533

C7_02600C_A	-18.4893	2.84E-18	3648286
CR_01510C_A	-18.6495	0.000489	3640104
C4_04520W_A	-18.9329	2.69E-17	3641110
FLU1	-19.0948	7.26E-12	3637048
CR_06140W_A	-19.1156	3.93E-24	3644862
C3_01430W_A	-19.4882	3.29E-08	3636633
ZCF1	-20.4925	4.58E-23	3638329
FYV5	-20.9015	6.19E-08	3647057
C4_07140W_A	-20.954	7.99E-22	3642936
C3_07570C_A	-21.6621	3.14E-12	3638522
FMP45	-21.7141	1.04E-20	3637004
CTA4	-22.2396	1.09E-27	3641743
PIR1	-22.4731	6.17E-37	3645801
C1_07790C_A	-22.4804	8.34E-10	3640706
C1_12630C_A	-23.4626	1.59E-08	3646838
C4_03340C_A	-23.7237	5.23E-09	3648090
C1_06350W_A	-23.7774	6.14E-25	3639562
HSP70	-24.0175	1.09E-26	3636229
CDA2	-24.6951	1.31E-06	3639716
SSU1	-25.2395	1.5E-22	3641882
CR_04710W_A	-25.3395	0.000192	3648141
C7_01430C_A	-26.4732	1.96E-26	3636961
C6_02660C_A	-26.9956	3.19E-07	3639224
C4_06200W_A	-27.5739	5.98E-05	30515282
C6_03440W_A	-27.7338	5.51E-15	3640274
FRE9	-28.5652	3.76E-16	3643452
EBP1	-28.9544	1.52E-15	3644060
C3_03460C_A	-29.0745	1.77E-10	3638359
CR_07680C_A	-29.4791	1.99E-06	3648193
KTI11	-29.8022	5.55E-05	30515125
C3_04510W_A	-30.674	7.59E-09	3635258
C6_01400W_A	-30.7185	0.001737	3641604
CDR4	-31.22	8.9E-29	3640711
BMT1	-31.449	2.36E-30	3638535
C1_13820C_A	-31.7359	6.29E-19	3636127
UGA6	-32.0386	1.59E-05	3642975
MET1	-33.8376	2.59E-29	3643014
CR_04940W_A	-34.7971	5.19E-10	3648209
C1_02690C_A	-34.8789	0.001183	3636823
C6_03810W_A	-34.975	1.13E-16	3640286
C2_05330C_A	-35.0731	2.25E-19	3643426
C2_06520C_A	-35.1019	1.02E-08	3646571
KSP1	-35.2907	1.68E-36	3644467
C1_11410C_A	-36.5033	5.92E-06	30515054
C7_00880C_A	-36.6241	2.45E-21	3638052
C7_01680C_A	-36.6511	1.89E-27	3636981
C2_08260W_A	-38.5441	2.05E-08	3642345

CR_09930W_A	-39.0202	1.63E-26	3639029
HSP31	-39.4583	2.28E-10	3644589
C2_07070W_A	-46.5243	4.75E-25	3643501
MRV4	-47.436	0.000205	3636547
C2_06530W_A	-49.6829	1.6E-11	3646572
HWP2	-51.4561	0.000402	3646803
GIT3	-54.541	1.84E-31	3642634
CR_01530C_A	-54.9045	1.06E-13	3640072
AAH1	-61.0514	3.16E-07	3643529
MNN1	-66.1914	2.93E-18	3637784
C5_04030W_A	-66.319	1.66E-34	3647737
C1_00190C_A	-72.2399	3.06E-30	3639337
ARG11	-72.5079	1.68E-05	3635193
C5_03080C_A	-74.7483	7.85E-27	3637701
C7_03560W_A	-94.0681	6.42E-59	3646236
C7_00770W_A	-95.485	2.6E-21	3637959
CR_08880C_A	-105.999	1.28E-21	3641878
YWP1	-116.625	7.01E-64	3643915
RME1	-128.461	6.53E-38	3644453
C2_02130C_A	-141.486	2.08E-10	3635747
PHO87	-143.656	1.83E-10	3639861
C4_06910W_A	-155.421	9.66E-17	3642933
GPI1	-190.254	1.44E-24	3643680
C3_07930C_A	-214.938	0.047674	3647452
PRX1	-216.818	5.06E-36	3641357
CR_00840C_A	-238.693	0.039689	3640411
C1_06430C_A	-271.108	0.03515	3639553
CRZ2	-365.892	4.02E-64	3645725
HAK1	-498.421	0.018606	3639546
C3_06170C_A	-660.771	0.013838	3642272
HSP30	-700.86	5.58E-67	3645419
C3_00230C_A	-725.629	0.012196	3640557
CFL4	-1179.89	0.00714	3642639
C4_07260W_A	-1602.41	0.004992	3642942
C1_14630C_A	-23569.9	0.000133	3643291
TAR1	-152062	6.1E-241	30515449
	**S45 vs Control**		
Name	Fold change	P-value	GeneID
ECE1	29315.18	1.5E-09	3646814
HWP1	7729.863	8.39E-23	3645372
C1_04180W_A	4137.347	1.02E-06	3644716
HGT12	2603.884	3.99E-06	3638048
C1_13100W_A	1904.235	1.03E-05	30515073
HHT21	1507.709	7.39E-16	3644707
ECM331	1163.648	3.47E-05	3637731
SAP5	793.1459	8.23E-05	3639268

CDC11	777.0125	8.43E-05	3647888
MAL31	538.1231	1.03E-11	3636420
HYR1	502.9258	1.62E-11	3636226
CRH11	426.1513	6.49E-11	3637905
HHT2	346.5971	1.91E-24	3647326
HTA2	322.3881	6.12E-24	3644715
CDC10	285.3759	5.24E-10	3640947
ALS3	255.8446	9.16E-71	3647965
MLC1	250.4467	0.001065	30515387
TUB2	243.9226	1.16E-17	3639329
ALI1	243.5633	0.00117	3636635
CR_06380C_A	240.8575	0.001218	3648139
RBT1	216.6491	3.27E-17	3645377
PGA6	208.5507	3.75E-09	3634774
FGR6-10	206.518	0.001623	3641770
C5_02780W_A	200.0764	0.001795	3637803
COG4	197.8325	0.001804	3645571
C4_05390W_A	196.4817	0.001831	3646951
SAM51	192.3798	0.001917	3641582
C6_02140W_A	191.7853	0.001924	3645886
C6_00400C_A	191.1845	0.001937	3646087
C1_06250W_A	190.3626	0.002052	3647525
C4_02600C_A	184.8314	0.002163	3637912
HHO1	175.9418	8.37E-16	30515358
C5_03000C_A	175.8943	0.002254	3637818
C1_07490C_A	171.5018	0.002605	3646666
SHE3	170.2838	7.79E-09	3639277
VMA10	163.7544	0.002562	3645215
SOD5	156.4144	8.59E-38	3638886
TUB4	155.7778	0.002767	3641774
UME6	146.1291	2.77E-08	3647522
DPM2	144.044	0.003329	30515325
KAR3	138.6747	0.003613	3644078
TUB1	136.4792	2.89E-24	3641930
CCN1	136.4378	0.003917	3643735
MCD1	134.1259	0.004238	3639044
TNA1	133.8215	0.005584	3637827
C3_02620C_A	126.4466	0.004323	3638326
C3_06670C_A	122.1558	0.00447	3642254
C7_01100C_A	121.0943	0.004605	3637948
CR_05310W_A	120.358	0.004579	3648125
HHF1	117.3514	1.46E-27	3644705
SAP6	113.172	1.44E-07	3639229
C2_05510C_A	108.999	0.00588	3643420
DPM1	104.3424	1.45E-07	3640700
MCD4	103.2656	0.006164	3642036
POL30	102.9885	3.33E-07	3635583

PRI2	100.9091	0.006508	3642788
CDC28	97.24045	2.41E-07	3644838
C3_07840C_A	94.05979	0.007357	3647465
C1_03150C_A	93.04448	0.0078	30514976
C6_00710W_A	92.88784	0.007556	3647073
C1_02100W_A	92.83823	0.007541	3644578
C5_03430W_A	90.66547	0.008296	3646346
FGR6-3	89.83755	0.007868	3647954
DUR3	89.68482	0.01174	3644760
ERG28	88.00192	0.008161	3638833
HRT2	86.31746	6.61E-07	3635709
C4_02460W_A	84.09652	0.009719	3637844
PHR1	82.91186	3.16E-39	3641108
CR_00280C_A	82.62809	0.00914	3639719
TEM1	81.90066	0.009056	3636923
PGA31	79.07791	0.010577	3641226
C2_01420C_A	79.00154	0.010098	3635841
TOS1	78.26475	3.49E-36	3636566
CR_10130W_A	78.23551	0.009803	3638970
C7_00240W_A	77.69146	0.010083	3637980
ERG11	77.112	2.73E-15	3641571
GUP1	77.01222	0.010111	3636171
C6_02180W_A	75.18013	0.010565	3645861
CR_06500C_A	75.1384	0.010682	3645274
HTA1	74.68119	1.35E-21	3645658
OGG1	72.30463	0.01196	3643106
C3_06750W_A	70.90707	0.011655	30515219
RFA2	70.87946	1.88E-06	3643506
C1_11940C_A	70.33293	0.011926	3642046
C1_03940W_A	69.67496	1.42E-09	3646415
LSC1	67.57347	2.08E-17	3640199
HHF22	67.06224	2.8E-22	3647327
ASK1	66.79357	2.11E-06	3635656
CFL11	65.01147	3.4E-09	3646622
C3_07540C_A	64.8939	0.014002	3638526
DDR48	64.14773	2.58E-18	3644061
ILV5	63.22592	2.01E-15	3644012
C1_11950W_A	62.97176	0.014543	3642045
CR_04490C_A	62.97145	0.0146	3641013
C1_05100W_A	61.14181	0.01545	3647669
ARF3	60.85342	4.26E-06	3636628
CDC21	58.98914	6.67E-06	3643463
C1_07920W_A	58.73351	0.016342	30515026
RBT4	58.07789	6.71E-17	3639534
C1_10560C_A	58.02794	0.016869	3647196
YNK1	57.52674	5.27E-17	3637746
C2_10010C_A	57.43769	0.017066	3643962

ERG6	55.68096	1.31E-14	3636655
C6_00660C_A	55.65729	0.017444	3647133
C4_03170W_A	55.14304	0.018251	3637840
CR_04650W_A	55.03585	0.017507	30515396
FAD1	55.03342	0.0176	3634930
C6_01880W_A	54.43699	0.01798	3641599
C4_04380C_A	54.0059	9.6E-06	30515266
C1_03600W_A	53.36001	1.01E-05	3636880
CR_09810W_A	53.24581	0.018957	3638985
HOM6	52.95858	1.54E-05	3636815
OST1	52.8184	1.11E-05	3643657
CWH43	52.81063	9.47E-11	3640389
LEU2	52.44588	1.48E-07	3638034
ASF1	52.08034	0.020309	3647348
SMC1	51.5213	2.03E-08	3643221
C1_06050C_A	51.22037	1.04E-05	30515003
C4_07080C_A	50.79141	1.77E-08	3642905
C2_09590C_A	50.23069	0.021433	3643800
C1_05190C_A	48.96972	0.022059	30514991
MLH1	48.41831	0.023132	3645758
IHD1	48.19392	8.53E-32	3640291
ASH2	47.87579	1.65E-05	3636445
EXG2	47.87218	2.71E-10	3636816
RAD54	47.85351	1.63E-05	3636192
ATP18	47.72265	1.7E-05	30515084
ACP12	46.72758	1.83E-05	3637361
DUR1,2	46.56742	1.66E-09	3644771
C1_05740C_A	46.53731	0.023795	3639893
CYT1	46.53147	1.35E-16	3643441
C4_00920C_A	46.46964	0.023617	3635678
DOG1	46.08286	1.86E-05	3641597
ATP19	44.96282	2.07E-05	30515061
C3_06640W_A	44.77795	0.026436	3642251
AGM1	44.63777	2.21E-05	3636138
SNZ1	43.78853	1.48E-10	3636803
ATO1	43.74432	1.11E-14	3647761
MAL2	43.42038	8.23E-17	3638946
C6_02190C_A	43.34777	3.24E-08	3645862
RNR1	43.06115	1.44E-16	3642989
SWI6	42.96484	2.76E-05	3634932
C5_04310W_A	42.95053	0.02868	3636477
CR_03340C_A	42.90692	0.027681	3645144
C2_06280C_A	42.82447	0.026702	3646923
PMI1	42.49608	5.13E-08	3643795
CR_09520C_A	42.05568	4.87E-08	3647103
POL1	42.05406	1.28E-13	3635144
CR_06170W_A	41.90815	2.66E-05	3644865

MCM2	41.83226	4.62E-05	3643197
PLB5	41.69451	1.97E-16	3640750
YOX1	41.66333	0.028556	3637952
DAD3	40.99258	0.028403	3644864
EMP24	40.57777	6.84E-08	3647247
PCL2	40.51641	0.031915	3646501
CR_03470W_A	40.02793	5.85E-05	3643198
C5_03930C_A	39.96669	2.31E-10	3647447
CLB4	39.8542	1.03E-07	3643102
C1_13770C_A	39.77311	0.029887	3636139
C3_06730W_A	39.77252	0.029707	3638504
PGA34	39.77114	0.029788	3642579
QCR2	39.11118	1.79E-16	3646352
BIO2	38.86179	3.61E-05	3640106
ERV46	38.43999	2.51E-07	3644129
ATP1	37.93715	1.64E-31	3646134
C6_00120W_A	37.38754	0.033436	30515323
C2_09960W_A	36.71955	0.033426	3643821
COX5	36.23156	3.97E-18	3634768
ARC18	36.09836	0.034639	3644056
C7_01750W_A	36.04727	0.03564	3637009
THR1	35.79982	7.47E-05	3641572
ERV25	35.3911	7.68E-11	3642243
C2_09100C_A	34.87668	1.54E-12	3644113
C3_02530W_A	34.84068	0.037115	30515189
C2_07540W_A	34.0487	8.8E-05	3645222
CTF8	33.71813	0.038945	3644120
ERP5	33.69495	2.63E-11	30515046
C7_01830W_A	33.6673	0.038005	3637053
C4_01140C_A	33.64424	0.038511	3635657
ECM33	33.63331	3.84E-26	30514977
GIN4	33.51628	9.01E-12	3644543
MSH6	33.27159	2.86E-11	3636161
CYB5	32.88056	4E-07	3637965
MPS1	32.76532	9.73E-05	3641888
CR_01920W_A	32.47204	0.040932	3640096
C3_02330C_A	32.44509	0.040394	3636550
PGA54	32.22657	1.42E-17	3637929
SSR1	31.9413	5.87E-16	3638055
DDC1	31.83592	0.041719	3638374
C3_07130W_A	31.83465	0.041467	3638440
PGA30	31.80823	9.79E-05	3641227
C7_03650W_A	31.22095	0.042922	3646224
SEC21	31.05252	7.96E-17	3644786
C2_05720C_A	30.9505	3.98E-09	3645574
PMM1	30.62489	6.35E-11	3636852
C2_02330W_A	30.01136	0.045694	3635811

C2_06270W_A	30.00331	0.045235	3646921
C3_07050W_A	30.00331	0.04524	3638515
C3_06400C_A	29.9838	1.83E-06	3642260
CR_05360C_A	29.95392	0.000147	3648033
BGL2	29.91075	4.91E-19	3635691
DPP3	29.87257	3.27E-10	3636942
CDC12	29.47292	1.75E-17	3636809
LYS9	29.45917	3.6E-10	3642279
CR_01910C_A	29.40683	0.046779	3640095
MET15	29.25746	2.2E-11	3645031
GDH3	29.25284	3.17E-15	3648086
PDI1	29.20049	6.42E-16	3641367
C3_02140C_A	28.8754	3.06E-08	3636656
KIP1	28.85637	1.86E-06	3645261
ERG26	28.76954	1.12E-08	3642803
QCR8	28.73812	3.08E-12	30515112
RGD1	28.67865	9.8E-09	3639868
EXO1	28.42577	2.35E-08	3641583
NHP6A	28.21404	8.65E-17	30515244
MSB1	27.84982	1.44E-11	3648218
HGT1	27.65126	1.32E-28	3645420
SEP7	27.54051	2E-10	3644596
CHO1	27.31946	8.3E-10	3644557
RFX2	27.19143	3.43E-10	3635658
C1_07260C_A	26.66639	0.000242	3644456
CDC46	26.62083	3.72E-08	3646912
CR_10440W_A	26.58146	0.000395	3638976
CSM3	26.47636	0.000469	3644083
CHT2	26.4269	3.9E-16	3636523
C1_04430C_A	26.3435	0.000259	30514986
LYS4	26.33979	2.2E-09	3641180
C4_00390W_A	26.18624	3.5E-06	3645016
SEC22	26.02242	0.000282	30515391
CR_05500C_A	25.86382	0.000302	3647598
PMT2	25.70476	2.42E-17	3638538
HSL1	25.46807	4.04E-14	3637810
RFC5	25.23116	0.000339	3638890
FAV2	24.89808	0.000347	3647236
C2_07650C_A	24.86526	3.14E-06	3642342
C7_00230W_A	24.85017	8.59E-10	3637982
SEF2	24.70415	9.46E-06	3647052
C2_05290C_A	24.5908	9.27E-08	3643472
SOL3	24.46463	9.1E-08	3646625
LEU4	24.39207	5.98E-05	3639339
C1_06700W_A	24.35459	0.000393	3639511
CR_10530W_A	24.25361	0.000414	3639042
ATP3	24.2293	1.5E-16	3640387

NUO1	24.08627	3.05E-11	3647100
RAD53	23.88633	8.99E-06	3645633
DUT1	23.88524	2.51E-07	3640188
C1_06920C_A	23.88409	0.0005	3639525
FRP2	23.75479	1.28E-09	3638008
C7_01500W_A	23.72935	1.72E-07	3637050
CR_03000C_A	23.70327	0.000535	3642597
PTP3	23.58286	1.78E-17	3638940
TSA1	23.55373	4E-17	3642269
C1_01080W_A	23.45624	0.000417	3640173
C6_00770C_A	23.45467	0.000417	3647079
SEC23	23.40097	2.51E-14	3641818
RFA1	23.3715	1.98E-16	3638823
ERV29	23.292	1.05E-10	3635672
SUN41	23.23796	1.89E-22	3647067
HGT3	23.13468	0.000434	3643185
C1_00700W_A	23.13306	0.000446	3639330
GIN1	23.11585	2.06E-08	3644539
SMC2	23.00026	2.82E-09	3643918
ACO2	22.98607	2.74E-07	3646708
CR_01490C_A	22.84207	1.76E-07	30515376
NRM1	22.72196	8.82E-06	3639343
LSP1	22.49008	1.49E-21	3648025
LYS12	22.26815	1.01E-14	3640134
IDP1	22.11493	1.2E-07	3647474
LAG1	22.1057	2.86E-10	3640383
C5_02380W_A	21.9994	1.41E-21	3637794
SRB1	21.8286	6.36E-21	3647454
C1_06630W_A	21.82077	3.56E-08	3639544
C5_02920W_A	21.68818	0.000486	3637751
ACO1	21.43046	1.18E-24	3642090
CR_09560C_A	21.36781	0.000563	3647099
ERG2	21.21327	0.000628	3639347
C1_09020W_A	21.08988	2.78E-10	3634917
C1_04200C_A	21.08976	2.76E-07	3644718
CBR1	20.9676	2.95E-10	3646961
C1_02760W_A	20.96218	1.22E-08	3636870
C1_05180C_A	20.88631	0.000604	30514990
CR_00990W_A	20.73603	0.000695	3640425
TSA1B	20.72221	1.07E-15	30515214
C6_04100W_A	20.62248	7.36E-12	3647836
CMP1	20.40285	3.3E-10	3639328
C2_09050C_A	20.34753	7.44E-09	3645791
HTA3	20.29826	7.07E-09	3638322
YVC1	20.21166	4.58E-16	3642303
HTB1	20.16557	5.89E-18	3645659
SEC13	20.0919	2.42E-12	3638425

C5_03120W_A	20.08397	0.00073	3637705
MCM6	19.99885	1.08E-06	3640142
C5_01230C_A	19.97232	1.94E-11	3642618
C2_02050C_A	19.854	3.18E-05	3635851
KTR4	19.76343	0.000816	3646425
SAC1	19.71142	3.64E-08	3634864
SOU1	19.70479	6.45E-05	3642799
CR_09330C_A	19.68565	1.89E-11	3641899
C2_07550W_A	19.6692	4.49E-12	3645221
ACT1	19.65227	2.04E-23	3636195
HAT1	19.58462	3.3E-05	3644770
LSC2	19.53577	7.53E-15	3646618
C6_03130W_A	19.31865	1.43E-06	30515341
OSH3	19.23652	6.96E-12	3645368
ERG13	18.98615	2.22E-11	3641881
EAF6	18.96175	0.000935	3646494
COF1	18.95099	2.49E-16	30515293
C1_04010C_A	18.759	0.001243	3646426
CR_00910W_A	18.73063	2.66E-16	30515371
CPH1	18.66348	2.33E-09	3644468
C5_05350W_A	18.61166	3.39E-07	3636401
C4_00710W_A	18.6026	3.03E-05	3645750
CR_03520C_A	18.569	8.85E-11	3643203
CAR1	18.55656	1.27E-14	3636503
DPB4	18.42687	1.89E-06	3638818
C1_12650C_A	18.3201	0.001098	3646836
GPX2	18.30155	5.87E-15	3644009
C1_09670C_A	18.29312	0.001512	3634872
ATP2	18.19427	6.51E-23	3645039
SMC3	17.96606	6.37E-05	3638408
RET2	17.93477	6.68E-12	3637714
CR_05800C_A	17.83971	0.001343	3646706
C3_01020W_A	17.82629	3.54E-05	3639828
SGA1	17.79842	5.21E-08	3636652
CEX1	17.7175	1.28E-06	3644913
SAS2	17.58753	4.29E-05	3638817
C2_06610C_A	17.51604	0.001372	30515140
RIP1	17.50453	7.91E-15	3635204
C2_02410W_A	17.46369	9.73E-12	3635755
C5_00080C_A	17.45908	2.59E-12	3647886
C7_01710W_A	17.35782	2.72E-12	3637012
C3_06630W_A	17.30756	0.001832	3642250
TOP2	17.29819	6.24E-11	3642777
C5_05020C_A	17.02455	2E-07	3636486
PHO15	17.02231	6.28E-09	3644459
C2_00590W_A	17.01329	3.87E-05	3638851
VMA2	17.00417	1.17E-14	3646710

SAR1	16.90377	2.17E-11	3645865
C1_02500W_A	16.87482	0.001544	3636795
PGA63	16.87136	5.63E-14	3639524
INO1	16.86929	1.54E-20	3638966
BUB1	16.80916	2.25E-12	3637922
C4_00910C_A	16.80728	1.85E-06	3635679
MYO2	16.74508	4.49E-17	3636140
PST1	16.67969	2.83E-15	3643520
C1_09980C_A	16.55306	4.66E-05	3634863
CAC2	16.27422	5.56E-05	3646978
C2_10200W_A	16.24407	0.002254	3643928
TAL1	16.23944	6.65E-14	3643225
SDH12	16.20524	1.66E-14	3642775
AXL2	16.0768	8.26E-16	3641184
ASM3	15.94185	0.00015	3639332
QCR7	15.92142	5.88E-12	3639233
C1_01190C_A	15.90397	6.46E-05	3640207
MDH1-1	15.85153	5.65E-14	3635636
C5_00340W_A	15.83516	1.17E-09	3641555
C1_04780C_A	15.78576	3.65E-07	3644774
APL4	15.64495	2.45E-11	3642565
HET1	15.61459	1.31E-10	3647897
LAT1	15.56992	1.27E-13	3636986
C2_00180C_A	15.55261	1.17E-05	3638905
RIC1	15.51106	3.6E-06	3639331
DOA1	15.42951	2.9E-12	3634871
C4_06140C_A	15.42945	0.002264	3642821
DOT1	15.39927	6.91E-12	3642236
CR_04760C_A	15.38878	0.000112	3647303
CRD2	15.32773	0.002829	30515234
PMS1	15.1481	1.6E-05	3647714
C1_07590C_A	15.04893	7.64E-07	3646673
CHS1	15.02031	1.25E-07	3641364
ACS2	14.99519	2.08E-14	3644710
NDH51	14.96621	1.93E-15	3645444
HGT2	14.95727	1.71E-13	3644579
CHL4	14.94789	0.002587	3646131
C1_14320C_A	14.92351	0.002981	3643316
LYS22	14.87223	1.11E-05	3645466
TOS4	14.82658	5E-13	3644548
DPM3	14.79525	0.000129	30515245
MNT1	14.79206	1.16E-13	3636585
C1_06340W_A	14.77245	1.86E-06	3639499
NUC2	14.69274	5.48E-17	3637030
UGA32	14.66614	1.05E-05	3639333
C1_09060C_A	14.62798	0.00253	3634772
CR_05130C_A	14.62763	0.002601	3646401

C4_02420C_A	14.62707	0.002532	3637849
SOD1	14.55625	1.29E-07	30515248
C4_05250W_A	14.54057	1.35E-05	3646158
C4_00020W_A	14.50586	1.64E-11	3648053
CLC1	14.4229	7.22E-12	3635662
C1_03720C_A	14.40715	3.02E-11	3648216
C3_01070C_A	14.40665	0.002858	3639832
PMT4	14.39752	1.89E-10	3644096
SPC34	14.30582	0.002608	3641173
C6_02990W_A	14.28393	2.13E-05	3639193
ESC4	14.21021	0.000261	3635727
FRE30	14.19834	2.19E-08	3646025
C2_01260W_A	14.1489	0.000149	3638792
YBL053	14.13379	2.07E-07	3647045
CR_09050C_A	14.0954	1.76E-05	3648197
CR_09230C_A	14.08335	1.98E-05	3641918
C1_06270W_A	14.06967	6.92E-08	3647523
SEC12	14.06958	1.6E-11	3641639
FRE7	14.05198	1.17E-09	3646024
RAS1	14.03411	1.27E-13	3643927
C1_12880C_A	14.00144	4.71E-10	3636151
C2_07140W_A	13.97905	3.88E-09	3643508
CDC54	13.92481	3.57E-05	3647606
HGC1	13.87153	1.46E-11	3639349
DUN1	13.86544	3.38E-05	3636494
LEU1	13.8517	2.14E-11	3639685
C2_07010W_A	13.83162	1.21E-09	3643535
CDC3	13.82946	6.17E-15	3644719
PSF1	13.7552	0.004352	3645894
C1_01210W_A	13.75126	9.15E-07	3640209
C2_03970W_A	13.64569	0.003554	3637419
C4_04980W_A	13.64291	3.43E-05	3641167
GND1	13.63795	5.98E-13	3636131
CR_10140W_A	13.56918	1.06E-13	3638972
C5_02220C_A	13.50501	0.003667	3637760
C4_03290W_A	13.5013	4.49E-12	3637887
C3_05990C_A	13.49578	1E-06	3641732
SEC20	13.4085	0.000207	3639206
ERG10	13.39054	2.65E-14	3648275
ATP5	13.39016	4.02E-13	3640521
C2_09040W_A	13.33803	1.33E-07	3645792
CR_00060C_A	13.31415	3.79E-11	3639673
GLT1	13.26749	2.77E-14	3639519
C3_00520W_A	13.13283	0.000236	3640513
CR_03270W_A	13.11363	2.08E-05	3645101
ABG1	13.05452	2.86E-08	3647715
MYO1	13.02629	3.47E-11	3647248

MMS21	12.99704	0.000376	3642265
MIS11	12.98466	1.08E-16	3645732
C1_08140W_A	12.9784	0.000252	3640730
C4_04800W_A	12.97625	2.14E-13	3641113
APL2	12.95386	2.27E-10	3638343
C5_01590W_A	12.93754	1.71E-06	3647041
CUP5	12.92387	1.59E-12	3635174
AAP1	12.92253	6.62E-05	3647397
C5_05230C_A	12.91024	1.86E-12	3636470
C3_07230W_A	12.89329	3.16E-11	3638531
CR_09070C_A	12.86376	1.74E-10	3641926
AMO1	12.86027	0.000377	3642994
DAP1	12.85555	2.15E-05	3641021
C7_01460C_A	12.85226	1.51E-05	3636958
C7_00670C_A	12.85183	5.66E-07	3638024
C6_02100W_A	12.8517	1.64E-06	3647554
C4_04970C_A	12.8517	1.46E-05	30515274
TPM2	12.82853	1E-12	30515441
PSF3	12.81792	0.006306	3637999
SDH2	12.79819	1.53E-14	3646397
C3_02820C_A	12.7977	7.08E-11	3638419
SNF5	12.78079	4.1E-08	3648297
SMC5	12.76365	4.21E-06	3645103
RAD52	12.75306	1.63E-06	3647148
OPI3	12.70169	0.004826	3642277
C5_03780C_A	12.6352	0.000439	3647232
USO5	12.59975	1.19E-06	3638398
C4_01510W_A	12.58796	6.69E-07	3635616
PDR17	12.5771	0.000203	3643007
CDC42	12.52475	3.45E-09	3646488
C4_05790W_A	12.52419	8.56E-08	3641784
C3_00210C_A	12.49539	0.00025	3640560
C4_03400W_A	12.48684	0.000213	30515256
CTF5	12.39034	0.005366	3647550
CR_08610W_A	12.27604	9.79E-07	3642109
C2_02310W_A	12.25849	4.88E-09	3635809
NUF2	12.2524	1.66E-06	3642613
ERG24	12.18175	6.86E-07	3647716
RHO1	12.17107	2.26E-14	3642564
GVP36	12.15978	4.93E-07	3641772
C5_01960C_A	12.14389	8.22E-11	3643738
C5_02210W_A	12.11721	5.25E-07	3637759
C4_04600C_A	12.11592	0.000293	3641156
C3_01610W_A	12.10196	3.15E-14	3636664
ACB1	12.06275	5.38E-08	30515350
PPH3	11.99565	0.007026	3643193
C1_14060W_A	11.92407	1.38E-11	3643344

IPP1	11.9123	1.94E-12	3643874
C3_06410C_A	11.87904	2.75E-05	3642261
ARP3	11.85045	1.61E-10	3643479
APE3	11.84251	3.71E-05	3643875
MEP1	11.82906	3.76E-08	3636553
INP51	11.80147	3.57E-06	3643806
FGR41	11.74337	0.00604	3634839
CR_03330W_A	11.7392	0.005776	3645095
PDX1	11.69623	1.86E-12	3636128
DPP2	11.66602	0.000374	3645341
PMT1	11.62497	7.25E-15	3641393
CDC19	11.58974	8.53E-13	3643438
SKN1	11.55545	3.78E-14	3641755
ATP17	11.54937	2.71E-11	30515364
C2_09180W_A	11.52186	0.008806	3644092
CIT1	11.48824	2.61E-11	3643201
PDS5	11.47594	3.52E-10	3642341
CR_09570W_A	11.47445	5.13E-05	3647098
SPC3	11.44871	5.36E-06	3636121
LYS1	11.44244	1.9E-09	6335429
MRP8	11.42881	6.08E-07	3641178
C2_06770W_A	11.40082	0.007064	3643515
BMH1	11.39212	5.04E-14	3636810
C4_03860C_A	11.37845	0.000456	3647854
ALD5	11.36115	3.02E-17	3643026
SSH1	11.29456	2.14E-07	3642504
C1_02370C_A	11.21687	1.08E-06	3644576
GFA1	11.1958	4.15E-10	3636557
CR_05760C_A	11.19183	0.000457	3646695
RFC2	11.16558	4.58E-05	3637939
IDP2	11.13737	2.02E-15	3644934
VMA11	11.12178	1.6E-05	3637054
C2_02260W_A	11.10367	2.07E-07	3635778
ZWF1	11.09911	2.65E-12	3634913
ADK1	11.0705	1.05E-10	3644533
ESP1	11.06861	2.24E-06	3640197
C5_05200C_A	11.06258	2.92E-06	3636467
C4_02930W_A	10.91601	0.000501	3637876
ALG8	10.91491	0.000484	3636562
C3_05880C_A	10.91274	2E-11	3641737
CR_01070W_A	10.8225	0.000514	3640382
VMA8	10.81622	5.59E-09	3642798
IFA4	10.80731	0.000591	3645454
C5_03950W_A	10.78285	1.31E-06	3647445
OPT6	10.7027	8.46E-09	3635603
TVP18	10.64689	8.61E-05	3634844
CR_03950W_A	10.63929	0.009191	3640960

CR_04770C_A	10.62686	0.009498	3647302
CR_04860C_A	10.61554	0.008629	3647294
MNS1	10.60795	4.41E-09	3644692
C6_04120C_A	10.60531	5.41E-09	3647974
GRP1	10.57122	0.000719	3634899
CDC7	10.5611	1.08E-06	3643424
C1_01800W_A	10.54643	0.000643	3645407
CBP1	10.46229	4.51E-16	3641920
C2_08890W_A	10.4572	0.017705	3645799
RAD51	10.45518	2.53E-10	3644944
C5_04950C_A	10.45192	1.38E-06	3636422
KGD2	10.43834	3.64E-11	3646036
CR_06070W_A	10.39917	2.57E-08	3644840
DPB2	10.36874	0.000235	3639026
C3_04080W_A	10.36754	1.13E-14	30515200
PDB1	10.32824	1.96E-10	3641186
VMA5	10.29484	2.88E-08	3642316
C3_07880C_A	10.29407	0.009288	3647470
SUC1	10.28629	4.47E-10	3641916
C6_02330W_A	10.28081	4.94E-14	3645877
C5_04260W_A	10.26554	0.000225	3636451
MTS1	10.25987	4.6E-12	3634873
ASE1	10.25771	0.00013	3641729
HXK2	10.25247	5.14E-10	3641015
CEK1	10.23848	0.000155	3642789
YCG1	10.23533	6.06E-10	3636639
LMO1	10.1636	3.96E-11	3641403
C1_11760C_A	10.15285	1.83E-05	3645328
TPD3	10.13759	2E-10	3638512
C1_11960C_A	10.13439	0.009532	30515060
C1_10310W_A	10.13351	0.009522	3634906
MCR1	10.12668	1.71E-12	3647561
CAT2	10.06675	6.69E-14	3635659
PIL1	10.06168	2.38E-13	3644769
PGA38	10.05275	0.000151	3637846
CWH41	10.04355	1.71E-06	3646533
ATP4	10.02631	4.69E-13	3643418
C1_02650W_A	9.971656	1.89E-10	3636818
PST2	9.963044	0.000206	3643909
MSH2	9.957326	7.14E-10	3642939
CHS2	9.926038	2E-10	3641893
POR1	9.902535	6.79E-14	3644698
C2_03210W_A	9.898869	0.00099	30515098
ARO9	9.888109	2.39E-06	3641773
CRN1	9.87458	1.59E-10	3637034
CR_00130C_A	9.860341	1.78E-07	3639709
VCX1	9.860028	1.7E-07	3646508

C6_01960W_A	9.840911	0.000271	3644559
C1_07830C_A	9.805499	0.011525	3640736
C5_04550W_A	9.803266	0.000149	3636508
CR_07170W_A	9.792285	6.96E-10	3645271
C3_04740C_A	9.701758	0.000263	3635251
C1_05320C_A	9.692795	2.97E-06	3642470
RSR1	9.666845	4.45E-15	3640145
C6_03090W_A	9.662474	0.012542	3639275
NGT1	9.652271	0.011294	3640573
C5_00060C_A	9.638743	2.25E-06	3647889
IRO1	9.603299	9.76E-11	3636648
FUM12	9.584904	4.8E-13	3638489
HMG1	9.513454	1.44E-08	3644687
FESUR1	9.510095	1.41E-10	3638514
ARC35	9.493468	1.34E-06	3639823
RPS17B	9.484877	3.55E-13	30515044
C2_07720C_A	9.482126	2.63E-15	3642304
C7_03800W_A	9.47853	0.000176	3643150
POL2	9.467993	1.23E-09	3645733
SEC18	9.446678	2.94E-11	3636208
C1_10750C_A	9.437349	0.001479	30515043
C1_08080C_A	9.436323	1.76E-07	30515029
C6_04280W_A	9.386194	6.57E-09	3646279
MET13	9.373946	1.4E-07	3638382
GLC3	9.359631	4.57E-15	3639200
ERG5	9.344448	2.18E-12	3641400
TRS20	9.343908	0.001625	3640214
C6_02250W_A	9.331064	0.012777	3645869
C1_08440C_A	9.310999	0.014665	3646487
VMA7	9.300675	0.000221	3637332
C6_00930C_A	9.244839	1.98E-08	3644032
FTR2	9.242276	4.56E-08	3643303
APM4	9.21976	0.000206	3642297
SPE3	9.181269	1.29E-06	3643528
CR_02620C_A	9.168958	3.84E-09	3642567
BRN1	9.156548	1.11E-06	3641815
KIP3	9.153625	2.87E-05	3641716
C1_00830W_A	9.151916	2.73E-05	3639344
C1_03170C_A	9.147193	0.014686	3636805
C3_04630W_A	9.120324	1.93E-06	3635189
ALS1	9.081059	3.26E-12	3640280
RNH35	9.065156	0.017378	3636987
DED81	9.044533	1.33E-14	3646227
CLA4	9.033886	1.05E-09	3634878
C1_08730W_A	9.025195	0.000209	3634938
STV1	9.01022	9.06E-10	3646070
SER33	8.999515	7.24E-11	3642030

IDH1	8.995368	1.51E-10	3634893
ARP1	8.983969	0.002188	3646349
ATP14	8.976509	3.84E-10	30515134
CR_00880W_A	8.97536	0.001755	3640397
CR_07110C_A	8.974158	0.001773	3648224
TRX1	8.968119	1.02E-10	3638950
PDA1	8.963139	2.22E-11	3642902
FMA1	8.93735	0.021285	3647986
C1_05490C_A	8.913931	1.76E-07	3642507
SPC2	8.882567	0.001778	3645769
C4_03770W_A	8.882307	0.001815	3645354
PET9	8.833088	3.07E-13	3641587
CAS1	8.830459	0.000228	3645324
VAM3	8.82991	0.000236	3635146
RKI1	8.788087	0.001978	3636574
CR_02500W_A	8.78643	2.54E-05	3647696
EAP1	8.77866	2.98E-10	3643814
ALG6	8.751692	5.05E-05	3647316
CR_04730W_A	8.73818	0.000227	3647305
ERG9	8.721946	1.01E-08	3643913
ILV6	8.72176	3.4E-07	3635598
C6_02880W_A	8.689884	0.015953	3639238
C3_04780C_A	8.688687	2.92E-07	3635214
RHO3	8.674353	1.72E-06	3643448
HIS1	8.602651	1.24E-06	3636501
GDE1	8.59845	9.7E-11	3636505
HEM14	8.56304	1.94E-06	3634923
C4_00190W_A	8.559777	0.002737	3645030
ABP2	8.552977	0.000316	3643443
ALG9	8.537892	1.15E-09	3644457
C1_12240C_A	8.534248	1.62E-06	3642023
FGR51	8.522608	1.73E-06	3647796
CR_05010W_A	8.513179	0.002295	3646389
SUB2	8.5077	3.57E-13	3645033
C4_04440W_A	8.498841	2.5E-07	3641177
BFR1	8.477533	2.51E-10	3635579
PGA33	8.472641	3.24E-07	3637350
SMI1B	8.436931	0.000343	3643675
C3_01940C_A	8.408506	9.82E-07	3636600
CR_09010C_A	8.387913	5.08E-07	3641892
MEP2	8.385093	0.000628	3645022
C1_10520W_A	8.36868	0.018254	3647191
RTT109	8.36868	0.018291	3639678
LIP6	8.368597	0.018675	3634890
MEU1	8.329214	2.61E-06	3645635
C6_01460C_A	8.319925	3.81E-05	3641623
SNG3	8.315827	4.95E-07	3646715

RFC4	8.307821	4.63E-06	3638994
C2_05870W_A	8.292107	0.025255	30515128
CR_03020C_A	8.284512	6.31E-07	3642598
MET6	8.25508	1.76E-09	3640084
C1_02700C_A	8.248635	4.08E-08	30514972
SEC8	8.181637	1.34E-07	3647078
LAP41	8.181328	2.04E-07	3636653
C6_00110C_A	8.17525	2.68E-05	3647896
HFL1	8.15811	0.002399	3636883
C6_02490C_A	8.15237	3.86E-06	3639171
C1_05920W_A	8.143221	2.02E-06	3639864
HBR2	8.140497	6.04E-14	3646273
C2_06920C_A	8.134043	2.26E-05	3643524
C6_03990C_A	8.131798	2.57E-05	3640271
RFC3	8.117662	0.000361	3647440
C1_11280W_A	8.103258	5.49E-07	3644554
C7_04000W_A	8.093082	2.21E-05	3643114
IRR1	8.06563	1.67E-09	3643305
CR_08480C_A	8.060055	7.98E-06	30515442
CAP4	8.050351	0.002352	3637335
CR_04850C_A	8.047113	0.021227	3647295
POL3	8.032295	2.8E-08	3641359
CR_03560W_A	8.025752	0.002799	3643217
CR_01280C_A	8.002071	0.000182	3640400
BNR1	7.966068	1.89E-06	3639672
PHO113	7.93674	0.030548	3644946
RPL24A	7.923298	1.27E-12	3641174
C2_10470C_A	7.907269	1.87E-07	3641196
C3_01590W_A	7.903444	0.000179	3636666
C1_03490W_A	7.891552	3.24E-07	3636914
C6_03050C_A	7.866425	0.002962	3639270
MNN2	7.849802	4.78E-07	3644405
SSA2	7.846883	8.02E-09	3644711
EGD2	7.843061	4.33E-09	3635284
RNH1	7.827031	4.94E-06	3639237
C1_01010W_A	7.821975	3.3E-07	3640181
C3_00790W_A	7.81903	1.37E-05	3648186
C6_02530C_A	7.815812	0.000626	3639175
STE24	7.767165	5.13E-09	3645040
ACS1	7.763806	5.32E-09	3643941
CYP1	7.75619	3.73E-12	3636978
SUR7	7.706893	1.19E-09	3641610
PRD1	7.693694	1.31E-12	3642471
C7_00410C_A	7.663677	2.3E-06	3638033
C5_05040W_A	7.654646	0.000217	3636488
CDC5	7.650964	7.24E-08	3639281
AAT1	7.650044	2.2E-07	3643468

C1_00450C_A	7.635932	4.58E-06	30514955
GLO3	7.624594	9.37E-14	3640556
C3_04420W_A	7.6243	8.98E-09	3635180
AGE3	7.606761	5.12E-07	3644565
RNR21	7.594973	3.35E-08	3643021
CR_09530C_A	7.58459	2.65E-09	3647101
RIM1	7.579211	1.39E-08	3639898
SCP1	7.571755	0.026798	3646554
C7_03350C_A	7.566382	0.024814	3646722
C5_04540C_A	7.566193	0.025142	3636509
C7_03160W_A	7.558537	5.55E-10	3641345
C2_09650W_A	7.546439	6.46E-11	3643794
FAS2	7.544842	7.02E-11	3635222
PMT5	7.521355	4.08E-05	3639011
IHD2	7.496636	6.35E-06	3639359
MSC7	7.48625	5.4E-08	3645214
MDH1	7.468483	1.51E-11	3639667
CR_06510W_A	7.452733	0.001013	3645264
C1_14240W_A	7.451793	8.67E-09	3643306
CDC24	7.44946	1.75E-10	3643737
CR_00690C_A	7.443414	4.07E-05	3640390
ALG5	7.407605	0.026878	3642558
C4_03990C_A	7.403923	0.004399	3648295
FGR38	7.40221	2.14E-06	3645412
CR_04560C_A	7.386867	0.000127	3640946
C1_06480C_A	7.378509	2.92E-06	30515008
SFH5	7.372171	2.35E-06	3634884
STT3	7.369814	1E-07	3635743
CR_09590W_A	7.340683	2.59E-05	3647095
INT1	7.335866	1.96E-11	3637761
LHS1	7.335113	1.98E-08	3643032
CR_07220C_A	7.32733	3.74E-07	3646040
C1_12180C_A	7.32362	0.000892	3642038
CR_01430W_A	7.290933	0.001063	3640138
MMS22	7.287089	0.000371	3639681
ERG251	7.282761	7.99E-08	3635716
PGA10	7.276242	0.005489	3645009
KSR1	7.254735	0.000247	3646052
C2_02670C_A	7.243369	0.029305	3643008
YPT72	7.221286	3.41E-06	3643523
C1_11740W_A	7.216234	0.001101	3645317
RDI1	7.213295	9.27E-12	3635167
VPS1	7.209433	6.1E-12	3642656
YHB5	7.202401	1.27E-10	3647343
CMK2	7.176987	5.79E-09	3643961
C2_10830W_A	7.170045	0.006415	30515170
C1_03240W_A	7.143187	0.001156	3636895

LTE1	7.141208	3.77E-10	3643495
C4_04230W_A	7.14074	0.001296	3646857
HIS5	7.12778	0.005571	3645756
PFY1	7.12612	2.88E-10	3640703
CR_05680C_A	7.108553	0.000366	3646684
C3_02190C_A	7.096758	3.56E-07	3636642
C1_00420W_A	7.074879	4.24E-07	3639286
SMP3	7.056837	0.032737	3642980
CR_01320C_A	7.026968	4.31E-05	3640386
PYC2	7.022745	1.38E-10	3637323
CRC1	7.014389	4.88E-06	3640112
C7_02080W_A	7.004266	0.000296	3637025
ERG4	6.994406	1.7E-12	3640511
GIT2	6.980066	1.73E-07	3642633
C3_03510C_A	6.959991	7.7E-06	3638310
C6_00270W_A	6.95989	2.39E-06	3646066
MNN24	6.955644	2.4E-09	3638789
EGD1	6.919573	1.88E-10	3645340
C1_01290C_A	6.912635	0.006429	3640184
CSH1	6.903981	3.49E-05	3646427
YPT31	6.887905	1.88E-08	3647999
C7_00600C_A	6.862371	0.000341	3638031
C3_01310W_A	6.858862	0.000245	3636620
RPP2A	6.847927	4.73E-09	30515439
BEM2	6.841657	4.18E-08	3637044
C5_04410C_A	6.803548	0.00158	3636410
AMO2	6.802478	0.002105	3648021
C1_11860W_A	6.798501	7.47E-08	3642009
ADH2	6.79738	8.16E-08	3640751
CR_01100C_A	6.766071	0.034912	3640378
C7_02520W_A	6.76398	0.034105	3636941
BET4	6.758127	0.006008	3644695
CR_01080W_A	6.736187	3.67E-08	3640381
DBF4	6.734481	3.83E-06	3641388
C6_02160W_A	6.721016	0.000177	3645889
C4_04920W_A	6.704455	0.000246	3641172
HOF1	6.676456	0.001769	3645014
CHT3	6.653313	0.000252	3638967
ENA21	6.651553	2.65E-07	3641392
PSF2	6.611154	0.03745	3647534
C5_02110W_A	6.605619	3.35E-06	3637732
C6_01560W_A	6.603673	0.000322	3641652
ILV2	6.593671	5.58E-07	3636552
C2_03020C_A	6.583469	2.22E-08	3643019
C5_01070C_A	6.569333	6.09E-06	3642627
COX4	6.564612	1.94E-10	3635737
ARC40	6.563982	3.73E-09	3644866

C2_05560W_A	6.560855	0.001965	3645561
VPS51	6.560824	0.001913	3639242
C5_01440C_A	6.553683	0.000384	3647043
APG7	6.549614	0.000174	3646615
C2_08650W_A	6.538214	6.64E-08	3643908
C2_01690W_A	6.537535	2.31E-08	3635728
MCM3	6.501489	1.56E-07	3645223
C4_02400C_A	6.496218	0.002345	3637851
PCK1	6.49435	2.66E-10	3639701
C7_01320W_A	6.493986	3.39E-06	3645896
SNL1	6.492948	3.31E-06	3647192
PHM7	6.488169	6.1E-10	3642329
TSC11	6.48033	0.007843	3645267
GPI7	6.478684	3.97E-05	3647672
RTS1	6.467708	1.6E-12	3646557
C6_02200C_A	6.455239	0.040015	3645863
MCT1	6.452952	1.92E-06	3646683
C2_01730W_A	6.449082	0.040744	3635732
HNT2	6.442981	0.038625	3642271
VRG4	6.43318	3.66E-06	3641768
CCC1	6.418649	1.41E-05	3645645
C3_05280C_A	6.409969	0.000805	3635202
COX6	6.40711	2.41E-10	30515099
PEX11	6.403901	0.000414	3646288
CR_02210W_A	6.401627	2.25E-10	3644943
DCK1	6.387307	9.11E-10	3637383
HOM3	6.378204	0.000497	3641771
STE13	6.319996	7.42E-05	3647597
GCF1	6.313843	3.24E-08	3646498
CR_05200C_A	6.304374	0.000452	3648080
C7_03640C_A	6.295587	0.008905	3646223
C2_09970C_A	6.292126	2.71E-09	3643820
CDC47	6.287148	3.23E-07	3645780
HGT13	6.282214	0.041733	3638047
PHO114	6.282045	0.04104	3635644
HSP12	6.271877	4.15E-06	3643745
C3_02210C_A	6.249299	0.000102	3636641
C4_01840C_A	6.247645	0.00018	3635592
RPS15	6.208103	2.17E-10	3635194
FRS2	6.199135	2.11E-09	3636824
C4_05080C_A	6.194019	0.000366	3641129
PLD1	6.193018	4.62E-08	3645329
C4_06460C_A	6.189042	0.012448	3642791
C6_02480W_A	6.18893	0.003269	3639170
CR_04880W_A	6.180917	0.000795	3647309
PUP2	6.178426	2.33E-08	3646617
C1_08800W_A	6.137277	9.81E-05	3634791

CDC50	6.123485	5.57E-06	3640302
C2_04450W_A	6.121891	0.044838	3645467
C5_03370C_A	6.121829	0.045151	3646350
CR_04870C_A	6.118776	0.012789	3647310
C5_00840W_A	6.113652	0.010533	3644130
C1_10540C_A	6.113129	0.000807	3647193
RHO2	6.110082	0.000763	30515143
MVB12	6.107445	0.00075	30515400
VMA13	6.045543	1.61E-08	30515402
NDE1	6.03486	9.76E-07	3638354
C1_13280C_A	6.021194	1.94E-05	3636145
MNN10	6.017072	1.4E-05	3644997
C4_06940C_A	6.012313	0.000495	3642928
TEF2	6.008055	1.81E-09	3646502
SEC62	5.999961	1.65E-05	3636860
C7_03410C_A	5.997671	0.000101	3646716
MDH1-3	5.984089	2.79E-07	3641197
SPC98	5.979155	2.48E-05	3640113
C6_00180C_A	5.961379	0.04732	3646081
C2_05860C_A	5.961069	0.048514	3647473
C2_01910W_A	5.961069	0.048696	3635790
C1_04030W_A	5.911789	0.003368	3646428
C2_10860C_A	5.910053	1.44E-09	3641233
C1_10460W_A	5.901853	8.04E-07	3647183
KAR9	5.898675	0.000457	3636136
CR_07230W_A	5.893197	5.06E-05	3646031
C6_03310W_A	5.864271	3.26E-05	3639197
CR_02260C_A	5.857713	0.000971	3644912
C2_09780C_A	5.840243	1.99E-05	3643805
GNA1	5.827846	0.000132	3637397
CR_04020C_A	5.827621	0.000121	3640969
C5_01410C_A	5.826341	8.83E-06	3647054
C3_07140C_A	5.818399	0.000396	3638439
CDC20	5.808094	8.19E-06	3644057
AHP2	5.807548	5.05E-05	3636977
C6_00200C_A	5.804897	3.42E-06	3646082
PRN4	5.804875	0.000867	3639877
PXA2	5.800421	7.61E-09	3642055
PLC2	5.782585	0.002859	3642985
CHS8	5.779322	5.7E-09	3640532
C1_05670W_A	5.773193	3.37E-06	3639899
CR_08560C_A	5.759844	2.65E-08	3642142
C2_03450W_A	5.754134	0.000876	3637349
C2_02020W_A	5.750284	5.48E-06	3635769
TFP1	5.746505	9.17E-09	3636662
FGR6-1	5.741761	0.012594	3647947
SMC4	5.734552	5.57E-09	3641578

C6_04250W_A	5.732911	2.63E-11	30515345
FCY2	5.729148	9.66E-05	3638365
C3_06610W_A	5.716756	5.08E-05	3642248
CR_08670C_A	5.705934	0.001052	3642126
C3_00850C_A	5.705703	4.92E-08	3647748
C4_06810C_A	5.694718	1.63E-08	3642916
PGA59	5.69294	4.6E-09	3637881
C2_02660W_A	5.686738	4.78E-05	3643009
KEX2	5.681236	6.11E-08	3634914
C2_05850C_A	5.671845	6.82E-05	3647472
HIS7	5.657027	0.000223	3647868
CR_07120C_A	5.653014	0.003063	3648225
TOM20	5.649452	0.000164	3636817
C3_03160C_A	5.648973	0.013054	3638424
RAD7	5.647853	3.18E-05	3635833
ACC1	5.639822	1.29E-08	3639688
TIP1	5.620211	0.000504	3636512
ATP7	5.616927	1.2E-07	3646672
RHB1	5.605438	0.017558	3635232
APM1	5.601195	1.16E-05	3636424
ZCF24	5.599659	0.001462	3645417
MMD1	5.591155	6.13E-09	30515015
C3_05550C_A	5.588403	1.18E-05	3643659
C2_02840C_A	5.588069	3.48E-06	3642976
C2_09760W_A	5.587354	0.003323	3643807
C1_02890C_A	5.55646	0.013674	3636867
BUB2	5.556226	0.013719	3643030
C1_05950C_A	5.548632	3.03E-06	3639859
MP65	5.546416	2.42E-09	3643951
SEC61	5.537931	5.06E-07	3647462
C1_01950C_A	5.522795	0.004086	3645421
TRP4	5.465725	0.016072	3642904
C2_08540C_A	5.464168	0.014757	3643920
C2_05170W_A	5.462861	0.000548	3643462
C2_03340W_A	5.441992	0.000213	3637392
C1_13320C_A	5.441274	1.6E-07	3636149
HXT5	5.427664	0.000585	3643209
C3_00170C_A	5.41567	4.17E-06	3640589
C6_02560W_A	5.408678	0.001142	3639205
MNN26	5.406483	1.45E-05	3646240
RDH54	5.39593	2.09E-05	3641231
KAR2	5.395789	5.81E-06	3638830
CR_01300W_A	5.391028	3.93E-05	30515373
MPT5	5.384591	1.44E-05	3636205
C4_00330C_A	5.380297	1.93E-07	30515227
SNG4	5.374003	1.56E-07	3646714
C4_01460C_A	5.3713	0.017159	3635622

CHS6	5.370905	0.000119	3641377
SPC19	5.367303	0.018332	3646423
ARE2	5.364474	3.25E-06	3643526
RIB5	5.357946	1.17E-08	3636499
TPI1	5.345601	1.69E-07	3638482
C1_10090C_A	5.345236	1.45E-07	3634817
C3_07460W_A	5.333868	2.27E-06	3638480
MCI4	5.332539	6.42E-06	3640118
RAD10	5.326851	0.008373	3639884
SRD1	5.321821	1.37E-05	3635764
ADE1	5.306125	6.74E-06	3639722
APN1	5.285297	0.000183	3642263
C1_01250W_A	5.279983	1.46E-05	3640212
C3_03270W_A	5.279496	0.018042	3638321
CDC6	5.279037	0.01754	3642034
ROB1	5.269637	8.43E-07	3636212
C6_03320W_A	5.265234	0.00647	3639198
C6_04340W_A	5.259641	1.17E-05	3646291
KIP2	5.254549	0.002012	3643954
CTA2	5.240884	1.48E-05	3639372
IQG1	5.233774	5.32E-06	3637052
C3_01900C_A	5.215383	0.001387	3636559
ORC3	5.206739	0.00012	3645639
CR_04080C_A	5.205575	2.92E-07	3641023
C3_01910C_A	5.204505	1.67E-06	30515182
C6_03960W_A	5.202717	2.23E-06	3640269
AYR1	5.202557	0.00077	3647754
C1_04580C_A	5.200727	0.000427	3646132
CR_07350W_A	5.19665	8.03E-09	3646055
C4_06160W_A	5.186989	0.018647	3642819
C1_02780W_A	5.181901	5.17E-05	3636871
APL5	5.179182	1.42E-05	3638377
CR_02680W_A	5.167768	0.000743	3642573
CLB2	5.165038	1.16E-07	3635840
CTF18	5.165003	5.09E-05	3640431
ARP9	5.162479	9.67E-05	3639829
C1_07340W_A	5.161156	1.62E-07	3644472
YSA1	5.153465	0.000198	3638516
C1_06200W_A	5.153035	1.07E-05	3648300
C2_05130W_A	5.14359	0.003476	3643458
CAK1	5.14038	0.000552	3637352
NCE4	5.132869	0.007503	3641194
TFC4	5.121373	8.17E-06	3638420
CAT1	5.119732	6.74E-06	3639495
C1_12700W_A	5.108551	0.000727	3646831
C4_03050C_A	5.101145	0.000227	3637930
C1_02680C_A	5.095175	0.000169	3636822

PHO89	5.090412	4.4E-09	3635633
MON2	5.08548	2.56E-07	3636201
C3_01510W_A	5.073075	0.000887	3636571
CAT8	5.063664	0.003915	3640745
SEC24	5.061266	4.29E-06	3634939
C1_03410W_A	5.058775	0.003513	3636905
C1_02970W_A	5.04345	4.74E-07	3636835
HYU1	5.042297	3.87E-07	3643024
CR_05840W_A	5.034992	0.000206	3646704
ADO1	5.030799	5.43E-07	3639274
C3_02290W_A	5.025611	0.0002	3636556
C3_07110W_A	5.022486	1.82E-05	3638442
CR_09670C_A	5.005694	7.92E-05	3647091
C2_07110C_A	5.005165	0.000902	3643505
C7_03200C_A	5.004187	0.000393	3641342
C5_04790C_A	5.001977	0.022624	3636446
C1_07850C_A	5.001928	0.022317	3640739
C1_00790W_A	5.001079	0.009034	3639348
LRO1	4.996568	1.35E-07	3639356
SEC34	4.993431	3.41E-06	3644455
C1_00210C_A	4.993343	3.2E-06	3639335
C3_00470W_A	4.97212	1.9E-05	3640520
C6_00910C_A	4.95972	3.35E-08	3644030
MES1	4.958134	6.71E-08	3636516
C6_00540W_A	4.955127	0.004117	3647146
PEX8	4.954602	0.003663	3647393
MAE1	4.946239	2.97E-10	3641615
HYS2	4.945402	0.000277	3636441
C1_07840W_A	4.931672	7.97E-08	3640737
C1_07440W_A	4.924076	8.9E-08	3644482
C1_01000C_A	4.92338	0.001127	3640180
YCS4	4.920929	6.78E-07	3645196
C7_00420C_A	4.908745	0.00026	3638032
WBP1	4.908324	6.53E-08	3644398
C1_07010W_A	4.905207	0.004479	3639537
C7_01740C_A	4.905016	0.004235	3637010
SWI4	4.902355	2.64E-05	3645408
SEC27	4.895247	1.21E-08	3645870
MUM2	4.878373	2.35E-07	3636432
C3_00910W_A	4.874062	0.008782	3647755
SSO2	4.866723	1.1E-08	3643809
C1_07780W_A	4.86401	1.11E-05	3647955
GPX3	4.854582	0.004111	3644471
TLO1	4.852362	2.28E-05	3639677
C1_12400C_A	4.846333	0.004738	3642059
IFA21	4.842473	0.006799	3641344
UBA2	4.840912	0.000944	3640701

DAM1	4.838004	4.97E-05	3634820
CPR3	4.831672	7.88E-06	3635812
CR_03530W_A	4.825962	2E-10	3643204
HOC1	4.823689	9.34E-05	3641605
C4_00800W_A	4.823254	0.032054	3645757
DCC1	4.820872	0.029231	3638037
C4_04280C_A	4.80902	0.009181	3646846
C6_03950C_A	4.80402	0.004233	3640268
ASN1	4.80265	1.96E-07	3645790
DSL1	4.801433	0.010247	3645718
C6_01360W_A	4.800771	0.004606	3641608
CSR1	4.798353	0.010579	3641162
FRP3	4.763627	5.59E-06	3648102
ATP16	4.762991	6.68E-07	3638963
C4_01910W_A	4.740172	0.000554	3635635
PLC1	4.718913	0.000174	3647860
C6_03040C_A	4.716147	0.000194	3639269
GSP1	4.711264	3.13E-10	3646925
C1_02290C_A	4.704742	0.004348	3644568
C7_01950W_A	4.703549	0.0002	3636970
ARO3	4.697084	2.07E-05	3635770
C2_07420W_A	4.696644	0.000828	3645202
UAP1	4.696102	9.52E-07	3637769
MNN12	4.688459	2.34E-08	3634904
IST2	4.688455	3.18E-07	3646662
CMD1	4.679079	9.54E-08	3646520
C4_04160W_A	4.670443	6.15E-07	3641185
RPL43A	4.655837	1.35E-06	30515319
C1_05900W_A	4.650568	4.57E-07	3639866
SFL2	4.649303	1.4E-05	3636434
DNM1	4.647173	5.82E-08	3643673
C3_04970C_A	4.641867	6.04E-07	3635164
C2_04170C_A	4.64148	2.28E-06	30515111
ERG27	4.640639	2.46E-05	3640432
C4_05340W_A	4.639429	0.000286	3646947
C1_03440C_A	4.637796	1.07E-05	3636908
C6_02280W_A	4.634447	0.000136	3645872
END3	4.633541	0.000292	3636644
C7_03780C_A	4.632326	0.03077	3643119
IPL1	4.629876	0.000979	3645876
C5_04420W_A	4.628748	0.000817	3636411
COX7	4.628554	9.01E-07	3638339
C7_00150W_A	4.61857	6.47E-08	3637973
GAL4	4.611891	3.04E-07	3641244
FGR34	4.604941	3.16E-05	3639033
IDH2	4.603447	5.09E-10	3642979
C2_05800C_A	4.603286	2.64E-05	3645584

C3_04650W_A	4.60256	0.00187	3635192
PDC11	4.584154	1.04E-05	3642780
SEC26	4.574293	1.5E-07	3641005
HUT1	4.566915	0.040181	3638505
ERG12	4.564335	0.000131	3634859
C4_00770C_A	4.562294	0.000847	3645760
ECM39	4.562207	0.000885	3641386
C2_07980W_A	4.558492	0.001855	3642312
C1_10630C_A	4.555031	0.005541	3647683
MEA1	4.554808	0.005453	3635154
ARP2	4.553145	4.88E-07	3641915
MNN21	4.551438	1.91E-05	3648124
NUO2	4.550327	1E-06	3638381
ILV3	4.549849	9.2E-06	3636428
SMF3	4.548387	5.67E-05	3638852
C2_00400C_A	4.542667	4.93E-08	3638821
AOX1	4.542652	0.015243	3634946
HEM4	4.542085	0.002693	3643942
C3_03340C_A	4.538204	0.000362	30515195
C4_00410W_A	4.536999	0.000263	3645019
C1_08050W_A	4.525187	1.53E-06	3640709
C6_01750C_A	4.520798	0.037083	3641640
CYC1	4.516209	7.71E-06	3643937
DNA2	4.504826	0.000102	3646072
ABP1	4.501505	2.34E-07	3637875
MTW1	4.484703	0.013482	3643783
PXA1	4.48181	1.17E-07	3639713
GEA2	4.480275	2.12E-05	3636646
STE23	4.473363	1.42E-05	3639236
UGP1	4.471173	7.54E-06	3648144
C5_04770W_A	4.459611	0.001228	3636444
C3_06920W_A	4.456419	0.001112	3638511
C3_04590W_A	4.451449	1.48E-06	30515204
C3_01420C_A	4.449413	0.004192	3636634
GAT1	4.449152	0.001426	3641794
FUM11	4.437804	7.03E-06	3641016
C4_06240W_A	4.435984	0.002727	3642807
PGA53	4.435099	0.000944	3635599
KGD1	4.415629	9.26E-06	3647752
C4_04250W_A	4.415405	0.000243	3646855
C7_01930C_A	4.414357	0.000906	3637027
PSD2	4.411249	8.33E-07	3636515
YHM2	4.40138	1.8E-06	3647137
CR_01180W_A	4.401343	0.000154	3640427
C2_08930W_A	4.396121	3.3E-05	3645795
ATP20	4.386884	4.41E-05	3647602
TFS1	4.381979	2.64E-06	3642655

RPL8B	4.381	5.48E-08	3635198
C3_07450C_A	4.370762	0.005891	3638481
SEC4	4.36042	2.27E-07	3640119
C1_00630W_A	4.354676	0.003211	3639380
GWT1	4.354455	0.001233	3645575
GYP1	4.34222	1.36E-05	3641138
SPF1	4.338655	4.59E-07	3646573
CR_00270C_A	4.327339	0.047599	3639720
KRE5	4.323129	0.000263	3638384
C1_01050C_A	4.317439	0.00024	3640170
LCB2	4.314051	1.7E-06	3636181
NIF3	4.302141	0.003929	3646527
CR_07600W_A	4.291347	0.000826	3644849
C2_08380C_A	4.286406	0.001243	3648253
NPT1	4.275597	0.00024	3643109
CR_02570C_A	4.273882	0.005042	3647685
C1_14170W_A	4.265276	1.46E-07	3643323
SEC10	4.249524	1.15E-05	3642961
C1_08780W_A	4.249179	2.98E-05	3634789
C1_14530W_A	4.247813	6.52E-05	3643333
GDB1	4.246562	1.43E-05	3647409
C1_13190W_A	4.240028	3.52E-08	3636163
C6_01050W_A	4.238121	0.000277	3644015
FMO2	4.237041	0.04674	3637440
ARC19	4.225909	0.017203	3640418
CPY1	4.225845	2.05E-05	3646721
SEC3	4.222032	2.88E-06	3642806
SGT2	4.220747	3.48E-07	3642978
FBA1	4.217607	8.19E-06	3635585
HEM13	4.20794	7.98E-05	3647390
ANB1	4.179347	9.1E-07	3641648
FEN12	4.176478	4.36E-05	3637404
ZCF7	4.160471	0.018418	3636667
CR_05880W_A	4.155096	0.007611	3646702
C4_01760W_A	4.149698	0.000175	3635584
CR_07460C_A	4.14082	0.000727	3646049
CAM1-1	4.1365	4.08E-05	3646358
C2_02950W_A	4.127724	0.00693	3643012
ERG7	4.124818	2.63E-07	3635761
C2_09560C_A	4.124289	0.000517	3643813
C4_07240W_A	4.123152	0.000247	3642945
C3_01800C_A	4.109931	4.3E-06	3636586
C3_04460W_A	4.107725	6.09E-05	3635208
C5_02900W_A	4.107609	7.14E-05	3637747
C5_02050W_A	4.10191	6.23E-07	3643768
DAD4	4.095913	0.019511	30515433
RPS12	4.094761	7.06E-06	3638438

BUD7	4.087938	6.81E-07	3647228
TLO9	4.083234	0.000925	3648051
C2_09910C_A	4.078411	0.045847	30515162
C7_03150W_A	4.076665	0.048928	3641346
SGO1	4.070276	0.000117	3643464
ADE6	4.047355	1.98E-05	3647304
C4_04860W_A	4.046734	0.012334	3641161
C1_00330C_A	4.046574	0.001846	3639296
THR4	4.038758	2.99E-06	3637711
APS3	4.034107	0.007543	3646491
ARO8	4.033546	8.58E-06	3638828
C1_11250W_A	4.030469	0.000862	3644556
CDC73	4.019971	0.000141	3636830
C3_05010C_A	4.015491	0.001611	3635169
C6_02090C_A	3.99192	4.26E-07	6335445
C2_07100W_A	3.989987	2.31E-05	3643504
C5_00610C_A	3.98634	0.00265	3641585
C7_01480W_A	3.978337	0.002105	3636956
MKK2	3.977241	3.4E-07	3645580
OBPA	3.975314	3.05E-06	3643764
PAN3	3.971254	9.46E-05	3636464
VPS23	3.969035	0.025143	3644401
C2_00730C_A	3.964431	0.001603	3638913
C1_02320C_A	3.964268	2.55E-07	3644572
C4_00380W_A	3.959156	0.008211	3645015
C1_00920W_A	3.959081	7.2E-05	3639283
SHP1	3.947311	8.97E-06	3640083
MET14	3.942548	0.02389	3641547
C4_04330C_A	3.94015	7.52E-07	3646840
TYR1	3.939658	0.001208	3635639
C4_06020C_A	3.939296	2.69E-05	3646519
C2_04180C_A	3.930381	0.000416	3637330
C2_06110W_A	3.93013	8.04E-05	3644094
C1_03540C_A	3.926257	0.027821	3636920
C1_02270C_A	3.916815	0.016149	3644566
C1_12740W_A	3.914867	9.13E-05	3646819
MAD2	3.901525	0.023913	3644696
C1_07820W_A	3.901361	0.025003	3640735
MED20	3.9011	0.025028	30515250
SAS3	3.899297	8.05E-05	3640073
FGR6-4	3.896272	0.000224	30515164
TOM22	3.889274	0.000967	3647650
ARC15	3.889237	0.000947	3646043
CAM1	3.872942	6.2E-06	3641734
RPP1B	3.867547	0.000112	3643104
C1_10680C_A	3.853066	3.4E-05	3647678
GAP2	3.844876	0.02963	3643662

RBD1	3.844434	4.48E-05	3642018
PMT6	3.844429	4.81E-05	3641116
C2_08340C_A	3.841341	0.002947	3648165
IFI3	3.836654	0.027568	3644694
OCA6	3.836293	0.026123	3643336
C1_02330C_A	3.83489	4.65E-05	30514969
NHX1	3.83099	4.19E-07	3647141
PKH3	3.829527	3.5E-06	3646075
CTA24	3.825295	4.38E-06	3636475
GRS1	3.822235	6.5E-06	3642474
CR_04330W_A	3.821829	0.001852	3640997
C2_04410W_A	3.820994	0.002527	3645455
SOD2	3.817728	0.0003	3640231
TLO34	3.811486	0.00067	3648392
ECM4	3.810959	0.000136	3640144
COQ3	3.80776	0.002051	3641630
ZCF32	3.806897	0.003626	3635213
C3_07820W_A	3.806005	0.003865	3647463
BET2	3.805733	0.002452	3639025
C2_03890W_A	3.805478	0.014939	3637396
C1_11120C_A	3.802738	0.000122	3644412
C3_00050C_A	3.795505	0.000835	3640570
PGA14	3.793265	6.56E-08	3641560
C1_13270W_A	3.783587	0.008488	3636144
C6_00920W_A	3.783026	0.000241	3644031
NIP100	3.779941	2.56E-05	3640970
TRP99	3.771601	0.000678	3642494
HGT10	3.770918	0.008434	3640309
C6_02030C_A	3.762159	0.000549	3647562
CR_05900W_A	3.755184	0.016682	3646681
C2_01320W_A	3.74303	0.001677	3638846
CR_07570W_A	3.742312	0.003801	3648004
BNI4	3.737514	7.21E-05	3646406
CR_08050C_A	3.730794	6.1E-05	3644133
C1_12660W_A	3.726345	0.001475	3646835
C3_01210C_A	3.711165	7E-05	3636612
PRS	3.706252	0.032332	3640100
C4_03810W_A	3.704038	0.002071	3645350
CNB1	3.703475	0.001526	3636463
VAC8	3.689247	5.92E-05	3647410
UBC15	3.676932	0.002271	3641260
YKU80	3.674114	0.002243	3645138
KIP4	3.672999	0.000155	3642032
C7_04310C_A	3.669927	0.000248	3643141
GPR1	3.659723	9.27E-05	3642616
FAA21	3.656748	0.003898	3638418
SWC4	3.655896	0.000181	3639679

C4_04180C_A	3.655798	0.015976	3641183
C2_01170C_A	3.652625	9.16E-05	3638860
C2_03290W_A	3.644806	0.002473	3637429
CSP37	3.637734	0.000367	3640098
RPS7A	3.637685	2.06E-05	3636573
OSM2	3.626695	0.000578	3636193
C7_03480W_A	3.624646	0.000102	3646230
C3_01180C_A	3.620771	0.00011	3647960
SER1	3.617534	0.003014	3646909
CDC68	3.611194	2.47E-05	3642787
CR_08620C_A	3.598679	0.001203	3642110
LAP3	3.592404	0.000208	3641012
C5_02750C_A	3.589237	0.000249	3637740
RAP1	3.585575	0.002912	3643945
C2_01160W_A	3.582423	0.008763	3638861
DAO1	3.579042	0.000205	3636885
GPA2	3.576969	2.01E-05	3636638
SRV2	3.575147	3.49E-06	3640938
C1_05390C_A	3.569554	0.000153	3642479
C7_02670W_A	3.569375	0.01157	3647651
C1_04190C_A	3.56702	0.006182	3644717
VMA4	3.56666	5.87E-06	3640111
CR_08640C_A	3.564494	6.33E-06	3642112
ERG1	3.556978	0.000568	3646509
PGA56	3.555664	6.2E-06	30515313
COX8	3.547955	3.61E-05	30515130
PSY2	3.545886	0.002589	3644567
C2_01340W_A	3.543209	3.41E-07	3638844
C3_05410W_A	3.534885	7.16E-06	3643665
ECI1	3.526825	0.001004	3642128
CR_08900C_A	3.523948	0.001427	3641908
CR_07370W_A	3.521063	0.00248	3646053
C5_03870C_A	3.520609	0.000745	3647223
C7_03660C_A	3.518391	8.4E-06	3646226
REV3	3.510597	0.000753	3641749
C3_01720C_A	3.504002	1.37E-05	3636601
VPS8	3.496641	0.003503	3640534
C1_08680C_A	3.490749	0.001024	3634933
CDC14	3.479508	5.72E-06	3647132
C6_00190W_A	3.477053	0.000923	3646080
CYB2	3.476422	0.00127	3636213
THS1	3.473689	3.9E-07	3647882
C6_02470W_A	3.472994	0.000865	3639169
SEC72	3.466085	0.004001	3645768
C7_04280C_A	3.463417	0.000228	3643173
C1_11140W_A	3.46061	0.000197	3644410
C1_04620W_A	3.456499	1.41E-05	3646135

C3_06990W_A	3.455128	0.024316	3638521
PIKA	3.452542	0.000284	3643765
MSO1	3.450547	0.000352	3642040
CR_02410W_A	3.447761	0.002903	3644928
CR_06790C_A	3.444206	0.003277	3647311
RFC1	3.44162	6.88E-06	3645582
C1_02490C_A	3.437756	0.002697	3636853
C6_03820C_A	3.425723	0.001708	3640288
CR_03460W_A	3.409383	0.002633	3643213
DQD1	3.408087	0.004106	3643488
C5_02060W_A	3.405756	0.026258	3643748
NUP159	3.405065	6.33E-05	3647458
HAT2	3.403269	0.000308	3645973
C3_03080W_A	3.396016	0.010938	3638397
ECM25	3.395673	0.011327	3636148
MUQ1	3.392705	0.000136	3638898
ASR1	3.389962	0.001935	3644402
NPL4	3.389607	0.006538	3639820
ASR3	3.389479	1.47E-05	3637402
GLG2	3.388198	0.000313	3642282
C3_07740W_A	3.386619	7.86E-06	3638453
YBN5	3.386117	4.52E-05	3644781
FAS1	3.378811	4.21E-05	3641593
GYP2	3.375667	5.88E-06	3641780
CSA1	3.371579	2.8E-05	3638009
DBF2	3.367836	0.002789	3648101
PPH21	3.362792	2.29E-05	3636665
ALT1	3.360109	2.34E-05	3638307
C1_08930C_A	3.355802	0.028058	30515031
C6_03580W_A	3.355461	0.026934	3640250
C1_04090C_A	3.355445	0.027102	3644697
GLX3	3.350211	3.97E-05	3638325
C1_14180W_A	3.346338	0.001417	3643324
MAC1	3.34549	0.002424	3637985
C6_02740W_A	3.344401	0.000819	3639216
PHB1	3.338354	0.001227	3645641
ARA1	3.33517	0.00755	3642331
C1_10960W_A	3.327541	0.014001	30515047
MRV2	3.318585	0.005275	3636545
C2_02530W_A	3.316342	0.019367	3635819
SCS7	3.316141	0.000336	3641157
C3_01820W_A	3.314686	0.00018	3636584
CR_10600C_A	3.314028	5.47E-06	3639004
C7_04120W_A	3.305511	0.028458	3643132
C2_01930C_A	3.295104	0.001587	3635793
IFK2	3.29271	0.013289	3637439
TOM70	3.282168	1.15E-05	3647645

YRB1	3.278314	4.89E-05	3639663
RAD16	3.277083	7.27E-05	3636874
C7_02540W_A	3.276698	0.000829	3636939
HOG1	3.27095	1.02E-05	3637393
PTC6	3.255584	0.004775	3647351
SEC1	3.252543	6.47E-06	3636935
ETR1	3.24874	0.000111	3640561
C6_01370W_A	3.240681	0.003771	3641607
LRG1	3.240428	1.43E-05	3639727
C7_00520W_A	3.236716	4.53E-06	3638001
C7_03840W_A	3.234379	0.00719	3643146
CR_10290C_A	3.229077	1.65E-05	3638935
PRO3	3.221189	0.000228	3645036
C7_03260C_A	3.217917	0.021215	3641366
C1_02510W_A	3.21344	0.016314	3636796
OPT4	3.212168	0.000643	3647697
HGT7	3.211676	0.000247	3638876
MPH1	3.210283	0.001602	3642817
ALG2	3.20921	0.001217	3647832
ISC1	3.205315	0.033562	3646705
EIF4E	3.204172	1.36E-05	3638981
C1_10730W_A	3.198298	0.004517	3644404
PHB2	3.196052	0.000199	3635238
C7_02100W_A	3.188891	0.00016	3637023
OCH1	3.187584	0.001167	3641751
C4_02160C_A	3.185818	0.000764	3635668
SRO77	3.185674	2.31E-05	3646088
C4_02470C_A	3.184394	0.000754	3637869
TAF14	3.176963	4.25E-05	3637357
C1_11430W_A	3.17104	0.009218	3644541
C1_07630W_A	3.168623	0.004146	3646660
SHM1	3.163064	2.12E-05	3646724
C7_01160C_A	3.162505	0.0106	30515351
C1_11920W_A	3.156991	0.001502	3642003
C3_05300C_A	3.15665	0.039651	3643646
C2_08270C_A	3.152112	0.00417	3642347
C2_00510W_A	3.150123	0.000774	3638868
C6_03330C_A	3.141227	0.010957	3639199
FAT1	3.139492	0.000566	3647083
C3_00610W_A	3.137142	0.011549	3640578
C1_02060W_A	3.134742	0.015022	30514968
GDA1	3.13403	0.000186	3641762
SAH1	3.133728	6.02E-05	3636452
MED18	3.130284	0.005572	3644103
ALG7	3.127835	0.003357	3642291
C5_01420W_A	3.127588	0.007638	3647055
C3_06860C_A	3.126708	0.0012	3638541

EMP70	3.125082	0.00011	3637919
C2_07440C_A	3.121402	0.004627	3645200
C1_11100W_A	3.120975	0.017952	3644414
CR_05450C_A	3.11854	0.018808	3648236
CYP5	3.116939	0.000231	3642257
C3_04810C_A	3.116392	0.000259	30515205
IFR2	3.114855	2.18E-05	3645100
C4_00590C_A	3.114125	4.84E-05	3645772
CDC27	3.113826	0.004657	3640405
MSK1	3.111616	0.007894	3637032
C1_07360W_A	3.110925	0.000148	3644470
C1_14050C_A	3.105766	0.001337	3643343
PPE1	3.105704	0.0397	3635802
C1_05990C_A	3.105596	0.037263	3639871
RNA1	3.099599	0.000374	3636595
C6_04560W_A	3.073811	0.000225	3645977
C4_05220C_A	3.063535	0.013823	3646155
C7_00790W_A	3.062971	0.000702	3637943
RPS8A	3.059458	0.000617	3645565
C4_02100C_A	3.058593	0.020556	3635641
ARG2	3.055629	0.037674	3647533
POL32	3.054684	0.001352	3639881
RPS1	3.054059	0.000631	3636924
CDC13	3.05213	0.006299	3639293
VPS52	3.050577	0.004963	3638996
C1_09070W_A	3.050328	0.000683	3634773
LPD1	3.045824	0.000993	3646037
RPL19A	3.043524	0.000615	3635257
SAM4	3.041541	0.000353	3646506
PNP1	3.038759	0.000791	3638426
ERG8	3.035579	0.002625	3635640
TMA19	3.032546	5.71E-05	3640399
C2_04330C_A	3.03211	0.004386	3645453
C2_00830C_A	3.027173	0.001163	3638808
RPD31	3.017315	0.000424	3638520
C2_09830C_A	3.009902	0.008287	30515161
C3_03840C_A	3.009684	0.006936	3645628
C3_04130W_A	3.007481	0.000782	3635283
C4_03320W_A	3.002001	0.001909	3648092
ARF2	3.001889	2.49E-05	3635271
C1_10320W_A	3.000163	0.00665	3634907
ANP1	2.993478	0.006079	3643917
C4_01730C_A	2.988608	0.002325	3635588
C4_01020C_A	2.983102	0.000465	3635606
NIT3	2.981871	0.005561	3644408
VAN1	2.980793	2.64E-05	3638527
MNN9	2.977404	0.001409	3641735

SFI1	2.975184	0.000825	3641385
C1_11600W_A	2.973703	0.007572	3645346
C2_07270W_A	2.96383	0.017094	3643474
GLR1	2.963775	6.39E-05	3647061
C6_00240C_A	2.956753	0.002669	3646062
RPP1A	2.95519	9.37E-05	3636839
RAV2	2.950486	0.015219	3639252
C5_03170C_A	2.945769	0.00045	3637801
C3_03760W_A	2.943335	0.001088	3645638
C5_03490C_A	2.94151	0.000871	3646967
C2_02870W_A	2.935136	0.005729	3642972
MDM10	2.932898	0.019151	3647778
XKS1	2.932845	0.00011	3648306
BMT6	2.931786	5.21E-05	3639258
ALG11	2.929368	0.032107	3645871
CR_05860W_A	2.927665	0.003989	3646703
MIR1	2.921335	0.001369	3634782
C5_04710W_A	2.916394	0.017538	3636517
CR_06740W_A	2.911768	0.000749	3646616
FDH1	2.907433	0.038993	3646398
PTC7	2.898112	0.00066	3645000
C3_01890C_A	2.894953	8.26E-06	3636561
CR_09090C_A	2.891538	0.030765	3641927
IPK1	2.889815	0.013454	3648091
SLC1	2.888578	0.003613	3638324
C1_01360C_A	2.880568	0.000532	3640191
C1_00320W_A	2.875021	0.02267	3639297
LYS21	2.873363	0.001101	3644763
HPC2	2.868078	0.002298	3636188
PMR1	2.865336	0.00082	3638043
TRP3	2.865257	0.007742	3642035
CR_06260W_A	2.859176	0.007243	3644853
CR_03600C_A	2.85686	0.001209	3643207
SET3	2.851267	6.83E-05	3643320
C2_07390C_A	2.84806	0.000384	3645229
LIP8	2.847004	0.025937	3646727
CR_00350W_A	2.846955	0.00011	3639686
VPS35	2.845576	0.000787	3645567
C1_01930W_A	2.844596	8.06E-06	30514966
SEN1	2.844089	6.66E-05	3635255
AIP2	2.842651	0.000138	3638393
RPL20B	2.841189	5.57E-05	3635615
C2_00320W_A	2.838597	0.002851	3638798
C2_01250W_A	2.837343	0.00617	3638793
CHS7	2.823268	0.006491	3639869
MED1	2.818774	0.00811	3637418
POB3	2.818537	0.000402	3635827

ARP8	2.816985	0.000365	3640200
C6_00080C_A	2.816018	0.022344	3647899
GRE3	2.810223	0.000346	3637811
CR_06560C_A	2.80088	0.002517	3648367
RLM1	2.797925	9.01E-05	3635703
ACP1	2.795304	0.001361	3639825
RPL18	2.789875	3.12E-05	3635156
GOR1	2.785252	0.029977	3636836
GLO2	2.780477	0.035916	3644067
CR_00290W_A	2.779666	0.001376	3639717
C1_07060C_A	2.77888	0.028908	30515018
YHM1	2.778117	0.007537	3644535
CSE4	2.777437	0.033621	3647750
EFB1	2.776631	0.000172	3641125
C7_00250C_A	2.776086	0.001816	3637979
CR_10020C_A	2.776017	0.030194	3639032
C1_14040W_A	2.771837	0.001257	3643342
CR_02770C_A	2.771461	0.028498	3642581
C4_02040W_A	2.767539	0.036405	3635649
C2_02170W_A	2.767183	0.004146	3635751
PGA49	2.765506	0.01772	3646525
C2_02390W_A	2.764556	0.00364	3635829
C4_05860W_A	2.764508	0.034135	3641792
C3_07770C_A	2.76304	0.010728	3647457
C1_01400C_A	2.758703	0.000511	3640237
C3_06710W_A	2.757685	0.000186	3638448
C5_01550C_A	2.753141	0.000359	3647063
ILV1	2.750938	0.001296	3646918
YPT7	2.745953	0.012884	3636864
ENO1	2.7389	0.001762	3646493
ARH2	2.736375	0.017271	3643411
C2_00570W_A	2.73319	0.000978	3638853
HDA1	2.728646	0.001605	3640154
VPH1	2.720264	1.26E-05	3646157
STP1	2.710687	0.013798	3635152
C4_06770W_A	2.707862	0.00175	3642920
RPS26A	2.703211	0.000239	3635787
INO4	2.702646	0.011082	30515105
C7_02470C_A	2.701248	0.015197	3636945
C5_01890W_A	2.701106	0.00473	3643750
C6_04110W_A	2.697102	0.000114	3647834
C1_09750W_A	2.693804	0.008844	3634822
GTT11	2.692615	0.012228	3645644
RPL28	2.690607	0.000145	30515386
MRP2	2.687466	0.011082	3636498
C1_08490W_A	2.684106	0.002119	3646492
C6_03420W_A	2.67556	0.040132	3639235

C1_09430W_A	2.675219	0.000523	3634855
C3_04360W_A	2.672436	0.008336	3635276
CR_05480W_A	2.672037	0.000376	3648238
RAC1	2.66753	0.000578	3639508
TIP20	2.666597	0.001195	3636457
TPO3	2.665503	0.005621	3634790
C4_01780C_A	2.663468	0.015466	3635582
RPL10A	2.662723	0.000363	3645868
C5_02400W_A	2.659309	0.002213	3637724
SCL1	2.657186	0.001301	3640510
CR_09510C_A	2.656307	0.004156	3647105
C6_02630C_A	2.655737	0.001523	3639221
C3_03210W_A	2.642794	0.042956	3638315
CR_06520C_A	2.642057	0.040663	3645263
C7_00140C_A	2.639314	0.016987	3637974
ZDS1	2.639141	3.41E-05	3641103
GCN4	2.63781	0.002916	3643823
ZCF39	2.624747	0.001211	3639037
C6_03880W_A	2.620336	0.002656	3640293
AGE1	2.617854	0.000299	3636913
RPS16A	2.617707	0.0003	30514974
CHC1	2.61434	0.003308	3647552
SGT1	2.612834	0.000852	3644068
MCM1	2.61152	0.001368	3638050
C3_06810W_A	2.610386	0.004109	3638545
C2_05590C_A	2.609704	0.011717	3645564
C4_00780C_A	2.609686	0.001601	3645759
ARL3	2.609616	0.026704	3644397
C7_03050W_A	2.608942	0.029783	3641378
HEM15	2.602201	0.000207	3645211
MDL1	2.601667	0.003027	3640146
LPT1	2.599277	0.00031	3645212
CTN1	2.596601	0.024395	3645414
TEF1	2.590409	0.001375	3648254
GLO1	2.58475	0.005181	3639312
C3_01950C_A	2.576847	0.003952	3636599
CR_02390W_A	2.571096	0.000196	3644930
USO1	2.570118	0.000613	3648280
URA6	2.568727	0.001592	3647988
CR_09750C_A	2.564662	0.008203	3639009
UGA33	2.56314	0.003128	3641886
RPN7	2.560509	0.000316	3641909
SIR2	2.553348	0.001191	3638845
C4_04240C_A	2.548489	0.001083	3646856
C5_01350W_A	2.548448	0.008893	3642640
C2_06320W_A	2.5479	0.000401	3646927
APR1	2.544562	0.003015	3645204

C6_00850W_A	2.543991	0.000566	3644010
C2_05220C_A	2.540928	0.002983	3643466
MRE11	2.540369	0.00669	3645898
C2_07220W_A	2.540043	0.000328	3643483
C7_02480W_A	2.539572	0.02148	3636944
VTC3	2.53787	0.000424	3643206
ADP1	2.533328	0.000313	3642458
C1_02240W_A	2.532014	0.000566	3644597
CDC55	2.529177	0.000214	3635785
C1_05780W_A	2.52678	0.001555	3639888
AGO1	2.526707	0.003875	3642828
YPT1	2.526398	9.22E-05	3636915
C4_03080W_A	2.524463	0.000244	3637928
MEC1	2.523564	0.002436	3647733
ZCF10	2.523338	0.010704	3643485
C5_00580W_A	2.523084	0.009421	3641588
CST20	2.521127	0.003033	3637790
C4_04090C_A	2.519598	0.029205	3641225
RPL17B	2.519334	8.38E-05	3645439
C1_06360W_A	2.516487	0.021402	3639561
C5_00790C_A	2.514568	0.001962	3644125
SEO1	2.512646	0.040092	3646621
RIM20	2.50932	0.001039	3634809
SPE1	2.508967	0.003934	3639327
C5_05440C_A	2.505557	0.001713	3636431
C1_14500C_A	2.503911	0.0003	3643331
C2_00350W_A	2.49883	0.023412	3638827
C2_10700C_A	2.492655	0.019095	3641207
RPP0	2.486215	0.006322	3638023
CR_10590W_A	2.485278	0.010055	3639003
C3_00360W_A	2.482034	0.007247	3640542
CAS4	2.480486	0.000242	3636568
C2_02700C_A	2.47907	0.038438	3643005
C1_12170C_A	2.475751	0.011469	3642039
CR_04120C_A	2.475669	0.013184	3640972
C4_03920W_A	2.475538	0.011454	3648314
C3_06350W_A	2.474028	0.011927	30515215
TKL1	2.47053	0.005769	3640767
COQ6	2.469916	0.007053	3636878
C2_08160C_A	2.467435	0.002861	30515152
GUK1	2.466457	0.020429	3647231
CDC23	2.463993	0.012375	3642111
CYS4	2.461851	0.0009	3645429
CR_07840C_A	2.456165	0.044581	3647346
ATC1	2.454447	0.000495	3639570
C7_00920C_A	2.452069	0.018271	3637957
CR_09190C_A	2.4506	0.017763	3641885

C1_04980C_A	2.445246	0.004482	3647529
RBF1	2.444667	0.028696	3639251
QCR9	2.442735	0.005215	30515251
LCB4	2.441711	0.044836	3642057
C3_00830C_A	2.436145	0.019699	3647757
C7_02170C_A	2.434501	0.019598	3636993
C1_02000W_A	2.433334	0.016891	3645418
FAA2-1	2.428813	0.016021	3644100
C1_14200W_A	2.420613	0.005372	3643301
INO2	2.420341	0.018899	3639674
CTA7	2.416892	0.019319	3637776
APT1	2.416474	0.023556	3635842
RPS30	2.416347	0.002721	30515390
TUF1	2.409385	0.000292	3639360
C3_03930W_A	2.408028	0.006818	3645657
C5_03140C_A	2.404365	0.002596	3637798
UGA11	2.401317	0.047352	3637437
C7_03370C_A	2.400707	0.003387	3646720
C1_12920C_A	2.399331	0.017002	3636155
C4_04760C_A	2.38734	0.004699	3641118
CCP1	2.385928	0.000696	3638350
RPS13	2.383632	0.002125	30515328
CR_10500C_A	2.383085	0.016195	3638982
CDC45	2.381867	0.025392	3648293
C1_00310W_A	2.3777	0.003291	3639298
PKC1	2.373145	0.000309	3635210
C2_05570C_A	2.370839	0.035156	3645562
C1_03960C_A	2.369526	0.011845	3646418
C4_06740C_A	2.369238	0.000322	3642923
SCW11	2.358001	0.014244	3636521
TOM40	2.357534	0.000588	3636968
DBP5	2.355492	0.000609	3636564
C1_04560W_A	2.353783	0.034613	3646130
HMO1	2.353708	0.00796	3646685
HEM2	2.350412	0.015005	3637428
C7_02780W_A	2.350248	0.001466	3641361
UBP13	2.348769	0.001152	3638879
C6_03620C_A	2.345931	0.019219	3640297
ERG20	2.344676	0.003932	3645440
FLC2	2.344403	0.001379	3647962
C3_04800C_A	2.339118	0.023177	3635216
C5_03980W_A	2.331196	0.001709	3647442
C1_11850W_A	2.329674	0.010082	3642010
RPS14B	2.329142	0.001646	30515006
PRO2	2.327344	0.017166	3638532
C7_02370W_A	2.326603	0.001882	3636930
HXK1	2.325529	0.001039	3645964

YPD1	2.321842	0.010419	3644458
YKT6	2.319631	0.007306	3636863
SWD2	2.315203	0.028392	3646677
C3_07850W_A	2.306429	0.006101	3647466
FLC3	2.305409	0.009387	3642322
C3_02090C_A	2.298909	0.017435	3636661
SHM2	2.298457	0.001523	3640306
CHK1	2.295782	0.008516	3637394
C1_03690W_A	2.294966	0.003529	3648426
PST3	2.281281	0.011541	3648031
C4_05810W_A	2.277296	0.005829	3641786
PGI1	2.275136	0.009455	3644847
DPS1-1	2.272918	0.008963	3645134
CR_03860C_A	2.272164	0.020464	3643186
GPM1	2.270917	0.01376	3637431
C2_08900W_A	2.264392	0.030447	30515156
GCV3	2.263309	0.002283	3636194
OPT2	2.263187	0.037285	3644915
PEX7	2.259959	0.034911	3644027
C2_03900C_A	2.258591	0.00291	3637395
LYS2	2.251576	0.004463	3636875
AVT7	2.250765	0.005792	3637433
C3_02970C_A	2.247117	0.03274	3638385
C4_06660W_A	2.240882	0.004911	3642809
C5_01880C_A	2.240426	0.025166	3643751
RPL35	2.237334	0.001231	30515207
C1_07050C_A	2.236752	0.034734	3639532
GRP2	2.235692	0.002823	3637744
DCW1	2.234867	0.001443	3638842
FGR24	2.22795	0.02398	3643288
RPN8	2.226971	0.00144	3643770
CR_04390C_A	2.223576	0.007479	3640980
KEL1	2.2211	0.001107	3639318
PCT1	2.216248	0.003062	3645743
GRE2	2.215652	0.034344	3648026
MDG1	2.200112	0.007195	3643311
C6_02410W_A	2.199358	0.008704	3645884
C4_02620C_A	2.199076	0.019753	3637834
C6_03470W_A	2.197888	0.020227	3640253
GAL10	2.196692	0.01558	3644583
C4_07200C_A	2.192723	0.006883	3642958
NAT2	2.189976	0.01411	3638837
SRT1	2.189426	0.049719	3642020
RCE1	2.189322	0.048666	3641104
C2_10460C_A	2.188761	0.008852	3641195
CR_06620W_A	2.187579	0.030436	3646631
PRC3	2.184552	0.013244	3639889

AVT4	2.179325	0.012497	3645316
PRE2	2.174669	0.006722	3643490
CMK1	2.171886	0.00068	3635263
CR_10510W_A	2.171393	0.035328	3639039
C4_06130W_A	2.168932	0.013346	3648085
CR_08980C_A	2.168482	0.049369	3641890
SAC6	2.162703	0.004892	3639213
C2_07190C_A	2.156871	0.03729	3643513
HAC1	2.156093	0.010573	3639849
VPS33	2.154607	0.04936	3647477
STB5	2.153888	0.011247	3640208
C6_02810C_A	2.153658	0.014249	3639248
C1_06190C_A	2.15135	0.013761	3648391
C1_02410C_A	2.150278	0.042061	3636793
C3_01280W_A	2.149906	0.037689	3636623
COX13	2.148684	0.00664	3635784
C2_05580W_A	2.145941	0.015802	3645563
PTC4	2.143342	0.016277	3646697
POM152	2.141636	0.002147	3638812
RAD32	2.140719	0.039117	3637340
HST1	2.140163	0.030452	3634770
CHS5	2.138387	0.005667	3637333
SMT3	2.136031	0.00257	3644550
TERT	2.13229	0.021962	3640729
C5_02500C_A	2.130572	0.010427	3637766
C2_07810W_A	2.125294	0.035282	3642324
C2_10630W_A	2.125243	0.035	3641248
C1_04860W_A	2.124219	0.002983	3644784
GBP2	2.123221	0.00762	3637992
C3_06970W_A	2.119457	0.042015	3638506
SLA1	2.117103	0.001682	3635739
CTA26	2.113977	0.017468	3638965
MNN15	2.112243	0.045482	3644794
SBP1	2.111041	0.019906	3635280
C1_04490W_A	2.110735	0.007176	3646145
PHO88	2.108527	0.027883	3641898
C1_14310W_A	2.107769	0.029245	3643315
C2_08100W_A	2.103176	0.005166	3642334
SAC7	2.101809	0.00541	3638010
ADE5,7	2.100093	0.010549	3640719
AAT21	2.099277	0.002222	3647241
C1_05980W_A	2.099231	0.021391	3639872
GDI1	2.09872	0.023288	3643298
VPS11	2.098386	0.017826	3646524
C6_02680W_A	2.092319	0.046449	3639226
C1_13810W_A	2.087072	0.013265	3636126
ADH5	2.084467	0.012731	3640156

C1_14090W_A	2.076965	0.031644	30515079
C2_01390W_A	2.072349	0.018279	3635726
HYM1	2.072104	0.043669	3637355
CDC53	2.071981	0.004602	3636603
KIN2	2.071864	0.003682	3639697
ARO2	2.067094	0.017788	3648190
FRS1	2.062781	0.003191	3640121
MSI3	2.058934	0.018533	3639821
C1_12530C_A	2.056179	0.044018	3647608
C6_02310W_A	2.055208	0.025459	3645875
CR_06640C_A	2.052849	0.003938	3646619
CAN3	2.052116	0.001179	3647066
RPL37B	2.05114	0.009589	30515053
C1_10920W_A	2.049346	0.049163	3644433
VPS16	2.048449	0.013905	3646150
CR_01820W_A	2.04508	0.012927	3640164
SMP2	2.044214	0.002777	3635779
CCT5	2.043739	0.011864	3643478
AHP1	2.042735	0.023109	3637850
C4_00170W_A	2.036743	0.013523	3645028
RBP1	2.036234	0.014852	3647647
C7_00910C_A	2.027274	0.032239	3637958
CR_01420W_A	2.025221	0.005838	3640137
C1_08690W_A	2.023705	0.042687	3634934
TIP41	2.023705	0.042756	3636506
C7_02560W_A	2.016769	0.041522	3637016
C5_01220W_A	2.012453	0.024323	3642620
CR_07340C_A	2.012425	0.024369	3646056
NAT4	2.010573	0.01544	3635704
CDC15	2.008938	0.01656	3643459
C2_05760C_A	2.008867	0.023652	3645578
NGG1	2.005563	0.02095	3636902
C1_02430C_A	2.004108	0.025739	3636846
CHS3	2.000365	0.02159	3636199
C2_00360C_A	-2.00127	0.004811	3638825
ATO2	-2.00165	0.036644	3639845
CWT1	-2.00751	0.003204	3647595
ADA2	-2.01256	0.005489	3644450
MSN5	-2.01585	0.023015	3637889
STD1	-2.01864	0.006034	3647459
TIM50	-2.03224	0.004778	3644530
FGR15	-2.03397	0.01016	3638882
IFM3	-2.03502	0.041371	3642335
GUT1	-2.03652	0.004365	3648082
ESA1	-2.03774	0.004689	3640518
C1_14250C_A	-2.03935	0.00122	3643307
SUI2	-2.04384	0.006423	3639569

C4_03110W_A	-2.04512	0.011536	3637924
C6_03910C_A	-2.04767	0.032886	3640263
C4_00060W_A	-2.04889	0.005866	3648061
HNM3	-2.04899	0.049315	3640093
ADAEC	-2.05011	0.006705	3637342
CRL1	-2.05249	0.009313	3640238
HSE1	-2.05871	0.004313	3640407
ARG4	-2.05996	0.030189	3646237
GPM2	-2.07314	0.023902	3644713
ZCF27	-2.07327	0.036046	3635631
CR_04140W_A	-2.0737	0.016617	3640974
TIF4631	-2.07521	0.000351	3643880
ASH1	-2.07842	0.019653	3641249
C1_12820C_A	-2.08078	0.024945	3646826
C3_02900W_A	-2.08363	0.023488	3638337
AFT2	-2.08435	0.008838	3643511
SKN2	-2.08901	0.021673	3638309
ECM3	-2.08933	0.007006	3635754
PGA25	-2.09409	0.013203	3647890
CPH2	-2.09659	0.027393	3646067
C1_13720W_A	-2.09764	0.030779	3636134
C1_04960C_A	-2.09971	0.028421	3647527
GRR1	-2.10066	0.001197	3636392
MRPL8	-2.10779	0.018303	3646039
CR_00370W_A	-2.11157	0.012565	3639684
C2_03560C_A	-2.11161	0.005421	3637337
C1_12350W_A	-2.11166	0.003728	3642063
BRG1	-2.11508	0.04739	3647664
IFE2	-2.11638	0.013329	3648027
C5_05140W_A	-2.12416	0.008804	3636460
CR_02590C_A	-2.12472	0.016639	3642595
C3_05950W_A	-2.12495	0.043656	3641728
SMX4	-2.12821	0.043985	30515427
PRP45	-2.12824	0.040722	3639166
C2_06780C_A	-2.12847	0.020839	3643516
C1_01820C_A	-2.12856	0.01583	3645435
C1_00270W_A	-2.13587	0.007201	3639300
C1_01710W_A	-2.13815	0.003514	3640201
SLU7	-2.1391	0.023042	3638501
C4_01220C_A	-2.13911	0.024223	3635650
C2_00770W_A	-2.14451	0.008882	3638909
FAA2	-2.1449	0.01021	3641731
SSN6	-2.14875	0.003896	3638518
CR_07320C_A	-2.15544	0.005246	3646057
C5_01050C_A	-2.15598	0.019428	3642648
MRPL3	-2.15747	0.009292	3640722
C4_07130W_A	-2.15996	0.001847	3642938

DSE1	-2.16101	0.031188	3643899
C2_08240W_A	-2.16394	0.000243	3642344
TBP1	-2.16764	0.005106	3647675
RPO26	-2.16787	0.012345	3646351
C6_04290W_A	-2.17145	0.003027	3646280
MIF2	-2.17263	0.000207	3639220
MBP1	-2.17738	0.003005	3635281
C4_02880C_A	-2.17761	0.021369	3637907
CR_06060W_A	-2.18039	0.010904	6335358
C2_00490W_A	-2.18329	0.000417	3638870
C1_09440W_A	-2.18709	0.022724	3634857
MUP1	-2.18794	0.009515	3642008
PPT1	-2.18886	0.015818	3636602
C3_01770C_A	-2.20205	0.009967	3636588
FGR27	-2.20799	0.001805	3646988
VPS27	-2.20862	0.000432	3639326
DCP2	-2.21122	0.00177	3642069
CR_07830C_A	-2.21334	0.00045	3647345
C3_04330C_A	-2.21484	0.003741	3635272
RTG1	-2.21592	0.006613	3634929
C3_01680C_A	-2.21845	0.023136	3636605
C1_07390W_A	-2.22259	0.001052	3644466
CR_07200W_A	-2.2248	0.020965	3646044
MAS2	-2.22564	0.015463	3647249
C2_02730W_A	-2.22565	0.00302	3643035
C6_02650C_A	-2.22683	0.008424	3639223
C1_01470W_A	-2.23313	0.001748	3640241
C7_01040C_A	-2.23441	0.010268	3638018
CR_02910W_A	-2.23771	0.004446	3642586
C2_00190C_A	-2.2389	0.000741	3638904
GAP5	-2.24581	0.000265	3646954
RIB1	-2.25165	0.005966	3642607
C7_01590C_A	-2.26023	0.042476	3637037
C4_02920W_A	-2.26043	0.000766	3637878
DOT5	-2.26048	0.000646	3640519
CKS1	-2.26504	0.001496	3647732
C5_00140C_A	-2.26738	0.038809	3647873
C3_01090W_A	-2.27529	0.01094	30515176
TIM17	-2.27613	0.010049	3647790
C3_04790W_A	-2.27968	0.009572	3635215
HCH1	-2.28158	0.028139	3641626
HAP43	-2.28195	0.000142	3644531
C5_00100C_A	-2.28729	0.009155	3647883
C4_06420W_A	-2.28852	0.001004	3642795
VPS13	-2.28852	0.000229	3646528
C1_09850C_A	-2.29178	0.001809	3634849
C4_04510W_A	-2.30344	0.001289	3641120

GCN1	-2.30698	0.000351	3641256
C3_07940W_A	-2.31034	0.000551	3647453
C5_00820W_A	-2.31463	0.002017	3644128
CR_09310W_A	-2.31609	0.003553	3641923
C3_04920C_A	-2.3171	8.75E-05	3635268
NBN1	-2.31858	0.000628	3637410
C1_03400W_A	-2.31875	0.00019	3636904
SOD6	-2.32047	0.012553	3638899
FET3	-2.3205	0.014358	3647153
C7_04150W_A	-2.32126	0.014204	3643128
C2_06090W_A	-2.3227	0.001366	3644097
SPL1	-2.32553	0.000766	3638035
CDC37	-2.3311	0.000981	3639210
C5_00030W_A	-2.33475	0.001495	3647879
CAP1	-2.33519	4.51E-05	3636640
TRM12	-2.33686	0.014122	3641738
C2_02520W_A	-2.33752	0.002211	3635818
PHO86	-2.34288	0.005015	3642323
C6_04400W_A	-2.34639	0.036607	3645949
SSU72	-2.3476	0.004143	3645129
CSC25	-2.35155	0.000295	3645660
C7_02110W_A	-2.35321	5.95E-05	3637022
C1_11610C_A	-2.3534	0.037387	3645345
CR_10420W_A	-2.35362	0.000166	3638974
C1_00530C_A	-2.35487	0.001002	3639368
C5_02730C_A	-2.35558	0.010861	3637738
C1_01850C_A	-2.35623	0.00099	3645431
CR_10040W_A	-2.35697	0.00643	3639034
TFB3	-2.35761	0.007053	3644115
C2_00940W_A	-2.36001	0.000648	3638891
C6_00360C_A	-2.36136	0.013501	3646083
DCK2	-2.36222	0.001297	3637384
CR_08200C_A	-2.36408	0.000657	3642087
C1_11770C_A	-2.36597	4.44E-05	3645327
CR_02460W_A	-2.36656	0.000494	3644922
C2_10850C_A	-2.36711	0.000848	3641232
ATG15	-2.37058	0.000502	3641641
C2_04770W_A	-2.37255	0.011059	3647783
GCA1	-2.37395	0.002168	3634903
PUS4	-2.38002	0.001388	3642661
CNH1	-2.38147	0.001149	3648056
C6_02720C_A	-2.38305	8.75E-05	3639212
PGA7	-2.3867	0.00028	3645006
C4_05740C_A	-2.38795	0.000813	3641806
C7_00350C_A	-2.39224	0.000236	3638039
RSN1	-2.3933	8.38E-05	3638308
CKA1	-2.39872	0.000103	3639018

C3_03110W_A	-2.39883	0.014774	3638400
NAB3	-2.40882	0.000537	3639209
CR_05440W_A	-2.41179	0.000503	3648245
C1_10230C_A	-2.4158	4.04E-05	3634880
MHP1	-2.41592	0.005738	3642460
HEM1	-2.41782	0.000146	3640149
VPS24	-2.41799	0.000286	3638892
PAM16	-2.41811	0.000483	3643321
C1_11290W_A	-2.41911	0.005422	3644553
GRX3	-2.42013	0.000513	3637858
C1_10200C_A	-2.42087	0.006034	3634877
MRPL37	-2.42255	0.030509	3644782
C7_03970C_A	-2.42785	0.000619	3643117
CR_05640C_A	-2.42938	0.026355	3646690
CEF1	-2.43063	0.000396	3634808
LIG1	-2.43093	0.000425	3636947
FHL1	-2.43277	0.000702	3643493
GAR1	-2.43408	0.000853	3645332
C3_03330C_A	-2.43681	0.000563	3638364
C1_01530C_A	-2.43742	0.000279	3640232
HAP41	-2.44162	0.003058	3647405
C1_07980C_A	-2.44512	0.000772	3640696
C7_00630C_A	-2.44804	0.024387	3638028
C2_07060W_A	-2.45006	0.02037	3643500
C4_04660C_A	-2.45173	0.000334	3641150
SHA3	-2.45521	0.002326	3644580
ARG81	-2.45541	0.001592	3634775
HTS1	-2.45721	4.91E-05	3636532
MLS1	-2.46076	0.004912	3634875
CCH1	-2.46386	0.000733	3640175
C1_14580C_A	-2.46437	0.000331	3643290
C6_03380W_A	-2.46634	0.000795	3639231
C3_04260W_A	-2.4669	0.00144	3635140
TOK1	-2.46701	0.000703	3645754
CR_00820C_A	-2.47572	0.001963	3640413
EAF3	-2.47754	0.007418	3648318
C3_07920W_A	-2.47958	0.000216	30515226
C4_06410W_A	-2.48027	0.012718	3642796
KRE6	-2.48093	0.000219	3641756
SGE1	-2.48377	0.003077	3642614
ELC1	-2.48452	0.036765	3646151
C6_03290W_A	-2.48645	0.000137	3639195
SFT1	-2.48682	0.009478	30515372
PHHB	-2.48775	0.000172	3638871
CR_02800C_A	-2.48895	0.000156	3642559
PRB1	-2.48998	8.9E-05	3643521
PEL1	-2.49044	0.005989	3637989

HMX1	-2.49112	0.000178	3639294
SLM2	-2.49224	4.45E-05	3647559
C1_12610W_A	-2.49266	0.000292	3646825
C3_05380W_A	-2.49281	0.020284	3643679
POP2	-2.49518	0.000305	3640301
C2_08950W_A	-2.4956	0.000135	3645788
FET34	-2.49604	0.003409	3647131
GCD6	-2.49644	0.00073	3646510
CR_00040C_A	-2.49981	0.000214	3639675
HGT4	-2.50122	4.9E-05	3635269
ARG1	-2.50303	0.0097	3639691
C1_03050W_A	-2.50515	0.002546	3636843
ROD1	-2.50521	0.000346	3635763
C6_00980C_A	-2.51033	0.000552	3644046
LPI9	-2.51044	0.000567	3637060
C5_05000C_A	-2.51123	0.001453	3636539
C1_14190C_A	-2.51737	0.000204	3643300
C5_00260W_A	-2.52761	0.000156	3641561
C7_03000C_A	-2.53129	0.002518	3641383
DAL1	-2.53344	0.021462	3640564
SFT2	-2.53384	0.001694	3644442
C2_03500W_A	-2.53697	4.98E-05	3637345
C1_01220C_A	-2.53751	0.005623	3640210
C1_06660W_A	-2.53782	4.4E-05	3639542
C7_01370W_A	-2.54058	0.027143	3645901
PTP2	-2.54446	0.000264	3647858
C5_04170W_A	-2.54525	1.23E-05	3636543
PMC1	-2.54749	0.000386	3636627
C1_14480W_A	-2.54753	0.004493	3643328
C5_00390C_A	-2.54832	0.002686	3641552
C2_09980W_A	-2.55098	0.000303	3643965
BLM3	-2.55234	1.04E-05	3642310
HGT8	-2.55295	0.004531	3638875
ZSF1	-2.55945	0.000305	3641257
SPT5	-2.561	0.000211	3635847
ANT1	-2.56357	0.034352	3639516
YCP4	-2.56445	0.002148	3648032
CR_00750C_A	-2.56665	0.000322	3640438
CR_10060W_A	-2.56665	0.00051	3639035
C1_00510W_A	-2.56721	0.005183	3639311
C7_04290W_A	-2.57162	0.00754	3643172
C2_10050W_A	-2.57252	5.2E-06	3643949
C7_01280C_A	-2.57451	0.003251	3645909
C5_01930W_A	-2.57536	0.00261	3643741
HAL9	-2.57738	0.001104	3643757
ZCF35	-2.57784	0.001018	3641740
C3_02420C_A	-2.58205	0.005497	3638342

LPG20	-2.58489	0.00585	3644780
CR_08400C_A	-2.58549	0.000925	3642115
STI1	-2.59345	3.86E-05	3643758
CDR11	-2.5968	0.002445	3647389
EFG1	-2.59995	0.00039	3644145
C4_06680C_A	-2.60031	0.000187	3642950
C3_07380W_A	-2.60547	0.001524	3638473
C5_03150W_A	-2.60578	0.001358	3637799
PTC5	-2.61013	6.1E-06	3642072
C5_00160W_A	-2.61236	0.001601	3647871
RIM21	-2.62041	0.00149	3643739
GPT1	-2.62161	0.031926	3647671
PUT1	-2.62237	0.003281	3637779
C6_01620W_A	-2.62279	0.000559	3641647
C1_10620W_A	-2.62288	0.000234	3647682
CR_04370W_A	-2.62305	0.00597	3641004
C4_05260W_A	-2.62348	0.003066	3648282
STF2	-2.62702	0.000918	30515083
C7_01150W_A	-2.62769	0.007143	3645913
SLR1	-2.63273	0.000256	3643957
MLP1	-2.63314	0.000246	3635688
NAG6	-2.63367	0.000412	3645961
NUP49	-2.63652	6.31E-05	3636173
PDX3	-2.63897	0.00108	3640949
C2_02770W_A	-2.63987	6.15E-06	3643031
C3_06830C_A	-2.64175	0.000771	3638543
C2_10600W_A	-2.64328	0.001645	3641245
CR_07250C_A	-2.64537	0.003605	3646028
DAO2	-2.65003	0.00259	3646797
IFR1	-2.65038	0.023938	3643930
CR_08040W_A	-2.65203	0.000607	3644135
CR_03230W_A	-2.65403	0.004023	3645127
C7_01490W_A	-2.65719	0.005179	3637051
C1_10080W_A	-2.66022	0.008374	3634814
CR_07160C_A	-2.6615	0.005	3648347
C4_02110W_A	-2.67399	0.001134	3635673
C5_00780C_A	-2.67527	0.000622	3644124
CR_00980C_A	-2.67898	5.67E-06	3640426
C5_00800C_A	-2.68439	0.000455	3644126
C3_06600C_A	-2.6894	0.002447	3642280
C3_03070W_A	-2.69874	0.003736	3638396
CR_10760C_A	-2.69918	0.003362	3638943
PRP22	-2.70071	0.000173	3636404
LYS144	-2.70402	0.000473	3640512
HNM1	-2.70478	0.000701	3638857
C4_03140C_A	-2.70698	0.006142	3637843
YAH1	-2.70963	0.00212	3638368

C3_07490W_A	-2.71894	4.26E-05	3638528
GCN3	-2.72422	0.001021	3645905
C2_10140W_A	-2.72453	0.020718	3643934
STP2	-2.72474	0.001275	3636214
PTR3	-2.72569	0.002445	3645428
C2_03950W_A	-2.72634	0.001981	3637420
CR_08420W_A	-2.72714	0.000355	3642117
CR_10640W_A	-2.73141	0.015705	3639014
ATX1	-2.73523	0.004834	30515426
DUR32	-2.73554	0.005395	3636142
C3_00840C_A	-2.73596	0.007131	3647758
C2_09790W_A	-2.73701	0.00199	3643804
MNN4-4	-2.73877	0.006316	3639250
C2_07800W_A	-2.73896	0.025054	3642325
C3_06420C_A	-2.74248	0.000342	3642262
NMA111	-2.74364	3.32E-05	3640179
BMT5	-2.74573	3.26E-06	3635781
MET16	-2.75009	0.012622	3642964
C4_03000C_A	-2.77233	4.94E-05	3637935
FCY24	-2.77369	0.021022	3641902
C1_07560W_A	-2.77784	0.000668	3646676
C6_01350W_A	-2.78178	0.000474	3641642
C6_03220W_A	-2.79693	0.003645	3639179
C2_07580W_A	-2.79748	0.004684	3645216
C1_04120C_A	-2.79938	0.009459	3644701
CR_08330W_A	-2.80846	0.005324	3642080
C1_05540C_A	-2.81667	0.002985	3642503
RPP1	-2.81759	0.013673	3644681
C2_10540W_A	-2.82085	0.000127	3641200
OYE2	-2.82098	0.000225	3641603
PDR6	-2.82161	1.6E-05	3638824
HSK3	-2.82286	0.001562	3635730
C3_03670W_A	-2.82529	9.15E-06	3645649
C6_02690C_A	-2.82539	5.41E-05	3639228
C2_07640W_A	-2.82725	6.27E-05	3645192
MIT1	-2.83051	2.87E-05	3644089
C3_04940W_A	-2.83355	5.42E-05	3635270
C3_06820C_A	-2.83906	1.26E-06	3638544
CR_01370C_A	-2.84015	6.35E-05	3640168
C5_03010W_A	-2.84059	0.003724	3637819
C1_09840C_A	-2.84687	2.52E-06	3634848
C3_03310C_A	-2.84925	0.009934	3638363
CR_04820W_A	-2.85225	5.34E-05	3647298
SDS24	-2.8621	0.000776	3640756
AAF1	-2.8654	7.32E-07	3642284
C6_04420W_A	-2.86576	1.66E-05	3645951
OPT8	-2.86841	0.013982	3640266

C4_04500C_A	-2.87168	0.016731	3641123
C2_09240C_A	-2.8726	0.003796	3644064
CR_07510W_A	-2.87486	0.013059	3647998
C1_08700W_A	-2.87685	3.78E-05	3634936
MNL1	-2.87936	4.4E-06	3646050
C4_02690W_A	-2.8825	7.39E-07	3637861
CR_09610C_A	-2.88558	0.0002	3647094
TRY2	-2.88787	0.000422	3647670
C2_09360W_A	-2.8882	0.002168	3644109
HMT1	-2.8884	0.000245	3640182
CDR1	-2.89022	0.000249	3635237
MOH1	-2.89095	0.000393	3646809
C2_06620W_A	-2.89132	0.011184	3646564
YTH1	-2.89239	1.68E-06	3645572
SRR1	-2.89241	0.014524	3647589
C4_04550C_A	-2.90115	0.000161	3641106
C6_00700C_A	-2.90185	0.000193	3647074
BMT9	-2.90293	6.3E-06	3635654
PEX5	-2.90346	4.55E-06	3645026
C1_11110C_A	-2.90381	0.017472	3644413
TBF1	-2.91108	4.38E-05	3637327
CR_03260W_A	-2.91272	4.05E-06	30515389
C3_02030W_A	-2.91783	0.001166	3636580
ZCF21	-2.91881	1.32E-05	3645762
CR_01570W_A	-2.91992	0.049404	3640077
CHA1	-2.92025	0.037262	3638791
C2_04850C_A	-2.92064	9.91E-05	3648114
BUB3	-2.9214	7.75E-07	3646362
C4_03930C_A	-2.92144	0.009355	30515262
C2_07680W_A	-2.92332	0.00021	3642339
C1_11580W_A	-2.92401	1.07E-05	3645330
SSY5	-2.92716	4.06E-07	3642140
CKA2	-2.92932	9.28E-05	3643444
RCA1	-2.9334	8.75E-05	3639303
C3_07700W_A	-2.93357	0.000522	3638457
OFD1	-2.93458	0.000286	3646962
C2_07040W_A	-2.93633	0.000197	3643499
C3_00950C_A	-2.93736	0.004791	3639852
YCF1	-2.93812	1.91E-05	3636934
CDC34	-2.93904	3.01E-05	3637028
LIG4	-2.94896	0.000137	3642986
EST1	-2.95081	1.87E-05	3636526
PGA41	-2.95308	0.027761	3642800
C1_04820C_A	-2.96152	0.004284	3644787
C3_04380C_A	-2.96703	0.000187	3635173
PNC1	-2.97095	0.000302	3646232
C2_06650C_A	-2.97199	0.015096	3648419

CYM1	-2.97658	0.000282	3642244
C3_02750W_A	-2.97711	0.012543	3638411
UGA1	-2.98112	0.000824	3637329
C3_07600W_A	-2.98393	0.004833	3638495
C3_02980C_A	-2.98616	0.002533	3638386
C3_01850W_A	-2.99076	0.000735	3636565
GNP3	-2.99303	0.001093	3639027
CR_03870W_A	-2.99366	0.005946	3643187
C3_00300W_A	-2.99384	0.000111	3640524
MEF2	-2.99404	0.000375	3639564
C2_05670C_A	-2.99466	0.0048	3645570
CR_10810C_A	-2.99533	0.000125	3638949
MNN14	-3.00023	1.66E-05	3643651
CR_09920W_A	-3.01287	0.001399	3639028
FZO1	-3.01537	4.93E-05	3646848
C7_02550C_A	-3.01865	0.000148	3637018
C2_01820C_A	-3.02189	0.000931	3635831
MRPL40	-3.02199	2.94E-05	3640968
C2_06200C_A	-3.02561	0.00114	3646908
C5_04050W_A	-3.02567	1.67E-07	3647735
C1_05210C_A	-3.02593	0.000369	3642465
MAS1	-3.02705	9.72E-06	3636855
CPP1	-3.03313	0.000119	3634865
C1_00540C_A	-3.03398	0.000128	3639367
GIR2	-3.03515	0.000115	3636962
C7_03880C_A	-3.03866	0.024219	3643142
C4_02330C_A	-3.04236	0.011394	3637884
DUR4	-3.04723	0.007007	3645012
C4_03750C_A	-3.04926	0.010428	3645356
C1_03040W_A	-3.05027	0.003357	3636842
DAL5	-3.05167	0.001654	3638401
RPL7	-3.06837	0.000703	6335362
C3_05850W_A	-3.07796	8.85E-05	3641758
C5_00130C_A	-3.08309	1.1E-05	3647881
IML1	-3.09068	4.02E-06	3636813
C1_02090C_A	-3.09796	0.002234	3644577
RPC53	-3.10171	0.001554	3637898
C2_07000W_A	-3.10984	0.000377	3643534
C2_03830W_A	-3.11521	0.001834	3637399
UGA6	-3.11878	0.005162	3642975
TIF3	-3.12157	8.11E-06	3641646
C6_00640C_A	-3.12159	0.000617	3647134
HRR25	-3.12484	1.01E-05	3645878
DFG16	-3.13562	1.4E-05	3637413
C1_06910C_A	-3.14023	6.61E-05	3639526
C1_05160C_A	-3.15345	0.000518	3642468
ISU1	-3.15652	3.29E-06	3637007

C1_02960C_A	-3.15843	2.51E-06	3636834
TRM1	-3.15852	0.002036	3640396
ATO10	-3.16076	0.024811	3640394
C6_03000C_A	-3.16094	0.033035	3639194
NMD5	-3.16806	5.98E-05	3645745
C2_07920W_A	-3.17078	0.002805	3642294
TRY3	-3.17872	1.69E-05	3642652
MSU1	-3.1817	0.009272	3643919
LEU3	-3.18304	1.05E-05	3637757
SFL1	-3.18333	6.9E-06	3642498
C2_01540W_A	-3.18548	0.000373	3635804
C2_02280W_A	-3.18824	3.03E-06	3635806
C5_04350C_A	-3.18994	0.000282	3636482
ITR1	-3.19034	1.76E-07	3643440
C2_01370C_A	-3.19347	7.26E-06	3635723
C1_01890C_A	-3.20839	2.02E-07	3645427
GCD7	-3.21855	3.58E-06	3637367
C4_04460C_A	-3.22241	3.92E-08	3641127
C7_01660C_A	-3.22968	7.66E-06	3636983
ROT1	-3.23694	0.001759	3639350
CR_07680C_A	-3.23718	0.01217	3648193
C6_02420W_A	-3.23832	0.000239	3645885
DOT6	-3.23928	5.68E-06	3640078
NHP2	-3.2393	5.86E-05	3641003
CR_01440C_A	-3.24507	2.3E-06	30515374
CR_08410W_A	-3.2493	6.31E-05	3642116
C6_01980C_A	-3.25045	6.31E-05	3644560
RAT1	-3.25097	1.01E-06	3635686
CPA2	-3.25207	4.71E-05	3640385
PDR16	-3.25555	0.004117	3644679
CR_07670W_A	-3.26751	2.02E-05	3647255
HOS3	-3.281	6.71E-07	3637886
DRE2	-3.28172	2.54E-06	3642571
OLE2	-3.28568	1.01E-06	3643503
CNT	-3.28831	1.11E-05	3644071
CR_07940W_A	-3.28835	0.001903	3644139
C3_07580W_A	-3.29919	0.000124	30515225
CRP1	-3.29992	0.000311	3634826
CWC22	-3.29993	0.004438	3643938
C1_06840C_A	-3.31082	2.4E-05	30515012
C3_07280C_A	-3.31185	1.81E-05	3638431
HIP1	-3.31533	3.3E-06	3643761
MRPL36	-3.31906	3.11E-06	3643733
C4_00100C_A	-3.32618	1.05E-08	3645004
BGL22	-3.32756	0.000368	3641935
C1_06470W_A	-3.33027	9.55E-09	30515007
C5_01310W_A	-3.33079	0.036167	3642645

PRS1	-3.33354	6.88E-06	3635817
C1_05650W_A	-3.33734	0.001174	3639838
C6_03780C_A	-3.34161	8.13E-06	3640308
C3_07100C_A	-3.34886	1.02E-06	3638443
C3_00940W_A	-3.35051	1.12E-08	3639851
SET2	-3.35211	2.12E-06	3643923
CR_03910C_A	-3.35966	8.72E-07	3640954
CR_05910W_A	-3.36001	0.000395	3646680
MRPL27	-3.36014	0.000117	3636884
CR_08190W_A	-3.3635	0.013493	3642084
RPC40	-3.36789	5.74E-05	3643427
C1_06800W_A	-3.37515	0.000427	3639496
NOT5	-3.38248	2.45E-06	3640763
C3_05250C_A	-3.38738	3.42E-06	3635199
CR_03660C_A	-3.38959	1.52E-05	3643219
C2_00200W_A	-3.39962	5.48E-05	3638903
CEM1	-3.40094	0.005616	3635242
C1_08240C_A	-3.40105	0.001649	3640759
C1_10120C_A	-3.40423	1.14E-06	3634779
C1_05200C_A	-3.40696	4.49E-06	3642466
C2_05980C_A	-3.40735	0.000616	3644075
C6_04180W_A	-3.41082	4.15E-07	3648176
C4_03020W_A	-3.41315	2.13E-06	3637934
C4_00460C_A	-3.41808	3.95E-08	3645010
C5_03700C_A	-3.42176	0.001598	3646990
C3_00500C_A	-3.4219	0.000112	3640515
C6_01550C_A	-3.42425	0.00011	3641653
C2_00540W_A	-3.4343	2.57E-07	3638865
CUP2	-3.43738	4.07E-07	3636189
C4_07100C_A	-3.43936	8.15E-07	3642903
SAP9	-3.44196	9.03E-05	3645625
MRV4	-3.44444	0.006046	3636547
GAP4	-3.44534	0.000183	3644463
MSS51	-3.45399	2.63E-07	3639031
C1_01140C_A	-3.45738	1.05E-06	3640202
ISW2	-3.4664	1.81E-05	3642235
CR_07910C_A	-3.4679	2.58E-07	3644142
C1_07640C_A	-3.47208	0.000605	3646659
C1_10140C_A	-3.48105	0.000393	3634780
SUI3	-3.48361	3.29E-07	3643130
C6_02980C_A	-3.48399	1.63E-06	3639192
C3_00160C_A	-3.4852	1.62E-05	3640591
TPT1	-3.48764	4.42E-05	3640543
OPY2	-3.48773	2.32E-06	3640765
TIM23	-3.4955	6.54E-05	3643777
C3_07900C_A	-3.50073	8.66E-05	3647450
CR_01780W_A	-3.50173	0.001923	3640157

MUC1	-3.50738	6.57E-09	3645771
HAP2	-3.51174	8.21E-07	3641800
RPC19	-3.51764	0.027518	3647693
C2_00220C_A	-3.52134	8.56E-06	3638902
C2_08850C_A	-3.52145	1.45E-07	3643890
RTG3	-3.5247	2.3E-07	3644423
C4_07170C_A	-3.53499	0.001061	30515289
C2_08920W_A	-3.53992	5.81E-07	3645797
ABC1	-3.55363	7.53E-05	3640239
C2_10170C_A	-3.55552	7.81E-07	3643931
PRP39	-3.55986	0.004176	3635832
FRP1	-3.56298	7.02E-10	3645005
C4_07010C_A	-3.57861	0.002668	3642966
CFL1	-3.5789	0.002482	3641810
C1_09540W_A	-3.58036	5.67E-05	3634885
C5_04120C_A	-3.58359	1.43E-05	3636522
IMH3	-3.5862	2.08E-06	3646556
RNR22	-3.58645	1.86E-07	3645217
YME1	-3.59605	4.77E-05	3641816
PGA55	-3.60323	1.31E-06	3645784
KRE30	-3.60328	1.74E-06	3642311
SEF1	-3.61071	6.16E-09	3644945
C7_02120C_A	-3.62326	0.0003	3637019
CCS1	-3.62945	5.01E-07	3644476
CR_09140C_A	-3.63197	5.75E-05	3641879
C3_00620C_A	-3.641	4.65E-06	30515171
TPK1	-3.64112	2.98E-07	3634879
SAM35	-3.64331	1.62E-05	3643329
C1_09390W_A	-3.65192	0.000283	3634851
C3_07430W_A	-3.66136	2.03E-07	3638484
C4_04010W_A	-3.66584	0.000282	3641218
CR_02970C_A	-3.66887	2.84E-07	3642603
C1_05250W_A	-3.67777	0.001305	3642462
CR_02700W_A	-3.69512	0.022669	3642575
SIK1	-3.69514	2.09E-07	3638925
CSP1	-3.69584	0.024946	3648242
C3_07290W_A	-3.6967	2.58E-07	3638430
CR_06970C_A	-3.70464	6.38E-06	3645736
TOR1	-3.70648	1.55E-07	3643480
URA7	-3.71008	8.02E-09	3636510
C5_03470C_A	-3.71287	0.000499	3646963
C2_03550C_A	-3.725	4.17E-07	3637338
TRK1	-3.7288	3.68E-10	3644153
PSO2	-3.72966	0.000866	3641241
C1_01580W_A	-3.73058	1.51E-05	3640222
C5_00570W_A	-3.73378	8.71E-07	3641589
C7_03380W_A	-3.74366	3.24E-05	30515359

C3_01520C_A	-3.74543	9.99E-07	3636570
CR_08680C_A	-3.75501	1.7E-09	3642127
C1_11900C_A	-3.75511	0.000103	3642005
SPO72	-3.77011	7.03E-10	3644072
C6_02900C_A	-3.77064	0.000817	3639240
COX19	-3.77782	7.93E-06	3636220
CR_03840C_A	-3.7835	0.029449	3643184
C1_00520W_A	-3.7903	1.17E-05	3639310
ARG8	-3.80444	3.18E-07	3641131
AUT7	-3.8101	2.51E-07	30514999
SNT1	-3.8407	6.66E-11	3642033
CR_03110W_A	-3.84157	0.000876	3645124
C3_02920W_A	-3.85093	1.25E-10	3638379
C7_02660C_A	-3.85623	0.010271	3647652
PRS5	-3.85968	2.3E-09	3642296
NBP2	-3.86267	6.92E-07	3636963
HST2	-3.86384	0.000221	3640162
TCO89	-3.87277	3.83E-08	3644788
CR_03250C_A	-3.87713	9.34E-08	3645102
MSN4	-3.89281	6.39E-07	3634911
VPS21	-3.89616	2.61E-11	3642092
C2_07410W_A	-3.89667	2.05E-05	3645203
TRY4	-3.8993	1.54E-05	3635240
DOT4	-3.90149	3.32E-07	3646810
C5_03640W_A	-3.90765	1.51E-05	3646984
RPD3	-3.9088	2.97E-08	3642580
BAS1	-3.91153	1.31E-08	3641136
C2_05400W_A	-3.91696	0.00667	3643435
C1_05230W_A	-3.91696	0.006758	3642463
C3_01130C_A	-3.92303	1.76E-08	3639901
C1_13880C_A	-3.92448	5.71E-07	3636133
C1_08710W_A	-3.94841	6.58E-05	3634937
PCL5	-3.951	7.75E-08	3636466
C1_14350W_A	-3.95302	3.54E-08	3643281
NAR1	-3.9628	1.24E-07	3634916
IFM1	-3.96872	1.05E-07	3641389
C3_05750C_A	-3.96904	0.004803	3641719
CR_02930W_A	-3.9759	2.46E-11	3642588
C3_03300C_A	-3.98178	4.17E-09	3638361
C2_02110C_A	-3.98768	0.000889	3635745
ICL1	-3.99447	0.004022	3646146
REG1	-3.99561	8.72E-06	3638858
C6_00170C_A	-3.99784	1.32E-11	3647903
C1_09650W_A	-4.007	1.56E-06	3634870
C4_01280C_A	-4.01732	1.05E-05	3635700
C2_00170C_A	-4.02133	4.19E-07	3638906
NPR2	-4.02898	1.08E-05	3638360

CR_06290C_A	-4.03169	1.15E-08	3644856
C3_07370W_A	-4.03553	8.37E-07	3638474
C1_10580C_A	-4.05392	0.006082	3647679
C5_03030W_A	-4.05946	0.000265	3637822
C2_06800C_A	-4.06198	1.9E-05	3643518
C3_05440C_A	-4.06718	0.000466	3643668
RFG1	-4.06892	2.29E-11	3642570
C2_02120W_A	-4.08166	2.28E-05	3635746
BUD23	-4.08413	0.00128	3642629
C5_01470C_A	-4.08532	0.001278	3647050
PGA13	-4.08739	0.032004	3642138
DUS4	-4.09689	3.93E-07	3641580
C6_03460W_A	-4.10028	6.84E-12	3640252
C1_05370C_A	-4.10637	6.2E-07	3642481
C6_04510C_A	-4.10723	1.73E-06	3645955
UGT51C1	-4.11477	2.38E-09	3640147
C2_00840W_A	-4.12193	6.4E-05	3638807
C2_08030W_A	-4.13144	2.01E-05	3642309
AGP3	-4.13147	0.001281	3641163
CR_04160C_A	-4.13631	3.27E-08	3640976
DAL8	-4.13906	4.89E-05	3635181
C1_05790W_A	-4.13931	8.27E-05	30515002
C5_04980W_A	-4.13984	2.68E-11	3636536
C4_03580W_A	-4.14468	0.001525	3645369
C1_08890C_A	-4.14914	0.013932	3634924
MTR2	-4.1502	1.59E-08	30515167
C1_05360C_A	-4.15552	9.83E-07	3642482
NDT80	-4.16856	3.4E-08	3638840
C5_02680W_A	-4.17024	2.6E-09	3637774
RIT1	-4.17354	0.000221	3638492
C3_03370C_A	-4.18369	2.19E-06	3638366
SET6	-4.18542	0.000338	3644590
C2_00810C_A	-4.22204	0.000242	3638810
C2_08860W_A	-4.22476	2.06E-10	3645776
C4_04560C_A	-4.23201	1.67E-08	3641105
C1_03140W_A	-4.23451	1.09E-06	3636929
RPF1	-4.24543	6.99E-07	3637890
C3_02630C_A	-4.24573	1.11E-06	3638328
C6_02660C_A	-4.24797	0.000452	3639224
C1_11200W_A	-4.24896	5.09E-05	3644396
C2_00090W_A	-4.25326	3.73E-05	3647910
LIP4	-4.26016	1.47E-06	3645987
C2_06570C_A	-4.27938	1.43E-09	3646561
CR_07480W_A	-4.27939	2.28E-07	3646047
C1_06770W_A	-4.29354	9.39E-09	3639504
C2_09000C_A	-4.29482	0.005197	3645782
C4_01090C_A	-4.29594	0.003777	3635685

HSX11	-4.31758	0.001684	3635660
ADE8	-4.32019	1.6E-06	3642999
C7_03590C_A	-4.32359	1.17E-05	3646239
C2_02270C_A	-4.3386	2.24E-06	3635805
DEF1	-4.34301	1.91E-06	3639023
C7_04160W_A	-4.3534	1.53E-05	3643127
RHD1	-4.35511	0.001015	3647531
EHT1	-4.36429	1.55E-07	3636894
C2_02300W_A	-4.36645	7.5E-06	3635808
CR_02510W_A	-4.38048	1.12E-12	3647694
C1_07130C_A	-4.41262	1.57E-08	3644462
PUF3	-4.4131	4.46E-08	3646950
SDH4	-4.42113	0.000313	3636497
CR_03760W_A	-4.43035	0.000183	3643178
C6_02380W_A	-4.44135	2.05E-12	3645883
C2_02820C_A	-4.444	7.69E-07	3643027
SBA1	-4.44477	5.07E-09	3640305
C1_13330C_A	-4.45536	8.88E-09	3636150
C4_05970W_A	-4.45728	2.78E-10	3646514
CTR2	-4.45804	2.32E-09	3648427
C7_00340C_A	-4.47901	1.41E-12	3638040
TCC1	-4.48275	9.17E-12	3638524
C7_03400C_A	-4.48732	2.05E-10	3646717
C7_02460C_A	-4.50047	1.77E-10	3636946
C5_04080C_A	-4.52145	3.75E-08	3647731
C5_04290C_A	-4.52578	1.14E-07	3636455
C4_01960C_A	-4.52708	2.24E-10	3635664
C3_05450C_A	-4.53678	4.25E-10	3643669
ZCF29	-4.56571	1.66E-09	3641370
BUD5	-4.57391	1.03E-07	3647315
C2_09570C_A	-4.59744	0.000642	3643811
C5_02010C_A	-4.60603	2.37E-10	3643772
ALR1	-4.61106	4.3E-09	3636616
C6_02970C_A	-4.61295	5.26E-07	3639190
C3_04620C_A	-4.61401	0.000111	3635155
C5_03410C_A	-4.62102	1.6E-06	3646347
C6_03390W_A	-4.62141	5.5E-06	3639232
ATG1	-4.62266	2.79E-08	3641175
ATG9	-4.62335	1.43E-07	3635239
C2_07280W_A	-4.62551	0.00617	3643475
ISA2	-4.64231	1.31E-09	3638513
URK1	-4.64293	1.76E-08	3635186
C1_01630W_A	-4.64651	1.45E-10	3640194
CR_00670C_A	-4.64977	9.15E-05	3639655
C5_04990W_A	-4.66295	0.000497	3636538
C6_02020C_A	-4.67266	3.6E-10	3647563
EPL1	-4.67279	1.73E-13	3639732

C2_07250C_A	-4.67307	0.00198	3643487
C4_07060W_A	-4.6767	7.73E-11	3642908
PFK26	-4.68109	9.62E-12	3634912
C2_05080C_A	-4.68904	0.004006	3643453
PRA1	-4.68967	0.003081	3642969
YOR1	-4.69005	1.94E-09	3643964
C7_03270W_A	-4.69189	9.21E-09	3641365
C1_06090C_A	-4.6924	4.8E-09	3639822
IFF6	-4.70151	1.01E-07	3644087
C4_02520C_A	-4.70338	2E-09	3637863
C1_08840W_A	-4.71688	0.007301	3634919
STE11	-4.71883	7.02E-13	3637369
C4_03230C_A	-4.73062	0.012645	3637836
CR_01950W_A	-4.73901	3.97E-07	3640107
C2_01070W_A	-4.75112	3.65E-07	3638834
SIP5	-4.75135	1.21E-08	3639865
C1_11880W_A	-4.76027	2.62E-06	3642007
TPO4	-4.76973	2.15E-08	3640956
HBR1	-4.77558	1.22E-09	3639295
CR_03160W_A	-4.77673	8.87E-06	3645117
MET2	-4.78065	6.64E-08	3640070
C7_03230C_A	-4.78392	0.033366	3641369
CCC2	-4.8188	1.59E-13	3637821
CR_07540C_A	-4.82935	0.043409	3648001
C6_03280W_A	-4.82937	0.041643	3639185
MDM34	-4.83306	5.33E-09	3647517
C4_06850C_A	-4.85115	2.32E-09	3642912
C4_02090C_A	-4.85586	6.72E-10	3635642
C3_06700C_A	-4.86346	6.6E-12	3642247
CR_06450W_A	-4.86944	0.000143	3645279
NOP1	-4.87102	6.97E-11	3642947
C3_07800C_A	-4.87366	3.3E-07	3647461
C3_04370C_A	-4.87767	9.31E-08	3635172
SLD1	-4.89395	4.23E-07	3638406
C3_00030C_A	-4.89524	3.08E-07	3640571
CR_06840W_A	-4.90535	2.3E-08	3647322
YPT53	-4.92036	3.26E-08	3642483
RGA2	-4.92818	3.78E-15	3635661
CYC3	-4.95854	1.04E-10	3642621
GZF3	-4.98552	9.2E-11	3642563
C5_02980C_A	-4.99006	1.1E-07	3637816
LEU42	-5.0055	6.69E-10	3643808
C7_01210C_A	-5.03616	1.4E-09	3645904
FLO8	-5.04059	4.93E-11	3646292
CR_08650C_A	-5.06407	1.05E-13	3642124
FGR23	-5.07334	3.51E-05	3636555
C5_02640W_A	-5.07854	1.31E-07	3637785

JEN2	-5.07889	0.007398	3641214
SSY1	-5.08057	4.92E-12	3637382
C1_00860W_A	-5.0815	4.86E-06	3639358
C2_02960C_A	-5.08737	2.59E-07	3643011
HSP104	-5.10592	7.06E-07	3642097
PAM18	-5.11181	8.32E-08	3645747
HGH1	-5.12811	5.61E-08	3635647
SHY1	-5.14018	4.24E-12	3634824
C5_03740W_A	-5.14708	3.36E-08	3647237
C6_02150C_A	-5.15378	5.38E-05	3645888
C2_09820W_A	-5.1727	1.67E-08	3643801
ZCF30	-5.18164	2.99E-09	3642051
C1_13380W_A	-5.18553	9.22E-05	3636217
PUS7	-5.1973	9.68E-08	3643960
C5_01560C_A	-5.20074	1.97E-08	3647064
SMD2	-5.20258	0.002377	30515133
CR_02890C_A	-5.20529	2.83E-07	3642585
C1_00090W_A	-5.22294	4.36E-05	3639304
C4_01500W_A	-5.22451	1.74E-06	3635617
C7_04140C_A	-5.24103	1.96E-08	3643129
C4_05650W_A	-5.25814	2.99E-06	3641814
C6_01340C_A	-5.25919	3.34E-06	30515331
C1_07530W_A	-5.26694	0.000897	30515025
C7_01020C_A	-5.27163	2.3E-07	3638020
C3_06560W_A	-5.2717	1.5E-14	3642276
CR_10430C_A	-5.27646	0.004099	3638975
C2_01510C_A	-5.27719	0.002432	3635801
C2_02920W_A	-5.28173	3.36E-14	3643016
OYE32	-5.28713	3.38E-09	3642919
CIRT4B	-5.3175	1.24E-14	3642560
VTC4	-5.32248	2.08E-13	3646795
DAC1	-5.32716	3.9E-10	3645967
C6_02290C_A	-5.33441	2.23E-09	3645873
C1_06540C_A	-5.34463	1.37E-08	3639520
C7_02930C_A	-5.36402	9.12E-11	3641390
C5_04530W_A	-5.37127	6.6E-12	3636507
C4_06040W_A	-5.38091	1.91E-05	3646521
PRN1	-5.39341	1.48E-06	3639882
CAG1	-5.40535	3.6E-05	3636469
C2_05060C_A	-5.40613	1.84E-05	3643451
BUL4	-5.4089	8.7E-12	3642037
C2_05270W_A	-5.43284	1.78E-10	3643470
NAG1	-5.43842	2.93E-09	3645966
UTP20	-5.44896	3.89E-11	3647956
NOP5	-5.46182	4.32E-14	3646084
MDN1	-5.46262	6.64E-13	3635614
PDE2	-5.46559	9.83E-14	3636877

C4_06990W_A	-5.47733	2.41E-11	3642968
MLH3	-5.48159	1.99E-09	3643294
C7_03210W_A	-5.48533	1.64E-05	3641371
C2_04520C_A	-5.50447	0.000613	3645447
C7_01360C_A	-5.50949	0.013823	3645900
IFH1	-5.50986	2.67E-15	3637770
C7_02160W_A	-5.51018	5.29E-08	3636994
PWP1	-5.51771	1.83E-09	3635623
C4_05590W_A	-5.53074	2.25E-12	3641776
C2_02710C_A	-5.54602	1.43E-09	3643003
CR_10400W_A	-5.5618	4.08E-12	3638956
C6_00380C_A	-5.57412	2.95E-09	3646085
C4_06200W_A	-5.59519	0.001248	30515282
ZCF3	-5.59545	1.69E-11	3645335
C1_10250C_A	-5.59559	0.000828	3634882
C5_03460C_A	-5.60396	5.53E-06	3646345
BRE1	-5.61396	5.82E-15	3641568
C2_04110W_A	-5.62937	0.000114	3637378
C3_00340W_A	-5.63649	2.27E-05	3640544
C1_14080W_A	-5.63764	1.7E-13	3643345
C1_11320C_A	-5.64424	3.52E-07	30515052
PHR3	-5.68331	2.05E-17	3645003
CR_04600W_A	-5.70353	7.35E-12	3640950
RXT3	-5.75069	1.41E-11	3643431
C5_04840C_A	-5.75271	9.89E-11	3636435
CR_06680C_A	-5.76638	7.56E-08	3646623
C1_09040C_A	-5.76869	2.39E-10	3634769
CR_10280W_A	-5.77571	4.14E-07	3638934
RPA190	-5.78153	1.57E-13	3647677
KNS1	-5.78769	6.5E-09	3636228
C1_09780C_A	-5.78943	3.38E-10	3634842
C1_11080W_A	-5.81774	1.04E-11	3644416
ARO10	-5.84576	9.58E-08	3647320
C1_09320C_A	-5.85034	1.43E-11	3634834
ELP3	-5.85738	1.73E-14	3641747
RRP42	-5.87122	6.48E-05	3647852
C2_01050W_A	-5.93727	0.02549	30515086
FGR46	-5.9377	0.018718	3638989
CR_02600W_A	-5.9377	0.018826	3642596
ISY1	-5.94898	2.34E-06	3646233
C2_03570C_A	-5.96059	1.41E-05	3637336
HIT1	-5.96136	1.27E-05	3637854
PWP2	-5.98848	2.23E-14	3640416
RMT2	-5.9971	2.43E-08	3641569
GYP7	-6.00423	2.46E-15	3646245
C2_05990C_A	-6.0047	0.000726	3644074
RIM2	-6.03518	4.45E-07	3645448

C5_02020C_A	-6.03756	8.13E-13	3643771
TPS2	-6.04752	6.5E-09	3636892
CR_05550C_A	-6.05354	1.01E-13	3647593
WAR1	-6.05552	2.03E-08	3644691
STB3	-6.06455	1.9E-15	3645781
C1_09520C_A	-6.07041	4.73E-17	3634766
C3_02670W_A	-6.07555	1.54E-05	3638405
C1_11160C_A	-6.08382	1.41E-06	3644399
IMP4	-6.0877	1.15E-12	3644138
C1_06510C_A	-6.14296	1.35E-09	3639549
C2_04820W_A	-6.14923	6.61E-08	3647776
FUN31	-6.15188	9.77E-13	3642249
SUA71	-6.16349	2.41E-13	3636879
C5_01730W_A	-6.1741	1.09E-05	3643730
RAD3	-6.18343	2.9E-17	3638014
MDJ1	-6.18356	5.23E-15	3646980
MET1	-6.19167	5.98E-07	3643014
ERO1	-6.19486	9.29E-09	3634810
C1_14380C_A	-6.19672	1.1E-08	3643285
RIO2	-6.20419	8.11E-13	3642066
C1_05270C_A	-6.2196	1.5E-09	3642476
C2_02630W_A	-6.24371	7.21E-06	3635839
FGR50	-6.24768	0.005067	3644859
WHI3	-6.27311	6.45E-14	3636991
C3_01150C_A	-6.27672	2.33E-07	3648401
C6_01590W_A	-6.28213	3.65E-18	3641650
NAG3	-6.2896	3.27E-07	3645969
C4_00260W_A	-6.29752	2.18E-13	3645037
RIM101	-6.32067	1.22E-09	3643280
C3_05140C_A	-6.32215	4.25E-07	3635161
C1_13370W_A	-6.32864	8.59E-05	3636216
C2_09610W_A	-6.33622	4.22E-09	3643798
C3_05860C_A	-6.36066	8.45E-09	3641759
C1_13590W_A	-6.36572	0.021273	3636209
C2_00280C_A	-6.42137	6.93E-08	3638802
C2_05830C_A	-6.44883	3.75E-11	3647483
MNN4	-6.46495	4.47E-16	3642784
PRP8	-6.49636	1.59E-17	3642125
CR_06980W_A	-6.49967	0.000256	3645735
C1_06750W_A	-6.5009	0.000125	3639506
C3_00100W_A	-6.54012	4.42E-09	3640567
C6_04300W_A	-6.56193	4.24E-09	3646286
C1_12750C_A	-6.56421	1.42E-08	3646818
C1_06820W_A	-6.57359	2.64E-13	3639493
ARO80	-6.62582	1.58E-09	3636808
DBP2	-6.63948	2.87E-13	3647692
C4_05160C_A	-6.64284	2.56E-15	3647411

CR_03200C_A	-6.65638	1.57E-09	3645131
SOK1	-6.68934	7.95E-14	3642495
CR_08830W_A	-6.69368	6.15E-05	30515450
PGA48	-6.69837	7.22E-16	3647905
C1_06380C_A	-6.70892	0.000159	3639559
STP4	-6.72165	6.2E-07	3637405
CR_10750C_A	-6.725	9.07E-08	3638942
C7_00590W_A	-6.73112	5.48E-11	3637994
C2_03000C_A	-6.73881	2.22E-08	3643022
RIX7	-6.77388	2.68E-15	3637735
ALS6	-6.78254	9.28E-10	3642266
COX17	-6.79264	1.26E-06	30515088
MTG1	-6.80343	1.29E-07	3645139
C3_07550C_A	-6.81359	6.27E-13	3638525
CR_04220C_A	-6.8394	2.63E-08	3640943
SAP10	-6.84143	1.84E-09	3641126
C1_12570C_A	-6.85962	5.08E-10	3647604
HAP42	-6.86409	1.51E-14	3635729
GCA2	-6.86975	1.24E-14	3647194
C1_13390W_A	-6.89328	8.44E-07	3636218
C4_03740W_A	-6.90647	8.91E-11	3645357
RAS2	-6.91535	4.45E-15	3635211
C2_08700C_A	-6.91684	1.62E-16	3643887
C6_04240W_A	-6.94318	6.09E-06	3646276
GCV2	-6.94972	1.1E-11	3646505
PAN6	-6.95534	3.23E-12	3647684
C2_02420C_A	-6.98212	1.32E-10	3635756
CPA1	-6.98637	2.22E-10	3635715
FAL1	-6.99442	1.25E-10	3639839
C3_00410C_A	-7.00176	0.001824	3640552
CYS3	-7.0018	7.68E-09	3642081
C5_01900C_A	-7.04593	0.008102	30515302
C3_03470W_A	-7.04687	0.008778	3638306
C5_01020C_A	-7.04694	0.008364	30515297
C1_00160C_A	-7.05922	1.37E-13	3639341
C6_03520C_A	-7.07694	0.00246	3640259
C5_03800W_A	-7.09127	1.6E-10	3647230
C4_00750C_A	-7.11042	4.02E-05	3645763
C1_13010W_A	-7.11501	3.66E-09	3636120
CR_03980W_A	-7.13257	4.49E-11	3640964
MRP17	-7.13535	1.3E-12	3641548
C2_10740C_A	-7.17998	2.02E-13	3641236
C7_01880C_A	-7.19299	0.040184	3637031
C3_05840W_A	-7.19303	0.033543	3641757
C6_01020W_A	-7.19351	0.047124	3644050
MRF1	-7.19619	4.59E-16	3645321
SOF1	-7.21822	5.74E-11	3640585

PES1	-7.25645	3.57E-13	3644105
PZF1	-7.28658	7.52E-13	3648113
UTP8	-7.32993	8.57E-18	3640547
C1_04720W_A	-7.33244	1.6E-08	30514988
GAC1	-7.35582	1.66E-10	3638025
C1_09790C_A	-7.36718	6.4E-16	3634843
C4_05230C_A	-7.38951	4.18E-12	3646156
JIP5	-7.39298	3.08E-13	3634922
C1_01750W_A	-7.40425	0.000598	3645413
C2_05640W_A	-7.40724	1.09E-16	3645566
C1_05930C_A	-7.41092	5.85E-14	3639862
YTM1	-7.41385	1.53E-10	3634765
AOX2	-7.43158	1.68E-06	3634945
FCR1	-7.44273	3.63E-15	3638542
RPA135	-7.45699	2.37E-15	3637996
C3_05900W_A	-7.46144	5.64E-14	3641739
C1_04510W_A	-7.46324	3.45E-07	3646147
C2_06850W_A	-7.46348	0.002951	30515142
C5_02280C_A	-7.47857	5.77E-12	3637712
C2_02540W_A	-7.48642	7.05E-18	3635820
OYE23	-7.50006	7.38E-05	3641618
MRT4	-7.51679	1.55E-08	3639219
CUP9	-7.52577	3.81E-07	3636950
KCS1	-7.5327	1.99E-10	3648120
C4_07040W_A	-7.53422	0.00012	3642963
TYE7	-7.53787	3.91E-12	3636203
C4_06210C_A	-7.56213	1.17E-12	3642815
C5_04850W_A	-7.56736	5.28E-06	3636436
C6_01780C_A	-7.57863	4.22E-11	3641636
CEK2	-7.60632	1.85E-05	3642459
NEP1	-7.67339	2.59E-09	3644545
ZCF28	-7.68476	2.56E-08	3634776
RPO41	-7.69348	3.67E-14	3639378
RCL1	-7.70752	3.02E-11	3645199
TRY6	-7.71299	4.91E-05	3638499
CR_03940W_A	-7.73899	2.58E-09	3648378
QDR1	-7.78526	2.65E-07	3640941
ZPR1	-7.80714	2.56E-16	3640177
SUT1	-7.81001	2.14E-17	3637706
C2_07020C_A	-7.81393	3.54E-07	3643497
RRP6	-7.81872	7.6E-13	3647535
MTLA2	-7.82772	0.01338	3643766
C1_10440W_A	-7.88437	1.61E-12	3647180
C1_02850W_A	-7.88979	3.74E-17	3636862
SUV3	-7.92113	3.33E-09	3645451
ATS1	-8.01264	3.37E-10	3642079
CR_08940W_A	-8.01441	1.01E-10	3641914

MIS12	-8.04277	6.08E-19	3639669
ALS5	-8.07235	7.51E-14	3640277
CDA2	-8.07785	4.93E-07	3639716
RRN11	-8.10955	1.31E-11	3645276
MIH1	-8.12684	9.8E-17	3648188
C3_00150W_A	-8.12931	0.000157	3640595
TAZ1	-8.133	6.26E-09	3644108
C6_00520W_A	-8.1552	0.002933	3647147
C1_09210C_A	-8.16473	9.18E-24	3634897
CR_04750W_A	-8.17874	0.004504	30515399
CR_09740W_A	-8.18747	2.3E-11	3639008
C5_03630C_A	-8.21667	1.13E-05	3646983
C7_01690W_A	-8.22056	8.27E-11	3636980
RLP24	-8.22673	5.57E-11	3645748
WSC1	-8.23839	5.4E-11	3635138
ARF1	-8.24084	5.16E-16	3642130
C1_11020W_A	-8.24591	1.94E-09	3644420
HAS1	-8.34127	1.64E-14	3636443
CR_07750C_A	-8.358	9.94E-13	3647350
C6_03800C_A	-8.3858	1.39E-19	3640310
CR_10410C_A	-8.40114	1.81E-14	3638957
CR_00810W_A	-8.41612	5.27E-06	3640415
UTP13	-8.50437	1.5E-17	3637718
C2_00760C_A	-8.50923	1.56E-17	3638910
RPA34	-8.52131	7.03E-13	3634883
CR_08470W_A	-8.55183	3.16E-16	3642134
UTP15	-8.55705	1.54E-12	3643906
C3_07650C_A	-8.56075	1.75E-07	3638488
C1_13250W_A	-8.56189	8.58E-24	3636143
CR_08720W_A	-8.57626	5.84E-09	3642122
PRP5	-8.63387	3.56E-19	3638447
C5_02070C_A	-8.63723	2.76E-15	3643746
MTM1	-8.65853	2.89E-13	3642487
LIP1	-8.66242	3.61E-05	3634888
FET31	-8.73541	1.73E-07	3647151
C3_02840W_A	-8.82752	8.11E-06	3638331
C4_03190W_A	-8.83178	9.79E-12	3637838
C7_02610C_A	-8.88027	1.02E-12	3648285
C3_02180C_A	-8.89001	7.54E-12	3636643
CR_06330C_A	-8.93946	2.06E-09	3644846
ALS9	-8.97223	1.53E-16	3640281
CR_04170W_A	-8.98282	1.24E-19	3640977
PNG2	-9.05476	3.51E-22	3645568
C5_03530C_A	-9.06464	0.030282	3646971
C7_01940C_A	-9.06917	5.15E-26	3636971
C4_03830W_A	-9.07357	1.67E-10	3647851
CR_03710C_A	-9.1044	5.46E-16	3643224

SFP1	-9.10888	4.97E-15	3635225
ARX1	-9.12362	1.87E-13	3636811
C3_03250W_A	-9.15772	1.65E-22	3638318
C4_04300C_A	-9.18869	1.66E-09	3646843
RPF2	-9.19349	2.21E-11	3643467
C6_01890C_A	-9.30379	3.58E-18	3641598
AQY1	-9.31789	7.21E-12	3642587
C1_06620C_A	-9.33568	0.012105	3639545
DHH1	-9.34223	1.13E-12	3639490
C1_07650W_A	-9.36382	2.44E-11	3646657
C2_00650W_A	-9.38792	7.76E-13	3638889
ARR3	-9.42735	4.78E-10	3642910
C6_04050W_A	-9.44432	2.79E-10	3647830
C4_06790W_A	-9.46447	1.01E-13	3642918
FUS1	-9.46651	0.048855	3645342
C4_02190C_A	-9.46651	0.048855	3635697
C4_02230C_A	-9.46656	0.048855	3635693
C1_12630C_A	-9.46987	8.91E-10	3646838
C5_02440C_A	-9.47347	7.23E-10	3637728
C1_14330W_A	-9.52201	2.91E-12	3643278
C5_00310C_A	-9.5222	8.65E-12	3641576
FLU1	-9.56398	2.18E-12	3637048
C4_02850W_A	-9.57924	1.08E-19	3637910
PTC8	-9.6115	1.5E-17	3635674
C4_06620C_A	-9.67103	2.09E-07	3642813
CR_02550C_A	-9.73636	3.75E-12	3647689
C6_02350C_A	-9.78863	4.48E-09	3645881
UTP21	-9.79052	7.79E-13	3635757
C1_05440C_A	-9.7929	2.82E-15	3642489
HPT1	-9.79701	1.36E-15	3643034
C6_00290W_A	-9.79724	1.34E-12	3646069
C1_03830C_A	-9.82128	9.15E-11	3644678
C2_06660W_A	-9.8213	3.95E-07	3648418
CR_04240C_A	-9.86831	1.08E-19	3640989
C1_08630W_A	-9.9404	1.75E-15	3634928
C5_01610W_A	-9.96607	1.02E-07	3647251
C1_10880W_A	-9.97277	4.36E-19	3644449
C4_07150W_A	-10.0011	0.004305	3642935
C6_02950C_A	-10.0096	5.6E-11	3639188
DIM1	-10.0389	5.73E-09	3636135
C4_00490W_A	-10.0787	4.85E-09	3645013
NRG1	-10.0792	4.39E-21	3643166
C7_00160C_A	-10.0803	1.22E-12	3637972
C1_01160C_A	-10.0845	5.38E-14	3640204
C1_12680W_A	-10.104	2.56E-17	3646833
RRP8	-10.1095	3.91E-13	3643900
ECM22	-10.1157	2.9E-19	3648000

C5_03510C_A	-10.1382	1.55E-10	3646969
NBP35	-10.1633	4.29E-18	3647412
C3_07670W_A	-10.1647	1.14E-16	3638460
MFG1	-10.1776	1.05E-15	3643884
IFA14	-10.1817	1.16E-08	3639012
POP4	-10.1838	3.43E-09	3639241
C1_07960W_A	-10.2234	9.76E-07	3640725
C4_05350W_A	-10.2255	1.95E-18	3646948
ELF1	-10.2424	3.13E-24	3641903
C2_06480W_A	-10.2512	3.84E-12	3646568
HSP78	-10.3007	2.65E-19	3637414
C3_05460W_A	-10.3441	5.63E-11	3643670
C3_01110C_A	-10.3469	0.009364	3639835
C2_04700C_A	-10.3552	4.7E-06	3647794
NOC2	-10.52	1.14E-20	3647596
LAS1	-10.5248	3.75E-16	3644073
CR_00310C_A	-10.563	8.25E-23	3639715
SEN15	-10.582	5.86E-06	3646411
RTA3	-10.6295	1.11E-10	3646566
BMS1	-10.7336	5.48E-20	3639857
C3_02350W_A	-10.7391	1.62E-16	3636618
C2_09880C_A	-10.744	1.26E-18	3643779
C3_04730C_A	-10.7483	0.002572	3635250
C6_01400W_A	-10.7504	0.002521	3641604
C4_05330C_A	-10.7578	2.66E-09	3646959
CNS1	-10.7612	5.66E-15	3639365
C7_01030C_A	-10.8182	2.64E-21	3638019
C4_02260C_A	-10.8849	8.8E-11	3635690
DRS1	-10.8993	9.22E-18	3639045
CR_00460C_A	-10.911	9.37E-11	3639726
HNT1	-10.9424	7.99E-20	3644441
C1_08610C_A	-10.9478	4.61E-13	3646512
C6_01040C_A	-10.9953	3.2E-14	3644014
C2_03690C_A	-11.0203	1.54E-09	3637376
QDR3	-11.0694	7.14E-12	3644023
GRF10	-11.1697	5.49E-23	3636492
CR_06230W_A	-11.2751	4.96E-09	3644867
PET127	-11.2782	9.14E-13	3644417
RGS2	-11.3139	7.2E-17	3646629
C6_04200C_A	-11.3921	2.96E-08	30515344
C3_05790C_A	-11.4826	2.53E-07	3641753
ZCF2	-11.489	5.9E-30	3642484
UGA3	-11.5291	9.72E-19	3638926
HEM3	-11.5425	6.11E-15	3643940
C3_07570C_A	-11.5707	5.46E-14	3638522
C3_06370C_A	-11.6498	1.37E-18	3642258
SPB4	-11.6848	6.55E-13	3647252

C1_10360C_A	-11.698	2.72E-26	3634836
C3_06940W_A	-11.7219	3.3E-28	3638509
C4_05010W_A	-11.8074	7.84E-11	3641148
SNQ2	-11.8449	7.32E-23	3640290
LTV1	-11.8516	4.56E-16	3639016
SPB1	-11.9103	1.11E-19	3648179
MAK21	-11.9223	1.88E-25	3635147
CSI2	-11.9359	2.2E-09	3642016
CR_01410C_A	-12.0535	2.19E-11	3640136
C7_04090C_A	-12.1213	4.28E-18	3643135
C1_11510C_A	-12.1283	4.8E-12	3645336
MPP10	-12.1635	1.48E-25	3647912
C4_00690C_A	-12.2191	4.53E-06	3645752
C1_07660W_A	-12.2367	2.83E-09	3646656
C5_05340W_A	-12.2583	6.32E-17	3636400
SSU81	-12.3022	3.82E-16	3634944
C1_01620C_A	-12.4339	4.57E-17	3640193
NOG1	-12.4592	9.45E-24	3641736
C5_03080C_A	-12.4742	6.29E-25	3637701
C2_06520C_A	-12.5145	5.3E-09	3646571
C2_05810W_A	-12.5208	9.87E-17	3647481
CR_04710W_A	-12.5878	0.000118	3648141
C2_08180C_A	-12.6129	8.31E-16	3642317
C7_01910C_A	-12.6273	3.78E-20	3637029
UTP4	-12.6487	4.2E-19	3636657
C4_02770C_A	-12.7034	2.68E-11	3637903
RRN3	-12.7307	1.08E-18	3647925
CTA8	-12.7415	3.43E-18	3634947
C3_05160C_A	-12.7562	6.87E-25	3635229
DBP8	-12.7896	3.31E-13	3646692
C2_06530W_A	-12.8026	3.22E-13	3646572
C2_10090C_A	-12.9111	4.75E-13	3643939
NOP8	-12.9488	4.96E-17	3646290
DED1	-13.0438	7.34E-27	3641752
C5_01430C_A	-13.0531	6.09E-11	3647056
MTG2	-13.1058	8.82E-13	3646693
C1_06440C_A	-13.1815	6.73E-11	3639552
NOP15	-13.1886	4.46E-13	3637966
SDA1	-13.1985	3.42E-20	3646688
CHR1	-13.2366	3.98E-26	3647601
C5_04380C_A	-13.2395	8.42E-10	3636485
TPS3	-13.274	2.2E-14	3641205
C7_02340C_A	-13.2914	1.1E-09	3636933
FGR47	-13.3632	3.29E-27	3641651
C5_01990W_A	-13.3912	0.001308	3643774
CR_04250W_A	-13.4831	0.021365	3640990
C4_07000W_A	-13.4832	0.02124	3642967

CR_01250C_A	-13.484	0.02163	3640403
CR_06870C_A	-13.484	0.022475	3647318
UTP9	-13.5044	6.13E-19	3638451
KRR1	-13.5425	4.11E-14	3644542
C2_02190C_A	-13.5904	1.01E-17	3635753
CR_09930W_A	-13.6449	7.18E-27	3639029
RNR3	-13.7154	5.12E-15	3647591
POP3	-13.7483	5.65E-10	3638993
HSP31	-13.7534	1.57E-12	3644589
MSS116	-13.7667	1.01E-26	3634792
C2_05160C_A	-13.7735	6.66E-29	3643461
C1_09710C_A	-13.8655	4.78E-23	3634818
SCH9	-13.9084	2.92E-28	3637421
COX11	-13.9573	1.73E-19	3646842
C6_04530C_A	-13.9645	2.26E-26	3645970
RPC10	-14.0736	3.28E-15	3643292
CR_04920W_A	-14.1145	3.68E-20	3648207
C3_02020W_A	-14.1834	3.54E-22	3636592
CR_07030C_A	-14.1989	6.57E-10	3645730
GPD1	-14.226	9.76E-19	3643924
C3_07400W_A	-14.2405	5.82E-15	3638471
SLY41	-14.3208	1.13E-32	3647139
ALD6	-14.3249	1.22E-17	3647407
C2_04080W_A	-14.5136	1.51E-19	3637381
C2_09160W_A	-14.5728	1.53E-10	3644090
NOG2	-14.6034	2.65E-19	3640299
C5_01710C_A	-14.6149	1.18E-11	3643732
NAN1	-14.7517	2.61E-18	3637932
SGD1	-14.7596	2.32E-23	3643191
NOP14	-14.779	1.93E-21	3635266
C2_10810W_A	-14.787	6.83E-17	3641243
BUD22	-14.9158	2.22E-21	3640391
C2_04670W_A	-14.9558	2.15E-14	3647789
NMD3	-15.0082	1.37E-20	3646614
UTP5	-15.0602	1.54E-21	3638922
CR_08080W_A	-15.1329	0.000139	3642094
SPO11	-15.1329	0.000141	3643873
C3_03460C_A	-15.1529	2.59E-13	3638359
C3_01560W_A	-15.3256	1.02E-20	3636669
RRP15	-15.3413	1.49E-16	3648397
CIC1	-15.533	1.44E-15	3644059
NOC4	-15.5621	5.56E-18	3645224
C5_03920C_A	-15.5857	5.37E-17	3647448
YVH1	-15.6581	1.99E-08	3643218
C3_07070C_A	-15.702	1.99E-13	3638446
TSR1	-15.7903	2.38E-28	3642135
NIP7	-15.8459	1.15E-12	3645880

RRP9	-15.9323	5.15E-16	3642576
C1_07950C_A	-15.9913	2.54E-10	3640724
CR_02420W_A	-16.0051	2.1E-17	3644926
C3_06950W_A	-16.1964	3.01E-17	3638507
C2_09500W_A	-16.2572	1.17E-19	3643817
C7_00330C_A	-16.4535	1.74E-15	3638042
RSM22	-16.5035	6.05E-18	3642506
C6_03210C_A	-16.6964	1.18E-08	3639178
C7_02600C_A	-16.7432	2.65E-21	3648286
PGA37	-16.7554	1.42E-10	3636484
C2_05330C_A	-16.8714	6.92E-21	3643426
C4_04810C_A	-16.8881	5.88E-16	3641112
C3_05800W_A	-16.9435	2.8E-19	3641754
C2_02580W_A	-17.0555	7.91E-17	3635860
NCS2	-17.3483	3.19E-27	3643216
C1_01610C_A	-17.3672	1.05E-11	3640225
ALS7	-17.4266	6.98E-29	3642234
C6_03200W_A	-17.4288	7.44E-29	3639177
SMM1	-17.4996	1.68E-18	3643778
C5_00810C_A	-17.5007	0.008647	3644127
C3_04870W_A	-17.5007	0.008679	3635226
C1_03840W_A	-17.5007	0.008709	30514984
C7_03550C_A	-17.5018	0.010469	3646235
NSA2	-17.5178	9.32E-22	3642259
CR_08500W_A	-17.7038	9.37E-20	3642136
HWP2	-17.7558	0.000163	3646803
GCS1	-17.8495	9.78E-28	3640742
CR_09800C_A	-17.9333	3.15E-35	3638984
C3_06760W_A	-17.9505	9.57E-14	3638502
CR_07500W_A	-18.143	0.015964	3647997
C6_00530C_A	-18.1777	1.99E-07	30515326
CR_10470C_A	-18.2188	1.34E-26	3638979
RPA12	-18.2297	2.17E-09	3643477
CR_08580C_A	-18.2655	5.79E-11	3642105
CR_01710W_A	-18.2656	7.91E-11	3640130
RFX1	-18.2666	1.94E-13	3644844
RMS1	-18.3131	7.84E-17	3646361
C1_10970W_A	-18.3395	2.71E-26	3644427
CLG1	-18.4199	1.46E-40	3646030
C1_01510W_A	-18.5779	1.97E-26	3640229
C4_03350C_A	-18.6122	1.1E-15	3646794
C1_04710C_A	-18.6709	5.33E-14	3644764
C1_05330C_A	-18.7429	4.16E-20	3642485
ROA1	-18.8426	1.96E-39	3645424
C1_09330W_A	-18.959	3.28E-09	3634802
C1_01150C_A	-19.0501	3.77E-29	3640203
C1_13130C_A	-19.1446	8.94E-21	3636202

C6_02230W_A	-19.169	4.3E-28	3645866
C2_00410C_A	-19.3411	1.3E-31	3638820
HCA4	-19.4615	7.82E-29	3637911
C1_06350W_A	-19.5393	5.75E-29	3639562
C1_04040C_A	-19.7298	1.38E-25	3646429
C3_06160C_A	-19.7801	1.62E-20	3641766
C4_02740W_A	-19.822	1.14E-32	3637855
NSA1	-19.8646	2.08E-16	3642313
DBP7	-19.8757	4.24E-30	3645903
C2_01060C_A	-20.1568	2.68E-10	3638835
FAR1	-20.1804	5.17E-13	3637970
RCH1	-20.3613	1.05E-18	3645002
CR_03360W_A	-20.3742	2.26E-20	3645141
HSP70	-20.4654	1.11E-30	3636229
C1_11000C_A	-20.7538	2.8E-15	3644422
GST2	-20.9614	3.7E-21	3637937
RIM8	-21.1576	4.77E-33	3639342
UTP18	-21.4788	1.33E-26	3643123
PRM9	-21.5198	0.006705	30515173
DIP2	-22.1038	1.1E-24	3640762
C2_03110W_A	-22.1946	0.000203	3642995
SSF1	-22.2345	6.48E-19	3647085
C6_00810C_A	-22.2948	9.82E-19	3647068
C2_04570W_A	-22.301	6.51E-24	3645441
PIR1	-22.4254	1.42E-43	3645801
NOP13	-22.4908	2.07E-26	3638469
FTH1	-22.5468	1.35E-31	3634852
C3_01540W_A	-22.565	2.78E-07	3636567
NOP4	-22.7012	2.11E-36	3647991
BUD21	-22.8558	1.17E-18	3640541
TSR2	-22.9556	9.34E-17	3636845
SRP40	-23.1829	7.72E-29	3642605
C1_01130W_A	-23.5835	1.02E-45	3640178
DBP3	-23.7559	1.49E-24	3634868
ELA1	-24.1575	5.18E-15	3644680
SAS10	-25.351	6.94E-34	3637899
C6_03810W_A	-25.5906	1.92E-29	3640286
C2_09660W_A	-25.7975	5.27E-24	3643793
PRX1	-26.0549	4.94E-26	3641357
C2_04120C_A	-26.171	3.46E-21	3637377
MAK5	-26.1875	8.44E-27	3643454
ENP1	-26.1882	5.6E-20	3647861
ENP2	-26.2076	9.78E-24	3646234
C4_07140W_A	-26.4191	2.51E-40	3642936
CR_06550C_A	-26.4801	5.08E-21	3645260
CR_01600C_A	-26.5908	1.38E-12	3640080
PGA57	-26.8176	9.36E-07	3635608

RMP1	-26.9615	0.00014	3636215
CDR4	-26.9629	3.61E-26	3640711
CFL4	-27.1762	1.77E-11	3642639
KTI12	-27.3352	2.15E-15	3645140
CR_02030C_A	-27.3929	1.9E-19	3640152
C1_10950C_A	-27.6652	2.36E-29	3644428
UTP22	-27.6796	4.48E-28	3635760
FYV5	-27.9514	4.82E-10	3647057
CR_04110W_A	-28.4827	2E-21	3641026
HSP21	-28.6038	5.05E-27	3637364
BMT1	-30.1427	4.7E-43	3638535
CR_06140W_A	-30.2081	1.29E-21	3644862
C4_03340C_A	-31.5681	4.78E-11	3648090
C1_09770W_A	-31.5837	4.36E-24	3634825
FTR1	-32.455	2.55E-30	3643318
C2_08580W_A	-32.9046	1.04E-05	3643916
C2_07070W_A	-33.1047	1.62E-32	3643501
EBP1	-33.2159	2.09E-18	3644060
C1_06430C_A	-33.5646	0.001257	3639553
C1_12440W_A	-33.5714	0.001142	3642012
CR_01510C_A	-33.5714	0.001146	3640104
C7_00770W_A	-34.1583	1.35E-25	3637959
C5_02590C_A	-34.8284	4.56E-16	3637723
C6_03360C_A	-35.5903	5.17E-08	3639203
C1_07790C_A	-36.3459	6.24E-17	3640706
RRS1	-36.3897	3.39E-20	3639352
C1_13820C_A	-36.4363	1.85E-23	3636127
RPN4	-36.5326	2.73E-43	3644684
CTA4	-36.5808	4.76E-39	3641743
FMP45	-36.7802	3.39E-31	3637004
SEN2	-36.8319	2.86E-19	3637832
C1_02450C_A	-37.8171	1.08E-11	3636849
C2_05840W_A	-37.8457	6.07E-20	3647484
C6_01870C_A	-39.0874	2.54E-20	3641625
QDR2	-39.4927	2.96E-37	3643661
KSP1	-39.662	1.06E-40	3644467
C6_03180C_A	-40.0169	6.41E-61	3639261
WOR2	-40.0503	4.19E-14	3635230
C2_05750W_A	-40.4916	2.01E-26	3645577
CR_04940W_A	-41.4118	4.3E-11	3648209
C5_04910W_A	-41.4733	1.09E-06	3636417
C4_00810C_A	-41.4926	1.42E-10	3645742
C6_03440W_A	-42.2797	8.3E-21	3640274
GIT3	-43.0088	1.77E-35	3642634
C1_06760C_A	-45.5875	5.64E-26	3639505
HGT17	-45.613	0.000304	3635687
C3_02040C_A	-45.896	2.85E-24	3636579

KAR4	-47.2785	7.84E-10	3644937
C3_00230C_A	-47.9096	3.92E-07	3640557
ZCF1	-48.0981	2.61E-60	3638329
C2_08260W_A	-48.7315	5.17E-10	3642345
C4_04520W_A	-49.049	1.37E-32	3641110
C1_00190C_A	-49.1348	9.05E-30	3639337
C7_03850W_A	-52.2812	5.9E-20	3643145
KTI11	-53.6461	0.000134	30515125
C7_00880C_A	-54.2473	3.02E-46	3638052
FRE10	-55.4065	2.43E-23	3646841
ECM1	-55.8081	9.19E-13	3641224
HBR3	-57.8144	1.47E-33	3645652
REI1	-59.7964	1.63E-25	3647536
C7_01680C_A	-60.0243	3.31E-45	3636981
C3_01430W_A	-66.2648	4.94E-11	3636633
ARG11	-69.7215	2.26E-05	3635193
C2_02130C_A	-70.2381	1.83E-14	3635747
C2_08530C_A	-73.2969	1.13E-08	3643921
NOP6	-74.4329	1.1E-17	3639507
C5_03440W_A	-75.7525	3.46E-09	30515310
CR_01530C_A	-78.0126	7.5E-15	3640072
MAK16	-80.1246	1.51E-26	3646920
C3_04510W_A	-84.3182	2.05E-09	3635258
RME1	-99.7606	9.87E-51	3644453
C7_01430C_A	-100.19	2.98E-53	3636961
AAH1	-109.879	1.94E-06	3643529
SSU1	-120.189	1.02E-52	3641882
GPI1	-127.682	3.17E-20	3643680
YWP1	-156.447	1.57E-72	3643915
CR_08880C_A	-231.101	3.16E-19	3641878
CR_00840C_A	-235.678	0.042954	3640411
CR_03320C_A	-249.101	0.044671	3645096
C3_00120W_A	-267.706	0.037777	3640566
C1_02690C_A	-269.513	0.038482	3636823
C5_04030W_A	-324.073	6.12E-26	3647737
FRE9	-356.356	5.44E-16	3643452
C2_02640C_A	-459.923	0.021892	30515095
MNN1	-507.485	1.09E-10	3637784
C1_11410C_A	-523.961	0.018969	30515054
C4_06910W_A	-540.981	1.14E-10	3642933
C1_09820C_A	-556.015	0.017898	3634846
C3_06170C_A	-652.188	0.015093	3642272
HSP30	-892.042	7.19E-59	3645419
C7_03560W_A	-943.888	4.59E-53	3646236
CRZ2	-1002.48	1.51E-56	3645725
C4_07260W_A	-1581.44	0.00555	3642942
PHO87	-2061.12	0.004023	3639861

C1_14630C_A	-23084	0.000165	3643291
TAR1	-57898.3	9.7E-262	30515449

S120 vs Control

Name	Fold change	P-value	GeneID
ECE1	25907.67	1.01E-08	3646814
HWP1	7404.873	2.04E-23	3645372
C1_04180W_A	1816.104	2.43E-05	3644716
C1_13100W_A	1182.998	7.1E-05	30515073
SAP5	1155.91	7.76E-05	3639268
ECM331	1139.382	7.78E-05	3637731
HGT12	1123.753	8.72E-05	3638048
CRH11	921.141	6.3E-14	3637905
HHT21	649.5999	4.33E-13	3644707
CDC11	624.8933	0.000287	3647888
MAL31	314.4351	4.34E-10	3636420
HYR1	286.334	1.01E-09	3636226
C4_05390W_A	280.3652	0.001459	3646951
SOD5	269.1401	4.52E-56	3638886
SAP6	238.1401	1.84E-09	3639229
ALS3	215.2594	1.3E-130	3647965
MLC1	214.0555	0.002574	30515387
PGA6	208.5507	3.75E-09	3634774
CDC10	201.5065	3.13E-09	3640947
TUB2	194.6661	6.83E-19	3639329
VMA10	186.9686	0.003166	3645215
ALI1	172.8347	0.003684	3636635
C5_03000C_A	158.7864	0.0042	3637818
CR_06500C_A	153.8465	0.004467	3645274
DPM2	150.6277	0.004602	30515325
COG4	139.7331	0.005293	3645571
CR_06380C_A	139.0102	0.005344	3648139
C6_00400C_A	138.2382	0.005409	3646087
HHT2	136.9696	3.47E-20	3647326
C6_02140W_A	132.4267	0.005767	3645886
C3_02620C_A	132.1478	0.005784	3638326
HTA2	131.0766	2.85E-19	3644715
C3_07840C_A	130.268	0.006076	3647465
SHE3	128.3289	6.45E-08	3639277
FGR6-10	118.1774	0.007118	3641770
SAM51	117.9032	0.007174	3641582
C3_06670C_A	115.0742	0.007399	3642254
C1_03150C_A	114.9287	0.007679	30514976
CR_05310W_A	108.351	0.008305	3648125
HRT2	103.9582	2.18E-07	3635709
C6_02180W_A	103.3995	0.008951	3645861
ERG11	102.2612	4.15E-21	3641571

CCN1	101.4518	0.009144	3643735
C4_02460W_A	101.3564	0.009187	3637844
FGR41	100.4444	9.5E-07	3634839
DPM1	97.62307	3.42E-07	3640700
C6_00710W_A	96.36837	0.009961	3647073
TUB1	96.11569	1.23E-26	3641930
C3_06750W_A	95.17098	0.010596	30515219
ASM3	91.8097	1.48E-10	3639332
ERG28	90.26678	0.011351	3638833
EXG2	88.58708	8.96E-14	3636816
ERG6	87.3051	2.67E-16	3636655
UME6	86.95036	6.1E-07	3647522
MCD4	86.85311	0.012043	3642036
PHR1	86.45929	2.06E-62	3641108
PGA31	82.6682	0.012841	3641226
PGA45	81.41293	7.67E-07	3639875
PGA23	81.22544	0.014881	3644941
FRP2	79.83861	1.1E-15	3638008
GUP1	78.9468	0.01394	3636171
C5_02780W_A	74.29708	0.015215	3637803
RBT4	73.36206	4.79E-20	3639534
HHO1	72.85366	3.31E-13	30515358
TOS1	72.37178	3.06E-50	3636566
CR_04650W_A	72.20191	0.017014	30515396
LSC1	70.52083	1.17E-22	3640199
FAD1	67.96825	0.017774	3634930
TUB4	65.61016	0.018784	3641774
C7_01100C_A	63.88077	0.019632	3637948
C4_02600C_A	63.87914	0.019579	3637912
C1_11950W_A	63.67502	0.019673	3642045
YNK1	63.06106	8.6E-19	3637746
C1_10560C_A	60.08761	0.021394	3647196
C1_02100W_A	59.04022	0.02199	3644578
C2_09590C_A	58.50324	0.022795	3643800
PGA34	58.43625	0.022476	3642579
C2_10010C_A	57.24483	0.023033	3643962
CR_10130W_A	56.9033	0.023669	3638970
C1_06250W_A	56.80114	0.023309	3647525
C3_07130W_A	56.64197	0.024334	3638440
POL30	56.52391	6.2E-06	3635583
HHF1	56.35825	8.51E-25	3644705
OST1	56.1464	6.44E-06	3643657
C1_07490C_A	56.08034	0.023845	3646666
PGA38	55.34272	1.66E-09	3637846
TEM1	55.25356	0.02436	3636923
C2_05510C_A	55.24473	0.024088	3643420
C3_01020W_A	55.18658	8.19E-09	3639828

DDR48	55.07801	2.83E-25	3644061
CR_04490C_A	54.85898	0.024242	3641013
C1_03600W_A	54.46304	7.46E-06	3636880
C3_01100W_A	54.39344	0.024729	3639834
AGM1	53.0375	8.69E-06	3636138
FGR6-3	50.78609	0.027157	3647954
C2_02050C_A	50.38293	1.75E-08	3635851
MCD1	50.05062	0.027626	3639044
CDC28	48.78972	1.41E-05	3644838
C4_04380C_A	46.88121	1.62E-05	30515266
ERG26	46.51957	5.62E-11	3642803
C1_06700W_A	45.81408	1.88E-05	3639511
ERV46	45.61996	2.43E-08	3644129
PRI2	44.94446	0.032106	3642788
C2_09960W_A	44.43002	0.032904	3643821
CWH43	44.17237	1.63E-10	3640389
C2_05720C_A	44.16518	1.83E-10	3645574
SNZ1	44.09828	2.35E-10	3636803
BIO2	43.50852	2.63E-05	3640106
C2_02330W_A	42.04205	0.03553	3635811
RBT1	41.93871	7.87E-10	3645377
GIG1	41.61863	0.03588	3644712
C1_07920W_A	41.57404	0.035905	30515026
ERG10	41.48762	6.02E-30	3648275
RFA2	40.99916	3.42E-05	3643506
C1_06050C_A	40.92611	3.38E-05	30515003
KAR3	40.62909	0.037203	3644078
C5_03930C_A	40.35438	3.3E-10	3647447
CHT3	40.24288	2.63E-10	3638967
C3_02330C_A	40.23604	0.037948	3636550
C3_02530W_A	39.71833	0.038236	30515189
CWH8	39.18072	0.039976	3644598
CFL11	38.69454	6.88E-08	3646622
DAD3	38.16401	0.041657	3644864
ARF3	37.93858	4.19E-05	3636628
PMI1	37.87707	7.52E-08	3643795
DOG1	37.72422	4.31E-05	3641597
CR_01910C_A	37.41502	0.041733	3640095
CR_00280C_A	37.40695	0.041702	3639719
CHO1	37.19543	1.03E-11	3644557
C6_00120W_A	36.80473	0.042757	30515323
ARC18	36.73014	0.043062	3644056
C6_00660C_A	36.40552	0.043704	3647133
DPP3	36.19875	4.55E-12	3636942
CR_06170W_A	35.89784	5.47E-05	3644865
HTA1	35.74504	4.36E-20	3645658
C6_02210W_A	35.47551	0.044884	3645864

CBR1	35.30851	3.64E-13	3646961
ERG2	35.15236	7.4E-05	3639347
C6_02100W_A	35.14137	2.01E-15	3647554
ASF1	35.04437	0.045708	3647348
C1_11940C_A	35.00655	0.045754	3642046
APE3	34.81753	4.88E-09	3643875
C1_03940W_A	34.61904	1.6E-07	3646415
C7_02310C_A	34.56738	0.046743	3636936
ATO1	34.33794	9.59E-12	3647761
ACP12	34.07311	9.03E-05	3637361
IHD1	33.85754	8.97E-38	3640291
ECM33	33.55228	1.06E-46	30514977
PTP3	33.16464	1.2E-38	3638940
C3_07540C_A	33.12279	0.049445	3638526
PMM1	32.85381	9.18E-11	3636852
ATP18	32.54227	8.66E-05	30515084
SSR1	32.29718	2.67E-16	3638055
PLB5	31.14378	5.43E-17	3640750
MAL2	30.35605	6.37E-13	3638946
C5_02380W_A	30.35272	3.3E-46	3637794
CYB5	29.53665	9.24E-07	3637965
PDI1	29.43708	1.59E-20	3641367
ATP19	29.28343	0.00017	30515061
C3_07470W_A	29.14874	4.16E-08	3638479
HHF22	29.05323	1.45E-17	3647327
CHS1	28.9221	1.59E-12	3641364
HOM6	28.75685	0.000174	3636815
C3_02140C_A	28.74908	6.33E-09	3636656
ERG13	28.74396	2.36E-16	3641881
BGL2	28.56694	1.92E-31	3635691
HGT1	27.8786	2.74E-41	3645420
CR_00990W_A	27.50132	0.000198	3640425
FAV2	27.49754	0.000213	3647236
SGA1	27.35965	4E-10	3636652
C1_02730W_A	27.0026	0.00022	3636826
ATP1	26.88715	2.26E-44	3646134
RFX2	26.75059	2.81E-10	3635658
HGT3	26.74329	0.000227	3643185
CSA1	26.6084	8.16E-30	3638009
CR_05500C_A	26.27493	0.000245	3647598
EMP24	26.21191	1.58E-06	3647247
GFA1	25.70344	1.44E-36	3636557
ASK1	25.63726	0.000287	3635656
SEC21	25.54321	1.14E-15	3644786
C6_02190C_A	25.44161	2.24E-06	3645862
LSC2	24.68975	1.29E-28	3646618
CR_00910W_A	24.63013	3.19E-22	30515371

CYT1	24.62745	8.31E-17	3643441
PGA10	24.48807	3.66E-06	3645009
PMT2	24.39702	6.78E-18	3638538
C1_14060W_A	24.33172	3.06E-21	3643344
CR_09330C_A	24.28902	6.56E-17	3641899
SOL3	24.26504	2.84E-08	3646625
SOD1	24.21585	1.53E-10	30515248
RAD54	24.19249	0.000358	3636192
C4_00710W_A	24.04229	4.01E-06	3645750
SEC22	23.88488	0.0004	30515391
ERV25	23.88412	1.05E-09	3642243
C1_09060C_A	23.00268	0.000517	3634772
ARO9	22.92479	6.56E-11	3641773
C2_07650C_A	22.84799	5.39E-06	3642342
C5_05200C_A	22.78496	4.86E-11	3636467
COX5	22.62841	5.83E-16	3634768
THR1	22.47825	0.000574	3641572
CHT2	22.47542	1.48E-15	3636523
QCR2	22.44674	2.71E-17	3646352
CR_09560C_A	22.24308	0.000589	3647099
SEC23	22.19735	3.11E-17	3641818
PGA54	22.03231	5.58E-17	3637929
MSB1	21.70079	1.16E-10	3648218
CDC12	21.60917	3.66E-17	3636809
OSH3	21.58983	6.34E-13	3645368
C5_02920W_A	21.56556	0.000647	3637751
ERV29	21.53349	1.24E-10	3635672
SSA2	21.30595	1.76E-14	3644711
C5_01230C_A	21.2183	1.34E-11	3642618
C1_02760W_A	21.21827	2.36E-09	3636870
C6_04100W_A	21.19501	1.6E-13	3647836
C5_05020C_A	21.05027	2.25E-09	3636486
C1_07260C_A	20.82136	0.000788	3644456
SMC1	20.77328	8.12E-06	3643221
TAL1	20.74321	8.48E-30	3643225
C2_09050C_A	20.44842	2.59E-09	3645791
ERG24	20.3643	4.16E-10	3647716
KTR4	20.30932	0.001177	3646425
ERP5	20.29853	2.77E-09	30515046
SUN41	20.1149	1.93E-36	3647067
C2_07010W_A	20.1088	7.38E-12	3643535
CSR1	19.84429	2.61E-07	3641162
C2_09100C_A	19.71139	3.78E-10	3644113
C1_04430C_A	19.67792	0.000902	30514986
C1_12650C_A	19.65176	0.000934	3646836
ASH2	19.48964	0.000919	3636445
LSP1	19.31179	3.67E-33	3648025

C2_07540W_A	19.24195	0.000943	3645222
GIN4	18.95061	7.94E-10	3644543
SWI6	18.91173	0.00103	3634932
C2_07140W_A	18.71057	4.49E-11	3643508
TSA1	18.68621	6.67E-24	3642269
POL1	18.54254	2.89E-09	3635144
MSH6	18.35239	2.95E-09	3636161
PHO15	18.24272	1.47E-09	3644459
C5_03120W_A	18.21076	0.001255	3637705
C1_01210W_A	18.10377	1.83E-08	3640209
CR_01490C_A	18.07979	6.03E-07	30515376
DOA1	18.05185	2.3E-13	3634871
CDC21	17.98319	0.001293	3643463
GND1	17.83615	2E-27	3636131
HGT2	17.81097	2.44E-27	3644579
LAP41	17.60739	1.77E-12	3636653
C3_02820C_A	17.55181	9.24E-13	3638419
MPS1	17.53413	0.001536	3641888
HOF1	17.4892	1.71E-06	3645014
RFC5	17.37944	0.001533	3638890
C3_06400C_A	17.3375	2.86E-05	3642260
C5_01070C_A	17.30014	5.18E-12	3642627
SCW11	17.25478	5E-13	3636521
DAP1	17.24126	1.34E-06	3641021
CR_09520C_A	17.21157	3.02E-05	3647103
HSL1	17.11037	4.98E-12	3637810
NHP6A	17.10157	1.67E-14	30515244
MCM2	17.07316	0.00165	3643197
CR_03520C_A	17.05097	9.41E-11	3643203
ACT1	17.04361	4.37E-36	3636195
CMP1	16.95596	1.29E-09	3639328
GPX2	16.88944	4.01E-23	3644009
RGD1	16.88671	1.31E-06	3639868
SAR1	16.80886	2.37E-11	3645865
CR_05130C_A	16.76696	0.001902	3646401
VMA2	16.76168	8.14E-18	3646710
TSA1B	16.64203	1.87E-21	30515214
CR_08450C_A	16.61399	3.12E-09	3642133
EAF6	16.58533	0.002062	3646494
ACS2	16.52656	1.5E-29	3644710
C4_05250W_A	16.48865	2.96E-06	3646158
CR_05800C_A	16.47041	0.001945	3646706
QCR8	16.46343	2.21E-10	30515112
ATP3	16.41498	1.14E-16	3640387
RNR1	16.33511	1.33E-16	3642989
C4_00390W_A	16.30467	5.13E-05	3645016
ERG9	16.30331	2.89E-15	3643913

NUO1	16.28398	2.83E-10	3647100
C1_06920C_A	16.24189	0.002128	3639525
SEP7	16.18678	7.27E-08	3644596
C1_02500W_A	16.172	0.002288	3636795
LAT1	16.16685	1.51E-21	3636986
CR_04770C_A	16.0222	0.002241	3647302
C6_02880W_A	15.98532	0.002273	3639238
C4_07080C_A	15.97987	5.46E-05	3642905
C2_00590W_A	15.94515	5.42E-05	3638851
DPM3	15.90962	6.03E-05	30515245
C2_10200W_A	15.88837	0.002278	3643928
HMG1	15.87954	7.76E-13	3644687
C2_05290C_A	15.87477	3.65E-06	3643472
SEF2	15.79999	6.9E-05	3647052
CR_08610W_A	15.76689	1.93E-09	3642109
C7_00230W_A	15.75493	9.13E-08	3637982
CLB4	15.73573	7.41E-05	3643102
C2_03020C_A	15.71582	3.77E-16	3643019
SEC13	15.71438	1.28E-11	3638425
CR_03330W_A	15.6741	0.002425	3645095
RET2	15.63177	6.85E-11	3637714
C1_04200C_A	15.56799	2.25E-06	3644718
C1_01080W_A	15.55701	0.002491	3640173
SAC1	15.54995	1.14E-07	3634864
HAT1	15.54863	5.7E-05	3644770
C1_03240W_A	15.35684	2.34E-06	3636895
FAS2	15.31214	4.22E-36	3635222
ZWF1	15.25998	1.48E-16	3634913
PLC2	15.23847	2.19E-06	3642985
CR_03270W_A	15.07747	2.25E-06	3645101
TVP18	15.02328	2.83E-06	3634844
CR_10530W_A	14.96465	0.002774	3639042
HTA3	14.91615	6.08E-07	3638322
MSC7	14.88001	8.65E-14	3645214
SRB1	14.8783	1.55E-23	3647454
PMT4	14.73193	3.05E-13	3644096
C2_06610C_A	14.605	0.00349	30515140
C7_00670C_A	14.59621	1.47E-08	3638024
PGA63	14.5946	4.82E-16	3639524
CR_05360C_A	14.5677	0.003282	3648033
C5_04950C_A	14.55577	1.89E-08	3636422
ACB1	14.52	1.09E-08	30515350
PDX1	14.47718	3.16E-16	3636128
C1_01190C_A	14.4517	0.000128	3640207
C1_09980C_A	14.44185	0.000101	3634863
C3_05990C_A	14.43267	2.49E-07	3641732
C7_01710W_A	14.34976	6.49E-11	3637012

NGT1	14.28793	0.0034	3640573
VCX1	14.02691	2.27E-10	3646508
C6_03130W_A	14.01954	6.33E-06	30515341
SEC12	14.01209	4.54E-11	3641639
CBP1	13.99711	1.44E-15	3641920
C4_04980W_A	13.99494	5.03E-06	3641167
C1_00700W_A	13.98533	0.003938	3639330
SPE3	13.97826	4.11E-10	3643528
ERG5	13.95357	9.53E-22	3641400
C4_02930W_A	13.9269	0.000123	3637876
C5_02220C_A	13.92059	0.003948	3637760
HET1	13.87745	1.07E-12	3647897
ATP2	13.82176	1.76E-33	3645039
CUP5	13.75874	1.73E-13	3635174
C2_06770W_A	13.6893	0.003869	3643515
PDB1	13.59833	1.11E-21	3641186
CR_09070C_A	13.57104	4.68E-13	3641926
MIS11	13.5305	1.06E-15	3645732
CR_09230C_A	13.52248	6.99E-06	3641918
SUC1	13.41094	1.51E-11	3641916
LAG1	13.31954	7.52E-08	3640383
LYS1	13.30931	2.71E-10	6335429
C2_03970W_A	13.26784	0.004315	3637419
CDC19	13.21427	3.33E-32	3643438
C1_10310W_A	13.15095	0.004357	3634906
CR_03470W_A	13.14537	0.004273	3643198
CR_03000C_A	13.14537	0.004306	3642597
KIP1	13.10285	0.000199	3645261
SOU1	13.02748	0.000217	3642799
C3_01070C_A	13.01474	0.004432	3639832
C6_03320W_A	13.00113	1.04E-05	3639198
COF1	12.99901	2.58E-12	30515293
CDC5	12.98358	1.56E-11	3639281
C1_03170C_A	12.91725	0.004574	3636805
C5_00080C_A	12.8058	1.21E-10	3647886
OPI3	12.79772	0.00467	3642277
C6_02200C_A	12.78229	0.00471	3645863
LYS9	12.71841	6.87E-08	3642279
C4_04800W_A	12.59042	3.67E-15	3641113
C5_04410C_A	12.53255	1.08E-05	3636410
SFH5	12.48035	6.17E-10	3634884
C4_06140C_A	12.45019	0.004981	3642821
CR_09570W_A	12.41977	1.2E-05	3647098
C1_01290C_A	12.30547	0.00034	3640184
SSH1	12.29968	1.98E-08	3642504
GVP36	12.29794	7.59E-08	3641772
RKI1	12.2262	0.000366	3636574

PIL1	12.22007	7.32E-28	3644769
PDR17	12.21893	0.000367	3643007
C6_02330W_A	12.19171	3.4E-29	3645877
CR_04860C_A	12.10616	0.005429	3647294
MCM6	12.10593	1.94E-05	3640142
IQG1	12.03896	1.13E-14	3637052
HXK2	12.0263	5.93E-34	3641015
C5_02210W_A	12.02373	1.8E-07	3637759
C5_01440C_A	12.01277	1.7E-06	3647043
C4_04860W_A	11.98991	1.42E-06	3641161
IPP1	11.9393	4.03E-20	3643874
C4_02420C_A	11.89128	0.006572	3637849
PST1	11.86926	1.65E-15	3643520
RPS17B	11.85149	2.48E-12	30515044
C1_07590C_A	11.81814	1.62E-06	3646673
C4_04600C_A	11.75994	0.000429	3641156
BTA1	11.75316	0.001649	3645347
CHS2	11.73448	1.68E-14	3641893
MNT1	11.71919	3.37E-20	3636585
RPL24A	11.68435	8.46E-13	3641174
C1_09020W_A	11.68125	2.68E-07	3634917
RFA1	11.58452	1.15E-10	3638823
C1_04780C_A	11.57231	1.53E-06	3644774
C3_07230W_A	11.57136	8.66E-11	3638531
CYP1	11.54777	6.32E-15	3636978
C1_14320C_A	11.52729	0.006399	3643316
MYO2	11.52467	1.25E-12	3636140
C2_02410W_A	11.43123	1.53E-09	3635755
ABG1	11.42344	5.64E-08	3647715
CR_08480C_A	11.38185	4.96E-07	30515442
MYO1	11.38105	1.06E-10	3647248
VMA5	11.32406	7.05E-10	3642316
GDE1	11.29875	9.15E-13	3636505
SER33	11.28739	3.15E-16	3642030
ARP3	11.21969	7.62E-12	3643479
ILV5	11.19789	4.9E-13	3644012
ARC35	11.15945	5.23E-08	3639823
QCR7	11.13618	8.34E-10	3639233
ERG251	11.05691	1.89E-24	3635716
C4_00020W_A	11.04896	3.21E-11	3648053
NDE1	11.02745	4.78E-27	3638354
CWH41	10.98264	4.41E-07	3646533
FTR2	10.97902	3.43E-10	3643303
C1_03720C_A	10.96743	2.87E-10	3648216
SPC3	10.95808	3.56E-06	3636121
ERG4	10.94191	4.02E-15	3640511
C1_11960C_A	10.93384	0.00809	30515060

SMC2	10.91691	4.06E-06	3643918
C2_09040W_A	10.88432	7.1E-07	3645792
TRX1	10.85121	1.29E-11	3638950
MDH1-1	10.84761	1.32E-15	3635636
C7_04000W_A	10.84761	6.71E-07	3643114
RHO1	10.84749	9.43E-16	3642564
CLC1	10.82782	1.31E-10	3635662
SEC20	10.80002	0.00057	3639206
MRP8	10.79127	1.33E-06	3641178
C5_01590W_A	10.76968	5.02E-06	3647041
C2_07550W_A	10.727	9.09E-10	3645221
APL4	10.70318	9.96E-10	3642565
EXO1	10.68534	4.78E-05	3641583
CR_10440W_A	10.59183	0.00899	3638976
GLC3	10.58265	7.13E-15	3639200
VMA11	10.5496	5.53E-06	3637054
YVC1	10.54932	8.19E-15	3642303
MNN12	10.5447	6.06E-15	3634904
C3_04420W_A	10.54163	3.4E-15	3635180
ADH2	10.465	5.56E-20	3640751
C6_03950C_A	10.44435	5.35E-06	3640268
IDH1	10.44366	9.08E-19	3634893
C2_07720C_A	10.38529	2.57E-14	3642304
CR_09590W_A	10.36537	2.35E-08	3647095
RAS1	10.35081	1.35E-20	3643927
C1_05180C_A	10.30981	0.010304	30514990
TRS20	10.28241	0.000787	3640214
C6_00770C_A	10.25555	0.00983	3647079
MEU1	10.25331	9.67E-08	3645635
C1_01800W_A	10.21706	0.00084	3645407
DPB4	10.18944	8.44E-05	3638818
ALD5	10.16838	2.25E-25	3643026
AXL2	10.15946	2.06E-11	3641184
C7_01500W_A	10.15372	7.95E-05	3637050
TPM2	10.14063	1.1E-18	30515441
CDC46	10.08845	9.16E-05	3646912
CR_07220C_A	10.06463	5.22E-10	3646040
C4_04970C_A	10.03118	8.07E-05	30515274
CR_04760C_A	10.02604	0.000929	3647303
C4_01510W_A	10.01549	1.61E-06	3635616
C1_06270W_A	10.01397	1.39E-06	3647523
MET6	10.01045	4.98E-20	3640084
DUT1	10.00712	8.47E-05	3640188
CDC3	9.987704	4.26E-16	3644719
PDA1	9.982906	6.29E-25	3642902
OPT6	9.97926	1.35E-09	3635603
C6_03090W_A	9.946713	0.010899	3639275

CEK1	9.941207	8.86E-05	3642789
C6_01460C_A	9.941129	2.05E-06	3641623
KGD2	9.919755	6.17E-15	3646036
RHO2	9.902147	7.68E-06	30515143
CDC42	9.879379	4.88E-08	3646488
C6_03960W_A	9.859484	1.62E-13	3640269
C6_02480W_A	9.792949	8.87E-05	3639170
DUN1	9.790309	9.24E-05	3636494
CR_07120C_A	9.777447	0.000136	3648225
CR_05760C_A	9.705363	0.001063	3646695
C3_05880C_A	9.70211	3.49E-12	3641737
CR_06070W_A	9.650586	3.09E-09	3644840
ATP17	9.595299	4.52E-12	30515364
C6_03050C_A	9.58327	0.001212	3639270
CR_03950W_A	9.579202	0.01233	3640960
PRN4	9.53016	1.33E-05	3639877
C5_04550W_A	9.528747	0.000133	3636508
POR1	9.509977	2.65E-24	3644698
FUM11	9.456687	3.75E-12	3641016
C3_00520W_A	9.452368	0.001342	3640513
SCP1	9.446528	0.012955	3646554
CR_07110C_A	9.432651	0.002017	3648224
C4_03290W_A	9.428173	2.14E-09	3637887
PHO89	9.39985	2.42E-10	3635633
IST2	9.382877	1.46E-14	3646662
BFR1	9.379688	1.11E-14	3635579
STT3	9.369388	8.3E-11	3635743
CR_05200C_A	9.35318	1.4E-05	3648080
SNG3	9.324976	1.26E-08	3646715
ACO1	9.31453	5.37E-21	3642090
MTS1	9.313708	4.87E-12	3634873
ASE1	9.311285	0.000138	3641729
INP51	9.29907	1.39E-05	3643806
C3_05290C_A	9.253093	0.001786	3635203
CPH1	9.248806	2.08E-06	3644468
C7_03350C_A	9.218156	0.013924	3646722
SEC18	9.214114	7.64E-11	3636208
CR_01280C_A	9.195649	1.86E-05	3640400
APL2	9.182041	2.02E-08	3638343
CAR1	9.14783	3.36E-12	3636503
ADE1	9.130677	5E-10	3639722
C2_09760W_A	9.122612	0.000189	3643807
C2_07390C_A	9.1038	6.89E-11	3645229
EGD2	9.091137	1.98E-13	3635284
HTB1	9.059746	4.32E-15	3645659
TPD3	9.039165	5.12E-11	3638512
SUB2	9.015351	5.52E-15	3645033

KIP2	8.960555	2.31E-05	3643954
C1_05950C_A	8.945637	4.3E-10	3639859
PMT1	8.941529	6.22E-14	3641393
MNS1	8.927177	6.79E-08	3644692
ADE6	8.898062	1.52E-14	3647304
HIS1	8.892705	1.14E-06	3636501
VMA8	8.885336	5E-08	3642798
ACC1	8.883258	1.52E-25	3639688
EGD1	8.880135	2.05E-11	3645340
CEX1	8.87234	0.000265	3644913
C1_06630W_A	8.814627	4.74E-05	3639544
GIN1	8.814083	3.24E-05	3644539
TRP4	8.76954	0.002422	3642904
SPC34	8.741347	0.018656	3641173
BUB1	8.740225	1E-07	3637922
C6_04280W_A	8.723266	2.05E-09	3646279
C2_02020W_A	8.696522	2.39E-09	3635769
BMH1	8.658374	1.77E-19	3636810
ATP5	8.645678	9.73E-15	3640521
ALG8	8.631882	0.002588	3636562
RPP2A	8.622932	1.83E-10	30515439
DPP2	8.60441	0.002425	3645341
C4_04440W_A	8.58362	3.08E-09	3641177
LHS1	8.548649	1.58E-10	3643032
CYK3	8.511661	0.000414	3639539
SDH12	8.495604	3.67E-13	3642775
CAS1	8.476569	0.000375	3645324
C7_00190W_A	8.436592	1.57E-07	3637969
VPS1	8.382092	1.95E-13	3642656
RIC1	8.350939	0.000421	3639331
C2_06920C_A	8.288313	1.69E-05	3643524
RHO3	8.283873	2.68E-07	3643448
CAM1-1	8.278231	5.52E-10	3646358
C5_00340W_A	8.263425	2.12E-05	3641555
FRS2	8.228056	3.04E-11	3636824
VMA7	8.210034	0.000592	3637332
MCR1	8.172543	1.3E-14	3647561
RPS15	8.147919	9.15E-13	3635194
PRD1	8.137728	4.02E-12	3642471
CR_00130C_A	8.1292	3.98E-07	3639709
ADK1	8.060314	4.22E-10	3644533
STV1	8.051537	3.45E-12	3646070
AAT1	8.032563	6.98E-13	3643468
NRM1	8.012891	0.003337	3639343
VAM3	8.005399	0.000439	3635146
SPC2	7.995808	0.003164	3645769
MET15	7.977717	4.24E-11	3645031

C2_02310W_A	7.970895	9.13E-07	3635809
C3_01940C_A	7.964975	1.95E-07	3636600
C1_09670C_A	7.953908	0.022494	3634872
CR_04850C_A	7.953374	0.022442	3647295
SKN1	7.946307	8.22E-13	3641755
C1_12180C_A	7.942181	0.000519	3642038
KAR2	7.896332	2.51E-17	3638830
AYR1	7.889951	9.37E-06	3647754
IDP2	7.889912	1.14E-10	3644934
CAP4	7.882325	0.003536	3637335
RIP1	7.865597	1.65E-11	3635204
CR_00880W_A	7.86341	0.003399	3640397
C1_10510W_A	7.842454	0.023028	3647189
C5_05040W_A	7.818676	9.22E-05	3636488
AAP1	7.812421	0.000516	3647397
C4_06460C_A	7.796443	0.003451	3642791
PHO113	7.781413	0.02444	3644946
ESP1	7.767328	1.53E-05	3640197
C7_03780C_A	7.75079	0.00379	3643119
CR_02210W_A	7.718089	2.22E-13	3644943
C1_02700C_A	7.708059	6.75E-10	30514972
C4_03400W_A	7.686008	0.004214	30515256
SDH2	7.685686	2.39E-09	3646397
PUP2	7.67011	1.05E-09	3646617
C4_00910C_A	7.665241	0.000616	3635679
C7_00410C_A	7.654614	1.52E-07	3638033
VRG4	7.633069	5.47E-08	3641768
RTT109	7.615836	0.025479	3639678
C4_03770W_A	7.603655	0.003762	3645354
GWT1	7.59385	1.76E-06	3645575
C2_02260W_A	7.583973	2.89E-06	3635778
C3_06410C_A	7.577567	0.000766	3642261
IRO1	7.569452	1.27E-12	3636648
RAD53	7.481906	0.003998	3645633
C1_08730W_A	7.478854	0.000646	3634938
CR_10140W_A	7.415088	1.67E-12	3638972
C2_01260W_A	7.410701	0.004161	3638792
CR_06510W_A	7.385056	0.000689	3645264
C6_00110C_A	7.383024	2.2E-05	3647896
C4_05080C_A	7.381074	2.16E-05	3641129
C7_02520W_A	7.374264	0.029796	3636941
C6_01360W_A	7.367841	0.000317	3641608
ATP4	7.365607	8.51E-13	3643418
HGC1	7.347499	6.18E-09	3639349
RAD52	7.333958	0.000137	3647148
C1_13270W_A	7.331266	2.98E-05	3636144
NUC2	7.303643	1.05E-09	3637030

SNF5	7.289486	5.68E-06	3648297
ARE2	7.285744	9.86E-09	3643526
C6_00930C_A	7.250979	6.78E-07	3644032
ALG6	7.244575	3.62E-05	3647316
TEF2	7.232087	9.92E-21	3646502
C2_09780C_A	7.222129	1.52E-07	3643805
RPL43A	7.211315	1.77E-11	30515319
CAT2	7.209105	2.64E-10	3635659
C6_02560W_A	7.20346	1.12E-05	3639205
CLB2	7.198186	7.29E-10	3635840
ABP2	7.196143	0.000806	3643443
SAP4	7.188097	0.031069	3640257
PEX8	7.183862	0.000187	3647393
SUR7	7.179139	1.33E-16	3641610
HIS5	7.178124	0.004982	3645756
DED81	7.148543	4.15E-12	3646227
CHL4	7.148132	0.030211	3646131
DBF2	7.147523	2.15E-07	3648101
C1_10540C_A	7.103274	0.000252	3647193
ATP14	7.094073	1.92E-09	30515134
C3_04780C_A	7.09259	6.63E-07	3635214
STE23	7.087355	4.68E-10	3639236
C4_01460C_A	7.067962	0.005598	3635622
C1_08080C_A	7.053401	1.04E-06	30515029
C7_01320W_A	7.041652	2.11E-07	3645896
C4_06160W_A	7.025781	0.004944	3642819
PCK1	7.022082	1.32E-12	3639701
CHS8	7.009776	5.71E-13	3640532
C6_02530C_A	6.98175	0.001239	3639175
AGE3	6.968304	3.01E-07	3644565
SMC3	6.957844	0.005142	3638408
C1_10520W_A	6.943515	0.03425	3647191
PST2	6.925735	0.001146	3643909
FRP3	6.920559	1.37E-14	3648102
YPT72	6.920542	1.71E-06	3643523
GLO3	6.91389	1.92E-13	3640556
SSO2	6.89545	6.04E-11	3643809
C1_14180W_A	6.88485	8.16E-08	3643324
TFC4	6.883027	4.15E-09	3638420
C5_03950W_A	6.883011	4.61E-05	3647445
C5_00060C_A	6.882171	5.27E-05	3647889
CR_09050C_A	6.879047	0.001157	3648197
C3_01590W_A	6.831143	0.000332	3636666
CMK2	6.82404	1.54E-09	3643961
SMF3	6.819669	5.01E-08	3638852
C4_05790W_A	6.818539	1.09E-05	3641784
C1_10750C_A	6.818205	0.00553	30515043

C1_08140W_A	6.816755	0.005589	3640730
C4_03860C_A	6.800757	0.006248	3647854
C1_00420W_A	6.781184	5.18E-08	3639286
FAS1	6.769636	2.24E-20	3641593
C5_05350W_A	6.769237	0.0004	3636401
CSM3	6.756903	0.03727	3644083
CAM1	6.749842	2.16E-15	3641734
STE24	6.736175	4.16E-11	3645040
CR_05680C_A	6.735029	0.000393	3646684
C3_02570W_A	6.731907	7.6E-07	3638375
C3_05280C_A	6.719871	0.000381	3635202
C3_00850C_A	6.715121	4.87E-14	3647748
C1_02330C_A	6.710716	4.66E-07	30514969
TOS4	6.693881	3.03E-09	3644548
SMP3	6.689076	0.038235	3642980
C2_02670C_A	6.687622	0.038387	3643008
C5_01960C_A	6.687296	7.72E-08	3643738
CR_01320C_A	6.668859	1.34E-05	3640386
RPS12	6.666639	7.72E-10	3638438
C1_07510W_A	6.652265	0.006532	3646664
APM4	6.648104	0.001419	3642297
C7_01460C_A	6.626322	0.001924	3636958
CR_07460C_A	6.618287	1.85E-07	3646049
RSR1	6.586073	1.1E-10	3640145
C3_00170C_A	6.575777	1.39E-06	3640589
C1_11760C_A	6.545806	0.000499	3645328
ADO1	6.542896	8E-10	3639274
C3_01610W_A	6.525595	4.95E-10	3636664
C1_05490C_A	6.523794	3.63E-06	3642507
RPL8B	6.522765	4.05E-10	3635198
C5_04260W_A	6.518286	0.00216	3636451
AHP2	6.509268	4.42E-06	3636977
KGD1	6.496019	7.97E-12	3647752
C1_03490W_A	6.494101	9.61E-07	3636914
RAP1	6.483809	6.93E-06	3643945
VMA13	6.481918	2.23E-09	30515402
ASN1	6.48163	1.05E-10	3645790
YBL053	6.470296	9.32E-05	3647045
CRD2	6.465838	0.040993	30515234
GAL4	6.457726	1.05E-12	3641244
NUF2	6.455082	0.000677	3642613
RDI1	6.451771	2.51E-11	3635167
CPY1	6.44833	3.49E-12	3646721
C1_05670W_A	6.4425	2.77E-09	3639899
C7_03160W_A	6.437378	8.79E-10	3641345
C5_05230C_A	6.419824	2.7E-11	3636470
GIT2	6.413379	1.83E-08	3642633

PHO114	6.410801	0.043618	3635644
C2_06890C_A	6.404977	0.007775	3643522
MDH1	6.396861	4.7E-11	3639667
C5_01260W_A	6.385111	0.043877	3642615
C2_08930W_A	6.370181	5.35E-08	3645795
INT1	6.340257	2.74E-12	3637761
CR_00060C_A	6.330469	3.36E-09	3639673
INO1	6.325914	8.8E-20	3638966
THI13	6.303743	0.007798	3641921
C2_09970C_A	6.287768	2.63E-09	3643820
SEC8	6.286974	1.85E-06	3647078
C1_01950C_A	6.279151	0.00273	3645421
PFY1	6.276943	6.92E-10	3640703
C7_03310W_A	6.274967	0.008463	3646726
KAR9	6.264609	0.000124	3636136
LMO1	6.258409	6.12E-09	3641403
C4_00770C_A	6.256192	9.7E-06	3645760
NDH51	6.250215	4.6E-10	3645444
UGA32	6.244484	0.002567	3639333
TOM20	6.240747	7.39E-06	3636817
C1_00830W_A	6.239427	0.000674	3639344
ORC3	6.227485	2.29E-06	3645639
C1_05320C_A	6.227263	0.000152	3642470
C3_02290W_A	6.224708	3.46E-07	3636556
VPS51	6.185246	0.002681	3639242
CDC20	6.180834	5.1E-06	3644057
C4_00190W_A	6.175423	0.008845	3645030
C1_11280W_A	6.171893	7.95E-06	3644554
FMO2	6.164036	0.009186	3637440
TMA19	6.160149	2.09E-12	3640399
IHD2	6.151718	1.31E-05	3639359
CR_00690C_A	6.139352	0.000138	3640390
C1_00210C_A	6.136751	6.31E-10	3639335
IFA21	6.13143	0.000591	3641344
FCY2	6.129315	1.43E-05	3638365
CDC54	6.105857	0.002932	3647606
C2_10470C_A	6.09665	1.33E-06	3641196
KSR1	6.090427	0.000633	3646052
PHM7	6.080002	1.44E-14	3642329
HOC1	6.063038	9.59E-09	3641605
TLO9	6.05133	3.76E-07	3648051
TFP1	6.044547	1.43E-11	3636662
C2_04830W_A	6.021559	0.013718	3648115
CIT1	6.011129	9.75E-09	3643201
C4_00410W_A	6.005538	3.48E-06	3645019
BNR1	5.976704	1.49E-05	3639672
FGR34	5.954773	3.17E-07	3639033

TPI1	5.950175	1.46E-16	3638482
CR_01070W_A	5.92746	0.011552	3640382
CDC50	5.919618	4.71E-06	3640302
ARC40	5.919195	3.41E-08	3644866
NIF3	5.914701	0.000242	3646527
C4_02470C_A	5.91234	1.23E-08	3637869
GRP1	5.911311	0.011015	3634899
CSE4	5.910877	9.48E-05	3647750
C1_02650W_A	5.902907	4.26E-07	3636818
C3_06920W_A	5.899007	6.84E-05	3638511
ZRT2	5.898691	1.55E-06	3635861
C4_04330C_A	5.88892	5.36E-10	3646840
C6_04120C_A	5.883169	5E-06	3647974
CR_01080W_A	5.882735	3.88E-08	3640381
CRN1	5.868345	1.57E-08	3637034
TLO1	5.866836	4.7E-07	3639677
SNL1	5.860615	9.82E-06	3647192
CTA2	5.853206	3.59E-08	3639372
CDC24	5.840882	8.64E-09	3643737
C3_03510C_A	5.829573	1.76E-05	3638310
PLD1	5.811747	1.19E-11	3645329
YPT31	5.803956	9.56E-09	3647999
C7_03800W_A	5.80235	0.00407	3643150
CR_09530C_A	5.800872	4.65E-10	3647101
PXA2	5.798773	5.31E-08	3642055
C2_10860C_A	5.785455	3.27E-09	3641233
C1_02370C_A	5.742169	7.24E-05	3644576
C1_12880C_A	5.741625	6.21E-06	3636151
C4_04920W_A	5.735818	0.000912	3641172
IDP1	5.732975	1.68E-05	3647474
ALG9	5.715766	3.95E-07	3644457
DOT1	5.712578	6.9E-06	3642236
C2_01160W_A	5.684851	0.000355	3638861
SPF1	5.678815	9.8E-10	3646573
USO5	5.659631	0.00125	3638398
C6_00270W_A	5.654231	7.84E-06	3646066
RTS1	5.64666	5.15E-12	3646557
ERG12	5.625544	6.71E-06	3634859
MES1	5.608782	7.88E-08	3636516
PST3	5.604963	3.27E-07	3648031
C1_00450C_A	5.602404	2.74E-05	30514955
C1_01250W_A	5.586444	2.82E-06	3640212
IFA4	5.58394	0.013291	3645454
C5_00840W_A	5.576299	0.013645	3644130
C7_03410C_A	5.568258	0.000102	3646716
CLA4	5.56094	1.38E-08	3634878
ACO2	5.552957	0.005359	3646708

MNN24	5.542913	1.88E-08	3638789
C2_07420W_A	5.539326	0.000165	3645202
C4_06810C_A	5.535985	1.52E-08	3642916
SMC5	5.527198	0.00116	3645103
ERG1	5.524911	2.49E-13	3646509
C5_04420W_A	5.520954	0.000114	3636411
FESUR1	5.50818	1.05E-07	3638514
CR_05010W_A	5.506191	0.014991	3646389
C3_01510W_A	5.503189	0.000136	3636571
GCF1	5.462695	5.2E-10	3646498
ZRT1	5.445165	0.018221	3642970
RBT5	5.440724	4.67E-15	3645007
CR_02620C_A	5.43208	6.94E-06	3642567
GPX3	5.430921	0.001406	3644471
C1_12400C_A	5.426034	0.001425	3642059
C1_02780W_A	5.421578	8.11E-07	3636871
C5_01410C_A	5.421089	4.67E-06	3647054
PMT5	5.41618	0.000717	3639011
FGR29	5.403552	0.001001	3641190
FUM12	5.399649	2.37E-08	3638489
C2_05560W_A	5.395828	0.005705	3645561
APG7	5.378236	0.000603	3646615
ERG27	5.376908	2.91E-06	3640432
C7_02080W_A	5.375502	0.001878	3637025
RPP1B	5.374586	1.09E-08	3643104
C1_10730W_A	5.353166	1.39E-05	3644404
SAH1	5.346672	1.2E-11	3636452
C1_14240W_A	5.334143	7.21E-07	3643306
TYR1	5.329202	5.68E-05	3635639
CR_04870C_A	5.320423	0.016499	3647310
CAC2	5.320423	0.016514	3646978
C2_05130W_A	5.312735	0.002104	3643458
C6_03310W_A	5.29924	1.24E-05	3639197
FGR38	5.288254	2.15E-05	3645412
SEC62	5.283358	1.05E-05	3636860
RFC2	5.273935	0.005711	3637939
C2_08340C_A	5.265967	0.000357	3648165
C3_03160C_A	5.254191	0.017646	3638424
LEU2	5.22374	0.005901	3638034
PYC2	5.221457	5.27E-11	3637323
ENA21	5.220159	1.91E-08	3641392
DSL1	5.182111	0.005824	3645718
ATP7	5.181914	7.27E-06	3646672
C4_04160W_A	5.176446	3.27E-08	3641185
ABP1	5.171068	3.99E-09	3637875
CPR3	5.16244	2.01E-08	3635812
TIP1	5.154437	0.001036	3636512

CR_07170W_A	5.15195	4.65E-06	3645271
C1_05920W_A	5.151578	0.000102	3639864
ACS1	5.142748	5.08E-10	3643941
C1_04030W_A	5.139041	0.007485	3646428
NPT1	5.132033	4.38E-06	3643109
C2_05850C_A	5.123361	0.000123	3647472
JEM1	5.123227	0.019188	3643876
SAS2	5.11847	0.019383	3638817
NPL4	5.116065	0.000126	3639820
PDC11	5.114137	7.33E-15	3642780
SEC61	5.094066	2.41E-09	3647462
PMS1	5.092798	0.006539	3647714
GPI7	5.091507	0.000293	3647672
C1_01050C_A	5.084319	9.94E-06	3640170
C2_05170W_A	5.05579	0.000244	3643462
C1_11860W_A	5.049583	4.71E-08	3642009
C1_07040C_A	5.042359	0.002647	3639533
C2_01320W_A	5.038038	1.4E-05	3638846
CR_04080C_A	5.0326	6.49E-09	3641023
RPS7A	5.025355	2.65E-09	3636573
RAD51	5.023679	2.09E-06	3644944
ZCF24	5.02241	0.002711	3645417
MDH1-3	5.003883	4.25E-06	3641197
C3_04970C_A	5.003507	6.68E-08	3635164
MAE1	5.000903	1.67E-10	3641615
C4_02400C_A	4.989734	0.007671	3637851
SET3	4.98443	1.47E-10	3643320
C2_02390W_A	4.980187	5.43E-12	3635829
MNN10	4.973065	2.29E-05	3644997
C6_02160W_A	4.968333	0.001104	3645889
PET9	4.945094	3.45E-15	3641587
C3_00210C_A	4.929666	0.022963	3640560
KIP4	4.92692	5.53E-08	3642032
CTA24	4.912857	2.52E-08	3636475
C2_03210W_A	4.912298	0.024345	30515098
C1_12240C_A	4.90766	0.000496	3642023
C1_08800W_A	4.903442	0.00012	3634791
FGR51	4.902489	0.000136	3647796
MPT5	4.901949	1.43E-05	3636205
C1_06480C_A	4.894841	0.000111	30515008
ARP2	4.876935	8.16E-09	3641915
TOP2	4.875902	2.39E-05	3642777
RPP1A	4.87197	4.61E-08	3636839
C1_07440W_A	4.866918	3.25E-08	3644482
ARP1	4.86523	0.026135	3646349
RCT1	4.862239	2.17E-12	3641727
APT1	4.86149	3.03E-05	3635842

C2_03340W_A	4.857356	0.000198	3637392
ALS1	4.844023	8.13E-15	3640280
CHS6	4.835612	5.72E-05	3641377
SGT2	4.834307	1.15E-08	3642978
RIM1	4.823543	1.34E-05	3639898
LAP3	4.812732	2.86E-06	3641012
APM1	4.811741	1.68E-05	3636424
AMN1	4.802065	0.001683	3635795
CR_09750C_A	4.79744	1.15E-07	3639009
CR_04500C_A	4.793084	6.23E-05	3641014
TSC11	4.791043	0.025969	3645267
CR_02380C_A	4.789297	0.027321	3644931
C2_09650W_A	4.781527	4.83E-09	3643794
C1_10460W_A	4.777476	1.33E-06	3647183
CR_04730W_A	4.77224	0.008317	3647305
END3	4.753791	7.13E-05	3636644
HIS7	4.751371	0.000724	3647868
WBP1	4.749936	3.71E-08	3644398
SER1	4.74074	6.43E-06	3646909
HEM14	4.729932	8.12E-05	3634923
EFB1	4.717119	2.18E-10	3641125
SEC72	4.71559	3.63E-05	3645768
LIP8	4.698333	0.000329	3646727
CR_07230W_A	4.690092	0.000113	3646031
C1_11120C_A	4.686563	4.58E-08	3644412
APN1	4.679668	8.8E-05	3642263
HBR2	4.676668	9.69E-08	3646273
GRS1	4.675801	9.01E-10	3642474
SHM2	4.65971	5.84E-10	3640306
HFL1	4.658173	0.030459	3636883
C3_04080W_A	4.608218	3.87E-07	30515200
C3_06610W_A	4.601129	0.000268	3642248
C6_01750C_A	4.594481	0.031586	3641640
EAP1	4.593713	1.45E-08	3643814
C1_04580C_A	4.590598	0.000295	3646132
PTC6	4.582483	5.29E-05	3647351
HYU1	4.56372	3.32E-07	3643024
C1_13280C_A	4.561967	0.000202	3636145
SHP1	4.560839	4.64E-08	3640083
GSP1	4.560464	2.22E-09	3646925
C1_02320C_A	4.559506	4.92E-08	3644572
C3_00910W_A	4.559009	0.010622	3647755
BEM2	4.544883	1.87E-08	3637044
MMD1	4.539701	1.04E-07	30515015
C5_03780C_A	4.536258	0.03366	3647232
C1_02970W_A	4.530646	2.21E-07	3636835
LEU1	4.530288	6.85E-07	3639685

C3_01820W_A	4.53021	9.9E-07	3636584
C2_09250W_A	4.527062	0.03431	3644065
POL3	4.523402	1.3E-05	3641359
CR_05450C_A	4.52183	0.001091	3648236
CR_04880W_A	4.512655	0.005846	3647309
C3_04630W_A	4.506323	0.001661	3635189
CR_09010C_A	4.499557	4.44E-07	3641892
RPL20B	4.497022	3E-07	3635615
C3_03080W_A	4.488886	0.001248	3638397
CR_09670C_A	4.485534	0.000195	3647091
SEC24	4.484766	9.27E-10	3634939
APL5	4.481823	1.65E-05	3638377
MSH2	4.471674	7.79E-06	3642939
SEC27	4.468814	1.35E-07	3645870
C3_04460W_A	4.46621	2.17E-05	3635208
C4_03720C_A	4.45977	7.75E-05	3645359
C1_13810W_A	4.455856	6.48E-06	3636126
C4_01840C_A	4.453632	0.000555	3635592
MON2	4.450766	1.77E-07	3636201
C4_04090C_A	4.449859	0.000427	3641225
C2_03450W_A	4.436123	0.006239	3637349
C2_09910C_A	4.427033	0.039452	30515162
IDH2	4.417006	2.33E-09	3642979
ARO3	4.417004	2.14E-05	3635770
C3_02190C_A	4.413552	1.55E-05	3636642
RFC4	4.411984	0.000454	3638994
C3_04740C_A	4.408356	0.012239	3635251
MP65	4.405542	1.57E-12	3643951
MNN2	4.403548	8.8E-05	3644405
ARL3	4.397841	0.000235	3644397
RNR21	4.368677	6.86E-08	3643021
SEC26	4.362426	3.45E-08	3641005
C4_03810W_A	4.360424	0.000591	3645350
CR_01430W_A	4.358867	0.0166	3640138
ADE5,7	4.358559	1.17E-09	3640719
C4_03990C_A	4.354535	0.042388	3648295
C3_04590W_A	4.3503	1.34E-07	30515204
C3_01910C_A	4.34721	8.4E-06	30515182
ARP9	4.344474	0.000233	3639829
C3_07450C_A	4.336863	0.007279	3638481
C7_00150W_A	4.326874	9.78E-08	3637973
C1_11250W_A	4.322569	3.07E-05	3644556
C1_01010W_A	4.321023	9.88E-05	3640181
C1_11740W_A	4.311867	0.013615	3645317
CR_08670C_A	4.310127	0.011438	3642126
FBA1	4.30633	1.43E-12	3635585
RPL18	4.305872	1.07E-07	3635156

SHM1	4.304275	1.98E-07	3646724
CRC1	4.303743	0.000269	3640112
ARO4	4.300636	3.21E-07	3647668
YBN5	4.291708	2.47E-07	3644781
GLO2	4.286105	0.001691	3644067
ANB1	4.279762	1.84E-07	3641648
C7_01480W_A	4.279339	0.001861	3636956
BUB2	4.271703	0.040318	3643030
ASR1	4.252986	4.76E-07	3644402
DCK1	4.248252	6.55E-12	3637383
RPL10A	4.244263	1.59E-07	3645868
RPL35	4.244227	1.24E-05	30515207
C1_00920W_A	4.243463	3.05E-05	3639283
C2_07110C_A	4.234596	0.003377	3643505
LTE1	4.233413	1.03E-07	3643495
C6_01560W_A	4.223842	0.008787	3641652
C1_06200W_A	4.210066	7.02E-05	3648300
ADE13	4.203371	3.84E-08	3644863
CR_06560C_A	4.202478	3.83E-06	3648367
C2_02530W_A	4.201696	0.00422	3635819
KTR2	4.200703	0.043864	3645443
CR_02570C_A	4.192683	0.004165	3647685
UAP1	4.192342	4.94E-07	3637769
C2_07270W_A	4.180216	0.001277	3643474
CR_01180W_A	4.17633	0.000283	3640427
C6_02030C_A	4.171694	6.1E-05	3647562
C1_03440C_A	4.16333	4.28E-06	3636908
KEX2	4.157737	1.65E-07	3634914
CDC7	4.151488	0.00226	3643424
C1_07840W_A	4.151236	1E-07	3640737
C2_10830W_A	4.141881	0.045652	30515170
C4_05810W_A	4.140344	4.04E-08	3641786
KIP3	4.122413	0.00905	3641716
TRP99	4.121301	7.22E-05	3642494
GDB1	4.120347	1.61E-07	3647409
C1_07340W_A	4.118047	2.76E-06	3644472
POL2	4.115241	3.48E-05	3645733
UBC15	4.112786	0.00071	3641260
COX4	4.109181	3.34E-07	3635737
CR_03020C_A	4.105325	0.000656	3642598
LYS12	4.102501	9.41E-06	3640134
C5_01550C_A	4.095191	1.22E-05	3647063
YCG1	4.094922	8.16E-05	3636639
C2_08650W_A	4.093664	1.36E-05	3643908
PXA1	4.090256	1.92E-07	3639713
STE13	4.087343	0.002152	3647597
SAC6	4.083395	5.75E-07	3639213

C1_09070W_A	4.082113	2.5E-07	3634773
C3_00470W_A	4.078698	4.87E-05	3640520
C1_12740W_A	4.077297	1.46E-05	3646819
CR_09840C_A	4.074896	0.047485	3638988
CR_08560C_A	4.067326	2.15E-05	3642142
C1_07780W_A	4.058419	1.97E-05	3647955
C7_01930C_A	4.05414	0.001394	3637027
PGA49	4.050576	0.00043	3646525
SEC4	4.046363	1.91E-07	3640119
C1_12700W_A	4.044141	0.002199	3646831
C1_10090C_A	4.036895	5.43E-07	3634817
RPL19A	4.034525	1.43E-09	3635257
MAK32	4.032849	0.020331	3645411
C2_09560C_A	4.021665	6.51E-05	3643813
C3_02210C_A	4.018605	0.001092	3636641
CR_02500W_A	4.018452	0.007254	3647696
SMI1B	4.017198	0.021273	3643675
C7_00600C_A	4.015888	0.009939	3638031
RPS1	4.008904	4.08E-09	3636924
C6_01960W_A	4.007659	0.023025	3644559
BET4	4.007087	0.048642	3644695
SNG4	4.005396	4.09E-06	3646714
CR_07350W_A	4.00125	8.38E-07	3646055
PSD2	4.001047	6.61E-08	3636515
C1_14170W_A	3.99716	1.24E-06	3643323
C4_00380W_A	3.995847	0.006964	3645015
C7_00120W_A	3.99424	0.00063	3637976
RBF1	3.990933	0.000102	3639251
C5_02400W_A	3.987987	3.93E-07	3637724
ERG7	3.980072	7.49E-07	3635761
RPL28	3.977912	3.47E-08	30515386
CAK1	3.969309	0.002017	3637352
PDS5	3.969215	3.89E-05	3642341
GLO1	3.960958	8.88E-06	3639312
C1_06340W_A	3.950688	0.010794	3639499
MVB12	3.947865	0.010601	30515400
GYP1	3.947422	6.93E-06	3641138
PGA33	3.946693	0.000613	3637350
C3_05550C_A	3.936698	0.000307	3643659
SEC34	3.934855	5.26E-06	3644455
C1_00330C_A	3.92947	0.003304	3639296
CDC47	3.923167	1.16E-05	3645780
C4_07240W_A	3.923013	0.000183	3642945
C4_00330C_A	3.903292	1.41E-05	30515227
RPS8A	3.902278	3.78E-08	3645565
C6_03260W_A	3.902167	2.14E-06	3639183
RFC3	3.90022	0.021599	3647440

MNN21	3.89618	6.44E-05	3648124
RBD1	3.895846	2.74E-07	3642018
THS1	3.894928	8.02E-08	3647882
C3_05410W_A	3.893253	1.23E-06	3643665
CHT4	3.891242	0.023823	3635768
HEM13	3.880623	4.22E-08	3647390
C4_03050C_A	3.880615	0.002421	3637930
PRE2	3.871251	1.65E-06	3643490
ERG8	3.869657	4.15E-05	3635640
COX6	3.867827	5.96E-07	30515099
FRE7	3.867645	0.000153	3646024
UBA2	3.865193	0.00246	3640701
C3_00790W_A	3.856582	0.002757	3648186
GNA1	3.855674	0.003339	3637397
C2_01690W_A	3.848701	7.75E-07	3635728
CR_04020C_A	3.844879	0.003815	3640969
UGP1	3.840224	3.87E-09	3648144
C1_13320C_A	3.823106	8.93E-07	3636149
C3_03340C_A	3.817888	0.000731	30515195
PLC1	3.810513	0.000768	3647860
SCL1	3.803621	1.01E-06	3640510
ZCF39	3.800071	9.23E-08	3639037
COQ3	3.799416	0.00091	3641630
C4_01760W_A	3.796863	6.93E-05	3635584
ILV6	3.783908	0.000125	3635598
CR_04560C_A	3.781311	0.003071	3640946
FRE30	3.776211	0.000456	3646025
DQD1	3.775649	0.003061	3643488
C6_03580W_A	3.773204	0.012214	3640250
PEX6	3.764986	0.000554	3643436
LRO1	3.763657	3.94E-06	3639356
ARC19	3.762429	0.026322	3640418
C6_01050W_A	3.762213	0.000744	3644015
C7_02100W_A	3.759138	7.91E-07	3637023
C5_04770W_A	3.755994	0.003314	3636444
HGT7	3.745356	1.99E-11	3638876
ATP16	3.741716	5.09E-07	3638963
CR_07370W_A	3.739389	0.001723	3646053
MCT1	3.739183	0.000106	3646683
C3_06690C_A	3.722534	0.009632	3642256
AMO2	3.72126	0.028424	3648021
ECM39	3.716236	0.003094	3641386
AIP2	3.711793	5.04E-07	3638393
C1_10320W_A	3.70886	0.001184	3634907
CNB1	3.707078	0.000764	3636463
C3_01210C_A	3.704748	1.45E-05	3636612
XKS1	3.69496	3.58E-06	3648306

HEM4	3.688801	0.010327	3643942
GPR1	3.685655	2.83E-06	3642616
CMD1	3.668275	2.77E-07	3646520
ISC1	3.668254	0.01436	3646705
IFK2	3.666597	0.004523	3637439
C6_00890W_A	3.660086	7.7E-08	3644028
KRE5	3.648251	0.000105	3638384
C5_01220W_A	3.641778	1.13E-05	3642620
C5_02050W_A	3.63684	1.67E-06	3643768
C2_05800C_A	3.632722	0.000603	3645584
C1_02290C_A	3.63204	0.014481	3644568
REV3	3.620795	0.000168	3641749
RIB5	3.616221	6.65E-07	3636499
C6_04250W_A	3.613162	2.14E-06	30515345
SEC3	3.610526	4.21E-06	3642806
C5_02900W_A	3.608352	8.89E-05	3637747
C3_05300C_A	3.602819	0.017078	3643646
EIF4E	3.585272	1.88E-05	3638981
C2_07980W_A	3.580029	0.014211	3642312
MUM2	3.5737	2.27E-05	3636432
CR_08050C_A	3.55979	1.28E-06	3644133
BUD7	3.559085	1.5E-06	3647228
ROB1	3.554709	1.1E-06	3636212
C6_00920W_A	3.554118	0.000294	3644031
GEA2	3.54232	2.63E-05	3636646
C3_06860C_A	3.538355	2.95E-05	3638541
PNP1	3.536791	3.78E-05	3638426
CR_10020C_A	3.536736	0.003019	3639032
C3_01720C_A	3.532007	2.01E-06	3636601
C4_04280C_A	3.531882	0.039524	3646846
YSA1	3.531622	0.003322	3638516
OBPA	3.531384	5.86E-07	3643764
OCA6	3.530182	0.035676	3643336
C1_10630C_A	3.526559	0.01657	3647683
PGA59	3.524902	7.74E-10	3637881
C2_04410W_A	3.516472	0.003245	3645455
C7_03660C_A	3.492643	3.17E-07	3646226
C6_03990C_A	3.491981	0.014574	3640271
C2_04490W_A	3.488397	3.1E-05	3645463
C6_00200C_A	3.485133	0.000165	3646082
MSO1	3.484604	4.86E-06	3642040
FLC2	3.481019	2.36E-06	3647962
C7_03200C_A	3.478662	0.001467	3641342
VMA4	3.475399	7.72E-06	3640111
C1_04190C_A	3.469086	0.006327	3644717
C1_02680C_A	3.467074	0.003939	3636822
C3_04810C_A	3.46589	1.71E-06	30515205

PGA13	3.46242	0.009748	3642138
APS3	3.456985	0.018028	3646491
C6_03820C_A	3.456443	0.000199	3640288
C2_02840C_A	3.452996	0.000257	3642976
C6_02470W_A	3.452356	0.000244	3639169
CR_05880W_A	3.450095	0.020397	3646702
AHP1	3.443966	1.11E-07	3637850
C7_03050W_A	3.429788	0.003968	3641378
C6_04340W_A	3.424175	0.001059	3646291
C7_00420C_A	3.420724	0.004433	3638032
RPN7	3.420214	3.62E-06	3641909
RPS26A	3.41782	1.04E-05	3635787
BNI4	3.412552	0.000142	3646406
C4_04250W_A	3.411449	0.001236	3646855
BRN1	3.409033	0.004821	3641815
C1_02060W_A	3.408492	0.0037	30514968
C1_13190W_A	3.405297	1.21E-07	3636163
NCE4	3.393351	0.043088	3641194
ARH2	3.387865	0.00141	3643411
PGA52	3.385157	3.16E-06	3647908
ALG7	3.381445	0.000706	3642291
C1_00320W_A	3.374982	0.004336	3639297
C1_04280C_A	3.371444	0.047646	3644709
C6_02490C_A	3.361524	0.001034	3639171
CR_01300W_A	3.360644	0.002653	30515373
MCI4	3.358571	0.000707	3640118
C1_06190C_A	3.357098	1.16E-06	3648391
MSI3	3.35631	7.3E-06	3639821
PIKA	3.354817	4.92E-05	3643765
C1_08780W_A	3.350181	3.45E-05	3634789
CR_02260C_A	3.346211	0.02357	3644912
C1_01360C_A	3.339631	3.54E-06	3640191
GTR1	3.333435	0.004717	3643914
AGO1	3.326907	6.97E-06	3642828
C3_01420C_A	3.324142	0.017443	3636634
TFS1	3.317916	9.29E-06	3642655
BET2	3.315102	0.006239	3639025
DPB2	3.311326	0.024344	3639026
GLX3	3.311239	4.51E-07	3638325
C1_14530W_A	3.303632	0.000259	3643333
CR_03530W_A	3.291479	4.37E-06	3643204
LPD1	3.290654	3.83E-07	3646037
BMT3	3.284622	0.003826	3640439
C2_01930C_A	3.276089	0.000373	3635793
GRE3	3.27566	3.35E-06	3637811
PPH21	3.274533	1.14E-05	3636665
C3_07460W_A	3.271449	0.00017	3638480

C1_04560W_A	3.267656	0.002106	3646130
CYP5	3.262051	5.06E-06	3642257
CDC73	3.257675	0.000478	3636830
ASR3	3.256755	7.7E-06	3637402
MMS22	3.248272	0.0273	3639681
ACE2	3.242796	2.71E-05	3646034
C4_05800C_A	3.24063	0.028883	3641785
C7_00520W_A	3.237006	1.82E-05	3638001
MCM1	3.217991	1.31E-05	3638050
C6_00540W_A	3.210291	0.029069	3647146
C1_08680C_A	3.195963	0.000694	3634933
CR_05330W_A	3.193649	0.032987	30515404
UBI3	3.193105	1.13E-06	3642934
PKH3	3.188807	3.6E-06	3646075
ENO1	3.187419	8.8E-09	3646493
C4_01910W_A	3.185594	0.006769	3635635
C3_01180C_A	3.183587	0.000183	3647960
ATP20	3.175793	0.002465	3647602
TKL1	3.171513	3.06E-08	3640767
PGA14	3.16679	4.45E-07	3641560
NUP159	3.166775	3.1E-06	3647458
C3_04650W_A	3.164162	0.020693	3635192
C2_06110W_A	3.161751	0.000338	3644094
C3_00010C_A	3.159788	1.21E-05	3640600
PMT6	3.159053	0.000155	3641116
RPP0	3.158354	9.97E-08	3638023
SRD1	3.158159	0.001883	3635764
C7_00920C_A	3.15636	0.000786	3637957
ALG2	3.150181	0.000878	3647832
C3_04800C_A	3.14958	0.001615	3635216
C4_06940C_A	3.148927	0.020906	3642928
FEN12	3.134875	0.000153	3637404
C2_04170C_A	3.134568	7.17E-06	30515111
RNH1	3.131154	0.004666	3639237
RPS16A	3.130037	2.88E-06	30514974
C5_00750C_A	3.129349	6.43E-05	3644121
PHO88	3.128386	7.37E-05	3641898
C2_08620W_A	3.126618	5.73E-05	3643912
ARC15	3.12561	0.005027	3646043
RPN8	3.122803	1.81E-05	3643770
COQ6	3.116387	3.35E-05	3636878
RPB11	3.116136	0.046753	30514957
RNA1	3.114852	3.38E-06	3636595
C6_04560W_A	3.114224	3.59E-06	3645977
FRS1	3.110569	1.7E-06	3640121
C4_00590C_A	3.095626	3.1E-05	3645772
C2_00870W_A	3.090306	0.022563	3638804

ARO2	3.088934	5.99E-05	3648190
CR_08900C_A	3.081089	0.000532	3641908
C2_00730C_A	3.080022	0.006171	3638913
PAN3	3.07884	0.000252	3636464
SSB1	3.076467	5.36E-08	3642095
C5_00790C_A	3.074347	6.37E-06	3644125
VAC8	3.06824	1.57E-05	3647410
C3_03840C_A	3.06594	0.002284	3645628
C1_00630W_A	3.064151	0.022475	3639380
C1_04620W_A	3.058129	2.41E-05	3646135
C6_00910C_A	3.053586	4.92E-05	3644030
ADP1	3.047736	4.46E-05	3642458
C3_01950C_A	3.042456	6.16E-05	3636599
CR_10600C_A	3.032234	1.55E-05	3639004
C5_03400C_A	3.031851	0.029072	30515308
C7_02370W_A	3.030207	5.25E-06	3636930
C1_01000C_A	3.030042	0.026361	3640180
C1_10500W_A	3.030009	0.02419	3647188
RPS14B	3.028002	1.24E-05	30515006
CYS4	3.021606	6.32E-05	3645429
RPL17B	3.015674	4.98E-05	3645439
MET13	3.015583	0.003421	3638382
GPM1	3.012334	4.86E-08	3637431
CR_02390W_A	3.00883	2.51E-05	3644930
C2_04330C_A	3.006725	0.001948	3645453
CR_09090C_A	3.00555	0.023927	3641927
C6_03330C_A	2.9996	0.012186	3639199
MEA1	2.998406	0.040377	3635154
C1_04640W_A	2.993562	0.000194	3644761
RPL13	2.992073	8.45E-06	3636841
MDL1	2.984889	0.000125	3640146
RBK1	2.983782	0.00219	3646830
PGA56	2.980901	3.94E-08	30515313
SWC4	2.978935	0.000734	3639679
YKU80	2.978614	0.012199	3645138
C4_06240W_A	2.977854	0.025358	3642807
CR_08640C_A	2.975811	1.1E-05	3642112
C7_03260C_A	2.975718	0.025227	3641366
MKK2	2.971887	7.74E-05	3645580
C4_06020C_A	2.971383	0.00051	3646519
HYM1	2.965219	0.000809	3637355
C1_09750W_A	2.964691	0.003491	3634822
DBF4	2.964254	0.001909	3641388
RFC1	2.961563	1.85E-05	3645582
C3_04130W_A	2.960584	0.000227	3635283
C6_00080C_A	2.958251	0.012589	3647899
CR_02770C_A	2.952083	0.007853	3642581

C2_10700C_A	2.948504	0.001672	3641207
SPC98	2.943513	0.007853	3640113
C1_10680C_A	2.937548	4.85E-05	3647678
ARP8	2.937063	5.54E-05	3640200
RPN6	2.929297	1.33E-05	3645352
SEC1	2.925449	2.1E-05	3636935
YMC1	2.924357	0.014008	3644474
C3_06990W_A	2.92389	0.047641	3638521
MNN26	2.923358	0.004054	3646240
PGA53	2.922257	0.008915	3635599
SEC10	2.918028	9.48E-05	3642961
RPS13	2.914791	1.23E-05	30515328
GPA2	2.914057	0.000131	3636638
C3_07820W_A	2.912589	0.028027	3647463
PHB2	2.912358	0.000103	3635238
C4_06770W_A	2.910597	0.000227	3642920
OCH1	2.909814	0.00066	3641751
RAD7	2.906874	0.00946	3635833
RPN2	2.896304	4.74E-05	3642027
C7_01740C_A	2.893098	0.04693	3637010
C5_00610C_A	2.892812	0.025812	3641585
CR_03130W_A	2.892397	0.028279	3645121
C4_05340W_A	2.891962	0.003994	3646947
RAV2	2.890591	0.024903	3639252
APR1	2.88774	4.65E-05	3645204
TLO34	2.887452	0.003151	3648392
UBA1	2.875064	7.06E-05	3642286
UGA11	2.872357	0.020111	3637437
HFL2	2.870415	0.025966	3637833
ETR1	2.860191	0.000141	3640561
CTA7	2.858754	0.001162	3637776
TRP3	2.857055	0.010478	3642035
LCB2	2.854189	0.000199	3636181
CDC68	2.849793	4.58E-05	3642787
OSM2	2.847362	0.010897	3636193
EMP70	2.846581	3.05E-05	3637919
C3_03210W_A	2.846153	0.028931	3638315
C5_01350W_A	2.842588	0.000519	3642640
COX7	2.838228	0.000945	3638339
C1_08900W_A	2.832757	0.01064	3634925
C2_07220W_A	2.829129	6.67E-05	3643483
MDM10	2.827889	0.021296	3647778
IRR1	2.826196	0.000806	3643305
RPL3	2.823648	4.54E-07	3647719
TAF14	2.823018	7.31E-05	3637357
CDC55	2.817762	8.33E-05	3635785
C3_02720W_A	2.810367	0.001661	3638410

FAT1	2.809509	0.000816	3647083
NHX1	2.8091	0.000134	3647141
SRV2	2.808149	4.19E-06	3640938
PHB1	2.808135	0.001124	3645641
URA6	2.808053	3.28E-05	3647988
C1_08050W_A	2.800824	0.000712	3640709
RBP1	2.79984	8.8E-05	3647647
C1_11600W_A	2.798256	0.012585	3645346
RPL37B	2.797341	2.41E-05	30515053
C1_02830W_A	2.795021	0.009841	3636876
C6_03620C_A	2.788125	0.003253	3640297
CR_00350W_A	2.785253	4.23E-05	3639686
PMR1	2.785142	0.000168	3638043
CR_04330W_A	2.784471	0.022664	3640997
C3_07140C_A	2.782516	0.037978	3638439
CR_05840W_A	2.778412	0.024556	3646704
C1_12660W_A	2.774241	0.012273	3646835
C7_02670W_A	2.771837	0.029888	3647651
DNM1	2.769578	1.97E-05	3643673
RPL6	2.76723	7.87E-05	30514975
CR_04120C_A	2.757449	0.002994	3640972
C1_01300W_A	2.753053	9.04E-05	3640185
RPS30	2.750157	0.001134	30515390
CYC1	2.749126	0.000631	3643937
CR_06790C_A	2.744873	0.015213	3647311
VPS11	2.744845	0.000299	3646524
C2_00320W_A	2.744154	0.003987	3638798
DPS1-1	2.738976	1.33E-05	3645134
PEX1	2.738971	0.001693	3636943
SLC1	2.738358	0.003333	3638324
VPS35	2.733799	4.94E-05	3645567
C1_02040C_A	2.733635	3.24E-05	3644586
TEF1	2.732296	2.63E-07	3648254
C1_09430W_A	2.730107	0.000159	3634855
YRB1	2.72904	0.000237	3639663
MUQ1	2.723162	0.000835	3638898
TOM70	2.715732	3.55E-05	3647645
PRO3	2.714781	0.000299	3645036
GDH3	2.713365	0.000102	3648086
C4_04720W_A	2.70802	0.004569	3641137
C6_03040C_A	2.707626	0.016587	3639269
C2_07100W_A	2.705711	0.000665	3643504
PEP1	2.704654	8.98E-05	3647612
USO1	2.699866	5.43E-05	3648280
HAT2	2.698204	0.001502	3645973
C1_05900W_A	2.697049	0.000175	3639866
HOG1	2.688325	7.47E-05	3637393

RPL5	2.685725	8.62E-06	3637057
VPH1	2.683229	1.28E-05	3646157
SPE1	2.682983	0.000377	3639327
C2_07440C_A	2.682978	0.011645	3645200
C7_04280C_A	2.680419	0.001928	3643173
C7_02470C_A	2.675936	0.011585	3636945
C3_01900C_A	2.674962	0.033376	3636559
ERG20	2.67487	8.68E-05	3645440
C1_11730W_A	2.674446	0.000488	3645318
ANP1	2.673574	0.006094	3643917
NCP1	2.671406	9.55E-06	3637839
EFT2	2.670722	1.37E-06	3642998
C1_02000W_A	2.670244	0.000926	3645418
RPS6A	2.670197	0.000108	3635701
C1_14050C_A	2.670171	0.001729	3643343
C4_00780C_A	2.669098	0.000387	3645759
GLT1	2.664095	2.86E-05	3639519
CR_02680W_A	2.66319	0.044508	3642573
C2_01340W_A	2.659629	0.000113	3638844
SMC4	2.657372	0.000415	3641578
CSP2	2.653075	0.02014	3645749
HEM2	2.653045	0.000724	3637428
SRO77	2.652884	0.000235	3646088
C1_05720W_A	2.649505	3.75E-05	30515000
C1_02510W_A	2.64623	0.04857	3636796
C4_06740C_A	2.64543	3.99E-05	3642923
YST1	2.642216	7.97E-05	3643678
C3_03560W_A	2.640329	0.032115	3638299
C3_00050C_A	2.63561	0.018317	3640570
TIP20	2.627824	0.00048	3636457
SAC7	2.626749	0.000208	3638010
C2_00830C_A	2.626431	0.001726	3638808
ADH1	2.624893	5.39E-06	3636489
C6_02630C_A	2.624045	0.000993	3639221
C4_05850C_A	2.624029	0.034123	3641791
CR_03460W_A	2.612259	0.01583	3643213
C5_00280C_A	2.609951	0.014293	3641581
C2_01170C_A	2.609416	0.000515	3638860
C1_10960W_A	2.609307	0.044415	30515047
C6_01370W_A	2.609205	0.014251	3641607
CR_08620C_A	2.60758	0.01821	3642110
CR_07840C_A	2.600881	0.005921	3647346
C2_04180C_A	2.598086	0.016583	3637330
C3_04360W_A	2.596074	0.001614	3635276
TOM22	2.590949	0.029166	3647650
SCS7	2.579998	4.03E-05	3641157
SFL2	2.577069	0.001397	3636434

C2_05220C_A	2.572491	0.000865	3643466
C1_12170C_A	2.571501	0.009278	3642039
C2_05590C_A	2.57077	0.016316	3645564
C1_11920W_A	2.566909	0.006677	3642003
ERG25	2.565365	0.000344	3644933
LPT1	2.564791	0.000389	3645212
MED1	2.564175	0.015345	3637418
NUO2	2.563732	9.03E-05	3638381
GLR1	2.561933	7.98E-05	3647061
C2_02930C_A	2.561117	0.000435	3643015
C1_01910W_A	2.559948	0.001248	3645425
RPL25	2.559255	0.001503	30515338
C1_06390W_A	2.559192	0.007908	3639558
C5_03170C_A	2.552954	0.000731	3637801
C2_05570C_A	2.551334	0.005247	3645562
VTC3	2.549657	0.000593	3643206
MCM3	2.543419	0.002108	3645223
CR_07780W_A	2.542792	0.002342	3647352
PEX22	2.539973	0.017901	3648103
LYS21	2.531917	0.000728	3644763
SUA72	2.526073	0.046853	3648239
C1_05390C_A	2.525371	0.00225	3642479
C3_06710W_A	2.524599	0.000134	3638448
ATC1	2.518503	0.000405	3639570
C1_10410W_A	2.517322	0.047045	3634840
CHS7	2.516274	0.004137	3639869
PTC7	2.511841	0.001247	3645000
C7_00140C_A	2.506561	0.00807	3637974
PIN3	2.505557	0.000956	3635228
ACP1	2.504566	0.001703	3639825
C2_02870W_A	2.502333	0.018755	3642972
C5_00580W_A	2.499878	0.002591	3641588
C5_03140C_A	2.498709	0.000398	3637798
C2_04370W_A	2.496773	0.003714	3645460
RPL2	2.495987	3.99E-05	30515049
CR_10510W_A	2.495146	0.003628	3639039
DSE1	2.494375	0.019992	3643899
C2_08160C_A	2.493832	0.002623	30515152
CHC1	2.49139	0.000201	3647552
ILV1	2.489388	0.001137	3646918
C1_01400C_A	2.486957	6.54E-05	3640237
C1_11140W_A	2.485571	0.003858	3644410
ACH1	2.485312	4.01E-05	3643773
POB3	2.484864	0.00042	3635827
C3_05010C_A	2.476196	0.038558	3635169
MSK1	2.469393	0.036482	3637032
RPT4	2.467398	7.83E-05	3640966

C2_09930W_A	2.466473	0.010157	3643824
PRO2	2.465428	0.004696	3638532
C2_08270C_A	2.463594	0.005256	3642347
RIM20	2.462843	0.000268	3634809
NIT3	2.462803	0.022502	3644408
RPD31	2.462458	0.000771	3638520
C1_12920C_A	2.458148	0.01943	3636155
YKT6	2.444545	0.001581	3636863
C5_02230W_A	2.443795	0.000498	3637708
CR_06620W_A	2.443206	0.010172	3646631
CSH3	2.442569	0.000222	3646798
CR_02410W_A	2.441737	0.03166	3644928
CR_07600W_A	2.440148	0.020838	3644849
YHM2	2.437793	0.003629	3647137
C7_01230C_A	2.437581	0.000587	3645906
HSP12	2.43094	0.000912	3643745
RPP2B	2.429587	0.000168	3635195
C7_03840W_A	2.428736	0.027511	3643146
UBP13	2.426522	0.000236	3638879
CMK1	2.426102	0.000272	3635263
C6_04110W_A	2.423632	0.000739	3647834
ECI1	2.422104	0.018819	3642128
INO4	2.419527	0.027364	30515105
RPN1	2.411282	0.000511	3636147
HGT5	2.401246	0.003331	3635201
MED18	2.400733	0.049199	3644103
C7_00790W_A	2.39856	0.003341	3637943
SAM4	2.398363	0.000909	3646506
GLK4	2.396707	0.001337	3646046
SGO1	2.39125	0.008654	3643464
CTF18	2.387685	0.013927	3640431
VPS41	2.384932	0.003325	3634800
C3_07110W_A	2.384887	0.00961	3638442
COQ4	2.381901	0.001677	3636804
C1_07630W_A	2.379148	0.026192	3646660
RPL32	2.378613	0.003896	30515335
PRE10	2.378327	0.000632	3636957
HOL4	2.378048	6.02E-05	3645409
YHB5	2.369613	0.000115	3647343
C5_02110W_A	2.364479	0.001542	3637732
AGE1	2.362393	0.000298	3636913
C7_02170C_A	2.351464	0.005198	3636993
CR_03600C_A	2.34886	0.00343	3643207
C6_00240C_A	2.346139	0.011757	3646062
PUP1	2.343463	0.000137	3638936
C7_02780W_A	2.343254	0.000353	3641361
RDH54	2.342878	0.009406	3641231

C4_01020C_A	2.340679	0.001042	3635606
C6_03880W_A	2.336707	0.003035	3640293
C4_04390W_A	2.334961	0.001583	30515267
C1_05630C_A	2.334283	0.000431	3639840
C1_11850W_A	2.333318	0.000712	3642010
DBP5	2.333087	0.001373	3636564
PTC4	2.332488	0.0015	3646697
HOM2	2.32795	0.000148	3635826
C5_02370C_A	2.324328	0.002188	3637793
CYB2	2.324193	0.034865	3636213
PUP3	2.324126	0.003602	3646718
C1_02240W_A	2.319475	0.000392	3644597
C2_09710C_A	2.315139	0.00156	3643786
MNN15	2.3131	0.024579	3644794
HDA1	2.310977	0.002697	3640154
SPE2	2.307059	0.001889	3644116
C2_10670W_A	2.306931	0.000689	3641251
SER2	2.306184	0.049284	3643006
PEX7	2.304115	0.008142	3644027
PCT1	2.303441	0.000584	3645743
ARC1	2.30298	0.00019	3645108
RLM1	2.30115	0.000288	3635703
C6_02280W_A	2.285278	0.027728	3645872
SWI4	2.284292	0.008701	3645408
C5_02750C_A	2.283327	0.005974	3637740
RPS5	2.276601	0.001567	3637700
ASC1	2.276038	0.000262	3645907
GCV1	2.273756	0.02521	3639172
GCV3	2.272909	0.001542	3636194
HEM15	2.270561	0.001424	3645211
RPS20	2.269391	0.003339	3642071
SEN1	2.267907	0.000601	3635255
C3_05110W_A	2.263926	0.028372	3635158
C2_10630W_A	2.263503	0.016078	3641248
DAO1	2.262264	0.0156	3636885
C6_00850W_A	2.262148	0.000279	3644010
GRP2	2.261674	0.001569	3637744
ARF2	2.261585	0.000485	3635271
THR4	2.257211	0.003701	3637711
C1_11890W_A	2.256902	0.011789	3642006
GRE2	2.256614	0.003946	3648026
ECM4	2.254606	0.000912	3640144
C2_01900C_A	2.247261	0.004663	3635789
MLT1	2.247027	0.000439	3640748
VPS33	2.243813	0.011242	3647477
GLG2	2.242792	0.006154	3642282
C2_04300C_A	2.241037	0.03533	3648276

UGA33	2.237842	0.004084	3641886
C4_03920W_A	2.237674	0.02982	3648314
CSH1	2.237003	0.01489	3646427
YCS4	2.235993	0.002631	3645196
C4_01780C_A	2.232991	0.034721	3635582
ILV2	2.230106	0.001064	3636552
RPL16A	2.22983	0.000985	3639338
RPS21B	2.224497	0.006442	30514961
MIR1	2.222856	0.000496	3634782
ADE17	2.21842	0.000547	3641024
C2_05580W_A	2.218123	0.002263	3645563
C7_02850W_A	2.216972	0.00097	3641399
MID1	2.214746	0.018647	3647441
MNN9	2.210706	0.004565	3641735
C3_01890C_A	2.210364	0.001948	3636561
KEL1	2.209017	0.001064	3639318
DDI1	2.208667	0.001618	3643295
CDC53	2.208175	0.000876	3636603
C1_07050C_A	2.192332	0.016643	3639532
PEP3	2.190301	0.030587	3639267
C2_07810W_A	2.186693	0.017027	3642324
C2_03900C_A	2.184338	0.000975	3637395
C1_14200W_A	2.183345	0.005967	3643301
CR_01820W_A	2.183318	0.009224	3640164
ADE2	2.180647	0.001993	3635259
C2_01390W_A	2.173298	0.000943	3635726
PGI1	2.16396	0.000253	3644847
C1_14500C_A	2.163607	0.002431	3643331
C3_01280W_A	2.162156	0.031353	3636623
SOD2	2.15952	0.013569	3640231
FGR24	2.158232	0.015989	3643288
C4_03200C_A	2.157106	0.001255	3637837
RPL4B	2.154285	0.000201	3643347
C2_00510W_A	2.153732	0.019495	3638868
C5_02500C_A	2.149203	0.007391	3637766
C7_00250C_A	2.145986	0.008064	3637979
C1_14040W_A	2.134937	0.009529	3643342
C1_12830C_A	2.134601	0.004412	3636176
GBP2	2.134057	0.000742	3637992
RPS4A	2.133759	0.002325	3641247
C3_07740W_A	2.133236	0.000535	3638453
SES1	2.128659	0.000715	3638415
CR_00470W_A	2.126784	0.002194	3639725
C3_05150W_A	2.122899	0.004512	3635162
CHS5	2.122824	0.001524	3637333
SMP2	2.122237	0.002204	3635779
CR_09510C_A	2.114186	0.02503	3647105

NPL3	2.112673	0.002527	3643310
VPS52	2.112258	0.040983	3638996
RPS18	2.10663	0.01392	3637953
RPL15A	2.104016	0.001179	3641025
CR_01380W_A	2.103317	0.02693	3640131
YPD1	2.103105	0.025762	3644458
CR_10500C_A	2.101881	0.025328	3638982
CR_03860C_A	2.100401	0.01296	3643186
HSP90	2.099427	0.00602	3636951
GPH1	2.096849	0.000156	3637956
COX8	2.096659	0.002678	30515130
PGM2	2.090814	0.000717	3642562
C1_07690C_A	2.089107	0.007608	3641767
C2_06320W_A	2.088	0.001134	3646927
SCT2	2.085331	0.005865	3643018
C7_03370C_A	2.079434	0.005493	3646720
C3_01800C_A	2.078923	0.006052	3636586
GDI1	2.077942	0.004185	3643298
PKH2	2.073131	0.002521	3642058
CST20	2.069377	0.012516	3637790
DNA2	2.066355	0.049789	3646072
C3_02160C_A	2.06622	0.00317	3636654
MDG1	2.065716	0.001286	3643311
C1_06970C_A	2.065692	0.001105	3639567
KIN2	2.0656	0.001612	3639697
VTI1	2.060041	0.008709	3638352
C4_04760C_A	2.059297	0.008168	3641118
AKR1	2.058658	0.005363	3636166
C3_03760W_A	2.05752	0.020077	3645638
UTR2	2.057026	0.001635	3636591
VPS16	2.053627	0.008484	3646150
HHT1	2.053466	0.000866	3638444
UBP6	2.051364	0.001286	3639317
CDC14	2.050233	0.004303	3647132
C1_08490W_A	2.049351	0.013198	3646492
C5_03490C_A	2.048484	0.003001	3646967
TIF	2.044246	0.002497	3640190
C1_00310W_A	2.04236	0.004939	3639298
RPL14	2.041693	0.005521	30515072
C7_02560W_A	2.03522	0.01636	3637016
C6_03470W_A	2.033594	0.024092	3640253
C5_01540W_A	2.032604	0.007835	30515301
PGA7	2.031765	0.002539	3645006
CTA1	2.030984	0.023684	3641631
ARO8	2.030285	0.0032	3638828
C2_00690W_A	2.028954	0.039822	3638885
RPN3	2.028714	0.001057	3636917

KRS1	2.027438	0.001617	3638486
C4_06690C_A	2.017048	0.008679	30515285
VPS4	2.015694	0.013975	3637703
IFR2	2.015642	0.005213	3645100
C3_06970W_A	2.01539	0.04987	3638506
C1_03790C_A	2.015067	0.006434	3644682
MAM33	2.011465	0.030572	3643103
LRG1	2.010774	0.003022	3639727
GUS1	2.010041	0.005733	3638029
YPT1	2.006835	0.006198	3636915
CUP1	2.00474	0.019383	30515318
SBP1	2.004631	0.004459	3635280
C5_04960W_A	2.001783	0.004215	3636423
CR_08350W_A	2.000029	0.005855	3642082
C1_14190C_A	-2.0006	9.82E-05	3643300
C2_00700W_A	-2.00176	0.001374	3638884
C6_02690C_A	-2.00637	0.012098	3639228
FOX2	-2.00712	0.002267	3648327
C3_07680W_A	-2.00764	8.3E-05	3638459
PEX5	-2.00841	0.004237	3645026
RER2	-2.00861	0.009963	3636399
MUC1	-2.01067	0.002778	3645771
CR_02670C_A	-2.01152	0.001459	3642572
C1_01470W_A	-2.01257	0.002083	3640241
C3_01150C_A	-2.01374	0.0476	3648401
OSM1	-2.01401	0.001152	3645573
C2_06090W_A	-2.01509	0.001164	3644097
C3_06050C_A	-2.01599	0.030246	3641746
C1_00530C_A	-2.01697	0.025985	3639368
ADE8	-2.01785	0.003258	3642999
C1_10910C_A	-2.01883	0.006504	3644445
C7_01070C_A	-2.0223	0.008652	3637949
PES1	-2.02335	0.003227	3644105
STP2	-2.02344	0.003834	3636214
C2_07030C_A	-2.02633	0.047503	3643498
C2_04280W_A	-2.02784	0.007935	3637326
CWT1	-2.03065	0.001666	3647595
AFT2	-2.03085	0.0107	3643511
C2_00940W_A	-2.03712	0.001305	3638891
EXM2	-2.0377	0.013336	3642929
ARG81	-2.04025	0.001116	3634775
C6_04550C_A	-2.04144	0.000975	3645976
NPR1	-2.04356	0.001752	3639498
C2_03410W_A	-2.04734	5.38E-05	3637412
DCP2	-2.04857	0.000569	3642069
WSC2	-2.0493	0.001128	3639225
ABD1	-2.0509	0.000787	3638456

IFE2	-2.05128	0.000424	3648027
CR_07140C_A	-2.05134	0.039256	3648227
C1_06470W_A	-2.05551	0.007697	30515007
C2_03830W_A	-2.05828	0.013528	3637399
CR_07830C_A	-2.05893	0.000739	3647345
C6_04140C_A	-2.06046	0.001501	3647970
ARX1	-2.06077	6.55E-05	3636811
CCT8	-2.06321	0.000863	3639324
C4_01710C_A	-2.06324	0.002413	3635708
PIF1	-2.069	0.006748	3646054
SPT14	-2.07245	0.001691	3641019
ESA1	-2.07274	4.87E-05	3640518
C5_01140C_A	-2.07426	0.004835	3642663
IMH3	-2.07546	0.001493	3646556
STP1	-2.077	0.031624	3635152
C2_01680C_A	-2.07743	0.00057	3635744
C5_00260W_A	-2.07985	6.8E-05	3641561
MRPS9	-2.08157	0.000601	3642064
C1_10230C_A	-2.08398	0.000261	3634880
C2_10600W_A	-2.08499	0.007733	3641245
CR_03070W_A	-2.08585	0.001315	3645105
C1_06530C_A	-2.08677	0.000335	3639521
C2_03150C_A	-2.08741	0.024398	3642990
KCH1	-2.09038	0.048327	3636988
RHR2	-2.09192	0.003438	3640522
CCC2	-2.09735	0.000667	3637821
C2_01070W_A	-2.09781	0.005697	3638834
MAS2	-2.09877	0.013214	3647249
C1_03050W_A	-2.10448	0.000796	3636843
C2_05380W_A	-2.10643	0.012084	3643432
C1_09850C_A	-2.10793	0.001865	3634849
BMT5	-2.10794	0.000209	3635781
C1_01820C_A	-2.10838	0.013977	3645435
C2_07760W_A	-2.11295	0.008593	3642298
C4_02700W_A	-2.11319	0.010426	3637859
FEN1	-2.11321	0.00443	3646829
CR_03740C_A	-2.11493	0.021986	3643176
CR_00370W_A	-2.11619	0.000472	3639684
SBA1	-2.11726	0.001795	3640305
SEF1	-2.11993	0.000542	3644945
ECM3	-2.12136	0.01418	3635754
C2_04100W_A	-2.13037	0.025818	3637379
C4_05400C_A	-2.13192	0.006226	3646952
C4_05010W_A	-2.13253	0.015604	3641148
AFL1	-2.13573	9.69E-06	3641594
CR_00630W_A	-2.13692	0.003581	3639690
CR_09460C_A	-2.13978	0.000294	30515453

BAT21	-2.13987	0.011843	3637356
REP1	-2.1429	0.030268	3639708
C1_13560W_A	-2.14312	0.015608	3636174
CR_02910W_A	-2.14599	0.000315	3642586
C3_07940W_A	-2.14635	0.001218	3647453
CR_04580W_A	-2.14756	0.011003	3640948
SLM2	-2.14836	0.001484	3647559
GPM2	-2.1491	0.00029	3644713
C1_06770W_A	-2.15057	0.003025	3639504
PWP1	-2.15086	0.001998	3635623
C7_01400C_A	-2.15096	0.010423	3645893
C6_02430W_A	-2.15201	0.013185	3639163
C6_01420C_A	-2.15211	0.019227	3641602
C2_07060W_A	-2.15376	0.001434	3643500
SPT7	-2.15598	0.000161	3638928
C1_02900C_A	-2.16019	0.008965	3636869
C2_06200C_A	-2.16044	0.012418	3646908
C2_08950W_A	-2.16258	0.000223	3645788
MST1	-2.16386	0.009184	3636831
SUI3	-2.16431	0.001169	3643130
SEC2	-2.16493	0.000204	3636119
STF2	-2.16935	9.41E-05	30515083
C7_01720W_A	-2.16963	0.00497	3637011
FCA1	-2.17284	0.004225	30515327
MBP1	-2.1756	0.000261	3635281
ROT1	-2.17584	0.026327	3639350
C4_03750C_A	-2.18036	0.040586	3645356
MRP7	-2.18076	0.022231	3643118
RLI1	-2.18108	0.0002	3636888
ROD1	-2.18292	0.00039	3635763
C4_01970W_A	-2.18339	0.000916	3635663
PRP39	-2.18351	0.035655	3635832
C5_05130C_A	-2.18388	0.040081	3636459
BOI2	-2.18431	0.000202	3640404
C6_04450W_A	-2.18571	0.01354	3645982
C1_04960C_A	-2.19216	0.001444	3647527
C1_11770C_A	-2.1924	0.00016	3645327
NMD5	-2.19303	0.000365	3645745
C1_09840C_A	-2.19972	4.58E-05	3634848
C5_03040W_A	-2.20713	0.024212	3637824
PPT1	-2.20987	0.008995	3636602
C7_01280C_A	-2.21075	0.007242	3645909
LPI9	-2.21107	0.001333	3637060
SMC6	-2.21136	6.36E-05	3637039
C1_00030C_A	-2.21704	0.015928	3639373
CR_04370W_A	-2.21844	0.011539	3641004
MLP1	-2.22041	0.000365	3635688

ATO6	-2.22336	0.040125	3643664
CR_05440W_A	-2.22486	0.000986	3648245
SOG2	-2.2249	5.88E-05	3644475
C2_06570C_A	-2.22525	0.003197	3646561
KRE30	-2.2304	7.55E-05	3642311
C2_07520C_A	-2.23385	0.006155	3645207
GCD7	-2.2384	0.000481	3637367
C7_04140C_A	-2.24192	0.009412	3643129
C5_05140W_A	-2.24574	0.013358	3636460
MED3	-2.24624	0.005672	3646522
C2_00190C_A	-2.25279	9.2E-06	3638904
C2_10850C_A	-2.25417	0.000105	3641232
MDN1	-2.25494	3.41E-05	3635614
C1_12610W_A	-2.25538	0.006319	3646825
C5_03640W_A	-2.2583	0.002551	3646984
CR_04140W_A	-2.25909	0.00019	3640974
MSW1	-2.25944	0.020875	3637743
C1_12950W_A	-2.26331	0.001806	3636157
C1_04370C_A	-2.26379	0.004077	3647994
VPS24	-2.26379	0.000191	3638892
C4_02690W_A	-2.26899	8.23E-05	3637861
CR_04820W_A	-2.27031	7.16E-05	3647298
GYP5	-2.27177	0.000141	3641246
C1_04120C_A	-2.27358	0.02437	3644701
CR_10040W_A	-2.2792	0.016626	3639034
DUR4	-2.28221	0.045985	3645012
C5_02730C_A	-2.28289	0.012091	3637738
LEU5	-2.28469	7.16E-05	3638838
C6_04290W_A	-2.28879	5.73E-05	3646280
C5_03560W_A	-2.29065	0.00724	3646975
GCA1	-2.29089	0.003454	3634903
ZCF21	-2.29225	6.34E-06	3645762
HGT18	-2.29417	0.011632	3645111
CCW14	-2.29531	0.042273	3642650
C2_00810C_A	-2.29585	0.015227	3638810
OPT7	-2.29595	0.029924	3645008
C2_00170C_A	-2.2989	0.001676	3638906
PAM16	-2.29936	0.001768	3643321
GRX3	-2.30227	3.24E-05	3637858
ASR2	-2.30799	0.002484	3641907
HNM3	-2.31233	0.046693	3640093
C4_01930C_A	-2.3149	0.00773	3635634
C2_02770W_A	-2.31757	5.45E-05	3643031
CR_07160C_A	-2.31999	0.006067	3648347
CR_03870W_A	-2.32177	0.016402	3643187
FGR27	-2.32844	0.000339	3646988
C6_02460C_A	-2.33078	0.005189	3639168

CR_03260W_A	-2.33351	0.000301	30515389
SLN1	-2.33617	1.1E-05	3640422
C1_13430C_A	-2.33876	0.012814	3636223
C3_01130C_A	-2.33882	0.000494	3639901
NMA111	-2.34628	0.000295	3640179
CTN3	-2.3504	0.030132	3647396
PDX3	-2.35078	0.000543	3640949
C2_00220C_A	-2.35132	7.43E-05	3638902
SLR1	-2.35272	4.49E-05	3643957
SHA3	-2.35716	1.08E-05	3644580
CSC25	-2.35837	2.72E-05	3645660
SSK1	-2.36209	4.19E-05	3636184
C5_03150W_A	-2.36214	0.000357	3637799
C4_06410W_A	-2.36408	0.016392	3642796
C4_00080C_A	-2.36618	0.007839	3648065
RIB1	-2.36799	4.33E-05	3642607
ATG15	-2.36983	9.43E-05	3641641
PBP2	-2.37091	2.49E-06	3640267
MRPL3	-2.37205	0.003602	3640722
CTR1	-2.37375	0.002836	3647077
C4_04500C_A	-2.37547	0.014733	3641123
RPC53	-2.37941	0.013043	3637898
C6_03370W_A	-2.38168	7.12E-05	3639230
C6_00980C_A	-2.38233	0.001639	3644046
SDH1	-2.38266	0.00021	3642477
DCK2	-2.38697	0.000948	3637384
GAP1	-2.38777	5.59E-05	3637806
PRS1	-2.39192	0.001617	3635817
HAS1	-2.39752	0.000131	3636443
C3_03330C_A	-2.40029	0.000622	3638364
C4_03780C_A	-2.40062	0.00597	3645353
HAP41	-2.4042	0.002293	3647405
FMT1	-2.40534	0.001362	3646530
CKA1	-2.41022	1.5E-05	3639018
BUD23	-2.4139	0.035234	3642629
SSN6	-2.41862	0.000359	3638518
PRP22	-2.41927	0.001295	3636404
RIM21	-2.42098	1.64E-05	3643739
HAP43	-2.42103	6.83E-06	3644531
C3_01520C_A	-2.424	0.000299	3636570
MRP20	-2.43005	0.00575	3640224
C2_05300C_A	-2.43807	0.001416	3643423
PDR6	-2.43858	0.000148	3638824
CNH1	-2.43992	0.000159	3648056
C3_07900C_A	-2.44611	0.001748	3647450
YML6	-2.44627	0.001799	3637954
C5_05250C_A	-2.44913	0.002199	3636472

RPA135	-2.45013	3.22E-05	3637996
C2_09240C_A	-2.45173	0.008288	3644064
VPS13	-2.45678	9.32E-06	3646528
POX1-3	-2.45691	0.000193	3636598
C2_01820C_A	-2.45746	0.001528	3635831
CR_02590C_A	-2.45748	0.00385	3642595
C7_03680W_A	-2.45923	0.021922	3646228
CR_07690W_A	-2.4654	0.037213	3648194
C2_07410W_A	-2.46559	0.001553	3645203
PHO4	-2.46947	0.00017	3641817
C6_02940C_A	-2.47764	0.002871	3639187
C5_04180W_A	-2.47811	0.005273	3636544
SPL1	-2.48025	6.36E-05	3638035
PRB1	-2.48334	0.000157	3643521
C3_01090W_A	-2.48795	0.007716	30515176
C7_01590C_A	-2.48839	0.025682	3637037
LPG20	-2.49498	0.003008	3644780
C2_05400W_A	-2.49525	0.045099	3643435
C6_00700C_A	-2.50114	0.000452	3647074
C6_01000C_A	-2.50266	0.011834	3644048
YVH1	-2.50918	0.005715	3643218
GAP5	-2.51953	4.09E-07	3646954
C4_00880W_A	-2.52065	0.003467	3635681
CEF1	-2.52174	1.24E-05	3634808
C4_07060W_A	-2.52182	3.8E-06	3642908
PUT2	-2.52724	0.000263	3636439
CR_04170W_A	-2.5277	4.35E-05	3640977
C3_00300W_A	-2.53273	3.55E-05	3640524
CR_09100C_A	-2.5344	0.009527	3641928
C1_09540W_A	-2.53927	0.000561	3634885
YTM1	-2.5469	0.006171	3634765
C1_11200W_A	-2.54818	0.000217	3644396
C1_05200C_A	-2.55119	0.000191	3642466
C3_07920W_A	-2.55423	5.34E-06	30515226
MIT1	-2.55658	3.01E-05	3644089
C6_02150C_A	-2.55794	0.00729	3645888
C4_00610W_A	-2.56808	0.000177	3645770
C4_02880C_A	-2.56814	0.007687	3637907
C4_05740C_A	-2.57721	2.85E-07	3641806
BGL22	-2.57908	0.001818	3641935
CWC22	-2.57973	0.013138	3643938
C3_02900W_A	-2.58114	0.001476	3638337
DAL81	-2.58307	0.00086	3640419
RMT2	-2.5848	0.00212	3641569
HEM1	-2.58731	8.1E-07	3640149
SSU72	-2.59472	0.000376	3645129
ALK2	-2.60234	0.015307	3639700

C5_00030W_A	-2.60423	0.00018	3647879
C1_09130W_A	-2.60891	6.99E-05	3634943
MNN22	-2.61196	0.00014	3641117
CR_08400C_A	-2.61531	0.000565	3642115
RAT1	-2.61768	1.28E-06	3635686
ERO1	-2.62098	7.37E-07	3634810
CDR11	-2.6214	5.18E-05	3647389
SIT1	-2.62747	4.84E-05	3642307
C2_10050W_A	-2.62777	4.95E-06	3643949
C3_04940W_A	-2.62984	4.87E-06	3635270
DFG16	-2.63019	9.59E-07	3637413
C1_06510C_A	-2.63463	1.32E-05	3639549
C4_00060W_A	-2.63847	0.000481	3648061
C2_00540W_A	-2.63994	2.27E-08	3638865
ECM17	-2.64122	6.69E-09	3644077
C3_07590W_A	-2.64174	0.025622	3638496
NAG6	-2.64279	0.000119	3645961
CR_00820C_A	-2.64782	0.001242	3640413
UGT51C1	-2.6519	4.2E-06	3640147
C6_00550W_A	-2.65893	2.64E-05	3647144
C4_03580W_A	-2.66111	0.010453	3645369
PGA62	-2.66113	4.61E-06	3637879
C4_02110W_A	-2.66172	0.001378	3635673
C1_09610W_A	-2.66292	0.009371	3634891
C3_00420W_A	-2.6656	0.000682	3640553
C3_03310C_A	-2.66658	0.021592	3638363
C4_07100C_A	-2.66686	1.92E-06	3642903
CR_10750C_A	-2.67117	0.000739	3638942
C3_05380W_A	-2.67211	0.012597	3643679
C3_07390C_A	-2.67578	0.002101	3638472
CR_07910C_A	-2.67968	9.01E-09	3644142
REG1	-2.68863	9.73E-06	3638858
C6_02380W_A	-2.68914	7.45E-06	3645883
C4_06420W_A	-2.69157	9.22E-06	3642795
PEA2	-2.6981	0.001899	3647673
C5_04860C_A	-2.69953	0.039903	3636437
C3_03920W_A	-2.70214	0.001729	30515197
ARO10	-2.70372	0.000893	3647320
CR_00660W_A	-2.71063	0.035857	3639658
CR_01570W_A	-2.71267	0.04185	3640077
CR_06060W_A	-2.71576	0.000489	6335358
WOR4	-2.71578	4.69E-06	3638454
NUP49	-2.72013	6.54E-06	3636173
C4_03410W_A	-2.72071	0.000209	3646807
C3_07430W_A	-2.72163	1.02E-06	3638484
C3_04370C_A	-2.72269	0.000457	3635172
TOK1	-2.72855	7.54E-06	3645754

C1_05540C_A	-2.73073	2.16E-06	3642503
FOX3	-2.73144	0.001454	3636629
TOR1	-2.73604	8.94E-07	3643480
GIR2	-2.73874	0.000605	3636962
MNL1	-2.7392	9.33E-06	3646050
CIS2	-2.74035	0.001176	3639366
MLS1	-2.74317	0.000343	3634875
EAF3	-2.74859	0.002409	3648318
C3_07660W_A	-2.74912	0.000565	3638462
C1_08770W_A	-2.75031	0.000429	3634788
SKN2	-2.75512	0.000838	3638309
CR_05150W_A	-2.76536	0.0375	30515403
C4_07130W_A	-2.76632	5.8E-06	3642938
SAM35	-2.76661	0.000612	3643329
BRF1	-2.7674	7.65E-07	3646689
CFL1	-2.76958	0.019449	3641810
C1_00540C_A	-2.77144	2.69E-05	3639367
CRP1	-2.77342	5.02E-06	3634826
C1_10710C_A	-2.77479	0.01556	3644407
SPO72	-2.77501	5.79E-08	3644072
C2_00620C_A	-2.77747	0.013354	3638849
C3_03740W_A	-2.77851	0.018526	3645640
DRE2	-2.77909	8.09E-10	3642571
PUS7	-2.78042	5.21E-05	3643960
CR_00750C_A	-2.78285	2.86E-05	3640438
TPT1	-2.78836	0.000671	3640543
LYS144	-2.79085	0.000524	3640512
FTH2	-2.79748	0.000439	3640401
PWP2	-2.80511	6.35E-07	3640416
GPT1	-2.80538	0.023571	3647671
PGA41	-2.8078	0.028407	3642800
C1_11620W_A	-2.80841	0.017507	3645344
CR_02460W_A	-2.80904	8.48E-06	3644922
C3_06420C_A	-2.80956	1.18E-05	3642262
ALP1	-2.80977	1.81E-06	3644437
C3_04540C_A	-2.81012	3.55E-05	3635262
C1_02960C_A	-2.81486	1.74E-07	3636834
C1_05210C_A	-2.81496	0.000172	3642465
FCY21	-2.81627	1.91E-07	3643822
C4_04510W_A	-2.81839	1.41E-06	3641120
C4_01240C_A	-2.81871	1.98E-06	3635705
C2_03500W_A	-2.82177	1.51E-06	3637345
BFA1	-2.82321	0.000206	3639301
GCV2	-2.82703	2.13E-06	3646505
CR_04160C_A	-2.82723	0.003193	3640976
TIM17	-2.8312	0.000747	3647790
CR_08040W_A	-2.83141	8.98E-08	3644135

SFT1	-2.83209	0.002423	30515372
ZPR1	-2.8399	9.29E-07	3640177
C2_01660C_A	-2.84086	0.000202	3635742
HBR1	-2.84533	7.5E-05	3639295
OPT1	-2.8512	0.009125	3640150
C6_01080C_A	-2.85294	0.038845	3644019
MIF2	-2.85626	2.68E-07	3639220
MSU1	-2.86348	0.006837	3643919
C4_00820W_A	-2.86604	0.001467	3645741
URK1	-2.86728	6.56E-08	3635186
C2_04110W_A	-2.86905	0.00999	3637378
C4_06210C_A	-2.87111	0.000254	3642815
C2_10540W_A	-2.87694	1.9E-06	3641200
OFD1	-2.87997	2.11E-06	3646962
ZCF35	-2.88145	0.001461	3641740
CR_02890C_A	-2.88305	0.000677	3642585
C5_00100C_A	-2.88447	2.95E-07	3647883
C4_04660C_A	-2.8857	1.6E-06	3641150
BUB3	-2.8858	7.44E-06	3646362
DBP2	-2.88968	9.13E-07	3647692
C1_10060C_A	-2.89078	0.002854	3634812
HRR25	-2.8909	1.04E-05	3645878
C1_10080W_A	-2.89457	0.002575	3634814
MAS1	-2.89463	3.81E-07	3636855
C5_04170W_A	-2.89543	6.03E-08	3636543
C3_07100C_A	-2.89926	0.000342	3638443
C3_00450C_A	-2.89944	1.66E-06	3640548
AGC1	-2.89961	5.36E-05	3636219
C6_04400W_A	-2.90033	0.010631	3645949
CR_05640C_A	-2.9084	0.024304	3646690
CPP1	-2.91497	1.04E-06	3634865
C3_01540W_A	-2.91933	0.034784	3636567
TRY2	-2.9214	0.000446	3647670
NOC2	-2.92161	0.00023	3647596
C5_02740W_A	-2.92463	0.00174	3637739
CR_00040C_A	-2.92599	2.34E-07	3639675
CR_09920W_A	-2.93192	2.22E-05	3639028
CR_08190W_A	-2.93565	0.025152	3642084
C2_06440C_A	-2.93579	0.000563	3646565
TIM23	-2.93744	0.000549	3643777
C1_08700W_A	-2.94567	1.38E-06	3634936
HGT8	-2.9483	5.03E-07	3638875
C6_01350W_A	-2.94866	7.06E-05	3641642
CR_09610C_A	-2.95211	0.000144	3647094
C3_02910W_A	-2.96556	0.005375	3638378
VPS21	-2.96846	6.5E-07	3642092
C2_03950W_A	-2.96934	0.000299	3637420

ISU1	-2.97153	1.68E-07	3637007
C1_12570C_A	-2.97211	6.32E-07	3647604
RPA190	-2.9732	2.16E-06	3647677
ZCF18	-2.97548	0.000684	3641635
C1_11580W_A	-2.97549	1.08E-06	3645330
C5_00780C_A	-2.97572	0.000316	3644124
SPT5	-2.98285	1.52E-07	3635847
C3_05850W_A	-2.98318	0.000196	3641758
BAS1	-2.98515	1.04E-07	3641136
C3_00500C_A	-2.9854	7.52E-05	3640515
C2_07920W_A	-2.986	0.005559	3642294
SET6	-2.9881	0.015182	3644590
C3_02980C_A	-2.98836	0.000572	3638386
MRT4	-2.98961	0.001846	3639219
WH11	-2.99005	1.69E-07	30515117
C1_14480W_A	-2.99137	0.000157	3643328
C1_00090W_A	-2.99201	0.005596	3639304
TRM12	-2.99421	0.000678	3641738
C1_06380C_A	-2.99881	0.010453	3639559
C1_03140W_A	-3.00386	1.18E-05	3636929
FGR15	-3.00554	0.000437	3638882
C1_05370C_A	-3.00687	1.14E-06	3642481
C7_01150W_A	-3.01074	0.000219	3645913
CR_03110W_A	-3.01307	0.002639	3645124
C2_09790W_A	-3.01348	0.000115	3643804
C3_06820C_A	-3.01515	3.43E-08	3638544
HST2	-3.01864	0.000832	3640162
YTH1	-3.03011	3.26E-08	3645572
C2_01370C_A	-3.03847	3.14E-07	3635723
CR_10420W_A	-3.04154	1.87E-06	3638974
C2_02300W_A	-3.04259	6.89E-05	3635808
C3_02920W_A	-3.04447	4.58E-08	3638379
PRP45	-3.05319	0.010222	3639166
URA7	-3.0579	8.14E-08	3636510
CR_02950C_A	-3.05805	0.000829	3642590
CKS1	-3.05822	0.000131	3647732
FUR4	-3.06002	0.00163	3638305
C1_07980C_A	-3.06021	1.35E-07	3640696
C3_00340W_A	-3.06384	0.003335	3640544
C4_04460C_A	-3.06401	4.39E-08	3641127
NAG1	-3.06902	3.3E-05	3645966
RIT1	-3.07333	0.001785	3638492
C6_03220W_A	-3.0757	0.002332	3639179
SET2	-3.07723	2.76E-07	3643923
CR_02510W_A	-3.07773	1.26E-05	3647694
AAF1	-3.08028	3.97E-07	3642284
C6_04180W_A	-3.0841	7.1E-07	3648176

CR_02800C_A	-3.08475	6.06E-07	3642559
C7_02460C_A	-3.08924	7.22E-07	3636946
C1_12370W_A	-3.08986	9.89E-05	3642061
C1_14580C_A	-3.09174	5.42E-08	3643290
C3_07490W_A	-3.09197	2.4E-12	3638528
C2_09820W_A	-3.09321	3.45E-06	3643801
C1_03400W_A	-3.09536	5.47E-06	3636904
C1_01890C_A	-3.10138	1.35E-06	3645427
C1_06540C_A	-3.10391	1.09E-05	3639520
CR_04750W_A	-3.10449	0.028501	30515399
GRR1	-3.11468	5.93E-07	3636392
C2_07680W_A	-3.11597	0.000113	3642339
CR_04240C_A	-3.11871	3.45E-07	3640989
RRP42	-3.12849	0.003647	3647852
C7_03000C_A	-3.13437	0.000106	3641383
FET3	-3.13576	0.000503	3647153
CR_03080C_A	-3.13691	0.004002	3645104
HGH1	-3.14018	0.000973	3635647
C2_10170C_A	-3.14859	3.23E-08	3643931
MRPL27	-3.15196	0.000631	3636884
C5_04980W_A	-3.15475	0.004243	3636536
CR_10060W_A	-3.16805	7.68E-07	3639035
ALR1	-3.16813	3.15E-08	3636616
C3_00160C_A	-3.16856	1.06E-07	3640591
ARO80	-3.16927	0.000177	3636808
UTP13	-3.17084	3.11E-07	3637718
C2_03560C_A	-3.1724	0.0002	3637337
C1_12680W_A	-3.17394	1.53E-06	3646833
LIG1	-3.17416	7.09E-09	3636947
C1_02090C_A	-3.17945	0.00095	3644577
PTC5	-3.18205	4.3E-07	3642072
C2_02420C_A	-3.18512	9.79E-06	3635756
TPS2	-3.1885	1.29E-08	3636892
POP2	-3.19517	1.1E-06	3640301
C1_06800W_A	-3.20541	0.000348	3639496
C1_05250W_A	-3.20646	0.030452	3642462
SDS24	-3.20735	2.51E-08	3640756
CR_08330W_A	-3.20883	6.06E-05	3642080
DAL5	-3.21231	0.003874	3638401
C4_03020W_A	-3.21438	3.67E-07	3637934
C4_02170C_A	-3.21529	0.00779	3635667
C1_09650W_A	-3.2169	8.06E-08	3634870
CDR1	-3.21727	6.27E-09	3635237
C1_00520W_A	-3.22065	0.000185	3639310
C4_04990C_A	-3.22311	0.038395	3641166
SAP9	-3.22423	1.26E-07	3645625
C7_01610W_A	-3.22694	0.000319	30515353

CR_10640W_A	-3.22872	0.004786	3639014
CPA1	-3.23082	1.01E-06	3635715
C2_06620W_A	-3.23631	0.005905	3646564
C2_02540W_A	-3.24208	1.72E-07	3635820
C4_06680C_A	-3.24251	6.06E-10	3642950
C2_04770W_A	-3.24935	0.000464	3647783
C2_09360W_A	-3.25066	0.000526	3644109
C3_07280C_A	-3.25459	0.001167	3638431
C3_00940W_A	-3.25488	5.26E-08	3639851
TCO89	-3.27219	3.62E-08	3644788
C5_00820W_A	-3.2744	8.03E-05	3644128
CR_01440C_A	-3.28134	9.32E-07	30515374
UTP21	-3.28257	6.21E-07	3635757
TRK1	-3.29799	2.38E-09	3644153
ARG8	-3.29948	2.1E-06	3641131
C7_02160W_A	-3.30324	2.61E-05	3636994
AGP3	-3.30811	0.004945	3641163
C1_01140C_A	-3.30856	5.44E-06	3640202
C6_00360C_A	-3.31369	0.000636	3646083
C7_02930C_A	-3.32698	2.22E-07	3641390
C2_01740C_A	-3.32885	0.008087	3635733
C2_03570C_A	-3.32946	0.003624	3637336
PSO2	-3.33645	0.002728	3641241
C4_00750C_A	-3.33651	0.005582	3645763
MRPL37	-3.33818	0.002519	3644782
CR_10000C_A	-3.34039	0.003832	3639030
CR_07670W_A	-3.34257	6.91E-05	3647255
PRS5	-3.34868	2.79E-07	3642296
MDJ1	-3.35741	4.47E-05	3646980
CR_05910W_A	-3.35784	4.05E-05	3646680
HAP2	-3.35872	3.1E-09	3641800
CAN2	-3.36534	1.81E-08	3644017
GNP3	-3.37523	0.000119	3639027
C1_11290W_A	-3.3813	0.000303	3644553
C1_09740C_A	-3.38261	0.019705	30515035
MET14	-3.38418	0.015128	3641547
YME1	-3.38429	1.53E-07	3641816
CR_06680C_A	-3.39304	2.43E-06	3646623
C2_05080C_A	-3.39566	0.015389	3643453
C3_03200C_A	-3.3971	0.021453	3638428
CYM1	-3.39761	4.52E-09	3642244
C2_07000W_A	-3.39767	1.53E-05	3643534
PDE1	-3.40364	0.00026	3637713
C1_01630W_A	-3.40579	8.1E-10	3640194
C1_13330C_A	-3.40658	1.16E-07	3636150
C7_01660C_A	-3.40669	5.24E-06	3636983
C3_07700W_A	-3.4121	3.38E-05	3638457

C4_06990W_A	-3.41847	3.64E-06	3642968
CR_03940W_A	-3.42003	3.53E-05	3648378
C5_00160W_A	-3.42485	7.58E-05	3647871
C1_08710W_A	-3.4349	0.000153	3634937
C7_00500W_A	-3.43823	0.046069	3637987
C2_05270W_A	-3.44184	1.75E-08	3643470
C1_10580C_A	-3.45184	0.00266	3647679
CR_10760C_A	-3.45903	0.000336	3638943
PHHB	-3.46382	1.05E-06	3638871
DOT6	-3.46507	1.53E-08	3640078
C7_00160C_A	-3.46883	8.03E-05	3637972
C1_00270W_A	-3.47005	2.6E-07	3639300
C1_04820C_A	-3.47158	0.000283	3644787
DAL1	-3.47391	0.000163	3640564
C1_05360C_A	-3.48183	3.23E-07	3642482
C2_05980C_A	-3.48584	0.001258	3644075
C1_07530W_A	-3.48833	0.005174	30515025
CDC34	-3.4938	1.53E-08	3637028
C4_04820C_A	-3.49961	0.00111	3641165
C7_01030C_A	-3.50416	5.6E-05	3638019
BRE1	-3.50764	5.62E-06	3641568
C1_09390W_A	-3.5204	0.000137	3634851
CR_07680C_A	-3.52221	0.001925	3648193
C1_10120C_A	-3.52267	5.75E-14	3634779
C3_01770C_A	-3.52273	4.04E-05	3636588
C5_01050C_A	-3.52294	0.000238	3642648
RPF2	-3.52374	6.27E-05	3643467
RSN1	-3.5249	5.09E-07	3638308
MEP2	-3.52803	1.32E-07	3645022
DUR32	-3.53787	0.001069	3636142
PGA55	-3.5485	1.17E-06	3645784
C7_00200W_A	-3.54857	6.83E-06	3637968
CR_07320C_A	-3.55062	1.97E-07	3646057
C3_07580W_A	-3.55576	2E-06	30515225
C5_02680W_A	-3.56812	1.44E-10	3637774
CR_06450W_A	-3.57528	0.000473	3645279
CKA2	-3.57905	7.74E-07	3643444
RPF1	-3.58637	1.46E-07	3637890
OLE2	-3.60087	5.87E-10	3643503
CR_03840C_A	-3.60684	0.012504	3643184
C5_03740W_A	-3.60777	4.43E-05	3647237
NBN1	-3.61071	7.2E-08	3637410
C2_06510W_A	-3.6109	1.48E-07	3646570
C6_03290W_A	-3.61939	5.38E-11	3639195
C3_02030W_A	-3.6196	7.68E-06	3636580
C2_02120W_A	-3.61962	6.34E-07	3635746
IFR1	-3.62357	0.003172	3643930

C4_07010C_A	-3.6251	0.003277	3642966
C2_07800W_A	-3.63387	0.004003	3642325
C5_00130C_A	-3.63415	5.33E-09	3647881
DAO2	-3.63914	7.34E-05	3646797
MTG1	-3.64097	0.000113	3645139
C7_03210W_A	-3.64559	0.000912	3641371
HSP78	-3.65101	5.58E-05	3637414
C1_10110W_A	-3.65133	0.013686	3634778
CUP2	-3.65227	9.13E-09	3636189
CR_02930W_A	-3.65285	3.43E-11	3642588
FLU1	-3.65555	2.02E-05	3637048
C2_10740C_A	-3.66019	1.02E-09	3641236
C3_01850W_A	-3.66116	7.69E-05	3636565
CR_04600W_A	-3.66168	3.09E-09	3640950
C4_07170C_A	-3.66538	0.001916	30515289
TRY3	-3.67126	1.1E-09	3642652
C5_02010C_A	-3.68275	1.17E-06	3643772
RHD1	-3.68866	0.003218	3647531
C7_03270W_A	-3.69074	3.4E-09	3641365
C7_02660C_A	-3.70262	0.012346	3647652
CR_03250C_A	-3.70312	3.99E-07	3645102
C1_14350W_A	-3.7087	9.28E-11	3643281
SWE1	-3.71786	2.57E-13	3634866
OYE2	-3.72008	3.35E-07	3641603
RTG3	-3.7219	1.06E-09	3644423
C2_09510C_A	-3.72288	3.27E-09	3643816
CR_07480W_A	-3.72318	4.24E-10	3646047
C5_04050W_A	-3.72738	4.4E-14	3647735
C2_02110C_A	-3.74446	0.000595	3635745
RIO2	-3.76386	6.31E-11	3642066
C6_01340C_A	-3.76543	6.03E-05	30515331
C1_00860W_A	-3.76561	8.81E-06	3639358
C7_01490W_A	-3.77019	1.96E-06	3637051
IFM3	-3.78394	7.21E-05	3642335
CSI2	-3.78631	0.003145	3642016
C2_01510C_A	-3.78734	0.005004	3635801
C2_00200W_A	-3.79168	1.42E-08	3638903
SSY5	-3.79664	1.79E-10	3642140
C6_00640C_A	-3.79783	1.86E-05	3647134
C1_01220C_A	-3.80158	0.000108	3640210
CR_02600W_A	-3.81168	0.036671	3642596
FET31	-3.81805	0.000542	3647151
C7_02550C_A	-3.82104	9.25E-06	3637018
OPY2	-3.82226	1.06E-09	3640765
CR_08500W_A	-3.83206	2.18E-07	3642136
C2_04820W_A	-3.83574	1.65E-06	3647776
EST1	-3.83596	1.91E-11	3636526

CR_10280W_A	-3.84153	0.000179	3638934
COX17	-3.85579	0.000294	30515088
CR_06840W_A	-3.85686	3.28E-10	3647322
C1_11900C_A	-3.85963	6.73E-06	3642005
CR_01370C_A	-3.86115	1.38E-07	3640168
PFK26	-3.86328	9.68E-10	3634912
DAL9	-3.86351	5.62E-05	3645653
CR_00670C_A	-3.86733	0.001411	3639655
MTR2	-3.87548	2.99E-06	30515167
TPK1	-3.87648	2.84E-12	3634879
C2_07040W_A	-3.87754	8.87E-06	3643499
SHY1	-3.87893	9.71E-09	3634824
FGR23	-3.88273	6.25E-09	3636555
FZO1	-3.88937	2.47E-09	3646848
PTR3	-3.88967	5.67E-05	3645428
C4_03710C_A	-3.89055	0.002346	3645360
RAD3	-3.89511	1.78E-10	3638014
CNT	-3.90665	3.92E-10	3644071
MNN4-4	-3.91297	0.000127	3639250
SLU7	-3.91555	0.000163	3638501
C1_06840C_A	-3.91775	3.03E-10	30515012
C4_00100C_A	-3.91897	1.15E-12	3645004
NOT5	-3.92009	1.92E-11	3640763
C2_03550C_A	-3.92176	8.09E-10	3637338
C6_03460W_A	-3.92225	4.13E-13	3640252
C3_00150W_A	-3.92976	1.25E-06	3640595
C4_05330C_A	-3.9319	4E-05	3646959
RRP6	-3.93569	1.78E-08	3647535
C2_08850C_A	-3.93847	2.43E-10	3643890
PRN1	-3.95014	7.92E-05	3639882
C1_01580W_A	-3.95144	3.8E-06	3640222
C3_02420C_A	-3.95825	2.65E-05	3638342
NEP1	-3.96498	6.07E-07	3644545
HOS3	-3.9755	1.04E-11	3637886
C3_02180C_A	-3.9802	6.04E-05	3636643
TPO4	-3.98167	2.38E-05	3640956
C3_05250C_A	-3.98372	2.75E-08	3635199
NOP15	-3.9916	7.59E-05	3637966
C3_00620C_A	-3.99372	4.36E-08	30515171
MSN4	-3.9978	7.79E-11	3634911
LEU3	-4.00099	1.43E-10	3637757
SNT1	-4.01767	6.33E-10	3642033
CR_03160W_A	-4.02485	2.31E-05	3645117
C5_00800C_A	-4.02957	1.73E-09	3644126
DEF1	-4.03037	1.73E-12	3639023
CTR2	-4.03561	3.97E-14	3648427
TOP1	-4.04305	6.53E-10	3644034

ISA2	-4.04645	3.65E-10	3638513
C1_11880W_A	-4.05087	1.01E-05	3642007
MSS51	-4.0519	1.5E-10	3639031
C4_06850C_A	-4.06844	4.15E-09	3642912
CR_09740W_A	-4.07386	2.1E-08	3639008
C2_00280C_A	-4.07504	7.9E-06	3638802
CR_03910C_A	-4.07667	5.45E-10	3640954
IFH1	-4.08215	2.56E-12	3637770
C3_05140C_A	-4.08246	2.4E-06	3635161
C1_09780C_A	-4.0829	2.07E-12	3634842
C5_01900C_A	-4.0832	0.01786	30515302
C7_02920W_A	-4.08465	0.019899	3641391
CEM1	-4.09753	0.000422	3635242
C2_01240C_A	-4.10523	0.000233	3638794
KNS1	-4.10588	4.99E-06	3636228
C3_07800C_A	-4.10743	0.000159	3647461
LIP4	-4.11653	1.81E-07	3645987
CR_02970C_A	-4.11662	3.09E-13	3642603
SDH4	-4.11916	0.001411	3636497
C5_01470C_A	-4.14551	0.002536	3647050
C3_03370C_A	-4.14601	2.19E-11	3638366
C7_02110W_A	-4.15083	3.89E-13	3637022
C2_05160C_A	-4.15102	4.54E-08	3643461
C2_02630W_A	-4.15388	1.41E-05	3635839
CR_08410W_A	-4.15723	1.66E-10	3642116
CR_06230W_A	-4.15932	8.97E-06	3644867
NIP7	-4.16091	8.35E-05	3645880
PEL1	-4.16715	5.79E-10	3637989
TBF1	-4.18122	7.22E-13	3637327
C3_03670W_A	-4.1831	3.1E-13	3645649
CCS1	-4.18753	3.67E-13	3644476
C3_07070C_A	-4.18878	7.65E-06	3638446
HSK3	-4.19222	2.7E-07	3635730
C5_03630C_A	-4.19562	0.003107	3646983
CR_08940W_A	-4.19859	1.29E-05	3641914
AUT7	-4.21796	2.11E-12	30514999
C5_03030W_A	-4.22115	5.83E-05	3637822
C6_01780C_A	-4.22551	2.24E-05	3641636
SLD1	-4.22876	5.46E-13	3638406
C5_02020C_A	-4.22956	7.82E-12	3643771
MLH3	-4.23247	1.65E-08	3643294
HAL9	-4.2379	1.75E-12	3643757
C5_03460C_A	-4.23879	7.49E-05	3646345
C2_04080W_A	-4.23993	2.14E-13	3637381
STB3	-4.24264	1E-09	3645781
C6_04300W_A	-4.24534	1.34E-07	3646286
C3_07670W_A	-4.25032	1.98E-10	3638460

C1_10360C_A	-4.25409	7.38E-07	3634836
DUS4	-4.26268	2.39E-07	3641580
C7_02340C_A	-4.27332	0.000303	3636933
RGA2	-4.27369	2.73E-11	3635661
EPL1	-4.27628	1.27E-10	3639732
C5_05240C_A	-4.27865	0.023031	3636471
FGR46	-4.27932	0.02525	3638989
ZCF30	-4.2802	9.01E-11	3642051
PGA48	-4.28268	4.53E-07	3647905
C4_02090C_A	-4.28294	2.58E-07	3635642
NPR2	-4.28592	9.84E-08	3638360
C6_00530C_A	-4.30456	0.000466	30515326
C1_02380C_A	-4.30649	0.007352	3636790
C4_01090C_A	-4.31379	0.002055	3635685
C7_01360C_A	-4.31545	0.020177	3645900
C2_02820C_A	-4.32885	1.7E-07	3643027
C7_03400C_A	-4.33008	9.98E-10	3646717
GAP4	-4.34032	2.61E-09	3644463
FCY24	-4.34062	0.000827	3641902
ISW2	-4.34424	6.74E-06	3642235
C6_01980C_A	-4.34516	5.1E-08	3644560
C5_01610W_A	-4.34907	0.000173	3647251
C3_05160C_A	-4.35043	5.28E-13	3635229
MRV4	-4.35056	0.008222	3636547
C2_00650W_A	-4.3613	1.49E-09	3638889
C7_02120C_A	-4.36482	5.77E-05	3637019
RIM101	-4.36615	6.63E-10	3643280
C3_05860C_A	-4.38909	2.85E-06	3641759
PUT1	-4.40477	1.99E-14	3637779
CR_06970C_A	-4.40788	3.06E-06	3645736
C2_00840W_A	-4.42293	7.08E-05	3638807
PUF3	-4.4299	9.02E-08	3646950
CR_06290C_A	-4.43729	8.6E-09	3644856
RLP24	-4.44472	1.91E-05	3645748
C4_02850W_A	-4.45963	8.66E-12	3637910
POP4	-4.46121	3.34E-06	3639241
C2_08920W_A	-4.46459	1.19E-08	3645797
ZCF29	-4.47939	7.69E-12	3641370
C6_03390W_A	-4.49078	1.66E-06	3639232
C1_11570W_A	-4.49354	0.003832	30515055
C2_06850W_A	-4.49384	0.00531	30515142
C6_02020C_A	-4.52026	1.31E-12	3647563
SPB1	-4.53167	7.46E-09	3648179
RPD3	-4.53784	8.12E-12	3642580
MIS12	-4.54568	3.71E-11	3639669
CR_08650C_A	-4.55491	6.96E-11	3642124
C4_07040W_A	-4.56656	0.000607	3642963

C1_09320C_A	-4.57069	3.06E-10	3634834
C3_03300C_A	-4.58366	3.25E-11	3638361
HIP1	-4.5873	1.65E-13	3643761
C1_13590W_A	-4.59769	0.039408	3636209
C5_00140C_A	-4.59769	0.000145	3647873
C6_02350C_A	-4.60268	2.45E-06	3645881
C1_09040C_A	-4.60322	6.71E-06	3634769
C2_07580W_A	-4.60501	0.000209	3645216
C4_03000C_A	-4.61743	2.89E-10	3637935
FGR13	-4.61815	4.2E-05	3648078
UTP8	-4.62451	1.43E-13	3640547
CR_10400W_A	-4.6275	3.87E-07	3638956
C1_05790W_A	-4.64224	9.92E-05	30515002
C4_07150W_A	-4.65066	0.004336	3642935
C4_03740W_A	-4.65545	3.45E-07	3645357
CR_05550C_A	-4.65561	9.16E-13	3647593
C2_03000C_A	-4.65601	6.5E-07	3643022
C3_06700C_A	-4.65614	1.45E-14	3642247
IMP4	-4.6637	4.23E-07	3644138
C7_03590C_A	-4.67242	7.52E-10	3646239
RIX7	-4.69496	2.72E-10	3637735
CR_00810W_A	-4.70774	2.64E-05	3640415
UTP15	-4.75349	9.62E-08	3643906
C2_02270C_A	-4.758	4.68E-07	3635805
C6_04510C_A	-4.76204	2.7E-08	3645955
DBP3	-4.76735	3.05E-09	3634868
C1_04040C_A	-4.76877	4.29E-09	3646429
SUR2	-4.76905	5.67E-12	3642973
YAH1	-4.77288	3.82E-06	3638368
C1_03830C_A	-4.77559	1.03E-08	3644678
WHI3	-4.79696	7.46E-15	3636991
CR_03710C_A	-4.7973	7.13E-10	3643224
BMS1	-4.80922	4.88E-10	3639857
IML1	-4.82052	1.82E-17	3636813
C7_01170C_A	-4.83502	5.06E-06	3645914
UTP4	-4.84101	4.6E-08	3636657
SSY1	-4.84806	5.69E-12	3637382
C4_05360C_A	-4.87325	0.018881	3646949
C7_03230C_A	-4.87466	0.021373	3641369
C1_12280C_A	-4.87477	0.020068	3642019
MNN4	-4.89016	1.33E-09	3642784
C5_01430C_A	-4.89065	9.62E-08	3647056
ATO10	-4.89697	0.0041	3640394
CR_03200C_A	-4.90243	3.8E-11	3645131
C2_05670C_A	-4.9153	3.7E-05	3645570
C2_08700C_A	-4.91645	2.46E-15	3643887
C7_04160W_A	-4.92383	1.42E-06	3643127

MET3	-4.9268	2.04E-06	3636132
RCL1	-4.92858	9.45E-07	3645199
HPT1	-4.92973	6.38E-11	3643034
OYE23	-4.93145	0.000781	3641618
ATG9	-4.93402	1.16E-10	3635239
PNC1	-4.95073	7.75E-08	3646232
C2_02920W_A	-4.957	2.07E-12	3643016
ZCF28	-4.95853	6.31E-07	3634776
C3_06560W_A	-4.97117	1.52E-15	3642276
DRS1	-4.97382	4.12E-13	3639045
C2_05060C_A	-4.97836	6.28E-05	3643451
C2_02960C_A	-4.9798	1.68E-08	3643011
C5_02980C_A	-4.98388	3.57E-08	3637816
C4_01960C_A	-4.98726	6.85E-14	3635664
C7_03380W_A	-4.989	5.39E-05	30515359
VTC4	-4.98979	6.18E-14	3646795
GYP7	-5.0243	2.65E-13	3646245
PTR22	-5.02645	1.74E-12	3645634
FAL1	-5.03871	3.28E-08	3639839
SOF1	-5.04086	9.95E-12	3640585
PRP8	-5.05627	1.17E-15	3642125
MRPL36	-5.05782	1.17E-08	3643733
CR_08680C_A	-5.07969	5.12E-15	3642127
STE11	-5.08724	2.02E-16	3637369
C5_04080C_A	-5.09859	5.66E-13	3647731
MNN14	-5.11573	1.99E-17	3643651
RPA34	-5.11632	8.85E-06	3634883
C4_05970W_A	-5.11899	1.61E-16	3646514
SPB4	-5.1286	6.41E-08	3647252
PRP5	-5.13199	3.99E-15	3638447
C2_02280W_A	-5.15592	8.55E-12	3635806
C5_03470C_A	-5.16242	7.02E-06	3646963
RFG1	-5.16656	1.08E-14	3642570
C1_07130C_A	-5.17061	4.41E-10	3644462
C1_10140C_A	-5.20076	8.12E-13	3634780
FUN31	-5.20413	7.3E-17	3642249
C2_05830C_A	-5.20975	1.69E-09	3647483
C1_11610C_A	-5.22038	7.74E-09	3645345
C4_03950C_A	-5.24638	0.005258	30515263
C5_05340W_A	-5.25915	9.44E-14	3636400
RRP8	-5.26232	2.57E-07	3643900
C6_03800C_A	-5.27515	3.48E-16	3640310
C1_12750C_A	-5.2769	1.48E-09	3646818
HNM1	-5.27878	3.7E-15	3638857
CR_01630C_A	-5.27933	0.000516	3640085
MUP1	-5.28488	1.59E-13	3642008
C1_09820C_A	-5.28658	0.01624	3634846

C5_02070C_A	-5.29359	4.11E-15	3643746
C1_14380C_A	-5.30267	3.77E-07	3643285
CR_07030C_A	-5.31731	4.93E-06	3645730
SDA1	-5.31883	4.85E-11	3646688
DBP8	-5.32215	3.19E-06	3646692
C3_02020W_A	-5.32878	1.24E-12	3636592
CR_01410C_A	-5.33697	2.4E-08	3640136
UTP5	-5.34291	3.59E-11	3638922
C7_00340C_A	-5.3455	1.46E-16	3638040
FHL1	-5.34571	2.55E-15	3643493
CAG1	-5.34882	3.24E-05	3636469
SFL1	-5.35646	3.21E-17	3642498
C3_03780W_A	-5.39691	0.048523	3645637
C5_04120C_A	-5.40693	3.94E-09	3636522
DHH1	-5.41568	4.71E-08	3639490
C6_04050W_A	-5.41832	1.13E-07	3647830
C5_04910W_A	-5.42009	0.000317	3636417
MOH1	-5.42298	1.17E-11	3646809
C2_02710C_A	-5.43049	1.87E-08	3643003
C1_09520C_A	-5.43369	1.35E-14	3634766
ATS1	-5.43691	2.9E-09	3642079
C4_02260C_A	-5.4526	1.66E-07	3635690
C4_01500W_A	-5.45468	3.11E-09	3635617
SRR1	-5.46118	1.49E-08	3647589
IFF6	-5.46342	2.98E-14	3644087
CR_07940W_A	-5.47876	7.96E-06	3644139
STP4	-5.48493	3.36E-07	3637405
C2_03110W_A	-5.48824	0.001355	3642995
CR_02940C_A	-5.48922	0.027435	3642589
C4_05590W_A	-5.49067	1.86E-16	3641776
JIP5	-5.52505	5.15E-11	3634922
NBP2	-5.52601	2.8E-11	3636963
CR_07750C_A	-5.54584	1.73E-09	3647350
DOT4	-5.54797	3.5E-18	3646810
C2_06480W_A	-5.54956	5.02E-10	3646568
PDR16	-5.55619	0.000199	3644679
DAC1	-5.56366	3.23E-11	3645967
SUV3	-5.57124	6.15E-09	3645451
PNG2	-5.57897	2.62E-12	3645568
YPT53	-5.59668	4.46E-09	3642483
FTH1	-5.60063	4.25E-18	3634852
CR_10410C_A	-5.60554	3.64E-11	3638957
MET10	-5.61783	6.31E-17	3644088
C1_11160C_A	-5.6291	3.22E-05	3644399
RXT3	-5.63121	2.04E-11	3643431
KRR1	-5.63713	5.46E-07	3644542
MAK21	-5.66309	2.73E-20	3635147

C1_11080W_A	-5.66361	7.99E-18	3644416
LEU42	-5.67801	3.18E-13	3643808
DIM1	-5.68994	0.000195	3636135
TYE7	-5.69333	4.61E-16	3636203
HAP42	-5.69555	1.24E-17	3635729
NOG1	-5.69932	7.3E-10	3641736
NSA1	-5.71066	5.24E-08	3642313
C6_00170C_A	-5.71748	1.55E-22	3647903
NAR1	-5.72508	7.95E-14	3634916
C3_00230C_A	-5.73567	0.00069	3640557
C1_05440C_A	-5.74475	8.3E-23	3642489
C6_02290C_A	-5.74952	8.87E-12	3645873
C2_04520C_A	-5.76908	8.12E-05	3645447
C5_04290C_A	-5.77779	1.02E-12	3636455
C5_01020C_A	-5.7878	0.010073	30515297
ISY1	-5.79154	4.38E-07	3646233
C1_04510W_A	-5.79528	8.13E-06	3646147
NOC4	-5.80215	1.41E-10	3645224
MRPL40	-5.80967	1.46E-10	3640968
PIR1	-5.82314	2.51E-09	3645801
C3_01110C_A	-5.85	0.023083	3639835
HRQ2	-5.85601	4.2E-07	3647850
C4_00460C_A	-5.8664	9.22E-19	3645010
CR_06980W_A	-5.86837	0.000249	3645735
PHR3	-5.88236	1.52E-17	3645003
C4_03230C_A	-5.8869	0.003863	3637836
C2_08030W_A	-5.89226	3.26E-09	3642309
C1_09330W_A	-5.90792	8.52E-07	3634802
C5_01560C_A	-5.91702	5.66E-11	3647064
C3_07650C_A	-5.92663	2.93E-06	3638488
PCL5	-5.93352	4.42E-20	3636466
C3_00840C_A	-5.93811	4.8E-05	3647758
CIC1	-5.93966	2.77E-07	3644059
COX19	-5.94204	1.45E-09	3636220
NOP14	-5.95277	9.46E-18	3635266
MPP10	-5.95974	3.55E-21	3647912
SPR28	-5.96176	0.001276	3637716
OYE32	-5.97023	4.7E-09	3642919
C7_01210C_A	-5.98183	7.31E-12	3645904
C7_03880C_A	-6.02003	0.000695	3643142
C1_07960W_A	-6.02447	9.87E-06	3640725
C6_01890C_A	-6.03534	5.35E-10	3641598
C2_06800C_A	-6.03944	2.49E-09	3643518
CR_01640C_A	-6.04056	0.044554	3640086
MET16	-6.0438	1.26E-09	3642964
CR_06330C_A	-6.04626	1.95E-06	3644846
C2_00090W_A	-6.06729	3.34E-06	3647910

GAC1	-6.06957	8.87E-18	3638025
C2_04570W_A	-6.09489	4.15E-08	3645441
SUA71	-6.10355	2.32E-15	3636879
C1_06090C_A	-6.10667	3.66E-22	3639822
BMT9	-6.11158	6.78E-14	3635654
MDM34	-6.12714	1.47E-12	3647517
HSX11	-6.14776	7.36E-05	3635660
C4_06040W_A	-6.15146	2.54E-06	3646521
C2_08860W_A	-6.15194	1.42E-19	3645776
C3_06370C_A	-6.15921	9.3E-19	3642258
C2_06660W_A	-6.167	2.15E-06	3648418
C3_00100W_A	-6.17205	8.05E-10	3640567
C1_07950C_A	-6.1723	6.8E-07	3640724
C1_02850W_A	-6.17945	3.05E-21	3636862
C3_06760W_A	-6.18438	4.34E-07	3638502
CR_00460C_A	-6.20341	5.73E-06	3639726
C2_05640W_A	-6.27906	9.03E-17	3645566
HIT1	-6.28405	1.53E-05	3637854
C1_13380W_A	-6.29573	7.58E-06	3636217
C4_02520C_A	-6.30197	6.85E-13	3637863
C3_05790C_A	-6.3091	2.84E-06	3641753
C3_05440C_A	-6.32282	1.88E-06	3643668
ELP3	-6.32598	1.67E-17	3641747
C4_00490W_A	-6.37549	2.68E-13	3645013
C2_04120C_A	-6.38536	2.77E-10	3637377
C1_13250W_A	-6.3899	1.91E-15	3636143
C2_01540W_A	-6.40045	2.63E-09	3635804
C3_05450C_A	-6.40094	7.59E-18	3643669
C1_13880C_A	-6.40181	1.12E-11	3636133
C5_00570W_A	-6.40339	4.48E-21	3641589
CIRT4B	-6.44934	2.53E-19	3642560
DBP7	-6.47231	1.53E-16	3645903
C6_00380C_A	-6.47333	5.96E-11	3646085
PDE2	-6.50717	1.65E-27	3636877
NRG1	-6.52457	1.48E-13	3643166
C6_01590W_A	-6.52691	2.03E-16	3641650
C4_05160C_A	-6.54075	1.53E-19	3647411
C1_09790C_A	-6.54276	1.01E-18	3634843
MAK5	-6.5514	6.58E-13	3643454
C4_03830W_A	-6.5615	1.03E-06	3647851
CR_03360W_A	-6.59482	8.53E-13	3645141
DAL8	-6.61396	9.97E-07	3635181
C1_08240C_A	-6.6189	1.82E-05	3640759
C3_02750W_A	-6.63836	4.88E-05	3638411
C2_07250C_A	-6.64907	4.92E-05	3643487
BRG1	-6.6529	9E-12	3647664
C4_00260W_A	-6.66502	2.6E-24	3645037

C4_04560C_A	-6.69151	3.52E-20	3641105
C2_08180C_A	-6.69737	2.09E-09	3642317
ABC1	-6.7196	2.74E-19	3640239
C6_02970C_A	-6.72162	2.81E-11	3639190
CR_02550C_A	-6.72923	3.69E-10	3647689
CR_09140C_A	-6.72964	6.44E-14	3641879
C1_06820W_A	-6.72973	1.38E-14	3639493
TCC1	-6.73032	1.49E-21	3638524
MEF2	-6.74017	4.23E-11	3639564
RRS1	-6.7454	4.16E-05	3639352
C2_01050W_A	-6.75809	0.008729	30515086
NMD3	-6.76821	1.99E-17	3646614
C3_02350W_A	-6.773	2.45E-13	3636618
C5_02440C_A	-6.7994	9.16E-09	3637728
PCL1	-6.80014	0.014697	3646356
C3_04870W_A	-6.80211	0.013487	3635226
HNT1	-6.80895	5.2E-23	3644441
C4_05230C_A	-6.83164	1.8E-10	3646156
C6_03210C_A	-6.8443	3.1E-06	3639178
CR_06870C_A	-6.87486	0.026539	3647318
KCS1	-6.87815	4.1E-10	3648120
FLO8	-6.90073	2.88E-23	3646292
SUT1	-6.90245	1.38E-22	3637706
ICL1	-6.93354	8.27E-07	3646146
SEN15	-6.9445	4.32E-06	3646411
QDR1	-6.95497	3.51E-19	3640941
IFM1	-6.9745	2.67E-17	3641389
C5_02280C_A	-6.97641	1.7E-12	3637712
C4_02770C_A	-6.98569	3.19E-09	3637903
HSP70	-7.00371	1.72E-05	3636229
C3_06160C_A	-7.0077	3.96E-09	3641766
PAM18	-7.00845	9.82E-12	3645747
C1_09550W_A	-7.00935	0.047174	3634886
C2_03710W_A	-7.00935	0.047181	3637374
C2_10000C_A	-7.00935	0.047188	30515163
C1_04800C_A	-7.01139	0.044906	3644789
C4_00530C_A	-7.01139	0.044908	3645746
KAR4	-7.04812	2.29E-07	3644937
ATG1	-7.07193	5.87E-25	3641175
C4_00690C_A	-7.08994	5.5E-12	3645752
FAR1	-7.09733	9.91E-11	3637970
BUD21	-7.12508	2.81E-11	3640541
CEK2	-7.12574	2.54E-05	3642459
ECM22	-7.16488	3.91E-15	3648000
C5_00310C_A	-7.18182	1.12E-09	3641576
RPC10	-7.18899	7.33E-10	3643292
C1_12630C_A	-7.21157	3.55E-08	3646838

C5_03410C_A	-7.21395	4.21E-09	3646347
C2_09160W_A	-7.21983	1.1E-08	3644090
C1_10250C_A	-7.24636	3.17E-05	3634882
RMP1	-7.24894	0.000112	3636215
RMS1	-7.26858	8.34E-12	3646361
C2_07020C_A	-7.27013	5.14E-07	3643497
C3_07910W_A	-7.27258	0.036399	3647449
SIP5	-7.28057	6.19E-18	3639865
C1_07660W_A	-7.28328	4.83E-08	3646656
C5_01990W_A	-7.32633	0.001385	3643774
NOP8	-7.32705	2.16E-12	3646290
C3_04620C_A	-7.33371	1.56E-06	3635155
C3_07400W_A	-7.33877	8.24E-13	3638471
TSR1	-7.34574	3.62E-18	3642135
CR_08470W_A	-7.34828	3.08E-19	3642134
C5_01730W_A	-7.35398	4.58E-06	3643730
C2_09570C_A	-7.35627	2.36E-05	3643811
C5_04840C_A	-7.37287	1.22E-11	3636435
SFP1	-7.38984	3.71E-25	3635225
CR_08080W_A	-7.39617	0.000184	3642094
C3_05460W_A	-7.42122	1.23E-09	3643670
WAR1	-7.42997	7.29E-15	3644691
C1_05270C_A	-7.43311	9.45E-12	3642476
C3_01560W_A	-7.44538	7.22E-13	3636669
C3_03460C_A	-7.45519	6.28E-10	3638359
C1_10880W_A	-7.46489	3.79E-13	3644449
C1_09210C_A	-7.47005	6.47E-15	3634897
C5_04530W_A	-7.47641	1.3E-12	3636507
C7_01020C_A	-7.48518	1.45E-10	3638020
C6_01550C_A	-7.53607	5.98E-10	3641653
SGD1	-7.58407	1.87E-20	3643191
OPT8	-7.5846	3.6E-11	3640266
WSC1	-7.59707	2.93E-19	3635138
C3_07570C_A	-7.60024	9.67E-14	3638522
PAN6	-7.60156	2.42E-13	3647684
SSF1	-7.6072	3.23E-12	3647085
IFF8	-7.62503	0.001154	3644117
GCA2	-7.63546	7.64E-16	3647194
C3_02630C_A	-7.69636	3.78E-12	3638328
C1_10970W_A	-7.71291	1.19E-18	3644427
BUD5	-7.72648	8.96E-22	3647315
ARR3	-7.72765	4.08E-09	3642910
RIM2	-7.73057	1.17E-08	3645448
C1_06750W_A	-7.73277	1.07E-05	3639506
SOK1	-7.74729	4.3E-18	3642495
MTG2	-7.75437	2.48E-10	3646693
C1_08630W_A	-7.7555	8.06E-13	3634928

C1_13390W_A	-7.76904	2.48E-08	3636218
C1_10440W_A	-7.77385	5.47E-15	3647180
TPS3	-7.82442	1.91E-21	3641205
C1_01160C_A	-7.87613	4.85E-14	3640204
MET2	-7.90587	2.2E-23	3640070
C6_04240W_A	-7.93921	1.66E-06	3646276
QDR3	-7.9415	2.76E-12	3644023
SAP10	-7.95728	1.58E-12	3641126
C2_07280W_A	-7.96899	0.00026	3643475
CNS1	-8.05404	6.93E-13	3639365
C1_06440C_A	-8.05664	1.85E-11	3639552
BUL4	-8.06183	4.65E-14	3642037
MRF1	-8.06541	4.06E-18	3645321
C1_05930C_A	-8.08452	1.64E-20	3639862
C7_00590W_A	-8.15234	1.08E-12	3637994
C3_07370W_A	-8.17044	1.24E-13	3638474
NCS2	-8.17212	1.19E-20	3643216
NOP4	-8.17219	1.57E-23	3647991
C6_02660C_A	-8.18044	1.59E-06	3639224
C5_02640W_A	-8.20727	9.69E-15	3637785
C3_06940W_A	-8.23399	1.57E-20	3638509
LIP1	-8.26985	4.86E-06	3634888
UTP18	-8.2719	1.05E-15	3643123
TRY6	-8.27499	1.88E-05	3638499
SPO11	-8.29619	0.000102	3643873
SRP40	-8.32803	1.06E-24	3642605
RPO41	-8.33629	1.38E-21	3639378
ARF1	-8.38708	8.71E-28	3642130
RRN3	-8.41885	6.51E-25	3647925
UGA6	-8.42384	2.1E-06	3642975
PET127	-8.46792	7.06E-13	3644417
CR_01710W_A	-8.48227	4.49E-09	3640130
C1_14330W_A	-8.48369	3.9E-15	3643278
NBP35	-8.4919	1.78E-21	3647412
CYC3	-8.58761	1.44E-28	3642621
UTP9	-8.59044	8.05E-16	3638451
C7_02610C_A	-8.62746	3.67E-24	3648285
HCA4	-8.6304	6.77E-18	3637911
NSA2	-8.66086	5.27E-13	3642259
C1_11020W_A	-8.6629	6.11E-09	3644420
C4_06790W_A	-8.6906	1.87E-17	3642918
C2_04090W_A	-8.73538	0.017031	3637380
ENP1	-8.76223	1.17E-08	3647861
C4_06200W_A	-8.83122	3.15E-05	30515282
C1_04720W_A	-8.865	1.4E-09	30514988
C1_06760C_A	-8.87284	4.38E-16	3639505
SOD3	-8.90882	9.03E-12	30515347

ALS6	-8.92157	1.59E-13	3642266
C2_05750W_A	-8.92563	4.42E-10	3645577
C1_03840W_A	-8.93991	0.008325	30514984
C1_02800W_A	-8.9433	0.012114	3636873
C2_10810W_A	-8.94701	5.63E-11	3641243
RRN11	-8.99199	3.44E-16	3645276
KRE6	-8.99989	1.93E-21	3641756
ELF1	-9.01904	5.26E-26	3641903
C1_02670C_A	-9.04228	0.025067	3636821
CTA8	-9.05742	5.08E-24	3634947
TRY4	-9.05997	3.56E-08	3635240
CR_03980W_A	-9.06106	1.71E-14	3640964
CR_04710W_A	-9.10306	4.94E-05	3648141
C3_07290W_A	-9.12202	3.81E-28	3638430
C4_03960W_A	-9.15723	8.32E-12	30515264
C1_09710C_A	-9.16458	7.12E-21	3634818
LAS1	-9.17398	5.02E-16	3644073
NAG3	-9.18454	4.46E-16	3645969
NOP13	-9.2537	1.92E-10	3638469
C1_01750W_A	-9.25589	1.95E-05	3645413
ALS5	-9.27355	3.39E-19	3640277
C6_01040C_A	-9.28787	5.09E-17	3644014
SPO1	-9.2956	0.00084	3647039
C4_03190W_A	-9.34875	6.06E-13	3637838
C7_01690W_A	-9.40791	2.43E-12	3636980
NAN1	-9.41195	9.59E-25	3637932
C4_04810C_A	-9.43111	3.2E-12	3641112
POP3	-9.45682	2.04E-09	3638993
CR_08580C_A	-9.48844	2.18E-10	3642105
ENP2	-9.49871	7.09E-18	3646234
C3_03250W_A	-9.51894	5.14E-32	3638318
GST2	-9.54139	3.45E-21	3637937
CR_02420W_A	-9.60784	1.37E-14	3644926
MRP17	-9.62917	2.56E-16	3641548
RRP15	-9.6321	5.56E-09	3648397
RAS2	-9.66506	1.47E-21	3635211
TSR2	-9.6806	1.16E-14	3636845
NDT80	-9.73351	1.33E-29	3638840
PZF1	-9.74281	4.99E-12	3648113
C3_06950W_A	-9.99767	4.17E-13	3638507
RRP9	-10.0128	4.95E-17	3642576
C1_11530C_A	-10.0264	0.012639	3645334
CR_01250C_A	-10.0264	0.012776	3640403
C7_01880C_A	-10.0287	0.008965	3637031
CR_04250W_A	-10.0302	0.01002	3640990
C7_01940C_A	-10.1414	5.69E-33	3636971
SLY41	-10.2068	1.43E-25	3647139

SEN2	-10.2643	2.18E-12	3637832
CR_08720W_A	-10.3749	1.05E-11	3642122
BUD22	-10.3829	1.54E-15	3640391
C2_09500W_A	-10.4064	2.97E-18	3643817
CR_09800C_A	-10.4139	9.71E-28	3638984
RNR22	-10.459	2.16E-30	3645217
C6_04530C_A	-10.5224	9.94E-28	3645970
C3_02670W_A	-10.6402	1.05E-07	3638405
SUL2	-10.7694	2.19E-07	3637835
C5_03920C_A	-10.7785	1.07E-15	3647448
C2_09660W_A	-10.8531	1.12E-17	3643793
C1_01610C_A	-10.8756	2.74E-11	3640225
CR_09930W_A	-10.8891	4.36E-35	3639029
C2_09610W_A	-10.9045	7.61E-19	3643798
C2_10090C_A	-10.9095	4.58E-17	3643939
KTI12	-10.9751	8.63E-15	3645140
ROA1	-11.0038	9.17E-31	3645424
ZCF25	-11.0086	0.002964	3635694
CR_03660C_A	-11.0335	1.52E-13	3643219
GZF3	-11.0823	9.14E-31	3642563
ALS9	-11.0892	1.55E-17	3640281
C3_05900W_A	-11.1448	1.16E-16	3641739
C3_07550C_A	-11.2106	1.64E-19	3638525
CHR1	-11.2886	7.57E-33	3647601
MTM1	-11.3759	8.38E-18	3642487
C6_02230W_A	-11.4536	2.88E-17	3645866
C5_03080C_A	-11.4558	4.7E-33	3637701
C6_04200C_A	-11.472	3.15E-08	30515344
SNQ2	-11.5092	1.39E-28	3640290
C6_00520W_A	-11.5263	0.000978	3647147
CR_00840C_A	-11.5271	0.001497	3640411
C4_03350C_A	-11.5286	1.39E-11	3646794
C3_01430W_A	-11.6611	1.28E-10	3636633
C6_03200W_A	-11.6883	6.39E-31	3639177
FCR1	-11.7065	4.64E-16	3638542
UGA3	-11.725	3.37E-34	3638926
C2_05810W_A	-11.7602	6.18E-24	3647481
ZCF3	-11.7654	1.21E-36	3645335
C2_04700C_A	-11.8413	2.01E-07	3647794
C1_01620C_A	-12.0019	2.33E-34	3640193
C1_08840W_A	-12.0323	0.000483	3634919
C2_09000C_A	-12.0419	5.67E-05	3645782
MIH1	-12.0508	7.07E-37	3648188
DIP2	-12.0688	3.84E-23	3640762
LTV1	-12.1665	1.06E-17	3639016
C1_02450C_A	-12.1688	1.62E-08	3636849
C7_00330C_A	-12.2366	1.1E-16	3638042

CR_04920W_A	-12.2629	2.64E-22	3648207
CR_10470C_A	-12.3424	1.94E-18	3638979
CLG1	-12.3459	9.83E-32	3646030
ELA1	-12.4408	1.22E-15	3644680
RFX1	-12.4462	2.7E-13	3644844
C2_05840W_A	-12.7285	6.36E-12	3647484
NOG2	-12.7704	5.53E-20	3640299
C1_13130C_A	-12.8227	2.01E-18	3636202
MSS116	-12.8308	2.77E-21	3634792
MFG1	-12.833	8.85E-25	3643884
C4_07020C_A	-12.9637	0.023698	3642965
C3_04450C_A	-12.9637	0.023698	3635207
C1_09300C_A	-12.9637	0.023698	3634831
FRP5	-12.9645	0.028296	3641600
CR_04110W_A	-12.9701	2.44E-24	3641026
C5_03800W_A	-12.9862	6.89E-29	3647230
C7_03550C_A	-13.0448	0.006727	3646235
C1_08830C_A	-13.0469	0.002995	3634918
FGR50	-13.0971	0.000152	3644859
C2_06520C_A	-13.1381	1.17E-10	3646571
C2_00410C_A	-13.169	6.19E-38	3638820
ZCF2	-13.171	2.5E-34	3642484
C4_04200C_A	-13.2356	0.009004	3641182
C1_08610C_A	-13.4165	2.41E-33	3646512
RCH1	-13.4537	1.56E-18	3645002
RPA12	-13.5642	4.9E-08	3643477
FYV5	-13.6438	1.65E-09	3647057
C1_06350W_A	-13.7374	1.16E-32	3639562
C4_05350W_A	-13.7735	5.08E-27	3646948
C1_11000C_A	-13.7871	5.35E-12	3644422
C1_13010W_A	-13.7994	7.97E-21	3636120
GRF10	-13.8328	6.83E-46	3636492
C1_07650W_A	-13.8934	5.63E-15	3646657
C5_03510C_A	-13.9502	6.8E-24	3646969
C1_04710C_A	-14.003	3.12E-13	3644764
RGS2	-14.0933	1.76E-18	3646629
DAL4	-14.1331	0.001444	3638422
XUT1	-14.1741	2.01E-06	3642785
HBR3	-14.2108	6.13E-32	3645652
C3_00410C_A	-14.3965	0.000119	3640552
C7_01910C_A	-14.468	2.26E-21	3637029
C1_10950C_A	-14.5666	9.66E-18	3644428
MAK16	-14.651	1.69E-17	3646920
DED1	-14.6563	3.66E-33	3641752
RNR3	-14.6726	1.95E-19	3647591
C2_09880C_A	-14.6991	1.27E-17	3643779
UTP22	-14.8465	1.68E-34	3635760

C2_05330C_A	-14.9487	1.24E-20	3643426
C3_05800W_A	-15.0911	7.7E-23	3641754
C3_04730C_A	-15.1435	0.000412	3635250
C1_08890C_A	-15.1445	0.000305	3634924
CR_02030C_A	-15.1491	4.08E-24	3640152
CDA2	-15.2642	1.03E-08	3639716
C1_11510C_A	-15.301	4.8E-19	3645336
SCH9	-15.456	6.38E-40	3637421
C4_04300C_A	-15.5036	1.86E-11	3646843
C2_00760C_A	-15.5288	1.19E-33	3638910
C5_01710C_A	-15.5963	8.88E-13	3643732
C1_05330C_A	-15.973	1.08E-24	3642485
C1_01510W_A	-16.0125	3.1E-25	3640229
C7_02600C_A	-16.0481	5.3E-46	3648286
C1_11320C_A	-16.0586	8.81E-30	30515052
PRM9	-16.0706	0.00325	30515173
TAZ1	-16.3708	3.01E-11	3644108
ECM1	-16.4083	5.1E-13	3641224
CYS3	-16.4145	2.08E-44	3642081
PTC8	-16.4436	6.94E-37	3635674
SAS10	-16.4823	4.99E-24	3637899
C2_02190C_A	-16.8422	1.15E-22	3635753
C1_07790C_A	-16.8973	1.02E-16	3640706
HEM3	-16.9632	3.9E-37	3643940
C1_01130W_A	-17.0401	1.09E-29	3640178
C1_12440W_A	-17.2145	0.00019	3642012
HGT17	-17.8155	2.62E-05	3635687
C7_04090C_A	-17.8904	3.16E-33	3643135
ALS7	-17.9089	4.91E-39	3642234
PGA37	-17.9781	3.74E-12	3636484
CUP9	-17.9858	6.07E-36	3636950
C3_05840W_A	-18.5425	0.008007	3641757
C4_07000W_A	-18.5454	0.006643	3642967
RTA3	-18.6041	9.94E-12	3646566
SSU81	-18.9381	1.36E-19	3634944
C7_03460W_A	-19.0852	0.012291	3646712
C2_02580W_A	-19.1069	1.45E-18	3635860
C3_02040C_A	-19.2165	3.22E-15	3636579
HWP2	-19.2961	3.52E-05	3646803
HAK1	-19.4515	4.33E-07	3639546
C5_04850W_A	-19.5016	2.46E-08	3636436
C3_04510W_A	-19.6208	5.28E-13	3635258
C5_02590C_A	-19.6544	3.11E-19	3637723
RSM22	-19.6799	5.21E-24	3642506
ALD6	-19.8827	2.71E-23	3647407
C2_01060C_A	-20.2302	1.99E-11	3638835
GAP2	-20.4688	8.85E-25	3643662

C3_02840W_A	-20.5713	1.26E-08	3638331
C1_11410C_A	-20.7328	2.24E-07	30515054
FGR47	-20.9504	1.49E-49	3641651
REI1	-21.1979	7.16E-24	3647536
RIM8	-21.2271	1.44E-43	3639342
C4_02740W_A	-21.2364	8.93E-44	3637855
C6_00290W_A	-21.8302	9.89E-36	3646069
C1_01150C_A	-22.1556	1.03E-31	3640203
CR_00310C_A	-22.5604	1.24E-38	3639715
C4_06620C_A	-22.6825	5.24E-09	3642813
C4_04520W_A	-22.7517	4.34E-32	3641110
C4_04020C_A	-22.8387	0.000614	3641215
C7_03850W_A	-23.0148	7.97E-17	3643145
C2_06530W_A	-23.0907	1.38E-16	3646572
GCS1	-23.5912	4.11E-42	3640742
NOP6	-23.6567	1.38E-21	3639507
GPD1	-23.9051	2.39E-27	3643924
C7_01680C_A	-24.0227	8.08E-56	3636981
C6_02950C_A	-24.0923	2.6E-12	3639188
MET1	-24.1142	1.17E-52	3643014
C6_03280W_A	-24.1283	0.002299	3639185
CDR4	-24.3183	1.56E-27	3640711
HSP31	-24.8724	2.81E-13	3644589
RPN4	-25.0181	6.06E-45	3644684
C1_02690C_A	-25.1245	0.000205	3636823
COX11	-25.4249	5.78E-49	3646842
HSP21	-25.4615	2.02E-34	3637364
CTA4	-26.1803	6.65E-49	3641743
C4_07260W_A	-26.6538	9.67E-14	3642942
C1_09770W_A	-27.1348	2.38E-23	3634825
C4_07140W_A	-27.2191	6.01E-50	3642936
FRE10	-27.6051	1.26E-23	3646841
CR_06550C_A	-28.1201	1.34E-30	3645260
QDR2	-28.2638	3.77E-55	3643661
C5_03440W_A	-28.3967	1.95E-12	30515310
PRX1	-28.5328	1.86E-51	3641357
PGA57	-28.8166	4.11E-08	3635608
SMM1	-28.8841	2.63E-23	3643778
CR_01600C_A	-29.6009	2.87E-14	3640080
C2_03690C_A	-29.736	2.82E-14	3637376
CAN1	-31.1094	4.69E-56	3644035
KSP1	-31.2592	2.6E-70	3644467
C6_03810W_A	-31.3909	3.28E-40	3640286
C1_13820C_A	-31.4248	7.38E-30	3636127
CR_04220C_A	-33.1619	6.33E-15	3640943
C2_04670W_A	-33.6217	1.87E-18	3647789
FRE9	-33.9704	8.62E-32	3643452

WOR2	-35.2227	7.17E-15	3635230
BMT1	-36.8506	9.91E-53	3638535
AQY1	-37.4069	1.93E-29	3642587
C6_03360C_A	-38.2784	6.37E-10	3639203
C4_00810C_A	-39.8599	2.65E-12	3645742
JEN2	-40.0793	4.52E-17	3641214
GIT3	-40.7891	4.9E-60	3642634
C6_01400W_A	-40.8796	0.000607	3641604
C2_07070W_A	-40.8953	2.96E-28	3643501
C7_00770W_A	-42.1863	2.29E-31	3637959
C6_03180C_A	-42.9771	1.94E-63	3639261
C2_08530C_A	-43.2456	2.3E-11	3643921
CR_01510C_A	-46.4611	0.00029	3640104
C6_03440W_A	-46.5519	2.29E-26	3640274
CR_04940W_A	-47.3973	7.67E-13	3648209
C6_01870C_A	-50.7162	1.52E-22	3641625
CR_08830W_A	-52.163	6.31E-33	30515450
FMP45	-52.5234	3.39E-32	3637004
C7_00880C_A	-60.5808	1.42E-73	3638052
IFA14	-64.8068	1.17E-10	3639012
C2_02130C_A	-67.3784	1.81E-17	3635747
C1_00190C_A	-70.1604	1.89E-43	3639337
MNN1	-74.3158	3.21E-31	3637784
ZCF1	-75.4146	6.4E-113	3638329
C6_00810C_A	-75.642	7.97E-33	3647068
C7_01430C_A	-76.832	7.09E-34	3636961
AAH1	-82.4632	3.34E-09	3643529
C2_08580W_A	-85.5081	1.07E-05	3643916
YWP1	-88.876	1.91E-34	3643915
EBP1	-93.1105	5.51E-19	3644060
SSU1	-98.5082	1.41E-68	3641882
C2_08260W_A	-100.567	7.26E-10	3642345
C4_03340C_A	-106.627	4.59E-10	3648090
AOX2	-125.854	6E-100	3634945
CR_01530C_A	-145.522	1.52E-15	3640072
CR_07540C_A	-159.887	0.036087	3648001
C5_00810C_A	-159.887	0.036087	3644127
FGR42	-159.887	0.036087	3647438
GPI1	-171.471	1.74E-27	3643680
CR_06140W_A	-177.28	4.47E-85	3644862
C5_04030W_A	-188.886	4.03E-42	3647737
PHO87	-194.163	8.03E-13	3639861
C2_09330C_A	-196.885	0.028734	3644106
C3_07930C_A	-246.29	0.02692	3647452
RME1	-269.953	6.63E-59	3644453
CR_03320C_A	-286.063	0.023665	3645096
C1_06430C_A	-307.813	0.018668	3639553

HSP30	-484.077	2.45E-75	3645419
KTI11	-492.736	0.010359	30515125
C2_02640C_A	-529.846	0.009273	30515095
ARG11	-640.77	0.007113	3635193
C4_06910W_A	-746.962	3.77E-12	3642933
C3_06170C_A	-751.873	0.005852	3642272
C7_03560W_A	-854.455	1.08E-71	3646236
C5_04380C_A	-973.677	0.003968	3636485
CFL4	-1343.35	0.002622	3642639
CRZ2	-2122.36	6.83E-61	3645725
CR_08880C_A	-5848.68	0.000273	3641878
C1_14630C_A	-26642	2.12E-05	3643291
TAR1	-48432	0	30515449

B 20 vs Control

Name	Fold change	P-value	GeneID
ASG7	#N/A	#N/A	3639173
ATO7	#N/A	#N/A	3635814
BUD20	#N/A	#N/A	3645800
C1_00020C_A	#N/A	#N/A	3639374
C1_01720C_A	#N/A	#N/A	30514964
C1_01970W_A	#N/A	#N/A	30514967
C1_03580W_A	#N/A	#N/A	30514980
C1_03880C_A	#N/A	#N/A	3646409
C1_04440W_A	#N/A	#N/A	3646138
C1_05550C_A	#N/A	#N/A	30514994
C1_05730W_A	#N/A	#N/A	30515001
C1_06300W_A	#N/A	#N/A	30515004
C1_06310W_A	#N/A	#N/A	30515005
C1_06490C_A	#N/A	#N/A	30515009
C1_06830W_A	#N/A	#N/A	30515011
C1_07320W_A	#N/A	#N/A	30515023
C1_07450W_A	#N/A	#N/A	30515024
C1_07940W_A	#N/A	#N/A	30515027
C1_08960W_A	#N/A	#N/A	30515032
C1_08970W_A	#N/A	#N/A	30515033
C1_09560C_A	#N/A	#N/A	30515034
C1_10050W_A	#N/A	#N/A	3634811
C1_10130W_A	#N/A	#N/A	30515040
C1_10590W_A	#N/A	#N/A	30515042
C1_10980W_A	#N/A	#N/A	3644425
C1_11180C_A	#N/A	#N/A	30515050
C1_11710C_A	#N/A	#N/A	3645320
C1_11780W_A	#N/A	#N/A	30515057
C1_12980W_A	#N/A	#N/A	30515068
C1_12990W_A	#N/A	#N/A	30515069
C1_13000W_A	#N/A	#N/A	30515070

C1_13040C_A	#N/A	#N/A	30515071
C1_13290W_A	#N/A	#N/A	3636146
C1_13500C_A	#N/A	#N/A	30515075
C2_00050C_A	#N/A	#N/A	3647922
C2_00310W_A	#N/A	#N/A	3638800
C2_00560W_A	#N/A	#N/A	3638854
C2_00900W_A	#N/A	#N/A	3638895
C2_02080W_A	#N/A	#N/A	30515093
C2_03580W_A	#N/A	#N/A	30515100
C2_03630W_A	#N/A	#N/A	3637438
C2_04070C_A	#N/A	#N/A	30515110
C2_04610C_A	#N/A	#N/A	30515113
C2_05310C_A	#N/A	#N/A	30515119
C2_05420C_A	#N/A	#N/A	30515121
C2_05600W_A	#N/A	#N/A	30515124
C2_05880W_A	#N/A	#N/A	30515129
C2_05900W_A	#N/A	#N/A	3647475
C2_06400C_A	#N/A	#N/A	30515135
C2_06410W_A	#N/A	#N/A	30515136
C2_06560W_A	#N/A	#N/A	30515139
C2_07710W_A	#N/A	#N/A	3642305
C2_07870C_A	#N/A	#N/A	3642319
C2_08520C_A	#N/A	#N/A	30515155
C2_09110C_A	#N/A	#N/A	3644114
C2_09540W_A	#N/A	#N/A	30515159
C2_09550C_A	#N/A	#N/A	30515160
C2_09800C_A	#N/A	#N/A	3643803
C2_10800W_A	#N/A	#N/A	30515169
C3_00140W_A	#N/A	#N/A	3640596
C3_01050C_A	#N/A	#N/A	3639831
C3_01740C_A	#N/A	#N/A	30515178
C3_02880W_A	#N/A	#N/A	3638335
C3_04150W_A	#N/A	#N/A	30515202
C3_05640W_A	#N/A	#N/A	3643654
C3_05660C_A	#N/A	#N/A	3643656
C3_05820W_A	#N/A	#N/A	30515212
C3_06390W_A	#N/A	#N/A	30515216
C4_00640W_A	#N/A	#N/A	3645767
C4_01570C_A	#N/A	#N/A	30515236
C4_01580C_A	#N/A	#N/A	30515237
C4_01590C_A	#N/A	#N/A	30515238
C4_01600C_A	#N/A	#N/A	30515239
C4_01610C_A	#N/A	#N/A	30515240
C4_01620C_A	#N/A	#N/A	30515241
C4_01630C_A	#N/A	#N/A	30515242
C4_01640C_A	#N/A	#N/A	30515243
C4_02650C_A	#N/A	#N/A	30515249

C4_03480C_A	#N/A	#N/A	3646815
C4_04040W_A	#N/A	#N/A	3641213
C4_04490C_A	#N/A	#N/A	30515269
C4_06180C_A	#N/A	#N/A	30515281
C4_06520W_A	#N/A	#N/A	30515284
C4_06630C_A	#N/A	#N/A	3642812
C4_06640C_A	#N/A	#N/A	3642811
C5_00530W_A	#N/A	#N/A	3641537
C5_01030W_A	#N/A	#N/A	30515298
C5_01150C_A	#N/A	#N/A	30515299
C5_01160C_A	#N/A	#N/A	30515300
C5_02130W_A	#N/A	#N/A	3637733
C5_02850W_A	#N/A	#N/A	30515305
C5_03450W_A	#N/A	#N/A	30515311
C5_03890C_A	#N/A	#N/A	30515312
C5_04450C_A	#N/A	#N/A	30515315
C5_04690C_A	#N/A	#N/A	30515320
C6_00220C_A	#N/A	#N/A	30515324
C6_01540C_A	#N/A	#N/A	30515333
C6_02390W_A	#N/A	#N/A	30515339
C6_02400W_A	#N/A	#N/A	30515340
C6_03570W_A	#N/A	#N/A	3640248
C6_04150W_A	#N/A	#N/A	30515343
C7_00540W_A	#N/A	#N/A	30515348
C7_01000C_A	#N/A	#N/A	3638022
C7_01060W_A	#N/A	#N/A	3637950
C7_01130C_A	#N/A	#N/A	3645912
C7_01260W_A	#N/A	#N/A	30515352
C7_02130W_A	#N/A	#N/A	3636997
C7_02190W_A	#N/A	#N/A	30515356
C7_02360W_A	#N/A	#N/A	3636931
C7_02420C_A	#N/A	#N/A	3636975
C7_04070C_A	#N/A	#N/A	3643153
CR_00450C_A	#N/A	#N/A	30515367
CR_00500C_A	#N/A	#N/A	30515368
CR_00720W_A	#N/A	#N/A	3640441
CR_02320W_A	#N/A	#N/A	30515381
CR_02470W_A	#N/A	#N/A	30515382
CR_02660C_A	#N/A	#N/A	30515383
CR_03190C_A	#N/A	#N/A	30515388
CR_04620C_A	#N/A	#N/A	3640952
CR_05350W_A	#N/A	#N/A	30515405
CR_05370W_A	#N/A	#N/A	30515406
CR_05470C_A	#N/A	#N/A	30515409
CR_06190C_A	#N/A	#N/A	30515418
CR_06200C_A	#N/A	#N/A	30515419
CR_06210C_A	#N/A	#N/A	30515420

CR_06220C_A	#N/A	#N/A	30515421
CR_06580W_A	#N/A	#N/A	3646627
CR_06600C_A	#N/A	#N/A	30515422
CR_06630W_A	#N/A	#N/A	30515423
CR_07300W_A	#N/A	#N/A	3646059
CR_08140W_A	#N/A	#N/A	30515435
CR_10320W_A	#N/A	#N/A	3638938
CR_10460W_A	#N/A	#N/A	30515457
CSA2	#N/A	#N/A	3642930
FGR18	#N/A	#N/A	3644112
GTT1	#N/A	#N/A	3643653
ITS1	#N/A	#N/A	30515447
ITS2	#N/A	#N/A	30515448
MFA1	#N/A	#N/A	30515153
MFALPHA	#N/A	#N/A	3646420
MRV3	#N/A	#N/A	3636546
PGA12	#N/A	#N/A	3638920
PGA19	#N/A	#N/A	3638894
PGA22	#N/A	#N/A	3644939
PGA44	#N/A	#N/A	3636647
PGA50	#N/A	#N/A	3647524
PTR2	#N/A	#N/A	3640089
RBR1	#N/A	#N/A	3640986
RDN18	#N/A	#N/A	3648135
RDN25	#N/A	#N/A	3648137
RDN5	#N/A	#N/A	3648138
RDN58	#N/A	#N/A	3648136
RHD2	#N/A	#N/A	3637891
RPR1	#N/A	#N/A	30515150
SCR1	#N/A	#N/A	30515392
SNR52	#N/A	#N/A	30515222
SNR6	#N/A	#N/A	30515211
SPR3	#N/A	#N/A	3635853
tA(AGC)1	#N/A	#N/A	30515148
tA(AGC)2	#N/A	#N/A	30515199
tA(AGC)3	#N/A	#N/A	30514965
tA(AGC)4	#N/A	#N/A	30514960
tA(AGC)5	#N/A	#N/A	30515321
tA(AGC)7	#N/A	#N/A	30515078
tA(UGC)1	#N/A	#N/A	30515336
tA(UGC)2	#N/A	#N/A	30515342
tC(GCA)1	#N/A	#N/A	30515411
tC(GCA)2	#N/A	#N/A	30515410
tD(GUC)2	#N/A	#N/A	30515261
tD(GUC)3	#N/A	#N/A	30515260
tD(GUC)4	#N/A	#N/A	30515259
tD(GUC)5	#N/A	#N/A	30515114

tD(GUC)6	#N/A	#N/A	30515233
tD(GUC)7	#N/A	#N/A	30515452
tE(CUC)1	#N/A	#N/A	30515101
tE(UUC)1	#N/A	#N/A	30515196
tE(UUC)2	#N/A	#N/A	30515184
tE(UUC)3	#N/A	#N/A	30515295
tE(UUC)4	#N/A	#N/A	30515294
tE(UUC)5	#N/A	#N/A	30515309
tE(UUC)6	#N/A	#N/A	30515198
tE(UUC)7	#N/A	#N/A	30515445
tF(GAA)1	#N/A	#N/A	30515191
tF(GAA)2	#N/A	#N/A	30515103
tF(GAA)3	#N/A	#N/A	30515107
tF(GAA)4	#N/A	#N/A	30515166
tF(GAA)5	#N/A	#N/A	30515224
tG(CCC)1	#N/A	#N/A	30515228
tG(GCC)1	#N/A	#N/A	30515432
tG(GCC)2	#N/A	#N/A	30515258
tG(GCC)3	#N/A	#N/A	30515115
tG(GCC)4	#N/A	#N/A	30515231
tG(GCC)5	#N/A	#N/A	30515230
tG(GCC)6	#N/A	#N/A	30515446
tG(UCC)1	#N/A	#N/A	30515172
tG(UCC)2	#N/A	#N/A	30515229
tH(GUG)1	#N/A	#N/A	30515401
tH(GUG)3	#N/A	#N/A	30515217
tI(AAU)1	#N/A	#N/A	30515138
tI(AAU)2	#N/A	#N/A	30515147
tI(AAU)3	#N/A	#N/A	30515122
tI(AAU)4	#N/A	#N/A	30515272
tI(AAU)5	#N/A	#N/A	30515440
tI(UAU)1	#N/A	#N/A	30515045
tK(CUU)1	#N/A	#N/A	30515193
tK(CUU)2	#N/A	#N/A	30515206
tK(UUU)1	#N/A	#N/A	30515181
tK(UUU)2	#N/A	#N/A	30515428
tK(UUU)3	#N/A	#N/A	30515436
tK(UUU)4	#N/A	#N/A	30515437
tK(UUU)5	#N/A	#N/A	30515438
tL(AAG)1	#N/A	#N/A	30515379
tL(AAG)2	#N/A	#N/A	30515022
tL(CAA)1	#N/A	#N/A	30515091
tL(CAA)2	#N/A	#N/A	30515087
tL(CAA)3	#N/A	#N/A	30515346
tL(CAA)4	#N/A	#N/A	30515444
tL(CAA)5	#N/A	#N/A	30515322
tL(CAA)6	#N/A	#N/A	30515362

tL(UAA)1	#N/A	#N/A	30515089
tL(UAA)2	#N/A	#N/A	30515375
tL(UAA)3	#N/A	#N/A	30514979
tL(UAA)4	#N/A	#N/A	30515017
tL(UAA)5	#N/A	#N/A	30515360
tM(CAU)1	#N/A	#N/A	30515188
tM(CAU)2	#N/A	#N/A	30515145
tM(CAU)3	#N/A	#N/A	30515132
tM(CAU)4	#N/A	#N/A	30515451
tN(GUU)1	#N/A	#N/A	30514997
tN(GUU)2	#N/A	#N/A	30514996
tN(GUU)3	#N/A	#N/A	30514978
tN(GUU)4	#N/A	#N/A	30515455
tP(AGG)1	#N/A	#N/A	30515016
tP(UGG)1	#N/A	#N/A	30514992
tP(UGG)2	#N/A	#N/A	30515157
tP(UGG)3	#N/A	#N/A	30514956
tP(UGG)4	#N/A	#N/A	30515413
tP(UGG)5	#N/A	#N/A	30515412
tQ(CUG)1	#N/A	#N/A	30515296
tQ(UUG)1	#N/A	#N/A	30515257
tQ(UUG)3	#N/A	#N/A	30515118
tQ(UUG)4	#N/A	#N/A	30515271
tQ(UUG)5	#N/A	#N/A	30515270
tR(ACG)1	#N/A	#N/A	30515408
tR(ACG)2	#N/A	#N/A	30515407
tR(CCG)1	#N/A	#N/A	30514970
tR(CCU)1	#N/A	#N/A	30515177
tR(UCU)1	#N/A	#N/A	30515192
tR(UCU)2	#N/A	#N/A	30515102
tR(UCU)3	#N/A	#N/A	30515106
tR(UCU)4	#N/A	#N/A	30514971
tR(UCU)5	#N/A	#N/A	30515223
tS(AGA)1	#N/A	#N/A	30515416
tS(AGA)2	#N/A	#N/A	30515425
tS(AGA)3	#N/A	#N/A	30515415
tS(AGA)4	#N/A	#N/A	30515126
tS(CAG)1	#N/A	#N/A	30515292
tS(CGA)1	#N/A	#N/A	30515036
tS(GCU)1	#N/A	#N/A	30515398
tS(GCU)2	#N/A	#N/A	30515397
tS(UGA)1	#N/A	#N/A	30514963
tS(UGA)2	#N/A	#N/A	30515279
tS(UGA)3	#N/A	#N/A	30515235
tT(AGU)1	#N/A	#N/A	30514998
tT(AGU)3	#N/A	#N/A	30515369
tT(AGU)4	#N/A	#N/A	30515116

tT(AGU)6	#N/A	#N/A	30514952
tT(CGU)1	#N/A	#N/A	30514983
tT(UGU)1	#N/A	#N/A	30515252
tT(UGU)2	#N/A	#N/A	30515278
tV(AAC)1	#N/A	#N/A	30515094
tV(AAC)2	#N/A	#N/A	30515180
tV(AAC)3	#N/A	#N/A	30515380
tV(AAC)4	#N/A	#N/A	30515414
tV(AAC)5	#N/A	#N/A	30515209
tV(AAC)6	#N/A	#N/A	30515210
tV(CAC)1	#N/A	#N/A	30515081
tV(UAC)1	#N/A	#N/A	30515165
tW(CCA)1	#N/A	#N/A	30515064
tW(CCA)2	#N/A	#N/A	30515220
tY(GUA)1	#N/A	#N/A	30515058
tY(GUA)2	#N/A	#N/A	30515183
tY(GUA)3	#N/A	#N/A	30515221
tY(GUA)4	#N/A	#N/A	30515059
ZCF4	#N/A	#N/A	3641799
CDC11	406.0459	0.000405	3647888
C1_04180W_A	275.1658	0.00097	3644716
C1_13100W_A	237.2895	0.00147	30515073
HOM6	223.57	2.29E-09	3636815
MCD4	192.4306	0.001931	3642036
COG4	179.0322	0.002182	3645571
DPM2	175.9478	0.002316	30515325
MLC1	162.1611	0.00288	30515387
SNZ1	154.2732	1.56E-16	3636803
LYS22	142.9577	5.43E-20	3645466
C3_06750W_A	142.5395	0.003657	30515219
DPM1	141.1045	4.01E-08	3640700
C6_02140W_A	136.9144	0.003689	3645886
VMA10	129.9214	0.00406	3645215
CR_05310W_A	126.3412	0.004293	3648125
C4_05390W_A	124.5216	0.004469	3646951
CDC10	119.6006	1.02E-07	3640947
C3_06670C_A	108.4452	0.006297	3642254
C3_02530W_A	108.3182	0.006189	30515189
C4_02460W_A	107.5906	0.006038	3637844
C7_00240W_A	103.5097	0.006161	3637980
SAM51	99.31514	0.006635	3641582
ALI1	96.26341	0.007021	3636635
PMI1	96.06303	1.05E-10	3643795
OST1	90.52435	3.71E-07	3643657
THR1	89.41438	4.15E-07	3641572
TNA1	88.85096	0.009031	3637827
FAD1	87.68012	0.008455	3634930

C3_02620C_A	86.03809	0.008813	3638326
C4_02600C_A	82.00375	0.009426	3637912
ECM331	73.75977	0.011475	3637731
ERV46	73.22009	1.13E-09	3644129
HHT21	73.1974	7.21E-06	3644707
PDI1	72.86423	6.02E-28	3641367
AMO2	70.75814	1.81E-12	3648021
CR_00280C_A	69.6498	0.012617	3639719
C2_10010C_A	68.89955	0.012723	3643962
C1_10560C_A	66.0944	0.013729	3647196
C1_07490C_A	65.68499	0.01403	3646666
C6_00400C_A	64.81734	0.014183	3646087
LYS9	64.53346	9.09E-15	3642279
PMT2	64.53029	3.99E-23	3638538
ACO2	60.92014	5.29E-12	3646708
FGR6-10	60.54686	0.01612	3641770
C5_03000C_A	59.96836	0.016217	3637818
C1_03600W_A	59.63087	4.75E-06	3636880
C1_06250W_A	59.33154	0.016317	3647525
HIS1	57.14062	2.39E-16	3636501
CR_06380C_A	55.47311	0.017879	3648139
DAG7	54.80046	3.27E-14	3635607
C6_01880W_A	53.89083	0.019453	3641599
C2_05510C_A	53.32149	0.019194	3643420
ILV5	52.94697	1.46E-23	3644012
C6_02180W_A	52.87934	0.019379	3645861
CCN1	52.85854	0.019346	3643735
C4_03170W_A	52.39649	0.01984	3637840
HRT2	52.05355	1.47E-05	3635709
LYS1	50.86196	1.62E-18	6335429
C1_03150C_A	50.54045	0.021426	30514976
KAR3	49.75862	0.021314	3644078
HIS5	49.06609	2.24E-08	3645756
MCD1	48.98444	0.022234	3639044
SHE3	48.9707	1.79E-05	3639277
C5_02780W_A	48.9067	0.022039	3637803
PMM1	48.82919	3.58E-12	3636852
DPP1	48.79069	0.022809	3644537
ASN1	48.38851	1.67E-29	3645790
TUB2	47.90142	1.2E-10	3639329
C1_14320C_A	47.17984	1.5E-05	3643316
POL30	46.88248	1.56E-05	3635583
CR_10130W_A	46.76085	0.023672	3638970
ERG28	46.1889	0.024003	3638833
SEC21	45.95567	4.78E-17	3644786
AGM1	45.83945	1.85E-05	3636138
PHA2	45.72024	0.024502	3638346

C6_00660C_A	44.87771	0.025219	3647133
C6_03320W_A	43.65185	8.63E-10	3639198
C6_00710W_A	43.00341	0.0273	3647073
TRP4	42.89982	4.25E-08	3642904
C2_09590C_A	42.42258	0.029786	3643800
C7_03650W_A	42.29636	0.027696	3646224
PGA6	42.09467	4.77E-05	3634774
ARF3	41.58626	9.11E-05	3636628
C1_07920W_A	41.28948	0.028951	30515026
YNK1	41.18569	7.34E-15	3637746
ERV29	41.1595	3.55E-12	3635672
ARO3	40.53567	4.86E-21	3635770
CR_01490C_A	40.51879	1.8E-09	30515376
LSC1	40.50232	7.09E-15	3640199
CWH43	40.41714	3.13E-10	3640389
C2_05720C_A	39.85327	3.24E-10	3645574
PMT4	39.1898	9.12E-17	3644096
EMP24	38.68005	1.49E-07	3647247
C5_04310W_A	38.67483	0.032178	3636477
CAR1	38.57203	1.62E-24	3636503
C4_02080W_A	38.37425	0.033095	3635643
IFE1	38.2529	0.036211	3644778
DDC1	38.22512	0.032867	3638374
C3_07130W_A	38.19304	0.033725	3638440
CWH8	38.11502	0.03336	3644598
CR_04650W_A	37.98332	0.033613	30515396
C2_01420C_A	37.63153	0.033851	3635841
CDC28	37.36951	4.38E-05	3644838
ASF1	37.32236	0.034049	3647348
C1_02100W_A	37.31863	0.034033	3644578
RFA2	36.63303	5.25E-05	3643506
MNT4	36.42031	0.035065	3647300
JEM1	36.104	4.27E-07	3643876
C3_06730W_A	35.11955	0.036811	3638504
C1_05100W_A	35.11251	0.036702	3647669
HGT12	35.08086	0.036943	3638048
YOX1	34.54032	0.038589	3637952
ACP12	34.51254	6.45E-05	3637361
DCG1	34.46972	0.038134	3638373
C1_02760W_A	34.11503	8.54E-11	3636870
RET2	33.89244	9.2E-15	3637714
TEM1	33.85644	0.041939	3636923
CR_04490C_A	33.78614	0.03868	3641013
C4_01140C_A	33.7658	0.038306	3635657
C5_03970W_A	33.60294	0.0399	3647443
CR_06500C_A	33.44098	0.039693	3645274
HIS7	33.25733	3.94E-14	3647868

CR_09330C_A	33.07476	6.17E-16	3641899
C7_00680W_A	32.88518	0.039754	3637967
GUP1	32.6537	0.040471	3636171
PXP2	32.13964	0.042191	3636558
C7_00220W_A	31.98172	0.042025	3637984
ARC18	31.95339	0.042201	3644056
C3_07540C_A	31.91293	0.04234	3638526
PRI2	31.79625	0.04308	3642788
C2_02330W_A	31.56205	0.042788	3635811
TUB4	30.70429	0.044614	3641774
C3_06640W_A	30.22917	0.045797	3642251
ERV25	30.16464	1.37E-10	3642243
SEC23	30.08194	1.17E-18	3641818
OGG1	29.32393	0.048141	3643106
CR_03470W_A	28.88377	0.000184	3643198
PHR1	27.89135	1.78E-24	3641108
SEC22	27.73284	0.000222	30515391
CR_05800C_A	27.23897	0.000233	3646706
CR_05130C_A	26.97175	0.000277	3646401
ERP5	26.93663	7.46E-10	30515046
HOM3	26.58608	1.04E-11	3641771
MET6	26.57654	4.95E-27	3640084
TUB1	26.22009	9.05E-14	3641930
CEK1	26.16379	2.78E-07	3642789
ATP19	26.15547	0.000228	30515061
C1_06700W_A	26.03628	0.00023	3639511
ASH2	25.31822	0.000282	3636445
C2_05290C_A	25.31005	2.09E-07	3643472
DOG1	25.23772	0.000297	3641597
C1_07260C_A	25.11474	0.000279	3644456
SSR1	24.81012	5.04E-13	3638055
C5_05020C_A	24.65637	2.91E-09	3636486
C4_06140C_A	24.43743	0.000512	3642821
SMC1	24.2106	8.24E-06	3643221
LEU4	23.88518	2.32E-07	3639339
ENA21	23.85014	1.28E-27	3641392
SWI6	23.77737	0.000372	3634932
MCM2	23.74465	0.000382	3643197
ATP18	23.72355	0.000358	30515084
IDP1	23.4614	2.36E-12	3647474
PMT1	23.37171	9.03E-21	3641393
C6_02190C_A	23.26296	7.75E-06	3645862
C6_01460C_A	23.24269	1.93E-10	3641623
SER33	22.96806	1.46E-20	3642030
POL1	22.84275	2.17E-10	3635144
RFC5	22.84046	0.000466	3638890
CEX1	22.52134	1.37E-07	3644913

C4_04380C_A	22.33243	0.001573	30515266
LYS12	21.91838	1.48E-14	3640134
CR_03950W_A	21.81382	0.000561	3640960
MCM6	21.66099	2.05E-07	3640142
PGA63	21.65504	1.56E-21	3639524
C7_00670C_A	21.34635	2.94E-10	3638024
CR_00990W_A	21.33279	0.000572	3640425
ATP1	21.31433	6.05E-25	3646134
ARO4	21.10176	2.86E-23	3647668
HET1	20.64012	1.61E-13	3647897
HTA2	20.58905	7.67E-08	3644715
CR_09230C_A	20.57057	2.83E-07	3641918
CRH11	20.4949	0.000673	3637905
C1_02970W_A	20.32729	3.68E-15	3636835
ERG11	20.31074	8.79E-10	3641571
MTS1	20.20753	1.38E-15	3634873
DPM3	20.16598	2.29E-05	30515245
APE3	19.95406	3.41E-07	3643875
ERG6	19.74397	4.63E-08	3636655
C1_06920C_A	19.57578	0.000954	3639525
HHT2	19.54691	1.06E-06	3647326
C5_01230C_A	19.36819	2.57E-11	3642618
C1_06050C_A	19.31331	0.000989	30515003
KAR2	19.14525	8.36E-20	3638830
SEC13	19.12796	7.19E-12	3638425
C3_02140C_A	19.11193	7.03E-07	3636656
C2_10200W_A	19.08437	0.001179	3643928
C5_02920W_A	18.94948	0.001079	3637751
SOU1	18.84178	4.33E-05	3642799
CR_03000C_A	18.84141	0.001105	3642597
CDC21	18.75703	0.001195	3643463
TSA1	18.74438	6.31E-21	3642269
FRP2	18.66683	5.73E-08	3638008
RBT4	18.50603	2.79E-10	3639534
MIS11	18.41703	2.3E-15	3645732
CR_06170W_A	18.37592	0.001217	3644865
SEC12	18.34094	2.91E-12	3641639
STT3	18.21213	3.23E-14	3635743
SAC1	18.18757	6.99E-08	3634864
BFR1	18.1865	4.47E-16	3635579
C2_07650C_A	17.99902	2.84E-05	3642342
SEP7	17.98058	8.42E-08	3644596
C1_04780C_A	17.93633	4.54E-08	3644774
CR_03330W_A	17.90707	0.001269	3645095
CMP1	17.87449	3.54E-09	3639328
C4_00710W_A	17.84933	3.17E-05	3645750
MET13	17.79704	4.07E-13	3638382

TSA1B	17.58265	1.13E-19	30515214
LYS4	17.50046	1.36E-09	3641180
RPS17B	17.46694	4.32E-15	30515044
C2_07140W_A	17.39697	5.48E-10	3643508
RGD1	17.34527	3.03E-06	3639868
C1_02500W_A	17.22083	0.001627	3636795
TOS1	17.12775	4.46E-15	3636566
C1_11860W_A	17.0753	3.23E-15	3642009
SPE3	16.94607	2.6E-11	3643528
QCR2	16.84526	2.22E-12	3646352
PHO15	16.76887	5.39E-08	3644459
CDC46	16.71384	5.68E-06	3646912
LEU2	16.7109	4.91E-06	3638034
PYC2	16.69224	1.54E-21	3637323
SSA2	16.62024	1.25E-14	3644711
MNT1	16.53563	6.12E-18	3636585
CDC12	16.50981	9.61E-13	3636809
CYB5	16.46188	7.96E-05	3637965
C1_01190C_A	16.3918	6.31E-05	3640207
CYT1	16.35053	1.84E-11	3643441
DOA1	16.2458	1.37E-11	3634871
LHS1	16.20511	7.03E-14	3643032
MYO2	15.97738	1.34E-13	3636140
MSH6	15.92072	6.52E-08	3636161
HAT1	15.91804	6.55E-05	3644770
RNR1	15.87371	2.91E-14	3642989
LAT1	15.83677	4.58E-18	3636986
CAR2	15.78708	1.04E-21	3645027
SEC18	15.69786	2.5E-14	3636208
RAD53	15.66208	6.84E-05	3645633
DDR48	15.59607	1.06E-07	3644061
MES1	15.56214	2.28E-14	3636516
C2_07010W_A	15.48026	2.44E-10	3643535
BGL2	15.27355	2.61E-15	3635691
SRB1	15.2707	7.25E-20	3647454
C2_01630W_A	15.26577	0.000176	3635738
ATP3	15.2309	2.34E-14	3640387
C1_04430C_A	15.22375	0.002396	30514986
C4_00390W_A	15.19026	7.18E-05	3645016
SAR1	15.13975	4.6E-10	3645865
CR_10530W_A	15.12212	0.002387	3639042
CWH41	15.01524	3.78E-08	3646533
CR_05500C_A	15.01049	0.002495	3647598
C1_03940W_A	14.80591	9.22E-05	3646415
ACT1	14.78859	1.65E-21	3636195
ERG26	14.68054	6.26E-06	3642803
CBR1	14.59997	3.42E-08	3646961

ASK1	14.54672	0.002722	3635656
SER1	14.51162	2.64E-12	3646909
C1_09980C_A	14.43771	0.000115	3634863
APL2	14.25341	1.69E-10	3638343
HUT1	14.12068	0.000264	3638505
CR_05360C_A	13.90819	0.003384	3648033
C5_00080C_A	13.87614	1.81E-10	3647886
KTR4	13.84255	0.003244	3646425
C2_07540W_A	13.83664	0.003244	3645222
SOL3	13.80437	2.11E-05	3646625
C3_06630W_A	13.73056	0.003377	3642250
GIN4	13.66342	8.52E-08	3644543
CR_10440W_A	13.64613	0.003734	3638976
SEC20	13.63676	0.000145	3639206
C5_03120W_A	13.49933	0.003626	3637705
GND1	13.4515	6.42E-14	3636131
SPC2	13.42051	0.000249	3645769
LSC2	13.40363	2.98E-13	3646618
PSF2	13.39695	0.004014	3647534
C6_02480W_A	13.26999	1.04E-05	3639170
SSH1	13.22844	6.86E-08	3642504
C2_03970W_A	13.13212	0.004004	3637419
RAD54	13.13111	0.005378	3636192
RHB1	13.06233	0.000251	3635232
CLC1	12.97633	1.67E-11	3635662
OPI3	12.91513	0.004242	3642277
CR_03520C_A	12.88094	5.13E-08	3643203
SEC62	12.84839	5.64E-10	3636860
C2_09040W_A	12.82866	1.46E-07	3645792
AAT1	12.81671	3.63E-16	3643468
CR_08480C_A	12.57128	7.06E-08	30515442
PDB1	12.56998	1.95E-16	3641186
C4_04160W_A	12.5144	2.53E-12	3641185
C4_07080C_A	12.50956	0.00029	3642905
PDX1	12.49573	9.17E-13	3636128
EGD2	12.4542	1.08E-12	3635284
C1_07590C_A	12.43572	4.19E-06	3646673
RPL24A	12.39965	8.28E-13	3641174
C3_00520W_A	12.3143	0.000287	3640513
MSB1	12.31125	2.34E-07	3648218
C4_04980W_A	12.23604	6.17E-05	3641167
CSM3	12.23378	0.005474	3644083
EAF6	12.20868	0.005221	3646494
PLC2	12.07308	4.87E-05	3642985
QCR8	12.03389	5.03E-08	30515112
FRS2	12.03274	6.69E-14	3636824
TEF2	12.00451	2.35E-18	3646502

THR4	11.98206	9.1E-13	3637711
C3_06400C_A	11.97039	0.000301	3642260
C1_09060C_A	11.93232	0.007717	3634772
COF1	11.9188	2.52E-11	30515293
HOM2	11.87905	8.26E-14	3635826
RFC2	11.87858	4.39E-05	3637939
CR_05010W_A	11.87009	0.000344	3646389
MNS1	11.85955	2.88E-09	3644692
APL4	11.82786	3.15E-09	3642565
C4_04440W_A	11.73857	3.35E-09	3641177
VRG4	11.71523	1.43E-09	3641768
C6_04100W_A	11.62362	2.63E-09	3647836
C1_12650C_A	11.55875	0.00738	3646836
SKN1	11.48118	1.67E-11	3641755
EGD1	11.4401	2.61E-13	3645340
CAS1	11.43106	5.9E-05	3645324
C6_00770C_A	11.41669	0.007058	3647079
COX5	11.39963	1.85E-09	3634768
C4_01510W_A	11.39051	1.59E-06	3635616
GDE1	11.31519	1.83E-11	3636505
RFA1	11.24159	1.36E-09	3638823
HMG1	11.22866	3.59E-10	3644687
HHO1	11.22623	0.000189	30515358
C5_01960C_A	11.17034	8.65E-11	3643738
OSH3	11.16694	1.08E-07	3645368
C2_01910W_A	11.16297	0.007332	3635790
C2_09780C_A	11.15201	1.96E-08	3643805
C4_02930W_A	11.12911	0.000953	3637876
ALG8	11.10155	0.000609	3636562
DED81	11.08953	1.43E-12	3646227
ARO8	11.06642	1.01E-16	3638828
MET15	11.04391	3.73E-12	3645031
C7_01710W_A	11.03878	8.31E-09	3637012
TVP18	11.00045	8.07E-05	3634844
ADK1	10.99047	3.76E-11	3644533
RPP2A	10.9492	2.83E-11	30515439
MEU1	10.94716	1.19E-07	3645635
RPS15	10.93509	2.61E-12	3635194
ALS4	10.88601	6.83E-14	3647972
C2_06610C_A	10.81072	0.00865	30515140
C1_03240W_A	10.78065	5.94E-05	3636895
CR_00130C_A	10.76485	5.41E-08	3639709
CTP1	10.72318	6.47E-05	3635141
C3_07230W_A	10.72238	1.46E-09	3638531
C3_01070C_A	10.70899	0.008924	3639832
CR_02060W_A	10.69427	1.42E-09	3640155
DPB4	10.6643	5.84E-05	3638818

ZWF1	10.63483	1.28E-10	3634913
ILV2	10.63139	1.98E-15	3636552
SAM4	10.63126	1.72E-12	3646506
RIC1	10.60785	5.56E-05	3639331
C2_06770W_A	10.54858	0.009716	3643515
CR_09520C_A	10.51288	0.001137	3647103
CAM1-1	10.45604	2.25E-12	3646358
C5_00840W_A	10.44894	0.001089	3644130
CHO1	10.39041	1.03E-05	3644557
ILV6	10.33833	2.74E-10	3635598
C1_04030W_A	10.32999	6.93E-05	3646428
SGA1	10.28961	1.49E-05	3636652
C1_06630W_A	10.2843	1.05E-05	3639544
C6_03130W_A	10.26933	7.83E-05	30515341
ERG2	10.23639	0.010629	3639347
C2_00590W_A	10.18429	0.001042	3638851
C5_02210W_A	10.15276	4.77E-06	3637759
SMC5	10.12788	1.08E-05	3645103
ARO2	10.11045	4.6E-13	3648190
C7_03310W_A	10.03312	0.00108	3646726
ALD5	9.973648	2.83E-16	3643026
C2_02020W_A	9.961019	1.21E-08	3635769
LYS2	9.958425	3.57E-14	3636875
C3_06920W_A	9.943139	3.5E-07	3638511
TOP2	9.897009	1.89E-08	3642777
ERG13	9.891973	6.82E-09	3641881
CAC2	9.89002	0.00119	3646978
C1_09670C_A	9.88735	0.012141	3634872
RPL8B	9.83142	9.76E-15	3635198
C5_05350W_A	9.789826	3.02E-05	3636401
ATP2	9.779436	3.52E-16	3645039
CYP1	9.752543	4.87E-11	3636978
ACS2	9.73279	9.97E-12	3644710
C2_07420W_A	9.731897	1.11E-06	3645202
GRP1	9.695598	0.001271	3634899
C1_05180C_A	9.673148	0.013357	30514990
TRP3	9.671622	1.81E-08	3642035
SMC2	9.636691	3.61E-05	3643918
HSL1	9.633713	9.97E-08	3637810
IDH1	9.577579	4.34E-14	3634893
OAC1	9.569628	0.015849	3642245
C4_03290W_A	9.464996	4.29E-09	3637887
DUT1	9.463191	0.000171	3640188
C4_05790W_A	9.405931	2.71E-06	3641784
C5_02380W_A	9.401487	2.38E-16	3637794
SMC3	9.369147	0.001873	3638408
C1_07510W_A	9.365794	0.001861	3646664

C7_04000W_A	9.343899	4.25E-06	3643114
PDR17	9.329259	0.001783	3643007
REX3	9.306021	0.01417	3636164
C6_02880W_A	9.304157	0.014306	3639238
CR_00880W_A	9.208763	0.002302	3640397
HYR1	9.208644	0.016633	3636226
MPS1	9.187556	0.014587	3641888
CR_04080C_A	9.18505	1.55E-10	3641023
VMA2	9.139385	5.95E-12	3646710
SOD1	9.136383	8.11E-06	30515248
C1_11960C_A	9.077334	0.014934	30515060
C6_02560W_A	9.050264	3.06E-05	3639205
C3_05280C_A	9.037409	4.67E-05	3635202
EXO1	9.015506	0.000227	3641583
PTP3	9.006547	6.33E-11	3638940
ECM33	8.997922	7.89E-11	30514977
TAL1	8.978829	4.67E-09	3643225
C1_04200C_A	8.972663	0.000216	3644718
DQD1	8.971236	2.82E-07	3643488
C2_07110C_A	8.965256	6.26E-06	3643505
INP51	8.9525	6.58E-05	3643806
ADE6	8.92592	1.65E-12	3647304
DSL1	8.920435	0.000229	3645718
CDC54	8.872699	0.000332	3647606
SPC3	8.854483	7.85E-05	3636121
HAM1	8.84441	2.44E-08	3647224
CR_04730W_A	8.836171	0.000296	3647305
MMS21	8.82169	0.002024	3642265
CLB4	8.787597	0.002218	3643102
C4_04970C_A	8.726785	0.00027	30515274
C4_00020W_A	8.704132	6.56E-09	3648053
IPP1	8.690845	1.16E-09	3643874
C2_09050C_A	8.634304	5.51E-05	3645791
THS1	8.624168	1.55E-13	3647882
C1_00700W_A	8.609057	0.016597	3639330
C5_03290C_A	8.567203	0.018509	30515307
APL5	8.553657	7.36E-09	3638377
SEC61	8.53935	1.86E-12	3647462
C2_03020C_A	8.522299	9.43E-11	3643019
SEC8	8.513353	1.09E-07	3647078
HFL1	8.508766	0.002184	3636883
ARC35	8.494841	5.46E-06	3639823
C1_12910W_A	8.486211	0.002485	30515066
GEA2	8.44741	1.46E-09	3636646
LEU1	8.425389	3.17E-10	3639685
GFA1	8.419144	4.71E-09	3636557
ABG1	8.413124	5.69E-06	3647715

TOM20	8.381722	4.03E-07	3636817
BIO2	8.379576	0.018683	3640106
PPT2	8.375903	0.018765	3634762
RAM1	8.368571	0.00224	3647859
C1_06480C_A	8.313301	5.59E-06	30515008
UBA2	8.281534	2.55E-06	3640701
PHO114	8.262969	0.019539	3635644
RPL43A	8.255183	6.69E-10	30515319
C4_06940C_A	8.242936	1.63E-05	3642928
FAS2	8.242185	1.26E-11	3635222
CDC42	8.241186	3.55E-06	3646488
C4_04800W_A	8.230399	7.74E-11	3641113
CDC19	8.228037	7.66E-13	3643438
C6_02250W_A	8.225596	0.021815	3645869
LAP41	8.222554	4.43E-07	3636653
C2_01260W_A	8.204708	0.002587	3638792
PRS	8.203115	0.000441	3640100
ADE1	8.201105	5.05E-09	3639722
PMT5	8.198749	1.53E-05	3639011
C2_02410W_A	8.179274	2.61E-07	3635755
TMA19	8.170876	4.53E-12	3640399
MRP8	8.152179	2.33E-05	3641178
RTT109	8.150998	0.021029	3639678
ARC40	8.141019	9.63E-11	3644866
RAD52	8.131569	6.85E-05	3647148
C7_03640C_A	8.099616	0.002586	3646223
RHO3	8.085924	1.59E-05	3643448
GRS1	8.074464	7.89E-12	3642474
C3_01310W_A	8.013649	2.25E-05	3636620
C7_03800W_A	8.009412	0.000694	3643150
C1_02330C_A	7.976665	2.64E-09	30514969
C2_09630C_A	7.951831	0.025076	3643796
STV1	7.941664	7E-10	3646070
CDC3	7.931708	2.33E-10	3644719
NPL4	7.912293	8.84E-06	3639820
KRE5	7.891782	1.92E-09	3638384
MCR1	7.882138	5.8E-09	3647561
C1_02290C_A	7.877088	9.1E-05	3644568
YPT31	7.851293	3.17E-09	3647999
C4_01460C_A	7.828443	0.003057	3635622
GLC3	7.821332	1.53E-10	3639200
CR_04500C_A	7.818015	7.61E-07	3641014
PDA1	7.772054	1.66E-13	3642902
CR_01320C_A	7.732949	7.23E-06	3640386
ACB1	7.709871	6.68E-06	30515350
ASM3	7.661342	0.003953	3639332
RFC3	7.655825	0.000803	3647440

PST3	7.650721	8.2E-10	3648031
TSC11	7.624203	0.003531	3645267
CR_04760C_A	7.621528	0.004454	3647303
C2_09100C_A	7.616783	5.71E-05	3644113
PRO2	7.61595	1.41E-09	3638532
RPS12	7.564102	1.82E-09	3638438
SEC27	7.533233	6.86E-10	3645870
C3_02210C_A	7.531265	9.7E-06	3636641
KEX2	7.525802	1.74E-10	3634914
C1_05320C_A	7.524919	0.000111	3642470
SPE1	7.518258	8.61E-10	3639327
C2_03340W_A	7.463353	8.92E-06	3637392
MDH1-1	7.462519	2.73E-07	3635636
C4_04920W_A	7.449416	0.00015	3641172
C1_03170C_A	7.44279	0.030963	3636805
C2_07550W_A	7.440494	3.67E-07	3645221
CUP5	7.438527	1.45E-08	3635174
MNN26	7.418743	1.55E-07	3646240
GLO3	7.410495	1.9E-11	3640556
AGE3	7.393084	1.6E-07	3644565
SUB2	7.361759	8.33E-11	3645033
C4_03050C_A	7.339626	1.23E-05	3637930
C1_01080W_A	7.332162	0.032322	3640173
UGA32	7.331749	0.001271	3639333
NIF3	7.321516	8.6E-05	3646527
LMO1	7.296223	4.14E-09	3641403
C7_01320W_A	7.292332	4.07E-07	3645896
C4_05730W_A	7.282043	0.037015	3641805
ARG2	7.265348	0.000202	3647533
NHP6A	7.263212	9.82E-10	30515244
C4_04600C_A	7.256836	0.004796	3641156
HHF1	7.241544	5.63E-07	3644705
C6_01960W_A	7.226764	0.001027	3644559
RBT1	7.225285	0.00273	3645377
APM4	7.210953	0.001089	3642297
SEC72	7.196325	5.52E-06	3645768
CDC7	7.157702	1.51E-05	3643424
C2_05800C_A	7.144016	7.45E-07	3645584
STE13	7.142128	2.07E-05	3647597
CR_07170W_A	7.139577	1.1E-06	3645271
C4_02420C_A	7.101891	0.033667	3637849
TRX1	7.099601	3.62E-08	3638950
C1_01800W_A	7.090705	0.005165	3645407
C4_04090C_A	7.084619	8.78E-06	3641225
C4_06810C_A	7.073923	3.66E-10	3642916
GIN1	7.064587	0.000429	3644539
TYR1	7.041527	8.56E-06	3635639

AIP2	7.006166	8.95E-10	3638393
APM1	7.005744	5.27E-07	3636424
SPF1	6.990776	2.1E-10	3646573
C4_01840C_A	6.986762	5.6E-05	3635592
CR_00710C_A	6.98606	0.036884	3640440
C7_01740C_A	6.939064	0.000266	3637010
C1_09020W_A	6.937987	9.27E-05	3634917
C6_03950C_A	6.928372	0.000512	3640268
C2_05850C_A	6.927677	7.86E-06	3647472
CR_08610W_A	6.920742	9.37E-05	3642109
PRO3	6.913997	1.1E-08	3645036
C1_05990C_A	6.904218	0.000418	3639871
C1_11740W_A	6.890458	0.001404	3645317
MAE1	6.866658	2.83E-09	3641615
ARP3	6.84482	1.28E-07	3643479
SAH1	6.831	1.86E-12	3636452
ALG6	6.82563	0.000111	3647316
ILV1	6.818384	7.17E-09	3646918
NIT3	6.816891	1.77E-06	3644408
C7_00600C_A	6.813585	0.000345	3638031
CR_07080W_A	6.808797	1.41E-08	3647964
C2_06270W_A	6.804393	0.269801	3646921
C6_04280W_A	6.80146	1.13E-06	3646279
RPS7A	6.777799	3.21E-09	3636573
C2_07270W_A	6.776599	8.9E-06	3643474
C5_00060C_A	6.772621	0.000101	3647889
HNT2	6.751383	0.03953	3642271
SMP3	6.74839	0.041517	3642980
ILV3	6.73848	6.26E-11	3636428
ATP5	6.710766	3.35E-10	3640521
VMA5	6.703852	1.84E-05	3642316
IFA21	6.700315	0.000435	3641344
NUO1	6.690364	3.29E-05	3647100
SAS2	6.689914	0.006785	3638817
PHO84	6.661232	0.000113	3645348
C2_02260W_A	6.636304	4.5E-05	3635778
C5_04950C_A	6.634084	0.000104	3636422
QCR7	6.623472	1.73E-05	3639233
GVP36	6.594524	0.0001	3641772
C4_05080C_A	6.581638	0.000154	3641129
RKI1	6.57924	0.008027	3636574
C1_01210W_A	6.561873	0.000545	3640209
YBL053	6.530374	0.000119	3647045
C2_06950C_A	6.527558	0.00223	3643527
C3_00470W_A	6.512487	1.81E-06	3640520
C1_07340W_A	6.508717	5.24E-09	3644472
C2_03450W_A	6.506118	0.000359	3637349

ADO1	6.460215	1.73E-08	3639274
SEC26	6.454165	6.61E-10	3641005
YBN5	6.447001	1.72E-09	3644781
CR_00870C_A	6.438188	0.049285	3640398
CR_05760C_A	6.421931	0.008081	3646695
C4_06160W_A	6.420655	0.008256	3642819
PET9	6.415708	7.69E-11	3641587
C7_02100W_A	6.382214	1.89E-08	3637023
C5_05040W_A	6.365587	0.000433	3636488
C3_03510C_A	6.365163	2.97E-05	3638310
YHB5	6.357313	1.7E-06	3647343
C2_00730C_A	6.350564	1.76E-05	3638913
C1_11280W_A	6.335701	1.8E-05	3644554
CAK1	6.312772	0.000116	3637352
VAS1	6.309483	1.3E-10	3648340
RNH35	6.303539	0.048752	3636987
C3_05880C_A	6.300741	1.85E-08	3641737
C2_02090W_A	6.295846	9.16E-05	3635854
DUN1	6.29213	0.002502	3636494
SEC24	6.290881	5E-11	3634939
VMA8	6.280749	1.27E-05	3642798
CR_01080W_A	6.271585	1.86E-08	3640381
INO1	6.25158	3.62E-11	3638966
FRE30	6.239456	7.22E-08	3646025
C1_13270W_A	6.21854	0.000249	3636144
C7_02920W_A	6.179736	0.000193	3641391
C2_09180W_A	6.175978	0.049547	3644092
RIP1	6.16901	1.01E-09	3635204
POL3	6.153346	3.86E-06	3641359
C2_05130W_A	6.152908	0.000757	3643458
C1_10540C_A	6.15212	0.000766	3647193
VMA11	6.148575	0.000751	3637054
TPD3	6.146478	9.43E-08	3638512
CR_09010C_A	6.114437	3.55E-09	3641892
KIP3	6.10957	0.000854	3641716
C3_01720C_A	6.105729	7.73E-10	3636601
HBR2	6.097537	1.08E-09	3646273
CRN1	6.083763	8.59E-08	3637034
RPL10A	6.053539	1.79E-10	3645868
C2_02310W_A	6.052685	2.66E-05	3635809
PMR1	6.052003	2.01E-08	3638043
CR_08000C_A	6.047339	7.56E-09	3644150
C1_11760C_A	6.040944	0.000865	3645328
HGT10	6.030827	2.39E-05	3640309
PUP2	6.027624	5.68E-08	3646617
RPP1B	6.019769	6.8E-09	3643104
CR_05200C_A	6.011023	0.001509	3648080

FRE7	6.005746	4.84E-08	3646024
C5_04410C_A	6.001958	0.003437	3636410
FTR2	5.998181	3.8E-06	3643303
ARE2	5.978174	1.54E-06	3643526
C4_03400W_A	5.958391	0.012282	30515256
NUC2	5.941332	3.87E-08	3637030
CR_02630C_A	5.938291	0.052872	3642568
ERG10	5.907373	7.57E-08	3648275
HTA3	5.891741	0.001676	3638322
DOT1	5.881604	3.65E-05	3642236
TPM2	5.876837	1.04E-07	30515441
ATP17	5.842354	1.53E-07	30515364
CR_09670C_A	5.834776	2.11E-05	3647091
RPP1A	5.831991	1.31E-09	3636839
C3_01020W_A	5.824862	0.013766	3639828
SHM1	5.824039	1.81E-09	3646724
C4_00380W_A	5.824005	0.000389	3645015
KSR1	5.822609	0.001381	3646052
C5_01410C_A	5.81772	1.69E-05	3647054
APT1	5.809779	1.69E-05	3635842
GIT2	5.807191	1.3E-05	3642633
IFA4	5.777697	0.015492	3645454
CR_07600W_A	5.773915	1.89E-06	3644849
RPS1	5.773699	2.5E-10	3636924
ATP4	5.768569	9.15E-10	3643418
CR_08670C_A	5.762241	0.001476	3642126
HEM15	5.757403	3.79E-08	3645211
C4_06460C_A	5.755404	0.015078	3642791
STE24	5.753352	3.89E-08	3645040
C4_00770C_A	5.716491	5.75E-05	3645760
WBP1	5.71607	3.25E-08	3644398
RPS27A	5.714142	0.003685	30514993
C1_12240C_A	5.703963	0.000128	3642023
MNN2	5.703786	2.68E-05	3644405
MAM33	5.681036	4.29E-07	3643103
MSM1	5.671003	0.003805	3636398
NPT1	5.6678	1.73E-06	3643109
C1_08800W_A	5.651955	7.43E-05	3634791
FAT1	5.639984	1.48E-07	3647083
C5_04550W_A	5.635055	0.003609	3636508
SNF5	5.630133	0.000151	3648297
PEX11	5.628035	0.001735	3646288
C4_01470W_A	5.626584	0.017205	3635621
C4_05310W_A	5.624535	0.016766	3646958
DPP2	5.622647	0.016936	3645341
C4_03770W_A	5.622286	0.016706	3645354
BMH1	5.620237	3.52E-10	3636810

ESC4	5.619737	0.017926	3635727
BNR1	5.6148	8.54E-05	3639672
C6_02470W_A	5.603728	1.52E-06	3639169
C7_01460C_A	5.584913	0.004111	3636958
MYO1	5.583055	1.82E-05	3647248
C1_05490C_A	5.574961	2.55E-05	3642507
DPB2	5.571733	0.001592	3639026
C1_12180C_A	5.564242	0.005614	3642038
ZCF24	5.53244	0.001705	3645417
CR_06070W_A	5.529448	3.84E-05	3644840
C1_01250W_A	5.516032	4.5E-05	3640212
DAP1	5.506948	0.004944	3641021
CR_07110C_A	5.486048	0.01761	3648224
SEC34	5.472538	9.36E-07	3644455
RPL19A	5.467882	6.11E-10	3635257
FGR34	5.445887	1.17E-05	3639033
C1_01050C_A	5.439976	7.41E-06	3640170
PHO89	5.435585	3.68E-08	3635633
C1_00210C_A	5.435489	4.76E-08	3639335
CR_00910W_A	5.434142	9.76E-08	30515371
C6_01750C_A	5.41842	0.019331	3641640
VPS1	5.410306	4.98E-08	3642656
CR_04880W_A	5.382258	0.002102	3647309
ERG12	5.368015	2.17E-05	3634859
ATP14	5.363392	1.82E-07	30515134
RHO1	5.361425	2.31E-08	3642564
C1_02890C_A	5.353836	0.019183	3636867
C1_01290C_A	5.35242	0.01971	3640184
RNH1	5.344427	3.39E-05	3639237
BAT21	5.326892	3.85E-08	3637356
C5_01590W_A	5.316898	0.002023	3647041
C3_04780C_A	5.308278	4.55E-05	3635214
NAM2	5.302605	0.000215	3640275
RPL18	5.299763	3.67E-08	3635156
PRD1	5.298716	1.37E-08	3642471
CR_03560W_A	5.290111	0.020532	3643217
MSH2	5.288046	1.72E-05	3642939
C1_14240W_A	5.279649	2.81E-06	3643306
PMS1	5.275261	0.006994	3647714
ATP7	5.272535	1.23E-06	3646672
C1_00630W_A	5.245818	0.000659	3639380
MSI3	5.240144	2.22E-09	3639821
POL2	5.233839	3.53E-06	3645733
CR_07350W_A	5.228175	1.28E-08	3646055
RPS8A	5.219429	7.86E-10	3645565
DNM1	5.217753	1.02E-07	3643673
BIO32	5.215862	0.005921	3643430

SMF3	5.210898	3.6E-05	3638852
C5_04150C_A	5.210202	0.006115	3636525
RFC4	5.206125	0.00032	3638994
C4_01730C_A	5.19266	3.63E-06	3635588
C3_07820W_A	5.181889	0.000908	3647463
CR_00060C_A	5.162645	2.96E-06	3639673
C6_03960W_A	5.162412	6.8E-07	3640269
C2_03170W_A	5.155861	0.021591	3642988
GLO2	5.154075	0.000418	3644067
C6_00930C_A	5.138709	7.7E-05	3644032
RDI1	5.133885	5.59E-07	3635167
C4_04180C_A	5.12665	0.005424	3641183
CSR1	5.126583	0.007696	3641162
PMT6	5.124051	5.01E-07	3641116
LSP1	5.120179	1.46E-07	3648025
SNL1	5.104261	8.49E-05	3647192
C3_04970C_A	5.097223	5.39E-07	3635164
CR_04870C_A	5.095471	0.0318	3647310
RPL28	5.093557	1.21E-09	30515386
MET3	5.088954	5.03E-08	3636132
C2_09250W_A	5.086846	0.022275	3644065
C5_03950W_A	5.083765	0.000943	3647445
END3	5.065069	9.16E-05	3636644
HFL2	5.059892	0.001147	3637833
C3_06610W_A	5.059	0.000553	3642248
C4_05250W_A	5.047525	0.009893	3646158
CR_10140W_A	5.04611	6.64E-08	3638972
MNN10	5.044683	3.88E-05	3644997
C1_14060W_A	5.042761	4.7E-05	3643344
CYK3	5.037869	0.007496	3639539
TOM22	5.031839	6.47E-05	3647650
C1_12400C_A	5.027137	0.003646	3642059
CAM1	5.007525	2.31E-08	3641734
C1_07820W_A	4.999662	0.007808	3640735
C1_12070C_A	4.997897	0.000469	3642025
SHP1	4.996628	1.1E-07	3640083
CAT8	4.975888	0.00012	3640745
ATF1	4.973109	1.63E-06	3635273
C6_03330C_A	4.970806	0.000246	3639199
C1_00450C_A	4.969091	0.00021	30514955
RAD10	4.967398	0.008079	3639884
NDH51	4.964949	2.25E-08	3645444
C3_04740C_A	4.961065	0.007977	3635251
APN1	4.957225	0.000156	3642263
C2_04830W_A	4.95617	0.023196	3648115
MNN24	4.955806	6.83E-06	3638789
C2_01160W_A	4.947681	0.001729	3638861

C1_02430C_A	4.945734	1.56E-07	3636846
RPL35	4.935694	1.79E-07	30515207
AAT22	4.931903	0.000564	3635651
C2_08930W_A	4.914037	3.91E-06	3645795
RPS16A	4.894593	3.89E-07	30514974
PGA53	4.892495	0.000176	3635599
POR1	4.888378	3.14E-09	3644698
TIP1	4.885867	0.002017	3636512
C2_08160C_A	4.87292	1.63E-06	30515152
SGT2	4.87238	1.77E-07	3642978
YHM1	4.864402	4.26E-05	3644535
C4_02040W_A	4.857918	0.000699	3635649
LAC1	4.852401	0.000995	3641717
RPS26A	4.816048	9.8E-07	3635787
C4_00910C_A	4.811309	0.010403	3635679
RAD7	4.803281	8.77E-05	3635833
EFB1	4.797786	3.01E-08	3641125
IDH2	4.779273	9.09E-08	3642979
CR_06770C_A	4.768241	0.002131	3647313
C1_04280C_A	4.765485	0.010267	3644709
NRM1	4.755326	0.027901	3639343
HTA1	4.745461	0.000227	3645658
C3_05300C_A	4.735547	0.004385	3643646
C5_01550C_A	4.726659	4.73E-07	3647063
CDC50	4.717538	7.34E-05	3640302
ACC1	4.717537	2.56E-06	3639688
MPT5	4.710817	4.36E-05	3636205
KGD2	4.707671	5.13E-08	3646036
C1_05670W_A	4.70125	1.56E-05	3639899
C2_05560W_A	4.700095	0.010777	3645561
BUB1	4.696992	0.000287	3637922
BAT22	4.693172	4.54E-07	3643663
ERG24	4.692888	0.003427	3647716
C1_07780W_A	4.691811	5.4E-06	3647955
C1_02910C_A	4.688869	0.029948	3636829
C2_09480W_A	4.687665	0.032245	3643819
MSC7	4.686602	0.000218	3645214
C3_01510W_A	4.682161	0.000984	3636571
TFP1	4.68061	2.54E-07	3636662
ARP1	4.652025	0.032692	3646349
SSB1	4.650623	1.95E-08	3642095
VMA7	4.650436	0.011412	3637332
C2_06890C_A	4.647223	0.041362	3643522
RPL20B	4.643989	1.03E-06	3635615
AOX1	4.643605	0.012741	3634946
C1_13030C_A	4.638471	5.75E-08	3636122
C1_04090C_A	4.630828	0.004964	3644697

C4_03720C_A	4.627934	9.43E-05	3645359
EXG2	4.626357	0.016161	3636816
IFG3	4.621338	9.08E-06	3641545
CR_01070W_A	4.618047	0.032432	3640382
CTA2	4.611751	2.13E-05	3639372
C3_06410C_A	4.60406	0.011892	3642261
C6_02990W_A	4.603132	0.017428	3639193
CR_07230W_A	4.600437	0.000403	3646031
GSP1	4.574307	7.07E-08	3646925
MVB12	4.572572	0.005731	30515400
C1_04470C_A	4.57209	0.004968	3646142
MET14	4.563955	0.000384	3641547
CDC47	4.559224	1.08E-05	3645780
CDC24	4.552297	1.52E-05	3643737
NCE103	4.549935	1.43E-06	3636621
C5_05230C_A	4.546426	8.86E-08	3636470
C6_00910C_A	4.537706	7.1E-08	3644030
C2_09560C_A	4.536696	7.29E-05	3643813
C3_01180C_A	4.52384	9.12E-06	3647960
C3_01610W_A	4.511429	1.01E-07	3636664
C7_01480W_A	4.506146	0.000864	3636956
BMT4	4.503457	0.002834	3639182
C3_01940C_A	4.501519	0.000587	3636600
NDE1	4.501024	8.81E-08	3638354
FGR51	4.500607	0.0007	3647796
PEX8	4.497323	0.005468	3647393
C4_04280C_A	4.495763	0.01412	3646846
MON2	4.488257	2.36E-06	3636201
C1_12060C_A	4.488039	0.036594	3642026
C1_10750C_A	4.48697	0.037152	30515043
C3_01590W_A	4.485217	0.004956	3636666
LIP8	4.45308	0.00078	3646727
TEF1	4.452613	6.11E-08	3648254
C1_14160W_A	4.447252	0.005264	3643322
STE23	4.438141	1.92E-05	3639236
C2_09970C_A	4.436596	2.33E-06	3643820
GNA1	4.435355	0.00128	3637397
C2_04330C_A	4.432003	0.000204	3645453
C7_02520W_A	4.430914	0.10732	3636941
DCC1	4.422291	0.039802	3638037
ZCF5	4.421886	0.005918	3641819
C4_00190W_A	4.420791	0.039646	3645030
C3_04810C_A	4.416172	1.02E-08	30515205
C5_05200C_A	4.413677	0.003152	3636467
TOM40	4.404223	2.06E-07	3636968
C1_14530W_A	4.399864	3.57E-05	3643333
C7_02670W_A	4.388279	0.001091	3647651

LYS21	4.387742	8.07E-07	3644763
C1_08660C_A	4.387578	0.017505	3634931
C1_04190C_A	4.385165	0.001537	3644717
PRN4	4.379989	0.005697	3639877
C1_06270W_A	4.376277	0.004431	3647523
C1_03490W_A	4.367843	0.0003	3636914
HIS3	4.366873	0.007017	3647777
CTA7	4.362475	4.74E-05	3637776
ADE5,7	4.360507	9.45E-08	3640719
SER2	4.35674	0.000333	3643006
HEM14	4.355543	0.000374	3634923
CR_04560C_A	4.340273	0.002082	3640946
C3_04590W_A	4.334837	1.85E-05	30515204
ARO9	4.330699	0.003647	3641773
MNN21	4.324294	2.23E-05	3648124
C6_00920W_A	4.324198	6.48E-05	3644031
C7_03660C_A	4.320427	6.43E-07	3646226
C3_02820C_A	4.316082	0.000535	3638419
C3_03130C_A	4.314512	0.112674	3638421
CDC68	4.309045	3.97E-07	3642787
HXK2	4.304752	3.37E-07	3641015
COQ3	4.304635	0.000364	3641630
PGA10	4.30172	0.046013	3645009
VPS23	4.295362	0.023256	3644401
BNI4	4.283886	8.05E-06	3646406
C3_01900C_A	4.280125	0.00234	3636559
ANB1	4.277301	2.76E-06	3641648
C2_10860C_A	4.272129	2.84E-06	3641233
AYR1	4.26939	0.004795	3647754
C7_02080W_A	4.266632	0.007075	3637025
MCM3	4.2499	1.21E-05	3645223
PDC11	4.240961	2.73E-06	3642780
C2_01320W_A	4.240328	0.000497	3638846
C1_00330C_A	4.237908	0.001849	3639296
LAG1	4.237317	0.004489	3640383
C1_03720C_A	4.234242	0.000521	3648216
PGA38	4.233899	0.029818	3637846
C1_10630C_A	4.232515	0.00709	3647683
C5_02520W_A	4.225779	0.012787	3637768
RPL17B	4.223426	5E-07	3645439
PST2	4.221207	0.035559	3643909
AAP1	4.217598	0.020772	3647397
C6_02030C_A	4.217012	0.001295	3647562
SHM2	4.216744	1.14E-06	3640306
ALS1	4.210867	2.73E-07	3640280
ERV1	4.19781	0.007647	30515385
CR_01280C_A	4.196714	0.007468	3640400

MUM2	4.196061	1.91E-05	3636432
CR_03600C_A	4.185293	4.5E-05	3643207
C7_01500W_A	4.175215	0.024141	3637050
C5_03930C_A	4.173703	0.022542	3647447
CR_00290W_A	4.173167	2.25E-06	3639717
TRP5	4.166045	3.09E-06	3648087
C1_08080C_A	4.16352	0.000428	30515029
PLC1	4.140614	0.000665	3647860
C5_04420W_A	4.139826	0.002389	3636411
C1_08730W_A	4.135155	0.021562	3634938
C1_01950C_A	4.133357	0.021649	3645421
CRC1	4.097481	0.000697	3640112
MMS22	4.087647	0.009052	3639681
C4_06950W_A	4.086793	0.02315	3642927
C7_03200C_A	4.085357	0.000861	3641342
TOM70	4.081911	2.39E-07	3647645
C3_03560W_A	4.080071	0.001411	3638299
VMA13	4.058947	2.25E-05	30515402
C3_06970W_A	4.054785	0.000114	3638506
LRO1	4.05036	2.78E-05	3639356
C6_02280W_A	4.047008	0.000147	3645872
C6_04560W_A	4.045905	1.67E-06	3645977
PFY1	4.044788	1.95E-06	3640703
SEF2	4.034528	0.063617	3647052
ROB1	4.032125	1.56E-06	3636212
RPS3	4.028718	1.04E-05	3647299
GWT1	4.015775	0.001651	3645575
C2_06320W_A	4.014908	9.51E-06	3646927
CR_05480W_A	4.010136	3.06E-06	3648238
ARC19	4.0029	0.027188	3640418
C2_05220C_A	3.99362	8.98E-06	3643466
C1_02650W_A	3.984299	0.000219	3636818
C1_10730W_A	3.981139	0.000534	3644404
TRS20	3.980819	0.065346	3640214
OCH1	3.978834	2.23E-05	3641751
PRP42	3.968508	0.006945	3643177
C6_01050W_A	3.968456	0.001061	3644015
CDC60	3.966968	9.48E-08	3640128
FRS1	3.962759	4.19E-06	3640121
FESUR1	3.954206	1.81E-05	3638514
C2_02950W_A	3.951076	2.97E-05	3643012
KAR9	3.948433	0.007894	3636136
C4_02400C_A	3.947534	0.029138	3637851
ECM39	3.946742	0.001746	3641386
ARC1	3.945332	3.52E-07	3645108
CPR3	3.944596	3.26E-05	3635812
MDH1	3.940879	2.37E-05	3639667

RAD16	3.934394	8.16E-07	3636874
SPE2	3.92485	2.14E-06	3644116
C1_13060C_A	3.919966	1.45E-06	3636123
PLD1	3.910105	5.09E-06	3645329
CR_03270W_A	3.902357	0.031414	3645101
BET2	3.888277	0.001161	3639025
C2_01930C_A	3.88676	0.000154	3635793
HHF22	3.883859	0.000952	3647327
C1_00420W_A	3.88146	0.000313	3639286
RPS14B	3.881186	8.38E-06	30515006
VPS51	3.874951	0.034077	3639242
MNN9	3.867741	0.000157	3641735
ARP9	3.864941	0.001084	3639829
CYS4	3.854876	2.83E-06	3645429
C6_00110C_A	3.854356	0.007826	3647896
RAD51	3.854121	0.000704	3644944
ERG8	3.854108	0.000439	3635640
CR_01180W_A	3.852062	0.000702	3640427
C7_00520W_A	3.84992	5.05E-06	3638001
C1_08040W_A	3.846335	0.011069	30515028
C2_10470C_A	3.843554	0.000758	3641196
CR_02260C_A	3.838089	0.013232	3644912
PIL1	3.832705	8.55E-07	3644769
C2_00870W_A	3.831519	0.008803	3638804
PDS5	3.82787	0.000785	3642341
TLO9	3.822263	0.000289	3648051
BUD7	3.81884	3.48E-06	3647228
YMC1	3.817161	0.00075	3644474
C4_05850C_A	3.812608	0.001702	3641791
CDC73	3.812538	0.000185	3636830
GOR1	3.809375	0.000922	3636836
C6_02530C_A	3.807826	0.032127	3639175
MMD1	3.783974	0.000174	30515015
RPP0	3.777615	1.76E-06	3638023
ERG9	3.776704	7.74E-05	3643913
PIKA	3.764413	3.17E-05	3643765
BTS1	3.763734	0.008004	3646982
C6_00340C_A	3.763654	9.52E-05	3646074
HOC1	3.759598	0.000192	3641605
CR_01260W_A	3.751987	0.001378	3640402
AAT21	3.746138	5.4E-06	3647241
RFC1	3.745851	1.05E-05	3645582
MP65	3.740646	2.8E-06	3643951
HXT5	3.740353	0.005733	3643209
HIS4	3.73414	1.91E-06	3645025
C2_09620W_A	3.732609	0.000811	3643797
EIF4E	3.730925	4.95E-06	3638981

TIP20	3.730295	6.63E-06	3636457
RTS1	3.728672	4.99E-06	3646557
CR_05900W_A	3.728265	0.015416	3646681
PAN3	3.720838	0.000513	3636464
CR_02410W_A	3.71763	0.001056	3644928
KRS1	3.71744	1.28E-05	3638486
HYU1	3.70941	0.000324	3643024
RPS30	3.68612	1.37E-05	30515390
C3_07630C_A	3.681509	9.58E-05	3638491
ESP1	3.680417	0.010115	3640197
SDH12	3.678591	2.6E-05	3642775
C6_03470W_A	3.676651	0.00023	3640253
MED1	3.674306	0.000936	3637418
RPL13	3.663819	1.01E-06	3636841
C3_05510W_A	3.652406	0.018911	3643672
ZRT2	3.64588	0.001438	3635861
CR_09510C_A	3.644233	0.000288	3647105
C1_14170W_A	3.639191	6.91E-06	3643323
CHS2	3.638366	9.75E-05	3641893
ARP2	3.634778	2.72E-06	3641915
RNR21	3.628318	1.96E-06	3643021
C1_00920W_A	3.628106	0.000224	3639283
C3_00310C_A	3.624626	0.003954	3640523
CHS6	3.622102	0.002325	3641377
C4_06670W_A	3.621136	0.019298	3642951
UBI3	3.612052	3.93E-06	3642934
SIM1	3.610087	5.44E-05	3636185
C5_04900C_A	3.586433	0.001276	3636416
C6_03580W_A	3.583966	0.019164	3640250
HEM13	3.583916	2.23E-05	3647390
GYP1	3.574052	6.51E-05	3641138
C2_09930W_A	3.573321	0.000252	3643824
C4_05340W_A	3.570937	0.002039	3646947
C7_00150W_A	3.567895	1.8E-05	3637973
GLO1	3.566465	0.002838	3639312
FAS1	3.56429	6.11E-05	3641593
C4_04390W_A	3.562975	1.16E-05	30515267
MRPL6	3.562387	0.002181	3639724
YST1	3.560831	9.27E-07	3643678
C7_03260C_A	3.560223	0.010734	3641366
BRN1	3.553454	0.005935	3641815
SLC1	3.545476	0.000564	3638324
C3_04420W_A	3.539542	0.000158	3635180
RPL6	3.535066	5.22E-05	30514975
C5_00340W_A	3.531143	0.011841	3641555
AHP2	3.5244	0.009361	3636977
CR_01430W_A	3.522625	0.040886	3640138

CR_00690C_A	3.50663	0.013286	3640390
CR_05880W_A	3.505061	0.022832	3646702
C1_03540C_A	3.501426	0.043845	3636920
YHM2	3.494803	0.000152	3647137
C3_05010C_A	3.489422	0.003179	3635169
TOM6	3.489247	0.044304	3636596
MAK32	3.488805	0.043491	3645411
C5_02050W_A	3.485265	1.73E-05	3643768
URA4	3.477022	0.000642	3642632
C3_04080W_A	3.474759	5.3E-05	30515200
C5_04770W_A	3.472577	0.011179	3636444
DPP3	3.471498	0.023173	3636942
CR_03530W_A	3.470014	1.74E-05	3643204
C1_02510W_A	3.46725	0.01203	3636796
RCK2	3.464862	5.7E-06	3643507
CSH1	3.464282	0.002965	3646427
C2_04990W_A	3.460822	0.044185	3643445
ALA1	3.458746	3.84E-05	3640285
C2_07190C_A	3.454371	0.000493	3643513
CMK2	3.452694	0.000694	3643961
C1_01010W_A	3.450756	0.001653	3640181
YBP1	3.450135	0.000115	3636187
MAC1	3.449067	0.001126	3637985
ERG5	3.438774	1.96E-06	3641400
C3_04460W_A	3.429521	0.000793	3635208
VCX1	3.425645	0.006577	3646508
MDL1	3.40938	9.13E-05	3640146
C2_04160W_A	3.401044	0.013342	3637331
CR_05140W_A	3.392283	0.020198	3646400
TIF5	3.39056	0.000102	3637765
C1_01000C_A	3.385295	0.014793	3640180
ERD1	3.384828	0.044082	3640723
CR_03460W_A	3.38104	0.001835	3643213
COQ5	3.37918	0.000566	3643416
GAL4	3.379079	1.29E-05	3641244
ACO1	3.369962	0.000176	3642090
CR_05450C_A	3.36932	0.014495	3648236
C7_03160W_A	3.359386	0.000146	3641345
C2_05170W_A	3.355194	0.013052	3643462
CR_02690W_A	3.352506	0.018285	3642574
C3_02390W_A	3.351438	0.013611	3636615
RPL37B	3.350947	7.21E-05	30515053
CYT2	3.341647	0.031568	3635671
CR_09590W_A	3.340659	0.007908	3647095
TUF1	3.339475	7.62E-05	3639360
DPS1-1	3.33786	2.05E-05	3645134
C2_03910C_A	3.33051	0.001674	3637426

GDA1	3.319883	2.21E-05	3641762
ZCF39	3.313445	4.81E-05	3639037
ATP20	3.310306	0.001279	3647602
C1_03440C_A	3.302297	0.00054	3636908
CR_09070C_A	3.302244	0.000652	3641926
C5_02110W_A	3.300633	0.000189	3637732
RPS13	3.299722	4.5E-05	30515328
CHS7	3.299517	0.003065	3639869
C1_07100C_A	3.297843	0.017232	3639487
APS3	3.29633	0.016006	3646491
RPS5	3.294639	5.6E-06	3637700
C1_09470C_A	3.28622	0.001167	3634761
C2_10830W_A	3.285146	0.102849	30515170
RBD1	3.284223	0.000227	3642018
CTA24	3.283995	2.53E-05	3636475
ERG27	3.28323	0.00202	3640432
FGR38	3.27623	0.005153	3645412
SUN41	3.274505	2.72E-05	3647067
OPT6	3.274287	0.004974	3635603
CR_02570C_A	3.26805	0.017368	3647685
GPI7	3.264667	0.017845	3647672
ARL3	3.261618	0.004607	3644397
IRR1	3.261295	0.000897	3643305
C3_07450C_A	3.259387	0.035763	3638481
C1_08490W_A	3.258776	0.000147	3646492
CR_06520C_A	3.257787	0.016991	3645263
CDC13	3.249459	0.006258	3639293
DNA2	3.247889	0.003701	3646072
C2_04370W_A	3.246169	0.000175	3645460
CR_02770C_A	3.245864	0.004678	3642581
C5_00750C_A	3.240642	0.000171	3644121
C4_04330C_A	3.239013	4.79E-05	3646840
TRP2	3.235336	2.97E-05	3640079
MDH1-3	3.232853	0.000983	3641197
C4_00590C_A	3.229378	7.22E-05	3645772
RPP2B	3.223054	1.95E-05	3635195
C7_00230W_A	3.221883	0.036493	3637982
C1_08780W_A	3.217749	0.000272	3634789
RPL2	3.215534	1.51E-05	30515049
C1_02780W_A	3.214981	0.004538	3636871
C6_01370W_A	3.209379	0.002294	3641607
YSA1	3.198908	0.011735	3638516
PHB1	3.198616	0.001307	3645641
ANP1	3.194466	0.001802	3643917
C3_03840C_A	3.19241	0.002794	3645628
RPL5	3.185517	1.43E-05	3637057
C1_07440W_A	3.180409	0.000189	3644482

C5_00280C_A	3.180361	0.003566	3641581
C3_01140W_A	3.174044	0.013367	3639902
C2_06920C_A	3.169995	0.025562	3643524
PHO88	3.168647	0.001773	3641898
C1_04970W_A	3.167566	1.03E-05	3647528
C3_04630W_A	3.166121	0.015865	3635189
C1_02370C_A	3.165056	0.018667	3644576
RPB11	3.159856	0.045991	30514957
EFT2	3.157148	3.62E-05	3642998
C2_07980W_A	3.153167	0.024819	3642312
PGA54	3.151548	0.00237	3637929
C1_13280C_A	3.147795	0.007244	3636145
RPS4A	3.134988	2.14E-05	3641247
C7_03050W_A	3.133759	0.012496	3641378
PNP1	3.133063	0.000501	3638426
C1_01360C_A	3.1301	0.000214	3640191
C1_09750W_A	3.129407	0.002794	3634822
MCI4	3.129036	0.002166	3640118
C1_09430W_A	3.119536	0.000202	3634855
CR_08130W_A	3.116227	0.002762	3642070
CLA4	3.113638	0.001011	3634878
HOG1	3.112493	1.12E-05	3637393
CPY1	3.103897	0.000111	3646721
ALG11	3.102304	0.026254	3645871
C5_04710W_A	3.101258	0.017294	3636517
YKU80	3.097826	0.016507	3645138
C4_00330C_A	3.097421	0.000853	30515227
C4_02470C_A	3.095738	0.00104	3637869
C5_02750C_A	3.095547	0.001735	3637740
FEN12	3.090042	0.002333	3637404
PSY2	3.089514	0.008908	3644567
C1_04580C_A	3.087543	0.014262	3646132
C2_05050C_A	3.085639	0.01156	3643450
C3_04130W_A	3.083076	0.000296	3635283
C6_04060W_A	3.082914	0.000896	3647828
C1_06200W_A	3.079636	0.004152	3648300
ORC3	3.077169	0.006489	3645639
C1_00830W_A	3.076858	0.046656	3639344
PXA2	3.07407	0.000485	3642055
GCN4	3.072249	0.000166	3643823
GLR1	3.07095	0.000356	3647061
C2_07080C_A	3.06278	0.028074	3643502
YPD1	3.062441	0.00178	3644458
C2_03700W_A	3.062381	0.025476	3637375
EMP70	3.059971	0.000249	3637919
C4_05640C_A	3.056188	0.033158	3641813
COX6	3.05549	5.24E-05	30515099

GPD2	3.051862	7.42E-05	3644563
CR_09990W_A	3.046132	0.002172	3638930
AXL2	3.042416	0.001236	3641184
SRO77	3.041615	1.94E-05	3646088
RPL32	3.039298	0.000363	30515335
RPL11	3.037509	0.000256	3643489
ABP1	3.034901	7.26E-05	3637875
USO1	3.032723	2.02E-05	3648280
SEC4	3.028425	7.41E-05	3640119
CR_02620C_A	3.026612	0.004176	3642567
TLO1	3.025507	0.002585	3639677
MDM10	3.023897	0.016091	3647778
C1_05720W_A	3.022764	3.41E-05	30515000
HSP12	3.022458	0.000521	3643745
CLB2	3.019298	0.001767	3635840
ADH2	3.018707	0.000223	3640751
C3_00790W_A	3.015354	0.019041	3648186
C6_00270W_A	3.013582	0.012242	3646066
C3_02570W_A	3.011199	0.004957	3638375
RPL25	3.008792	0.000185	30515338
CTF18	3.005916	0.004424	3640431
HYS2	3.004714	0.004838	3636441
C3_06690C_A	3.000863	0.028333	3642256
C2_10230W_A	2.99539	0.007917	3643925
PEX3	2.995247	0.006309	3644484
URA5	2.98942	0.013176	3640087
FAA21	2.978081	0.003097	3638418
RNA1	2.971207	0.000328	3636595
ASC1	2.969927	0.000123	3645907
PST1	2.969469	0.008348	3643520
CR_01700C_A	2.969384	0.003092	3640129
SWC4	2.968453	0.002786	3639679
CR_08980C_A	2.965952	0.005488	3641890
CR_08900C_A	2.964858	0.003875	3641908
GUA1	2.963212	0.000514	3634763
SNO1	2.956181	0.004281	3636812
NOP10	2.945684	0.007427	30515434
FGR50	2.944875	0.017156	3644859
RPS6A	2.944435	5.3E-05	3635701
CR_07570W_A	2.94052	0.013758	3648004
XOG1	2.930666	0.001307	3636837
ECM15	2.930467	0.006505	30515218
C1_01910W_A	2.924533	0.000639	3645425
FAD3	2.9222	0.002102	3636124
C4_01020C_A	2.914032	0.000186	3635606
C1_07690C_A	2.913556	0.000306	3641767
C5_03400C_A	2.912537	0.03425	30515308

C3_07770C_A	2.909482	0.005326	3647457
SWI4	2.903166	0.002104	3645408
C1_11120C_A	2.901723	0.001184	3644412
CR_08450C_A	2.900997	0.021335	3642133
C1_10110W_A	2.89215	0.022286	3634778
MSK1	2.889795	0.016449	3637032
VPS52	2.888671	0.006335	3638996
LTP1	2.887916	0.006375	3640760
CR_07780W_A	2.885864	0.001918	3647352
YVC1	2.879651	0.000504	3642303
LCB2	2.872639	0.000553	3636181
MRP2	2.870864	0.004438	3636498
TRP99	2.869413	0.01015	3642494
ROD1	2.865357	0.000116	3635763
LIP5	2.862835	0.042246	3641356
C5_05440C_A	2.862329	0.001278	3636431
ALT1	2.860443	0.000823	3638307
OCA1	2.855923	0.037312	3643929
C1_07050C_A	2.850383	0.00309	3639532
PGA33	2.84716	0.014685	3637350
RAD6	2.840116	0.003416	3643143
ADE4	2.834716	0.000236	3641769
SPC98	2.834469	0.017144	3640113
RPL16A	2.833363	7.18E-05	3639338
CR_04020C_A	2.825409	0.025621	3640969
YCG1	2.819095	0.013858	3636639
LEA1	2.818382	0.028864	3641807
IFR2	2.811885	0.000302	3645100
UBA1	2.81018	0.000476	3642286
C4_05860W_A	2.806124	0.023216	3641792
SSO2	2.802926	0.000612	3643809
C1_10090C_A	2.801757	0.000996	3634817
RHR2	2.794367	0.000242	3640522
C2_01900C_A	2.794286	0.001793	3635789
C1_08110W_A	2.790388	0.000147	3640716
TFC4	2.787815	0.006499	3638420
C6_02410W_A	2.777775	0.000745	3645884
RHD1	2.77686	0.005072	3647531
C3_03080W_A	2.77523	0.029236	3638397
C3_02190C_A	2.770736	0.007063	3636642
PGA52	2.769881	0.000228	3647908
CAT2	2.769227	0.000447	3635659
C7_03840W_A	2.76771	0.012631	3643146
C2_07100W_A	2.767164	0.002654	3643504
C1_12760W_A	2.764793	0.005764	3646817
RPL3	2.763807	0.000147	3647719
C2_01170C_A	2.760919	0.001431	3638860

C1_11100W_A	2.751881	0.029872	3644414
YPT72	2.751879	0.017687	3643523
CR_05860W_A	2.751309	0.004644	3646703
CHT2	2.750528	0.008302	3636523
CNB1	2.749629	0.017617	3636463
ERG4	2.745778	0.00048	3640511
C7_02470C_A	2.743875	0.013961	3636945
C1_02240W_A	2.742696	0.000311	3644597
C7_00210C_A	2.741526	0.002694	3637983
FUM11	2.732773	0.01768	3641016
CR_06620W_A	2.732739	0.006852	3646631
ARF2	2.732724	0.001335	3635271
TOS4	2.732464	0.007509	3644548
C7_00140C_A	2.727499	0.006982	3637974
C5_02400W_A	2.725647	0.001948	3637724
GCV1	2.721829	0.011369	3639172
ARA1	2.712856	0.006197	3642331
C2_10700C_A	2.710234	0.005354	3641207
C2_00880W_A	2.701544	0.036035	3638897
C2_02170W_A	2.69515	0.00446	3635751
C5_01420W_A	2.694459	0.009136	3647055
CCT2	2.679026	0.000372	3643815
C3_05550C_A	2.678097	0.018841	3643659
C5_01440C_A	2.677358	0.074574	3647043
RPN7	2.673313	0.000313	3641909
FLC2	2.671508	0.000218	3647962
C1_02830W_A	2.664035	0.026776	3636876
C4_00230W_A	2.660304	0.032086	3645034
C1_14090W_A	2.660163	0.005376	30515079
UAP1	2.659047	0.000735	3637769
C5_00610C_A	2.654349	0.038926	3641585
C4_03730C_A	2.650619	0.027529	3645358
C4_00780C_A	2.646322	0.000724	3645759
C2_01390W_A	2.645754	0.000522	3635726
OBPA	2.645577	0.00024	3643764
TIM44	2.644263	0.00042	3640529
C5_03140C_A	2.64047	0.000266	3637798
FUM12	2.638742	0.000749	3638489
CCT5	2.637196	0.00025	3643478
C5_01540W_A	2.636122	0.000529	30515301
VPS8	2.635617	0.02588	3640534
C7_01930C_A	2.635527	0.026396	3637027
RPL15A	2.630417	0.00025	3641025
BUL1	2.626518	0.021657	3640734
TIM8	2.626236	0.039939	3647469
C1_14050C_A	2.625731	0.004098	3643343
GCF1	2.619019	0.001282	3646498

RPS21	2.613645	0.000366	3640242
C1_06670W_A	2.613452	0.004391	3639541
C1_06890C_A	2.610809	0.000385	30515014
PTC7	2.608861	0.001049	3645000
C2_08650W_A	2.608056	0.008269	3643908
CR_10020C_A	2.60732	0.029973	3639032
RPS18	2.606218	0.000908	3637953
C2_05590C_A	2.605053	0.015229	3645564
ZCF32	2.603666	0.043544	3635213
ADE12	2.601634	0.000292	3634869
C4_00410W_A	2.600207	0.031554	3645019
C1_00320W_A	2.592532	0.029481	3639297
CR_04330W_A	2.591602	0.029425	3640997
C7_01350C_A	2.59116	0.028647	3645899
SRV2	2.581991	0.000217	3640938
C4_04720W_A	2.580818	0.004417	3641137
C1_05390C_A	2.579229	0.006953	3642479
C2_02870W_A	2.576386	0.028711	3642972
SCL1	2.57528	0.000863	3640510
PTC6	2.568067	0.032297	3647351
C4_07240W_A	2.56766	0.018463	3642945
C1_11250W_A	2.564545	0.012921	3644556
CCC1	2.564029	0.041174	3645645
C6_02370C_A	2.561854	0.001456	3645882
C2_09600C_A	2.559431	0.023571	3643799
RAV2	2.555637	0.04696	3639252
POB3	2.553709	0.000677	3635827
YKT6	2.551705	0.003424	3636863
SUC1	2.549436	0.02722	3641916
C6_00190W_A	2.548246	0.01307	3646080
C1_10460W_A	2.544491	0.008158	3647183
PPH21	2.543331	0.001733	3636665
C6_03310W_A	2.541668	0.018789	3639197
LHP1	2.541122	0.003245	3646665
CR_01820W_A	2.541014	0.001526	3640164
RPL14	2.540898	0.001299	30515072
VAN1	2.53966	0.000855	3638527
GUS1	2.536409	0.001325	3638029
NTH1	2.533743	0.001622	3639665
COX4	2.533335	0.000194	3635737
CR_01380W_A	2.532678	0.008337	3640131
PKH3	2.524895	0.001637	3646075
C2_08270C_A	2.524422	0.009374	3642347
C2_07610C_A	2.521122	0.001171	3645195
C7_01600W_A	2.5145	0.001225	3637036
CR_01050C_A	2.512217	0.004959	3640384
SES1	2.510261	0.001967	3638415

ALG2	2.510198	0.010958	3647832
C5_00050W_A	2.502538	0.024699	3647875
RPD31	2.498386	0.00171	3638520
C2_01690W_A	2.495924	0.002746	3635728
C2_07440C_A	2.49306	0.026509	3645200
AHA1	2.485344	0.006575	3638933
VMA4	2.485243	0.001174	3640111
CMD1	2.483353	0.002551	3646520
TFS1	2.482973	0.005606	3642655
RPS21B	2.482159	0.001142	30514961
CR_09640C_A	2.481152	0.018374	30515456
HEM2	2.476078	0.005086	3637428
C3_00050C_A	2.475748	0.023432	3640570
C6_00200C_A	2.474936	0.011036	3646082
DBF4	2.47255	0.014588	3641388
CHC1	2.469867	0.001166	3647552
RPL23A	2.469348	0.004156	3647558
C1_06190C_A	2.466594	0.001915	3648391
C6_01420C_A	2.466169	0.011151	3641602
TPO3	2.456545	0.013236	3634790
C1_02270C_A	2.454121	0.021181	3644566
C7_03830C_A	2.452941	0.138018	3643147
C3_00830C_A	2.451268	0.007387	3647757
ARO7	2.450556	0.04051	3645337
C7_04280C_A	2.449145	0.012615	3643173
C3_06860C_A	2.446729	0.018356	3638541
TAF14	2.443815	0.00175	3637357
C5_01890W_A	2.443393	0.009792	3643750
C2_04490W_A	2.438664	0.004531	3645463
C6_03820C_A	2.431458	0.013715	3640288
C4_01780C_A	2.42938	0.024024	3635582
SEC3	2.427876	0.006339	3642806
RPL27A	2.42591	0.003279	30515062
DBP5	2.425747	0.000557	3636564
CR_07370W_A	2.425181	0.044665	3646053
RPL4B	2.422534	0.000834	3643347
CR_08350W_A	2.419772	0.001494	3642082
SDH2	2.417778	0.004901	3646397
C1_01900C_A	2.417169	0.015114	3645426
CR_04230W_A	2.415086	0.009773	3640944
C3_06800C_A	2.412463	0.017071	3638497
SMC4	2.408426	0.008949	3641578
BEM2	2.40799	0.000647	3637044
HAT2	2.4068	0.005846	3645973
TIF	2.406503	0.000532	3640190
HPC2	2.402855	0.009509	3636188
C2_07810W_A	2.402258	0.009548	3642324

C1_04980C_A	2.400842	0.01056	3647529
RPS20	2.398222	0.005194	3642071
CR_00490W_A	2.397523	0.002322	3639723
C3_01280W_A	2.396025	0.023296	3636623
C6_02940C_A	2.395941	0.005395	3639187
C7_00410C_A	2.392904	0.040946	3638033
SPO75	2.392502	0.011521	3645364
SEC10	2.388256	0.002632	3642961
C4_06020C_A	2.388194	0.006648	3646519
C2_05580W_A	2.386829	0.00179	3645563
C6_01950C_A	2.385505	0.006053	3644558
PKH2	2.38509	0.000585	3642058
HGT7	2.384254	0.00168	3638876
URA3	2.381224	0.013438	3636649
CYP5	2.374752	0.000375	3642257
C3_01210C_A	2.373884	0.007227	3636612
ERG20	2.372683	0.003382	3645440
ALG9	2.372306	0.017539	3644457
C1_12660W_A	2.370789	0.041652	3646835
AHP1	2.366512	0.032549	3637850
PHB2	2.366418	0.005945	3635238
C6_02680W_A	2.365336	0.022022	3639226
ERF1	2.363412	0.002871	3643455
C2_00830C_A	2.360968	0.005531	3638808
C4_02780W_A	2.360934	0.001235	3637902
C5_03980W_A	2.356079	0.00176	3647442
C3_03760W_A	2.351623	0.010457	3645638
C5_01350W_A	2.350552	0.010314	3642640
CR_10290C_A	2.347154	0.002646	3638935
AKR1	2.34715	0.005068	3636166
TIM21	2.347073	0.014822	3644573
C3_07740W_A	2.345201	0.002226	3638453
IDP2	2.340606	0.013762	3644934
COQ6	2.340424	0.003474	3636878
C2_02840C_A	2.334785	0.023516	3642976
C1_02490C_A	2.330318	0.042423	3636853
YCS4	2.329959	0.004021	3645196
CBP1	2.316461	0.019153	3641920
C1_11920W_A	2.312392	0.031676	3642003
VPS33	2.308513	0.012914	3647477
DUR1,2	2.306728	0.022933	3644771
TCP1	2.305661	0.000655	3646499
TYS1	2.303628	0.00222	3637870
C3_00170C_A	2.303587	0.040103	3640589
ENT3	2.300973	0.005094	3635813
UBC15	2.300738	0.047456	3641260
HAC1	2.297698	0.00149	3639849

IPL1	2.296144	0.110178	3645876
CR_03400W_A	2.293458	0.006844	3645137
VAM3	2.289479	0.205181	3635146
CHS5	2.288634	0.001886	3637333
SYS3	2.287636	0.003454	3645119
PEX7	2.28584	0.011722	3644027
DAO2	2.285758	0.003681	3646797
NHX1	2.284051	0.00322	3647141
C1_05900W_A	2.275843	0.016828	3639866
CSP37	2.275821	0.007898	3640098
C1_13260W_A	2.273766	0.030325	30515074
CR_10170C_A	2.273543	0.004839	3638916
C1_07840W_A	2.272855	0.012887	3640737
C7_01270C_A	2.269382	0.045222	3645908
C2_09650W_A	2.263158	0.003133	3643794
CR_08640C_A	2.261688	0.003828	3642112
RPS23A	2.243322	0.001523	3639515
SFL2	2.241545	0.035778	3636434
YRB1	2.238032	0.002611	3639663
C2_08380C_A	2.237885	0.11074	3648253
C6_02490C_A	2.235229	0.04512	3639171
MKK2	2.234546	0.00345	3645580
RPN2	2.234425	0.005785	3642027
SAL6	2.233342	0.000869	3640289
RPG1A	2.232721	0.002734	3646816
SSC1	2.231566	0.00164	3645232
ATP16	2.230311	0.001689	3638963
UBC4	2.225201	0.0093	3638927
FBA1	2.223999	0.004619	3635585
RPN1	2.222136	0.005525	3636147
C2_00350W_A	2.220847	0.025373	3638827
MIR1	2.219877	0.002267	3634782
UBP13	2.21739	0.003592	3638879
PEP3	2.216753	0.049622	3639267
C4_03320W_A	2.215545	0.027965	3648092
C1_08050W_A	2.204281	0.011911	3640709
ADE2	2.203822	0.001658	3635259
C2_03900C_A	2.200039	0.009304	3637395
C2_03360W_A	2.198276	0.002575	3637389
C7_02560W_A	2.197843	0.016571	3637016
C3_03410C_A	2.196789	0.011949	3638353
C1_09810W_A	2.193573	0.018562	3634845
C5_01980C_A	2.191506	0.049925	3643775
KEL1	2.1879	0.002409	3639318
NUP159	2.178103	0.005022	3647458
UGP1	2.177318	0.007928	3648144
C4_00700C_A	2.175461	0.019824	3645751

PRE2	2.175137	0.005044	3643490
MUQ1	2.17408	0.020719	3638898
CYC1	2.170444	0.006109	3643937
MCA1	2.170268	0.001499	3635233
GYP2	2.166272	0.00723	3641780
C1_09570W_A	2.164179	0.020453	3634887
C6_02810C_A	2.163822	0.011629	3639248
C1_09360C_A	2.163056	0.003513	3634807
C2_06110W_A	2.162847	0.025145	3644094
LRG1	2.160149	0.003254	3639727
SGT1	2.160056	0.001168	3644068
ADE13	2.156927	0.002715	3644863
ULP1	2.155375	0.033899	3638314
MNT2	2.155245	0.015543	3636583
ERG251	2.154595	0.002513	3635716
ATM1	2.149868	0.01438	3646284
NIP1	2.147592	0.003482	3635618
RPN6	2.144733	0.02105	3645352
CR_10590W_A	2.141792	0.027775	3639003
VPS35	2.136072	0.014042	3645567
PPZ1	2.132447	0.030524	3645265
MRE11	2.129371	0.032766	3645898
C7_00490C_A	2.129084	0.020392	3637986
ACS1	2.128117	0.027103	3643941
CR_05970C_A	2.127733	0.014806	3642502
RTF1	2.118689	0.004732	3637964
C1_13720W_A	2.116922	0.014356	3636134
URA6	2.114616	0.007118	3647988
MED16	2.114154	0.026052	3646725
RPL12	2.113038	0.007765	3636659
CR_08050C_A	2.11169	0.01691	3644133
LPD1	2.10678	0.007038	3646037
C6_04250W_A	2.102672	0.005866	30515345
HDA1	2.102151	0.016209	3640154
GDB1	2.101875	0.026444	3647409
C1_12430W_A	2.099808	0.024383	3642013
C4_03110W_A	2.098832	0.004465	3637924
C4_05810W_A	2.097446	0.008833	3641786
MNT3	2.08965	0.0399	3648123
C2_02930C_A	2.088426	0.013675	3643015
MSH3	2.085688	0.043609	3643889
RIM20	2.08492	0.008956	3634809
GUT2	2.081602	0.005759	3642921
C1_05780W_A	2.080705	0.008786	3639888
UBP1	2.079237	0.00972	3641760
ADP1	2.077037	0.021088	3642458
NPL3	2.073631	0.013168	3643310

C1_02320C_A	2.069717	0.009633	3644572
FCY23	2.067808	0.010073	3647681
CR_09110C_A	2.067208	0.046555	3641929
C1_03790C_A	2.066293	0.011503	3644682
MID1	2.063795	0.040614	3647441
PHO85	2.062581	0.009392	3646148
C2_10460C_A	2.061333	0.009214	3641195
C2_04400W_A	2.059212	0.00505	3645457
C6_02450W_A	2.055757	0.008441	3639167
CR_10600C_A	2.055218	0.008644	3639004
APA2	2.050799	0.02727	3639234
C1_08690W_A	2.050052	0.041311	3634934
C2_06850W_A	2.0475	0.115864	30515142
RPS22A	2.044089	0.010647	3639551
CR_05890C_A	2.042655	0.036471	3646682
C3_00660W_A	2.042003	0.031517	3640538
FAD2	2.039131	0.005348	3644053
CIT1	2.038346	0.017516	3643201
MRS7	2.037987	0.007178	3640187
SRP101	2.037431	0.010448	3636513
C4_03130W_A	2.03585	0.013405	3637921
BIG1	2.035643	0.043422	3644434
IST2	2.034608	0.043932	3646662
C1_12830C_A	2.028566	0.01781	3636176
C1_10620W_A	2.023659	0.019587	3647682
PGA14	2.023413	0.005138	3641560
RPC53	2.018355	0.035244	3637898
RPB8	2.016928	0.044401	3647301
TPI1	2.013905	0.028277	3638482
EXO84	2.008765	0.011763	3644022
PHM5	2.005692	0.011702	3637951
DCK1	2.003436	0.027908	3637383
C7_00250C_A	2.002314	0.043597	3637979
CR_08560C_A	2.00122	0.021759	3642142
CSA1	1.997034	0.039592	3638009
SAS3	1.997032	0.051277	3640073
C1_12170C_A	1.993797	0.078453	3642039
C1_06120C_A	1.991532	0.467272	3639850
GBP2	1.990402	0.005974	3637992
SPP1	1.983878	0.053535	3636897
C1_12920C_A	1.983175	0.085016	3636155
MNN12	1.980729	0.043113	3634904
VTC3	1.979989	0.014407	3643206
C4_03810W_A	1.97923	0.13707	3645350
C5_02900W_A	1.977067	0.065725	3637747
C5_02500C_A	1.976371	0.024326	3637766
C3_01970C_A	1.973617	0.712293	3636597

C6_02580W_A	1.972947	0.05094	3639207
GLT1	1.972256	0.017634	3639519
C5_01940W_A	1.970793	0.02661	3643740
PEX17	1.970265	0.092125	3643886
CR_08170C_A	1.970112	0.088879	3642073
TFA1	1.966222	0.016938	3634850
ADE17	1.966116	0.014176	3641024
ERG7	1.960198	0.018956	3635761
C3_02290W_A	1.959688	0.096495	3636556
C7_02480W_A	1.953599	0.098211	3636944
TRM1	1.950441	0.049212	3640396
C1_11430W_A	1.950424	0.142621	3644541
C3_00640W_A	1.949263	0.019593	3640574
CZF1	1.949246	0.032025	3642915
CR_00890C_A	1.948105	0.37242	30515370
C1_12640W_A	1.94783	0.371519	3646837
C4_06290W_A	1.94743	0.374417	30515283
CR_03860C_A	1.946192	0.049477	3643186
CDC27	1.944563	0.10272	3640405
C3_07480W_A	1.943962	0.060237	3638529
SRD1	1.940965	0.155479	3635764
YCP4	1.934904	0.04452	3648032
MLT1	1.93207	0.010799	3640748
CAT1	1.931898	0.079773	3639495
C3_01570W_A	1.929465	0.139944	3636668
C3_00610W_A	1.927259	0.161752	3640578
C6_00760W_A	1.92717	0.046469	3647080
RPL10	1.92692	0.018463	3636850
C5_00140C_A	1.92479	0.045261	3647873
RAP1	1.924277	0.171362	3643945
RPN8	1.922049	0.016815	3643770
ZUO1	1.921622	0.017257	3637908
MRPL19	1.920251	0.108617	3639497
C4_01120C_A	1.919239	0.111823	3635683
TIM10	1.917329	0.067589	30515247
SEC14	1.917144	0.012225	3641542
C1_09070W_A	1.916203	0.050497	3634773
C5_01140C_A	1.915736	0.026776	3642663
C1_03370W_A	1.914099	0.020721	3636891
CR_01560W_A	1.913774	0.219849	3640075
RPL30	1.913773	0.016813	30515273
RPS24	1.913732	0.028231	3640568
C4_06470W_A	1.913279	0.052683	3642790
SNG3	1.913035	0.14672	3646715
RPC31	1.911653	0.083598	3642577
C6_00560W_A	1.911403	0.028655	3647143
OXR1	1.908248	0.061709	3638372

SLP2	1.906551	0.058381	3644091
C1_07090C_A	1.906275	0.012821	3639488
AGC1	1.906062	0.017688	3636219
C6_03040C_A	1.904627	0.131888	3639269
C1_04620W_A	1.90418	0.056026	3646135
C1_12740W_A	1.899701	0.060418	3646819
C2_05020W_A	1.899465	0.234951	3643447
C1_09610W_A	1.898384	0.05567	3634891
MGM101	1.898008	0.09247	3636820
TOA2	1.893366	0.110882	3635710
PEP1	1.88931	0.022366	3647612
BMT3	1.885721	0.119016	3640439
CR_09050C_A	1.883753	0.324436	3648197
C7_04210C_A	1.883652	0.017602	3643121
CR_09570W_A	1.883105	0.324295	3647098
CR_05960W_A	1.880506	0.403074	30515417
SPC19	1.880192	0.401359	3646423
ZCF22	1.879999	0.406669	3637727
C3_02120W_A	1.879198	0.04577	3636658
RPS28B	1.87898	0.018628	30515349
CR_10510W_A	1.878155	0.0759	3639039
DCR1	1.877216	0.025497	3641164
IML2	1.877211	0.013595	3643302
UME7	1.875075	0.5037	3637918
C3_04100W_A	1.875002	0.505681	30515201
ALS2	1.872231	0.03367	3645724
C1_10820C_A	1.870931	0.028841	3644435
C1_07060C_A	1.869691	0.231593	30515018
C3_07460W_A	1.869036	0.089847	3638480
KGD1	1.867657	0.036575	3647752
MED17	1.867037	0.043041	3636918
HSP60	1.860731	0.023722	3645275
SSN8	1.860307	0.107508	3641718
C4_02160C_A	1.859771	0.048058	3635668
CR_09660W_A	1.856458	0.113292	3647092
AVT4	1.856021	0.0431	3645316
RPL21A	1.856004	0.041883	3637400
C3_04360W_A	1.854117	0.072491	3635276
BCY1	1.853754	0.014608	3638832
C4_01760W_A	1.852585	0.106943	3635584
C7_03010W_A	1.850143	0.010903	3641382
C1_10500W_A	1.848545	0.261861	3647188
LTE1	1.846909	0.068057	3643495
C2_00390C_A	1.845189	0.021437	3638822
C3_00890C_A	1.844239	0.116339	3647753
CMK1	1.843771	0.01654	3635263
C6_04340W_A	1.843372	0.12354	3646291

PRT1	1.842799	0.022874	3636959
ZCF10	1.840599	0.086406	3643485
C6_03600C_A	1.83877	0.733327	3640295
C6_02550W_A	1.83877	0.733378	3639204
MRV8	1.83877	0.733401	3636450
C3_01440C_A	1.83877	0.733491	3636632
C3_04310C_A	1.838579	0.730018	3635145
FIG1	1.838579	0.730225	3644025
C7_02140W_A	1.838579	0.730433	3636996
NUP	1.838579	0.730438	3637041
CR_00350W_A	1.838498	0.047282	3639686
C3_03020W_A	1.838388	0.731568	3638391
CR_01910C_A	1.838388	0.731579	3640095
PGA31	1.838388	0.731634	3641226
C1_11260C_A	1.838388	0.731724	30515051
RBF1	1.838215	0.191089	3639251
C1_04860W_A	1.836662	0.026828	3644784
C2_07790C_A	1.836164	0.344479	3642326
C1_03260W_A	1.836046	0.080153	3636898
RPS25B	1.83553	0.04174	3646972
CR_08520C_A	1.835414	0.349558	30515443
C5_05150C_A	1.835029	0.143467	3636461
C6_02430W_A	1.833034	0.024817	3639163
C2_10720C_A	1.831575	0.051071	3641209
C2_02000W_A	1.829734	0.035942	3635767
PEX1	1.828692	0.083073	3636943
AGE2	1.825543	0.048703	3643810
C1_04560W_A	1.821438	0.192186	3646130
C1_01530C_A	1.820125	0.034898	3640232
SOD2	1.819196	0.219943	3640231
C7_00790W_A	1.818456	0.058842	3637943
C5_02330W_A	1.818	0.147589	3637789
NCP1	1.814661	0.025742	3637839
C2_07910C_A	1.81452	0.437481	30515146
BEM3	1.814197	0.03894	3637885
MTO1	1.813349	0.203302	3640707
C1_08930C_A	1.811888	0.30739	30515031
C3_02850C_A	1.810056	0.222118	3638332
WRS1	1.808958	0.050567	3642060
KEM1	1.808921	0.043263	3636222
ACP1	1.808837	0.068324	3639825
C4_02720C_A	1.808724	0.10278	3637857
C1_02770W_A	1.80834	0.265086	30514973
C2_04170C_A	1.807317	0.033827	30515111
MRS4	1.805861	0.056994	3642306
SAC6	1.804432	0.056845	3639213
NAT2	1.802322	0.044943	3638837

CEF3	1.800732	0.036291	3647040
CR_04460C_A	1.800477	0.216958	3640988
C2_08990C_A	1.800306	0.062362	3645783
HGT5	1.799748	0.086654	3635201
C1_08680C_A	1.79764	0.134854	3634933
C3_04650W_A	1.797614	0.277605	3635192
C6_00260W_A	1.797612	0.028668	3646065
ERB1	1.795799	0.027295	3644702
C3_03820C_A	1.794958	0.078789	3645631
NUO2	1.793752	0.0362	3638381
NMT1	1.792761	0.052712	3635624
C3_01790C_A	1.792022	0.08554	3636587
C1_12810W_A	1.790188	0.151859	3646827
C1_11970C_A	1.79002	0.143457	3642044
ULP3	1.789595	0.081596	3640526
OCA6	1.789522	0.367768	3643336
C4_01830C_A	1.789069	0.205525	3635593
C2_04320W_A	1.78903	0.142853	3648274
COI1	1.788575	0.075372	3640721
C6_01330C_A	1.788493	0.365246	3641643
EMP46	1.786999	0.043101	3645270
SWD3	1.785879	0.228244	3645890
C5_02310C_A	1.784601	0.155208	3637787
CRK1	1.784306	0.071071	3643414
CR_06270W_A	1.782071	0.459977	3644854
CST20	1.78133	0.049781	3637790
C5_01110W_A	1.780815	0.044909	3642623
GTR1	1.77785	0.237654	3643914
HTB1	1.776655	0.104908	3645659
C2_00570W_A	1.775081	0.085278	3638853
PGA49	1.774362	0.189367	3646525
GPX2	1.771391	0.162531	3644009
TLO34	1.770992	0.153988	3648392
CDC53	1.769572	0.047846	3636603
C5_04620C_A	1.769501	0.168297	3636394
C1_06390W_A	1.76941	0.151619	3639558
RIB5	1.768095	0.090353	3636499
C1_06500W_A	1.767872	0.298315	3639550
C1_09940W_A	1.766687	0.298365	30515037
CHS8	1.765573	0.046764	3640532
C1_03430W_A	1.765364	0.168093	3636906
MCU1	1.765333	0.143884	3636169
CR_09620C_A	1.763849	0.105001	6335435
C1_09110W_A	1.762606	0.24283	3634940
C1_05830W_A	1.761823	0.756357	3639883
LDG3	1.761823	0.756384	3637001
C1_05450W_A	1.761823	0.756391	3642490

CR_01350C_A	1.759593	0.039762	3640166
SIT4	1.759538	0.087737	3647993
C1_12480W_A	1.759313	0.549084	30515063
C1_14500C_A	1.759149	0.054713	3643331
POL32	1.756928	0.0883	3639881
NRP1	1.756498	0.176254	3640698
C1_01300W_A	1.756182	0.079789	3640185
PRC3	1.755686	0.084922	3639889
C4_01910W_A	1.75425	0.269731	3635635
PRP3	1.752248	0.100714	3637406
C6_03730C_A	1.752229	0.087542	3640303
PSD2	1.751828	0.048669	3636515
C1_10350C_A	1.751659	0.037335	3634835
RPL82	1.751635	0.043648	3644419
C4_03920W_A	1.748617	0.156053	3648314
C4_01310W_A	1.747217	0.460641	3635604
CR_01550C_A	1.747135	0.153924	3640074
GCR3	1.746674	0.027058	3646485
RIM13	1.746435	0.090795	3636487
URA2	1.743903	0.052539	3645728
CR_06560C_A	1.743797	0.10461	3648367
C5_01090C_A	1.742308	0.089938	3642625
MAD2	1.742088	0.392235	3644696
CIS2	1.741551	0.072571	3639366
ARP4	1.741086	0.092905	3639201
RUB1	1.741005	0.256454	30515194
C1_07010W_A	1.740489	0.338787	3639537
C2_04410W_A	1.739854	0.269673	3645455
CDC5	1.738939	0.179923	3639281
C3_02690C_A	1.738873	0.076704	3638407
TIF11	1.737844	0.052333	3641208
CCT3	1.73554	0.029367	3636458
SWD2	1.734755	0.164777	3646677
C2_08420W_A	1.734293	0.207962	3643897
C4_02430W_A	1.734244	0.105264	3637848
C6_01490C_A	1.733925	0.342727	3641620
RPL42	1.733511	0.07215	30515041
C1_05010C_A	1.733175	0.081471	3647532
TIM54	1.732936	0.049033	3641349
MEC1	1.731212	0.121974	3647733
RSR1	1.730618	0.073521	3640145
C3_06350W_A	1.730153	0.194751	30515215
CR_04120C_A	1.730132	0.174455	3640972
RAD50	1.729574	0.092649	3636594
STN1	1.723093	0.578867	3643901
C2_10630W_A	1.721586	0.148055	3641248
RPL29	1.721191	0.059795	30515048

REX2	1.719131	0.168413	3635783
CDC20	1.7172	0.195326	3644057
SYN8	1.717148	0.215577	3645120
FLO9	1.71697	0.110606	3640581
CR_03690W_A	1.716603	0.070038	3643222
PEX22	1.716301	0.208652	3648103
C3_03340C_A	1.715548	0.221036	30515195
C7_00420C_A	1.715127	0.237371	3638032
SIW14	1.714805	0.264672	3647323
C1_02060W_A	1.711216	0.284246	30514968
C4_01670C_A	1.707199	0.169437	3635711
ARP8	1.705903	0.047265	3640200
C5_02060W_A	1.705741	0.384386	3643748
RPC19	1.705	0.282074	3647693
MRPL8	1.704384	0.074237	3646039
HYM1	1.702127	0.130565	3637355
DAK2	1.702126	0.08514	3634895
C2_03530W_A	1.69863	0.079683	3637341
C4_02590C_A	1.697847	0.099274	3637913
C7_02780W_A	1.696882	0.04356	3641361
C2_10060C_A	1.696493	0.428903	3643948
SMF12	1.693281	0.063044	3643509
C6_04120C_A	1.691959	0.25342	3647974
GPA2	1.691513	0.081938	3636638
CDC55	1.691208	0.040411	3635785
CR_09240C_A	1.690598	0.122406	3641919
DRG1	1.690168	0.068923	3640714
ZDS1	1.687395	0.049188	3641103
URE2	1.686209	0.101372	3647795
C7_03370C_A	1.685043	0.102852	3646720
MRS2	1.684168	0.207088	3640110
RAM2	1.683727	0.142908	3634767
RPS27	1.683568	0.052348	30515429
C2_00400C_A	1.683069	0.10917	3638821
KRE30	1.682502	0.088907	3642311
C4_00170W_A	1.68033	0.038399	3645028
C2_06440C_A	1.680145	0.11575	3646565
CR_02700W_A	1.678987	0.322074	3642575
C7_03990C_A	1.676032	0.13404	3643115
C6_00430C_A	1.675806	0.111678	3646089
CCT6	1.674828	0.032335	3642914
C6_03990C_A	1.672386	0.398077	3640271
CR_02430C_A	1.670386	0.042003	3644924
C6_03620C_A	1.669919	0.193019	3640297
SSZ1	1.669859	0.074942	3641139
RAS1	1.669569	0.040613	3643927
KRE9	1.668514	0.097087	3635183

LCB4	1.668312	0.218758	3642057
SEC1	1.66791	0.050913	3636935
MIA40	1.665089	0.052968	3636866
IMP2	1.662591	0.301119	3642636
ARO1	1.658733	0.060211	3635680
C1_12670C_A	1.658093	0.199931	3646834
C5_02390C_A	1.657633	0.086176	3637795
FGR24	1.656923	0.159545	3643288
C1_08920W_A	1.654582	0.123156	3634910
FAV3	1.651382	0.29405	3647911
MCM1	1.650336	0.083693	3638050
CR_02680W_A	1.650326	0.364229	3642573
ABP2	1.647954	0.443384	3643443
C2_00320W_A	1.647772	0.200818	3638798
C2_05410W_A	1.647707	0.090628	30515120
C3_06710W_A	1.646142	0.123954	3638448
CR_07470W_A	1.644653	0.331335	3646048
PXA1	1.644555	0.045199	3639713
C1_10810W_A	1.643057	0.60053	3644436
C1_12110C_A	1.642612	0.602475	3642054
CR_03020C_A	1.641533	0.267396	3642598
C3_01220W_A	1.641042	0.270181	3636610
C2_04360W_A	1.639936	0.141417	3645461
C1_03960C_A	1.639449	0.178943	3646418
RPP1	1.636479	0.215972	3644681
C3_06110C_A	1.634842	0.0926	3641761
FOL1	1.634772	0.135281	3644123
IFD3	1.631194	0.256225	3640211
SMD3	1.631158	0.336686	3647060
GIS2	1.630014	0.056742	3643744
TOM7	1.627639	0.263095	30515355
C1_06720C_A	1.62554	0.248147	3639509
C1_10320W_A	1.624992	0.291244	3634907
PCK1	1.624373	0.073004	3639701
COX8	1.622553	0.096191	30515130
C1_02160W_A	1.6224	0.288723	3644584
DAM1	1.619116	0.234774	3634820
EDC3	1.616811	0.20901	3646152
CR_03240C_A	1.61549	0.223367	3645126
C6_01200W_A	1.613877	0.531877	3644041
C1_03040W_A	1.613133	0.181822	3636842
GRE3	1.612684	0.217261	3637811
RCE1	1.612676	0.227999	3641104
RTT101	1.612333	0.146461	3639827
C1_05630C_A	1.611764	0.112528	3639840
NGG1	1.611357	0.112326	3636902
C3_03830W_A	1.610868	0.23652	3645630

C4_06170C_A	1.610395	0.146085	3642818
RPL39	1.609757	0.079515	30515109
C3_00850C_A	1.609685	0.115568	3647748
C2_08720W_A	1.607272	0.164504	3643885
IMP1	1.606572	0.319059	3636881
C2_05570C_A	1.604972	0.253293	3645562
C3_00960W_A	1.603408	0.296391	3639853
RPT4	1.6034	0.046129	3640966
C5_02450W_A	1.603329	0.138103	3637729
MED22	1.602949	0.418486	3640217
C5_00040C_A	1.602268	0.054446	3647874
GCV3	1.601818	0.073334	3636194
C3_05150W_A	1.601599	0.156879	3635162
C1_13750C_A	1.601282	0.473619	3636137
CHT3	1.600503	0.540427	3638967
C1_14030W_A	1.600205	0.144134	3643340
C3_05320W_A	1.599143	0.089033	3643648
VPS11	1.598783	0.171295	3646524
C1_06640C_A	1.598577	0.382011	30515010
HRT1	1.5976	0.244969	30515187
RPC40	1.597172	0.098908	3643427
C6_03390W_A	1.595838	0.159937	3639232
CR_01020C_A	1.595075	0.219699	3640420
ETR1	1.594892	0.110002	3640561
KIP2	1.594776	0.41143	3643954
TRR1	1.594755	0.078835	3637778
C7_00920C_A	1.593204	0.215895	3637957
SSP96	1.592623	0.561292	3641401
C7_04240C_A	1.591534	0.082798	3643165
C5_04580C_A	1.590775	0.395487	3636511
C6_02160W_A	1.590554	0.394666	3645889
RAD32	1.590464	0.220404	3637340
C5_02230W_A	1.590277	0.082474	3637708
IPK1	1.58993	0.213346	3648091
GUK1	1.58945	0.24373	3647231
C5_03490C_A	1.589193	0.100167	3646967
C1_13370W_A	1.588907	0.214927	3636216
RPS19A	1.58875	0.13043	30515208
NCB2	1.588149	0.159125	3643028
PGA56	1.58677	0.081192	30515313
C3_06830C_A	1.586416	0.087503	3638543
RAD23	1.586229	0.076742	3635834
C2_00290W_A	1.585452	0.311499	3638801
PKC1	1.585445	0.077663	3635210
C1_00530C_A	1.585328	0.112887	3639368
MXR1	1.584685	0.147375	3638881
SLA1	1.58326	0.107788	3635739

C1_09620C_A	1.581803	0.248644	3634892
TIP41	1.5816	0.243505	3636506
C1_04640W_A	1.578145	0.189616	3644761
RDH54	1.577896	0.227947	3641231
STT4	1.57748	0.078702	3647963
C2_05520W_A	1.576299	0.335224	3643421
MIS12	1.575796	0.114099	3639669
CR_00470W_A	1.572253	0.119351	3639725
C5_00580W_A	1.571526	0.214386	3641588
UBP6	1.571046	0.093119	3639317
MRPL10	1.570786	0.132611	3643446
VPS16	1.57055	0.180893	3646150
C2_06710W_A	1.570487	0.415858	3648020
C1_11890W_A	1.570082	0.222908	3642006
C1_06710W_A	1.56983	0.223027	3639510
PRE10	1.568874	0.08945	3636957
MSU1	1.568763	0.22359	3643919
C3_05410W_A	1.565742	0.060637	3643665
SRB9	1.565181	0.162212	3635845
FUN12	1.562138	0.123527	3640713
GAP1	1.560465	0.121193	3637806
C6_02640C_A	1.560197	0.168914	3639222
RCT1	1.560118	0.099879	3641727
C6_00240C_A	1.55906	0.282385	3646062
GAR1	1.558388	0.104939	3645332
PIN4	1.558324	0.115206	3640101
C3_06990W_A	1.558303	0.439491	3638521
C4_06590W_A	1.55683	0.267886	3642778
SET3	1.555964	0.110577	3643320
DCW1	1.55539	0.090196	3638842
C4_07150W_A	1.555345	0.317836	3642935
REV3	1.55429	0.253175	3641749
HEM4	1.553652	0.431715	3643942
GDS1	1.553629	0.081962	3642626
UME1	1.553366	0.106396	3643101
EFH1	1.553049	0.654867	3646929
SOD4	1.552817	0.450481	3638888
C7_01190W_A	1.550849	0.340066	3645902
C2_10730W_A	1.546751	0.572908	3641210
C2_05080C_A	1.545339	0.290632	3643453
CDC48	1.545101	0.100206	3644440
C6_00080C_A	1.542937	0.342636	3647899
CCT7	1.541878	0.08552	3643734
C1_09350W_A	1.5394	0.171039	3634805
C1_11140W_A	1.537588	0.234436	3644410
ECM4	1.536853	0.095055	3640144
AGO1	1.536833	0.140387	3642828

C1_02000W_A	1.535307	0.192904	3645418
C2_07360W_A	1.534162	0.268795	3645236
C1_00410C_A	1.534026	0.281541	3639287
PGI1	1.533611	0.188137	3644847
CHK1	1.533021	0.133418	3637394
IFD6	1.532564	0.431706	3644703
RIA1	1.529532	0.111406	3644478
C2_02700C_A	1.529176	0.364009	3643005
NGT1	1.526874	0.658545	3640573
C7_03690W_A	1.526814	0.656042	3647862
C3_04350C_A	1.526656	0.657377	3635275
TLO16	1.5264	0.660626	3643137
CBK1	1.525388	0.078334	3634838
CR_05700C_A	1.524434	0.204686	3646701
COX7	1.524293	0.189876	3638339
ARV1	1.523869	0.530239	3635248
CR_10820W_A	1.52301	0.285795	3638958
C1_08390C_A	1.52286	0.176249	3646504
RHO2	1.522605	0.468983	30515143
C2_07030C_A	1.522289	0.253949	3643498
CR_05840W_A	1.522245	0.357583	3646704
TIF35	1.521882	0.104746	3643308
PGA1	1.52147	0.314142	3638980
C3_07330W_A	1.521364	0.55422	3638478
C7_02510W_A	1.521319	0.294422	30515357
C1_13190W_A	1.517774	0.110497	3636163
C4_05820W_A	1.517587	0.36621	30515277
C4_02680C_A	1.516643	0.550695	3637828
SAM50	1.515927	0.329052	3641721
PR26	1.514159	0.106154	3642981
SAM2	1.51289	0.133018	3644538
VPS17	1.511969	0.128503	3640218
C3_04370C_A	1.510332	0.155146	3635172
CR_07840C_A	1.509876	0.278781	3647346
CR_06020W_A	1.50874	0.484213	3644873
C2_01220W_A	1.508153	0.111838	3638856
RPN3	1.506958	0.130737	3636917
MET18	1.505949	0.308216	3636631
TIF34	1.505719	0.135762	3636872
C7_04310C_A	1.505238	0.237939	3643141
C4_04870C_A	1.505058	0.12568	3641160
C3_06050C_A	1.504588	0.196505	3641746
CTA1	1.502483	0.209019	3641631
C4_06960W_A	1.50223	0.459254	3642926
C4_03680C_A	1.501847	0.312937	3645363
CSH3	1.501519	0.205022	3646798
MED5	1.500649	0.197979	3648211

SFI1	1.500506	0.198349	3641385
CR_10260W_A	1.500219	0.371997	3638932
C6_01220C_A	1.499725	0.174337	3644042
C7_03610C_A	1.499575	0.163214	3646241
GDH3	1.497463	0.231638	3648086
C2_03800C_A	1.495575	0.108751	3637401
C3_01910C_A	1.493366	0.286293	30515182
CR_06570C_A	1.492351	0.157153	3648366
C3_01800C_A	1.490198	0.205432	3636586
PHO91	1.489134	0.115701	3644588
RPS9B	1.488111	0.159386	30515104
MRP7	1.487958	0.193717	3643118
GSG1	1.487688	0.394528	3647609
C1_04460C_A	1.486867	0.499971	3646140
C1_13320C_A	1.486608	0.170962	3636149
CR_00380W_A	1.486507	0.490994	3639682
APN2	1.485764	0.286888	3647674
IRS4	1.484595	0.23718	3645650
C4_01650C_A	1.484564	0.282063	3635713
CLN3	1.484551	0.150374	3642624
C6_03540W_A	1.48323	0.188263	3640244
PHO81	1.482919	0.274193	3639662
KIN2	1.481797	0.152619	3639697
YPT1	1.481498	0.124248	3636915
MRPL33	1.481486	0.371298	30515175
CR_04300W_A	1.479365	0.139822	3640995
C1_06400C_A	1.479085	0.173855	3639557
C1_14040W_A	1.477918	0.230641	3643342
C2_04790C_A	1.474337	0.214887	3647779
C2_09810C_A	1.472627	0.391964	3643802
CR_06260W_A	1.471764	0.318904	3644853
DLD1	1.470655	0.312478	3643025
NAP1	1.469828	0.188713	3639714
C3_00730W_A	1.469277	0.247547	3640530
C4_04240C_A	1.469022	0.16291	3646856
MEX67	1.4685	0.161166	3641020
C1_00660C_A	1.468333	0.233074	3639376
CR_05120W_A	1.46819	0.133265	3646402
C5_05510C_A	1.467317	0.835051	3636476
C2_06630C_A	1.467317	0.835139	30515141
KCH1	1.466392	0.282099	3636988
MET8	1.466106	0.302029	3638533
LPT1	1.464883	0.239128	3645212
C3_07110W_A	1.463247	0.301377	3638442
GPI19	1.461356	0.567618	3643471
C1_06420C_A	1.461247	0.281084	3639554
C1_04850C_A	1.460794	0.565051	30514989

GDT1	1.460722	0.222358	3645445
RVB2	1.459918	0.135361	3637055
IST1	1.458548	0.418958	3640587
BUD23	1.458371	0.333815	3642629
C6_03880W_A	1.454434	0.225074	3640293
AMN1	1.453729	0.455858	3635795
MNN11	1.453109	0.370811	3636792
MEP1	1.451524	0.348975	3636553
C2_00630C_A	1.450948	0.644017	3638848
TES1	1.450483	0.521268	3647480
C1_03630W_A	1.449117	0.257759	3636882
CR_06930W_A	1.448816	0.392504	3648382
PDC2	1.448757	0.261087	3634862
MNN15	1.448339	0.345597	3644794
C1_05380C_A	1.448272	0.246258	3642480
ARH2	1.447824	0.457123	3643411
C2_04300C_A	1.446588	0.366823	3648276
C2_02720W_A	1.445223	0.13177	3643002
C5_03690W_A	1.443949	0.503051	3646989
VPS2	1.443434	0.280615	3641546
SMP2	1.443071	0.225231	3635779
C3_03210W_A	1.442962	0.519735	3638315
QDR1	1.441976	0.180301	3640941
C3_07030C_A	1.440965	0.281889	3638517
AVT1	1.440514	0.265049	3646928
LAP3	1.440323	0.356053	3641012
C3_05290C_A	1.439786	0.642625	3635203
ALS3	1.439167	0.138379	3647965
C1_05950C_A	1.438741	0.354527	3639859
C3_00270C_A	1.434904	0.190949	3640527
C1_05660C_A	1.433509	0.177667	3639900
C5_02350C_A	1.432753	0.555299	3637791
C7_01230C_A	1.4303	0.224799	3645906
C4_05630W_A	1.430101	0.268222	3641812
C1_01940C_A	1.42919	0.440371	3645422
C1_05210C_A	1.428434	0.203063	3642465
C1_03460C_A	1.427704	0.391932	3636911
SSN3	1.426904	0.271906	3637353
SAP1	1.424321	0.569326	3640256
C7_01670W_A	1.424279	0.605753	3636982
AGP2	1.42311	0.227162	3635684
CR_07340C_A	1.422332	0.270891	3646056
XKS1	1.421676	0.22006	3648306
C4_02100C_A	1.421428	0.516805	3635641
DDI1	1.4203	0.206846	3643295
C3_06810W_A	1.417834	0.348352	3638545
C5_01190W_A	1.41782	0.273583	3642659

C2_03130W_A	1.415911	0.344066	3642993
C2_10670W_A	1.415786	0.171047	3641251
YOR1	1.415727	0.214433	3643964
C1_03910C_A	1.415653	0.378661	3646412
RAD14	1.415503	0.353957	3636953
C4_00320C_A	1.414837	0.439871	3644999
CWC22	1.414702	0.334552	3643938
C3_07140C_A	1.414541	0.521177	3638439
CR_04720C_A	1.414326	0.153282	3648140
SLF1	1.414065	0.36882	3638500
C1_05220C_A	1.413956	0.600561	3642464
C4_02950C_A	1.413407	0.652423	3637874
C1_09910C_A	1.413245	0.527314	3634798
RBP1	1.413024	0.254657	3647647
VPH1	1.412931	0.119829	3646157
VMA22	1.410785	0.721017	3647086
DAL7	1.410639	0.716547	3636130
C6_00630W_A	1.410583	0.716619	3647135
C6_03070C_A	1.410493	0.71649	3639272
TLO5	1.410493	0.716848	3647926
GLY1	1.410339	0.173344	3647181
C6_02200C_A	1.410291	0.723467	3645863
C2_08750W_A	1.410201	0.723693	3643881
SKI8	1.409773	0.276572	3640294
POL5	1.407513	0.269191	3639254
STR2	1.407465	0.13234	3644689
C6_02420W_A	1.407453	0.268632	3645885
TIM50	1.406134	0.181076	3644530
VRP1	1.405483	0.345061	3642293
PAP1	1.405405	0.245873	3643763
TKL1	1.40513	0.360907	3640767
C7_02540W_A	1.404788	0.366226	3636939
C7_04010W_A	1.403941	0.244336	3643112
MED14	1.401457	0.22995	3637800
GPM1	1.401452	0.255127	3637431
C5_03170C_A	1.400359	0.295727	3637801
C3_04380C_A	1.399892	0.225589	3635173
C6_02090C_A	1.399625	0.231908	6335445
DBF2	1.399541	0.406052	3648101
CR_04750W_A	1.398422	0.471901	30515399
C1_07400C_A	1.398346	0.290425	3644464
C2_07220W_A	1.398328	0.199962	3643483
C3_05420W_A	1.397854	0.29029	3643666
C4_04750W_A	1.397491	0.298321	3641133
CR_09150W_A	1.397462	0.47073	3641880
BMT8	1.397418	0.382825	3637442
YDJ1	1.397311	0.209588	3640939

C1_02390W_A	1.396923	0.285282	3636791
C1_04630C_A	1.395731	0.242807	3644762
CR_01120C_A	1.395227	0.272521	3640434
C3_02200W_A	1.395104	0.852125	30515185
C7_02210W_A	1.395104	0.85221	3636990
KAR5	1.394959	0.851266	3644049
ATO5	1.394959	0.851292	3643652
C6_01130W_A	1.394959	0.851514	3644055
PGA34	1.394959	0.851713	3642579
CR_01920W_A	1.394959	0.851781	3640096
C6_02620C_A	1.394959	0.851852	3639211
C4_05220C_A	1.394603	0.489429	3646155
C1_04600C_A	1.391169	0.207401	3646133
C6_00800C_A	1.391165	0.206452	3647069
CR_00830W_A	1.390988	0.55613	3640412
C1_03690W_A	1.389889	0.257417	3648426
C1_02720W_A	1.389806	0.361452	3636825
C3_07420W_A	1.389232	0.221522	3638485
ECM29	1.388276	0.157432	3638433
CR_09190C_A	1.388264	0.418008	3641885
PHO8	1.388117	0.679016	3647179
C1_13180W_A	1.387146	0.857361	3636162
RFC52	1.387146	0.857368	30514953
C5_05270C_A	1.386231	0.29865	3636496
RPS10	1.384551	0.278702	30515149
PCT1	1.38445	0.224946	3645743
C4_03700W_A	1.383624	0.327062	3645361
C1_08160W_A	1.383471	0.261776	3640733
C2_01230W_A	1.383104	0.492811	3638795
VPS4	1.380471	0.313904	3637703
LKH1	1.379202	0.192304	3647187
C2_05710C_A	1.379151	0.244898	30515127
CR_06780W_A	1.378866	0.263035	3647312
C1_02740C_A	1.378029	0.587555	3636827
SNF4	1.378005	0.22976	3640264
CR_06790C_A	1.377997	0.475216	3647311
CR_07310W_A	1.377306	0.405217	3646058
SHE9	1.377149	0.311653	3642984
C4_03040W_A	1.37589	0.428339	30515253
C4_00500W_A	1.374854	0.250291	3648379
C1_04230W_A	1.37427	0.263341	3644722
MED21	1.371748	0.510602	3639010
C1_04530C_A	1.370337	0.32081	3646149
HLJ1	1.369185	0.33753	3643158
C4_04500C_A	1.368627	0.324258	3641123
C3_03930W_A	1.368368	0.349107	3645657
C2_05960C_A	1.367255	0.639411	3647479

MTW1	1.367079	0.635708	3643783
C2_07200W_A	1.364855	0.55809	3643481
C1_03620C_A	1.364521	0.319319	30514981
C2_02620W_A	1.362394	0.336583	3635838
SUA72	1.357766	0.49743	3648239
C1_08860C_A	1.357234	0.320026	3634921
C3_05120C_A	1.353982	0.334032	3635159
GPI13	1.353263	0.439411	3637424
NUP85	1.352901	0.246746	3635175
C2_02660W_A	1.351601	0.531498	3643009
UGA11	1.350969	0.530325	3637437
THI6	1.350379	0.576841	3638333
RER2	1.347854	0.293281	3636399
C2_08690C_A	1.347724	0.625747	3643888
HGH1	1.347155	0.39174	3635647
C5_03880C_A	1.346071	0.698292	3647222
C4_00210W_A	1.346037	0.698169	3645032
NUP188	1.345528	0.25026	3634858
C2_00820W_A	1.3453	0.316206	3638809
VPS53	1.343724	0.258201	3639319
C6_01560W_A	1.342188	0.611391	3641652
C7_02350C_A	1.341462	0.371232	3636932
TRY5	1.340447	0.597059	3641619
C1_11560C_A	1.34018	0.485427	3645331
C7_00910C_A	1.339758	0.384328	3637958
C6_04370W_A	1.338882	0.419394	3646294
ISN1	1.338854	0.195681	3640196
SCT2	1.338672	0.435089	3643018
C5_03100C_A	1.338636	0.339848	3637704
PRE9	1.338378	0.351354	3638311
ENO1	1.338141	0.355642	3646493
CR_04450C_A	1.337739	0.274503	3640987
C2_08630C_A	1.337431	0.279834	3643910
MRPL3	1.335823	0.304483	3640722
C1_06560W_A	1.335165	0.448126	3639517
GCN20	1.334425	0.251785	3639314
C3_01950C_A	1.333765	0.346183	3636599
DAO1	1.333548	0.477071	3636885
C4_02620C_A	1.331163	0.342434	3637834
C1_11010C_A	1.330378	0.441488	3644421
C4_04130W_A	1.329918	0.418589	3641221
ASR2	1.329497	0.366435	3641907
SUR7	1.32918	0.278486	3641610
C3_02090C_A	1.32897	0.437131	3636661
IDI1	1.328791	0.319703	3637894
SIK1	1.328772	0.369698	3638925
MDL2	1.328575	0.351917	3639256

C1_07990C_A	1.327953	0.424883	3640697
C1_05520W_A	1.326681	0.597051	3642505
C1_03280W_A	1.326475	0.318423	3636901
C1_12280C_A	1.32626	0.591321	3642019
GPI2	1.326225	0.587729	3641011
C3_06260C_A	1.325572	0.383434	3642240
MAL31	1.325313	0.777506	3636420
C4_01950W_A	1.324756	0.318833	3635665
C3_00360W_A	1.324648	0.333336	3640542
SUP35	1.323536	0.329255	3643785
C7_01010W_A	1.32267	0.799067	3638021
FGR14	1.322365	0.264492	3648083
C7_04170W_A	1.321703	0.692935	3643126
C5_03710C_A	1.320234	0.673183	3647240
C3_05400C_A	1.319861	0.682048	3643681
CR_06850C_A	1.319726	0.685268	30515424
PRO1	1.318474	0.257321	3639000
C2_05390C_A	1.317761	0.781188	3643434
NOP1	1.317732	0.352653	3642947
POT1	1.316368	0.328057	3639707
GCD11	1.316143	0.276842	3637755
PIM1	1.315241	0.296827	3640999
C4_06770W_A	1.314223	0.384566	3642920
SGO1	1.313983	0.472075	3643464
CAS5	1.313573	0.280924	3635652
C4_05400C_A	1.312655	0.332833	3646952
NOP15	1.311418	0.336747	3637966
TIM23	1.310756	0.36149	3643777
C1_01400C_A	1.310177	0.347241	3640237
C4_03080W_A	1.307893	0.296726	3637928
NAT5	1.30697	0.464088	3641022
C6_00500C_A	1.306084	0.563318	3647149
C5_03670C_A	1.30404	0.504113	3646987
PRK1	1.302704	0.274695	3640153
RPT5	1.302257	0.302844	3642911
C1_14310W_A	1.302007	0.449866	3643315
C1_10680C_A	1.301509	0.372745	3647678
SEC15	1.300978	0.305662	3646845
PUS4	1.300226	0.344808	3642661
RPL7	1.299336	0.419045	6335362
C3_01820W_A	1.298655	0.439871	3636584
CDC15	1.298652	0.361965	3643459
C1_12900W_A	1.298241	0.459652	3636154
CR_03130W_A	1.297519	0.616419	3645121
C5_02040W_A	1.29724	0.400364	3643769
PHO23	1.295503	0.363544	3643926
IFI3	1.295502	0.724159	3644694

PEA2	1.294793	0.41033	3647673
C4_03300C_A	1.294769	0.79124	30515254
PHO113	1.294501	0.78762	3644946
C2_07700C_A	1.294417	0.787428	3642337
C1_02040C_A	1.294233	0.297296	3644586
GAD1	1.293607	0.473802	3645339
C1_04790W_A	1.293341	0.410824	3644772
C5_00950C_A	1.293064	0.352537	3642653
CR_01950W_A	1.292897	0.40054	3640107
ZCF17	1.292632	0.454786	3640205
C4_04820C_A	1.291766	0.416407	3641165
GAT1	1.291577	0.520701	3641794
NSP1	1.291263	0.380424	3637430
C3_02580C_A	1.291041	0.621964	3638376
RPN5	1.29078	0.274633	3636405
RAD59	1.290443	0.6114	3648281
C4_05180C_A	1.289943	0.319242	3647413
C5_01310W_A	1.289148	0.634874	3642645
TEA1	1.289041	0.384212	3643671
C2_09070C_A	1.286985	0.439816	3644111
C4_00740W_A	1.286214	0.610197	3645764
C4_02670W_A	1.285699	0.498757	3637830
RPS42	1.283952	0.396555	3640195
C5_03550W_A	1.281231	0.27474	3646973
C5_03470C_A	1.280893	0.41266	3646963
C2_05040C_A	1.280039	0.347197	3643449
C1_12470W_A	1.279444	0.747374	3648182
PGA15	1.279312	0.747505	3642781
C3_01120W_A	1.278918	0.557362	3639836
CR_07220C_A	1.278418	0.570535	3646040
C5_04300C_A	1.278089	0.400359	3636456
C6_03150C_A	1.278055	0.447022	3639257
APR1	1.277918	0.385536	3645204
SUI1	1.277517	0.385497	3647730
C6_01300W_A	1.277376	0.54355	3644024
ERG25	1.27702	0.421887	3644933
C1_01490W_A	1.27655	0.349193	3640226
C1_02410C_A	1.273627	0.519693	3636793
CR_10380C_A	1.272862	0.717901	3638953
SIN3	1.269971	0.332221	3639282
C3_02870C_A	1.269466	0.581873	3638334
HOS2	1.269193	0.52956	3640509
CR_00420W_A	1.268315	0.656738	3639728
TRP1	1.267819	0.421318	3639321
C3_07760C_A	1.267458	0.653975	3638450
C4_06740C_A	1.267208	0.367618	3642923
CR_02120C_A	1.266516	0.645951	3640143

C2_01740C_A	1.26078	0.547855	3635733
C7_00780W_A	1.260575	0.404599	3637945
MPH1	1.260016	0.505157	3642817
C7_02370W_A	1.258384	0.384409	3636930
PTH2	1.25837	0.525348	3637709
C1_00470C_A	1.254889	0.458226	3639315
C5_02820C_A	1.254357	0.473928	3637808
C2_03830W_A	1.254349	0.405131	3637399
C2_08620W_A	1.25149	0.50372	3643912
CR_08930C_A	1.25084	0.444793	3641913
MKC1	1.250708	0.400586	3639710
FGR28	1.250526	0.541439	3648315
C4_05010W_A	1.250407	0.431087	3641148
C1_08760W_A	1.250402	0.544471	3634787
GSL1	1.248849	0.534239	3639844
SEC5	1.248124	0.483275	3648178
C1_14520W_A	1.247259	0.384217	3643332
INT1	1.245117	0.477351	3637761
C6_02740W_A	1.244537	0.544774	3639216
CR_06690C_A	1.244389	0.522087	3646624
C1_03990W_A	1.243917	0.572031	3646424
C3_06040W_A	1.243556	0.663941	3641745
SKO1	1.242069	0.425839	3644688
SPT20	1.241635	0.52905	3642492
RAD2	1.241516	0.4973	3645374
C6_02900C_A	1.241266	0.568609	3639240
C1_02470W_A	1.239205	0.678371	3636851
SBP1	1.238815	0.427637	3635280
C4_03150W_A	1.238669	0.431072	3637842
C2_08060W_A	1.237838	0.695973	30515151
GRP2	1.234647	0.408576	3637744
C2_00840W_A	1.234481	0.483867	3638807
AGP3	1.233351	0.569601	3641163
C3_03060W_A	1.233168	0.731213	3638395
C6_00690W_A	1.232792	0.63245	3647075
C7_04110W_A	1.231303	0.497397	3643133
MGE1	1.231159	0.466366	3640133
OPI1	1.231023	0.50332	3635777
C6_01940W_A	1.230837	0.739657	30515337
AMO1	1.230622	0.720264	3642994
PUP3	1.229673	0.497621	3646718
YPT52	1.229319	0.47883	3643346
RPO26	1.228517	0.451106	3646351
C2_03950W_A	1.228259	0.504441	3637420
SET6	1.227864	0.547899	3644590
RPL9B	1.227828	0.478957	3638348
C7_00580C_A	1.227233	0.646888	3637995

HOL1	1.226986	0.6469	3636838
C2_01030W_A	1.226773	0.453929	3638836
C2_07240C_A	1.226327	0.765074	3643486
YIM1	1.225607	0.475432	3637372
NMD5	1.225253	0.422184	3645745
C3_03380W_A	1.225034	0.576451	3638367
HGT14	1.223529	0.586118	3648320
C1_05610W_A	1.222753	0.597168	3639843
CR_10390W_A	1.222068	0.692093	3638954
C4_06690C_A	1.221777	0.495615	30515285
NUP84	1.221665	0.433268	3645351
IQG1	1.221339	0.611929	3637052
RIB4	1.220534	0.505326	30514995
WH11	1.219289	0.485812	30515117
C7_03030W_A	1.218712	0.648069	3641380
C5_01470C_A	1.217706	0.614	3647050
C2_07290W_A	1.216576	0.463131	3643476
ATC1	1.215991	0.559251	3639570
YHB4	1.215773	0.460672	3638999
VTI1	1.215318	0.495912	3638352
YAE1	1.213217	0.684694	3637941
C2_05300C_A	1.212306	0.459974	3643423
VPS41	1.211146	0.607984	3634800
C5_04920C_A	1.211092	0.466771	3636419
PRE8	1.210043	0.458827	3641906
ECM14	1.209619	0.559617	3638392
C4_03560W_A	1.209112	0.537199	3645373
C4_05360C_A	1.208815	0.72476	3646949
C1_01860W_A	1.208579	0.493807	3645430
C1_05750C_A	1.207823	0.484797	3639891
C2_04780W_A	1.207563	0.496025	3647781
DIM1	1.207108	0.600327	3636135
C3_02160C_A	1.206517	0.441094	3636654
C5_03010W_A	1.205547	0.534467	3637819
C1_02030C_A	1.205131	0.72462	3645415
C7_02630W_A	1.20418	0.648362	3647646
C5_02740W_A	1.203544	0.543915	3637739
C7_02690C_A	1.202908	0.660042	3647649
CR_08550W_A	1.201982	0.425519	3642141
TAF60	1.201577	0.538807	3642252
YTM1	1.201204	0.50965	3634765
C5_02660C_A	1.201046	0.575587	3637771
C4_05260W_A	1.20069	0.543433	3648282
GCN2	1.19871	0.549521	3645897
C5_01000C_A	1.198155	0.568774	3642651
C5_03810C_A	1.197951	0.762332	3647229
C7_01570C_A	1.197506	0.760562	3637040

C2_00110W_A	1.197306	0.678213	3647921
C4_03950C_A	1.1948	0.748564	30515263
C4_01560C_A	1.194619	0.546869	3635714
C1_13690C_A	1.194107	0.51303	30515076
STB5	1.192791	0.597116	3640208
FUR1	1.191974	0.537685	3646348
EAF7	1.191929	0.515487	3640973
C6_00890W_A	1.190941	0.530924	3644028
TRM2	1.190921	0.600337	3640235
C1_04910C_A	1.190305	0.566671	3644793
C1_12530C_A	1.189267	0.663075	3647608
C5_00030W_A	1.188789	0.508493	3647879
MSS4	1.187708	0.470235	3647957
CR_02300C_A	1.187324	0.504502	3644938
C2_05760C_A	1.185984	0.583582	3645578
CR_03760W_A	1.185035	0.620478	3643178
ORC4	1.184613	0.579496	3637753
HSP90	1.184459	0.53015	3636951
C1_11370C_A	1.184427	0.6298	3644547
C6_02630C_A	1.184262	0.659447	3639221
C2_09830C_A	1.183975	0.715136	30515161
CR_02210W_A	1.183306	0.634066	3644943
CR_07870W_A	1.183062	0.733274	3644146
CR_09500C_A	1.183027	0.692417	3641897
DTD2	1.182541	0.592889	3638390
C2_10330C_A	1.18059	0.80724	3643943
C1_03950C_A	1.180326	0.807782	3646417
BUD31	1.179599	0.741882	3634796
AYR2	1.178813	0.620019	3639184
RLP24	1.178491	0.553435	3645748
C4_05040W_A	1.178331	0.864991	30515275
SIT1	1.177795	0.53234	3642307
C7_01650W_A	1.17747	0.476308	3636984
RPL40B	1.17729	0.513601	30515232
PUS7	1.176912	0.521322	3643960
C1_01680C_A	1.176831	0.652189	3640198
URA1	1.176443	0.50848	3634819
ROT2	1.176007	0.556798	3641566
C3_01850W_A	1.175686	0.608655	3636565
SNG4	1.175426	0.655657	3646714
C6_01000C_A	1.175371	0.647801	3644048
CR_00570W_A	1.175158	0.553064	3639664
PES1	1.173617	0.568867	3644105
C2_00060C_A	1.173181	0.535571	3647914
C2_05530C_A	1.172945	0.544991	3643422
C3_05110W_A	1.172085	0.712964	3635158
AQY1	1.170757	0.663777	3642587

C1_02700C_A	1.170575	0.663473	30514972
C3_06550C_A	1.17031	0.652209	3642275
AKL1	1.169456	0.521134	3641237
C2_04380C_A	1.16909	0.587185	3645459
TRS33	1.168332	0.68558	3636992
PEX6	1.168325	0.676881	3643436
C1_06030C_A	1.167616	0.779004	3639867
CDC14	1.167603	0.613794	3647132
RAC1	1.167031	0.585723	3639508
C1_03510C_A	1.166931	0.680107	3636916
C3_05090C_A	1.165633	0.606597	3635245
C1_08470W_A	1.165547	0.709365	3646490
ATO1	1.164968	0.800383	3647761
C3_02080W_A	1.164876	0.61884	3636575
C3_01890C_A	1.163162	0.534867	3636561
C7_02340C_A	1.163159	0.591612	3636933
SEN1	1.162605	0.56454	3635255
KIS2	1.161974	0.564696	3636211
PUP1	1.161352	0.522255	3638936
C1_14080W_A	1.160965	0.57643	3643345
MRPS9	1.160737	0.588217	3642064
CR_08430W_A	1.160721	0.565848	3642120
CHS4	1.160199	0.511729	3641726
VPS15	1.159396	0.617023	3644040
COQ4	1.15923	0.607035	3636804
CR_06230W_A	1.159118	0.626138	3644867
C5_05250C_A	1.157983	0.613923	3636472
C1_13840W_A	1.15776	0.526239	3636129
TPK2	1.157513	0.593631	3643482
C5_02990W_A	1.155987	0.782453	3637817
C3_02760C_A	1.155537	0.622214	3638413
C5_00150C_A	1.155444	0.524888	3647872
YDC1	1.154992	0.745085	3642962
C2_07860W_A	1.154436	0.6498	3642320
CR_09490W_A	1.154077	0.609739	3641896
HPD1	1.152871	0.79934	3639239
CR_01570W_A	1.152003	0.764937	3640077
MOB2	1.15198	0.638766	3639381
C2_03780C_A	1.151951	0.585547	3637368
RTA2	1.151805	0.575108	3646567
C1_10200C_A	1.15172	0.655626	3634877
VAC8	1.151351	0.58236	3647410
C2_06160W_A	1.149883	0.798483	3644079
NUP82	1.148983	0.611411	3640261
CR_04580W_A	1.147464	0.658663	3640948
CR_03540W_A	1.147454	0.754848	3643215
C4_05210W_A	1.146367	0.744903	3646154

C2_04130W_A	1.146096	0.656379	3637334
HCR1	1.146042	0.583945	3638952
C6_03740W_A	1.146011	0.860337	3640304
INO4	1.145705	0.7509	30515105
C1_07860W_A	1.14542	0.861385	3640740
ATO9	1.145391	0.860002	3640392
C5_04940W_A	1.144814	0.839065	3636421
C3_04120C_A	1.144322	0.730853	3635282
NAM7	1.144285	0.567511	3641540
CR_03350C_A	1.14169	0.624036	3645142
MNR2	1.139791	0.610275	3645017
C2_10870W_A	1.139221	0.681862	3641234
SSK2	1.138228	0.616757	3641144
ILS1	1.137705	0.607055	3645956
C4_06450W_A	1.13692	0.795948	3642792
GPD1	1.136529	0.609105	3643924
MRR1	1.134551	0.683028	3641741
CR_07020W_A	1.134169	0.648947	3645731
C2_10120W_A	1.133016	0.743695	3643936
C4_03140C_A	1.132801	0.730576	3637843
FGR10	1.131065	0.815365	3641159
C1_08910C_A	1.129968	0.775372	3634926
C4_05330C_A	1.126176	0.730023	3646959
C7_00850W_A	1.125239	0.784099	3638056
HOF1	1.124945	0.870661	3645014
RLI1	1.124276	0.669905	3636888
UGA2	1.124271	0.758128	3645436
RPC11	1.124133	0.78668	3641239
QCR9	1.124034	0.700936	30515251
SPA2	1.122626	0.646611	3646824
C3_02490C_A	1.122293	0.631749	3638351
ASG1	1.121623	0.668238	3647687
C5_04610W_A	1.120893	0.652358	3636393
CR_05100W_A	1.119729	0.753994	3646403
EMC9	1.119506	0.656462	3647918
C4_03780C_A	1.119275	0.669919	3645353
GGA2	1.119184	0.647103	3636928
C5_00250C_A	1.118989	0.814135	3641562
FLC3	1.117399	0.714624	3642322
CR_07120C_A	1.116723	0.885106	3648225
SMT3	1.115869	0.683124	3644550
C3_04190W_A	1.115722	0.915895	30515203
SNU114	1.115597	0.688253	3647798
MSW1	1.115547	0.753475	3637743
NTF2	1.114132	0.702231	30515039
PRE6	1.113172	0.672688	30515395
FAA4	1.112995	0.666686	3638973

C4_06730C_A	1.112151	0.655528	3642924
C1_07570C_A	1.111323	0.814057	3646675
C2_01440C_A	1.111131	0.833449	30515090
C5_01880C_A	1.110798	0.791699	3643751
FCY21	1.110281	0.645944	3643822
CR_03960C_A	1.109217	0.777285	3640963
XUT1	1.10908	0.79431	3642785
C4_03600C_A	1.108881	0.74409	3645366
NIT2	1.108615	0.806001	3641874
DUR35	1.10765	0.926655	3635150
C1_06530C_A	1.107185	0.647328	3639521
C1_10920W_A	1.106621	0.785457	3644433
C3_07850W_A	1.106005	0.726737	3647466
TEL1	1.106004	0.777508	3639263
GRE2	1.104544	0.801754	3648026
RPN10	1.103646	0.712526	3644080
C7_04200C_A	1.102372	0.729031	3643122
CR_10620C_A	1.102285	0.698893	3639006
CR_04140W_A	1.101288	0.711576	3640974
C7_00070C_A	1.101122	0.706362	3638011
GCN5	1.100343	0.740389	3646626
TUP1	1.098508	0.726919	3639369
CR_01790C_A	1.098445	0.784636	3640161
TIF3	1.097911	0.681127	3641646
C3_02770C_A	1.097505	0.756475	3638414
C5_04330W_A	1.097416	0.787601	30515314
CSI2	1.09735	0.770845	3642016
DEM1	1.097071	0.817124	3643200
SPT3	1.096858	0.761319	3638977
C2_03490C_A	1.095637	0.766718	3637346
MRP20	1.09551	0.736738	3640224
PGA59	1.094773	0.726021	3637881
C4_00660W_A	1.094442	0.762994	3645755
C1_09280W_A	1.093982	0.779304	3634829
FDH3	1.093614	0.723187	3638923
C5_00320W_A	1.093588	0.804123	30515291
C1_08340C_A	1.09313	0.838359	3640752
CR_08110W_A	1.091993	0.849124	3642067
RPB4	1.091855	0.76837	3647800
TLO4	1.091233	0.87492	30515080
YEA4	1.09101	0.877379	3643787
C2_01340W_A	1.090526	0.73532	3638844
BZZ1	1.090022	0.782207	3636572
C1_00910W_A	1.089744	0.890684	3639284
C6_01120C_A	1.089339	0.886809	3644054
CR_10180W_A	1.089003	0.888543	3638917
C7_02860C_A	1.087804	0.74866	3641397

NOT3	1.087315	0.76222	3638829
C1_11090C_A	1.087164	0.784952	3644415
C6_00900C_A	1.087152	0.867151	3644029
C6_00490W_A	1.086156	0.822504	3647150
C6_02690C_A	1.085435	0.754397	3639228
C3_01690W_A	1.083664	0.779939	3636604
NOT4	1.083506	0.752346	3645115
PEX14	1.08349	0.797148	3648208
CHS1	1.083312	0.897814	3641364
HHT1	1.083292	0.734503	3638444
C4_06840W_A	1.083098	0.847532	3642913
C1_03920C_A	1.080174	0.881835	3646413
C2_04960C_A	1.078866	0.861111	3643442
C2_02290C_A	1.078705	0.92036	3635807
C5_04960W_A	1.078645	0.7653	3636423
CDC23	1.078084	0.837402	3642111
GLC7	1.07794	0.738583	3647257
LYS142	1.077854	0.836883	3634896
ACF2	1.077211	0.85915	3641613
EHT1	1.076426	0.830536	3636894
CR_04390C_A	1.076305	0.798231	3640980
CR_04170W_A	1.075543	0.778749	3640977
TAF4	1.075179	0.786465	3645198
C3_03590W_A	1.074853	0.815818	3638302
HMT1	1.074834	0.791622	3640182
TPO5	1.074052	0.861696	3647791
C2_03560C_A	1.073429	0.816098	3637337
ALK2	1.073313	0.857556	3639700
CR_03430W_A	1.072315	0.890099	3645114
C4_01130C_A	1.071765	0.865538	3635682
CR_07200W_A	1.071527	0.840563	3646044
SAP10	1.071439	0.819007	3641126
RPA135	1.070188	0.803468	3637996
CR_03230W_A	1.06967	0.808149	3645127
C5_03830C_A	1.069184	0.839547	3647227
C3_03470W_A	1.068189	0.8946	3638306
C5_00380W_A	1.06745	0.85438	3641553
C2_07390C_A	1.066985	0.82303	3645229
C1_09000W_A	1.066957	0.89509	3634915
HMX1	1.066648	0.778345	3639294
BNI1	1.066585	0.78494	3636118
C6_04440C_A	1.066386	0.822388	3645983
C7_01590C_A	1.064942	0.873687	3637037
NGS1	1.064804	0.811593	3639703
CDS1	1.064293	0.806146	3647729
C4_06360C_A	1.062303	0.950755	3642825
ALK8	1.062192	0.950413	3648226

C1_09930W_A	1.062192	0.950493	3634860
CR_06310W_A	1.062082	0.950979	3644860
C4_04950W_A	1.062014	0.951014	3641168
C1_14200W_A	1.061935	0.851011	3643301
C1_00540C_A	1.06145	0.829989	3639367
C5_00560W_A	1.061008	0.834313	3648267
NOP5	1.060835	0.828703	3646084
CHS3	1.060255	0.81013	3636199
C2_02540W_A	1.06009	0.831772	3635820
FLC1	1.059229	0.817214	3639854
C1_12350W_A	1.058958	0.821691	3642063
C1_00760W_A	1.057478	0.875443	3639351
C3_00420W_A	1.05709	0.8354	3640553
C1_05400C_A	1.057049	0.91128	3642478
SAC3	1.055713	0.832964	3635823
YPT7	1.055381	0.900278	3636864
TIP120	1.055024	0.847477	3638494
RRP42	1.054166	0.878876	3647852
CR_02390W_A	1.053865	0.875198	3644930
ECM7	1.052912	0.863353	3645029
C4_01660W_A	1.052442	0.838279	3635712
C6_00210W_A	1.051565	0.894532	3646060
CR_02610C_A	1.051458	0.853078	3642566
ANT1	1.049913	0.908361	3639516
C2_08830W_A	1.049419	0.858302	3643894
C1_07360W_A	1.049309	0.852817	3644470
C4_00820W_A	1.048844	0.889277	3645741
HST3	1.047962	0.867935	3642641
C4_04930C_A	1.047025	0.918764	3641170
ERG1	1.046784	0.845355	3646509
SET1	1.046209	0.881531	3639280
YML6	1.044253	0.873516	3637954
LAB5	1.043414	0.863518	3637893
GLN1	1.043	0.883919	3646386
PIN3	1.042881	0.868978	3635228
C1_06800W_A	1.04244	0.87978	3639496
IMH3	1.041626	0.888113	3646556
TSC2	1.04094	0.898185	3646953
HST1	1.040389	0.910274	3634770
C1_04990C_A	1.039463	0.915903	3647530
C1_00970W_A	1.038754	0.955434	30514958
C2_02150C_A	1.037279	0.899995	3635749
MSF1	1.03551	0.896931	3638806
C3_01150C_A	1.035258	0.918684	3648401
SEC9	1.035061	0.891089	3644052
CR_09310W_A	1.034615	0.914196	3641923
RPL38	1.034288	0.905688	30515082

RAD9	1.032688	0.925235	3637780
C4_05620C_A	1.032361	0.954211	3641783
HXK1	1.031457	0.905827	3645964
C1_06600W_A	1.030527	0.9003	3639513
C7_02170C_A	1.029891	0.939506	3636993
CDC43	1.029629	0.953897	3648206
C3_03070W_A	1.028774	0.925602	3638396
C4_06800W_A	1.028161	0.910882	3642917
SKI2	1.027668	0.913465	3642143
C6_00230W_A	1.027635	0.953819	3646061
C7_02850W_A	1.027214	0.921162	3641399
FCR3	1.026715	0.926339	3643760
CR_01420W_A	1.02567	0.923267	3640137
C2_09680W_A	1.024445	0.934509	3643791
ECM17	1.022685	0.930213	3644077
C7_03480W_A	1.021863	0.941703	3646230
C1_03400W_A	1.02089	0.938428	3636904
C4_04620C_A	1.019433	0.971475	3641155
C6_04110W_A	1.019171	0.946462	3647834
VPS22	1.018754	0.97123	3647250
C5_04720C_A	1.016538	0.956022	3636518
C2_07520C_A	1.015959	0.956124	3645207
TAC1	1.015804	0.959235	3643755
PHO112	1.015746	0.987989	3644929
MAS2	1.014387	0.959904	3647249
C2_09310C_A	1.013985	0.973645	3644104
C6_02310W_A	1.01374	0.969137	3645875
C2_02500W_A	1.013435	0.966209	3635816
C7_01030C_A	1.013338	0.960598	3638019
C5_00230C_A	1.012148	0.987297	3641564
C6_03240W_A	1.012148	0.987321	3639181
C5_02360C_A	1.012109	0.987306	3637792
C1_12800W_A	1.012109	0.987321	3646828
C5_04890C_A	1.01181	0.97937	3636415
C4_02660W_A	1.011795	0.988016	3637831
PET100	1.010862	0.97507	30515038
CR_04160C_A	1.010851	0.972193	3640976
C3_00300W_A	1.009099	0.973032	3640524
C1_10280C_A	1.008934	0.98023	3634902
C1_11790W_A	1.00884	0.985723	3645326
PGA27	1.008608	0.986264	3638811
C4_02150C_A	1.008224	0.986885	3635670
C7_00010C_A	1.007374	0.97977	3638006
C1_10470W_A	1.006878	0.979098	3647184
C5_01870W_A	1.006361	0.980573	3643752
ARO10	1.004442	0.990892	3647320
C3_07660W_A	1.003344	0.990658	3638462

C5_05260W_A	1.003341	0.995139	3636473
C3_07350W_A	1.003011	0.990939	3638476
C2_02490C_A	1.002687	0.991051	3635815
GSH2	1.00258	0.993042	3642083
ECI1	1.002395	0.996009	3642128
SYG1	1.000547	0.9985	3644777
C2_03930C_A	-1.00035	0.998996	3637423
NOC2	-1.00047	0.998497	3647596
C2_01750C_A	-1.00189	0.995329	3635734
BFA1	-1.00285	0.992653	3639301
PTC1	-1.00296	0.991582	3634827
FRP3	-1.00299	0.992117	3648102
CR_09530C_A	-1.00329	0.989717	3647101
SUL2	-1.00409	0.990129	3637835
RBT5	-1.00519	0.986798	3645007
C6_03260W_A	-1.00538	0.987228	3639183
FGR17	-1.00561	0.991373	3640296
IFF5	-1.00565	0.98389	3642782
C1_00160C_A	-1.00586	0.985769	3639341
C2_08900W_A	-1.00638	0.988342	30515156
FOX3	-1.0074	0.98125	3636629
PPT1	-1.00794	0.978059	3636602
DAD4	-1.00842	0.990058	30515433
C7_03460W_A	-1.00845	0.990387	3646712
CR_01170W_A	-1.00993	0.964201	3640429
CR_06250W_A	-1.01017	0.9809	3644852
C3_05140C_A	-1.01018	0.972688	3635161
UTP20	-1.01058	0.968567	3647956
ERO1	-1.01196	0.968677	3634810
C1_10330C_A	-1.0131	0.968467	3634908
C7_00130W_A	-1.01357	0.967445	3637975
SUI2	-1.0139	0.955573	3639569
MRR2	-1.01419	0.962722	3647468
C1_07210C_A	-1.01433	0.958342	3644473
C2_10820C_A	-1.01469	0.956326	3641230
CR_08590W_A	-1.01486	0.955977	3642106
CR_06960W_A	-1.01515	0.965615	3645737
BCK1	-1.01556	0.959582	3641384
UFE1	-1.0161	0.953099	3643174
C2_06030W_A	-1.01612	0.956941	3644070
C2_01490C_A	-1.01615	0.95501	3635800
SNF1	-1.01663	0.94637	3642643
C2_05540C_A	-1.01664	0.962714	3645559
C4_06880C_A	-1.01838	0.959145	30515287
C1_03410W_A	-1.01897	0.975821	3636905
MODF	-1.0198	0.95846	3636182
C4_04760C_A	-1.01988	0.955079	3641118

C2_01070W_A	-1.0204	0.936002	3638834
POM152	-1.02226	0.938094	3638812
SDH4	-1.02344	0.948718	3636497
PHM7	-1.02386	0.923135	3642329
C3_01170W_A	-1.02606	0.926562	3647958
CDR3	-1.02629	0.951271	3645365
C1_01180C_A	-1.02703	0.962343	3640206
C5_05090W_A	-1.02729	0.939325	3636493
CR_06680C_A	-1.02778	0.910319	3646623
CR_00670C_A	-1.02877	0.937972	3639655
IFU5	-1.03008	0.906933	3640116
C4_00880W_A	-1.03016	0.91682	3635681
CR_05980W_A	-1.03198	0.899077	3642501
C2_10760C_A	-1.03281	0.896556	3641238
C1_06340W_A	-1.03294	0.960339	3639499
TOP1	-1.03451	0.894668	3644034
C4_03410W_A	-1.03535	0.891511	3646807
C7_01360C_A	-1.03572	0.9433	3645900
FAA2	-1.03617	0.898875	3641731
C3_01260C_A	-1.03781	0.920615	3636625
C5_00820W_A	-1.03833	0.886661	3644128
CR_01780W_A	-1.03857	0.919836	3640157
GSL2	-1.0389	0.889467	3640410
C4_02880C_A	-1.04022	0.910292	3637907
C3_06150W_A	-1.04025	0.895475	3641765
GAP6	-1.04312	0.882413	3646968
CR_10610C_A	-1.04371	0.93801	3639005
C1_00510W_A	-1.04374	0.89707	3639311
C2_01450C_A	-1.04418	0.966822	3635843
C5_01260W_A	-1.04418	0.966842	3642615
PRE1	-1.04441	0.873367	3636500
C7_02960C_A	-1.04577	0.910086	3641387
PRE5	-1.04589	0.855508	3643111
C4_00850C_A	-1.04609	0.949566	3635718
C3_01520C_A	-1.04622	0.863191	3636570
C2_07920W_A	-1.04701	0.897522	3642294
RRD1	-1.04822	0.926111	3638445
C6_04080W_A	-1.04829	0.836706	3647837
CR_05460W_A	-1.04933	0.887443	3648237
C3_07600W_A	-1.04985	0.891302	3638495
AGE1	-1.04987	0.877907	3636913
C1_10170W_A	-1.05088	0.978168	3634783
C2_06980W_A	-1.05088	0.978168	3643530
C1_12940C_A	-1.05098	0.978122	30515067
C1_11390W_A	-1.05098	0.978122	3644544
C2_02790C_A	-1.05098	0.978122	3643029
SCW4	-1.05098	0.978122	3636797

C2_06140C_A	-1.05098	0.978122	3644082
C2_06350C_A	-1.05098	0.978122	3646919
OPT5	-1.05098	0.978122	3640768
DAL52	-1.05098	0.978122	3643736
C4_03490C_A	-1.05098	0.978122	3646799
RBT7	-1.05098	0.978122	3637925
CR_09350C_A	-1.05098	0.978122	3641901
CR_08740W_A	-1.05098	0.978122	3642123
JEN1	-1.05098	0.978122	3642278
C7_01380W_A	-1.05098	0.978122	3645891
CR_02340W_A	-1.05098	0.978122	3644936
RNY11	-1.05098	0.978122	3636408
GTT12	-1.05098	0.978122	3638304
CR_00530W_A	-1.05098	0.978122	3639668
CR_03300C_A	-1.05109	0.978076	3645098
C4_04400C_A	-1.05109	0.978076	30515268
GTT13	-1.05109	0.978076	3638301
C1_05890W_A	-1.05109	0.978076	3639876
C3_07690C_A	-1.05434	0.846976	3638458
PEP8	-1.05454	0.849565	3645624
UGA33	-1.05469	0.88863	3641886
ORC1	-1.05511	0.831315	3636922
C1_07960W_A	-1.05544	0.88172	3640725
EAP1	-1.05562	0.840286	3643814
C4_06650W_A	-1.05576	0.821718	3642810
C6_00550W_A	-1.05631	0.83189	3647144
SAP8	-1.05687	0.956289	3638371
NIP100	-1.05689	0.876603	3640970
C1_05480C_A	-1.05769	0.942715	3642486
COX13	-1.05798	0.82522	3635784
C1_11530C_A	-1.05817	0.933402	3645334
C2_02910W_A	-1.05823	0.941993	30515097
TEP1	-1.05837	0.919028	3647653
RRP6	-1.05849	0.819497	3647535
PCL1	-1.05856	0.925728	3646356
C5_00550C_A	-1.05867	0.911328	3641535
DFG10	-1.05869	0.917227	3645786
C2_06650C_A	-1.05885	0.890485	3648419
CR_02440W_A	-1.05889	0.92733	3644923
BUR2	-1.05899	0.864129	3637772
CFL5	-1.05909	0.918029	3642612
C7_01950W_A	-1.05919	0.902196	3636970
C2_01920C_A	-1.05921	0.856395	3635792
C1_01090C_A	-1.05936	0.869667	3640174
MRPL37	-1.05937	0.87345	3644782
C5_03850W_A	-1.05942	0.853036	3647225
C5_04320C_A	-1.06001	0.834766	3636480

C2_04480W_A	-1.06062	0.910653	3645464
C5_04910W_A	-1.0615	0.872811	3636417
C1_06590C_A	-1.06157	0.8356	3639514
CR_08300C_A	-1.06205	0.809917	3642076
C2_00620C_A	-1.06209	0.885641	3638849
C4_01820C_A	-1.06276	0.808656	3635594
VPS34	-1.06471	0.83765	3639540
MVD	-1.06514	0.803978	3639306
CR_05560W_A	-1.06569	0.823335	3647592
UGA1	-1.06581	0.828448	3637329
C4_06210C_A	-1.06609	0.800598	3642815
UTP15	-1.06641	0.804361	3643906
GIR2	-1.06653	0.812463	3636962
ASR3	-1.06676	0.789275	3637402
C1_14450C_A	-1.06761	0.794351	3643299
C2_01510C_A	-1.06809	0.892167	3635801
C3_01670W_A	-1.06882	0.805681	3636606
C3_03260W_A	-1.07092	0.786967	3638320
YMX6	-1.07094	0.862007	3640255
SGE1	-1.07139	0.812459	3642614
C3_04750W_A	-1.07263	0.849593	3635252
C5_02480W_A	-1.07354	0.795016	3637762
C3_05680W_A	-1.07445	0.818108	3641724
CR_03700C_A	-1.07462	0.789066	3643223
RPF2	-1.07468	0.804606	3643467
GCD6	-1.07511	0.74503	3646510
BUD16	-1.07582	0.867875	3647519
FGR6-4	-1.07613	0.824482	30515164
C1_13790C_A	-1.07665	0.867051	3636141
INO2	-1.07693	0.868624	3639674
CYM1	-1.07703	0.766583	3642244
HPA2	-1.07799	0.823963	3647907
SKN7	-1.07891	0.796414	3641563
CR_02590C_A	-1.07966	0.804635	3642595
CHO2	-1.07967	0.780029	3647690
YTA6	-1.08024	0.747711	3638990
C6_04260C_A	-1.08047	0.871694	3646277
C7_04260W_A	-1.08061	0.774391	3643160
ARL1	-1.08085	0.794614	3635637
FET33	-1.08094	0.808717	3641544
C4_05650W_A	-1.08188	0.787361	3641814
CR_06090W_A	-1.08306	0.75784	6335359
C3_06140W_A	-1.0843	0.836751	3641764
GSC1	-1.08447	0.780601	3636794
ZFU2	-1.08482	0.80912	3638534
C3_02410C_A	-1.08698	0.784421	3638341
C1_05230W_A	-1.08723	0.85969	3642463

RPT1	-1.08782	0.738044	3642461
RBR2	-1.08987	0.874654	3640983
PLB4.5	-1.09014	0.874465	3635724
C1_04840C_A	-1.09086	0.830974	3644785
C1_09540W_A	-1.09104	0.743249	3634885
CR_07510W_A	-1.09159	0.831171	3647998
CCP1	-1.09195	0.822049	3638350
NRG1	-1.09241	0.765347	3643166
RFX2	-1.09331	0.891349	3635658
C3_03680W_A	-1.09412	0.806064	3645648
UTP21	-1.09418	0.729608	3635757
C1_06770W_A	-1.09427	0.747501	3639504
SEC7	-1.09535	0.720015	3635220
CDL1	-1.09659	0.742157	3641132
CRM1	-1.09678	0.714866	3639721
HCH1	-1.09713	0.782593	3641626
PCL5	-1.09756	0.731618	3636466
C1_02750C_A	-1.09776	0.728822	3636828
C2_08740W_A	-1.09794	0.798477	3643882
C5_01040W_A	-1.09815	0.909474	3642649
FAA2-1	-1.09995	0.84444	3644100
C5_01300C_A	-1.10079	0.868167	3642646
C3_05940C_A	-1.10294	0.836526	3641744
C5_01170W_A	-1.10302	0.840397	3642662
MCT1	-1.10304	0.815754	3646683
C1_02090C_A	-1.10304	0.785168	3644577
ZCF11	-1.10321	0.771276	3645109
PUT3	-1.10361	0.734755	3639535
SNM1	-1.1038	0.80524	3642609
C3_03540W_A	-1.10408	0.706768	3638313
HAL21	-1.10604	0.707998	3644045
C3_07170C_A	-1.10656	0.67929	3638536
C2_00920W_A	-1.10744	0.802338	3638893
C2_04500W_A	-1.10774	0.723424	3645462
C7_04150W_A	-1.10786	0.697339	3643128
GLN4	-1.10876	0.62962	3637998
C3_03430C_A	-1.10881	0.828626	3638355
FCY24	-1.11124	0.807794	3641902
C5_05210W_A	-1.11127	0.703162	3636468
C5_02370C_A	-1.11229	0.71851	3637793
CGT1	-1.11302	0.70217	3641808
C5_01610W_A	-1.11316	0.767855	3647251
C2_05670C_A	-1.11351	0.739653	3645570
C1_09440W_A	-1.11489	0.745775	3634857
CFL2	-1.11578	0.879783	3641811
CR_03110W_A	-1.11659	0.73683	3645124
RPR2	-1.11692	0.835359	3641017

IRO1	-1.11751	0.665588	3636648
C1_13650C_A	-1.11755	0.745073	3636191
C6_01210W_A	-1.11887	0.726825	30515330
C4_00840W_A	-1.11905	0.674111	3635722
C2_09690C_A	-1.11988	0.695017	3643789
C1_01390C_A	-1.11989	0.685245	3640236
C7_00500W_A	-1.12041	0.883652	3637987
MNN23	-1.12109	0.652492	3634813
CR_06530W_A	-1.12192	0.752615	3645262
CR_07160C_A	-1.122	0.706728	3648347
MDS3	-1.12253	0.646928	3638464
FGR32	-1.12284	0.593346	3644136
C4_06410W_A	-1.12309	0.739838	3642796
C6_02460C_A	-1.12345	0.702124	3639168
C2_01760C_A	-1.12556	0.810259	3635736
KRE1	-1.12629	0.701798	3643180
GCD1	-1.12844	0.627084	3640965
CSO99	-1.12855	0.832777	3642786
CR_06450W_A	-1.12864	0.717524	3645279
PET127	-1.12936	0.653402	3644417
SDS22	-1.13084	0.672639	3637362
BMT6	-1.13103	0.647961	3639258
WSC4	-1.13114	0.674494	3643282
C3_01680C_A	-1.13237	0.71537	3636605
BCR1	-1.13339	0.63864	3645280
NEP1	-1.13425	0.64059	3644545
MYO5	-1.13457	0.629784	3647404
C2_00790C_A	-1.13503	0.69687	3638907
CR_03930C_A	-1.13537	0.668189	3640957
C4_01790W_A	-1.13552	0.703983	3635597
MNN13	-1.13555	0.794025	3637720
CR_03940W_A	-1.13629	0.659068	3648378
KRR1	-1.13686	0.692187	3644542
C4_01720C_A	-1.13698	0.605771	3635589
C5_03700C_A	-1.13775	0.699085	3646990
RAX2	-1.13776	0.603824	3647610
C1_14560C_A	-1.13803	0.654961	3648376
CR_06100C_A	-1.13828	0.870136	3644842
C5_02800C_A	-1.13861	0.869113	3637807
C6_04190C_A	-1.13864	0.867062	3646282
C6_04230W_A	-1.13864	0.867304	3646274
CR_01770C_A	-1.13865	0.600465	3640122
C7_03400C_A	-1.14023	0.586723	3646717
CR_00090C_A	-1.14031	0.656782	3639734
C4_01290W_A	-1.14108	0.61532	3635699
KIS1	-1.14343	0.590371	3644063
C4_05610C_A	-1.14388	0.62927	3641782

C5_05070W_A	-1.14414	0.738914	3636491
C1_12880C_A	-1.14535	0.802696	3636151
PRE3	-1.14634	0.599645	3643660
CR_06740W_A	-1.14646	0.700163	3646616
CR_09750C_A	-1.1474	0.715005	3639009
ARG4	-1.14886	0.629283	3646237
C2_04700C_A	-1.14928	0.691726	3647794
C1_12820C_A	-1.14928	0.670052	3646826
C2_05350C_A	-1.14947	0.790509	3643428
C6_00250W_A	-1.15034	0.621173	3646063
FGR43	-1.15072	0.765564	3634828
C5_03480C_A	-1.15208	0.632182	3646965
ARG83	-1.15224	0.716308	3637862
C5_00260W_A	-1.15241	0.537977	3641561
CR_10640W_A	-1.15307	0.724799	3639014
C6_01920C_A	-1.1532	0.597682	3644534
GLX3	-1.15324	0.585949	3638325
SKI3	-1.15338	0.621552	3634847
C3_00840C_A	-1.15465	0.695412	3647758
DUR4	-1.15481	0.716361	3645012
RAD57	-1.1552	0.676192	3642333
PAM17	-1.15552	0.605812	3638369
SPT10	-1.1571	0.649105	3645729
ZCF8	-1.15718	0.651146	3636651
MTR10	-1.15957	0.583619	3647235
HAL22	-1.15965	0.692624	3644051
C5_01930W_A	-1.15983	0.611597	3643741
UBA4	-1.1607	0.593917	3644432
C4_02260C_A	-1.16115	0.580989	3635690
C4_03830W_A	-1.16131	0.571309	3647851
ASR1	-1.16174	0.69728	3644402
C3_03200C_A	-1.16437	0.756118	3638428
CR_03740C_A	-1.16448	0.59788	3643176
CR_09700W_A	-1.16502	0.696104	3647088
CR_05030W_A	-1.16515	0.535668	3646388
C2_06200C_A	-1.16672	0.596228	3646908
PWP1	-1.16703	0.53709	3635623
CR_00340C_A	-1.16727	0.786352	30515365
C2_04730W_A	-1.16778	0.590597	3647787
C6_03530C_A	-1.16786	0.785717	3640260
MBF1	-1.1679	0.585663	3640171
CR_02600W_A	-1.16798	0.787318	3642596
C1_01220C_A	-1.16919	0.688708	3640210
UTP13	-1.17021	0.526328	3637718
C1_02900C_A	-1.17167	0.640994	3636869
GDI1	-1.17184	0.533827	3643298
C3_00530C_A	-1.17193	0.630934	3640588

C6_03300C_A	-1.1723	0.595638	3639196
AVT7	-1.17356	0.58115	3637433
C3_02990C_A	-1.17442	0.6959	3638388
C6_01890C_A	-1.17488	0.525528	3641598
C5_04260W_A	-1.17501	0.813436	3636451
CR_08280W_A	-1.17516	0.69316	3642102
C2_02180W_A	-1.17629	0.565068	3635752
C2_04100W_A	-1.17649	0.657871	3637379
C2_04180C_A	-1.17659	0.726845	3637330
CR_02890C_A	-1.17672	0.58008	3642585
MEF2	-1.17739	0.539061	3639564
C3_06760W_A	-1.17833	0.594424	3638502
C5_03640W_A	-1.17878	0.573041	3646984
C1_14460W_A	-1.1792	0.488064	3643326
MED4	-1.17928	0.621414	3645209
C1_14430C_A	-1.1795	0.619444	3643297
CKB2	-1.17964	0.584668	3637741
VPS36	-1.18085	0.616067	3637354
C5_03110C_A	-1.18246	0.747465	30515306
C3_03150W_A	-1.18253	0.55971	3638423
CR_06030C_A	-1.1844	0.647186	3644874
C6_02350C_A	-1.18522	0.574199	3645881
C2_06300W_A	-1.18557	0.713903	3646924
C2_07680W_A	-1.18595	0.512826	3642339
SPS20	-1.18617	0.652599	3645739
C5_01050C_A	-1.18696	0.584161	3642648
C1_05980W_A	-1.18801	0.581878	3639872
NMA111	-1.18806	0.509644	3640179
CR_07760W_A	-1.18869	0.740456	30515431
RPT2	-1.18909	0.505356	3640525
ECM42	-1.18935	0.613228	3636995
C3_05460W_A	-1.1911	0.530375	3643670
GYP8	-1.19218	0.576051	3637750
C3_07700W_A	-1.19269	0.510065	3638457
C6_02800W_A	-1.19313	0.598378	3639246
HEX1	-1.1932	0.508895	3646981
C1_02380C_A	-1.19362	0.718465	3636790
NPL6	-1.19428	0.495313	3638883
YUH2	-1.19469	0.520551	3636412
C1_07390W_A	-1.19511	0.461846	3644466
C5_04470C_A	-1.19583	0.448324	3636414
C2_05910W_A	-1.19587	0.575539	3647476
HAP5	-1.19604	0.521239	3642654
TIM17	-1.19646	0.547377	3647790
YVH1	-1.19658	0.61784	3643218
HGT16	-1.19756	0.797958	3646026
DBP2	-1.19837	0.567719	3647692

NHP2	-1.19842	0.551455	3641003
C2_07040W_A	-1.19964	0.522043	3643499
CSE4	-1.19968	0.739776	3647750
TIM22	-1.19973	0.508304	3648168
C2_03420C_A	-1.20009	0.472716	3637411
C1_09310C_A	-1.20056	0.61192	3634833
C5_00170W_A	-1.2017	0.523263	3647870
CR_04830C_A	-1.20331	0.694071	3647297
C5_02730C_A	-1.20476	0.491463	3637738
CRD2	-1.20481	0.858836	30515234
C2_04390W_A	-1.20493	0.85536	3645458
C2_01960C_A	-1.20498	0.853684	3635796
C1_03660W_A	-1.205	0.80346	30514982
PGA58	-1.20506	0.853397	3637826
C7_02090C_A	-1.20521	0.636179	3637024
HGT1	-1.20565	0.531221	3645420
DSE1	-1.20669	0.613935	3643899
RMT2	-1.20826	0.47288	3641569
C5_02830W_A	-1.20851	0.504528	3637809
MED8	-1.20987	0.683069	3645446
CR_09040W_A	-1.21006	0.625487	3641894
C3_00950C_A	-1.21072	0.588902	3639852
PTC2	-1.211	0.449798	3640105
C4_04120C_A	-1.21194	0.51929	3641222
C6_03380W_A	-1.21214	0.468122	3639231
AFL1	-1.21233	0.422728	3641594
CR_04240C_A	-1.21417	0.450521	3640989
CR_07700W_A	-1.21491	0.685945	3648195
C3_03230C_A	-1.21565	0.493247	3638316
C7_00870W_A	-1.21596	0.684419	3638054
C1_04700C_A	-1.21646	0.530446	3644766
C4_03750C_A	-1.21717	0.612018	3645356
C5_01200W_A	-1.21834	0.704351	3642657
C7_03730C_A	-1.2191	0.495771	3647867
C2_02420C_A	-1.21932	0.478572	3635756
GPH1	-1.21995	0.442261	3637956
MOB1	-1.2234	0.526414	3639208
C3_06740W_A	-1.22441	0.493722	3638503
C4_04990C_A	-1.2247	0.687712	3641166
CR_05710C_A	-1.22568	0.680909	3646700
PHR2	-1.22569	0.441636	3639334
C7_03680W_A	-1.22606	0.592584	3646228
C2_08170W_A	-1.22696	0.798891	3642318
SAP98	-1.22836	0.608356	3637435
C1_01470W_A	-1.2296	0.395913	3640241
C1_03330C_A	-1.22979	0.513405	3636859
YCK2	-1.23018	0.397956	3643655

C4_03710C_A	-1.23069	0.600848	3645360
CHA1	-1.23097	0.645155	3638791
AAH1	-1.23107	0.531958	3643529
C1_01930W_A	-1.23177	0.548557	30514966
VID21	-1.23281	0.404748	3642946
C6_04450W_A	-1.23297	0.478682	3645982
C4_05230C_A	-1.23448	0.494865	3646156
C1_10340W_A	-1.23476	0.57879	3634909
SPB1	-1.23489	0.385329	3648179
C6_01520W_A	-1.23524	0.743028	3641617
PRP39	-1.23545	0.569577	3635832
HIR1	-1.23553	0.379681	3638796
CR_08920W_A	-1.23566	0.706544	3641911
C2_06230W_A	-1.23667	0.391655	3646911
CCR4	-1.23675	0.364696	3640749
SEC65	-1.23934	0.456475	3640123
C2_01240C_A	-1.23967	0.521442	3638794
DUR7	-1.24003	0.527711	3643335
MEC3	-1.24033	0.636459	3646910
C6_03420W_A	-1.24097	0.694467	3639235
CR_06040W_A	-1.24127	0.393416	3644836
C2_06120C_A	-1.2413	0.624283	3644084
RPA190	-1.24172	0.430855	3647677
PRP45	-1.24225	0.590066	3639166
C3_01660W_A	-1.2425	0.506446	3636607
C3_01230C_A	-1.24354	0.744128	3636609
NUP60	-1.24468	0.359604	3642826
CCW14	-1.24492	0.574915	3642650
DOA4	-1.24533	0.38558	3643337
ECM3	-1.24595	0.433206	3635754
CR_10500C_A	-1.24661	0.579731	3638982
C1_06540C_A	-1.24679	0.394319	3639520
RPT6	-1.24695	0.310975	3643877
C1_04110W_A	-1.24708	0.576596	3644699
FAA2-3	-1.24855	0.525102	3643125
C2_02040W_A	-1.25023	0.408482	3635848
C5_00350C_A	-1.25258	0.382447	3641554
APE2	-1.25361	0.41689	3647990
C1_02950W_A	-1.25376	0.702211	3636833
C2_01050W_A	-1.25384	0.692167	30515086
GPI8	-1.2542	0.425842	3646669
C5_04360C_A	-1.25496	0.436977	3636483
C1_01820C_A	-1.25555	0.435655	3645435
C1_10190W_A	-1.25727	0.424379	3634785
CR_06820W_A	-1.25806	0.651013	3647325
C2_09360W_A	-1.25817	0.488682	3644109
CPR6	-1.25903	0.319864	3639020

TIF4631	-1.2598	0.43022	3643880
MED7	-1.26059	0.621962	3638344
C3_06600C_A	-1.26064	0.478414	3642280
MET28	-1.26111	0.442256	3637962
C2_04570W_A	-1.26131	0.355265	3645441
ISW2	-1.26256	0.40682	3642235
CR_09580C_A	-1.26294	0.379121	3647097
SMC6	-1.26338	0.334841	3637039
C5_04630W_A	-1.26364	0.566916	3636395
C7_01370W_A	-1.26388	0.545609	3645901
C4_06430C_A	-1.26445	0.545018	3642794
C1_11150W_A	-1.26469	0.498774	3648356
C7_00450C_A	-1.26497	0.557646	3637991
KIP4	-1.26584	0.533933	3642032
C3_05970C_A	-1.2669	0.310756	3641730
TERT	-1.267	0.486366	3640729
C1_07950C_A	-1.26848	0.488766	3640724
C1_09220W_A	-1.26943	0.375897	3634898
PIF1	-1.2695	0.358924	3646054
C1_11770C_A	-1.27005	0.355501	3645327
C6_02380W_A	-1.27092	0.389408	3645883
PCD1	-1.2711	0.434113	3647087
PEX19	-1.27149	0.381402	3642108
MRT4	-1.27153	0.393882	3639219
C2_04080W_A	-1.27511	0.39235	3637381
PGA25	-1.27522	0.379155	3647890
C1_14470W_A	-1.27573	0.436673	3643327
GEF2	-1.27598	0.360859	3644935
SNX4	-1.2782	0.334224	3638843
C6_03430C_A	-1.27859	0.404198	3640273
C7_03590C_A	-1.27978	0.375557	3646239
RIM11	-1.28032	0.312466	3648277
C2_01250W_A	-1.28138	0.511541	3638793
LYS144	-1.2822	0.382052	3640512
C3_05020W_A	-1.28222	0.528824	3635170
FGR44	-1.28227	0.33728	3635166
LAP4	-1.28566	0.499186	3646979
C2_06780C_A	-1.28672	0.427563	3643516
C7_00810W_A	-1.28784	0.330325	3637940
C2_05980C_A	-1.28812	0.467396	3644075
CR_10630W_A	-1.28835	0.58942	3639007
C7_04140C_A	-1.28958	0.403811	3643129
C2_06080C_A	-1.29058	0.434376	3644098
ENG1	-1.29351	0.449754	3636886
CR_04180C_A	-1.29423	0.279242	3640937
C4_00950C_A	-1.29478	0.3713	3635675
C2_06430C_A	-1.295	0.53342	3646558

C4_02340W_A	-1.2963	0.361531	3637883
FGR16	-1.29691	0.618294	3644411
PBS2	-1.29713	0.27797	3641748
HYR3	-1.29841	0.269341	3644119
C4_05980C_A	-1.29893	0.43295	3646516
C3_03780W_A	-1.30152	0.659673	3645637
RRP9	-1.30232	0.369015	3642576
C4_03190W_A	-1.30245	0.385161	3637838
UTR2	-1.30252	0.3154	3636591
TRA1	-1.30283	0.423016	3641644
C5_00090C_A	-1.30452	0.353326	3647885
C4_06440C_A	-1.30616	0.612404	3642793
RCL1	-1.30624	0.384538	3645199
GCN3	-1.30716	0.348867	3645905
SPT23	-1.30749	0.288272	3643958
SEC6	-1.30838	0.291506	3640565
CR_10740W_A	-1.3112	0.427892	3638941
C2_04870C_A	-1.31252	0.244038	3648111
RPO21	-1.31396	0.279047	3638991
C3_02660W_A	-1.31586	0.808062	3638404
C1_03100W_A	-1.31633	0.361448	3636925
C1_12710C_A	-1.31692	0.366285	3646822
C3_03330C_A	-1.31714	0.334606	3638364
HYR4	-1.31722	0.346685	3640436
GLE1	-1.31801	0.335599	3642340
POX1	-1.31826	0.30178	3640247
NIK1	-1.31919	0.262144	3641358
C4_00470C_A	-1.3212	0.358206	3645011
UTP4	-1.32125	0.303468	3636657
PRS1	-1.32147	0.330906	3635817
C7_01180W_A	-1.32201	0.411074	3645915
PWP2	-1.32307	0.255802	3640416
GCD7	-1.3234	0.356131	3637367
STE50	-1.32386	0.364039	3636660
C1_03050W_A	-1.32391	0.250453	3636843
USO6	-1.32582	0.248997	3643647
ABZ1	-1.32612	0.269177	3648307
CR_06640C_A	-1.32989	0.337406	3646619
C4_07130W_A	-1.33028	0.24562	3642938
BDF1	-1.33108	0.28849	3641592
C1_09040C_A	-1.33246	0.383342	3634769
C2_10140W_A	-1.33298	0.534976	3643934
C5_03570W_A	-1.3331	0.287333	3646976
KAP120	-1.3338	0.215123	3643110
CR_07660C_A	-1.33439	0.305664	3647256
MTG1	-1.33451	0.359438	3645139
C2_04820W_A	-1.33467	0.295646	3647776

C2_07000W_A	-1.33529	0.322999	3643534
C4_06680C_A	-1.33583	0.216865	3642950
SKN2	-1.3367	0.383903	3638309
C5_04990W_A	-1.33677	0.472376	3636538
OFD1	-1.33712	0.211923	3646962
DOT4	-1.34093	0.315036	3646810
C3_07390C_A	-1.34196	0.323462	3638472
C3_05330C_A	-1.34248	0.708835	3643650
C1_04800C_A	-1.34262	0.703234	3644789
C1_08180C_A	-1.34449	0.26054	3640743
C4_02580W_A	-1.34659	0.756678	3637915
CPP1	-1.34678	0.23679	3634865
ADH1	-1.34699	0.30998	3636489
FOX2	-1.34711	0.24804	3648327
C6_04650W_A	-1.3484	0.242393	3645979
C3_00400C_A	-1.34889	0.309082	3640554
C6_01850W_A	-1.34893	0.277404	3641629
C1_04040C_A	-1.34966	0.266768	3646429
SLA2	-1.35028	0.202714	3643149
C6_01980C_A	-1.35068	0.290091	3644560
LEM3	-1.35099	0.215194	3643456
C1_10420C_A	-1.35181	0.274082	3647178
SNP3	-1.35291	0.520529	3640991
C1_14410W_A	-1.35321	0.349364	3643293
C1_12870C_A	-1.35334	0.597696	3636180
SUV3	-1.35352	0.24302	3645451
CR_01440C_A	-1.35393	0.284086	30515374
C3_04230W_A	-1.35408	0.301599	3635187
RIX7	-1.35489	0.26749	3637735
ECM38	-1.35591	0.528598	3645375
RIB3	-1.35646	0.243206	3642062
RIT1	-1.35717	0.38357	3638492
RAD18	-1.35763	0.488224	3641637
ALG1	-1.35824	0.419244	3646517
ZCF6	-1.3585	0.508369	3635837
C5_05130C_A	-1.35942	0.457013	3636459
CEM1	-1.36058	0.456386	3635242
HMI1	-1.3611	0.328298	3638997
ZPR1	-1.36164	0.152524	3640177
C3_04940W_A	-1.36232	0.219928	3635270
TTR1	-1.36393	0.194871	3639313
C5_04350C_A	-1.36397	0.331242	3636482
C1_00980W_A	-1.3645	0.366559	30514959
CTF1	-1.36454	0.3122	3635788
CR_04370W_A	-1.36469	0.337809	3641004
C7_00830C_A	-1.36888	0.240347	3637938
C4_03160C_A	-1.36902	0.630145	3637841

C1_07280C_A	-1.37225	0.647051	30515021
LPG20	-1.37532	0.481682	3644780
C3_03440C_A	-1.37572	0.449718	3638356
RAX1	-1.37578	0.251431	3635256
C2_03140C_A	-1.37622	0.174025	3642992
C7_02220C_A	-1.37634	0.268527	3636989
C7_00270W_A	-1.37641	0.268144	3637977
DBP3	-1.37719	0.199578	3634868
APC1	-1.37757	0.214145	3639383
GLE2	-1.37759	0.223225	3646293
TAF19	-1.37885	0.395543	3641396
SLU7	-1.37924	0.345735	3638501
MIG1	-1.37935	0.261628	3637812
C2_03260W_A	-1.37938	0.311683	3637432
C5_01920C_A	-1.37952	0.255399	3643742
CIC1	-1.38048	0.265728	3644059
RBR3	-1.38169	0.275822	3646728
CRZ1	-1.38202	0.282585	3641722
SNF7	-1.383	0.1721	3639377
CTA6	-1.38369	0.329072	3642907
C5_00790C_A	-1.38466	0.220837	3644125
C5_03040W_A	-1.38514	0.431972	3637824
KIC1	-1.38515	0.186958	3647784
C1_06410W_A	-1.3853	0.363702	3639555
RVS162	-1.38538	0.44417	3646820
UTP5	-1.38562	0.169664	3638922
PRR2	-1.38596	0.542889	3646723
MST1	-1.38628	0.288899	3636831
C6_03220W_A	-1.38719	0.378984	3639179
CHT1	-1.38731	0.444622	3639704
NSA1	-1.38747	0.297449	3642313
DJP1	-1.38758	0.176213	3647186
CFL1	-1.38801	0.412865	3641810
C7_01960W_A	-1.38846	0.348968	3636969
C2_08240W_A	-1.38917	0.212862	3642344
ECM1	-1.38947	0.269596	3641224
SGS1	-1.39046	0.281885	3641258
SOL1	-1.39112	0.316576	3645627
C2_07740W_A	-1.39243	0.16236	3642302
C2_09860C_A	-1.39344	0.538053	3643781
ABD1	-1.39454	0.275418	3638456
LIG1	-1.39647	0.170855	3636947
FGR27	-1.39682	0.190303	3646988
C5_00400C_A	-1.39846	0.277227	3641550
SDA1	-1.39871	0.149465	3646688
ARX1	-1.39905	0.173734	3636811
C7_00840C_A	-1.39967	0.431458	3638057

SKP1	-1.39992	0.174406	3644485
CR_02780W_A	-1.40085	0.746902	3642582
C5_04480C_A	-1.40085	0.747039	30515317
C2_01040W_A	-1.40114	0.743628	30515085
C4_01340W_A	-1.40129	0.750999	3635601
SAP6	-1.40138	0.751875	3639229
CR_06320C_A	-1.40233	0.277809	3644845
CR_08500W_A	-1.40274	0.183542	3642136
MNN22	-1.40359	0.262778	3641117
C3_04840C_A	-1.40385	0.318864	3635224
C1_00520W_A	-1.40411	0.213733	3639310
CR_05750W_A	-1.40429	0.308311	3646696
ALR1	-1.40591	0.201143	3636616
C1_00570C_A	-1.40648	0.243598	3639362
PTC4	-1.40665	0.325205	3646697
C7_01170C_A	-1.40716	0.278401	3645914
C5_02140C_A	-1.40735	0.18566	3637752
EST3	-1.40744	0.617217	3640551
BMS1	-1.40782	0.182889	3639857
C2_04740C_A	-1.40973	0.565056	3647786
C1_10890C_A	-1.41029	0.264149	3644447
LYS143	-1.41035	0.495824	3634894
CR_06010W_A	-1.41093	0.399702	3644870
C1_11110C_A	-1.41094	0.395378	3644413
C2_08510W_A	-1.41134	0.442862	3643922
STP1	-1.41186	0.273036	3635152
MSO1	-1.41209	0.298993	3642040
CR_06330C_A	-1.41673	0.29945	3644846
C3_05990C_A	-1.41928	0.591826	3641732
C2_06490W_A	-1.41935	0.189999	3646569
RVS167	-1.42082	0.146285	3647831
C2_09460C_A	-1.42171	0.214693	3647713
C2_08100W_A	-1.42209	0.196375	3642334
PEP7	-1.42261	0.353222	3645001
C1_02220C_A	-1.42272	0.298351	3644595
C1_10250C_A	-1.42275	0.440178	3634882
C5_05460C_A	-1.42384	0.426589	3636527
CR_03220C_A	-1.42793	0.49136	3645128
AFG3	-1.42883	0.123483	3636589
C2_10300C_A	-1.42884	0.464177	3643955
C7_03040W_A	-1.42977	0.455083	3641379
C1_05420W_A	-1.4304	0.362186	3642493
EAF3	-1.43102	0.259918	3648318
CR_09740W_A	-1.4313	0.15515	3639008
C1_13810W_A	-1.43233	0.26406	3636126
C2_07020C_A	-1.43413	0.28269	3643497
RAT1	-1.43481	0.129001	3635686

JAB1	-1.43582	0.158945	3646811
DOS2	-1.43597	0.143623	3636183
C1_06910C_A	-1.43682	0.208902	3639526
C3_06280W_A	-1.43702	0.205628	3642238
HAP3	-1.43819	0.665792	3635629
C1_02200C_A	-1.43861	0.418394	3644593
C5_02620C_A	-1.43895	0.194005	3637783
C1_06380C_A	-1.43919	0.393052	3639559
C1_12610W_A	-1.43968	0.177321	3646825
ESS1	-1.44108	0.202511	3647989
HAS1	-1.44538	0.140746	3636443
C2_00750W_A	-1.44582	0.552819	3638911
C4_07060W_A	-1.44608	0.140507	3642908
SPT7	-1.44639	0.131897	3638928
PPS1	-1.44963	0.395394	3646526
CR_10230W_A	-1.44976	0.126071	3638921
C1_09950C_A	-1.44984	0.187845	3634861
C2_10740C_A	-1.45067	0.167336	3641236
C4_04550C_A	-1.45345	0.177985	3641106
IPK2	-1.45513	0.20538	3643784
CR_00040C_A	-1.45535	0.163291	3639675
ALO1	-1.45811	0.124314	3638983
ZCF13	-1.45892	0.18397	3646354
C4_06090C_A	-1.45894	0.2347	3646532
C4_00560C_A	-1.45934	0.267795	3645744
GLK4	-1.45997	0.277289	3646046
DBR1	-1.46008	0.364544	3647560
C2_10850C_A	-1.4611	0.126731	3641232
C1_13380W_A	-1.46126	0.27846	3636217
C3_05160C_A	-1.46128	0.100967	3635229
C4_02960W_A	-1.46198	0.182408	3637873
SSD1	-1.46302	0.132795	3636520
GAL7	-1.46311	0.227817	3644591
C1_00100C_A	-1.46322	0.213755	3639325
CR_05550C_A	-1.4633	0.094285	3647593
C7_03860W_A	-1.46339	0.107356	3643144
C2_00890W_A	-1.4646	0.643933	3638896
CR_07850W_A	-1.46479	0.231038	3647347
C5_05000C_A	-1.46556	0.16572	3636539
ZCF31	-1.46616	0.157416	3635191
CR_03310C_A	-1.47039	0.123101	3645097
C6_02330W_A	-1.47082	0.307554	3645877
ENP1	-1.47174	0.169521	3647861
C4_04200C_A	-1.47282	0.570054	3641182
C2_01530C_A	-1.4739	0.125712	3635803
C2_00860C_A	-1.47479	0.627429	3638805
C5_03560W_A	-1.47482	0.16102	3646975

IRA2	-1.47533	0.086331	3642011
C3_07240W_A	-1.47535	0.623486	3638530
C2_04750W_A	-1.47605	0.242647	3647785
C1_00030C_A	-1.4767	0.24528	3639373
IPT1	-1.47727	0.120849	3634941
GLG2	-1.47819	0.289345	3642282
MIM1	-1.47847	0.269456	30515394
C1_13240W_A	-1.48027	0.171232	3636167
FGR22	-1.48176	0.393173	3635862
CR_07320C_A	-1.4825	0.117673	3646057
DBP8	-1.48332	0.131621	3646692
C2_02440W_A	-1.48382	0.133097	3635758
ZCF21	-1.4853	0.087438	3645762
C3_00510W_A	-1.4859	0.354967	3640514
C4_01930C_A	-1.48617	0.335789	3635634
C2_03690C_A	-1.48741	0.146158	3637376
CDC4	-1.48754	0.057853	3640125
CR_01360W_A	-1.4877	0.457902	3640167
C3_03530W_A	-1.48814	0.466035	3638312
PGA13	-1.48824	0.468394	3642138
C4_00030C_A	-1.48881	0.224775	3648055
SRT1	-1.49031	0.36473	3642020
DOT5	-1.49031	0.139364	3640519
MUB1	-1.49197	0.11011	3642246
C2_09280C_A	-1.49234	0.265167	3644102
PDE1	-1.49279	0.143525	3637713
CR_08940W_A	-1.49331	0.116513	3641914
C7_03980W_A	-1.4964	0.271993	3643116
CR_02950C_A	-1.49678	0.200382	3642590
C6_01350W_A	-1.4976	0.113967	3641642
C6_01080C_A	-1.49946	0.383634	3644019
SRB8	-1.49984	0.122182	3641128
C2_03890W_A	-1.50081	0.537281	3637396
TRM9	-1.50083	0.243314	3642074
HGT8	-1.50611	0.124838	3638875
CR_10810C_A	-1.50622	0.196336	3638949
C1_00790W_A	-1.50691	0.553412	3639348
UBR1	-1.50726	0.067746	3637871
C3_02180C_A	-1.50755	0.113332	3636643
C3_00380C_A	-1.50807	0.214235	3640540
C4_01500W_A	-1.50858	0.145679	3635617
C1_10880W_A	-1.51079	0.177848	3644449
SPR28	-1.51093	0.391385	3637716
MDG1	-1.51105	0.162632	3643311
C1_01750W_A	-1.51147	0.33658	3645413
C2_04620W_A	-1.51281	0.09124	3645438
C1_06440C_A	-1.51384	0.130589	3639552

C3_01560W_A	-1.51551	0.088868	3636669
C4_06150C_A	-1.51573	0.713541	3642820
C7_00610C_A	-1.51596	0.125706	3638030
CTA3	-1.51706	0.113783	3645333
CUE5	-1.52041	0.123854	3642467
C4_03930C_A	-1.52172	0.258318	30515262
CR_07750C_A	-1.52175	0.100319	3647350
C2_05400W_A	-1.52288	0.369363	3643435
C7_00310C_A	-1.52453	0.352242	3638045
DAD2	-1.52481	0.507709	3643465
C1_03360W_A	-1.52499	0.105997	3636889
NTG1	-1.5253	0.255077	3640746
C3_02360C_A	-1.52534	0.501869	3636617
C3_03690W_A	-1.52569	0.426753	3645647
GYP5	-1.52696	0.059157	3641246
C3_06490W_A	-1.52808	0.129901	3642285
SAP2	-1.52895	0.121881	3647354
C1_01040W_A	-1.52988	0.226922	3640183
ENP2	-1.53052	0.116132	3646234
SUI3	-1.5306	0.060985	3643130
FKH2	-1.53149	0.104815	3640536
RRP8	-1.53239	0.102412	3643900
C2_02630W_A	-1.53313	0.183288	3635839
C2_00700W_A	-1.53423	0.057985	3638884
C2_07760W_A	-1.53569	0.093032	3642298
RIM2	-1.53592	0.172993	3645448
C1_11300C_A	-1.53669	0.115253	3644552
C3_05950W_A	-1.53787	0.25675	3641728
CTA26	-1.5393	0.201898	3638965
MSS116	-1.54014	0.094959	3634792
CR_03180W_A	-1.54033	0.115348	3645133
C2_08180C_A	-1.54229	0.110936	3642317
C6_01380C_A	-1.54533	0.248466	3641606
C1_06070W_A	-1.54549	0.11604	3639824
C6_00310W_A	-1.54824	0.046536	3646071
C1_05360C_A	-1.54864	0.068672	3642482
COX15	-1.54912	0.067393	3647071
C3_00390W_A	-1.55051	0.382969	3640539
MSS11	-1.55061	0.174134	3647296
CAS4	-1.55135	0.106765	3636568
C4_01280C_A	-1.55161	0.121534	3635700
IMP4	-1.55449	0.067354	3644138
C4_00070C_A	-1.55534	0.057107	3648063
C7_01490W_A	-1.55567	0.100517	3637051
NOG1	-1.5562	0.106098	3641736
KCS1	-1.55872	0.170457	3648120
LYP1	-1.55874	0.33129	6335417

C2_07410W_A	-1.55937	0.155978	3645203
SLN1	-1.56096	0.073689	3640422
C4_01990W_A	-1.5611	0.578675	30515246
CR_08190W_A	-1.56141	0.307052	3642084
HTS1	-1.56189	0.079161	3636532
FRP6	-1.56334	0.074428	3641601
ZCF23	-1.56538	0.109933	3644477
CKB1	-1.56601	0.075886	3638799
C2_01820C_A	-1.56637	0.177093	3635831
SFU1	-1.56669	0.157654	3634867
MTLA1	-1.56714	0.301559	3643729
C5_03410C_A	-1.56853	0.149799	3646347
DAL1	-1.57004	0.266204	3640564
ZCF15	-1.57031	0.123134	3637867
C4_07200C_A	-1.57038	0.159391	3642958
C1_00880W_A	-1.57151	0.314252	3639355
SAC7	-1.57181	0.121565	3638010
C3_00450C_A	-1.57235	0.08582	3640548
DRS1	-1.58172	0.060538	3639045
C2_01370C_A	-1.58179	0.073626	3635723
CRL1	-1.58268	0.077209	3640238
SCO1	-1.58314	0.083331	3641922
C3_01780C_A	-1.58333	0.490241	30515179
C6_00390W_A	-1.58503	0.071261	3646086
PRP13	-1.58724	0.157832	3643194
C2_09420W_A	-1.58755	0.140357	3647718
C1_09340C_A	-1.58793	0.166121	3634804
ARG5,6	-1.5895	0.07144	3634830
DFG5	-1.59157	0.055216	3638867
ZCF18	-1.59231	0.124106	3641635
CR_00980C_A	-1.59285	0.051699	3640426
C3_02750W_A	-1.593	0.304436	3638411
C1_08240C_A	-1.59365	0.215733	3640759
C1_10080W_A	-1.59411	0.19325	3634814
ELF1	-1.59428	0.123777	3641903
URA7	-1.59587	0.069324	3636510
C3_03310C_A	-1.59643	0.249719	3638363
C2_03410W_A	-1.59664	0.041779	3637412
C7_00940W_A	-1.597	0.075334	3637955
C2_00170C_A	-1.59755	0.088087	3638906
DOT6	-1.59909	0.068509	3640078
C3_07590W_A	-1.5992	0.301955	3638496
C1_14550C_A	-1.59934	0.267768	3643334
C7_02460C_A	-1.60014	0.088224	3636946
C2_04120C_A	-1.6006	0.071291	3637377
DUO1	-1.60069	0.303656	3646858
C2_03880C_A	-1.60174	0.446604	30515108

C5_04640C_A	-1.602	0.128563	3636397
CR_10750C_A	-1.60216	0.145819	3638942
HAP31	-1.60232	0.386694	3640994
CR_01720W_A	-1.60416	0.14052	3640114
C4_02170C_A	-1.60419	0.295133	3635667
C5_00760W_A	-1.60598	0.054577	3644122
UBI4	-1.6061	0.091205	3638432
C1_06970C_A	-1.60656	0.094155	3639567
YAF9	-1.60916	0.130702	3647864
PEX2	-1.60949	0.154816	3643460
C4_00420C_A	-1.60984	0.116307	3645021
SWR1	-1.61019	0.092329	3645220
C5_01220W_A	-1.61237	0.193368	3642620
C2_00810C_A	-1.61312	0.188508	3638810
NOP14	-1.61317	0.054678	3635266
C1_04490W_A	-1.61381	0.048481	3646145
C1_02800W_A	-1.61389	0.442503	3636873
TGL99	-1.6139	0.125118	3636168
PDK2	-1.61501	0.065129	3641876
POS5	-1.61511	0.166596	3638519
BUD6	-1.61685	0.096734	3640727
TRY2	-1.61794	0.081561	3647670
C1_05280W_A	-1.61831	0.16423	3642475
CDH1	-1.61881	0.07308	3638814
TPK1	-1.62077	0.036805	3634879
CR_03080C_A	-1.62121	0.185574	3645104
C1_13950C_A	-1.62163	0.485361	3636186
C7_03270W_A	-1.62166	0.057001	3641365
C3_07380W_A	-1.62235	0.068986	3638473
MED15	-1.62595	0.056189	3640761
C4_02860W_A	-1.62601	0.063769	3637909
C1_05650W_A	-1.62852	0.133316	3639838
C5_02560C_A	-1.62885	0.089334	3637719
C6_00520W_A	-1.62893	0.39534	3647147
C5_04290C_A	-1.6311	0.045468	3636455
C2_05380W_A	-1.63154	0.078212	3643432
SPB4	-1.63235	0.094281	3647252
CCT8	-1.6327	0.040196	3639324
C4_07010C_A	-1.63601	0.280848	3642966
C4_02850W_A	-1.63611	0.063916	3637910
STE2	-1.63642	0.534108	3646630
C7_00460W_A	-1.63649	0.544787	3637990
C7_03150W_A	-1.63653	0.539221	3641346
C2_02230C_A	-1.63659	0.538636	3635775
GCD2	-1.63679	0.087813	3638437
C1_00890W_A	-1.63729	0.076719	3639354
CGR1	-1.63741	0.140007	3637853

C4_06230C_A	-1.63773	0.052428	3642808
DAL81	-1.63839	0.126686	3640419
C2_09160W_A	-1.63885	0.098034	3644090
C2_03150C_A	-1.63926	0.165595	3642990
C5_00330C_A	-1.64082	0.16383	3641556
SRP40	-1.64208	0.074581	3642605
C5_02010C_A	-1.64347	0.047806	3643772
C1_00430W_A	-1.64408	0.091961	3639285
CR_00820C_A	-1.6441	0.097004	3640413
C5_02690W_A	-1.64575	0.176346	3637775
C2_06620W_A	-1.64612	0.212286	3646564
ADE8	-1.64665	0.072819	3642999
SSF1	-1.64718	0.062857	3647085
C2_05270W_A	-1.6476	0.068627	3643470
FTH2	-1.65236	0.124489	3640401
C1_08540C_A	-1.65242	0.019954	3646497
C3_07100C_A	-1.65867	0.062046	3638443
PAM18	-1.65994	0.070033	3645747
C3_03920W_A	-1.66123	0.115663	30515197
AFG1	-1.66135	0.195222	3634920
C2_02120W_A	-1.66234	0.045325	3635746
CR_06470W_A	-1.66471	0.042256	3645277
CR_03070W_A	-1.66552	0.050442	3645105
MNN14	-1.66789	0.047727	3643651
C3_00340W_A	-1.66792	0.158408	3640544
PGA5	-1.66999	0.633798	3644575
C4_00580W_A	-1.67296	0.169906	3645773
SAP4	-1.67321	0.618899	3640257
C1_12000C_A	-1.67321	0.619052	3642041
MRV1	-1.67332	0.616816	3635610
C4_06220C_A	-1.67332	0.61741	3642814
C3_04170W_A	-1.67332	0.617668	3635182
THI4	-1.67349	0.616191	3635160
AFT2	-1.67419	0.063419	3643511
STI1	-1.67505	0.019424	3643758
MTG2	-1.67528	0.071667	3646693
SCW11	-1.67649	0.350216	3636521
C7_03210W_A	-1.67649	0.120879	3641371
MET16	-1.6765	0.062348	3642964
PAN1	-1.6767	0.099761	3637386
C2_01190C_A	-1.67779	0.16732	3638859
C3_02720W_A	-1.67843	0.189203	3638410
C1_03830C_A	-1.68026	0.061281	3644678
C2_02710C_A	-1.68079	0.057514	3643003
C1_00310W_A	-1.68159	0.078528	3639298
PRP22	-1.68205	0.041167	3636404
C6_00640C_A	-1.68308	0.110853	3647134

C3_01190C_A	-1.68316	0.033128	3647961
C1_12950W_A	-1.6835	0.050745	3636157
ZNC1	-1.68366	0.029	3643754
ROT1	-1.68377	0.115078	3639350
CR_00230W_A	-1.68391	0.02856	3639698
SFL1	-1.68461	0.01883	3642498
C4_00290C_A	-1.68501	0.545872	3644994
NOC4	-1.68665	0.048303	3645224
OLE1	-1.68704	0.058155	3640755
YPS7	-1.68758	0.053039	3636937
C7_03850W_A	-1.68809	0.040477	3643145
C4_06280C_A	-1.68895	0.26889	3642802
CR_00370W_A	-1.68904	0.071899	3639684
C2_09670C_A	-1.68926	0.034566	3643792
PRN2	-1.69077	0.179988	3639879
CR_08710W_A	-1.69079	0.027525	3642131
FUR4	-1.69143	0.145061	3638305
C1_07150W_A	-1.69263	0.227218	30515019
C1_07080W_A	-1.69357	0.145451	3639489
C3_05380W_A	-1.70016	0.176891	3643679
CR_01410C_A	-1.70095	0.046546	3640136
PHO4	-1.70136	0.071928	3641817
COX9	-1.70204	0.063521	30515131
C1_06470W_A	-1.70463	0.048805	30515007
CR_03780C_A	-1.70544	0.28974	3643179
CR_01370C_A	-1.70885	0.053308	3640168
PGK1	-1.70886	0.068656	3647081
NMD3	-1.7091	0.023929	3646614
SET2	-1.70917	0.026682	3643923
MRPL27	-1.70931	0.04702	3636884
C1_09390W_A	-1.71057	0.109329	3634851
C1_03840W_A	-1.71459	0.390223	30514984
C2_05160C_A	-1.71535	0.027354	3643461
CR_03620C_A	-1.71635	0.023842	3643182
GUT1	-1.71709	0.051666	3648082
C2_08860W_A	-1.71765	0.021952	3645776
C1_01160C_A	-1.71796	0.029611	3640204
VAC7	-1.71877	0.05052	3646849
C5_02510C_A	-1.71968	0.035009	3637767
BEM1	-1.72086	0.031044	3635627
PTH1	-1.72136	0.030147	3637710
PDC12	-1.72156	0.301724	3635591
C6_01090C_A	-1.7222	0.305245	3644020
TEC1	-1.72254	0.117267	3635261
C3_02020W_A	-1.72312	0.020378	3636592
C1_00380C_A	-1.72316	0.059851	3639292
SRP54	-1.7234	0.023591	3640377

ZCF38	-1.72403	0.039636	3639705
ROM2	-1.72417	0.025901	3637434
C1_08710W_A	-1.7247	0.066008	3634937
CR_02460W_A	-1.7273	0.038984	3644922
ERG3	-1.72809	0.055614	3644776
C1_11850W_A	-1.72892	0.02651	3642010
ZCF20	-1.7293	0.038194	3647059
MED9	-1.73091	0.148945	3643134
CR_08330W_A	-1.73183	0.070375	3642080
MRPL36	-1.73249	0.044099	3643733
MSS51	-1.73285	0.020571	3639031
GCN1	-1.73293	0.044297	3641256
HAP43	-1.73371	0.011304	3644531
CR_09480W_A	-1.73561	0.048692	3641939
CYR1	-1.73594	0.019277	3641407
C1_11160C_A	-1.73669	0.094511	3644399
C1_04510W_A	-1.73692	0.119736	3646147
C1_12250C_A	-1.73749	0.027003	3642022
C1_04820C_A	-1.73763	0.129612	3644787
CDC37	-1.73826	0.030303	3639210
C2_07510W_A	-1.73915	0.202845	3645208
CR_04270C_A	-1.7394	0.390299	3640992
WOR1	-1.74089	0.623158	3634781
C7_00160C_A	-1.74318	0.036574	3637972
C2_03460C_A	-1.74583	0.050899	3637348
CR_07030C_A	-1.74635	0.077413	3645730
GRX3	-1.74674	0.027482	3637858
FPG1	-1.74699	0.018971	3641632
C4_00750C_A	-1.74836	0.155629	3645763
ACE2	-1.74859	0.082793	3646034
RMP1	-1.74982	0.242722	3636215
C4_01300W_A	-1.75032	0.029935	3635605
BRF1	-1.75057	0.019522	3646689
C7_03000C_A	-1.75134	0.081598	3641383
C5_04050W_A	-1.75213	0.022202	3647735
GCY1	-1.75514	0.418452	3638477
SCS7	-1.75718	0.008908	3641157
C2_06550W_A	-1.76121	0.244067	3646559
NIP7	-1.76159	0.07484	3645880
MRPL40	-1.76231	0.022089	3640968
POP4	-1.76251	0.067115	3639241
C4_06790W_A	-1.76556	0.01676	3642918
C5_02320C_A	-1.76606	0.075215	3637788
TPT1	-1.76638	0.059499	3640543
NOG2	-1.76756	0.023253	3640299
RIB1	-1.76843	0.043796	3642607
C1_02940C_A	-1.76929	0.037778	3636832

C6_02110W_A	-1.77019	0.159296	3647553
EST1	-1.77247	0.011553	3636526
C2_03000C_A	-1.77833	0.052563	3643022
C5_05140W_A	-1.77859	0.079829	3636460
C7_01720W_A	-1.78042	0.034576	3637011
MAK21	-1.78754	0.015662	3635147
C2_02200W_A	-1.78783	0.080588	3635772
C1_01540W_A	-1.78804	0.015829	3640233
C3_03100C_A	-1.78947	0.024929	3638399
IFM1	-1.79068	0.012443	3641389
C1_12540W_A	-1.79397	0.23161	3647607
CR_06840W_A	-1.79504	0.022062	3647322
NOP8	-1.79523	0.035879	3646290
C3_02350W_A	-1.79646	0.019187	3636618
C3_05760W_A	-1.79678	0.079419	3641720
C2_08450W_A	-1.7973	0.030128	3643905
C1_05800C_A	-1.79753	0.157259	3639887
C2_02580W_A	-1.79901	0.021932	3635860
C1_13560W_A	-1.79932	0.051982	3636174
TDH3	-1.8006	0.085677	3638540
C7_00430W_A	-1.80084	0.049617	3637993
IAH1	-1.8009	0.049641	3638945
C5_01430C_A	-1.80158	0.058586	3647056
C4_03850W_A	-1.80217	0.013322	3647853
SWI1	-1.80359	0.041864	3644996
C5_03460C_A	-1.80742	0.077712	3646345
FLU1	-1.80887	0.024254	3637048
C1_11900C_A	-1.8091	0.069729	3642005
JIP5	-1.80919	0.023608	3634922
CR_01130W_A	-1.81105	0.038172	3640433
C2_07630C_A	-1.8121	0.011176	3645193
FET3	-1.81258	0.084713	3647153
PEL1	-1.8151	0.04196	3637989
PGM2	-1.816	0.020623	3642562
CR_09610C_A	-1.81697	0.028728	3647094
CR_04310C_A	-1.81738	0.069023	3640996
CRH12	-1.81755	0.012696	3636447
C7_03580C_A	-1.81848	0.075552	3646238
PPG1	-1.81905	0.102794	3641143
C1_12440W_A	-1.8216	0.220699	3642012
GAP4	-1.82346	0.034008	3644463
CR_00010C_A	-1.82426	0.03038	3639712
C5_00160W_A	-1.82593	0.04437	3647871
C3_04690C_A	-1.826	0.18045	3635196
ZSF1	-1.82668	0.010392	3641257
HEX3	-1.82681	0.01198	3635776
C1_11720W_A	-1.82696	0.3411	3645319

C2_08660C_A	-1.82765	0.336339	3643907
C5_04010C_A	-1.82772	0.335569	3647439
CDC39	-1.82778	0.01486	3646278
CTA9	-1.83141	0.015723	3640215
FGR15	-1.83162	0.030344	3638882
C2_08910C_A	-1.83303	0.018637	3645798
C4_06370C_A	-1.83436	0.101718	3642824
UTP8	-1.83463	0.010423	3640547
RSP5	-1.83683	0.003922	3643898
SOU2	-1.83743	0.449202	3642823
C1_05150C_A	-1.83762	0.460693	3642469
C5_00310C_A	-1.83909	0.025885	3641576
C3_06290W_A	-1.83946	0.022465	3642237
C5_00130C_A	-1.84021	0.020842	3647881
BUD2	-1.84076	0.036685	3641541
TFG1	-1.84279	0.011378	3635645
NAN1	-1.84279	0.009748	3637932
C1_14180W_A	-1.8437	0.218642	3643324
CR_00220W_A	-1.84434	0.09453	3639699
FCA1	-1.84465	0.016447	30515327
C1_13330C_A	-1.8461	0.03002	3636150
RPA34	-1.84644	0.058913	3634883
C1_02450C_A	-1.84674	0.061517	3636849
C1_12570C_A	-1.84754	0.013213	3647604
PAM16	-1.84756	0.033601	3643321
C7_02660C_A	-1.84924	0.193809	3647652
C2_09510C_A	-1.85052	0.021188	3643816
CR_04820W_A	-1.85292	0.039655	3647298
PGA18	-1.8538	0.040466	3638394
C1_05120W_A	-1.85403	0.024139	3647667
RPB7	-1.85616	0.100248	3640221
C2_00270C_A	-1.85944	0.053304	3638803
C4_04250W_A	-1.86052	0.262685	3646855
C4_06850C_A	-1.86072	0.032379	3642912
HEM1	-1.86108	0.009883	3640149
CSC25	-1.86124	0.025243	3645660
RPO41	-1.86262	0.023537	3639378
C7_01660C_A	-1.86343	0.028105	3636983
C4_02450W_A	-1.86369	0.147995	3637845
TSR1	-1.86431	0.007327	3642135
C3_05900W_A	-1.86478	0.020931	3641739
KNS1	-1.86487	0.022577	3636228
C4_06130W_A	-1.86659	0.059044	3648085
C1_06320W_A	-1.867	0.199931	3648118
C4_06990W_A	-1.87164	0.04013	3642968
CR_00430C_A	-1.87203	0.140676	30515366
RLM1	-1.87241	0.057639	3635703

PFK2	-1.87624	0.018598	3637056
C1_10970W_A	-1.87641	0.01683	3644427
C6_04290W_A	-1.87659	0.006546	3646280
C1_09330W_A	-1.877	0.09448	3634802
C3_02670W_A	-1.88157	0.09504	3638405
C4_00860C_A	-1.88353	0.133949	3635717
CR_05150W_A	-1.88451	0.20918	30515403
C6_01040C_A	-1.88481	0.011014	3644014
C3_03770C_A	-1.88524	0.010269	3645636
CSP2	-1.88599	0.208761	3645749
C1_04960C_A	-1.88646	0.010957	3647527
C1_10840C_A	-1.88673	0.003154	3644452
C3_07900C_A	-1.88676	0.036837	3647450
C4_01230C_A	-1.88739	0.055257	3635706
C3_04330C_A	-1.8876	0.02416	3635272
C4_01090C_A	-1.88783	0.193368	3635685
C3_07800C_A	-1.88889	0.041912	3647461
C7_02530C_A	-1.88891	0.06357	3636940
UTP18	-1.88929	0.00855	3643123
C2_00510W_A	-1.89001	0.233048	3638868
MET10	-1.89319	0.007546	3644088
C1_12680W_A	-1.89452	0.007506	3646833
C2_00230W_A	-1.89603	0.034948	3638901
CR_00600C_A	-1.89643	0.018994	3639660
CR_00110W_A	-1.89818	0.008417	3639731
C3_03110W_A	-1.90098	0.083977	3638400
C3_06440W_A	-1.90183	0.011201	3642281
C4_04810C_A	-1.90361	0.012752	3641112
C1_11880W_A	-1.90638	0.046619	3642007
C5_03940C_A	-1.90667	0.03016	3647446
CR_09340W_A	-1.9076	0.018804	3641900
C3_06240C_A	-1.90996	0.243947	30515213
DAL4	-1.9112	0.444474	3638422
C3_01340W_A	-1.91285	0.348508	3636650
C4_02700W_A	-1.91362	0.036628	3637859
C1_01850C_A	-1.91597	0.018134	3645431
FRP5	-1.91628	0.462739	3641600
C4_03200C_A	-1.9171	0.04499	3637837
C7_03620C_A	-1.91827	0.019431	3646242
C3_04260W_A	-1.91957	0.016456	3635140
PCL7	-1.91963	0.004913	3639491
SWD1	-1.9199	0.048179	3640535
C7_02120C_A	-1.92009	0.057757	3637019
KRE6	-1.92128	0.012417	3641756
CNH1	-1.92177	0.009276	3648056
FEN1	-1.9238	0.024596	3646829
C4_03740W_A	-1.92692	0.007925	3645357

CR_08200C_A	-1.92854	0.003799	3642087
C3_02260C_A	-1.93011	0.00441	3636636
MED19	-1.93373	0.075779	30515334
C1_10410W_A	-1.93559	0.209621	3634840
SCT1	-1.9364	0.009027	3648346
C7_03890C_A	-1.93878	0.010843	3643108
C3_07920W_A	-1.93981	0.011786	30515226
TES15	-1.94027	0.132365	3647478
C4_07210W_A	-1.94127	0.02576	3642959
C7_00300W_A	-1.94203	0.028788	3638046
CR_03640C_A	-1.94241	0.014216	3643190
CKA2	-1.94276	0.017564	3643444
PEX13	-1.94567	0.006062	3641877
SMX4	-1.94645	0.122581	30515427
EHD3	-1.9485	0.054513	3636858
SNF2	-1.94892	0.012855	3635855
C4_06420W_A	-1.94973	0.005284	3642795
HRK1	-1.95422	0.0101	3640586
C1_04370C_A	-1.95433	0.012825	3647994
C2_07720C_A	-1.95458	0.035029	3642304
C7_00190W_A	-1.95552	0.100794	3637969
CR_01640C_A	-1.95717	0.295732	3640086
C3_00160C_A	-1.96162	0.005704	3640591
YKE2	-1.96183	0.023706	30515454
WAL1	-1.96279	0.006678	3647093
LSM6	-1.96384	0.06898	30515363
CR_08290W_A	-1.96409	0.020605	3642103
C1_09300C_A	-1.96543	0.470523	3634831
RIM15	-1.96643	0.00682	3637961
HGC1	-1.96835	0.124331	3639349
C2_09340W_A	-1.96859	0.724572	3644107
C1_13600W_A	-1.96859	0.724572	3636210
C1_12040W_A	-1.96859	0.724572	3642029
C1_09500W_A	-1.96859	0.724572	3634764
C1_06220C_A	-1.96859	0.724572	3647518
CR_01220W_A	-1.96859	0.724572	3640406
KRE62	-1.96859	0.724572	3641543
C6_04070C_A	-1.96859	0.724572	3647826
C2_10150W_A	-1.96859	0.724572	3643933
PGA28	-1.96859	0.724572	3641350
FGR37	-1.96859	0.724572	3641609
C4_05580C_A	-1.96859	0.724572	3641775
C4_03500C_A	-1.96859	0.724572	3646801
C3_03620C_A	-1.96859	0.724572	3645654
C1_03470C_A	-1.9688	0.724534	3636912
C2_02140C_A	-1.9688	0.724534	3635748
C1_05970W_A	-1.9688	0.724534	3639874

C2_01460C_A	-1.9688	0.724534	3635844
C1_12090C_A	-1.9688	0.724534	3642056
C2_08150W_A	-1.9688	0.724534	3642328
C1_09700W_A	-1.9688	0.724534	3634876
C4_00990W_A	-1.9688	0.724534	3635609
CR_08260C_A	-1.9688	0.724534	3642100
C4_02240C_A	-1.9688	0.724534	3635692
CR_04980C_A	-1.9688	0.724534	3646395
C5_03770C_A	-1.9688	0.724534	3647233
C4_03760W_A	-1.9688	0.724534	3645355
YCF1	-1.96881	0.011554	3636934
C5_03300C_A	-1.96947	0.028158	3646357
TRM12	-1.9701	0.031648	3641738
C2_09870W_A	-1.97188	0.03487	3643780
C6_03910C_A	-1.97222	0.084642	3640263
C2_10680W_A	-1.97394	0.015815	3641252
HGT2	-1.97688	0.024954	3644579
IFF11	-1.97908	0.552975	3640580
NAT4	-1.98008	0.034149	3635704
C2_07370W_A	-1.98249	0.02728	3645233
C5_03210C_A	-1.98449	0.255699	3646364
C2_05490W_A	-1.98498	0.241403	3643417
C7_03230C_A	-1.98503	0.255895	3641369
TFB3	-1.98507	0.023488	3644115
SFT1	-1.98663	0.077804	30515372
CR_07480W_A	-1.99203	0.00426	3646047
GLN3	-1.99213	0.003732	3636453
C7_01210C_A	-1.99477	0.005759	3645904
VID27	-1.99541	0.003217	3647894
GAP5	-1.99723	0.004119	3646954
C6_02230W_A	-1.99723	0.0041	3645866
C2_00220C_A	-1.99825	0.005486	3638902
C3_04410C_A	-2.00149	0.004855	3635178
C1_06660W_A	-2.00203	0.012379	3639542
C5_01290C_A	-2.0039	0.003701	3642647
C1_01590C_A	-2.00593	0.004717	3640223
SAP9	-2.00691	0.006509	3645625
C1_11930W_A	-2.00768	0.003847	3642002
C2_08020C_A	-2.00845	0.012185	3642308
EXO70	-2.01142	0.005289	3636948
SOG2	-2.01159	0.004354	3644475
HRQ2	-2.01194	0.029925	3647850
DUS4	-2.01806	0.005901	3641580
CR_02670C_A	-2.01947	0.011872	3642572
C3_04700W_A	-2.02096	0.025141	3635247
C7_01280C_A	-2.02171	0.036379	3645909
C1_10910C_A	-2.02224	0.022792	3644445

RSM22	-2.02325	0.004975	3642506
C7_01990C_A	-2.02427	0.017218	3636966
MAK5	-2.02453	0.007747	3643454
MUP1	-2.0259	0.013362	3642008
CR_08400C_A	-2.02638	0.021667	3642115
CR_03200C_A	-2.02644	0.003007	3645131
PBP2	-2.02724	0.00203	3640267
C2_02520W_A	-2.02732	0.027523	3635818
CTM1	-2.02772	0.013876	3638951
HST7	-2.02816	0.009443	3640953
C7_02450W_A	-2.02891	0.015745	3636972
CDR2	-2.03111	0.021471	3635265
NOP13	-2.03599	0.01204	3638469
C3_02030W_A	-2.03616	0.022447	3636580
RRS1	-2.03777	0.00743	3639352
KIN3	-2.03844	0.003586	3641199
C5_02440C_A	-2.03942	0.018329	3637728
C4_03090W_A	-2.04475	0.015717	3637926
C1_09840C_A	-2.04514	0.003061	3634848
POP2	-2.04564	0.006398	3640301
FGR3	-2.05082	0.003453	3641179
TPS1	-2.05217	0.005197	3646699
C4_04670C_A	-2.0534	0.004332	3641140
CAF16	-2.05789	0.007482	3646486
C4_05740C_A	-2.06224	0.003642	3641806
MPP10	-2.06305	0.005846	3647912
C2_08960C_A	-2.06375	0.025359	3645787
C3_07940W_A	-2.06408	0.011225	3647453
CR_10410C_A	-2.0648	0.0105	3638957
PHO86	-2.06601	0.028114	3642323
CR_06060W_A	-2.07491	0.018787	6335358
C7_00200W_A	-2.07647	0.008461	3637968
C4_00610W_A	-2.07755	0.007089	3645770
GPM2	-2.07839	0.012628	3644713
C6_02720C_A	-2.07924	0.010673	3639212
C2_05830C_A	-2.08218	0.007031	3647483
C7_01040C_A	-2.08629	0.013188	3638018
PFK1	-2.0865	0.00693	3636448
C7_02930C_A	-2.08811	0.005087	3641390
SIR2	-2.08973	0.01376	3638845
C4_02090C_A	-2.09029	0.01218	3635642
HCA4	-2.09087	0.006823	3637911
HPT1	-2.09711	0.017277	3643034
CR_09410W_A	-2.09875	0.027079	3641933
PDE2	-2.09975	0.00238	3636877
C3_05860C_A	-2.1034	0.023892	3641759
C2_02730W_A	-2.10419	0.001035	3643035

C4_01710C_A	-2.1045	0.000382	3635708
TOM1	-2.10483	0.00995	3642987
MSN4	-2.10508	0.005797	3634911
ARD	-2.10616	0.004188	3647906
PSD1	-2.10695	0.000321	3639382
IFF9	-2.10894	0.004688	3641008
NUP49	-2.11095	0.001046	3636173
ADAEC	-2.11307	0.008577	3637342
BGL22	-2.11531	0.020482	3641935
C2_05550W_A	-2.11627	0.040876	3645560
MAS1	-2.11696	0.001242	3636855
MBP1	-2.11904	0.001946	3635281
C3_06370C_A	-2.12001	0.004724	3642258
C5_01560C_A	-2.12116	0.003924	3647064
BUD5	-2.12445	0.003755	3647315
C5_04530W_A	-2.12758	0.007551	3636507
SPT6	-2.12804	0.007018	3643171
CNT	-2.1295	0.001613	3644071
CR_10400W_A	-2.12968	0.001943	3638956
RTG3	-2.13026	0.011656	3644423
C7_01400C_A	-2.13264	0.01123	3645893
TBP1	-2.13682	0.008026	3647675
YFH1	-2.14107	0.016041	3646853
C5_01490C_A	-2.14191	0.001346	3647058
OYE32	-2.14337	0.045547	3642919
HBR1	-2.14416	0.001706	3639295
HST2	-2.1451	0.029256	3640162
C1_01580W_A	-2.14515	0.005396	3640222
DCP2	-2.14569	0.001234	3642069
C3_00100W_A	-2.14869	0.008312	3640567
DIP2	-2.15241	0.000507	3640762
PTK2	-2.15331	0.000387	3641611
FMT1	-2.15562	0.004602	3646530
MET4	-2.15736	0.002854	3641219
C1_07130C_A	-2.16603	0.001167	3644462
C1_10440W_A	-2.17416	0.002387	3647180
C1_00460W_A	-2.17753	0.009102	3639316
C2_02800W_A	-2.18019	0.028335	30515096
C6_00360C_A	-2.1806	0.026689	3646083
MTR2	-2.18259	0.002861	30515167
GRR1	-2.18375	0.000655	3636392
C3_07290W_A	-2.18418	0.000535	3638430
NCS2	-2.18439	0.004201	3643216
CR_10280W_A	-2.18729	0.015808	3638934
C4_00050W_A	-2.18887	0.006489	3648060
C4_06660W_A	-2.19067	0.029888	3642809
TCC1	-2.19562	0.001644	3638524

C1_04590W_A	-2.1976	0.003979	30514987
CR_00460C_A	-2.19804	0.010868	3639726
POX1-3	-2.20107	0.002987	3636598
C2_06480W_A	-2.20147	0.003419	3646568
CR_05770W_A	-2.20203	0.02977	3646694
ZCF9	-2.20528	0.006962	3638869
RRP15	-2.20542	0.005255	3648397
GAL102	-2.20563	0.008455	3644585
C2_01680C_A	-2.20683	0.009509	3635744
PEP12	-2.20766	0.007301	3637737
PRS5	-2.20777	0.00057	3642296
C6_01550C_A	-2.20797	0.01179	3641653
C1_11000C_A	-2.20833	0.010304	3644422
C1_04120C_A	-2.21033	0.041047	3644701
C4_07220C_A	-2.21176	0.002595	3642955
CCH1	-2.21176	0.006414	3640175
PTR3	-2.2144	0.013475	3645428
C5_02280C_A	-2.21566	0.000973	3637712
WSC2	-2.21937	0.003051	3639225
C1_05270C_A	-2.22322	0.007307	3642476
C4_03350C_A	-2.22531	0.002612	3646794
IFH1	-2.22886	0.00341	3637770
TAF145	-2.23056	0.002582	3639486
FCR1	-2.23074	0.001213	3638542
C6_04550C_A	-2.23143	0.000392	3645976
C1_06820W_A	-2.23208	0.001225	3639493
CKS1	-2.23631	0.004931	3647732
C5_02590C_A	-2.24014	0.001639	3637723
C4_02510W_A	-2.25182	0.005055	3637865
DBP7	-2.25551	0.001145	3645903
RIO2	-2.25554	0.000806	3642066
C5_01530C_A	-2.25612	0.007122	3647062
VPS20	-2.25706	0.023993	3637427
C2_00360C_A	-2.25931	0.000759	3638825
C6_01340C_A	-2.26501	0.024099	30515331
HMO1	-2.26595	0.004853	3646685
LIG4	-2.26639	0.000435	3642986
C1_11610C_A	-2.26762	0.001328	3645345
CR_10730C_A	-2.26796	0.000918	3638998
PEX12	-2.26797	0.01138	3638862
ENA2	-2.27208	0.001582	3639291
GSY1	-2.27348	0.002784	3640435
C2_02960C_A	-2.27624	0.009592	3643011
C2_06660W_A	-2.27624	0.015157	3648418
PTP1	-2.2773	0.003892	3642093
ISA1	-2.27882	0.001327	3639174
C4_02110W_A	-2.27891	0.004852	3635673

CR_02420W_A	-2.27977	0.001195	3644926
KOG1	-2.28042	0.000425	3642488
C7_04090C_A	-2.28321	0.004212	3643135
ATO2	-2.28343	0.0068	3639845
C1_01840C_A	-2.28746	0.006536	3645432
BRO1	-2.28884	0.003481	3636590
SHA3	-2.29057	0.001917	3644580
C7_01020C_A	-2.29168	0.00354	3638020
HSM3	-2.294	0.00395	3646713
CNS1	-2.29604	0.001414	3639365
C2_08330W_A	-2.29729	0.004605	3648166
C6_00850W_A	-2.30229	0.003295	3644010
C1_11730W_A	-2.30238	0.001451	3645318
PSO2	-2.30341	0.045837	3641241
C1_13530W_A	-2.30426	0.000653	3636170
C7_03760W_A	-2.30585	0.011576	3647865
DAL9	-2.30681	0.013014	3645653
C1_06650W_A	-2.30826	0.003461	3639543
SDH1	-2.3089	0.001285	3642477
C3_07550C_A	-2.31162	0.000765	3638525
C2_00420W_A	-2.31216	0.003148	3638819
C1_14380C_A	-2.31322	0.003649	3643285
C1_09710C_A	-2.31364	0.001585	3634818
YAH1	-2.31544	0.00895	3638368
C5_03920C_A	-2.31918	0.005548	3647448
C4_04190C_A	-2.32496	0.239354	3646860
ORM1	-2.32556	0.003143	3640307
C3_03670W_A	-2.32608	0.000412	3645649
C3_00570C_A	-2.32774	0.000376	3640584
C5_05340W_A	-2.32804	8.21E-05	3636400
SEF1	-2.32849	0.000758	3644945
RMS1	-2.3304	0.001325	3646361
CR_09100C_A	-2.33388	0.048215	3641928
C3_07280C_A	-2.33491	0.003018	3638431
C2_09240C_A	-2.33525	0.021044	3644064
CAP1	-2.33648	2.68E-05	3636640
CR_07940W_A	-2.34169	0.012364	3644139
SMI1	-2.34211	0.003817	3640741
C4_04350W_A	-2.3478	0.003445	3646852
URK1	-2.35439	0.001588	3635186
PSA2	-2.3593	0.001645	3636159
POT1-2	-2.35979	0.059592	3638908
OPY2	-2.36119	0.000341	3640765
C2_02770W_A	-2.36453	0.000207	3643031
C1_02850W_A	-2.36482	0.00089	3636862
MAK16	-2.36949	0.001481	3646920
C5_03510C_A	-2.37061	0.004204	3646969

UCF1	-2.37137	0.001579	3648170
C4_00490W_A	-2.37201	0.001027	3645013
SSU72	-2.37902	0.000944	3645129
C3_03190C_A	-2.37966	0.004631	3638427
C3_05430W_A	-2.38648	0.004257	3643667
CR_04280C_A	-2.39062	0.025065	3640993
CR_07670W_A	-2.39259	0.004826	3647255
RRN11	-2.39546	0.001292	3645276
C3_04790W_A	-2.39798	0.010136	3635215
C2_09790W_A	-2.4024	0.003325	3643804
C2_00090W_A	-2.40328	0.0129	3647910
ISU1	-2.40998	0.000384	3637007
SIS1	-2.41028	0.000222	3644841
C1_04720W_A	-2.41328	0.003777	30514988
C2_09500W_A	-2.41328	0.003566	3643817
RIM9	-2.41663	0.015716	3644047
C4_04510W_A	-2.4167	0.000784	3641120
C3_05440C_A	-2.41697	0.014637	3643668
VPS70	-2.42406	0.002278	3637971
C4_07100C_A	-2.4249	0.000108	3642903
CR_02030C_A	-2.43221	0.000466	3640152
NAR1	-2.43334	0.001315	3634916
ZCF35	-2.43543	0.00203	3641740
ESA1	-2.43545	9.86E-05	3640518
ASH1	-2.43682	0.003317	3641249
CR_10470C_A	-2.43781	0.000102	3638979
C5_00800C_A	-2.43992	0.000204	3644126
SEH1	-2.44029	0.000344	3642290
PRN3	-2.44312	0.046672	3639878
REI1	-2.45118	0.000562	3647536
LAS1	-2.45123	0.000278	3644073
REP1	-2.45139	0.038682	3639708
C4_00810C_A	-2.4522	0.00935	3645742
C3_06160C_A	-2.45821	0.003027	3641766
C2_01310W_A	-2.4611	0.002319	3638788
C7_00030W_A	-2.4631	0.000552	3638004
LEU42	-2.46366	0.001516	3643808
IFM3	-2.46388	0.008101	3642335
C1_11690W_A	-2.46841	0.000381	3645322
RPM2	-2.46929	8.91E-05	3644790
PSP1	-2.47014	0.000298	3644551
C2_08390W_A	-2.47106	0.005873	3648252
NSA2	-2.47131	0.000255	3642259
UBC8	-2.47465	0.002715	3645433
STE4	-2.47481	0.029333	3637358
C3_07070C_A	-2.4754	0.002331	3638446
FAL1	-2.47613	0.000239	3639839

SAS10	-2.47698	0.001364	3637899
RGD3	-2.48354	9.06E-06	3648003
C1_13430C_A	-2.48398	0.030236	3636223
PEX4	-2.48419	0.00384	3636429
C2_04770W_A	-2.49093	0.012023	3647783
RPA12	-2.49177	0.027209	3643477
C3_00130C_A	-2.49251	1.48E-05	3640593
CKA1	-2.49276	0.000144	3639018
CWT1	-2.49352	0.000287	3647595
CR_05910W_A	-2.4936	0.003061	3646680
C6_04480C_A	-2.49371	0.000183	3645986
CR_10760C_A	-2.49544	0.018707	3638943
C7_03240W_A	-2.49574	0.003904	3641368
RNR3	-2.49631	0.001793	3647591
ACH1	-2.49908	0.003117	3643773
C7_02550C_A	-2.50027	0.003268	3637018
CR_03480W_A	-2.50061	0.117135	3643199
C2_09660W_A	-2.50394	0.003043	3643793
C1_01070C_A	-2.51388	0.031819	3640172
BLM3	-2.514	1.67E-05	3642310
C3_04620C_A	-2.51541	0.011143	3635155
C5_05360C_A	-2.51693	0.003066	3636402
C5_02190C_A	-2.51818	0.0007	30515303
HSX11	-2.52129	0.04105	3635660
FHL1	-2.52316	0.000649	3643493
CR_09440C_A	-2.52343	8.57E-05	3641937
C1_06750W_A	-2.52408	0.02268	3639506
CR_10770W_A	-2.52427	0.003679	3638944
C1_00580W_A	-2.53161	0.010171	3639361
C3_00630W_A	-2.53429	0.000303	3640576
C5_03260C_A	-2.5384	0.001447	3646360
C6_03210C_A	-2.53851	0.004916	3639178
C4_03370C_A	-2.54011	0.027376	3646796
CR_02970C_A	-2.54041	6.89E-05	3642603
C3_04540C_A	-2.54355	0.000619	3635262
FRE10	-2.54414	0.005541	3646841
HAL9	-2.54634	0.000338	3643757
C2_02270C_A	-2.54829	0.002565	3635805
SHY1	-2.54972	7.96E-05	3634824
FRE3	-2.54974	0.035721	3641789
MAF1	-2.551	0.000435	3642332
BBC1	-2.5519	0.000433	3646661
C5_03030W_A	-2.55526	0.017486	3637822
C6_03780C_A	-2.57089	0.001154	3640308
TAZ1	-2.57147	0.006144	3644108
EFG1	-2.57234	0.00017	3644145
SAM35	-2.57428	0.000656	3643329

CR_06650C_A	-2.5743	0.037205	3646620
CR_06970C_A	-2.58775	0.000768	3645736
SVF1	-2.59083	0.001308	3639289
C3_05790C_A	-2.59169	0.032368	3641753
C6_02650C_A	-2.59864	0.000838	3639223
CR_00630W_A	-2.59873	0.001158	3639690
HMS1	-2.59968	1.75E-05	3641570
C3_03300C_A	-2.59977	2.99E-05	3638361
ELP3	-2.60176	2.67E-05	3641747
CR_07190W_A	-2.60306	0.000193	3645269
CEF1	-2.60394	0.000722	3634808
LEU3	-2.60683	3.65E-05	3637757
ZCF29	-2.60722	0.000191	3641370
C6_04140C_A	-2.60835	0.000183	3647970
TSM1	-2.60844	0.001657	3645953
C2_09570C_A	-2.6091	0.032281	3643811
C4_02920W_A	-2.61177	6.83E-05	3637878
C1_08630W_A	-2.61208	8.41E-05	3634928
TBF1	-2.61257	6.85E-05	3637327
C2_10600W_A	-2.61387	0.000791	3641245
BUD22	-2.61389	0.000167	3640391
NOP4	-2.61749	7.82E-06	3647991
C2_06090W_A	-2.62393	0.000341	3644097
C2_04520C_A	-2.62612	0.037118	3645447
C1_03310W_A	-2.62871	0.00021	3636856
DCK2	-2.62964	0.01448	3637384
OYE2	-2.63297	0.000258	3641603
C6_02290C_A	-2.63317	6.54E-05	3645873
SMM1	-2.64078	0.000283	3643778
C1_06760C_A	-2.64207	5.05E-05	3639505
C4_03960W_A	-2.6428	0.000691	30515264
GNP1	-2.6485	0.000169	3646073
LTV1	-2.65115	0.000131	3639016
GDH2	-2.65399	0.000287	3642295
C3_02420C_A	-2.65734	0.001947	3638342
C1_01460W_A	-2.65768	0.000236	3640240
C3_07680W_A	-2.65899	5.79E-05	3638459
PTR22	-2.65988	0.000705	3645634
C2_00490W_A	-2.66072	0.000204	3638870
C3_00500C_A	-2.66382	0.001027	3640515
NOT5	-2.66576	9.58E-05	3640763
CST5	-2.67042	0.006033	3645981
RAD1	-2.67243	0.000161	3641192
C1_04220C_A	-2.67738	0.001768	3644721
C2_03500W_A	-2.67994	2.87E-06	3637345
KTI11	-2.68087	0.039913	30515125
SST2	-2.68091	0.022545	3637754

GCS1	-2.68131	0.000169	3640742
BUD21	-2.68163	6.27E-05	3640541
C6_04470C_A	-2.68355	1.86E-05	3645985
NAG3	-2.68385	0.00124	3645969
SIZ1	-2.68419	1.48E-06	3640219
C4_00680W_A	-2.6842	0.000388	3645753
C6_04410C_A	-2.68437	0.005758	3645950
EXM2	-2.68498	0.001897	3642929
ZCF27	-2.68524	3.56E-05	3635631
SFP1	-2.68764	1.34E-05	3635225
C1_10710C_A	-2.69347	0.028771	3644407
C1_02960C_A	-2.69393	5.52E-05	3636834
YWP1	-2.695	0.005442	3643915
C3_07650C_A	-2.6959	0.004132	3638488
IRE1	-2.69871	2.3E-05	3640726
AHR1	-2.69914	4.88E-05	3641733
HOL4	-2.70047	7.35E-06	3645409
ULP2	-2.70128	0.002261	3643196
C2_05750W_A	-2.70289	0.000178	3645577
C2_02820C_A	-2.70522	0.000223	3643027
SSN6	-2.70973	0.000343	3638518
BOI2	-2.70989	0.000104	3640404
GAL10	-2.71177	0.000238	3644583
C4_01420W_A	-2.71444	0.001342	3635626
C4_06620C_A	-2.71506	0.047354	3642813
GNP3	-2.71689	0.001527	3639027
TPO4	-2.71812	0.000371	3640956
C2_00740C_A	-2.71972	2.82E-05	3638912
FAB1	-2.72036	0.000478	3635766
C7_03500W_A	-2.72317	0.001565	3646246
HSE1	-2.72948	1.81E-05	3640407
C2_07430C_A	-2.73071	0.001014	3645201
C1_12370W_A	-2.7323	0.002972	3642061
OPT8	-2.73237	0.000137	3640266
C3_02900W_A	-2.73702	0.00542	3638337
RPF1	-2.73724	4.42E-05	3637890
CR_02960W_A	-2.7394	0.036113	3642591
CR_03910C_A	-2.7401	7.84E-05	3640954
C6_02150C_A	-2.74337	0.016329	3645888
ARO80	-2.74689	0.002019	3636808
CR_10040W_A	-2.74925	0.003812	3639034
UPC2	-2.75338	1.26E-05	3646489
C3_02910W_A	-2.75523	0.013889	3638378
WOR2	-2.75548	0.000597	3635230
CR_00160C_A	-2.76429	0.000777	3639706
C1_07660W_A	-2.77466	0.00265	3646656
C1_08770W_A	-2.77545	0.000955	3634788

C7_00330C_A	-2.77562	7.97E-05	3638042
PMA1	-2.78255	1.04E-05	3640531
C5_00780C_A	-2.78364	6.62E-05	3644124
C1_03450C_A	-2.7842	7.55E-05	3636910
C2_03550C_A	-2.78465	9.48E-05	3637338
C7_02160W_A	-2.78808	0.000562	3636994
MET2	-2.79232	1.63E-06	3640070
C7_03380W_A	-2.79402	0.00104	30515359
CR_08420W_A	-2.80093	1.1E-05	3642117
C3_07430W_A	-2.81192	1.01E-05	3638484
GPT1	-2.8122	0.041247	3647671
C7_00040C_A	-2.81729	0.006256	3638002
C2_00410C_A	-2.81974	2.78E-06	3638820
C3_07400W_A	-2.82172	0.000621	3638471
PPR1	-2.82715	0.000348	3636535
SOF1	-2.82721	4.16E-05	3640585
CR_04110W_A	-2.83237	0.000476	3641026
SPL1	-2.83746	3.3E-05	3638035
C1_12860C_A	-2.8411	0.017022	3636179
C1_02210W_A	-2.84805	0.000167	3644594
C2_07060W_A	-2.85757	0.000477	3643500
CTR1	-2.86056	0.000338	3647077
MUC1	-2.86726	4.82E-06	3645771
C7_01610W_A	-2.86775	0.002001	30515353
CR_01630C_A	-2.86833	0.034728	3640085
MSB2	-2.87722	4.93E-07	3635830
C5_04180W_A	-2.88729	0.014631	3636544
DUR32	-2.89019	0.010434	3636142
CR_03410W_A	-2.89027	0.000966	3645136
C3_03740W_A	-2.89318	0.020356	3645640
OFR1	-2.8986	0.001538	3640710
C4_05440C_A	-2.89881	4.42E-06	3646960
CR_07920W_A	-2.90114	1.15E-05	3644141
UTP9	-2.90381	3.88E-06	3638451
C6_04240W_A	-2.907	0.009237	3646276
C6_04510C_A	-2.91476	0.000146	3645955
DFI1	-2.92448	1.45E-05	3638038
CCE1	-2.92621	0.047839	3640395
C1_13130C_A	-2.92671	6.39E-06	3636202
MDN1	-2.93423	0.000209	3635614
RFX1	-2.93478	0.000956	3644844
C1_11080W_A	-2.93547	7.5E-06	3644416
C3_06270C_A	-2.93553	0.000295	3642239
CR_10420W_A	-2.9374	4.45E-06	3638974
C7_01510W_A	-2.94561	3.07E-05	3637049
ATS1	-2.94667	5.91E-05	3642079
C2_00640W_A	-2.95039	0.001128	3638847

C1_13390W_A	-2.95819	0.003727	3636218
C3_06420C_A	-2.95845	6.44E-05	3642262
C3_01090W_A	-2.96035	0.004311	30515176
C1_07810C_A	-2.96054	0.00402	3640708
CPH2	-2.96079	5.32E-05	3646067
CR_09800C_A	-2.96237	5.41E-07	3638984
CR_02100C_A	-2.96536	0.001284	3640141
SBA1	-2.9697	4.34E-06	3640305
C4_01960C_A	-2.973	3.65E-06	3635664
C5_02580W_A	-2.97582	0.000476	3637721
C4_01860C_A	-2.9782	0.000248	3635590
C2_08530C_A	-2.98337	0.001414	3643921
C2_09000C_A	-2.98856	0.023147	3645782
C5_04120C_A	-2.99948	9.24E-05	3636522
C2_00650W_A	-3.00008	3.34E-05	3638889
RNR22	-3.0021	2.4E-05	3645217
BUD14	-3.00389	1.4E-06	3643469
C1_12580W_A	-3.00455	3.88E-05	3647603
RPN4	-3.00929	1.81E-05	3644684
CR_08040W_A	-3.01008	3.21E-05	3644135
BNA4	-3.01472	1.07E-06	3640528
STD1	-3.01568	5.46E-05	3647459
MNN4	-3.02017	6.49E-06	3642784
PRP5	-3.02495	9.5E-07	3638447
CR_06980W_A	-3.02599	0.011727	3645735
C1_05540C_A	-3.02832	5.57E-05	3642503
C1_10580C_A	-3.03672	0.011782	3647679
RTA3	-3.03858	0.000263	3646566
C4_06040W_A	-3.04274	0.001713	3646521
C1_07640C_A	-3.04639	0.001801	3646659
PGA55	-3.05375	1.04E-05	3645784
CET1	-3.05661	1.25E-05	3640140
CCC2	-3.05678	1.02E-05	3637821
C2_02110C_A	-3.05883	0.004524	3635745
IML1	-3.06116	0.000103	3636813
DUG3	-3.06336	3E-05	3641381
CR_08410W_A	-3.06399	4.52E-06	3642116
C4_03580W_A	-3.06418	0.012959	3645369
DAL5	-3.07459	0.004756	3638401
C6_02970C_A	-3.07486	7.37E-05	3639190
C6_03290W_A	-3.08025	2.16E-06	3639195
BAS1	-3.08123	1.95E-06	3641136
C6_04050W_A	-3.08138	0.000241	3647830
C2_02570W_A	-3.08223	0.00556	3635858
C1_05920W_A	-3.08676	0.03914	3639864
MNL1	-3.09105	6.15E-07	3646050
C2_05060C_A	-3.09119	0.008025	3643451

PUT4	-3.09678	0.036166	3636801
CR_00810W_A	-3.09762	0.001028	3640415
CR_03120W_A	-3.10443	4.11E-06	3645123
LEU5	-3.11571	3.68E-06	3638838
C4_00460C_A	-3.11583	1.26E-06	3645010
ARG81	-3.11718	1.33E-06	3634775
CR_03980W_A	-3.11783	0.000112	3640964
CR_04710W_A	-3.11805	0.017959	3648141
SGD1	-3.11843	2.91E-06	3643191
DFG16	-3.1266	1.3E-05	3637413
C3_05360C_A	-3.12733	0.000189	3643676
C1_10950C_A	-3.13157	1.46E-05	3644428
C6_01450C_A	-3.13535	0.00014	3641624
PDX3	-3.13559	2.62E-06	3640949
C4_00690C_A	-3.13695	9.11E-06	3645752
C6_03590C_A	-3.14051	0.002682	3640251
VPH2	-3.14108	0.005547	3642021
C3_02840W_A	-3.14244	0.005364	3638331
C1_05200C_A	-3.14579	3.14E-05	3642466
C2_09710C_A	-3.146	0.000295	3643786
C1_12750C_A	-3.14654	0.000282	3646818
SSY5	-3.14988	8.58E-07	3642140
FZO1	-3.15482	3.29E-06	3646848
CR_03730C_A	-3.15542	8.59E-07	3643226
C2_06520C_A	-3.15605	0.000753	3646571
C3_02630C_A	-3.15753	2.05E-05	3638328
CR_05440W_A	-3.162	0.000155	3648245
TSR2	-3.16696	0.000127	3636845
C2_05840W_A	-3.19559	0.000116	3647484
SPT5	-3.1979	3.68E-06	3635847
SFT2	-3.20094	0.000115	3644442
ABC1	-3.20175	2.6E-06	3640239
GLK1	-3.20235	0.001055	3648348
CR_03360W_A	-3.20755	1.09E-06	3645141
C3_02040C_A	-3.20761	2.03E-05	3636579
C1_11670W_A	-3.2142	2.74E-05	3645338
C1_05790W_A	-3.2151	0.002562	30515002
SEC2	-3.22156	2.59E-06	3636119
C4_02570C_A	-3.22613	0.001099	3637916
SPO7	-3.22704	0.000118	3645576
C2_00190C_A	-3.22905	9.51E-07	3638904
NBP2	-3.23079	1.97E-06	3636963
C4_00060W_A	-3.2315	2.59E-05	3648061
CDG1	-3.23197	3.9E-05	3641883
SSK1	-3.23502	5.09E-05	3636184
IFF4	-3.23993	0.003138	3639694
KTI12	-3.2405	7.28E-05	3645140

C3_04720C_A	-3.24168	5.09E-05	3635249
C5_01710C_A	-3.24198	4.78E-05	3643732
CDC34	-3.24231	9.71E-07	3637028
C2_10090C_A	-3.24421	1.38E-05	3643939
CR_02550C_A	-3.24458	1.51E-05	3647689
C4_02770C_A	-3.24678	0.000327	3637903
CR_01710W_A	-3.24956	0.000471	3640130
C3_00940W_A	-3.25243	1.63E-08	3639851
PHHB	-3.25752	0.00047	3638871
PAN6	-3.26445	2.33E-05	3647684
C5_00390C_A	-3.26816	4.01E-06	3641552
VPS27	-3.27556	2.13E-07	3639326
PTP2	-3.28337	1.27E-06	3647858
NDT80	-3.28545	1.78E-06	3638840
NOP6	-3.28855	4.95E-06	3639507
MRP17	-3.28887	4.45E-06	3641548
C3_00010C_A	-3.28897	0.015649	3640600
VPS24	-3.29658	2.6E-06	3638892
ISY1	-3.29663	0.0009	3646233
FET34	-3.30118	0.000158	3647131
C6_00530C_A	-3.30576	0.002757	30515326
CTA8	-3.31656	2E-06	3634947
RTG1	-3.31852	3.17E-05	3634929
C1_09850C_A	-3.32323	9.47E-08	3634849
HNM1	-3.32945	2.19E-05	3638857
RGT1	-3.33215	3.72E-05	3637920
C6_04530C_A	-3.34818	4.68E-06	3645970
SLK19	-3.35099	4.26E-05	3638466
C6_01620W_A	-3.35334	4.66E-05	3641647
C2_00600C_A	-3.36052	3.99E-07	3638850
MRF1	-3.36066	0.000176	3645321
RFG1	-3.36589	3.17E-09	3642570
C7_01940C_A	-3.36711	6.59E-06	3636971
AXL1	-3.3694	0.002628	3641938
C5_03740W_A	-3.37007	1.43E-05	3647237
NPR2	-3.37235	1.4E-05	3638360
MLH3	-3.37247	3.48E-06	3643294
ZCF28	-3.37537	0.000143	3634776
C3_07490W_A	-3.37852	3.72E-07	3638528
CYS3	-3.38083	4.58E-06	3642081
SLR1	-3.3828	3.06E-07	3643957
C2_02920W_A	-3.39798	9.51E-07	3643016
PUF3	-3.40293	3.39E-07	3646950
C1_04710C_A	-3.40661	1.27E-05	3644764
C1_04360C_A	-3.4113	2.34E-05	30514985
KAR4	-3.41267	0.007962	3644937
NPR1	-3.41627	8.83E-08	3639498

CUP2	-3.41753	4.75E-07	3636189
CIP1	-3.43046	0.002363	3644018
C3_07580W_A	-3.43239	3.69E-05	30515225
CR_01090W_A	-3.43298	1.63E-06	3640379
IFF3	-3.43303	3.45E-06	3643189
MTM1	-3.435	1.57E-05	3642487
PGA4	-3.44206	3.61E-07	3636406
FMP27	-3.44414	2.28E-07	3641645
CR_04350C_A	-3.44757	2.09E-06	3641001
C1_10120C_A	-3.45514	6.94E-08	3634779
RVS161	-3.45826	3.15E-08	3638005
C2_00280C_A	-3.47503	0.00026	3638802
CR_10800C_A	-3.47634	1.45E-06	3638948
OLE2	-3.47955	1.96E-07	3643503
C1_03120W_A	-3.48029	6.76E-08	3636926
C3_06700C_A	-3.48747	5.08E-06	3642247
C6_04300W_A	-3.49055	1.84E-05	3646286
OSM1	-3.49537	8.51E-06	3645573
GAL1	-3.51389	2.71E-06	3644581
BPH1	-3.51642	0.000252	3639547
CR_06290C_A	-3.52033	5.44E-05	3644856
C2_02280W_A	-3.52218	2.38E-07	3635806
YAK1	-3.52479	7.85E-08	3647801
C2_00770W_A	-3.52838	8.47E-05	3638909
C2_01060C_A	-3.53756	0.000546	3638835
RPD3	-3.55096	1.52E-06	3642580
C5_00100C_A	-3.5519	0.000346	3647883
C3_03370C_A	-3.55802	8.39E-07	3638366
CR_05730C_A	-3.56523	8.36E-08	3646698
C3_01770C_A	-3.57855	1.32E-05	3636588
C5_01730W_A	-3.5833	0.003895	3643730
C2_08340C_A	-3.59143	0.035798	3648165
CR_01600C_A	-3.59448	2.99E-05	3640080
C1_12150C_A	-3.5945	0.003149	3642049
C3_06820C_A	-3.60775	7.37E-07	3638544
FRP1	-3.60838	4.14E-07	3645005
CR_03160W_A	-3.61337	7.38E-05	3645117
C5_02070C_A	-3.61445	8.54E-08	3643746
NCR1	-3.61822	9.49E-06	3643314
ZCF30	-3.62608	2.43E-07	3642051
HNM3	-3.62682	0.002168	3640093
DES1	-3.63975	9.19E-09	3636529
C2_10790C_A	-3.64317	7.42E-06	3641242
MDJ1	-3.64647	9.15E-09	3646980
SPT14	-3.64673	1.13E-07	3641019
PGA62	-3.65	9.62E-07	3637879
COX17	-3.65193	0.001771	30515088

CR_07250C_A	-3.65595	5.29E-05	3646028
VPS13	-3.68002	1.03E-05	3646528
TCO89	-3.68285	1.37E-08	3644788
GPR1	-3.68882	2.18E-07	3642616
C2_05820W_A	-3.70078	4.46E-07	3647482
CR_00310C_A	-3.70177	5.83E-06	3639715
C1_07790C_A	-3.70628	6.52E-06	3640706
C1_05250W_A	-3.71186	0.008931	3642462
HGT6	-3.71712	1.58E-06	3638873
C2_02530W_A	-3.7224	0.048128	3635819
C2_05770W_A	-3.73945	6.5E-06	3645579
SEN2	-3.74345	0.000402	3637832
C6_02930W_A	-3.75035	1.2E-08	3639243
HBR3	-3.75419	9.54E-06	3645652
SNT1	-3.75755	3.13E-09	3642033
HOS3	-3.75996	8.73E-08	3637886
RGS2	-3.76803	6.32E-06	3646629
C1_14480W_A	-3.77371	8.61E-05	3643328
FDH1	-3.7744	0.003909	3646398
GRF10	-3.77969	2.77E-10	3636492
C1_13010W_A	-3.78025	5.73E-08	3636120
BMT5	-3.78181	3.82E-08	3635781
CR_04920W_A	-3.78355	5.39E-08	3648207
C2_03570C_A	-3.7862	0.001923	3637336
C1_11580W_A	-3.78669	5.51E-07	3645330
OPT3	-3.78926	3.22E-08	3644918
MNN4-4	-3.79032	0.000792	3639250
CHR1	-3.81794	1.59E-09	3647601
RRN3	-3.83161	5.49E-07	3647925
CR_07140C_A	-3.84032	0.00021	3648227
QDR3	-3.84046	4.58E-06	3644023
C6_02980C_A	-3.84497	2.74E-09	3639192
DRE2	-3.85269	1.59E-07	3642571
C1_01510W_A	-3.85794	0.001399	3640229
RCA1	-3.86009	1.5E-08	3639303
C2_07250C_A	-3.868	0.008835	3643487
HAP41	-3.86947	2.06E-08	3647405
C5_04860C_A	-3.87126	0.014919	3636437
C1_10230C_A	-3.87971	3.12E-07	3634880
PMC1	-3.88024	1.04E-07	3636627
PNC1	-3.88964	1.09E-05	3646232
C1_08840W_A	-3.89773	0.018907	3634919
C5_04080C_A	-3.89773	4.68E-08	3647731
C2_10540W_A	-3.89933	1.88E-07	3641200
C4_04460C_A	-3.90485	3.53E-08	3641127
C2_06800C_A	-3.90943	7.9E-06	3643518
ATX1	-3.91919	0.001086	30515426

C6_03460W_A	-3.9224	4.82E-09	3640252
C1_14250C_A	-3.92394	1.85E-06	3643307
MOH1	-3.93594	4.55E-09	3646809
CR_03660C_A	-3.95153	2.82E-06	3643219
C1_14580C_A	-3.95559	1.91E-07	3643290
C1_03750W_A	-3.96997	5.1E-06	3644690
C5_00570W_A	-3.97325	5.08E-07	3641589
C3_00410C_A	-3.97475	0.014055	3640552
FGR23	-3.97775	1.55E-08	3636555
COX19	-3.99665	2.51E-06	3636220
C2_10050W_A	-3.99684	1.45E-07	3643949
NAB3	-3.99712	3.45E-07	3639209
C1_01150C_A	-4.00193	7.37E-08	3640203
NBN1	-4.00261	2.04E-07	3637410
C2_08850C_A	-4.00787	1E-07	3643890
IFR1	-4.01269	0.003147	3643930
C3_05800W_A	-4.01411	1.07E-08	3641754
CR_03870W_A	-4.01501	0.000346	3643187
POP3	-4.02039	6.84E-05	3638993
C2_02300W_A	-4.03182	1.73E-05	3635808
CR_07910C_A	-4.03894	6.5E-07	3644142
CR_10000C_A	-4.04315	0.001822	3639030
C2_06510W_A	-4.04651	5.66E-06	3646570
C4_00260W_A	-4.054	1.49E-10	3645037
CCS1	-4.05545	1.08E-09	3644476
C1_07560W_A	-4.06265	2.85E-05	3646676
UTP22	-4.06323	2.6E-09	3635760
C2_00200W_A	-4.07083	9.66E-08	3638903
C3_02890C_A	-4.07491	0.00954	3638336
C1_09790C_A	-4.08791	7.14E-09	3634843
C4_04560C_A	-4.09517	2.6E-09	3641105
C7_02110W_A	-4.09929	8.07E-10	3637022
C2_10810W_A	-4.10949	5.63E-09	3641243
ALP1	-4.11888	1.75E-09	3644437
ADR1	-4.13077	1.07E-07	3637866
TOK1	-4.13924	4.09E-08	3645754
CR_09460C_A	-4.14043	1.57E-05	30515453
SOK1	-4.14444	4.37E-07	3642495
C3_07370W_A	-4.15015	7.3E-08	3638474
C6_00170C_A	-4.15085	4.95E-10	3647903
C4_03000C_A	-4.15111	9.4E-07	3637935
VPS21	-4.1569	1.74E-09	3642092
MLP1	-4.15919	5.24E-08	3635688
SUT1	-4.16506	7.88E-06	3637706
PUT2	-4.17366	4.08E-09	3636439
CYC3	-4.17515	1.16E-09	3642621
CR_08720W_A	-4.17784	0.00017	3642122

DAC1	-4.18984	2.29E-07	3645967
C1_11020W_A	-4.19093	4.87E-05	3644420
PZF1	-4.20689	9.35E-07	3648113
CR_04600W_A	-4.21645	3.94E-07	3640950
C1_11290W_A	-4.22358	0.000205	3644553
C7_01150W_A	-4.23968	2.17E-05	3645913
PGA7	-4.25526	1.13E-08	3645006
PNG2	-4.25543	3.38E-06	3645568
C6_03440W_A	-4.25564	2.27E-08	3640274
PTC8	-4.26244	2.02E-09	3635674
OPT7	-4.27566	0.002623	3645008
HGT4	-4.2869	2.95E-06	3635269
TRY6	-4.30119	0.017559	3638499
C6_04180W_A	-4.30404	4.55E-07	3648176
C4_02520C_A	-4.30735	1.83E-09	3637863
C7_00260C_A	-4.30994	1.63E-07	3637978
REG1	-4.3174	2.96E-07	3638858
C3_07670W_A	-4.32211	3.59E-10	3638460
C1_06510C_A	-4.32512	1.64E-08	3639549
CR_03710C_A	-4.35504	3.38E-08	3643224
CR_07830C_A	-4.35677	4.29E-13	3647345
C3_01430W_A	-4.35966	8.49E-06	3636633
C2_00540W_A	-4.37058	1.56E-07	3638865
C4_06080W_A	-4.37157	0.007363	30515280
C3_03000W_A	-4.37269	0.00868	3638389
C2_04280W_A	-4.37807	3.2E-05	3637326
CR_08680C_A	-4.38055	1.09E-10	3642127
PRB1	-4.38752	3.84E-09	3643521
C1_08700W_A	-4.40136	3.09E-11	3634936
C1_11680C_A	-4.40709	0.001036	30515056
C6_03200W_A	-4.41261	2.52E-08	3639177
C7_03410C_A	-4.41577	0.01298	3646716
HRR25	-4.4217	1.97E-10	3645878
SOD3	-4.42419	4.38E-09	30515347
ZCF16	-4.43604	0.000294	3647395
C2_00940W_A	-4.43785	5.78E-11	3638891
C2_04850C_A	-4.44474	4.51E-09	3648114
C2_06600W_A	-4.44618	1.35E-09	3646563
C5_03870C_A	-4.46123	8.33E-06	3647223
THI20	-4.46258	0.000377	3637387
HIT1	-4.4635	0.000527	3637854
NAG6	-4.46441	1.61E-10	3645961
C6_00380C_A	-4.46534	1.24E-06	3646085
MIH1	-4.46554	8.56E-07	3648188
BRE1	-4.46885	1.94E-10	3641568
ZCF3	-4.46886	6.75E-11	3645335
C4_04010W_A	-4.47888	0.000195	3641218

HNT1	-4.4894	2.6E-08	3644441
C3_05850W_A	-4.51835	1.72E-05	3641758
AUR1	-4.53177	2.32E-11	3642617
TRY3	-4.53666	2.51E-07	3642652
C3_02710W_A	-4.55663	3.35E-07	30515190
RPC10	-4.56698	4.37E-09	3643292
FBP1	-4.58184	7.92E-09	3647464
PRN1	-4.58214	0.000408	3639882
GCV2	-4.58414	3.01E-08	3646505
C6_00700C_A	-4.59656	6.21E-07	3647074
MED3	-4.60005	2.2E-05	3646522
C1_08350C_A	-4.60138	5.44E-08	30515030
CR_08080W_A	-4.60335	0.014188	3642094
CR_08580C_A	-4.60745	3.07E-06	3642105
LPI9	-4.61099	1.37E-10	3637060
VPS28	-4.61749	1.05E-07	3645794
PDR16	-4.62706	5.3E-06	3644679
C1_03270W_A	-4.63134	1.31E-08	3636900
BUL4	-4.63398	2.61E-06	3642037
XYL2	-4.63612	6.96E-06	3638961
UGT51C1	-4.64188	1.42E-07	3640147
C6_03800C_A	-4.6447	1.03E-11	3640310
MEP2	-4.66073	9.85E-08	3645022
DEF1	-4.66224	2.35E-09	3639023
ADA2	-4.69729	1.65E-07	3644450
FLO8	-4.69955	5.6E-12	3646292
TYE7	-4.71144	3.35E-09	3636203
TRK1	-4.71441	1.93E-09	3644153
C7_03970C_A	-4.71627	4.21E-07	3643117
CR_02930W_A	-4.74421	1.19E-07	3642588
HAP2	-4.74629	2.62E-12	3641800
CR_05540C_A	-4.74883	1.72E-09	3647594
C2_06570C_A	-4.75852	1.77E-10	3646561
WOR4	-4.76343	2.09E-10	3638454
C3_06560W_A	-4.76658	6.99E-11	3642276
CDR1	-4.7784	7.68E-11	3635237
ALS5	-4.77997	1.57E-08	3640277
C7_00590W_A	-4.78632	2.72E-08	3637994
DYN1	-4.78687	1.33E-06	3635236
RXT3	-4.78808	2.94E-08	3643431
ARG8	-4.80505	1.25E-05	3641131
C2_10170C_A	-4.81615	1.12E-10	3643931
C1_10060C_A	-4.84443	0.000556	3634812
RAS2	-4.85772	2.15E-11	3635211
C3_04510W_A	-4.86076	0.000297	3635258
NCE102	-4.8694	1.9E-09	3635267
CAN3	-4.91441	3E-10	3647066

C1_11410C_A	-4.9269	0.001036	30515054
RIM21	-4.93111	6.13E-09	3643739
C1_05330C_A	-4.94098	2.47E-08	3642485
C1_11620W_A	-4.94915	0.001672	3645344
C2_05810W_A	-4.9523	3.49E-12	3647481
C6_03370W_A	-4.95289	1.52E-10	3639230
C1_01890C_A	-4.96243	3.75E-12	3645427
C1_13880C_A	-4.96506	6.07E-11	3636133
STP2	-4.96921	3.46E-10	3636214
C2_04110W_A	-4.98027	0.000722	3637378
C3_05450C_A	-4.98283	4.96E-11	3643669
C5_02020C_A	-4.98346	3.18E-09	3643771
CBF1	-4.98586	1.3E-06	3642779
SEN15	-4.99851	0.000187	3646411
C1_09320C_A	-4.99997	5.11E-11	3634834
C3_05250C_A	-5.0065	1.89E-06	3635199
PGA41	-5.0069	0.00277	3642800
PRA1	-5.0073	0.002266	3642969
SLM2	-5.01683	2.93E-16	3647559
HGT18	-5.02615	0.000348	3645111
C2_07580W_A	-5.04095	0.000224	3645216
SOD6	-5.06723	7.5E-06	3638899
NAG1	-5.08907	8.24E-09	3645966
FGR13	-5.09258	3.27E-05	3648078
EBP1	-5.11369	1.09E-09	3644060
C1_13820C_A	-5.11906	3.74E-09	3636127
C4_05970W_A	-5.12328	1.34E-11	3646514
OYE23	-5.1316	0.001855	3641618
C3_07470W_A	-5.14372	0.046846	3638479
GZF3	-5.15622	1.82E-13	3642563
CR_02910W_A	-5.15804	3.56E-11	3642586
C5_04170W_A	-5.15816	2.69E-14	3636543
CR_09140C_A	-5.15927	2E-06	3641879
FUN31	-5.16009	7.59E-10	3642249
PDR6	-5.16412	1.07E-08	3638824
ATG15	-5.16683	2.9E-13	3641641
C1_03140W_A	-5.18093	3.23E-08	3636929
C2_07640W_A	-5.18695	1.97E-11	3645192
C1_14330W_A	-5.21325	1.4E-08	3643278
C7_01700W_A	-5.23514	7.38E-10	3637013
C4_01970W_A	-5.24072	1.11E-09	3635663
C3_02980C_A	-5.24274	5.59E-05	3638386
PRP8	-5.24567	1.92E-12	3642125
C2_05990C_A	-5.25356	0.001603	3644074
C4_03020W_A	-5.25794	7.56E-14	3637934
C4_07040W_A	-5.2593	0.001413	3642963
C1_07220W_A	-5.26279	5.44E-10	3644460

C1_00860W_A	-5.27918	3.99E-05	3639358
C1_01140C_A	-5.28358	8.02E-09	3640202
C4_00080C_A	-5.2851	8.25E-06	3648065
FTR1	-5.31194	1.79E-12	3643318
OPT1	-5.31497	8.83E-05	3640150
YME1	-5.36412	4.3E-09	3641816
C1_09130W_A	-5.36952	6.13E-09	3634943
C7_03550C_A	-5.3795	0.026176	3646235
POL93	-5.38109	6.02E-11	3639299
HEM3	-5.38753	1.04E-10	3643940
CAG1	-5.39537	6.01E-05	3636469
CPA2	-5.39742	2.84E-07	3640385
CR_07820W_A	-5.39745	0.022579	3647344
C1_10140C_A	-5.40877	1.59E-09	3634780
CR_10060W_A	-5.41056	9.54E-14	3639035
C2_03110W_A	-5.41911	0.00107	3642995
UGA3	-5.42906	9.29E-13	3638926
C6_00290W_A	-5.43919	5.57E-12	3646069
C7_03280C_A	-5.44915	1.05E-09	3641353
RAD3	-5.47842	2.89E-14	3638014
C2_07280W_A	-5.48795	0.002199	3643475
IHD1	-5.50418	4.31E-06	3640291
TOR1	-5.51965	4.15E-12	3643480
ADH5	-5.5265	4.11E-07	3640156
C1_06840C_A	-5.54102	1.52E-12	30515012
C2_06740W_A	-5.55208	1.12E-09	3648024
C6_02020C_A	-5.56123	3.92E-09	3647563
SPO72	-5.57868	7.02E-10	3644072
NBP35	-5.63134	2.24E-13	3647412
HSK3	-5.63864	2.3E-07	3635730
FAV1	-5.65279	0.005068	3641115
C2_01660C_A	-5.66278	3.06E-09	3635742
C1_05370C_A	-5.67236	1.21E-10	3642481
C2_08030W_A	-5.68619	1.14E-06	3642309
AMS1	-5.71329	3.81E-10	3637882
ATG9	-5.71849	7.95E-10	3635239
C4_04660C_A	-5.72139	2.8E-13	3641150
DED1	-5.72961	3.79E-09	3641752
SCH9	-5.74413	4.66E-12	3637421
QDR2	-5.76669	1.28E-12	3643661
PFK26	-5.7832	3.17E-09	3634912
C2_03760C_A	-5.81621	3.95E-16	3637371
YPT53	-5.82035	9.9E-10	3642483
ALS9	-5.83574	1.29E-10	3640281
IFF6	-5.85001	3.57E-14	3644087
C1_05930C_A	-5.85653	2.19E-14	3639862
C1_01630W_A	-5.85913	5.46E-13	3640194

C4_05590W_A	-5.87381	5.71E-13	3641776
HSP70	-5.88316	9.59E-07	3636229
CEK2	-5.90117	7.52E-05	3642459
SPO11	-5.90347	0.000859	3643873
CR_00750C_A	-5.9058	8.78E-09	3640438
C4_00100C_A	-5.90667	2.31E-15	3645004
C3_00620C_A	-5.9475	3.67E-10	30515171
SLD1	-5.97665	3.74E-10	3638406
MFG1	-5.98025	2.16E-10	3643884
GST2	-6.00732	2.15E-12	3637937
MSN5	-6.01499	5.07E-09	3637889
SDS24	-6.03496	1.37E-11	3640756
ARR3	-6.0665	3.01E-06	3642910
C7_00340C_A	-6.06805	4.31E-10	3638040
SFC1	-6.06998	2.58E-06	3636413
CR_02510W_A	-6.09883	3.75E-12	3647694
CRG1	-6.14729	5.49E-06	3646393
ALS6	-6.17359	6.75E-09	3642266
ATO10	-6.17659	0.006942	3640394
DHH1	-6.17857	4.49E-10	3639490
C1_01710W_A	-6.18305	3.29E-17	3640201
C1_14350W_A	-6.20825	7.47E-16	3643281
CPA1	-6.24631	4.27E-08	3635715
PEX5	-6.25	1.17E-15	3645026
CR_02800C_A	-6.25187	4.24E-09	3642559
TUS1	-6.26582	9.64E-13	3646144
C1_11270W_A	-6.29682	1.98E-10	3644555
C4_02690W_A	-6.36921	3.1E-11	3637861
CR_06550C_A	-6.3736	1.28E-12	3645260
C5_03150W_A	-6.37434	4.1E-14	3637799
C1_12630C_A	-6.3939	2.64E-06	3646838
FYV5	-6.39688	2.61E-06	3647057
SNQ2	-6.40419	9.76E-18	3640290
C1_00090W_A	-6.42704	6.96E-05	3639304
SUA71	-6.53064	5.79E-14	3636879
C2_02550C_A	-6.53993	0.002804	3635856
C2_05640W_A	-6.54959	7.04E-17	3645566
C1_13250W_A	-6.56183	5.45E-11	3636143
C6_02100W_A	-6.57287	0.004913	3647554
SSU81	-6.62856	5.97E-09	3634944
DAL8	-6.67359	7.96E-06	3635181
C1_09240C_A	-6.67651	0.015471	3634901
C6_01590W_A	-6.69691	1.53E-18	3641650
C4_01220C_A	-6.70302	5.1E-06	3635650
C3_01540W_A	-6.74694	4.51E-05	3636567
AUT7	-6.75164	2.17E-16	30514999
C3_02920W_A	-6.75239	1.95E-12	3638379

C1_06090C_A	-6.77062	2.03E-12	3639822
WSC1	-6.77105	2.65E-14	3635138
C6_00730W_A	-6.77739	0.037757	3647070
GIT1	-6.77924	0.024421	3646562
C5_02680W_A	-6.79835	9.64E-17	3637774
WAR1	-6.80037	5.39E-11	3644691
C5_04850W_A	-6.80456	3.22E-05	3636436
C2_09820W_A	-6.81182	1.49E-06	3643801
C2_04090W_A	-6.8156	0.043305	3637380
DIP5	-6.82386	2.28E-11	3636798
FET31	-6.84112	1.6E-09	3647151
SLY41	-6.85822	2.19E-16	3647139
C1_09520C_A	-6.89232	3.41E-15	3634766
C1_09650W_A	-6.90898	3.13E-18	3634870
C2_09610W_A	-6.94219	2.34E-12	3643798
C5_04840C_A	-6.9469	8.19E-13	3636435
RGA2	-6.95514	1.99E-17	3635661
AOX2	-6.96816	1.55E-13	3634945
PIR32	-6.97559	0.048267	3646670
CR_08310C_A	-6.97604	0.040704	3642078
C6_00980C_A	-6.98769	3.92E-06	3644046
GYP7	-7.01472	1.79E-18	3646245
C3_04920C_A	-7.03003	1.4E-16	3635268
MET1	-7.04965	2.42E-15	3643014
CR_08470W_A	-7.10875	4.06E-15	3642134
C2_10130W_A	-7.15177	0.001576	3643935
COX11	-7.18757	5.04E-12	3646842
C4_05160C_A	-7.19067	3.11E-16	3647411
HGT19	-7.19428	8.8E-10	3640558
MIT1	-7.23386	2.01E-15	3644089
LIP4	-7.26935	3.66E-08	3645987
AAF1	-7.28012	1.95E-18	3642284
C5_02980C_A	-7.29834	4.3E-07	3637816
SEO1	-7.29898	0.000377	3646621
SWE1	-7.30496	1.89E-10	3634866
MDM34	-7.30948	7.56E-17	3647517
C6_02660C_A	-7.30962	0.000136	3639224
ISA2	-7.32069	1.62E-12	3638513
C5_03960W_A	-7.36144	3.92E-18	3647444
RIM101	-7.38059	7.11E-14	3643280
C1_06980C_A	-7.38745	5.06E-10	3639565
C4_07140W_A	-7.41785	1.06E-13	3642936
C3_07570C_A	-7.51689	2.33E-10	3638522
C5_03800W_A	-7.6003	4.01E-14	3647230
SPO1	-7.60538	0.001289	3647039
C4_03230C_A	-7.60605	0.001751	3637836
CSP1	-7.60605	0.00197	3648242

ELA1	-7.60623	9.66E-08	3644680
C4_04520W_A	-7.74309	6.12E-15	3641110
C7_01070C_A	-7.74722	1.46E-16	3637949
INN1	-7.76414	1.14E-16	3642578
MIF2	-7.80888	4.05E-17	3639220
C1_14190C_A	-7.8182	4.34E-16	3643300
C1_11320C_A	-7.85543	1.37E-12	30515052
SIP5	-8.02445	1.11E-24	3639865
SUR2	-8.05051	1.06E-13	3642973
VTC4	-8.06176	5.16E-19	3646795
C5_04980W_A	-8.14661	2E-13	3636536
HIP1	-8.15488	9.16E-19	3643761
MHP1	-8.16097	1.72E-16	3642460
C7_04160W_A	-8.1776	2.19E-08	3643127
STB3	-8.1852	9.28E-19	3645781
HAP42	-8.21109	3.59E-21	3635729
C5_05240C_A	-8.21165	0.009445	3636471
C2_08950W_A	-8.23723	1.01E-13	3645788
C4_02740W_A	-8.3209	1.58E-13	3637855
C3_01130C_A	-8.39306	9.16E-20	3639901
ECM21	-8.40456	6.54E-22	3634784
CAN2	-8.42946	7.53E-20	3644017
YTH1	-8.43254	1.56E-19	3645572
C2_06530W_A	-8.44626	2.49E-11	3646572
EPL1	-8.45897	3.86E-17	3639732
C4_06200W_A	-8.5887	0.000182	30515282
PLB3	-8.60663	1.2E-23	3647089
ITR1	-8.6216	5.38E-24	3643440
CAN1	-8.63528	1.01E-21	3644035
C4_07170C_A	-8.69497	2.09E-05	30515289
CR_08650C_A	-8.70534	8.61E-18	3642124
C6_01780C_A	-8.71586	9.07E-16	3641636
C3_06950W_A	-8.74559	6.14E-12	3638507
CR_08990C_A	-8.79509	4.32E-17	3641891
CR_07540C_A	-8.79884	0.008171	3648001
C2_01540W_A	-8.80991	3.17E-08	3635804
PTC5	-8.82516	3.33E-18	3642072
ARG1	-8.86383	2.14E-20	3639691
C2_09880C_A	-8.87931	3.77E-14	3643779
CR_04220C_A	-8.88736	8.8E-09	3640943
CTR2	-8.89444	1.2E-20	3648427
CUP1	-8.98909	1.02E-09	30515318
RIM8	-9.02243	2.57E-20	3639342
C1_07980C_A	-9.03345	2.15E-17	3640696
C1_11200W_A	-9.03658	7.59E-06	3644396
BLP1	-9.12426	1.98E-18	30515065
C2_09980W_A	-9.16294	7.54E-14	3643965

FMP45	-9.22163	4.18E-11	3637004
C6_04400W_A	-9.28204	4.2E-05	3645949
WOR3	-9.47539	2.52E-12	3641009
C2_08700C_A	-9.57476	3.56E-19	3643887
FRE9	-9.58111	1.46E-18	3643452
C7_00350C_A	-9.58137	4.4E-21	3638039
C6_03360C_A	-9.58956	3.71E-07	3639203
C4_05900C_A	-9.60072	5.76E-26	3641796
HSP104	-9.67793	1.82E-08	3642097
C1_08610C_A	-9.69024	3.41E-20	3646512
TPS2	-9.72949	1.89E-14	3636892
BRG1	-9.74074	1.06E-15	3647664
STF2	-9.87105	1.31E-13	30515083
C4_01240C_A	-9.92429	3.58E-16	3635705
FAR1	-9.92748	8.17E-11	3637970
CR_06870C_A	-9.93891	0.012574	3647318
CLG1	-9.98552	3.98E-21	3646030
STE11	-10.0476	2.27E-25	3637369
C1_09780C_A	-10.1207	9.12E-26	3634842
ATG1	-10.2471	1.27E-18	3641175
C6_01020W_A	-10.3149	0.023509	3644050
MLS1	-10.3501	5.58E-21	3634875
C5_04030W_A	-10.3646	6.11E-20	3647737
CR_03250C_A	-10.4668	4.44E-26	3645102
C5_02640W_A	-10.5072	8.75E-13	3637785
C6_01870C_A	-10.5189	1.66E-15	3641625
PHR3	-10.5985	3.13E-26	3645003
CRP1	-10.669	2.94E-25	3634826
C6_03180C_A	-10.7036	2.56E-24	3639261
C6_02950C_A	-10.7168	6.25E-10	3639188
FGR46	-10.8202	0.003227	3638989
MTLA2	-10.8215	0.005043	3643766
WHI3	-10.8382	6.16E-18	3636991
OPT2	-10.9698	1.3E-06	3644915
C1_01610C_A	-11.0054	2.06E-11	3640225
C3_00030C_A	-11.0263	9.78E-07	3640571
C6_01400W_A	-11.2771	0.000576	3641604
C1_07160C_A	-11.3184	1.69E-17	30515020
C5_01990W_A	-11.4122	0.000404	3643774
C2_08920W_A	-11.4456	1.82E-26	3645797
RSN1	-11.5785	1.4E-25	3638308
GCA1	-11.6013	1.14E-09	3634903
C6_02790C_A	-11.6344	1.89E-23	3639245
BMT9	-11.7109	3.58E-12	3635654
FTH1	-11.7173	2.12E-21	3634852
C4_03460C_A	-11.7435	7.53E-05	3646813
CR_03260W_A	-11.8704	8.5E-24	30515389

C3_02790W_A	-11.8978	2.2E-08	3638416
C2_02190C_A	-11.9524	6.35E-13	3635753
GAP2	-12.079	2.59E-17	3643662
ECM22	-12.1491	1.5E-24	3648000
C1_01130W_A	-12.4164	3E-19	3640178
CIRT4B	-12.4429	4.33E-19	3642560
C1_11510C_A	-12.5578	9.77E-14	3645336
CR_01510C_A	-12.8114	0.000204	3640104
C1_13590W_A	-12.8998	0.005443	3636209
CR_03670W_A	-12.9011	0.00398	3643220
C3_03250W_A	-12.9816	5.36E-23	3638318
GRX1	-13.0659	0.024246	3637026
AGA1	-13.0659	0.024246	3641536
CR_10310W_A	-13.0659	0.024246	3638937
C4_02230C_A	-13.0668	0.024241	3635693
C7_02000C_A	-13.0676	0.026386	3636964
C4_07020C_A	-13.0681	0.024241	3642965
FUS1	-13.0681	0.024241	3645342
CFL11	-13.069	0.024599	3646622
CTA4	-13.0787	4.95E-28	3641743
C7_01690W_A	-13.2477	1.75E-10	3636980
C3_00150W_A	-13.296	9.04E-10	3640595
C1_06350W_A	-13.3528	1.33E-30	3639562
C3_06940W_A	-13.4721	2.03E-29	3638509
C1_07530W_A	-13.4724	4.43E-05	30515025
LIP1	-13.5957	1.15E-05	3634888
C3_03460C_A	-13.7732	1.37E-08	3638359
ROA1	-13.9559	5.6E-25	3645424
C4_04300C_A	-14.0943	1.05E-10	3646843
CR_08830W_A	-14.2121	2.03E-10	30515450
C4_05350W_A	-14.4384	5.65E-31	3646948
C1_10360C_A	-14.4509	2.27E-14	3634836
BUB3	-14.7045	6.26E-19	3646362
C6_04200C_A	-14.7304	9.45E-09	30515344
C1_07650W_A	-14.7938	7.58E-11	3646657
SOD5	-14.8446	1.13E-06	3638886
ZCF2	-14.9755	1.98E-28	3642484
C4_04020C_A	-15.7131	0.00104	3641215
PIR1	-15.7818	5.82E-28	3645801
SSY1	-16.1001	2.58E-26	3637382
C7_01910C_A	-16.1264	8.54E-20	3637029
TPS3	-16.7197	3.06E-23	3641205
CR_09920W_A	-16.8996	2.77E-20	3639028
C3_07910W_A	-17.2225	0.005813	3647449
GAC1	-17.2482	3.77E-28	3638025
RCH1	-17.3783	1.27E-22	3645002
C7_02610C_A	-17.3869	3.85E-26	3648285

ARF1	-17.4509	1.51E-28	3642130
C1_01620C_A	-17.453	5.72E-29	3640193
FGR47	-17.5629	6.52E-35	3641651
C2_05330C_A	-17.6642	3.55E-23	3643426
UGA6	-17.73	7.07E-07	3642975
C4_07000W_A	-18.6153	0.008121	3642967
HSP78	-18.708	4.04E-33	3637414
C7_00630C_A	-18.9985	1.23E-07	3638028
GCA2	-19.0589	6.56E-19	3647194
C6_04420W_A	-19.35	2.45E-12	3645951
YHB1	-19.488	4.51E-45	3647353
BMT1	-19.5401	1.76E-24	3638535
IFA14	-19.643	9.09E-10	3639012
PUT1	-20.5372	2.37E-25	3637779
CTN3	-20.8799	1.77E-17	3647396
CR_06140W_A	-21.2135	1.28E-32	3644862
C7_02600C_A	-21.7199	6.78E-30	3648286
C2_07070W_A	-22.1462	3.37E-20	3643501
C1_00270W_A	-22.5076	1.1E-35	3639300
C7_01680C_A	-22.5846	3.81E-44	3636981
CUP9	-22.8698	7.92E-35	3636950
C2_04670W_A	-23.103	1.15E-14	3647789
C6_03280W_A	-24.1676	0.003251	3639185
FGR42	-24.1676	0.003286	3647438
CDR11	-24.4726	6.39E-42	3647389
C1_06430C_A	-24.7483	0.000202	3639553
CTN1	-25.3593	1.11E-16	3645414
C1_09210C_A	-25.4483	8.39E-34	3634897
C1_05440C_A	-25.7147	1.27E-29	3642489
SRR1	-26.1289	2.93E-18	3647589
C7_01430C_A	-26.3699	1.51E-50	3636961
ARG11	-26.6101	1.35E-07	3635193
ZCF1	-27.0755	1.57E-29	3638329
C5_03080C_A	-29.0749	2.66E-29	3637701
TRY4	-29.3333	1.59E-16	3635240
C2_08260W_A	-29.6603	7.38E-11	3642345
C6_03810W_A	-29.6611	5.21E-33	3640286
PRM9	-29.7171	0.002689	30515173
CDR4	-30.1516	6.94E-13	3640711
C1_09770W_A	-30.2487	9.1E-27	3634825
C2_08580W_A	-31.0222	4.79E-07	3643916
HAK1	-31.0249	1.51E-06	3639546
C6_00810C_A	-31.0458	2E-29	3647068
OPT4	-32.1718	3.4E-27	3647697
CR_09930W_A	-32.2966	2.85E-19	3639029
SSU1	-32.5166	1.03E-35	3641882
ALS7	-33.2278	9.23E-46	3642234

HGT17	-33.6375	2.26E-05	3635687
PHO87	-33.6812	9.73E-15	3639861
PGA48	-36.9615	2.83E-32	3647905
ICL1	-37.2415	8.04E-48	3646146
GIT3	-37.8186	2.18E-55	3642634
C2_02390W_A	-38.2371	2.04E-32	3635829
CR_07680C_A	-39.099	1.36E-07	3648193
C7_00880C_A	-39.8809	2.36E-27	3638052
C1_05160C_A	-40.2152	1.73E-31	3642468
C3_04730C_A	-40.813	0.000691	3635250
C7_00770W_A	-40.8738	1.31E-28	3637959
CDA2	-43.1521	1.92E-08	3639716
C2_00760C_A	-45.0281	8.3E-36	3638910
C1_02690C_A	-46.6007	0.000518	3636823
RHD3	-48.6244	5.83E-31	3641212
STP4	-49.7659	1.1E-47	3637405
KSP1	-52.0796	1.81E-44	3644467
IFE2	-52.2835	1.73E-34	3648027
ALD6	-53.1957	2.22E-32	3647407
C7_03560W_A	-54.9282	3.81E-42	3646236
CR_04940W_A	-57.2785	2.22E-11	3648209
C4_07260W_A	-60.9861	1.28E-13	3642942
CR_08880C_A	-62.2809	5.52E-32	3641878
C2_02130C_A	-65.5388	1.63E-17	3635747
JEN2	-65.6098	6.51E-31	3641214
CFL4	-73.4567	3.04E-10	3642639
HSP31	-85.5597	3.04E-11	3644589
PRX1	-102.178	5.54E-39	3641357
C1_14630C_A	-102.652	1.93E-13	3643291
C4_03340C_A	-104.734	2.89E-09	3648090
GPI1	-106.355	1.41E-23	3643680
CR_01530C_A	-107.903	8.94E-16	3640072
HSP21	-138.662	2.05E-87	3637364
C5_04380C_A	-146.258	7.48E-07	3636485
MNN1	-156.065	9.73E-22	3637784
PGA37	-185.083	1.84E-07	3636484
C1_06620C_A	-190.557	0.049158	3639545
C3_04870W_A	-190.557	0.049158	3635226
C5_00810C_A	-190.557	0.0492	3644127
CRZ2	-199.772	1.16E-71	3645725
C2_09330C_A	-234.324	0.04117	3644106
RME1	-261.804	2.75E-51	3644453
C1_00190C_A	-267.164	3.76E-30	3639337
C3_07930C_A	-292.374	0.038072	3647452
C3_06660C_A	-321.845	0.030687	3642253
CR_00840C_A	-321.865	0.031346	3640411
CR_03320C_A	-339.357	0.033251	3645096

C3_00120W_A	-365.617	0.027524	3640566
MRV4	-496.857	0.019622	3636547
HWP2	-528.72	0.019289	3646803
C2_02640C_A	-628.194	0.015253	30515095
C4_06910W_A	-745.678	1.85E-11	3642933
C1_09820C_A	-759.46	0.012435	3634846
PGA57	-803.044	0.01179	3635608
C3_00230C_A	-978.244	0.009367	3640557
HSP30	-5260.32	6.78E-33	3645419
TAR1	-16143.3	1.07E-74	30515449

B45 vs Control

Name	Fold change	P-value	GeneID
ASG7	#N/A	#N/A	3639173
ATO7	#N/A	#N/A	3635814
BUD20	#N/A	#N/A	3645800
C1_00020C_A	#N/A	#N/A	3639374
C1_01720C_A	#N/A	#N/A	30514964
C1_01970W_A	#N/A	#N/A	30514967
C1_03580W_A	#N/A	#N/A	30514980
C1_03880C_A	#N/A	#N/A	3646409
C1_04440W_A	#N/A	#N/A	3646138
C1_05550C_A	#N/A	#N/A	30514994
C1_05730W_A	#N/A	#N/A	30515001
C1_06300W_A	#N/A	#N/A	30515004
C1_06310W_A	#N/A	#N/A	30515005
C1_06490C_A	#N/A	#N/A	30515009
C1_06830W_A	#N/A	#N/A	30515011
C1_07320W_A	#N/A	#N/A	30515023
C1_07450W_A	#N/A	#N/A	30515024
C1_07940W_A	#N/A	#N/A	30515027
C1_08960W_A	#N/A	#N/A	30515032
C1_08970W_A	#N/A	#N/A	30515033
C1_09500W_A	#N/A	#N/A	3634764
C1_09560C_A	#N/A	#N/A	30515034
C1_10130W_A	#N/A	#N/A	30515040
C1_10590W_A	#N/A	#N/A	30515042
C1_11180C_A	#N/A	#N/A	30515050
C1_11710C_A	#N/A	#N/A	3645320
C1_11780W_A	#N/A	#N/A	30515057
C1_12040W_A	#N/A	#N/A	3642029
C1_12980W_A	#N/A	#N/A	30515068
C1_12990W_A	#N/A	#N/A	30515069
C1_13000W_A	#N/A	#N/A	30515070
C1_13040C_A	#N/A	#N/A	30515071
C1_13500C_A	#N/A	#N/A	30515075
C2_00050C_A	#N/A	#N/A	3647922

C2_00900W_A	#N/A	#N/A	3638895
C2_01460C_A	#N/A	#N/A	3635844
C2_02080W_A	#N/A	#N/A	30515093
C2_02140C_A	#N/A	#N/A	3635748
C2_03580W_A	#N/A	#N/A	30515100
C2_03630W_A	#N/A	#N/A	3637438
C2_04070C_A	#N/A	#N/A	30515110
C2_04610C_A	#N/A	#N/A	30515113
C2_05310C_A	#N/A	#N/A	30515119
C2_05420C_A	#N/A	#N/A	30515121
C2_05600W_A	#N/A	#N/A	30515124
C2_05880W_A	#N/A	#N/A	30515129
C2_06400C_A	#N/A	#N/A	30515135
C2_06410W_A	#N/A	#N/A	30515136
C2_06560W_A	#N/A	#N/A	30515139
C2_07710W_A	#N/A	#N/A	3642305
C2_08150W_A	#N/A	#N/A	3642328
C2_08520C_A	#N/A	#N/A	30515155
C2_09110C_A	#N/A	#N/A	3644114
C2_09340W_A	#N/A	#N/A	3644107
C2_09540W_A	#N/A	#N/A	30515159
C2_09550C_A	#N/A	#N/A	30515160
C2_09800C_A	#N/A	#N/A	3643803
C2_10800W_A	#N/A	#N/A	30515169
C3_00140W_A	#N/A	#N/A	3640596
C3_01050C_A	#N/A	#N/A	3639831
C3_01740C_A	#N/A	#N/A	30515178
C3_02880W_A	#N/A	#N/A	3638335
C3_03620C_A	#N/A	#N/A	3645654
C3_04150W_A	#N/A	#N/A	30515202
C3_05660C_A	#N/A	#N/A	3643656
C3_05820W_A	#N/A	#N/A	30515212
C4_00990W_A	#N/A	#N/A	3635609
C4_01570C_A	#N/A	#N/A	30515236
C4_01580C_A	#N/A	#N/A	30515237
C4_01590C_A	#N/A	#N/A	30515238
C4_01600C_A	#N/A	#N/A	30515239
C4_01610C_A	#N/A	#N/A	30515240
C4_01620C_A	#N/A	#N/A	30515241
C4_01630C_A	#N/A	#N/A	30515242
C4_01640C_A	#N/A	#N/A	30515243
C4_02650C_A	#N/A	#N/A	30515249
C4_03480C_A	#N/A	#N/A	3646815
C4_03760W_A	#N/A	#N/A	3645355
C4_04040W_A	#N/A	#N/A	3641213
C4_04400C_A	#N/A	#N/A	30515268
C4_04490C_A	#N/A	#N/A	30515269

C4_06180C_A	#N/A	#N/A	30515281
C4_06520W_A	#N/A	#N/A	30515284
C4_06630C_A	#N/A	#N/A	3642812
C4_06640C_A	#N/A	#N/A	3642811
C5_00530W_A	#N/A	#N/A	3641537
C5_01030W_A	#N/A	#N/A	30515298
C5_01150C_A	#N/A	#N/A	30515299
C5_01160C_A	#N/A	#N/A	30515300
C5_02130W_A	#N/A	#N/A	3637733
C5_02850W_A	#N/A	#N/A	30515305
C5_03450W_A	#N/A	#N/A	30515311
C5_03890C_A	#N/A	#N/A	30515312
C5_04450C_A	#N/A	#N/A	30515315
C5_05510C_A	#N/A	#N/A	3636476
C6_00220C_A	#N/A	#N/A	30515324
C6_01540C_A	#N/A	#N/A	30515333
C6_02390W_A	#N/A	#N/A	30515339
C6_02400W_A	#N/A	#N/A	30515340
C6_03570W_A	#N/A	#N/A	3640248
C6_04150W_A	#N/A	#N/A	30515343
C7_00540W_A	#N/A	#N/A	30515348
C7_01000C_A	#N/A	#N/A	3638022
C7_01060W_A	#N/A	#N/A	3637950
C7_01130C_A	#N/A	#N/A	3645912
C7_01260W_A	#N/A	#N/A	30515352
C7_02190W_A	#N/A	#N/A	30515356
C7_02420C_A	#N/A	#N/A	3636975
C7_04070C_A	#N/A	#N/A	3643153
CR_00450C_A	#N/A	#N/A	30515367
CR_00500C_A	#N/A	#N/A	30515368
CR_00720W_A	#N/A	#N/A	3640441
CR_02320W_A	#N/A	#N/A	30515381
CR_02470W_A	#N/A	#N/A	30515382
CR_02660C_A	#N/A	#N/A	30515383
CR_03190C_A	#N/A	#N/A	30515388
CR_04620C_A	#N/A	#N/A	3640952
CR_04980C_A	#N/A	#N/A	3646395
CR_05350W_A	#N/A	#N/A	30515405
CR_05370W_A	#N/A	#N/A	30515406
CR_05470C_A	#N/A	#N/A	30515409
CR_06190C_A	#N/A	#N/A	30515418
CR_06200C_A	#N/A	#N/A	30515419
CR_06210C_A	#N/A	#N/A	30515420
CR_06220C_A	#N/A	#N/A	30515421
CR_06580W_A	#N/A	#N/A	3646627
CR_06600C_A	#N/A	#N/A	30515422
CR_06630W_A	#N/A	#N/A	30515423

CR_07300W_A	#N/A	#N/A	3646059
CR_08140W_A	#N/A	#N/A	30515435
CR_10320W_A	#N/A	#N/A	3638938
CR_10460W_A	#N/A	#N/A	30515457
CSA2	#N/A	#N/A	3642930
FGR18	#N/A	#N/A	3644112
FGR37	#N/A	#N/A	3641609
GTT12	#N/A	#N/A	3638304
ITS1	#N/A	#N/A	30515447
ITS2	#N/A	#N/A	30515448
MFALPHA	#N/A	#N/A	3646420
PGA12	#N/A	#N/A	3638920
PGA44	#N/A	#N/A	3636647
PGA50	#N/A	#N/A	3647524
RDN18	#N/A	#N/A	3648135
RDN25	#N/A	#N/A	3648137
RDN5	#N/A	#N/A	3648138
RDN58	#N/A	#N/A	3648136
RFC52	#N/A	#N/A	30514953
RHD2	#N/A	#N/A	3637891
RPR1	#N/A	#N/A	30515150
SCR1	#N/A	#N/A	30515392
SNR52	#N/A	#N/A	30515222
SNR6	#N/A	#N/A	30515211
tA(AGC)1	#N/A	#N/A	30515148
tA(AGC)2	#N/A	#N/A	30515199
tA(AGC)3	#N/A	#N/A	30514965
tA(AGC)4	#N/A	#N/A	30514960
tA(AGC)5	#N/A	#N/A	30515321
tA(AGC)7	#N/A	#N/A	30515078
tA(UGC)1	#N/A	#N/A	30515336
tA(UGC)2	#N/A	#N/A	30515342
tC(GCA)1	#N/A	#N/A	30515411
tC(GCA)2	#N/A	#N/A	30515410
tD(GUC)2	#N/A	#N/A	30515261
tD(GUC)3	#N/A	#N/A	30515260
tD(GUC)4	#N/A	#N/A	30515259
tD(GUC)5	#N/A	#N/A	30515114
tD(GUC)6	#N/A	#N/A	30515233
tD(GUC)7	#N/A	#N/A	30515452
tE(CUC)1	#N/A	#N/A	30515101
tE(UUC)1	#N/A	#N/A	30515196
tE(UUC)2	#N/A	#N/A	30515184
tE(UUC)3	#N/A	#N/A	30515295
tE(UUC)4	#N/A	#N/A	30515294
tE(UUC)5	#N/A	#N/A	30515309
tE(UUC)6	#N/A	#N/A	30515198

tE(UUC)7	#N/A	#N/A	30515445
tF(GAA)1	#N/A	#N/A	30515191
tF(GAA)2	#N/A	#N/A	30515103
tF(GAA)3	#N/A	#N/A	30515107
tF(GAA)4	#N/A	#N/A	30515166
tF(GAA)5	#N/A	#N/A	30515224
tG(CCC)1	#N/A	#N/A	30515228
tG(GCC)1	#N/A	#N/A	30515432
tG(GCC)2	#N/A	#N/A	30515258
tG(GCC)3	#N/A	#N/A	30515115
tG(GCC)4	#N/A	#N/A	30515231
tG(GCC)5	#N/A	#N/A	30515230
tG(GCC)6	#N/A	#N/A	30515446
tG(UCC)1	#N/A	#N/A	30515172
tG(UCC)2	#N/A	#N/A	30515229
tH(GUG)1	#N/A	#N/A	30515401
tH(GUG)3	#N/A	#N/A	30515217
tI(AAU)1	#N/A	#N/A	30515138
tI(AAU)2	#N/A	#N/A	30515147
tI(AAU)3	#N/A	#N/A	30515122
tI(AAU)4	#N/A	#N/A	30515272
tI(AAU)5	#N/A	#N/A	30515440
tI(UAU)1	#N/A	#N/A	30515045
tK(CUU)1	#N/A	#N/A	30515193
tK(CUU)2	#N/A	#N/A	30515206
tK(UUU)1	#N/A	#N/A	30515181
tK(UUU)2	#N/A	#N/A	30515428
tK(UUU)3	#N/A	#N/A	30515436
tK(UUU)4	#N/A	#N/A	30515437
tK(UUU)5	#N/A	#N/A	30515438
tL(AAG)1	#N/A	#N/A	30515379
tL(AAG)2	#N/A	#N/A	30515022
tL(CAA)1	#N/A	#N/A	30515091
tL(CAA)2	#N/A	#N/A	30515087
tL(CAA)3	#N/A	#N/A	30515346
tL(CAA)4	#N/A	#N/A	30515444
tL(CAA)5	#N/A	#N/A	30515322
tL(CAA)6	#N/A	#N/A	30515362
tL(UAA)1	#N/A	#N/A	30515089
tL(UAA)2	#N/A	#N/A	30515375
tL(UAA)3	#N/A	#N/A	30514979
tL(UAA)4	#N/A	#N/A	30515017
tL(UAA)5	#N/A	#N/A	30515360
tM(CAU)1	#N/A	#N/A	30515188
tM(CAU)2	#N/A	#N/A	30515145
tM(CAU)3	#N/A	#N/A	30515132
tM(CAU)4	#N/A	#N/A	30515451

tN(GUU)1	#N/A	#N/A	30514997
tN(GUU)2	#N/A	#N/A	30514996
tN(GUU)3	#N/A	#N/A	30514978
tN(GUU)4	#N/A	#N/A	30515455
tP(AGG)1	#N/A	#N/A	30515016
tP(UGG)1	#N/A	#N/A	30514992
tP(UGG)2	#N/A	#N/A	30515157
tP(UGG)3	#N/A	#N/A	30514956
tP(UGG)4	#N/A	#N/A	30515413
tP(UGG)5	#N/A	#N/A	30515412
tQ(CUG)1	#N/A	#N/A	30515296
tQ(UUG)1	#N/A	#N/A	30515257
tQ(UUG)3	#N/A	#N/A	30515118
tQ(UUG)4	#N/A	#N/A	30515271
tQ(UUG)5	#N/A	#N/A	30515270
tR(ACG)1	#N/A	#N/A	30515408
tR(ACG)2	#N/A	#N/A	30515407
tR(CCG)1	#N/A	#N/A	30514970
tR(CCU)1	#N/A	#N/A	30515177
tR(UCU)1	#N/A	#N/A	30515192
tR(UCU)2	#N/A	#N/A	30515102
tR(UCU)3	#N/A	#N/A	30515106
tR(UCU)4	#N/A	#N/A	30514971
tR(UCU)5	#N/A	#N/A	30515223
tS(AGA)1	#N/A	#N/A	30515416
tS(AGA)2	#N/A	#N/A	30515425
tS(AGA)3	#N/A	#N/A	30515415
tS(AGA)4	#N/A	#N/A	30515126
tS(CAG)1	#N/A	#N/A	30515292
tS(CGA)1	#N/A	#N/A	30515036
tS(GCU)1	#N/A	#N/A	30515398
tS(GCU)2	#N/A	#N/A	30515397
tS(UGA)1	#N/A	#N/A	30514963
tS(UGA)2	#N/A	#N/A	30515279
tS(UGA)3	#N/A	#N/A	30515235
tT(AGU)1	#N/A	#N/A	30514998
tT(AGU)3	#N/A	#N/A	30515369
tT(AGU)4	#N/A	#N/A	30515116
tT(AGU)6	#N/A	#N/A	30514952
tT(CGU)1	#N/A	#N/A	30514983
tT(UGU)1	#N/A	#N/A	30515252
tT(UGU)2	#N/A	#N/A	30515278
tV(AAC)1	#N/A	#N/A	30515094
tV(AAC)2	#N/A	#N/A	30515180
tV(AAC)3	#N/A	#N/A	30515380
tV(AAC)4	#N/A	#N/A	30515414
tV(AAC)5	#N/A	#N/A	30515209

tV(AAC)6	#N/A	#N/A	30515210
tV(CAC)1	#N/A	#N/A	30515081
tV(UAC)1	#N/A	#N/A	30515165
tW(CCA)1	#N/A	#N/A	30515064
tW(CCA)2	#N/A	#N/A	30515220
tY(GUA)1	#N/A	#N/A	30515058
tY(GUA)2	#N/A	#N/A	30515183
tY(GUA)3	#N/A	#N/A	30515221
tY(GUA)4	#N/A	#N/A	30515059
C1_04180W_A	2166.965	9.75E-06	3644716
HHT21	862.7474	1.88E-13	3644707
PXP2	252.8188	0.001772	3636558
CDC11	247.7979	0.001399	3647888
C1_13100W_A	236.3041	0.002501	30515073
HHT2	196.5096	2.97E-20	3647326
COG4	187.9012	0.002382	3645571
C4_05390W_A	185.0709	0.002463	3646951
HGT12	174.0884	0.002787	3638048
ALI1	164.6847	0.003133	3636635
HTA2	159.1083	2.08E-18	3644715
C2_09590C_A	146.18	0.004144	3643800
C5_03000C_A	143.1225	0.004052	3637818
VMA10	139.9069	0.004193	3645215
C1_03150C_A	136.7983	0.004359	30514976
MLC1	134.5805	0.004499	30515387
ECM331	128.232	0.004985	3637731
SAM51	116.7657	0.005879	3641582
CRH11	114.1947	1.14E-07	3637905
HOM6	113.0353	1.28E-07	3636815
HRT2	95.64974	2.78E-07	3635709
C6_00660C_A	93.70142	0.00871	3647133
C6_02140W_A	92.87445	0.008826	3645886
CDC10	92.47391	4.25E-07	3640947
CR_05210W_A	92.32608	0.00989	3648081
HHO1	92.23814	1.36E-13	30515358
TNA1	88.14619	0.009783	3637827
KAR3	87.11876	0.009742	3644078
FGR6-10	82.64575	0.010573	3641770
C3_07540C_A	81.75328	0.010888	3638526
C3_02620C_A	81.31457	0.01091	3638326
IFE1	78.20751	0.011505	3644778
C7_00240W_A	72.74629	0.013452	3637980
TUB4	70.21221	0.013824	3641774
C1_06700W_A	70.03219	2.53E-06	3639511
CR_05310W_A	69.71725	0.014095	3648125
CR_06380C_A	66.66372	0.014909	3648139
HHF1	64.58899	1.12E-20	3644705

DPM2	60.89383	0.017394	30515325
SOU1	59.99438	2.43E-09	3642799
TUB2	59.72865	5.71E-12	3639329
PGA6	57.08214	8.47E-06	3634774
C6_02180W_A	56.89437	0.019575	3645861
C3_01100W_A	56.63476	0.021064	3639834
C6_00710W_A	56.45123	0.019908	3647073
C3_06730W_A	55.86659	0.021103	3638504
LEU2	55.71417	2.65E-10	3638034
LSC1	54.87289	1.6E-18	3640199
GUP1	54.67702	0.021052	3636171
CCN1	54.23321	0.021174	3643735
C6_00400C_A	53.34345	0.021629	3646087
ILV5	51.4566	5.19E-26	3644012
C4_02460W_A	50.27491	0.023589	3637844
C1_02100W_A	49.7939	0.023822	3644578
DDR48	49.72886	1.42E-17	3644061
CWH8	49.49135	0.025278	3644598
C2_10010C_A	49.35008	0.024097	3643962
CR_00280C_A	47.57422	0.02565	3639719
C3_07840C_A	47.5734	0.025544	3647465
C2_06760C_A	46.351	0.027539	3648022
YOX1	45.25529	0.028639	3637952
C2_02050C_A	43.76637	3.93E-07	3635851
AGM1	43.07282	3.04E-05	3636138
AMO2	42.71079	1.87E-10	3648021
CR_10130W_A	42.68826	0.030188	3638970
C3_02140C_A	42.48132	2E-10	3636656
C5_02780W_A	42.33482	0.031466	3637803
ERG28	42.26361	0.03075	3638833
C7_01750W_A	41.80063	0.03077	3637009
C1_11950W_A	41.75979	0.031193	3642045
C1_13770C_A	40.91322	0.031648	3636139
DPM1	40.87069	3.32E-05	3640700
ARC18	40.46986	0.032142	3644056
C3_06750W_A	40.46456	0.032295	30515219
CR_04490C_A	40.44631	0.032511	3641013
C7_01100C_A	39.5801	0.033318	3637948
C1_10560C_A	39.17789	0.034108	3647196
FAD1	38.69472	0.034429	3634930
TUB1	38.63581	8.44E-17	3641930
CDC28	38.12393	4.78E-05	3644838
HHF22	37.99188	1.25E-18	3647327
TEM1	36.93082	0.037357	3636923
ACP12	35.888	6.1E-05	3637361
C1_11990W_A	35.69336	0.041385	3642042
C2_02330W_A	35.18801	0.041119	3635811

C7_01830W_A	34.69877	0.041551	3637053
CWH43	34.69127	3.97E-09	3640389
HTA1	34.42255	1.68E-17	3645658
C2_06280C_A	33.82988	0.043151	3646923
PHA2	33.81006	0.043065	3638346
C2_05720C_A	32.17936	1.08E-08	3645574
C2_09960W_A	31.5906	0.047348	3643821
PMI1	31.42779	1.52E-06	3643795
SHE3	31.36665	0.000118	3639277
LYS9	31.16137	5.44E-12	3642279
C4_02600C_A	31.15929	0.048596	3637912
C1_07920W_A	31.14546	0.048473	30515026
PGA38	31.12234	2.02E-07	3637846
C2_05510C_A	30.70306	0.048529	3643420
C3_07130W_A	30.70288	0.048468	3638440
OST1	30.68756	0.000126	3643657
THR1	30.57367	0.000128	3641572
MET15	29.35811	6.81E-22	3645031
ASH2	28.99961	0.000175	3636445
LEU4	28.9904	8.07E-08	3639339
CHS1	28.70319	6.15E-11	3641364
C1_09060C_A	28.30326	0.000223	3634772
ALS4	28.20492	3.86E-26	3647972
C1_03940W_A	28.18335	1.59E-06	3646415
SOL3	27.81889	2.08E-08	3646625
C2_09100C_A	27.66253	6.02E-12	3644113
ASK1	27.62902	0.000244	3635656
SSR1	27.14681	5.72E-14	3638055
PHR1	26.99618	4.74E-26	3641108
FGR41	26.99195	0.000541	3634839
ERG11	26.66072	1.6E-11	3641571
CR_03000C_A	26.6311	0.000275	3642597
CLB4	25.94598	2.6E-06	3643102
C6_02190C_A	25.75747	2.79E-06	3645862
C1_03600W_A	25.70437	0.000283	3636880
CYT1	25.67835	1.2E-15	3643441
C1_04430C_A	25.61279	0.000317	30514986
ATP18	25.29403	0.000413	30515084
C1_06050C_A	25.13861	0.000349	30515003
BIO2	24.81582	0.000402	3640106
IDP1	24.36888	3.88E-12	3647474
C1_14320C_A	24.22961	0.000406	3643316
CAT1	24.0958	1.15E-18	3639495
SNZ1	23.84286	2.49E-07	3636803
PHO15	23.57425	3.42E-11	3644459
GDH3	23.5637	3.39E-16	3648086
QCR2	23.24859	1.05E-16	3646352

ATP1	23.05777	6.08E-25	3646134
BGL2	22.82861	1.41E-21	3635691
CDC5	22.67828	4.73E-13	3639281
C2_00180C_A	22.57644	1.57E-08	3638905
C2_07140W_A	22.35038	5.46E-12	3643508
AMO1	22.31737	1.88E-06	3642994
ATP19	21.63119	0.000629	30515061
C2_07540W_A	21.17796	0.000696	3645222
CR_04760C_A	20.70813	1.3E-05	3647303
C5_02920W_A	20.3854	0.000769	3637751
C1_10170W_A	20.38223	0.099223	3634783
PDI1	20.26597	5.56E-17	3641367
CR_06170W_A	20.25821	0.00081	3644865
HOF1	20.20212	2.23E-06	3645014
YNK1	19.9981	1.2E-11	3637746
SEC21	19.99402	1.58E-12	3644786
PLC2	19.50833	5.18E-07	3642985
RGD1	19.40861	4.76E-07	3639868
LYS4	19.3754	5.6E-10	3641180
DUR1,2	19.32104	2.39E-08	3644771
RFA2	18.96228	0.001145	3643506
C5_01230C_A	18.87863	3.47E-10	3642618
RAD54	18.83325	0.001113	3636192
ERV46	18.82375	2.67E-05	3644129
GND1	18.77185	8.06E-20	3636131
HAT1	18.63479	2.96E-05	3644770
TAL1	18.50021	1.12E-20	3643225
CR_09520C_A	18.43657	2.99E-05	3647103
LAT1	18.32011	2.67E-19	3636986
ACO2	18.27477	9.89E-07	3646708
LYS22	18.24696	2.34E-07	3645466
HTA3	18.05301	2.51E-07	3638322
C1_02760W_A	17.9854	2.28E-07	3636870
TSA1	17.82827	1.01E-18	3642269
LSC2	17.70064	1.2E-18	3646618
OPI3	17.67022	0.001386	3642277
CR_05500C_A	17.55805	0.001374	3647598
CRC1	17.52641	2.9E-10	3640112
C4_01510W_A	17.50183	6.35E-09	3635616
AAP1	17.47746	7.16E-06	3647397
DOG1	17.44454	0.001372	3641597
SPE3	17.20832	1.34E-11	3643528
QCR8	17.11912	4.35E-09	30515112
ERG6	16.97448	5.28E-07	3636655
C1_09980C_A	16.93481	4.83E-05	3634863
LEU1	16.8882	3.04E-20	3639685
APE3	16.87908	3.23E-06	3643875

C1_07260C_A	16.76558	0.001534	3644456
TSA1B	16.66488	4.46E-18	30515214
C2_03970W_A	16.53856	0.001657	3637419
LYS12	16.49661	1.56E-16	3640134
CYB5	16.48032	6.48E-05	3637965
CR_09330C_A	16.35669	5.62E-12	3641899
POL30	16.32521	0.001852	3635583
PTP3	16.22599	2.77E-19	3638940
CR_03520C_A	16.05925	1.84E-09	3643203
NHP6A	15.92446	6.08E-16	30515244
ERG26	15.91254	4.16E-06	3642803
COX5	15.89234	2.4E-12	3634768
CR_03330W_A	15.8603	0.00197	3645095
COF1	15.48671	8.35E-13	30515293
KIP1	15.43935	0.000102	3645261
C4_07080C_A	15.37895	0.000105	3642905
C2_06770W_A	15.28792	0.002629	3643515
CHO1	15.25488	3.37E-07	3644557
C5_02220C_A	14.73603	0.003297	3637760
OSH3	14.62775	6.66E-09	3645368
C2_03020C_A	14.5568	4.6E-16	3643019
TOS1	14.5508	3.8E-16	3636566
ATP3	14.49465	5.12E-16	3640387
MDH1-1	14.38717	1.08E-17	3635636
MAL31	14.30603	0.004245	3636420
SWI6	14.27584	0.003439	3634932
FMA1	14.2456	0.005938	3647986
CEX1	14.23096	7.84E-06	3644913
CR_04770C_A	14.19421	0.003748	3647302
MSB1	14.18702	7.19E-07	3648218
EAF6	14.16685	0.003567	3646494
ZWF1	14.1636	1.32E-15	3634913
DOA1	14.09431	6.09E-12	3634871
CHT3	14.09039	6.57E-06	3638967
CR_05360C_A	14.04989	0.003659	3648033
ACT1	14.03461	1.09E-19	3636195
C6_02560W_A	14.01154	1.29E-08	3639205
RFC5	13.94967	0.003882	3638890
GRP1	13.89863	0.000206	3634899
PMT2	13.84039	4.26E-12	3638538
C1_00700W_A	13.83053	0.004184	3639330
EMP24	13.82541	0.000187	3647247
SEC23	13.77015	2.19E-14	3641818
C1_01190C_A	13.627	0.000211	3640207
SAC1	13.60943	9.3E-07	3634864
C2_06890C_A	13.59094	0.000254	3643522
C1_03240W_A	13.50214	1.3E-05	3636895

C6_04100W_A	13.28715	3.18E-09	3647836
C3_05290C_A	13.20606	0.000532	3635203
C7_00230W_A	13.15707	2.23E-06	3637982
CR_03950W_A	13.14497	0.00434	3640960
MCR1	13.12818	4.14E-16	3647561
C2_02410W_A	13.09904	8.1E-09	3635755
HIS1	13.02829	5.94E-08	3636501
C5_02380W_A	12.90739	9.77E-19	3637794
PEX11	12.89688	7.61E-06	3646288
MPS1	12.7809	0.005754	3641888
CHT2	12.7762	7.99E-11	3636523
HSL1	12.73627	4.89E-09	3637810
C2_09040W_A	12.7085	3.14E-07	3645792
CMP1	12.65437	2.79E-07	3639328
C1_04200C_A	12.59167	2.06E-05	3644718
C1_10310W_A	12.5335	0.005283	3634906
CBR1	12.50737	2.98E-07	3646961
ERV25	12.42231	2.21E-06	3642243
BMT4	12.39892	2.7E-07	3639182
CR_09670C_A	12.39213	4.14E-09	3647091
C1_12650C_A	12.35159	0.005518	3646836
HOM3	12.34756	9.27E-07	3641771
CLB2	12.34543	1.48E-14	3635840
VMA2	12.27472	1.65E-15	3646710
C6_03320W_A	12.24058	3.7E-05	3639198
CR_01490C_A	12.20106	2.69E-05	30515376
CDC19	12.19308	1.05E-16	3643438
RBT4	12.1705	2.8E-07	3639534
SEC22	12.15299	0.006984	30515391
CR_03470W_A	12.12504	0.005741	3643198
PMM1	12.0849	7.72E-06	3636852
C3_01020W_A	12.07146	0.000379	3639828
TPO3	11.99311	7.81E-12	3634790
FAA21	11.98479	1.29E-09	3638418
RET2	11.93859	8E-09	3637714
C1_05180C_A	11.88014	0.006248	30514990
MIS11	11.87681	3.06E-16	3645732
ACS2	11.79416	5.84E-15	3644710
CDC21	11.78596	0.006134	3643463
CYK3	11.74802	7.87E-05	3639539
C4_00390W_A	11.71854	0.000453	3645016
SMC2	11.70461	4.96E-06	3643918
SGA1	11.68308	5.64E-06	3636652
CAR1	11.6508	1.07E-11	3636503
HGT10	11.62219	2.68E-09	3640309
C4_00710W_A	11.57702	0.00048	3645750
RPS17B	11.5714	1.02E-14	30515044

C1_01080W_A	11.5606	0.006833	3640173
KTR4	11.55985	0.006832	3646425
C2_00590W_A	11.55299	0.000427	3638851
C7_00670C_A	11.51837	6.17E-07	3638024
C1_02270C_A	11.47638	3.08E-08	3644566
PST2	11.46243	0.000105	3643909
ASE1	11.39692	0.000139	3641729
ECM33	11.38635	3.59E-16	30514977
HNT2	11.33394	0.007334	3642271
ILV6	11.30177	3.12E-11	3635598
ERG13	11.17751	3.03E-08	3641881
ACO1	11.15697	3.22E-16	3642090
ALD5	11.14921	1.02E-17	3643026
CR_09230C_A	11.14478	7.45E-05	3641918
HTB1	11.08818	5.1E-14	3645659
C4_01840C_A	11.08281	3.01E-07	3635592
C2_05860C_A	11.03457	0.009343	3647473
ASR1	10.95087	3.23E-11	3644402
SEP7	10.94705	7.54E-06	3644596
SEC13	10.943	1.14E-08	3638425
ARP3	10.93729	1.36E-11	3643479
MET3	10.90997	1.55E-14	3636132
SEC20	10.90382	0.00062	3639206
ATP2	10.87424	6.99E-17	3645039
C7_01710W_A	10.87193	1.8E-08	3637012
HSP12	10.8461	7.26E-14	3643745
CYP1	10.84566	2.52E-14	3636978
CR_07120C_A	10.83582	0.000295	3648225
C2_07550W_A	10.80244	4.84E-09	3645221
HYR1	10.76931	0.009029	3636226
C2_07010W_A	10.74807	3.49E-07	3643535
GIN4	10.73045	2.51E-06	3644543
HET1	10.72867	6.57E-10	3647897
ACB1	10.70781	2.43E-07	30515350
C5_02110W_A	10.67862	3.74E-14	3637732
PST3	10.67478	2.04E-12	3648031
HIS5	10.64756	0.000716	3645756
IPP1	10.58564	3.06E-15	3643874
C6_02480W_A	10.54955	7.42E-05	3639170
TRX1	10.47812	1.38E-11	3638950
C1_04460C_A	10.41696	6.68E-05	3646140
SAR1	10.4071	3.36E-07	3645865
LAP41	10.3268	3.64E-09	3636653
GVP36	10.32418	2.13E-06	3641772
CR_00990W_A	10.31582	0.009371	3640425
DBF2	10.2279	7.72E-08	3648101
SPC34	10.20197	0.009649	3641173

SEC12	10.14849	4.51E-09	3641639
C4_00020W_A	10.10986	5.25E-10	3648053
C3_01070C_A	10.08906	0.010531	3639832
NUO1	9.980297	7.5E-07	3647100
LYS1	9.98023	2.91E-08	6335429
PDB1	9.975561	2.23E-13	3641186
C1_02500W_A	9.975442	0.010585	3636795
MET6	9.975231	1.12E-14	3640084
C1_09020W_A	9.953088	4.62E-06	3634917
C4_03290W_A	9.915437	2.05E-09	3637887
APM4	9.913275	0.00013	3642297
C1_07590C_A	9.902728	1.78E-05	3646673
IDH1	9.880403	3.74E-15	3634893
C5_00080C_A	9.869909	1.67E-07	3647886
DUN1	9.868424	0.000151	3636494
HGT3	9.862764	0.010803	3643185
C5_00340W_A	9.856325	6.88E-07	3641555
PIL1	9.820645	7.51E-16	3644769
C4_04600C_A	9.788027	0.001163	3641156
PDX1	9.787909	4.3E-13	3636128
C1_14060W_A	9.762363	2.62E-10	3643344
SEF2	9.737164	0.001024	3647052
C6_01360W_A	9.663895	4.76E-05	3641608
C2_09050C_A	9.658931	1.93E-05	3645791
C2_07650C_A	9.651127	0.001224	3642342
PGA63	9.649538	7.18E-14	3639524
GLO2	9.567841	1.38E-06	3644067
SSH1	9.562455	1.49E-06	3642504
CWH41	9.556823	4.49E-06	3646533
APL4	9.525645	6.15E-07	3642565
SCP1	9.523702	0.012547	3646554
CRD2	9.482864	0.014065	30515234
SER33	9.435803	6.02E-12	3642030
INP51	9.414225	2.53E-05	3643806
OSM2	9.40754	2.78E-07	3636193
SNF5	9.367053	9E-07	3648297
HGT2	9.346394	1.34E-10	3644579
RPL24A	9.334271	2.41E-14	3641174
CDC12	9.267449	2.11E-08	3636809
NRM1	9.248632	0.001536	3639343
IQG1	9.233926	3.31E-09	3637052
PGA45	9.230548	0.019246	3639875
GLT1	9.177768	3.25E-13	3639519
NUC2	9.153955	1.19E-10	3637030
CLC1	9.134909	1.3E-08	3635662
HMG1	9.10757	1.17E-07	3644687
RIP1	9.08455	1.93E-12	3635204

C5_02210W_A	9.038274	7E-06	3637759
CR_08480C_A	9.031499	3.23E-06	30515442
SRB1	8.991735	6.57E-14	3647454
C6_03130W_A	8.983957	0.00029	30515341
DED81	8.833137	2.19E-14	3646227
ADK1	8.832744	3.34E-09	3644533
C3_03510C_A	8.814987	4.11E-07	3638310
CR_07170W_A	8.79814	7.02E-09	3645271
ERG10	8.793385	1.37E-11	3648275
C4_04800W_A	8.781594	1.59E-11	3641113
C4_04970C_A	8.770218	0.000325	30515274
KIP2	8.695625	0.000235	3643954
C5_04950C_A	8.67548	9.29E-06	3636422
C6_00930C_A	8.672617	1.56E-07	3644032
FRS2	8.670156	7.75E-11	3636824
C2_02260W_A	8.648824	2.98E-06	3635778
MNT1	8.59365	1.32E-13	3636585
ATP5	8.589924	2.17E-14	3640521
SEC18	8.583426	3.93E-11	3636208
TOP2	8.57883	7.59E-07	3642777
C1_01800W_A	8.570027	0.0024	3645407
C6_00110C_A	8.561993	1.14E-05	3647896
EGD1	8.560097	1.16E-10	3645340
CR_04880W_A	8.539595	9.98E-05	3647309
FRP2	8.505931	0.000105	3638008
SUB2	8.495974	7.98E-13	3645033
ERV29	8.47654	1.49E-05	3635672
ERP5	8.462063	0.000131	30515046
C4_01460C_A	8.439097	0.002626	3635622
C2_02020W_A	8.405604	7.85E-07	3635769
BMH1	8.351104	2.51E-13	3636810
NUF2	8.330328	9.97E-05	3642613
MET13	8.31746	1.45E-07	3638382
C6_04280W_A	8.312369	9.65E-07	3646279
CR_10440W_A	8.302457	0.023409	3638976
C1_13270W_A	8.276623	2.16E-05	3636144
RPS15	8.261988	2.36E-13	3635194
TEF2	8.260696	1.11E-13	3646502
LSP1	8.248248	5.14E-13	3648025
C1_11280W_A	8.225708	1.44E-06	3644554
MEP1	8.179253	6.71E-09	3636553
EXO1	8.142866	0.000503	3641583
CR_00130C_A	8.132645	1.48E-06	3639709
C5_05230C_A	8.097068	1.59E-12	3636470
CR_03560W_A	8.073457	0.003847	3643217
C5_01960C_A	8.069524	3.25E-08	3643738
C7_02520W_A	8.060107	0.026137	3636941

APL2	8.044194	7.37E-07	3638343
TVP18	8.004739	0.000453	3634844
EGD2	8.001392	2.64E-10	3635284
RHB1	7.986076	0.003596	3635232
ARC35	7.983354	1.06E-05	3639823
CSH1	7.98235	4.58E-08	3646427
CAT2	7.977744	5.45E-12	3635659
PDA1	7.964682	2.49E-13	3642902
SOD1	7.964237	8.92E-05	30515248
CR_03270W_A	7.961393	0.000502	3645101
CR_10140W_A	7.956831	6.61E-13	3638972
C6_00770C_A	7.938882	0.024925	3647079
CR_10530W_A	7.938789	0.024768	3639042
ADE6	7.931815	1.81E-12	3647304
C2_01260W_A	7.921272	0.003656	3638792
MEU1	7.917262	8.51E-06	3645635
CEK1	7.914233	0.000496	3642789
RPP2A	7.880257	3.16E-09	30515439
TRP4	7.867648	0.004568	3642904
C1_12240C_A	7.858526	9.51E-06	3642023
ESP1	7.852226	2.85E-05	3640197
C7_02100W_A	7.833026	5.49E-11	3637023
C5_03120W_A	7.825839	0.026042	3637705
C1_06920C_A	7.825563	0.02591	3639525
C2_06610C_A	7.825287	0.026227	30515140
ERG2	7.817166	0.035927	3639347
DOT1	7.769428	2.01E-06	3642236
HBR2	7.766753	9.67E-14	3646273
C1_04780C_A	7.737373	0.00013	3644774
CDC20	7.725812	1.27E-05	3644057
C2_10200W_A	7.72461	0.026207	3643928
C4_06140C_A	7.712613	0.025553	3642821
CR_09010C_A	7.705783	1.87E-09	3641892
RFA1	7.705173	1.15E-06	3638823
C4_04920W_A	7.70055	0.00013	3641172
C7_04000W_A	7.669293	4.31E-05	3643114
CUP5	7.656331	6.7E-08	3635174
C5_04550W_A	7.640674	0.000719	3636508
QCR7	7.636371	1.49E-06	3639233
C1_02700C_A	7.6161	7.36E-07	30514972
SDH12	7.588178	5.09E-12	3642775
SMC1	7.520947	0.00658	3643221
ABG1	7.518021	1.38E-05	3647715
TPD3	7.515184	1.56E-08	3638512
RIC1	7.505374	0.000753	3639331
C1_08040W_A	7.494531	1E-04	30515028
C1_04010C_A	7.489999	0.030053	3646426

ARE2	7.483729	2.17E-08	3643526
CHS8	7.480968	1.87E-11	3640532
C3_07880C_A	7.47514	0.028598	3647470
C5_05020C_A	7.455769	0.000216	3636486
CR_00060C_A	7.40028	1.82E-08	3639673
MES1	7.397176	4.39E-10	3636516
AHP2	7.382927	8.34E-06	3636977
POT1	7.377204	3.62E-08	3639707
CR_08610W_A	7.368315	5.59E-05	3642109
BUB1	7.339932	1.2E-06	3637922
C4_00910C_A	7.322281	0.000963	3635679
ASN1	7.318663	3.65E-08	3645790
C1_02290C_A	7.315708	0.000281	3644568
ATP17	7.299493	2.43E-08	30515364
ADE1	7.286557	6.6E-08	3639722
LIP6	7.260279	0.030829	3634890
C1_08800W_A	7.243593	9.49E-06	3634791
DAP1	7.23259	0.001036	3641021
C2_05290C_A	7.2319	0.001105	3643472
ARC40	7.230648	1.15E-08	3644866
CPY1	7.229095	4.8E-11	3646721
NDH51	7.226601	3.43E-10	3645444
PMT4	7.204461	3.14E-06	3644096
HIS7	7.203904	1.85E-05	3647868
MDH1	7.191796	2.06E-11	3639667
C3_07230W_A	7.171784	1.24E-06	3638531
ALS1	7.15604	1.49E-10	3640280
TOS4	7.151277	2.54E-08	3644548
KIP3	7.13671	0.000556	3641716
CR_07110C_A	7.133871	0.005547	3648224
PLB5	7.119876	1.49E-06	3640750
C1_11760C_A	7.105755	0.000308	3645328
C2_02310W_A	7.10149	1.24E-05	3635809
ALG8	7.077986	0.005088	3636562
FGR29	7.064609	0.000329	3641190
AOX1	7.063044	0.001487	3634946
C7_01460C_A	7.050898	0.001072	3636958
C3_05280C_A	7.038504	0.000352	3635202
C1_10750C_A	7.015732	0.005901	30515043
VMA5	7.013356	5.82E-06	3642316
VPS51	7.00608	0.00119	3639242
WH11	7.001238	7.46E-08	30515117
CRN1	6.997696	7.9E-09	3637034
REX3	6.996203	0.043549	3636164
C1_11860W_A	6.980828	5.45E-09	3642009
C2_05170W_A	6.94819	3.11E-05	3643462
CAS1	6.914394	0.001175	3645324

CR_01070W_A	6.883441	0.006101	3640382
INO1	6.869789	1.27E-10	3638966
C1_12180C_A	6.868758	0.001154	3642038
CR_00910W_A	6.840022	6.49E-10	30515371
ENA21	6.834258	4.82E-12	3641392
PYC2	6.830886	7.34E-12	3637323
RAD53	6.82819	0.007698	3645633
ZCF5	6.824694	0.000385	3641819
SOD2	6.820349	1.44E-07	3640231
C7_03640C_A	6.818895	0.006536	3646223
C1_07510W_A	6.818855	0.006383	3646664
C1_10520W_A	6.807548	0.036406	3647191
VMA11	6.791209	0.000372	3637054
MYO1	6.790893	3.42E-06	3647248
CDC42	6.767214	1.45E-05	3646488
SKN1	6.743155	1.68E-07	3641755
C6_04320C_A	6.729441	0.279383	3646289
C1_14240W_A	6.728489	1.06E-07	3643306
FAS2	6.718843	1.31E-10	3635222
DAG7	6.713754	0.000164	3635607
CIT1	6.713244	1.98E-10	3643201
CCC1	6.685775	1.26E-06	3645645
CAM1-1	6.675436	1.19E-07	3646358
C3_01610W_A	6.662433	4.92E-10	3636664
C7_00410C_A	6.652541	1.77E-05	3638033
C1_02650W_A	6.643918	4.13E-06	3636818
VPS1	6.630053	1.24E-10	3642656
RKI1	6.623953	0.007256	3636574
C3_06860C_A	6.620996	9.79E-11	3638541
RPL8B	6.618596	2.91E-11	3635198
C1_07820W_A	6.59552	0.001585	3640735
CR_05800C_A	6.590344	0.041464	3646706
VMA8	6.585132	8.29E-06	3642798
MET14	6.584723	8.47E-06	3641547
POL1	6.579488	0.000371	3635144
GLC3	6.560233	6.93E-10	3639200
CAP4	6.49397	0.008477	3637335
CR_01100C_A	6.468083	0.041663	3640378
RTT109	6.468083	0.041659	3639678
PDC11	6.458444	3.35E-11	3642780
C1_00450C_A	6.442262	2.68E-05	30514955
C1_12400C_A	6.440275	0.000554	3642059
TFS1	6.427911	3.88E-10	3642655
C1_04030W_A	6.421364	0.001912	3646428
KGD2	6.404568	4.79E-11	3646036
NIF3	6.400499	0.000165	3646527
C3_00520W_A	6.364818	0.009049	3640513

MDH1-3	6.359944	3.17E-07	3641197
CR_01430W_A	6.326982	0.003105	3640138
RAM1	6.324815	0.010521	3647859
C1_05670W_A	6.316024	3.31E-07	3639899
GFA1	6.300353	2.13E-09	3636557
DPP2	6.300187	0.009802	3645341
RNR1	6.297802	3.61E-06	3642989
CR_05200C_A	6.277335	0.000807	3648080
JEM1	6.272742	0.011502	3643876
CYB2	6.26334	6.62E-05	3636213
PDR17	6.258088	0.012057	3643007
CR_07230W_A	6.254472	2.18E-05	3646031
VRG4	6.253959	3.08E-06	3641768
CR_05130C_A	6.241796	0.046355	3646401
C4_06460C_A	6.235314	0.010045	3642791
BRN1	6.232651	2.67E-05	3641815
SDH2	6.220707	4.7E-08	3646397
SCW11	6.205674	5.38E-05	3636521
PRN4	6.204669	0.000816	3639877
USO5	6.180711	0.000759	3638398
C1_00420W_A	6.177113	2.11E-06	3639286
POR1	6.158476	3.76E-11	3644698
ATP4	6.156497	4.89E-11	3643418
ILV2	6.142483	1.03E-10	3636552
CHS2	6.140342	3.81E-07	3641893
STV1	6.140194	4.41E-08	3646070
C3_06920W_A	6.134032	0.0001	3638511
C4_02420C_A	6.12811	0.046568	3637849
C4_03400W_A	6.122643	0.011896	30515256
GDE1	6.117566	1.28E-07	3636505
UGA32	6.095861	0.002771	3639333
C2_05130W_A	6.054936	0.000806	3643458
MNS1	6.029786	1.72E-05	3644692
ADH2	6.027469	9.06E-11	3640751
RPL43A	5.998833	8.56E-12	30515319
THR4	5.979792	4.61E-09	3637711
DLD1	5.976843	1.67E-07	3643025
TSC11	5.97597	0.010592	3645267
CR_07460C_A	5.956264	3.46E-05	3646049
FRE30	5.916825	1.09E-06	3646025
RAD10	5.91511	0.003824	3639884
ARP1	5.91097	0.011026	3646349
ACC1	5.908111	2.5E-09	3639688
C1_12140W_A	5.887463	0.000677	3642050
PEX8	5.881603	0.001072	3647393
C4_00770C_A	5.877988	5.34E-05	3645760
C1_02370C_A	5.870507	0.000204	3644576

CR_04730W_A	5.868274	0.003934	3647305
TOM20	5.849719	2.66E-05	3636817
C1_02910C_A	5.84594	0.011565	3636829
C1_10540C_A	5.804267	0.00155	3647193
SMF3	5.802754	3.58E-06	3638852
NPL4	5.801837	8.04E-05	3639820
ILV3	5.793283	5.86E-09	3636428
DPM3	5.778946	0.012901	30515245
CR_01320C_A	5.775006	0.000247	3640386
YSA1	5.773675	7.24E-05	3638516
BFR1	5.742031	1.91E-09	3635579
APL5	5.740953	4.02E-06	3638377
C1_01050C_A	5.733685	3.58E-06	3640170
CR_02260C_A	5.731343	0.001565	3644912
CR_05680C_A	5.725782	0.001985	3646684
C6_01750C_A	5.716154	0.012911	3641640
INT1	5.715845	2.91E-09	3637761
VCX1	5.712592	6.56E-05	3646508
C6_03330C_A	5.711887	6E-05	3639199
C2_08200W_A	5.697094	0.000288	3648342
C3_00210C_A	5.693884	0.019586	3640560
GLO1	5.652079	2.98E-07	3639312
DPB4	5.642266	0.005009	3638818
C1_08730W_A	5.641415	0.005165	3634938
MMD1	5.636684	2.16E-09	30515015
CR_02880W_A	5.62957	0.020545	3642584
FRE7	5.614624	1.22E-06	3646024
FTR2	5.611899	2.55E-05	3643303
CPR3	5.608074	9.53E-07	3635812
CDC3	5.603376	1.92E-09	3644719
C1_05490C_A	5.590814	2.49E-05	3642507
HXT5	5.570556	0.000401	3643209
C2_05560W_A	5.551218	0.005348	3645561
PMT1	5.551192	7.24E-09	3641393
EXG2	5.550784	0.006349	3636816
C4_03770W_A	5.521975	0.014902	3645354
PUP2	5.50868	1.11E-06	3646617
AGE3	5.507034	2.41E-05	3644565
SOU2	5.498613	0.024307	3642823
SSA2	5.496686	1.22E-06	3644711
PRS	5.474411	0.00701	3640100
HFL1	5.468663	0.01639	3636883
C3_01420C_A	5.460972	0.00087	3636634
C3_04630W_A	5.450273	0.000397	3635189
VMA13	5.446242	2.97E-07	30515402
VMA7	5.445143	0.007207	3637332
YCG1	5.440339	3.71E-05	3636639

C1_03440C_A	5.434821	7.63E-07	3636908
PMT5	5.432613	0.000736	3639011
ERG5	5.425501	2.97E-09	3641400
RPS7A	5.425276	4.36E-10	3636573
ERG24	5.419682	0.000887	3647716
C6_02530C_A	5.415046	0.006807	3639175
C2_03210W_A	5.392003	0.017721	30515098
SNG3	5.391887	5.99E-05	3646715
MSC7	5.373102	5.07E-06	3645214
C3_02820C_A	5.365627	3.85E-05	3638419
C5_04410C_A	5.357206	0.008961	3636410
STT3	5.355189	8.44E-06	3635743
C5_01070C_A	5.299235	0.000269	3642627
C2_07110C_A	5.263565	0.000874	3643505
C6_03050C_A	5.256979	0.022751	3639270
MSH6	5.252721	0.001436	3636161
C2_03450W_A	5.252023	0.001741	3637349
ZCF24	5.251255	0.001879	3645417
TPI1	5.246609	7.88E-10	3638482
CR_03020C_A	5.245983	8.13E-05	3642598
C3_04420W_A	5.232064	1.4E-07	3635180
MCM6	5.229292	0.012392	3640142
C7_03200C_A	5.219319	5.13E-05	3641342
RAD52	5.217558	0.001967	3647148
C3_05300C_A	5.216972	0.001854	3643646
C4_04860W_A	5.214003	0.002121	3641161
CR_05760C_A	5.197588	0.02151	3646695
RNH1	5.184566	7.39E-05	3639237
C2_02950W_A	5.176996	1.2E-06	3643012
FESUR1	5.176244	9.03E-07	3638514
C4_04160W_A	5.174977	1.24E-06	3641185
FOX3	5.155347	1.74E-05	3636629
C1_01250W_A	5.142195	3.9E-05	3640212
C6_02030C_A	5.134504	4.14E-05	3647562
NPT1	5.130168	1.31E-05	3643109
CMK2	5.128402	1.18E-05	3643961
MYO2	5.117601	5.33E-05	3636140
GCF1	5.116354	1.03E-08	3646498
SER1	5.096415	3.96E-06	3646909
FAA2-1	5.088345	1.76E-05	3644100
C4_01470W_A	5.06838	0.025291	3635621
C1_02890C_A	5.067815	0.025901	3636867
CR_06070W_A	5.065142	0.00012	3644840
AHP1	5.055044	1.57E-07	3637850
C1_06480C_A	5.046364	0.00012	30515008
C6_03950C_A	5.045722	0.002695	3640268
PGA10	5.042936	0.028227	3645009

C1_02970W_A	5.026444	1.02E-06	3636835
PET9	5.020347	3.25E-08	3641587
BNR1	5.009566	0.000381	3639672
GLO3	5.006096	7.55E-08	3640556
FAA2-3	5.005764	8.47E-05	3643125
DSL1	5.005491	0.007357	3645718
C1_01290C_A	5.002782	0.027532	3640184
C5_01410C_A	4.99555	3.92E-05	3647054
DNM1	4.984823	3.75E-08	3643673
C6_01560W_A	4.976411	0.003213	3641652
C1_08080C_A	4.959854	0.000221	30515029
C1_12880C_A	4.945698	0.000156	3636151
CR_01080W_A	4.939913	3.48E-06	3640381
LRO1	4.935507	5.12E-07	3639356
YPT31	4.92626	9.12E-06	3647999
PFY1	4.923416	9.43E-08	3640703
VPS23	4.915276	0.00813	3644401
PRD1	4.914413	7.06E-08	3642471
C1_01010W_A	4.906541	6.9E-05	3640181
ARP2	4.902807	1.62E-07	3641915
MTS1	4.887412	1.87E-06	3634873
TYR1	4.883943	0.000482	3635639
C2_07440C_A	4.879539	0.000127	3645200
C3_05880C_A	4.878534	2.77E-06	3641737
C3_02210C_A	4.872615	0.000497	3636641
CSP37	4.87006	2.95E-08	3640098
UBA2	4.866982	0.000637	3640701
C1_05320C_A	4.866522	0.001418	3642470
C3_01900C_A	4.860323	0.000943	3636559
ECI1	4.858585	4.99E-05	3642128
CR_04870C_A	4.856664	0.039929	3647310
IFD6	4.836424	0.001684	3644703
C7_00600C_A	4.83245	0.003074	3638031
SEC8	4.830078	9.23E-05	3647078
C1_11740W_A	4.824213	0.009624	3645317
C2_01320W_A	4.819669	0.00015	3638846
C1_03720C_A	4.818129	0.000108	3648216
C3_00850C_A	4.815724	7.79E-09	3647748
C1_02330C_A	4.803879	5.72E-07	30514969
C3_04080W_A	4.79482	6.74E-07	30515200
GRS1	4.793975	8.39E-10	3642474
TRP99	4.789115	7.35E-05	3642494
MAL2	4.787553	0.000463	3638946
C7_00150W_A	4.784335	5.07E-08	3637973
RTS1	4.769769	5.69E-08	3646557
C4_05790W_A	4.767678	0.000939	3641784
C6_03960W_A	4.758172	3.71E-08	3640269

GLR1	4.756128	1.46E-07	3647061
ARO9	4.752457	0.001798	3641773
C1_00210C_A	4.750482	8.78E-08	3639335
CAT8	4.741031	0.000446	3640745
C4_04090C_A	4.738215	0.000452	3641225
C1_07340W_A	4.721799	2.99E-06	3644472
ARO3	4.712065	6.9E-05	3635770
ABP1	4.704066	1.74E-08	3637875
KSR1	4.692708	0.004668	3646052
TPM2	4.689628	1.12E-09	30515441
C1_10090C_A	4.685886	1.1E-07	3634817
C7_03780C_A	4.678742	0.035372	3643119
RCK2	4.677816	8.23E-09	3643507
C2_05850C_A	4.677589	0.000452	3647472
C2_10860C_A	4.655657	2.87E-06	3641233
CR_00690C_A	4.638003	0.002033	3640390
C1_07010W_A	4.63582	0.005512	3639537
C1_07440W_A	4.630952	1.47E-05	3644482
ARO4	4.626754	3.83E-06	3647668
C1_10730W_A	4.625811	7E-05	3644404
C4_00190W_A	4.614349	0.035941	3645030
APM1	4.614221	8.66E-05	3636424
CDC14	4.604785	1.73E-07	3647132
C1_00330C_A	4.601263	0.001086	3639296
IDH2	4.601153	8.14E-10	3642979
ASR2	4.600388	6.7E-07	3641907
C2_03340W_A	4.595621	0.000506	3637392
SNL1	4.590616	0.000401	3647192
AAT1	4.586314	3.98E-08	3643468
PEX6	4.586089	1.3E-05	3643436
ATP14	4.584114	5.11E-06	30515134
RPS12	4.58385	7.57E-09	3638438
IPL1	4.57842	0.001273	3645876
SHP1	4.561122	6.27E-06	3640083
C2_01930C_A	4.543582	1.97E-05	3635793
C2_08930W_A	4.535987	5E-05	3645795
ARA1	4.534344	2.5E-05	3642331
TRS20	4.525319	0.044973	3640214
C7_01320W_A	4.521916	0.000193	3645896
C3_01440C_A	4.517315	0.401033	3636632
GSP1	4.517022	2.24E-09	3646925
ACS1	4.515386	3.56E-08	3643941
C3_02570W_A	4.503259	0.000207	3638375
CR_02500W_A	4.498662	0.00309	3647696
CSR1	4.493223	0.020391	3641162
C1_00630W_A	4.492052	0.003043	3639380
CR_05010W_A	4.487458	0.042429	3646389

C1_07990C_A	4.486226	1.02E-07	3640697
CAC2	4.484445	0.039772	3646978
C4_06160W_A	4.484286	0.040598	3642819
NDE1	4.473821	1.85E-08	3638354
STE24	4.467307	1.64E-06	3645040
MAK32	4.460116	0.014736	3645411
C5_04420W_A	4.454906	0.001578	3636411
HXK2	4.451651	1.01E-06	3641015
XUT1	4.449896	0.000846	3642785
C7_00520W_A	4.448411	3.38E-07	3638001
CR_02620C_A	4.443864	0.000143	3642567
RHO1	4.44342	9.23E-06	3642564
LHS1	4.442844	0.000117	3643032
C6_04120C_A	4.426831	0.000619	3647974
C2_10470C_A	4.422678	0.000164	3641196
ARP9	4.418347	0.000785	3639829
C3_03270W_A	4.413936	0.041585	3638321
CR_03530W_A	4.405057	1.64E-08	3643204
C2_06920C_A	4.381967	0.00306	3643524
OBPA	4.380691	2.93E-07	3643764
GRE3	4.368451	3.71E-07	3637811
MNN26	4.364347	0.000153	3646240
C4_01730C_A	4.362519	7.05E-05	3635588
C4_00380W_A	4.354827	0.003258	3645015
END3	4.353582	0.000686	3636644
C6_01420C_A	4.348461	2.69E-06	3641602
C2_00730C_A	4.342922	0.00092	3638913
COX6	4.311949	2.82E-08	30515099
SAC6	4.305262	1.04E-07	3639213
COQ3	4.299399	0.000705	3641630
C2_07980W_A	4.29739	0.002985	3642312
C2_07420W_A	4.289359	0.00206	3645202
DUT1	4.284268	0.018928	3640188
C4_04140W_A	4.282011	0.011843	3641220
PEX1	4.257856	2.9E-05	3636943
C2_09970C_A	4.253372	6.82E-06	3643820
GEA2	4.250071	7.45E-05	3636646
IFR2	4.242577	9.95E-07	3645100
CR_09570W_A	4.240312	0.018676	3647098
CR_05880W_A	4.237565	0.009458	3646702
SHM1	4.235374	4.25E-07	3646724
PHB1	4.219669	4.65E-05	3645641
SUR7	4.218936	5.93E-08	3641610
MNN24	4.207759	0.000182	3638789
TFC4	4.204738	0.000169	3638420
HYU1	4.204463	0.000105	3643024
LYS21	4.203907	3.04E-06	3644763

C1_13320C_A	4.203619	3.39E-06	3636149
CR_05900W_A	4.202481	0.009643	3646681
CYS4	4.182591	1.93E-06	3645429
RPS8A	4.176155	1.82E-07	3645565
ANB1	4.175762	1.2E-07	3641648
C4_02400C_A	4.172961	0.021573	3637851
RPP1A	4.168832	7.73E-07	3636839
C4_06810C_A	4.166427	7.4E-05	3642916
ARL3	4.165897	0.000916	3644397
YCP4	4.150023	3.66E-07	3648032
SPF1	4.12357	2.36E-05	3646573
SUN41	4.122526	3.27E-07	3647067
C2_09560C_A	4.099057	0.00015	3643813
C5_05200C_A	4.09829	0.003876	3636467
ATP7	4.097686	0.000392	3646672
C2_06950C_A	4.09697	0.018401	3643527
C5_01550C_A	4.093292	1.14E-06	3647063
C1_02780W_A	4.090323	0.000216	3636871
FUM12	4.075597	3.83E-07	3638489
C3_01510W_A	4.073814	0.002738	3636571
SMC4	4.067979	4.71E-06	3641578
C3_01590W_A	4.066357	0.012157	3636666
MNN2	4.060072	0.000918	3644405
C3_04810C_A	4.056738	9.88E-08	30515205
NAM2	4.054828	0.001953	3640275
LIP8	4.052459	0.003164	3646727
C6_02470W_A	4.052064	0.00021	3639169
CR_06770C_A	4.041355	0.004406	3647313
C4_06940C_A	4.040864	0.004515	3642928
CR_07780W_A	4.040415	3.82E-05	3647352
PAN3	4.039926	2.29E-05	3636464
C1_01210W_A	4.033945	0.013063	3640209
C2_07270W_A	4.033689	0.00339	3643474
SAH1	4.033283	4E-07	3636452
CR_07350W_A	4.022807	4.21E-06	3646055
YBN5	4.019142	5.39E-07	3644781
PGA14	4.011755	4.92E-08	3641560
C6_01050W_A	4.000316	0.001088	3644015
CR_02680W_A	3.984732	0.004817	3642573
RPS1	3.984499	6.74E-07	3636924
LAG1	3.984384	0.004717	3640383
RPL18	3.979595	2.7E-07	3635156
ALS2	3.964829	1.79E-06	3645724
MPT5	3.964565	0.000394	3636205
C3_00790W_A	3.962777	0.003973	3648186
RAD51	3.961649	0.000167	3644944
HEM14	3.960744	0.001175	3634923

C3_06410C_A	3.960306	0.021888	3642261
CTP1	3.960235	0.02257	3635141
C7_01740C_A	3.957879	0.011399	3637010
C2_08650W_A	3.95152	0.000335	3643908
GCV1	3.951309	0.000164	3639172
YPD1	3.951101	0.00011	3644458
GTT11	3.943492	0.000474	3645644
CR_08900C_A	3.940475	0.000212	3641908
HGT1	3.939947	2.64E-06	3645420
C2_01690W_A	3.936957	5.98E-05	3635728
C4_05810W_A	3.927903	5.99E-07	3641786
RPL19A	3.923047	6.3E-07	3635257
MUQ1	3.907762	0.000123	3638898
CR_01180W_A	3.902384	0.001331	3640427
HEM15	3.901109	2.14E-06	3645211
ALG6	3.899134	0.005546	3647316
C4_02620C_A	3.89676	2.2E-06	3637834
MVB12	3.894104	0.013322	30515400
CDC7	3.883575	0.003607	3643424
TFP1	3.882453	3.22E-07	3636662
C3_07140C_A	3.873528	0.006462	3638439
GOA1	3.86977	0.024196	3641153
RFC2	3.869587	0.024429	3637939
RPL10A	3.869253	5.19E-07	3645868
ERG9	3.869122	0.000161	3643913
C6_00910C_A	3.866852	5.8E-06	3644030
SEC27	3.866666	2E-06	3645870
CR_08450C_A	3.86336	0.006153	3642133
GDB1	3.863021	4.8E-07	3647409
CDC50	3.837507	0.001414	3640302
STE13	3.835971	0.004669	3647597
PLD1	3.830172	4.59E-07	3645329
MUM2	3.830155	0.000357	3636432
ETR1	3.829265	1.51E-07	3640561
C3_04460W_A	3.828703	0.000206	3635208
CDC73	3.828481	0.000228	3636830
C4_04230W_A	3.822463	0.028248	3646857
C7_02080W_A	3.819103	0.013938	3637025
RIM1	3.816919	0.000502	3639898
C5_01590W_A	3.813729	0.015388	3647041
CR_02690W_A	3.813505	0.007806	3642574
RPP1B	3.8098	0.000187	3643104
C6_03620C_A	3.790273	0.000291	3640297
SPC3	3.782649	0.01352	3636121
C1_10460W_A	3.781307	0.000237	3647183
CR_01700C_A	3.781128	0.000391	3640129
SHM2	3.777157	4.73E-07	3640306

RPS26A	3.775835	5.14E-07	3635787
C2_03290W_A	3.768041	0.002944	3637429
ADO1	3.760542	6.93E-05	3639274
C4_04440W_A	3.758488	0.001593	3641177
ORC3	3.758406	0.001571	3645639
C2_01160W_A	3.756832	0.008106	3638861
FBA1	3.756218	2.01E-06	3635585
KAR2	3.753245	2.07E-06	3638830
RPL28	3.750818	3.57E-07	30515386
ERV1	3.748015	0.014649	30515385
C1_05990C_A	3.747972	0.014911	3639871
CR_01280C_A	3.747556	0.015513	3640400
STP1	3.740921	0.000555	3635152
XKS1	3.733274	1.31E-07	3648306
SFH5	3.732854	0.004319	3634884
EFB1	3.730971	1.02E-06	3641125
MRP8	3.729068	0.009682	3641178
MON2	3.727079	0.000201	3636201
CR_09590W_A	3.726721	0.003141	3647095
GWT1	3.726334	0.003449	3645575
SPE1	3.72281	1.84E-05	3639327
MAM33	3.713949	0.000103	3643103
C6_02410W_A	3.712931	4.96E-05	3645884
FLC2	3.708819	2.72E-06	3647962
ECM39	3.706305	0.003783	3641386
RPS16A	3.704572	3.38E-07	30514974
C6_02160W_A	3.700408	0.010371	3645889
PGA33	3.698465	0.002363	3637350
MCI4	3.696198	0.000587	3640118
CTA2	3.69464	0.000358	3639372
COX4	3.682752	3.52E-06	3635737
CDC24	3.660835	7.37E-05	3643737
TLO9	3.657805	0.000566	3648051
ADE5,7	3.654069	1.8E-06	3640719
RNA1	3.652082	2.18E-05	3636595
RAD7	3.645627	0.001476	3635833
CSO99	3.642974	0.01967	3642786
CR_02770C_A	3.625939	0.002443	3642581
CHS6	3.625685	0.002427	3641377
C7_03410C_A	3.624226	0.011	3646716
OCA1	3.614858	0.012246	3643929
C3_04970C_A	3.612411	0.000403	3635164
RPL17B	3.60915	9.88E-07	3645439
C5_05350W_A	3.608596	0.019559	3636401
C1_03410W_A	3.608216	0.018496	3636905
FCY2	3.608123	0.00272	3638365
CR_07640C_A	3.596799	0.040951	3647258

C7_04280C_A	3.595174	0.000325	3643173
C2_05800C_A	3.59007	0.000574	3645584
TIP1	3.586837	0.012678	3636512
HPD1	3.579506	0.022397	3639239
AYR1	3.558078	0.013359	3647754
PKH2	3.55718	4.92E-06	3642058
CR_08000C_A	3.553824	4.72E-06	3644150
MED1	3.546507	0.00164	3637418
IST2	3.545615	5.9E-05	3646662
CR_08920W_A	3.54413	0.001748	3641911
C2_00880W_A	3.54026	0.003909	3638897
CR_08670C_A	3.538343	0.020608	3642126
C4_02720C_A	3.53828	0.000329	3637857
C5_01440C_A	3.538074	0.020382	3647043
C3_04590W_A	3.53289	0.000153	30515204
C1_07690C_A	3.530206	7.81E-05	3641767
C5_01420W_A	3.530033	0.004194	3647055
RPL20B	3.527839	4.3E-07	3635615
SGT2	3.527344	1.87E-05	3642978
C4_02470C_A	3.510979	0.000184	3637869
C4_06240W_A	3.50104	0.01517	3642807
C1_07780W_A	3.497045	0.000443	3647955
CR_05860W_A	3.494455	0.001249	3646703
C1_11120C_A	3.48669	0.000148	3644412
C1_07840W_A	3.485454	0.000169	3640737
C3_07110W_A	3.482979	0.000768	3638442
CBP1	3.476388	0.000239	3641920
C5_04770W_A	3.476028	0.006288	3636444
TAF14	3.472395	1.68E-05	3637357
PGA54	3.468086	0.001325	3637929
YHM2	3.466713	0.000446	3647137
PGA53	3.46181	0.003309	3635599
C3_04780C_A	3.461703	0.003545	3635214
C3_01140W_A	3.452684	0.00645	3639902
GLX3	3.448076	1.47E-05	3638325
PST1	3.444442	0.000145	3643520
CR_05750W_A	3.438599	1.18E-05	3646696
SPC98	3.437303	0.00541	3640113
RTA2	3.436408	5.56E-06	3646567
FGR38	3.433921	0.004427	3645412
CR_01300W_A	3.433149	0.002974	30515373
APG7	3.43141	0.021263	3646615
CAM1	3.429144	3.33E-06	3641734
SUC1	3.423902	0.002289	3641916
CR_09150W_A	3.42285	0.005321	3641880
MNN10	3.418541	0.003263	3644997
SMC5	3.404166	0.042486	3645103

POL3	3.403139	0.000665	3641359
C5_00280C_A	3.400455	0.0032	3641581
C3_03560W_A	3.398038	0.006437	3638299
DQD1	3.39686	0.006117	3643488
C1_01910W_A	3.39646	0.000487	3645425
PTC6	3.396214	0.004546	3647351
C1_02440C_A	3.395914	0.020077	3636848
C2_05220C_A	3.391038	0.000394	3643466
C2_09780C_A	3.387166	0.00371	3643805
C1_04090C_A	3.364436	0.030022	3644697
C5_05040W_A	3.363441	0.028084	3636488
ARG2	3.363339	0.029393	3647533
FAS1	3.362742	3.84E-05	3641593
GPX3	3.362608	0.029294	3644471
C2_07100W_A	3.360494	0.000414	3643504
C4_05080C_A	3.359366	0.01892	3641129
C4_00590C_A	3.359325	2.72E-05	3645772
RPN7	3.355918	6.16E-06	3641909
CR_02410W_A	3.351883	0.00424	3644928
C1_03490W_A	3.351363	0.004223	3636914
SEC34	3.350795	0.000628	3644455
CR_07700W_A	3.34906	0.002926	3648195
C3_01910C_A	3.34821	0.000723	30515182
RHD1	3.347783	0.001581	3647531
HDA1	3.346321	0.000268	3640154
ACE2	3.338907	0.000371	3646034
RIB5	3.336063	0.000869	3636499
CR_04020C_A	3.332152	0.00848	3640969
CR_04500C_A	3.330921	0.014974	3641014
C5_03950W_A	3.330749	0.019255	3647445
C3_01310W_A	3.319964	0.031548	3636620
NUP159	3.313424	1.18E-05	3647458
C3_06350W_A	3.309086	0.000893	30515215
C1_02430C_A	3.307588	0.000458	3636846
C6_01460C_A	3.302812	0.021252	3641623
TOM22	3.300819	0.004397	3647650
FAT1	3.29866	0.000186	3647083
C5_02400W_A	3.297771	0.000838	3637724
SEC26	3.294189	2.18E-06	3641005
SSB1	3.293691	1.74E-05	3642095
C6_01370W_A	3.291898	0.002564	3641607
GNA1	3.284766	0.010695	3637397
CDC68	3.284272	1.25E-05	3642787
C5_05440C_A	3.283277	7.76E-05	3636431
RBK1	3.281855	0.003249	3646830
SEC24	3.280379	5.53E-06	3634939
SEC10	3.279974	7.51E-06	3642961

FUM11	3.271822	0.000205	3641016
IFA21	3.270178	0.03628	3641344
C3_07460W_A	3.26509	0.001203	3638480
SEC3	3.259934	0.00027	3642806
CR_05480W_A	3.257999	7.22E-05	3648238
C3_03340C_A	3.25545	0.007212	30515195
RPS14B	3.255128	1.19E-06	30515006
GIN1	3.247316	0.042307	3644539
C4_06710W_A	3.245507	0.020348	3642948
C2_00870W_A	3.244994	0.0209	3638804
C2_09930W_A	3.243148	0.001683	3643824
KRE5	3.242833	0.001032	3638384
CR_09510C_A	3.234259	0.000943	3647105
C5_02900W_A	3.234096	0.001424	3637747
RHO3	3.230005	0.009129	3643448
C1_01490W_A	3.229585	3.02E-05	3640226
C1_11920W_A	3.226229	0.001905	3642003
YPT72	3.226025	0.006322	3643523
CR_07600W_A	3.223533	0.005872	3644849
PKH3	3.219103	0.000224	3646075
C4_01830C_A	3.216966	0.001331	3635593
C1_01000C_A	3.216541	0.021287	3640180
C2_08160C_A	3.21359	0.000313	30515152
C7_01480W_A	3.212716	0.01227	3636956
STE23	3.210106	0.000633	3639236
C3_01940C_A	3.207247	0.005735	3636600
C6_03040C_A	3.207049	0.006227	3639269
NIT3	3.204857	0.003849	3644408
RAD16	3.201873	0.000256	3636874
C6_04250W_A	3.200743	1.4E-05	30515345
PHM7	3.199355	2.18E-05	3642329
MNN21	3.199315	0.00063	3648124
C1_08780W_A	3.198036	0.000425	3634789
LAP3	3.195651	0.001323	3641012
C3_05550C_A	3.188671	0.004342	3643659
GYP1	3.186341	0.000448	3641138
PPE1	3.184616	0.036072	3635802
C5_02750C_A	3.180285	0.000931	3637740
ERG8	3.17855	0.000891	3635640
C7_03660C_A	3.175239	2.82E-05	3646226
MSI3	3.171352	4.75E-06	3639821
FOX2	3.167592	0.000688	3648327
GAT1	3.154265	0.014147	3641794
DAM1	3.153382	0.004398	3634820
C3_05510W_A	3.153182	0.035062	3643672
RAP1	3.151974	0.010699	3643945
HGT7	3.150341	2.73E-05	3638876

RPL6	3.148178	1.1E-05	30514975
HOC1	3.142631	0.001611	3641605
C6_03470W_A	3.142052	0.001034	3640253
ARO2	3.13832	0.001321	3648190
COQ5	3.137043	0.001023	3643416
ZCF39	3.135291	4.43E-06	3639037
PEX3	3.132599	0.005334	3644484
LYS2	3.13254	4.62E-05	3636875
C1_02320C_A	3.131304	5.14E-05	3644572
BUD7	3.128229	8.51E-05	3647228
TOM70	3.128178	0.00014	3647645
C1_09070W_A	3.118471	0.000492	3634773
APN1	3.116021	0.006554	3642263
CR_08050C_A	3.114194	0.000605	3644133
PMT6	3.113565	0.000848	3641116
ECM4	3.112309	5.56E-05	3640144
CAR2	3.105354	7.48E-06	3645027
C5_03400C_A	3.103058	0.023593	30515308
AGP3	3.096279	0.000889	3641163
TKL1	3.095222	2.58E-05	3640767
C4_03720C_A	3.090452	0.006721	3645359
C4_03810W_A	3.08565	0.009402	3645350
C4_01910W_A	3.085545	0.008856	3635635
C1_14160W_A	3.085138	0.040849	3643322
C5_00060C_A	3.074621	0.024467	3647889
C6_02490C_A	3.073178	0.008853	3639171
SRV2	3.072723	5.03E-06	3640938
PGA52	3.064042	5.34E-05	3647908
C6_00200C_A	3.062663	0.002172	3646082
PLC1	3.060983	0.007898	3647860
YMC1	3.057785	0.007895	3644474
EIF4E	3.053105	0.00011	3638981
THS1	3.047925	6.27E-06	3647882
C4_04720W_A	3.046814	0.001862	3641137
C2_07080C_A	3.0464	0.025068	3643502
C3_00050C_A	3.044486	0.010604	3640570
C3_02190C_A	3.041447	0.003491	3636642
C4_00410W_A	3.040345	0.010441	3645019
RPS30	3.039023	9.55E-05	30515390
RFC1	3.031285	0.0002	3645582
C3_07450C_A	3.013782	0.041534	3638481
SWC4	3.007539	0.00157	3639679
C3_01820W_A	3.000889	0.000493	3636584
C3_04800C_A	3.000757	0.00419	3635216
C3_03840C_A	2.998388	0.006992	3645628
C2_02930C_A	2.99493	0.000147	3643015
YDC1	2.990775	0.011376	3642962

C2_00830C_A	2.987527	0.000447	3638808
CR_01380W_A	2.98747	0.002323	3640131
EBP7	2.98468	0.008772	3642971
IFD3	2.980717	0.009494	3640211
C7_00420C_A	2.979631	0.018154	3638032
POX1-3	2.979092	0.00125	3636598
ARC15	2.977229	0.008168	3646043
C2_10700C_A	2.973869	0.003813	3641207
ATC1	2.968106	1.3E-05	3639570
GAD1	2.967662	7.42E-05	3645339
C6_04560W_A	2.96183	0.000902	3645977
CR_05840W_A	2.960836	0.012245	3646704
RPL13	2.958736	5.09E-05	3636841
C1_01360C_A	2.958527	0.000114	3640191
SEC62	2.954841	0.007882	3636860
SEC4	2.949947	0.000169	3640119
RPD31	2.944649	0.000919	3638520
C5_04940W_A	2.943528	0.045888	3636421
RPL35	2.941121	2.79E-05	30515207
C7_01930C_A	2.940597	0.013406	3637027
C1_03430W_A	2.938848	0.006701	3636906
C1_06270W_A	2.936776	0.036873	3647523
CCP1	2.934265	0.00012	3638350
CR_05450C_A	2.933039	0.032972	3648236
C6_03820C_A	2.929264	0.004137	3640288
C1_12660W_A	2.928841	0.008668	3646835
TOM40	2.927655	0.000102	3636968
ERG251	2.92526	2.52E-05	3635716
CR_07570W_A	2.921711	0.014758	3648004
C2_00510W_A	2.914803	0.00489	3638868
C4_00330C_A	2.911686	0.002272	30515227
ISC1	2.908304	0.048223	3646705
TMA19	2.906384	4.36E-05	3640399
CYC1	2.901159	0.000422	3643937
ENO1	2.899775	0.000138	3646493
HAM1	2.894448	0.007361	3647224
C6_02100W_A	2.887368	0.048454	3647554
AQY1	2.887315	0.000157	3642587
C6_04340W_A	2.880582	0.006041	3646291
C2_01870C_A	2.878718	0.014503	3635836
APT1	2.877676	0.007648	3635842
ATP16	2.874053	2.33E-05	3638963
ERG12	2.873917	0.012914	3634859
ERG4	2.869359	0.000241	3640511
FGR34	2.867689	0.006254	3639033
C5_00610C_A	2.853747	0.032633	3641585
ZCF32	2.853428	0.032702	3635213

IFK2	2.853195	0.032809	3637439
C7_03840W_A	2.851914	0.010364	3643146
PEX7	2.850533	0.003335	3644027
CR_04080C_A	2.850409	0.000699	3641023
ARC1	2.849672	0.000131	3645108
CR_01820W_A	2.848108	0.002499	3640164
APR1	2.843008	0.000162	3645204
TEF1	2.836941	0.000189	3648254
CR_08130W_A	2.83667	0.007815	3642070
RFC3	2.83633	0.113197	3647440
YST1	2.835917	5.22E-05	3643678
C1_02490C_A	2.835408	0.008736	3636853
DAO1	2.82365	0.00357	3636885
UBA1	2.820511	6.59E-05	3642286
C2_03700W_A	2.818752	0.043155	3637375
ILV1	2.818003	0.001073	3646918
SER2	2.817567	0.011478	3643006
TLO1	2.813785	0.008706	3639677
C2_01170C_A	2.808176	0.000526	3638860
C2_02840C_A	2.807479	0.005588	3642976
C1_10960W_A	2.805503	0.033765	30515047
C1_11100W_A	2.801827	0.036974	3644414
RDI1	2.800146	0.000922	3635167
GIT2	2.796699	0.012136	3642633
C1_02060W_A	2.795854	0.019758	30514968
VPS8	2.79577	0.01856	3640534
C2_06110W_A	2.786132	0.004375	3644094
C1_13280C_A	2.782211	0.017214	3636145
PMR1	2.777085	0.002947	3638043
C1_13260W_A	2.776972	0.010806	30515074
PRO3	2.775162	0.001367	3645036
BET2	2.770069	0.015385	3639025
RPP0	2.769389	0.000182	3638023
C3_03410C_A	2.768918	0.000184	3638353
CR_08620C_A	2.763712	0.012289	3642110
HEM4	2.761882	0.04741	3643942
C3_05410W_A	2.759297	2.72E-05	3643665
DLD2	2.757432	0.035848	3638475
CR_04330W_A	2.754253	0.02195	3640997
C4_05850C_A	2.754064	0.02447	3641791
ADP1	2.749013	0.000587	3642458
GPM1	2.748744	0.000432	3637431
GAL4	2.746369	0.000209	3641244
C4_00320C_A	2.745743	0.019526	3644999
HOG1	2.735309	0.000532	3637393
VPS52	2.733049	0.01221	3638996
C5_02050W_A	2.729699	0.000474	3643768

CR_07370W_A	2.728168	0.019734	3646053
C1_02240W_A	2.725256	0.001742	3644597
ADE2	2.722824	0.000238	3635259
CR_06620W_A	2.721227	0.006695	3646631
PRC3	2.717393	0.004343	3639889
CR_04560C_A	2.717051	0.041243	3640946
SCL1	2.716954	0.003106	3640510
RCY1	2.715111	0.027662	3643731
PGI1	2.713812	0.000244	3644847
DSE1	2.71337	0.012841	3643899
C1_00320W_A	2.713013	0.02486	3639297
CAK1	2.709639	0.035867	3637352
C3_04130W_A	2.70797	0.004188	3635283
UAP1	2.706645	0.001068	3637769
WBP1	2.703661	0.001505	3644398
BMT3	2.70082	0.017191	3640439
C3_01720C_A	2.698087	7.31E-05	3636601
C7_02170C_A	2.695104	0.00499	3636993
GRE2	2.691159	0.003721	3648026
MAC1	2.68957	0.012876	3637985
CDC27	2.689253	0.013666	3640405
C3_01180C_A	2.689131	0.002997	3647960
UGA2	2.688282	0.001506	3645436
MED18	2.679162	0.017108	3644103
KGD1	2.67906	0.000359	3647752
SLC1	2.67767	0.012321	3638324
FGR51	2.671861	0.031979	3647796
C4_01020C_A	2.671045	0.000288	3635606
CR_09070C_A	2.666707	0.009309	3641926
GCN4	2.663164	0.000147	3643823
C6_02940C_A	2.657709	0.003531	3639187
C2_01390W_A	2.65325	0.000558	3635726
EMP70	2.649862	0.003268	3637919
ANT1	2.646778	0.00933	3639516
CTA7	2.646075	0.00716	3637776
CR_00350W_A	2.642939	0.000626	3639686
C2_02090W_A	2.637886	0.039139	3635854
C5_03490C_A	2.634964	0.000404	3646967
CR_00290W_A	2.631573	0.000832	3639717
RIB4	2.630679	0.001966	30514995
LTP1	2.627867	0.017278	3640760
C2_00400C_A	2.627711	0.00082	3638821
ERG20	2.625046	0.003224	3645440
C1_10820C_A	2.621203	0.000753	3644435
C6_00240C_A	2.62015	0.010754	3646062
C6_00920W_A	2.61894	0.008849	3644031
RPL37B	2.617117	5.25E-05	30515053

PHB2	2.614797	0.002207	3635238
C7_02670W_A	2.6143	0.043667	3647651
MSH2	2.612268	0.012465	3642939
CR_07840C_A	2.609149	0.009144	3647346
URA5	2.606397	0.019886	3640087
CMK1	2.604916	0.000115	3635263
C4_07240W_A	2.601135	0.016936	3642945
SUL2	2.593367	0.006332	3637835
C6_00270W_A	2.587791	0.021087	3646066
PXA2	2.585171	0.004887	3642055
ATP20	2.582773	0.009255	3647602
POL2	2.582469	0.027856	3645733
C5_01220W_A	2.582089	0.011265	3642620
FRS1	2.581721	0.000642	3640121
C2_04370W_A	2.577269	0.003061	3645460
VMA4	2.575498	0.001217	3640111
C2_04170C_A	2.572753	0.002465	30515111
AKR1	2.572469	0.001054	3636166
C1_06200W_A	2.571828	0.016267	3648300
CR_08560C_A	2.566503	0.003646	3642142
ALG9	2.561417	0.013364	3644457
RAD32	2.560011	0.007579	3637340
C3_01210C_A	2.557503	0.004972	3636612
C2_09650W_A	2.550852	0.003112	3643794
C4_05860W_A	2.547488	0.042862	3641792
MAE1	2.546869	9.42E-05	3641615
C4_05340W_A	2.542633	0.017101	3646947
C3_07480W_A	2.542426	0.005532	3638529
LPD1	2.54085	0.000509	3646037
C3_00470W_A	2.540047	0.009617	3640520
SLA1	2.5343	7.49E-05	3635739
SAM4	2.534065	0.004166	3646506
VPS35	2.533583	0.003811	3645567
C6_04060W_A	2.533215	0.007957	3647828
CR_07080W_A	2.52969	0.001344	3647964
URA4	2.528743	0.009846	3642632
PTC7	2.528072	0.001841	3645000
CR_03460W_A	2.527906	0.016198	3643213
C7_03160W_A	2.522651	0.005182	3641345
C3_07740W_A	2.520764	0.001305	3638453
CR_09990W_A	2.51841	0.004691	3638930
CYP5	2.511092	0.000313	3642257
DPS1-1	2.503563	0.000334	3645134
SRO77	2.499242	0.001787	3646088
ERG27	2.492356	0.019348	3640432
TRP3	2.489628	0.027022	3642035
YCS4	2.488172	0.005536	3645196

RPS13	2.487588	0.000298	30515328
C6_00190W_A	2.487497	0.020257	3646080
C1_08050W_A	2.486993	0.005854	3640709
SEC72	2.473863	0.027006	3645768
C4_00780C_A	2.471997	0.004025	3645759
CTA24	2.468577	0.005869	3636475
C1_05900W_A	2.462711	0.001516	3639866
CR_09750C_A	2.462701	0.007133	3639009
MOB1	2.462004	0.006959	3639208
C1_05720W_A	2.459185	0.000516	30515000
C3_06970W_A	2.454576	0.021515	3638506
PRE2	2.453339	0.004691	3643490
C1_01900C_A	2.449031	0.012692	3645426
EFT2	2.446013	0.00118	3642998
CHC1	2.445387	0.000576	3647552
C1_10320W_A	2.444357	0.045043	3634907
GPA2	2.44413	0.002414	3636638
C3_05010C_A	2.441976	0.035125	3635169
C1_02830W_A	2.440717	0.02673	3636876
C2_03910C_A	2.440248	0.027534	3637426
CHS7	2.435739	0.008459	3639869
C4_02160C_A	2.433909	0.006655	3635668
C6_02280W_A	2.425732	0.022115	3645872
MSK1	2.424447	0.036804	3637032
C2_10460C_A	2.423413	0.006997	3641195
ECM15	2.418466	0.03603	30515218
PCK1	2.414148	0.003601	3639701
UBP13	2.413794	0.00461	3638879
C4_04330C_A	2.412366	0.003866	3646840
ANP1	2.410712	0.0283	3643917
HOM2	2.407474	0.001485	3635826
NUO2	2.40114	0.003989	3638381
C2_09620W_A	2.392128	0.028412	3643797
C5_04900C_A	2.390086	0.022216	3636416
C4_01780C_A	2.384834	0.023279	3635582
C4_04390W_A	2.377027	0.001206	30515267
C6_00890W_A	2.37389	0.005305	3644028
SGO1	2.373682	0.019537	3643464
C1_14180W_A	2.372211	0.046566	3643324
C1_05390C_A	2.370838	0.008018	3642479
LMO1	2.361374	0.010142	3641403
RPL3	2.36051	0.001015	3647719
RPN6	2.356105	0.007656	3645352
GUS1	2.353639	0.001806	3638029
AHA1	2.344386	0.011901	3638933
ENG1	2.336935	0.016768	3636886
SSO2	2.330251	0.002731	3643809

MRE11	2.328273	0.021677	3645898
C2_03360W_A	2.327879	0.000518	3637389
TIP20	2.327574	0.004103	3636457
C1_03690W_A	2.323247	0.011484	3648426
ERG7	2.322338	0.007266	3635761
PPH21	2.320409	0.005638	3636665
RAS1	2.31933	0.00184	3643927
URA2	2.318638	0.001749	3645728
MP65	2.318085	0.001478	3643951
C1_07050C_A	2.31778	0.020195	3639532
DAK2	2.314454	0.001546	3634895
PNP1	2.309622	0.009763	3638426
C1_10680C_A	2.309226	0.000491	3647678
CMD1	2.308905	0.004148	3646520
RPS5	2.307373	0.001331	3637700
GRP2	2.301284	0.001833	3637744
C5_00750C_A	2.299992	0.008819	3644121
C3_02120W_A	2.29482	0.011992	3636658
C4_04380C_A	2.290019	0.401147	30515266
C7_03030W_A	2.286638	0.034137	3641380
ADE17	2.285796	0.001221	3641024
RBP1	2.280759	0.004965	3647647
NHX1	2.279979	0.012989	3647141
C1_14500C_A	2.279778	0.002993	3643331
C3_03080W_A	2.27887	0.090375	3638397
ECM25	2.278617	0.086712	3636148
C6_00560W_A	2.278332	0.006212	3647143
RPL2	2.276699	0.002326	30515049
VAS1	2.270931	0.003787	3648340
CCT5	2.261564	0.002154	3643478
NAT2	2.25897	0.008646	3638837
C6_03880W_A	2.253122	0.011141	3640293
C5_02230W_A	2.250861	0.001406	3637708
RPN8	2.245724	0.0066	3643770
C6_00850W_A	2.243718	0.005154	3644010
C3_03760W_A	2.24261	0.021987	3645638
C6_00760W_A	2.24167	0.026348	3647080
C2_03900C_A	2.241641	0.005028	3637395
COQ6	2.240348	0.009472	3636878
ALA1	2.239482	0.001089	3640285
RPL16A	2.238254	0.002167	3639338
FGR24	2.237218	0.022659	3643288
C5_01350W_A	2.234538	0.018241	3642640
ARO8	2.232214	0.005072	3638828
CDC55	2.231221	0.000674	3635785
FDH3	2.229448	0.004507	3638923
RPS6A	2.227523	0.002729	3635701

CR_06570C_A	2.225811	0.003409	3648366
MRP2	2.224748	0.0378	3636498
C1_06670W_A	2.224142	0.01085	3639541
C7_02370W_A	2.222388	0.013023	3636930
URA6	2.221733	0.007141	3647988
C4_03320W_A	2.221445	0.026603	3648092
ROB1	2.217467	0.003767	3636212
SAS3	2.214652	0.029328	3640073
C2_01220W_A	2.213213	0.001106	3638856
C7_00140C_A	2.21118	0.039088	3637974
IDP2	2.209254	0.010233	3644934
COX8	2.208662	0.011193	30515130
AIP2	2.206114	0.003655	3638393
C2_05590C_A	2.204939	0.042491	3645564
SWD2	2.204668	0.044554	3646677
NSP1	2.201878	0.001094	3637430
C1_14170W_A	2.201021	0.009282	3643323
C2_07190C_A	2.199038	0.02338	3643513
C1_00920W_A	2.198507	0.029294	3639283
UBI3	2.194237	0.00386	3642934
AAT21	2.194103	0.000998	3647241
NMT1	2.19339	0.014647	3635624
C2_02170W_A	2.193094	0.02702	3635751
GCV3	2.191493	0.005086	3636194
GOR1	2.18672	0.042589	3636836
MED17	2.182995	0.013367	3636918
ADE13	2.181734	0.001107	3644863
RPN2	2.180574	0.000982	3642027
C1_08680C_A	2.179467	0.041598	3634933
RPN1	2.176737	0.00109	3636147
OXR1	2.169169	0.025019	3638372
SEC14	2.168452	0.000919	3641542
C1_03510C_A	2.166532	0.010656	3636916
HEM2	2.165182	0.024145	3637428
C1_11890W_A	2.164627	0.030118	3642006
RVB2	2.164439	0.004533	3637055
C7_04310C_A	2.161578	0.038607	3643141
C5_01540W_A	2.160371	0.002783	30515301
C1_13060C_A	2.15895	0.013049	3636123
C3_00890C_A	2.155623	0.04521	3647753
YBP1	2.151959	0.033077	3636187
BCY1	2.151589	0.00072	3638832
CR_09620C_A	2.147973	0.031586	6335435
SFI1	2.144131	0.019854	3641385
YVC1	2.143974	0.017868	3642303
C5_03550W_A	2.140723	0.001535	3646973
C2_08270C_A	2.140294	0.042653	3642347

C3_03930W_A	2.136925	0.030354	3645657
C1_08490W_A	2.135964	0.019833	3646492
C7_02560W_A	2.131888	0.024135	3637016
LTE1	2.13116	0.021013	3643495
SMP2	2.129471	0.001459	3635779
DBP5	2.128264	0.008379	3636564
GUT2	2.126708	0.004812	3642921
RIM20	2.124089	0.021685	3634809
C4_01760W_A	2.119189	0.042973	3635584
C1_12830C_A	2.118145	0.010123	3636176
C7_00250C_A	2.117688	0.022573	3637979
USO1	2.117252	0.011309	3648280
RPS4A	2.110327	0.004867	3641247
ADE12	2.10723	0.003325	3634869
COX7	2.106684	0.022986	3638339
ALG2	2.105947	0.041119	3647832
RPL5	2.105814	0.005488	3637057
C7_02780W_A	2.105438	0.004322	3641361
C1_09430W_A	2.103871	0.030965	3634855
KEX2	2.094382	0.013651	3634914
KEM1	2.090382	0.003166	3636222
C5_01940W_A	2.089869	0.01859	3643740
CR_10590W_A	2.085286	0.038371	3639003
C2_07610C_A	2.084398	0.022782	3645195
C7_04210C_A	2.083479	0.002481	3643121
C1_01930W_A	2.080831	0.003258	30514966
C3_07030C_A	2.079511	0.029259	3638517
C1_05780W_A	2.077794	0.014067	3639888
C1_10280C_A	2.074406	0.025713	3634902
ARF2	2.065669	0.002687	3635271
PKC1	2.061892	0.005748	3635210
YRB1	2.060019	0.013152	3639663
MIR1	2.059104	0.004742	3634782
CTA1	2.058841	0.040452	3641631
C5_00580W_A	2.057872	0.043387	3641588
TIF5	2.055814	0.023735	3637765
C3_04360W_A	2.05573	0.044375	3635276
C6_02370C_A	2.053351	0.029536	3645882
C5_03170C_A	2.053347	0.027037	3637801
APA2	2.051093	0.026088	3639234
C4_03110W_A	2.049828	0.007636	3637924
PGA59	2.049397	0.008299	3637881
RPL11	2.046266	0.010059	3643489
C2_07630C_A	2.04624	0.028672	3645193
ACP1	2.045952	0.039356	3639825
SET3	2.0431	0.010859	3643320
NGG1	2.034834	0.014856	3636902

C6_02630C_A	2.034436	0.033024	3639221
RPP2B	2.028187	0.002232	3635195
C2_07810W_A	2.024157	0.046478	3642324
C2_08620W_A	2.022134	0.028149	3643912
CR_03600C_A	2.021621	0.034195	3643207
GDS1	2.018434	0.003066	3642626
KRS1	2.017693	0.005086	3638486
RPG1A	2.013345	0.009494	3646816
C2_01900C_A	2.013175	0.044813	3635789
RCT1	2.007684	0.014397	3641727
C1_01300W_A	2.003378	0.016754	3640185
RPS21	2.003178	0.009064	3640242
GBP2	1.996011	0.003594	3637992
RPL4B	1.992748	0.008865	3643347
UME1	1.992283	0.012309	3643101
CR_10600C_A	1.986144	0.023948	3639004
SYS3	1.977644	0.013216	3645119
C5_04300C_A	1.975855	0.006859	3636456
RPS3	1.971202	0.002633	3647299
VPS11	1.966333	0.028491	3646524
HGT5	1.964575	0.037318	3635201
GYP2	1.958846	0.013272	3641780
HEM13	1.957047	0.005406	3647390
C5_03880C_A	1.955631	0.366429	3647222
DFR1	1.955425	0.369651	3641348
SRB9	1.955146	0.019935	3635845
CCT2	1.953221	0.003356	3643815
MDL1	1.951441	0.039304	3640146
IML2	1.950878	0.018345	3643302
SIT1	1.948023	0.012396	3642307
C2_00570W_A	1.947461	0.022289	3638853
MED16	1.943778	0.039254	3646725
C3_01680C_A	1.94238	0.034668	3636605
PSD2	1.941222	0.040166	3636515
CDC53	1.934129	0.013425	3636603
RBD1	1.929509	0.041099	3642018
C1_00310W_A	1.929492	0.013681	3639298
ARP8	1.926448	0.026241	3640200
MKK2	1.924569	0.017267	3645580
SEC61	1.922134	0.005522	3647462
CR_01420W_A	1.917481	0.016195	3640137
LHP1	1.915913	0.020034	3646665
IRS4	1.909785	0.031026	3645650
PHM5	1.90948	0.023823	3637951
C1_06890C_A	1.909399	0.010487	30515014
C1_11850W_A	1.906676	0.016976	3642010
SPT20	1.904564	0.041544	3642492

GUA1	1.901768	0.00384	3634763
KEL1	1.901251	0.014751	3639318
SIM1	1.899856	0.019313	3636185
TUF1	1.893037	0.006811	3639360
CR_10120C_A	1.890725	0.396635	3638969
C1_02000W_A	1.888591	0.070281	3645418
SAC7	1.887142	0.024044	3638010
GDA1	1.883064	0.058398	3641762
TYS1	1.882378	0.006355	3637870
C1_06720C_A	1.88171	0.118863	3639509
CR_00420W_A	1.880823	0.237445	3639728
C3_06690C_A	1.880646	0.232957	3642256
C1_10920W_A	1.875665	0.084301	3644433
ABP140	1.871743	0.212081	3644592
C3_07820W_A	1.871183	0.223247	3647463
C6_00680C_A	1.870699	0.21641	30515329
DDI1	1.867702	0.03738	3643295
SEC1	1.866896	0.039379	3636935
C5_01200W_A	1.866037	0.236192	3642657
C1_04560W_A	1.862553	0.174362	3646130
DPB2	1.862349	0.323206	3639026
HAT2	1.860395	0.0597	3645973
RPL15A	1.859884	0.017429	3641025
C1_07100C_A	1.859517	0.250852	3639487
C2_10670W_A	1.859255	0.012296	3641251
C1_13030C_A	1.857544	0.030702	3636122
GSG1	1.85713	0.151101	3647609
OCH1	1.855596	0.087235	3641751
C2_04160W_A	1.854347	0.228615	3637331
LRG1	1.853651	0.031067	3639727
RPL14	1.849267	0.015093	30515072
GPI7	1.847638	0.216569	3647672
RPT4	1.8462	0.017977	3640966
C3_01060W_A	1.845912	0.733185	30515174
C5_03910C_A	1.845912	0.733218	3647221
C1_11050W_A	1.845901	0.731259	3644418
C1_13180W_A	1.845901	0.731524	3636162
C2_04430W_A	1.845901	0.731417	3645469
C2_10150W_A	1.845901	0.731435	3643933
C7_04340C_A	1.845901	0.731475	30515361
PGA34	1.845901	0.731376	3642579
TEF4	1.845901	0.731454	3646359
C3_04310C_A	1.84589	0.733001	3635145
C5_03730W_A	1.84589	0.732879	3647238
C6_03600C_A	1.84589	0.733357	3640295
C7_02140W_A	1.84589	0.733178	3636996
CR_03300C_A	1.84589	0.733011	3645098

CR_08260C_A	1.84589	0.73322	3642100
CR_01350C_A	1.845677	0.048616	3640166
MCT1	1.844101	0.150942	3646683
C7_01190W_A	1.841543	0.157656	3645902
RBR1	1.839958	0.741975	3640986
C7_00380W_A	1.839613	0.187155	3638036
ARP4	1.837842	0.058427	3639201
AVT4	1.834958	0.043997	3645316
BIG1	1.834388	0.100249	3644434
HYM1	1.833043	0.088898	3637355
C1_07060C_A	1.832252	0.270632	30515018
ZCF17	1.832091	0.071116	3640205
C2_01040W_A	1.831496	0.536956	30515085
C5_03980W_A	1.830709	0.0293	3647442
C5_02450W_A	1.830391	0.066008	3637729
C1_04640W_A	1.828688	0.06631	3644761
C1_12000C_A	1.828476	0.530668	3642041
ZCF19	1.828331	0.521818	3644869
RPL25	1.82614	0.009903	30515338
C2_08170W_A	1.825938	0.425949	3642318
PXA1	1.825005	0.009583	3639713
ERD1	1.824915	0.347592	3640723
CR_07340C_A	1.824159	0.082155	3646056
FGR50	1.824092	0.174157	3644859
C5_04580C_A	1.823879	0.255868	3636511
C1_04580C_A	1.823484	0.179221	3646132
C4_04500C_A	1.823175	0.076789	3641123
C4_06470W_A	1.822933	0.083426	3642790
CSA1	1.822683	0.048977	3638009
C3_00360W_A	1.821787	0.127679	3640542
CR_06520C_A	1.821438	0.237796	3645263
C4_05630W_A	1.818632	0.038839	3641812
CR_01120C_A	1.818173	0.069308	3640434
RPL32	1.817132	0.015065	30515335
UBC4	1.814011	0.018501	3638927
VTC3	1.813884	0.060918	3643206
RPC31	1.812666	0.106303	3642577
MID1	1.812616	0.096727	3647441
C2_09600C_A	1.806918	0.157359	3643799
C1_09110W_A	1.802857	0.254433	3634940
UGA11	1.80272	0.182635	3637437
CR_09190C_A	1.79693	0.141326	3641885
C7_02510W_A	1.793824	0.153621	30515357
C4_05180C_A	1.793202	0.016553	3647413
C3_01950C_A	1.79306	0.101332	3636599
C7_02480W_A	1.790762	0.159779	3636944
C5_03810C_A	1.789443	0.303478	3647229

GLK4	1.789216	0.01432	3646046
C3_01280W_A	1.786482	0.145292	3636623
C1_03180W_A	1.78344	0.376014	3636806
C3_06710W_A	1.782979	0.028495	3638448
C2_07790C_A	1.782826	0.413373	3642326
MAD2	1.779517	0.3694	3644696
C7_01160C_A	1.779124	0.22207	30515351
C3_00660W_A	1.778301	0.118112	3640538
LPT1	1.776188	0.04198	3645212
C5_00400C_A	1.77555	0.070073	3641550
DBF4	1.774693	0.145103	3641388
SCT2	1.77454	0.066117	3643018
C2_02870W_A	1.774407	0.161334	3642972
C1_02510W_A	1.774328	0.271532	3636796
C6_00430C_A	1.773212	0.080884	3646089
C1_12640W_A	1.772634	0.462006	3646837
CR_07310W_A	1.772361	0.161094	3646058
C4_03710C_A	1.768766	0.194657	3645360
C3_01890C_A	1.766776	0.022861	3636561
C3_02360C_A	1.766054	0.339112	3636617
C5_04470C_A	1.766042	0.04892	3636414
CR_09500C_A	1.765194	0.125765	3641897
C2_04300C_A	1.764662	0.17617	3648276
ULP1	1.762093	0.124984	3638314
C1_03790C_A	1.761409	0.037221	3644682
PEP1	1.761273	0.038107	3647612
C4_01310W_A	1.761139	0.453249	3635604
CR_06790C_A	1.7611	0.251551	3647311
CHK1	1.760901	0.06647	3637394
BAT21	1.759775	0.067765	3637356
VPS4	1.759161	0.063435	3637703
CR_02060W_A	1.757992	0.174704	3640155
C2_04780W_A	1.756471	0.072505	3647781
C2_04490W_A	1.75559	0.102119	3645463
C4_03990C_A	1.752692	0.471661	3648295
FGR17	1.751863	0.267829	3640296
RPL27A	1.748547	0.0196	30515062
HMO1	1.745067	0.048882	3646685
SES1	1.744021	0.023711	3638415
C1_06360W_A	1.743397	0.187111	3639561
MNN9	1.742832	0.10466	3641735
C1_04970W_A	1.741939	0.110439	3647528
CR_05890C_A	1.739904	0.102818	3646682
CR_05120W_A	1.739583	0.074324	3646402
CR_09660W_A	1.739522	0.246143	3647092
CR_04230W_A	1.738333	0.123432	3640944
MSH3	1.73787	0.14286	3643889

CDC23	1.734768	0.128176	3642111
ECM17	1.734433	0.027227	3644077
DAD4	1.733955	0.385724	30515433
SPS20	1.731054	0.12631	3645739
C6_00250W_A	1.730241	0.023438	3646063
VRP1	1.729011	0.066921	3642293
PEX17	1.726953	0.190412	3643886
ERB1	1.725472	0.06969	3644702
C1_05660C_A	1.72537	0.020358	3639900
ASC1	1.725195	0.031702	3645907
OPT3	1.720858	0.131747	3644918
THI6	1.720107	0.221264	3638333
C1_12670C_A	1.719334	0.203943	3646834
C1_11250W_A	1.716249	0.170234	3644556
NUP85	1.715399	0.047429	3635175
MDJ2	1.715303	0.567644	3643437
C2_07700C_A	1.715203	0.569677	3642337
VAN1	1.714938	0.08691	3638527
PGA49	1.714194	0.22597	3646525
PSY2	1.714055	0.223127	3644567
ADE4	1.711603	0.056937	3641769
RPS20	1.711337	0.013978	3642071
C4_05620C_A	1.710217	0.314364	3641783
ZUO1	1.709549	0.038302	3637908
ZCF10	1.706521	0.138511	3643485
RPL23A	1.705382	0.021535	3647558
C4_02100C_A	1.704081	0.302093	3635641
NUP82	1.7038	0.049254	3640261
C4_02780W_A	1.702755	0.061855	3637902
C2_05390C_A	1.701576	0.600001	3643434
CR_05960W_A	1.696964	0.490533	30515417
C4_02950C_A	1.696266	0.480107	3637874
C2_03170W_A	1.696056	0.482434	3642988
C6_03240W_A	1.694266	0.493067	3639181
C1_09000W_A	1.692926	0.208284	3634915
DNA2	1.691265	0.223727	3646072
C5_04960W_A	1.689801	0.055982	3636423
C1_03540C_A	1.688618	0.412106	3636920
CR_08520C_A	1.688519	0.403715	30515443
C2_08690C_A	1.688022	0.371217	3643888
CR_10290C_A	1.686833	0.076881	3638935
HST3	1.686766	0.101255	3642641
C7_03370C_A	1.685859	0.07764	3646720
TIF	1.684875	0.042306	3640190
C3_03060W_A	1.684115	0.364583	3638395
C3_07760C_A	1.681664	0.333139	3638450
MXR1	1.681504	0.070793	3638881

DCW1	1.681445	0.052753	3638842
RPS18	1.679626	0.033291	3637953
LIP5	1.678989	0.352943	3641356
C3_00610W_A	1.678698	0.248338	3640578
C5_00790C_A	1.678023	0.067612	3644125
C3_02290W_A	1.677783	0.265956	3636556
CR_06930W_A	1.676816	0.220248	3648382
C6_04110W_A	1.675819	0.068898	3647834
CR_10170C_A	1.675017	0.085635	3638916
CR_00490W_A	1.67264	0.088983	3639723
C7_04010W_A	1.671772	0.092414	3643112
URE2	1.670773	0.136567	3647795
C2_06320W_A	1.670383	0.085805	3646927
CR_09100C_A	1.669154	0.175017	3641928
YHB5	1.668699	0.107824	3647343
RSR1	1.666943	0.097632	3640145
SAL6	1.66615	0.048572	3640289
RPS23A	1.665292	0.035906	3639515
ALS3	1.665161	0.049025	3647965
ULP3	1.66409	0.164825	3640526
CR_08990C_A	1.662304	0.087635	3641891
C1_09360C_A	1.66132	0.103642	3634807
C1_12700W_A	1.656039	0.319898	3646831
C6_00210W_A	1.655473	0.102156	3646060
NIP1	1.65537	0.073634	3635618
C5_02690W_A	1.652702	0.193643	3637775
CDC47	1.65208	0.167791	3645780
C4_03600C_A	1.651903	0.056964	3645366
AYR2	1.651471	0.09348	3639184
VPH1	1.651298	0.032693	3646157
UBP1	1.65067	0.105258	3641760
C1_02390W_A	1.648057	0.144884	3636791
TCP1	1.646686	0.05341	3646499
CDC54	1.643035	0.436426	3647606
C2_10720C_A	1.642474	0.113469	3641209
C1_08390C_A	1.64122	0.082652	3646504
C1_06390W_A	1.640896	0.197957	3639558
TIP41	1.640855	0.164423	3636506
PRE10	1.639244	0.124653	3636957
CHS5	1.639007	0.031043	3637333
VAC8	1.637846	0.045264	3647410
C3_00830C_A	1.637069	0.216916	3647757
C1_03460C_A	1.634217	0.239312	3636911
NPL3	1.634173	0.053286	3643310
C5_01880C_A	1.633689	0.23851	3643751
C4_02430W_A	1.633468	0.116421	3637848
C1_12740W_A	1.632485	0.177211	3646819

LYS14	1.63206	0.341755	3639217
C7_04060W_A	1.631328	0.512762	3643156
C2_09910C_A	1.631308	0.512185	30515162
CDC60	1.630491	0.044544	3640128
CRK1	1.629901	0.098136	3643414
GGA2	1.627169	0.056686	3636928
C3_01790C_A	1.626633	0.175809	3636587
C5_00950C_A	1.623469	0.110144	3642653
C3_03380W_A	1.623311	0.168714	3638367
C2_02700C_A	1.623154	0.255715	3643005
NUP188	1.617011	0.037434	3634858
GTR1	1.616773	0.30331	3643914
C1_10110W_A	1.616612	0.297865	3634778
IMP1	1.61646	0.300986	3636881
SRP101	1.614665	0.095191	3636513
C1_02040C_A	1.612857	0.041225	3644586
C2_08380C_A	1.609426	0.372951	3648253
NAT5	1.607888	0.210007	3641022
WRS1	1.607306	0.138881	3642060
C5_00850C_A	1.60637	0.633203	3642637
SAP4	1.60637	0.633134	3640257
CDC45	1.606085	0.257113	3648293
RTF1	1.604988	0.135432	3637964
C6_00500C_A	1.604633	0.285332	3647149
C4_05000W_A	1.604479	0.6282	3641149
CR_00870C_A	1.602146	0.620773	3640398
PGA26	1.602146	0.620738	3639890
C2_01570W_A	1.602136	0.619175	3635782
RIA1	1.601774	0.090742	3644478
C3_06540C_A	1.600168	0.468541	3642274
C2_10630W_A	1.599255	0.20134	3641248
C4_05380C_A	1.597645	0.457429	30515276
C3_06680C_A	1.596568	0.381685	3642255
C1_10500W_A	1.596337	0.387962	3647188
BMT8	1.595663	0.198386	3637442
AAT22	1.594315	0.437089	3635651
NIP100	1.591548	0.168487	3640970
PEX4	1.591446	0.153215	3636429
CR_05970C_A	1.591431	0.143233	3642502
C1_12110C_A	1.590873	0.640216	3642054
GUK1	1.590072	0.197488	3647231
C3_03830W_A	1.58892	0.23346	3645630
CR_04720C_A	1.588739	0.044085	3648140
ALG11	1.586015	0.414692	3645871
C3_06800C_A	1.584725	0.271142	3638497
SMD3	1.584364	0.377511	3647060
TLO13	1.584337	0.373957	3647891

C2_08320C_A	1.583395	0.565993	3648167
C6_01300W_A	1.582425	0.275193	3644024
NIT2	1.581212	0.184451	3641874
POB3	1.579803	0.108686	3635827
SHE9	1.579583	0.187431	3642984
CR_08640C_A	1.579508	0.122647	3642112
C1_02740C_A	1.579366	0.428087	3636827
C1_04980C_A	1.5777	0.18034	3647529
CR_02390W_A	1.576119	0.098072	3644930
C1_06190C_A	1.575953	0.145642	3648391
C4_06660W_A	1.575534	0.172846	3642809
C1_01220C_A	1.575511	0.123778	3640210
C7_00790W_A	1.575244	0.180057	3637943
CR_06820W_A	1.575124	0.343023	3647325
C1_00760W_A	1.574256	0.169397	3639351
C3_00640W_A	1.573679	0.151846	3640574
MGM101	1.572769	0.207617	3636820
CR_09640C_A	1.5724	0.246927	30515456
BAT22	1.571898	0.078616	3643663
SSN8	1.570584	0.251291	3641718
C5_03140C_A	1.570573	0.098986	3637798
CR_08930C_A	1.570231	0.131143	3641913
PHO89	1.569918	0.207957	3635633
ADH1	1.567008	0.10051	3636489
DCC1	1.566479	0.553743	3638037
C6_04230W_A	1.566378	0.556359	3646274
C3_06810W_A	1.565351	0.200214	3638545
MYO5	1.565264	0.066019	3647404
SWI4	1.563536	0.250899	3645408
C2_02910W_A	1.56153	0.581497	30515097
EMC9	1.557997	0.05965	3647918
LCB2	1.55641	0.192626	3636181
C2_09830C_A	1.555896	0.289282	30515161
C2_06040C_A	1.555873	0.384505	3644069
C4_04750W_A	1.55512	0.178191	3641133
GLG2	1.554181	0.214031	3642282
MCM1	1.551876	0.150235	3638050
C1_09470C_A	1.551582	0.206762	3634761
C3_01570W_A	1.550132	0.309099	3636668
C3_05150W_A	1.54914	0.127041	3635162
LCB4	1.546795	0.306993	3642057
LKH1	1.546609	0.052594	3647187
C3_06110C_A	1.545378	0.136044	3641761
HYS2	1.544686	0.287909	3636441
RAD14	1.541991	0.23026	3636953
C1_04860W_A	1.541933	0.159767	3644784
CSH3	1.540854	0.13269	3646798

SIN3	1.540743	0.057279	3639282
UBP6	1.540512	0.090198	3639317
FLC3	1.539394	0.158791	3642322
C4_01120C_A	1.539266	0.338874	3635683
C7_00490C_A	1.538665	0.185197	3637986
C5_03830C_A	1.537989	0.172685	3647227
CR_04450C_A	1.536928	0.116452	3640987
SGT1	1.536875	0.096844	3644068
RHR2	1.536402	0.125577	3640522
SSC1	1.53318	0.089777	3645232
C2_09070C_A	1.530685	0.131041	3644111
SEC5	1.528282	0.213604	3648178
CDC15	1.528005	0.161031	3643459
MCA1	1.525871	0.118378	3635233
CR_03860C_A	1.524352	0.214867	3643186
C4_00170W_A	1.521301	0.136363	3645028
SBP1	1.521277	0.096319	3635280
YPT1	1.520156	0.08925	3636915
C5_04360C_A	1.520102	0.192501	3636483
AGP2	1.518567	0.129773	3635684
C2_08340C_A	1.517565	0.457213	3648165
C7_00920C_A	1.515673	0.297643	3637957
C4_04870C_A	1.513646	0.091551	3641160
C4_05610C_A	1.511242	0.10766	3641782
CR_01560W_A	1.511149	0.438636	3640075
C5_01190W_A	1.509813	0.192661	3642659
TEA1	1.509025	0.162143	3643671
MEX67	1.507748	0.194402	3641020
AMN1	1.507164	0.467826	3635795
C1_08180C_A	1.506743	0.105915	3640743
RAM2	1.506308	0.253856	3634767
C5_01110W_A	1.506055	0.171678	3642623
CST20	1.505842	0.187344	3637790
GLN1	1.505762	0.167018	3646386
C2_01960C_A	1.505347	0.691123	3635796
C6_02810C_A	1.503873	0.198686	3639248
FCY21	1.503572	0.106288	3643822
C1_04150C_A	1.502404	0.595892	3644704
C6_01200W_A	1.501747	0.593131	3644041
C1_12060C_A	1.501659	0.595753	3642026
TIM13	1.501622	0.587322	3637868
C7_00850W_A	1.501621	0.315781	3638056
C1_07850C_A	1.501525	0.588477	3640739
C2_10730W_A	1.501457	0.597363	3641210
CR_01050C_A	1.499497	0.250237	3640384
ROD1	1.497887	0.193945	3635763
C7_01650W_A	1.497044	0.113846	3636984

RPL12	1.496609	0.108897	3636659
RPS21B	1.496524	0.074511	30514961
ENT3	1.494424	0.092155	3635813
CR_07850W_A	1.494306	0.167191	3647347
C1_00470C_A	1.492093	0.223924	3639315
TAF60	1.490532	0.176333	3642252
C2_08720W_A	1.490436	0.243704	3643885
C4_06360C_A	1.48897	0.678895	3642825
C6_00180C_A	1.488952	0.686087	3646081
C4_04950W_A	1.488873	0.678302	3641168
SUP35	1.488742	0.109327	3643785
RUB1	1.488493	0.430585	30515194
MED14	1.488479	0.220024	3637800
MNR2	1.486789	0.087338	3645017
C1_09910C_A	1.486379	0.475289	3634798
AGE1	1.486038	0.181428	3636913
DAL4	1.48388	0.483158	3638422
PEP8	1.482477	0.176514	3645624
PEX2	1.480508	0.248089	3643460
AGE2	1.479937	0.194969	3643810
C1_03630W_A	1.477118	0.239223	3636882
CR_09530C_A	1.476881	0.231191	3647101
PDK2	1.476768	0.136588	3641876
FOL1	1.474054	0.235836	3644123
C5_02350C_A	1.473946	0.510858	3637791
C6_02740W_A	1.473877	0.284674	3639216
POM152	1.472714	0.205106	3638812
MGE1	1.472468	0.134834	3640133
FDH1	1.472465	0.43855	3646398
C4_02340W_A	1.472062	0.18685	3637883
CR_01360W_A	1.471691	0.435713	3640167
C7_00310C_A	1.471011	0.348346	3638045
HOS2	1.469884	0.263544	3640509
SKI8	1.469326	0.215543	3640294
CR_07160C_A	1.46889	0.196447	3648347
C1_14030W_A	1.468242	0.272322	3643340
VPS16	1.467073	0.243217	3646150
RPN3	1.466445	0.137896	3636917
TUP1	1.465855	0.128164	3639369
C1_14090W_A	1.464698	0.262142	30515079
RPS22A	1.464675	0.129555	3639551
TIM8	1.464447	0.449509	3647469
ATM1	1.46442	0.258722	3646284
CZF1	1.464379	0.154584	3642915
WSC4	1.464289	0.154511	3643282
C5_03670C_A	1.463792	0.347187	3646987
C7_03010W_A	1.463276	0.187021	3641382

ZRT2	1.462989	0.391116	3635861
KRE9	1.461857	0.181705	3635183
SMI1B	1.461255	0.549787	3643675
C1_13690C_A	1.460629	0.265806	30515076
C3_01220W_A	1.459241	0.374709	3636610
RPB8	1.459042	0.314447	3647301
PCT1	1.458454	0.16798	3645743
VPS33	1.456704	0.277885	3647477
C4_01950W_A	1.456229	0.21651	3635665
C1_10620W_A	1.455529	0.109307	3647682
CCT6	1.454782	0.195353	3642914
C1_12430W_A	1.453859	0.259944	3642013
CR_09490W_A	1.452878	0.178916	3641896
GAP6	1.451616	0.168881	3646968
C1_11090C_A	1.451174	0.215793	3644415
C2_05050C_A	1.451166	0.452558	3643450
C7_00210C_A	1.450997	0.282239	3637983
SIW14	1.450996	0.455324	3647323
C7_01350C_A	1.449349	0.378573	3645899
RVS162	1.449185	0.290853	3646820
C1_11010C_A	1.448959	0.326968	3644421
C2_00390C_A	1.4484	0.217321	3638822
GSH2	1.445394	0.223344	3642083
SPO75	1.441922	0.297523	3645364
C2_01720C_A	1.441489	0.361225	3635731
EHD3	1.441406	0.315748	3636858
C4_03160C_A	1.440692	0.472882	3637841
C1_12070C_A	1.440565	0.473471	3642025
C5_00230C_A	1.436801	0.630705	3641564
C2_02500W_A	1.434965	0.243076	3635816
ACF2	1.434833	0.276636	3641613
C6_00800C_A	1.432611	0.14486	3647069
CR_00830W_A	1.43222	0.506418	3640412
C3_02970C_A	1.431403	0.307636	3638385
CDC48	1.431138	0.178797	3644440
PUP1	1.429633	0.158266	3638936
CR_05460W_A	1.427622	0.304502	3648237
C1_06560W_A	1.426847	0.30019	3639517
RCE1	1.426666	0.384276	3641104
CR_05700C_A	1.426633	0.310449	3646701
C4_03960W_A	1.426488	0.254036	30515264
FAD2	1.424442	0.117071	3644053
C2_03130W_A	1.424427	0.358178	3642993
TRM1	1.424292	0.333404	3640396
PUP3	1.423263	0.249034	3646718
C6_02430W_A	1.422057	0.264547	3639163
IFF5	1.421675	0.219184	3642782

C2_04330C_A	1.421499	0.370931	3645453
PTC4	1.420902	0.235042	3646697
NGS1	1.419903	0.180866	3639703
C3_07630C_A	1.419499	0.262778	3638491
C2_00110W_A	1.418124	0.408745	3647921
C1_03920C_A	1.416764	0.495061	3646413
INO4	1.415985	0.415951	30515105
C6_00260W_A	1.415277	0.190794	3646065
C7_03990C_A	1.413964	0.322586	3643115
C4_03150W_A	1.413479	0.251134	3637842
FAA4	1.413237	0.186495	3638973
C6_02640C_A	1.413163	0.328818	3639222
RRD1	1.412284	0.411733	3638445
C3_01120W_A	1.411962	0.40365	3639836
C6_03310W_A	1.411912	0.40404	3639197
CR_02610C_A	1.41122	0.218058	3642566
SPE2	1.409742	0.263126	3644116
RPC19	1.40951	0.427632	3647693
C4_05010W_A	1.408837	0.229175	3641148
C2_00920W_A	1.408105	0.379528	3638893
VPS2	1.408042	0.349323	3641546
C3_05110W_A	1.407882	0.377992	3635158
RPS24	1.407589	0.135759	3640568
C1_14020W_A	1.404744	0.853792	3643339
C4_00250W_A	1.404744	0.853903	3645035
RHO2	1.404621	0.558016	30515143
C1_08110W_A	1.404305	0.220556	3640716
C1_04230W_A	1.403504	0.258774	3644722
C3_02070C_A	1.401957	0.851269	3636576
C3_05640W_A	1.401957	0.851177	3643654
C1_06220C_A	1.401948	0.85118	3647518
C2_06140C_A	1.401948	0.85118	3644082
C6_01130W_A	1.401948	0.85118	3644055
C6_04070C_A	1.401948	0.851272	3647826
C7_02360W_A	1.401948	0.851272	3636931
PGA28	1.401948	0.851272	3641350
PGA61	1.401948	0.851272	3640292
SAP5	1.401948	0.85118	3639268
ORC4	1.401691	0.286042	3637753
C7_00580C_A	1.400846	0.489584	3637995
PEX22	1.400544	0.408511	3648103
TRP5	1.400455	0.196534	3648087
C3_03680W_A	1.400304	0.235036	3645648
AXL2	1.399635	0.345558	3641184
C1_12090C_A	1.399435	0.85475	3642056
C5_04690C_A	1.399435	0.855297	30515320
GTT1	1.399435	0.85507	3643653

RNY11	1.399435	0.855395	3636408
RDH54	1.399244	0.336978	3641231
C5_00560W_A	1.397954	0.260626	3648267
C1_13190W_A	1.397522	0.203396	3636163
C5_05260W_A	1.397512	0.516299	3636473
RFX2	1.397468	0.578277	3635658
CR_03130W_A	1.397381	0.542427	3645121
SSZ1	1.397026	0.156751	3641139
C2_04360W_A	1.394821	0.37933	3645461
SYN8	1.393696	0.501364	3645120
C3_02850C_A	1.392818	0.517836	3638332
LEA1	1.392639	0.507972	3641807
CR_02120C_A	1.392623	0.509207	3640143
TIM44	1.390689	0.20398	3640529
KIN2	1.389474	0.193705	3639697
SMT3	1.388311	0.215223	3644550
IST1	1.388211	0.511449	3640587
C1_04270C_A	1.387825	0.757653	3644708
MRS7	1.385555	0.253537	3640187
C7_02220C_A	1.383282	0.295127	3636989
C5_03100C_A	1.382526	0.32579	3637704
C5_00150C_A	1.382209	0.189666	3647872
C1_12920C_A	1.381324	0.411498	3636155
CR_06780W_A	1.381254	0.255869	3647312
CTF18	1.379287	0.422008	3640431
VPS15	1.378728	0.312824	3644040
C5_02390C_A	1.378408	0.293883	3637795
C2_04450W_A	1.377123	0.747067	3645467
RPL10	1.376543	0.203291	3636850
NCB2	1.376429	0.345184	3643028
VPS41	1.376117	0.346482	3634800
CR_03440W_A	1.375804	0.741154	3645112
STN1	1.375804	0.739635	3643901
C4_05730W_A	1.375723	0.739879	3641805
C2_08750W_A	1.375707	0.743924	3643881
C6_01950C_A	1.375483	0.336546	3644558
C2_02620W_A	1.374733	0.338963	3635838
PEX19	1.372915	0.213719	3642108
CCT7	1.372515	0.229664	3643734
IPK1	1.371256	0.375682	3648091
C3_07850W_A	1.370635	0.314827	3647466
IFI3	1.370414	0.631299	3644694
CR_03700C_A	1.370281	0.235319	3643223
C1_09940W_A	1.369087	0.5445	30515037
YAE1	1.368406	0.495066	3637941
ALG7	1.368033	0.428114	3642291
C4_01650C_A	1.367654	0.370146	3635713

C5_01000C_A	1.36753	0.300128	3642651
RTT101	1.367364	0.346855	3639827
MSO1	1.366681	0.384546	3642040
CR_06260W_A	1.363358	0.456128	3644853
MET18	1.362714	0.491589	3636631
C2_10230W_A	1.361931	0.477359	3643925
C1_13720W_A	1.36192	0.344958	3636134
NTF2	1.35967	0.349765	30515039
C1_07360W_A	1.356724	0.301718	3644470
NTH1	1.356622	0.178544	3639665
MNN12	1.355732	0.336198	3634904
PPZ1	1.355266	0.346923	3645265
C7_03610C_A	1.354432	0.326037	3646241
C7_01270C_A	1.354407	0.513962	3645908
C6_02680W_A	1.354384	0.444913	3639226
C5_01140C_A	1.354236	0.288639	3642663
C1_11730W_A	1.353326	0.323202	3645318
PRE1	1.353002	0.307958	3636500
PHO91	1.35288	0.23285	3644588
C4_06690C_A	1.352873	0.302674	30515285
C5_05150C_A	1.352003	0.464702	3636461
C5_01090C_A	1.351135	0.341408	3642625
VTI1	1.350299	0.342343	3638352
SWD3	1.349709	0.588707	3645890
C3_03430C_A	1.347772	0.542647	3638355
C1_06710W_A	1.347593	0.387962	3639510
C1_06590C_A	1.347365	0.330119	3639514
CR_08350W_A	1.347279	0.260149	3642082
CR_05980W_A	1.347187	0.203244	3642501
MEC1	1.346958	0.395865	3647733
TIM10	1.346465	0.406416	30515247
LYS142	1.344974	0.361715	3634896
C6_03420W_A	1.344629	0.558393	3639235
C7_00190W_A	1.344454	0.444769	3637969
C4_03040W_A	1.343018	0.446728	30515253
C1_01400C_A	1.34249	0.322877	3640237
NOP1	1.342476	0.265325	3642947
C1_05630C_A	1.342171	0.191332	3639840
RPN5	1.341602	0.202046	3636405
RAD50	1.341371	0.310268	3636594
C5_03690W_A	1.341201	0.587518	3646989
C3_00730W_A	1.34109	0.380496	3640530
SNF4	1.340031	0.256976	3640264
LAC1	1.339145	0.606409	3641717
TFA1	1.338329	0.323571	3634850
ROT2	1.337302	0.368815	3641566
C3_03150W_A	1.335822	0.314032	3638423

PRE6	1.334991	0.3732	30515395
C5_04920C_A	1.334974	0.27976	3636419
C1_11720W_A	1.334873	0.624529	3645319
C4_06670W_A	1.334793	0.625096	3642951
C5_03530C_A	1.334777	0.62132	3646971
C3_06830C_A	1.334616	0.357102	3638543
DIT2	1.334612	0.617196	3640962
C4_01660W_A	1.334493	0.277727	3635712
C2_01340W_A	1.334339	0.279186	3638844
PHO88	1.333595	0.424362	3641898
GCR3	1.332067	0.298613	3646485
C2_05520W_A	1.331703	0.544629	3643421
RPL21A	1.331462	0.237533	3637400
CR_03540W_A	1.331021	0.455188	3643215
PHO81	1.330705	0.397501	3639662
RIB3	1.329562	0.317643	3642062
MDG1	1.329158	0.275772	3643311
RPS25B	1.328714	0.226166	3646972
TDH3	1.328256	0.29758	3638540
C3_03820C_A	1.32823	0.414832	3645631
PR26	1.327575	0.230098	3642981
C2_05040C_A	1.325628	0.328465	3643449
YKT6	1.325223	0.373454	3636863
C1_09620C_A	1.322289	0.509682	3634892
C1_09570W_A	1.321136	0.406638	3634887
AVT7	1.320284	0.34248	3637433
C1_12470W_A	1.319592	0.730848	3648182
MSS4	1.318105	0.266084	3647957
TPK2	1.317052	0.272179	3643482
C1_01530C_A	1.317029	0.257796	3640232
C1_08860C_A	1.316231	0.423196	3634921
GCN2	1.31592	0.421967	3645897
HST1	1.315245	0.413432	3634770
ZCF6	1.314383	0.489266	3635837
C2_10300C_A	1.313987	0.570043	3643955
C2_00290W_A	1.313566	0.566071	3638801
C4_00420C_A	1.313479	0.304886	3645021
C2_05580W_A	1.312232	0.347911	3645563
BEM2	1.311463	0.345107	3637044
PHO85	1.311193	0.316501	3646148
C2_00320W_A	1.310678	0.488575	3638798
PRE8	1.310303	0.36452	3641906
C1_07090C_A	1.309384	0.357676	3639488
C2_07290W_A	1.309073	0.316637	3643476
C1_10240C_A	1.308642	0.496051	3634881
CR_09840C_A	1.307175	0.71872	3638988
C2_02000W_A	1.306561	0.304394	3635767

CCT3	1.305131	0.355631	3636458
C1_08160W_A	1.305055	0.381719	3640733
TIF34	1.303537	0.245656	3636872
VPS53	1.30257	0.356745	3639319
TRP2	1.301666	0.404084	3640079
C1_03910C_A	1.301217	0.513189	3646412
MED5	1.300308	0.428987	3648211
PAP1	1.299731	0.388034	3643763
CR_06850C_A	1.298636	0.719427	30515424
PTH2	1.298245	0.445068	3637709
C4_04130W_A	1.297343	0.443586	3641221
RAD23	1.297239	0.325967	3635834
BMT7	1.297016	0.611792	3638357
ECM29	1.296235	0.353556	3638433
CR_08430W_A	1.292504	0.329837	3642120
GIS2	1.290867	0.317069	3643744
C2_04320W_A	1.287474	0.526249	3648274
C4_06740C_A	1.287176	0.408825	3642923
REX2	1.286781	0.537856	3635783
ERF1	1.285624	0.360792	3643455
C1_12120W_A	1.285541	0.600008	3642053
ARV1	1.284493	0.715978	3635248
RPS27	1.284136	0.386909	30515429
DFG10	1.283788	0.636441	3645786
CR_06690C_A	1.282652	0.471115	3646624
PRE9	1.282526	0.405545	3638311
RPC53	1.282297	0.510747	3637898
MET8	1.281767	0.514483	3638533
C4_06730C_A	1.280952	0.302792	3642924
C3_02490C_A	1.280387	0.304101	3638351
INO2	1.279788	0.534362	3639674
C2_04180C_A	1.279729	0.538606	3637330
CR_09830W_A	1.279616	0.654327	3638986
C2_08990C_A	1.279518	0.462239	3645783
C7_03460W_A	1.27942	0.715371	3646712
CR_08300C_A	1.278458	0.339667	3642076
C1_02160W_A	1.277015	0.614142	3644584
C2_10120W_A	1.275872	0.550629	3643936
ALT1	1.275674	0.453725	3638307
KIP4	1.27477	0.47783	3642032
URA1	1.274539	0.264843	3634819
CR_03690W_A	1.274236	0.442108	3643222
C2_05530C_A	1.273948	0.357756	3643422
C1_06420C_A	1.273567	0.514916	3639554
SNG4	1.273499	0.488082	3646714
C1_08520C_A	1.272927	0.585006	3646495
AXL1	1.27211	0.446041	3641938

C4_04240C_A	1.271565	0.436956	3646856
PIN4	1.271149	0.436004	3640101
HIR1	1.270531	0.351039	3638796
C1_00910W_A	1.270467	0.68845	3639284
MNT2	1.268811	0.497349	3636583
TSC2	1.267222	0.474743	3646953
STB5	1.26605	0.499051	3640208
C4_02670W_A	1.265128	0.543311	3637830
C6_00690W_A	1.265116	0.559593	3647075
MNN13	1.264996	0.626993	3637720
GPD2	1.264899	0.420014	3644563
DAD2	1.26466	0.681333	3643465
RPL29	1.26401	0.378084	30515048
C1_14560C_A	1.263222	0.474906	3648376
C4_03300C_A	1.262654	0.807825	30515254
THI4	1.262557	0.809967	3635160
PEX14	1.262274	0.379537	3648208
C6_02580W_A	1.261768	0.538062	3639207
PIN3	1.260916	0.411771	3635228
C7_04240C_A	1.258785	0.357154	3643165
TIM54	1.258613	0.458667	3641349
C3_02770C_A	1.257414	0.453872	3638414
CR_04300W_A	1.253874	0.311804	3640995
IFU5	1.253849	0.418952	3640116
LAP4	1.251924	0.460937	3646979
CR_04390C_A	1.25131	0.443861	3640980
PCD1	1.251124	0.417239	3647087
C3_01800C_A	1.25039	0.495951	3636586
APE2	1.250006	0.356137	3647990
CRM1	1.249744	0.371645	3639721
TES15	1.249135	0.591172	3647478
C3_06550C_A	1.248707	0.533112	3642275
RIM11	1.245598	0.399145	3648277
HHT1	1.245499	0.369545	3638444
FUR1	1.245228	0.495019	3646348
CDC43	1.245184	0.655943	3648206
PHR2	1.244012	0.391746	3639334
KRE30	1.242085	0.376668	3642311
C2_09480W_A	1.241955	0.777239	3643819
C2_06850W_A	1.240677	0.657206	30515142
SET1	1.239557	0.470628	3639280
MNN15	1.235698	0.617271	3644794
RPL30	1.235527	0.340262	30515273
C2_10650W_A	1.234591	0.759211	30515168
C2_10060C_A	1.234109	0.750813	3643948
C2_10330C_A	1.234022	0.752384	3643943
C3_06150W_A	1.233415	0.472942	3641765

C1_02800W_A	1.233387	0.732793	3636873
C7_01600W_A	1.232546	0.52752	3637036
C3_01690W_A	1.232354	0.508503	3636604
C6_04190C_A	1.231435	0.791032	3646282
C2_10870W_A	1.230683	0.529028	3641234
C6_01450C_A	1.228518	0.492037	3641624
C1_14040W_A	1.227361	0.536037	3643342
C2_00350W_A	1.226434	0.551898	3638827
ZCF16	1.225676	0.534097	3647395
C6_02310W_A	1.225581	0.525629	3645875
C2_07200W_A	1.225123	0.680976	3643481
C3_02580C_A	1.225108	0.679619	3638376
BUD23	1.22437	0.614283	3642629
C5_00550C_A	1.224322	0.686822	3641535
C4_02150C_A	1.223664	0.674385	3635670
C3_02760C_A	1.222983	0.447853	3638413
PRR2	1.222725	0.695685	3646723
CR_04830C_A	1.222692	0.66912	3647297
C1_14200W_A	1.22262	0.529521	3643301
C3_00310C_A	1.221076	0.615732	3640523
CR_09040W_A	1.219782	0.593154	3641894
C1_08690W_A	1.219731	0.589441	3634934
C1_02720W_A	1.21878	0.541819	3636825
FLO9	1.218502	0.559724	3640581
ZCF11	1.218074	0.543899	3645109
C1_14520W_A	1.218021	0.426491	3643332
C3_05090C_A	1.217698	0.531114	3635245
SMF12	1.216974	0.504546	3643509
RPS9B	1.216851	0.437557	30515104
RPL7	1.215871	0.509956	6335362
C1_03370W_A	1.214902	0.452574	3636891
RBT5	1.211776	0.508938	3645007
EMP46	1.209183	0.514533	3645270
TRY5	1.208543	0.643054	3641619
COI1	1.207094	0.563488	3640721
RPS28B	1.205657	0.470805	30515349
MRS2	1.205583	0.673789	3640110
C4_06170C_A	1.204609	0.559171	3642818
GAL7	1.202557	0.508075	3644591
C1_04910C_A	1.202461	0.536891	3644793
SNU114	1.20029	0.526138	3647798
C2_08630C_A	1.200012	0.508779	3643910
SLP2	1.199304	0.622798	3644091
SPP1	1.198007	0.640809	3636897
SYS1	1.197913	0.665302	3643138
C5_03870C_A	1.197305	0.626952	3647223
EDC3	1.196603	0.63631	3646152

C5_02520W_A	1.196554	0.763188	3637768
COQ4	1.196531	0.575802	3636804
C2_08830W_A	1.196414	0.548924	3643894
C7_04110W_A	1.196385	0.576171	3643133
C1_10630C_A	1.195059	0.762889	3647683
SPT3	1.194713	0.578909	3638977
C1_01860W_A	1.194664	0.537081	3645430
C2_05760C_A	1.194322	0.587143	3645578
C2_04870C_A	1.194268	0.429379	3648111
C6_02800W_A	1.194161	0.602723	3639246
C1_08340C_A	1.19413	0.662065	3640752
C2_05080C_A	1.191917	0.661328	3643453
TRP1	1.191615	0.600677	3639321
C2_04130W_A	1.189793	0.552977	3637334
C6_00230W_A	1.189183	0.706521	3646061
BIO32	1.188663	0.797028	3643430
C2_02150C_A	1.188496	0.537062	3635749
CR_10500C_A	1.187593	0.652674	3638982
BZZ1	1.187563	0.516985	3636572
FUN12	1.187485	0.489348	3640713
C6_01960W_A	1.187469	0.801026	3644559
C1_13810W_A	1.186514	0.63642	3636126
C6_04260C_A	1.186287	0.720949	3646277
C4_02660W_A	1.18617	0.830029	3637831
PRE3	1.184759	0.531314	3643660
TRR1	1.184481	0.520109	3637778
CR_07870W_A	1.183045	0.737456	3644146
C4_05820W_A	1.182009	0.736258	30515277
C4_01790W_A	1.181432	0.610529	3635597
C1_03960C_A	1.179418	0.656177	3646418
PRE5	1.179319	0.573659	3643111
C1_10190W_A	1.178751	0.553445	3634785
NUP84	1.178414	0.582497	3645351
SIT4	1.177974	0.58679	3647993
C4_06290W_A	1.177488	0.825249	30515283
C4_00290C_A	1.177405	0.825054	3644994
C1_05480C_A	1.177391	0.828767	3642486
CR_06100C_A	1.177315	0.830416	3644842
STT4	1.175832	0.520527	3647963
C1_04620W_A	1.175389	0.616951	3646135
MIA40	1.175261	0.560522	3636866
MNN22	1.174673	0.580857	3641117
PGK1	1.174133	0.561394	3647081
C4_03080W_A	1.172126	0.554229	3637928
C1_05750C_A	1.172125	0.573228	3639891
RVS167	1.170512	0.527807	3647831
TEP1	1.170115	0.772544	3647653

C4_00700C_A	1.169557	0.677152	3645751
CR_00470W_A	1.167799	0.545619	3639725
C2_04790C_A	1.167767	0.618303	3647779
C3_07770C_A	1.166913	0.696632	3647457
C1_07080W_A	1.166719	0.656382	3639489
CR_09110C_A	1.166305	0.646346	3641929
ISN1	1.166137	0.601504	3640196
RNR21	1.164529	0.621855	3643021
SET6	1.164451	0.679694	3644590
RTA3	1.163453	0.593735	3646566
C5_05070W_A	1.162822	0.681022	3636491
SEN1	1.162801	0.615552	3635255
C3_07280C_A	1.162754	0.65451	3638431
BFA1	1.162701	0.628664	3639301
CR_05100W_A	1.161897	0.676103	3646403
C3_03260W_A	1.161403	0.562575	3638320
C6_00340C_A	1.160489	0.671959	3646074
C3_02160C_A	1.158925	0.553057	3636654
TIM22	1.158828	0.63346	3648168
TRM9	1.158601	0.655036	3642074
POS5	1.158352	0.608606	3638519
COX13	1.157223	0.621867	3635784
C5_04620C_A	1.156748	0.7499	3636394
C4_06800W_A	1.156529	0.568212	3642917
CLA4	1.155302	0.669711	3634878
MLT1	1.154615	0.535575	3640748
RPN10	1.154062	0.609348	3644080
C7_01180W_A	1.15389	0.652078	3645915
HAL21	1.153835	0.603121	3644045
SDS22	1.152338	0.598705	3637362
C5_00600C_A	1.151998	0.888993	3641586
C2_05570C_A	1.151543	0.707376	3645562
C1_11930W_A	1.15142	0.580023	3642002
C4_00860C_A	1.149983	0.67473	3635717
PGA42	1.149421	0.886646	3642801
C6_04080W_A	1.148058	0.592088	3647837
C4_01720C_A	1.147979	0.559179	3635589
CR_02430C_A	1.146882	0.523707	3644924
C6_03150C_A	1.146698	0.675163	3639257
C4_00050W_A	1.146518	0.622581	3648060
C6_03730C_A	1.145866	0.675038	3640303
CUE5	1.145502	0.557316	3642467
RAD9	1.144314	0.689731	3637780
CDS1	1.144082	0.588437	3647729
C1_12350W_A	1.143763	0.656823	3642063
C1_13750C_A	1.143306	0.841105	3636137
C2_08910C_A	1.143063	0.606472	3645798

C1_07210C_A	1.142738	0.638599	3644473
C2_04960C_A	1.140529	0.773735	3643442
MRR2	1.139212	0.656664	3647468
CEF3	1.132895	0.6477	3647040
RIM13	1.132625	0.702201	3636487
UGP1	1.132538	0.578566	3648144
CDC13	1.13218	0.795486	3639293
CR_06640C_A	1.131425	0.721816	3646619
CR_06650C_A	1.130652	0.759479	3646620
CR_01780W_A	1.129537	0.754126	3640157
C3_02390W_A	1.129378	0.808576	3636615
C1_06640C_A	1.128993	0.813415	30515010
C7_04260W_A	1.128888	0.640652	3643160
C3_05120C_A	1.128568	0.718493	3635159
C2_07360W_A	1.128258	0.756731	3645236
CR_01020C_A	1.126981	0.760609	3640420
HXK1	1.126961	0.665337	3645964
C1_02200C_A	1.126949	0.754977	3644593
TAF4	1.126728	0.663387	3645198
CR_07200W_A	1.126494	0.727598	3646044
C5_02040W_A	1.125759	0.680667	3643769
CR_03760W_A	1.125301	0.723602	3643178
C2_00750W_A	1.124729	0.838156	3638911
SLF1	1.124252	0.747085	3638500
C5_04890C_A	1.123545	0.807073	3636415
NPL6	1.122908	0.667274	3638883
C2_03490C_A	1.12057	0.729342	3637346
CR_04460C_A	1.119723	0.824306	3640988
C2_07220W_A	1.119175	0.676776	3643483
CRG1	1.118019	0.703617	3646393
HGC1	1.117207	0.769091	3639349
MRR1	1.116285	0.739623	3641741
RPT5	1.115269	0.65641	3642911
C3_02930W_A	1.114575	0.889949	3638380
ERG1	1.113978	0.645026	3646509
CR_03400W_A	1.11393	0.763605	3645137
MODF	1.113396	0.752419	3636182
RPL9B	1.113375	0.669015	3638348
C4_06410W_A	1.112985	0.76047	3642796
STE2	1.112579	0.886787	3646630
C4_00500W_A	1.112073	0.716405	3648379
C7_00010C_A	1.111929	0.736485	3638006
RPO26	1.110924	0.710835	3646351
CR_10830C_A	1.110206	0.780562	3638960
PRP3	1.109858	0.78598	3637406
C1_04530C_A	1.109195	0.759037	3646149
C1_01070C_A	1.10886	0.786098	3640172

C1_09440W_A	1.108488	0.763101	3634857
C5_05270C_A	1.107964	0.750398	3636496
C4_03560W_A	1.107703	0.713744	3645373
C2_09280C_A	1.106875	0.755072	3644102
C1_11560C_A	1.106775	0.820612	3645331
GLY1	1.106288	0.682316	3647181
VID21	1.105159	0.699466	3642946
RAD59	1.102178	0.849444	3648281
ARG3	1.10212	0.875111	3639180
C1_12810W_A	1.10027	0.809222	3646827
C5_00380W_A	1.10022	0.801048	3641553
C3_05430W_A	1.099672	0.77013	3643667
LYS143	1.099426	0.846508	3634894
DRG1	1.09867	0.750628	3640714
HIS4	1.09817	0.703508	3645025
C5_02500C_A	1.097637	0.765165	3637766
C7_02690C_A	1.096394	0.800073	3647649
DTD2	1.095953	0.784317	3638390
FKH2	1.095461	0.743556	3640536
C1_01940C_A	1.09531	0.84514	3645422
RPL82	1.094993	0.760598	3644419
C4_04250W_A	1.094442	0.851659	3646855
C5_02560C_A	1.094157	0.733334	3637719
CR_06030C_A	1.093273	0.803199	3644874
C6_04370W_A	1.092343	0.807371	3646294
CR_03240C_A	1.091721	0.82679	3645126
C1_10350C_A	1.091534	0.780773	3634835
MRS4	1.091016	0.810934	3642306
AGC1	1.090587	0.744981	3636219
C4_02860W_A	1.090557	0.750373	3637909
FGR39	1.09039	0.884972	3644529
YOR1	1.090133	0.749363	3643964
RPC40	1.090082	0.755038	3643427
C1_06400C_A	1.089968	0.738372	3639557
C1_05010C_A	1.089698	0.803326	3647532
C1_02750C_A	1.08925	0.735453	3636828
C5_00050W_A	1.089033	0.84239	3647875
C3_02090C_A	1.088854	0.819522	3636661
GAR1	1.088233	0.74879	3645332
ZDS1	1.08803	0.741387	3641103
CR_06960W_A	1.087607	0.792247	3645737
C1_01390C_A	1.085223	0.793546	3640236
FGR14	1.085007	0.7567	3648083
C1_06530C_A	1.084455	0.750936	3639521
MDL2	1.084027	0.78581	3639256
MSU1	1.08047	0.867234	3643919
C1_05610W_A	1.080427	0.833629	3639843

CR_01550C_A	1.080006	0.851127	3640074
C7_00070C_A	1.079821	0.76863	3638011
PRP13	1.079314	0.766563	3643194
C3_05380W_A	1.079266	0.842715	3643679
ZCF15	1.079079	0.791407	3637867
YTM1	1.078785	0.784824	3634765
C1_10410W_A	1.078635	0.879071	3634840
BCK1	1.078366	0.788617	3641384
YPT52	1.074995	0.808863	3643346
PRT1	1.074915	0.793437	3636959
TRA1	1.074757	0.808081	3641644
EAF7	1.074363	0.803185	3640973
C2_08900W_A	1.072914	0.866968	30515156
C3_03590W_A	1.072615	0.811269	3638302
GLE2	1.072228	0.79855	3646293
MET10	1.070622	0.763405	3644088
C1_11140W_A	1.070537	0.852333	3644410
UGA33	1.070145	0.844792	3641886
RPL42	1.070091	0.783921	30515041
C2_01920C_A	1.069181	0.811819	3635792
HOS1	1.067666	0.881288	3646518
IDI1	1.067436	0.824522	3637894
GPH1	1.067323	0.804595	3637956
QCR9	1.066664	0.85216	30515251
CR_06250W_A	1.066512	0.893074	3644852
GCN5	1.065193	0.823449	3646626
C1_03260W_A	1.064146	0.871554	3636898
C7_01960W_A	1.063309	0.839697	3636969
NOT4	1.062841	0.814465	3645115
PUS4	1.062375	0.828595	3642661
C1_04790W_A	1.062181	0.857848	3644772
EXO84	1.061847	0.846486	3644022
MIM1	1.0613	0.857031	30515394
C2_07920W_A	1.060865	0.873404	3642294
C1_12250C_A	1.060767	0.81814	3642022
HPA2	1.060743	0.86094	3647907
C1_02410C_A	1.060611	0.882403	3636793
SKI2	1.059856	0.831491	3642143
CR_10260W_A	1.059069	0.904044	3638932
C1_13370W_A	1.058598	0.884978	3636216
POX1	1.057339	0.838603	3640247
C2_03800C_A	1.056618	0.831535	3637401
C1_04470C_A	1.056457	0.919669	3646142
SSK2	1.055969	0.83033	3641144
C6_01520W_A	1.055807	0.928843	3641617
C6_02540C_A	1.054791	0.926424	3639176
C7_04120W_A	1.054779	0.926023	3643132

C3_07470W_A	1.05446	0.939214	3638479
C6_01850W_A	1.052995	0.852037	3641629
YMX6	1.05221	0.887903	3640255
SKO1	1.052035	0.843887	3644688
OFR1	1.051369	0.883105	3640710
EHT1	1.051001	0.864559	3636894
BEM3	1.050966	0.862029	3637885
C2_08390W_A	1.050466	0.823807	3648252
PRN2	1.050296	0.890179	3639879
C1_06600W_A	1.05005	0.836064	3639513
TAF19	1.049988	0.895913	3641396
CFL5	1.049054	0.935066	3642612
PHO23	1.048562	0.873706	3643926
SNM1	1.047869	0.903367	3642609
FGR6-1	1.0478	0.951093	3647947
C7_03620C_A	1.045706	0.869309	3646242
SLA2	1.045367	0.86238	3643149
SRT1	1.044998	0.922075	3642020
FCR3	1.044921	0.881899	3643760
C1_11370C_A	1.044565	0.898645	3644547
SAM2	1.043465	0.899639	3644538
C2_01230W_A	1.042779	0.931639	3638795
GSL2	1.042764	0.884062	3640410
FGR43	1.041536	0.932161	3634828
HGT8	1.041514	0.88428	3638875
C6_02420W_A	1.041001	0.891815	3645885
C3_01260C_A	1.040005	0.904383	3636625
C2_10760C_A	1.038661	0.88735	3641238
GLC7	1.037344	0.864303	3647257
PGA5	1.037233	0.971669	3644575
C7_01010W_A	1.03633	0.971117	3638021
CR_06310W_A	1.036324	0.971125	3644860
C1_06000W_A	1.036269	0.971599	3639870
IFF11	1.036263	0.97121	3640580
FET99	1.03625	0.971589	3647152
C5_01870W_A	1.036037	0.889276	3643752
RAD2	1.035286	0.914001	3645374
HSP60	1.034328	0.892001	3645275
C2_05410W_A	1.033967	0.894099	30515120
C1_03100W_A	1.033146	0.89096	3636925
TIM50	1.032282	0.909727	3644530
GCD1	1.031986	0.911404	3640965
C5_04610W_A	1.031693	0.906815	3636393
C3_01170W_A	1.031001	0.922171	3647958
CTA3	1.029528	0.896721	3645333
NAP1	1.029207	0.906321	3639714
CR_09700W_A	1.029171	0.941336	3647088

CGR1	1.029085	0.931963	3637853
C4_04760C_A	1.028821	0.930513	3641118
PTC1	1.028528	0.93039	3634827
C2_06300W_A	1.026826	0.952758	3646924
ARG83	1.026361	0.948067	3637862
C4_06130W_A	1.025553	0.920134	3648085
RPL39	1.025527	0.926084	30515109
C1_00660C_A	1.024988	0.931718	3639376
CR_06740W_A	1.024814	0.949294	3646616
C4_06770W_A	1.024076	0.938732	3642920
ECM14	1.02327	0.936048	3638392
CGT1	1.023243	0.943506	3641808
C3_03230C_A	1.022403	0.942414	3638316
PDC2	1.020826	0.956964	3634862
CR_05330W_A	1.020067	0.97388	30515404
C3_05990C_A	1.019831	0.973681	3641732
C1_02210W_A	1.019795	0.934091	3644594
C2_08660C_A	1.019753	0.973748	3643907
C1_11970C_A	1.019744	0.961483	3642044
C4_05400C_A	1.019379	0.948696	3646952
C2_03830W_A	1.019176	0.948465	3637399
C2_00060C_A	1.019123	0.942035	3647914
C3_06260C_A	1.018845	0.953261	3642240
C7_01390W_A	1.018055	0.98729	3645892
GPI13	1.018048	0.963683	3637424
ASH1	1.017549	0.960052	3641249
GAL102	1.017041	0.954861	3644585
MED21	1.016781	0.971644	3639010
BUD31	1.016519	0.973054	3634796
ABZ1	1.016143	0.948516	3648307
C5_03010W_A	1.014087	0.966518	3637819
CBK1	1.013557	0.959043	3634838
C7_00060C_A	1.012607	0.971789	3638013
CHS3	1.012572	0.959362	3636199
C2_09710C_A	1.011882	0.963634	3643786
C5_04640C_A	1.011553	0.957021	3636397
ASR3	1.011462	0.969618	3637402
HSP90	1.011323	0.965823	3636951
C1_03990W_A	1.011297	0.978399	3646424
CR_08110W_A	1.010852	0.980942	3642067
SNO1	1.010833	0.977456	3636812
CR_05140W_A	1.009921	0.984663	3646400
CR_07020W_A	1.009807	0.970945	3645731
ASG1	1.009336	0.97412	3647687
SSN3	1.007956	0.980197	3637353
TIF11	1.007319	0.975997	3641208
CAF16	1.007256	0.978943	3646486

C2_10160W_A	1.006902	0.991694	3643932
C1_10340W_A	1.005276	0.988483	3634909
RPO21	1.00515	0.98369	3638991
C4_03700W_A	1.00453	0.988393	3645361
C3_00170C_A	1.004126	0.991618	3640589
TIM9	1.004105	0.993036	3646243
FAA2	1.003531	0.990616	3641731
C1_08540C_A	1.003188	0.990071	3646497
CR_00630W_A	1.002948	0.991246	3639690
NOP5	1.002361	0.992538	3646084
C4_02590C_A	1.002091	0.995482	3637913
C1_14470W_A	1.001702	0.995639	3643327
TIF35	1.001644	0.995218	3643308
PES1	1.001289	0.996163	3644105
CDL1	1.000832	0.997747	3641132
C2_04380C_A	1.00054	0.998661	3645459
LPG20	-1.00148	0.996416	3644780
RPT2	-1.00348	0.988954	3640525
CR_10390W_A	-1.00413	0.993181	3638954
HOS3	-1.00505	0.98164	3637886
GSC1	-1.00523	0.98365	3636794
MDS3	-1.00542	0.983015	3638464
PIM1	-1.0058	0.97848	3640999
C4_05210W_A	-1.00596	0.989311	3646154
CR_01950W_A	-1.0065	0.981828	3640107
PGA56	-1.00658	0.980016	30515313
RPC11	-1.00696	0.987026	3641239
C3_04370C_A	-1.00772	0.977555	3635172
CR_10820W_A	-1.00785	0.98365	3638958
C2_09690C_A	-1.00895	0.978166	3643789
C1_12900W_A	-1.00925	0.980308	3636154
C1_11670W_A	-1.01029	0.971186	3645338
AKL1	-1.01157	0.964813	3641237
MCM3	-1.01239	0.973342	3645223
C2_03930C_A	-1.01334	0.965435	3637423
C1_05230W_A	-1.01394	0.975481	3642463
C6_00900C_A	-1.01414	0.976388	3644029
C1_02030C_A	-1.01456	0.977934	3645415
CR_02300C_A	-1.01565	0.954764	3644938
C4_00800W_A	-1.01589	0.984507	3645757
SPT10	-1.01654	0.956682	3645729
C1_02090C_A	-1.01661	0.962001	3644577
HCM1	-1.01741	0.98197	3634794
C2_02290C_A	-1.01742	0.981847	3635807
CR_06270W_A	-1.01742	0.981878	3644854
SAP3	-1.0175	0.98228	3635197
C7_03860W_A	-1.01797	0.945263	3643144

ZFU2	-1.01868	0.964809	3638534
DOT5	-1.01877	0.948165	3640519
CIS2	-1.01897	0.945886	3639366
C7_02960C_A	-1.01969	0.958013	3641387
FET33	-1.0199	0.951591	3641544
C6_04440C_A	-1.0206	0.946996	3645983
C2_03140C_A	-1.02195	0.933674	3642992
C5_02330W_A	-1.022	0.962462	3637789
CR_04170W_A	-1.02274	0.921568	3640977
C1_03280W_A	-1.02356	0.922448	3636901
C6_02090C_A	-1.02383	0.936558	6335445
ZCF13	-1.02463	0.929357	3646354
ALK2	-1.02471	0.948465	3639700
C3_02410C_A	-1.02599	0.93048	3638341
COX15	-1.02639	0.904365	3647071
RAT1	-1.02721	0.923144	3635686
POL32	-1.02757	0.930748	3639881
C3_06490W_A	-1.02795	0.921201	3642285
C7_00870W_A	-1.02898	0.954314	3638054
FGR22	-1.02898	0.954272	3635862
TLO4	-1.02978	0.956014	30515080
C7_04200C_A	-1.02985	0.923915	3643122
ATO1	-1.0299	0.957254	3647761
RPS19A	-1.03167	0.895711	30515208
C1_08760W_A	-1.03181	0.93395	3634787
GSL1	-1.03212	0.932114	3639844
OPT2	-1.03221	0.943518	3644915
NTG1	-1.03308	0.925609	3640746
YIM1	-1.03329	0.918078	3637372
TIP120	-1.03482	0.909057	3638494
DFG5	-1.03489	0.894621	3638867
C2_09310C_A	-1.03505	0.936418	3644104
C4_06440C_A	-1.03778	0.940701	3642793
CR_04180C_A	-1.03793	0.882511	3640937
C4_06090C_A	-1.03951	0.902493	3646532
C7_02540W_A	-1.03956	0.916871	3636939
DEM1	-1.03972	0.919048	3643200
BUR2	-1.03983	0.914708	3637772
FLC1	-1.03985	0.858778	3639854
LYP1	-1.03985	0.931779	6335417
C1_03950C_A	-1.04009	0.952246	3646417
PSA2	-1.04104	0.885084	3636159
BMT6	-1.04139	0.892485	3639258
C7_01030C_A	-1.04171	0.874035	3638019
HAL22	-1.04181	0.901338	3644051
CR_08590W_A	-1.04288	0.876203	3642106
C1_05890W_A	-1.04383	0.981411	3639876

C2_00310W_A	-1.04383	0.981411	3638800
C2_07870C_A	-1.04383	0.981411	3642319
C5_03770C_A	-1.04383	0.981411	3647233
C2_01800W_A	-1.04384	0.981409	30515092
C3_01970C_A	-1.04384	0.981409	3636597
C4_00730C_A	-1.04384	0.981409	3645765
C4_03490C_A	-1.04384	0.981409	3646799
C5_04140W_A	-1.04384	0.981409	3636524
C7_02260W_A	-1.04384	0.981409	3637002
CR_00530W_A	-1.04384	0.981409	3639668
CR_01220W_A	-1.04384	0.981409	3640406
CR_09350C_A	-1.04384	0.981409	3641901
PGA22	-1.04384	0.981409	3644939
SCW4	-1.04384	0.981409	3636797
C1_10980W_A	-1.04384	0.981406	3644425
C6_02620C_A	-1.04384	0.981406	3639211
C7_02130W_A	-1.04384	0.981406	3636997
FIG1	-1.04384	0.981406	3644025
DOA4	-1.04473	0.865434	3643337
C4_06430C_A	-1.04486	0.909533	3642794
CR_01570W_A	-1.04504	0.929569	3640077
VPS36	-1.04555	0.88588	3637354
IFG3	-1.04623	0.900822	3641545
FGR10	-1.04753	0.923751	3641159
C4_01300W_A	-1.04778	0.840056	3635605
ATF1	-1.04872	0.89687	3635273
CR_04120C_A	-1.04879	0.913606	3640972
MAF1	-1.05153	0.853054	3642332
RPT1	-1.05171	0.836618	3642461
C1_14080W_A	-1.05229	0.837337	3643345
C6_04650W_A	-1.05275	0.828394	3645979
MED7	-1.05325	0.911782	3638344
C7_02090C_A	-1.0533	0.892181	3637024
POL5	-1.05452	0.857206	3639254
CR_05030W_A	-1.05459	0.848748	3646388
GAP1	-1.05461	0.817623	3637806
C3_00270C_A	-1.0549	0.846285	3640527
C4_06880C_A	-1.0556	0.865944	30515287
CR_08550W_A	-1.05594	0.858677	3642141
VPS34	-1.05629	0.859953	3639540
UFE1	-1.05661	0.835836	3643174
C5_02820C_A	-1.0572	0.857072	3637808
C6_01000C_A	-1.0579	0.8806	3644048
C1_06970C_A	-1.05812	0.843181	3639567
MVD	-1.05952	0.816077	3639306
C7_03880C_A	-1.05999	0.909326	3643142
SIK1	-1.06059	0.79899	3638925

C2_06650C_A	-1.06072	0.886606	3648419
CRZ1	-1.06081	0.838325	3641722
PLB2	-1.06098	0.91358	3644562
C2_02180W_A	-1.06163	0.853538	3635752
DAP2	-1.06194	0.862267	3637815
MNN23	-1.06208	0.826397	3634813
C2_05710C_A	-1.06375	0.806426	30515127
YPT7	-1.06428	0.88874	3636864
CTA6	-1.06429	0.862465	3642907
AVT1	-1.06448	0.869039	3646928
PCL7	-1.06481	0.789089	3639491
C1_14460W_A	-1.0652	0.77867	3643326
SAM50	-1.06525	0.895554	3641721
C1_01470W_A	-1.06535	0.830139	3640241
CR_04160C_A	-1.06675	0.819095	3640976
C2_01490C_A	-1.06718	0.837554	3635800
C7_00780W_A	-1.06771	0.848683	3637945
C2_07620W_A	-1.06784	0.953827	3645194
CTN1	-1.06795	0.902939	3645414
LAB5	-1.06816	0.790025	3637893
C1_10200C_A	-1.06915	0.835424	3634877
C1_00430W_A	-1.06964	0.80485	3639285
ORC1	-1.0701	0.807826	3636922
C4_04620C_A	-1.07025	0.936458	3641155
C7_02850W_A	-1.07085	0.798988	3641399
MED19	-1.07148	0.824934	30515334
SAC3	-1.07176	0.806531	3635823
NMA111	-1.0722	0.755506	3640179
MRPL10	-1.07248	0.82149	3643446
C3_05680W_A	-1.07345	0.831235	3641724
CR_03220C_A	-1.07363	0.880728	3645128
C1_03400W_A	-1.074	0.74944	3636904
RPB4	-1.07466	0.823134	3647800
MET28	-1.07568	0.787833	3637962
CR_07480W_A	-1.07603	0.762316	3646047
C1_02220C_A	-1.07729	0.815764	3644595
GPR1	-1.0777	0.833549	3642616
CTA26	-1.0779	0.808022	3638965
C5_03470C_A	-1.07914	0.816539	3646963
MET4	-1.07961	0.77972	3641219
PGA1	-1.08003	0.859954	3638980
CR_09240C_A	-1.08109	0.793505	3641919
C1_01840C_A	-1.08121	0.788906	3645432
C4_04550C_A	-1.08257	0.770566	3641106
FGR28	-1.08261	0.832109	3648315
C1_04700C_A	-1.08265	0.781376	3644766
CR_04280C_A	-1.0827	0.809074	3640993

GDI1	-1.0842	0.753299	3643298
HMT1	-1.08422	0.772162	3640182
UCF1	-1.08437	0.77697	3648170
SAP98	-1.08455	0.831479	3637435
C5_02480W_A	-1.0847	0.772765	3637762
C5_04350C_A	-1.08518	0.79209	3636482
C5_00100C_A	-1.08532	0.796218	3647883
VPS22	-1.08542	0.882517	3647250
ISA1	-1.08636	0.736197	3639174
PRK1	-1.08685	0.741964	3640153
C1_10470W_A	-1.08783	0.774336	3647184
C1_02900C_A	-1.0881	0.792359	3636869
PAN1	-1.08853	0.737356	3637386
C1_00160C_A	-1.08867	0.720851	3639341
YVH1	-1.08899	0.793804	3643218
MNN11	-1.08903	0.834623	3636792
C5_02360C_A	-1.08921	0.910807	3637792
C4_02580W_A	-1.08926	0.911365	3637915
C3_05330C_A	-1.08928	0.909928	3643650
C5_01040W_A	-1.08929	0.911097	3642649
FAV2	-1.08986	0.935304	3647236
C3_05760W_A	-1.09079	0.782464	3641720
GCN20	-1.0911	0.779935	3639314
C4_01560C_A	-1.0917	0.785937	3635714
PUT4	-1.09262	0.851325	3636801
NCP1	-1.09268	0.751528	3637839
C7_00810W_A	-1.09272	0.740992	3637940
MEA1	-1.09302	0.88397	3635154
GLN4	-1.09325	0.705354	3637998
ESS1	-1.09354	0.747264	3647989
CR_10020C_A	-1.09487	0.848343	3639032
MNT3	-1.0962	0.805272	3648123
C4_02880C_A	-1.09632	0.768467	3637907
AGO1	-1.0967	0.75493	3642828
C4_00840W_A	-1.09682	0.708554	3635722
CR_06510W_A	-1.09724	0.893112	3645264
C4_01290W_A	-1.09867	0.709898	3635699
MRPL3	-1.10031	0.767361	3640722
RBR3	-1.10118	0.73601	3646728
C5_04980W_A	-1.10124	0.809022	3636536
SEC7	-1.10166	0.698387	3635220
PRS1	-1.10223	0.696128	3635817
C1_04630C_A	-1.1026	0.752365	3644762
PTC2	-1.10281	0.675558	3640105
PDE1	-1.10382	0.739559	3637713
C2_01530C_A	-1.10401	0.64753	3635803
C3_05320W_A	-1.10407	0.736015	3643648

C2_01760C_A	-1.10694	0.837136	3635736
C2_01440C_A	-1.10701	0.834856	30515090
C1_04990C_A	-1.10707	0.773078	3647530
CR_01170W_A	-1.10771	0.690351	3640429
C3_07660W_A	-1.10808	0.742845	3638462
SKI3	-1.10852	0.687928	3634847
IRA2	-1.10862	0.695103	3642011
DBR1	-1.10991	0.776431	3647560
C4_03130W_A	-1.11087	0.73521	3637921
RLP24	-1.11107	0.714299	3645748
CR_00570W_A	-1.11128	0.72692	3639664
IRO1	-1.11242	0.677425	3636648
KIS2	-1.11292	0.702649	3636211
C1_13790C_A	-1.11353	0.802035	3636141
C1_09310C_A	-1.11359	0.759564	3634833
C7_02530C_A	-1.11465	0.721609	3636940
C5_01920C_A	-1.11467	0.731128	3643742
CBF1	-1.11496	0.679117	3642779
C3_00420W_A	-1.11537	0.714023	3640553
C5_00140C_A	-1.11597	0.763721	3647873
NRP1	-1.11717	0.811665	3640698
YML6	-1.11795	0.73604	3637954
YTA6	-1.11812	0.639943	3638990
SEC65	-1.11929	0.690901	3640123
C6_01220C_A	-1.12024	0.715028	3644042
C1_09280W_A	-1.12065	0.714217	3634829
SEC9	-1.12102	0.64195	3644052
ALP1	-1.12172	0.649272	3644437
GCD11	-1.12204	0.643292	3637755
RPA135	-1.12249	0.678014	3637996
RPS10	-1.12373	0.578022	30515149
USO6	-1.12381	0.700989	3643647
APN2	-1.12401	0.7561	3647674
SKN7	-1.12406	0.699192	3641563
MSF1	-1.12425	0.681387	3638806
C1_11680C_A	-1.12459	0.763508	30515056
C4_05650W_A	-1.12518	0.702871	3641814
CR_04750W_A	-1.12539	0.80804	30515399
C2_10820C_A	-1.12632	0.671724	3641230
FAD3	-1.12671	0.717209	3636124
NMD5	-1.12676	0.662645	3645745
C5_03480C_A	-1.12678	0.684587	3646965
SFL2	-1.12696	0.735133	3636434
C2_07370W_A	-1.1288	0.695803	3645233
C6_03530C_A	-1.12892	0.818643	3640260
CR_04270C_A	-1.129	0.818784	3640992
PUS7	-1.12905	0.690539	3643960

C6_01550C_A	-1.13059	0.728163	3641653
C6_01210W_A	-1.13105	0.688364	30515330
LEM3	-1.1323	0.690099	3643456
UTP20	-1.13275	0.688324	3647956
C2_02490C_A	-1.13441	0.60456	3635815
C1_08920W_A	-1.13496	0.70357	3634910
C1_14310W_A	-1.1357	0.724371	3643315
SEC15	-1.13578	0.637906	3646845
CR_05560W_A	-1.13578	0.678214	3647592
TPO5	-1.13591	0.730384	3647791
SAP1	-1.13639	0.834297	3640256
AFL1	-1.13647	0.574301	3641594
CR_02940C_A	-1.13653	0.834882	3642589
CR_10620C_A	-1.13954	0.627032	3639006
C2_04400W_A	-1.1398	0.601538	3645457
C7_00910C_A	-1.14036	0.740591	3637958
CR_00230W_A	-1.14038	0.586156	3639698
C5_01170W_A	-1.14047	0.773284	3642662
LYS144	-1.14139	0.661054	3640512
C4_01130C_A	-1.14152	0.765473	3635682
GYP8	-1.14203	0.669939	3637750
SWI1	-1.14248	0.589801	3644996
C2_07030C_A	-1.14288	0.687051	3643498
C2_06430C_A	-1.14305	0.733082	3646558
C3_05420W_A	-1.14354	0.659209	3643666
C6_02450W_A	-1.14485	0.617261	3639167
MPH1	-1.1453	0.704111	3642817
C2_06490W_A	-1.147	0.646734	3646569
C5_04330W_A	-1.14721	0.715824	30515314
HNM4	-1.14722	0.846343	3636802
NOC2	-1.14774	0.604765	3647596
C2_05960C_A	-1.14857	0.834568	3647479
EST3	-1.14859	0.833657	3640551
CR_03070W_A	-1.14886	0.607582	3645105
C1_05980W_A	-1.14931	0.66599	3639872
C3_07420W_A	-1.15012	0.600306	3638485
STE50	-1.15121	0.635638	3636660
MEP2	-1.15233	0.741661	3645022
KIS1	-1.15312	0.604871	3644063
FGR6-4	-1.15313	0.680605	30515164
C3_01670W_A	-1.15391	0.604059	3636606
C5_00170W_A	-1.15414	0.617255	3647870
HOL4	-1.1548	0.545614	3645409
SAP9	-1.15549	0.594996	3645625
MRPL8	-1.15666	0.660326	3646039
C3_07350W_A	-1.15716	0.610991	3638476
KCH1	-1.15787	0.703365	3636988

C2_07860W_A	-1.15864	0.628497	3642320
CCR4	-1.15999	0.522625	3640749
MED4	-1.16052	0.635503	3645209
C2_07520C_A	-1.16063	0.626188	3645207
C5_04720C_A	-1.16071	0.627503	3636518
C3_03780W_A	-1.16088	0.792321	3645637
SPA2	-1.16113	0.509246	3646824
GPI8	-1.16252	0.613379	3646669
C1_03620C_A	-1.16277	0.619452	30514981
C1_03360W_A	-1.1637	0.540763	3636889
PEP7	-1.16377	0.679361	3645001
C3_07690C_A	-1.16439	0.626522	3638458
C2_08240W_A	-1.16517	0.542266	3642344
C2_04740C_A	-1.16627	0.775572	3647786
NAM7	-1.16713	0.544158	3641540
C3_01520C_A	-1.16808	0.563493	3636570
GAL10	-1.16968	0.513035	3644583
C2_06230W_A	-1.1701	0.523307	3646911
SNF1	-1.17056	0.53282	3642643
MED9	-1.17068	0.645555	3643134
C5_00260W_A	-1.17186	0.455135	3641561
C1_07860W_A	-1.17198	0.838141	3640740
C3_07240W_A	-1.17206	0.835417	3638530
FGR32	-1.1721	0.451865	3644136
ATO9	-1.17216	0.84323	3640392
HYR3	-1.17255	0.486622	3644119
SSP96	-1.17372	0.843974	3641401
C3_03330C_A	-1.1738	0.561895	3638364
RPP1	-1.17443	0.736862	3644681
C4_06450W_A	-1.17458	0.74001	3642792
C2_00620C_A	-1.17506	0.70176	3638849
CHS4	-1.17553	0.502075	3641726
SEC6	-1.17617	0.519951	3640565
PPS1	-1.17622	0.709687	3646526
HGT19	-1.17644	0.558923	3640558
C1_01180C_A	-1.17696	0.775596	3640206
C3_04380C_A	-1.177	0.533309	3635173
C1_07400C_A	-1.17772	0.643142	3644464
C2_05540C_A	-1.17835	0.666687	3645559
C2_08770C_A	-1.17948	0.717847	3643878
TRS33	-1.18168	0.688358	3636992
TRM2	-1.18258	0.630044	3640235
NPR1	-1.18313	0.554474	3639498
MED22	-1.18351	0.782866	3640217
C3_02690C_A	-1.18367	0.636141	3638407
YDJ1	-1.18381	0.465435	3640939
RBR2	-1.18395	0.74906	3640983

HGH1	-1.18496	0.579402	3635647
C2_03780C_A	-1.18518	0.533344	3637368
C7_02340C_A	-1.18564	0.606074	3636933
CR_08280W_A	-1.18625	0.641836	3642102
C2_00790C_A	-1.18634	0.627101	3638907
HAP31	-1.18767	0.725898	3640994
C1_02770W_A	-1.18779	0.736733	30514973
C2_05350C_A	-1.18779	0.736596	3643428
CR_09310W_A	-1.18927	0.571427	3641923
RAC1	-1.18999	0.551216	3639508
SPT23	-1.19069	0.474843	3643958
SRD1	-1.19145	0.686571	3635764
OPI1	-1.19206	0.57044	3635777
RPT6	-1.19269	0.425884	3643877
C1_08770W_A	-1.19383	0.559073	3634788
HGT14	-1.19389	0.601575	3648320
ZCF31	-1.19417	0.523312	3635191
C1_14430C_A	-1.19476	0.556231	3643297
C3_04230W_A	-1.19607	0.579936	3635187
C4_07150W_A	-1.19634	0.732776	3642935
C7_00940W_A	-1.19937	0.509696	3637955
C3_06440W_A	-1.19953	0.438983	3642281
SWD1	-1.20139	0.58082	3640535
DUO1	-1.20234	0.6862	3646858
C5_04870W_A	-1.20282	0.587069	3636438
C1_05210C_A	-1.20316	0.570821	3642465
C5_00040C_A	-1.20357	0.532528	3647874
C3_02080W_A	-1.20357	0.552554	3636575
CR_05710C_A	-1.20391	0.707352	3646700
CR_03430W_A	-1.20401	0.707181	3645114
C6_03550C_A	-1.20411	0.715439	3640246
C1_04600C_A	-1.20557	0.513041	3646133
C3_07600W_A	-1.20557	0.568667	3638495
CR_08330W_A	-1.20583	0.54376	3642080
C5_02830W_A	-1.20618	0.519163	3637809
C2_01050W_A	-1.20627	0.734967	30515086
CR_06090W_A	-1.20761	0.493849	6335359
MIH1	-1.20843	0.439857	3648188
NUP60	-1.20922	0.464292	3642826
CYM1	-1.2097	0.425694	3642244
ZCF23	-1.21001	0.520373	3644477
C2_00420W_A	-1.21158	0.486606	3638819
C2_09460C_A	-1.21183	0.448381	3647713
C1_00970W_A	-1.21197	0.774736	30514958
ALG1	-1.21265	0.584425	3646517
C4_01820C_A	-1.21425	0.477434	3635594
CR_03780C_A	-1.21548	0.685907	3643179

C1_00890W_A	-1.21602	0.530165	3639354
UTP13	-1.21631	0.442684	3637718
C5_00250C_A	-1.21639	0.669399	3641562
PRC2	-1.21715	0.57052	3647044
HRQ2	-1.21985	0.516307	3647850
C2_02540W_A	-1.22144	0.471471	3635820
CR_09140C_A	-1.22155	0.456939	3641879
CWC22	-1.22192	0.590131	3643938
CR_10740W_A	-1.22316	0.528147	3638941
HAC1	-1.2234	0.484982	3639849
KAP120	-1.22461	0.338211	3643110
C1_12710C_A	-1.22531	0.497765	3646822
ARO1	-1.22581	0.34638	3635680
MED8	-1.22655	0.653617	3645446
C3_03530W_A	-1.22773	0.721187	3638312
C5_02660C_A	-1.22864	0.485146	3637771
DCR1	-1.22887	0.49301	3641164
C1_00880W_A	-1.22958	0.563952	3639355
NOT3	-1.23061	0.442706	3638829
C4_03750C_A	-1.23124	0.596253	3645356
RRP6	-1.23139	0.486097	3647535
CR_03310C_A	-1.23335	0.392411	3645097
SCS7	-1.23355	0.389008	3641157
LIP9	-1.23455	0.833797	3641395
C4_02210W_A	-1.23456	0.829521	3635695
CR_02440W_A	-1.23457	0.737121	3644923
PGA58	-1.23464	0.831801	3637826
C3_04170W_A	-1.23465	0.831549	3635182
C5_00510W_A	-1.23465	0.833825	3641539
C6_03070C_A	-1.23465	0.833774	3639272
PGA27	-1.23475	0.666783	3638811
CR_07250C_A	-1.23478	0.480105	3646028
C5_00330C_A	-1.23534	0.464231	3641556
C7_00830C_A	-1.23602	0.395086	3637938
CDR3	-1.23634	0.64274	3645365
CR_03230W_A	-1.23726	0.484095	3645127
C4_00880W_A	-1.23731	0.506873	3635681
VPS17	-1.23765	0.441203	3640218
PEX13	-1.23818	0.42656	3641877
PUT3	-1.2387	0.423026	3639535
C4_03140C_A	-1.23896	0.557337	3637843
CSI2	-1.23916	0.506489	3642016
C1_08470W_A	-1.24212	0.619562	3646490
C1_13650C_A	-1.24222	0.525291	3636191
SCT1	-1.24291	0.459711	3648346
C4_00950C_A	-1.24311	0.439952	3635675
IME2	-1.24352	0.833454	3645099

C7_00130W_A	-1.24429	0.477965	3637975
C2_03530W_A	-1.24443	0.447552	3637341
MKC1	-1.2448	0.366964	3639710
C4_06210C_A	-1.24603	0.399383	3642815
DJP1	-1.24727	0.394936	3647186
CR_07820W_A	-1.24913	0.693128	3647344
FGR27	-1.25016	0.458403	3646988
STR2	-1.25051	0.438555	3644689
C5_02620C_A	-1.25242	0.403355	3637783
MET16	-1.25282	0.445228	3642964
C4_06650W_A	-1.25295	0.381727	3642810
C3_07380W_A	-1.25379	0.436612	3638473
JAB1	-1.25481	0.414679	3646811
C3_04120C_A	-1.25633	0.550117	3635282
C5_02730C_A	-1.25677	0.413316	3637738
C6_03380W_A	-1.25687	0.426808	3639231
SUI2	-1.25745	0.350693	3639569
C1_03450C_A	-1.25851	0.37516	3636910
YPS7	-1.26112	0.452805	3636937
NOP15	-1.26156	0.45945	3637966
C1_14450C_A	-1.26189	0.416525	3643299
PWP1	-1.26265	0.42028	3635623
C4_03830W_A	-1.26288	0.426664	3647851
C5_02140C_A	-1.26399	0.433028	3637752
C7_00030W_A	-1.26409	0.444253	3638004
C3_05970C_A	-1.26637	0.375952	3641730
C1_11790W_A	-1.26698	0.634339	3645326
C3_07170C_A	-1.26816	0.344472	3638536
C4_04120C_A	-1.26835	0.427158	3641222
C2_00860C_A	-1.2684	0.765906	3638805
C2_00890W_A	-1.26855	0.76358	3638896
ZPR1	-1.26936	0.258754	3640177
VPS20	-1.26981	0.570262	3637427
RPS42	-1.26988	0.448428	3640195
ZRT1	-1.27198	0.776571	3642970
C2_08420W_A	-1.27276	0.603072	3643897
C3_07100C_A	-1.2735	0.380355	3638443
CAS4	-1.27527	0.456261	3636568
C3_03540W_A	-1.27537	0.376242	3638313
C7_00450C_A	-1.27634	0.493072	3637991
C6_01920C_A	-1.27677	0.39736	3644534
C2_04620W_A	-1.27685	0.273376	3645438
C1_07390W_A	-1.27702	0.295293	3644466
KIC1	-1.27744	0.375965	3647784
CR_00010C_A	-1.27819	0.355856	3639712
CR_06040W_A	-1.27819	0.334225	3644836
C2_00270C_A	-1.27881	0.472596	3638803

BNI1	-1.27896	0.322941	3636118
PHO8	-1.28109	0.71481	3647179
TIF4631	-1.28136	0.311982	3643880
ECM18	-1.28248	0.707422	3645655
CR_10380C_A	-1.28263	0.701311	3638953
C4_00850C_A	-1.28271	0.706417	3635718
APC1	-1.28273	0.374189	3639383
TAC1	-1.28422	0.365772	3643755
DOS2	-1.28422	0.391723	3636183
HAS1	-1.28552	0.25258	3636443
TIF3	-1.28572	0.367962	3641646
C1_12280C_A	-1.2861	0.672134	3642019
C4_00470C_A	-1.28664	0.331585	3645011
GLK1	-1.28864	0.329479	3648348
C3_06050C_A	-1.28865	0.465143	3641746
C1_12530C_A	-1.28899	0.545326	3647608
C1_01040W_A	-1.2897	0.48848	3640183
C3_03100C_A	-1.28975	0.347821	3638399
C2_02720W_A	-1.29005	0.339055	3643002
CEM1	-1.2941	0.467621	3635242
C4_00560C_A	-1.29411	0.39371	3645744
C1_00530C_A	-1.29412	0.348005	3639368
PGM2	-1.29415	0.273287	3642562
NCE102	-1.29464	0.329383	3635267
ALO1	-1.29491	0.281705	3638983
YEA4	-1.29518	0.643712	3643787
C2_05490W_A	-1.29519	0.643718	3643417
GPI2	-1.29529	0.64241	3641011
CDC39	-1.29557	0.235273	3646278
C5_01930W_A	-1.29603	0.397932	3643741
EAP1	-1.29696	0.352328	3643814
C5_03040W_A	-1.29878	0.444559	3637824
CR_04140W_A	-1.29889	0.344198	3640974
WAL1	-1.29984	0.25957	3647093
TEL1	-1.30065	0.440034	3639263
HYR4	-1.30142	0.335288	3640436
TPS1	-1.30153	0.29898	3646699
C2_06440C_A	-1.30294	0.368643	3646565
XOG1	-1.30413	0.431735	3636837
CR_02590C_A	-1.30432	0.402281	3642595
C4_04510W_A	-1.30506	0.234354	3641120
C2_08510W_A	-1.30632	0.600579	3643922
C2_07240C_A	-1.30639	0.706979	3643486
ECM38	-1.30646	0.54049	3645375
C4_03680C_A	-1.30687	0.545251	3645363
CR_04240C_A	-1.30749	0.297496	3640989
C6_00390W_A	-1.3083	0.340779	3646086

C1_13240W_A	-1.30943	0.325729	3636167
VPS70	-1.30945	0.407432	3637971
C5_05090W_A	-1.30961	0.474719	3636493
C4_02450W_A	-1.3099	0.439693	3637845
POT1-2	-1.30992	0.461362	3638908
C4_06370C_A	-1.31012	0.463382	3642824
ARX1	-1.3102	0.213896	3636811
C2_00820W_A	-1.31191	0.401668	3638809
YCK2	-1.31229	0.307598	3643655
C3_03070W_A	-1.31317	0.401199	3638396
GCD6	-1.31506	0.333843	3646510
C3_06280W_A	-1.31507	0.315318	3642238
LIG4	-1.3153	0.326627	3642986
C3_04410C_A	-1.31567	0.290754	3635178
PIF1	-1.31569	0.329283	3646054
C2_01070W_A	-1.31658	0.325267	3638834
CR_03940W_A	-1.31692	0.36873	3648378
YUH2	-1.31913	0.34044	3636412
C7_01230C_A	-1.31942	0.313303	3645906
ZCF8	-1.32178	0.355093	3636651
C4_05260W_A	-1.32242	0.350726	3648282
CAN1	-1.32284	0.354815	3644035
C7_00430W_A	-1.32293	0.326215	3637993
ERG25	-1.32402	0.396755	3644933
CR_02460W_A	-1.32558	0.293239	3644922
C6_03390W_A	-1.32583	0.372833	3639232
C1_13840W_A	-1.32588	0.237604	3636129
CUP1	-1.32668	0.414351	30515318
TTR1	-1.32815	0.381848	3639313
C5_04340W_A	-1.32968	0.532955	3636481
C2_06080C_A	-1.33039	0.339719	3644098
CR_08710W_A	-1.33062	0.197917	3642131
GCD2	-1.33118	0.298209	3638437
ZNC1	-1.33135	0.260221	3643754
SFC1	-1.33211	0.467339	3636413
C1_11270W_A	-1.333	0.300558	3644555
C2_02040W_A	-1.33417	0.27767	3635848
PRO1	-1.33485	0.263213	3639000
HEX1	-1.33501	0.336325	3646981
C4_00740W_A	-1.33694	0.555986	3645764
C1_02380C_A	-1.33705	0.55451	3636790
C1_01090C_A	-1.33729	0.434592	3640174
C2_02420C_A	-1.33736	0.333938	3635756
C2_04500W_A	-1.33799	0.337609	3645462
C5_00090C_A	-1.33855	0.34111	3647885
RPL40B	-1.33943	0.236055	30515232
CR_10610C_A	-1.34047	0.569326	3639005

C6_03590C_A	-1.3406	0.468933	3640251
PGA25	-1.34207	0.325623	3647890
CAS5	-1.34266	0.333046	3635652
HAP5	-1.34299	0.303747	3642654
C6_01490C_A	-1.34473	0.604083	3641620
C4_07200C_A	-1.34514	0.341089	3642958
CR_03960C_A	-1.34621	0.469766	3640963
C7_04140C_A	-1.34699	0.260385	3643129
SFL1	-1.34744	0.189449	3642498
RLI1	-1.34749	0.297496	3636888
C1_06770W_A	-1.34929	0.275105	3639504
TAF145	-1.3507	0.153915	3639486
IMH3	-1.35077	0.231762	3646556
C7_03980W_A	-1.35184	0.410421	3643116
C2_09860C_A	-1.35366	0.513423	3643781
C1_05800C_A	-1.354	0.481123	3639887
C2_05380W_A	-1.3546	0.310099	3643432
C2_04730W_A	-1.35723	0.329722	3647787
C1_05380C_A	-1.35735	0.355245	3642480
FUR4	-1.35777	0.498633	3638305
C2_03950W_A	-1.3578	0.401677	3637420
C3_00950C_A	-1.35804	0.407463	3639852
FMP27	-1.36082	0.259161	3641645
DAL81	-1.3613	0.363585	3640419
C3_00400C_A	-1.36152	0.328023	3640554
C1_04850C_A	-1.36185	0.648923	30514989
DUR4	-1.36203	0.416489	3645012
GST3	-1.36204	0.653372	3645278
ULP2	-1.36371	0.287214	3643196
C4_03850W_A	-1.3648	0.180592	3647853
TSM1	-1.36521	0.209262	3645953
DUR7	-1.36711	0.357929	3643335
CR_10230W_A	-1.36716	0.204354	3638921
C6_03300C_A	-1.36766	0.235224	3639196
C3_05940C_A	-1.36787	0.504768	3641744
SUA72	-1.36797	0.505055	3648239
RAD18	-1.36801	0.476119	3641637
CR_03350C_A	-1.36804	0.295307	3645142
C1_05360C_A	-1.36935	0.292701	3642482
C1_01540W_A	-1.36997	0.274365	3640233
C3_01850W_A	-1.37024	0.355478	3636565
C2_00740C_A	-1.37031	0.24163	3638912
C6_03540W_A	-1.37168	0.308858	3640244
PFK2	-1.37303	0.25462	3637056
SOL1	-1.37463	0.326136	3645627
MRP7	-1.3747	0.394895	3643118
YAF9	-1.37586	0.336113	3647864

C3_02720W_A	-1.37671	0.367405	3638410
C5_01610W_A	-1.37874	0.356213	3647251
SNF7	-1.37994	0.180472	3639377
C5_00320W_A	-1.38073	0.394071	30515291
C7_00460W_A	-1.38206	0.678236	3637990
C2_02230C_A	-1.38216	0.680504	3635775
CR_01790C_A	-1.38304	0.363273	3640161
CR_00110W_A	-1.38304	0.199308	3639731
C1_03830C_A	-1.38427	0.277401	3644678
C1_09220W_A	-1.38431	0.238194	3634898
C4_01090C_A	-1.38464	0.477583	3635685
C2_05160C_A	-1.38495	0.133275	3643461
C1_00510W_A	-1.38564	0.290794	3639311
C1_12820C_A	-1.38579	0.281756	3646826
CR_10730C_A	-1.38637	0.221759	3638998
C4_06590W_A	-1.38638	0.486637	3642778
CTA9	-1.38687	0.249308	3640215
GNP1	-1.38698	0.260328	3646073
COX9	-1.38776	0.236939	30515131
C4_05980C_A	-1.38884	0.301286	3646516
C1_03330C_A	-1.38929	0.299928	3636859
C7_00610C_A	-1.38979	0.239357	3638030
CR_01770C_A	-1.39013	0.166927	3640122
MSS11	-1.39082	0.350259	3647296
C3_04690C_A	-1.39115	0.443731	3635196
C3_00510W_A	-1.39129	0.450849	3640514
CR_01410C_A	-1.39216	0.293565	3640136
C5_03940C_A	-1.39255	0.307008	3647446
C1_12540W_A	-1.39289	0.465886	3647607
C1_05650W_A	-1.39523	0.343316	3639838
C7_01370W_A	-1.39695	0.484014	3645901
C5_02740W_A	-1.39731	0.348072	3637739
CR_00340C_A	-1.39818	0.557002	30515365
BUD2	-1.39992	0.25806	3641541
VID27	-1.40098	0.143006	3647894
HMI1	-1.40158	0.262256	3638997
C4_04020C_A	-1.40173	0.597245	3641215
C1_08900W_A	-1.40283	0.477173	3634925
C3_07590W_A	-1.40289	0.4508	3638496
RPA190	-1.40372	0.2417	3647677
CRL1	-1.40398	0.208594	3640238
CRH12	-1.40466	0.159612	3636447
C4_06230C_A	-1.40524	0.188488	3642808
C5_01310W_A	-1.40566	0.510769	3642645
MAS2	-1.40604	0.247337	3647249
C3_05020W_A	-1.40645	0.421956	3635170
GPX2	-1.4098	0.345824	3644009

C2_01030W_A	-1.4102	0.258283	3638836
ZCF18	-1.41092	0.294514	3641635
C4_04820C_A	-1.41096	0.366881	3641165
SLK19	-1.4118	0.19365	3638466
RLM1	-1.41205	0.242597	3635703
DIM1	-1.41247	0.365634	3636135
C4_00580W_A	-1.41322	0.362535	3645773
C2_08100W_A	-1.4141	0.225587	3642334
C3_06040W_A	-1.41522	0.472754	3641745
C3_05140C_A	-1.41574	0.264343	3635161
C1_04490W_A	-1.41586	0.235668	3646145
HST6	-1.41789	0.754201	3642288
BUD6	-1.41854	0.181266	3640727
C2_07430C_A	-1.42031	0.297592	3645201
C4_02700W_A	-1.42042	0.236321	3637859
KRR1	-1.42063	0.228057	3644542
GCN3	-1.42079	0.246261	3645905
GDH2	-1.42097	0.21644	3642295
C6_01890C_A	-1.421	0.21949	3641598
HSM3	-1.42191	0.261472	3646713
C2_10140W_A	-1.42253	0.456009	3643934
FCY23	-1.42267	0.241733	3647681
C4_02960W_A	-1.42302	0.150904	3637873
GPM2	-1.4249	0.149482	3644713
C1_00200C_A	-1.42513	0.427515	3639336
MRPL33	-1.4255	0.423747	30515175
C1_09350W_A	-1.42559	0.244058	3634805
MRPS9	-1.42671	0.193408	3642064
SKN2	-1.42708	0.230507	3638309
C6_02900C_A	-1.42716	0.393736	3639240
CR_10630W_A	-1.42723	0.439479	3639007
ECM7	-1.42732	0.229395	3645029
NSA1	-1.4299	0.26133	3642313
C2_07000W_A	-1.43039	0.228393	3643534
C3_04350C_A	-1.4319	0.729612	3635275
AFG1	-1.43201	0.279266	3634920
UTP21	-1.43206	0.281186	3635757
PMA1	-1.43217	0.145791	3640531
FGR44	-1.43285	0.203237	3635166
C5_03850W_A	-1.4332	0.270882	3647225
PTR22	-1.43333	0.188514	3645634
C4_01800W_A	-1.43389	0.728266	3635595
CR_02780W_A	-1.43389	0.728439	3642582
C1_07810C_A	-1.43426	0.292788	3640708
C7_02350C_A	-1.43455	0.226795	3636932
CR_01720W_A	-1.43504	0.217498	3640114
C2_07770C_A	-1.43506	0.718928	30515144

CR_04990C_A	-1.43515	0.719028	3646391
C5_00760W_A	-1.43527	0.186002	3644122
C4_06420W_A	-1.43606	0.180974	3642795
C6_03260W_A	-1.4364	0.27702	3639183
TERT	-1.43666	0.282586	3640729
SPT7	-1.43847	0.174628	3638928
OLE1	-1.43909	0.18685	3640755
BCR1	-1.44072	0.185857	3645280
CR_06450W_A	-1.44127	0.306176	3645279
C7_03240W_A	-1.44727	0.087874	3641368
UBA4	-1.44779	0.185381	3644432
PEX12	-1.44947	0.212967	3638862
PAM17	-1.45104	0.193293	3638369
C3_01230C_A	-1.45187	0.576132	3636609
C6_01330C_A	-1.45187	0.57603	3641643
C1_11530C_A	-1.45187	0.57618	3645334
C5_04630W_A	-1.45215	0.396113	3636395
KIN3	-1.4525	0.123464	3641199
C1_00030C_A	-1.45313	0.312766	3639373
C7_02860C_A	-1.45432	0.165861	3641397
CKB2	-1.45491	0.226577	3637741
C3_05540C_A	-1.45621	0.52424	3643658
PDC12	-1.45799	0.436753	3635591
C7_03580C_A	-1.45834	0.234683	3646238
NRG1	-1.45882	0.116154	3643166
TOM7	-1.45926	0.409032	30515355
C2_08020C_A	-1.45958	0.162564	3642308
C5_05250C_A	-1.4604	0.212788	3636472
RGT1	-1.46236	0.192667	3637920
CR_03640C_A	-1.46241	0.162816	3643190
UTR2	-1.46257	0.197349	3636591
C1_05280W_A	-1.46361	0.29612	3642475
CSC25	-1.46483	0.161573	3645660
SWR1	-1.46523	0.118962	3645220
CR_03620C_A	-1.46601	0.094921	3643182
HAP3	-1.4661	0.594064	3635629
FET31	-1.46821	0.15056	3647151
C1_10330C_A	-1.46824	0.280637	3634908
TOM1	-1.46847	0.100655	3642987
TOP1	-1.46898	0.17753	3644034
C2_08960C_A	-1.46904	0.270503	3645787
C7_04150W_A	-1.46958	0.184946	3643128
C3_06140W_A	-1.47052	0.372383	3641764
C2_03260W_A	-1.47162	0.195492	3637432
CR_03180W_A	-1.47358	0.155523	3645133
C1_14410W_A	-1.47798	0.198191	3643293
C5_03440W_A	-1.47808	0.197512	30515310

C4_07130W_A	-1.4784	0.138169	3642938
ROM2	-1.47884	0.108527	3637434
C7_00270W_A	-1.47892	0.176949	3637977
CR_10770W_A	-1.47969	0.192978	3638944
C4_00080C_A	-1.48181	0.179722	3648065
C6_02690C_A	-1.48209	0.196236	3639228
TIM17	-1.48215	0.208741	3647790
C1_11610C_A	-1.48405	0.143757	3645345
CR_08940W_A	-1.48463	0.192993	3641914
TIM23	-1.48498	0.195609	3643777
C2_01740C_A	-1.48502	0.328225	3635733
C7_01590C_A	-1.48512	0.329463	3637037
C5_01530C_A	-1.48684	0.182502	3647062
C6_02110W_A	-1.48806	0.3559	3647553
C6_01940W_A	-1.49244	0.51682	30515337
C2_03560C_A	-1.49364	0.207898	3637337
SEO1	-1.49391	0.387679	3646621
C1_07570C_A	-1.49464	0.376204	3646675
WSC2	-1.49465	0.053701	3639225
C5_02310C_A	-1.495	0.344417	3637787
SDA1	-1.49506	0.081541	3646688
SMI1	-1.49507	0.117668	3640741
OFD1	-1.49646	0.139903	3646962
TFG1	-1.49662	0.053981	3635645
SPB1	-1.4973	0.168709	3648179
C3_00380C_A	-1.49868	0.211317	3640540
C6_03910C_A	-1.49868	0.213174	3640263
CR_06010W_A	-1.49892	0.2531	3644870
DCK1	-1.49932	0.077376	3637383
C7_03400C_A	-1.49975	0.15161	3646717
C4_06840W_A	-1.50056	0.336557	3642913
UBC8	-1.50064	0.091044	3645433
C2_05910W_A	-1.50084	0.225227	3647476
TGL99	-1.50304	0.161784	3636168
C3_00390W_A	-1.50325	0.409241	3640539
CR_04580W_A	-1.50848	0.136348	3640948
C4_06680C_A	-1.50909	0.116056	3642950
CR_10310W_A	-1.50926	0.626639	3638937
CCH1	-1.50984	0.124638	3640175
TPO2	-1.51001	0.505311	3643164
C7_00840C_A	-1.51029	0.319192	3638057
C3_02990C_A	-1.51052	0.30673	3638388
CDR2	-1.51292	0.180943	3635265
CR_06470W_A	-1.51353	0.08447	3645277
CR_09410W_A	-1.51388	0.174833	3641933
MRP20	-1.5145	0.179256	3640224
RRP42	-1.51635	0.251085	3647852

ILS1	-1.51681	0.088678	3645956
CR_01130W_A	-1.51737	0.117173	3640433
CDH1	-1.51748	0.141363	3638814
C6_03740W_A	-1.51811	0.59935	3640304
C2_08060W_A	-1.51893	0.482469	30515151
C1_05400C_A	-1.51902	0.390736	3642478
SPR28	-1.51919	0.372305	3637716
CR_00670C_A	-1.51921	0.233057	3639655
C2_07410W_A	-1.51962	0.161933	3645203
C1_11200W_A	-1.52082	0.164881	3644396
C1_11300C_A	-1.52133	0.159236	3644552
C6_03430C_A	-1.52158	0.106175	3640273
C7_01170C_A	-1.52319	0.132673	3645914
ENA2	-1.52451	0.110664	3639291
EXO70	-1.52471	0.089839	3636948
IMP4	-1.52794	0.093478	3644138
HRK1	-1.53047	0.077296	3640586
C5_02010C_A	-1.53092	0.12393	3643772
SOG2	-1.53099	0.081096	3644475
RMT2	-1.53105	0.178992	3641569
C7_02460C_A	-1.5311	0.071936	3636946
PBS2	-1.53148	0.079621	3641748
C4_04930C_A	-1.53192	0.348199	3641170
SPT6	-1.53258	0.089324	3643171
C3_01190C_A	-1.53303	0.110494	3647961
PGA62	-1.53329	0.125329	3637879
CR_07660C_A	-1.53676	0.11667	3647256
C3_01660W_A	-1.5369	0.216882	3636607
CR_08500W_A	-1.53698	0.086452	3642136
RPR2	-1.53772	0.413811	3641017
C1_11150W_A	-1.53819	0.241589	3648356
CR_04310C_A	-1.53876	0.199268	3640996
C1_13330C_A	-1.53899	0.094179	3636150
C1_01750W_A	-1.5413	0.350338	3645413
C2_03420C_A	-1.54259	0.116861	3637411
CR_08290W_A	-1.54325	0.085745	3642103
C3_00530C_A	-1.54333	0.218313	3640588
C1_02470W_A	-1.5439	0.402321	3636851
C6_00490W_A	-1.54393	0.322929	3647150
REP1	-1.54525	0.198466	3639708
CHO2	-1.54627	0.178417	3647690
HCR1	-1.5468	0.126638	3638952
C1_01820C_A	-1.54742	0.140633	3645435
C2_01750C_A	-1.54774	0.205297	3635734
CR_06320C_A	-1.54976	0.130713	3644845
C5_03110C_A	-1.55145	0.379361	30515306
CKB1	-1.55335	0.109231	3638799

C7_04170W_A	-1.55432	0.506349	3643126
CR_00980C_A	-1.55525	0.057306	3640426
C4_03200C_A	-1.55594	0.111372	3637837
CR_06680C_A	-1.55628	0.134244	3646623
C5_03700C_A	-1.55766	0.187417	3646990
C5_04320C_A	-1.55781	0.05913	3636480
GLN3	-1.55881	0.094763	3636453
C3_02260C_A	-1.55886	0.117553	3636636
C5_05210W_A	-1.56011	0.135816	3636468
C5_03640W_A	-1.56029	0.138929	3646984
C2_03410W_A	-1.56045	0.051572	3637412
SFU1	-1.56165	0.117766	3634867
ARL1	-1.56362	0.126498	3635637
AFT2	-1.56402	0.128334	3643511
ECM3	-1.56437	0.123202	3635754
DUG3	-1.56479	0.134025	3641381
C1_10910C_A	-1.56634	0.126581	3644445
BMS1	-1.5664	0.065815	3639857
C7_01400C_A	-1.56663	0.124279	3645893
C2_03880C_A	-1.56725	0.512467	30515108
PET100	-1.56916	0.189205	30515038
CR_06230W_A	-1.57064	0.239549	3644867
IFF4	-1.57217	0.188538	3639694
SRB8	-1.57355	0.082652	3641128
RHD3	-1.5737	0.057666	3641212
HGT16	-1.57447	0.456075	3646026
PBP2	-1.57669	0.047072	3640267
PTH1	-1.5773	0.096213	3637710
C2_10740C_A	-1.57855	0.117291	3641236
BPH1	-1.57889	0.076371	3639547
SLU7	-1.57935	0.172129	3638501
CR_01640C_A	-1.57976	0.473869	3640086
IPT1	-1.58004	0.074258	3634941
OPT8	-1.581	0.109419	3640266
AFG3	-1.58257	0.082593	3636589
ABD1	-1.5826	0.085862	3638456
C4_00070C_A	-1.58316	0.042474	3648063
C1_07960W_A	-1.58475	0.275439	3640725
C1_01590C_A	-1.58485	0.047259	3640223
SEC2	-1.58548	0.037203	3636119
DAO2	-1.58552	0.193226	3646797
CR_06530W_A	-1.58742	0.21289	3645262
C5_01300C_A	-1.58746	0.421897	3642646
C4_06960W_A	-1.58757	0.420506	3642926
C2_07510W_A	-1.58865	0.270502	3645208
CR_01630C_A	-1.58881	0.276099	3640085
C3_02030W_A	-1.58926	0.114891	3636580

C2_10680W_A	-1.58937	0.072525	3641252
C2_03460C_A	-1.59028	0.088865	3637348
PHO84	-1.5914	0.140859	3645348
BRO1	-1.59266	0.061204	3636590
FRP3	-1.59372	0.166467	3648102
C2_05300C_A	-1.59467	0.121302	3643423
MDR1	-1.59692	0.401067	3639260
C2_02440W_A	-1.59839	0.088947	3635758
ZCF38	-1.6031	0.107526	3639705
RIX7	-1.60468	0.102823	3637735
C7_00300W_A	-1.60551	0.063465	3638046
C1_06800W_A	-1.60566	0.112341	3639496
C3_04750W_A	-1.60659	0.180318	3635252
C3_03110W_A	-1.6068	0.172046	3638400
CPR6	-1.60706	0.046333	3639020
C6_01980C_A	-1.60708	0.081175	3644560
PRP39	-1.6079	0.290107	3635832
C1_05120W_A	-1.60799	0.076726	3647667
BRF1	-1.60935	0.058812	3646689
ACH1	-1.61021	0.045612	3643773
C6_00640C_A	-1.61054	0.099492	3647134
C1_01680C_A	-1.61066	0.17588	3640198
C2_02730W_A	-1.61212	0.101787	3643035
C3_00130C_A	-1.61215	0.06489	3640593
GLE1	-1.61222	0.097621	3642340
DBP8	-1.61248	0.098583	3646692
SIS1	-1.61299	0.055432	3644841
ADH5	-1.61384	0.116739	3640156
SUI1	-1.61429	0.088653	3647730
UBR1	-1.61686	0.063839	3637871
C3_05060W_A	-1.61737	0.277576	3635241
CR_00370W_A	-1.61742	0.082403	3639684
CFL1	-1.62235	0.248906	3641810
MLS1	-1.62393	0.114779	3634875
NOP14	-1.62575	0.099975	3635266
C6_03220W_A	-1.62689	0.172163	3639179
UTP4	-1.62727	0.078408	3636657
C2_00170C_A	-1.62925	0.064947	3638906
MUP1	-1.62928	0.052048	3642008
C2_04570W_A	-1.63156	0.129979	3645441
C2_09360W_A	-1.6318	0.152068	3644109
C5_00350C_A	-1.63195	0.091405	3641554
CR_09340W_A	-1.63236	0.056573	3641900
BDF1	-1.63354	0.021745	3641592
SSD1	-1.63491	0.02993	3636520
SEH1	-1.63524	0.051656	3642290
DIP5	-1.63566	0.063004	3636798

C2_09680W_A	-1.63637	0.093289	3643791
SNF2	-1.63714	0.085368	3635855
C2_00230W_A	-1.63737	0.087475	3638901
DRS1	-1.63795	0.082081	3639045
MDN1	-1.63965	0.037372	3635614
PFK1	-1.63968	0.0742	3636448
C6_02930W_A	-1.64605	0.034271	3639243
PTP1	-1.64654	0.056357	3642093
C5_03300C_A	-1.64761	0.101857	3646357
C1_01460W_A	-1.64767	0.047985	3640240
CR_03410W_A	-1.64817	0.091098	3645136
MTO1	-1.64832	0.325225	3640707
ECM42	-1.64854	0.13949	3636995
C3_02020W_A	-1.64855	0.046023	3636592
C3_01150C_A	-1.64942	0.097693	3648401
C3_05950W_A	-1.64981	0.222616	3641728
C5_05130C_A	-1.65125	0.227828	3636459
VPS13	-1.6522	0.027208	3646528
XYL2	-1.65232	0.086223	3638961
C1_05420W_A	-1.65431	0.209865	3642493
MCU1	-1.65448	0.248015	3636169
C1_08910C_A	-1.65543	0.276041	3634926
C1_05540C_A	-1.65582	0.058604	3642503
CR_07760W_A	-1.65655	0.278635	30515431
C6_04480C_A	-1.65795	0.05401	3645986
C7_01360C_A	-1.65821	0.306479	3645900
HLJ1	-1.65928	0.091341	3643158
GCN1	-1.65973	0.06282	3641256
IPK2	-1.65993	0.085028	3643784
IHD1	-1.66071	0.111137	3640291
C5_03030W_A	-1.66151	0.126602	3637822
C1_06030C_A	-1.6625	0.377787	3639867
C2_01820C_A	-1.66327	0.115506	3635831
UGA1	-1.66447	0.133371	3637329
ALS9	-1.66509	0.086884	3640281
FBP1	-1.66616	0.100481	3647464
CR_10180W_A	-1.66624	0.400041	3638917
SGE1	-1.66835	0.118833	3642614
PPT1	-1.66877	0.120871	3636602
CR_03730C_A	-1.67045	0.05114	3643226
C3_04840C_A	-1.67077	0.125198	3635224
SOD3	-1.67095	0.132887	30515347
C1_13950C_A	-1.67255	0.442522	3636186
C5_04910W_A	-1.67357	0.242061	3636417
C2_09510C_A	-1.67667	0.023577	3643816
C7_03730C_A	-1.6769	0.076215	3647867
CR_02270C_A	-1.67941	0.21006	3644911

C3_01560W_A	-1.68072	0.045525	3636669
CHT1	-1.68152	0.206895	3639704
CR_05190W_A	-1.68304	0.640555	3648079
MTR10	-1.68387	0.061169	3647235
NAG4	-1.684	0.511684	3645978
FRP5	-1.68411	0.517995	3641600
FLU1	-1.68436	0.08949	3637048
IAH1	-1.68468	0.071251	3638945
C2_04120C_A	-1.68534	0.041358	3637377
CR_09480W_A	-1.68591	0.065064	3641939
C1_07640C_A	-1.68626	0.205783	3646659
TSR1	-1.68684	0.044765	3642135
C3_03740W_A	-1.68738	0.221609	3645640
FCA1	-1.68814	0.070133	30515327
GRX3	-1.68862	0.03609	3637858
C7_01510W_A	-1.69028	0.034644	3637049
PSD1	-1.69035	0.031418	3639382
C2_00700W_A	-1.69215	0.055867	3638884
BBC1	-1.69222	0.034441	3646661
C5_01490C_A	-1.69384	0.031896	3647058
C3_00010C_A	-1.69441	0.119316	3640600
C6_02150C_A	-1.69637	0.149964	3645888
C1_06410W_A	-1.69709	0.15092	3639555
C1_00380C_A	-1.69752	0.054875	3639292
C5_05360C_A	-1.69765	0.035581	3636402
RPL38	-1.69807	0.026837	30515082
RSP5	-1.69981	0.019781	3643898
C4_01280C_A	-1.70125	0.061574	3635700
C4_01710C_A	-1.70146	0.024444	3635708
C1_04120C_A	-1.70318	0.188243	3644701
C7_00160C_A	-1.70346	0.044959	3637972
RPF2	-1.70388	0.059403	3643467
C5_00820W_A	-1.70396	0.089235	3644128
UBI4	-1.70396	0.05756	3638432
C1_06650W_A	-1.70458	0.086667	3639543
RRP8	-1.70647	0.059745	3643900
STI1	-1.70717	0.01069	3643758
CR_04370W_A	-1.70732	0.122051	3641004
NHP2	-1.70783	0.038078	3641003
C4_03410W_A	-1.70816	0.066284	3646807
HOL1	-1.70817	0.260387	3636838
C1_06540C_A	-1.71162	0.061366	3639520
C2_09670C_A	-1.71217	0.040075	3643792
PET127	-1.71333	0.148956	3644417
C3_00990C_A	-1.71338	0.59236	3639856
C3_02660W_A	-1.71338	0.592118	3638404
C3_04190W_A	-1.71338	0.59188	30515203

C4_05040W_A	-1.71339	0.59273	30515275
C4_03370C_A	-1.71652	0.214375	3646796
FPG1	-1.71685	0.037857	3641632
C2_01680C_A	-1.71735	0.032685	3635744
CR_02960W_A	-1.72314	0.182256	3642591
MED15	-1.72483	0.028546	3640761
CR_00090C_A	-1.72592	0.078451	3639734
C3_03470W_A	-1.72879	0.313088	3638306
VPS24	-1.72948	0.04582	3638892
HTS1	-1.73017	0.01445	3636532
SAP10	-1.73039	0.045812	3641126
RAX1	-1.73218	0.03878	3635256
C4_05330C_A	-1.73235	0.151229	3646959
C2_06780C_A	-1.7351	0.109889	3643516
C1_00100C_A	-1.73564	0.085288	3639325
C1_03310W_A	-1.73594	0.056961	3636856
MTG1	-1.73615	0.171444	3645139
CR_02890C_A	-1.73716	0.066034	3642585
CR_05550C_A	-1.73746	0.034367	3647593
UTP5	-1.73776	0.038553	3638922
SPB4	-1.73926	0.081549	3647252
C1_03270W_A	-1.74067	0.055771	3636900
MSW1	-1.74087	0.102561	3637743
C2_00220C_A	-1.74088	0.031441	3638902
C3_04700W_A	-1.7416	0.101347	3635247
C2_00600C_A	-1.74324	0.020516	3638850
GDT1	-1.74337	0.054049	3645445
RIM15	-1.74396	0.041339	3637961
C2_05820W_A	-1.74409	0.032765	3647482
CNT	-1.74497	0.011824	3644071
C7_03230C_A	-1.74519	0.34026	3641369
NAG3	-1.74554	0.125744	3645969
CR_09580C_A	-1.74621	0.054271	3647097
C2_10070W_A	-1.74764	0.615469	3643946
C5_03520W_A	-1.74764	0.614871	3646970
C1_04370C_A	-1.74797	0.034294	3647994
PEA2	-1.74882	0.12176	3647673
CCW14	-1.75153	0.169551	3642650
C3_07390C_A	-1.75421	0.063324	3638472
GCD7	-1.75446	0.040575	3637367
C1_03050W_A	-1.755	0.023262	3636843
RIM9	-1.75519	0.100721	3644047
MAS1	-1.75619	0.051044	3636855
C2_05550W_A	-1.75658	0.125163	3645560
MUB1	-1.75689	0.026029	3642246
PPG1	-1.75887	0.085164	3641143
C2_02520W_A	-1.759	0.058258	3635818

C3_04330C_A	-1.75918	0.073444	3635272
PRN3	-1.76056	0.176555	3639878
YCF1	-1.76171	0.025153	3636934
NIK1	-1.76192	0.036414	3641358
C2_07720C_A	-1.76224	0.099753	3642304
C1_06910C_A	-1.76424	0.023815	3639526
CR_03110W_A	-1.76508	0.107032	3645124
C1_13560W_A	-1.76513	0.085265	3636174
TFB3	-1.76572	0.076735	3644115
C2_01370C_A	-1.76613	0.0604	3635723
C3_05160C_A	-1.76637	0.031075	3635229
BEM1	-1.76806	0.035489	3635627
C7_01570C_A	-1.76949	0.339915	3637040
C7_01720W_A	-1.76998	0.034402	3637011
C2_00940W_A	-1.77061	0.046854	3638891
C4_07060W_A	-1.77072	0.018944	3642908
STE4	-1.77222	0.142663	3637358
CAN2	-1.77433	0.050704	3644017
C5_00030W_A	-1.77449	0.051605	3647879
HST7	-1.77475	0.024867	3640953
C5_00800C_A	-1.77526	0.015593	3644126
CR_07140C_A	-1.77609	0.067688	3648227
C5_01430C_A	-1.77898	0.059223	3647056
CCT8	-1.77927	0.01479	3639324
IFF3	-1.78113	0.013634	3643189
C1_11770C_A	-1.78139	0.014153	3645327
C1_14550C_A	-1.78466	0.204995	3643334
C2_10850C_A	-1.7854	0.01672	3641232
C4_00030C_A	-1.78642	0.042579	3648055
C3_03440C_A	-1.78724	0.17415	3638356
PRN1	-1.78791	0.089739	3639882
C1_10840C_A	-1.78809	0.024523	3644452
CYS3	-1.79112	0.030534	3642081
C2_09420W_A	-1.79213	0.095175	3647718
URA7	-1.79289	0.024657	3636510
C2_05770W_A	-1.79393	0.049474	3645579
C1_04960C_A	-1.79464	0.049371	3647527
C2_07640W_A	-1.7954	0.016807	3645192
CR_08200C_A	-1.79633	0.023724	3642087
MBF1	-1.7969	0.025521	3640171
SYG1	-1.79692	0.074561	3644777
C2_04750W_A	-1.79734	0.092116	3647785
CYR1	-1.79748	0.029655	3641407
MST1	-1.79777	0.08279	3636831
HEM1	-1.79872	0.025517	3640149
C6_01380C_A	-1.79914	0.145	3641606
SNP3	-1.80088	0.199949	3640991

C3_06290W_A	-1.80237	0.021554	3642237
C5_03560W_A	-1.80598	0.046841	3646975
POL93	-1.80713	0.031059	3639299
C5_05000C_A	-1.80789	0.065408	3636539
C3_07680W_A	-1.80865	0.011246	3638459
C7_03760W_A	-1.80891	0.079611	3647865
YFH1	-1.80901	0.080348	3646853
C1_10420C_A	-1.80969	0.040125	3647178
C1_05220C_A	-1.80988	0.402602	3642464
C4_02680C_A	-1.80989	0.398081	3637828
NOG1	-1.81151	0.007086	3641736
C2_09000C_A	-1.81434	0.167059	3645782
EAF3	-1.81448	0.137531	3648318
C3_02350W_A	-1.81654	0.028558	3636618
C7_03040W_A	-1.82131	0.201426	3641379
FET3	-1.82254	0.054321	3647153
MTLA1	-1.8242	0.157741	3643729
C4_00680W_A	-1.82598	0.047871	3645753
MAK21	-1.82685	0.016481	3635147
C4_03780C_A	-1.8279	0.064919	3645353
C1_04360C_A	-1.83011	0.019251	30514985
QDR1	-1.83158	0.047813	3640941
CR_02700W_A	-1.83431	0.275043	3642575
C2_06160W_A	-1.83589	0.316027	3644079
SKP1	-1.83695	0.018317	3644485
RCL1	-1.83902	0.01776	3645199
CWT1	-1.83993	0.014408	3647595
OPT1	-1.8401	0.181693	3640150
C5_03570W_A	-1.84034	0.049195	3646976
C1_09950C_A	-1.84057	0.036245	3634861
DCP2	-1.84417	0.005219	3642069
C1_04040C_A	-1.84591	0.020203	3646429
C1_00570C_A	-1.8462	0.033108	3639362
C6_04470C_A	-1.84687	0.014847	3645985
C4_04990C_A	-1.84793	0.24177	3641166
CR_10410C_A	-1.85233	0.014726	3638957
SNX4	-1.85259	0.019953	3638843
C6_02350C_A	-1.85459	0.042139	3645881
ZCF21	-1.85472	0.023904	3645762
ATO6	-1.85528	0.130957	3643664
CR_00220W_A	-1.85737	0.080773	3639699
MBP1	-1.85814	0.007715	3635281
CR_10750C_A	-1.85916	0.030997	3638942
C1_00540C_A	-1.85917	0.033026	3639367
SLN1	-1.8609	0.003624	3640422
CR_09740W_A	-1.86226	0.046301	3639008
KOG1	-1.86311	0.022671	3642488

WOR4	-1.86394	0.022681	3638454
ELF1	-1.86495	0.019445	3641903
VAC7	-1.86513	0.026719	3646849
C2_10540W_A	-1.8653	0.030739	3641200
RPB7	-1.86856	0.074162	3640221
C2_07680W_A	-1.86949	0.045686	3642339
OYE2	-1.86969	0.017537	3641603
BLM3	-1.87005	0.011157	3642310
C1_06660W_A	-1.87037	0.010309	3639542
GAP5	-1.87118	0.016039	3646954
ERG3	-1.87195	0.034634	3644776
CR_03930C_A	-1.87307	0.05774	3640957
C5_02370C_A	-1.87313	0.051549	3637793
C3_03770C_A	-1.87751	0.026995	3645636
PGA18	-1.88197	0.027236	3638394
HNM3	-1.88237	0.117906	3640093
C2_00190C_A	-1.88243	0.007324	3638904
C3_03310C_A	-1.88289	0.119322	3638363
CR_10640W_A	-1.883	0.12061	3639014
C5_04010C_A	-1.88623	0.298964	3647439
PRP22	-1.88665	0.014864	3636404
C7_03890C_A	-1.88668	0.00811	3643108
C1_12570C_A	-1.88709	0.015563	3647604
C2_07740W_A	-1.88994	0.003184	3642302
C3_06880W_A	-1.89031	0.44757	3638539
C1_04800C_A	-1.89042	0.434492	3644789
MIS12	-1.89046	0.020108	3639669
C2_06600W_A	-1.89065	0.009936	3646563
CIC1	-1.89237	0.050131	3644059
UTP15	-1.89396	0.035148	3643906
NEP1	-1.89663	0.057242	3644545
C4_00660W_A	-1.89757	0.027497	3645755
C7_03680W_A	-1.89775	0.127836	3646228
C3_00450C_A	-1.89848	0.025183	3640548
C5_03510C_A	-1.90543	0.034894	3646969
C2_06200C_A	-1.90692	0.069498	3646908
C1_09540W_A	-1.90716	0.03358	3634885
C1_06980C_A	-1.90801	0.018045	3639565
C2_00840W_A	-1.9095	0.083748	3638807
MNN14	-1.91356	0.009051	3643651
C4_05230C_A	-1.91589	0.051954	3646156
C2_06030W_A	-1.91674	0.02406	3644070
DCK2	-1.9179	0.028766	3637384
ALR1	-1.9184	0.032003	3636616
C1_12580W_A	-1.9198	0.01084	3647603
RAD57	-1.92043	0.055908	3642333
C7_02550C_A	-1.92243	0.027852	3637018

SRP54	-1.92263	0.006669	3640377
C2_04700C_A	-1.92481	0.138389	3647794
C2_09870W_A	-1.92526	0.018885	3643780
CLN3	-1.92686	0.021783	3642624
C5_02190C_A	-1.92881	0.045087	30515303
MEF2	-1.92947	0.022753	3639564
C5_05460C_A	-1.93044	0.133736	3636527
C3_00300W_A	-1.93129	0.012603	3640524
C2_02800W_A	-1.93172	0.045022	30515096
C3_06760W_A	-1.93529	0.033492	3638502
C1_02950W_A	-1.93695	0.259311	3636833
CET1	-1.93815	0.006521	3640140
CDC37	-1.93821	0.01182	3639210
NOC4	-1.93906	0.022749	3645224
TPK1	-1.94058	0.016331	3634879
GYP5	-1.94078	0.012791	3641246
DBP2	-1.94155	0.007203	3647692
SUV3	-1.94227	0.057119	3645451
C2_03690C_A	-1.94259	0.029259	3637376
PGA4	-1.9433	0.004739	3636406
HBR1	-1.94434	0.009425	3639295
CR_06840W_A	-1.94479	0.010194	3647322
C1_05450W_A	-1.94531	0.732498	3642490
C2_06350C_A	-1.94531	0.732498	3646919
C4_00640W_A	-1.94531	0.732498	3645767
C4_05580C_A	-1.94531	0.732498	3641775
OPT9	-1.94531	0.732498	3640090
PGA19	-1.94531	0.732498	3638894
C1_03470C_A	-1.94532	0.732496	3636912
C1_05970W_A	-1.94532	0.732496	3639874
C1_09700W_A	-1.94532	0.732496	3634876
C1_10050W_A	-1.94532	0.732496	3634811
C1_11390W_A	-1.94532	0.732496	3644544
C2_00560W_A	-1.94532	0.732496	3638854
C2_06980W_A	-1.94532	0.732496	3643530
C4_02240C_A	-1.94532	0.732496	3635692
C4_03500C_A	-1.94532	0.732496	3646801
CR_02340W_A	-1.94532	0.732496	3644936
DAL52	-1.94532	0.732496	3643736
GIT4	-1.94532	0.732496	3642635
KRE62	-1.94532	0.732496	3641543
MFA1	-1.94532	0.732496	30515153
MRV3	-1.94532	0.732496	3636546
OPT5	-1.94532	0.732496	3640768
PTR2	-1.94532	0.732496	3640089
SPR3	-1.94532	0.732496	3635853
C7_01660C_A	-1.94609	0.019749	3636983

SMX4	-1.94641	0.132703	30515427
C7_02450W_A	-1.94698	0.016924	3636972
C4_02260C_A	-1.94704	0.04283	3635690
GEF2	-1.94707	0.034783	3644935
CDC4	-1.94867	0.003913	3640125
C7_01280C_A	-1.95044	0.05019	3645909
SDH4	-1.95152	0.089734	3636497
C4_03090W_A	-1.95342	0.019832	3637926
VPH2	-1.95479	0.082524	3642021
C1_05250W_A	-1.95525	0.353952	3642462
C6_02460C_A	-1.95585	0.023461	3639168
NUP49	-1.9567	0.005918	3636173
ORM1	-1.95741	0.003612	3640307
EXM2	-1.95837	0.020602	3642929
C1_13430C_A	-1.96096	0.090325	3636223
C5_04990W_A	-1.9613	0.105605	3636538
C3_05400C_A	-1.96412	0.36587	3643681
C4_04350W_A	-1.96432	0.016225	3646852
SCO1	-1.9652	0.009266	3641922
RVS161	-1.96659	0.003495	3638005
DAL9	-1.96805	0.070621	3645653
C2_01310W_A	-1.96906	0.02339	3638788
JIP5	-1.97228	0.019321	3634922
C4_04200C_A	-1.97244	0.331527	3641182
CR_00600C_A	-1.97534	0.030224	3639660
C4_04670C_A	-1.97823	0.022824	3641140
RGD3	-1.97836	0.003222	3648003
RNR22	-1.97887	0.001119	3645217
C2_04280W_A	-1.98616	0.018534	3637326
C4_07100C_A	-1.98769	0.005445	3642903
C1_11110C_A	-1.98968	0.088572	3644413
GRR1	-1.99027	0.002613	3636392
GSY1	-1.99065	0.008764	3640435
C1_02940C_A	-1.99109	0.022304	3636832
C3_07800C_A	-1.99345	0.020298	3647461
C7_03590C_A	-1.99744	0.024003	3646239
PEL1	-1.99773	0.009494	3637989
HMS1	-1.99926	0.00513	3641570
C4_02920W_A	-2.00009	0.004372	3637878
SAM35	-2.00146	0.018871	3643329
SDH1	-2.0018	0.011435	3642477
CTR1	-2.00518	0.044472	3647077
RIO2	-2.00792	0.014106	3642066
SAP2	-2.0082	0.029739	3647354
GAL1	-2.01729	0.000534	3644581
AMS1	-2.01752	0.013549	3637882
CR_07030C_A	-2.01967	0.040029	3645730

C3_07430W_A	-2.02076	0.001877	3638484
RIB1	-2.02192	0.004368	3642607
C3_03920W_A	-2.02479	0.033851	30515197
C1_06070W_A	-2.02561	0.010271	3639824
CR_07320C_A	-2.02562	0.015208	3646057
HEX3	-2.02942	0.008881	3635776
MTR2	-2.03087	0.011689	30515167
CR_07750C_A	-2.03196	0.020508	3647350
UTP8	-2.03306	0.003562	3640547
MOB2	-2.03373	0.021518	3639381
CR_03120W_A	-2.03423	0.005183	3645123
CR_00160C_A	-2.03548	0.015421	3639706
C4_02850W_A	-2.0362	0.008881	3637910
C3_07290W_A	-2.03682	0.008322	3638430
RRP9	-2.03701	0.041742	3642576
CTN3	-2.0448	0.024313	3647396
GIR2	-2.04551	0.009709	3636962
C3_03670W_A	-2.05078	0.004268	3645649
C4_06990W_A	-2.05111	0.018688	3642968
MTG2	-2.05458	0.017971	3646693
C2_04820W_A	-2.0551	0.015103	3647776
CR_03200C_A	-2.05778	0.009494	3645131
CR_05440W_A	-2.05881	0.011931	3648245
PNG2	-2.05951	0.00786	3645568
CR_03740C_A	-2.06006	0.027627	3643176
MLP1	-2.06073	0.003988	3635688
C1_12610W_A	-2.06177	0.012744	3646825
CR_02670C_A	-2.06208	0.010601	3642572
HAL9	-2.06481	0.003218	3643757
RPM2	-2.06499	0.010294	3644790
PWP2	-2.07361	0.005397	3640416
MIG1	-2.08122	0.002007	3637812
SIZ1	-2.08195	0.002607	3640219
C2_02770W_A	-2.08419	0.00033	3643031
ENP2	-2.08422	0.009624	3646234
RIT1	-2.09032	0.029793	3638492
YAK1	-2.09171	0.002035	3647801
C4_01500W_A	-2.09264	0.020784	3635617
C6_03290W_A	-2.09436	0.002039	3639195
C7_01990C_A	-2.0957	0.014899	3636966
ESA1	-2.096	0.009149	3640518
C5_04050W_A	-2.09644	0.000276	3647735
FGR15	-2.09789	0.010335	3638882
C1_09340C_A	-2.09804	0.009429	3634804
C7_03480W_A	-2.10003	0.007868	3646230
C1_00520W_A	-2.10003	0.009917	3639310
CR_07190W_A	-2.10168	0.001342	3645269

C6_04290W_A	-2.10378	0.001346	3646280
C3_00570C_A	-2.10419	0.005207	3640584
C3_04920C_A	-2.10475	0.00955	3635268
HGT6	-2.10629	0.006752	3638873
CEF1	-2.11817	0.003831	3634808
C3_07700W_A	-2.11836	0.006181	3638457
CR_08400C_A	-2.11924	0.007754	3642115
RAX2	-2.12042	0.004186	3647610
C2_07760W_A	-2.12104	0.006854	3642298
C1_09040C_A	-2.12201	0.029707	3634769
YKE2	-2.1226	0.009707	30515454
LEU3	-2.12312	0.005751	3637757
C6_02330W_A	-2.12417	0.001843	3645877
C2_01240C_A	-2.12519	0.047836	3638794
C2_08330W_A	-2.12562	0.019962	3648166
C1_12680W_A	-2.12737	0.003349	3646833
C2_07020C_A	-2.12759	0.029922	3643497
C3_00100W_A	-2.12838	0.010427	3640567
C1_06440C_A	-2.13191	0.0193	3639552
CTF1	-2.13379	0.017067	3635788
HCH1	-2.13514	0.018791	3641626
C1_01850C_A	-2.13624	0.008013	3645431
C5_01050C_A	-2.13707	0.023861	3642648
CR_04350C_A	-2.13955	0.003094	3641001
SPL1	-2.14479	0.001205	3638035
PRS5	-2.14504	0.006646	3642296
HSE1	-2.1487	0.002716	3640407
ZCF9	-2.14931	0.00502	3638869
ZCF20	-2.15088	0.007906	3647059
C1_11900C_A	-2.15107	0.018773	3642005
FGR3	-2.15148	0.003241	3641179
HCA4	-2.15186	0.009478	3637911
EST1	-2.15283	0.00089	3636526
C1_11690W_A	-2.15283	0.006974	3645322
C2_09240C_A	-2.15652	0.04664	3644064
C1_14250C_A	-2.15817	0.005088	3643307
C5_04180W_A	-2.15859	0.036436	3636544
C6_00360C_A	-2.16288	0.026752	3646083
POP2	-2.16404	0.003451	3640301
C1_12950W_A	-2.1642	0.005967	3636157
CST5	-2.16541	0.022173	3645981
MAK5	-2.16761	0.007179	3643454
RTG1	-2.16931	0.004971	3634929
C4_03740W_A	-2.16947	0.00674	3645357
C6_04450W_A	-2.17034	0.017483	3645982
ADE8	-2.17178	0.008069	3642999
C7_03280C_A	-2.1798	0.004701	3641353

C2_06740W_A	-2.18026	0.001162	3648024
NAB3	-2.1807	0.001628	3639209
CR_02950C_A	-2.18821	0.013313	3642590
CR_03080C_A	-2.18908	0.047119	3645104
C2_01190C_A	-2.19033	0.049652	3638859
CR_05770W_A	-2.1909	0.013395	3646694
ARD	-2.19159	0.003651	3647906
DBP7	-2.19217	0.001369	3645903
PDR16	-2.19417	0.005291	3644679
SUI3	-2.19479	0.000656	3643130
IFF9	-2.19967	0.001647	3641008
SSK1	-2.20087	0.002595	3636184
NIP7	-2.20276	0.009065	3645880
C7_01490W_A	-2.20773	0.014801	3637051
C2_02120W_A	-2.20779	0.006533	3635746
RER2	-2.2078	0.005428	3636399
MRT4	-2.20963	0.008314	3639219
OYE32	-2.2115	0.001674	3642919
C5_05140W_A	-2.21466	0.014659	3636460
C3_02180C_A	-2.2147	0.005711	3636643
CR_09440C_A	-2.21784	0.001395	3641937
TBF1	-2.21911	0.015202	3637327
C1_11290W_A	-2.22037	0.014048	3644553
C2_00360C_A	-2.2223	0.000211	3638825
CKA1	-2.22336	0.002047	3639018
C3_05360C_A	-2.22348	0.00155	3643676
CR_01440C_A	-2.22455	0.000456	30515374
C2_05670C_A	-2.2262	0.042728	3645570
C6_00310W_A	-2.23037	0.000891	3646071
C5_04290C_A	-2.23214	0.003295	3636455
ISW2	-2.2411	0.012025	3642235
TRY2	-2.24224	0.005361	3647670
VPS28	-2.24344	0.00684	3645794
C7_03210W_A	-2.24354	0.018008	3641371
CR_02800C_A	-2.24394	0.002461	3642559
BGL22	-2.24645	0.018249	3641935
C2_04080W_A	-2.24871	0.003054	3637381
BLP1	-2.24891	0.003337	30515065
C3_06740W_A	-2.24906	0.013879	3638503
C2_02200W_A	-2.25374	0.011481	3635772
C4_05440C_A	-2.25475	0.001368	3646960
CPP1	-2.25682	0.001381	3634865
C1_04220C_A	-2.25911	0.011658	3644721
PHHB	-2.25934	0.006665	3638871
ENP1	-2.26011	0.011746	3647861
C6_01350W_A	-2.26358	0.003422	3641642
C1_10080W_A	-2.26401	0.037259	3634814

CR_07670W_A	-2.26786	0.007044	3647255
CR_10800C_A	-2.27083	0.001821	3638948
C6_04410C_A	-2.27106	0.014084	3645950
C1_00460W_A	-2.27192	0.014246	3639316
C7_02930C_A	-2.27554	0.001744	3641390
DUS4	-2.27576	0.002173	3641580
C2_04770W_A	-2.27688	0.01511	3647783
C1_09390W_A	-2.27715	0.011499	3634851
RTG3	-2.27725	0.001801	3644423
MPP10	-2.27847	0.000434	3647912
C3_07940W_A	-2.27915	0.001228	3647453
C2_03550C_A	-2.28238	0.000555	3637338
C1_03120W_A	-2.2846	0.000607	3636926
C7_00200W_A	-2.28465	0.002342	3637968
C3_02670W_A	-2.28787	0.046178	3638405
FGR13	-2.28887	0.024971	3648078
C1_01160C_A	-2.28897	0.00443	3640204
SSF1	-2.29126	0.001697	3647085
C2_06480W_A	-2.29313	0.003309	3646568
SET2	-2.29406	0.004727	3643923
C1_10970W_A	-2.29496	0.007555	3644427
C1_09840C_A	-2.29522	0.000422	3634848
VPS27	-2.29805	0.000385	3639326
C2_02960C_A	-2.29869	0.001603	3643011
C6_00550W_A	-2.30046	0.003977	3647144
FRE3	-2.30233	0.041572	3641789
C7_01700W_A	-2.30252	0.000773	3637013
C1_05200C_A	-2.30326	0.005498	3642466
C2_02630W_A	-2.30402	0.013259	3635839
GPD1	-2.305	0.000143	3643924
C4_06850C_A	-2.30542	0.001196	3642912
C1_03040W_A	-2.30874	0.026166	3636842
PDE2	-2.30917	0.001028	3636877
LIG1	-2.31392	0.000841	3636947
C2_05270W_A	-2.31431	0.004351	3643470
THI20	-2.3157	0.030494	3637387
C3_00500C_A	-2.31654	0.009925	3640515
C2_05990C_A	-2.3183	0.07747	3644074
PSP1	-2.31844	0.000482	3644551
SWE1	-2.32587	2.18E-05	3634866
SEF1	-2.32839	0.002669	3644945
RAD1	-2.33042	0.000714	3641192
SPO7	-2.33081	0.008808	3645576
NAN1	-2.33257	0.006812	3637932
C3_00840C_A	-2.33604	0.048344	3647758
INN1	-2.33713	2.66E-05	3642578
C6_02650C_A	-2.34299	0.00285	3639223

ZCF35	-2.34853	0.004352	3641740
C3_03190C_A	-2.34877	0.001859	3638427
C7_04090C_A	-2.35065	0.000324	3643135
C4_03580W_A	-2.35118	0.039932	3645369
ADAEC	-2.35756	0.000795	3637342
C1_06820W_A	-2.35763	0.002484	3639493
LEU5	-2.36163	0.000528	3638838
NDT80	-2.36418	3.52E-05	3638840
C2_05980C_A	-2.36737	0.02583	3644075
C2_10600W_A	-2.36868	0.003201	3641245
C2_08180C_A	-2.37313	0.005576	3642317
PPR1	-2.3735	0.004641	3636535
DOT4	-2.37361	0.001652	3646810
C6_03210C_A	-2.37572	0.007461	3639178
C7_03850W_A	-2.37713	0.01869	3643145
CR_00820C_A	-2.37757	0.007076	3640413
SRP40	-2.37866	0.000751	3642605
C1_08710W_A	-2.38029	0.008896	3634937
C6_03780C_A	-2.38064	0.000623	3640308
C6_02380W_A	-2.38861	0.001473	3645883
CR_01090W_A	-2.38925	0.000393	3640379
SBA1	-2.39071	0.000802	3640305
CR_04110W_A	-2.39248	0.001772	3641026
NMD3	-2.39741	0.000807	3646614
C2_08860W_A	-2.40439	2.24E-05	3645776
CR_03910C_A	-2.40885	0.000698	3640954
C4_05740C_A	-2.40987	0.001286	3641806
ZCF29	-2.41036	0.00075	3641370
CR_08410W_A	-2.41685	0.000973	3642116
C1_10890C_A	-2.41996	0.000406	3644447
UTP18	-2.42494	0.002765	3643123
C1_09710C_A	-2.42552	0.003168	3634818
SSN6	-2.42597	0.000371	3638518
FAL1	-2.42615	0.001967	3639839
PCL5	-2.42855	0.001642	3636466
DOT6	-2.43703	0.000753	3640078
C2_02710C_A	-2.43759	0.003134	3643003
MRF1	-2.44123	0.000547	3645321
C2_10050W_A	-2.4459	2.33E-05	3643949
CR_10420W_A	-2.44601	0.000198	3638974
BNA4	-2.45061	0.000289	3640528
C4_02170C_A	-2.45072	0.038824	3635667
C3_07900C_A	-2.4525	0.004213	3647450
C5_00160W_A	-2.45277	0.004864	3647871
CAN3	-2.45307	0.001309	3647066
MRPL40	-2.45467	0.002052	3640968
C1_12860C_A	-2.45657	0.0237	3636179

C1_02960C_A	-2.4578	0.000159	3636834
C3_00630W_A	-2.45822	0.000138	3640576
PTK2	-2.46278	0.000303	3641611
DYN1	-2.46746	0.000869	3635236
CR_09460C_A	-2.46763	0.00064	30515453
C1_08350C_A	-2.46893	0.000815	30515030
CR_06330C_A	-2.47056	0.004698	3644846
DBP3	-2.47187	0.00083	3634868
C2_00810C_A	-2.47206	0.018563	3638810
C4_00750C_A	-2.4722	0.031368	3645763
MEC3	-2.4766	0.040264	3646910
CR_07690W_A	-2.4767	0.039295	3648194
SGS1	-2.48049	0.007256	3641258
NSA2	-2.48277	0.000367	3642259
TRM12	-2.48362	0.014216	3641738
C2_00770W_A	-2.48665	0.006802	3638909
C4_00820W_A	-2.48722	0.025606	3645741
C2_01250W_A	-2.49345	0.015592	3638793
C5_00780C_A	-2.49394	0.002574	3644124
MUC1	-2.49475	0.000743	3645771
C3_04940W_A	-2.4952	0.000237	3635270
C4_07010C_A	-2.49897	0.029184	3642966
C4_06620C_A	-2.50264	0.01939	3642813
C4_02570C_A	-2.50309	0.006828	3637916
C7_04290W_A	-2.50314	0.02173	3643172
C1_06320W_A	-2.50784	0.048271	3648118
PGA55	-2.50965	0.001383	3645784
CR_08190W_A	-2.51012	0.047418	3642084
YHB4	-2.51049	0.005757	3638999
C3_05460W_A	-2.51124	0.008122	3643670
C4_01930C_A	-2.513	0.015807	3635634
C7_01040C_A	-2.51643	0.003879	3638018
C3_02790W_A	-2.51708	0.003319	3638416
SPT5	-2.52029	9.58E-05	3635847
PIR1	-2.52094	0.001697	3645801
HSX11	-2.5269	0.031858	3635660
C2_00490W_A	-2.52705	9.12E-05	3638870
CR_06290C_A	-2.52873	0.000715	3644856
C4_02110W_A	-2.52995	0.004349	3635673
C4_00490W_A	-2.53292	0.001758	3645013
CR_08040W_A	-2.53507	0.000995	3644135
KNS1	-2.53546	0.00095	3636228
C7_03970C_A	-2.536	0.003885	3643117
CR_03710C_A	-2.53844	0.000305	3643224
CR_05640C_A	-2.54166	0.025215	3646690
C2_09160W_A	-2.54248	0.0154	3644090
URK1	-2.54556	0.00026	3635186

SFP1	-2.54917	0.000517	3635225
C3_06370C_A	-2.54923	0.000605	3642258
RPO41	-2.54935	0.000154	3639378
SOF1	-2.55039	0.00012	3640585
C7_03000C_A	-2.55256	0.001944	3641383
CR_07910C_A	-2.55393	9.41E-05	3644142
HAP43	-2.55776	9.19E-05	3644531
C5_02280C_A	-2.55778	0.00022	3637712
SGD1	-2.56005	0.002145	3643191
C2_06800C_A	-2.5611	0.001691	3643518
C3_07580W_A	-2.56159	0.001076	30515225
MSN4	-2.56289	8.09E-05	3634911
EFG1	-2.56913	7.95E-05	3644145
C1_07950C_A	-2.57081	0.007728	3640724
C6_02230W_A	-2.57274	0.000362	3645866
CR_07920W_A	-2.57734	0.000124	3644141
C1_10880W_A	-2.57749	0.001182	3644449
MNN4	-2.57755	9.69E-05	3642784
NOP8	-2.57905	0.007059	3646290
C3_07920W_A	-2.58093	0.000101	30515226
C6_02720C_A	-2.58152	0.00015	3639212
AOX2	-2.58843	0.046947	3634945
C3_00160C_A	-2.5894	0.000265	3640591
HAP41	-2.59354	0.000842	3647405
SLR1	-2.59827	7.1E-05	3643957
KRE1	-2.59904	0.000564	3643180
C7_02120C_A	-2.60029	0.010425	3637019
ARG5,6	-2.60201	0.000118	3634830
C1_09850C_A	-2.60592	8.97E-05	3634849
CR_02420W_A	-2.60602	0.000993	3644926
C1_00370W_A	-2.61125	0.028532	30514954
CNH1	-2.61147	8.38E-05	3648056
C1_09740C_A	-2.61551	0.086398	30515035
SOD6	-2.61708	0.000301	3638899
C6_04550C_A	-2.61766	0.000335	3645976
C1_01580W_A	-2.61825	0.000766	3640222
ARG4	-2.61877	0.000325	3646237
C5_05340W_A	-2.61977	7.42E-05	3636400
TBP1	-2.62127	0.000196	3647675
TEC1	-2.62198	6.01E-05	3635261
C3_04790W_A	-2.62269	0.001712	3635215
PTR3	-2.62285	0.002606	3645428
C2_05830C_A	-2.63032	0.002469	3647483
FTH2	-2.63129	0.003855	3640401
LEU42	-2.6323	6.23E-05	3643808
ARO10	-2.63658	0.005766	3647320
NAR1	-2.63979	0.000269	3634916

C1_13530W_A	-2.64377	0.000591	3636170
SST2	-2.64455	0.015311	3637754
ATX1	-2.64575	0.009372	30515426
C1_12370W_A	-2.64662	0.00976	3642061
C5_02810W_A	-2.64727	0.012893	30515304
CDC34	-2.64966	2.5E-05	3637028
C3_00940W_A	-2.65089	7.03E-05	3639851
FHL1	-2.65114	0.00018	3643493
C4_02330C_A	-2.65209	0.02701	3637884
CR_02510W_A	-2.66362	6.67E-05	3647694
C5_02580W_A	-2.6702	0.000436	3637721
ERO1	-2.67235	4.08E-05	3634810
YAH1	-2.67437	0.0029	3638368
GUT1	-2.67692	0.000815	3648082
C3_04260W_A	-2.68284	0.000328	3635140
SVF1	-2.68349	0.000161	3639289
LSM6	-2.68374	0.004645	30515363
C5_02510C_A	-2.68482	0.000547	3637767
C5_01290C_A	-2.68571	0.00041	3642647
CR_05730C_A	-2.68576	3.23E-05	3646698
CR_10810C_A	-2.68817	0.00017	3638949
C4_04810C_A	-2.6905	0.00043	3641112
CR_05910W_A	-2.69479	0.002675	3646680
C4_02510W_A	-2.69974	0.000975	3637865
AAH1	-2.70179	0.011057	3643529
C7_00040C_A	-2.70268	0.007743	3638002
TRY3	-2.70399	2.6E-05	3642652
C3_02710W_A	-2.70597	0.000309	30515190
C3_05860C_A	-2.70664	0.002297	3641759
IRE1	-2.70701	8.75E-05	3640726
FCR1	-2.71613	0.000241	3638542
ATG15	-2.71792	6.59E-05	3641641
CR_00430C_A	-2.71877	0.012576	30515366
C3_07490W_A	-2.7218	1.94E-05	3638528
C3_06270C_A	-2.72213	0.001325	3642239
DAL1	-2.72464	0.004929	3640564
SSU72	-2.72488	0.000394	3645129
RMS1	-2.73442	0.000872	3646361
C6_01040C_A	-2.73696	0.000327	3644014
CKA2	-2.74039	0.000111	3643444
C7_01070C_A	-2.74153	2.69E-05	3637949
AUR1	-2.74384	4.28E-05	3642617
C4_00610W_A	-2.74597	0.000848	3645770
C2_09500W_A	-2.74922	8.1E-05	3643817
FRP6	-2.75105	4.03E-05	3641601
C2_02820C_A	-2.75109	0.000416	3643027
C4_07210W_A	-2.75143	0.001584	3642959

C2_07580W_A	-2.75512	0.01763	3645216
C5_00310C_A	-2.7601	0.000261	3641576
IFM3	-2.76148	0.009523	3642335
C5_03740W_A	-2.76373	0.00016	3647237
C2_03000C_A	-2.76731	0.00157	3643022
CR_00460C_A	-2.76899	0.000738	3639726
C4_06790W_A	-2.76907	0.000536	3642918
HNM1	-2.76916	7.06E-06	3638857
PMC1	-2.77615	1.33E-05	3636627
MSS116	-2.77634	0.000398	3634792
TOK1	-2.78042	9.17E-05	3645754
C1_13880C_A	-2.78602	8.45E-07	3636133
SIR2	-2.78642	0.00148	3638845
C2_04850C_A	-2.78679	5.12E-05	3648114
MNL1	-2.79056	1.78E-06	3646050
C2_00410C_A	-2.79095	0.000218	3638820
TPT1	-2.79147	0.000916	3640543
C1_01890C_A	-2.79545	5.35E-05	3645427
CR_06060W_A	-2.79572	0.001881	6335358
GCS1	-2.79644	4.14E-05	3640742
C7_01150W_A	-2.79749	0.001188	3645913
CR_05540C_A	-2.80109	4.55E-05	3647594
CPH2	-2.80962	2.41E-05	3646067
C4_00460C_A	-2.8128	1.95E-05	3645010
C1_13380W_A	-2.81395	0.011673	3636217
C1_01140C_A	-2.81526	2.24E-05	3640202
C2_03150C_A	-2.81559	0.010677	3642990
C2_04520C_A	-2.81929	0.018258	3645447
C1_00980W_A	-2.82023	0.007852	30514959
NOP13	-2.82448	3.2E-05	3638469
C1_07130C_A	-2.82623	6.5E-05	3644462
C1_10360C_A	-2.82693	2.97E-07	3634836
UGT51C1	-2.82849	4.98E-06	3640147
NOT5	-2.82971	9.74E-06	3640763
MOH1	-2.83242	0.003251	3646809
RPA34	-2.83943	0.000829	3634883
CR_02970C_A	-2.84114	1.73E-05	3642603
CR_06980W_A	-2.84769	0.015742	3645735
C2_06620W_A	-2.85467	0.015133	3646564
C7_03270W_A	-2.85663	0.001507	3641365
CR_07830C_A	-2.8569	1.99E-05	3647345
DIP2	-2.86413	0.00025	3640762
C5_04170W_A	-2.86791	1.32E-05	3636543
MRPL36	-2.86808	0.001009	3643733
FAB1	-2.87221	0.000149	3635766
C2_07060W_A	-2.87258	0.000313	3643500
BUD21	-2.87549	9.87E-05	3640541

C6_04300W_A	-2.8817	0.000329	3646286
IML1	-2.88447	1.29E-05	3636813
C2_06510W_A	-2.88462	0.000145	3646570
C1_10250C_A	-2.88635	0.018362	3634882
STF2	-2.88676	0.000223	30515083
C1_07660W_A	-2.8917	0.002779	3646656
PSO2	-2.89606	0.006833	3641241
KTI12	-2.89738	0.000114	3645140
ATO2	-2.90277	0.001358	3639845
STD1	-2.90343	5.23E-05	3647459
BUD22	-2.90367	0.000215	3640391
C4_03190W_A	-2.90524	0.000145	3637838
C6_04140C_A	-2.90917	1.44E-05	3647970
FMT1	-2.91017	0.000306	3646530
FZO1	-2.9118	9.03E-06	3646848
C3_03300C_A	-2.91291	1.29E-05	3638361
C1_02850W_A	-2.91769	0.000303	3636862
C4_07220C_A	-2.91914	5.07E-05	3642955
PUT2	-2.92312	1.28E-05	3636439
PEP12	-2.92358	0.000133	3637737
CAP1	-2.92427	2.64E-05	3636640
C5_03410C_A	-2.93605	0.007711	3646347
C4_03460C_A	-2.93783	0.040435	3646813
KRE6	-2.93959	0.00024	3641756
IFH1	-2.94086	0.000534	3637770
C2_03500W_A	-2.9495	6.84E-08	3637345
VPS21	-2.94998	2.56E-06	3642092
C3_06600C_A	-2.96565	0.000927	3642280
HST2	-2.97174	0.003963	3640162
PHO4	-2.97845	9.4E-05	3641817
CUP2	-2.98735	7.73E-06	3636189
LAS1	-2.98928	5.09E-05	3644073
C4_01420W_A	-2.99582	0.001492	3635626
C1_03750W_A	-2.99632	0.000109	3644690
BOI2	-2.99879	0.000256	3640404
NCS2	-2.99885	4.28E-07	3643216
MNN4-4	-3.00077	0.005213	3639250
C6_04050W_A	-3.00715	0.000223	3647830
CR_02930W_A	-3.00773	1.11E-07	3642588
STP2	-3.00846	1.02E-05	3636214
C2_03760C_A	-3.00866	3.63E-07	3637371
C1_14480W_A	-3.00873	0.000731	3643328
MRPL27	-3.0142	4.1E-05	3636884
SHA3	-3.01655	0.00013	3644580
C3_06160C_A	-3.0166	2.82E-05	3641766
C4_04660C_A	-3.01762	7.05E-06	3641150
PTP2	-3.01905	6.35E-07	3647858

NOG2	-3.0493	0.000669	3640299
CR_05150W_A	-3.05157	0.02617	30515403
C2_01510C_A	-3.0516	0.025954	3635801
C2_08850C_A	-3.05286	1.31E-05	3643890
HSP104	-3.05393	4.06E-05	3642097
RRS1	-3.05566	0.000141	3639352
C6_03460W_A	-3.06215	1.07E-08	3640252
CR_10400W_A	-3.06645	2.23E-05	3638956
RAS2	-3.06806	1.62E-05	3635211
ADA2	-3.06948	1E-05	3644450
NOP4	-3.07113	0.000115	3647991
SOK1	-3.08073	8.85E-06	3642495
C1_07160C_A	-3.08159	0.000199	30515020
C2_07040W_A	-3.08394	0.000134	3643499
C3_07550C_A	-3.09277	7.74E-06	3638525
ZSF1	-3.09451	5.82E-05	3641257
C3_04540C_A	-3.09865	6.37E-06	3635262
OSM1	-3.101	3.25E-06	3645573
C2_06660W_A	-3.10144	0.008762	3648418
C7_01210C_A	-3.10241	0.000554	3645904
C5_03920C_A	-3.10805	6.52E-05	3647448
C4_01860C_A	-3.11005	7.52E-06	3635590
C1_04510W_A	-3.11024	0.005035	3646147
BMT9	-3.11072	0.000184	3635654
C3_05440C_A	-3.11211	0.004013	3643668
C1_10230C_A	-3.11237	2.36E-05	3634880
C1_11620W_A	-3.11936	0.010405	3645344
C2_00090W_A	-3.11972	0.002322	3647910
C1_06380C_A	-3.11978	0.007021	3639559
C1_06760C_A	-3.12153	2.12E-05	3639505
PDR6	-3.12179	0.000184	3638824
CDG1	-3.13025	0.000202	3641883
MSB2	-3.1323	3.58E-06	3635830
CR_00750C_A	-3.13565	8.52E-06	3640438
RCA1	-3.13575	2.02E-05	3639303
C2_01540W_A	-3.13665	0.00118	3635804
C1_09330W_A	-3.14306	0.005501	3634802
ZCF27	-3.14686	2.67E-05	3635631
NAG1	-3.14722	0.000248	3645966
C5_00390C_A	-3.14792	1.1E-05	3641552
TCC1	-3.14825	4.46E-06	3638524
ARO80	-3.15083	0.000586	3636808
SFT2	-3.15185	0.001085	3644442
DFG16	-3.15187	1.59E-05	3637413
CNS1	-3.15449	8.24E-05	3639365
C4_04460C_A	-3.16896	8.75E-06	3641127
CR_10470C_A	-3.17171	1.07E-05	3638979

CR_01370C_A	-3.17244	5.55E-05	3640168
PLB3	-3.17365	2.26E-06	3647089
NCR1	-3.18098	3.14E-07	3643314
C1_14580C_A	-3.18409	0.000175	3643290
CCS1	-3.19009	3.99E-07	3644476
ROT1	-3.19181	0.001762	3639350
C7_01610W_A	-3.19549	0.000228	30515353
RRP15	-3.19713	1.29E-05	3648397
RIM2	-3.19726	0.002596	3645448
BUD14	-3.19906	1.1E-07	3643469
CR_03870W_A	-3.19957	0.000974	3643187
C1_11570W_A	-3.20169	0.024444	30515055
C3_06820C_A	-3.20665	3.53E-07	3638544
NBP2	-3.20713	2.24E-05	3636963
CR_10430C_A	-3.21043	0.026425	3638975
C7_00590W_A	-3.21125	7.42E-05	3637994
PNC1	-3.21311	0.000247	3646232
RGS2	-3.22102	4.32E-06	3646629
C5_02440C_A	-3.2298	0.000676	3637728
MET2	-3.23518	1.08E-05	3640070
SAS10	-3.23647	0.000406	3637899
C6_00700C_A	-3.23745	4.81E-05	3647074
C4_04010W_A	-3.24085	0.000453	3641218
C2_00200W_A	-3.24527	1.21E-05	3638903
C6_02970C_A	-3.247	0.000972	3639190
RSM22	-3.25	0.000137	3642506
C5_03960W_A	-3.25121	4.21E-07	3647444
HSP70	-3.25129	3.15E-05	3636229
C2_05060C_A	-3.25456	0.004758	3643451
BAS1	-3.25774	1.68E-06	3641136
C6_03520C_A	-3.26754	0.034464	3640259
C2_00280C_A	-3.26837	9.7E-05	3638802
C1_07560W_A	-3.26851	0.000108	3646676
C1_06470W_A	-3.30131	5.31E-07	30515007
CR_00040C_A	-3.30205	4.13E-06	3639675
C6_03370W_A	-3.31536	1.63E-05	3639230
C1_05270C_A	-3.31712	7.43E-05	3642476
C7_02160W_A	-3.31777	0.000682	3636994
UPC2	-3.3193	5.61E-05	3646489
C6_00290W_A	-3.3215	6.61E-06	3646069
NBN1	-3.32208	1.89E-05	3637410
C1_10140C_A	-3.32771	1.04E-05	3634780
SHY1	-3.33782	1.36E-05	3634824
OPT7	-3.33949	0.002156	3645008
C6_04240W_A	-3.34029	0.002248	3646276
RPF1	-3.34144	1.27E-06	3637890
C2_10170C_A	-3.34284	1.17E-06	3643931

C4_05590W_A	-3.34723	5.1E-06	3641776
C1_12150C_A	-3.34942	0.005135	3642049
SDS24	-3.35288	5.96E-06	3640756
PRB1	-3.36239	1.16E-05	3643521
C4_02090C_A	-3.36395	2.85E-06	3635642
TSR2	-3.36546	2.8E-05	3636845
ARG81	-3.37665	2.32E-06	3634775
SMD2	-3.37835	0.011809	30515133
C1_11000C_A	-3.38329	2.51E-05	3644422
C2_10810W_A	-3.40095	8.64E-06	3641243
C3_01090W_A	-3.40323	0.001074	30515176
C1_10060C_A	-3.41261	0.003562	3634812
C1_07220W_A	-3.41362	1.06E-06	3644460
PDX3	-3.4137	6.45E-10	3640949
ELP3	-3.41429	1.95E-06	3641747
BUL4	-3.41627	0.000678	3642037
C1_08700W_A	-3.41788	7.52E-10	3634936
C2_00650W_A	-3.4251	1.14E-05	3638889
C6_02290C_A	-3.43065	9.15E-06	3645873
HPT1	-3.43583	4.67E-07	3643034
GZF3	-3.43677	3.67E-09	3642563
PHO86	-3.43801	0.000218	3642323
SPO72	-3.44545	2.45E-07	3644072
C5_03150W_A	-3.44693	2.52E-07	3637799
C2_09820W_A	-3.44946	9.2E-07	3643801
C3_07070C_A	-3.45387	9.57E-05	3638446
GNP3	-3.4547	4.28E-05	3639027
MSS51	-3.45482	4.06E-05	3639031
PRP5	-3.45524	2.88E-06	3638447
C4_00060W_A	-3.45734	6.06E-05	3648061
IFM1	-3.45801	2.25E-06	3641389
C4_01970W_A	-3.45854	8.23E-06	3635663
C1_04720W_A	-3.46466	3.25E-05	30514988
PAM18	-3.47079	0.000995	3645747
C1_08630W_A	-3.47234	8.12E-06	3634928
C1_08840W_A	-3.47623	0.043266	3634919
C3_02920W_A	-3.47815	1.99E-07	3638379
ZCF30	-3.47853	1.3E-06	3642051
POP4	-3.48883	0.000554	3639241
FCY24	-3.49244	0.003729	3641902
DAL5	-3.49958	0.000786	3638401
TUS1	-3.50317	1.38E-07	3646144
C1_09240C_A	-3.50476	0.057284	3634901
C2_07250C_A	-3.50896	0.006435	3643487
C5_04860C_A	-3.50927	0.009034	3636437
C1_14190C_A	-3.50962	5.35E-06	3643300
C2_05750W_A	-3.51284	8.88E-05	3645577

C7_02110W_A	-3.52402	1.26E-06	3637022
C1_10440W_A	-3.5352	0.000124	3647180
CTM1	-3.54765	7.3E-09	3638951
DUR32	-3.55125	0.001726	3636142
UGA3	-3.55529	1.63E-06	3638926
C2_09980W_A	-3.55556	2.58E-06	3643965
CR_10040W_A	-3.55699	0.000715	3639034
C6_04530C_A	-3.56005	4.39E-07	3645970
C6_02980C_A	-3.57156	8.22E-06	3639192
RXT3	-3.57297	5.79E-06	3643431
CDR4	-3.5836	2.96E-05	3640711
C3_04720C_A	-3.58586	8.6E-07	3635249
CAG1	-3.58823	0.000653	3636469
C1_13390W_A	-3.58923	0.000228	3636218
C1_02450C_A	-3.6036	5.95E-05	3636849
DES1	-3.60377	2.64E-07	3636529
CSP2	-3.60519	0.024199	3645749
ADR1	-3.60675	1.99E-06	3637866
MLH3	-3.61386	2.15E-06	3643294
C2_04110W_A	-3.61721	0.005979	3637378
C1_11880W_A	-3.61767	0.000237	3642007
C3_06420C_A	-3.61887	2.22E-06	3642262
C1_14350W_A	-3.62138	1.1E-07	3643281
SLM2	-3.62321	3.35E-08	3647559
C1_11580W_A	-3.62434	4.06E-06	3645330
CR_09610C_A	-3.63622	4.35E-05	3647094
PAM16	-3.63712	3.41E-05	3643321
ISU1	-3.64232	3.82E-07	3637007
CR_09800C_A	-3.64497	2.26E-06	3638984
CR_06970C_A	-3.65183	1.15E-05	3645736
BUD5	-3.65295	2.57E-05	3647315
MSN5	-3.66681	1.07E-06	3637889
CR_08680C_A	-3.66995	1.04E-07	3642127
C3_03200C_A	-3.6744	0.013744	3638428
C7_03500W_A	-3.67542	2.35E-05	3646246
ISY1	-3.68334	4.3E-05	3646233
CPA2	-3.68389	2.69E-07	3640385
HNT1	-3.6857	2.54E-09	3644441
C6_00980C_A	-3.68655	3.96E-05	3644046
NAT4	-3.68806	6.02E-05	3635704
C2_00540W_A	-3.69072	5.11E-08	3638865
C6_01340C_A	-3.69247	0.000725	30515331
C7_00260C_A	-3.70413	3.64E-08	3637978
C1_06510C_A	-3.70777	2.19E-07	3639549
GAP4	-3.71968	2.25E-07	3644463
C2_10790C_A	-3.72788	1.14E-05	3641242
C6_04510C_A	-3.7326	2.62E-06	3645955

C4_05970W_A	-3.73413	2.57E-08	3646514
C1_06840C_A	-3.7373	2.93E-06	30515012
CR_03160W_A	-3.74083	0.000557	3645117
CR_03480W_A	-3.74249	0.019886	3643199
C1_03140W_A	-3.75653	4.31E-05	3636929
C5_00570W_A	-3.77226	5.48E-10	3641589
C6_02020C_A	-3.77269	2.37E-06	3647563
C5_02320C_A	-3.7733	6.87E-06	3637788
C1_00860W_A	-3.77428	0.000391	3639358
OPY2	-3.77676	7.29E-08	3640765
C2_08450W_A	-3.7796	1.11E-05	3643905
C1_07150W_A	-3.79366	0.002485	30515019
HGT18	-3.79734	0.000435	3645111
REI1	-3.80962	3.74E-05	3647536
REG1	-3.81112	7.28E-08	3638858
SRR1	-3.81373	0.002217	3647589
SPT14	-3.8239	6.69E-07	3641019
RRN11	-3.82621	6.45E-05	3645276
C5_02070C_A	-3.83493	1.52E-08	3643746
ATS1	-3.84086	3.5E-06	3642079
MIF2	-3.84256	1.07E-06	3639220
JEN2	-3.84528	0.020478	3641214
C3_02630C_A	-3.84851	4.26E-06	3638328
SSY5	-3.8558	1.53E-08	3642140
SMC6	-3.85831	2.06E-06	3637039
RPD3	-3.8586	5.69E-07	3642580
C1_10120C_A	-3.85877	1.06E-06	3634779
C5_03800W_A	-3.87303	2.62E-06	3647230
NPR2	-3.87533	3E-06	3638360
PRP8	-3.88249	1.39E-10	3642125
CR_04710W_A	-3.8843	0.008513	3648141
C2_02920W_A	-3.88778	3.75E-08	3643016
TPO4	-3.90346	2.61E-06	3640956
C5_02590C_A	-3.91171	0.001487	3637723
C3_07370W_A	-3.91552	1.29E-07	3638474
C3_01770C_A	-3.92672	0.000229	3636588
ICL1	-3.94927	0.000104	3646146
C7_01020C_A	-3.9529	1.66E-05	3638020
C3_05850W_A	-3.95498	5.87E-06	3641758
HRR25	-3.9552	4.92E-10	3645878
C1_00580W_A	-3.95935	0.000108	3639361
C2_00690W_A	-3.96028	0.000555	3638885
C3_07670W_A	-3.96088	1.09E-11	3638460
IFR1	-3.96428	0.00326	3643930
SPO11	-3.9691	0.006474	3643873
PGA41	-3.96954	0.00788	3642800
C2_09660W_A	-3.97047	9.07E-07	3643793

PEX5	-3.97562	1.67E-07	3645026
C2_09790W_A	-3.97857	2.52E-05	3643804
HSK3	-4.00441	4.24E-05	3635730
DAC1	-4.01463	4.09E-05	3645967
MHP1	-4.02443	2.78E-09	3642460
C5_04120C_A	-4.02685	9.17E-06	3636522
C5_03460C_A	-4.02802	8.27E-05	3646345
ALD6	-4.02964	5.79E-05	3647407
C2_00640W_A	-4.04453	0.0001	3638847
UTP9	-4.04708	1.3E-08	3638451
C1_01630W_A	-4.04763	2.38E-08	3640194
C6_04200C_A	-4.04804	0.001029	30515344
CR_06550C_A	-4.05012	7.85E-07	3645260
HAP2	-4.05461	7.14E-09	3641800
C1_01150C_A	-4.0566	1.94E-07	3640203
C1_12750C_A	-4.05786	3.7E-05	3646818
C3_04620C_A	-4.06369	0.000754	3635155
C5_02020C_A	-4.06379	5.64E-08	3643771
GCA1	-4.06696	1.5E-06	3634903
CEK2	-4.0713	0.001131	3642459
LIP4	-4.07331	2.9E-06	3645987
C3_02420C_A	-4.07648	0.000136	3638342
GYP7	-4.07708	6.34E-08	3646245
C6_01620W_A	-4.07769	1.79E-05	3641647
C2_07800W_A	-4.07883	0.001915	3642325
WAR1	-4.083	3.99E-08	3644691
C3_06700C_A	-4.09329	1.45E-08	3642247
C5_00130C_A	-4.1037	1.09E-07	3647881
ISA2	-4.11204	1.38E-06	3638513
C1_11020W_A	-4.12561	4.95E-06	3644420
C4_00690C_A	-4.12883	3.09E-07	3645752
MAK16	-4.13329	4.93E-05	3646920
C1_05370C_A	-4.14354	1.76E-07	3642481
NOP6	-4.14733	1.49E-07	3639507
C4_01230C_A	-4.16147	9.83E-06	3635706
C1_05930C_A	-4.16586	3.71E-08	3639862
MRPL37	-4.18164	0.000891	3644782
C3_02750W_A	-4.1843	0.009426	3638411
CCE1	-4.18532	0.010249	3640395
CDR1	-4.18853	3.24E-08	3635237
DFI1	-4.1963	2.68E-09	3638038
CR_01710W_A	-4.19812	0.00023	3640130
ALS5	-4.20306	5.56E-09	3640277
C3_05450C_A	-4.21204	2.54E-07	3643669
C2_06090W_A	-4.22132	5.67E-09	3644097
C5_01560C_A	-4.23277	1.34E-07	3647064
HIP1	-4.23695	4.24E-11	3643761

BRE1	-4.23818	5.1E-08	3641568
C2_10090C_A	-4.2497	7.12E-06	3643939
C5_01470C_A	-4.26267	0.00119	3647050
C3_02910W_A	-4.27687	0.000324	3638378
ROA1	-4.28398	1.03E-07	3645424
C2_02110C_A	-4.28948	0.000393	3635745
CHR1	-4.30648	5.77E-08	3647601
NAG6	-4.30894	3.61E-11	3645961
TRK1	-4.31609	6.03E-10	3644153
CR_02030C_A	-4.32563	3.96E-06	3640152
C6_00520W_A	-4.333	0.007107	3647147
CSP1	-4.33333	0.010008	3648242
CR_04820W_A	-4.33605	3.92E-08	3647298
FLO8	-4.33961	8.75E-10	3646292
TOR1	-4.35114	2.9E-10	3643480
C7_00350C_A	-4.35184	6.4E-09	3638039
C4_07170C_A	-4.35227	0.00072	30515289
OLE2	-4.35433	2.02E-09	3643503
C3_00340W_A	-4.36538	0.000385	3640544
IFF6	-4.36721	2.07E-09	3644087
CCC2	-4.36801	1.27E-08	3637821
YME1	-4.37722	4.66E-09	3641816
C5_01710C_A	-4.39686	2.28E-05	3643732
TCO89	-4.39782	1.75E-08	3644788
C1_14380C_A	-4.40413	3.32E-07	3643285
CKS1	-4.41179	8.61E-07	3647732
C1_09520C_A	-4.42014	2.08E-09	3634766
FUN31	-4.43455	1.34E-09	3642249
C6_04180W_A	-4.44797	3.76E-09	3648176
C6_03800C_A	-4.45094	1.79E-07	3640310
CIRT4B	-4.45167	4.53E-09	3642560
FGR23	-4.45996	1.09E-07	3636555
ECM1	-4.46274	1.09E-06	3641224
C3_00030C_A	-4.47272	2.22E-07	3640571
CR_02550C_A	-4.47896	8.14E-07	3647689
C2_05840W_A	-4.47952	1.79E-05	3647484
C3_02890C_A	-4.49275	0.00199	3638336
MED3	-4.4983	5E-06	3646522
CR_03980W_A	-4.50191	4.79E-07	3640964
C4_00100C_A	-4.50694	4.88E-08	3645004
CR_03840C_A	-4.51802	0.007075	3643184
C3_02040C_A	-4.52288	5.56E-07	3636579
C3_07400W_A	-4.53207	1.81E-06	3638471
CR_08420W_A	-4.54692	3.18E-09	3642117
CR_03360W_A	-4.54927	3.86E-08	3645141
C4_03020W_A	-4.55202	8.34E-14	3637934
ECM21	-4.55474	2.6E-12	3634784

SNQ2	-4.55633	1.5E-10	3640290
C3_01430W_A	-4.55739	7.19E-06	3636633
C3_06240C_A	-4.55984	0.013344	30515213
C6_00170C_A	-4.57347	3.37E-11	3647903
KTI11	-4.58212	0.002562	30515125
C7_00330C_A	-4.58361	3.34E-07	3638042
LPI9	-4.61016	2.8E-12	3637060
YTH1	-4.61469	1.18E-12	3645572
BMT5	-4.62735	2.3E-11	3635781
C6_02660C_A	-4.63528	6.25E-05	3639224
SIP5	-4.63821	7.9E-09	3639865
ABC1	-4.65579	2.63E-07	3640239
C5_04530W_A	-4.66229	2.29E-08	3636507
C2_02280W_A	-4.67732	4.64E-10	3635806
C4_03350C_A	-4.69815	1.36E-06	3646794
C2_02270C_A	-4.71277	5.57E-07	3635805
RIM21	-4.7147	6E-09	3643739
CYC3	-4.71473	1.53E-11	3642621
RRN3	-4.71936	4.5E-08	3647925
C1_09130W_A	-4.72419	5.14E-11	3634943
CR_00810W_A	-4.72569	9.94E-05	3640415
LTV1	-4.7344	1.22E-07	3639016
C5_04080C_A	-4.73779	8.38E-09	3647731
CR_10760C_A	-4.74568	1.5E-05	3638943
C1_07980C_A	-4.74903	4.56E-10	3640696
C1_01710W_A	-4.75956	2.4E-10	3640201
C3_01130C_A	-4.76524	4.62E-09	3639901
C1_05790W_A	-4.76743	5.36E-05	30515002
EBP1	-4.7735	6.35E-09	3644060
CR_10060W_A	-4.7737	1.91E-12	3639035
C2_10130W_A	-4.7747	0.004274	3643935
PAN6	-4.77965	5.54E-08	3647684
RFG1	-4.79205	6.15E-12	3642570
HIT1	-4.8008	0.000133	3637854
C4_03000C_A	-4.80272	4.58E-09	3637935
PRA1	-4.81012	0.001515	3642969
C3_07650C_A	-4.81092	4.4E-05	3638488
C7_01940C_A	-4.81459	7.4E-11	3636971
SEN15	-4.81837	0.000123	3646411
HMX1	-4.84589	3.1E-07	3639294
C3_02900W_A	-4.85544	2.62E-05	3638337
C1_11080W_A	-4.87754	6.18E-11	3644416
HBR3	-4.88015	6.75E-08	3645652
CR_10280W_A	-4.88435	0.000141	3638934
C1_11160C_A	-4.88534	7.51E-06	3644399
C3_03370C_A	-4.8965	6.87E-09	3638366
YPT53	-4.89817	2.5E-07	3642483

AHR1	-4.90633	8.99E-09	3641733
ATG9	-4.90842	5.46E-08	3635239
C1_00270W_A	-4.91001	3.85E-07	3639300
C2_02530W_A	-4.91476	0.016749	3635819
FMP45	-4.92605	5.94E-08	3637004
C4_05900C_A	-4.93165	5.12E-13	3641796
FEN1	-4.9368	1.8E-07	3646829
PUF3	-4.93694	1.04E-09	3646950
C6_00380C_A	-4.98372	1.69E-07	3646085
C2_06570C_A	-4.9909	1.65E-11	3646561
C1_10950C_A	-4.99379	4.83E-08	3644428
SMM1	-4.99744	1.63E-07	3643778
YHB1	-5.00854	2.56E-07	3647353
C2_09570C_A	-5.00884	0.000241	3643811
C2_08950W_A	-5.00934	4.47E-09	3645788
CR_10000C_A	-5.01142	0.000572	3639030
C5_03260C_A	-5.0209	3.35E-08	3646360
GST2	-5.03062	6.26E-10	3637937
SNT1	-5.03382	4.69E-12	3642033
DAL8	-5.03544	3.4E-05	3635181
C2_08920W_A	-5.05534	8.26E-10	3645797
CR_03250C_A	-5.06858	3.86E-12	3645102
POP3	-5.08685	4.93E-06	3638993
PFK26	-5.08712	6.96E-13	3634912
CR_04920W_A	-5.10275	1.48E-10	3648207
C3_05800W_A	-5.11022	7.07E-08	3641754
MDM34	-5.11808	1.37E-11	3647517
C2_01660C_A	-5.12348	8.01E-07	3635742
C1_07530W_A	-5.14731	0.001005	30515025
C1_04710C_A	-5.15295	3.5E-06	3644764
GCV2	-5.16244	5.34E-11	3646505
ARG8	-5.17908	9.01E-09	3641131
CR_03660C_A	-5.18105	5.42E-08	3643219
SCH9	-5.2082	1.74E-10	3637421
C4_02520C_A	-5.23218	8.82E-10	3637863
KCS1	-5.24502	4.29E-07	3648120
CTA8	-5.25422	6.06E-11	3634947
C3_03690W_A	-5.27897	0.003443	3645647
YWP1	-5.28376	8.93E-07	3643915
CR_04250W_A	-5.29257	0.036435	3640990
C4_04560C_A	-5.29883	3.93E-12	3641105
C6_02790C_A	-5.31603	7.7E-11	3639245
MDJ1	-5.33222	5.71E-11	3646980
STP4	-5.33663	0.000235	3637405
CR_09920W_A	-5.35082	7.82E-12	3639028
C1_10710C_A	-5.36194	0.000568	3644407
C1_06090C_A	-5.38548	1.98E-11	3639822

PTC5	-5.39576	3.53E-12	3642072
C4_02690W_A	-5.4247	2.75E-08	3637861
SFT1	-5.4594	3.04E-05	30515372
C3_00120W_A	-5.4607	0.01579	3640566
CR_04600W_A	-5.50446	1.72E-11	3640950
C3_02840W_A	-5.50465	7.57E-05	3638331
MRP17	-5.50552	5.22E-09	3641548
ZCF28	-5.52537	8.7E-06	3634776
SUR2	-5.54165	6.29E-12	3642973
RNR3	-5.54211	3.95E-11	3647591
CR_03670W_A	-5.54322	0.01872	3643220
DHH1	-5.54861	1.67E-09	3639490
C5_02680W_A	-5.57642	5.88E-11	3637774
C6_01590W_A	-5.58157	5E-17	3641650
C4_06040W_A	-5.59684	8.14E-06	3646521
ELC1	-5.60061	0.000556	3646151
HGT4	-5.60102	5.3E-10	3635269
COX19	-5.61657	3.92E-07	3636220
C2_02300W_A	-5.63176	1.91E-06	3635808
C1_01510W_A	-5.64095	7.32E-06	3640229
TPS2	-5.64123	1.73E-10	3636892
C7_00340C_A	-5.64301	1.75E-14	3638040
IFE2	-5.64965	3.15E-10	3648027
CR_02100C_A	-5.65445	1.22E-06	3640141
EPL1	-5.65668	4.85E-16	3639732
AAF1	-5.65936	1.17E-12	3642284
RAD3	-5.71151	2.4E-15	3638014
PRM9	-5.71287	0.013767	30515173
SUA71	-5.72828	2.87E-14	3636879
DRE2	-5.73687	1.69E-13	3642571
MET1	-5.75743	7.07E-12	3643014
C2_08030W_A	-5.90943	1.47E-07	3642309
BRG1	-5.91035	1.36E-08	3647664
GRF10	-5.9169	8.45E-14	3636492
BUB3	-5.92733	4.5E-12	3646362
WHI3	-5.93257	1.47E-11	3636991
C1_13130C_A	-5.93294	2.48E-11	3636202
SLY41	-5.93544	9.09E-14	3647139
C4_00260W_A	-5.95312	5.8E-10	3645037
C1_09790C_A	-5.98622	3.15E-12	3634843
RPA12	-5.98865	1.88E-06	3643477
C2_03570C_A	-5.99096	1.18E-05	3637336
C3_06560W_A	-5.99676	2.41E-14	3642276
ATO10	-5.99815	0.001005	3640394
RGA2	-6.01453	1.5E-15	3635661
CR_03260W_A	-6.02018	1.42E-09	30515389
C3_05250C_A	-6.0238	2.39E-08	3635199

C6_00530C_A	-6.04487	4.48E-06	30515326
MTM1	-6.04526	9.83E-09	3642487
C3_05790C_A	-6.04565	6.25E-06	3641753
C1_14330W_A	-6.05032	1.13E-07	3643278
C7_03380W_A	-6.08442	9.82E-07	30515359
ARG1	-6.09024	1.55E-08	3639691
C2_02570W_A	-6.10826	6.77E-06	3635858
HAP42	-6.11995	2.33E-14	3635729
CRP1	-6.12063	4.42E-10	3634826
C3_05900W_A	-6.14417	4.42E-08	3641739
RIM101	-6.17321	5.53E-12	3643280
C6_04400W_A	-6.20468	0.000338	3645949
C3_05750C_A	-6.20721	0.001224	3641719
NBP35	-6.21105	1.34E-13	3647412
TAZ1	-6.21265	7.2E-07	3644108
PGA7	-6.22971	1.16E-11	3645006
C1_06750W_A	-6.23046	0.000121	3639506
DED1	-6.26562	5.87E-14	3641752
C1_07790C_A	-6.31791	1.57E-07	3640706
AUT7	-6.32421	1.78E-10	30514999
C5_01730W_A	-6.33044	1.9E-05	3643730
TYE7	-6.33373	6.17E-12	3636203
C7_01690W_A	-6.35742	6.94E-09	3636980
C2_05810W_A	-6.36764	1.43E-13	3647481
C1_13010W_A	-6.38454	7.26E-10	3636120
MIT1	-6.38462	6.81E-16	3644089
C1_05330C_A	-6.45866	2.66E-12	3642485
C1_04590W_A	-6.50992	2.05E-10	30514987
C2_00760C_A	-6.55805	9.78E-09	3638910
C2_09880C_A	-6.56095	6.76E-14	3643779
C3_01110C_A	-6.56934	0.008117	3639835
CR_09930W_A	-6.58791	9.83E-12	3639029
C2_05640W_A	-6.65088	4.22E-15	3645566
STE11	-6.65166	1.37E-17	3637369
CR_08650C_A	-6.75275	1.86E-16	3642124
C1_08890C_A	-6.75402	0.001967	3634924
C1_09320C_A	-6.78056	1.76E-22	3634834
C5_03210C_A	-6.82004	0.005964	3646364
C4_00810C_A	-6.83886	9.9E-07	3645742
C1_09650W_A	-6.86893	6.25E-16	3634870
C3_02980C_A	-6.88343	7.54E-07	3638386
CR_00310C_A	-6.95541	8.43E-15	3639715
STB3	-6.95912	5.64E-14	3645781
CPA1	-6.96776	2.18E-11	3635715
UTP22	-7.01704	1.44E-08	3635760
C3_06940W_A	-7.04491	1.25E-20	3638509
C2_08530C_A	-7.10194	0.000299	3643921

C1_05160C_A	-7.14117	2.18E-15	3642468
C1_05690C_A	-7.1462	0.043838	3639896
QDR3	-7.14642	5.95E-10	3644023
C4_07020C_A	-7.14671	0.043891	3642965
DLH1	-7.14671	0.044338	3647605
C1_13820C_A	-7.1643	3.47E-07	3636127
PHR3	-7.16518	9.48E-17	3645003
C7_01910C_A	-7.17373	4.07E-11	3637029
MFG1	-7.17601	2.54E-15	3643884
C1_01610C_A	-7.18085	7.32E-08	3640225
C2_02390W_A	-7.18977	5.2E-13	3635829
ATG1	-7.25146	2.14E-14	3641175
ALS6	-7.27553	1.89E-10	3642266
WOR2	-7.29639	9.23E-10	3635230
C3_00230C_A	-7.29863	2.36E-06	3640557
C1_08610C_A	-7.37166	1.2E-11	3646512
CTR2	-7.43545	2.21E-18	3648427
CR_08080W_A	-7.43618	0.000205	3642094
CR_08580C_A	-7.44234	5.64E-08	3642105
C4_01960C_A	-7.45723	1.46E-15	3635664
RPN4	-7.4797	1.88E-16	3644684
C3_07570C_A	-7.48263	1.32E-10	3638522
C6_03200W_A	-7.49436	5.25E-14	3639177
C3_00620C_A	-7.50139	1.39E-10	30515171
C6_01780C_A	-7.52558	5.56E-12	3641636
C7_02660C_A	-7.53996	0.000487	3647652
GCA2	-7.59559	4.57E-10	3647194
HSP78	-7.61582	8.11E-14	3637414
C1_04820C_A	-7.62387	3.02E-07	3644787
C2_08700C_A	-7.65459	3.52E-14	3643887
C6_03000C_A	-7.67468	0.001074	3639194
C7_00630C_A	-7.73952	4.8E-07	3638028
CDR11	-7.77363	5.94E-12	3647389
C1_05920W_A	-7.85065	0.001561	3639864
C4_03230C_A	-7.85181	0.001286	3637836
PZF1	-7.8634	1.09E-08	3648113
C4_07040W_A	-7.87465	3.52E-05	3642963
C2_01060C_A	-7.88775	9.21E-08	3638835
FRP1	-7.90741	3.19E-19	3645005
TRY4	-7.93371	9.46E-11	3635240
CFL2	-8.09536	0.004242	3641811
PGA13	-8.09703	0.002229	3642138
C7_04160W_A	-8.14315	6.21E-08	3643127
QDR2	-8.15455	4.27E-15	3643661
RSN1	-8.16723	7.16E-17	3638308
C4_05160C_A	-8.18446	2E-15	3647411
CR_04220C_A	-8.22131	2.24E-08	3640943

ITR1	-8.24749	4.67E-25	3643440
CR_08470W_A	-8.25124	3.65E-14	3642134
C1_09780C_A	-8.33306	7.35E-27	3634842
CR_07680C_A	-8.40085	1.32E-05	3648193
ZCF3	-8.41123	1.17E-15	3645335
C6_02950C_A	-8.43375	8.45E-10	3639188
FGR46	-8.45983	0.004014	3638989
C5_05240C_A	-8.46037	0.004127	3636471
MTLA2	-8.46082	0.006093	3643766
CR_01600C_A	-8.46491	4.41E-09	3640080
SLD1	-8.69881	1.04E-14	3638406
C1_11320C_A	-8.73187	6.86E-10	30515052
CR_08720W_A	-8.73516	1.58E-09	3642122
RPC10	-8.79914	6.22E-13	3643292
C4_06200W_A	-8.87447	4.25E-05	30515282
OYE23	-8.95506	4.72E-05	3641618
CR_08830W_A	-8.98186	6.34E-12	30515450
C1_10580C_A	-9.05273	8.61E-06	3647679
C1_08830C_A	-9.05385	0.0049	3634918
C7_03550C_A	-9.05443	0.006432	3646235
CR_02910W_A	-9.08548	2.13E-15	3642586
RCH1	-9.14498	1.2E-15	3645002
C1_13250W_A	-9.15371	7.33E-19	3636143
C1_00090W_A	-9.17941	3.2E-06	3639304
DEF1	-9.23254	1.59E-14	3639023
SPO1	-9.37225	0.000731	3647039
C6_01400W_A	-9.37401	0.000817	3641604
C6_03440W_A	-9.43739	4.79E-10	3640274
C1_12630C_A	-9.50422	3.11E-07	3646838
CR_00840C_A	-9.54329	0.002603	3640411
SSY1	-9.58861	2.51E-17	3637382
C3_03250W_A	-9.65418	1.1E-20	3638318
C5_02640W_A	-9.68207	1.77E-13	3637785
HEM3	-9.68335	1.94E-18	3643940
FYV5	-9.74975	3.6E-08	3647057
PTC8	-9.78163	3.13E-20	3635674
RIM8	-9.82622	1.84E-18	3639342
C4_07140W_A	-9.90598	1.47E-20	3642936
ARF1	-9.99222	8.66E-23	3642130
C5_01900C_A	-10.0431	0.001441	30515302
C2_02580W_A	-10.044	3.2E-15	3635860
OPT4	-10.0534	5E-14	3647697
C5_02980C_A	-10.1548	1.66E-11	3637816
ECM22	-10.1764	3.7E-24	3648000
C3_05840W_A	-10.1984	0.008738	3641757
C3_04510W_A	-10.2947	4.67E-10	3635258
RFX1	-10.3614	5.83E-10	3644844

C2_07280W_A	-10.3997	9.32E-05	3643475
COX11	-10.4155	1.64E-24	3646842
C2_09610W_A	-10.4611	1.42E-16	3643798
C5_04840C_A	-10.5708	8.29E-17	3636435
C6_03360C_A	-10.575	2.23E-08	3639203
FTH1	-10.6044	1.08E-18	3634852
C3_00150W_A	-10.6374	5.08E-11	3640595
HSP31	-10.6472	7.26E-08	3644589
FET34	-10.6475	2.32E-19	3647131
C1_11510C_A	-10.7982	2.07E-15	3645336
CUP9	-10.9531	2.41E-15	3636950
C4_04520W_A	-10.9694	2.46E-14	3641110
C2_06520C_A	-11.0728	3.23E-07	3646571
KAR4	-11.1158	1.27E-08	3644937
VTC4	-11.1411	1.46E-34	3646795
C3_06950W_A	-11.2351	2.33E-16	3638507
C6_03180C_A	-11.2543	1.15E-29	3639261
C4_02770C_A	-11.3088	1.1E-10	3637903
TPS3	-11.3138	9.39E-23	3641205
FRE10	-11.3214	9.66E-22	3646841
WSC1	-11.4837	3.95E-19	3635138
C4_02740W_A	-12.0617	1.09E-23	3637855
C2_02190C_A	-12.0801	8.61E-18	3635753
HGT17	-12.1264	2.56E-05	3635687
MRV4	-12.1281	3.05E-05	3636547
CTA4	-12.13	1.06E-27	3641743
ARR3	-12.2071	2.52E-09	3642910
C1_06350W_A	-12.2162	1.07E-23	3639562
C7_02610C_A	-12.3031	8.03E-20	3648285
TRY6	-12.3817	1.03E-06	3638499
HAK1	-12.3849	9.44E-07	3639546
SEN2	-12.472	1.91E-14	3637832
C1_09820C_A	-12.4964	2.37E-05	3634846
C2_07070W_A	-12.5653	6.73E-18	3643501
C6_01870C_A	-12.5851	4.81E-16	3641625
COX17	-12.6806	1.4E-08	30515088
C5_01020C_A	-13.225	0.000827	30515297
C2_08350C_A	-13.3179	0.023044	3640769
C2_10000C_A	-13.3179	0.02283	30515163
C3_04450C_A	-13.3187	0.022825	3635207
SSU81	-13.3554	2.84E-17	3634944
ELA1	-13.9595	1.83E-12	3644680
C1_01130W_A	-13.9842	3.49E-27	3640178
C2_08580W_A	-14.1126	5.97E-07	3643916
C4_01240C_A	-14.4275	7.99E-18	3635705
C4_04300C_A	-14.509	3.38E-10	3646843
FAR1	-14.5391	2.4E-11	3637970

C5_01990W_A	-14.5542	0.000252	3643774
C2_04670W_A	-14.5606	5.37E-13	3647789
PGA48	-14.6278	2.54E-25	3647905
C5_04850W_A	-14.8072	1.81E-07	3636436
C4_05350W_A	-15.0473	2.56E-30	3646948
FTR1	-15.5486	1.03E-24	3643318
C1_07650W_A	-15.7604	4.85E-12	3646657
GAC1	-15.8388	5.33E-26	3638025
ZCF2	-16.1431	8.98E-24	3642484
C3_01540W_A	-16.4187	2.86E-08	3636567
C2_06530W_A	-16.5948	3E-10	3646572
CR_01510C_A	-17.3958	0.000137	3640104
PGA37	-18.0574	3.49E-12	3636484
C1_09210C_A	-18.486	1.23E-40	3634897
GIT1	-19.0061	0.007193	3646562
C3_00590W_A	-19.3163	0.011395	6335207
C1_05440C_A	-19.7732	4.15E-22	3642489
C5_03080C_A	-19.8345	2.08E-30	3637701
C7_03560W_A	-20.0478	5.28E-17	3646236
C7_00880C_A	-20.2943	1.46E-26	3638052
FRE9	-20.5985	4.91E-31	3643452
C6_03810W_A	-20.6664	5.84E-35	3640286
C7_02600C_A	-20.9744	9.75E-28	3648286
WOR3	-21.1252	2.25E-13	3641009
CLG1	-21.3623	2.33E-46	3646030
C4_01220C_A	-21.4702	9.83E-09	3635650
C6_04420W_A	-22.1141	6.55E-14	3645951
SUT1	-22.3533	3.65E-33	3637706
C3_06660C_A	-22.4047	0.000204	3642253
C7_01430C_A	-22.481	7.88E-18	3636961
IFA14	-22.5334	6.68E-11	3639012
FGR47	-23.1897	4.39E-34	3641651
ALS7	-23.3069	6.05E-37	3642234
PGA57	-23.4119	5.68E-08	3635608
PRX1	-23.4625	5.52E-37	3641357
C2_05330C_A	-24.1733	1.96E-27	3643426
LIP1	-24.2996	4.78E-07	3634888
FGR42	-24.692	0.002396	3647438
C6_03280W_A	-24.6922	0.002435	3639185
C5_04030W_A	-26.3099	4.69E-34	3647737
BMT1	-26.4009	1.68E-32	3638535
ARG11	-27.4639	7.09E-08	3635193
CR_08880C_A	-28.6962	1.79E-19	3641878
UGA6	-29.9059	5.75E-07	3642975
C1_01620C_A	-30.0636	6.55E-35	3640193
ZCF1	-30.2303	9.59E-36	3638329
PUT1	-30.3718	1.27E-35	3637779

C1_00190C_A	-30.8314	6.55E-36	3639337
KSP1	-31.3795	3.56E-45	3644467
GIT3	-34.1502	2.85E-45	3642634
HSP21	-36.4567	9.27E-29	3637364
RME1	-36.806	5.7E-41	3644453
C1_09770W_A	-37.666	8.09E-24	3634825
C6_00810C_A	-40.6757	2.5E-29	3647068
CR_06140W_A	-41.4117	1.18E-32	3644862
CDA2	-44.5016	5.93E-09	3639716
C1_06430C_A	-47.4333	0.000274	3639553
C1_02690C_A	-47.6452	0.000348	3636823
CR_04940W_A	-47.6739	1.27E-12	3648209
C3_03460C_A	-55.3378	6.55E-14	3638359
C4_03340C_A	-55.9612	2.34E-11	3648090
HWP2	-68.7656	7.89E-05	3646803
C2_08260W_A	-69.5115	1.9E-10	3642345
SSU1	-75.1902	4.98E-55	3641882
C5_04380C_A	-80.3798	9.51E-09	3636485
C2_02130C_A	-80.8713	3.9E-17	3635747
C7_01680C_A	-82.3703	8.21E-55	3636981
C1_11410C_A	-92.9351	6.91E-06	30515054
PHO87	-101.876	6.82E-16	3639861
CFL4	-110.874	1.04E-09	3642639
HSP30	-133.299	8.39E-34	3645419
C2_04090W_A	-175.745	0.047536	3637380
C1_06620C_A	-181.056	0.042471	3639545
C3_04870W_A	-181.056	0.042535	3635226
C5_00810C_A	-181.056	0.042535	3644127
CR_07540C_A	-181.056	0.042535	3648001
C1_07040C_A	-181.067	0.043619	3639533
MNN1	-200.045	1.02E-21	3637784
CR_01530C_A	-214.7	3.28E-13	3640072
C2_09330C_A	-222.761	0.034696	3644106
C7_00770W_A	-225.442	3.28E-23	3637959
C3_07910W_A	-233.322	0.037145	3647449
CRZ2	-251.913	8.85E-83	3645725
C3_07930C_A	-278.198	0.031524	3647452
C4_07260W_A	-280.664	8.97E-09	3642942
GPI1	-285.772	3.82E-28	3643680
CR_03320C_A	-322.997	0.027477	3645096
C2_02640C_A	-598.085	0.012099	30515095
C4_06910W_A	-766.138	6.97E-12	3642933
C3_06170C_A	-848.358	0.008112	3642272
C1_14630C_A	-30088.4	5.05E-05	3643291
TAR1	-102569	0	30515449

B120 vs Control

Name	Fold change	P-value	GeneID
ASG7	#N/A	#N/A	3639173
ATO7	#N/A	#N/A	3635814
BUD20	#N/A	#N/A	3645800
C1_00020C_A	#N/A	#N/A	3639374
C1_01720C_A	#N/A	#N/A	30514964
C1_01970W_A	#N/A	#N/A	30514967
C1_03580W_A	#N/A	#N/A	30514980
C1_03880C_A	#N/A	#N/A	3646409
C1_04440W_A	#N/A	#N/A	3646138
C1_05550C_A	#N/A	#N/A	30514994
C1_05730W_A	#N/A	#N/A	30515001
C1_05890W_A	#N/A	#N/A	3639876
C1_06300W_A	#N/A	#N/A	30515004
C1_06310W_A	#N/A	#N/A	30515005
C1_06490C_A	#N/A	#N/A	30515009
C1_06830W_A	#N/A	#N/A	30515011
C1_07320W_A	#N/A	#N/A	30515023
C1_07450W_A	#N/A	#N/A	30515024
C1_07940W_A	#N/A	#N/A	30515027
C1_08960W_A	#N/A	#N/A	30515032
C1_08970W_A	#N/A	#N/A	30515033
C1_09500W_A	#N/A	#N/A	3634764
C1_09560C_A	#N/A	#N/A	30515034
C1_09700W_A	#N/A	#N/A	3634876
C1_10050W_A	#N/A	#N/A	3634811
C1_10130W_A	#N/A	#N/A	30515040
C1_10590W_A	#N/A	#N/A	30515042
C1_11180C_A	#N/A	#N/A	30515050
C1_11710C_A	#N/A	#N/A	3645320
C1_11780W_A	#N/A	#N/A	30515057
C1_12040W_A	#N/A	#N/A	3642029
C1_12940C_A	#N/A	#N/A	30515067
C1_12980W_A	#N/A	#N/A	30515068
C1_12990W_A	#N/A	#N/A	30515069
C1_13000W_A	#N/A	#N/A	30515070
C1_13040C_A	#N/A	#N/A	30515071
C1_13500C_A	#N/A	#N/A	30515075
C2_00310W_A	#N/A	#N/A	3638800
C2_00560W_A	#N/A	#N/A	3638854
C2_00900W_A	#N/A	#N/A	3638895
C2_01460C_A	#N/A	#N/A	3635844
C2_02080W_A	#N/A	#N/A	30515093
C2_02140C_A	#N/A	#N/A	3635748
C2_03580W_A	#N/A	#N/A	30515100
C2_03630W_A	#N/A	#N/A	3637438

C2_04070C_A	#N/A	#N/A	30515110
C2_04610C_A	#N/A	#N/A	30515113
C2_05310C_A	#N/A	#N/A	30515119
C2_05420C_A	#N/A	#N/A	30515121
C2_05600W_A	#N/A	#N/A	30515124
C2_05880W_A	#N/A	#N/A	30515129
C2_06400C_A	#N/A	#N/A	30515135
C2_06410W_A	#N/A	#N/A	30515136
C2_06560W_A	#N/A	#N/A	30515139
C2_06980W_A	#N/A	#N/A	3643530
C2_07710W_A	#N/A	#N/A	3642305
C2_08150W_A	#N/A	#N/A	3642328
C2_08520C_A	#N/A	#N/A	30515155
C2_09110C_A	#N/A	#N/A	3644114
C2_09340W_A	#N/A	#N/A	3644107
C2_09540W_A	#N/A	#N/A	30515159
C2_09550C_A	#N/A	#N/A	30515160
C2_09800C_A	#N/A	#N/A	3643803
C2_10150W_A	#N/A	#N/A	3643933
C2_10800W_A	#N/A	#N/A	30515169
C3_00140W_A	#N/A	#N/A	3640596
C3_00680C_A	#N/A	#N/A	3640537
C3_01050C_A	#N/A	#N/A	3639831
C3_01740C_A	#N/A	#N/A	30515178
C3_02880W_A	#N/A	#N/A	3638335
C3_04150W_A	#N/A	#N/A	30515202
C3_05660C_A	#N/A	#N/A	3643656
C3_05820W_A	#N/A	#N/A	30515212
C3_06390W_A	#N/A	#N/A	30515216
C4_00990W_A	#N/A	#N/A	3635609
C4_01570C_A	#N/A	#N/A	30515236
C4_01580C_A	#N/A	#N/A	30515237
C4_01590C_A	#N/A	#N/A	30515238
C4_01600C_A	#N/A	#N/A	30515239
C4_01610C_A	#N/A	#N/A	30515240
C4_01620C_A	#N/A	#N/A	30515241
C4_01630C_A	#N/A	#N/A	30515242
C4_01640C_A	#N/A	#N/A	30515243
C4_02650C_A	#N/A	#N/A	30515249
C4_03480C_A	#N/A	#N/A	3646815
C4_04040W_A	#N/A	#N/A	3641213
C4_04400C_A	#N/A	#N/A	30515268
C4_04490C_A	#N/A	#N/A	30515269
C4_06180C_A	#N/A	#N/A	30515281
C4_06520W_A	#N/A	#N/A	30515284
C4_06630C_A	#N/A	#N/A	3642812
C5_01030W_A	#N/A	#N/A	30515298

C5_01150C_A	#N/A	#N/A	30515299
C5_01160C_A	#N/A	#N/A	30515300
C5_02850W_A	#N/A	#N/A	30515305
C5_03450W_A	#N/A	#N/A	30515311
C5_03890C_A	#N/A	#N/A	30515312
C5_04450C_A	#N/A	#N/A	30515315
C6_00220C_A	#N/A	#N/A	30515324
C6_01540C_A	#N/A	#N/A	30515333
C6_02390W_A	#N/A	#N/A	30515339
C6_02400W_A	#N/A	#N/A	30515340
C6_02620C_A	#N/A	#N/A	3639211
C6_03570W_A	#N/A	#N/A	3640248
C6_03600C_A	#N/A	#N/A	3640295
C6_04150W_A	#N/A	#N/A	30515343
C7_00540W_A	#N/A	#N/A	30515348
C7_01000C_A	#N/A	#N/A	3638022
C7_01060W_A	#N/A	#N/A	3637950
C7_01130C_A	#N/A	#N/A	3645912
C7_01260W_A	#N/A	#N/A	30515352
C7_02190W_A	#N/A	#N/A	30515356
C7_02420C_A	#N/A	#N/A	3636975
C7_04070C_A	#N/A	#N/A	3643153
CR_00450C_A	#N/A	#N/A	30515367
CR_00500C_A	#N/A	#N/A	30515368
CR_00530W_A	#N/A	#N/A	3639668
CR_02320W_A	#N/A	#N/A	30515381
CR_02470W_A	#N/A	#N/A	30515382
CR_02660C_A	#N/A	#N/A	30515383
CR_03190C_A	#N/A	#N/A	30515388
CR_04980C_A	#N/A	#N/A	3646395
CR_05350W_A	#N/A	#N/A	30515405
CR_05370W_A	#N/A	#N/A	30515406
CR_05470C_A	#N/A	#N/A	30515409
CR_06190C_A	#N/A	#N/A	30515418
CR_06200C_A	#N/A	#N/A	30515419
CR_06210C_A	#N/A	#N/A	30515420
CR_06220C_A	#N/A	#N/A	30515421
CR_06580W_A	#N/A	#N/A	3646627
CR_06600C_A	#N/A	#N/A	30515422
CR_06630W_A	#N/A	#N/A	30515423
CR_08140W_A	#N/A	#N/A	30515435
CR_10320W_A	#N/A	#N/A	3638938
CR_10460W_A	#N/A	#N/A	30515457
CSA2	#N/A	#N/A	3642930
FGR18	#N/A	#N/A	3644112
FGR37	#N/A	#N/A	3641609
FIG1	#N/A	#N/A	3644025

GTT1	#N/A	#N/A	3643653
GTT12	#N/A	#N/A	3638304
ITS1	#N/A	#N/A	30515447
ITS2	#N/A	#N/A	30515448
MFALPHA	#N/A	#N/A	3646420
MRV3	#N/A	#N/A	3636546
OPT5	#N/A	#N/A	3640768
PGA12	#N/A	#N/A	3638920
PGA22	#N/A	#N/A	3644939
PGA50	#N/A	#N/A	3647524
RDN18	#N/A	#N/A	3648135
RDN25	#N/A	#N/A	3648137
RDN5	#N/A	#N/A	3648138
RDN58	#N/A	#N/A	3648136
RFC52	#N/A	#N/A	30514953
RHD2	#N/A	#N/A	3637891
RPR1	#N/A	#N/A	30515150
SCR1	#N/A	#N/A	30515392
SNR52	#N/A	#N/A	30515222
SNR6	#N/A	#N/A	30515211
SPR3	#N/A	#N/A	3635853
tA(AGC)1	#N/A	#N/A	30515148
tA(AGC)2	#N/A	#N/A	30515199
tA(AGC)3	#N/A	#N/A	30514965
tA(AGC)4	#N/A	#N/A	30514960
tA(AGC)5	#N/A	#N/A	30515321
tA(AGC)7	#N/A	#N/A	30515078
tA(UGC)1	#N/A	#N/A	30515336
tA(UGC)2	#N/A	#N/A	30515342
tC(GCA)1	#N/A	#N/A	30515411
tC(GCA)2	#N/A	#N/A	30515410
tD(GUC)2	#N/A	#N/A	30515261
tD(GUC)3	#N/A	#N/A	30515260
tD(GUC)4	#N/A	#N/A	30515259
tD(GUC)5	#N/A	#N/A	30515114
tD(GUC)6	#N/A	#N/A	30515233
tD(GUC)7	#N/A	#N/A	30515452
tE(CUC)1	#N/A	#N/A	30515101
tE(UUC)1	#N/A	#N/A	30515196
tE(UUC)2	#N/A	#N/A	30515184
tE(UUC)3	#N/A	#N/A	30515295
tE(UUC)4	#N/A	#N/A	30515294
tE(UUC)5	#N/A	#N/A	30515309
tE(UUC)6	#N/A	#N/A	30515198
tE(UUC)7	#N/A	#N/A	30515445
tF(GAA)1	#N/A	#N/A	30515191
tF(GAA)2	#N/A	#N/A	30515103

tF(GAA)3	#N/A	#N/A	30515107
tF(GAA)4	#N/A	#N/A	30515166
tF(GAA)5	#N/A	#N/A	30515224
tG(CCC)1	#N/A	#N/A	30515228
tG(GCC)1	#N/A	#N/A	30515432
tG(GCC)2	#N/A	#N/A	30515258
tG(GCC)3	#N/A	#N/A	30515115
tG(GCC)4	#N/A	#N/A	30515231
tG(GCC)5	#N/A	#N/A	30515230
tG(GCC)6	#N/A	#N/A	30515446
tG(UCC)1	#N/A	#N/A	30515172
tG(UCC)2	#N/A	#N/A	30515229
tH(GUG)1	#N/A	#N/A	30515401
tH(GUG)3	#N/A	#N/A	30515217
tI(AAU)1	#N/A	#N/A	30515138
tI(AAU)2	#N/A	#N/A	30515147
tI(AAU)3	#N/A	#N/A	30515122
tI(AAU)4	#N/A	#N/A	30515272
tI(AAU)5	#N/A	#N/A	30515440
tI(UAU)1	#N/A	#N/A	30515045
tK(CUU)1	#N/A	#N/A	30515193
tK(CUU)2	#N/A	#N/A	30515206
tK(UUU)1	#N/A	#N/A	30515181
tK(UUU)2	#N/A	#N/A	30515428
tK(UUU)3	#N/A	#N/A	30515436
tK(UUU)4	#N/A	#N/A	30515437
tK(UUU)5	#N/A	#N/A	30515438
tL(AAG)1	#N/A	#N/A	30515379
tL(AAG)2	#N/A	#N/A	30515022
tL(CAA)1	#N/A	#N/A	30515091
tL(CAA)2	#N/A	#N/A	30515087
tL(CAA)3	#N/A	#N/A	30515346
tL(CAA)4	#N/A	#N/A	30515444
tL(CAA)5	#N/A	#N/A	30515322
tL(CAA)6	#N/A	#N/A	30515362
tL(UAA)1	#N/A	#N/A	30515089
tL(UAA)2	#N/A	#N/A	30515375
tL(UAA)3	#N/A	#N/A	30514979
tL(UAA)4	#N/A	#N/A	30515017
tL(UAA)5	#N/A	#N/A	30515360
tM(CAU)1	#N/A	#N/A	30515188
tM(CAU)2	#N/A	#N/A	30515145
tM(CAU)3	#N/A	#N/A	30515132
tM(CAU)4	#N/A	#N/A	30515451
tN(GUU)1	#N/A	#N/A	30514997
tN(GUU)2	#N/A	#N/A	30514996
tN(GUU)3	#N/A	#N/A	30514978

tN(GUU)4	#N/A	#N/A	30515455
tP(AGG)1	#N/A	#N/A	30515016
tP(UGG)1	#N/A	#N/A	30514992
tP(UGG)2	#N/A	#N/A	30515157
tP(UGG)3	#N/A	#N/A	30514956
tP(UGG)4	#N/A	#N/A	30515413
tP(UGG)5	#N/A	#N/A	30515412
tQ(CUG)1	#N/A	#N/A	30515296
tQ(UUG)1	#N/A	#N/A	30515257
tQ(UUG)3	#N/A	#N/A	30515118
tQ(UUG)4	#N/A	#N/A	30515271
tQ(UUG)5	#N/A	#N/A	30515270
tR(ACG)1	#N/A	#N/A	30515408
tR(ACG)2	#N/A	#N/A	30515407
tR(CCG)1	#N/A	#N/A	30514970
tR(CCU)1	#N/A	#N/A	30515177
tR(UCU)1	#N/A	#N/A	30515192
tR(UCU)2	#N/A	#N/A	30515102
tR(UCU)3	#N/A	#N/A	30515106
tR(UCU)4	#N/A	#N/A	30514971
tR(UCU)5	#N/A	#N/A	30515223
tS(AGA)1	#N/A	#N/A	30515416
tS(AGA)2	#N/A	#N/A	30515425
tS(AGA)3	#N/A	#N/A	30515415
tS(AGA)4	#N/A	#N/A	30515126
tS(CAG)1	#N/A	#N/A	30515292
tS(CGA)1	#N/A	#N/A	30515036
tS(GCU)1	#N/A	#N/A	30515398
tS(GCU)2	#N/A	#N/A	30515397
tS(UGA)1	#N/A	#N/A	30514963
tS(UGA)2	#N/A	#N/A	30515279
tS(UGA)3	#N/A	#N/A	30515235
tT(AGU)1	#N/A	#N/A	30514998
tT(AGU)3	#N/A	#N/A	30515369
tT(AGU)4	#N/A	#N/A	30515116
tT(AGU)6	#N/A	#N/A	30514952
tT(CGU)1	#N/A	#N/A	30514983
tT(UGU)1	#N/A	#N/A	30515252
tT(UGU)2	#N/A	#N/A	30515278
tV(AAC)1	#N/A	#N/A	30515094
tV(AAC)2	#N/A	#N/A	30515180
tV(AAC)3	#N/A	#N/A	30515380
tV(AAC)4	#N/A	#N/A	30515414
tV(AAC)5	#N/A	#N/A	30515209
tV(AAC)6	#N/A	#N/A	30515210
tV(CAC)1	#N/A	#N/A	30515081
tV(UAC)1	#N/A	#N/A	30515165

tW(CCA)1	#N/A	#N/A	30515064
tW(CCA)2	#N/A	#N/A	30515220
tY(GUA)1	#N/A	#N/A	30515058
tY(GUA)2	#N/A	#N/A	30515183
tY(GUA)3	#N/A	#N/A	30515221
tY(GUA)4	#N/A	#N/A	30515059
C1_04180W_A	1838.009	2.44E-05	3644716
HHT21	687.4192	3.45E-13	3644707
C4_05390W_A	365.7476	0.000913	3646951
HGT12	356.9393	0.00117	3638048
CRH11	259.2182	9.02E-10	3637905
C1_03150C_A	228.3427	0.002216	30514976
ALI1	211.9259	0.002599	3636635
CDC11	202.9542	0.002835	3647888
C2_09590C_A	183.3749	0.003417	3643800
VMA10	168.7248	0.003997	3645215
HRT2	156.5937	1.55E-08	3635709
C3_07840C_A	154.6042	0.004735	3647465
SAM51	153.5386	0.004687	3641582
COG4	152.0432	0.004734	3645571
TNA1	145.2899	0.006371	3637827
C3_02620C_A	141.9118	0.005387	3638326
HHT2	141.278	7.51E-21	3647326
C1_11990W_A	136.0189	0.005713	3642042
HTA2	122.987	6.43E-20	3644715
C6_02140W_A	112.1675	0.007998	3645886
FGR41	109.0029	1.56E-07	3634839
PXP2	103.623	0.010247	3636558
SOU1	96.51473	2.85E-11	3642799
HOM6	96.06665	3.39E-07	3636815
C6_00710W_A	91.24766	0.011193	3647073
PGA38	91.10256	2.25E-13	3637846
C5_03000C_A	89.18306	0.011658	3637818
MLC1	86.9366	0.01214	30515387
C1_13100W_A	86.61008	0.013682	30515073
ECM331	81.09085	0.013749	3637731
CDC10	80.54509	7.4E-07	3640947
ERG28	77.18923	0.014466	3638833
LSC1	76.38602	1.03E-24	3640199
FGR6-10	76.35584	0.014889	3641770
CR_06380C_A	74.58674	0.015345	3648139
CR_05310W_A	74.22469	0.01543	3648125
C6_02180W_A	74.17393	0.015405	3645861
GUP1	73.3657	0.015691	3636171
DPM2	70.25507	0.016958	30515325
CR_10130W_A	68.25876	0.017607	3638970
C3_02140C_A	64.07053	9.18E-13	3636656

C3_06750W_A	61.07263	0.020849	30515219
CR_00280C_A	59.80364	0.021404	3639719
HHO1	59.61921	2.53E-12	30515358
C6_00660C_A	58.96976	0.021812	3647133
C2_02330W_A	58.96065	0.021778	3635811
CR_05210W_A	58.11305	0.022346	3648081
C4_02460W_A	55.97889	0.023544	3637844
DPM1	55.92615	5.24E-06	3640700
CR_04490C_A	55.84606	0.024013	3641013
TUB4	54.69301	0.024324	3641774
BIO2	54.68603	6.59E-06	3640106
C1_11950W_A	54.26734	0.024617	3642045
C3_06730W_A	53.247	0.025616	3638504
ERG11	52.62181	1.7E-16	3641571
FAD1	52.54865	0.026021	3634930
C2_10010C_A	52.14356	0.026197	3643962
PGA6	51.4342	9.52E-06	3634774
ILV5	51.0439	1.68E-28	3644012
HHF1	50.64913	1.77E-25	3644705
C1_10560C_A	50.44964	0.027769	3647196
C6_00400C_A	50.05936	0.028112	3646087
CHT3	49.59319	1.64E-12	3638967
C7_00240W_A	48.81486	0.030285	3637980
POL30	46.53292	2.46E-05	3635583
C2_09960W_A	45.80774	0.031869	3643821
C7_03650W_A	45.79697	0.03188	3646224
C3_07540C_A	45.52861	0.032421	3638526
TUB2	45.23504	4.47E-11	3639329
C3_01100W_A	43.70186	0.033806	3639834
C2_05720C_A	42.18395	6.34E-11	3645574
ARC18	41.13062	0.037203	3644056
ACP12	40.92523	3.17E-05	3637361
SOL3	40.84536	8.92E-11	3646625
IFE1	40.53337	0.038312	3644778
C1_07920W_A	40.28704	0.038256	30515026
C7_01750W_A	40.18398	0.03912	3637009
CCN1	40.05794	0.038887	3643735
RBE1	39.93976	0.039342	3643317
CWH43	39.35949	2.27E-10	3640389
RFA2	39.04335	5.05E-05	3643506
C1_06700W_A	38.29284	4.22E-05	3639511
C1_02100W_A	38.06428	0.04152	3644578
HHF22	37.97656	6.84E-26	3647327
SNZ1	37.85121	4.79E-10	3636803
C1_03600W_A	37.26534	4.86E-05	3636880
C2_05510C_A	37.23845	0.042502	3643420
AGM1	37.1082	5.15E-05	3636138

C4_02600C_A	36.92031	0.042839	3637912
C7_01100C_A	36.8408	0.043711	3637948
LEU2	36.69563	7.47E-09	3638034
OST1	36.59624	5.41E-05	3643657
THR1	35.67744	6.16E-05	3641572
C1_03940W_A	35.65093	2.04E-07	3646415
CWH8	35.22868	0.045807	3644598
PHA2	35.21192	0.045825	3638346
YNK1	34.72237	4.59E-15	3637746
PMI1	34.70729	1.87E-07	3643795
C1_09060C_A	34.30913	7.31E-05	3634772
C1_07490C_A	33.6558	0.049359	3646666
ATP18	33.52918	8.07E-05	30515084
C1_06050C_A	33.36543	8.24E-05	30515003
PGA45	33.15621	0.000108	3639875
PHO15	32.46646	1.2E-13	3644459
ERG6	32.4299	7.49E-12	3636655
CYT1	31.6298	3.65E-19	3643441
ATP19	30.76045	0.000116	30515061
CR_03000C_A	30.26503	0.000118	3642597
HTA1	29.87498	1.59E-20	3645658
ATP1	29.82967	1.37E-45	3646134
SHE3	29.64287	0.000137	3639277
CR_03470W_A	29.29306	0.000147	3643198
ERG26	29.26096	4.01E-09	3642803
CDC28	27.41372	0.000193	3644838
QCR2	26.23998	7.16E-19	3646352
C2_07140W_A	26.09914	8.14E-14	3643508
ERV46	25.96754	2.37E-06	3644129
CR_09330C_A	25.89895	1.55E-17	3641899
C2_02050C_A	25.54991	2.81E-06	3635851
TAL1	25.53146	9.72E-37	3643225
TUB1	25.37593	3.83E-15	3641930
C5_02380W_A	25.36484	2.31E-44	3637794
PHR1	25.07557	1.35E-35	3641108
GND1	24.47076	4.8E-38	3636131
C1_14320C_A	24.32981	0.00038	3643316
PDI1	24.24315	1.29E-21	3641367
C5_02920W_A	23.93852	0.000369	3637751
HAT1	23.58362	3.39E-06	3644770
SEC21	23.10166	1.57E-15	3644786
C2_07540W_A	22.78709	0.000503	3645222
C4_03170W_A	22.73576	0.083578	3637840
LSC2	22.22575	1.28E-28	3646618
CR_06170W_A	22.10278	0.000519	3644865
CR_03330W_A	21.97744	0.000537	3645095
C1_02760W_A	21.9228	1.71E-09	3636870

CR_05500C_A	21.86455	0.000594	3647598
AAP1	21.76539	6.84E-08	3647397
DDR48	21.76286	1.79E-14	3644061
TSA1	21.44504	1.2E-28	3642269
DOG1	21.31599	0.000602	3641597
APE3	21.14587	8.7E-08	3643875
PMM1	21.11455	3.19E-09	3636852
FRP2	21.00679	3.94E-09	3638008
CR_04760C_A	20.94452	8.53E-06	3647303
ASH2	20.85535	0.000689	3636445
ERG13	20.74262	1.4E-15	3641881
LAT1	20.62469	1.14E-27	3636986
ZWF1	20.36406	9.16E-22	3634913
IDP1	19.66598	2.71E-12	3647474
CYB5	19.61352	1.3E-05	3637965
C1_07260C_A	19.50037	0.000858	3644456
COX5	19.36686	1.16E-15	3634768
SPE3	19.22154	4.17E-13	3643528
QCR8	19.11081	4.86E-11	30515112
ACS2	19.07977	8.65E-33	3644710
AMO2	19.06399	3.93E-06	3648021
CDC19	18.92144	2.99E-39	3643438
EMP24	18.91416	1.94E-05	3647247
C5_01230C_A	18.87226	4.53E-11	3642618
C1_04430C_A	18.86443	0.000939	30514986
TSA1B	18.80112	1.34E-26	30515214
PST3	18.63648	1.48E-20	3648031
C2_07650C_A	18.60677	1.73E-05	3642342
SSR1	18.47764	5.76E-13	3638055
C2_03970W_A	18.38394	0.001036	3637419
LEU1	18.31934	1.04E-24	3639685
CR_03520C_A	18.25413	7.96E-11	3643203
ATP3	18.21657	1.05E-21	3640387
PMT2	18.20375	1.46E-15	3638538
C2_03020C_A	18.17033	2.11E-21	3643019
CBR1	17.99706	5.6E-10	3646961
RPS17B	17.93804	4.12E-21	30515044
C6_02190C_A	17.89396	2.18E-05	3645862
EAF6	17.75288	0.001221	3646494
RFC5	17.74404	0.001254	3638890
CDC21	17.73972	0.001329	3643463
CHO1	17.6637	1.7E-08	3644557
C4_00710W_A	17.56656	2.17E-05	3645750
C4_01510W_A	17.55835	1.14E-09	3635616
CAR1	17.53611	2.09E-17	3636503
ALD5	17.32618	2.71E-32	3643026
CHT2	17.3235	1.12E-12	3636523

CR_00990W_A	17.31264	0.001407	3640425
PHO8	17.28495	4.98E-05	3647179
C4_00390W_A	17.25032	2.93E-05	3645016
MDH1-1	17.18595	3.37E-28	3635636
ASK1	17.17152	0.001671	3635656
C1_01190C_A	17.16693	2.61E-05	3640207
SCW11	17.11685	3.36E-15	3636521
C6_02560W_A	16.94474	5.28E-11	3639205
MIS11	16.87701	1.78E-21	3645732
ERG10	16.87007	1.93E-19	3648275
C2_07010W_A	16.77273	2.9E-11	3643535
CAT1	16.68428	6.41E-27	3639495
PLC2	16.59256	1.16E-06	3642985
SEC22	16.43395	0.00171	30515391
EGD2	16.41417	9.79E-18	3635284
RPL24A	16.26565	2.01E-21	3641174
CLB4	16.08615	5.76E-05	3643102
TOS1	15.97822	2.26E-20	3636566
LYS9	15.83943	7.26E-09	3642279
CYP1	15.83687	6E-23	3636978
C2_09040W_A	15.7738	4.45E-09	3645792
DOA1	15.75416	8.22E-14	3634871
RAD54	15.72087	0.002036	3636192
OSH3	15.66866	8.85E-11	3645368
C5_02220C_A	15.40786	0.002213	3637760
C4_03050C_A	15.37045	7.8E-11	3637930
C1_05180C_A	15.206	0.00228	30514990
SWI6	15.19313	0.002268	3634932
HGT3	15.09436	0.002311	3643185
C2_09100C_A	15.04437	1.49E-08	3644113
RGD1	15.0054	3.16E-06	3639868
C5_04410C_A	14.96721	3.25E-06	3636410
C1_09980C_A	14.93896	7.92E-05	3634863
ATP2	14.84413	4.31E-35	3645039
ERV25	14.76595	2.46E-07	3642243
HIS1	14.71901	1.81E-09	3636501
CR_03950W_A	14.71511	0.002664	3640960
LAP41	14.7109	1.92E-13	3636653
ACO2	14.66116	5.05E-06	3646708
C1_06920C_A	14.57765	0.002669	3639525
BGL2	14.5723	2.01E-19	3635691
TRP4	14.45655	0.000101	3642904
SEC23	14.41655	3.04E-16	3641818
COF1	14.35135	1.35E-15	30515293
RET2	14.31734	5.33E-11	3637714
C7_01710W_A	14.26044	3.28E-11	3637012
PDX1	14.26037	2.51E-19	3636128

RBT4	14.20971	4.46E-09	3639534
C2_07550W_A	14.19059	5.64E-13	3645221
SGA1	14.08709	5.16E-07	3636652
C1_00700W_A	14.0716	0.00294	3639330
VMA2	14.01203	8.43E-20	3646710
NHP6A	14.01073	5.24E-19	30515244
HTA3	13.9788	6.48E-07	3638322
MCR1	13.97497	1.52E-20	3647561
C2_06770W_A	13.89158	0.003171	3643515
TRX1	13.78433	5.19E-16	3638950
EGD1	13.76647	1.04E-16	3645340
CR_05360C_A	13.76533	0.00318	3648033
OPI3	13.76344	0.003179	3642277
PDB1	13.75554	1.2E-23	3641186
C6_03050C_A	13.71845	0.00013	3639270
C2_06890C_A	13.62791	0.000193	3643522
KTR4	13.61894	0.003509	3646425
HWP1	13.51326	0.003815	3645372
C1_03240W_A	13.46082	6.81E-06	3636895
C2_02410W_A	13.3515	3.58E-11	3635755
CWH41	13.34932	1.95E-08	3646533
FAS2	13.33568	7.28E-33	3635222
RPP2A	13.286	2.07E-13	30515439
C6_03130W_A	13.27253	8.84E-06	30515341
C2_09050C_A	13.13027	7.86E-07	3645791
C6_04100W_A	13.11986	4.87E-11	3647836
C2_00590W_A	13.05847	0.000196	3638851
C1_10310W_A	13.04766	0.003809	3634906
ERG24	12.99919	3.81E-08	3647716
DAP1	12.9599	9.68E-06	3641021
C7_00670C_A	12.9564	3.74E-08	3638024
C5_05020C_A	12.9287	8.94E-07	3636486
CR_08480C_A	12.89856	7.05E-09	30515442
DAG7	12.84864	8.06E-08	3635607
CMP1	12.83466	4.87E-08	3639328
SEC13	12.8303	2.85E-11	3638425
ACT1	12.8107	5.71E-31	3636195
RPS15	12.76949	4.95E-19	3635194
LEU4	12.74658	3.86E-05	3639339
CHS1	12.73321	7.17E-08	3641364
RFA1	12.62997	1.84E-12	3638823
C1_12650C_A	12.62905	0.004468	3646836
SRB1	12.62123	2.91E-24	3647454
C4_04600C_A	12.58514	0.00022	3641156
HMG1	12.56945	2.16E-12	3644687
ADK1	12.55946	1.28E-14	3644533
C1_09020W_A	12.38657	1.33E-07	3634917

CR_01490C_A	12.35493	1.07E-05	30515376
SAC1	12.34319	1.14E-06	3634864
SER33	12.31887	3.64E-17	3642030
CR_08610W_A	12.27599	4.34E-07	3642109
ERP5	12.25139	1.79E-06	30515046
C5_00080C_A	12.15978	8.65E-11	3647886
ERG2	12.15374	0.005599	3639347
C1_04200C_A	12.12938	1.26E-05	3644718
C5_02110W_A	12.08456	4.75E-23	3637732
ADE1	12.05989	3.08E-13	3639722
SEC20	12.02082	0.000282	3639206
C1_14060W_A	11.99738	7.47E-16	3643344
FRS2	11.98153	3.46E-16	3636824
ARP3	11.96765	1.37E-14	3643479
HSP12	11.92655	1.7E-22	3643745
C3_01070C_A	11.92014	0.005767	3639832
RPL8B	11.90277	1.1E-20	3635198
C6_02480W_A	11.80705	4.29E-05	3639170
ACO1	11.79003	1.29E-26	3642090
PGA63	11.69737	9E-19	3639524
TEF2	11.65805	1.79E-31	3646502
ARC35	11.61564	8.49E-09	3639823
C4_04970C_A	11.61297	2.83E-05	30515274
POL1	11.50899	9.17E-07	3635144
RKI1	11.44311	0.000417	3636574
C1_01080W_A	11.40988	0.006784	3640173
IDH1	11.36372	1.53E-24	3634893
C4_01840C_A	11.34004	5.51E-09	3635592
SMC1	11.30749	0.000487	3643221
PMT4	11.30533	5.34E-11	3644096
HXK2	11.29445	8.59E-30	3641015
PGA10	11.27947	0.000416	3645009
SEC12	11.24843	9.97E-11	3641639
IPP1	11.23418	1.65E-20	3643874
HYR1	11.19754	0.007094	3636226
C5_05200C_A	11.16652	5.27E-07	3636467
ACB1	11.13361	2.29E-08	30515350
HGT13	11.09998	0.009139	3638047
HTB1	11.08604	5.93E-22	3645659
HIS5	11.07781	0.000463	3645756
ASN1	11.04034	2.74E-17	3645790
CRC1	10.89127	1.47E-10	3640112
ARE2	10.87502	9.47E-13	3643526
C1_12180C_A	10.81329	4.82E-05	3642038
SSA2	10.68246	4.44E-15	3644711
CAM1-1	10.64827	2.83E-15	3646358
C1_11760C_A	10.59087	4.75E-06	3645328

C1_02500W_A	10.58613	0.00826	3636795
APL4	10.57854	1.54E-09	3642565
C4_07080C_A	10.5631	0.000535	3642905
CR_10530W_A	10.54734	0.008554	3639042
DUT1	10.53783	7.26E-05	3640188
CAT2	10.52959	2E-16	3635659
C1_07590C_A	10.48875	5.28E-06	3646673
CR_09670C_A	10.4243	1.07E-09	3647091
LYS12	10.40542	3.21E-14	3640134
C1_00420W_A	10.39157	8.9E-11	3639286
C4_00020W_A	10.37825	1.25E-11	3648053
SAR1	10.37601	7.06E-09	3645865
RIP1	10.34997	3.24E-15	3635204
ERV29	10.34737	7.43E-07	3635672
SEC18	10.31585	4.57E-14	3636208
MDH1	10.29508	1.78E-18	3639667
BMT4	10.26576	7.93E-07	3639182
C6_02880W_A	10.23887	0.010976	3639238
PDA1	10.23438	6.69E-26	3642902
GLC3	10.20591	1.34E-17	3639200
CR_03270W_A	10.19432	6.7E-05	3645101
C5_04550W_A	10.18278	6.99E-05	3636508
NUC2	10.15506	1.28E-14	3637030
PIL1	10.1486	8.59E-28	3644769
C1_11960C_A	10.13518	0.010331	30515060
NUO1	10.1285	2.17E-07	3647100
HET1	10.08937	2.08E-10	3647897
MSH6	10.05593	2.14E-06	3636161
GRP1	10.01927	0.000874	3634899
C2_00180C_A	10.01756	2.6E-06	3638905
SUB2	10.01	4.08E-17	3645033
MCM6	9.958443	0.000104	3640142
CR_09230C_A	9.953533	8.1E-05	3641918
QCR7	9.952857	5.68E-09	3639233
C3_06860C_A	9.945282	6.38E-15	3638541
C1_13270W_A	9.927411	1.54E-06	3636144
C5_01960C_A	9.888138	1.53E-10	3643738
CEX1	9.796031	8.84E-05	3644913
C2_02020W_A	9.773477	8.18E-10	3635769
ATP5	9.730191	4.73E-17	3640521
DED81	9.719106	3.23E-17	3646227
ADE6	9.703398	3.74E-17	3647304
CR_00130C_A	9.691789	1.49E-08	3639709
HGT2	9.63479	8.38E-15	3644579
CR_04860C_A	9.606546	0.012276	3647294
C1_04780C_A	9.590933	8.76E-06	3644774
HOF1	9.563019	0.0002	3645014

C2_06610C_A	9.556247	0.012088	30515140
CLC1	9.526619	2.28E-10	3635662
C3_03510C_A	9.519302	2.97E-08	3638310
C5_04950C_A	9.51792	1.73E-06	3636422
SDH12	9.482635	5.05E-19	3642775
GVP36	9.479023	2.35E-06	3641772
ABG1	9.475219	6.07E-07	3647715
HGT10	9.459689	2.7E-08	3640309
C2_05290C_A	9.41889	0.000145	3643472
EXG2	9.382717	0.000159	3636816
VMA8	9.378104	9.59E-09	3642798
MET15	9.365122	9.76E-11	3645031
C2_10200W_A	9.35755	0.012913	3643928
CR_05130C_A	9.353852	0.01266	3646401
C1_07510W_A	9.348398	0.001071	3646664
SEF2	9.348245	0.001067	3647052
MTS1	9.299234	3.58E-13	3634873
ALS4	9.29918	3.3E-16	3647972
SSH1	9.298413	9.36E-07	3642504
INP51	9.27662	1.2E-05	3643806
LYS4	9.259959	6.87E-06	3641180
RPS12	9.218562	1.1E-15	3638438
C5_02210W_A	9.210499	3.01E-06	3637759
PDC11	9.199961	7.14E-22	3642780
CDC5	9.196171	1.8E-10	3639281
C1_10520W_A	9.179259	0.014305	3647191
MET6	9.146335	2.39E-21	3640084
RIC1	9.139621	0.000148	3639331
FTR2	9.112545	7.84E-10	3643303
C1_01800W_A	9.111195	0.00131	3645407
LIP6	9.107916	0.014414	3634890
STV1	9.102005	1.53E-14	3646070
C7_04000W_A	9.079604	3.17E-06	3643114
ATP17	9.030875	4.04E-12	30515364
ECM33	8.989139	2.55E-18	30514977
C6_03320W_A	8.984097	0.000227	3639198
RPL43A	8.981992	5.37E-15	30515319
C2_02310W_A	8.965554	2.55E-07	3635809
CRD2	8.944269	0.014348	30515234
CAP4	8.929689	0.001445	3637335
MSC7	8.920142	8.23E-11	3645214
CR_10140W_A	8.886184	4.71E-16	3638972
MPS1	8.844632	0.015309	3641888
VMA11	8.839008	2.17E-05	3637054
GDH3	8.810927	5.21E-19	3648086
C7_02100W_A	8.805456	5.91E-14	3637023
PRN4	8.773161	2.66E-05	3639877

ILV6	8.763517	1.52E-09	3635598
RTT109	8.761953	0.016201	3639678
C7_02520W_A	8.725516	0.016478	3636941
NIF3	8.712736	5.14E-06	3646527
C2_02260W_A	8.67693	8.39E-07	3635778
CDC12	8.676501	2.27E-10	3636809
ADH2	8.6686	2.44E-20	3640751
MSB1	8.660026	5.58E-06	3648218
JEM1	8.627393	0.00181	3643876
PTP3	8.607839	7.37E-15	3638940
HOM3	8.570892	1.53E-05	3641771
ENA21	8.557637	7.05E-23	3641392
C5_03120W_A	8.533618	0.016998	3637705
MCM2	8.530397	0.016971	3643197
GLO2	8.527836	1E-06	3644067
C1_11280W_A	8.514242	9.04E-09	3644554
VCX1	8.500969	7.85E-07	3646508
C4_03290W_A	8.481667	1.84E-09	3637887
DPB4	8.442559	0.000266	3638818
C5_05230C_A	8.429344	8.84E-17	3636470
CR_04880W_A	8.424807	4.39E-05	3647309
C6_02530C_A	8.415596	0.000279	3639175
SEP7	8.377203	3.42E-05	3644596
ACC1	8.340154	2.04E-24	3639688
C6_02250W_A	8.330526	0.01863	3645869
C1_02330C_A	8.315534	1.56E-14	30514969
C4_06160W_A	8.296779	0.001963	3642819
C1_12400C_A	8.258471	6.2E-05	3642059
C6_00930C_A	8.245865	3.14E-09	3644032
C3_01020W_A	8.242534	0.002082	3639828
TVP18	8.197944	0.000342	3634844
HSL1	8.187977	6.32E-08	3637810
NDE1	8.179892	2.03E-22	3638354
C4_01460C_A	8.158487	0.002268	3635622
LSP1	8.141147	8.25E-19	3648025
ARF3	8.135405	0.020972	3636628
RPS7A	8.117182	3.73E-15	3636573
SER1	8.110336	3.52E-10	3646909
HIS7	8.103035	4.42E-06	3647868
CR_01100C_A	8.077892	0.023063	3640378
C3_06920W_A	8.076741	1.97E-06	3638511
PEX6	8.032538	1.97E-09	3643436
CR_05800C_A	8.020212	0.021308	3646706
SCP1	8.018936	0.021328	3646554
OSM2	8.017323	2.22E-09	3636193
ASR1	8.01293	1.31E-17	3644402
VPS51	8.00038	0.000358	3639242

C2_05860C_A	7.998921	0.023943	3647473
APL2	7.995099	1.39E-07	3638343
LYS1	7.984475	1.67E-07	6335429
C4_04800W_A	7.940051	1.03E-11	3641113
C6_03960W_A	7.936324	3.68E-14	3640269
C1_04030W_A	7.932285	0.0004	3646428
SNF5	7.917592	3.88E-06	3648297
CR_10440W_A	7.916025	0.021795	3638976
ERG5	7.88777	1.96E-15	3641400
APM4	7.884545	0.000431	3642297
SDH2	7.873263	8.71E-12	3646397
TPI1	7.864224	1.01E-23	3638482
ATP4	7.8503	1.5E-15	3643418
CR_00910W_A	7.825181	1.81E-12	30515371
PRD1	7.813247	4.04E-15	3642471
CAS1	7.802475	0.000398	3645324
MDH1-3	7.800968	1.13E-09	3641197
RPP1A	7.800917	2.92E-14	3636839
MMD1	7.792773	4.84E-14	30515015
AAT1	7.791468	2.58E-14	3643468
C4_04160W_A	7.767464	2.19E-12	3641185
C4_03400W_A	7.766041	0.00316	30515256
CUP5	7.765994	5.29E-10	3635174
PEX8	7.750729	7.44E-05	3647393
GDE1	7.750483	9.1E-09	3636505
AHP1	7.740095	3.84E-19	3637850
CR_04850C_A	7.725135	0.02515	3647295
MNS1	7.71835	3.13E-07	3644692
C1_02650W_A	7.715827	6.09E-09	3636818
NDH51	7.691366	1.32E-13	3645444
C1_05670W_A	7.688274	1.96E-10	3639899
RNR1	7.682349	4.43E-09	3642989
C1_02700C_A	7.672419	1.56E-12	30514972
C3_01610W_A	7.668465	8.95E-14	3636664
C2_06260W_A	7.665574	0.26227	3646913
ERG9	7.659297	1.3E-11	3643913
C2_01260W_A	7.651946	0.002791	3638792
GCV1	7.64758	1.14E-09	3639172
VMA5	7.636608	3.45E-07	3642316
CR_09010C_A	7.612967	1.52E-13	3641892
C6_04280W_A	7.567113	3.27E-08	3646279
BMH1	7.555297	1.92E-19	3636810
TPD3	7.546685	7.84E-10	3638512
HGT1	7.5446	1.56E-22	3645420
C1_05490C_A	7.533166	5.79E-07	3642507
C7_03350C_A	7.518639	0.029762	3646722
PEX11	7.51636	0.000117	3646288

C4_04920W_A	7.492965	0.000107	3641172
KIP1	7.480809	0.003329	3645261
MEU1	7.457566	1.6E-05	3645635
MNT1	7.453139	2.5E-16	3636585
CPR3	7.449862	3.57E-10	3635812
SOD1	7.421232	2.59E-05	30515248
TOM20	7.402684	1.49E-06	3636817
DPP2	7.360321	0.003333	3645341
C6_02030C_A	7.35537	1.35E-08	3647562
C1_04460C_A	7.336242	0.000116	3646140
GIN4	7.324827	2.5E-05	3644543
POR1	7.320476	3.45E-20	3644698
SMC2	7.320295	0.000144	3643918
C1_12240C_A	7.30542	7.3E-06	3642023
ARC40	7.302213	3.05E-10	3644866
CIT1	7.256125	1.25E-16	3643201
PYC2	7.237156	3.31E-15	3637323
C3_00520W_A	7.229116	0.003898	3640513
C2_03210W_A	7.18398	0.003984	30515098
C1_08800W_A	7.179433	4.16E-06	3634791
SHM2	7.152145	9.39E-19	3640306
KGD2	7.138535	2.32E-13	3646036
CYK3	7.122176	0.000747	3639539
CSR1	7.099592	0.000679	3641162
CR_09560C_A	7.096446	0.030543	3647099
C1_03490W_A	7.087974	6.72E-07	3636914
GLO1	7.07578	1.03E-09	3639312
C4_02420C_A	7.069668	0.031899	3637849
PMT1	7.063282	1.3E-12	3641393
C1_08080C_A	7.037117	3.13E-06	30515029
CR_07230W_A	7.010151	1.37E-06	3646031
CSM3	7.001523	0.033983	3644083
C4_06140C_A	6.99482	0.031327	3642821
C1_01950C_A	6.975805	0.000801	3645421
PST2	6.972634	0.001248	3643909
FRE30	6.906975	3.97E-13	3646025
STT3	6.906323	5.44E-09	3635743
C1_08730W_A	6.895941	0.000855	3634938
C7_00230W_A	6.876814	0.000253	3637982
AHP2	6.852312	3.5E-06	3636977
GLT1	6.840914	7.56E-18	3639519
TPO3	6.839913	1.31E-18	3634790
BFR1	6.833918	1.14E-12	3635579
CAM1	6.796194	1.75E-17	3641734
HNT2	6.789456	0.034447	3642271
C1_02890C_A	6.787293	0.006354	3636867
ALG8	6.783245	0.00576	3636562

TFS1	6.780277	4.72E-14	3642655
FRE7	6.779014	6.07E-16	3646024
VMA7	6.772082	0.001065	3637332
NPT1	6.761929	3.04E-08	3643109
MES1	6.755443	3.99E-12	3636516
CR_01320C_A	6.706802	1.09E-05	3640386
RPL10A	6.70306	1.88E-17	3645868
CR_04770C_A	6.688718	0.036892	3647302
FBA1	6.682164	4.23E-19	3635585
C1_00450C_A	6.673701	5.55E-06	30514955
FMA1	6.66779	0.040017	3647986
PDR17	6.665375	0.007274	3643007
C2_05560W_A	6.653168	0.001099	3645561
C6_00770C_A	6.648109	0.038196	3647079
C4_06460C_A	6.633094	0.006604	3642791
CPY1	6.617944	7.12E-14	3646721
C1_14240W_A	6.615953	3.6E-09	3643306
RPP1B	6.613034	2.74E-11	3643104
EXO1	6.612015	0.001172	3641583
C6_03950C_A	6.588619	0.000308	3640268
C4_00770C_A	6.5855	6.21E-06	3645760
TOP2	6.58452	3.08E-06	3642777
FAA21	6.560447	9.66E-08	3638418
C3_00210C_A	6.550568	0.007573	3640560
CRN1	6.550025	2.35E-09	3637034
TOS4	6.517065	2.89E-08	3644548
C2_05170W_A	6.516457	1.33E-05	3643462
C1_01010W_A	6.51618	2.79E-06	3640181
C1_01050C_A	6.510054	4.3E-08	3640170
RNH35	6.478249	0.040537	3636987
C2_09780C_A	6.474852	3.8E-06	3643805
FUM11	6.463995	6.66E-11	3641016
DPM3	6.462668	0.007387	30515245
CR_06770C_A	6.461552	0.0001	3647313
C1_11860W_A	6.45925	1.1E-11	3642009
C3_07230W_A	6.449655	1.87E-06	3638531
DUR1,2	6.434535	3.53E-07	3644771
HEM14	6.431241	2.28E-06	3634923
ASM3	6.427277	0.00723	3639332
C3_04420W_A	6.405807	9.66E-12	3635180
CR_05200C_A	6.402248	0.000336	3648080
C3_05290C_A	6.388873	0.008138	3635203
CR_03560W_A	6.358117	0.00803	3643217
C3_01510W_A	6.335304	2.26E-05	3636571
HBR2	6.329786	5.81E-12	3646273
YSA1	6.326478	8.5E-06	3638516
CR_09520C_A	6.315515	0.007797	3647103

CR_05760C_A	6.314525	0.007741	3646695
RAD53	6.31403	0.007725	3645633
SKN1	6.306224	2.61E-12	3641755
C1_10750C_A	6.289134	0.009024	30515043
C5_05350W_A	6.287215	0.000528	3636401
CR_02260C_A	6.284092	0.000458	3644912
C1_03170C_A	6.277787	0.044352	3636805
ASE1	6.271207	0.001781	3641729
C1_03720C_A	6.267331	1.25E-06	3648216
CR_00060C_A	6.256668	2.09E-08	3639673
IFA4	6.193483	0.008321	3645454
C5_04420W_A	6.185616	2.72E-05	3636411
AGE3	6.175083	8.39E-07	3644565
SPC34	6.174908	0.045739	3641173
C2_06750C_A	6.173299	0.045157	3648023
CR_05680C_A	6.169514	0.000538	3646684
PUP2	6.163621	1.19E-09	3646617
C2_07420W_A	6.155076	2.95E-05	3645202
SMF3	6.139635	5.9E-08	3638852
RPS26A	6.138237	2.3E-13	3635787
C7_03310W_A	6.1373	0.00859	3646726
C5_00340W_A	6.136474	5.75E-05	3641555
MET13	6.131471	1.07E-06	3638382
RPL18	6.127428	1.75E-15	3635156
C4_00380W_A	6.107242	0.000202	3645015
DUN1	6.105266	0.001924	3636494
C5_01550C_A	6.096026	1.34E-10	3647063
IST2	6.088737	1.6E-10	3646662
RPS8A	6.085989	5.77E-15	3645565
LHS1	6.083757	1.26E-09	3643032
RTS1	6.079976	2.62E-12	3646557
FAS1	6.07583	2.91E-18	3641593
ATP14	6.05898	1.02E-08	30515134
SEC8	6.049678	3.02E-06	3647078
C1_00210C_A	6.049395	3.67E-12	3639335
CR_09590W_A	6.032873	1.19E-05	3647095
CDC42	6.032377	1.23E-05	3646488
C3_03270W_A	6.020923	0.008905	3638321
C3_04080W_A	6.020881	6.3E-10	30515200
C1_02290C_A	6.019467	0.000647	3644568
C6_01750C_A	6.018196	0.009163	3641640
C3_02820C_A	6.015497	6.98E-06	3638419
CR_00690C_A	6.006395	0.000155	3640390
SPC3	5.992998	0.000512	3636121
CR_02620C_A	5.978234	3.17E-06	3642567
CR_06070W_A	5.976799	7.95E-06	3644840
TSC11	5.961847	0.009186	3645267

DSE1	5.961064	2.84E-07	3643899
FESUR1	5.94994	1.37E-09	3638514
CAR2	5.943874	2.25E-13	3645027
C1_07340W_A	5.943268	1.26E-09	3644472
RPS1	5.934958	1E-16	3636924
C1_01290C_A	5.906609	0.009868	3640184
RAM1	5.905462	0.009814	3647859
C3_04810C_A	5.891638	1.94E-14	30515205
ZCF5	5.864084	0.000637	3641819
RHB1	5.852705	0.011186	3635232
TMA19	5.83485	3.69E-13	3640399
ERG251	5.816651	2.37E-15	3635716
SAH1	5.81624	1.85E-15	3636452
C1_02780W_A	5.81169	6E-08	3636871
APM1	5.810877	7.58E-07	3636424
ADE5,7	5.802585	3.07E-13	3640719
HXT5	5.797631	0.000161	3643209
CMK2	5.781838	9.37E-09	3643961
RAD10	5.773529	0.003184	3639884
EFB1	5.742462	1.08E-14	3641125
CR_05010W_A	5.727934	0.011821	3646389
SNL1	5.716529	9.55E-06	3647192
C2_08930W_A	5.701649	1.31E-08	3645795
RPL28	5.690551	1.4E-13	30515386
C5_01410C_A	5.68769	3.35E-06	3647054
SAC6	5.652498	2.63E-14	3639213
AMO1	5.650111	0.000759	3642994
NPL4	5.647093	1.92E-05	3639820
SOD2	5.646145	1.4E-08	3640231
AYR1	5.629517	0.000357	3647754
ACS1	5.62452	1.96E-13	3643941
CR_04870C_A	5.621806	0.018734	3647310
C7_03640C_A	5.611657	0.012997	3646223
C1_10540C_A	5.582762	0.000768	3647193
C3_02570W_A	5.578936	6.03E-06	3638375
C2_03340W_A	5.57542	2.49E-05	3637392
C2_07980W_A	5.568084	0.000506	3642312
C1_01250W_A	5.561431	3.09E-06	3640212
ARP2	5.553459	4.76E-11	3641915
RPL19A	5.549315	1.47E-14	3635257
ARO4	5.546603	5.73E-11	3647668
DQD1	5.543162	2.78E-05	3643488
ARO3	5.52984	3.74E-06	3635770
RPS16A	5.517098	1.89E-12	30514974
GLO3	5.496334	3.19E-12	3640556
PMT5	5.490781	0.000416	3639011
VPS1	5.487047	6.89E-12	3642656

TFP1	5.472819	3.13E-11	3636662
ARC19	5.471655	0.003313	3640418
CEK1	5.468878	0.003362	3642789
C1_02270C_A	5.466904	3.36E-07	3644566
C4_06810C_A	5.457723	3.4E-09	3642916
COX6	5.455292	1.55E-12	30515099
CYB2	5.452434	7.11E-06	3636213
C5_05040W_A	5.445535	0.00107	3636488
C7_00600C_A	5.445448	0.00103	3638031
GRS1	5.431191	5.54E-13	3642474
C2_06950C_A	5.423927	0.004328	3643527
DSL1	5.404437	0.003725	3645718
RPL35	5.383264	7.43E-12	30515207
C1_10730W_A	5.368796	7.24E-06	3644404
C4_04140W_A	5.368423	0.001162	3641220
ABP1	5.359814	1.01E-12	3637875
TYR1	5.347187	3.88E-05	3635639
C4_00910C_A	5.331697	0.004047	3635679
C3_06400C_A	5.325917	0.018652	3642260
VMA13	5.298794	2E-08	30515402
RAP1	5.290834	4.72E-05	3643945
YBN5	5.289948	1.47E-11	3644781
C2_01930C_A	5.269838	2.02E-07	3635793
APT1	5.264679	1E-05	3635842
C6_00090W_A	5.262646	0.01869	3647898
FUM12	5.242811	1.07E-09	3638489
COQ3	5.226264	2.26E-05	3641630
C6_01360W_A	5.210236	0.001375	3641608
DOT1	5.205295	1.28E-05	3642236
RAD52	5.19075	0.001691	3647148
C3_05300C_A	5.141999	0.001637	3643646
THR4	5.138371	1.55E-10	3637711
C6_02470W_A	5.13064	3.13E-06	3639169
C2_07110C_A	5.117042	0.00066	3643505
DBF2	5.108505	1.87E-05	3648101
SUN41	5.106095	7.16E-13	3647067
RPL20B	5.102767	3.66E-11	3635615
IDH2	5.098195	4.84E-12	3642979
APL5	5.074075	4.6E-06	3638377
C1_07990C_A	5.056838	6.44E-10	3640697
GPX3	5.033956	0.001869	3644471
C1_06630W_A	5.033842	0.001882	3639544
TRP99	5.023769	1.05E-05	3642494
GLR1	5.022126	1.46E-10	3647061
CR_01080W_A	4.998284	8.52E-08	3640381
C7_01320W_A	4.988729	2.77E-05	3645896
SSB1	4.981428	7.25E-14	3642095

FCY2	4.977917	2.98E-05	3638365
UBA2	4.977542	8.81E-05	3640701
C1_02910C_A	4.971844	0.019995	3636829
RFC3	4.96747	0.006506	3647440
MUM2	4.954608	7.09E-09	3636432
VPS23	4.943061	0.005874	3644401
C3_00850C_A	4.941521	4.52E-12	3647748
C7_03820C_A	4.940548	0.08332	3643148
C3_02210C_A	4.925435	0.000101	3636641
PEX1	4.915592	2.41E-07	3636943
SPF1	4.915234	2.47E-09	3646573
CR_07110C_A	4.914138	0.021542	3648224
KAR2	4.911254	1.55E-12	3638830
MCI4	4.904653	3.22E-05	3640118
ECI1	4.899577	1.69E-05	3642128
PET9	4.896564	4.26E-14	3641587
MVB12	4.893855	0.002175	30515400
BUB1	4.886884	3.45E-05	3637922
POT1	4.881967	3.19E-09	3639707
C4_04440W_A	4.881063	3.58E-05	3641177
RPS14B	4.874681	5.1E-10	30515006
C1_02970W_A	4.871933	1.02E-07	3636835
C2_04480W_A	4.866503	0.000119	3645464
C1_00330C_A	4.86456	0.000336	3639296
C2_05130W_A	4.860908	0.002471	3643458
C2_01160W_A	4.855745	0.001079	3638861
END3	4.851561	2.69E-05	3636644
ADE13	4.84921	8.5E-13	3644863
CR_08450C_A	4.847181	0.000436	3642133
SHM1	4.846876	1.24E-10	3646724
ANB1	4.844189	3.24E-11	3641648
ZRT1	4.836115	0.024951	3642970
ATP7	4.831249	3.1E-07	3646672
CDC46	4.824044	0.008623	3646912
C2_07270W_A	4.817535	0.000191	3643474
ESP1	4.815058	0.001115	3640197
HEM13	4.812616	1.07E-11	3647390
C1_12880C_A	4.810871	0.000115	3636151
CR_07120C_A	4.807038	0.009764	3648225
CLB2	4.805404	3.17E-07	3635840
YCP4	4.802347	3.93E-11	3648032
ZCF24	4.79986	0.002743	3645417
C4_01470W_A	4.797172	0.02391	3635621
ADO1	4.792085	6.89E-08	3639274
HYU1	4.790267	1.86E-07	3643024
SHP1	4.7879	9.63E-08	3640083
PGA54	4.784819	5.98E-06	3637929

CR_08670C_A	4.784242	0.004562	3642126
C4_00190W_A	4.784157	0.026083	3645030
C4_05810W_A	4.774123	3.98E-11	3641786
ALS1	4.773698	2.72E-12	3640280
C1_07820W_A	4.766812	0.008476	3640735
VRG4	4.765142	2.21E-05	3641768
CR_03530W_A	4.764333	6E-11	3643204
COX4	4.761933	2E-10	3635737
C1_07840W_A	4.74667	3.32E-08	3640737
C2_09250W_A	4.73521	0.025772	3644065
CCC1	4.735147	4.15E-05	3645645
CHS2	4.726487	2.82E-07	3641893
RAD51	4.721485	1.7E-06	3644944
C6_01460C_A	4.713532	0.001102	3641623
MRP8	4.703489	0.000999	3641178
ARL3	4.70155	8.2E-05	3644397
CR_02770C_A	4.686288	6.33E-05	3642581
RHD1	4.678515	2.18E-06	3647531
ECM39	4.677118	0.000282	3641386
RPP0	4.67709	1.57E-12	3638023
MNN10	4.675716	3.84E-05	3644997
BNR1	4.673159	0.000238	3639672
PLB5	4.669618	7.88E-05	3640750
C4_02930W_A	4.667741	0.030369	3637876
SUC1	4.647869	2.14E-05	3641916
BET4	4.644508	0.029015	3644695
C3_05280C_A	4.643136	0.0029	3635202
C7_03200C_A	4.642943	8.24E-05	3641342
C6_00110C_A	4.642241	0.000998	3647896
RPL17B	4.636212	5.49E-12	3645439
C1_03440C_A	4.63618	2.73E-07	3636908
XKS1	4.626103	8.78E-11	3648306
C3_04590W_A	4.620455	4.11E-08	30515204
C7_03780C_A	4.620165	0.028384	3643119
PRS	4.616869	0.010077	3640100
ERG4	4.605261	3.99E-10	3640511
PST1	4.592851	1.24E-05	3643520
CDC73	4.576266	1.23E-05	3636830
C1_07440W_A	4.576005	8.94E-08	3644482
CR_07640C_A	4.574011	0.010049	3647258
C3_04780C_A	4.573436	0.00011	3635214
C1_11740W_A	4.57214	0.010421	3645317
C3_04970C_A	4.565463	2.45E-07	3635164
AOX1	4.564104	0.012332	3634946
C7_00520W_A	4.555587	1.73E-10	3638001
C3_04460W_A	4.553939	1.17E-05	3635208
C4_04090C_A	4.54992	0.000271	3641225

C7_00150W_A	4.53436	3.77E-08	3637973
SOD5	4.528373	9.43E-05	3638886
RNH1	4.527781	6.15E-05	3639237
RPL13	4.522181	7.61E-11	3636841
CTA2	4.510811	3.26E-05	3639372
TLO9	4.492328	5.46E-05	3648051
C4_01730C_A	4.484703	1.57E-05	3635588
OBPA	4.483625	6.28E-10	3643764
C2_10860C_A	4.481975	4.23E-08	3641233
ARO2	4.461283	6.85E-08	3648190
DNM1	4.447179	6.48E-10	3643673
CR_00880W_A	4.446845	0.032787	3640397
HOC1	4.442325	1.63E-05	3641605
PHB1	4.437346	8.52E-06	3645641
IQG1	4.43387	1.97E-06	3637052
C2_08160C_A	4.421769	5.35E-08	30515152
GCF1	4.419751	3.75E-08	3646498
HEM4	4.40646	0.001477	3643942
ALG6	4.404784	0.001578	3647316
RFC4	4.39987	0.000458	3638994
C4_00590C_A	4.398026	5.44E-10	3645772
C1_05990C_A	4.392687	0.004265	3639871
UBI3	4.391692	1.26E-10	3642934
RPL3	4.383679	6.25E-13	3647719
MYO2	4.381377	9.29E-06	3636140
CR_02880W_A	4.374263	0.038063	3642584
UGA32	4.370585	0.012341	3639333
RPS30	4.370089	1.14E-08	30515390
GRE3	4.366848	1.65E-09	3637811
TKL1	4.365052	4.15E-13	3640767
LIP8	4.363147	0.000491	3646727
C3_07450C_A	4.361694	0.00434	3638481
C1_10090C_A	4.34984	5.51E-09	3634817
CHS6	4.346294	0.000159	3641377
C4_03770W_A	4.34528	0.046655	3645354
ENO1	4.336003	2.8E-12	3646493
MYO1	4.333659	5.83E-05	3647248
MMS21	4.327314	0.037069	3642265
CR_01180W_A	4.315798	4.58E-05	3640427
ILV2	4.312168	1.84E-08	3636552
CR_07460C_A	4.311358	5.98E-05	3646049
C6_03330C_A	4.309888	0.000419	3639199
ENG1	4.299672	4.28E-06	3636886
C4_02400C_A	4.298154	0.013966	3637851
CDC3	4.298065	5.59E-08	3644719
RIM1	4.296706	1.55E-05	3639898
C5_01070C_A	4.295627	0.000313	3642627

SOU2	4.293898	0.038642	3642823
ERG12	4.28544	6E-05	3634859
RPL5	4.284489	6.89E-12	3637057
GFA1	4.277351	2.07E-09	3636557
NIT3	4.276937	8.4E-05	3644408
C1_01910W_A	4.272056	8.96E-08	3645425
C2_03450W_A	4.268959	0.007832	3637349
GYP1	4.268027	4.52E-06	3641138
IFA21	4.265162	0.005069	3641344
RPL6	4.261851	2.4E-10	30514975
SMC5	4.252986	0.005488	3645103
SMC3	4.243202	0.043385	3638408
C4_01760W_A	4.242486	2.22E-05	3635584
C2_05850C_A	4.239102	0.000488	3647472
CDC68	4.234715	1.56E-09	3642787
ERG8	4.215725	2.35E-05	3635640
C4_03860C_A	4.211368	0.040365	3647854
C2_07440C_A	4.204022	0.000168	3645200
CR_02690W_A	4.201904	0.002546	3642574
GDB1	4.200819	3.2E-09	3647409
GSP1	4.196436	7.01E-10	3646925
C3_03560W_A	4.193113	0.000705	3638299
C6_02100W_A	4.192912	0.002285	3647554
CSP37	4.189841	1.87E-10	3640098
YPT31	4.186633	2.54E-05	3647999
C2_08650W_A	4.185107	2.8E-05	3643908
MEP1	4.178704	1.65E-06	3636553
MED1	4.167546	0.000114	3637418
LAP3	4.166183	3.03E-05	3641012
GIN1	4.164229	0.00763	3644539
C2_09560C_A	4.164156	4.73E-05	3643813
C6_01050W_A	4.157014	0.000111	3644015
C5_04770W_A	4.156334	0.001418	3636444
TFC4	4.153391	2.75E-05	3638420
C3_03340C_A	4.152087	0.000395	30515195
C3_01420C_A	4.138995	0.003243	3636634
C3_01590W_A	4.138928	0.006016	3636666
C2_01320W_A	4.125847	0.000143	3638846
C6_03470W_A	4.121491	3.63E-06	3640253
IFR2	4.119613	4.43E-09	3645100
MAK32	4.115919	0.016915	3645411
ARA1	4.11289	1.07E-06	3642331
TRS20	4.110238	0.045712	3640214
C1_05320C_A	4.108892	0.003495	3642470
C1_01360C_A	4.099856	1.28E-08	3640191
RIB5	4.09884	2.89E-07	3636499
C2_06920C_A	4.097639	0.003275	3643524

GPM1	4.094811	1.1E-11	3637431
C4_05790W_A	4.093592	0.001493	3641784
SGT2	4.091994	6.11E-09	3642978
C6_03620C_A	4.090275	3.4E-05	3640297
MAM33	4.089564	4.31E-06	3643103
POL3	4.088238	2.63E-05	3641359
PTC6	4.080382	0.000175	3647351
LRO1	4.076609	9.71E-07	3639356
PAN3	4.075926	1.8E-05	3636464
TEF1	4.057489	2.81E-12	3648254
APN1	4.055585	0.00042	3642263
C3_05880C_A	4.054707	8.26E-07	3641737
ETR1	4.053903	2.56E-09	3640561
CR_01300W_A	4.050269	0.000193	30515373
C3_01820W_A	4.044433	5.21E-07	3636584
RFC2	4.042349	0.017617	3637939
SDC1	4.036051	0.047579	3636542
GWT1	4.035319	0.000939	3645575
CBP1	4.034595	1.64E-07	3641920
C2_01690W_A	4.030003	1.13E-06	3635728
FGR38	4.028997	0.000822	3645412
C5_02400W_A	4.01733	6.66E-07	3637724
PGA53	4.00596	0.000596	3635599
CR_08900C_A	4.004752	4.06E-05	3641908
NUF2	4.000134	0.009743	3642613
NAM2	3.9956	0.000951	3640275
SEC27	3.986826	1.86E-09	3645870
CR_03460W_A	3.98451	0.000102	3643213
FAA2-1	3.976596	3.15E-05	3644100
RPL37B	3.968491	1.92E-08	30515053
RTA2	3.966534	3.42E-09	3646567
RPS3	3.960462	2.57E-09	3647299
MAL2	3.958848	0.001163	3638946
ARP9	3.954072	0.000538	3639829
EIF4E	3.949179	7.03E-08	3638981
C1_02440C_A	3.949013	0.00381	3636848
SEC26	3.948852	1.44E-09	3641005
C3_02190C_A	3.947474	9.35E-05	3636642
RFC1	3.941991	4.86E-07	3645582
YMC1	3.936085	0.000845	3644474
FGR51	3.926193	0.001023	3647796
RPS13	3.920084	4.08E-09	30515328
ORC3	3.913576	0.000265	3645639
C1_08040W_A	3.900356	0.010291	30515028
CMK1	3.888962	1.87E-09	3635263
C1_11120C_A	3.885287	4.71E-06	3644412
CSE4	3.884084	0.001935	3647750

CR_07350W_A	3.881453	6.36E-07	3646055
C6_00270W_A	3.874674	0.000671	3646066
C4_02620C_A	3.863685	1.87E-10	3637834
MON2	3.86338	4.16E-06	3636201
ECM4	3.861272	3.48E-09	3640144
HEM15	3.860449	7.65E-09	3645211
RBT1	3.852977	0.026366	3645377
YST1	3.847571	2.76E-09	3643678
C4_06710W_A	3.844873	0.004744	3642948
C5_00060C_A	3.844642	0.004779	3647889
C6_04560W_A	3.838336	1.95E-07	3645977
DLD2	3.833643	0.002306	3638475
CR_05900W_A	3.828715	0.009064	3646681
C4_00330C_A	3.811174	4.68E-05	30515227
C6_00200C_A	3.810169	0.000105	3646082
GEA2	3.808547	3.25E-06	3636646
TOM22	3.805063	0.000344	3647650
ILV3	3.801724	2.26E-07	3636428
CR_09570W_A	3.799081	0.024863	3647098
THS1	3.798406	2.21E-09	3647882
C4_04980W_A	3.79642	0.029166	3641167
BET2	3.78926	0.000764	3639025
DAK2	3.7853	1.29E-09	3634895
C1_09070W_A	3.780526	1.56E-05	3634773
TLO1	3.773943	0.000151	3639677
CSA1	3.773755	2.77E-07	3638009
C6_02490C_A	3.767172	0.000972	3639171
RCK2	3.759158	1.44E-08	3643507
YHM2	3.756415	3.34E-05	3647137
RPS4A	3.75226	2.61E-09	3641247
TIP1	3.746488	0.00522	3636512
C2_10470C_A	3.739362	0.000222	3641196
C7_01740C_A	3.735914	0.010711	3637010
YPT72	3.730405	0.000819	3643523
CR_02570C_A	3.725871	0.006134	3647685
COQ6	3.725703	1.21E-06	3636878
C1_11920W_A	3.723107	0.000149	3642003
ERV1	3.722706	0.012526	30515385
CR_09510C_A	3.718582	5.35E-05	3647105
MED20	3.716479	0.026857	30515250
SNG3	3.715989	0.00198	3646715
C2_00730C_A	3.714478	0.000884	3638913
ARC1	3.713175	3.58E-08	3645108
C5_03400C_A	3.709467	0.009008	30515308
C4_02470C_A	3.707083	1.94E-06	3637869
C1_08660C_A	3.704048	0.029196	3634931
C2_00880W_A	3.700457	0.001921	3638897

PMT6	3.694695	6.03E-06	3641116
CR_01070W_A	3.693822	0.067295	3640382
CDC50	3.693412	0.000563	3640302
CHS8	3.690719	1.26E-06	3640532
MNN24	3.689339	0.000136	3638789
URA5	3.686915	0.001503	3640087
C6_04250W_A	3.679997	3.62E-08	30515345
C2_02950W_A	3.675702	4.33E-05	3643012
KSR1	3.673992	0.012754	3646052
C1_01210W_A	3.673771	0.012633	3640209
RPP2B	3.673728	2.53E-07	3635195
MSH2	3.66911	8.11E-05	3642939
CR_07780W_A	3.667162	6.54E-05	3647352
MPT5	3.664488	0.000327	3636205
C2_03290W_A	3.66258	0.002208	3637429
DPS1-1	3.662083	9.61E-09	3645134
C7_03160W_A	3.658124	4.83E-06	3641345
EFT2	3.654489	1.75E-10	3642998
C3_05550C_A	3.646172	0.000673	3643659
C2_08200W_A	3.642934	0.003074	3648342
C5_01440C_A	3.641763	0.014128	3647043
C2_09970C_A	3.637506	6.43E-06	3643820
C7_01460C_A	3.637454	0.02884	3636958
ATC1	3.636418	4.66E-07	3639570
C3_06410C_A	3.635222	0.029186	3642261
C3_04800C_A	3.633897	0.000162	3635216
RPL11	3.628115	5.57E-07	3643489
ZRT2	3.625316	0.000525	3635861
CYC1	3.621439	2.08E-06	3643937
C3_01900C_A	3.618585	0.003961	3636559
C1_13260W_A	3.617375	0.000165	30515074
PRO3	3.610474	1.53E-06	3645036
STE24	3.607919	4.26E-06	3645040
C1_08050W_A	3.605464	3.36E-06	3640709
CSH1	3.6024	0.000209	3646427
C4_04230W_A	3.596364	0.031119	3646857
C4_06940C_A	3.592489	0.007206	3642928
SPE1	3.588007	2E-06	3639327
C2_06110W_A	3.582587	9.4E-05	3644094
PKH2	3.581673	4.7E-07	3642058
RPS21	3.580468	1.07E-09	3640242
GCV3	3.576153	2.32E-08	3636194
C7_00410C_A	3.575138	0.001513	3638033
SUR7	3.571597	5.53E-08	3641610
CR_07370W_A	3.569751	0.00184	3646053
MSM1	3.567683	0.034058	3636398
C2_08620W_A	3.566245	4.15E-06	3643912

CR_01820W_A	3.565505	1.34E-06	3640164
C6_00910C_A	3.561318	1.25E-07	3644030
CDC24	3.560837	6.93E-05	3643737
CR_00350W_A	3.551928	6.24E-07	3639686
LTP1	3.54924	0.000578	3640760
C1_06480C_A	3.548362	0.001613	30515008
DPP3	3.545335	0.016889	3636942
ZCF39	3.543958	6.06E-08	3639037
C1_10690W_A	3.543819	0.009303	3644443
CR_01430W_A	3.542037	0.037066	3640138
PLD1	3.541658	7.58E-09	3645329
OCA1	3.540632	0.007449	3643929
C2_00830C_A	3.539496	2.23E-06	3638808
C6_00760W_A	3.537615	7.77E-05	3647080
FRS1	3.537092	5.94E-08	3640121
CR_08050C_A	3.536733	5.84E-06	3644133
RAD7	3.532291	0.000828	3635833
PCK1	3.532053	3.09E-07	3639701
CR_02410W_A	3.530893	0.000954	3644928
RHO1	3.520112	1.9E-06	3642564
DAO1	3.516713	5.09E-05	3636885
C7_03660C_A	3.515835	7.45E-08	3646226
C2_09760W_A	3.513593	0.0325	3643807
CR_05480W_A	3.513257	1.56E-06	3648238
YPD1	3.510288	0.000141	3644458
SER2	3.508777	0.00132	3643006
C4_04390W_A	3.505198	2.33E-07	30515267
ILV1	3.497688	5.45E-06	3646918
TAF14	3.490952	9.38E-07	3637357
SEC62	3.490582	0.000763	3636860
RPL2	3.488	3E-09	30515049
ERG7	3.47695	1.63E-06	3635761
TPM2	3.463702	1.76E-08	30515441
TOM70	3.459765	3.86E-06	3647645
RPS5	3.45377	7.48E-08	3637700
C2_07100W_A	3.452099	4.83E-06	3643504
C3_01910C_A	3.44955	0.000243	30515182
LYS21	3.448996	5.96E-06	3644763
RPL25	3.446974	6.11E-08	30515338
C1_05720W_A	3.446578	1.36E-08	30515000
SEC24	3.436669	1.46E-07	3634939
C4_04330C_A	3.435872	9.58E-07	3646840
ARH2	3.42532	0.001696	3643411
C7_02080W_A	3.422608	0.019878	3637025
IDP2	3.422345	6.06E-06	3644934
FGR34	3.421452	0.000314	3639033
C1_08780W_A	3.41164	5.97E-05	3634789

PGI1	3.410834	5.02E-09	3644847
WBP1	3.407076	2.02E-06	3644398
C4_07240W_A	3.397752	0.000794	3642945
BUB2	3.396079	0.085358	3643030
C5_01420W_A	3.395195	0.000845	3647055
PFY1	3.394346	1.22E-06	3640703
RPS6A	3.393747	3.42E-08	3635701
OCA6	3.392126	0.037995	3643336
CTA24	3.389604	1.72E-06	3636475
C2_05800C_A	3.388256	0.000426	3645584
C6_03880W_A	3.383004	6.76E-06	3640293
RPN7	3.381411	8.06E-08	3641909
C7_04280C_A	3.377739	0.000135	3643173
MSI3	3.377126	5.54E-08	3639821
C1_07780W_A	3.373855	0.000244	3647955
RNA1	3.373659	8.42E-06	3636595
CR_05750W_A	3.369767	1.48E-07	3646696
PNP1	3.368778	5.12E-05	3638426
ASR2	3.36718	4.86E-08	3641907
C2_02090W_A	3.366693	0.006003	3635854
CTA7	3.361841	0.000142	3637776
C3_04630W_A	3.361808	0.007469	3635189
C1_06200W_A	3.348214	0.000984	3648300
C4_05080C_A	3.33982	0.010635	3641129
C1_02320C_A	3.337994	1.26E-06	3644572
FLC2	3.336579	1.73E-07	3647962
ATP16	3.335138	1.21E-07	3638963
YDC1	3.331511	0.0014	3642962
LPD1	3.331129	2.59E-08	3646037
CR_07700W_A	3.330587	0.001462	3648195
C3_04360W_A	3.32699	0.000117	3635276
C4_05850C_A	3.326485	0.003313	3641791
CAK1	3.323578	0.006592	3637352
CYS4	3.323463	6.26E-08	3645429
HGT7	3.316587	3.15E-09	3638876
C1_13320C_A	3.313585	2.15E-05	3636149
CDC54	3.312477	0.046825	3647606
RPL4B	3.310956	1.68E-09	3643347
VAM3	3.310933	0.044664	3635146
RPL16A	3.304252	8.53E-08	3639338
C7_01930C_A	3.297231	0.00346	3637027
GUS1	3.290358	4.98E-07	3638029
WH11	3.281778	8.45E-05	30515117
APS3	3.279583	0.012261	3646491
THI6	3.277897	0.003495	3638333
CR_04730W_A	3.270788	0.048514	3647305
RPL32	3.268913	6.92E-07	30515335

C6_01420C_A	3.259617	1.48E-05	3641602
PLC1	3.256842	0.00319	3647860
C4_05860W_A	3.256047	0.003956	3641792
C7_03050W_A	3.256047	0.003958	3641378
CR_04500C_A	3.254641	0.003585	3641014
C1_02060W_A	3.253492	0.004263	30514968
ARC15	3.247678	0.002252	3646043
ERG27	3.243112	0.000643	3640432
C1_10320W_A	3.242574	0.004605	3634907
GTT11	3.240905	0.0007	3645644
C1_00320W_A	3.240631	0.003855	3639297
CR_04020C_A	3.239609	0.008339	3640969
TOM40	3.235929	5.18E-07	3636968
CR_01280C_A	3.232545	0.026337	3640400
C5_04150C_A	3.229732	0.048425	3636525
CR_08920W_A	3.229366	0.001026	3641911
MUQ1	3.228865	6.95E-05	3638898
MNN21	3.226883	0.000246	3648124
CNB1	3.226404	0.002157	3636463
PEX7	3.217779	0.000363	3644027
KRE5	3.213178	0.000315	3638384
C1_02370C_A	3.212249	0.016902	3644576
C1_00630W_A	3.212211	0.012995	3639380
AMN1	3.20945	0.00699	3635795
C3_01940C_A	3.20822	0.003895	3636600
BRN1	3.207037	0.006495	3641815
C3_03080W_A	3.197662	0.008964	3638397
C5_00280C_A	3.195959	0.002423	3641581
C3_03410C_A	3.194977	9.52E-07	3638353
KGD1	3.194708	1.59E-07	3647752
C2_10700C_A	3.194401	0.000351	3641207
CR_07570W_A	3.193407	0.003922	3648004
C4_00410W_A	3.190955	0.00367	3645019
C1_06890C_A	3.189397	1.05E-06	30515014
C2_00400C_A	3.188389	3.17E-06	3638821
CDC20	3.185867	0.002577	3644057
RBF1	3.185442	0.00107	3639251
NUP159	3.18531	2.49E-07	3647458
C2_00870W_A	3.184447	0.013272	3638804
RPD31	3.178408	5.89E-06	3638520
CR_05880W_A	3.178199	0.029891	3646702
PXA2	3.177556	0.000113	3642055
C4_01910W_A	3.168856	0.005648	3635635
FGR29	3.15931	0.013557	3641190
C1_02430C_A	3.156165	1.86E-05	3636846
MNN2	3.153347	0.002726	3644405
RPL15A	3.153339	2.16E-08	3641025

C2_01390W_A	3.152792	1.02E-05	3635726
C5_01540W_A	3.15278	6.85E-07	30515301
GAL4	3.145353	2.31E-05	3641244
C4_03110W_A	3.145093	5.78E-07	3637924
PEX3	3.14311	0.001918	3644484
C4_02720C_A	3.133483	0.000476	3637857
RHO3	3.130774	0.005785	3643448
ADE17	3.130072	8.91E-08	3641024
PMR1	3.126437	1.56E-05	3638043
FOX3	3.125536	0.000161	3636629
C3_03840C_A	3.124989	0.001634	3645628
CAT8	3.122091	0.022345	3640745
SAM4	3.119238	0.000247	3646506
C1_10460W_A	3.117114	0.000449	3647183
C1_02040C_A	3.114025	6.81E-07	3644586
SFH5	3.109683	0.005962	3634884
C2_04370W_A	3.109333	0.000152	3645460
C4_01830C_A	3.102724	0.00052	3635593
SEC3	3.101258	5.75E-05	3642806
DLD1	3.100797	0.000209	3643025
C4_03720C_A	3.096938	0.002809	3645359
C3_01720C_A	3.092851	8.05E-07	3636601
ATP20	3.088392	0.000427	3647602
RBK1	3.087825	0.001613	3646830
HYM1	3.087208	0.000469	3637355
PPH21	3.082837	9.93E-06	3636665
AKR1	3.081755	4.37E-06	3636166
C6_03580W_A	3.074944	0.033298	3640250
C1_10410W_A	3.072768	0.006397	3634840
STE23	3.072536	0.000277	3639236
ISC1	3.068456	0.047479	3646705
YBL053	3.059137	0.024462	3647045
COQ5	3.056621	0.000516	3643416
C3_06350W_A	3.056456	0.000474	30515215
SEC10	3.056427	2.06E-05	3642961
MAE1	3.053469	3.3E-05	3641615
C2_00510W_A	3.050046	0.000943	3638868
RPB11	3.044876	0.035519	30514957
SET3	3.043204	1.03E-05	3643320
C4_05640C_A	3.042784	0.019957	3641813
C4_00320C_A	3.040844	0.005102	3644999
LAG1	3.032881	0.018501	3640383
C1_01000C_A	3.03199	0.018862	3640180
UBA1	3.027938	6.53E-07	3642286
C7_00420C_A	3.027152	0.005447	3638032
C3_01210C_A	3.027107	0.000355	3636612
C1_06670W_A	3.025106	5.3E-05	3639541

YKU80	3.016921	0.008108	3645138
YCG1	3.015795	0.002591	3636639
CR_07600W_A	3.015243	0.002198	3644849
PPE1	3.013709	0.037741	3635802
C3_01280W_A	3.011952	0.003493	3636623
C2_09620W_A	2.99534	0.004312	3643797
C3_01140W_A	2.988167	0.015101	3639902
YVC1	2.984122	7.71E-05	3642303
LYS2	2.977979	8.68E-07	3636875
RPL27A	2.972314	5.41E-06	30515062
C2_07390C_A	2.971217	2.06E-05	3645229
RPS21B	2.971079	2.26E-06	30514961
CR_01380W_A	2.96741	0.000606	3640131
ERG1	2.965366	2.09E-06	3646509
PGA33	2.9649	0.004982	3637350
C2_04410W_A	2.962673	0.008864	3645455
CR_09070C_A	2.960733	0.000686	3641926
RAV2	2.960576	0.013868	3639252
C1_12140W_A	2.957727	0.023154	3642050
C3_06690C_A	2.955933	0.024324	3642256
C2_02930C_A	2.953754	2.46E-05	3643015
CR_04080C_A	2.952827	8.34E-06	3641023
C1_10500W_A	2.952463	0.026342	3647188
C6_03820C_A	2.950293	0.000662	3640288
PRE2	2.947765	6.98E-06	3643490
CCP1	2.946338	3.57E-06	3638350
CR_09750C_A	2.937202	0.000497	3639009
PIKA	2.93437	0.000552	3643765
C3_01180C_A	2.930273	0.0002	3647960
APG7	2.929679	0.026463	3646615
RPS20	2.927931	2.52E-06	3642071
C2_07080C_A	2.92757	0.029737	3643502
NUO2	2.921966	8.77E-06	3638381
VMA4	2.91528	3.65E-05	3640111
IFK2	2.915276	0.013626	3637439
CR_04560C_A	2.915175	0.014027	3640946
C7_00120W_A	2.915055	0.005279	3637976
POL2	2.914919	0.000741	3645733
RDI1	2.908801	2.43E-05	3635167
C1_14170W_A	2.906942	2.52E-05	3643323
HOG1	2.905987	1.56E-05	3637393
C4_06240W_A	2.90412	0.02833	3642807
PEP3	2.899453	0.003518	3639267
C1_11100W_A	2.894866	0.014224	3644414
C2_04170C_A	2.893219	9.1E-06	30515111
SWC4	2.891548	0.00045	3639679
KIP2	2.890161	0.047484	3643954

C3_05410W_A	2.89012	2.58E-06	3643665
ADP1	2.889479	4.15E-05	3642458
C5_02060W_A	2.888281	0.045989	3643748
TRP3	2.888259	0.007084	3642035
C4_03810W_A	2.888163	0.010842	3645350
MNN26	2.88769	0.00784	3646240
C6_00920W_A	2.885206	0.000932	3644031
MRP2	2.881909	0.002855	3636498
AIP2	2.877053	9.24E-06	3638393
C7_01480W_A	2.875012	0.015331	3636956
ZCF32	2.873059	0.015974	3635213
ADE12	2.871562	1.18E-06	3634869
C7_00380W_A	2.871104	0.023128	3638036
RPL14	2.868383	2.41E-06	30515072
COX7	2.866055	0.000528	3638339
PTC7	2.862764	4.2E-05	3645000
SEC34	2.861241	0.000751	3644455
HAM1	2.857411	0.005263	3647224
C4_02100C_A	2.854572	0.017253	3635641
SEC61	2.85047	1.75E-06	3647462
C5_02050W_A	2.84619	4.46E-05	3643768
PHB2	2.842031	3.11E-05	3635238
IFD3	2.837813	0.002509	3640211
BUD7	2.835784	3.86E-05	3647228
C2_09930W_A	2.835625	0.002086	3643824
C1_14050C_A	2.83307	0.000547	3643343
ADE2	2.831325	1.24E-06	3635259
C3_04130W_A	2.828222	0.000492	3635283
C1_03430W_A	2.825236	0.002501	3636906
PEP1	2.824036	3.04E-06	3647612
C3_06610W_A	2.815454	0.014679	3642248
C5_00750C_A	2.81511	0.000668	3644121
C1_02490C_A	2.813498	0.005971	3636853
ARO9	2.805316	0.034795	3641773
C1_00920W_A	2.796011	0.001553	3639283
C5_02900W_A	2.794995	0.001522	3637747
ALG9	2.792449	0.002755	3644457
CDC55	2.792061	5.84E-06	3635785
C7_02170C_A	2.790296	0.000904	3636993
STE13	2.788092	0.019749	3647597
C4_02040W_A	2.787254	0.019445	3635649
CR_07170W_A	2.786722	0.013651	3645271
C5_05440C_A	2.785316	9.82E-06	3636431
C6_04120C_A	2.784618	0.009569	3647974
ANT1	2.782418	0.004532	3639516
VPS35	2.773687	0.00017	3645567
C2_07630C_A	2.773548	3.69E-06	3645193

C1_01490W_A	2.770204	9.06E-07	3640226
C4_01020C_A	2.768522	3.6E-06	3635606
C1_02830W_A	2.762806	0.007787	3636876
CR_08130W_A	2.76044	0.008527	3642070
C2_02840C_A	2.750528	0.003247	3642976
GAD1	2.746896	0.000112	3645339
HOM2	2.741336	2.24E-05	3635826
BTS1	2.728473	0.039927	3646982
C1_01900C_A	2.727511	0.002158	3645426
SEC4	2.722688	5.42E-05	3640119
PKH3	2.722609	6.86E-05	3646075
RAS1	2.721531	2.1E-06	3643927
PGA52	2.719656	8.97E-06	3647908
RPS18	2.717289	1.21E-05	3637953
FAA2-3	2.715986	0.00372	3643125
C4_04650W_A	2.715945	0.044354	3641152
C1_08490W_A	2.7105	0.000883	3646492
C7_02670W_A	2.709112	0.02555	3647651
GIM5	2.707005	0.025483	3639886
CR_03020C_A	2.700824	0.01349	3642598
CR_08560C_A	2.700071	0.000627	3642142
CR_08620C_A	2.69415	0.009033	3642110
C1_03510C_A	2.692845	1.11E-05	3636916
C1_07630W_A	2.688007	0.009776	3646660
C3_05010C_A	2.687054	0.011896	3635169
APA2	2.684451	0.000166	3639234
CDC7	2.682668	0.023895	3643424
SCL1	2.682212	0.000102	3640510
RPN8	2.678861	3.36E-05	3643770
C6_04060W_A	2.673602	0.001335	3647828
RIM20	2.673375	3.59E-05	3634809
C3_06970W_A	2.671178	0.003965	3638506
SRV2	2.669026	8.6E-06	3640938
C2_03900C_A	2.668709	3.87E-05	3637395
C7_03840W_A	2.667077	0.00958	3643146
URA4	2.667065	0.001453	3642632
C4_04720W_A	2.663376	0.000219	3641137
C1_13190W_A	2.662752	4.34E-06	3636163
GUA1	2.657238	4.61E-05	3634763
C6_02410W_A	2.654473	0.000736	3645884
C7_03260C_A	2.65271	0.046621	3641366
AHA1	2.652142	0.001211	3638933
RPS22A	2.64506	8.83E-05	3639551
C2_05220C_A	2.643415	0.000731	3643466
C7_01160C_A	2.641136	0.016541	30515351
FGR17	2.639792	0.025971	3640296
ERG20	2.639327	0.000166	3645440

MDL1	2.636826	0.001143	3640146
C7_02780W_A	2.633207	9.2E-06	3641361
C3_00050C_A	2.631199	0.010415	3640570
CR_06560C_A	2.629067	0.00154	3648367
HEM2	2.628258	0.002392	3637428
SMC4	2.624822	0.000654	3641578
MED18	2.62443	0.015696	3644103
C3_00470W_A	2.624198	0.003024	3640520
C7_00250C_A	2.623933	0.001433	3637979
C3_07820W_A	2.623656	0.030689	3647463
CR_01700C_A	2.617543	0.004422	3640129
C1_04640W_A	2.613821	0.001277	3644761
NPL3	2.613433	5.58E-06	3643310
C7_02370W_A	2.611227	8.09E-06	3636930
CR_09150W_A	2.610071	0.021572	3641880
SRO77	2.609067	0.000112	3646088
KEM1	2.604211	2.27E-05	3636222
C4_01780C_A	2.603947	0.005193	3635582
C3_05150W_A	2.5999	0.000138	3635162
ARP8	2.597074	9.27E-06	3640200
C1_06720C_A	2.597024	0.011671	3639509
C3_02120W_A	2.594592	0.001303	3636658
SCT2	2.593016	0.000123	3643018
PGA49	2.592273	0.019043	3646525
GBP2	2.591581	1.56E-05	3637992
RAD16	2.589443	0.000137	3636874
C1_07690C_A	2.589143	0.001555	3641767
CR_06820W_A	2.586807	0.024907	3647325
VPS52	2.584517	0.007992	3638996
C6_00890W_A	2.580543	1.3E-05	3644028
SMP2	2.579622	6.55E-06	3635779
CR_09990W_A	2.577623	0.001455	3638930
RPN6	2.573168	0.000125	3645352
NOP10	2.572325	0.014807	30515434
CYP5	2.570767	1.23E-05	3642257
C2_09650W_A	2.570229	0.00027	3643794
C1_01300W_A	2.568828	0.000124	3640185
VPS11	2.568648	0.000536	3646524
RPL23A	2.566013	4.01E-05	3647558
CRG1	2.561731	0.00029	3646393
ALG2	2.55969	0.00721	3647832
CHC1	2.557964	2.76E-05	3647552
PRC3	2.557316	0.000293	3639889
C1_02680C_A	2.553009	0.027778	3636822
CDC47	2.541887	0.002262	3645780
CR_05860W_A	2.541563	0.007208	3646703
C7_02470C_A	2.539555	0.013597	3636945

RCY1	2.536007	0.024623	3643731
C5_02750C_A	2.535948	0.002298	3637740
RPN2	2.531661	2.71E-05	3642027
C2_01870C_A	2.525655	0.02058	3635836
PXA1	2.525168	1.33E-05	3639713
CR_08000C_A	2.52346	0.000117	3644150
RIB4	2.523105	3.22E-05	30514995
VAS1	2.522468	3.53E-05	3648340
C1_10820C_A	2.521161	0.000184	3644435
COX8	2.519493	3.96E-05	30515130
C1_11890W_A	2.514738	0.003625	3642006
C2_08380C_A	2.509597	0.038696	3648253
MRPL6	2.509182	0.024482	3639724
AAT22	2.508663	0.040531	3635651
RPL12	2.50806	3.95E-05	3636659
C1_13280C_A	2.492515	0.022281	3636145
UBP13	2.490365	0.00043	3638879
RBP1	2.4849	6.69E-05	3647647
C5_03490C_A	2.475073	0.000129	3646967
RCT1	2.474717	1.02E-05	3641727
C5_04900C_A	2.465399	0.004697	3636416
C2_02700C_A	2.463975	0.027582	3643005
C6_00190W_A	2.463915	0.00797	3646080
MDM10	2.463405	0.028603	3647778
RPL42	2.463099	0.000172	30515041
UBC15	2.460482	0.025684	3641260
C3_01570W_A	2.458067	0.02652	3636668
CR_10510W_A	2.45781	0.004395	3639039
SSO2	2.457189	0.000966	3643809
RPS23A	2.455572	8.23E-05	3639515
CR_10600C_A	2.452579	0.000152	3639004
GLK4	2.449055	7.49E-05	3646046
GPI7	2.444845	0.048888	3647672
C1_09610W_A	2.440823	0.003527	3634891
SLA1	2.432947	6.33E-05	3635739
C3_06710W_A	2.430998	0.000424	3638448
MAC1	2.429153	0.015976	3637985
C2_01170C_A	2.429046	0.002148	3638860
CR_03860C_A	2.418678	0.002578	3643186
C3_03760W_A	2.416464	0.003159	3645638
SEC72	2.415993	0.021435	3645768
C2_07810W_A	2.408725	0.007816	3642324
C1_14180W_A	2.406406	0.017692	3643324
ASC1	2.398784	6.49E-05	3645907
C7_00190W_A	2.396703	0.00451	3637969
KRS1	2.3959	8.81E-05	3638486
C5_01350W_A	2.395533	0.003273	3642640

C6_03040C_A	2.394206	0.028631	3639269
C1_11600W_A	2.393694	0.028638	3645346
C2_04490W_A	2.391905	0.00303	3645463
HAT2	2.390658	0.003222	3645973
PHM7	2.387006	0.000103	3642329
C6_00240C_A	2.385279	0.011401	3646062
ALA1	2.3827	9.71E-05	3640285
C1_03690W_A	2.379688	0.000572	3648426
KEL1	2.378594	0.000206	3639318
C1_11850W_A	2.367947	4.06E-05	3642010
C5_02690W_A	2.36689	0.004542	3637775
C2_05020W_A	2.365269	0.080676	3643447
C5_00580W_A	2.359794	0.003898	3641588
RPG1A	2.356664	8.31E-05	3646816
C7_00140C_A	2.352993	0.016037	3637974
C1_12170C_A	2.345719	0.018025	3642039
POB3	2.342409	6.89E-05	3635827
TUF1	2.340644	0.000113	3639360
C6_01370W_A	2.337912	0.015875	3641607
C6_02280W_A	2.33786	0.021099	3645872
GAT1	2.335742	0.019287	3641794
C1_04560W_A	2.333752	0.027674	3646130
C1_04580C_A	2.332114	0.041056	3646132
CR_06620W_A	2.331245	0.014183	3646631
C5_04960W_A	2.324337	0.00013	3636423
C5_01890W_A	2.323363	0.011846	3643750
C7_03030W_A	2.3154	0.019902	3641380
CDC60	2.30498	0.000152	3640128
RBD1	2.304979	0.00356	3642018
C1_03790C_A	2.302005	0.000292	3644682
IRR1	2.301679	0.008303	3643305
C1_05390C_A	2.300145	0.003688	3642479
SLC1	2.299892	0.01491	3638324
RPN1	2.296617	9.14E-05	3636147
EBP7	2.296557	0.019911	3642971
C4_00230W_A	2.296028	0.041348	3645034
YBP1	2.294396	0.00503	3636187
GIT2	2.293948	0.016373	3642633
CR_08990C_A	2.29287	0.000297	3641891
CR_10590W_A	2.292382	0.004042	3639003
OXR1	2.292357	0.008406	3638372
C5_02230W_A	2.288961	0.000174	3637708
C5_00790C_A	2.286177	0.000149	3644125
C6_04340W_A	2.2854	0.02012	3646291
C6_02630C_A	2.284877	0.004564	3639221
SRB9	2.28369	0.000705	3635845
PDS5	2.283634	0.012433	3642341

CR_00290W_A	2.283513	0.000745	3639717
C6_00560W_A	2.281888	0.005376	3647143
C7_04310C_A	2.273739	0.008251	3643141
C1_14500C_A	2.272886	0.000256	3643331
C2_07190C_A	2.271774	0.010321	3643513
C1_11010C_A	2.268064	0.014091	3644421
ARO8	2.264606	0.00091	3638828
MSK1	2.264456	0.040598	3637032
C1_09360C_A	2.261582	0.000262	3634807
C4_00780C_A	2.261474	0.002377	3645759
NMT1	2.258241	0.002921	3635624
C1_12830C_A	2.253884	0.001409	3636176
C7_01650W_A	2.253027	0.000226	3636984
C1_14530W_A	2.252178	0.013532	3643333
TLO34	2.250107	0.022379	3648392
UGA2	2.248015	0.00055	3645436
URA2	2.247725	0.000241	3645728
BCY1	2.247233	0.000185	3638832
C2_08270C_A	2.241639	0.013326	3642347
VPS4	2.240563	0.003238	3637703
RPL21A	2.238891	0.000396	3637400
ALS2	2.234465	0.001316	3645724
C3_07480W_A	2.233765	0.005839	3638529
C1_10680C_A	2.232642	0.000425	3647678
C5_01940W_A	2.229495	0.004009	3643740
C2_09600C_A	2.224411	0.035576	3643799
RPS24	2.220066	0.001052	3640568
C2_03910C_A	2.216661	0.032992	3637426
FAT1	2.212525	0.007331	3647083
C6_02740W_A	2.209416	0.011833	3639216
C7_02480W_A	2.209082	0.03833	3636944
C1_14090W_A	2.208659	0.011984	30515079
NAT2	2.200404	0.008646	3638837
NHX1	2.199802	0.001507	3647141
TIF	2.197968	0.000127	3640190
PEX4	2.196796	0.002456	3636429
BNI4	2.194218	0.006245	3646406
RPL10	2.194015	0.000334	3636850
PGA14	2.19325	0.000258	3641560
PRO2	2.192178	0.020977	3638532
SES1	2.189834	0.000378	3638415
OCH1	2.187863	0.006856	3641751
TIP20	2.185746	0.007347	3636457
CDC53	2.185688	0.00062	3636603
C2_00570W_A	2.181093	0.002254	3638853
LMO1	2.173453	0.006288	3641403
RNR21	2.169672	0.000228	3643021

C4_03920W_A	2.166543	0.028108	3648314
MP65	2.166142	9.38E-05	3643951
VTC3	2.164663	0.001164	3643206
ROB1	2.163969	0.000851	3636212
C4_03600C_A	2.163772	0.00325	3645366
RPL29	2.160572	0.000671	30515048
RBT5	2.159695	0.000205	3645007
C2_01900C_A	2.150463	0.009146	3635789
C1_00410C_A	2.148618	0.016315	3639287
INO4	2.147809	0.046567	30515105
ADH1	2.145808	0.000387	3636489
C6_00430C_A	2.144056	0.005928	3646089
CR_04230W_A	2.143584	0.019396	3640944
FOX2	2.14037	0.000885	3648327
NSP1	2.140094	0.000275	3637430
C6_03310W_A	2.139352	0.035435	3639197
HPC2	2.136889	0.018469	3636188
GRE2	2.13591	0.010131	3648026
C2_03360W_A	2.12994	0.000542	3637389
C5_03550W_A	2.128704	0.000529	3646973
RCE1	2.127907	0.038559	3641104
LCB4	2.12443	0.049988	3642057
C4_05340W_A	2.123846	0.042739	3646947
URA6	2.122485	0.004846	3647988
GLX3	2.12239	0.006528	3638325
INT1	2.121336	0.002053	3637761
C3_00830C_A	2.11953	0.009564	3647757
USO1	2.118283	0.001065	3648280
AAT21	2.116614	0.001294	3647241
RPS25B	2.115656	0.001989	3646972
UBC4	2.112027	0.000486	3638927
CR_09620C_A	2.107281	0.017056	6335435
NAT5	2.100995	0.02851	3641022
TRY5	2.100745	0.045069	3641619
RVB2	2.098795	0.000668	3637055
CR_08170C_A	2.09536	0.030466	3642073
C3_01950C_A	2.095227	0.007318	3636599
CMD1	2.093833	0.000566	3646520
C3_07740W_A	2.093025	0.001973	3638453
RPL39	2.092658	0.000692	30515109
C5_04470C_A	2.086796	0.00079	3636414
C2_04330C_A	2.084197	0.032551	3645453
C1_02000W_A	2.078329	0.010832	3645418
C6_02370C_A	2.077146	0.008636	3645882
ZUO1	2.06865	0.001303	3637908
COQ4	2.06438	0.007447	3636804
C4_03320W_A	2.062616	0.033052	3648092

ACP1	2.061519	0.013596	3639825
CR_09500C_A	2.06014	0.026812	3641897
STP1	2.057547	0.017139	3635152
C1_08680C_A	2.05689	0.037458	3634933
C1_10280C_A	2.055667	0.003122	3634902
CR_07080W_A	2.053764	0.002311	3647964
KEX2	2.041583	0.009754	3634914
PTC4	2.041419	0.011791	3646697
TIF5	2.040476	0.013886	3637765
CR_07840C_A	2.03548	0.033372	3647346
POX1-3	2.035178	0.002924	3636598
APR1	2.0315	0.000809	3645204
C3_00890C_A	2.026902	0.038979	3647753
C5_03140C_A	2.026388	0.000781	3637798
C2_10460C_A	2.025915	0.00882	3641195
CR_03600C_A	2.025272	0.012005	3643207
TIP41	2.024499	0.032484	3636506
DBP5	2.023781	0.004437	3636564
GOR1	2.022555	0.039642	3636836
GPA2	2.016448	0.022326	3636638
SKI8	2.014892	0.010656	3640294
PSD2	2.013251	0.004198	3636515
REV3	2.013221	0.046159	3641749
RPT4	2.005125	0.000577	3640966
C1_06190C_A	2.000529	0.014113	3648391
C4_02950C_A	1.999918	0.340362	3637874
C1_12920C_A	1.999099	0.045504	3636155
CR_09190C_A	1.998805	0.049585	3641885
C3_03210W_A	1.996191	0.131177	3638315
C5_03880C_A	1.995248	0.337182	3647222
EMP70	1.993997	0.013712	3637919
MRE11	1.990768	0.029383	3645898
C1_04470C_A	1.989891	0.159852	3646142
GCN4	1.987451	0.000825	3643823
C3_03930W_A	1.986809	0.012409	3645657
HGT5	1.986766	0.016377	3635201
CYT2	1.983618	0.219035	3635671
CCT5	1.982837	0.001128	3643478
C2_08340C_A	1.973904	0.137009	3648165
MKK2	1.968446	0.010724	3645580
C3_07030C_A	1.968332	0.020804	3638517
C1_12930C_A	1.967166	0.472982	3636156
CR_06690C_A	1.966348	0.020881	3646624
C1_06360W_A	1.966077	0.075654	3639561
C6_01490C_A	1.963611	0.172592	3641620
C3_05110W_A	1.963379	0.053669	3635158
UME1	1.961	0.002676	3643101

FGR50	1.955656	0.085171	3644859
C6_00850W_A	1.95468	0.005793	3644010
C1_04980C_A	1.95329	0.019476	3647529
BIG1	1.952565	0.054434	3644434
SNO1	1.946173	0.043446	3636812
PGK1	1.944757	0.00162	3647081
C1_09750W_A	1.944434	0.071342	3634822
C5_03980W_A	1.943631	0.003869	3647442
ECM15	1.94264	0.072851	30515218
TLG2	1.941647	0.095401	3635773
MEC1	1.940961	0.017335	3647733
C1_12060C_A	1.936515	0.354267	3642026
C5_04300C_A	1.936497	0.001645	3636456
CR_05960W_A	1.936159	0.353776	30515417
CPH1	1.935344	0.197752	3644468
IST1	1.931261	0.140692	3640587
CR_05890C_A	1.928299	0.02617	3646682
BMT3	1.927302	0.087189	3640439
C4_04180C_A	1.925384	0.24679	3641183
C1_09470C_A	1.922927	0.033758	3634761
NCP1	1.920839	0.00153	3637839
C4_02680C_A	1.920779	0.343056	3637828
RPS28B	1.920002	0.003646	30515349
HRT1	1.916932	0.079317	30515187
C3_01890C_A	1.915134	0.001572	3636561
CR_01120C_A	1.914648	0.019702	3640434
TPK2	1.914642	0.001624	3643482
C1_13030C_A	1.913275	0.00389	3636122
C2_07610C_A	1.912979	0.004807	3645195
C3_06680C_A	1.91241	0.194977	3642255
ALS3	1.912006	0.002286	3647965
CR_02120C_A	1.910814	0.155678	3640143
SHE9	1.910728	0.029536	3642984
C7_00920C_A	1.908571	0.067255	3637957
ESC4	1.904362	0.380036	3635727
C1_05780W_A	1.902277	0.012754	3639888
ZCF10	1.900952	0.050235	3643485
SSC1	1.899357	0.001382	3645232
C7_04210C_A	1.899272	0.003124	3643121
C4_06470W_A	1.899011	0.037395	3642790
C1_01940C_A	1.898945	0.110783	3645422
IML2	1.892987	0.003877	3643302
WRS1	1.892551	0.014796	3642060
C3_03830W_A	1.890923	0.058381	3645630
C5_02450W_A	1.887833	0.021044	3637729
C2_02360C_A	1.887642	0.197003	3635824
CZF1	1.885005	0.007594	3642915

HMO1	1.883525	0.003822	3646685
ARF2	1.883011	0.002445	3635271
MYO5	1.880317	0.003297	3647404
CR_09640C_A	1.877759	0.064977	30515456
C4_00210W_A	1.877498	0.378764	3645032
C2_00630C_A	1.877419	0.384202	3638848
C1_02240W_A	1.876967	0.005811	3644597
C1_09000W_A	1.876832	0.09683	3634915
C3_01680C_A	1.873488	0.023523	3636605
C4_04620C_A	1.873008	0.13102	3641155
C3_07330W_A	1.8707	0.335764	3638478
TIM21	1.866832	0.063029	3644573
LHP1	1.866059	0.031235	3646665
C1_09810W_A	1.86514	0.051528	3634845
DIT1	1.864979	0.508626	3648147
UME7	1.864714	0.506665	3637918
MCA1	1.864154	0.00317	3635233
C1_08390C_A	1.862977	0.015405	3646504
CTA1	1.86241	0.039148	3641631
C2_04180C_A	1.861991	0.107492	3637330
C5_05260W_A	1.861967	0.208898	3636473
C6_02960W_A	1.861158	0.272821	3639189
C2_09710C_A	1.859692	0.003682	3643786
LYS142	1.85822	0.025729	3634896
YCS4	1.85526	0.02116	3645196
CDC14	1.853068	0.020965	3647132
SWD2	1.851246	0.092253	3646677
C5_04790C_A	1.850345	0.407352	3636446
C3_07460W_A	1.850274	0.064636	3638480
C3_06490W_A	1.848732	0.005394	3642285
LPT1	1.848232	0.009598	3645212
C2_05570C_A	1.845395	0.07437	3645562
C2_07200W_A	1.845239	0.18311	3643481
C1_04910C_A	1.845238	0.025453	3644793
C2_00110W_A	1.845155	0.111894	3647921
SBP1	1.843257	0.004847	3635280
MAD2	1.842797	0.3293	3644696
ABP2	1.842613	0.333361	3643443
C6_01330C_A	1.84198	0.325953	3641643
C3_06550C_A	1.841074	0.064049	3642275
C1_13060C_A	1.841017	0.008372	3636123
HDA1	1.840638	0.031384	3640154
CR_00830W_A	1.840353	0.197038	3640412
C3_07140C_A	1.840338	0.234538	3638439
MIR1	1.839914	0.004634	3634782
C1_12740W_A	1.839848	0.081901	3646819
CR_05120W_A	1.83923	0.010996	3646402

C4_06740C_A	1.839021	0.004775	3642923
C5_01980C_A	1.83771	0.097915	3643775
C1_12660W_A	1.836683	0.137967	3646835
CR_01670W_A	1.836665	0.228866	3640124
TEA1	1.835999	0.02704	3643671
C7_01190W_A	1.834265	0.150787	3645902
UGA11	1.833886	0.148617	3637437
C2_01220W_A	1.831288	0.012222	3638856
C2_05410W_A	1.831148	0.005885	30515120
DBF4	1.826855	0.075599	3641388
MET3	1.826039	0.033321	3636132
LYS14	1.824538	0.200751	3639217
C6_02580W_A	1.823817	0.076333	3639207
C5_02520W_A	1.823335	0.27291	3637768
CR_05330W_A	1.823079	0.272939	30515404
MMS22	1.822384	0.277627	3639681
C4_02780W_A	1.82114	0.009056	3637902
C2_08540C_A	1.820072	0.407078	3643920
CDC6	1.819814	0.40328	3642034
CR_02060W_A	1.817673	0.110561	3640155
C2_05050C_A	1.815318	0.154029	3643450
C2_10670W_A	1.814718	0.00208	3641251
C3_02770C_A	1.814134	0.030351	3638414
TRP1	1.811583	0.033181	3639321
CR_01260W_A	1.809864	0.149683	3640402
MED16	1.807218	0.031814	3646725
C7_03370C_A	1.806765	0.022939	3646720
C2_01920C_A	1.805408	0.016003	3635792
CR_05970C_A	1.805359	0.036094	3642502
C3_06810W_A	1.803852	0.062814	3638545
ADE4	1.80124	0.006984	3641769
CR_08980C_A	1.80007	0.109665	3641890
C3_00610W_A	1.797743	0.166993	3640578
RPL9B	1.796963	0.004583	3638348
C2_00920W_A	1.796478	0.100969	3638893
RAD32	1.79555	0.076382	3637340
YRB1	1.795307	0.010082	3639663
C4_01670C_A	1.794745	0.108234	3635711
C6_04110W_A	1.792484	0.008513	3647834
C7_00580C_A	1.79189	0.156546	3637995
C1_10960W_A	1.791635	0.204253	30515047
C2_00320W_A	1.791374	0.070237	3638798
C1_14200W_A	1.791043	0.039959	3643301
C1_12070C_A	1.790748	0.221986	3642025
AGO1	1.789804	0.03476	3642828
CSH3	1.787807	0.012361	3646798
C2_02870W_A	1.787582	0.122756	3642972

C1_14030W_A	1.78708	0.03836	3643340
C6_02940C_A	1.78571	0.037219	3639187
C2_08390W_A	1.78457	0.006685	3648252
MET14	1.78401	0.185782	3641547
C6_03990C_A	1.780818	0.268889	3640271
VPH1	1.779083	0.004849	3646157
RPN3	1.778804	0.005533	3636917
CDC27	1.773771	0.120275	3640405
TYS1	1.773627	0.003621	3637870
AGP2	1.772964	0.007119	3635684
DCW1	1.772411	0.009405	3638842
DDI1	1.77096	0.015111	3643295
C1_07050C_A	1.770696	0.083876	3639532
C1_03470C_A	1.770211	0.758169	3636912
C1_14020W_A	1.770211	0.758336	3643339
C7_02210W_A	1.770065	0.755917	3636990
CR_04400W_A	1.770065	0.755917	30515393
C3_02070C_A	1.769919	0.756301	3636576
C4_00730C_A	1.769919	0.756373	3645765
C7_01380W_A	1.769919	0.756638	3645891
RNY11	1.769919	0.756774	3636408
ZCF4	1.769919	0.756206	3641799
C6_00080C_A	1.769741	0.19138	3647899
VRP1	1.767481	0.010664	3642293
SWI4	1.766744	0.061846	3645408
C1_00310W_A	1.766619	0.026692	3639298
C4_06170C_A	1.76477	0.040707	3642818
CR_00490W_A	1.764487	0.03848	3639723
C1_01400C_A	1.76368	0.033988	3640237
C1_01220C_A	1.763266	0.037582	3640210
C6_03110C_A	1.762501	0.553606	3639278
C4_05000W_A	1.762463	0.549796	3641149
C2_01040W_A	1.762174	0.54909	30515085
C2_01570W_A	1.762174	0.549323	3635782
CTF18	1.762042	0.104522	3640431
EFH1	1.761886	0.556165	3646929
ZCF26	1.761479	0.429053	3635666
C2_10730W_A	1.760943	0.429071	3641210
C4_02160C_A	1.759124	0.028852	3635668
GRP2	1.758948	0.015082	3637744
C1_12430W_A	1.758702	0.06129	3642013
CHS5	1.757859	0.003904	3637333
PEX19	1.756846	0.021663	3642108
ACE2	1.756629	0.042905	3646034
C3_00010C_A	1.756355	0.049234	3640600
C3_01310W_A	1.756123	0.269064	3636620
CST20	1.753875	0.044039	3637790

SIN3	1.753583	0.005768	3639282
C2_10720C_A	1.753177	0.04842	3641209
QCR9	1.749939	0.051711	30515251
C1_04970W_A	1.748525	0.013088	3647528
MSO1	1.74769	0.033068	3642040
VPS2	1.745668	0.055263	3641546
RPS27	1.74533	0.006306	30515429
GGA2	1.744866	0.008488	3636928
C1_12120W_A	1.742056	0.200853	3642053
AGE1	1.741659	0.091479	3636913
MCM1	1.741572	0.044768	3638050
C6_02160W_A	1.734789	0.266727	3645889
CR_05460W_A	1.73308	0.099744	3648237
ZCF19	1.731829	0.573656	3644869
AYR2	1.731571	0.041572	3639184
PEX2	1.730986	0.039458	3643460
URA1	1.730346	0.006401	3634819
C1_08690W_A	1.729782	0.086973	3634934
C3_00960W_A	1.728783	0.18655	3639853
VPS16	1.727972	0.043476	3646150
ASH1	1.726778	0.081536	3641249
RAM2	1.725706	0.095466	3634767
VPS33	1.72418	0.111505	3647477
MID1	1.721714	0.097827	3647441
C1_00790W_A	1.720141	0.376534	3639348
C7_01670W_A	1.720037	0.379907	3636982
C3_06540C_A	1.71941	0.381402	3642274
C1_00510W_A	1.719354	0.051498	3639311
PCT1	1.718016	0.020983	3645743
DFG10	1.714641	0.294878	3645786
GPH1	1.714466	0.037448	3637956
C3_01220W_A	1.71349	0.200056	3636610
ANP1	1.713096	0.128203	3643917
RPL30	1.712687	0.025249	30515273
C1_11250W_A	1.712611	0.12724	3644556
C5_01110W_A	1.711459	0.047373	3642623
PSY2	1.711449	0.189819	3644567
CCT2	1.709674	0.020936	3643815
STB5	1.707748	0.048424	3640208
ROT2	1.705698	0.02266	3641566
FAV3	1.705661	0.223412	3647911
CR_10120C_A	1.702346	0.461937	3638969
IPL1	1.699029	0.267562	3645876
C1_14040W_A	1.698712	0.053098	3643342
SGT1	1.697913	0.012734	3644068
C4_00170W_A	1.6968	0.023205	3645028
VPS41	1.695946	0.092544	3634800

C2_02000W_A	1.695673	0.022192	3635767
HYS2	1.695083	0.144386	3636441
SWD3	1.694945	0.259941	3645890
YAE1	1.692923	0.209723	3637941
SEC1	1.690691	0.047279	3636935
C1_04860W_A	1.685619	0.01802	3644784
C5_04920C_A	1.684323	0.015362	3636419
RAD6	1.682982	0.132472	3643143
RDH54	1.682975	0.10216	3641231
STT4	1.681366	0.008926	3647963
CR_07220C_A	1.681066	0.154421	3646040
C4_06950W_A	1.679015	0.399602	3642927
C1_05900W_A	1.678175	0.084511	3639866
PHO88	1.673865	0.089383	3641898
C6_00250W_A	1.673668	0.020615	3646063
ALT1	1.673342	0.068668	3638307
BMT8	1.673122	0.125495	3637442
C1_13810W_A	1.671714	0.083622	3636126
SEC5	1.671256	0.048698	3648178
SSZ1	1.670159	0.019567	3641139
C3_01790C_A	1.668534	0.105146	3636587
MGM101	1.667718	0.121736	3636820
CR_04460C_A	1.667589	0.237264	3640988
MRPL19	1.665664	0.207377	3639497
C4_04870C_A	1.663313	0.011034	3641160
C4_03730C_A	1.662784	0.209998	3645358
MGE1	1.662469	0.028686	3640133
C1_06710W_A	1.661662	0.117744	3639510
C2_01960C_A	1.659939	0.596369	3635796
C2_04450W_A	1.659939	0.596284	3645467
C2_07770C_A	1.659803	0.594344	30515144
C6_02300C_A	1.659803	0.593963	3645874
PHO112	1.659431	0.596005	3644929
C4_04750W_A	1.658711	0.057796	3641133
SNF4	1.658037	0.025248	3640264
C1_10630C_A	1.65793	0.381454	3647683
FDH3	1.655973	0.012693	3638923
C2_10630W_A	1.655915	0.105549	3641248
ARP4	1.654074	0.083029	3639201
C4_05630W_A	1.653871	0.048679	3641812
RPS19A	1.653549	0.025101	30515208
PUP3	1.653365	0.035616	3646718
RPS9B	1.653315	0.015132	30515104
C1_05950C_A	1.653122	0.158653	3639859
CRK1	1.649838	0.05859	3643414
C7_04060W_A	1.644738	0.487142	3643156
GUT2	1.641198	0.014792	3642921

ALG7	1.641095	0.163996	3642291
TCP1	1.640348	0.025905	3646499
ULP1	1.638663	0.159243	3638314
PRT1	1.638327	0.023724	3636959
SMI1B	1.637905	0.42689	3643675
C1_02210W_A	1.637	0.042285	3644594
C2_08690C_A	1.635892	0.389216	3643888
PGA59	1.635122	0.011098	3637881
C3_03060W_A	1.634445	0.39087	3638395
AQY1	1.632578	0.174222	3642587
KIN2	1.630047	0.017555	3639697
C5_05150C_A	1.629675	0.216864	3636461
C2_05590C_A	1.628752	0.174327	3645564
UBP6	1.627684	0.016715	3639317
SFI1	1.626541	0.102631	3641385
TIM10	1.626358	0.153656	30515247
NIP1	1.625839	0.013799	3635618
C4_06020C_A	1.622981	0.110369	3646519
COX13	1.622601	0.020137	3635784
C7_04010W_A	1.622078	0.022591	3643112
SUP35	1.621845	0.022202	3643785
URE2	1.620858	0.111865	3647795
MCM3	1.620469	0.123834	3645223
C5_03100C_A	1.620329	0.098991	3637704
C2_02620W_A	1.619658	0.098669	3635838
PRE1	1.618882	0.028821	3636500
C1_06390W_A	1.618051	0.178993	3639558
C2_04300C_A	1.616517	0.184204	3648276
NRG1	1.613871	0.026131	3643166
PUP1	1.613827	0.017256	3638936
ATM1	1.612339	0.103347	3646284
DTD2	1.611829	0.081144	3638390
PKC1	1.611791	0.019843	3635210
SIW14	1.611712	0.268247	3647323
TDH3	1.610474	0.024986	3638540
C1_03370W_A	1.610355	0.018385	3636891
SAL6	1.610293	0.040388	3640289
C7_00210C_A	1.609437	0.141867	3637983
RIM11	1.60931	0.038208	3648277
C4_06690C_A	1.607812	0.068747	30515285
CR_06260W_A	1.605434	0.181028	3644853
C3_06800C_A	1.605129	0.177274	3638497
C5_02500C_A	1.604108	0.073057	3637766
TOA2	1.602385	0.184557	3635710
C2_07290W_A	1.60209	0.026375	3643476
C2_00290W_A	1.601858	0.251743	3638801
C4_01950W_A	1.601476	0.070615	3635665

C3_05120C_A	1.599322	0.107407	3635159
C7_02510W_A	1.599239	0.19659	30515357
CR_08930C_A	1.59857	0.074775	3641913
C1_14520W_A	1.595471	0.041014	3643332
LKH1	1.594967	0.017871	3647187
C7_02560W_A	1.591004	0.116877	3637016
CR_05980W_A	1.588677	0.028948	3642501
GAR1	1.588577	0.056911	3645332
CR_01350C_A	1.588499	0.028339	3640166
TLO13	1.588489	0.321772	3647891
ABP140	1.588236	0.325852	3644592
PRE10	1.587175	0.036088	3636957
C4_00940W_A	1.586399	0.521984	3635677
PDK2	1.58637	0.026319	3641876
CR_06930W_A	1.585854	0.241856	3648382
FOL1	1.585351	0.138784	3644123
TIM22	1.581636	0.0873	3648168
UBP1	1.581198	0.062187	3641760
C6_02680W_A	1.580515	0.179538	3639226
ACF2	1.580299	0.115753	3641613
TIM44	1.580025	0.031263	3640529
TIF34	1.57934	0.031415	3636872
TAF60	1.574247	0.079177	3642252
C7_02920W_A	1.573735	0.418651	3641391
C2_05760C_A	1.573596	0.096991	3645578
TRM1	1.573297	0.145148	3640396
NUP82	1.568361	0.029741	3640261
C1_13690C_A	1.568115	0.055356	30515076
OPT6	1.567205	0.296948	3635603
C1_08520C_A	1.566044	0.311191	3646495
C1_10620W_A	1.565238	0.042477	3647682
C3_00660W_A	1.564272	0.122361	3640538
C2_09070C_A	1.563785	0.150141	3644111
ISN1	1.560247	0.033675	3640196
C1_08110W_A	1.559816	0.044304	3640716
CR_04720C_A	1.558892	0.0469	3648140
C6_00210W_A	1.557857	0.08568	3646060
C2_02660W_A	1.557472	0.307638	3643009
C4_03300C_A	1.557408	0.646809	30515254
C2_09850C_A	1.557314	0.646921	3643782
IHD2	1.557041	0.307177	3639359
EMC9	1.55429	0.02623	3647918
SAM2	1.553248	0.087539	3644538
SAS3	1.552916	0.172177	3640073
NCE103	1.552459	0.227925	3636621
CHS7	1.552403	0.169241	3639869
VTI1	1.551818	0.091122	3638352

C3_01620W_A	1.550806	0.445412	3636663
C2_05710C_A	1.550721	0.04109	30515127
NCB2	1.548302	0.152991	3643028
C6_00500C_A	1.548254	0.286312	3647149
COI1	1.548076	0.099264	3640721
C7_02630W_A	1.547594	0.194597	3647646
NGG1	1.547365	0.090374	3636902
C3_05090C_A	1.546311	0.103557	3635245
C7_00310C_A	1.546309	0.253714	3638045
RHR2	1.545759	0.073769	3640522
C7_00790W_A	1.545337	0.160239	3637943
PAP1	1.545317	0.096736	3643763
C2_00350W_A	1.544291	0.164608	3638827
C1_06560W_A	1.54346	0.160917	3639517
RUB1	1.541253	0.361278	30515194
SAC7	1.539425	0.082468	3638010
SPE2	1.53877	0.125307	3644116
DAM1	1.535533	0.256873	3634820
NUP188	1.534444	0.06279	3634858
C1_10190W_A	1.53245	0.04141	3634785
C2_08830W_A	1.531953	0.069915	3643894
PEX14	1.528442	0.064801	3648208
C1_12640W_A	1.527841	0.5562	3646837
PR26	1.524727	0.034628	3642981
CR_02390W_A	1.524648	0.071649	3644930
C1_04230W_A	1.524557	0.094543	3644722
VPS53	1.524346	0.068127	3639319
MNN9	1.523712	0.157936	3641735
RPS42	1.52131	0.039411	3640195
GTR1	1.52055	0.333859	3643914
MDG1	1.519808	0.04819	3643311
C3_07850W_A	1.51966	0.109342	3647466
NIP100	1.518615	0.143515	3640970
OFR1	1.516403	0.049511	3640710
C2_03130W_A	1.516107	0.215404	3642993
C6_02810C_A	1.513339	0.149156	3639248
FEN12	1.512911	0.186828	3637404
UGP1	1.511641	0.058367	3648144
C2_03170W_A	1.510263	0.595054	3642988
C1_06970C_A	1.509157	0.087071	3639567
CR_08350W_A	1.50799	0.04872	3642082
C5_01880C_A	1.506996	0.235334	3643751
CR_01560W_A	1.505233	0.426947	3640075
C5_00550C_A	1.502962	0.380667	3641535
C3_03430C_A	1.50248	0.346465	3638355
C5_04360C_A	1.502237	0.072155	3636483
C2_08720W_A	1.501939	0.201187	3643885

AGE2	1.501937	0.116986	3643810
RPS10	1.501423	0.081977	30515149
SYS3	1.500488	0.1194	3645119
C3_01120W_A	1.497543	0.275996	3639836
BAT21	1.496622	0.128973	3637356
PHO89	1.495978	0.203978	3635633
EDC3	1.495171	0.235494	3646152
C1_04790W_A	1.494783	0.145034	3644772
NIT2	1.490664	0.219363	3641874
C6_00900C_A	1.489759	0.347267	3644029
PRE9	1.486859	0.117633	3638311
C4_02450W_A	1.486759	0.204581	3637845
GPI19	1.486554	0.52808	3643471
TUP1	1.48601	0.056279	3639369
RTF1	1.48535	0.069205	3637964
ENT3	1.481639	0.060354	3635813
HSP60	1.481167	0.053724	3645275
C1_03630W_A	1.48029	0.196194	3636882
C3_07760C_A	1.480209	0.439753	3638450
SRT1	1.478817	0.3084	3642020
MCT1	1.478514	0.267219	3646683
ECM29	1.478371	0.073997	3638433
RPN5	1.475665	0.059618	3636405
C2_03890W_A	1.475578	0.50426	3637396
C1_08180C_A	1.475274	0.083551	3640743
SGO1	1.473893	0.254291	3643464
RIM13	1.473885	0.170576	3636487
C1_08470W_A	1.473258	0.26583	3646490
C6_03260W_A	1.473141	0.14863	3639183
C4_05180C_A	1.47303	0.103664	3647413
C2_08660C_A	1.471253	0.497115	3643907
C1_09620C_A	1.470957	0.308471	3634892
CAF16	1.47086	0.068138	3646486
C2_06320W_A	1.46821	0.135433	3646927
CR_10500C_A	1.467895	0.242547	3638982
CR_07340C_A	1.467249	0.185206	3646056
CDC13	1.466871	0.354304	3639293
C1_13490C_A	1.465581	0.386697	3636230
IFF5	1.465066	0.153481	3642782
C7_02850W_A	1.464767	0.066218	3641399
C1_11970C_A	1.464217	0.280306	3642044
IRS4	1.464126	0.155353	3645650
APE2	1.462326	0.062117	3647990
PHM5	1.461904	0.145902	3637951
GYP2	1.461674	0.132464	3641780
C5_03830C_A	1.461438	0.185317	3647227
C1_09910C_A	1.460646	0.422027	3634798

C1_09110W_A	1.460325	0.41891	3634940
PTH2	1.460156	0.225383	3637709
PRE8	1.460124	0.090788	3641906
RPL82	1.456953	0.061607	3644419
C1_01530C_A	1.455503	0.064527	3640232
ALG11	1.454967	0.460735	3645871
GSG1	1.45449	0.350029	3647609
RIB3	1.450225	0.069055	3642062
EXO84	1.449806	0.15057	3644022
CR_04450C_A	1.446188	0.083322	3640987
C3_03590W_A	1.445874	0.100985	3638302
C1_06590C_A	1.445079	0.097064	3639514
C7_02220C_A	1.443797	0.098954	3636989
C7_01350C_A	1.440001	0.367515	3645899
SPO75	1.439622	0.269083	3645364
TRP2	1.436866	0.176914	3640079
SNG4	1.435564	0.234663	3646714
C3_03150W_A	1.435375	0.172128	3638423
C6_01960W_A	1.434352	0.557507	3644559
HGT19	1.434158	0.070252	3640558
PHO85	1.433467	0.150299	3646148
SRP101	1.431595	0.141781	3636513
CR_04120C_A	1.431028	0.298829	3640972
C4_06730C_A	1.430572	0.083169	3642924
RAD14	1.429187	0.274313	3636953
SEC14	1.427705	0.078475	3641542
C4_05220C_A	1.42759	0.413063	3646155
C5_03480C_A	1.427533	0.181403	3646965
NTF2	1.426969	0.140837	30515039
TIF35	1.426583	0.125871	3643308
POM152	1.426038	0.104929	3638812
TOM7	1.425878	0.395906	30515355
C5_02390C_A	1.425501	0.158523	3637795
GDS1	1.425304	0.09099	3642626
C2_01720C_A	1.424257	0.3644	3635731
GSH2	1.422991	0.104409	3642083
CR_09490W_A	1.422987	0.179989	3641896
C2_10870W_A	1.422835	0.223759	3641234
CR_10620C_A	1.422227	0.102365	3639006
CRM1	1.421973	0.09471	3639721
C2_00750W_A	1.419394	0.540567	3638911
CCT7	1.418936	0.115644	3643734
SUA72	1.417693	0.400291	3648239
MDL2	1.417623	0.189674	3639256
C6_02640C_A	1.416211	0.251179	3639222
C1_05660C_A	1.415936	0.07996	3639900
C5_01090C_A	1.415274	0.179454	3642625

REX2	1.413159	0.317888	3635783
MRV2	1.412834	0.463909	3636545
C7_03010W_A	1.412192	0.102171	3641382
C2_07910C_A	1.410884	0.636129	30515146
C6_03150C_A	1.409961	0.222942	3639257
C3_05760W_A	1.409168	0.182354	3641720
C1_08860C_A	1.408515	0.226477	3634921
VAC8	1.40739	0.11834	3647410
CR_00470W_A	1.407351	0.113442	3639725
C7_00010C_A	1.406524	0.183065	3638006
PRE6	1.405865	0.141748	30515395
C1_00760W_A	1.405529	0.273444	3639351
C1_11560C_A	1.405484	0.374419	3645331
CDC45	1.4054	0.372917	3648293
NUP85	1.404606	0.107955	3635175
C6_00800C_A	1.402972	0.104914	3647069
C1_08160W_A	1.402574	0.185832	3640733
C1_14470W_A	1.402124	0.185257	3643327
HHT1	1.397841	0.097584	3638444
FET31	1.397652	0.179847	3647151
DAP2	1.3974	0.216415	3637815
C1_06640C_A	1.396716	0.497271	30515010
PRP13	1.396488	0.149716	3643194
C2_04360W_A	1.394971	0.269789	3645461
C2_10060C_A	1.39383	0.591342	3643948
BZZ1	1.392953	0.132674	3636572
RVS162	1.392917	0.288036	3646820
CR_03690W_A	1.391186	0.213633	3643222
C1_10110W_A	1.39089	0.460102	3634778
C3_02970C_A	1.390725	0.326241	3638385
MED17	1.390271	0.250189	3636918
C3_00170C_A	1.387685	0.387164	3640589
MIA40	1.38605	0.194132	3636866
CR_02610C_A	1.38555	0.110417	3642566
C1_05010C_A	1.385249	0.324202	3647532
CEF3	1.384338	0.09197	3647040
C5_03530C_A	1.384267	0.573973	3646971
RPC31	1.384236	0.362588	3642577
TRP5	1.383363	0.129792	3648087
C3_03680W_A	1.382774	0.172498	3645648
SMT3	1.382443	0.129491	3644550
C4_01790W_A	1.381347	0.247035	3635597
CR_08640C_A	1.381212	0.193474	3642112
C1_08340C_A	1.378246	0.366244	3640752
C2_02180W_A	1.37539	0.257762	3635752
SDS22	1.374312	0.155715	3637362
CCT3	1.37425	0.186131	3636458

C4_05820W_A	1.371757	0.476268	30515277
POS5	1.371356	0.242294	3638519
C4_02860W_A	1.370754	0.11867	3637909
PEP8	1.369878	0.214668	3645624
UAP1	1.368188	0.197662	3637769
CR_09830W_A	1.368132	0.564898	3638986
MODF	1.367446	0.287311	3636182
C5_01190W_A	1.367067	0.283724	3642659
C7_04240C_A	1.366752	0.134935	3643165
C1_12000C_A	1.366193	0.754609	3642041
C1_14560C_A	1.363987	0.21226	3648376
C4_02430W_A	1.363086	0.25036	3637848
ZCF16	1.362694	0.330815	3647395
C2_02170W_A	1.362103	0.336623	3635751
C4_04130W_A	1.361377	0.289624	3641221
C1_12250C_A	1.361024	0.145	3642022
KRE9	1.360891	0.217268	3635183
HSP90	1.360772	0.122683	3636951
BAT22	1.359651	0.152379	3643663
LAP4	1.359311	0.234955	3646979
CR_08430W_A	1.359133	0.153798	3642120
RAD50	1.357908	0.228618	3636594
C5_05270C_A	1.357607	0.243005	3636496
C1_06420C_A	1.356527	0.352968	3639554
MLT1	1.356134	0.131102	3640748
BEM2	1.356104	0.181431	3637044
YPT1	1.355115	0.141279	3636915
ERF1	1.354671	0.206209	3643455
VPS15	1.354329	0.244252	3644040
SPT20	1.35383	0.280015	3642492
SMD3	1.353454	0.516678	3647060
C1_03660W_A	1.353073	0.624416	30514982
TIM8	1.352966	0.516049	3647469
RVS167	1.352896	0.132845	3647831
C2_08320C_A	1.352807	0.677229	3648167
FGR6-1	1.352697	0.677483	3647947
PGA32	1.352476	0.681705	3638537
C7_02010C_A	1.352018	0.756035	3636954
C2_07620W_A	1.351989	0.754623	3645194
SAP8	1.351989	0.754508	3638371
C3_02160C_A	1.351917	0.186097	3636654
C4_04950W_A	1.351908	0.755425	3641168
C2_09630C_A	1.351797	0.755208	3643796
FAV2	1.351797	0.756548	3647236
C5_03370C_A	1.351767	0.758548	3646350
C5_01260W_A	1.351686	0.758191	3642615
GUK1	1.351467	0.358257	3647231

C5_00150C_A	1.34987	0.119244	3647872
C3_00730W_A	1.347703	0.266257	3640530
GIS2	1.347117	0.139085	3643744
C2_01800W_A	1.346687	0.872605	30515092
C2_02790C_A	1.346687	0.872605	3643029
CR_08260C_A	1.346687	0.872605	3642100
DAL52	1.346687	0.872605	3643736
KRE62	1.346687	0.872605	3641543
PGA19	1.346687	0.872605	3638894
C1_06160W_A	1.346576	0.87264	3639846
C4_00250W_A	1.346576	0.87264	3645035
CR_03300C_A	1.346576	0.87264	3645098
PGA61	1.346576	0.87264	3640292
C2_04500W_A	1.345858	0.18414	3645462
C5_00850C_A	1.342472	0.768209	3642637
C1_10240C_A	1.342373	0.448854	3634881
RPT5	1.341008	0.138106	3642911
C1_12760W_A	1.339507	0.411892	3646817
PRR2	1.338723	0.568459	3646723
CR_10290C_A	1.33629	0.255404	3638935
CR_06780W_A	1.336132	0.266928	3647312
MSH3	1.335628	0.383162	3643889
GCR3	1.334604	0.180578	3646485
CDC48	1.334372	0.170846	3644440
C2_05900W_A	1.334002	0.877484	3647475
RPB8	1.330783	0.438741	3647301
C1_05750C_A	1.330598	0.218983	3639891
ACH1	1.328141	0.214098	3643773
C4_01430C_A	1.327738	0.530246	3635625
C3_07420W_A	1.326988	0.165787	3638485
PIN4	1.326965	0.188652	3640101
C3_00640W_A	1.326722	0.245155	3640574
C1_09940W_A	1.324017	0.597839	30515037
C3_07110W_A	1.323371	0.387897	3638442
C3_07690C_A	1.321719	0.273805	3638458
YIM1	1.317784	0.288942	3637372
ORC4	1.317285	0.293195	3637753
CR_03430W_A	1.315952	0.536583	3645114
NOP1	1.315816	0.20584	3642947
CR_01050C_A	1.315166	0.391351	3640384
PEP7	1.315085	0.40116	3645001
RRD1	1.314514	0.473436	3638445
RPN10	1.313804	0.190057	3644080
AGP3	1.312736	0.385796	3641163
DAD4	1.312477	0.65836	30515433
C2_00390C_A	1.312382	0.252952	3638822
CR_10380C_A	1.312184	0.656121	3638953

TIF11	1.311432	0.254615	3641208
TIM54	1.310895	0.286021	3641349
C3_02390W_A	1.310706	0.573104	3636615
C4_01300W_A	1.310696	0.29871	3635605
FGR22	1.309538	0.496574	3635862
C6_01210W_A	1.308219	0.300493	30515330
TRR1	1.307443	0.198643	3637778
C3_03380W_A	1.30722	0.360952	3638367
CUE5	1.305906	0.170745	3642467
RPL40B	1.30509	0.180386	30515232
PRE5	1.304929	0.225487	3643111
CR_03240C_A	1.304341	0.445495	3645126
FAA4	1.303936	0.217786	3638973
ATO1	1.302834	0.653175	3647761
CFL5	1.301929	0.616353	3642612
C2_02150C_A	1.300777	0.302037	3635749
PCL7	1.298526	0.210628	3639491
C4_00860C_A	1.297587	0.389209	3635717
C5_00230C_A	1.294523	0.724402	3641564
C3_02580C_A	1.294486	0.597744	3638376
C6_04230W_A	1.294234	0.724052	3646274
FUN12	1.294122	0.204334	3640713
CCT6	1.293413	0.211172	3642914
C1_03620C_A	1.29293	0.31173	30514981
IFU5	1.291244	0.251493	3640116
C7_04120W_A	1.290467	0.643871	3643132
C6_02540C_A	1.290363	0.64451	3639176
CDC15	1.289247	0.359721	3643459
C2_08900W_A	1.287423	0.454758	30515156
C3_02870C_A	1.287196	0.504427	3638334
C3_06830C_A	1.286484	0.290974	3638543
C3_05990C_A	1.286461	0.671724	3641732
C6_04370W_A	1.283641	0.467759	3646294
CR_09040W_A	1.283146	0.450714	3641894
C4_01650C_A	1.282906	0.434436	3635713
LEA1	1.280023	0.619635	3641807
C4_03960W_A	1.279582	0.275369	30515264
C7_01600W_A	1.279031	0.385818	3637036
CR_08300C_A	1.278991	0.212388	3642076
C4_00700C_A	1.278888	0.463098	3645751
C4_05610C_A	1.278222	0.229385	3641782
PRE3	1.272843	0.249748	3643660
C3_06110C_A	1.272027	0.357262	3641761
RPS27A	1.271609	0.697049	30514993
VPS36	1.269853	0.361028	3637354
C1_09570W_A	1.269711	0.447575	3634887
ASR3	1.269338	0.224193	3637402

C2_06040C_A	1.26752	0.623055	3644069
CR_06520C_A	1.267418	0.619096	3645263
C4_04250W_A	1.266665	0.551764	3646855
CR_09240C_A	1.266623	0.388944	3641919
C5_00560W_A	1.266274	0.336706	3648267
C2_06430C_A	1.266097	0.470173	3646558
C7_01180W_A	1.264048	0.428184	3645915
MRS7	1.263151	0.239124	3640187
IDI1	1.261054	0.374902	3637894
C1_11790W_A	1.260275	0.609912	3645326
C4_00500W_A	1.259637	0.351622	3648379
C7_00060C_A	1.259626	0.421192	3638013
C1_11910W_A	1.258871	0.678274	3642004
C4_06880C_A	1.25875	0.441941	30515287
HIR1	1.258738	0.248535	3638796
CR_03760W_A	1.258446	0.432721	3643178
ZCF6	1.257979	0.533109	3635837
C7_03990C_A	1.25604	0.473758	3643115
TIM9	1.255607	0.581865	3646243
C3_02850C_A	1.253471	0.647213	3638332
MEX67	1.25206	0.360181	3641020
CDC23	1.252058	0.465819	3642111
C7_04260W_A	1.250247	0.322202	3643160
DAL7	1.249487	0.820974	3636130
C2_05390C_A	1.249384	0.819711	3643434
PGA26	1.249357	0.820013	3639890
PGA42	1.249179	0.821069	3642801
C2_04390W_A	1.249077	0.824597	3645458
C4_05040W_A	1.249077	0.824384	30515275
MVD	1.246553	0.2614	3639306
C1_02510W_A	1.245748	0.649727	3636796
GSY1	1.243622	0.404	3640435
MNN13	1.242754	0.629684	3637720
C1_09220W_A	1.242562	0.392274	3634898
CR_10820W_A	1.23833	0.544507	3638958
CR_04300W_A	1.237987	0.255127	3640995
C4_05210W_A	1.23767	0.59342	3646154
SLP2	1.236418	0.519237	3644091
C6_04190C_A	1.235627	0.776625	3646282
C4_03150W_A	1.235375	0.403839	3637842
SPS20	1.234527	0.466533	3645739
C4_00420C_A	1.234018	0.324687	3645021
PGM2	1.233922	0.359975	3642562
POX1	1.233182	0.34009	3640247
C3_03260W_A	1.232432	0.320614	3638320
C3_01170W_A	1.232399	0.42016	3647958
C2_04780W_A	1.232018	0.383243	3647781

ZCF22	1.232006	0.781055	3637727
C1_03950C_A	1.230999	0.738698	3646417
BIO32	1.230798	0.735784	3643430
MET8	1.230714	0.557089	3638533
CR_08520C_A	1.230398	0.738695	30515443
C1_10920W_A	1.230057	0.511408	3644433
MRPL33	1.228955	0.618429	30515175
C1_02200C_A	1.228112	0.59511	3644593
C1_00910W_A	1.228032	0.715053	3639284
GAP6	1.227086	0.297857	3646968
CDS1	1.226408	0.324806	3647729
C1_02030C_A	1.224823	0.669233	3645415
C1_07060C_A	1.224701	0.674733	30515018
C1_03920C_A	1.224602	0.674686	3646413
C1_09440W_A	1.222639	0.488948	3634857
TES15	1.220855	0.579804	3647478
NTH1	1.220835	0.373358	3639665
C6_01950C_A	1.220482	0.496879	3644558
SIT4	1.219757	0.393597	3647993
C4_03560W_A	1.219607	0.4527	3645373
C1_02220C_A	1.217373	0.514561	3644595
C2_08630C_A	1.216226	0.430601	3643910
C7_04110W_A	1.215143	0.526728	3643133
RTT101	1.21289	0.50774	3639827
IFD6	1.21232	0.731809	3644703
TRA1	1.211864	0.387567	3641644
ZCF25	1.210994	0.721127	3635694
RPC11	1.209792	0.609304	3641239
SFL2	1.209238	0.553132	3636434
C1_04530C_A	1.20831	0.515592	3646149
C5_04980W_A	1.208285	0.522623	3636536
C4_01660W_A	1.208141	0.377343	3635712
C2_04960C_A	1.207789	0.635472	3643442
LYP1	1.207789	0.635839	6335417
C4_02670W_A	1.207613	0.573492	3637830
AVT1	1.207255	0.538611	3646928
C1_08540C_A	1.206263	0.373635	3646497
MIM1	1.206105	0.506081	30515394
TIP120	1.205277	0.376732	3638494
C1_02720W_A	1.204829	0.545113	3636825
C2_06300W_A	1.20329	0.664786	3646924
C7_01950W_A	1.202025	0.649258	3636970
LRG1	1.202015	0.450524	3639727
FUR1	1.19906	0.502237	3646348
C3_07770C_A	1.198156	0.626458	3647457
C1_11670W_A	1.197682	0.41112	3645338
STN1	1.19747	0.864336	3643901

C5_00380W_A	1.195848	0.584527	3641553
VAN1	1.195597	0.475668	3638527
C1_05630C_A	1.195301	0.383741	3639840
ZCF11	1.194296	0.547729	3645109
C5_02560C_A	1.193802	0.452767	3637719
C4_03130W_A	1.192517	0.488818	3637921
C1_12470W_A	1.191226	0.818627	3648182
C2_05520W_A	1.190731	0.681657	3643421
MED14	1.189243	0.495569	3637800
UGA33	1.188436	0.557309	3641886
C4_03040W_A	1.187946	0.632997	30515253
C4_06770W_A	1.186676	0.557996	3642920
C1_03960C_A	1.185321	0.63437	3646418
C5_00090C_A	1.184409	0.496412	3647885
SEN1	1.183992	0.496546	3635255
MRR2	1.183765	0.492004	3647468
EMP46	1.183418	0.508644	3645270
VPS22	1.181571	0.732766	3647250
COX15	1.181005	0.381912	3647071
C1_11090C_A	1.18046	0.543819	3644415
DOS2	1.179473	0.460537	3636183
C3_07630C_A	1.179192	0.55754	3638491
TAF4	1.178493	0.493472	3645198
MAF1	1.178265	0.440799	3642332
C1_04150C_A	1.177956	0.827473	3644704
MXR1	1.177841	0.553655	3638881
SSN8	1.177773	0.649347	3641718
BEM3	1.175718	0.528692	3637885
C1_07100C_A	1.175286	0.755219	3639487
FLO9	1.174609	0.595255	3640581
C4_03160C_A	1.173432	0.747552	3637841
C1_13750C_A	1.172748	0.811327	3636137
SET1	1.172689	0.505796	3639280
C1_13950C_A	1.172261	0.805689	3636186
YKT6	1.171802	0.552768	3636863
C6_03550C_A	1.171362	0.729785	3640246
C2_05530C_A	1.171271	0.471577	3643422
C7_00910C_A	1.171017	0.579564	3637958
PIN3	1.169686	0.434288	3635228
YHM1	1.169395	0.686312	3644535
AXL2	1.168959	0.642234	3641184
C2_00060C_A	1.168664	0.46617	3647914
SPT3	1.1684	0.565385	3638977
NPL6	1.167995	0.524746	3638883
UCF1	1.166402	0.48529	3648170
C2_02500W_A	1.166265	0.53609	3635816
HIS4	1.165571	0.418981	3645025

C7_03610C_A	1.16513	0.548755	3646241
RPT1	1.164398	0.421516	3642461
SIM1	1.163952	0.452452	3636185
C1_14310W_A	1.163755	0.593006	3643315
C1_11430W_A	1.163489	0.741091	3644541
LCB2	1.16272	0.595149	3636181
POT1-2	1.16087	0.628384	3638908
CR_07470W_A	1.160738	0.760426	3646048
C6_00260W_A	1.159832	0.481424	3646065
C1_04700C_A	1.158367	0.51526	3644766
PTC1	1.157332	0.552008	3634827
C4_06440C_A	1.156801	0.718989	3642793
SKI2	1.15303	0.504984	3642143
C1_02770W_A	1.152395	0.775911	30514973
FAD2	1.151055	0.478294	3644053
C2_08240W_A	1.150681	0.511213	3642344
C1_11140W_A	1.150421	0.645858	3644410
C5_03670C_A	1.150055	0.696508	3646987
RIA1	1.149969	0.507912	3644478
C7_02960C_A	1.149709	0.675993	3641387
C1_04620W_A	1.148208	0.604755	3646135
TSC2	1.147402	0.629686	3646953
C3_02690C_A	1.147236	0.61947	3638407
PGA58	1.146853	0.889883	3637826
IME2	1.146759	0.889701	3645099
SLA2	1.146384	0.538031	3643149
C3_02090C_A	1.145517	0.666529	3636661
C5_01870W_A	1.145149	0.511238	3643752
C2_03800C_A	1.144724	0.518079	3637401
CR_05700C_A	1.144493	0.636585	3646701
SMF12	1.144048	0.564791	3643509
C2_09280C_A	1.143601	0.650072	3644102
GSL2	1.143424	0.598472	3640410
C3_03820C_A	1.141918	0.668027	3645631
SYN8	1.141223	0.761425	3645120
C1_06600W_A	1.140855	0.485969	3639513
ULP3	1.140781	0.645748	3640526
C1_08760W_A	1.139633	0.703053	3634787
C2_03260W_A	1.139208	0.574102	3637432
C6_04080W_A	1.138832	0.512022	3647837
C2_04320W_A	1.138811	0.720447	3648274
C5_05090W_A	1.137043	0.686144	3636493
C5_02140C_A	1.13699	0.588499	3637752
AVT4	1.136316	0.649406	3645316
C4_01120C_A	1.135441	0.747042	3635683
C1_12910W_A	1.134454	0.885715	30515066
C1_05610W_A	1.134422	0.689508	3639843

C7_00490C_A	1.134178	0.679983	3637986
MED5	1.13407	0.668065	3648211
FGR39	1.133993	0.821627	3644529
MRS2	1.133737	0.761284	3640110
C1_06340W_A	1.133465	0.823197	3639499
C5_04630W_A	1.132997	0.732931	3636395
MCU1	1.131291	0.757331	3636169
ECM7	1.131234	0.648908	3645029
C3_05420W_A	1.130009	0.655766	3643666
C2_00820W_A	1.129401	0.675954	3638809
GLK1	1.129205	0.570323	3648348
C1_13720W_A	1.129114	0.678548	3636134
AKL1	1.128602	0.54284	3641237
SKO1	1.127762	0.602793	3644688
C6_04650W_A	1.127281	0.551185	3645979
MNR2	1.12681	0.597661	3645017
PPZ1	1.126251	0.684643	3645265
C1_14460W_A	1.126034	0.552624	3643326
C1_02160W_A	1.125654	0.781582	3644584
C3_02490C_A	1.125596	0.595821	3638351
CR_07870W_A	1.125339	0.778585	3644146
SEC65	1.123598	0.621157	3640123
TIM50	1.123125	0.594743	3644530
C2_06490W_A	1.122602	0.645274	3646569
C4_00050W_A	1.121419	0.652693	3648060
C2_01760C_A	1.120273	0.796368	3635736
CR_03700C_A	1.119858	0.650771	3643223
C4_01310W_A	1.119443	0.879485	3635604
PGA15	1.119351	0.878839	3642781
SCS7	1.119244	0.632556	3641157
TIM13	1.119101	0.87943	3637868
ATO9	1.119034	0.880516	3640392
C1_05480C_A	1.119009	0.880427	3642486
C5_00950C_A	1.117849	0.638489	3642653
MOB1	1.117705	0.701482	3639208
BMT7	1.117455	0.822815	3638357
C2_01340W_A	1.117407	0.603987	3638844
C4_01720C_A	1.117266	0.580762	3635589
CR_05560W_A	1.117215	0.648027	3647592
TFA1	1.116256	0.668949	3634850
RAD2	1.115677	0.695609	3645374
PRP3	1.115492	0.730683	3637406
C1_12810W_A	1.114438	0.768648	3646827
FBP1	1.113742	0.600999	3647464
C4_06800W_A	1.112225	0.61281	3642917
C7_00270W_A	1.11173	0.667394	3637977
C7_00070C_A	1.111387	0.595343	3638011

HMT1	1.111263	0.633445	3640182
CR_03540W_A	1.111207	0.773465	3643215
RPO21	1.108193	0.620187	3638991
C2_04130W_A	1.108177	0.671014	3637334
C1_00970W_A	1.10815	0.867134	30514958
CR_05710C_A	1.107998	0.829627	3646700
LAB5	1.106908	0.639324	3637893
C5_04620C_A	1.106187	0.800192	3636394
PFK2	1.106155	0.640592	3637056
C4_03200C_A	1.104886	0.68764	3637837
MRPL10	1.102619	0.759637	3643446
C5_02350C_A	1.102446	0.859909	3637791
CR_01020C_A	1.10232	0.791844	3640420
HOS1	1.100911	0.789101	3646518
C6_01200W_A	1.100464	0.903085	3644041
C6_01220C_A	1.099787	0.710205	3644042
GLE2	1.098677	0.6919	3646293
C2_07220W_A	1.097171	0.694061	3643483
EAF7	1.096109	0.697154	3640973
CR_10830C_A	1.093861	0.79147	3638960
C4_04500C_A	1.0932	0.754933	3641123
C2_10300C_A	1.091585	0.863177	3643955
CDR3	1.091344	0.820117	3645365
C1_13790C_A	1.09115	0.819597	3636141
C2_08420W_A	1.090035	0.843561	3643897
CR_06030C_A	1.088744	0.766142	3644874
C5_05130C_A	1.088242	0.795295	3636459
ECM14	1.087818	0.731862	3638392
C6_01300W_A	1.085843	0.836362	3644024
PHO23	1.085463	0.742284	3643926
C5_04340W_A	1.08355	0.840061	3636481
HXK1	1.081317	0.749486	3645964
IRO1	1.081256	0.748055	3636648
RPL38	1.079974	0.719626	30515082
CR_02300C_A	1.079934	0.759119	3644938
MSS4	1.079752	0.693727	3647957
C7_00850W_A	1.078737	0.845234	3638056
GPD2	1.078468	0.735565	3644563
RAD23	1.077785	0.713449	3635834
IMP1	1.077381	0.876855	3636881
HPD1	1.074819	0.890304	3639239
C2_05350C_A	1.074574	0.884881	3643428
C1_01860W_A	1.074357	0.764379	3645430
SIK1	1.074191	0.735364	3638925
CGT1	1.074108	0.764716	3641808
PDC12	1.073207	0.882282	3635591
C6_03530C_A	1.073147	0.893181	3640260

EHD3	1.073088	0.768454	3636858
C5_01300C_A	1.07306	0.893935	3642646
CR_03400W_A	1.071824	0.811014	3645137
C1_00660C_A	1.071493	0.774028	3639376
C5_04720C_A	1.071028	0.804662	3636518
C1_03990W_A	1.070519	0.836063	3646424
DOA4	1.069796	0.754923	3643337
C7_02530C_A	1.068582	0.777934	3636940
GAL10	1.068416	0.750833	3644583
GCN2	1.068285	0.815773	3645897
C7_04170W_A	1.067745	0.916556	3643126
ARV1	1.067722	0.916799	3635248
DEM1	1.067683	0.852638	3643200
C2_05960C_A	1.067461	0.91771	3647479
C1_11930W_A	1.066285	0.741595	3642002
GCD11	1.065386	0.769293	3637755
ATF1	1.065115	0.837115	3635273
PRN2	1.064596	0.840183	3639879
SYS1	1.064546	0.871676	3643138
KIC1	1.064305	0.753285	3647784
C5_04640C_A	1.064072	0.747681	3636397
C5_01000C_A	1.063865	0.819707	3642651
C1_03280W_A	1.06386	0.778178	3636901
C6_00340C_A	1.063626	0.8333	3646074
C4_00580W_A	1.063094	0.83972	3645773
POL32	1.062779	0.839	3639881
CR_08110W_A	1.0627	0.883026	3642067
C3_03100C_A	1.060219	0.806137	3638399
TAF19	1.060122	0.858679	3641396
SAP98	1.058666	0.872036	3637435
GCN20	1.058521	0.795736	3639314
C1_03450C_A	1.057467	0.808616	3636910
PAN1	1.055818	0.784132	3637386
PHO81	1.055165	0.860987	3639662
LPG20	1.054856	0.84678	3644780
MNN15	1.054786	0.886696	3644794
C6_01850W_A	1.054661	0.782569	3641629
THI20	1.054198	0.867675	3637387
C1_07090C_A	1.054026	0.825998	3639488
C5_00040C_A	1.053795	0.803887	3647874
C1_00470C_A	1.053668	0.855489	3639315
DRG1	1.053595	0.822031	3640714
C7_00500W_A	1.052365	0.934341	3637987
C1_03100W_A	1.052291	0.83333	3636925
C4_05010W_A	1.051299	0.826442	3641148
C3_04120C_A	1.051164	0.8821	3635282
C6_00230W_A	1.048425	0.907898	3646061

CR_01420W_A	1.048236	0.847658	3640137
PRK1	1.047324	0.819621	3640153
SRD1	1.045748	0.908387	3635764
ZCF13	1.045464	0.864801	3646354
C3_04350C_A	1.044292	0.965015	3635275
UME6	1.044292	0.965009	3647522
C5_00510W_A	1.044229	0.965179	3641539
THI4	1.044229	0.965169	3635160
C2_10320C_A	1.044143	0.965269	3643944
CR_04390C_A	1.043402	0.873942	3640980
YPS7	1.042213	0.863654	3636937
CR_09660W_A	1.041911	0.918356	3647092
ECM38	1.041377	0.920232	3645375
C3_06280W_A	1.040763	0.87274	3642238
C1_11720W_A	1.039912	0.944478	3645319
CR_01950W_A	1.039767	0.869857	3640107
WSC4	1.039704	0.902959	3643282
ERB1	1.038829	0.846419	3644702
RAD59	1.038751	0.933245	3648281
C5_02040W_A	1.038638	0.880081	3643769
C2_01530C_A	1.037031	0.848098	3635803
C1_11730W_A	1.036768	0.869838	3645318
C3_02080W_A	1.036717	0.904938	3636575
GDA1	1.035016	0.882734	3641762
C2_10120W_A	1.034864	0.925745	3643936
SKI3	1.033659	0.866295	3634847
C4_04550C_A	1.033395	0.884134	3641106
C3_04410C_A	1.033054	0.867457	3635178
C5_00330C_A	1.031952	0.889092	3641556
C7_03980W_A	1.031492	0.917273	3643116
GLG2	1.031201	0.919824	3642282
ZCF17	1.029383	0.917649	3640205
SNF1	1.028657	0.886856	3642643
FGR28	1.028615	0.936883	3648315
C4_06430C_A	1.028049	0.937683	3642794
CR_06790C_A	1.028047	0.947674	3647311
MED7	1.027821	0.948267	3638344
FRP3	1.027673	0.896611	3648102
CR_06850C_A	1.025736	0.970593	30515424
DCR1	1.025531	0.916015	3641164
C1_06400C_A	1.025178	0.921085	3639557
DOT5	1.024556	0.920113	3640519
C1_01930W_A	1.02266	0.941662	30514966
C1_05520W_A	1.022547	0.965859	3642505
C5_01220W_A	1.0201	0.947487	3642620
LYS143	1.020013	0.965736	3634894
DBR1	1.017826	0.959612	3647560

GYP8	1.017187	0.950865	3637750
C2_08910C_A	1.017172	0.934053	3645798
NOT4	1.016436	0.936262	3645115
CR_02430C_A	1.01471	0.947428	3644924
CR_07310W_A	1.013134	0.971759	3646058
CR_06250W_A	1.012684	0.973622	3644852
CR_00570W_A	1.012193	0.958264	3639664
AVT7	1.012162	0.963721	3637433
MRPL8	1.010631	0.970714	3646039
C4_01290W_A	1.01003	0.969144	3635699
C1_07210C_A	1.009597	0.968092	3644473
C1_02740C_A	1.008384	0.987989	3636827
CR_00380W_A	1.008138	0.988501	3639682
C5_04320C_A	1.00804	0.969906	3636480
C1_09350W_A	1.007646	0.978799	3634805
NUP84	1.007583	0.974136	3645351
UBA4	1.00729	0.976115	3644432
SFC1	1.007042	0.985845	3636413
C4_00850C_A	1.006796	0.991771	3635718
C6_04260C_A	1.005992	0.989086	3646277
C5_02820C_A	1.00514	0.985251	3637808
C2_04790C_A	1.004753	0.988069	3647779
C5_02480W_A	1.004715	0.984438	3637762
C1_08910C_A	1.004539	0.991216	3634926
SLF1	1.003854	0.991018	3638500
C7_00300W_A	1.002789	0.988733	3638046
C7_00430W_A	1.002663	0.990444	3637993
C6_03240W_A	1.002648	0.997195	3639181
C2_03490C_A	1.002478	0.993063	3637346
ASG1	1.001586	0.994823	3647687
C1_09430W_A	1.000798	0.997463	3634855
C2_03140C_A	1.000473	0.998182	3642992
C1_12670C_A	-1.00032	0.999345	3646834
C5_00050W_A	-1.00052	0.99892	3647875
C3_01690W_A	-1.00153	0.995478	3636604
C1_02410C_A	-1.00179	0.995956	3636793
C5_01140C_A	-1.0021	0.992956	3642663
CR_09310W_A	-1.0024	0.992255	3641923
YPT52	-1.00268	0.991347	3643346
GLC7	-1.0027	0.989868	3647257
TLO4	-1.00316	0.995148	30515080
FGR14	-1.00344	0.987238	3648083
CR_01170W_A	-1.00349	0.986432	3640429
SEC7	-1.00416	0.983566	3635220
C4_02150C_A	-1.00435	0.992562	3635670
GCN5	-1.00447	0.985418	3646626
CR_07160C_A	-1.00476	0.984488	3648347

C4_06280C_A	-1.00544	0.988422	3642802
CR_03070W_A	-1.0059	0.977788	3645105
C4_06660W_A	-1.00704	0.985107	3642809
VID21	-1.00816	0.969201	3642946
UFE1	-1.00833	0.967053	3643174
C4_00560C_A	-1.00846	0.97536	3645744
KRE30	-1.00895	0.965657	3642311
RSR1	-1.00965	0.97298	3640145
SSN3	-1.01027	0.970947	3637353
C6_03420W_A	-1.01087	0.982383	3639235
FDH1	-1.01199	0.973396	3646398
YUH2	-1.01212	0.949788	3636412
CR_06960W_A	-1.0123	0.963083	3645737
FMP27	-1.01273	0.953629	3641645
RPT2	-1.01324	0.946043	3640525
C2_00740C_A	-1.01398	0.944506	3638912
KIS2	-1.01438	0.956876	3636211
SAC3	-1.01606	0.948653	3635823
C4_02880C_A	-1.01689	0.952292	3637907
C1_07400C_A	-1.01852	0.951223	3644464
C3_05680W_A	-1.01864	0.944972	3641724
C2_01490C_A	-1.01896	0.94298	3635800
SOD4	-1.01897	0.97595	3638888
GAL102	-1.0193	0.935769	3644585
CR_09340W_A	-1.01968	0.918808	3641900
PUS4	-1.0199	0.936082	3642661
C6_02800W_A	-1.0211	0.945968	3639246
C5_01920C_A	-1.02156	0.929966	3643742
TEP1	-1.02189	0.967576	3647653
C3_02410C_A	-1.02243	0.922074	3638341
C2_03530W_A	-1.0225	0.922035	3637341
COX9	-1.02291	0.930687	30515131
SAP9	-1.02307	0.908698	3645625
LIG4	-1.02548	0.888007	3642986
ARO1	-1.02573	0.898474	3635680
C1_13840W_A	-1.02577	0.905064	3636129
BCK1	-1.02731	0.909161	3641384
RPC40	-1.02795	0.908852	3643427
C1_02750C_A	-1.02907	0.89815	3636828
CR_00340C_A	-1.02948	0.955112	30515365
SEC9	-1.03094	0.880292	3644052
SSK2	-1.03141	0.873975	3641144
IFG3	-1.03142	0.926122	3641545
C2_05580W_A	-1.03183	0.902583	3645563
C1_00380C_A	-1.0322	0.874431	3639292
PSA2	-1.03252	0.873241	3636159
CDL1	-1.03287	0.894817	3641132

PCD1	-1.03453	0.887483	3647087
C5_00100C_A	-1.03503	0.888352	3647883
C1_04490W_A	-1.03521	0.856984	3646145
C2_00420W_A	-1.03568	0.885591	3638819
CTA3	-1.03616	0.867398	3645333
C1_13650C_A	-1.03712	0.892622	3636191
C1_00030C_A	-1.0373	0.903496	3639373
C5_02730C_A	-1.03881	0.873896	3637738
C1_12900W_A	-1.03888	0.898472	3636154
BMT6	-1.0398	0.871408	3639258
C2_10230W_A	-1.04004	0.925255	3643925
MET28	-1.04096	0.841279	3637962
C2_02490C_A	-1.041	0.857831	3635815
CR_06640C_A	-1.04101	0.899683	3646619
C3_01260C_A	-1.04116	0.890153	3636625
TEL1	-1.04151	0.880637	3639263
GDI1	-1.04191	0.838885	3643298
NRP1	-1.04213	0.917598	3640698
C2_08990C_A	-1.04399	0.883278	3645783
C1_03330C_A	-1.04439	0.877418	3636859
C2_04380C_A	-1.04568	0.868731	3645459
FLC3	-1.04603	0.861736	3642322
C2_10160W_A	-1.04626	0.944881	3643932
C1_03260W_A	-1.04654	0.893255	3636898
C2_04870C_A	-1.04752	0.803484	3648111
LEM3	-1.04837	0.821833	3643456
CR_07510W_A	-1.04944	0.903366	3647998
C7_01510W_A	-1.04971	0.822813	3637049
SEC15	-1.0499	0.841272	3646845
PIM1	-1.04997	0.813758	3640999
C3_05380W_A	-1.05093	0.87827	3643679
C2_10760C_A	-1.05181	0.833223	3641238
C3_00310C_A	-1.05379	0.89921	3640523
HYR3	-1.05419	0.789648	3644119
C7_03860W_A	-1.05432	0.795833	3643144
MNN12	-1.05581	0.848675	3634904
RPC19	-1.05621	0.894658	3647693
C1_04990C_A	-1.05635	0.854725	3647530
GPM2	-1.05639	0.813995	3644713
HAL21	-1.05645	0.817446	3644045
C5_00250C_A	-1.05663	0.90059	3641562
YMC2	-1.05664	0.914501	3634786
C4_06450W_A	-1.05689	0.906454	3642792
C6_01520W_A	-1.05728	0.919927	3641617
NOP5	-1.0578	0.786559	3646084
VPS17	-1.05787	0.814009	3640218
HAP3	-1.0582	0.929328	3635629

DCK1	-1.05827	0.791865	3637383
C4_02660W_A	-1.05924	0.94097	3637831
C4_06650W_A	-1.05938	0.782802	3642810
C7_01960W_A	-1.06096	0.82252	3636969
GLN1	-1.06108	0.755433	3646386
GAL7	-1.06139	0.782009	3644591
IFF4	-1.06181	0.843847	3639694
PGA11	-1.06194	0.951925	3638939
C4_06360C_A	-1.06212	0.953165	3642825
PES1	-1.0626	0.761837	3644105
CR_08550W_A	-1.0636	0.756148	3642141
ISA1	-1.06365	0.758356	3639174
CRH12	-1.06429	0.774344	3636447
C5_03690W_A	-1.06447	0.909197	3646989
ZCF15	-1.06454	0.791289	3637867
C1_00890W_A	-1.06621	0.778136	3639354
C3_06150W_A	-1.06706	0.787561	3641765
RBR3	-1.07012	0.772464	3646728
C7_01270C_A	-1.07152	0.862386	3645908
TRM9	-1.07239	0.819296	3642074
MED9	-1.07261	0.812474	3643134
MED4	-1.07316	0.800429	3645209
SUI2	-1.07337	0.736445	3639569
CCH1	-1.07374	0.756765	3640175
C1_05420W_A	-1.074	0.813609	3642493
ROD1	-1.07447	0.724468	3635763
C2_02040W_A	-1.07465	0.747209	3635848
FGR43	-1.07468	0.87027	3634828
ARG83	-1.07494	0.822147	3637862
C1_10340W_A	-1.07508	0.818932	3634909
CBF1	-1.0757	0.751384	3642779
CR_05100W_A	-1.07631	0.816518	3646403
VPS34	-1.07655	0.782164	3639540
SIT1	-1.07658	0.728467	3642307
C3_07350W_A	-1.07743	0.74352	3638476
PGA27	-1.07752	0.874879	3638811
CTA26	-1.08008	0.775671	3638965
CR_05140W_A	-1.08112	0.874298	3646400
C2_07870C_A	-1.0831	0.966412	3642319
C5_03770C_A	-1.0831	0.966393	3647233
C6_02550W_A	-1.0831	0.966393	3639204
C6_04070C_A	-1.0831	0.966393	3647826
C7_02360W_A	-1.0831	0.966535	3636931
CR_09350C_A	-1.0831	0.966606	3641901
C1_13180W_A	-1.08319	0.966359	3636162
C3_01970C_A	-1.08319	0.966359	3636597
C3_02200W_A	-1.08319	0.966359	30515185

C5_03730W_A	-1.08319	0.966359	3647238
C6_02760W_A	-1.08319	0.966359	3639218
RBR1	-1.08319	0.966359	3640986
SCW4	-1.08319	0.966359	3636797
C1_06220C_A	-1.08328	0.966324	3647518
CR_07300W_A	-1.08328	0.966324	3646059
CR_07740W_A	-1.08328	0.966324	30515430
CR_08740W_A	-1.08328	0.966324	3642123
GIT4	-1.08328	0.966324	3642635
PGA28	-1.08328	0.966324	3641350
PTR2	-1.08328	0.966324	3640089
CR_01550C_A	-1.08404	0.82956	3640074
C1_02390W_A	-1.08407	0.784857	3636791
CR_00010C_A	-1.0842	0.735292	3639712
C2_09480W_A	-1.08443	0.920086	3643819
GCD1	-1.08471	0.694635	3640965
SNM1	-1.08662	0.822022	3642609
PPS1	-1.08799	0.827235	3646526
C2_04620W_A	-1.08801	0.707365	3645438
C1_07360W_A	-1.08885	0.730103	3644470
C2_08770C_A	-1.09019	0.836975	3643878
C1_06650W_A	-1.09087	0.713532	3639543
C3_00400C_A	-1.09266	0.747597	3640554
C1_00530C_A	-1.09269	0.707527	3639368
MEA1	-1.09336	0.873806	3635154
C4_06670W_A	-1.09368	0.875535	3642951
C3_04380C_A	-1.09393	0.700765	3635173
CR_01570W_A	-1.09538	0.839446	3640077
KAP120	-1.09572	0.621244	3643110
CR_08590W_A	-1.09705	0.689641	3642106
RPO26	-1.09825	0.707748	3646351
PDC2	-1.09883	0.754767	3634862
FGR32	-1.09915	0.619195	3644136
C6_03540W_A	-1.09924	0.717726	3640244
C1_00160C_A	-1.10142	0.650301	3639341
C5_00400C_A	-1.10144	0.687263	3641550
NOT3	-1.10201	0.625337	3638829
MET18	-1.10231	0.804045	3636631
CR_09700W_A	-1.10251	0.784661	3647088
HOS2	-1.10353	0.763226	3640509
C2_06230W_A	-1.10357	0.608681	3646911
SMI1	-1.1062	0.649255	3640741
C7_00130W_A	-1.10625	0.70431	3637975
RAD9	-1.10694	0.736335	3637780
HAP31	-1.10762	0.810271	3640994
C2_07520C_A	-1.10778	0.703212	3645207
C1_12350W_A	-1.1078	0.656437	3642063

CR_04280C_A	-1.108	0.698027	3640993
C1_07280C_A	-1.11078	0.858486	30515021
C3_00950C_A	-1.11114	0.724581	3639852
NPR1	-1.11176	0.601735	3639498
PGA1	-1.11282	0.778435	3638980
ABZ1	-1.11457	0.592794	3648307
STR2	-1.11547	0.57788	3644689
C1_14430C_A	-1.11547	0.689209	3643297
C4_03700W_A	-1.11558	0.706545	3645361
C1_09310C_A	-1.11635	0.72977	3634833
TGL99	-1.11728	0.68293	3636168
MNN22	-1.11769	0.737497	3641117
C1_10350C_A	-1.11809	0.632523	3634835
ERG3	-1.11926	0.665164	3644776
MDS3	-1.1199	0.575718	3638464
BUL1	-1.12083	0.787971	3640734
CR_09110C_A	-1.12084	0.711491	3641929
C1_11370C_A	-1.12163	0.704508	3644547
CR_07140C_A	-1.12212	0.63974	3648227
C3_00270C_A	-1.12218	0.617523	3640527
MED21	-1.12264	0.8021	3639010
FCY23	-1.12354	0.648924	3647681
CR_03960C_A	-1.1236	0.72602	3640963
ALK2	-1.12364	0.728217	3639700
ORC1	-1.12664	0.57433	3636922
C1_03360W_A	-1.12696	0.546964	3636889
C5_01490C_A	-1.12707	0.538826	3647058
CR_06530W_A	-1.12714	0.683321	3645262
C2_05540C_A	-1.12738	0.684455	3645559
CGR1	-1.12822	0.642371	3637853
PFK1	-1.12848	0.569123	3636448
C5_02830W_A	-1.12858	0.588383	3637809
C2_03830W_A	-1.12862	0.612159	3637399
C2_01630W_A	-1.12884	0.875662	3635738
CR_08280W_A	-1.12906	0.721726	3642102
C3_04690C_A	-1.12915	0.73313	3635196
C3_03070W_A	-1.12938	0.659567	3638396
SEC6	-1.1296	0.531443	3640565
RFX2	-1.13274	0.827692	3635658
C2_09690C_A	-1.13279	0.63936	3643789
C6_02430W_A	-1.13284	0.597111	3639163
CR_10610C_A	-1.13402	0.804954	3639005
CR_06470W_A	-1.13428	0.575394	3645277
CR_00110W_A	-1.1348	0.517763	3639731
PEX13	-1.13485	0.546807	3641877
C6_03380W_A	-1.13666	0.529296	3639231
ENA2	-1.13668	0.502387	3639291

GPR1	-1.13721	0.618511	3642616
C5_03850W_A	-1.13736	0.639501	3647225
TPS1	-1.13857	0.580787	3646699
SCT1	-1.13944	0.530982	3648346
BUR2	-1.14016	0.668249	3637772
C2_03930C_A	-1.14084	0.600314	3637423
ZCF31	-1.14156	0.572459	3635191
C2_03780C_A	-1.14227	0.577072	3637368
PLB2	-1.1423	0.807756	3644562
C5_04610W_A	-1.14312	0.56883	3636393
CR_10630W_A	-1.14458	0.748204	3639007
CR_03410W_A	-1.14557	0.594869	3645136
C5_05000C_A	-1.14886	0.530329	3636539
PIR1	-1.1492	0.545645	3645801
FLC1	-1.1494	0.517167	3639854
TTR1	-1.14957	0.50731	3639313
C2_08060W_A	-1.14969	0.784134	30515151
MKC1	-1.14999	0.496424	3639710
CR_06010W_A	-1.15002	0.65198	3644870
SKN7	-1.15197	0.587037	3641563
BNI1	-1.15206	0.467326	3636118
ZDS1	-1.15215	0.491595	3641103
C1_14080W_A	-1.15262	0.424923	3643345
C3_07380W_A	-1.15449	0.541362	3638473
C1_12530C_A	-1.15508	0.683394	3647608
C2_01230W_A	-1.15546	0.744004	3638795
DAD2	-1.15592	0.806408	3643465
C4_03680C_A	-1.15827	0.710443	3645363
DFG5	-1.16026	0.448112	3638867
C1_04930C_A	-1.16056	0.787081	3644792
C3_02760C_A	-1.1606	0.534681	3638413
STE50	-1.16106	0.582884	3636660
C1_10470W_A	-1.16135	0.5122	3647184
CR_06090W_A	-1.16184	0.515499	6335359
C6_03300C_A	-1.16249	0.518673	3639196
C4_01820C_A	-1.16269	0.516193	3635594
C5_02330W_A	-1.1631	0.71775	3637789
C3_03540W_A	-1.16327	0.516196	3638313
C1_07570C_A	-1.16343	0.715484	3646675
C7_01230C_A	-1.16352	0.513654	3645906
C5_00140C_A	-1.16443	0.603221	3647873
GSL1	-1.16466	0.600341	3639844
CAS5	-1.16473	0.50178	3635652
NAP1	-1.16485	0.444683	3639714
C7_00810W_A	-1.16525	0.492684	3637940
ESS1	-1.16553	0.512524	3647989
FCR3	-1.16576	0.534676	3643760

CR_08710W_A	-1.16752	0.443212	3642131
HGH1	-1.16758	0.529183	3635647
HOL1	-1.16821	0.735246	3636838
INO2	-1.16941	0.696599	3639674
RPL7	-1.16947	0.523969	6335362
SPT23	-1.16957	0.484188	3643958
ULP2	-1.17002	0.549262	3643196
CR_03730C_A	-1.17054	0.533513	3643226
LTE1	-1.17069	0.585086	3643495
C1_14450C_A	-1.17086	0.411734	3643299
FGR10	-1.17115	0.736265	3641159
ZCF8	-1.17121	0.562178	3636651
C2_01750C_A	-1.1717	0.586825	3635734
YOR1	-1.17208	0.483624	3643964
HAL22	-1.17244	0.615915	3644051
C5_02370C_A	-1.17273	0.543041	3637793
C3_05020W_A	-1.17297	0.64615	3635170
C1_03460C_A	-1.1731	0.717721	3636911
C4_06130W_A	-1.17429	0.374956	3648085
CR_00220W_A	-1.17511	0.581366	3639699
EAP1	-1.17555	0.493057	3643814
C3_06040W_A	-1.17657	0.740931	3641745
BPH1	-1.17714	0.44418	3639547
C4_06090C_A	-1.17791	0.55117	3646532
MLS1	-1.17792	0.400479	3634875
C4_02340W_A	-1.17794	0.515718	3637883
C1_04630C_A	-1.17894	0.478507	3644762
C5_00320W_A	-1.17917	0.63074	30515291
C3_05430W_A	-1.17948	0.581583	3643667
C1_05280W_A	-1.18001	0.559912	3642475
C2_00270C_A	-1.18012	0.543747	3638803
HST1	-1.18029	0.54948	3634770
C1_03910C_A	-1.18124	0.688099	3646412
C1_01840C_A	-1.18143	0.437501	3645432
C6_01450C_A	-1.18177	0.501088	3641624
C5_03170C_A	-1.1828	0.547381	3637801
RPT6	-1.18438	0.35078	3643877
GPI2	-1.18466	0.743927	3641011
C4_00840W_A	-1.18482	0.475204	3635722
CRZ1	-1.18509	0.471219	3641722
C2_10820C_A	-1.18589	0.458356	3641230
C6_00390W_A	-1.19047	0.458431	3646086
IPK2	-1.19078	0.489636	3643784
TOM1	-1.19096	0.397396	3642987
CDG1	-1.19152	0.383665	3641883
C3_04190W_A	-1.19169	0.861488	30515203
C5_00600C_A	-1.19171	0.859654	3641586

ALK8	-1.19181	0.859669	3648226
C6_01000C_A	-1.19266	0.578029	3644048
ALG1	-1.19305	0.588216	3646517
C1_00430W_A	-1.19331	0.472704	3639285
C7_03620C_A	-1.19456	0.419195	3646242
CR_06510W_A	-1.19557	0.792252	3645264
C3_06290W_A	-1.19581	0.394071	3642237
JAB1	-1.196	0.46537	3646811
CR_07200W_A	-1.19629	0.594599	3646044
C7_01590C_A	-1.19642	0.624356	3637037
C6_04440C_A	-1.19713	0.507868	3645983
C7_04150W_A	-1.19849	0.415334	3643128
CR_03180W_A	-1.19888	0.39456	3645133
C3_04370C_A	-1.19923	0.434003	3635172
CCR4	-1.19964	0.394446	3640749
NGS1	-1.19968	0.43198	3639703
RAT1	-1.19972	0.363104	3635686
C6_00490W_A	-1.19991	0.597917	3647150
CR_03220C_A	-1.20059	0.682194	3645128
CR_01130W_A	-1.20165	0.452218	3640433
C2_06030W_A	-1.20177	0.411593	3644070
C1_11150W_A	-1.20221	0.48995	3648356
C2_07030C_A	-1.20389	0.547428	3643498
C4_04930C_A	-1.20423	0.662106	3641170
C5_02660C_A	-1.20474	0.50168	3637771
SPT10	-1.20511	0.486288	3645729
RPB4	-1.20512	0.506519	3647800
C2_02910W_A	-1.20832	0.806783	30515097
BRO1	-1.20855	0.357482	3636590
HCM1	-1.20872	0.809678	3634794
SNU114	-1.20889	0.440786	3647798
C2_07360W_A	-1.20947	0.603291	3645236
CR_02210W_A	-1.21113	0.461582	3644943
CR_07020W_A	-1.21158	0.405489	3645731
OFD1	-1.21239	0.369979	3646962
NHP2	-1.2146	0.336044	3641003
CR_10740W_A	-1.2146	0.452654	3638941
RTA3	-1.21486	0.439753	3646566
C3_05320W_A	-1.21549	0.397723	3643648
GCN3	-1.21639	0.385228	3645905
C3_01670W_A	-1.21691	0.386429	3636606
SNP3	-1.21723	0.656006	3640991
GLN4	-1.21731	0.379026	3637998
RHD3	-1.21762	0.335494	3641212
C5_00390C_A	-1.21792	0.331903	3641552
PEX12	-1.21879	0.383669	3638862
C3_02360C_A	-1.21914	0.726481	3636617

CR_09140C_A	-1.21915	0.46331	3641879
HRK1	-1.2196	0.282178	3640586
C1_11300C_A	-1.22018	0.410542	3644552
C2_05490W_A	-1.22145	0.702457	3643417
C1_07160C_A	-1.223	0.28055	30515020
C4_03090W_A	-1.22313	0.374543	3637926
HSM3	-1.2233	0.359064	3646713
CR_01360W_A	-1.22368	0.670889	3640167
MSF1	-1.22552	0.411215	3638806
C2_07510W_A	-1.2272	0.57306	3645208
C7_02090C_A	-1.2272	0.574257	3637024
C3_02720W_A	-1.22735	0.536568	3638410
TRS33	-1.22744	0.564096	3636992
KIP4	-1.2275	0.537314	3642032
CR_04180C_A	-1.22807	0.300751	3640937
HST3	-1.22897	0.446086	3642641
IRA2	-1.23509	0.292197	3642011
MRP7	-1.23562	0.412482	3643118
GPX2	-1.23577	0.409646	3644009
C1_06530C_A	-1.23618	0.289639	3639521
PRO1	-1.23643	0.25063	3639000
ALO1	-1.23653	0.279358	3638983
GCN1	-1.2366	0.29379	3641256
MNT2	-1.23789	0.453151	3636583
CLA4	-1.2385	0.461546	3634878
C4_04760C_A	-1.23857	0.458448	3641118
XUT1	-1.23881	0.560815	3642785
YML6	-1.2398	0.417526	3637954
OLE1	-1.24004	0.316419	3640755
SIS1	-1.24016	0.298745	3644841
C1_01470W_A	-1.24018	0.272403	3640241
POL5	-1.2407	0.388828	3639254
SOL1	-1.24102	0.459853	3645627
C7_00450C_A	-1.24164	0.5109	3637991
EHT1	-1.24495	0.342905	3636894
CR_06270W_A	-1.24682	0.797092	3644854
C1_01460W_A	-1.24686	0.315402	3640240
ADH5	-1.24734	0.310485	3640156
C1_01820C_A	-1.2479	0.391189	3645435
C4_04120C_A	-1.24796	0.389249	3641222
IPK1	-1.24821	0.525276	3648091
ATO2	-1.24941	0.325908	3639845
SRB8	-1.25197	0.34138	3641128
CR_10390W_A	-1.252	0.637267	3638954
RAC1	-1.25237	0.378588	3639508
C1_03400W_A	-1.25487	0.261803	3636904
C1_00880W_A	-1.25511	0.494549	3639355

C6_02650C_A	-1.25551	0.357998	3639223
RPR2	-1.25609	0.645964	3641017
C4_06410W_A	-1.25661	0.49382	3642796
C5_05210W_A	-1.25794	0.319909	3636468
TRM2	-1.25801	0.419074	3640235
TIF3	-1.25802	0.257146	3641646
KIS1	-1.25942	0.216841	3644063
C6_02310W_A	-1.25955	0.411629	3645875
CR_07660C_A	-1.25997	0.284789	3647256
C2_02440W_A	-1.26003	0.333175	3635758
ARG3	-1.26075	0.65797	3639180
CR_07480W_A	-1.26233	0.262358	3646047
C6_03590C_A	-1.26266	0.455899	3640251
C7_02860C_A	-1.26275	0.254633	3641397
C7_00840C_A	-1.26276	0.522432	3638057
C4_00470C_A	-1.26283	0.348822	3645011
C5_04870W_A	-1.2636	0.434915	3636438
C3_07600W_A	-1.26361	0.433142	3638495
MEC3	-1.26376	0.541786	3646910
CR_10170C_A	-1.26478	0.376138	3638916
TSM1	-1.26577	0.219851	3645953
C5_03810C_A	-1.2674	0.677131	3647229
HGT16	-1.2675	0.679769	3646026
MED19	-1.26869	0.3992	30515334
C1_01090C_A	-1.26981	0.42688	3640174
C5_04890C_A	-1.27077	0.565228	3636415
PET100	-1.27215	0.440641	30515038
C1_08240C_A	-1.27226	0.409471	3640759
C3_00510W_A	-1.27557	0.493556	3640514
NUP60	-1.27801	0.278957	3642826
ECM18	-1.27867	0.707243	3645655
C2_07790C_A	-1.27875	0.704255	3642326
NTG1	-1.27987	0.441775	3640746
C2_00230W_A	-1.28056	0.316255	3638901
HOL4	-1.28081	0.211934	3645409
IAH1	-1.28152	0.25522	3638945
C4_00740W_A	-1.28221	0.603872	3645764
C3_00390W_A	-1.28231	0.607252	3640539
C5_04350C_A	-1.28347	0.348774	3636482
C3_06880W_A	-1.28358	0.758777	3638539
SMX4	-1.28457	0.471911	30515427
C2_05820W_A	-1.28511	0.23584	3647482
C6_01920C_A	-1.28634	0.278036	3644534
C2_01030W_A	-1.28672	0.346494	3638836
C2_08960C_A	-1.28681	0.404527	3645787
PUT3	-1.28769	0.254498	3639535
C4_03080W_A	-1.28987	0.293281	3637928

C4_02700W_A	-1.2899	0.334538	3637859
C6_03730C_A	-1.29097	0.390859	3640303
ERG25	-1.29114	0.347405	3644933
C2_07860W_A	-1.29116	0.342597	3642320
C1_10200C_A	-1.29187	0.328912	3634877
C5_00260W_A	-1.29434	0.238508	3641561
CYR1	-1.29465	0.199396	3641407
SWI1	-1.29506	0.183337	3644996
MED8	-1.29521	0.548214	3645446
YTA6	-1.29551	0.218147	3638990
HCR1	-1.29612	0.19597	3638952
CR_04170W_A	-1.29648	0.235971	3640977
C1_01070C_A	-1.29749	0.449238	3640172
PHO91	-1.29839	0.17446	3644588
C1_10840C_A	-1.29841	0.195476	3644452
C1_07860W_A	-1.30019	0.737402	3640740
C5_02360C_A	-1.3003	0.740843	3637792
C7_00780W_A	-1.30265	0.314823	3637945
C2_06850W_A	-1.30334	0.566303	30515142
C1_05380C_A	-1.30452	0.317438	3642480
C1_08920W_A	-1.30521	0.387838	3634910
LYS144	-1.30602	0.299037	3640512
C2_05910W_A	-1.30978	0.283256	3647476
C3_06260C_A	-1.31017	0.336051	3642240
C4_05260W_A	-1.31253	0.315596	3648282
C4_05650W_A	-1.31257	0.323603	3641814
C6_01380C_A	-1.31289	0.432601	3641606
C3_03110W_A	-1.31357	0.405609	3638400
C3_05970C_A	-1.31416	0.147607	3641730
BDF1	-1.31469	0.1492	3641592
SKN2	-1.31497	0.32461	3638309
C1_01680C_A	-1.3154	0.336893	3640198
C5_01290C_A	-1.3155	0.135344	3642647
GLY1	-1.31754	0.194105	3647181
CR_03230W_A	-1.31784	0.278042	3645127
C2_07720C_A	-1.31824	0.292052	3642304
C2_03880C_A	-1.31858	0.617139	30515108
CR_10770W_A	-1.31859	0.250165	3638944
C4_07200C_A	-1.3188	0.284953	3642958
C3_02260C_A	-1.32202	0.169451	3636636
GDH2	-1.32216	0.206029	3642295
C3_06440W_A	-1.32278	0.148416	3642281
SWR1	-1.32396	0.134007	3645220
C1_04600C_A	-1.32492	0.216122	3646133
C3_06140W_A	-1.3267	0.432507	3641764
C5_03010W_A	-1.32697	0.312135	3637819
C5_00170W_A	-1.3279	0.243993	3647870

CR_03310C_A	-1.32959	0.15615	3645097
C1_05400C_A	-1.33103	0.521321	3642478
SRP54	-1.33257	0.165147	3640377
C3_05060W_A	-1.33264	0.418434	3635241
C1_14550C_A	-1.33287	0.486838	3643334
RGT1	-1.33296	0.196216	3637920
SPT7	-1.33343	0.184943	3638928
AFG1	-1.33358	0.316341	3634920
RPB7	-1.33382	0.328088	3640221
C1_09280W_A	-1.33536	0.319411	3634829
SPP1	-1.33541	0.416516	3636897
GPI8	-1.33577	0.265883	3646669
CR_01720W_A	-1.33605	0.260774	3640114
BBC1	-1.337	0.148067	3646661
CAS4	-1.337	0.115801	3636568
C1_06410W_A	-1.33706	0.364245	3639555
CR_04140W_A	-1.33816	0.232604	3640974
C3_03230C_A	-1.339	0.275216	3638316
C6_02900C_A	-1.33921	0.403653	3639240
C4_06210C_A	-1.33987	0.155551	3642815
MRR1	-1.34084	0.296482	3641741
CHS4	-1.34251	0.13872	3641726
C4_00950C_A	-1.34315	0.198831	3635675
DUR7	-1.34383	0.339454	3643335
C4_00070C_A	-1.34473	0.112499	3648063
C3_02990C_A	-1.34478	0.404017	3638388
C7_03240W_A	-1.34644	0.10942	3641368
RIM15	-1.34843	0.117581	3637961
C2_10650W_A	-1.34871	0.643771	30515168
CR_01770C_A	-1.34934	0.12518	3640122
YDJ1	-1.34969	0.182892	3640939
FKH2	-1.3517	0.181458	3640536
PRS1	-1.35182	0.199977	3635817
C2_08020C_A	-1.35243	0.187613	3642308
AFL1	-1.35285	0.145104	3641594
C6_03430C_A	-1.35373	0.185473	3640273
YTM1	-1.35496	0.174757	3634765
C2_06080C_A	-1.35601	0.216277	3644098
C1_04850C_A	-1.35615	0.680744	30514989
MRPS9	-1.35644	0.207464	3642064
C1_01540W_A	-1.35663	0.190266	3640233
C2_07700C_A	-1.35771	0.760755	3642337
C6_03070C_A	-1.35771	0.761108	3639272
C2_08750W_A	-1.35783	0.759016	3643881
C4_01800W_A	-1.35786	0.761176	3635595
CR_06920W_A	-1.35786	0.761184	3647314
DUR35	-1.35786	0.761307	3635150

C1_06000W_A	-1.35794	0.761591	3639870
C2_05040C_A	-1.35865	0.1511	3643449
GLE1	-1.35907	0.180085	3642340
CR_05030W_A	-1.36062	0.142086	3646388
C1_12870C_A	-1.36253	0.58744	3636180
C1_14410W_A	-1.36289	0.27335	3643293
C7_03580C_A	-1.36364	0.288646	3646238
PAM17	-1.36372	0.176408	3638369
HCH1	-1.36402	0.261369	3641626
C6_04480C_A	-1.36511	0.167557	3645986
C6_03390W_A	-1.36549	0.356341	3639232
C4_00660W_A	-1.36661	0.23888	3645755
C1_11200W_A	-1.36745	0.278854	3644396
HST6	-1.36967	0.762719	3642288
LIP9	-1.36967	0.762453	3641395
PHO100	-1.36967	0.762611	3644483
C2_02720W_A	-1.36982	0.162205	3643002
CSI2	-1.37028	0.301334	3642016
C1_00100C_A	-1.3707	0.284159	3639325
C4_03710C_A	-1.37119	0.365531	3645360
C2_05550W_A	-1.37156	0.260587	3645560
MRS4	-1.37228	0.2468	3642306
C1_07390W_A	-1.37358	0.094635	3644466
C5_03870C_A	-1.37365	0.171417	3647223
C3_00420W_A	-1.3739	0.180744	3640553
C5_04330W_A	-1.37397	0.356419	30515314
C2_06650C_A	-1.37553	0.394477	3648419
C5_03470C_A	-1.37584	0.228846	3646963
VPS70	-1.37643	0.109845	3637971
FMO1	-1.37693	0.581736	3635852
HGT8	-1.37797	0.127122	3638875
INO1	-1.37898	0.096495	3638966
PRC2	-1.37932	0.252227	3647044
C4_03750C_A	-1.38004	0.371448	3645356
CR_00230W_A	-1.38051	0.08612	3639698
C2_09870W_A	-1.3809	0.205096	3643780
PGA56	-1.38165	0.121998	30515313
CTA9	-1.38218	0.159657	3640215
C5_05070W_A	-1.38232	0.372518	3636491
CR_06650C_A	-1.38271	0.288993	3646620
C3_01520C_A	-1.38351	0.159152	3636570
C1_03270W_A	-1.3836	0.110577	3636900
GAL1	-1.3858	0.10353	3644581
C1_05650W_A	-1.38752	0.219996	3639838
C2_03950W_A	-1.38761	0.250992	3637420
C7_04140C_A	-1.38771	0.133785	3643129
DIP5	-1.38794	0.085611	3636798

C1_06980C_A	-1.3899	0.196967	3639565
C5_02620C_A	-1.38996	0.193856	3637783
PGA7	-1.39001	0.114	3645006
CR_03740C_A	-1.39045	0.198686	3643176
NCE102	-1.39085	0.14291	3635267
CR_03780C_A	-1.39099	0.454557	3643179
HNM3	-1.39201	0.302673	3640093
C1_09950C_A	-1.39344	0.187821	3634861
C1_13370W_A	-1.39405	0.322718	3636216
SEH1	-1.39467	0.160382	3642290
BUD6	-1.39524	0.179639	3640727
C5_03940C_A	-1.40022	0.241116	3647446
RPP1	-1.40261	0.403814	3644681
C4_06370C_A	-1.40296	0.306382	3642824
HAC1	-1.403	0.101378	3639849
C5_01530C_A	-1.40327	0.206342	3647062
RAD57	-1.40358	0.240777	3642333
RIM9	-1.40364	0.230711	3644047
C5_02190C_A	-1.40564	0.164606	30515303
C4_04360W_A	-1.40694	0.666253	3646851
C3_07240W_A	-1.40709	0.665688	3638530
CPR6	-1.40755	0.062563	3639020
C2_08510W_A	-1.40843	0.449671	3643922
C1_13240W_A	-1.40857	0.131407	3636167
CR_06570C_A	-1.41043	0.188354	3648366
VPH2	-1.41071	0.272243	3642021
AGC1	-1.41147	0.238993	3636219
C2_03460C_A	-1.41253	0.121472	3637348
SNX4	-1.41266	0.106213	3638843
CR_00370W_A	-1.41291	0.125559	3639684
RPC53	-1.41377	0.239007	3637898
C4_06230C_A	-1.41671	0.150805	3642808
XYL2	-1.41699	0.105544	3638961
SPT6	-1.41868	0.091086	3643171
C1_06800W_A	-1.41897	0.16133	3639496
C3_00360W_A	-1.41937	0.258526	3640542
CR_01790C_A	-1.4194	0.220464	3640161
APC1	-1.41987	0.131263	3639383
CSC25	-1.42041	0.115536	3645660
HGT14	-1.42101	0.275614	3648320
C1_01390C_A	-1.42139	0.19156	3640236
C3_03920W_A	-1.42165	0.278126	30515197
SLK19	-1.42186	0.102105	3638466
CR_10230W_A	-1.4223	0.070793	3638921
IMH3	-1.42388	0.090893	3646556
C5_01930W_A	-1.42597	0.161653	3643741
C4_02580W_A	-1.42609	0.717458	3637915

MDN1	-1.42622	0.052449	3635614
CBK1	-1.42645	0.063532	3634838
WAL1	-1.42665	0.060221	3647093
TPO5	-1.42669	0.307537	3647791
VPS24	-1.42751	0.115955	3638892
C1_08900W_A	-1.42756	0.469355	3634925
RPA135	-1.42889	0.08732	3637996
ZNC1	-1.42901	0.086326	3643754
C7_00030W_A	-1.42922	0.103659	3638004
CHK1	-1.4293	0.141075	3637394
CR_10260W_A	-1.42968	0.433433	3638932
GCD6	-1.43058	0.062568	3646510
C7_00830C_A	-1.43174	0.06533	3637938
C1_08770W_A	-1.43197	0.242002	3634788
BFA1	-1.4326	0.174433	3639301
HYR4	-1.43347	0.161798	3640436
C1_11270W_A	-1.43383	0.074312	3644555
CRL1	-1.43644	0.07863	3640238
YPT7	-1.437	0.366438	3636864
SFU1	-1.43731	0.173373	3634867
DIT2	-1.43887	0.522341	3640962
ZCF38	-1.43893	0.126005	3639705
SWD1	-1.43893	0.171192	3640535
ILS1	-1.44041	0.078601	3645956
C3_03330C_A	-1.444	0.103186	3638364
OPI1	-1.44412	0.14361	3635777
C1_06030C_A	-1.44552	0.485263	3639867
MPH1	-1.44651	0.179542	3642817
C3_07660W_A	-1.44666	0.147332	3638462
YFH1	-1.44711	0.193351	3646853
BEM1	-1.44896	0.134943	3635627
C5_00030W_A	-1.4497	0.086289	3647879
C2_07640W_A	-1.45105	0.064714	3645192
C2_08100W_A	-1.45181	0.132049	3642334
C2_07370W_A	-1.45221	0.161508	3645233
C4_03140C_A	-1.45247	0.240987	3637843
MSS11	-1.45376	0.205173	3647296
C2_01190C_A	-1.45443	0.230957	3638859
C5_03040W_A	-1.45472	0.238192	3637824
CR_00160C_A	-1.45507	0.109973	3639706
C7_04200C_A	-1.45731	0.08548	3643122
C3_05940C_A	-1.45751	0.408986	3641744
SKP1	-1.46519	0.045848	3644485
HGC1	-1.46543	0.33773	3639349
C1_11680C_A	-1.46586	0.261092	30515056
CR_09530C_A	-1.46753	0.093894	3647101
C1_10330C_A	-1.46803	0.214437	3634908

HTS1	-1.46809	0.054948	3636532
C1_06660W_A	-1.46839	0.100846	3639542
C3_06050C_A	-1.47018	0.162972	3641746
SCO1	-1.4704	0.079016	3641922
CR_04160C_A	-1.47089	0.074891	3640976
CR_02590C_A	-1.47211	0.161607	3642595
PUT4	-1.47335	0.427846	3636801
ECM17	-1.47367	0.031974	3644077
C3_03470W_A	-1.474	0.455897	3638306
CR_05440W_A	-1.47464	0.08131	3648245
C2_04400W_A	-1.47844	0.070177	3645457
C4_03930C_A	-1.47919	0.265045	30515262
C1_12710C_A	-1.47987	0.13235	3646822
C3_07170C_A	-1.48056	0.092568	3638536
PUS7	-1.48096	0.070741	3643960
UTP20	-1.48142	0.054061	3647956
C7_01280C_A	-1.48204	0.137172	3645909
HMI1	-1.4826	0.140551	3638997
C3_03770C_A	-1.48416	0.075178	3645636
ECM42	-1.48611	0.157626	3636995
APN2	-1.48708	0.280789	3647674
SPA2	-1.48769	0.033017	3646824
PGA18	-1.48788	0.104222	3638394
CR_07760W_A	-1.48954	0.426373	30515431
C3_04230W_A	-1.48965	0.129345	3635187
MRPL3	-1.49071	0.116189	3640722
PDE1	-1.4918	0.163538	3637713
C6_00690W_A	-1.49391	0.396613	3647075
C5_01310W_A	-1.49439	0.386664	3642645
CDH1	-1.49547	0.124318	3638814
PWP1	-1.49573	0.034497	3635623
CR_04150W_A	-1.49614	0.434373	3640975
C3_07100C_A	-1.4962	0.071503	3638443
GSC1	-1.49658	0.036776	3636794
C2_05080C_A	-1.4967	0.356941	3643453
CDR2	-1.49689	0.110036	3635265
C1_07080W_A	-1.49752	0.190322	3639489
FGR6-4	-1.49955	0.26229	30515164
C2_06160W_A	-1.50092	0.428828	3644079
C1_02950W_A	-1.50111	0.445583	3636833
C4_06960W_A	-1.50126	0.428721	3642926
PSD1	-1.50126	0.027659	3639382
C5_05360C_A	-1.50323	0.039724	3636402
C6_00360C_A	-1.50442	0.134364	3646083
MNT3	-1.50456	0.229464	3648123
CR_02270C_A	-1.50513	0.218424	3644911
C5_02310C_A	-1.50748	0.262884	3637787

DUG3	-1.51033	0.0566	3641381
CR_02960W_A	-1.51246	0.270016	3642591
PRA1	-1.51254	0.352269	3642969
HAP5	-1.51314	0.113125	3642654
C3_07590W_A	-1.51477	0.305088	3638496
PBP2	-1.5165	0.038035	3640267
VPS13	-1.51803	0.029195	3646528
CR_04310C_A	-1.51941	0.126868	3640996
C2_08740W_A	-1.51945	0.20913	3643882
ZCF23	-1.5218	0.095678	3644477
C2_06780C_A	-1.52283	0.153259	3643516
C4_06840W_A	-1.5231	0.281246	3642913
DUO1	-1.52395	0.333213	3646858
C2_00940W_A	-1.52738	0.04263	3638891
MAS2	-1.52795	0.136909	3647249
DJP1	-1.52839	0.0658	3647186
C3_03310C_A	-1.52986	0.247994	3638363
TAF145	-1.53158	0.052108	3639486
C2_03410W_A	-1.53278	0.023194	3637412
C7_00460W_A	-1.53292	0.606044	3637990
C6_04410C_A	-1.53306	0.161099	3645950
C1_11110C_A	-1.53353	0.244248	3644413
TIF4631	-1.53567	0.031609	3643880
C3_06270C_A	-1.53585	0.079996	3642239
C5_00760W_A	-1.53633	0.080156	3644122
C5_03640W_A	-1.53704	0.081979	3646984
C3_03200C_A	-1.53749	0.359611	3638428
STI1	-1.5398	0.016657	3643758
GCD2	-1.5399	0.050965	3638437
C5_03300C_A	-1.54073	0.097406	3646357
SNF7	-1.54218	0.057546	3639377
C7_04290W_A	-1.54221	0.16129	3643172
SOD6	-1.5425	0.085162	3638899
CEM1	-1.54268	0.199825	3635242
C2_00600C_A	-1.54459	0.022916	3638850
CR_09580C_A	-1.54549	0.090012	3647097
YCF1	-1.54669	0.030023	3636934
USO6	-1.54844	0.050118	3643647
C1_06770W_A	-1.5487	0.042051	3639504
C1_08350C_A	-1.5491	0.0244	30515030
C3_01800C_A	-1.54954	0.147044	3636586
CR_00630W_A	-1.55341	0.079872	3639690
FAA2	-1.55612	0.099476	3641731
FRE3	-1.55702	0.233523	3641789
RAD18	-1.55709	0.238704	3641637
C7_02450W_A	-1.55799	0.028533	3636972
CDC37	-1.5583	0.02255	3639210

ZCF21	-1.55962	0.019691	3645762
SDH1	-1.56139	0.050481	3642477
POL93	-1.56176	0.024619	3639299
C2_01250W_A	-1.5624	0.174282	3638793
HAS1	-1.56243	0.015931	3636443
FCA1	-1.56257	0.035832	30515327
HEX1	-1.56345	0.084122	3646981
CR_08290W_A	-1.56389	0.045551	3642103
C2_01070W_A	-1.56491	0.035304	3638834
C6_04450W_A	-1.56618	0.061023	3645982
C3_07280C_A	-1.56687	0.07232	3638431
C1_12540W_A	-1.56785	0.33013	3647607
ZPR1	-1.56808	0.033643	3640177
CR_04830C_A	-1.56834	0.316831	3647297
C3_05950W_A	-1.57207	0.175754	3641728
C3_07390C_A	-1.57498	0.080505	3638472
C2_10680W_A	-1.57586	0.055589	3641252
C2_07920W_A	-1.57621	0.161916	3642294
CR_09410W_A	-1.57637	0.115455	3641933
C3_04920C_A	-1.5765	0.011125	3635268
C4_04240C_A	-1.57676	0.068915	3646856
FCR1	-1.57711	0.015434	3638542
C4_02210W_A	-1.57737	0.644453	3635695
C1_12820C_A	-1.57751	0.087716	3646826
PGA17	-1.57763	0.657618	3637391
PRY1	-1.57773	0.656866	3646674
C3_00030C_A	-1.58002	0.035077	3640571
MBF1	-1.58062	0.017742	3640171
C1_02800W_A	-1.581	0.433597	3636873
C7_01570C_A	-1.58122	0.427849	3637040
MTLA1	-1.58191	0.213358	3643729
CDC43	-1.58197	0.32749	3648206
BUD31	-1.58245	0.329508	3634796
C5_03110C_A	-1.58461	0.340253	30515306
VPS20	-1.58538	0.095169	3637427
LSM6	-1.58733	0.088953	30515363
CFL1	-1.5882	0.223206	3641810
CDC4	-1.58924	0.031033	3640125
C4_03370C_A	-1.58948	0.17706	3646796
MBP1	-1.5918	0.046424	3635281
C5_03440W_A	-1.59277	0.12069	30515310
C7_01700W_A	-1.5934	0.007478	3637013
FCY21	-1.59451	0.042359	3643822
KOG1	-1.59471	0.024384	3642488
UTP21	-1.59609	0.01471	3635757
SAP1	-1.59612	0.448397	3640256
C5_03700C_A	-1.59625	0.115305	3646990

C6_04470C_A	-1.59631	0.020322	3645985
RSP5	-1.59798	0.011182	3643898
C4_00030C_A	-1.59835	0.047533	3648055
C4_01710C_A	-1.59885	0.010031	3635708
C2_00190C_A	-1.59985	0.027971	3638904
C3_03530W_A	-1.59985	0.329084	3638312
C5_02990W_A	-1.60021	0.333333	3637817
C1_05120W_A	-1.60025	0.030601	3647667
CR_09100C_A	-1.60088	0.125658	3641928
C2_09310C_A	-1.60226	0.236915	3644104
C7_01720W_A	-1.60296	0.049865	3637011
C5_05250C_A	-1.604	0.089293	3636472
ORM1	-1.6046	0.011973	3640307
ARL1	-1.60647	0.05775	3635637
C7_03040W_A	-1.60909	0.296179	3641379
GDT1	-1.6102	0.093418	3645445
C3_01660W_A	-1.61085	0.14466	3636607
C2_09460C_A	-1.61212	0.062278	3647713
C3_00380C_A	-1.61275	0.118178	3640540
C2_01440C_A	-1.61295	0.302016	30515090
CR_07850W_A	-1.61512	0.083572	3647347
C2_07240C_A	-1.61539	0.493017	3643486
HNM4	-1.61546	0.469741	3636802
C1_05220C_A	-1.61549	0.480133	3642464
CDC39	-1.6171	0.008991	3646278
C3_07940W_A	-1.61728	0.024634	3647453
C6_01980C_A	-1.61731	0.049968	3644560
TFG1	-1.61752	0.019162	3635645
CR_02460W_A	-1.61876	0.022315	3644922
C7_03280C_A	-1.61895	0.006331	3641353
PRN3	-1.6204	0.219674	3639878
AFT2	-1.62196	0.085101	3643511
CR_00980C_A	-1.623	0.007089	3640426
ROM2	-1.62376	0.010508	3637434
C6_01090C_A	-1.62454	0.340356	3644020
C7_03970C_A	-1.62458	0.037996	3643117
NIK1	-1.62505	0.039676	3641358
FUR4	-1.62695	0.094759	3638305
YHB5	-1.62775	0.052211	3647343
SNF2	-1.62798	0.008777	3635855
BGL22	-1.63131	0.071404	3641935
MRP20	-1.63143	0.077974	3640224
C1_14250C_A	-1.6319	0.007267	3643307
CR_04580W_A	-1.63213	0.043138	3640948
SSD1	-1.6348	0.019201	3636520
C7_01490W_A	-1.63506	0.046815	3637051
GAP1	-1.63516	0.007235	3637806

YMX6	-1.6359	0.136419	3640255
RLP24	-1.6361	0.02302	3645748
C4_07060W_A	-1.63798	0.007275	3642908
C1_02090C_A	-1.6386	0.115283	3644577
C6_02450W_A	-1.64171	0.098365	3639167
GLN3	-1.64326	0.011885	3636453
C2_03560C_A	-1.64494	0.051364	3637337
PGA4	-1.64583	0.013283	3636406
C3_04750W_A	-1.64681	0.133712	3635252
HMX1	-1.64982	0.008463	3639294
ZCF18	-1.65094	0.057993	3641635
C1_01590C_A	-1.65119	0.010763	3640223
DCK2	-1.65149	0.039725	3637384
C2_06550W_A	-1.65258	0.262221	3646559
C1_01040W_A	-1.65335	0.076111	3640183
MOB2	-1.65337	0.050864	3639381
C3_03440C_A	-1.6537	0.219046	3638356
C2_05300C_A	-1.65475	0.01634	3643423
HSE1	-1.65614	0.008538	3640407
FGR16	-1.65714	0.312096	3644411
SGE1	-1.6575	0.058948	3642614
C7_02690C_A	-1.65761	0.155713	3647649
C7_01030C_A	-1.65865	0.009788	3638019
ADE8	-1.65961	0.010803	3642999
C4_04350W_A	-1.65982	0.044281	3646852
CR_01780W_A	-1.6599	0.152403	3640157
CR_04370W_A	-1.6624	0.093004	3641004
C3_07430W_A	-1.66308	0.006763	3638484
C2_01740C_A	-1.66335	0.170126	3635733
C1_05980W_A	-1.66344	0.062199	3639872
CHO2	-1.66573	0.031835	3647690
TERT	-1.6668	0.055427	3640729
TIM23	-1.66688	0.039823	3643777
VPS28	-1.66695	0.019203	3645794
C4_04820C_A	-1.66797	0.089717	3641165
YCK2	-1.66894	0.006823	3643655
CR_03620C_A	-1.67006	0.018613	3643182
C2_02540W_A	-1.6701	0.014629	3635820
C2_05380W_A	-1.67126	0.094114	3643432
C1_01750W_A	-1.67437	0.180677	3645413
MNN11	-1.67498	0.169467	3636792
YVH1	-1.67537	0.068388	3643218
HOS3	-1.67663	0.020632	3637886
AMS1	-1.67699	0.044604	3637882
CKB2	-1.67716	0.054629	3637741
NAM7	-1.67754	0.007268	3641540
TAC1	-1.67813	0.028413	3643755

CKB1	-1.67884	0.029926	3638799
C7_03680W_A	-1.67926	0.119753	3646228
PHHB	-1.67965	0.007157	3638871
MNN23	-1.68206	0.029808	3634813
C1_04370C_A	-1.68234	0.027836	3647994
CR_04270C_A	-1.68245	0.34881	3640992
C1_04220C_A	-1.68312	0.04748	3644721
PLB1	-1.68339	0.519373	3644561
C6_03740W_A	-1.68343	0.532647	3640304
DUR4	-1.68397	0.143563	3645012
C2_00490W_A	-1.68411	0.002102	3638870
C2_06600W_A	-1.68545	0.009532	3646563
C3_04170W_A	-1.68574	0.634574	3635182
C2_05400W_A	-1.68684	0.212777	3643435
C3_02180C_A	-1.68816	0.039915	3636643
C4_02590C_A	-1.68867	0.106713	3637913
C1_06470W_A	-1.69203	0.013337	30515007
CR_02950C_A	-1.69206	0.051262	3642590
UPC2	-1.69264	0.004482	3646489
C7_00610C_A	-1.69383	0.042338	3638030
C6_01080C_A	-1.69517	0.204517	3644019
C1_03040W_A	-1.69559	0.102398	3636842
NOC2	-1.69821	0.009529	3647596
C4_01280C_A	-1.69849	0.050489	3635700
HLJ1	-1.69883	0.024195	3643158
CR_04240C_A	-1.70405	0.009934	3640989
CR_08940W_A	-1.70494	0.021922	3641914
CIS2	-1.70729	0.035748	3639366
C7_00350C_A	-1.70753	0.027165	3638039
C1_00460W_A	-1.71056	0.028249	3639316
C1_00580W_A	-1.7136	0.077831	3639361
C1_03310W_A	-1.71362	0.010192	3636856
MRT4	-1.71369	0.0142	3639219
ZFU2	-1.71683	0.101159	3638534
C4_03850W_A	-1.7178	0.005006	3647853
NOP15	-1.71793	0.02481	3637966
C4_07130W_A	-1.71845	0.004623	3642938
CR_01090W_A	-1.71868	0.018622	3640379
C1_13530W_A	-1.71925	0.016591	3636170
FGR27	-1.7195	0.023807	3646988
C3_06240C_A	-1.72382	0.287975	30515213
C6_00640C_A	-1.72398	0.043283	3647134
C3_01190C_A	-1.72479	0.01495	3647961
C4_03410W_A	-1.72535	0.026313	3646807
C7_02540W_A	-1.72904	0.146069	3636939
C3_05360C_A	-1.72909	0.008409	3643676
BUD23	-1.72919	0.132646	3642629

CR_07190W_A	-1.72929	0.014817	3645269
C4_01090C_A	-1.72933	0.200909	3635685
C7_02340C_A	-1.73056	0.040967	3636933
C2_04730W_A	-1.73245	0.045313	3647787
MUB1	-1.73331	0.008098	3642246
PGA25	-1.73389	0.029401	3647890
MST1	-1.73482	0.036569	3636831
ADAEC	-1.73482	0.008011	3637342
GCD7	-1.73584	0.012434	3637367
C2_04850C_A	-1.73717	0.003986	3648114
ARG5,6	-1.73802	0.00645	3634830
C1_05200C_A	-1.74066	0.01811	3642466
BUD2	-1.74102	0.033273	3641541
CR_02800C_A	-1.74121	0.028703	3642559
AFG3	-1.74242	0.002571	3636589
C6_00310W_A	-1.74296	0.007768	3646071
RNR22	-1.74551	0.012296	3645217
C5_01170W_A	-1.74643	0.219127	3642662
C4_06680C_A	-1.74684	0.004463	3642950
DAL81	-1.74699	0.032271	3640419
OSM1	-1.74771	0.022868	3645573
CR_00670C_A	-1.74906	0.087393	3639655
VID27	-1.74919	0.005047	3647894
CR_03120W_A	-1.74966	0.00126	3645123
C1_08710W_A	-1.75013	0.055318	3634937
C2_02730W_A	-1.75014	0.003355	3643035
GNP1	-1.75049	0.007368	3646073
SDH4	-1.75118	0.096498	3636497
C2_00620C_A	-1.7517	0.199363	3638849
C3_05140C_A	-1.75175	0.031051	3635161
C4_03780C_A	-1.75369	0.026254	3645353
C1_04360C_A	-1.75439	0.002897	30514985
C3_04790W_A	-1.75492	0.029144	3635215
RLI1	-1.75574	0.003423	3636888
C1_06910C_A	-1.75613	0.010143	3639526
C6_00550W_A	-1.75621	0.007352	3647144
RPF2	-1.75808	0.010305	3643467
RMT2	-1.75935	0.022132	3641569
C6_02930W_A	-1.76123	0.00614	3639243
YAK1	-1.76245	0.004714	3647801
C2_00700W_A	-1.76403	0.001879	3638884
C1_05230W_A	-1.76556	0.174298	3642463
C7_03730C_A	-1.76598	0.017376	3647867
MTR10	-1.7662	0.0113	3647235
FRP6	-1.76666	0.009488	3641601
C2_00220C_A	-1.76708	0.012522	3638902
BCR1	-1.76741	0.006516	3645280

BNA4	-1.76898	0.001422	3640528
C2_00170C_A	-1.77123	0.010163	3638906
CR_00600C_A	-1.77513	0.00889	3639660
PHR2	-1.77921	0.027989	3639334
CST5	-1.77933	0.035131	3645981
UBR1	-1.78328	0.002664	3637871
C2_09670C_A	-1.7842	0.009629	3643792
PGA62	-1.78566	0.017804	3637879
CET1	-1.78637	0.002848	3640140
CR_08330W_A	-1.79104	0.025293	3642080
GPI13	-1.79125	0.114726	3637424
DCP2	-1.79276	0.003512	3642069
C1_03120W_A	-1.79294	0.008129	3636926
FET33	-1.793	0.048738	3641544
CR_04350C_A	-1.79503	0.006538	3641001
C3_01850W_A	-1.79924	0.046899	3636565
C7_03880C_A	-1.80354	0.166408	3643142
C1_02940C_A	-1.80615	0.018063	3636832
CYM1	-1.80689	0.004782	3642244
C6_03910C_A	-1.80816	0.047731	3640263
C3_00530C_A	-1.81029	0.028304	3640588
C2_10140W_A	-1.81098	0.145438	3643934
C1_05800C_A	-1.81124	0.14755	3639887
C4_01130C_A	-1.8128	0.136813	3635682
PBS2	-1.81328	0.001197	3641748
SUI3	-1.81418	0.005341	3643130
CCW14	-1.81623	0.119115	3642650
C2_07000W_A	-1.81723	0.034705	3643534
CR_03350C_A	-1.8178	0.025035	3645142
HRQ2	-1.81845	0.074908	3647850
C6_02110W_A	-1.81967	0.095569	3647553
C7_02460C_A	-1.82008	0.003911	3636946
CR_03110W_A	-1.82102	0.09389	3645124
UTR2	-1.82398	0.009899	3636591
HMS1	-1.82445	0.001876	3641570
C1_09340C_A	-1.82706	0.010533	3634804
CR_03640C_A	-1.82711	0.003076	3643190
C4_00750C_A	-1.82843	0.091015	3645763
C3_01090W_A	-1.82943	0.04611	30515176
HGT6	-1.83038	0.010539	3638873
C3_04700W_A	-1.83313	0.020251	3635247
CR_02940C_A	-1.83405	0.339731	3642589
C1_03050W_A	-1.83645	0.004884	3636843
C4_02110W_A	-1.83726	0.016326	3635673
POP2	-1.83727	0.00807	3640301
NEP1	-1.83786	0.012814	3644545
C2_07680W_A	-1.83822	0.017396	3642339

C5_02010C_A	-1.83846	0.004983	3643772
BLM3	-1.83968	0.001596	3642310
C3_04940W_A	-1.84226	0.009192	3635270
C5_03570W_A	-1.84244	0.006477	3646976
C4_01560C_A	-1.84478	0.030235	3635714
NMA111	-1.84539	0.001284	3640179
CHS3	-1.84669	0.0048	3636199
C2_02520W_A	-1.84731	0.01429	3635818
C7_00940W_A	-1.84852	0.000528	3637955
C1_06070W_A	-1.84863	0.008723	3639824
C1_02900C_A	-1.84896	0.021482	3636869
C2_01820C_A	-1.84968	0.018151	3635831
CR_04750W_A	-1.8521	0.169735	30515399
C1_10080W_A	-1.85252	0.075446	3634814
ESA1	-1.8528	0.002707	3640518
LEU42	-1.85354	0.002535	3643808
YAF9	-1.85463	0.052256	3647864
CR_08400C_A	-1.85694	0.023167	3642115
RAX1	-1.85818	0.004237	3635256
C1_13330C_A	-1.85881	0.003056	3636150
CR_07250C_A	-1.85886	0.00758	3646028
ARX1	-1.85894	0.001419	3636811
C3_05400C_A	-1.86019	0.385365	3643681
C1_05540C_A	-1.86553	0.003521	3642503
C5_01040W_A	-1.86639	0.451723	3642649
BLP1	-1.86667	0.002801	30515065
C3_02930W_A	-1.86673	0.451639	3638380
STE2	-1.86685	0.444437	3646630
SAP3	-1.86696	0.452893	3635197
NCR1	-1.86846	0.001434	3643314
C4_00880W_A	-1.87334	0.012157	3635681
CHT1	-1.8756	0.119852	3639704
ECM3	-1.8777	0.014855	3635754
WOR4	-1.87889	0.005062	3638454
PPR1	-1.87972	0.00892	3636535
CR_03930C_A	-1.8811	0.014253	3640957
DAO2	-1.88117	0.035047	3646797
HPA2	-1.88144	0.069742	3647907
SAP6	-1.88176	0.542229	3639229
C7_01010W_A	-1.8818	0.524187	3638021
C2_02420C_A	-1.88189	0.006449	3635756
IFF11	-1.88191	0.524838	3640580
OP4	-1.88195	0.522065	3636125
MRV1	-1.88207	0.521062	3635610
UTP13	-1.88306	0.001458	3637718
CR_08040W_A	-1.88663	0.003682	3644135
C2_07740W_A	-1.8888	0.000307	3642302

RGD3	-1.8922	0.000463	3648003
C3_07700W_A	-1.89227	0.054694	3638457
CTA6	-1.89265	0.04635	3642907
MAS1	-1.89383	0.000389	3636855
PIF1	-1.89428	0.009284	3646054
SST2	-1.89479	0.055218	3637754
CR_10730C_A	-1.89541	0.001644	3638998
STF2	-1.89802	0.001705	30515083
MLP1	-1.89943	0.001875	3635688
CR_09480W_A	-1.90001	0.017385	3641939
UBC8	-1.90005	0.000292	3645433
ABD1	-1.90094	0.003663	3638456
STE4	-1.90126	0.042094	3637358
C1_00200C_A	-1.90275	0.146027	3639336
SAM50	-1.90379	0.155835	3641721
C2_07430C_A	-1.90656	0.004998	3645201
C6_03520C_A	-1.90945	0.194795	3640259
C1_07810C_A	-1.90951	0.030563	3640708
RPM2	-1.91146	0.000994	3644790
MSU1	-1.91285	0.075244	3643919
C3_00450C_A	-1.91338	0.001059	3640548
NMD5	-1.91644	0.003666	3645745
C7_02350C_A	-1.91907	0.004643	3636932
C6_02720C_A	-1.91959	0.00793	3639212
CR_06040W_A	-1.92261	0.002407	3644836
C2_04750W_A	-1.92679	0.035261	3647785
C2_06200C_A	-1.92887	0.012027	3646908
CR_05910W_A	-1.93002	0.017096	3646680
PTC2	-1.93361	0.000258	3640105
C6_02090C_A	-1.93483	0.010305	6335445
RLM1	-1.93539	0.019399	3635703
CR_00820C_A	-1.93609	0.007496	3640413
CCT8	-1.93669	0.00043	3639324
C3_07920W_A	-1.94046	0.000361	30515226
IRE1	-1.94082	0.00153	3640726
C4_02960W_A	-1.94225	0.002103	3637873
C3_07680W_A	-1.94234	0.000751	3638459
CR_08200C_A	-1.94436	0.000463	3642087
C2_00810C_A	-1.94466	0.032658	3638810
CR_08190W_A	-1.94559	0.125177	3642084
C2_09820W_A	-1.94591	0.022948	3643801
EXO70	-1.95319	0.006326	3636948
C4_00680W_A	-1.95351	0.009791	3645753
C2_07410W_A	-1.95392	0.010393	3645203
C2_00360C_A	-1.95466	0.001531	3638825
RCL1	-1.95725	0.002953	3645199
PEP12	-1.95749	0.005101	3637737

VPS27	-1.96128	0.00125	3639326
RIB1	-1.96326	0.001698	3642607
PRP22	-1.96417	0.003783	3636404
CR_06740W_A	-1.96513	0.067056	3646616
C2_07800W_A	-1.96622	0.100748	3642325
C2_09240C_A	-1.96638	0.041733	3644064
C4_05400C_A	-1.96845	0.005739	3646952
PMC1	-1.96956	0.00104	3636627
C1_05210C_A	-1.96998	0.024949	3642465
LAC1	-1.97082	0.134374	3641717
MED22	-1.97215	0.259037	3640217
PCL1	-1.97219	0.262582	3646356
C6_01940W_A	-1.97247	0.262578	30515337
CR_02440W_A	-1.97251	0.276163	3644923
C5_04010C_A	-1.97263	0.27187	3647439
C7_01150W_A	-1.97358	0.010981	3645913
CR_05730C_A	-1.97664	0.002362	3646698
C2_03420C_A	-1.98028	0.004639	3637411
GRX3	-1.98223	0.001001	3637858
NAG3	-1.98244	0.00388	3645969
CR_04820W_A	-1.98556	0.002638	3647298
FGR44	-1.9884	0.005217	3635166
EFG1	-1.98889	0.002659	3644145
FPG1	-1.98994	0.000489	3641632
C3_00630W_A	-1.9902	0.000192	3640576
C4_07210W_A	-1.99364	0.004592	3642959
C1_03750W_A	-1.99436	0.009681	3644690
MED15	-1.99468	0.000356	3640761
MTR2	-1.99563	0.002641	30515167
IPT1	-1.99707	0.002893	3634941
C1_01140C_A	-1.99922	0.003561	3640202
C1_10420C_A	-1.9995	0.005289	3647178
C6_02460C_A	-2.00031	0.006146	3639168
SOG2	-2.00045	0.000115	3644475
C2_02770W_A	-2.00129	4.27E-05	3643031
C2_10850C_A	-2.00316	0.00192	3641232
C1_12580W_A	-2.00317	0.002639	3647603
C1_04960C_A	-2.00392	0.00432	3647527
C4_00080C_A	-2.00549	0.002519	3648065
ZCF20	-2.00823	0.003878	3647059
RER2	-2.01527	0.002752	3636399
SSN6	-2.01753	0.001574	3638518
YKE2	-2.01779	0.006727	30515454
CR_10750C_A	-2.01932	0.010652	3638942
C5_02740W_A	-2.02154	0.031042	3637739
C5_01050C_A	-2.02238	0.0175	3642648
MUC1	-2.0231	3.53E-05	3645771

TBP1	-2.02966	0.001499	3647675
NAB3	-2.03045	8.62E-05	3639209
SFL1	-2.03147	9.45E-05	3642498
RVS161	-2.03165	0.000219	3638005
PTR22	-2.03372	0.00072	3645634
C4_07100C_A	-2.03409	9.89E-05	3642903
FGR3	-2.0354	0.00126	3641179
INN1	-2.03749	5.38E-05	3642578
C2_00840W_A	-2.03865	0.016253	3638807
C1_01850C_A	-2.04117	0.003731	3645431
C3_06760W_A	-2.04326	0.00373	3638502
HBR1	-2.04502	0.000694	3639295
C3_07580W_A	-2.04547	0.002582	30515225
C2_09510C_A	-2.04788	0.000404	3643816
LEU5	-2.04945	0.000825	3638838
CWT1	-2.0514	0.001025	3647595
GAP5	-2.0527	0.000129	3646954
RPA190	-2.05314	0.000325	3647677
C3_00100W_A	-2.0556	0.002877	3640567
CWC22	-2.05732	0.033909	3643938
SIZ1	-2.06384	0.000129	3640219
C2_04820W_A	-2.06543	0.002674	3647776
CR_06290C_A	-2.06932	0.002854	3644856
CR_08500W_A	-2.06983	0.00057	3642136
C2_01310W_A	-2.07443	0.002404	3638788
C5_00350C_A	-2.07621	0.0018	3641554
C4_04510W_A	-2.07759	0.00053	3641120
C3_03190C_A	-2.079	0.00314	3638427
BRF1	-2.08077	0.000591	3646689
RCA1	-2.08222	0.000221	3639303
C2_02800W_A	-2.08306	0.017971	30515096
C5_03560W_A	-2.08423	0.005751	3646975
C2_10050W_A	-2.08565	0.001103	3643949
C1_05360C_A	-2.08629	0.000795	3642482
C2_00770W_A	-2.09107	0.005793	3638909
C4_02570C_A	-2.0929	0.00868	3637916
C5_03740W_A	-2.09673	0.002451	3647237
PTH1	-2.09832	0.001082	3637710
C4_01860C_A	-2.10085	0.0057	3635590
C3_04840C_A	-2.10749	0.019718	3635224
FAB1	-2.10981	4.93E-05	3635766
C7_00160C_A	-2.11285	0.000531	3637972
C4_05440C_A	-2.11288	0.003358	3646960
C3_00130C_A	-2.11361	2.36E-05	3640593
SLU7	-2.11475	0.023537	3638501
C4_02920W_A	-2.11634	0.000583	3637878
C1_07640C_A	-2.11979	0.022097	3646659

C3_06600C_A	-2.12135	0.005967	3642280
C4_01970W_A	-2.12482	0.000549	3635663
KCH1	-2.12802	0.041301	3636988
C7_00040C_A	-2.13222	0.014632	3638002
C1_06540C_A	-2.13372	0.001593	3639520
RRP6	-2.13459	0.000509	3647535
C7_03400C_A	-2.13909	0.000369	3646717
GAP2	-2.13924	5.97E-05	3643662
C1_04040C_A	-2.14115	0.001455	3646429
C5_03030W_A	-2.14119	0.005524	3637822
SET6	-2.14198	0.022385	3644590
MET4	-2.14351	0.002168	3641219
C2_02200W_A	-2.14374	0.002728	3635772
CUP1	-2.14379	0.000937	30515318
ZSF1	-2.14383	2.3E-05	3641257
C1_10910C_A	-2.14468	0.000965	3644445
UBI4	-2.14505	5.53E-05	3638432
C4_04670C_A	-2.15115	0.000405	3641140
C5_00820W_A	-2.15205	0.001875	3644128
CAN3	-2.15462	0.000515	3647066
CR_01410C_A	-2.15475	0.001336	3640136
C6_04550C_A	-2.15502	0.000134	3645976
XOG1	-2.15909	0.010931	3636837
C7_03890C_A	-2.16058	0.00016	3643108
CR_02670C_A	-2.16561	0.000889	3642572
PPG1	-2.16917	0.01459	3641143
SFP1	-2.17505	0.000123	3635225
C5_05140W_A	-2.17524	0.002159	3636460
TPK1	-2.1763	3.01E-05	3634879
C6_04290W_A	-2.17648	0.000364	3646280
GIR2	-2.17664	0.000578	3636962
CR_06450W_A	-2.1819	0.010748	3645279
C3_06740W_A	-2.18506	0.001099	3638503
PSP1	-2.18557	5.37E-05	3644551
C7_02550C_A	-2.19038	0.001119	3637018
C1_14480W_A	-2.19437	0.003591	3643328
MTG2	-2.19616	0.002107	3646693
C6_01890C_A	-2.19719	0.000152	3641598
C2_05160C_A	-2.19811	2.75E-05	3643461
RAD1	-2.20173	0.000467	3641192
C1_00540C_A	-2.20496	0.00024	3639367
RTG1	-2.20616	0.000565	3634929
C1_11690W_A	-2.2095	0.000156	3645322
C5_05460C_A	-2.20954	0.04982	3636527
C5_00800C_A	-2.20992	2.79E-05	3644126
C3_04260W_A	-2.21166	0.001274	3635140
SUL2	-2.21285	0.0195	3637835

TFB3	-2.21494	0.003147	3644115
CR_08410W_A	-2.2176	0.000706	3642116
CR_00090C_A	-2.22052	0.001694	3639734
CR_03710C_A	-2.22074	0.000163	3643224
C4_05740C_A	-2.22247	1.06E-05	3641806
C5_02580W_A	-2.22837	0.001236	3637721
SGS1	-2.2359	0.000167	3641258
C5_02510C_A	-2.23835	0.001318	3637767
UTP15	-2.23908	0.000444	3643906
C3_07900C_A	-2.24141	0.002131	3647450
MET10	-2.2417	6.63E-06	3644088
CR_10640W_A	-2.24209	0.03511	3639014
C7_03000C_A	-2.24639	0.00198	3641383
C2_10740C_A	-2.24745	0.000229	3641236
C4_06850C_A	-2.24824	0.000325	3642912
STD1	-2.25073	1.21E-06	3647459
C2_03760C_A	-2.25144	0.000489	3637371
AXL1	-2.25206	0.009554	3641938
C1_12610W_A	-2.25277	0.000818	3646825
RIX7	-2.25348	0.00018	3637735
PRP45	-2.25617	0.028862	3639166
PMA1	-2.25837	8.97E-05	3640531
NUP49	-2.25906	0.000357	3636173
TSR1	-2.26057	6.24E-05	3642135
PDX3	-2.26169	0.000166	3640949
RIT1	-2.26305	0.008451	3638492
CR_03940W_A	-2.26405	0.001095	3648378
BMT9	-2.26966	0.000279	3635654
URA7	-2.27118	6.75E-06	3636510
C3_07490W_A	-2.27467	2.84E-06	3638528
C5_01430C_A	-2.27535	0.001147	3647056
C6_03780C_A	-2.27632	0.000227	3640308
C1_07150W_A	-2.27963	0.040382	30515019
C1_12680W_A	-2.28105	2.07E-05	3646833
C1_12860C_A	-2.2835	0.021875	3636179
SPL1	-2.28427	1.88E-05	3638035
CR_07320C_A	-2.28433	0.000346	3646057
DES1	-2.2866	6.23E-06	3636529
C6_03220W_A	-2.2866	0.013979	3639179
C3_04330C_A	-2.29423	2.41E-05	3635272
NSA1	-2.29454	0.000129	3642313
SBA1	-2.29745	0.00012	3640305
C3_00570C_A	-2.29814	8.3E-05	3640584
HEM1	-2.30361	0.00013	3640149
C6_02980C_A	-2.30386	0.001109	3639192
C5_04910W_A	-2.30399	0.02938	3636417
C2_10600W_A	-2.30596	0.00089	3641245

C2_09680W_A	-2.30657	0.002166	3643791
C2_04570W_A	-2.3087	0.000162	3645441
YAH1	-2.30918	0.001943	3638368
HSP104	-2.30995	0.000123	3642097
C1_03830C_A	-2.31257	0.000163	3644678
IMP4	-2.31417	0.000137	3644138
NAT4	-2.31564	0.000861	3635704
DRS1	-2.31691	1.07E-05	3639045
C3_02790W_A	-2.31693	0.006443	3638416
C1_12950W_A	-2.32037	0.000194	3636157
C1_00570C_A	-2.33254	0.000753	3639362
C4_07010C_A	-2.33298	0.03096	3642966
CR_02510W_A	-2.33819	0.00023	3647694
C1_04590W_A	-2.33991	0.001314	30514987
UGA1	-2.34134	0.002467	3637329
C6_00980C_A	-2.34204	0.001075	3644046
SPT5	-2.34429	1.99E-05	3635847
CR_06680C_A	-2.3459	4.31E-05	3646623
C7_01990C_A	-2.34928	0.000865	3636966
C3_00500C_A	-2.35117	0.000884	3640515
SUI1	-2.35167	0.000172	3647730
CR_03080C_A	-2.35556	0.018416	3645104
NIP7	-2.35563	0.00046	3645880
TRY2	-2.35651	0.000385	3647670
CR_07030C_A	-2.35666	0.001709	3645730
C1_11290W_A	-2.36163	0.004504	3644553
C1_13560W_A	-2.36641	0.004124	3636174
C1_11900C_A	-2.36903	0.000728	3642005
C1_10230C_A	-2.37178	3.24E-06	3634880
PRN1	-2.3729	0.002819	3639882
CR_07670W_A	-2.3734	0.000223	3647255
C3_07670W_A	-2.37386	5.38E-06	3638460
PEX5	-2.37391	3.73E-06	3645026
C1_00980W_A	-2.37453	0.012932	30514959
CR_10400W_A	-2.38056	0.000162	3638956
C2_10540W_A	-2.38179	6.52E-05	3641200
ALR1	-2.38346	3.52E-06	3636616
HST7	-2.38607	4.61E-05	3640953
C2_02390W_A	-2.38676	0.006831	3635829
C6_00700C_A	-2.38732	0.000204	3647074
C1_12570C_A	-2.39201	6.74E-06	3647604
TIM17	-2.39446	0.000879	3647790
CTM1	-2.39566	0.000276	3638951
ATG15	-2.39974	1.9E-05	3641641
HNT1	-2.40563	1.82E-06	3644441
C3_02030W_A	-2.40664	0.00044	3636580
PNC1	-2.40706	0.000413	3646232

C1_09850C_A	-2.40906	7.33E-07	3634849
DIM1	-2.40924	0.005192	3636135
C1_06840C_A	-2.41063	2.36E-05	30515012
EXM2	-2.41256	0.000972	3642929
C1_07960W_A	-2.41294	0.009056	3640725
C6_02150C_A	-2.41366	0.007636	3645888
C1_11770C_A	-2.41565	1.98E-06	3645327
C3_07800C_A	-2.41641	0.000409	3647461
IFF3	-2.41687	5.42E-05	3643189
C6_02690C_A	-2.41746	0.00025	3639228
C2_04120C_A	-2.4189	2.96E-05	3637377
PDR6	-2.42143	3.94E-05	3638824
CR_10040W_A	-2.42868	0.002144	3639034
UTP5	-2.4312	3.09E-05	3638922
C4_06420W_A	-2.43519	0.000101	3642795
IFF9	-2.43712	0.000132	3641008
C2_09810C_A	-2.44234	0.044737	3643802
HAP43	-2.44299	5.26E-07	3644531
C3_00940W_A	-2.44725	8.84E-07	3639851
C3_03740W_A	-2.44837	0.035406	3645640
BMS1	-2.4489	1.29E-06	3639857
C2_06120C_A	-2.45133	0.039468	3644084
SLR1	-2.4515	5.72E-06	3643957
C4_04010W_A	-2.45517	0.002818	3641218
ISY1	-2.4561	0.000448	3646233
C7_02930C_A	-2.45967	2.28E-05	3641390
OPT3	-2.46089	2.66E-05	3644918
C7_01400C_A	-2.46434	0.000427	3645893
ARO80	-2.46473	0.000313	3636808
C5_04290C_A	-2.46654	2.58E-05	3636455
C7_00260C_A	-2.47821	2.64E-06	3637978
C4_01930C_A	-2.48978	0.005656	3635634
FGR13	-2.48991	0.002145	3648078
TUS1	-2.49059	7.69E-06	3646144
C4_03580W_A	-2.49191	0.015339	3645369
DAL5	-2.49203	0.00576	3638401
C3_00300W_A	-2.49438	1.25E-05	3640524
UTP4	-2.49476	5.29E-05	3636657
KNS1	-2.49796	0.000904	3636228
C1_06320W_A	-2.49823	0.047225	3648118
CKA1	-2.50473	1.43E-05	3639018
CR_10000C_A	-2.50565	0.016753	3639030
DBP8	-2.50656	0.000154	3646692
MRPL37	-2.50721	0.019903	3644782
C2_06480W_A	-2.51115	0.000221	3646568
SOD3	-2.51388	1.57E-07	30515347
EST1	-2.51537	2.41E-06	3636526

C2_04100W_A	-2.51985	0.003706	3637379
PET127	-2.5218	0.000116	3644417
C4_05900C_A	-2.52828	1.21E-06	3641796
C3_02710W_A	-2.52883	7.73E-05	30515190
C5_00780C_A	-2.52889	0.000402	3644124
DAL1	-2.53076	0.000252	3640564
C2_09980W_A	-2.53294	0.000159	3643965
C2_03150C_A	-2.53369	0.004624	3642990
C4_02510W_A	-2.53533	0.000124	3637865
PGA55	-2.5354	2.68E-05	3645784
SAM35	-2.53978	0.000133	3643329
C2_04280W_A	-2.54069	4.19E-05	3637326
MRPL27	-2.54296	0.000359	3636884
MHP1	-2.54376	2.99E-05	3642460
CR_10430C_A	-2.54415	0.046243	3638975
OPT7	-2.54479	0.008941	3645008
SVF1	-2.54491	1.7E-05	3639289
C1_02960C_A	-2.54937	3.58E-06	3636834
C2_01680C_A	-2.55032	6.32E-05	3635744
DUR32	-2.55032	0.009594	3636142
C1_01580W_A	-2.55268	8.54E-05	3640222
CR_02100C_A	-2.55439	0.000166	3640141
C3_02910W_A	-2.55582	0.010224	3638378
PRP39	-2.55648	0.011149	3635832
C2_07760W_A	-2.56055	7.53E-05	3642298
C7_00200W_A	-2.56112	6.86E-05	3637968
C1_04120C_A	-2.56377	0.009981	3644701
TRM12	-2.56736	0.001499	3641738
MNN4	-2.57336	1.49E-06	3642784
FGR15	-2.57341	0.000191	3638882
C6_03290W_A	-2.58203	1.27E-05	3639195
C2_05770W_A	-2.58323	3.69E-05	3645579
CTR1	-2.58559	1.08E-05	3647077
PTP1	-2.58645	3.5E-05	3642093
GEF2	-2.58784	2.1E-06	3644935
MSW1	-2.5882	0.005922	3637743
C3_05160C_A	-2.5892	6.85E-08	3635229
MEF2	-2.58949	5.71E-05	3639564
C3_00840C_A	-2.60131	0.025941	3647758
CIP1	-2.60145	0.007726	3644018
SEC2	-2.60191	2.29E-05	3636119
C2_07060W_A	-2.60681	5.46E-05	3643500
IFE2	-2.60764	1.14E-06	3648027
C4_07220C_A	-2.60948	2.49E-05	3642955
ARG4	-2.61606	0.000134	3646237
C1_00520W_A	-2.6188	0.000381	3639310
PAM16	-2.6188	5E-05	3643321

C4_00610W_A	-2.61895	3.3E-05	3645770
POP4	-2.62415	0.000604	3639241
CAP1	-2.62813	1.52E-05	3636640
C1_11160C_A	-2.62922	0.001618	3644399
RTG3	-2.63524	1.83E-05	3644423
PNG2	-2.63666	0.000129	3645568
KRR1	-2.63694	4.22E-05	3644542
GRR1	-2.63829	2.52E-07	3636392
KIN3	-2.6406	2.45E-05	3641199
RXT3	-2.64164	0.000744	3643431
JIP5	-2.64287	6.14E-06	3634922
NOC4	-2.6442	9.98E-07	3645224
C1_06380C_A	-2.64676	0.016911	3639559
MNL1	-2.65074	3E-07	3646050
CR_00660W_A	-2.65203	0.033959	3639658
C2_03550C_A	-2.65208	1.09E-07	3637338
C7_01370W_A	-2.65231	0.023019	3645901
CTF1	-2.65302	9.02E-05	3635788
CAN2	-2.65668	4.86E-07	3644017
OYE2	-2.65707	2.37E-05	3641603
C6_02420W_A	-2.65734	9.48E-05	3645885
TOP1	-2.65809	5.1E-08	3644034
CR_02890C_A	-2.65847	6.59E-05	3642585
PPT1	-2.66345	0.000248	3636602
STP2	-2.66461	5.56E-06	3636214
CR_10800C_A	-2.66502	1.29E-07	3638948
PWP2	-2.66788	1.59E-07	3640416
CR_03200C_A	-2.66901	1.8E-06	3645131
C6_03370W_A	-2.6706	2.31E-06	3639230
LEU3	-2.67129	7.17E-07	3637757
C4_06990W_A	-2.67279	2.24E-05	3642968
C6_02380W_A	-2.67784	5.83E-08	3645883
QDR1	-2.68055	1.4E-06	3640941
CR_00430C_A	-2.68378	0.008927	30515366
C2_09360W_A	-2.68538	0.002246	3644109
C3_01150C_A	-2.69066	0.000546	3648401
RRP8	-2.69068	1.91E-05	3643900
DFG16	-2.69653	5.33E-06	3637413
DAL9	-2.69759	0.001546	3645653
ARG81	-2.70034	1.19E-07	3634775
C5_04990W_A	-2.70078	0.011952	3636538
C1_10890C_A	-2.70477	1.02E-05	3644447
PRS5	-2.70819	1.6E-06	3642296
YHB4	-2.70865	4.47E-05	3638999
C4_03740W_A	-2.7098	8.25E-05	3645357
GUT1	-2.71096	9.94E-05	3648082
C5_00130C_A	-2.7175	5.9E-05	3647881

C4_02850W_A	-2.71992	3.07E-07	3637910
FCY24	-2.72053	0.015172	3641902
MIS12	-2.72132	8.04E-07	3639669
FMT1	-2.72509	3E-05	3646530
C2_01370C_A	-2.72512	3.98E-07	3635723
C2_02710C_A	-2.72693	2.58E-05	3643003
C1_08840W_A	-2.72956	0.033704	3634919
C7_02120C_A	-2.73337	0.001452	3637019
HEX3	-2.73437	1.97E-05	3635776
MIG1	-2.73513	2.43E-07	3637812
C2_00200W_A	-2.74192	5.67E-07	3638903
CR_10410C_A	-2.7422	1.84E-06	3638957
C3_00160C_A	-2.74454	7.3E-07	3640591
VAC7	-2.74704	9.57E-05	3646849
CR_05550C_A	-2.7564	2.98E-07	3647593
C5_05340W_A	-2.7591	1.94E-06	3636400
UTP8	-2.7608	2.24E-09	3640547
C2_09790W_A	-2.76177	5.34E-05	3643804
DYN1	-2.76386	1.5E-06	3635236
MET16	-2.76528	6.48E-05	3642964
ERO1	-2.77107	6.09E-08	3634810
C1_14190C_A	-2.77177	2.74E-06	3643300
C2_02570W_A	-2.77196	0.005933	3635858
C4_03190W_A	-2.77201	9.41E-06	3637838
C1_02850W_A	-2.77418	7.13E-09	3636862
SPB1	-2.77509	2.69E-07	3648179
C2_08850C_A	-2.77919	1.3E-06	3643890
SSU72	-2.78101	1.35E-05	3645129
MSN4	-2.78135	7.52E-07	3634911
C7_03760W_A	-2.78819	0.001142	3647865
C3_06820C_A	-2.79074	1.6E-08	3638544
C5_03960W_A	-2.79921	2.89E-10	3647444
C2_05670C_A	-2.79943	0.004377	3645570
C4_02090C_A	-2.80818	6.42E-06	3635642
CR_07830C_A	-2.81051	9.22E-09	3647345
C1_09390W_A	-2.81055	0.000421	3634851
C5_02810W_A	-2.8154	0.008876	30515304
ADA2	-2.81941	9.82E-06	3644450
CNH1	-2.82118	3.27E-08	3648056
C6_02330W_A	-2.83554	4.57E-07	3645877
C4_03830W_A	-2.83738	1.33E-05	3647851
LIG1	-2.8382	8.65E-07	3636947
C4_05330C_A	-2.84138	3.47E-05	3646959
GCV2	-2.84281	1.22E-07	3646505
SWE1	-2.84579	3.5E-06	3634866
C1_11580W_A	-2.85052	1.91E-07	3645330
FET3	-2.85099	0.000325	3647153

C2_02960C_A	-2.85279	5.98E-06	3643011
DUS4	-2.86132	4.43E-06	3641580
CEF1	-2.86182	1.93E-06	3634808
C2_09420W_A	-2.8628	0.000826	3647718
NAG1	-2.86289	6.75E-06	3645966
SET2	-2.86438	1.14E-06	3643923
CR_06840W_A	-2.86592	6.97E-08	3647322
SDA1	-2.86931	3.05E-08	3646688
CPA2	-2.87005	5.38E-05	3640385
CR_03870W_A	-2.8701	0.000506	3643187
CLN3	-2.87308	0.000157	3642624
C1_14580C_A	-2.87386	7.12E-08	3643290
C7_01660C_A	-2.87745	1.04E-05	3636983
C4_02690W_A	-2.88076	1.57E-09	3637861
C2_02820C_A	-2.89557	1.89E-05	3643027
CPH2	-2.8972	1.3E-07	3646067
REP1	-2.89747	0.005094	3639708
SPR28	-2.90176	0.02033	3637716
CR_06060W_A	-2.90608	0.000118	6335358
C2_05060C_A	-2.90695	0.001367	3643451
C3_02890C_A	-2.91247	0.011634	3638336
HAL9	-2.91276	1.05E-05	3643757
C3_02020W_A	-2.91342	1.29E-10	3636592
C3_01540W_A	-2.91939	0.016384	3636567
CR_09740W_A	-2.9203	1.8E-07	3639008
C2_10790C_A	-2.92913	4.24E-05	3641242
CR_01440C_A	-2.93015	1.73E-05	30515374
TCO89	-2.93176	8.31E-09	3644788
C4_00060W_A	-2.93267	5.1E-06	3648061
RIO2	-2.93771	4.21E-07	3642066
CR_07910C_A	-2.94186	1.71E-08	3644142
C1_11880W_A	-2.94266	0.00014	3642007
C1_03140W_A	-2.94688	4.7E-07	3636929
PRB1	-2.94818	9.13E-08	3643521
URK1	-2.94852	4.31E-07	3635186
C7_00870W_A	-2.95496	0.020924	3638054
RPA34	-2.95696	1.01E-05	3634883
GNP3	-2.95737	7.85E-05	3639027
CNT	-2.95831	3.91E-08	3644071
CR_09460C_A	-2.96095	3.1E-08	30515453
HST2	-2.96192	0.000425	3640162
C1_12150C_A	-2.96392	0.004875	3642049
C2_08860W_A	-2.96554	9.48E-06	3645776
C1_09540W_A	-2.96772	2.17E-05	3634885
CR_07690W_A	-2.96773	0.009325	3648194
DBP3	-2.96951	1.08E-06	3634868
C4_07150W_A	-2.96993	0.022133	3642935

C2_00790C_A	-2.97306	9.51E-06	3638907
C4_03950C_A	-2.9748	0.030664	30515263
HGT18	-2.9749	0.00122	3645111
C3_02920W_A	-2.97619	3.5E-07	3638379
ARO10	-2.97662	0.001729	3647320
C2_00650W_A	-2.97773	1.57E-08	3638889
CR_07920W_A	-2.9801	3.75E-06	3644141
C2_04770W_A	-2.9846	0.000537	3647783
C6_01350W_A	-2.98497	1.1E-05	3641642
OYE32	-2.98689	4.03E-08	3642919
C3_03300C_A	-2.98727	4.84E-08	3638361
C1_13430C_A	-2.9933	0.000778	3636223
CR_02970C_A	-2.99389	4.01E-10	3642603
CR_06320C_A	-2.99612	0.000189	3644845
ECM21	-2.99905	2.8E-07	3634784
C7_01040C_A	-3.0027	4.7E-06	3638018
C2_01660C_A	-3.00283	1.58E-05	3635742
CIC1	-3.00431	6.3E-06	3644059
SFT1	-3.01226	0.000747	30515372
C6_03460W_A	-3.01566	2.1E-11	3640252
C6_01550C_A	-3.02056	2.03E-05	3641653
RAX2	-3.02434	9.69E-07	3647610
C3_03000W_A	-3.03077	0.030565	3638389
EAF3	-3.03572	0.000599	3648318
CR_01370C_A	-3.03901	1.41E-05	3640168
MNN4-4	-3.04386	0.001269	3639250
IFF8	-3.04569	0.016921	3644117
BUD21	-3.05142	1.95E-06	3640541
NOG1	-3.05145	6.52E-09	3641736
RRS1	-3.05834	4.11E-06	3639352
C7_03210W_A	-3.0621	0.001261	3641371
PLB3	-3.06308	7.36E-08	3647089
CDR4	-3.06557	3.61E-06	3640711
C3_05860C_A	-3.06559	3.82E-06	3641759
C5_00160W_A	-3.06994	0.000602	3647871
FAD3	-3.07066	0.001797	3636124
CR_00750C_A	-3.07438	1.26E-06	3640438
LIP7	-3.07477	0.040476	3641917
C2_00540W_A	-3.07681	1.57E-09	3638865
SAP2	-3.07976	2.01E-05	3647354
C6_02350C_A	-3.08069	6.66E-06	3645881
OPT8	-3.08128	1.07E-06	3640266
C1_08700W_A	-3.0832	1.97E-08	3634936
C7_01070C_A	-3.08924	1.27E-07	3637949
CR_06230W_A	-3.0962	7.36E-05	3644867
MAK21	-3.09858	1.39E-08	3635147
C1_01630W_A	-3.10033	4.9E-09	3640194

C4_01500W_A	-3.10062	1.3E-06	3635617
TOR1	-3.10268	1.04E-08	3643480
SHY1	-3.10505	4E-08	3634824
CR_05770W_A	-3.10693	0.000346	3646694
GYP5	-3.11185	4.3E-09	3641246
FRP1	-3.11293	2.68E-09	3645005
C3_05850W_A	-3.11364	2.3E-06	3641758
C2_09160W_A	-3.11714	0.000105	3644090
CR_03160W_A	-3.11838	0.000104	3645117
NMD3	-3.12025	2.38E-10	3646614
C3_04720C_A	-3.12267	1.59E-07	3635249
TOK1	-3.1254	2.4E-08	3645754
C2_07580W_A	-3.1298	0.003021	3645216
SDS24	-3.13972	3.81E-09	3640756
SLM2	-3.14081	1.16E-08	3647559
C3_01560W_A	-3.14135	1.04E-07	3636669
C6_02790C_A	-3.14173	3.41E-08	3639245
PTP2	-3.14326	2.46E-09	3647858
PTR3	-3.14604	6.65E-05	3645428
C2_06620W_A	-3.14618	0.008632	3646564
C2_06440C_A	-3.14747	0.003422	3646565
SLN1	-3.1515	4.03E-10	3640422
MEP2	-3.15269	1.48E-07	3645022
HAP41	-3.15364	1.47E-09	3647405
C5_03630C_A	-3.15787	0.002093	3646983
C1_09840C_A	-3.1596	1.79E-08	3634848
CTN1	-3.16012	0.000448	3645414
C4_05980C_A	-3.16216	0.000313	3646516
C1_07950C_A	-3.16239	0.000117	3640724
C2_04080W_A	-3.16872	3.37E-10	3637381
CR_10810C_A	-3.17194	4.93E-07	3638949
CIRT4B	-3.17429	2.21E-08	3642560
C5_01610W_A	-3.1765	0.000432	3647251
ROT1	-3.17902	0.000386	3639350
C5_00310C_A	-3.18757	1.24E-06	3641576
C2_00640W_A	-3.18791	3.85E-05	3638847
MAK5	-3.18984	6E-08	3643454
C4_04810C_A	-3.19326	1.3E-07	3641112
C1_05270C_A	-3.19499	8.93E-06	3642476
C5_04170W_A	-3.19832	4.23E-11	3636543
GYP7	-3.1985	1.27E-09	3646245
C5_02280C_A	-3.2129	1.69E-08	3637712
C6_01340C_A	-3.21601	0.000256	30515331
ARD	-3.21664	1.26E-06	3647906
C3_03670W_A	-3.21751	5E-07	3645649
C4_04460C_A	-3.21765	4.15E-09	3641127
FAL1	-3.21782	1.64E-07	3639839

BUL4	-3.21968	8.17E-06	3642037
CR_02930W_A	-3.22678	5.41E-08	3642588
CR_07750C_A	-3.22714	9.27E-08	3647350
HSX11	-3.23002	0.004797	3635660
C1_05160C_A	-3.2317	3.78E-09	3642468
NAG6	-3.23389	6.87E-09	3645961
C3_01130C_A	-3.23632	3.23E-06	3639901
C6_02020C_A	-3.23808	1.14E-09	3647563
C2_02120W_A	-3.24287	2.74E-07	3635746
CR_08420W_A	-3.244	2.08E-10	3642117
TBF1	-3.24828	3.53E-08	3637327
CR_05540C_A	-3.25108	3.58E-07	3647594
UGT51C1	-3.25228	2.2E-08	3640147
CKA2	-3.25519	2.61E-06	3643444
MRPL36	-3.25665	5.94E-06	3643733
SUV3	-3.26166	7.15E-07	3645451
YTH1	-3.26949	3.67E-09	3645572
C2_08330W_A	-3.27144	5.24E-07	3648166
REG1	-3.27439	9.71E-09	3638858
SPT14	-3.27475	6.81E-08	3641019
SPB4	-3.27632	1.6E-06	3647252
C1_09740C_A	-3.28008	0.025123	30515035
C1_06820W_A	-3.28561	1.27E-08	3639493
CR_10280W_A	-3.28639	5.38E-05	3638934
C2_00090W_A	-3.28676	0.000343	3647910
C4_05970W_A	-3.28955	1.12E-07	3646514
C1_12370W_A	-3.29291	1.91E-05	3642061
C7_02160W_A	-3.29303	3.55E-07	3636994
WSC2	-3.29559	1.52E-06	3639225
C1_01160C_A	-3.29638	3.22E-07	3640204
C1_09520C_A	-3.29931	1.13E-09	3634766
CPP1	-3.30075	9.39E-08	3634865
CRP1	-3.30111	8.09E-10	3634826
C1_04840C_A	-3.30195	0.006687	3644785
C3_00340W_A	-3.30217	0.003005	3640544
OPT1	-3.30556	0.000889	3640150
STB3	-3.31726	1.12E-06	3645781
C1_01890C_A	-3.32249	1.69E-11	3645427
C4_05230C_A	-3.32802	4.24E-08	3646156
NOT5	-3.32965	3.45E-11	3640763
RGA2	-3.33916	7.83E-09	3635661
C1_10970W_A	-3.34234	8.92E-10	3644427
MRPL40	-3.34954	2.37E-08	3640968
HSK3	-3.35533	4.37E-06	3635730
CR_03910C_A	-3.35876	8.51E-11	3640954
C1_14350W_A	-3.37485	3.34E-13	3643281
SFT2	-3.37758	6E-06	3644442

ATX1	-3.37791	0.002532	30515426
C4_06080W_A	-3.38064	0.012286	30515280
C7_00590W_A	-3.38084	3.25E-07	3637994
C1_11570W_A	-3.38152	0.012072	30515055
ZCF9	-3.38156	2.36E-07	3638869
SYG1	-3.38398	3.01E-05	3644777
C2_05270W_A	-3.38493	2.46E-09	3643470
SPO7	-3.38546	5.1E-06	3645576
HPT1	-3.38619	9.85E-08	3643034
C1_06440C_A	-3.39022	9.83E-07	3639552
C1_04510W_A	-3.3903	0.001355	3646147
CCS1	-3.39209	7.19E-12	3644476
C3_02900W_A	-3.39556	4.95E-05	3638337
DOT6	-3.39835	2.98E-08	3640078
PDE2	-3.40052	6.22E-10	3636877
C2_00280C_A	-3.40184	1.34E-05	3638802
C4_05590W_A	-3.40364	1.57E-11	3641776
SSY5	-3.40674	6.4E-12	3642140
PHO86	-3.40888	4.7E-05	3642323
C2_06090W_A	-3.41873	2.55E-07	3644097
C2_07020C_A	-3.42308	0.00039	3643497
CR_10420W_A	-3.42496	1.36E-08	3638974
NBP2	-3.42716	3.94E-08	3636963
BAS1	-3.43114	3.96E-11	3641136
SPO11	-3.43775	0.004575	3643873
C4_00100C_A	-3.43789	3.12E-11	3645004
SAP10	-3.44638	2.38E-07	3641126
HGT4	-3.45073	4.96E-10	3635269
BUB3	-3.45249	6.18E-08	3646362
C1_07560W_A	-3.45606	2.29E-05	3646676
C7_01610W_A	-3.45632	1E-05	30515353
C1_10360C_A	-3.45937	1.08E-08	3634836
C3_06370C_A	-3.46306	1.97E-09	3642258
TEC1	-3.46633	3.3E-10	3635261
PHO4	-3.46685	2.5E-08	3641817
MLH3	-3.46856	4.03E-08	3643294
C6_02970C_A	-3.47393	3.92E-07	3639190
MRF1	-3.47476	3.44E-10	3645321
C5_02070C_A	-3.47582	3.76E-13	3643746
C4_06790W_A	-3.48396	1.67E-08	3642918
MPP10	-3.48442	1.06E-09	3647912
SMC6	-3.48735	2.04E-08	3637039
C1_11620W_A	-3.49076	0.002096	3645344
C5_02440C_A	-3.49622	0.000201	3637728
C7_01210C_A	-3.50182	4.76E-08	3645904
C3_02670W_A	-3.50526	0.000652	3638405
C1_10880W_A	-3.50543	1.03E-11	3644449

CR_06330C_A	-3.50651	0.000148	3644846
UTP18	-3.50954	1.81E-08	3643123
YME1	-3.51158	1.44E-10	3641816
C2_02630W_A	-3.51479	5.89E-05	3635839
C2_01050W_A	-3.51502	0.044919	30515086
ZCF27	-3.5269	4.61E-10	3635631
PSO2	-3.52804	0.001299	3641241
PEA2	-3.53441	5.7E-05	3647673
MSN5	-3.55085	3.75E-09	3637889
NOP14	-3.55105	1.62E-10	3635266
RIM21	-3.55379	3.03E-09	3643739
C1_10120C_A	-3.56018	1.61E-11	3634779
C5_03410C_A	-3.56743	1.25E-05	3646347
C3_04620C_A	-3.59361	6.87E-05	3635155
C2_03690C_A	-3.59438	9.41E-07	3637376
ALS9	-3.59972	1.28E-09	3640281
PGA41	-3.60007	0.003277	3642800
CDC34	-3.60394	6.19E-11	3637028
VPS21	-3.60601	5.94E-10	3642092
PTK2	-3.60886	1.39E-12	3641611
DBP7	-3.6089	6.98E-10	3645903
CUP2	-3.60999	7.55E-10	3636189
PUT2	-3.61026	1.2E-09	3636439
ELP3	-3.61221	3.37E-10	3641747
C1_10710C_A	-3.6148	0.001572	3644407
C3_04540C_A	-3.61542	1.83E-08	3635262
RMP1	-3.61553	0.002933	3636215
IML1	-3.62022	4.82E-10	3636813
ISW2	-3.62207	1.18E-06	3642235
C2_07040W_A	-3.62525	3.7E-06	3643499
ICL1	-3.63199	1.68E-08	3646146
C2_00690W_A	-3.64308	0.000305	3638885
HSP70	-3.64701	1.66E-05	3636229
C5_04180W_A	-3.64835	0.000216	3636544
CR_00040C_A	-3.65605	1.17E-09	3639675
NSA2	-3.66917	4.86E-10	3642259
C4_06040W_A	-3.67246	0.000189	3646521
C4_01230C_A	-3.67264	1.2E-06	3635706
C6_04510C_A	-3.67663	2.13E-08	3645955
C2_05830C_A	-3.67724	1.61E-08	3647483
C7_01170C_A	-3.68941	0.000216	3645914
C2_06510W_A	-3.70847	3.68E-08	3646570
CR_03260W_A	-3.71052	2.25E-07	30515389
C2_03000C_A	-3.71597	2.82E-07	3643022
C2_06570C_A	-3.71808	5.39E-09	3646561
ELF1	-3.72375	1.65E-13	3641903
CR_07940W_A	-3.72767	4.74E-05	3644139

C1_11020W_A	-3.73531	9.82E-06	3644420
C1_13380W_A	-3.73926	0.000391	3636217
C2_02110C_A	-3.74086	0.000307	3635745
C5_02020C_A	-3.74619	1.22E-12	3643771
C5_04050W_A	-3.74955	2.88E-10	3647735
LIP4	-3.75194	9.47E-09	3645987
NOP13	-3.75296	8.53E-12	3638469
C3_05450C_A	-3.75377	3.02E-09	3643669
C3_00410C_A	-3.75406	0.005491	3640552
SSF1	-3.75743	2.04E-09	3647085
HIP1	-3.76706	6.66E-12	3643761
C3_02350W_A	-3.77047	3.09E-12	3636618
C4_00690C_A	-3.77091	3.39E-09	3645752
MSS51	-3.77732	1.82E-10	3639031
CKS1	-3.78812	1.25E-08	3647732
C1_05790W_A	-3.78913	0.000552	30515002
ENP2	-3.79367	8.79E-12	3646234
C1_10140C_A	-3.79863	2.96E-07	3634780
C2_02270C_A	-3.81176	1.29E-07	3635805
C1_02470W_A	-3.81228	0.01603	3636851
CSP1	-3.81351	0.011985	3648242
C4_03230C_A	-3.81382	0.012116	3637836
SOF1	-3.8158	2.29E-11	3640585
SEN15	-3.82396	0.000331	3646411
PEL1	-3.83096	2.98E-09	3637989
C4_04660C_A	-3.83315	2.45E-14	3641150
C2_10170C_A	-3.83559	7.55E-10	3643931
MSB2	-3.83858	1.34E-13	3635830
C6_04240W_A	-3.84705	0.000278	3646276
C5_03150W_A	-3.85455	1.23E-08	3637799
C2_08180C_A	-3.85912	7.6E-09	3642317
RPF1	-3.87085	1.85E-09	3637890
C1_11610C_A	-3.873	1.34E-10	3645345
C3_06160C_A	-3.88174	1.08E-10	3641766
CR_02420W_A	-3.89143	1.7E-08	3644926
CR_05640C_A	-3.89285	0.000453	3646690
C5_01470C_A	-3.89332	0.000686	3647050
C1_07130C_A	-3.89453	9.06E-11	3644462
C6_03210C_A	-3.89666	2.56E-06	3639178
TPS2	-3.90761	1.52E-10	3636892
TCC1	-3.91376	1.24E-12	3638524
C6_04050W_A	-3.91479	4.18E-07	3647830
C2_02280W_A	-3.92087	5E-10	3635806
C2_07250C_A	-3.92601	0.000883	3643487
CR_03840C_A	-3.93072	0.01729	3643184
SLD1	-3.9337	1.12E-10	3638406
SGD1	-3.94069	5.53E-11	3643191

RIM2	-3.95	5.59E-07	3645448
BRE1	-3.95566	8.52E-13	3641568
C1_09040C_A	-3.95777	4.92E-08	3634769
DBP2	-3.95825	3.69E-08	3647692
C1_13880C_A	-3.96016	1.98E-11	3636133
C6_04300W_A	-3.96453	3.98E-08	3646286
DAL8	-3.96579	4.07E-05	3635181
IFH1	-3.96944	3.78E-09	3637770
C1_01710W_A	-3.9702	1.44E-10	3640201
NBN1	-3.9736	2.17E-09	3637410
C1_09710C_A	-3.98053	1.78E-12	3634818
SHA3	-3.98962	4.67E-10	3644580
ADR1	-3.99508	2.14E-10	3637866
DFI1	-3.99791	7.08E-15	3638038
C6_04140C_A	-3.99976	8.1E-10	3647970
C4_00820W_A	-4.00537	0.0001	3645741
CTN3	-4.00796	8.31E-10	3647396
CR_09440C_A	-4.00926	4.62E-10	3641937
C2_09500W_A	-4.01076	1.43E-11	3643817
BOI2	-4.01291	3.94E-11	3640404
C1_04820C_A	-4.01438	0.000153	3644787
LPI9	-4.01622	2.05E-12	3637060
C1_00860W_A	-4.02311	9.93E-07	3639358
C2_05990C_A	-4.02702	0.001614	3644074
C7_03480W_A	-4.02964	4.09E-08	3646230
PCL5	-4.03594	2.9E-11	3636466
CR_00460C_A	-4.04829	2.56E-06	3639726
NPR2	-4.04899	2.07E-08	3638360
MED3	-4.06064	4.98E-07	3646522
NAN1	-4.06115	6.15E-11	3637932
RPD3	-4.06181	1.41E-11	3642580
C3_07070C_A	-4.06984	8.45E-06	3638446
BUD14	-4.07078	1.94E-09	3643469
TPT1	-4.07212	1.83E-07	3640543
MTG1	-4.08746	4.58E-06	3645139
UGA3	-4.09129	7.8E-14	3638926
ISA2	-4.09585	8.9E-13	3638513
SSK1	-4.09656	7.73E-10	3636184
C6_00520W_A	-4.10244	0.007265	3647147
CR_02700W_A	-4.10473	0.012952	3642575
C6_00380C_A	-4.10506	3.9E-10	3646085
C7_01020C_A	-4.12171	2.9E-09	3638020
C5_03260C_A	-4.1221	6.08E-07	3646360
C1_05370C_A	-4.12861	1.18E-09	3642481
RRP15	-4.13	4.22E-10	3648397
ZCF29	-4.13165	3.35E-13	3641370
MIF2	-4.15186	1.19E-15	3639220

CR_09610C_A	-4.1566	7.25E-08	3647094
MNN14	-4.16376	2.76E-12	3643651
C2_07280W_A	-4.16503	0.004063	3643475
PFK26	-4.17486	1.67E-10	3634912
PRP8	-4.18433	4.14E-14	3642125
CR_06980W_A	-4.20133	0.000612	3645735
CR_06970C_A	-4.21397	7.87E-08	3645736
SIR2	-4.21601	7.34E-06	3638845
FZO1	-4.21917	9.68E-11	3646848
PTC5	-4.22874	2.51E-17	3642072
ISU1	-4.23126	6.19E-13	3637007
C6_04400W_A	-4.24447	0.000316	3645949
SOK1	-4.24848	4.06E-11	3642495
C3_05460W_A	-4.24909	6.52E-07	3643670
FHL1	-4.25649	1.83E-14	3643493
RRP9	-4.2588	1.88E-11	3642576
C7_02110W_A	-4.26644	5.7E-12	3637022
NAR1	-4.27099	4.71E-14	3634916
CR_09920W_A	-4.27332	3.68E-12	3639028
C7_03590C_A	-4.27966	1.06E-11	3646239
C2_08950W_A	-4.2801	3.21E-11	3645788
PRM9	-4.28195	0.033636	30515173
HCA4	-4.28786	2.99E-12	3637911
C4_04190C_A	-4.30079	0.039188	3646860
C6_02230W_A	-4.30167	9.26E-12	3645866
C3_07910W_A	-4.30607	0.044644	3647449
DAC1	-4.31197	4.06E-09	3645967
ENP1	-4.33419	2.99E-10	3647861
ZCF35	-4.34523	5.53E-08	3641740
RPA12	-4.34644	6.1E-06	3643477
MOH1	-4.34949	9.58E-05	3646809
CR_04110W_A	-4.35716	8.44E-14	3641026
BMT5	-4.35919	2.07E-12	3635781
CR_04710W_A	-4.35945	0.000677	3648141
C1_14380C_A	-4.36299	1.86E-09	3643285
COX19	-4.36475	1.58E-06	3636220
C2_05980C_A	-4.37396	0.000192	3644075
SPO72	-4.37462	4.93E-15	3644072
C7_03270W_A	-4.37551	8.2E-11	3641365
FTH2	-4.38644	8.42E-08	3640401
C3_07290W_A	-4.38725	1.45E-11	3638430
FGR23	-4.40485	2.21E-09	3636555
DIP2	-4.40797	1.77E-18	3640762
LAS1	-4.41864	1.05E-10	3644073
CR_08680C_A	-4.43076	1.16E-16	3642127
ECM1	-4.4326	9.67E-06	3641224
C7_03380W_A	-4.43464	5.13E-07	30515359

C2_03500W_A	-4.43601	1.18E-16	3637345
GPT1	-4.43678	0.000914	3647671
CR_02550C_A	-4.43682	7.17E-11	3647689
C6_01400W_A	-4.44008	0.007154	3641604
AAF1	-4.45325	5.94E-18	3642284
IHD1	-4.45483	0.001357	3640291
C2_05810W_A	-4.46435	2.48E-09	3647481
C1_09650W_A	-4.46985	3.28E-17	3634870
CR_03250C_A	-4.47441	1.28E-13	3645102
AHR1	-4.47694	1.57E-10	3641733
C2_03570C_A	-4.48375	4.44E-05	3637336
NDT80	-4.49197	1.63E-17	3638840
SEF1	-4.49688	1.53E-15	3644945
HNM1	-4.49874	9.39E-11	3638857
C4_00460C_A	-4.5088	3.37E-19	3645010
C1_10250C_A	-4.51032	0.000214	3634882
C7_00340C_A	-4.51659	5.45E-14	3638040
CNS1	-4.52897	7.68E-12	3639365
IFM3	-4.53155	1E-06	3642335
C4_00260W_A	-4.54129	3.96E-17	3645037
C6_04530C_A	-4.55277	1.15E-18	3645970
C4_02260C_A	-4.56039	1.51E-07	3635690
YWP1	-4.5628	2.09E-13	3643915
SIP5	-4.5752	8.94E-14	3639865
FUN31	-4.59889	3.47E-13	3642249
C2_09000C_A	-4.59917	0.000671	3645782
C4_06620C_A	-4.60726	2.56E-05	3642813
TSR2	-4.60777	3.84E-09	3636845
C6_01040C_A	-4.62725	4.1E-12	3644014
ALP1	-4.65449	7.53E-15	3644437
PDR16	-4.65452	9.93E-11	3644679
CR_10760C_A	-4.65523	7.95E-06	3638943
KAR4	-4.65524	7.17E-06	3644937
C4_03020W_A	-4.65584	2.9E-13	3637934
ATO10	-4.66239	0.003146	3640394
C6_03000C_A	-4.66382	0.002894	3639194
PAM18	-4.67314	1.26E-08	3645747
C6_00170C_A	-4.67721	2.4E-19	3647903
C1_00270W_A	-4.67884	1.7E-16	3639300
RPO41	-4.68321	7.15E-18	3639378
TPO4	-4.6839	3.26E-11	3640956
C5_04530W_A	-4.68587	3.5E-09	3636507
C3_02840W_A	-4.6907	0.000317	3638331
NCS2	-4.69942	2.85E-16	3643216
GCA1	-4.70082	3.23E-05	3634903
C1_10440W_A	-4.70608	5.53E-12	3647180
C1_00090W_A	-4.71013	0.000201	3639304

MDJ1	-4.71451	2.8E-14	3646980
IFF6	-4.7179	4.23E-13	3644087
C1_04720W_A	-4.72755	2.54E-07	30514988
C3_02420C_A	-4.73019	2.44E-07	3638342
C1_06760C_A	-4.7302	3.92E-17	3639505
C6_04200C_A	-4.73155	5.82E-06	30515344
C2_06660W_A	-4.73475	1.25E-05	3648418
GST3	-4.73475	0.047253	3645278
EPL1	-4.74001	1.51E-14	3639732
CR_01630C_A	-4.74597	0.000263	3640085
C5_03460C_A	-4.7606	8.82E-06	3646345
C1_09330W_A	-4.77549	7.68E-06	3634802
C1_09130W_A	-4.78524	1.35E-12	3634943
DOT4	-4.79464	1.47E-17	3646810
BUD5	-4.80028	1.39E-13	3647315
C3_02630C_A	-4.82308	6.92E-08	3638328
C3_03690W_A	-4.83605	0.00443	3645647
C1_08890C_A	-4.83685	0.004511	3634924
C5_02680W_A	-4.84172	4.19E-14	3637774
C1_06510C_A	-4.84468	5.61E-16	3639549
C7_02660C_A	-4.85504	0.001218	3647652
C2_09660W_A	-4.85611	2.06E-15	3643793
C1_07530W_A	-4.85738	0.000529	30515025
CR_08650C_A	-4.87366	1.15E-15	3642124
ZCF30	-4.88806	8.53E-14	3642051
NOP8	-4.88933	5.81E-12	3646290
C3_07550C_A	-4.88957	5.55E-14	3638525
C3_06560W_A	-4.89485	1.3E-11	3642276
MIH1	-4.91109	1.83E-10	3648188
C4_03350C_A	-4.91343	1.64E-12	3646794
C2_08450W_A	-4.91405	1.83E-11	3643905
REI1	-4.91499	2.03E-12	3647536
NOP4	-4.93205	3.93E-18	3647991
C3_07400W_A	-4.93254	1.64E-11	3638471
FLU1	-4.94634	2.89E-09	3637048
C3_07650C_A	-4.96455	2.32E-06	3638488
C4_01420W_A	-4.97296	6.16E-12	3635626
HIT1	-4.9799	1.47E-05	3637854
RAD3	-5.00374	3.68E-20	3638014
C4_02170C_A	-5.00522	0.000185	3635667
C1_05930C_A	-5.01775	1.63E-13	3639862
MUP1	-5.02397	2.13E-20	3642008
C4_01960C_A	-5.03159	3.14E-14	3635664
C7_03500W_A	-5.03441	1.59E-10	3646246
CSP2	-5.04551	0.00195	3645749
CR_01710W_A	-5.04602	7.37E-08	3640130
CCE1	-5.04612	0.001809	3640395

MIT1	-5.04666	1.65E-14	3644089
OLE2	-5.04802	2.98E-15	3643503
C2_09570C_A	-5.04894	8.64E-05	3643811
YHB1	-5.07632	2.45E-16	3647353
C6_01620W_A	-5.08499	1.1E-08	3641647
RMS1	-5.09067	4.42E-12	3646361
C1_06350W_A	-5.09596	9.77E-20	3639562
C3_00230C_A	-5.11751	1.97E-05	3640557
ARG1	-5.13218	5.87E-12	3639691
CR_00810W_A	-5.15239	1.76E-06	3640415
C1_08830C_A	-5.16249	0.029578	3634918
SRP40	-5.171	3.05E-16	3642605
SNT1	-5.17267	5.75E-18	3642033
HAP2	-5.1767	5.47E-15	3641800
TRK1	-5.18113	2.31E-19	3644153
C5_03800W_A	-5.18387	1.23E-14	3647230
DHH1	-5.18966	2.16E-12	3639490
C3_01110C_A	-5.19239	0.011155	3639835
ARG8	-5.19341	2.38E-12	3641131
CAG1	-5.21714	1.45E-05	3636469
C2_05640W_A	-5.23505	5.87E-12	3645566
PAN6	-5.24785	1.06E-13	3647684
C4_07170C_A	-5.2499	2.29E-05	30515289
C2_06800C_A	-5.26534	9.47E-10	3643518
C4_04560C_A	-5.27352	3.18E-20	3641105
CR_10470C_A	-5.3069	1E-15	3638979
KRE1	-5.31272	4.02E-10	3643180
C4_00490W_A	-5.33192	4.24E-13	3645013
C2_06740W_A	-5.35005	1.16E-14	3648024
MET2	-5.35908	3.87E-18	3640070
C2_02300W_A	-5.37462	2.88E-08	3635808
C7_01690W_A	-5.37553	4.76E-11	3636980
MTLA2	-5.38951	0.027783	3643766
C5_03510C_A	-5.39498	2.22E-10	3646969
C2_01240C_A	-5.39922	1.61E-05	3638794
C2_02920W_A	-5.40693	3.49E-25	3643016
HSP78	-5.41188	6.13E-13	3637414
IFR1	-5.41223	7.95E-05	3643930
CR_09800C_A	-5.42138	1.44E-21	3638984
C1_07220W_A	-5.42209	1.85E-19	3644460
C3_01770C_A	-5.42289	5.53E-09	3636588
OPT2	-5.43812	7.37E-07	3644915
C5_03920C_A	-5.46672	4.81E-13	3647448
C1_08630W_A	-5.46802	2.14E-16	3634928
C5_00570W_A	-5.47272	4.66E-16	3641589
C1_07980C_A	-5.47286	1.12E-19	3640696
SUA71	-5.47448	1.58E-17	3636879

C2_00410C_A	-5.47474	3.52E-27	3638820
C3_07570C_A	-5.47475	4.33E-14	3638522
AUR1	-5.48801	1.9E-19	3642617
YPT53	-5.49122	5.55E-15	3642483
C1_01150C_A	-5.49651	3.13E-20	3640203
TRY3	-5.49771	1.36E-15	3642652
CR_10060W_A	-5.51503	5.72E-18	3639035
C3_06700C_A	-5.51544	3.69E-17	3642247
C6_00530C_A	-5.52235	3.36E-06	30515326
PRP5	-5.52432	4.49E-17	3638447
HRR25	-5.53051	8.41E-21	3645878
ATS1	-5.53251	1.83E-10	3642079
C4_03000C_A	-5.54568	2.13E-09	3637935
RSN1	-5.56779	8.21E-10	3638308
C1_11080W_A	-5.58728	1.1E-22	3644416
UTP9	-5.5884	9.98E-21	3638451
RIM101	-5.61683	3.69E-19	3643280
BUD22	-5.62256	8.89E-14	3640391
CR_00310C_A	-5.62325	2.08E-18	3639715
C5_04860C_A	-5.62461	8.57E-05	3636437
C7_01940C_A	-5.63776	2.1E-18	3636971
C3_00620C_A	-5.63819	3.68E-15	30515171
CAN1	-5.6407	5.3E-24	3644035
GAP4	-5.69972	2.22E-18	3644463
KTI12	-5.70194	4.11E-12	3645140
C1_09790C_A	-5.7082	7.47E-23	3634843
C5_03210C_A	-5.73915	0.025862	3646364
C3_07370W_A	-5.74772	2.08E-16	3638474
HAP42	-5.77922	6.34E-14	3635729
CR_03360W_A	-5.78288	1.92E-15	3645141
IFM1	-5.7957	8.35E-13	3641389
CR_04600W_A	-5.79781	1.12E-17	3640950
C6_04180W_A	-5.79831	1.35E-17	3648176
C2_08920W_A	-5.80308	2.58E-21	3645797
C2_05750W_A	-5.83428	1.19E-19	3645577
C5_04120C_A	-5.85568	1.88E-10	3636522
AUT7	-5.85706	1.39E-17	30514999
OPY2	-5.85977	4.73E-23	3640765
C3_05840W_A	-5.86668	0.020502	3641757
C6_00290W_A	-5.86677	8.06E-16	3646069
C3_01340W_A	-5.86716	0.022401	3636650
RPC10	-5.90306	7.79E-16	3643292
C2_01540W_A	-5.90311	1.42E-13	3635804
C6_01590W_A	-5.90746	1.22E-25	3641650
FET34	-5.91869	9.63E-21	3647131
C1_11000C_A	-5.93999	9.3E-12	3644422
C7_04090C_A	-5.94406	1.61E-22	3643135

C5_01730W_A	-5.96345	5.05E-06	3643730
C4_02520C_A	-5.97036	8.94E-17	3637863
ATO6	-5.97417	9.64E-06	3643664
C3_06420C_A	-5.98049	3.94E-15	3642262
DRE2	-5.98202	1.07E-18	3642571
CR_03320C_A	-5.99941	0.032394	3645096
C2_10130W_A	-6.01483	0.001082	3643935
NOP6	-6.02766	1.67E-13	3639507
RGS2	-6.03159	3.85E-16	3646629
C2_04110W_A	-6.03928	1.27E-05	3637378
C1_14330W_A	-6.04508	1.13E-09	3643278
PHO84	-6.07279	2.28E-10	3645348
C5_04080C_A	-6.0812	3.09E-15	3647731
C5_02320C_A	-6.09999	3.31E-09	3637788
ALS5	-6.16116	6.85E-18	3640277
C2_10810W_A	-6.19402	1.41E-16	3641243
C1_07660W_A	-6.19785	3.2E-07	3646656
PHR3	-6.21502	1.44E-23	3645003
CR_02030C_A	-6.22322	1.01E-15	3640152
KRE6	-6.24519	3.24E-14	3641756
PUF3	-6.25731	1.28E-17	3646950
CPA1	-6.26036	2.75E-10	3635715
C7_03850W_A	-6.34449	1.55E-14	3643145
C6_02290C_A	-6.34486	1.65E-16	3645873
C2_04700C_A	-6.39203	4.64E-06	3647794
C6_03800C_A	-6.40506	3.25E-13	3640310
PGA13	-6.41719	0.00982	3642138
C1_13390W_A	-6.44571	1.39E-08	3636218
C4_07040W_A	-6.48264	2.92E-05	3642963
C5_01020C_A	-6.5031	0.004294	30515297
C7_00330C_A	-6.54887	1.34E-13	3638042
VTC4	-6.56184	2.12E-25	3646795
GST2	-6.56577	6.21E-18	3637937
C3_02040C_A	-6.58129	4.07E-18	3636579
C5_02980C_A	-6.59484	6.75E-10	3637816
CR_09930W_A	-6.60746	4.3E-11	3639029
ALD6	-6.62763	2.62E-14	3647407
WAR1	-6.64449	4.81E-19	3644691
ROA1	-6.65362	4.7E-24	3645424
C5_01560C_A	-6.66243	2.82E-14	3647064
C6_01780C_A	-6.6818	8.49E-08	3641636
GPD1	-6.69392	8.75E-20	3643924
TPS3	-6.71532	1.28E-13	3641205
C1_09780C_A	-6.73014	2.06E-19	3634842
C3_06940W_A	-6.74345	4.22E-25	3638509
ZCF28	-6.74892	5.84E-08	3634776
MDM34	-6.77899	3.91E-23	3647517

HBR3	-6.7972	1.67E-17	3645652
TYE7	-6.81311	1.73E-24	3636203
C1_06090C_A	-6.8296	1.04E-17	3639822
C3_05800W_A	-6.84326	1.14E-16	3641754
CR_06550C_A	-6.86932	1.18E-17	3645260
COX17	-6.88291	3.8E-07	30515088
KCS1	-6.89409	2.88E-12	3648120
ATG9	-6.91695	1.01E-14	3635239
C3_02980C_A	-6.91823	4.96E-08	3638386
C2_04520C_A	-6.93731	1.85E-05	3645447
SLY41	-6.94398	5.54E-19	3647139
MSS116	-6.95057	7.81E-20	3634792
C1_12750C_A	-6.97146	1.1E-12	3646818
NBP35	-7.00915	4.53E-21	3647412
SNQ2	-7.03992	5.52E-27	3640290
STE11	-7.06143	6.12E-25	3637369
CTA8	-7.07198	5.48E-19	3634947
LTV1	-7.08012	1.64E-19	3639016
RRN11	-7.08818	3.38E-14	3645276
C3_03370C_A	-7.09298	2.25E-12	3638366
C2_05840W_A	-7.09563	3.08E-16	3647484
RRN3	-7.1154	6.2E-28	3647925
C6_02660C_A	-7.12591	1.55E-07	3639224
FMP45	-7.14511	4.94E-20	3637004
AAH1	-7.16276	1.07E-07	3643529
WHI3	-7.17093	6.46E-22	3636991
CCC2	-7.19306	8.88E-19	3637821
FTH1	-7.22689	1.7E-25	3634852
PGA48	-7.38104	3.51E-20	3647905
SSY1	-7.38816	1.95E-27	3637382
SAS10	-7.395	8.67E-24	3637899
ALS6	-7.39595	9.86E-15	3642266
MRP17	-7.40383	4.89E-19	3641548
C4_01220C_A	-7.43199	4.83E-08	3635650
C1_10060C_A	-7.43551	9.2E-07	3634812
MAK16	-7.44279	7.83E-20	3646920
RFG1	-7.48407	4.89E-34	3642570
OYE23	-7.48788	8.21E-06	3641618
C1_05920W_A	-7.5296	0.001455	3639864
C2_08030W_A	-7.57969	2.57E-10	3642309
FGR46	-7.58623	0.003399	3638989
C1_02450C_A	-7.5911	1.58E-11	3636849
C6_03200W_A	-7.60552	8.16E-23	3639177
C1_12630C_A	-7.63205	1.66E-09	3646838
C4_04200C_A	-7.63278	0.010367	3641182
GZF3	-7.63552	2.34E-31	3642563
POP3	-7.6467	3.81E-09	3638993

FLO8	-7.67333	1.51E-29	3646292
C1_13010W_A	-7.69926	3.51E-19	3636120
C3_05250C_A	-7.76167	1.26E-14	3635199
FYV5	-7.79494	9.02E-07	3647057
CYC3	-7.8125	2.7E-28	3642621
RAS2	-7.85692	1.23E-16	3635211
C2_03710W_A	-7.85855	0.037247	3637374
GRX1	-7.8592	0.029491	3637026
NRG2	-7.8592	0.029491	3647893
C2_02220C_A	-7.85967	0.029116	3635774
C4_07020C_A	-7.85967	0.029116	3642965
C5_02800C_A	-7.85985	0.034004	3637807
C3_04450C_A	-7.86032	0.029112	3635207
C4_02190C_A	-7.86032	0.029112	3635697
C2_08700C_A	-7.89303	6.41E-26	3643887
CR_04220C_A	-7.92069	1.94E-11	3640943
C4_05160C_A	-7.98831	5.69E-22	3647411
C1_01610C_A	-8.04303	1.15E-09	3640225
CR_03660C_A	-8.09509	5.73E-13	3643219
C1_06750W_A	-8.09778	2.01E-05	3639506
SUR2	-8.13911	2.15E-14	3642973
C7_02610C_A	-8.14457	2.73E-24	3648285
MTM1	-8.16764	3.71E-18	3642487
C1_10950C_A	-8.21152	2.93E-24	3644428
CR_04920W_A	-8.39487	1.11E-18	3648207
SEO1	-8.41484	0.000215	3646621
EBP1	-8.42221	8.01E-15	3644060
C1_09820C_A	-8.4264	0.000634	3634846
C3_00150W_A	-8.45345	3.49E-06	3640595
FTR1	-8.46961	7.6E-21	3643318
C4_04300C_A	-8.53907	1.55E-08	3646843
CR_08830W_A	-8.58539	5.88E-12	30515450
C3_05440C_A	-8.66387	1.14E-08	3643668
C2_02550C_A	-8.74994	0.001456	3635856
SPO1	-8.74994	0.001411	3647039
C3_04510W_A	-8.75271	5.78E-11	3635258
ARF1	-8.75744	1.48E-23	3642130
C5_01710C_A	-8.78345	3.23E-13	3643732
KTI11	-8.80141	1.13E-05	30515125
CR_03980W_A	-8.82352	4.54E-15	3640964
GAC1	-8.8508	1.09E-22	3638025
TAZ1	-8.89479	1.14E-09	3644108
NOG2	-8.91403	3.08E-27	3640299
ARR3	-8.92648	8.35E-11	3642910
C2_00760C_A	-8.94171	1.31E-19	3638910
ZCF3	-8.98792	4.58E-23	3645335
RSM22	-8.99134	2.34E-23	3642506

GCA2	-9.01361	4.15E-22	3647194
C4_00810C_A	-9.0399	2.53E-09	3645742
C6_02950C_A	-9.08111	2.5E-09	3639188
C7_04160W_A	-9.12359	1.62E-09	3643127
CHR1	-9.12782	1.57E-31	3647601
C2_06520C_A	-9.26622	2.55E-09	3646571
C4_01240C_A	-9.32393	6.95E-24	3635705
CR_04680C_A	-9.40041	0.002383	3648142
ECM22	-9.45964	1.25E-20	3648000
UTP22	-9.61616	1.83E-24	3635760
CDR1	-9.65595	7.4E-30	3635237
GCS1	-9.7155	4.76E-33	3640742
C2_10090C_A	-9.73087	2.66E-19	3643939
C1_05330C_A	-9.78682	1.47E-26	3642485
CEK2	-9.80063	1.27E-07	3642459
C6_03360C_A	-9.84441	3.15E-08	3639203
C3_01430W_A	-9.84666	3.4E-12	3636633
CDR11	-9.92484	1.56E-24	3647389
CYS3	-9.92507	1.4E-31	3642081
C1_12440W_A	-9.94521	0.000235	3642012
ATG1	-9.95089	1.93E-33	3641175
CR_02910W_A	-9.97492	6.18E-23	3642586
CR_08470W_A	-9.99736	1.48E-29	3642134
SRR1	-10.0261	3.31E-18	3647589
WOR3	-10.0884	2.67E-14	3641009
RNR3	-10.1754	3.03E-18	3647591
C1_13250W_A	-10.1907	1.41E-39	3636143
PZF1	-10.204	1.23E-15	3648113
C4_03460C_A	-10.2209	2.3E-05	3646813
CR_08580C_A	-10.248	2.18E-11	3642105
BRG1	-10.3587	4.38E-25	3647664
STP4	-10.3602	7.21E-26	3637405
C3_04730C_A	-10.4378	0.000532	3635250
C3_05900W_A	-10.4609	5.95E-17	3641739
QDR3	-10.597	8.44E-15	3644023
CTR2	-10.6415	1.98E-28	3648427
C5_02590C_A	-10.8757	5.34E-16	3637723
C1_04710C_A	-10.9831	8.05E-15	3644764
ELA1	-11.1971	1.06E-15	3644680
C6_00730W_A	-11.2467	0.007424	3647070
CR_01250C_A	-11.2473	0.008001	3640403
C3_05790C_A	-11.391	2.76E-09	3641753
C2_01060C_A	-11.4649	8.46E-11	3638835
C1_13130C_A	-11.5185	1.38E-25	3636202
UGA6	-11.5216	7.1E-07	3642975
C4_02770C_A	-11.576	1.15E-11	3637903
C1_11510C_A	-11.6151	1.65E-23	3645336

C1_01510W_A	-11.6795	7.18E-28	3640229
PGA37	-11.7365	3.26E-09	3636484
SCH9	-11.8877	1.4E-37	3637421
C5_02640W_A	-11.895	5.58E-18	3637785
MFG1	-12.1363	2.06E-37	3643884
C6_04420W_A	-12.231	8.12E-17	3645951
FAR1	-12.2423	4.55E-16	3637970
C1_11320C_A	-12.2568	5.35E-22	30515052
C1_07790C_A	-12.2667	8.73E-16	3640706
C2_08580W_A	-12.3282	2.36E-07	3643916
C1_10580C_A	-12.3285	8.35E-07	3647679
C2_02530W_A	-12.3585	0.001724	3635819
C2_02580W_A	-12.3811	2.27E-20	3635860
C2_02190C_A	-12.9277	2.29E-21	3635753
C1_08610C_A	-12.9307	7.47E-41	3646512
QDR2	-13.1507	6.14E-38	3643661
C1_05440C_A	-13.2301	9.97E-15	3642489
GRF10	-13.401	3.52E-30	3636492
DEF1	-13.4988	1.39E-35	3639023
HGT17	-13.5297	1.71E-05	3635687
SUT1	-13.5503	3.93E-20	3637706
ITR1	-13.8712	8.02E-53	3643440
C5_04840C_A	-13.9483	8.72E-26	3636435
FEN1	-13.9796	3.17E-18	3646829
HEM3	-14.0117	4.69E-40	3643940
C3_06950W_A	-14.0535	1.77E-21	3638507
ABC1	-14.095	4.65E-32	3640239
C2_09610W_A	-14.1862	6.37E-22	3643798
RFX1	-14.2167	1.53E-15	3644844
C4_04520W_A	-14.2226	1.66E-32	3641110
C6_03180C_A	-14.2364	3.32E-61	3639261
C1_06870C_A	-14.5173	0.018295	30515013
C1_12800W_A	-14.5173	0.018295	3646828
C4_00530C_A	-14.5181	0.018107	3645746
AGA1	-14.5184	0.01811	3641536
C1_05690C_A	-14.5184	0.018295	3639896
C2_08350C_A	-14.5184	0.018295	3640769
C2_10000C_A	-14.5184	0.018295	30515163
OFI1	-14.519	0.019824	3636225
ALK6	-14.5193	0.018107	3637045
CR_04610C_A	-14.5193	0.018107	3640951
C7_02000C_A	-14.5202	0.019438	3636964
C2_04090W_A	-14.5851	0.00482	3637380
C3_04870W_A	-14.6362	0.001344	3635226
C7_03550C_A	-14.6371	0.001854	3646235
CTA4	-14.6457	2.69E-42	3641743
TRY4	-14.7061	1.73E-18	3635240

C3_03250W_A	-14.7263	7.18E-43	3638318
C5_04850W_A	-14.7443	1.22E-08	3636436
C4_04020C_A	-14.8212	0.000898	3641215
C7_01910C_A	-14.9196	2.22E-26	3637029
SEN2	-14.9296	3.22E-24	3637832
IFA14	-15.2375	2.49E-12	3639012
RPN4	-15.2975	1.57E-44	3644684
COX11	-15.334	3.27E-38	3646842
WSC1	-15.4207	8.29E-31	3635138
LIP1	-15.7431	1.45E-06	3634888
C3_05750C_A	-16.1447	7.27E-06	3641719
DED1	-16.2609	8.62E-44	3641752
CR_01600C_A	-16.7845	1.51E-13	3640080
C1_09320C_A	-16.7867	7.75E-51	3634834
ZCF2	-17.0157	3.71E-49	3642484
RCH1	-17.0327	1.81E-29	3645002
C1_13820C_A	-17.3556	8.57E-34	3636127
C2_07070W_A	-17.4091	7.57E-40	3643501
CUP9	-17.4647	2.48E-29	3636950
C4_06200W_A	-17.5709	2.91E-06	30515282
PTC8	-17.5718	1.19E-40	3635674
SMM1	-17.7178	6.73E-21	3643778
C6_03810W_A	-17.8375	5.1E-32	3640286
C2_08530C_A	-18.088	1.2E-10	3643921
C6_01870C_A	-18.118	2.5E-24	3641625
CR_08080W_A	-18.2385	1.49E-05	3642094
C7_02600C_A	-18.3847	2.97E-44	3648286
MET1	-18.496	3.78E-45	3643014
C6_03440W_A	-18.5428	1.71E-30	3640274
C4_02740W_A	-19.0194	2.48E-29	3637855
C2_04670W_A	-19.0277	6.71E-18	3647789
SSU81	-19.0663	3.7E-22	3634944
C5_03080C_A	-19.1169	1.58E-40	3637701
C7_00630C_A	-19.2482	6.37E-09	3638028
C7_00880C_A	-19.3614	4.23E-26	3638052
CR_07680C_A	-19.8739	4.98E-09	3648193
FRE10	-20.0088	2.09E-38	3646841
CR_08720W_A	-20.1007	9.42E-14	3642122
C3_02750W_A	-20.1962	2.42E-06	3638411
C1_07650W_A	-20.3098	1.76E-16	3646657
ALS7	-20.8784	2.07E-50	3642234
RIM8	-21.1266	1.25E-49	3639342
C6_01020W_A	-21.2116	0.00694	3644050
KSP1	-21.267	2.5E-60	3644467
MNN1	-21.3818	2.1E-17	3637784
WOR2	-21.6015	6.91E-13	3635230
RME1	-21.6374	1.27E-33	3644453

HSP21	-21.7154	2.07E-28	3637364
C2_09880C_A	-21.8566	7.15E-24	3643779
C1_01130W_A	-22.5388	1.28E-59	3640178
PUT1	-22.7263	1.12E-61	3637779
OPT4	-23.1833	5.13E-42	3647697
C4_07140W_A	-23.2754	1.54E-48	3642936
C1_09210C_A	-24.2635	8.41E-46	3634897
CR_04940W_A	-24.6581	2.05E-15	3648209
FGR47	-24.7305	1.17E-66	3641651
C3_06660C_A	-24.7998	0.000155	3642253
C1_09770W_A	-25.5724	5.41E-36	3634825
C1_06620C_A	-27.0355	0.001599	3639545
CR_07540C_A	-27.0355	0.00158	3648001
C6_03280W_A	-27.0377	0.001576	3639185
FGR42	-27.0377	0.001579	3647438
C1_07040C_A	-27.0394	0.002002	3639533
C5_04380C_A	-27.0768	1.55E-12	3636485
C1_01620C_A	-28.466	6.82E-49	3640193
C1_11410C_A	-28.8406	3.1E-08	30515054
CDA2	-30.3991	5.57E-10	3639716
C4_05350W_A	-30.4761	6.36E-71	3646948
C5_01990W_A	-31.3485	0.000193	3643774
C5_05240C_A	-33.2952	0.00068	3636471
C1_00190C_A	-33.5611	6.43E-36	3639337
HSP31	-34.0461	3.97E-16	3644589
JEN2	-35.4732	4.13E-25	3641214
HAK1	-35.5909	2.38E-07	3639546
C7_01430C_A	-36.8909	1.91E-81	3636961
C2_05330C_A	-38.2871	5.52E-32	3643426
C2_06530W_A	-38.6995	2.66E-18	3646572
ARG11	-40.2359	5.59E-08	3635193
PRX1	-40.3126	1.62E-61	3641357
GIT3	-41.1804	1.37E-68	3642634
FRE9	-42.0899	1.34E-50	3643452
C4_03340C_A	-42.2182	3.09E-14	3648090
PGA57	-42.5493	3.4E-08	3635608
CFL4	-43.4123	3E-14	3642639
AOX2	-44.5952	1.11E-45	3634945
C5_04030W_A	-45.7296	1.44E-49	3647737
BMT1	-46.4745	1.33E-64	3638535
CLG1	-47.8743	2.86E-89	3646030
ZCF1	-50.4125	4.82E-56	3638329
TRY6	-51.9003	7.31E-07	3638499
CR_08880C_A	-60.4004	2.74E-40	3641878
SSU1	-64.727	1.23E-73	3641882
CR_06140W_A	-67.7026	3.21E-41	3644862
C4_07260W_A	-70.351	1.78E-16	3642942

C2_08260W_A	-77.3753	4.69E-12	3642345
C3_03460C_A	-100.623	1.19E-13	3638359
C7_01680C_A	-114.134	2.18E-85	3636981
C6_00810C_A	-135.115	2.65E-40	3647068
C4_07000W_A	-136.008	0.042247	3642967
CR_06870C_A	-136.016	0.042244	3647318
C2_02130C_A	-147.088	1.2E-15	3635747
PHO87	-149.396	1.5E-15	3639861
CR_01530C_A	-163.387	3.46E-16	3640072
C5_00810C_A	-176.984	0.031332	3644127
C2_09330C_A	-217.962	0.024789	3644106
C7_00770W_A	-251.543	6.66E-26	3637959
C3_07930C_A	-271.31	0.022461	3647452
HSP30	-275.508	2.7E-44	3645419
CR_00840C_A	-299.927	0.017681	3640411
C1_06430C_A	-340.831	0.01539	3639553
CR_01510C_A	-340.893	0.015099	3640104
C1_02690C_A	-342.692	0.015583	3636823
C7_03560W_A	-383.892	2.3E-124	3646236
GPI1	-384.925	4E-34	3643680
MRV4	-463.776	0.010505	3636547
HWP2	-494.125	0.010453	3646803
C1_05250W_A	-791.506	0.005147	3642462
C3_06170C_A	-832.65	0.004823	3642272
CRZ2	-2384.7	1E-70	3645725
C4_06910W_A	-5520.62	0.000275	3642933
C1_14630C_A	-29635.3	1.54E-05	3643291
TAR1	-72057.2	0	30515449
